# High resolution chromosomal microarray analysis in paediatric obsessive-compulsive disorder

**DOI:** 10.1186/s12920-017-0299-5

**Published:** 2017-11-28

**Authors:** Edna Grünblatt, Beatrice Oneda, Arif B. Ekici, Juliane Ball, Julia Geissler, Steffen Uebe, Marcel Romanos, Anita Rauch, Susanne Walitza

**Affiliations:** 1 Department of Child and Adolescent Psychiatry and Psychotherapy, University Hospital of Psychiatry Zurich, University of Zurich, Neumünsterallee 9, 8032 Zürich, Switzerland; 2 Neuroscience Center Zurich, University of Zurich and ETH Zurich, Zurich, Switzerland; 3 Department of Psychiatry, Psychosomatic and Psychotherapy, University Hospital of Würzburg, Würzburg, Germany; 4 Zurich Center for Integrative Human Physiology, University of Zurich, Zurich, Switzerland; 5 Institute of Medical Genetics, University of Zurich, Zurich-Schlieren, Switzerland; 6 Center of Mental Health, Department of Child and Adolescent Psychiatry, Psychosomatics and Psychotherapy, University Hospital of Würzburg, Würzburg, Germany; 7 Institute of Human Genetics, University Hospital Erlangen, Erlangen, Germany; 8 Department of Child and Adolescent Psychiatry and Psychotherapy, University Hospital of Psychiatry Zurich, University of Zurich, Wagistrasse 12, 8952 Schlieren, Switzerland

**Keywords:** OCD, CNV, Enrichment analysis, De-novo, Early-onset

## Abstract

**Background:**

Obsessive-Compulsive Disorder (OCD) is a common and chronic disorder in which a person has uncontrollable, reoccurring thoughts and behaviours. It is a complex genetic condition and, in case of early onset (EO), the patients manifest a more severe phenotype, and an increased heritability. Large (>500 kb) copy number variations (CNVs) previously associated with autism and schizophrenia have been reported in OCD. Recently, rare CNVs smaller than 500 kb overlapping risk loci for other neurodevelopmental conditions have also been reported in OCD, stressing the importance of examining CNVs of any size range. The aim of this study was to further investigate the role of rare and small CNVs in the aetiology of EO-OCD.

**Methods:**

We performed high-resolution chromosomal microarray analysis in 121 paediatric OCD patients and in 124 random controls to identify rare CNVs (>50 kb) which might contribute to EO-OCD.

**Results:**

The frequencies and the size of the observed rare CNVs in the patients did not differ from the controls. However, we observed a significantly higher frequency of rare CNVs affecting brain related genes, especially deletions, in the patients (OR = 1.98, 95% CI 1.02–3.84; OR = 3.61, 95% CI 1.14–11.41, respectively). Similarly, enrichment-analysis of CNVs gene content, performed with three independent methods, confirmed significant clustering of predefined genes involved in synaptic/brain related functional pathways in the patients but not in the controls. In two patients we detected *de-novo* CNVs encompassing genes previously associated with different neurodevelopmental disorders (*NRXN1, ANKS1B, UHRF1BP1*).

**Conclusions:**

Our results further strengthen the role of small rare CNVs, particularly deletions, as susceptibility factors for paediatric OCD.

**Electronic supplementary material:**

The online version of this article (10.1186/s12920-017-0299-5) contains supplementary material, which is available to authorized users.

## Background

Obsessive-compulsive disorder (OCD) is characterized by distressing, intrusive obsessive thoughts and/or repetitive compulsive behaviours [1]. According to a survey of adult US citizens, OCD is a frequent psychiatric disorder with 2.3% of respondents meeting full OCD DSM-IV criteria for lifetime and 1.2% for 12-month prevalence [2]. Similar prevalence rates of 0.1 to 2.3% have been reported in European countries [3]. In up to 50% of the cases, OCD emerges already during childhood or adolescence [4], and a bimodal age of onset, during early puberty or in the early twenties [5], has been described. The definition of “early-onset” OCD (EO-OCD) differs among studies regarding the cut-off for age of onset; below 7 years of age was the lowest and under 18 years of age was the highest cut-off age, which thus marks the “minimal” consensus for definition criteria. Accordingly, patients with “late onset” (LO) must be older than 18 years at onset. EO-OCD may represent a more severe or more “biological” subtype of the disorder in comparison to LO [6], with an increased heritability as well as differences in gender distribution, symptomatology, and comorbidity. In addition, in OCD with early and childhood onset, triggering life events seem to be observed to a lesser extent [2, 7]. Therefore, it has been hypothesized that EO-OCD represents a specific highly heritable subtype of OCD [8].

Despite the strong heritability estimates between 40 and 80% [9–11], two recent genome-wide studies [12, 13] failed to detect association of common single nucleotide polymorphisms (SNPs) with OCD. However, in a meta-analysis [14] an association between OCD and polymorphisms in the serotonin-related genes (*5-HTTLPR*/ *SLC6A4* and *HTR2A*) and in the catecholamine modulation genes (*COMT* and *MAOA*) have been reported. Similarly, we showed for the first time the association of 5-*HTTLPR* polymorphisms with EO-OCD [15, 16] as well as association to *HTR2A* polymorphism [17].

In order to further explain the heritability, it has been suggested that copy number variations (CNVs) play an important role in neurodevelopmental disorders [18–20]. There is rising awareness that common variants may explain a relatively small amount of common diseases, while rare variants with relatively large functional effects may add up to a significant disease contribution [21].

Up to now, five CNV studies in patients with OCD have been conducted [22–26]. The first study assessed the recurrent 15q11-q13 Prader-Willi syndrome and the 22q11.2 DiGeorge syndrome regions [22] in adults with OCD, since both disorders are often accompanied by OCD symptoms. However, in 236 OCD-patients no CNVs affecting the two regions were detected [22]. In a study, conducted by our group, an association between a ~100 base pairs (bp) deletion in the *HTR2A* promoter region and paediatric EO-OCD [23], as well as increased OCD-severity and an earlier age-of-onset, were reported. Recently, the first genome-wide analysis in a cohort of 1613 OCD and 1086 Tourette syndrome (TS) patients was performed. The study focused on CNVs larger than 500 kb and revealed aberrations in 16p13.11, 22q11.21 and in 6q25.2-q27 [24]. Additionally, a study on CNVs smaller than 500 kb screened 16 adults with EO-OCD and 12 controls [25] and a rare intragenic *FMN1* microdeletion in 15q13.3 was detected. In a very recent study 307 EO-OCD (259 of European ancestry) have been investigated for small and rare CNVs [26]. Interestingly, the authors found CNVs in genes involved in neuronal migration, synapse formation and postsynaptic scaffolding, which might be relevant to the pathogenesis of OCD. Four cases had CNVs involving known genomic disorder loci (1q21.1, 15q11.2-q13.1, 16p13.11 and 17p12). [26].

In light of these current findings and the notion of a stronger genetic contribution in patients with EO-OCD, we conducted a genome-wide CNVs analysis, using high-resolution chromosomal microarray analysis (CMA) in 121 homogenous phenotyped paediatric EO-OCD patients.

## Methods

### Paediatric patients with OCD

121 paediatric patients with EO-OCD were recruited from the Departments of Child and Adolescent Psychiatry at the University of Würzburg, Germany and the University of Zürich, Switzerland (all Caucasian). The mean age ± SD of the patients was 12.99 ± 2.8; mean age of onset = 10.43 ± 3.2; 75 were males and 46 females. Although an EO-OCD sample could in principle also consist of adult patients with a retrospectively analysed onset, the present study includes only paediatric patients that guaranteed an early-onset of the disorder.

All patients fulfilled the diagnostic criteria for OCD according to the Diagnostic and Statistical Manual of Mental Disorders, 4th edition (DSM-IV) [27] and the International Statistical Classification of Diseases and Related Health Problems, 10th Revision (ICD-10) [28]. Patients and parents from Würzburg were interviewed separately by senior clinicians with a semi-structured diagnostic interview of psychiatric disorders in children and adolescents (Kinder-DIPS; children and parents version) [29]. The patients and parents of Zürich underwent the German version of a semi-structured clinical interview (K-SADS-PL) [30] to investigate their phenotype, including psychiatric comorbidities. Autistic spectrum disorders were screened within an ascertainment for psychopathology (CASCAP-D) [31].

The Children’s Yale Brown Obsessive Compulsive Scale (CY-BOCS) [32] was used in all patients to assess severity and characteristics of OCD symptomatology. A summary score above 16 points was determined to be the cut-off for clinical impairment caused by OCD. In the present patients’ sample, the mean CY-BOCS score was 22.1, SD = 6.98. The DSM-IV and DSM-5 [1] do not require children to fulfil the criterion of insight into the irrationality of their symptoms. Therefore, it is possible that the CY-BOCS scores underestimated the severity of OCD in cases of reduced insight into the disorder. For the assessments of TS and tic-disorders, the German version [33] of the Child and Adult Schedule for Tourette and Other Behavioural Syndromes (STOBS) [34] was used in the Würzburg patients, the Yale Global Tic Severity Scale (YGTSS) [35] in the Zürich patients.

Patients with comorbid disorders (*n* = 75 /121) were included in the study when the OCD was the primary diagnosis, as assessed by two senior clinicians independently (see Additional file 1: Table S1).

Exclusion criteria were: lifetime history of psychotic disorders, Tourette’s disorder (TS), autistic spectrum disorder (ASD), alcohol dependence, intellectual disability (ID; IQ < 70). IQ was assessed by the Wechsler Intelligence Scale (WISC) [36] in the majority of cases (over 85%) while in few cases (less than 15%) the IQ was assessed by Culture Fair Intelligence Test (CFT) [37]. Since IQ < 70 was used only for exclusion criteria to avoid inclusion of any ID probands, using two scales do not affect the paediatric OCD population selection. Mean IQ score in our patient cohort was 108.27 (SD = 13.29).

The study was approved by the respective local ethics committees with the latest version of the Declaration of Helsinki, including an ethical permission granted by the Ethic Committees from Würzburg and the Cantonal Ethic Commission of Zürich (Ref. Nr. 39/97, 140/3 and EK: KEK-ZH-Nr. 2010–0340/3) and written informed consent was obtained in all cases from the participants or/and their parents.

### Control sample for statistical comparison

We analysed the data of 124 random population samples of Caucasian origin (DNA was extracted from native tissue), recruited at the Institute of Medical Genetics, University of Zurich, in the same manner, on the Cytoscan HD Array as the patient samples (76 males and 48 females, X^2^ = 0.012, *p* > 0.999 compared to EO-OCD).

### DNA extraction and chromosomal microarray analysis (CMA)

Constitutional genomic DNA was extracted from whole blood (EDTA-tubes; *n* = 74 cases and family) with the desalting Proteinase K methodology [38] or from saliva samples (Oragene DNA, DNA Genotek Inc., Ontario, Canada; *n* = 47 cases and family) using the manufacturer’s protocol. To assess genomic DNA purity, the ratio of absorbance at 260 nm and 280 nm was used. 74/121 cases were part of the previous reported genome-wide CNV (>500 kb) analysis [24], in which only one large CNV was reported and confirmed in the current study (patient #9025079001 7q21.11 deletion), without any previous findings that were not confirmed in the current study. DNA from available parents and/or siblings of 30 patients was investigated consecutively due to CNV findings in the patients.

DNA was analysed with the Cytoscan HD Array (containing about 750,000 SNPs and 1.9 million non-polymorphic probes) (Affymetrix Inc., Santa Clara, CA, USA) at a genome-wide resolution of 50 kb for both duplications and deletions. We have chosen this resolution in order to minimize the possibility of false positive CNVs, which according to our previous assessment have a median size of 19 kb [20]. Array hybridization and quality control was performed according to the manufacturer’s protocol. Data were analysed with Chromosome Analysis Suite (ChAS) software (Affymetrix) for changes of relative intensities. The CNV analysis was based on build 32.1. Genomic coordinates are based on GRCh37/hg19. In order to exclude common benign CNVs, we used a reference set of 820 in-house healthy controls and 1038 Affymetrix controls in combination with the Database of Genomic Variants (DGV) from the Centre for Applied Genomics (February 2009, hg19).

CNVs including coding sequences of genes that were absent in our in-house and Affymetrix primary control cohort and either unreported or reported only from limited sources in the DGV database were defined as rare CNVs in both the EO-OCD and the random control cohorts and used for statistical comparison.

### Statistical analysis and enrichment analysis

Frequency analysis was conducted using Χ^2^ test and the power was calculated using G*Power v.3.1.9.2 [39]. For continuous measures, the two-tailed t-test was used.

The genes within the rare CNVs identified in the patients and controls were investigated for their enrichment in pathways and functional groups using the Enrichment Analysis of Selected Entities (EASE) tool, based on Gene Set Enrichment Analysis (GSEA) method [40, 41], with the Pathway Studio software v.11.2.04 (Elsevier; Mammal database). Significant enriched pathways were considered with *p* < 0.005. As additional validation, we used the Database for Annotation, Visualization and Integration Discovery (DAVID) [42] for functional annotation clustering. The genes within CNVs were entered using the identifier “Official_gene_symbol” to create gene lists for EO-OCD and for control samples. We reported clusters with enrichment score > 1.3 as suggested by Huang da W et al. [42]. Significant enriched pathways were considered with *p* < 0.05 following Bonferroni, Benjamini and FDR corrections. In addition, as a third confirmation analysis, we performed a CNV enrichment analysis using PLINK [43] version 1.07 and its --cnv-enrichment-test option on all CNVs called [44]. Following multiple testing correction (6 tests) *p* < 0.008 was considered as significant, and nominal significant for *p* < 0.05.

Statistical analysis was performed with SPSS v.21 (IBM) and StatView v.5.0 (SAS Inst.). The level of significance was alpha = 0.05.

## Results

We detected rare CNVs (mean size = 258 ± 308 kb, min. 50 kb max. 1500 kb) in 41 out of 121 patients (Table 1; for details see Additional file 2: Table S2a) and in 39 out of 124 controls (mean size = 274 ± 234 kb, min. 50 kb max. 1027 kb; for details see Additional file 2: Table S2b). There was no significant difference in the number of all detected rare CNVs between patients (*n* = 41/121; 34%) and controls (*n* = 39/124; 31%; Χ^2^ = 0.165, *p* = 0.685) nor in the CNVs’ size (t(−74.47) = 0.255, *p* = 0.8). Moreover, no significant difference was observed in IQ scores or OCD severity (measured with CY-BOCS) between carriers and non-carriers of rare CNVs.

**Table 1:** Rare CNVs discovered in the paediatric obsessive compulsive disorder (EO-OCD) patients including inheritance pattern

Code	Gender	Diagnosis	Comorbid tics	CNV size (kb)	chromosomal location (hg19)	Genes within CNV	Inheritance	Comments
a) Deletions carriers								
*9025014001*	female	OCD	yes	51	2p16.3:51234059–51285498	***NRXN1***	de-novo	–
9925026001	male	OCD	no	170	3p22.1:42928225–43098107	*ZNF662, KRBOX1, FAM198A*	Maternal	Absent in healthy brother
*9025082001*	female	OCD	no	113	4p12:46952619–47065270	***GABRA4, GABRB1***	Maternal	–
*9025015001*	male	OCD	no	210	4q28.3:139075297–139285096	***SLC7A11*** *, LINC00499*	Maternal	–
*9025019001*	male	OCD	no	134	6p25.1:6645654–6779499	*LY86*	n.a.	–
9025107001	female	OCD	no	125	6q22.31:125494942–125619539	*TPD52L1, HDDC2*	Maternal	–
*9025079001*	male	OCD	yes	731	7q21.11:83743960–84475183	***SEMA3A***	Paternal	–
9025101001	male	OCD	no	83	10p11.21:34672540–34755348	***PARD3***	Paternal	–
*9025043001*	male	OCD	yes	310	12q23.1:100148198–100458394	***ANKS1B*** *, UHRF1BP1L*	de-novo	overlap to 190 kb deletion Chr12 (*ANKS1B*) in European paediatric OCD female [26]
*9025045001*	female	OCD	no	297	15q21.3:53716860–54014105	***WDR72***	Maternal	–
*9025069001*	female	OCD	no	105	15q24.2:76235495–76340932	***NRG4***	Maternal	–
9025100001	male	OCD	no	1500	16p13.11:15509406–16516109	*C16orf45, KIAA0430, NDE1, MIR484,* ***MYH11*** *, FOPNL,* ***ABCC1*** *, ABCC6, NOMO3, MIR3179–1, MIR3179–3, MIR3179–2, MIR3180–1, MIR3180–3, MIR3180–2, PKD1P1*	n.a.	overlap to 783 kb duplication Chr16 (*MYH11,KIAA0430,MPV17L,NDE1,FOPNL,C16orf45,NPIPA5,ABCC1*) in European paediatric OCD male maternally inherited [26]
*9025040001*	female	OCD	no	100	16p13.3:6294808–6394343	***RBFOX1***	Maternal	–
*9025076001*	female	OCD	no	373	19q13.12:37378717–37752059	*ZNF829, ZNF568, ZNF420, ZNF585A, ZNF585B, ZNF383*	Maternal	–
9925012001	male	OCD	yes	54	21q21.1:22856032-22910383	***NCAM2***	n.a.	–
9925015001	male	OCD	no	279	Xq11.2:63540728–63819338	***MTMR8***	Maternal	Brother (Anxiety) carrier of CNV as well; Mother (Anxiety)
*9025093001*	male	OCD	no	101	Xq27.3:142869149-142970485	***UBE2NL***	Maternal	–
b) Duplication carriers								
*9025030001*	male	OCD	no	166	1p21.2:101039885–101205680	***VCAM1***	n.a.	–
9025106001	female	OCD	no	487	1p31.1:74037092–74524344	*LRRIQ3*	n.a.	–
*9025016001*	male	OCD	no	147	1p36.12:22984535–23131772	*C1QB,* ***EPHB2***	Maternal	–
*9025094004*	male	OCD	yes	437	1q21.1:145372549–145809279	*NBPF10,* ***HFE2*** *, TXNIP, POLR3GL, ANKRD34A, LIX1L, RBM8A, GNRHR2, PEX11B, ITGA10, ANKRD35,* ***PIAS3*** *, NUDT17, POLR3C, RNF115, CD160, PDZK1, GPR89A, GPR89C*	Maternal	Sister (sub-threshold OCD) carrier of CNV as well; Mother (sub-threshold OCD); overlap to; (1) 1799 kb duplication Chr1 (*ACP6,PRKAB2,CHD1L,LOC100288142,FMO5,GJA8,NBPF24,NBPF8,BCL9,NBPF12,NBPF11,NBPF10,GPR89B,GPR89C,GJA5*) in European paediatric OCD male maternally inherited. (2) 170 kb duplication Chr1 (*CD160,RNF115,POLR3C,GPR89A,PDZK1*) in European paediatric OCD female, and (3) 165 kb duplication Chr1 (*CD160,RNF115,GPR89A,PDZK1*) in European paediatric OCD female, [26]
9925022001	female	OCD	no	1380	3p14.2: 60835192–62214802	***FHIT*** *, PTPRG*	n.a.	–
*9025027001*	male	OCD	no	278	3p25.2:12374585-12652539	*PPARG, TSEN2, LOC100129480, MKRN2,* ***RAF1***	Paternal	–
*9025029001*	female	OCD	no	58	4q13.3:72291016–72349103	***SLC4A4***	n.a.	–
*9025013001*	female	OCD	no	67	4q35.1:186023710–186090501	***SLC25A4,*** *KIAA1430*	n.a.	–
*9025091001*	male	OCD	no	484	6p21.31:34446474–34930648	***PACSIN1*** *, SPDEF, C6orf106, SNRPC, UHRF1BP1, TAF11, ANKS1A*	Paternal	–
9925001001	male	OCD	no	300, 162 & 79	6q14.1:76417785–76718017 76774410–76935941(two) & 17p13.2: 4309542–4388334	*SENP6,* ***MYO6*** *, IMPG1, SPNS3*	Paternal	Father CNV on Chr6q14:1; overlap to 73 kb duplication Chr17 (*SPNS3*) in non-European paediatric OCD male [26]
*9025089001*	male	OCD	no	246	7q11.23:72576872–72822709	*LOC100093631, GTF2IP1, NCF1B, GTF2IRD2P1, NSUN5, TRIM50, FKBP6*	Paternal	–
9925007001	female	OCD	yes	258	7q31.33:125882371–126140769	***GRM8***	Paternal	Absent in sister (OCD)
*9025036001*	female	OCD	no	89	7q36.1:149318934–149408392	*ZNF767*	n.a.	–
*9025049001*	male	OCD	no	153	7q36.3:157134943–157287531	***DNAJB6,*** *LGMD1E*	Paternal	–
*9025077001*	male	OCD	yes	158	9p13.3:33989242–34146776	*UBAP2, DCAF12*	Maternal	–
*9025020001*	female	OCD	no	52	9q34.13:134065786–134117305	*NUP214*	n.a.	–
*9025067001*	female	OCD	no	134	10p15.3:143252–277232	*ZMYND11*	n.a.	–
9925035001	male	OCD	no	131	11q12.1:58547531–58678042	*GLYATL2*	n.a.	–
*9025073001*	female	OCD	no	82	13q14.11:40310481–40392725	***COG6***	Paternal	–
9925030001	male	OCD	yes	109	13q34:110917586–111026140	***COL4A1, COL4A2***	Maternal	Absent in healthy sister
9925021001	female	OCD	no	176	14q21.1:42004775–42180366	***LRFN5***	Maternal	–
9025112001	male	OCD	no	100	14q23.1:58476607–58576320	*C14orf37*	n.a.	–
*9025082001*	female	OCD	no	66	18p11.32:2825037–2890695	***EMILIN2***	not maternal	Mother has only the deletion CNV; father not available
*9025043001*	male	OCD	yes	80	19p13.11:19749997–19830351	*GMIP, ATP13A1, ZNF101, ZNF14*	de-novo	–
9925027001	male	OCD	yes	50	19q13.2:39113548–39163701	***EIF3K, ACTN4***	Maternal	–
*9025040001*	female	OCD	no	73	20q12:37604490–37677926	***DHX35***	not maternal	Mother has only the deletion CNV; father not available
*9025078001*	female	OCD	no	313	Xp11.3:44750716–45063967	***KDM6A,*** *CXorf36*	Paternal	–

*Abbreviation: n.a.* not available, −-, not applicable, Underlines, proband carrying both deletions and duplications; **Bold**, brain/synapse related genes according to gene ontology, PubMed & GEO; *Cursive*, patients’ codes that were analysed in the previous publication for CNVs larger than 500 kb [24]; Patient # 90–25–079-001, with deletion on 7q21.11:83,743,960–84,475,183(hg19) of 731 kb /previously reported as 7q21.11:83,580,426–84,291,036(hg18) 711 kb [24]. For further details, as well as for control sample findings (e.g. CYBOCA, comorbidities etc.), see Additional file 2: Table S2a & b

However, we observed a significantly higher number of CNV carrying brain/synaptic genes in the patients in comparison to the controls (Χ^2^ = 4.225, *p* = 0.0398; odds ratio (OR) = 1.98, 95% CI 1.02–3.84; Power 1-β=0.858). In particular, the number of rare deletion carrying brain/synaptic genes was significantly higher in patients (*n* = 13 vs. 108 non-carriers) compared to controls (n = 4 vs. 120 non-carriers; Χ^2^ = 5.360, *p* = 0.021; OR = 3.611, 95% CI 1.14–11.41; Power 1-β=0.8909).

In line with this finding, both enrichment cluster analyses (Pathway Studio and DAVID), revealed a higher number of synaptic and brain related functional pathways in the patients (10 brain/synapse clusters, 103 none brain/synapse clusters in Pathway studio; 6 brain/synapse clusters, 31 none brain/synapse clusters in DAVID) in comparison to the controls (2 brain/synapse clusters, 98 none brain/synapse clusters in Pathway studio; 0 brain/synapse clusters, 25 none brain/synapse clusters in DAVID; Χ^2^ = 4.682, *p* = 0.03 for Pathway Studio; Χ^2^ = 4.488, *p* = 0.033 for DAVID Additional file 3: Table S3a & b).

Both enrichment cluster analyses resulted in similar top clusters for the patients group (KRAB domain, zinc-finger protein family) and for the control group (serum amyloid A, high-density lipoprotein), confirming the integrity of both analyses. The most significant (*p* < 0.0001) GO functional group enrichment cluster in the patient cohort belong to: axon guidance (genes within the CNVs: *RAF1, EPHB2, MYH11, SEMA3A, ITGA10, HFE2, NRXN1, COL4A1, COL4A2*), axonal fasciculation (genes within the CNVs: *EPHB2*, *NCAM2*, *SEMA3A*), synapse (genes within the CNVs: *EPHB2, GABRA4, GABRB1, NRXN1, MYO6, PIAS3, ANKS1B, PACSIN1*) and neuron cell-cell adhesion (genes within the CNVs: *NCAM2*, *NRXN1*), as analysed by Pathway Studio (for details see Additional file 3: Table S3a). Similarly, DAVID analysis revealed in the patients several GO related to synaptic and brain related genes, like in cluster 5: axonogenesis, cell morphogenesis involved in neuron differentiation, and neuron projection morphogenesis (gene cluster: *NCAM2*, *PARD3*, *NRXN1*, *SEMA3A*, *EPHB2*) (for details see Additional file 3: Table S3b). Interestingly, the control group did not show any synaptic or brain related clusters when the DAVID software was used, while only 2 clusters (*p* < 0.005) were found using Pathway Studio analysis: catecholamine metabolic process (genes within the CNVs: *SULT1A3, SULT1A4*) and axonal fasciculation (genes within the CNVs: *CNTN4, NRCAM*; for details see Additional file 3: Table S3a).

We could further confirm a significant enrichment (*p* = 1.28 x 10^−3^) of CNVs in brain expressed genes by applying the PLINK enrichment analysis [44]. Notably, there was no significance for the enrichment analysis for overall genes. The significance threshold was 8.33 x 10^−3^ (0.05/6, because 6 tests were performed) (Additional file 4: Table S4).

Where possible, the heritability of rare CNVs was assessed by analysing the parents/siblings of the index patients. We detected two *de-novo* CNVs in two patients with EO-OCD (one male and one female), both of whom had a comorbid tic disorder (Table1 & Additional file 2: Table S2a). Twenty-six patients inherited the CNVs (16 from the mother and 10 from the father). In two additional patients (9025100001 & 9925022001) CNVs were not maternally inherited (paternal DNA was unavailable). Three patients carried CNVs located on the X-chromosome: two deletions (affecting the genes *UBE2NL* and *MTMR8*) and one duplication (affecting the genes *KDM6A* and *CXorf36*). Interestingly, both deletions are hemizygous aberrations.

## Discussion

We did not detect a higher number of rare CNVs in paediatric patients with EO-OCD compared to controls, which is in line with previous publications [24, 26]. However, cluster analyses of the gene content of the rare CNVs revealed a significantly higher number of genes involved in synaptic and brain related pathways in the cases compared to controls, similarly to previous findings [24, 26]. Our results, therefore, further support the hypothesis that the aetiopathology of EO-OCD may be related to neurodevelopmental processes [9, 45]. In addition, we demonstrate that the OR of 3.6 (*p* = 0.021) for EO-OCD in carriers of rare small deletions is in the same range as that reported previously for larger deletions (OR = 4.4, *p* = 0.04) [24]. Therefore, as Gazzellone et al. [26] recently postulated, not only large CNVs >500 kb but also smaller rare CNVs (≥50 kb), particularly deletions in brain related genes, might represent a risk factor for paediatric EO-OCD.

We observed two large deletions (>500 Kb) in our cohort. The first one was a 1.5 Mb deletion in 16p13.11 with undeterminable inheritance in a male patient. The second was a 731 kb deletion in 7q21.11 (finding of this patient previously reported [24]) affecting the *SEMA3A* gene, which was inherited from the healthy father. While *SEMA3A* has not been described to be associated with human psychiatric disorders so far, the 16p13.11 deletion has previously been found to be associated with OCD [24] and with a variety of neurodevelopmental disorders, such as ASD, ID, epilepsy, and schizophrenia [46, 47], with reduced and male-biased penetrance [48]. Furthermore, a maternally inherited duplication overlapping the 16p13.11 was reported in an EO-OCD patient [26]. Therefore, the aforementioned findings support the possible causality of this aberration in OCD.

We detected another variant in a recurrent CNV locus in a male patient with EO-OCD and comorbid tics, who carries a 437 kb duplication encompassing the region 1q21.1. The duplication was inherited from the mother and present in the sister, both according to the CY-BOCS presenting sub-threshold OCD with similar phenotype including ordering behaviours. Notably, a duplication of that region has also been detected in a paediatric EO-OCD patient and his unaffected mother and dizygotic twin brother in the recently published cohort of Gazzellone et al. [26]. We, therefore, suggest that CNVs on the chromosome region 1q21.1, which have been reported to associate with congenital heart defects, developmental delay, ASD, and psychosis [49, 50], represent also a susceptibility locus for OCD. These phenotypes are subject to incomplete penetrance and variable expressivity, since in most of the cases they are inherited from apparently healthy parents [51].

We found several other smaller rare CNVs encompassing interesting candidate genes that have already been described in neurodevelopmental disorders, for which, however, additional evidence is needed to prove the possible relation with EO-OCD. One of these findings is the 258 kb duplication encompassing the gene *GRM8*, coding for a glutamate receptor, in 7q31.33, paternally inherited, observed in a female patient with EO-OCD and comorbid tics, but not in her sister with EO-OCD. CNVs encompassing the gene *GRM8* have been reported in patients with attention-deficit hyperactivity disorder (ADHD) [52] and with developmental delay, hypotonia, and strabismus [20]. Recent evidence has highlighted the role of glutamatergic synaptic dysfunctions in the cortico-striatal-thalamo-cortical circuit in the aetiology of OCD and related disorders [9, 53, 54].

A further noteworthy CNV, 113 kb deletion encompassing the two genes *GABRA4* and *GABRB1* in 4p12, was found in a female patient of maternal origin that carried also a 66 kb duplication (*EMILIN2* gene in 18p11.32). Involvement of GABA receptor subunit genes in the aetiology of autism has been reported [55–57]. Both *GABRA4* and *GABRB1* mRNA and protein were found to alter their expression in the parietal and frontal cortex and cerebellum of patients with ASD and in the lateral cerebellum of patients with schizophrenia and with affective disorders [58, 59]. In peripheral blood samples of patients with TS, mRNA expression of GABA receptors including *GABRA4* correlated with tic severity. Furthermore, there was indication of *GABRA4* being alternatively spliced in TS compared to healthy controls [60]. For the duplication, little is known about the *EMILIN2* gene and neurodevelopment. However, patients carrying large deletions in 18p11.32 were reported to have developmental delay and mental retardation [61] while linkage study could show some evidence for schizophrenia susceptibility near this region [62].

A 279 kb deletion in Xq11.2 encompassing the gene *MTMR8* was found in a patient with EO-OCD as well as in his mother and brother, who both suffer from anxiety disorder. Anxiety is very often a core feature of OCD symptomatology and in the DSM-IV [27], OCD was even classified among the anxiety disorders, which shows the close relationship between the two diseases. *MTMR8* encodes a phosphatidylinositol kinase and reduced protein expression is associated with impaired survival of specific neuronal populations [63]. Loss-of-function mutations occurring in the phosphatidylinositol kinase gene family are known to cause different X-linked neurological diseases, including schizophrenia, bipolar disorder and age-related neurodegeneration, probably due to endosomal trafficking defects and accumulations of the lipid substrates [64, 65].

A *de-novo* deletion of 51 kb in 2p16.3 was detected in a male diagnosed with very EO-OCD (4 years old at age of onset) who suffered also from comorbid tics and hyperkinetic symptoms. The deletion encompasses the *NRXN1* gene, for which an association with TS, ASD, ID, and schizophrenia has been described [24, 46, 66, 67]. Interestingly, the *NRXN1*-α knock-out mouse model supports the role of *NRXN1* in neurodevelopmental disorders, since these mice displayed non-social behaviour as well as hyperactivity and learning deficits [68].

In another patient with comorbid tics we found a *de-novo* deletion of 310 kb in 12q23.1 harbouring the genes *ANKS1B* and *UHRF1BP1* and a duplication of 80 kb in 19p13.11 harbouring the genes *GMIP*, *ATP13A1*, *ZNF101* and *ZNF14. ANKS1B is* predominantly expressed in the brain and known to interact with the amyloid beta protein precursor that may play a role in normal brain development and in the pathogenesis of Alzheimer’s disease [69, 70]. CNVs affecting *ANKS1B* have been reported in ASD and ID including a *de-novo* deletion in a male with ASD and delayed early language development but average language abilities and IQ [47, 71]. In addition, a 190 kb deletion encompassing the *ANKS1B* gene was reported in a patient with EO-OCD [26], enhancing the possible role of the gene in neurodevelopmental disorders. Within the duplication, *GMIP*, coding for RhoA-specific GTPase-activating protein, was reported to be a key factor for neuronal migration in the postnatal brain [72] as well as regulating vesicular trafficking [73]. Moreover, in a large population-based twin-family study exploring genome-wide association of obsessive-compulsive symptoms, a significant association in 4 genes (*MEF2BNB*, *RFXANK*, *MEF2BNB*-*MEF2B* and *MEF2B*) located in proximity to *GMIP* gene was reported [74].

## Conclusions

Our findings further support the role of rare CNVs in the aetiology of OCD and emphasize the role of rare small deletions encompassing brain genes as potential susceptibility factors in the aetiology of paediatric EO-OCD. Further studies are necessary to confirm the contribution of the individual variants in OCD.

## Additional files

Additional file 1: Table S1. Comorbidities according to ICD-10 of the pediatric OCD cohort. (DOCX 13 kb)

Additional file 2: Table S2a. Rare CNVs discovered in the paediatric obsessive compulsive disorder (OCD) patients; and inheritance pattern in cases with available parents. **Table S2b.** Rare CNVs discovered in the population control cohort. (PDF 236 kb)

Additional file 3: Table S3a. According to Pathway Studio. **Table S3b.** According to DAVID. (PDF 115 kb)

Additional file 4: Table S4. (**a**) CNV burden analysis results from PLINK. The *p*-values shown are for the GCNT test statistic from PLINK’s cnv-enrichment-test algorithm, applied to the particular gene list versus the entire genome. Although Brain expressed and ID genes resulted in *p* values below 0.05, only the brain-expressed genes list remains significantly enriched in CNVs after correction for multiple testing (6 tests). (**b**) CNV burden analysis results from PLINK. The Gene List used. (PDF 1722 kb)

## Abbreviations

ADHD: Attention-deficit hyperactivity disorder
ASD: Autism spectrum disorder
CMA: Chromosomal microarray analysis
CNV: Copy-number variation
CY-BOCS: Children’s Yale Brown Obsessive Compulsive Scale
DAVID: Database for Annotation, Visualization and Integration Discovery
DGV: Database of Genomic Variants
EO: Early-onset
GEO: Gene Expression Omnibus
GO: Gene ontology
ID: Intellectual disability
IQ: Intelligence quotient
LO: Late-onset
OCD: Obsessive-compulsive disorder
OR: Odds ratio
SNPs: Single nucleotide polymorphisms
TS: Tourette syndrome
