# Supplementary material for: High resolution chromosomal microarray analysis in paediatric obsessive-compulsive disorder
Source: BMC Med Genomics. 2017 Nov 28;10:68. doi: 10.1186/s12920-017-0299-5 (PMC5704537; doi:10.1186/s12920-017-0299-5)
Supplement: Supplementary file 1 — Comorbidities according to ICD-10 of the pediatric OCD cohort. (DOCX 13 kb) [file 12920_2017_299_MOESM1_ESM.docx]

**Table S1:** Comorbidities according to ICD-10 of the pediatric OCD cohort

| Comorbidity according to ICD-10 | OCD (n, % of total sample) | |
| --- | --- | --- |
|  | First comorbidity | Secondary comorbidity |
| ADHD (90, 90.1, 98, 98.1, 98.5, 98.8) | 21 (17.4%) | 2 (1.6%) |
| Social behavior disorders (91, 91.1, 91.2, 91.3, 92, 92.8) | 13 (10.7%) | non |
| Developmental disorders (80, 81, 82, 83) | 1 (0.8%) | 1 (0.8%) |
| Tics (95, 95.1, 95.2, 95.8) | 8 (6.6%) | 1 (0.8%) |
| Emotional disorders (93, 93.3, 93.8) | 2 (1.6%) | 3 (2.5%) |
| Eating disorders (50, 50.1) | 5 (4.1%) | non |
| Anxiety (41) | 2 (1.6%) | 3 (2.5%) |
| Neurotic (40) | 7 (5.7%) | 2 (1.6%) |
