# Supplementary material for: High resolution chromosomal microarray analysis in paediatric obsessive-compulsive disorder
Source: BMC Med Genomics. 2017 Nov 28;10:68. doi: 10.1186/s12920-017-0299-5 (PMC5704537; doi:10.1186/s12920-017-0299-5)
Supplement: Supplementary file 2 — Rare CNVs discovered in the paediatric obsessive compulsive disorder (OCD) patients; and inheritance pattern in cases with available parents. Table S2b. Rare CNVs discovered in the population control cohort. (PDF 236 kb) [file 12920_2017_299_MOESM2_ESM.pdf]

Table S2a: Rare CNVs discovered in the paediatric obsessive compulsive disorder (OCD) patients; and inheritance pattern in cases with available parents.

Patients Code	Gender	CY-BOCS	Comorbid tics	Comorbidieties (acording to ICD-10)	CNV size (kb)	chromosomal location (hg19)	Genes within CNV	GTEx transcriptomic database (brain versus all tissue)	Inheritance	Comments
**Deletions**										
9025014001	female	16	yes	ADHD	51	2p16.3:51234059-51285498	***NRXN1***	https://gtexportal.org/home/gene/NRXN1	de-novo	
9925026001	male	13	no	ADHD	170	3p22.1:42928225-43098107	*ZNF662, KRBOX1, FAM198A*		Maternal	Absent in brother (control)
9025082001	female	15	no	Anxiety disorder	113	4p12:46952619-47065270	***GABRA4*, *GABRB1***	https://gtexportal.org/home/gene/GABRA4 https://gtexportal.org/home/gene/GABRB1	Maternal	
9025015001	male	10	no	no	210	4q28.3:139075297-139285096	***SLC7A11***, *LINC00499*	https://gtexportal.org/home/gene/SLC7A11	Maternal	
9025019001	male	23	no	ADHD	134	6p25.1:6645654-6779499	*LY86*		n.a.	
9025107001	female	22	no	no	125	6q22.31:125494942-125619539	*TPD52L1, HDDC2*		Maternal	
9025079001	male	26	yes	no	731	7q21.11:83743960-84475183	***SEMA3A***	https://gtexportal.org/home/gene/SEMA3A	Paternal	
9025101001	male	26	no	no	83	10p11.21:34672540-34755348	***PARD3***	https://gtexportal.org/home/gene/PARD3	Paternal	
9025043001	male	26	yes	no	310	12q23.1:100148198-100458394	***ANKS1B***, *UHRF1BP1L*	https://gtexportal.org/home/gene/ANKS1B	de-novo	
9025045001	female	21	no	no	297	15q21.3:53716860-54014105	***WDR72***	https://gtexportal.org/home/gene/WDR72	Maternal	
9025069001	female	19	no	no	105	15q24.2:76235495-76340932	***NRG4***	https://gtexportal.org/home/gene/NRG4	Maternal	
9025100001	male	18	no	no	1500	16p13.11:15509406-16516109	*C16orf45, KIAA0430, NDE1, MIR484,* ***MYH11***, *FOPNL,* ***ABCC1***, *ABCC6, NOMO3, MIR3179-1, MIR3179-3, MIR3179-2, MIR3180-1, MIR3180-3, MIR3180-2, PKD1P1*	https://gtexportal.org/home/gene/MYH11 https://gtexportal.org/home/gene/ABCC1	n.a.	
9025040001	female	19	no	no	100	16p13.3:6294808-6394343	***RBFOX1***		Maternal	
9025076001	female	19	no	no	373	19q13.12:37378717-37752059	*ZNF829, ZNF568, ZNF420, ZNF585A, ZNF585B, ZNF383*		Maternal	
9925012001	male	16	yes	no	54	21q21.1:22856032 22910383	***NCAM2***	https://gtexportal.org/home/gene/NCAM2	n.a.	
9925015001	male	18	no	no	279	Xq11.2:63540728-63819338	***MTMR8***	https://gtexportal.org/home/gene/MTMR8	Maternal	Brother (Anxiety) carrier of CNV as well; Mother (Anxiety)
9025093001	male	7	no	Social behavior disorder; ADHD	101	Xq27.3:142869149 142970485	***UBE2NL***	https://gtexportal.org/home/gene/UBE2NL	Maternal	
**Duplication**										
9025030001	male	29	no	no	166	1p21.2:101039885-101205680	***VCAM1***	https://gtexportal.org/home/gene/VCAM1	n.a.	
9025106001	female	22	no	no	487	1p31.1:74037092-74524344	*LRRIQ3*		n.a.	
9025016001	male	13	no	no	147	1p36.12:22984535-23131772	*C1QB,* ***EPHB2***	https://gtexportal.org/home/gene/EPHB2	Maternal	
9025094004	male	13	yes	no	437	1q21.1:145372549-145809279	*NBPF10,* ***HFE2***, *TXNIP, POLR3GL, ANKRD34A, LIX1L, RBM8A, GNRHR2, PEX11B, ITGA10, ANKRD35,* ***PIAS3***, *NUDT17, POLR3C, RNF115, CD160, PDZK1, GPR89A, GPR89C*	https://gtexportal.org/home/gene/HFE2 https://gtexportal.org/home/gene/PIAS3	Maternal	Sister (sub-threshold OCD) carrier of CNV as well; Mother (sub-threshold OCD)
9925022001	female	22	no	no	1380	3p14.2: 60835192-62214802	***FHIT***, *PTPRG*	https://gtexportal.org/home/gene/FHIT	n.a.	
9025027001	male	29	no	no	278	3p25.2:1237458512652539	*PPARG, TSEN2, LOC100129480, MKRN2,* ***RAF1***	https://gtexportal.org/home/gene/RAF1	Paternal	
9025029001	female	32	no	Social behavior disorder	58	4q13.3:72291016-72349103	***SLC4A4***	https://gtexportal.org/home/gene/SLC4A4	n.a.	
9025013001	female	28	no	no	67	4q35.1:186023710-186090501	***SLC25A4***, *KIAA1430*	https://gtexportal.org/home/gene/SLC4A4	n.a.	
9025091001	male	13	no	no	484	6p21.31:34446474-34930648	***PACSIN1***, *SPDEF, C6orf106, SNRPC, UHRF1BP1, TAF11, ANKS1A*	https://gtexportal.org/home/gene/PACSIN1	Paternal	
9925001001	male	29	no	no	300, 162 & 79	6q14.1:76417785-76718017, 76774410-76935941(two) & 17p13.2: 4309542-4388334	*SENP6,* ***MYO6***, *IMPG1, SPNS3*	https://gtexportal.org/home/gene/MYO6	Paternal	Father CNV on Chr6q14:1
9025089001	male	7	no	ADHD	246	7q11.23:72576872-72822709	*LOC100093631, GTF2IP1, NCF1B, GTF2IRD2P1, NSUN5, TRIM50, FKBP6*		Paternal	
9925007001	female	22	yes	Emotional disorder	258	7q31.33:125882371-126140769	***GRM8***	https://gtexportal.org/home/gene/GRM8	Paternal	Absent in sister (OCD)
9025036001	female	32	no	no	89	7q36.1:149318934-149408392	*ZNF767*		n.a.	
9025049001	male	22	no	Social behavior disorder	153	7q36.3:157134943-157287531	***DNAJB6***, *LGMD1E*	https://gtexportal.org/home/gene/DNAJB6	Paternal	
9025077001	male	30	yes	no	158	9p13.3:33989242-34146776	*UBAP2, DCAF12*		Maternal	
9025020001	female	28	no	ADHD	52	9q34.13:134065786-134117305	*NUP214*		n.a.	
9025067001	female	n.a.	no	Social behavior disorder	134	10p15.3:143252-277232	*ZMYND11*		n.a.	
9925035001	male	32	no	Social behaviour disorder	131	11q12.1:58547531-58678042	*GLYATL2*		n.a.	
9025073001	female	20	no	ADHD	82	13q14.11:40310481-40392725	***COG6***	https://gtexportal.org/home/gene/COG6	Paternal	
9925030001	male	16	yes	no	109	13q34:110917586-111026140	***COL4A1, COL4A2***	https://gtexportal.org/home/gene/COL4A1 https://gtexportal.org/home/gene/COL4A2	Maternal	Absent in sister (control)
9925021001	female	32	no	no	176	14q21.1:42004775-42180366	***LRFN5***	https://gtexportal.org/home/gene/LRFN5	Maternal	
9025112001	male	15	no	Social behavior disorder	100	14q23.1:58476607-58576320	*C14orf37*		n.a.	
9025082001	female	15	no	Anxiety disorder	66	18p11.32:2825037-2890695	***EMILIN2***	https://gtexportal.org/home/gene/EMILIN2	not maternal	Mother has only the deletion CNV (see above)
9025043001	male	26	yes	no	80	19p13.11:19749997-19830351	*GMIP, ATP13A1, ZNF101, ZNF14*		de-novo	
9925027001	male	14	yes	ADHD	50	19q13.2:39113548-39163701	***EIF3K, ACTN4***	https://gtexportal.org/home/gene/EIF3K https://gtexportal.org/home/gene/ACTN4	Maternal	
9025040001	female	19	no	no	73	20q12:37604490-37677926	***DHX35***	https://gtexportal.org/home/gene/DHX35	not maternal	Mother has only the deletion CNV (see above)
9025078001	female	23	no	no	313	Xp11.3:44750716-45063967	***KDM6A***, *CXorf36*	https://gtexportal.org/home/gene/KDM6A	Paternal	

Abbreviation: n.a. not available; yellow, CNVs on X chromosome; green, *de-novo* CNVs; orange, proband carrying both deletions and duplications; blue, brain/synapse related CNVs; Bold, brain/synapse related genes according to gene ontology, PubMed & GEO; Cursive, patients' codes that were analyzed in the previous publication for CNVs larger than 500 kb (McGarth et al. 2014); Patient # 90-25-079-001, with deletion on 7q21.11:83743960-84475183(hg19) of 731 kb /previously reported as 7q21.11:83580426-84291036(hg18) 711 kb (McGarth et al. 2014)

Table S2b: Rare CNVs discovered in the population control cohort

Patients Code	Gender	CNV size (kb)	chromosomal location (hg19)	Genes within CNV	GTEx transcriptomic database (brain versus all tissue)
**Deletions**					
M42335	female	328	1q44:246416575-246744498	SMYD3	
M41153	male	165	2q24.3:168028287-168193218	XIRP2	
M41047	male	475	2q24.3:168087284-168562035	XIRP2	
M40799	male	132	3p26.3:2663624-2795377	**CNTN4**	https://gtexportal.org/home/gene/CNTN4
M40405	female	272	4q28.3:134918952-135191179	PABPC4L	
M40884	male	154	6p25.1:5241358-5395099	LYRM4, FARS2	
M36037	female	127	6q11.1:62682539-62809870	**KHDRBS2**	https://gtexportal.org/home/gene/KHDRBS2
M42369	male	355	8q12.3:62517788 -62872425	ASPH, MIR4470	
M41046	female	144	10q21.3:67937347-68081418	CTNNA3	
M40457	male	507	11p14.3:24986009-25492675	**LUZP2**	https://gtexportal.org/home/gene/LUZP2
M40695	male	236	11p15.1:18099953-18335684	SAAL1, SAA3P, MRGPRX3, MRGPRX4, LOC494141, SAA4, SAA2-SAA4, SAA2, SAA1, **HPS5**	https://gtexportal.org/home/gene/HPS5
M35622	female	212	16p12.3:20321492-20533136	GP2, UMOD, PDILT, ACSM5, ACSM2A	
M41045	female	50	18p11.32:1221686-1271917	LINC00470	
M40755	male	114	20p11.23:20552561-20666840	RALGAPA2	
M40124	male	130	Xp22.11:22925103-23055103	DDX53	
**Duplications**					
M40123	female	63	1q23.3:164530910-164594194	**PBX1**	https://gtexportal.org/home/gene/PBX1
M42299	female	62	1q44:249067554-249128801	SH3BP5L, MIR3124	
M40427	male	200	2q13:114183361-114383664	CBWD2, FOXD4L1, FAM138B, WASH2P, DDX11L2, RPL23AP7	
M39991	male	164	2q36.3:229724462-229888809	PID1	
M42369	male	150	4q13.3:73089231-73239699	**ADAMTS3**	https://gtexportal.org/home/gene/ADAMTS3
M41049	female	294	5q12.1:61666795-61961098	**KIF2A** , DIMT1, IPO11, LRRC70	https://gtexportal.org/home/gene/KIF2A
M41232	male	100	5q21.1:101630286-101730438	SLCO4C1, SLCO6A1	
M35942	male	209	6p22.3:17641236-17850142	**KIF13A**	https://gtexportal.org/home/gene/KIF13A
M40235	female	67	7q31.1:107937464-108004927	**NRCAM**	https://gtexportal.org/home/gene/NRCAM
M40800	male	549 & 148	7q34:141937588-142486547, 142568933-142716851	**MOXD2P** , PRSS58, LOC730441, MTRNR2L6, PRSS1, PRSS3P2, PRSS2, TRPV6, TRPV5, C7orf34, KEL	https://gtexportal.org/home/gene/MOXD2P
M40756	male	1027	8p11.21:40708420-41735763	ZMAT4, SFRP1, GOLGA7, GINS4, AGPAT6, NKX6-3, **ANK1** , MIR486	https://gtexportal.org/home/gene/ANK1
M39992	male	194 & 137	8p22:18306031-18499620 & 15q25.3:85356528-85493541	PSD3, ALPK3, SLC28A1	
M40802	male	232 & 119	8q11.21:48660845-48892710, 8q23.3:114118874-114238099	PRKDC, MCM4, CSMD3	
M40976	female	501	8q24.12:119622994-120124241	SAMD12, SAMD12-AS1, TNFRSF11B, COLEC10	
M41099	male	204	10p11.21:35019879-35223482	**PARD3**	https://gtexportal.org/home/gene/PARD3
M42617	female	827	10p11.23:30741708-31568886	MAP3K8, LYZL2, ZNF438	
M40404	female	96	11p14.3:22316255-22411969	**SLC17A6**	https://gtexportal.org/home/gene/SLC17A6
M42621	male	118	14q11.2:23153066-23271258	OXA1L, SLC7A7	
M40460	female	518	15q11.2:22770421-23288336	TUBGCP5, CYFIP1, **NIPA2** , **NIPA1** , LOC283683, WHAMMP3, GOLGA8IP, HERC2P2, **NIPA**	https://gtexportal.org/home/gene/NIPA2 https://gtexportal.org/home/gene/NIPA1
M40904	female	838	16p11.2:29402299-30240227	LOC606724, BOLA2B, BOLA2, SLX1B, SLX1A, **SLX1B-SULT1A4** , **SLX1A-SULT1A3** , **SULT1A4** , **SULT1A3** , LOC388242, LOC613038, LOC440354, SLC7A5P1, SPN, QPRT, C16orf54, ZG16, KIF22, MAZ, PRRT2, C16orf53, MVP, CDIPT, LOC440356, SEZ6L2, ASPHD1, KCTD13, TMEM219, TAOK2, HIRIP3, INO80E, DOC2A, C16orf92, FAM57B, ALDOA, PPP4C, TBX6, YPEL3, GDPD3, MAPK3, CORO1A, LOC613037	https://gtexportal.org/home/gene/SULT1A4 https://gtexportal.org/home/gene/SULT1A3
M40468	female	128	17q12:36410719-36538232	LOC440434, MRPL45, **GPR179** , **SOCS7**	https://gtexportal.org/home/gene/GPR179 https://gtexportal.org/home/gene/SOCS7
M41159	male	130	18q21.1:44283553-44413968	**PIAS2** , ST8SIA5	https://gtexportal.org/home/gene/PIAS2
M35999	female	529	20p11.23:18621787-19150529	DTD1, HSPC072, LOC100270804, C20orf78, C20orf79	
M40787	male	138	22q13.2:43038823-43176393	CYB5R3, A4GALT	
M41044	female	91	Xq26.3:134208593-134299774	LINC00087	

Abbreviation: n.a. not available; yellow, CNVs on X chromosome; orange, proband carrying both deletions and duplications; blue, synaptic/ brain CNVs; Bold, the gene involved in synaptic /brain related
