# Supplementary material for: High resolution chromosomal microarray analysis in paediatric obsessive-compulsive disorder
Source: BMC Med Genomics. 2017 Nov 28;10:68. doi: 10.1186/s12920-017-0299-5 (PMC5704537; doi:10.1186/s12920-017-0299-5)
Supplement: Supplementary file 3 — According to Pathway Studio. Table S3b. According to DAVID. (PDF 115 kb) [file 12920_2017_299_MOESM3_ESM.pdf]

**Table S3a-** According to Pathway Studio

**Enriched pathways/Groups according to GO for deletions and duplications CNVs in EO-OCD according to Pathway Studio (cutoff p<0.005)**

| Name | # of Entities | Expanded # of Entities | Overlap | Percent Overlap | Overlapping Entities | p-value | Jaccard similarity | Hit type |
|---|---|---|---|---|---|---|---|---|
| Secreted proteins | 7196 | 12585 | 56 | 0 | RAF1;PARD3;ANKS1B;DNAJB6;PACSIN1;ACTN4;MYH11;NRXN1;TXNIP;CD160;EMILIN2;GRM8;FKBP6;TRIM50;SLC25A4;NUP214;VCAM1;UBE2NL;ATP13A1;RNF115;ANKS1A;SEMA3A;ITGA10;NCAM2;SPDEF;COL4A1;COL4A2;SLC7A11;C14orf37;FAM198A;CXorf36;PPARG;GNRHR2;EPHB2;GABRA4;NRG4;HFE2;PTPRG;MIR484;MYO6;FHIT;ABCC6;SLC4A4;PDZK1;ABCC1;C1QB;NUDT17;IMPG1;EIF3K;SNRPC;NSUN5;POLR3GL;POLR3C;ANKRD35;MKRN2;MTMR8 | 9.25E-13 | 0.004 | Pathway Studio Ontology |
| KRAB domain | 343 | 343 | 10 | 2 | ZNF662;ZNF585A;KRBOX1;ZNF383;ZNF585B;ZNF829;ZNF101;ZNF568;ZNF420;ZNF14 | 7.43E-10 | 0.022 | Pathway Studio Ontology |
| krueppel C2H2-type zinc-finger protein family | 501 | 501 | 8 | 1 | ZNF662;ZNF585A;ZNF383;ZNF585B;ZNF829;ZNF101;ZNF568;ZNF14 | 3.69E-06 | 0.013 | Pathway Studio Ontology |
| axon guidance | 385 | 385 | 9 | 2 | RAF1;EPHB2;MYH11;SEMA3A;ITGA10;HFE2;NRXN1;COL4A1;COL4A2 | 2.78E-05 | 0.018 | biological_process |
| KLF | 325 | 325 | 6 | 1 | ZNF383;ZNF585B;ZNF101;ZNF568;ZNF420;ZNF14 | 2.85E-05 | 0.014 | Pathway Studio Ontology |
| collagen type IV | 6 | 6 | 2 | 33 | COL4A1;COL4A2 | 4.76E-05 | 0.017 | Pathway Studio Ontology |
| axonal fasciculation | 19 | 19 | 3 | 15 | SEMA3A;NCAM2;EPHB2 | 6.03E-05 | 0.023 | biological_process |
| TC 3.A.1.208 | 7 | 7 | 2 | 28 | ABCC6;ABCC1 | 6.66E-05 | 0.017 | Pathway Studio Ontology |
| sphingolipid transporter activity | 4 | 4 | 2 | 50 | SPNS3;ABCC1 | 9.77E-05 | 0.018 | molecular_function |
| Proteins Involved in Pathogenesis of Melanoma | 245 | 264 | 6 | 2 | ITGA10;RAF1;VCAM1;TXNIP;PIAS3;PPARG | 0.0001 | 0.016 | Disease Collections |
| drug transporter | 10 | 10 | 2 | 20 | ABCC6;ABCC1 | 0.0001 | 0.017 | Pathway Studio Ontology |
| RNA polymerase III | 10 | 10 | 2 | 20 | POLR3C;POLR3GL | 0.0001 | 0.017 | Pathway Studio Ontology |
| extracellular matrix constituent conferring elasticity | 5 | 5 | 2 | 40 | EMILIN2;COL4A1 | 0.0002 | 0.017 | molecular_function |
| peroxisome proliferator activated receptor signaling pathway | 6 | 6 | 2 | 33 | PPARG;ACTN4 | 0.0002 | 0.017 | biological_process |
| peptide synthase | 193 | 193 | 4 | 2 | UBE2NL;TRIM50;RNF115;MKRN2 | 0.0004 | 0.013 | Pathway Studio Ontology |
| chorio-allantoic fusion | 8 | 8 | 2 | 25 | DNAJB6;VCAM1 | 0.0005 | 0.017 | biological_process |
| collagen type IV trimer | 8 | 8 | 2 | 25 | COL4A1;COL4A2 | 0.0005 | 0.017 | cellular_component |
| collagen trimer | 91 | 91 | 4 | 4 | EMILIN2;C1QB;COL4A1;COL4A2 | 0.0005 | 0.020 | cellular_component |
| extracellular matrix polymerization proteins | 88 | 88 | 3 | 3 | EMILIN2;COL4A1;COL4A2 | 0.0006 | 0.015 | Pathway Studio Ontology |
| ABC transmembrane type-1 domain | 20 | 20 | 2 | 10 | ABCC6;ABCC1 | 0.0006 | 0.015 | Pathway Studio Ontology |
| synapse | 466 | 466 | 8 | 1 | EPHB2;GABRA4;GABRB1;NRXN1;MYO6;PIAS3;ANKS1B;PACSIN1 | 0.0006 | 0.014 | cellular_component |
| Role of Protein Kinase C in Diabetic Microangiopathy | 23 | 55 | 3 | 5 | COL4A1;COL4A2;RAF1 | 0.0006 | 0.018 | Disease Collections |
| ABC transporter domain | 22 | 22 | 2 | 9 | ABCC6;ABCC1 | 0.0007 | 0.015 | Pathway Studio Ontology |
| Peripheral Tissue Microangiopathy in Insulin Resistance | 87 | 139 | 4 | 2 | RAF1;VCAM1;COL4A1;COL4A2 | 0.0009 | 0.016 | Disease Collections |
| hydrolase acting on acid anhydrides in phosphorous-containing anhydrides | 25 | 25 | 2 | 7 | NUDT17;DHX35 | 0.0009 | 0.015 | Pathway Studio Ontology |
| Glioma Invasion Signaling | 91 | 148 | 4 | 2 | RAF1;EPHB2;COL4A1;COL4A2 | 0.0011 | 0.016 | Disease Collections |
| PID domain | 28 | 28 | 2 | 7 | ANKS1B;ANKS1A | 0.0012 | 0.014 | Pathway Studio Ontology |
| Synovial Fibroblast Proliferation in Rheumatoid Arthritis | 64 | 151 | 4 | 2 | RAF1;ACTN4;COL4A1;COL4A2 | 0.0012 | 0.015 | Disease Collections |
| C1q domain | 30 | 30 | 2 | 6 | EMILIN2;C1QB | 0.0013 | 0.014 | Pathway Studio Ontology |
| transporter activity | 299 | 299 | 6 | 2 | ABCC6;ABCC1;SLC4A4;PDZK1;SLC25A4;NUP214 | 0.0014 | 0.015 | molecular_function |
| centrosome localization | 14 | 14 | 2 | 14 | NDE1;PARD3 | 0.0014 | 0.016 | biological_process |
| transmembrane transport | 805 | 805 | 10 | 1 | RAF1;GABRA4;GABRB1;ABCC6;ABCC1;SLC4A4;SPNS3;SLC25A4;NUP214;SLC7A11 | 0.0016 | 0.011 | biological_process |
| neuron cell-cell adhesion | 15 | 15 | 2 | 13 | NCAM2;NRXN1 | 0.0017 | 0.016 | biological_process |
| cystine transporter | 1 | 1 | 1 | 100 | SLC7A11 | 0.0018 | 0.009 | Pathway Studio Ontology |
| TC 8.A.15.1 | 1 | 1 | 1 | 100 | PIAS3 | 0.0018 | 0.009 | Pathway Studio Ontology |
| pseudopodium | 16 | 16 | 2 | 12 | RAF1;ACTN4 | 0.0019 | 0.016 | cellular_component |
| negative regulation of epithelial cell migration | 17 | 17 | 2 | 11 | SEMA3A;PTPRG | 0.0021 | 0.016 | biological_process |
| extracellular matrix organization | 329 | 329 | 6 | 1 | ITGA10;NRXN1;VCAM1;DNAJB6;COL4A1;COL4A2 | 0.0023 | 0.014 | biological_process |
| transcription elongation from RNA polymerase III promoter | 18 | 18 | 2 | 11 | POLR3C;POLR3GL | 0.0024 | 0.016 | biological_process |
| termination of RNA polymerase III transcription | 18 | 18 | 2 | 11 | POLR3C;POLR3GL | 0.0024 | 0.016 | biological_process |
| establishment of protein localization to plasma membrane | 19 | 19 | 2 | 10 | PDZK1;PACSIN1 | 0.0027 | 0.016 | biological_process |
| RNA polymerase III activity | 19 | 19 | 2 | 10 | POLR3C;POLR3GL | 0.0027 | 0.016 | molecular_function |
| DNA-directed RNA polymerase III complex | 19 | 19 | 2 | 10 | POLR3C;POLR3GL | 0.0027 | 0.016 | cellular_component |
| GABA-A receptor activity | 20 | 20 | 2 | 10 | GABRA4;GABRB1 | 0.0030 | 0.015 | molecular_function |
| axonal growth cone | 20 | 20 | 2 | 10 | PARD3;NRXN1 | 0.0030 | 0.015 | cellular_component |
| heart development | 238 | 240 | 5 | 2 | RAF1;SLC25A4;VCAM1;KDM6A;PPARG | 0.0030 | 0.014 | biological_process |
| RRM (RNA recognition motif) domain | 159 | 159 | 3 | 1 | RBM8A;RBFOX1;KIAA0430 | 0.0030 | 0.011 | Pathway Studio Ontology |
| extracellular matrix structural constituent | 73 | 73 | 3 | 4 | COL4A1;COL4A2;IMPG1 | 0.0033 | 0.016 | molecular_function |
| muscle myosin | 2 | 2 | 1 | 50 | MYH11 | 0.0036 | 0.009 | Pathway Studio Ontology |
| PAR3 family | 2 | 2 | 1 | 50 | PARD3 | 0.0036 | 0.009 | Pathway Studio Ontology |
| bis(5'-adenosyl)-triphosphatase | 2 | 2 | 1 | 50 | FHIT | 0.0036 | 0.009 | Pathway Studio Ontology |
| GNRH receptor | 2 | 2 | 1 | 50 | GNRHR2 | 0.0036 | 0.009 | Pathway Studio Ontology |
| HIT domain | 2 | 2 | 1 | 50 | FHIT | 0.0036 | 0.009 | Pathway Studio Ontology |
| receptor phosphatase class 5 family | 2 | 2 | 1 | 50 | PTPRG | 0.0036 | 0.009 | Pathway Studio Ontology |
| alpha-carbonic anhydrase domain | 2 | 2 | 1 | 50 | PTPRG | 0.0036 | 0.009 | Pathway Studio Ontology |
| DIA1 family | 2 | 2 | 1 | 50 | CXorf36 | 0.0036 | 0.009 | Pathway Studio Ontology |
| FAM198 family | 2 | 2 | 1 | 50 | FAM198A | 0.0036 | 0.009 | Pathway Studio Ontology |
| tRNA-intron endonuclease family | 2 | 2 | 1 | 50 | TSEN2 | 0.0036 | 0.009 | Pathway Studio Ontology |
| eukaryotic RPC7 RNA polymerase subunit family | 2 | 2 | 1 | 50 | POLR3GL | 0.0036 | 0.009 | Pathway Studio Ontology |
| LIX1 family | 2 | 2 | 1 | 50 | LIX1L | 0.0036 | 0.009 | Pathway Studio Ontology |
| alpha10beta1 integrin | 2 | 2 | 1 | 50 | ITGA10 | 0.0036 | 0.009 | Pathway Studio Ontology |
| tRNA-intron endonuclease | 2 | 2 | 1 | 50 | TSEN2 | 0.0036 | 0.009 | Pathway Studio Ontology |
| Proteins Involved in Pathogenesis of Polycystic Ovary Syndrome | 100 | 100 | 3 | 2 | RAF1;PPARG;VCAM1 | 0.0036 | 0.014 | Disease Collections |
| collagen-like domain | 50 | 50 | 2 | 3 | EMILIN2;C1QB | 0.0037 | 0.013 | Pathway Studio Ontology |
| Biofluids assayable substances | 3881 | 8943 | 27 | 0 | RAF1;DNAJB6;PACSIN1;ACTN4;MYH11;TXNIP;SLC25A4;VCAM1;ABCC1;ATP13A1;C1QB;SEMA3A;ITGA10;NCAM2;COL4A1;COL4A2;C14orf37;EIF3K;PPARG;NSUN5;EPHB2;GABRA4;NRG4;HFE2;MTMR8;MIR484;FHIT | 0.0038 | 0.003 | Pathway Studio Ontology |
| regulation of anion transport | 23 | 23 | 2 | 8 | GPR89A;PDZK1 | 0.0039 | 0.015 | biological_process |
| tRNA Transcription and Processing | 20 | 78 | 3 | 3 | POLR3GL;POLR3C;TSEN2 | 0.0040 | 0.016 | Cell Process Pathways |
| death-inducing signaling complex assembly | 1 | 1 | 1 | 100 | RAF1 | 0.0041 | 0.009 | biological_process |
| regulation of ephrin receptor signaling pathway | 1 | 1 | 1 | 100 | ANKS1A | 0.0041 | 0.009 | biological_process |
| negative regulation of peptide hormone secretion | 1 | 1 | 1 | 100 | PPARG | 0.0041 | 0.009 | biological_process |
| negative regulation of cell motility involved in cerebral cortex radial glia guided migration | 1 | 1 | 1 | 100 | C16orf45 | 0.0041 | 0.009 | biological_process |
| neural crest cell migration involved in sympathetic nervous system development | 1 | 1 | 1 | 100 | SEMA3A | 0.0041 | 0.009 | biological_process |
| protein complex assembly involved in synapse maturation | 1 | 1 | 1 | 100 | NRXN1 | 0.0041 | 0.009 | biological_process |
| diadenosine triphosphate catabolic process | 1 | 1 | 1 | 100 | FHIT | 0.0041 | 0.009 | biological_process |
| cystine:glutamate antiporter activity | 1 | 1 | 1 | 100 | SLC7A11 | 0.0041 | 0.009 | molecular_function |
| glutathione transmembrane transport | 1 | 1 | 1 | 100 | ABCC1 | 0.0041 | 0.009 | biological_process |
| glutathione transmembrane transporter activity | 1 | 1 | 1 | 100 | ABCC1 | 0.0041 | 0.009 | molecular_function |
| glutathione S-conjugate-exporting ATPase activity | 1 | 1 | 1 | 100 | ABCC1 | 0.0041 | 0.009 | molecular_function |
| low-density lipoprotein particle receptor biosynthetic process | 1 | 1 | 1 | 100 | PPARG | 0.0041 | 0.009 | biological_process |
| peptidyl-proline modification | 1 | 1 | 1 | 100 | FKBP6 | 0.0041 | 0.009 | biological_process |

| | | | | | | | | |
|---|---|---|---|---|---|---|---|---|
| protein modification by small protein removal | 1 | 1 | 1 | 100 | SENP6 | 0.0041 | 0.009 | biological_process |
| negative regulation of pancreatic stellate cell proliferation | 1 | 1 | 1 | 100 | PPARG | 0.0041 | 0.009 | biological_process |
| negative regulation of cellular response to insulin stimulus | 1 | 1 | 1 | 100 | PPARG | 0.0041 | 0.009 | biological_process |
| cellular response to tumor cell | 1 | 1 | 1 | 100 | TXNIP | 0.0041 | 0.009 | biological_process |
| regulation of cholesterol transporter activity | 1 | 1 | 1 | 100 | PPARG | 0.0041 | 0.009 | biological_process |
| minus-end directed microfilament motor activity | 1 | 1 | 1 | 100 | MYO6 | 0.0041 | 0.009 | molecular_function |
| response to metformin | 1 | 1 | 1 | 100 | PPARG | 0.0041 | 0.009 | biological_process |
| protein localization to bicellular tight junction | 1 | 1 | 1 | 100 | ACTN4 | 0.0041 | 0.009 | biological_process |
| scavenger receptor binding | 1 | 1 | 1 | 100 | PDZK1 | 0.0041 | 0.009 | molecular_function |
| ankyrin repeat | 179 | 179 | 3 | 1 | ANKRD35;ANKS1B;ANKS1A | 0.0042 | 0.010 | Pathway Studio Ontology |
| anchored component of membrane | 161 | 161 | 4 | 2 | CD160;NCAM2;HFE2;VCAM1 | 0.0043 | 0.015 | cellular_component |
| Ig-like C2-type (immunoglobulin-like) domain | 182 | 182 | 3 | 1 | SEMA3A;NCAM2;VCAM1 | 0.0044 | 0.010 | Pathway Studio Ontology |
| SAM (sterile alpha motif) domain | 57 | 57 | 2 | 3 | ANKS1B;ANKS1A | 0.0048 | 0.012 | Pathway Studio Ontology |
| WD repeat DCAF12 family | 3 | 3 | 1 | 33 | DCAF12 | 0.0054 | 0.009 | Pathway Studio Ontology |
| unconventional myosin | 3 | 3 | 1 | 33 | MYO6 | 0.0054 | 0.009 | Pathway Studio Ontology |
| Raf family | 3 | 3 | 1 | 33 | RAF1 | 0.0054 | 0.009 | Pathway Studio Ontology |
| group III mGluR | 3 | 3 | 1 | 33 | GRM8 | 0.0054 | 0.009 | Pathway Studio Ontology |
| UTX family | 3 | 3 | 1 | 33 | KDM6A | 0.0054 | 0.009 | Pathway Studio Ontology |
| PACSIN family | 3 | 3 | 1 | 33 | PACSIN1 | 0.0054 | 0.009 | Pathway Studio Ontology |
| ANKRD34 family | 3 | 3 | 1 | 33 | ANKRD34A | 0.0054 | 0.009 | Pathway Studio Ontology |
| major facilitator (TC 2.A.1) superfamily. Spinster (TC 2.A.1.49) family | 3 | 3 | 1 | 33 | SPNS3 | 0.0054 | 0.009 | Pathway Studio Ontology |
| repulsive guidance molecule (RGM) family | 3 | 3 | 1 | 33 | HFE2 | 0.0054 | 0.009 | Pathway Studio Ontology |
| peroxin-11 family | 3 | 3 | 1 | 33 | PEX11B | 0.0054 | 0.009 | Pathway Studio Ontology |
| sialoglycoprotein | 3 | 3 | 1 | 33 | C14orf37 | 0.0054 | 0.009 | Pathway Studio Ontology |
| PPAR | 3 | 3 | 1 | 33 | PPARG | 0.0054 | 0.009 | Pathway Studio Ontology |
| U1 snRNP | 3 | 3 | 1 | 33 | SNRPC | 0.0054 | 0.009 | Pathway Studio Ontology |
| peroxisome division proteins | 3 | 3 | 1 | 33 | PEX11B | 0.0054 | 0.009 | Pathway Studio Ontology |
| HTH OST-type domain | 3 | 3 | 1 | 33 | KIAA0430 | 0.0054 | 0.009 | Pathway Studio Ontology |
| HD domain | 3 | 3 | 1 | 33 | HDDC2 | 0.0054 | 0.009 | Pathway Studio Ontology |
| positive regulation of synapse assembly | 28 | 28 | 2 | 7 | EPHB2;NRXN1 | 0.0058 | 0.014 | biological_process |
| protein sumoylation | 28 | 28 | 2 | 7 | PIAS3;SENP6 | 0.0058 | 0.014 | biological_process |
| spindle assembly proteins | 65 | 65 | 2 | 3 | NDE1;PARD3 | 0.0062 | 0.011 | Pathway Studio Ontology |
| gamma-aminobutyric acid signaling pathway | 29 | 29 | 2 | 6 | GABRA4;GABRB1 | 0.0062 | 0.014 | biological_process |

**Enriched pathways/Groups according to GO for deletions and duplications CNVs in control according to Pathway Studio (cutoff p<0.005)**

| Name | # of Entities | Expanded # of Entities | Overlap | Percent Overlap | Overlapping Entities | p-value | Jaccard similarity | Hit type |
|---|---|---|---|---|---|---|---|---|
| SAA family | 4 | 4 | 3 | 75 | SAA1;SAA2;SAA4 | 3.99E-08 | 0.021 | Pathway Studio Ontology |
| high-density lipoprotein particle | 29 | 29 | 5 | 17 | HPS5;SAA1;SAA2;SAA2-SAA4;SAA4 | 2.27E-07 | 0.030 | cellular_component |
| trypsin | 7 | 7 | 3 | 42 | PRSS1;PRSS2;PRSS58 | 3.48E-07 | 0.021 | Pathway Studio Ontology |
| SLX1 family | 2 | 2 | 2 | 100 | SLX1A;SLX1B | 4.68E-06 | 0.014 | Pathway Studio Ontology |
| SLX1-type zinc finger | 2 | 2 | 2 | 100 | SLX1A;SLX1B | 4.68E-06 | 0.014 | Pathway Studio Ontology |
| acute-phase response | 53 | 53 | 5 | 9 | HPS5;SAA1;SAA2;SAA2-SAA4;SAA4 | 5.01E-06 | 0.027 | biological_process |
| aspartyl/asparaginyl beta-hydroxylase family | 4 | 4 | 2 | 50 | ASPHD1;ASPH | 2.80E-05 | 0.014 | Pathway Studio Ontology |
| TC 2.A.7.25 | 4 | 4 | 2 | 50 | NIPA1;NIPA2 | 2.80E-05 | 0.014 | Pathway Studio Ontology |
| Slx1-Slx4 complex | 3 | 3 | 2 | 66 | SLX1A;SLX1B | 6.54E-05 | 0.014 | cellular_component |
| vanilloid receptor | 6 | 6 | 2 | 33 | TRPV6;TRPV5 | 6.97E-05 | 0.014 | Pathway Studio Ontology |
| aryl sulfotransferase | 6 | 6 | 2 | 33 | SULT1A3;SULT1A4 | 6.97E-05 | 0.014 | Pathway Studio Ontology |
| butyrate-CoA ligase | 8 | 8 | 2 | 25 | ACSM5;ACSM2A | 0.0001 | 0.014 | Pathway Studio Ontology |
| peptidyl-amino acid modification | 4 | 4 | 2 | 50 | ASPHD1;ASPH | 0.0001 | 0.014 | biological_process |
| DNA double-strand break processing involved in repair via single-strand annealing | 5 | 5 | 2 | 40 | SLX1A;SLX1B | 0.0002 | 0.014 | biological_process |
| blood group Ag | 12 | 12 | 2 | 16 | A4GALT;KEL | 0.0003 | 0.013 | Pathway Studio Ontology |
| ZP domain | 12 | 12 | 2 | 16 | GP2;UMOD | 0.0003 | 0.013 | Pathway Studio Ontology |
| 5'-flap endonuclease activity | 6 | 6 | 2 | 33 | SLX1A;SLX1B | 0.0003 | 0.014 | molecular_function |
| spindle assembly proteins | 65 | 65 | 3 | 4 | TUBGCP5;PARD3;KIF22 | 0.0004 | 0.015 | Pathway Studio Ontology |
| Paneth Cell Function in Crohn's Disease | 27 | 60 | 3 | 5 | PRSS2;PRSS58;PRSS1 | 0.0004 | 0.015 | Disease Collections |
| non-voltage Ca++ import proteins | 16 | 16 | 2 | 12 | TRPV6;TRPV5 | 0.0006 | 0.013 | Pathway Studio Ontology |
| peptidase S1 family | 73 | 73 | 3 | 4 | PRSS1;PRSS2;PRSS58 | 0.0006 | 0.014 | Pathway Studio Ontology |
| crossover junction endodeoxyribonuclease activity | 8 | 8 | 2 | 25 | SLX1A;SLX1B | 0.0006 | 0.014 | molecular_function |
| aryl sulfotransferase activity | 8 | 8 | 2 | 25 | SULT1A4;SULT1A3 | 0.0006 | 0.014 | molecular_function |
| butyrate-CoA ligase activity | 8 | 8 | 2 | 25 | ACSM2A;ACSM5 | 0.0006 | 0.014 | molecular_function |
| MAP kinase kinase kinase activity | 37 | 37 | 3 | 8 | MAP3K8;TAOK2;ALPK3 | 0.0007 | 0.017 | molecular_function |
| DNA replication | 157 | 157 | 5 | 3 | GINS4;MCM4;KCTD13;DTD1;INO80E | 0.0009 | 0.017 | biological_process |
| peptidase S1 domain | 91 | 91 | 3 | 3 | PRSS1;PRSS2;PRSS58 | 0.0011 | 0.013 | Pathway Studio Ontology |
| regulation of calcium ion-dependent exocytosis | 11 | 11 | 2 | 18 | DOC2A;TRPV6 | 0.0012 | 0.013 | biological_process |
| glycosphingolipid biosynthetic process | 11 | 11 | 2 | 18 | A4GALT;ST8SIA5 | 0.0012 | 0.013 | biological_process |
| DNA-dependent protein kinase activity | 13 | 13 | 2 | 15 | PRKDC;ALPK3 | 0.0016 | 0.013 | molecular_function |
| catecholamine metabolic process | 13 | 13 | 2 | 15 | SULT1A3;SULT1A4 | 0.0016 | 0.013 | biological_process |
| NF-kappaB-inducing kinase activity | 14 | 14 | 2 | 14 | PPP4C;ALPK3 | 0.0018 | 0.013 | molecular_function |
| magnesium ion transmembrane transporter activity | 14 | 14 | 2 | 14 | NIPA1;NIPA2 | 0.0018 | 0.013 | molecular_function |
| retroviral 3' processing activity | 54 | 54 | 3 | 5 | GDPD3;SLX1B;DTD1 | 0.0020 | 0.016 | molecular_function |
| T-G mismatch-specific endonuclease activity | 54 | 54 | 3 | 5 | GDPD3;SLX1B;DTD1 | 0.0020 | 0.016 | molecular_function |
| actin cytoskeleton organization | 192 | 192 | 5 | 2 | CORO1A;SFRP1;KCTD13;XIRP2;TAOK2 | 0.0021 | 0.015 | biological_process |
| rRNA adenine N(6)-methyltransferase family | 1 | 1 | 1 | 100 | DIMT1 | 0.0022 | 0.007 | Pathway Studio Ontology |
| DNA-PK | 1 | 1 | 1 | 100 | PRKDC | 0.0022 | 0.007 | Pathway Studio Ontology |
| TC 2.A.9.1 | 1 | 1 | 1 | 100 | OXA1L | 0.0022 | 0.007 | Pathway Studio Ontology |
| lactosylceramide 4-alpha-galactosyltransferase | 1 | 1 | 1 | 100 | A4GALT | 0.0022 | 0.007 | Pathway Studio Ontology |
| PP-4 (PP-X) family | 1 | 1 | 1 | 100 | PPP4C | 0.0022 | 0.007 | Pathway Studio Ontology |
| protein phosphatase 4 | 1 | 1 | 1 | 100 | PPP4C | 0.0022 | 0.007 | Pathway Studio Ontology |
| CDP-diacylglycerol-inositol 3-phosphatidyltransferase | 1 | 1 | 1 | 100 | CDIPT | 0.0022 | 0.007 | Pathway Studio Ontology |
| nicotinate-nucleotide diphosphorylase (carboxylating) | 1 | 1 | 1 | 100 | QPRT | 0.0022 | 0.007 | Pathway Studio Ontology |
| 18S rRNA (adenine1779-N6/adenine1780-N6)-dimethyltransferase | 1 | 1 | 1 | 100 | DIMT1 | 0.0022 | 0.007 | Pathway Studio Ontology |
| kinesin complex | 57 | 57 | 3 | 5 | KIF13A;KIF22;KIF2A | 0.0025 | 0.015 | cellular_component |
| negative regulation of androgen receptor signaling pathway | 16 | 16 | 2 | 12 | SFRP1;PIAS2 | 0.0025 | 0.013 | biological_process |
| magnesium ion transport | 16 | 16 | 2 | 12 | NIPA1;NIPA2 | 0.0025 | 0.013 | biological_process |
| phosphoric ester hydrolase activity | 61 | 61 | 3 | 4 | GDPD3;SLX1B;DTD1 | 0.0028 | 0.015 | molecular_function |
| regulation of ossification | 17 | 17 | 2 | 11 | SFRP1;PBX1 | 0.0028 | 0.013 | biological_process |
| protein complex assembly | 132 | 132 | 4 | 3 | MAPK3;SLC7A7;PARD3;OXA1L | 0.0035 | 0.015 | biological_process |
| axonal fasciculation | 19 | 19 | 2 | 10 | CNTN4;NRCAM | 0.0035 | 0.013 | biological_process |
| activation of MAPKK activity | 65 | 65 | 3 | 4 | MAP3K8;MAPK3;TAOK2 | 0.0036 | 0.015 | biological_process |
| early endosome | 217 | 217 | 5 | 2 | CORO1A;MAPK3;NIPA1;NIPA2;SLC17A6 | 0.0036 | 0.014 | cellular_component |
| readthrough transcript | 142 | 142 | 3 | 2 | SLX1A-SULT1A3;SLX1B-SULT1A4;SAA2-SAA4 | 0.0037 | 0.011 | Pathway Studio Ontology |
| WNT Inhibition by DKK1 in Osteoblast in Multiple Myeloma | 16 | 38 | 2 | 5 | SFRP1;TNFRSF11B | 0.0041 | 0.011 | Disease Collections |
| regulation of cell shape | 140 | 140 | 4 | 2 | CORO1A;CYFIP1;TAOK2;ALDOA | 0.0043 | 0.014 | biological_process |
| FDX-ACB domain | 2 | 2 | 1 | 50 | FARS2 | 0.0043 | 0.007 | Pathway Studio Ontology |
| GPCR | 2 | 2 | 1 | 50 | MRGPRX3 | 0.0043 | 0.007 | Pathway Studio Ontology |

| | | | | | | | | |
|---|---|---|---|---|---|---|---|---|
| PAR3 family | 2 | 2 | 1 | 50 | PARD3 | 0.0043 | 0.007 | Pathway Studio Ontology |
| peptide-aspartate beta-dioxygenase | 2 | 2 | 1 | 50 | ASPH | 0.0043 | 0.007 | Pathway Studio Ontology |
| RANKL receptor | 2 | 2 | 1 | 50 | TNFRSF11B | 0.0043 | 0.007 | Pathway Studio Ontology |
| ERK1/2 | 2 | 2 | 1 | 50 | MAPK3 | 0.0043 | 0.007 | Pathway Studio Ontology |
| VGLUT subfamily | 2 | 2 | 1 | 50 | SLC17A6 | 0.0043 | 0.007 | Pathway Studio Ontology |
| DTD family | 2 | 2 | 1 | 50 | DTD1 | 0.0043 | 0.007 | Pathway Studio Ontology |
| COLEC10/COLEC11 family | 2 | 2 | 1 | 50 | COLEC10 | 0.0043 | 0.007 | Pathway Studio Ontology |
| ERF4 family | 2 | 2 | 1 | 50 | GOLGA7 | 0.0043 | 0.007 | Pathway Studio Ontology |
| OXA1/ALB3/YidC family | 2 | 2 | 1 | 50 | OXA1L | 0.0043 | 0.007 | Pathway Studio Ontology |
| glycosyltransferase 32 family | 2 | 2 | 1 | 50 | A4GALT | 0.0043 | 0.007 | Pathway Studio Ontology |
| SH3BP5 family | 2 | 2 | 1 | 50 | SH3BP5L | 0.0043 | 0.007 | Pathway Studio Ontology |
| CYFIP family | 2 | 2 | 1 | 50 | CYFIP1 | 0.0043 | 0.007 | Pathway Studio Ontology |
| Xin family | 2 | 2 | 1 | 50 | XIRP2 | 0.0043 | 0.007 | Pathway Studio Ontology |
| jacalin lectin family | 2 | 2 | 1 | 50 | ZG16 | 0.0043 | 0.007 | Pathway Studio Ontology |
| Mg2+ transporter | 2 | 2 | 1 | 50 | NIPA1 | 0.0043 | 0.007 | Pathway Studio Ontology |
| Xin repeat | 2 | 2 | 1 | 50 | XIRP2 | 0.0043 | 0.007 | Pathway Studio Ontology |
| crossover junction endodeoxyribonuclease | 2 | 2 | 1 | 50 | SLX1A | 0.0043 | 0.007 | Pathway Studio Ontology |
| 18S rRNA (adenine(1779)-N(6)-adenine(1780)-N(6))-dimethyltransferase activity | 1 | 1 | 1 | 100 | DIMT1 | 0.0046 | 0.007 | molecular_function |
| peptide-aspartate beta-dioxygenase activity | 1 | 1 | 1 | 100 | ASPH | 0.0046 | 0.007 | molecular_function |
| lactosylceramide 4-alpha-galactosyltransferase activity | 1 | 1 | 1 | 100 | A4GALT | 0.0046 | 0.007 | molecular_function |
| CDP-diacylglycerol-inositol 3-phosphatidyltransferase activity | 1 | 1 | 1 | 100 | CDIPT | 0.0046 | 0.007 | molecular_function |
| stromal-epithelial cell signaling involved in prostate gland development | 1 | 1 | 1 | 100 | SFRP1 | 0.0047 | 0.007 | biological_process |
| signal transduction involved in mitotic G1 DNA damage checkpoint | 1 | 1 | 1 | 100 | PRKDC | 0.0047 | 0.007 | biological_process |
| regulation of mitochondrial fusion | 1 | 1 | 1 | 100 | PID1 | 0.0047 | 0.007 | biological_process |
| negative regulation of canonical Wnt signaling pathway involved in controlling type B pancreatic cell proliferation | 1 | 1 | 1 | 100 | SFRP1 | 0.0047 | 0.007 | biological_process |
| glandular epithelial cell maturation | 1 | 1 | 1 | 100 | AGPAT6 | 0.0047 | 0.007 | biological_process |
| thymocyte aggregation | 1 | 1 | 1 | 100 | SPN | 0.0047 | 0.007 | biological_process |
| positive regulation of interleukin-1 secretion | 1 | 1 | 1 | 100 | SAA1 | 0.0047 | 0.007 | biological_process |
| quinolinate catabolic process | 1 | 1 | 1 | 100 | QPRT | 0.0047 | 0.007 | biological_process |
| CDP-diacylglycerol metabolic process | 1 | 1 | 1 | 100 | CDIPT | 0.0047 | 0.007 | biological_process |
| globoside biosynthetic process | 1 | 1 | 1 | 100 | A4GALT | 0.0047 | 0.007 | biological_process |
| regulation of protein depolymerization | 1 | 1 | 1 | 100 | ASPH | 0.0047 | 0.007 | biological_process |
| convergent extension involved in somitogenesis | 1 | 1 | 1 | 100 | SFRP1 | 0.0047 | 0.007 | biological_process |
| antigen transcytosis by M cells in mucosal-associated lymphoid tissue | 1 | 1 | 1 | 100 | GP2 | 0.0047 | 0.007 | biological_process |
| protein activation cascade | 1 | 1 | 1 | 100 | MVP | 0.0047 | 0.007 | biological_process |
| negative regulation of signaling | 1 | 1 | 1 | 100 | MVP | 0.0047 | 0.007 | biological_process |
| negative regulation of response to gamma radiation | 1 | 1 | 1 | 100 | PRKDC | 0.0047 | 0.007 | biological_process |
| mitochondrial respiratory chain complex I biogenesis | 1 | 1 | 1 | 100 | OXA1L | 0.0047 | 0.007 | biological_process |
| menstrual cycle phase | 1 | 1 | 1 | 100 | SFRP1 | 0.0047 | 0.007 | biological_process |
| negative regulation of apolipoprotein binding | 1 | 1 | 1 | 100 | MAPK3 | 0.0047 | 0.007 | biological_process |
| M band | 22 | 22 | 2 | 9 | ANK1;ALDOA | 0.0048 | 0.013 | cellular_component |

**Abbreviations:**

**Yellow**, pathways and groups of brain/synapse related genes;

**Red**, pathways and groups similar between Pathway Studio and DAVID.

**Table S3b-** According to DAVID

**Enriched functional annotation cluster analysisof genes in deletions and duplications CNVs in EO-OCD according to DAVID (cutoff p<0.05 and Enritchment score>1.3)**

**Annotation Cluster 1** **Enrichment Score: 2.211**

| Category | Term | Count | % | PValue | Genes | List Total | Pop Hits | Pop Total | Fold Enrichment | Bonferroni | Benjamini | FDR |
|---|---|---|---|---|---|---|---|---|---|---|---|---|
| UP_SEQ_FEATURE | domain:KRAB | 9 | 8.57 | 3.09E-04 | ZNF568, ZNF383, ZNF420, ZNF585A, ZNF662, ZNF585B, ZNF829, ZNF101, ZNF14 | 99 | 332 | 19113 | 5.23357065 | 0.11948366 | 0.11948366 | 0.43170599 |
| INTERPRO | IPR001909:Krueppel-associated box | 9 | 8.57 | 5.74E-04 | ZNF568, ZNF383, ZNF420, ZNF585A, ZNF662, ZNF585B, ZNF829, ZNF101, ZNF14 | 89 | 355 | 16659 | 4.74540275 | 0.125626 | 0.125626 | 0.73482555 |
| UP_SEQ_FEATURE | zinc finger region:C2H2-type 8 | 9 | 8.57 | 0.0014 | ZNF568, ZNF383, ZNF420, ZNF585A, ZNF662, ZNF585B, ZNF829, ZNF101, ZNF14 | 99 | 417 | 19113 | 4.16677567 | 0.43039343 | 0.24527716 | 1.89536596 |
| SMART | SM00349:KRAB | 9 | 8.57 | 0.0017 | ZNF568, ZNF383, ZNF420, ZNF585A, ZNF662, ZNF585B, ZNF829, ZNF101, ZNF14 | 59 | 355 | 9079 | 3.90121747 | 0.10289255 | 0.10289255 | 1.75178391 |
| UP_SEQ_FEATURE | zinc finger region:C2H2-type 6 | 9 | 8.57 | 0.0036 | ZNF568, ZNF383, ZNF420, ZNF585A, ZNF662, ZNF585B, ZNF829, ZNF101, ZNF14 | 99 | 488 | 19113 | 3.56054396 | 0.77448068 | 0.21981555 | 4.93773135 |
| UP_SEQ_FEATURE | zinc finger region:C2H2-type 5 | 9 | 8.57 | 0.0064 | ZNF568, ZNF383, ZNF420, ZNF585A, ZNF662, ZNF585B, ZNF829, ZNF101, ZNF14 | 99 | 538 | 19113 | 3.22963839 | 0.9299294 | 0.25573739 | 8.64168353 |
| UP_SEQ_FEATURE | zinc finger region:C2H2-type 1 | 9 | 8.57 | 0.0067 | ZNF568, ZNF383, ZNF420, ZNF585A, ZNF662, ZNF585B, ZNF829, ZNF101, ZNF14 | 99 | 542 | 19113 | 3.20580342 | 0.93768476 | 0.24236605 | 9.00530589 |
| INTERPRO | IPR013087:Zinc finger, C2H2-type/integrase, DNA-binding | 9 | 8.57 | 0.0168 | ZNF568, ZNF383, ZNF420, ZNF585A, ZNF662, ZNF585B, ZNF829, ZNF101, ZNF14 | 89 | 621 | 16659 | 2.71275037 | 0.98098217 | 0.86209486 | 19.5624218 |
| INTERPRO | IPR007087:Zinc finger, C2H2-type | 9 | 8.57 | 0.0550 | ZNF568, ZNF383, ZNF420, ZNF585A, ZNF662, ZNF585B, ZNF829, ZNF101, ZNF14 | 89 | 784 | 16659 | 2.14874742 | 0.99999822 | 0.98787281 | 51.6750246 |
| INTERPRO | IPR015880:Zinc finger, C2H2-like | 9 | 8.57 | 0.0594 | ZNF568, ZNF383, ZNF420, ZNF585A, ZNF662, ZNF585B, ZNF829, ZNF101, ZNF14 | 89 | 797 | 16659 | 2.11369884 | 0.99999941 | 0.97224776 | 54.5110388 |
| SMART | SM00355:ZnF_C2H2 | 9 | 8.57 | 0.1327 | ZNF568, ZNF383, ZNF420, ZNF585A, ZNF662, ZNF585B, ZNF829, ZNF101, ZNF14 | 59 | 797 | 9079 | 1.73768156 | 0.9998537 | 0.82898 | 76.2409042 |

**Annotation Cluster 2** **Enrichment Score: 2.179**

| Category | Term | Count | % | PValue | Genes | List Total | Pop Hits | Pop Total | Fold Enrichment | Bonferroni | Benjamini | FDR |
|---|---|---|---|---|---|---|---|---|---|---|---|---|
| UP_SEQ_FEATURE | zinc finger region:C2H2-type 19 | 4 | 3.81 | 0.0028 | ZNF420, ZNF585A, ZNF585B, ZNF14 | 99 | 55 | 19113 | 14.0407713 | 0.68850996 | 0.25293044 | 3.88812621 |
| UP_SEQ_FEATURE | zinc finger region:C2H2-type 18 | 4 | 3.81 | 0.0047 | ZNF420, ZNF585A, ZNF585B, ZNF14 | 99 | 66 | 19113 | 11.7006428 | 0.85849349 | 0.24372047 | 6.43222506 |
| UP_SEQ_FEATURE | zinc finger region:C2H2-type 17 | 4 | 3.81 | 0.0089 | ZNF420, ZNF585A, ZNF585B, ZNF14 | 99 | 83 | 19113 | 9.30412559 | 0.97517921 | 0.24746922 | 11.8091464 |
| UP_SEQ_FEATURE | zinc finger region:C2H2-type 16 | 4 | 3.81 | 0.0160 | ZNF420, ZNF585A, ZNF585B, ZNF14 | 99 | 103 | 19113 | 7.49749926 | 0.99868682 | 0.37746177 | 20.1963594 |

**Annotation Cluster 3** **Enrichment Score: 1.862**

| Category | Term | Count | % | PValue | Genes | List Total | Pop Hits | Pop Total | Fold Enrichment | Bonferroni | Benjamini | FDR |
|---|---|---|---|---|---|---|---|---|---|---|---|---|
| UP_SEQ_FEATURE | zinc finger region:C2H2-type 15 | 5 | 4.76 | 0.0050 | ZNF568, ZNF420, ZNF585A, ZNF585B, ZNF14 | 99 | 134 | 19113 | 7.20375396 | 0.87420678 | 0.22828462 | 6.80593744 |
| UP_SEQ_FEATURE | zinc finger region:C2H2-type 14 | 5 | 4.76 | 0.0087 | ZNF568, ZNF420, ZNF585A, ZNF585B, ZNF14 | 99 | 157 | 19113 | 6.14842694 | 0.97280266 | 0.25946793 | 11.5345438 |
| UP_SEQ_FEATURE | zinc finger region:C2H2-type 13 | 5 | 4.76 | 0.0199 | ZNF568, ZNF420, ZNF585A, ZNF585B, ZNF14 | 99 | 201 | 19113 | 4.80250264 | 0.99974943 | 0.40442897 | 24.5666402 |
| UP_SEQ_FEATURE | zinc finger region:C2H2-type 12 | 5 | 4.76 | 0.0408 | ZNF568, ZNF420, ZNF585A, ZNF585B, ZNF14 | 99 | 252 | 19113 | 3.83056758 | 0.99999996 | 0.57569639 | 44.1762085 |

**Annotation Cluster 4** Enrichment Score: 1.635

| Category | Term | Count | % | PValue | Genes | List Total | Pop Hits | Pop Total | Fold Enrichment | Bonferroni | Benjamini | FDR |
|---|---|---|---|---|---|---|---|---|---|---|---|---|
| SP_PIR_KEYWORDS | hydroxylysine | 3 | 2.86 | 0.0109 | C1QB, COL4A2, COL4A1 | 99 | 31 | 19235 | 18.8025415 | 0.86571538 | 0.48791496 | 12.619727 |
| SP_PIR_KEYWORDS | triple helix | 3 | 2.86 | 0.0109 | C1QB, COL4A2, COL4A1 | 99 | 31 | 19235 | 18.8025415 | 0.86571538 | 0.48791496 | 12.619727 |
| SP_PIR_KEYWORDS | hydroxyproline | 3 | 2.86 | 0.0152 | C1QB, COL4A2, COL4A1 | 99 | 37 | 19235 | 15.7534808 | 0.94073175 | 0.37558927 | 17.291953 |
| SP_PIR_KEYWORDS | hydroxylation | 3 | 2.86 | 0.0509 | C1QB, COL4A2, COL4A1 | 99 | 71 | 19235 | 8.20956039 | 0.99993291 | 0.65621075 | 47.5678587 |
| INTERPRO | IPR008160:Collagen triple helix repeat | 3 | 2.86 | 0.0728 | C1QB, COL4A2, COL4A1 | 89 | 84 | 16659 | 6.68499197 | 0.99999998 | 0.94759033 | 62.1666131 |

**Annotation Cluster 5** **Enrichment Score: 1.596**

| Category | Term | Count | % | PValue | Genes | List Total | Pop Hits | Pop Total | Fold Enrichment | Bonferroni | Benjamini | FDR |
|---|---|---|---|---|---|---|---|---|---|---|---|---|
| GOTERM_BP_FAT | GO:0007409~axonogenesis | 5 | 4.76 | 0.0153 | NCAM2, PARD3, NRXN1, SEMA3A, EPHB2 | 68 | 193 | 13528 | 5.15391649 | 0.99998811 | 0.97717563 | 20.8786243 |
| GOTERM_BP_FAT | GO:0048667~cell morphogenesis involved in neuron differentiation | 5 | 4.76 | 0.0199 | NCAM2, PARD3, NRXN1, SEMA3A, EPHB2 | 68 | 209 | 13528 | 4.75935829 | 0.99999962 | 0.9751462 | 26.3033485 |
| GOTERM_BP_FAT | GO:0048812~neuron projection morphogenesis | 5 | 4.76 | 0.0212 | NCAM2, PARD3, NRXN1, SEMA3A, EPHB2 | 68 | 213 | 13528 | 4.66998067 | 0.99999985 | 0.95698571 | 27.7383169 |
| GOTERM_BP_FAT | GO:0000904~cell morphogenesis involved in differentiation | 5 | 4.76 | 0.0327 | NCAM2, PARD3, NRXN1, SEMA3A, EPHB2 | 68 | 244 | 13528 | 4.07666345 | 1 | 0.93376876 | 39.6224892 |
| GOTERM_BP_FAT | GO:0048858~cell projection morphogenesis | 5 | 4.76 | 0.0331 | NCAM2, PARD3, NRXN1, SEMA3A, EPHB2 | 68 | 245 | 13528 | 4.06002401 | 1 | 0.91585642 | 40.0212718 |
| GOTERM_BP_FAT | GO:0032990~cell part morphogenesis | 5 | 4.76 | 0.0380 | NCAM2, PARD3, NRXN1, SEMA3A, EPHB2 | 68 | 256 | 13528 | 3.88556985 | 1 | 0.86897662 | 44.4347815 |

**Annotation Cluster 6** **Enrichment Score: 1.569**

| Category | Term | Count | % | PValue | Genes | List Total | Pop Hits | Pop Total | Fold Enrichment | Bonferroni | Benjamini | FDR |
|---|---|---|---|---|---|---|---|---|---|---|---|---|
| GOTERM_BP_FAT | GO:0031175~neuron projection development | 6 | 5.71 | 0.0087 | NCAM2, PARD3, MYO6, NRXN1, SEMA3A, EPHB2 | 68 | 256 | 13528 | 4.66268382 | 0.99835831 | 0.99835831 | 12.4027091 |
| GOTERM_BP_FAT | GO:0048666~neuron development | 6 | 5.71 | 0.0261 | NCAM2, PARD3, MYO6, NRXN1, SEMA3A, EPHB2 | 68 | 339 | 13528 | 3.52108277 | 1 | 0.93808421 | 33.1133433 |
| GOTERM_BP_FAT | GO:0030030~cell projection organization | 6 | 5.71 | 0.0354 | NCAM2, PARD3, MYO6, NRXN1, SEMA3A, EPHB2 | 68 | 368 | 13528 | 3.24360614 | 1 | 0.91033775 | 42.181837 |
| GOTERM_BP_FAT | GO:0030182~neuron differentiation | 6 | 5.71 | 0.0656 | NCAM2, PARD3, MYO6, NRXN1, SEMA3A, EPHB2 | 68 | 438 | 13528 | 2.7252216 | 1 | 0.91722862 | 64.2690076 |

**Annotation Cluster 7** **Enrichment Score: 1.535**

| Category | Term | Count | % | PValue | Genes | List Total | Pop Hits | Pop Total | Fold Enrichment | Bonferroni | Benjamini | FDR |
|---|---|---|---|---|---|---|---|---|---|---|---|---|
| SP_PIR_KEYWORDS | zinc-finger | 18 | 17.14 | 0.0058 | ZMYND11, ZNF568, PPARG, TRIM50, RAF1, ZNF585A, ZNF662, ZNF585B, ZNF829, ZNF14, MKRN2, RNF115, GMIP, PIAS3, ZNF383, SNRPC, ZNF420, ZNF101 | 99 | 1718 | 19235 | 2.03566515 | 0.65782616 | 0.41504373 | 6.95206409 |
| SP_PIR_KEYWORDS | zinc | 18 | 17.14 | 0.0501 | ZMYND11, ZNF568, PPARG, TRIM50, RAF1, ZNF585A, ZNF662, ZNF585B, ZNF829, ZNF14, MKRN2, RNF115, GMIP, PIAS3, ZNF383, SNRPC, ZNF420, ZNF101 | 99 | 2189 | 19235 | 1.59765771 | 0.99992156 | 0.69322783 | 47.0142001 |
| GOTERM_MF_FAT | GO:0008270~zinc ion binding | 18 | 17.14 | 0.0851 | ZMYND11, ZNF568, PPARG, TRIM50, RAF1, ZNF585A, ZNF662, ZNF585B, ZNF829, ZNF14, MKRN2, RNF115, GMIP, PIAS3, ZNF383, SNRPC, ZNF420, ZNF101 | 69 | 2311 | 12983 | 1.46554287 | 1 | 0.95865342 | 67.6164616 |

**Enriched functional annotation cluster analysisof genes in deletions and duplications CNVs in controls according to DAVID (cutoff p<0.05 and Enritchment score>1.3)**

**Annotation Cluster 1** **Enrichment Score: 5.332**

| Category | Term | Count | % | PValue | Genes | List Total | Pop Hits | Pop Total | Fold Enrichment | Bonferroni | Benjamini | FDR |
|---|---|---|---|---|---|---|---|---|---|---|---|---|
| SMART | SM00197:SAA | 5 | 3.91 | 5.15E-09 | SAA2, SAA1, SAA3P, SAAL1, SAA4 | 54 | 5 | 9079 | 168.12963 | 2.89E-07 | 2.89E-07 | 5.09E-06 |
| INTERPRO | IPR000096:Serum amyloid A protein | 5 | 3.91 | 6.59E-09 | SAA2, SAA1, SAA3P, SAAL1, SAA4 | 103 | 5 | 16659 | 161.737864 | 1.56E-06 | 1.56E-06 | 8.49E-06 |
| PIR_SUPERFAMILY | PIRSF002472:Serum_amyloid_A | 5 | 3.91 | 7.77E-09 | SAA2, SAA1, SAA3P, SAAL1, SAA4 | 49 | 5 | 7396 | 150.938776 | 4.20E-07 | 4.20E-07 | 7.61E-06 |
| GOTERM_BP_FAT | GO:0006953~acute-phase response | 5 | 3.91 | 9.71E-05 | SAA2, SAA1, SAA3P, SAAL1, SAA4 | 83 | 40 | 13528 | 20.373494 | 0.07507222 | 0.07507222 | 0.14898045 |
| GOTERM_BP_FAT | GO:0002526~acute inflammatory response | 5 | 3.91 | 0.0029 | SAA2, SAA1, SAA3P, SAAL1, SAA4 | 83 | 98 | 13528 | 8.31571183 | 0.90633093 | 0.69394598 | 4.42313917 |
| GOTERM_BP_FAT | GO:0006954~inflammatory response | 5 | 3.91 | 0.1347 | SAA2, SAA1, SAA3P, SAAL1, SAA4 | 83 | 325 | 13528 | 2.50750695 | 1 | 0.97652589 | 89.1608878 |

**Annotation Cluster 2** **Enrichment Score: 1.904**

| Category | Term | Count | % | PValue | Genes | List Total | Pop Hits | Pop Total | Fold Enrichment | Bonferroni | Benjamini | FDR |
|---|---|---|---|---|---|---|---|---|---|---|---|---|
| SP_PIR_KEYWORDS | hdl | 3 | 2.34 | 0.0048 | SAA2, SAA1, SAA4 | 113 | 18 | 19235 | 28.3702065 | 0.64401609 | 0.64401609 | 5.95581666 |
| SP_PIR_KEYWORDS | amyloid | 3 | 2.34 | 0.0092 | SAA2, SAA1, SAA4 | 113 | 25 | 19235 | 20.4265487 | 0.86146183 | 0.62779284 | 11.0870206 |
| SP_PIR_KEYWORDS | acute phase | 3 | 2.34 | 0.0123 | SAA2, SAA1, SAA4 | 113 | 29 | 19235 | 17.6090937 | 0.92856578 | 0.58507579 | 14.5202568 |
| GOTERM_CC_FAT | GO:0034364~high-density lipoprotein particle | 3 | 2.34 | 0.0124 | SAA2, SAA1, SAA4 | 88 | 25 | 12782 | 17.43 | 0.89828299 | 0.68106896 | 14.2953056 |
| GOTERM_CC_FAT | GO:0034358~plasma lipoprotein particle | 3 | 2.34 | 0.0236 | SAA2, SAA1, SAA4 | 88 | 35 | 12782 | 12.45 | 0.98726389 | 0.66406197 | 25.5095566 |
| GOTERM_CC_FAT | GO:0032994~protein-lipid complex | 3 | 2.34 | 0.0236 | SAA2, SAA1, SAA4 | 88 | 35 | 12782 | 12.45 | 0.98726389 | 0.66406197 | 25.5095566 |

**Annotation Cluster 3** **Enrichment Score: 1.547**

| Category | Term | Count | % | PValue | Genes | List Total | Pop Hits | Pop Total | Fold Enrichment | Bonferroni | Benjamini | FDR |
|---|---|---|---|---|---|---|---|---|---|---|---|---|
| GOTERM_BP_FAT | GO:0030595~leukocyte chemotaxis | 3 | 2.34 | 0.0211 | CORO1A, SAA2, SAA1 | 83 | 37 | 13528 | 13.2152393 | 0.99999996 | 0.91338886 | 27.9026267 |
| GOTERM_BP_FAT | GO:0060326~cell chemotaxis | 3 | 2.34 | 0.0233 | CORO1A, SAA2, SAA1 | 83 | 39 | 13528 | 12.5375348 | 0.99999999 | 0.906124 | 30.3423892 |
| GOTERM_BP_FAT | GO:0050900~leukocyte migration | 3 | 2.34 | 0.0467 | CORO1A, SAA2, SAA1 | 83 | 57 | 13528 | 8.57831325 | 1 | 0.935999 | 52.060052 |

**Annotation Cluster 4** **Enrichment Score: 1.470**

| Category | Term | Count | % | PValue | Genes | List Total | Pop Hits | Pop Total | Fold Enrichment | Bonferroni | Benjamini | FDR |
|---|---|---|---|---|---|---|---|---|---|---|---|---|
| GOTERM_BP_FAT | GO:0016477~cell migration | 6 | 4.69 | 0.0260 | NRCAM, CORO1A, SAA2, TAOK2, SAA1, PRKDC | 83 | 276 | 13528 | 3.54321634 | 1 | 0.90536624 | 33.3285368 |
| GOTERM_BP_FAT | GO:0048870~cell motility | 6 | 4.69 | 0.0386 | NRCAM, CORO1A, SAA2, TAOK2, SAA1, PRKDC | 83 | 307 | 13528 | 3.18543228 | 1 | 0.92833042 | 45.3513241 |
| GOTERM_BP_FAT | GO:0051674~localization of cell | 6 | 4.69 | 0.0386 | NRCAM, CORO1A, SAA2, TAOK2, SAA1, PRKDC | 83 | 307 | 13528 | 3.18543228 | 1 | 0.92833042 | 45.3513241 |

**Annotation Cluster 5** **Enrichment Score: 1.331**

| Category | Term | Count | % | PValue | Genes | List Total | Pop Hits | Pop Total | Fold Enrichment | Bonferroni | Benjamini | FDR |
|---|---|---|---|---|---|---|---|---|---|---|---|---|
| GOTERM_MF_FAT | GO:0005524~ATP binding | 15 | 11.72 | 0.0193 | KIF22, TAOK2, ALPK3, FARS2, ACSM2A, PRKDC, MCM4, DTD1, KIF13A, CBWD2, MAPK3, MAP3K8, DDX53, ACSM5, KIF2A | 69 | 1477 | 12983 | 1.91089459 | 0.9820283 | 0.86594142 | 21.7854586 |
| GOTERM_MF_FAT | GO:0032559~adenyl ribonucleotide binding | 15 | 11.72 | 0.0215 | KIF22, TAOK2, ALPK3, FARS2, ACSM2A, PRKDC, MCM4, DTD1, KIF13A, CBWD2, MAPK3, MAP3K8, DDX53, ACSM5, KIF2A | 69 | 1497 | 12983 | 1.88536493 | 0.98858894 | 0.77486553 | 23.9276121 |
| GOTERM_MF_FAT | GO:0030554~adenyl nucleotide binding | 15 | 11.72 | 0.0320 | KIF22, TAOK2, ALPK3, FARS2, ACSM2A, PRKDC, MCM4, DTD1, KIF13A, CBWD2, MAPK3, MAP3K8, DDX53, ACSM5, KIF2A | 69 | 1577 | 12983 | 1.78972182 | 0.99877159 | 0.81278722 | 33.6187285 |
| GOTERM_MF_FAT | GO:0001883~purine nucleoside binding | 15 | 11.72 | 0.0358 | KIF22, TAOK2, ALPK3, FARS2, ACSM2A, PRKDC, MCM4, DTD1, KIF13A, CBWD2, MAPK3, MAP3K8, DDX53, ACSM5, KIF2A | 69 | 1601 | 12983 | 1.76289276 | 0.99945419 | 0.77745962 | 36.8306415 |
| GOTERM_MF_FAT | GO:0032553~ribonucleotide binding | 15 | 11.72 | 0.0920 | KIF22, TAOK2, ALPK3, FARS2, ACSM2A, PRKDC, MCM4, DTD1, KIF13A, CBWD2, MAPK3, MAP3K8, DDX53, ACSM5, KIF2A | 69 | 1836 | 12983 | 1.53725017 | 1 | 0.83577728 | 70.3273071 |
| GOTERM_MF_FAT | GO:0032555~purine ribonucleotide binding | 15 | 11.72 | 0.0920 | KIF22, TAOK2, ALPK3, FARS2, ACSM2A, PRKDC, MCM4, DTD1, KIF13A, CBWD2, MAPK3, MAP3K8, DDX53, ACSM5, KIF2A | 69 | 1836 | 12983 | 1.53725017 | 1 | 0.83577728 | 70.3273071 |
| GOTERM_MF_FAT | GO:0017076~purine nucleotide binding | 15 | 11.72 | 0.1205 | KIF22, TAOK2, ALPK3, FARS2, ACSM2A, PRKDC, MCM4, DTD1, KIF13A, CBWD2, MAPK3, MAP3K8, DDX53, ACSM5, KIF2A | 69 | 1918 | 12983 | 1.47152831 | 1 | 0.88973422 | 80.1632472 |

**Abbreviations:**

**Yellow**, pathways and groups of brain/synapse related genes;

**Red**, pathways and groups similar between Pathway Studio and DAVID.
