# Supplementary material for: High resolution chromosomal microarray analysis in paediatric obsessive-compulsive disorder
Source: BMC Med Genomics. 2017 Nov 28;10:68. doi: 10.1186/s12920-017-0299-5 (PMC5704537; doi:10.1186/s12920-017-0299-5)
Supplement: Supplementary file 4 — (a) CNV burden analysis results from PLINK. The p-values shown are for the GCNT test statistic from PLINK’s cnv-enrichment-test algorithm, applied to the particular gene list versus the entire genome. Although Brain expressed and ID genes resulted in p values below 0.05, only the brain-expressed genes list remains significantly enriched in CNVs after correction for multiple testing (6 tests). (b) CNV burden analysis results from PLINK. The Gene List used. (PDF 1722 kb) [file 12920_2017_299_MOESM4_ESM.pdf]

**Table S4:** (a) CNV burden analysis results from PLINK. The p-values shown are for the GCNT test statistic from PLINK's cnv-enrichment-test algorithm, applied to the particular gene list versus the entire genome. Although Brain expressed and ID genes resulted in p values below 0.05, only the brain-expressed genes list remains significantly enriched in CNVs after correction for multiple testing (6 tests).

In the ID genes list, evidence for enrichment remains suggestive, but not significant after correction.

| GENE LIST | BETA | P VALUE |
|---|---|---|
| Behavior and Learning | 0.200877 | 0.406048 |
| Brain Expressed | 0.294754 | **0.0012815** |
| ID Genes | 0.174355 | **0.0110462** |
| Neuronal Activity | 0.261905 | 0.0938133 |
| Synapse Genes | 0.367178 | 0.0811691 |
| GenicAuto | 0.00344507 | **0.791454** |

**Table S4:** (b) CNV burden analysis results from PLINK. The Gene List used

| Chromosome | Start Position | End Position | ResfSeq Genes | Behavior and Learning | Brain Expressed | ID Genes | Neuronal Activity | Synapse Genes |
|---|---|---|---|---|---|---|---|---|
| 1 | 11873 | 14409 | DDX11L1 | | | | | |
| 1 | 14361 | 29370 | WASH7P | | | | | |
| 1 | 17368 | 17436 | MIR6859 | | | | | |
| 1 | 30365 | 30503 | MIR1302 | | | | | |
| 1 | 34610 | 36081 | FAM138A | | | | | |
| 1 | 34610 | 36081 | FAM138F | | | | | |
| 1 | 69090 | 70008 | OR4F5 | | | | | |
| 1 | 134772 | 140566 | LOC729737 | | | | | |
| 1 | 323891 | 328581 | LOC100132062 | | | | | |
| 1 | 323891 | 328581 | LOC100132287 | | | | | |
| 1 | 323891 | 328581 | LOC100133331 | | | | | |
| 1 | 367658 | 368597 | OR4F16 | | | | | |
| 1 | 367658 | 368597 | OR4F29 | | | | | |
| 1 | 367658 | 368597 | OR4F3 | | | | | |
| 1 | 562759 | 564389 | LOC101928626 | | | | | |
| 1 | 567704 | 567793 | MIR6723 | | | | | |
| 1 | 621095 | 622034 | OR4F16 | | | | | |
| 1 | 621095 | 622034 | OR4F29 | | | | | |
| 1 | 621095 | 622034 | OR4F3 | | | | | |
| 1 | 661138 | 665731 | LOC100133331 | | | | | |
| 1 | 700244 | 714068 | LOC100288069 | | | | | |
| 1 | 752750 | 755214 | FAM87B | | | | | |
| 1 | 761585 | 762902 | LINC00115 | | | | | |
| 1 | 762970 | 794826 | LINC01128 | | | | | |
| 1 | 803450 | 812182 | FAM41C | | | | | |
| 1 | 852197 | 855072 | LOC100130417 | | | | | |
| 1 | 861120 | 879961 | SAMD11 | | | | | |
| 1 | 879582 | 894679 | NOC2L | | | | | |
| 1 | 895966 | 901099 | KLHL17 | | | KLHL17 | KLHL17 | |
| 1 | 901876 | 910484 | PLEKHN1 | | | | | |
| 1 | 910578 | 917497 | PERM1 | | | | | |
| 1 | 934341 | 935552 | HES4 | | | | | |
| 1 | 948846 | 949919 | ISG15 | | | | | |
| 1 | 955502 | 991499 | AGRN | | | | | |
| 1 | 1007125 | 1009687 | RNF223 | | | | | |
| 1 | 1017197 | 1051736 | C1orf159 | | | | | |
| 1 | 1072396 | 1079434 | LINC01342 | | | | | |
| 1 | 1102483 | 1102578 | MIR200B | | | | | |
| 1 | 1103242 | 1103332 | MIR200A | | | | | |
| 1 | 1104384 | 1104467 | MIR429 | | | | | |
| 1 | 1109285 | 1121243 | TTLL10 | | | | | |
| 1 | 1138887 | 1142089 | TNFRSF18 | | | | | |
| 1 | 1146705 | 1149548 | TNFRSF4 | | | | | |
| 1 | 1152287 | 1167447 | SDF4 | | | | | |
| 1 | 1167628 | 1170420 | B3GALT6 | | | | | |
| 1 | 1177825 | 1182102 | FAM132A | | | | | |
| 1 | 1189291 | 1209234 | UBE2J2 | | | | | |
| 1 | 1215815 | 1227409 | SCNN1D | | | SCNN1D | | |
| 1 | 1227763 | 1243269 | ACAP3 | | | | | |
| 1 | 1231489 | 1231550 | MIR6726 | | | | | |
| 1 | 1243993 | 1247057 | PUSL1 | | | | | |
| 1 | 1246964 | 1260067 | CPSF3L | | | | | |
| 1 | 1247881 | 1247946 | MIR6727 | | | | | |
| 1 | 1260142 | 1264276 | CPTP | | | | | |
| 1 | 1266725 | 1270700 | TAS1R3 | | | | | |
| 1 | 1270657 | 1284492 | DVL1 | | | | | |
| 1 | 1275029 | 1275088 | MIR6808 | | | | | |
| 1 | 1288068 | 1298921 | MXRA8 | | | | | |
| 1 | 1309109 | 1310562 | AURKAIP1 | | | | | |
| 1 | 1321090 | 1334718 | CCNL2 | | | | | |
| 1 | 1334909 | 1337426 | LOC148413 | | | | | |
| 1 | 1337275 | 1342693 | MRPL20 | | | | | |
| 1 | 1353799 | 1356824 | ANKRD65 | | | | | |

| | | | | | | | | |
|---|---|---|---|---|---|---|---|---|
| 1 | 1361507 | 1363167 | TMEM88B | | | | | |
| 1 | 1365536 | 1369953 | LOC102724312 | | | | | |
| 1 | 1370902 | 1378262 | VWA1 | | | | | |
| 1 | 1385068 | 1405538 | ATAD3C | | | | | |
| 1 | 1407134 | 1431584 | ATAD3B | | | | | |
| 1 | 1447522 | 1470067 | ATAD3A | | | | | |
| 1 | 1470157 | 1475740 | TMEM240 | | | | | |
| 1 | 1477052 | 1510262 | SSU72 | | | | | |
| 1 | 1533387 | 1535476 | C1orf233 | | | | | |
| 1 | 1550794 | 1565990 | MIB2 | | | MIB2 | | |
| 1 | 1567559 | 1570030 | MMP23B | | | | | |
| 1 | 1568158 | 1570027 | MMP23A | | | | | |
| 1 | 1570602 | 1655859 | CDK11B | | | | | |
| 1 | 1592938 | 1624243 | SLC35E2B | | | | | |
| 1 | 1631377 | 1633247 | MMP23A | | | | | |
| 1 | 1633822 | 1656004 | CDK11A | | | | | |
| 1 | 1656276 | 1677438 | SLC35E2 | | | | | |
| 1 | 1682670 | 1711508 | NADK | | | | | |
| 1 | 1716723 | 1822556 | GNB1 | | GNB1 | | | |
| 1 | 1846265 | 1848733 | CALML6 | | | | | |
| 1 | 1849028 | 1850740 | TMEM52 | | | | | |
| 1 | 1853389 | 1935276 | CFAP74 | | | | | |
| 1 | 1950767 | 1962192 | GABRD | GABRD | GABRD | | GABRD | GABRD |
| 1 | 1981908 | 2116834 | PRKCZ | | PRKCZ | PRKCZ | | |
| 1 | 2115898 | 2126214 | FAAP20 | | | | | |
| 1 | 2160133 | 2241652 | SKI | | | | | |
| 1 | 2252691 | 2323190 | MORN1 | | | | | |
| 1 | 2281852 | 2284100 | LOC100129534 | | | | | |
| 1 | 2286613 | 2323190 | MORN1 | | | | | |
| 1 | 2323213 | 2336885 | RER1 | | | | | |
| 1 | 2336240 | 2344010 | PEX10 | | PEX10 | | | |
| 1 | 2398897 | 2436969 | PLCH2 | | | | | |
| 1 | 2439969 | 2458067 | PANK4 | | | | | |
| 1 | 2460183 | 2461684 | HES5 | | | | | |
| 1 | 2481358 | 2488450 | LOC115110 | | | | | |
| 1 | 2487803 | 2497061 | TNFRSF14 | | | | | |
| 1 | 2497973 | 2515973 | LOC100996583 | | | | | |
| 1 | 2517898 | 2522908 | FAM213B | | | | | |
| 1 | 2522080 | 2564481 | MMEL1 | | | | | |
| 1 | 2572806 | 2706230 | TTC34 | | | | | |
| 1 | 2938045 | 2939467 | ACTRT2 | | | | | |
| 1 | 2976180 | 2984289 | LINC00982 | | | | | |
| 1 | 2985741 | 3355185 | PRDM16 | | | PRDM16 | | |
| 1 | 3044538 | 3044599 | MIR4251 | | | | | |
| 1 | 3371146 | 3397677 | ARHGEF16 | | | | | |
| 1 | 3404505 | 3528059 | MEGF6 | | | | | |
| 1 | 3477259 | 3477354 | MIR551A | | | | | |
| 1 | 3541555 | 3546694 | TPRG1L | | | | | |
| 1 | 3547330 | 3566671 | WRAP73 | | | | | |
| 1 | 3569128 | 3663937 | TP73 | | | | | |
| 1 | 3668964 | 3688209 | CCDC27 | | | | | |
| 1 | 3689324 | 3692546 | SMIM1 | | | | | |
| 1 | 3696783 | 3713068 | LRRC47 | | | | | |
| 1 | 3728644 | 3773797 | CEP104 | | | | | |
| 1 | 3773830 | 3801993 | DFFB | | | | | |
| 1 | 3805696 | 3816857 | C1orf174 | | | | | |
| 1 | 3816967 | 3832011 | LINC01134 | | | | | |
| 1 | 4000671 | 4012643 | LINC01346 | | | | | |
| 1 | 4472110 | 4484744 | LOC284661 | | | | | |
| 1 | 4715104 | 4843851 | AJAP1 | | | | | |
| 1 | 5624130 | 5624203 | MIR4417 | | | | | |
| 1 | 5922731 | 5922801 | MIR4689 | | | | | |
| 1 | 5922867 | 6052533 | NPHP4 | | | | | |
| 1 | 6052357 | 6161253 | KCNAB2 | | KCNAB2 | | | |
| 1 | 6161846 | 6240194 | CHD5 | | | | | |
| 1 | 6245079 | 6259679 | RPL22 | | | | | |

| | | | | | | | | |
|---|---|---|---|---|---|---|---|---|
| 1 | 6264899 | 6265840 | LOC102724450 | | | | | |
| 1 | 6266188 | 6281359 | RNF207 | | | | | |
| 1 | 6281252 | 6296044 | ICMT | | | | | |
| 1 | 6296299 | 6299502 | LINC00337 | | | | | |
| 1 | 6304251 | 6305638 | HES3 | | | | | |
| 1 | 6307405 | 6321035 | GPR153 | | | | | |
| 1 | 6324331 | 6453826 | ACOT7 | | | | | |
| 1 | 6475293 | 6479979 | HES2 | | | | | |
| 1 | 6484847 | 6521004 | ESPN | | | | | |
| 1 | 6489893 | 6489956 | MIR4252 | | | | | |
| 1 | 6521213 | 6526255 | TNFRSF25 | | | | | |
| 1 | 6526151 | 6580121 | PLEKHG5 | | | | | |
| 1 | 6581406 | 6614658 | NOL9 | | | | | |
| 1 | 6615337 | 6639817 | TAS1R1 | | | TAS1R1 | | |
| 1 | 6640050 | 6649340 | ZBTB48 | | | | | |
| 1 | 6650783 | 6662958 | KLHL21 | | | | | |
| 1 | 6673755 | 6684093 | PHF13 | | | | | |
| 1 | 6684924 | 6695646 | THAP3 | | | | | |
| 1 | 6694227 | 6761966 | DNAJC11 | | | | | |
| 1 | 6784804 | 6790072 | LOC100505887 | | | | | |
| 1 | 6845383 | 7829766 | CAMTA1 | | | | | |
| 1 | 7831328 | 7841492 | VAMP3 | | | VAMP3 | | |
| 1 | 7844488 | 7905240 | PER3 | | | | | |
| 1 | 7907671 | 7913551 | UTS2 | | | | | |
| 1 | 7975930 | 8003225 | TNFRSF9 | | | | | |
| 1 | 8021713 | 8045342 | PARK7 | | | | | |
| 1 | 8071778 | 8086393 | ERRFI1 | | | | | |
| 1 | 8268731 | 8275268 | LOC102724539 | | | | | |
| 1 | 8378144 | 8404227 | SLC45A1 | | SLC45A1 | | | |
| 1 | 8412463 | 8877699 | RERE | | | | | |
| 1 | 8484704 | 8495076 | LOC102724552 | | | | | |
| 1 | 8554859 | 8554973 | SNORD128 | | | | | |
| 1 | 8921058 | 8939151 | ENO1 | | | | | |
| 1 | 8926560 | 8926649 | MIR6728 | | | | | |
| 1 | 8938893 | 8939943 | ENO1 | | | | | |
| 1 | 9005892 | 9035148 | CA6 | | | | | |
| 1 | 9063358 | 9086404 | SLC2A7 | | | | | |
| 1 | 9097004 | 9129887 | SLC2A5 | | | | | |
| 1 | 9164475 | 9189229 | GPR157 | | | | | |
| 1 | 9208069 | 9242397 | MIR34AHG | | | | | |
| 1 | 9211726 | 9211836 | MIR34A | | | | | |
| 1 | 9242262 | 9252144 | LOC102724571 | | | | | |
| 1 | 9294862 | 9331394 | H6PD | | | | | |
| 1 | 9352940 | 9429590 | SPSB1 | | | | | |
| 1 | 9485152 | 9489055 | LOC100506022 | | | | | |
| 1 | 9599527 | 9642831 | SLC25A33 | | | | | |
| 1 | 9648931 | 9674935 | TMEM201 | | | | | |
| 1 | 9711789 | 9747632 | PIK3CD | | | | | |
| 1 | 9789078 | 9884584 | CLSTN1 | | | | | |
| 1 | 9908333 | 9970316 | CTNNBIP1 | | | | | |
| 1 | 9982170 | 10003485 | LZIC | | | | | |
| 1 | 10002980 | 10045556 | NMNAT1 | | | | | |
| 1 | 10027438 | 10027516 | MIR5697 | | | | | |
| 1 | 10057254 | 10076078 | RBP7 | | | | | |
| 1 | 10093040 | 10241296 | UBE4B | | | | | |
| 1 | 10270763 | 10441661 | KIF1B | | | KIF1B | | |
| 1 | 10359023 | 10359128 | RNU6-2 | | | | | |
| 1 | 10459048 | 10480568 | PGD | | | | | |
| 1 | 10490158 | 10502872 | APITD1 | | | | | |
| 1 | 10490158 | 10512060 | APITD1-CORT | | | | | |
| 1 | 10490803 | 10502872 | APITD1 | | | | | |
| 1 | 10490803 | 10512060 | APITD1-CORT | | | | | |
| 1 | 10509775 | 10512060 | CORT | | | | | |
| 1 | 10520602 | 10532613 | DFFA | | | | | |
| 1 | 10535002 | 10690815 | PEX14 | | | | | |
| 1 | 10696665 | 10856733 | CASZ1 | | | | | |

| | | | | | | | | | |
|---|---|---|---|---|---|---|---|---|---|
| 1 | 11006529 | 11042094 | C1orf127 | | | | | | |
| 1 | 11072678 | 11085549 | TARDBP | | | | | | |
| 1 | 11086579 | 11107296 | MASP2 | | | MASP2 | | | |
| 1 | 11114648 | 11120091 | SRM | | | | | | |
| 1 | 11126669 | 11159967 | EXOSC10 | | | | | | |
| 1 | 11159731 | 11162162 | LOC105376736 | | | | | | |
| 1 | 11166587 | 11209595 | MTOR | | | | | | |
| 1 | 11249345 | 11256038 | ANGPTL7 | | | | | | |
| 1 | 11333254 | 11348491 | UBIAD1 | | | | | | |
| 1 | 11539294 | 11597640 | DISP3 | | | | | | |
| 1 | 11669587 | 11673411 | LOC101929181 | | | | | | |
| 1 | 11708417 | 11714888 | FBXO2 | | | | | | |
| 1 | 11714431 | 11723384 | FBXO44 | | | | | | |
| 1 | 11724149 | 11734409 | FBXO6 | | | | | | |
| 1 | 11734536 | 11751678 | MAD2L2 | | | | | | |
| 1 | 11751780 | 11780336 | DRAXIN | | | | | | |
| 1 | 11796141 | 11810828 | AGTRAP | | | | | | |
| 1 | 11822249 | 11849642 | C1orf167 | | | | | | |
| 1 | 11837133 | 11839676 | LOC102724659 | | | | | | |
| 1 | 11845786 | 11866160 | MTHFR | | | MTHFR | | | |
| 1 | 11866152 | 11903201 | CLCN6 | | CLCN6 | | | | |
| 1 | 11900375 | 11907840 | NPPA | | | | | | |
| 1 | 11917520 | 11918992 | NPPB | | | | | | |
| 1 | 11979644 | 11986485 | KIAA2013 | | | | | | |
| 1 | 11994723 | 12035599 | PLOD1 | | | | | | |
| 1 | 12040237 | 12073572 | MFN2 | | | MFN2 | | | |
| 1 | 12079298 | 12092106 | MIIP | | | | | | |
| 1 | 12089214 | 12089279 | MIR6729 | | | | | | |
| 1 | 12123433 | 12204264 | TNFRSF8 | | | | | | |
| 1 | 12226999 | 12227095 | MIR7846 | | | | | | |
| 1 | 12227059 | 12269277 | TNFRSF1B | | | | | | |
| 1 | 12251769 | 12251830 | MIR4632 | | | | | | |
| 1 | 12290095 | 12572098 | VPS13D | | | | | | |
| 1 | 12567299 | 12567451 | SNORA59A | | | | | | |
| 1 | 12567299 | 12567451 | SNORA59B | | | | | | |
| 1 | 12627938 | 12677737 | DHRS3 | | | | | | |
| 1 | 12638984 | 12639051 | MIR6730 | | | | | | |
| 1 | 12704565 | 12727097 | AADACL4 | | | | | | |
| 1 | 12776117 | 12788726 | AADACL3 | | | | | | |
| 1 | 12806133 | 12821102 | C1orf158 | | | | | | |
| 1 | 12834990 | 12838048 | PRAMEF12 | | | | | | |
| 1 | 12851545 | 12856777 | PRAMEF1 | | | | | | |
| 1 | 12884617 | 12891264 | PRAMEF11 | | | | | | |
| 1 | 12907229 | 12908157 | HNRNPCL4 | | | | | | |
| 1 | 12907229 | 12908235 | HNRNPCL3 | | | | | | |
| 1 | 12907229 | 12908609 | HNRNPCL1 | | | | | | |
| 1 | 12916940 | 12921764 | PRAMEF2 | | | | | | |
| 1 | 12939032 | 12946025 | PRAMEF4 | | | | | | |
| 1 | 12976466 | 12980300 | PRAMEF7 | | | | | | |
| 1 | 12976466 | 12980300 | PRAMEF8 | | | | | | |
| 1 | 12998301 | 13007406 | PRAMEF6 | | | | | | |
| 1 | 12998504 | 13002353 | PRAMEF27 | | | | | | |
| 1 | 12998504 | 13002353 | PRAMEF9 | | | | | | |
| 1 | 13035498 | 13039011 | PRAMEF18 | | | | | | |
| 1 | 13035542 | 13038381 | PRAMEF22 | | | | | | |
| 1 | 13052239 | 13112702 | PRAMEF25 | | | | | | |
| 1 | 13052393 | 13112702 | PRAMEF26 | | | | | | |
| 1 | 13108513 | 13117751 | PRAMEF5 | | | | | | |
| 1 | 13161985 | 13167193 | PRAMEF34P | | | | | | |
| 1 | 13161985 | 13167193 | PRAMEF36P | | | | | | |
| 1 | 13182959 | 13184326 | HNRNPCL2 | | | | | | |
| 1 | 13194517 | 13199727 | PRAMEF34P | | | | | | |
| 1 | 13194517 | 13199727 | PRAMEF36P | | | | | | |
| 1 | 13328195 | 13698405 | PRAMEF18 | | | | | | |
| 1 | 13328832 | 13331671 | PRAMEF22 | | | | | | |
| 1 | 13359818 | 13369057 | PRAMEF5 | | | | | | |

| | | | | | | | | |
|---|---|---|---|---|---|---|---|---|
| 1 | 13359832 | 13368936 | PRAMEF6 | | | | | |
| 1 | 13364885 | 13368733 | PRAMEF27 | | | | | |
| 1 | 13386914 | 13390748 | PRAMEF7 | | | | | |
| 1 | 13386914 | 13390748 | PRAMEF8 | | | | | |
| 1 | 13409127 | 13414502 | PRAMEF10 | | | | | |
| 1 | 13409134 | 13414502 | PRAMEF33P | | | | | |
| 1 | 13421175 | 13428190 | PRAMEF15 | | | | | |
| 1 | 13424002 | 13427859 | PRAMEF27 | | | | | |
| 1 | 13447413 | 13452656 | PRAMEF13 | | | | | |
| 1 | 13447413 | 13452656 | PRAMEF14 | | | | | |
| 1 | 13474052 | 13477569 | PRAMEF19 | | | | | |
| 1 | 13495257 | 13498257 | PRAMEF17 | | | | | |
| 1 | 13516065 | 13526943 | PRAMEF20 | | | | | |
| 1 | 13607699 | 13611533 | PRAMEF7 | | | | | |
| 1 | 13607699 | 13611533 | PRAMEF8 | | | | | |
| 1 | 13629924 | 13635299 | PRAMEF10 | | | | | |
| 1 | 13629931 | 13635299 | PRAMEF33P | | | | | |
| 1 | 13641972 | 13648987 | PRAMEF15 | | | | | |
| 1 | 13644799 | 13648656 | PRAMEF27 | | | | | |
| 1 | 13668268 | 13673511 | PRAMEF13 | | | | | |
| 1 | 13668268 | 13673511 | PRAMEF14 | | | | | |
| 1 | 13694888 | 13698405 | PRAMEF19 | | | | | |
| 1 | 13716091 | 13719089 | PRAMEF17 | | | | | |
| 1 | 13736906 | 13747803 | PRAMEF20 | | | | | |
| 1 | 13801444 | 13840242 | LRRC38 | | | | | |
| 1 | 13910251 | 13944452 | PDPN | | | | | |
| 1 | 14026734 | 14114574 | PRDM2 | | | | | |
| 1 | 14925212 | 15394651 | KAZN | | KAZN | | | |
| 1 | 15438310 | 15546974 | TMEM51 | | | | | |
| 1 | 15490691 | 15498120 | C1orf195 | | | | | |
| 1 | 15573767 | 15724622 | FHAD1 | | | | | |
| 1 | 15736390 | 15756839 | EFHD2 | | | | | |
| 1 | 15764937 | 15773153 | CTRC | | | | | |
| 1 | 15783222 | 15798586 | CELA2A | | | | | |
| 1 | 15802595 | 15817895 | CELA2B | | | | | |
| 1 | 15817895 | 15851285 | CASP9 | | | | | |
| 1 | 15853307 | 15898228 | DNAJC16 | | | | | |
| 1 | 15898193 | 15911605 | AGMAT | | | | | |
| 1 | 15943952 | 15987552 | DDI2 | | | | | |
| 1 | 15986207 | 15988525 | RSC1A1 | | | | | |
| 1 | 16010826 | 16061264 | PLEKHM2 | | | | | |
| 1 | 16062808 | 16067887 | SLC25A34 | | | | | |
| 1 | 16068986 | 16074292 | TMEM82 | | | | | |
| 1 | 16085254 | 16113084 | FBLIM1 | | | | | |
| 1 | 16133656 | 16134194 | UQCRHL | | | | | |
| 1 | 16160709 | 16174642 | FLJ37453 | | | | | |
| 1 | 16174358 | 16266950 | SPEN | | | | | |
| 1 | 16197643 | 16231962 | MIR5096 | | | | | |
| 1 | 16268363 | 16302627 | ZBTB17 | | | | | |
| 1 | 16330730 | 16333190 | C1orf64 | | | | | |
| 1 | 16340522 | 16345285 | HSPB7 | | | | | |
| 1 | 16348485 | 16360545 | CLCNKA | | | CLCNKA | | |
| 1 | 16370230 | 16383821 | CLCNKB | | | CLCNKB | | |
| 1 | 16384263 | 16400127 | FAM131C | | | | | |
| 1 | 16450831 | 16482582 | EPHA2 | | | | | |
| 1 | 16524598 | 16539104 | ARHGEF19 | | | | | |
| 1 | 16558181 | 16563659 | RSG1 | | | | | |
| 1 | 16573334 | 16678986 | FBXO42 | | | | | |
| 1 | 16693524 | 16724643 | SZRD1 | | | | | |
| 1 | 16725137 | 16763919 | SPATA21 | | | | | |
| 1 | 16767166 | 16786584 | NECAP2 | | | | | |
| 1 | 16793930 | 16819196 | CROCCP3 | | | | | |
| 1 | 16865218 | 16866530 | FAM231B | | | | | |
| 1 | 16875408 | 16875482 | MIR3675 | | | | | |
| 1 | 16888921 | 16940100 | NBPF1 | | | | | |
| 1 | 16944750 | 16957401 | CROCCP2 | | | | | |

| | | | | | | | | |
|---|---|---|---|---|---|---|---|---|
| 1 | 16972068 | 16976915 | MST1P2 | | | | | |
| 1 | 16999497 | 17000007 | FAM231A | | | | | |
| 1 | 16999497 | 17000152 | FAM231C | | | | | |
| 1 | 17007749 | 17007823 | MIR3675 | | | | | |
| 1 | 17017712 | 17046652 | ESPNP | | | | | |
| 1 | 17060301 | 17060956 | FAM231C | | | | | |
| 1 | 17060446 | 17060956 | FAM231A | | | | | |
| 1 | 17081128 | 17090975 | MST1L | | | | | |
| 1 | 17185443 | 17185516 | MIR3675 | | | | | |
| 1 | 17215032 | 17216161 | LOC105376805 | | | | | |
| 1 | 17248444 | 17299474 | CROCC | | | | | |
| 1 | 17300998 | 17308081 | MFAP2 | | | | | |
| 1 | 17312452 | 17338467 | ATP13A2 | | ATP13A2 | | | |
| 1 | 17345224 | 17380665 | SDHB | | | | | |
| 1 | 17393255 | 17445948 | PADI2 | | PADI2 | | | |
| 1 | 17531620 | 17572501 | PADI1 | | | | | |
| 1 | 17575592 | 17610727 | PADI3 | | | | | |
| 1 | 17604383 | 17604470 | MIR3972 | | | | | |
| 1 | 17634689 | 17690495 | PADI4 | | | | | |
| 1 | 17698690 | 17728195 | PADI6 | | | | | |
| 1 | 17733250 | 17766250 | RCC2 | | | | | |
| 1 | 17866329 | 18024370 | ARHGEF10L | | | | | |
| 1 | 18081807 | 18153558 | ACTL8 | | | | | |
| 1 | 18392150 | 18400905 | LOC101927876 | | | | | |
| 1 | 18434239 | 18704977 | IGSF21 | | | | | |
| 1 | 18807423 | 18812480 | KLHDC7A | | | | | |
| 1 | 18957499 | 19075360 | PAX7 | | | | | |
| 1 | 19166092 | 19186155 | TAS1R2 | | | | | |
| 1 | 19197923 | 19229293 | ALDH4A1 | | ALDH4A1 | | | |
| 1 | 19209695 | 19209769 | MIR4695 | | | | | |
| 1 | 19223564 | 19223642 | MIR1290 | | | | | |
| 1 | 19230773 | 19282826 | IFFO2 | | | | | |
| 1 | 19400999 | 19536746 | UBR4 | | | | | |
| 1 | 19536879 | 19567198 | LOC101927895 | | | | | |
| 1 | 19542157 | 19578053 | EMC1 | | EMC1 | | | |
| 1 | 19578074 | 19586622 | MRTO4 | | | | | |
| 1 | 19592475 | 19600568 | AKR7L | | | | | |
| 1 | 19609056 | 19615280 | AKR7A3 | | | | | |
| 1 | 19619740 | 19622230 | LOC100506730 | | | | | |
| 1 | 19629201 | 19638640 | AKR7A2 | | | | | |
| 1 | 19638739 | 19658922 | PQLC2 | | | | | |
| 1 | 19665266 | 19812135 | CAPZB | | | | | |
| 1 | 19923470 | 19956315 | MINOS1 | | | | | |
| 1 | 19923470 | 19984949 | MINOS1-NBL1 | | | | | |
| 1 | 19934300 | 19935138 | RPS14P3 | | | | | |
| 1 | 19969722 | 19984949 | NBL1 | | | | | |
| 1 | 19991779 | 20007459 | HTR6 | | | HTR6 | | |
| 1 | 20008705 | 20126410 | TMCO4 | | | | | |
| 1 | 20140521 | 20141771 | RNF186 | | | | | |
| 1 | 20208887 | 20239437 | OTUD3 | | | | | |
| 1 | 20246799 | 20250110 | PLA2G2E | | | | | |
| 1 | 20301923 | 20306932 | PLA2G2A | | | | | |
| 1 | 20396700 | 20418394 | PLA2G5 | | PLA2G5 | | | |
| 1 | 20439142 | 20446059 | PLA2G2D | | | | | |
| 1 | 20465822 | 20476879 | PLA2G2F | | | | | |
| 1 | 20489584 | 20503857 | PLA2G2C | | | | | |
| 1 | 20510735 | 20522541 | UBXN10 | | | | | |
| 1 | 20617411 | 20681387 | VWA5B1 | | | | | |
| 1 | 20686293 | 20755287 | LINC01141 | | | | | |
| 1 | 20808883 | 20812728 | CAMK2N1 | | | | | |
| 1 | 20825940 | 20834674 | MUL1 | | | | | |
| 1 | 20878931 | 20881513 | FAM43B | | | | | |
| 1 | 20915443 | 20945400 | CDA | | | | | |
| 1 | 20959947 | 20978004 | PINK1 | | PINK1 | | | |
| 1 | 20960171 | 20960281 | MIR6084 | | | | | |
| 1 | 20969149 | 20978686 | PINK1 | | PINK1 | | | |

| | | | | | | | | |
|---|---|---|---|---|---|---|---|---|
| 1 | 20978259 | 20988037 | DDOST | | | | | |
| 1 | 20990506 | 21044510 | KIF17 | | | | | |
| 1 | 21046224 | 21059133 | SH2D5 | | | | | |
| 1 | 21069170 | 21113181 | HP1BP3 | | | | | |
| 1 | 21132784 | 21501251 | EIF4G3 | | EIF4G3 | | | |
| 1 | 21314806 | 21314925 | MIR1256 | | | | | |
| 1 | 21543739 | 21672034 | ECE1 | | | ECE1 | | |
| 1 | 21619782 | 21626362 | LOC100506801 | | | | | |
| 1 | 21766582 | 21811393 | NBPF3 | | | | | |
| 1 | 21835850 | 21904905 | ALPL | | | | | |
| 1 | 21922707 | 21995856 | RAP1GAP | | RAP1GAP | | | |
| 1 | 22004791 | 22109688 | USP48 | | | | | USP48 |
| 1 | 22138757 | 22151714 | LDLRAD2 | | | | | |
| 1 | 22148724 | 22263790 | HSPG2 | | | | | |
| 1 | 22303417 | 22315847 | CELA3B | | | | | |
| 1 | 22328148 | 22339035 | CELA3A | | | | | |
| 1 | 22350486 | 22352541 | LOC101928043 | | | | | |
| 1 | 22351683 | 22357717 | LINC00339 | | | | | |
| 1 | 22379119 | 22419436 | CDC42 | | | CDC42 | | |
| 1 | 22443797 | 22469519 | WNT4 | | | | | |
| 1 | 22592731 | 22592793 | MIR4418 | | | | | |
| 1 | 22778343 | 22857650 | ZBTB40 | | | | | |
| 1 | 22890003 | 22930087 | EPHA8 | | | | | |
| 1 | 22959750 | 22959859 | MIR6127 | | | | | |
| 1 | 22963117 | 22966175 | C1QA | | | | | |
| 1 | 22970117 | 22974603 | C1QC | | | | | |
| 1 | 22979681 | 22988029 | C1QB | | | | | |
| 1 | 23037330 | 23247993 | EPHB2 | EPHB2 | EPHB2 | | | EPHB2 |
| 1 | 23046009 | 23046091 | MIR4684 | | | | | |
| 1 | 23189651 | 23189719 | MIR4253 | | | | | |
| 1 | 23279535 | 23291923 | LACTBL1 | | | | | |
| 1 | 23337326 | 23342343 | C1orf234 | | | | | |
| 1 | 23345940 | 23410184 | KDM1A | | | | | |
| 1 | 23370797 | 23370865 | MIR3115 | | | | | |
| 1 | 23384350 | 23384427 | MIR4419A | | | | | |
| 1 | 23410515 | 23495351 | LUZP1 | | | | | |
| 1 | 23518387 | 23521222 | HTR1D | | | | | |
| 1 | 23607801 | 23612011 | LINC01355 | | | | | |
| 1 | 23631180 | 23670857 | HNRNPR | | | | | |
| 1 | 23685940 | 23698330 | ZNF436 | | | | | |
| 1 | 23707401 | 23751272 | TCEA3 | | | | | |
| 1 | 23755055 | 23810750 | ASAP3 | | | | | |
| 1 | 23832919 | 23857712 | E2F2 | | | | | |
| 1 | 23848421 | 23854911 | LOC101928163 | | | | | |
| 1 | 23884420 | 23886285 | ID3 | | | | | |
| 1 | 23953823 | 23967056 | MDS2 | | | | | |
| 1 | 24018268 | 24022915 | RPL11 | | | | | |
| 1 | 24069855 | 24104787 | TCEB3 | | | | | |
| 1 | 24104875 | 24114722 | PITHD1 | | | | | |
| 1 | 24117645 | 24122029 | LYPLA2 | | | | | |
| 1 | 24122088 | 24127294 | GALE | | | | | |
| 1 | 24128366 | 24151949 | HMGCL | | | HMGCL | | |
| 1 | 24171571 | 24194859 | FUCA1 | | | | | |
| 1 | 24200459 | 24239817 | CNR2 | | | | | |
| 1 | 24255559 | 24255637 | MIR378F | | | | | |
| 1 | 24286300 | 24289949 | PNRC2 | | | | | |
| 1 | 24290836 | 24306953 | SRSF10 | | | | | |
| 1 | 24382530 | 24438665 | MYOM3 | | | | | |
| 1 | 24446260 | 24469775 | IL22RA1 | | | | | |
| 1 | 24480646 | 24513765 | IFNLR1 | | | | | |
| 1 | 24526729 | 24538180 | LOC284632 | | | | | |
| 1 | 24645811 | 24681808 | GRHL3 | | | | | |
| 1 | 24683488 | 24741587 | STPG1 | | | | | |
| 1 | 24742244 | 24799473 | NIPAL3 | | | | | |
| 1 | 24822822 | 24828850 | RCAN3AS | | | | | |
| 1 | 24828840 | 24863510 | RCAN3 | | | | | |

| | | | | | | | | | |
|---|---|---|---|---|---|---|---|---|---|
| 1 | 24865763 | 24882515 | LOC100506985 | | | | | | |
| 1 | 24882566 | 24935818 | NCMAP | | | | | | |
| 1 | 24969593 | 24999772 | SRRM1 | | | | | | |
| 1 | 25071759 | 25170815 | CLIC4 | | | | CLIC4 | | |
| 1 | 25226001 | 25291501 | RUNX3 | | | | | | |
| 1 | 25245835 | 25245907 | MIR6731 | | | | | | |
| 1 | 25349993 | 25350077 | MIR4425 | | | | | | |
| 1 | 25548766 | 25559013 | SYF2 | | | | | | |
| 1 | 25568739 | 25573985 | RSRP1 | | | | | | |
| 1 | 25598976 | 25656936 | RHD | | | | | | |
| 1 | 25664788 | 25688852 | TMEM50A | | | | | | |
| 1 | 25688739 | 25747363 | RHCE | | | | RHCE | | |
| 1 | 25757348 | 25826698 | TMEM57 | | | | | | |
| 1 | 25870075 | 25895377 | LDLRAP1 | | | | | | |
| 1 | 25943958 | 26112698 | MAN1C1 | | | | | | |
| 1 | 26126666 | 26144713 | SEPN1 | | | | | | |
| 1 | 26146396 | 26159433 | MTFR1L | | | | | | |
| 1 | 26146444 | 26150097 | LOC646471 | | | | | | |
| 1 | 26146683 | 26159433 | MTFR1L | | | | | | |
| 1 | 26158403 | 26185949 | AUNIP | | | | | | |
| 1 | 26187974 | 26197744 | PAQR7 | | | | | | |
| 1 | 26210676 | 26233368 | STMN1 | | | | | | |
| 1 | 26232852 | 26232945 | MIR3917 | | | | | | |
| 1 | 26286257 | 26324648 | PAFAH2 | | | | | | |
| 1 | 26348270 | 26362954 | EXTL1 | | | | | | |
| 1 | 26364512 | 26372629 | SLC30A2 | | | | | | |
| 1 | 26377795 | 26394125 | TRIM63 | | | | | | |
| 1 | 26437655 | 26452039 | PDIK1L | | | | | | |
| 1 | 26485510 | 26489119 | FAM110D | | | | | | |
| 1 | 26496387 | 26497364 | ZNF593 | | | | | | |
| 1 | 26503980 | 26516376 | CNKSR1 | | | | | | |
| 1 | 26517118 | 26529033 | CATSPER4 | | | | | | |
| 1 | 26551810 | 26556331 | LOC101928303 | | | | | | |
| 1 | 26560643 | 26605301 | CEP85 | | | | | | |
| 1 | 26606212 | 26608013 | SH3BGRL3 | | | | | | |
| 1 | 26608772 | 26644756 | UBXN11 | | | | | | |
| 1 | 26644410 | 26647014 | CD52 | | | | | | |
| 1 | 26648349 | 26680621 | AIM1L | | | | | | |
| 1 | 26688124 | 26699285 | ZNF683 | | | | | | |
| 1 | 26737258 | 26756219 | LIN28A | | | | | | |
| 1 | 26758772 | 26797795 | DHDDS | | | | | | |
| 1 | 26789247 | 26794028 | LOC101928324 | | | | | | |
| 1 | 26798901 | 26803133 | HMGN2 | | | | | | |
| 1 | 26856248 | 26901520 | RPS6KA1 | | | | | | |
| 1 | 26881032 | 26881084 | MIR1976 | | | | | | |
| 1 | 27022521 | 27108601 | ARID1A | | | | | | |
| 1 | 27114453 | 27124894 | PIGV | | | | | | |
| 1 | 27153200 | 27182211 | ZDHHC18 | | | | | | |
| 1 | 27189632 | 27190947 | SFN | | | | SFN | | |
| 1 | 27205872 | 27216869 | GPN2 | | | | | | |
| 1 | 27216978 | 27226962 | GPATCH3 | | | | | | |
| 1 | 27237974 | 27240567 | NR0B2 | | | | | | |
| 1 | 27248212 | 27273362 | NUDC | | | | | | |
| 1 | 27276046 | 27286901 | KDF1 | | | | | | |
| 1 | 27320194 | 27327377 | TRNP1 | | | | | | |
| 1 | 27331510 | 27339333 | FAM46B | | | | | | |
| 1 | 27425299 | 27481621 | SLC9A1 | | | | | | |
| 1 | 27561006 | 27635124 | WDTC1 | | | | | | |
| 1 | 27648635 | 27662891 | TMEM222 | | | | | | |
| 1 | 27650364 | 27653016 | ACTG1P20 | | | | | | |
| 1 | 27668482 | 27680423 | SYTL1 | | | | | | |
| 1 | 27681669 | 27693357 | MAP3K6 | | | | | | |
| 1 | 27695600 | 27701315 | FCN3 | | | | | | |
| 1 | 27705589 | 27709823 | CD164L2 | | | | | | |
| 1 | 27719147 | 27722317 | GPR3 | | | | | | |
| 1 | 27730729 | 27816698 | WASF2 | | | | | | |

| | | | | | | | | |
|---|---|---|---|---|---|---|---|---|
| 1 | 27860755 | 27930005 | AHDC1 | | | | | |
| 1 | 27938800 | 27961727 | FGR | | | | | |
| 1 | 27992571 | 27998724 | IFI6 | | | | | |
| 1 | 28052489 | 28089423 | FAM76A | | | | | |
| 1 | 28099693 | 28150963 | STX12 | | | | | |
| 1 | 28157251 | 28178183 | PPP1R8 | | | | | |
| 1 | 28160911 | 28161077 | SCARNA1 | | | | | |
| 1 | 28199053 | 28213196 | THEMIS2 | | | | | |
| 1 | 28218035 | 28241308 | RPA2 | | | | | |
| 1 | 28261465 | 28285668 | SMPDL3B | | | | | |
| 1 | 28285972 | 28294607 | XKR8 | | | | | |
| 1 | 28296854 | 28415148 | EYA3 | | | | | |
| 1 | 28473676 | 28520447 | PTAFR | | | | | |
| 1 | 28526789 | 28559542 | DNAJC8 | | | | | |
| 1 | 28562601 | 28564616 | ATPIF1 | | | | | |
| 1 | 28585962 | 28609002 | SESN2 | | | | | |
| 1 | 28655512 | 28662478 | MED18 | | | | | |
| 1 | 28696092 | 28826881 | PHACTR4 | | | | | |
| 1 | 28832454 | 28837404 | SNHG3 | | | | | |
| 1 | 28832454 | 28865708 | RCC1 | | | | | |
| 1 | 28835069 | 28835227 | SNORA73B | | | | | |
| 1 | 28844744 | 28865708 | RCC1 | | | | | |
| 1 | 28879528 | 28905057 | TRNAU1AP | | | | | |
| 1 | 28905049 | 28908366 | SNHG12 | | | | | |
| 1 | 28905254 | 28905334 | SNORD99 | | | | | |
| 1 | 28906275 | 28906405 | SNORA61 | | | | | |
| 1 | 28906892 | 28907024 | SNORA44 | | | | | |
| 1 | 28907431 | 28907565 | SNORA16A | | | | | |
| 1 | 28918711 | 28921088 | RAB42 | | | | | |
| 1 | 28929608 | 28969604 | TAF12 | | | | | |
| 1 | 28975111 | 28975246 | RNU11 | | | | | |
| 1 | 28995239 | 29042115 | GMEB1 | | | | | |
| 1 | 29063132 | 29096287 | YTHDF2 | | | | | |
| 1 | 29138653 | 29190208 | OPRD1 | | | | | |
| 1 | 29213602 | 29391733 | EPB41 | EPB41 | | | | |
| 1 | 29445936 | 29450421 | TMEM200B | | | | | |
| 1 | 29474249 | 29508637 | SRSF4 | | | | | |
| 1 | 29519168 | 29557470 | MECR | | | | | |
| 1 | 29563027 | 29653325 | PTPRU | | PTPRU | | | |
| 1 | 29656131 | 29676626 | LOC101928460 | | | | | |
| 1 | 30486798 | 30510459 | LOC101929406 | | | | | |
| 1 | 31184123 | 31199593 | MATN1 | | | | | |
| 1 | 31205314 | 31230683 | LAPTM5 | | | | | |
| 1 | 31212002 | 31212079 | MIR4420 | | | | | |
| 1 | 31342312 | 31381480 | SDC3 | | SDC3 | | | |
| 1 | 31404352 | 31538564 | PUM1 | | | | | |
| 1 | 31408535 | 31408623 | SNORD103A | | | | | |
| 1 | 31408535 | 31408623 | SNORD103B | | | | | |
| 1 | 31421964 | 31422052 | SNORD103A | | | | | |
| 1 | 31421964 | 31422052 | SNORD103B | | | | | |
| 1 | 31441009 | 31441084 | SNORD103C | | | | | |
| 1 | 31652591 | 31712734 | NKAIN1 | | | | | |
| 1 | 31732414 | 31769644 | SNRNP40 | | | | | |
| 1 | 31769828 | 31837800 | ZCCHC17 | | | | | |
| 1 | 31838099 | 31845923 | FABP3 | | | | | |
| 1 | 31882411 | 31907527 | SERINC2 | | | | | |
| 1 | 31971838 | 31974167 | LINC01225 | | | | | |
| 1 | 31984035 | 31989846 | LINC01226 | | | | | |
| 1 | 32042085 | 32053287 | TINAGL1 | | | | | |
| 1 | 32083300 | 32092919 | HCRTR1 | | | HCRTR1 | | |
| 1 | 32095462 | 32110838 | PEF1 | | | | | |
| 1 | 32117847 | 32169768 | COL16A1 | | | | | |
| 1 | 32192705 | 32229664 | ADGRB2 | | | | | |
| 1 | 32224260 | 32224336 | MIR4254 | | | | | |
| 1 | 32256022 | 32281652 | SPOCD1 | | | | | |
| 1 | 32372021 | 32403988 | PTP4A2 | | | | | |

| | | | | | | | | | |
|---|---|---|---|---|---|---|---|---|---|
| 1 | 32479294 | 32526460 | KHDRBS1 | | | | | | |
| 1 | 32537631 | 32568467 | TMEM39B | | | | | | |
| 1 | 32552549 | 32552608 | MIR5585 | | | | | | |
| 1 | 32573643 | 32642168 | KPNA6 | | | | | | |
| 1 | 32645344 | 32663886 | TXLNA | | | | | | |
| 1 | 32665986 | 32670991 | CCDC28B | | | | | | |
| 1 | 32671235 | 32674288 | IQCC | | | | | | |
| 1 | 32674694 | 32681797 | DCDC2B | | | | | | |
| 1 | 32680077 | 32687972 | TMEM234 | | | | | | |
| 1 | 32687184 | 32697205 | EIF3I | | | | | | |
| 1 | 32697260 | 32707311 | MTMR9LP | | | | | | |
| 1 | 32712817 | 32714461 | FAM167B | | | | | | |
| 1 | 32716839 | 32751768 | LCK | | | LCK | | | |
| 1 | 32757707 | 32799224 | HDAC1 | | | | | | |
| 1 | 32799429 | 32801840 | MARCKSL1 | | | | | | |
| 1 | 32826870 | 32827844 | FAM229A | | | | | | |
| 1 | 32827861 | 32829924 | TSSK3 | | | | | | |
| 1 | 32830233 | 32860062 | BSDC1 | | | | | | |
| 1 | 32930657 | 32953459 | ZBTB8B | | | | | | |
| 1 | 33004745 | 33071551 | ZBTB8A | | | | | | |
| 1 | 33086816 | 33116191 | ZBTB8OS | | | | | | |
| 1 | 33116748 | 33151812 | RBBP4 | | | | | | |
| 1 | 33145506 | 33168361 | SYNC | | | | | | SYNC |
| 1 | 33207511 | 33240571 | KIAA1522 | | | | | | |
| 1 | 33240839 | 33283633 | YARS | | YARS | | | | |
| 1 | 33283042 | 33324480 | S100PBP | | | | | | |
| 1 | 33327868 | 33338093 | FNDC5 | | | | | | |
| 1 | 33352097 | 33360247 | HPCA | | HPCA | | | | |
| 1 | 33360195 | 33366953 | TMEM54 | | | | | | |
| 1 | 33402046 | 33430414 | RNF19B | | | | | | |
| 1 | 33473540 | 33502512 | AK2 | | | | | | |
| 1 | 33546713 | 33586132 | AZIN2 | | | | | | |
| 1 | 33611002 | 33647671 | TRIM62 | | | | | | |
| 1 | 33722173 | 33766320 | ZNF362 | | | | | | |
| 1 | 33772366 | 33786699 | A3GALT2 | | | | | | |
| 1 | 33789223 | 33841194 | PHC2 | | | | | | |
| 1 | 33797993 | 33798093 | MIR3605 | | | | | | |
| 1 | 33815952 | 33828846 | LOC101929464 | | | | | | |
| 1 | 33938231 | 33961995 | ZSCAN20 | | | | | | |
| 1 | 33979598 | 34631443 | CSMD2 | | | | | | |
| 1 | 34326075 | 34330392 | HMGB4 | | | | | | |
| 1 | 34334556 | 34351059 | CSMD2 | | | | | | |
| 1 | 34632483 | 34684731 | C1orf94 | | | | | | |
| 1 | 35135199 | 35135295 | MIR552 | | | | | | |
| 1 | 35220647 | 35224113 | GJB5 | | | GJB5 | | | |
| 1 | 35225341 | 35229325 | GJB4 | | | | | | |
| 1 | 35246789 | 35251967 | GJB3 | | | | | | |
| 1 | 35258598 | 35261348 | GJA4 | | | | | | |
| 1 | 35315962 | 35325417 | SMIM12 | | | | | | |
| 1 | 35331036 | 35395186 | DLGAP3 | | | | | | |
| 1 | 35441299 | 35444307 | LOC653160 | | | | | | |
| 1 | 35447126 | 35450948 | ZMYM6NB | | | | | | |
| 1 | 35451766 | 35497569 | ZMYM6 | | | | | | |
| 1 | 35525386 | 35581459 | ZMYM1 | | | | | | |
| 1 | 35649200 | 35658743 | SFPQ | | | | | | |
| 1 | 35734567 | 35887545 | ZMYM4 | | | | | | |
| 1 | 35899090 | 36023037 | KIAA0319L | | | | | | |
| 1 | 36023392 | 36032380 | NCDN | | | | | | |
| 1 | 36038970 | 36060927 | TFAP2E | | | | | | |
| 1 | 36065142 | 36107445 | PSMB2 | | | | | | |
| 1 | 36179476 | 36184790 | C1orf216 | | | | | | |
| 1 | 36197712 | 36235551 | CLSPN | | | | | | |
| 1 | 36273827 | 36323490 | AGO4 | | | | | | |
| 1 | 36335408 | 36389899 | AGO1 | | | | | | |
| 1 | 36396682 | 36522063 | AGO3 | | | | | | |
| 1 | 36549675 | 36553876 | TEKT2 | | | TEKT2 | | | |

| | | | | | | | | |
|---|---|---|---|---|---|---|---|---|
| 1 | 36554452 | 36559533 | ADPRHL2 | | | | | |
| 1 | 36560836 | 36590685 | COL8A2 | | | | | |
| 1 | 36602169 | 36621654 | TRAPPC3 | | | | | |
| 1 | 36621565 | 36646451 | MAP7D1 | | | | | |
| 1 | 36690016 | 36770957 | THRAP3 | | | | | |
| 1 | 36771993 | 36786948 | SH3D21 | | | | | |
| 1 | 36787630 | 36789755 | EVA1B | | | | | |
| 1 | 36805219 | 36851528 | STK40 | | | | | |
| 1 | 36859020 | 36863560 | LSM10 | | | | | |
| 1 | 36883506 | 36916086 | OSCP1 | | | | | |
| 1 | 36921361 | 36930040 | MRPS15 | | | | | |
| 1 | 36931643 | 36948915 | CSF3R | | | | | |
| 1 | 37261127 | 37499844 | GRIK3 | | | | GRIK3 | |
| 1 | 37627163 | 37627235 | MIR4255 | | | | | |
| 1 | 37920479 | 37940044 | LINC01137 | | | | | |
| 1 | 37940118 | 37949978 | ZC3H12A | | | | | |
| 1 | 37945830 | 37945890 | MIR6732 | | | | | |
| 1 | 37955560 | 37980420 | MEAF6 | | | | | |
| 1 | 37966535 | 37966595 | MIR5581 | | | | | |
| 1 | 38000049 | 38019945 | SNIP1 | | | | | |
| 1 | 38022519 | 38032458 | DNALI1 | | | | | |
| 1 | 38032412 | 38061586 | GNL2 | | | | | |
| 1 | 38076950 | 38100595 | RSPO1 | | | | | |
| 1 | 38147241 | 38156267 | C1orf109 | | | | | |
| 1 | 38158072 | 38175391 | CDCA8 | | | | | |
| 1 | 38181645 | 38230824 | EPHA10 | | | | | |
| 1 | 38259773 | 38267278 | MANEAL | | | | | |
| 1 | 38268613 | 38273865 | YRDC | | | | | |
| 1 | 38273472 | 38275126 | C1orf122 | | | | | |
| 1 | 38275238 | 38325292 | MTF1 | | | | | |
| 1 | 38326368 | 38412729 | INPP5B | | | | | |
| 1 | 38422651 | 38455761 | SF3A3 | | | | | |
| 1 | 38462441 | 38471187 | FHL3 | | | | | |
| 1 | 38478383 | 38490497 | UTP11L | | | | | |
| 1 | 38509522 | 38512450 | POU3F1 | | | | | |
| 1 | 38554902 | 38555001 | MIR3659 | | | | | |
| 1 | 38674705 | 38680439 | LINC01343 | | | | | |
| 1 | 39303868 | 39325495 | RRAGC | | | | | |
| 1 | 39328161 | 39339050 | MYCBP | | | | | |
| 1 | 39328161 | 39347298 | GJA9-MYCBP | | | | | |
| 1 | 39339072 | 39341898 | LOC105378663 | | | | | |
| 1 | 39339738 | 39347298 | GJA9 | | | GJA9 | | |
| 1 | 39351477 | 39407502 | RHBDL2 | | | | | |
| 1 | 39456915 | 39471737 | AKIRIN1 | | | | | |
| 1 | 39491966 | 39500308 | NDUFS5 | | | | | |
| 1 | 39549838 | 39952810 | MACF1 | | | | | |
| 1 | 39875175 | 39882154 | KIAA0754 | | | | | |
| 1 | 39957317 | 39995541 | BMP8A | | | | | |
| 1 | 39980537 | 39982341 | OXCT2P1 | | | | | |
| 1 | 39987951 | 40025370 | PPIEL | | | | | |
| 1 | 40026484 | 40042521 | PABPC4 | | | | | |
| 1 | 40030741 | 40038875 | LOC101929516 | | | | | |
| 1 | 40033045 | 40033182 | SNORA55 | | | | | |
| 1 | 40089102 | 40105348 | HEYL | | | | | |
| 1 | 40124792 | 40137710 | NT5C1A | | | | | |
| 1 | 40144319 | 40157382 | HPCAL4 | | | | | |
| 1 | 40204516 | 40229586 | PPIE | | | | | |
| 1 | 40223902 | 40254533 | BMP8B | | | | | |
| 1 | 40235196 | 40237020 | OXCT2 | | | | | |
| 1 | 40306705 | 40349183 | TRIT1 | | | | | |
| 1 | 40361095 | 40367687 | MYCL | | | | | |
| 1 | 40420783 | 40435640 | MFSD2A | | | | | |
| 1 | 40506254 | 40538321 | CAP1 | | | | | |
| 1 | 40538381 | 40563142 | PPT1 | PPT1 | | | | PPT1 |
| 1 | 40627040 | 40706593 | RLF | | | | | |
| 1 | 40713572 | 40717365 | TMCO2 | | | | | |

| | | | | | | | | |
|---|---|---|---|---|---|---|---|---|
| 1 | 40723721 | 40759856 | ZMPSTE24 | | | | | |
| 1 | 40766162 | 40782939 | COL9A2 | | COL9A2 | COL9A2 | | |
| 1 | 40839377 | 40888998 | SMAP2 | | | | | |
| 1 | 40916336 | 40929390 | ZFP69B | | | | | |
| 1 | 40943301 | 40962015 | ZFP69 | | | | | |
| 1 | 40974432 | 40982214 | EXO5 | | | | | |
| 1 | 40997232 | 41013841 | ZNF684 | | | | | |
| 1 | 41086351 | 41131324 | RIMS3 | | | | | |
| 1 | 41154751 | 41237276 | NFYC | | | | | |
| 1 | 41220026 | 41220118 | MIR30E | | | | | |
| 1 | 41222955 | 41223044 | MIR30C1 | | | | | |
| 1 | 41249683 | 41306124 | KCNQ4 | | | | | |
| 1 | 41326727 | 41328018 | CITED4 | | | | | |
| 1 | 41444970 | 41478237 | CTPS1 | | | | | |
| 1 | 41480261 | 41487427 | SLFNL1 | | | | | |
| 1 | 41492870 | 41707815 | SCMH1 | | | | | |
| 1 | 41827602 | 41849263 | FOXO6 | | | | | |
| 1 | 41944445 | 41950354 | EDN2 | | | | | |
| 1 | 41972035 | 42501596 | HIVEP3 | | | | | |
| 1 | 42619091 | 42621495 | GUCA2B | | | | | |
| 1 | 42628361 | 42630395 | GUCA2A | | | | | |
| 1 | 42642209 | 42801548 | FOXJ3 | | | | | |
| 1 | 42846467 | 42889900 | RIMKLA | | | | | |
| 1 | 42896000 | 42921938 | ZMYND12 | | | | | |
| 1 | 42922011 | 42939049 | PPCS | | | | | |
| 1 | 43000559 | 43120335 | CCDC30 | | | | | |
| 1 | 43124047 | 43142429 | PPIH | | | | | |
| 1 | 43147905 | 43169474 | YBX1 | | | | | |
| 1 | 43198763 | 43205925 | CLDN19 | | | | | |
| 1 | 43212005 | 43232755 | P3H1 | | | | | |
| 1 | 43232915 | 43241413 | C1orf50 | | | | | |
| 1 | 43242336 | 43250566 | LOC105378683 | | | | | |
| 1 | 43253660 | 43263901 | LOC100129924 | | | | | |
| 1 | 43272722 | 43283059 | SVBP | | | | | |
| 1 | 43282775 | 43310660 | ERMAP | | | | | |
| 1 | 43312243 | 43318146 | ZNF691 | | | | | |
| 1 | 43323292 | 43354463 | LOC339539 | | | | | |
| 1 | 43391045 | 43449029 | SLC2A1 | | SLC2A1 | | | |
| 1 | 43613593 | 43622067 | FAM183A | | | | | |
| 1 | 43629844 | 43638241 | EBNA1BP2 | | | | | |
| 1 | 43637322 | 43637383 | MIR6733 | | | | | |
| 1 | 43638000 | 43720029 | CFAP57 | | | | | |
| 1 | 43735664 | 43739673 | TMEM125 | | | | | |
| 1 | 43747556 | 43751288 | C1orf210 | | | | | |
| 1 | 43766565 | 43788781 | TIE1 | | | | | |
| 1 | 43803474 | 43820135 | MPL | | | MPL | | |
| 1 | 43824625 | 43828873 | CDC20 | | | | | |
| 1 | 43829067 | 43833745 | ELOVL1 | | | | | |
| 1 | 43830318 | 43830386 | MIR6734 | | | | | |
| 1 | 43849578 | 43855483 | MED8 | | | | | |
| 1 | 43855555 | 43919918 | SZT2 | | | | | |
| 1 | 43914209 | 43914282 | MIR6735 | | | | | |
| 1 | 43916673 | 43919660 | HYI | | | | | |
| 1 | 43996546 | 44089343 | PTPRF | | | | | |
| 1 | 44115796 | 44173012 | KDM4A | | | | | |
| 1 | 44173203 | 44396837 | ST3GAL3 | | | | | |
| 1 | 44175075 | 44193014 | LOC101929592 | | | | | |
| 1 | 44304293 | 44304355 | MIR6079 | | | | | |
| 1 | 44398991 | 44402912 | ARTN | | | | | |
| 1 | 44412477 | 44433694 | IPO13 | | | | | |
| 1 | 44435652 | 44439043 | DPH2 | | | | | |
| 1 | 44440319 | 44443972 | ATP6V0B | | | | | |
| 1 | 44444873 | 44456843 | B4GALT2 | | | | | |
| 1 | 44457279 | 44462198 | CCDC24 | | | | | |
| 1 | 44462154 | 44497164 | SLC6A9 | | | | | |
| 1 | 44584521 | 44600809 | KLF17 | | | | | |

| | | | | | | | | |
|---|---|---|---|---|---|---|---|---|
| 1 | 44679124 | 44686351 | DMAP1 | | | | | |
| 1 | 44686741 | 44820951 | ERI3 | | | | | |
| 1 | 44688266 | 44709955 | ERI3-IT1 | | | | | |
| 1 | 44718015 | 44718231 | SNORA110 | | | | | |
| 1 | 44870831 | 45117396 | RNF220 | | | | | |
| 1 | 45011164 | 45011224 | MIR5584 | | | | | |
| 1 | 45118918 | 45140280 | TMEM53 | | | | | |
| 1 | 45140393 | 45191263 | C1orf228 | | | | | |
| 1 | 45205489 | 45233438 | KIF2C | | | | | |
| 1 | 45241245 | 45244412 | RPS8 | | | | | |
| 1 | 45241536 | 45241610 | SNORD55 | | | | | |
| 1 | 45242163 | 45242261 | SNORD46 | | | | | |
| 1 | 45243513 | 45243584 | SNORD38A | | | | | |
| 1 | 45244061 | 45244130 | SNORD38B | | | | | |
| 1 | 45249256 | 45253426 | BEST4 | | | | | |
| 1 | 45265896 | 45271667 | PLK3 | | | | | |
| 1 | 45271581 | 45272957 | TCTEX1D4 | | | | | |
| 1 | 45274153 | 45279801 | BTBD19 | | | | | |
| 1 | 45285515 | 45308616 | PTCH2 | | | PTCH2 | | |
| 1 | 45316193 | 45452394 | EIF2B3 | | | | | |
| 1 | 45468219 | 45477027 | HECTD3 | | | | | |
| 1 | 45477804 | 45481341 | UROD | | | | | |
| 1 | 45482075 | 45672250 | ZSWIM5 | | | | | |
| 1 | 45769581 | 45771291 | LINC01144 | | | | | |
| 1 | 45792544 | 45794346 | HPDL | | | | | |
| 1 | 45794913 | 45806142 | MUTYH | | | | | |
| 1 | 45805341 | 45809650 | TOE1 | | | | | |
| 1 | 45809554 | 45956840 | TESK2 | | | | | |
| 1 | 45959597 | 45965751 | CCDC163P | | | | | |
| 1 | 45965855 | 45976739 | MMACHC | | | | | |
| 1 | 45976706 | 45988562 | PRDX1 | | | | | |
| 1 | 46016454 | 46035723 | AKR1A1 | | | | | |
| 1 | 46049659 | 46084578 | NASP | | | | | |
| 1 | 46085715 | 46089731 | CCDC17 | | | | | |
| 1 | 46092975 | 46152302 | GPBP1L1 | | | | | |
| 1 | 46111451 | 46112357 | RPS15AP10 | | | | | |
| 1 | 46153846 | 46160108 | TMEM69 | | | | | |
| 1 | 46159997 | 46216485 | IPP | | | | | |
| 1 | 46269284 | 46501796 | MAST2 | | | MAST2 | | |
| 1 | 46505811 | 46642167 | PIK3R3 | | | | | |
| 1 | 46599042 | 46604752 | LOC101929626 | | | | | |
| 1 | 46640748 | 46651634 | TSPAN1 | | | | | |
| 1 | 46654352 | 46685977 | POMGNT1 | | | | | |
| 1 | 46669005 | 46686928 | LURAP1 | | | | | |
| 1 | 46713366 | 46744145 | RAD54L | | | | | |
| 1 | 46744071 | 46769038 | LRRC41 | | | | | |
| 1 | 46769284 | 46782449 | UQCRH | | | | | |
| 1 | 46805848 | 46830824 | NSUN4 | | | | | |
| 1 | 46859938 | 46879520 | FAAH | | FAAH | | | |
| 1 | 46899498 | 46911374 | FAAHP1 | | | | | |
| 1 | 46912344 | 46915376 | LINC01398 | | | | | |
| 1 | 46972667 | 46979886 | DMBX1 | | | | | |
| 1 | 47004367 | 47035927 | MKNK1 | | | | | |
| 1 | 47011315 | 47016887 | KNCN | | | | | |
| 1 | 47023078 | 47069966 | MKNK1 | | | | | |
| 1 | 47073386 | 47082563 | MOB3C | | | | | |
| 1 | 47100710 | 47134099 | ATPAF1 | | | | | |
| 1 | 47137504 | 47139256 | TEX38 | | | | | |
| 1 | 47139707 | 47184736 | EFCAB14 | | | | | |
| 1 | 47264669 | 47285021 | CYP4B1 | | | | | |
| 1 | 47308766 | 47366147 | CYP4Z2P | | | | | |
| 1 | 47394845 | 47407156 | CYP4A11 | | | | | |
| 1 | 47489239 | 47516423 | CYP4X1 | | | | | |
| 1 | 47533159 | 47583992 | CYP4Z1 | | | | | |
| 1 | 47603096 | 47614526 | CYP4A22 | | | | | |
| 1 | 47644921 | 47646011 | LINC00853 | | | | | |

| | | | | | | | | |
|---|---|---|---|---|---|---|---|---|
| 1 | 47649260 | 47655771 | PDZK1IP1 | | | | | |
| 1 | 47681961 | 47698007 | TAL1 | | | | | |
| 1 | 47715810 | 47779819 | STIL | | | | | |
| 1 | 47799468 | 47844511 | CMPK1 | | | | | |
| 1 | 47846467 | 47902989 | LINC01389 | | | | | |
| 1 | 47881743 | 47883724 | FOXE3 | | | | | |
| 1 | 47897806 | 47906363 | FOXD2 | | | | | |
| 1 | 48226199 | 48462562 | TRABD2B | | | | | |
| 1 | 48567386 | 48648100 | SKINT1L | | | | | |
| 1 | 48688356 | 48714316 | SLC5A9 | | | | | |
| 1 | 48761043 | 48937880 | SPATA6 | | | | | |
| 1 | 48998526 | 50489626 | AGBL4 | | | | | |
| 1 | 49193194 | 49242641 | BEND5 | | | | | |
| 1 | 49723082 | 49734966 | LOC101929721 | | | | | |
| 1 | 49839872 | 49937757 | AGBL4-IT1 | | | | | |
| 1 | 50513685 | 50669457 | ELAVL4 | | | | | |
| 1 | 50883222 | 50889119 | DMRTA2 | | | | | |
| 1 | 50906934 | 51425936 | FAF1 | | | FAF1 | | |
| 1 | 51434366 | 51440309 | CDKN2C | | | | | |
| 1 | 51525508 | 51525577 | MIR4421 | | | | | |
| 1 | 51525689 | 51525775 | MIR6500 | | | | | |
| 1 | 51567905 | 51613754 | C1orf185 | | | | | |
| 1 | 51701944 | 51739119 | RNF11 | | | | | |
| 1 | 51752929 | 51796953 | TTC39A | | | | | |
| 1 | 51819934 | 51984995 | EPS15 | | | | | |
| 1 | 52082545 | 52254891 | OSBPL9 | | | | | |
| 1 | 52254865 | 52344609 | NRDC | | | | | |
| 1 | 52302015 | 52302074 | MIR761 | | | | | |
| 1 | 52373627 | 52456436 | RAB3B | | | | | |
| 1 | 52485803 | 52521843 | TXNDC12 | | | | | |
| 1 | 52497776 | 52499472 | KTI12 | | | | | |
| 1 | 52516589 | 52518355 | TXNDC12 | | | | | |
| 1 | 52521856 | 52556388 | BTF3L4 | | | | | |
| 1 | 52607765 | 52812358 | ZFYVE9 | | | ZFYVE9 | | |
| 1 | 52816264 | 52831877 | CC2D1B | | | | | |
| 1 | 52838500 | 52870143 | ORC1 | | | | | |
| 1 | 52870218 | 52883992 | PRPF38A | | | | | |
| 1 | 52888947 | 53018762 | ZCCHC11 | | | | | |
| 1 | 53068042 | 53074723 | GPX7 | | | | | |
| 1 | 53099065 | 53122737 | FAM159A | | | | | |
| 1 | 53152013 | 53164038 | COA7 | | | | | |
| 1 | 53192130 | 53293013 | ZYG11B | | | | | |
| 1 | 53308182 | 53360247 | ZYG11A | | | | | |
| 1 | 53361581 | 53387457 | ECHDC2 | | | | | |
| 1 | 53392900 | 53517289 | SCP2 | | | | | |
| 1 | 53394345 | 53394444 | MIR1273F | | | | | |
| 1 | 53400601 | 53400689 | MIR5095 | | | | | |
| 1 | 53405985 | 53406085 | MIR1273G | | | | | |
| 1 | 53480609 | 53517289 | SCP2 | | | | | |
| 1 | 53527723 | 53551174 | PODN | | | | | |
| 1 | 53552850 | 53608304 | SLC1A7 | | | | | |
| 1 | 53662100 | 53679869 | CPT2 | | | CPT2 | | |
| 1 | 53679771 | 53686311 | C1orf123 | | | | | |
| 1 | 53692563 | 53704282 | MAGOH | | | | | |
| 1 | 53704281 | 53708455 | LOC100507564 | | | | | |
| 1 | 53708040 | 53793821 | LRP8 | | LRP8 | | | |
| 1 | 53793904 | 53802181 | LOC105378732 | | | | | |
| 1 | 53904042 | 53905693 | SLC25A3P1 | | | | | |
| 1 | 53925071 | 53933160 | DMRTB1 | | | | | |
| 1 | 53971905 | 54199877 | GLIS1 | | | | | |
| 1 | 54231133 | 54304225 | NDC1 | | | | | |
| 1 | 54317391 | 54355504 | YIPF1 | | | | | |
| 1 | 54359860 | 54376759 | DIO1 | | | | | |
| 1 | 54387233 | 54411981 | HSPB11 | | | | | |
| 1 | 54411998 | 54433841 | LRRC42 | | | | | |
| 1 | 54472970 | 54483859 | LDLRAD1 | | | | | |

| | | | | | | | | |
|---|---|---|---|---|---|---|---|---|
| 1 | 54492353 | 54519111 | TMEM59 | | | | | |
| 1 | 54519244 | 54578192 | TCEANC2 | | | | | |
| 1 | 54519751 | 54519827 | MIR4781 | | | | | |
| 1 | 54604667 | 54618679 | CDCP2 | | | | | |
| 1 | 54638026 | 54665746 | CYB5RL | | | | | |
| 1 | 54665839 | 54684056 | MRPL37 | | | | | |
| 1 | 54691103 | 54704736 | SSBP3 | | | | | |
| 1 | 55013806 | 55100417 | ACOT11 | | | ACOT11 | | |
| 1 | 55074849 | 55089200 | FAM151A | | | | | |
| 1 | 55107412 | 55175940 | MROH7 | | | | | |
| 1 | 55107412 | 55208328 | MROH7-TTC4 | | | | | |
| 1 | 55181494 | 55208328 | TTC4 | | | | | |
| 1 | 55222570 | 55230226 | PARS2 | | | | | |
| 1 | 55246751 | 55266941 | TTC22 | | | | | |
| 1 | 55271735 | 55307937 | LEXM | | | | | |
| 1 | 55315299 | 55352921 | DHCR24 | | DHCR24 | | | |
| 1 | 55446336 | 55457966 | TMEM61 | | | | | |
| 1 | 55464616 | 55474465 | BSND | | | BSND | | |
| 1 | 55505148 | 55530526 | PCSK9 | | | | | |
| 1 | 55532031 | 55681039 | USP24 | | | | | |
| 1 | 55681080 | 55683128 | LOC100507634 | | | | | |
| 1 | 55691313 | 55691396 | MIR4422 | | | | | |
| 1 | 56960418 | 57045257 | PLPP3 | | | | | |
| 1 | 57093463 | 57110991 | LOC101929935 | | | | | |
| 1 | 57110989 | 57181008 | PRKAA2 | | | PRKAA2 | | |
| 1 | 57184476 | 57285369 | C1orf168 | | | | | |
| 1 | 57320442 | 57383894 | C8A | | | C8A | | |
| 1 | 57394882 | 57431813 | C8B | | | | | |
| 1 | 57463578 | 58328786 | DAB1 | | | | | |
| 1 | 58946390 | 59012471 | OMA1 | | | | | |
| 1 | 59041094 | 59043166 | TACSTD2 | | | | | |
| 1 | 59120410 | 59165747 | MYSM1 | | | | | |
| 1 | 59246462 | 59249785 | JUN | JUN | | | | |
| 1 | 59250822 | 59365384 | LINC01135 | | | | | |
| 1 | 59486147 | 59553919 | LINC01358 | | | | | |
| 1 | 59597607 | 59612479 | HSD52 | | | | | |
| 1 | 59762624 | 60228402 | FGGY | | | | | |
| 1 | 60198898 | 60198968 | MIR4711 | | | | | |
| 1 | 60238466 | 60254501 | LOC101926944 | | | | | |
| 1 | 60280532 | 60342050 | HOOK1 | | | HOOK1 | | |
| 1 | 60358979 | 60392470 | CYP2J2 | | CYP2J2 | | | |
| 1 | 60456065 | 60539442 | C1orf87 | | | | | |
| 1 | 61125302 | 61291256 | LOC101926964 | | | | | |
| 1 | 61405915 | 61719190 | NFIA | | | NFIA | | |
| 1 | 62119913 | 62121800 | MGC34796 | | | | | |
| 1 | 62146718 | 62191095 | TM2D1 | | | | | |
| 1 | 62208148 | 62629591 | INADL | | | | | |
| 1 | 62544457 | 62544528 | MIR3116 | | | | | |
| 1 | 62660473 | 62678001 | L1TD1 | | | | | |
| 1 | 62701836 | 62785083 | KANK4 | | | | | |
| 1 | 62901974 | 62917475 | USP1 | | | | | |
| 1 | 62920396 | 63154039 | DOCK7 | | | | | |
| 1 | 63063157 | 63071976 | ANGPTL3 | | | | | |
| 1 | 63249776 | 63330941 | ATG4C | | | | | |
| 1 | 63624753 | 63782928 | LINC00466 | | | | | |
| 1 | 63786554 | 63790797 | FOXD3 | | | | | |
| 1 | 63792595 | 63792655 | MIR6068 | | | | | |
| 1 | 63833260 | 63904233 | ALG6 | | | | | |
| 1 | 63906440 | 63988944 | ITGB3BP | | | | | |
| 1 | 63988971 | 64038364 | EFCAB7 | | | | | |
| 1 | 64014650 | 64016307 | DLEU2L | | | | | |
| 1 | 64058946 | 64125916 | PGM1 | | | | | |
| 1 | 64239689 | 64579474 | ROR1 | | | | | |
| 1 | 64669489 | 64710027 | UBE2U | | | | | |
| 1 | 64936475 | 65158741 | CACHD1 | | | | | |
| 1 | 65045529 | 65045604 | MIR4794 | | | | | |

| | | | | | | | | |
|---|---|---|---|---|---|---|---|---|
| 1 | 65210777 | 65298914 | RAVER2 | | | | | |
| 1 | 65298905 | 65432187 | JAK1 | | | | | |
| 1 | 65445259 | 65468159 | LINC01359 | | | | | |
| 1 | 65523437 | 65523525 | MIR3671 | | | | | |
| 1 | 65524116 | 65524191 | MIR101 | | | | | |
| 1 | 65613231 | 65697828 | AK4 | | | | | |
| 1 | 65730376 | 65881552 | DNAJC6 | | DNAJC6 | | | |
| 1 | 65886130 | 65901690 | LEPROT | | | | | |
| 1 | 65886334 | 66103176 | LEPR | | | LEPR | | |
| 1 | 65886398 | 65901690 | LEPROT | | | | | |
| 1 | 65991371 | 66101111 | LEPR | | | LEPR | | |
| 1 | 66258192 | 66840262 | PDE4B | | PDE4B | PDE4B | | |
| 1 | 66508182 | 66516401 | LOC101927139 | | | | | |
| 1 | 66797790 | 66840262 | PDE4B | | PDE4B | PDE4B | | |
| 1 | 66999251 | 67216822 | SGIP1 | | | | | |
| 1 | 67094122 | 67094200 | MIR3117 | | | | | |
| 1 | 67218139 | 67244730 | TCTEX1D1 | | | | | |
| 1 | 67263423 | 67266942 | INSL5 | | | | | |
| 1 | 67278571 | 67390570 | WDR78 | | | | | |
| 1 | 67390577 | 67454302 | MIER1 | | | | | |
| 1 | 67465014 | 67520080 | SLC35D1 | | | | | |
| 1 | 67557858 | 67600654 | C1orf141 | | | | | |
| 1 | 67632168 | 67725650 | IL23R | | | | | |
| 1 | 67773046 | 67862583 | IL12RB2 | | | IL12RB2 | | |
| 1 | 67873492 | 67896123 | SERBP1 | | | | | |
| 1 | 68150859 | 68154021 | GADD45A | | | | | |
| 1 | 68167148 | 68668670 | GNG12 | | | | | |
| 1 | 68511644 | 68517314 | DIRAS3 | | | | | |
| 1 | 68564141 | 68698284 | WLS | | | | | |
| 1 | 68649200 | 68649293 | MIR1262 | | | | | |
| 1 | 68894506 | 68915642 | RPE65 | | RPE65 | | | |
| 1 | 68939834 | 69004310 | DEPDC1 | | | | | |
| 1 | 70225857 | 70589171 | LRRC7 | | | | | |
| 1 | 70385004 | 70386000 | PIN1P1 | | | | | |
| 1 | 70610484 | 70671361 | LRRC40 | | | | | |
| 1 | 70671364 | 70717701 | SRSF11 | | | | | |
| 1 | 70724684 | 70820417 | ANKRD13C | | | | | |
| 1 | 70820492 | 70833705 | HHLA3 | | | | | |
| 1 | 70876900 | 70905534 | CTH | | | | | |
| 1 | 71172135 | 71252151 | LOC101927244 | | | | | |
| 1 | 71318035 | 71513491 | PTGER3 | | | | | |
| 1 | 71512188 | 71546972 | ZRANB2 | | | | | |
| 1 | 71533313 | 71533399 | MIR186 | | | | | |
| 1 | 71547006 | 71703406 | ZRANB2 | | | | | |
| 1 | 71868624 | 72748277 | NEGR1 | | | | | |
| 1 | 72259914 | 72302695 | NEGR1-IT1 | | | | | |
| 1 | 73771852 | 73804560 | LINC01360 | | | | | |
| 1 | 74491701 | 74663871 | LRRIQ3 | | | | | |
| 1 | 74663895 | 74674386 | FPGT | | | | | |
| 1 | 74663895 | 75010116 | FPGT-TNNI3K | | | | | |
| 1 | 74701070 | 75010116 | TNNI3K | | TNNI3K | | | |
| 1 | 75033794 | 75091782 | ERICH3 | | | | | |
| 1 | 75171171 | 75199092 | CRYZ | | | | | |
| 1 | 75198835 | 75232360 | TYW3 | | | | | |
| 1 | 75594118 | 75627218 | LHX8 | | | | | |
| 1 | 75667815 | 76076799 | SLC44A5 | | | | | |
| 1 | 76190031 | 76229363 | ACADM | | | ACADM | | |
| 1 | 76207686 | 76210698 | DLSTP1 | | | | | |
| 1 | 76251878 | 76260775 | RABGGTB | | | | | |
| 1 | 76252756 | 76252834 | SNORD45C | | | | | |
| 1 | 76253573 | 76253657 | SNORD45A | | | | | |
| 1 | 76255161 | 76255232 | SNORD45B | | | | | |
| 1 | 76262555 | 76378923 | MSH4 | | | | | |
| 1 | 76384557 | 76398116 | ASB17 | | | | | |
| 1 | 76477008 | 76485041 | LOC101927342 | | | | | |
| 1 | 76540388 | 77096669 | ST6GALNAC3 | | | | | |

| | | | | | | | | |
|---|---|---|---|---|---|---|---|---|
| 1 | 77333185 | 77529737 | ST6GALNAC5 | | | | | |
| 1 | 77525827 | 77525887 | MIR7156 | | | | | |
| 1 | 77554666 | 77685132 | PIGK | | | | | |
| 1 | 77747661 | 78025654 | AK5 | | | | | |
| 1 | 78028100 | 78149112 | ZZZ3 | | | | | |
| 1 | 78161673 | 78225564 | USP33 | | | | | |
| 1 | 78245308 | 78345225 | FAM73A | | | | | |
| 1 | 78347032 | 78409578 | NEXN | | | | | |
| 1 | 78412166 | 78444889 | FUBP1 | | | | | |
| 1 | 78444841 | 78483648 | DNAJB4 | | | | | |
| 1 | 78510645 | 78604133 | GIPC2 | | | | | |
| 1 | 78695282 | 78835147 | MGC27382 | | | | | |
| 1 | 78956727 | 79006386 | PTGFR | | | | | |
| 1 | 79086066 | 79111830 | IFI44L | | | | | |
| 1 | 79115476 | 79129763 | IFI44 | | | | | |
| 1 | 79355448 | 79472495 | ADGRL4 | | | | | |
| 1 | 81001439 | 81112473 | LOC101927412 | | | | | |
| 1 | 81979564 | 82023387 | LOC101927434 | | | | | |
| 1 | 82165454 | 82458422 | ADGRL2 | | | | | |
| 1 | 83439565 | 83451891 | LINC01361 | | | | | |
| 1 | 84041470 | 84326679 | LOC101927587 | | | | | |
| 1 | 84259597 | 84379059 | MIR548AP | | | | | |
| 1 | 84267198 | 84326229 | LOC101927560 | | | | | |
| 1 | 84335056 | 84464833 | TTLL7 | | | | | |
| 1 | 84543657 | 84704181 | PRKACB | | PRKACB | PRKACB | | |
| 1 | 84764048 | 84816481 | SAMD13 | | | | | |
| 1 | 84830640 | 84850484 | UOX | | | | | |
| 1 | 84864214 | 84880691 | DNASE2B | | | | | |
| 1 | 84944919 | 84964033 | RPF1 | | | | | |
| 1 | 84964005 | 84972262 | GNG5 | | | | | |
| 1 | 84971983 | 85022178 | SPATA1 | | | | | |
| 1 | 85018803 | 85040163 | CTBS | | | | | |
| 1 | 85063646 | 85086714 | LINC01461 | | | | | |
| 1 | 85093912 | 85100703 | LINC01555 | | | | | |
| 1 | 85109389 | 85156240 | SSX2IP | | | | | |
| 1 | 85279085 | 85358896 | LPAR3 | | | | | |
| 1 | 85391265 | 85462805 | MCOLN2 | | | | | |
| 1 | 85483764 | 85514223 | MCOLN3 | | | | | |
| 1 | 85527980 | 85598821 | WDR63 | | | | | |
| 1 | 85599476 | 85599556 | MIR4423 | | | | | |
| 1 | 85623355 | 85666728 | SYDE2 | | | | | |
| 1 | 85715636 | 85725355 | C1orf52 | | | | | |
| 1 | 85731459 | 85742587 | BCL10 | | | | | |
| 1 | 85742040 | 85743771 | LOC646626 | | | | | |
| 1 | 85784167 | 86044046 | DDAH1 | | | | | |
| 1 | 86046443 | 86049648 | CYR61 | | | | | |
| 1 | 86115105 | 86174116 | ZNHIT6 | | | | | |
| 1 | 86194915 | 86622121 | COL24A1 | | | | | |
| 1 | 86812506 | 86862025 | ODF2L | | | | | |
| 1 | 86823314 | 86823370 | MIR7856 | | | | | |
| 1 | 86889768 | 86922240 | CLCA2 | | | | | |
| 1 | 86934525 | 86965974 | CLCA1 | | | | | |
| 1 | 87012758 | 87046432 | CLCA4 | | | | | |
| 1 | 87099958 | 87121059 | CLCA3P | | | | | |
| 1 | 87170252 | 87213867 | SH3GLB1 | | | SH3GLB1 | SH3GLB1 | |
| 1 | 87328127 | 87380107 | SEP15 | | | | | |
| 1 | 87380334 | 87575681 | HS2ST1 | | | | | |
| 1 | 87595447 | 87602352 | LINC01140 | | | | | |
| 1 | 87678351 | 87717014 | LOC101927844 | | | | | |
| 1 | 87794150 | 87814607 | LMO4 | | | | | |
| 1 | 87819209 | 87837338 | LINC01364 | | | | | |
| 1 | 89003195 | 89301938 | PKN2 | | | | | |
| 1 | 89318320 | 89357301 | GTF2B | | | | | |
| 1 | 89401455 | 89458643 | CCBL2 | | | | | |
| 1 | 89445138 | 89458643 | RBMXL1 | | | | | |
| 1 | 89472359 | 89488549 | GBP3 | | | | | |

| | | | | | | | | |
|---|---|---|---|---|---|---|---|---|
| 1 | 89517986 | 89531043 | GBP1 | | | | | |
| 1 | 89571815 | 89591842 | GBP2 | | | | | |
| 1 | 89597433 | 89641723 | GBP7 | | | | | |
| 1 | 89646830 | 89664633 | GBP4 | | | | | |
| 1 | 89724633 | 89738544 | GBP5 | | | | | |
| 1 | 89754940 | 89756045 | LOC729930 | | | | | |
| 1 | 89829435 | 89853719 | GBP6 | | | | | |
| 1 | 89873237 | 89890493 | GBP1P1 | | | | | |
| 1 | 89990396 | 90063420 | LRRC8B | | | | | |
| 1 | 90090407 | 90098453 | FLJ27354 | | | | | |
| 1 | 90098643 | 90185094 | LRRC8C | | | | | |
| 1 | 90286572 | 90401989 | LRRC8D | | | | | |
| 1 | 90458823 | 90460525 | GEMIN8P4 | | | | | |
| 1 | 90460677 | 90494094 | ZNF326 | | | | | |
| 1 | 91177578 | 91182794 | BARHL2 | | | | | |
| 1 | 91248540 | 91317195 | LOC105378853 | | | | | |
| 1 | 91380856 | 91487812 | ZNF644 | | | | | |
| 1 | 91726322 | 91870426 | HFM1 | | | | | |
| 1 | 91966403 | 91991321 | CDC7 | | | | | |
| 1 | 92145899 | 92371559 | TGFBR3 | | | | | |
| 1 | 92414927 | 92479985 | BRDT | | | | | |
| 1 | 92495532 | 92529093 | EPHX4 | | | | | |
| 1 | 92539992 | 92541029 | SETSIP | | | | | |
| 1 | 92545861 | 92613401 | BTBD8 | | | | | |
| 1 | 92632608 | 92650280 | KIAA1107 | | | | | |
| 1 | 92683572 | 92711367 | C1orf146 | | | | | |
| 1 | 92711954 | 92764566 | GLMN | | | | | |
| 1 | 92764521 | 92853732 | RPAP2 | | | | | |
| 1 | 92940317 | 92952433 | GFI1 | | | | | |
| 1 | 92974252 | 93257961 | EVI5 | | | | | |
| 1 | 93297593 | 93307481 | RPL5 | | | | | |
| 1 | 93298285 | 93427079 | FAM69A | | FAM69A | | | |
| 1 | 93302845 | 93302940 | SNORD21 | | | | | |
| 1 | 93306275 | 93306408 | SNORA66 | | | | | |
| 1 | 93307716 | 93427079 | FAM69A | | FAM69A | | | |
| 1 | 93544791 | 93604638 | MTF2 | | | | | |
| 1 | 93615298 | 93646246 | TMED5 | | | | | |
| 1 | 93646272 | 93811368 | CCDC18 | | | | | |
| 1 | 93811477 | 93828148 | DR1 | | | | | |
| 1 | 93913687 | 94020218 | FNBP1L | | | | | |
| 1 | 94027297 | 94312706 | BCAR3 | | | | | |
| 1 | 94057524 | 94065587 | LOC100129046 | | | | | |
| 1 | 94312387 | 94312467 | MIR760 | | | | | |
| 1 | 94335013 | 94344762 | DNTTIP2 | | | | | |
| 1 | 94350755 | 94375154 | GCLM | | | | | |
| 1 | 94458393 | 94586705 | ABCA4 | | | | | |
| 1 | 94634462 | 94703307 | ARHGAP29 | | | | | |
| 1 | 94883932 | 94984219 | ABCD3 | | | | | |
| 1 | 94994731 | 95007413 | F3 | | | | | |
| 1 | 95123088 | 95285837 | LINC01057 | | | | | |
| 1 | 95211415 | 95211456 | MIR378G | | | | | |
| 1 | 95285897 | 95360803 | SLC44A3 | | | | | |
| 1 | 95362504 | 95392779 | CNN3 | | | | | |
| 1 | 95393583 | 95428826 | LOC729970 | | | | | |
| 1 | 95448278 | 95538507 | ALG14 | | | | | |
| 1 | 95527151 | 95533101 | LOC101928098 | | | | | |
| 1 | 95558072 | 95663161 | TMEM56 | | | | | |
| 1 | 95583478 | 95710509 | TMEM56-RWDD3 | | | | | |
| 1 | 95628774 | 95699538 | LOC101928118 | | | | | |
| 1 | 95699710 | 95712781 | RWDD3 | | | | | |
| 1 | 95940292 | 95944912 | FLJ31662 | | | | | |
| 1 | 95975671 | 95981020 | LOC100996635 | | | | | |
| 1 | 96457623 | 96488436 | LOC102723661 | | | | | |
| 1 | 96719624 | 96839681 | LOC101928241 | | | | | |
| 1 | 97187160 | 97280605 | PTBP2 | | | | | |
| 1 | 97543299 | 98263607 | DPYD | | | | | |

| | | | | | | | | | |
|---|---|---|---|---|---|---|---|---|---|
| 1 | 98453555 | 98515249 | MIR137HG | | | | | | |
| 1 | 98510798 | 98510907 | MIR2682 | | | | | | |
| 1 | 98511625 | 98511727 | MIR137 | | | | | | |
| 1 | 98676266 | 98738214 | LOC729987 | | | | | | |
| 1 | 99127235 | 99226056 | SNX7 | | | SNX7 | | | |
| 1 | 99355800 | 99470449 | PLPPR5 | | | | | | |
| 1 | 99469831 | 99614408 | LOC100129620 | | | | | | |
| 1 | 99729847 | 99775138 | PLPPR4 | | | | | | |
| 1 | 99937975 | 99953360 | LOC101928270 | | | | | | |
| 1 | 100111430 | 100160097 | PALMD | | | | | | |
| 1 | 100154610 | 100178513 | MIR548AA1 | | | | | | |
| 1 | 100154610 | 100178513 | MIR548D1 | | | | | | |
| 1 | 100174258 | 100231349 | FRRS1 | | | | | | |
| 1 | 100315639 | 100389579 | AGL | | | AGL | | | |
| 1 | 100435344 | 100492534 | SLC35A3 | | | | | | |
| 1 | 100503788 | 100548929 | MFSD14A | | | | | | |
| 1 | 100549100 | 100598651 | SASS6 | | | | | | |
| 1 | 100598705 | 100616054 | TRMT13 | | | | | | |
| 1 | 100614003 | 100643829 | LRRC39 | | | | | | |
| 1 | 100652477 | 100715409 | DBT | | | DBT | | | |
| 1 | 100730297 | 100758325 | RTCA | | | | | | |
| 1 | 100746796 | 100746864 | MIR553 | | | | | | |
| 1 | 100810570 | 100985833 | CDC14A | | | | | | |
| 1 | 101003727 | 101007583 | GPR88 | | | | | | |
| 1 | 101092605 | 101112560 | LINC01349 | | | | | | |
| 1 | 101185195 | 101204601 | VCAM1 | | | | | | |
| 1 | 101337927 | 101360735 | EXTL2 | | | | | | |
| 1 | 101361631 | 101447311 | SLC30A7 | | | | | | |
| 1 | 101455179 | 101491362 | DPH5 | | | | | | |
| 1 | 101491401 | 101552935 | LOC102606465 | | | | | | |
| 1 | 101700428 | 101702084 | LOC101928370 | | | | | | |
| 1 | 101702304 | 101707076 | S1PR1 | | | | | | |
| 1 | 101789392 | 101842869 | LINC01307 | | | | | | |
| 1 | 102268122 | 102462790 | OLFM3 | | | | | | |
| 1 | 102337567 | 102360299 | DNAJA1P5 | | | | | | |
| 1 | 103342022 | 103574052 | COL11A1 | | | COL11A1 | | | |
| 1 | 103960700 | 104068105 | LOC101928436 | | | | | | |
| 1 | 104068577 | 104097859 | RNPC3 | | | | | | |
| 1 | 104097265 | 104122156 | AMY2B | | | | | | |
| 1 | 104112025 | 104114008 | ACTG1P4 | | | | | | |
| 1 | 104159953 | 104168402 | AMY2A | | | | | | |
| 1 | 104198140 | 104207173 | AMY1A | | | | | | |
| 1 | 104198302 | 104207172 | AMY1C | | | | | | |
| 1 | 104198324 | 104207172 | AMY1B | | | | | | |
| 1 | 104230039 | 104239073 | AMY1A | | | | | | |
| 1 | 104230040 | 104238889 | AMY1B | | | | | | |
| 1 | 104230040 | 104238911 | AMY1C | | | | | | |
| 1 | 104292278 | 104301311 | AMY1A | | | | | | |
| 1 | 104292440 | 104301310 | AMY1C | | | | | | |
| 1 | 104292462 | 104301310 | AMY1B | | | | | | |
| 1 | 104615644 | 104619693 | LOC100129138 | | | | | | |
| 1 | 106132315 | 106161557 | LOC101928476 | | | | | | |
| 1 | 107599266 | 107601916 | PRMT6 | | | | | | |
| 1 | 107682539 | 108027521 | NTNG1 | | NTNG1 | | NTNG1 | | |
| 1 | 108113781 | 108507545 | VAV3 | | | | | | |
| 1 | 108439844 | 108439926 | MIR7852 | | | | | | |
| 1 | 108507064 | 108537229 | VAV3 | | | | | | |
| 1 | 108677343 | 108742980 | SLC25A24 | | | | | | |
| 1 | 108765962 | 108786703 | NBPF4 | | | | | | |
| 1 | 108992903 | 109013260 | NBPF6 | | | | | | |
| 1 | 109102970 | 109181949 | FAM102B | | | | | | |
| 1 | 109190909 | 109204148 | HENMT1 | | | | | | |
| 1 | 109234931 | 109244422 | PRPF38B | | | | | | |
| 1 | 109255555 | 109285367 | FNDC7 | | | | | | |
| 1 | 109289284 | 109352148 | STXBP3 | | | STXBP3 | | | |
| 1 | 109358519 | 109400864 | AKNAD1 | | | | | | |

| | | | | | | | | |
|---|---|---|---|---|---|---|---|---|
| 1 | 109399838 | 109401146 | SPATA42 | | | | | |
| 1 | 109419602 | 109473044 | GPSM2 | | GPSM2 | | | |
| 1 | 109472129 | 109506121 | CLCC1 | | | | | |
| 1 | 109512837 | 109584850 | WDR47 | | | | | |
| 1 | 109606997 | 109618624 | TAF13 | | | | | |
| 1 | 109633402 | 109639554 | TMEM167B | | | | | |
| 1 | 109642814 | 109643234 | SCARNA2 | | | | | |
| 1 | 109648572 | 109656479 | C1orf194 | | | | | |
| 1 | 109656584 | 109749403 | KIAA1324 | | | | | |
| 1 | 109756514 | 109780804 | SARS | | | | | |
| 1 | 109792640 | 109818378 | CELSR2 | | | | | |
| 1 | 109822175 | 109825790 | PSRC1 | | | | | |
| 1 | 109834986 | 109849663 | MYBPHL | | | | | |
| 1 | 109852187 | 109940567 | SORT1 | | SORT1 | | | |
| 1 | 109941652 | 109969108 | PSMA5 | | | | | |
| 1 | 110009099 | 110024764 | SYPL2 | | | | | |
| 1 | 110026560 | 110035420 | ATXN7L2 | | | | | |
| 1 | 110036657 | 110043063 | CYB561D1 | | | | | |
| 1 | 110049445 | 110052336 | AMIGO1 | | | | | |
| 1 | 110082493 | 110088455 | GPR61 | | | | | |
| 1 | 110091185 | 110138454 | GNAI3 | | | | | |
| 1 | 110141514 | 110141589 | MIR197 | | | | | |
| 1 | 110145888 | 110155705 | GNAT2 | | GNAT2 | | | |
| 1 | 110162434 | 110174677 | AMPD2 | | AMPD2 | | | |
| 1 | 110198697 | 110208123 | GSTM4 | | | | | |
| 1 | 110210643 | 110226619 | GSTM2 | | | | | |
| 1 | 110230417 | 110236367 | GSTM1 | | | | | |
| 1 | 110254863 | 110260890 | GSTM5 | | | | | |
| 1 | 110276553 | 110283660 | GSTM3 | | | | | |
| 1 | 110292701 | 110306644 | EPS8L3 | | | | | |
| 1 | 110453232 | 110473616 | CSF1 | | | | | |
| 1 | 110527386 | 110566364 | AHCYL1 | | AHCYL1 | | | |
| 1 | 110574198 | 110597263 | STRIP1 | | | | | |
| 1 | 110602996 | 110613322 | ALX3 | | | | | |
| 1 | 110625309 | 110652341 | LINC01397 | | | | | |
| 1 | 110655061 | 110656569 | UBL4B | | | | | |
| 1 | 110693131 | 110744823 | SLC6A17 | | | | | |
| 1 | 110751072 | 110776674 | KCNC4 | | | | | |
| 1 | 110828998 | 110881793 | LOC440600 | | | | | |
| 1 | 110881944 | 110889303 | RBM15 | | | | | |
| 1 | 110905472 | 110933704 | SLC16A4 | | | | | |
| 1 | 110943876 | 110958896 | LAMTOR5 | | | | | |
| 1 | 110993787 | 110999976 | PROK1 | | | | | |
| 1 | 111023387 | 111033891 | CYMP | | | | | |
| 1 | 111030301 | 111032880 | LOC440602 | | | | | |
| 1 | 111059838 | 111061797 | KCNA10 | | | | | |
| 1 | 111136201 | 111148975 | KCNA2 | | KCNA2 | | KCNA2 | |
| 1 | 111196185 | 111217655 | KCNA3 | | | | | |
| 1 | 111413820 | 111442558 | CD53 | | | | | |
| 1 | 111489811 | 111506566 | LRIF1 | | | | | |
| 1 | 111659953 | 111682838 | DRAM2 | | | | | |
| 1 | 111682248 | 111727724 | CEPT1 | | | | | |
| 1 | 111728590 | 111747160 | DENND2D | | | | | |
| 1 | 111770280 | 111786062 | CHI3L2 | | | | | |
| 1 | 111823145 | 111828730 | CHIAP2 | | | | | |
| 1 | 111833473 | 111863188 | CHIA | | | | | |
| 1 | 111889181 | 111895639 | PIFO | | | | | |
| 1 | 111927140 | 111932473 | PGCP1 | | | | | |
| 1 | 111956936 | 111970399 | OVGP1 | | | | | |
| 1 | 111982511 | 111991930 | WDR77 | | | | | |
| 1 | 111991742 | 112004525 | ATP5F1 | | | ATP5F1 | | |
| 1 | 112016490 | 112021134 | C1orf162 | | | | | |
| 1 | 112025969 | 112106625 | TMIGD3 | | | | | |
| 1 | 112042050 | 112046743 | ADORA3 | | ADORA3 | ADORA3 | | ADORA3 |
| 1 | 112141628 | 112150940 | LINC01160 | | | | | |
| 1 | 112162404 | 112259317 | RAP1A | | | | | |

| | | | | | | | | |
|---|---|---|---|---|---|---|---|---|
| 1 | 112264685 | 112290420 | FAM212B | | | | | |
| 1 | 112287938 | 112298131 | LOC101928718 | | | | | |
| 1 | 112298189 | 112310199 | DDX20 | | | | | |
| 1 | 112318453 | 112531777 | KCND3 | | KCND3 | KCND3 | | |
| 1 | 112396383 | 112399527 | KCND3-IT1 | | | | | |
| 1 | 112452278 | 112453552 | KCND3 | | KCND3 | KCND3 | | |
| 1 | 112533189 | 112541463 | LOC643355 | | | | | |
| 1 | 112938799 | 113003786 | CTTNBP2NL | | | | | |
| 1 | 113004391 | 113004455 | MIR4256 | | | | | |
| 1 | 113009162 | 113063910 | WNT2B | | | | | |
| 1 | 113066140 | 113162040 | ST7L | | | | | |
| 1 | 113162074 | 113214241 | CAPZA1 | | | | | |
| 1 | 113216933 | 113243368 | MOV10 | | | | | |
| 1 | 113243748 | 113250025 | RHOC | | | | | |
| 1 | 113252615 | 113257950 | PPM1J | | | | | |
| 1 | 113263188 | 113269856 | FAM19A3 | | | | | |
| 1 | 113362790 | 113393265 | LINC01356 | | | | | |
| 1 | 113454469 | 113498975 | SLC16A1 | | | | | |
| 1 | 113465971 | 113467295 | AKR7A2P1 | | | | | |
| 1 | 113499036 | 113506690 | SLC16A1 | | | | | |
| 1 | 113554308 | 113615724 | LOC100996251 | | | | | |
| 1 | 113615791 | 113667824 | LRIG2 | | | | | |
| 1 | 113739403 | 113748875 | LOC643441 | | | | | |
| 1 | 113933474 | 114228545 | MAGI3 | | | | | |
| 1 | 114239823 | 114301777 | PHTF1 | | | | | |
| 1 | 114304453 | 114355098 | RSBN1 | | | | | |
| 1 | 114355233 | 114443859 | AP4B1 | | | AP4B1 | | |
| 1 | 114356432 | 114414381 | PTPN22 | | | | | |
| 1 | 114399256 | 114443859 | AP4B1 | | | AP4B1 | | |
| 1 | 114419435 | 114430169 | BCL2L15 | | | | | |
| 1 | 114436816 | 114447746 | AP4B1 | | | AP4B1 | | |
| 1 | 114447240 | 114456708 | DCLRE1B | | | | | |
| 1 | 114466622 | 114520491 | HIPK1 | | | | | |
| 1 | 114522012 | 114524876 | OLFML3 | | | | | |
| 1 | 114631913 | 114696472 | SYT6 | | SYT6 | SYT6 | SYT6 | SYT6 |
| 1 | 114935398 | 115053781 | TRIM33 | | | | | |
| 1 | 115110180 | 115124265 | BCAS2 | | | BCAS2 | | |
| 1 | 115127195 | 115212732 | DENND2C | | | | | |
| 1 | 115215719 | 115238239 | AMPD1 | | | | | |
| 1 | 115247084 | 115259515 | NRAS | | | | | |
| 1 | 115259533 | 115300671 | CSDE1 | | | | | |
| 1 | 115312104 | 115323308 | SIKE1 | | | | | |
| 1 | 115397423 | 115537990 | SYCP1 | | | | | |
| 1 | 115572444 | 115576930 | TSHB | | | | | |
| 1 | 115590632 | 115632121 | TSPAN2 | | | | | |
| 1 | 115828536 | 115880857 | NGF | NGF | | | | |
| 1 | 116184573 | 116240845 | VANGL1 | | | | | |
| 1 | 116242625 | 116311426 | CASQ2 | | | | | |
| 1 | 116378998 | 116383747 | NHLH2 | | | | | |
| 1 | 116461996 | 116468529 | LOC101928995 | | | | | |
| 1 | 116465193 | 116519897 | LOC101928977 | | | | | |
| 1 | 116519118 | 116612675 | SLC22A15 | | | SLC22A15 | | |
| 1 | 116654375 | 116677861 | MAB21L3 | | | | | |
| 1 | 116915794 | 116961181 | ATP1A1 | | | ATP1A1 | | |
| 1 | 116966345 | 117021448 | LOC101929023 | | | | | |
| 1 | 117057155 | 117113715 | CD58 | | | | | |
| 1 | 117117019 | 117210377 | IGSF3 | | | | | |
| 1 | 117214370 | 117214449 | MIR320B1 | | | | | |
| 1 | 117236733 | 117249225 | C1orf137 | | | | | |
| 1 | 117297085 | 117311851 | CD2 | | | | | |
| 1 | 117452544 | 117532980 | PTGFRN | | | | | |
| 1 | 117544371 | 117579173 | CD101 | | | | | |
| 1 | 117568103 | 117602112 | LOC101929099 | | | | | |
| 1 | 117602948 | 117645491 | TTF2 | | | | | |
| 1 | 117637264 | 117637350 | MIR942 | | | | | |
| 1 | 117653676 | 117664411 | TRIM45 | | | TRIM45 | | |

| | | | | | | | | |
|---|---|---|---|---|---|---|---|---|
| 1 | 117686208 | 117753582 | VTCN1 | | | | | |
| 1 | 117838087 | 117863958 | LINC01525 | | | | | |
| 1 | 117910084 | 118068320 | MAN1A2 | | | | | |
| 1 | 118139453 | 118148392 | LOC100996263 | | | | | |
| 1 | 118148603 | 118171011 | FAM46C | | | | | |
| 1 | 118406106 | 118472302 | GDAP2 | | | | | |
| 1 | 118472371 | 118503049 | WDR3 | | | | | |
| 1 | 118496287 | 118727848 | SPAG17 | | | | | |
| 1 | 119425665 | 119532179 | TBX15 | | | | | |
| 1 | 119542966 | 119544028 | LOC105378933 | | | | | |
| 1 | 119573838 | 119683295 | WARS2 | | | | | |
| 1 | 119590027 | 119605910 | WARS2-IT1 | | | | | |
| 1 | 119683018 | 119818596 | LOC101929147 | | | | | |
| 1 | 119911398 | 119936753 | HAO2 | | | | | |
| 1 | 119911568 | 119912953 | HAO2-IT1 | | | | | |
| 1 | 119957553 | 119965662 | HSD3B2 | | | HSD3B2 | | |
| 1 | 120049825 | 120057681 | HSD3B1 | | | | | |
| 1 | 120106502 | 120115199 | HSD3BP4 | | | | | |
| 1 | 120140324 | 120141914 | LINC00622 | | | | | |
| 1 | 120161999 | 120190390 | ZNF697 | | | | | |
| 1 | 120254418 | 120286849 | PHGDH | | PHGDH | | | |
| 1 | 120290618 | 120311555 | HMGCS2 | | | HMGCS2 | | |
| 1 | 120336640 | 120354203 | REG4 | | | | | |
| 1 | 120377387 | 120387503 | NBPF7 | | | | | |
| 1 | 120436155 | 120439147 | ADAM30 | | | | | |
| 1 | 120454175 | 120612317 | NOTCH2 | | | NOTCH2 | | |
| 1 | 120839004 | 120855681 | FAM72B | | | | | |
| 1 | 120906033 | 120914842 | HIST2H2BA | | | | | |
| 1 | 120926127 | 120935944 | FCGR1B | | FCGR1B | | | |
| 1 | 121102016 | 121139765 | SRGAP2 | | SRGAP2 | | | |
| 1 | 121107151 | 121131061 | SRGAP2D | | | | | |
| 1 | 121107151 | 121134685 | SRGAP2B | | | | | |
| 1 | 121260909 | 121313686 | EMBP1 | | | | | |
| 1 | 142697420 | 142713605 | ANKRD20A12P | | | | | |
| 1 | 143134602 | 143202239 | LOC102723769 | | | | | |
| 1 | 143672920 | 143673002 | MIR6077 | | | | | |
| 1 | 143687126 | 143717173 | LOC100132057 | | | | | |
| 1 | 143717587 | 143744519 | LINC01138 | | | | | |
| 1 | 143767143 | 143767881 | PPIAL4G | | | | | |
| 1 | 143896451 | 143913143 | FAM72D | | | | | |
| 1 | 143896524 | 143913160 | FAM72C | | | | | |
| 1 | 144146810 | 146467744 | NBPF20 | | | | | |
| 1 | 144298163 | 144341756 | LINC00623 | | | | | |
| 1 | 144300467 | 144341077 | LINC00869 | | | | | |
| 1 | 144363461 | 144364246 | PPIAL4A | | | | | |
| 1 | 144363461 | 144364246 | PPIAL4C | | | | | |
| 1 | 144610814 | 144612719 | PFN1P2 | | | | | |
| 1 | 144614958 | 144830407 | NBPF8 | | | | | |
| 1 | 144614958 | 145370304 | NBPF9 | | | | | |
| 1 | 144851423 | 145076186 | PDE4DIP | | PDE4DIP | | | |
| 1 | 145096406 | 145116997 | SEC22B | | | | | |
| 1 | 145209112 | 145286270 | NOTCH2NL | | | | | |
| 1 | 145289769 | 145370303 | NBPF25P | | | | | |
| 1 | 145293370 | 146466121 | NBPF10 | | | | | |
| 1 | 145376205 | 145382404 | LOC101928979 | | | | | |
| 1 | 145413190 | 145417545 | HFE2 | | | | | |
| 1 | 145438437 | 145442644 | TXNIP | | | | | |
| 1 | 145456235 | 145470387 | POLR3GL | | | | | |
| 1 | 145470507 | 145475647 | ANKRD34A | | | | | |
| 1 | 145477066 | 145501669 | LIX1L | | | | | |
| 1 | 145507556 | 145513535 | RBM8A | | | | | |
| 1 | 145509751 | 145515899 | GNRHR2 | | | | | |
| 1 | 145516164 | 145523732 | PEX11B | | | | | |
| 1 | 145524890 | 145543868 | ITGA10 | | | | | |
| 1 | 145549208 | 145568526 | ANKRD35 | | | | | |
| 1 | 145575987 | 145586546 | PIAS3 | | | | | |

| | | | | | | | | | |
|---|---|---|---|---|---|---|---|---|---|
| 1 | 145584422 | 145584481 | MIR6736 | | | | | | |
| 1 | 145586492 | 145589435 | NUDT17 | | | NUDT17 | | | |
| 1 | 145590666 | 145611044 | POLR3C | | | | | | |
| 1 | 145610989 | 145689005 | RNF115 | | | | | | |
| 1 | 145695797 | 145715639 | CD160 | | | | | | |
| 1 | 145727665 | 145764206 | PDZK1 | | | PDZK1 | | | |
| 1 | 145764594 | 145827103 | GPR89A | | | | | | |
| 1 | 145827204 | 146467743 | NBPF25P | | | | | | |
| 1 | 145924387 | 145945477 | PDZK1P1 | | | | | | |
| 1 | 146032541 | 146082633 | NBPF11 | | | | | | |
| 1 | 146373856 | 146467639 | NBPF12 | | | | | | |
| 1 | 146490894 | 146514599 | LOC728989 | | | | | | |
| 1 | 146556162 | 146556303 | RNVU1-8 | | | | | | |
| 1 | 146571065 | 146585928 | NBPF13P | | | | | | |
| 1 | 146626684 | 146644168 | PRKAB2 | | | | | | |
| 1 | 146649429 | 146651528 | PDIA3P1 | | | | | | |
| 1 | 146655883 | 146697230 | FMO5 | | | | | | |
| 1 | 146714290 | 146767447 | CHD1L | | | | | | |
| 1 | 146853913 | 146989699 | LINC00624 | | | | | | |
| 1 | 147013270 | 147098020 | BCL9 | | | | | | |
| 1 | 147119167 | 147142634 | ACP6 | | | ACP6 | | | |
| 1 | 147228331 | 147245466 | GJA5 | | | | | | |
| 1 | 147374945 | 147381395 | GJA8 | | | GJA8 | | | |
| 1 | 147400505 | 147465753 | GPR89B | | | | | | |
| 1 | 147466093 | 147487188 | PDZK1P1 | | | | | | |
| 1 | 147574322 | 147624469 | NBPF11 | | | | | | |
| 1 | 147574322 | 148346929 | NBPF8 | | | | | | |
| 1 | 147680369 | 147719342 | LOC101927468 | | | | | | |
| 1 | 147806602 | 147806678 | MIR5087 | | | | | | |
| 1 | 147860433 | 147860515 | MIR6077 | | | | | | |
| 1 | 147874635 | 147904682 | LOC100132057 | | | | | | |
| 1 | 147954634 | 147955419 | PPIAL4A | | | | | | |
| 1 | 147954634 | 147955419 | PPIAL4C | | | | | | |
| 1 | 147994145 | 147994313 | RNVU1-19 | | | | | | |
| 1 | 148201751 | 148202536 | PPIAL4D | | | | | | |
| 1 | 148201751 | 148202536 | PPIAL4E | | | | | | |
| 1 | 148201751 | 148202536 | PPIAL4F | | | | | | |
| 1 | 148250248 | 148346756 | NBPF14 | | | | | | |
| 1 | 148250248 | 148346929 | NBPF9 | | | | | | |
| 1 | 148250249 | 149109725 | NBPF25P | | | | | | |
| 1 | 148558187 | 148596267 | NBPF15 | | | | | | |
| 1 | 148806014 | 148806799 | PPIAL4D | | | | | | |
| 1 | 148806014 | 148806799 | PPIAL4E | | | | | | |
| 1 | 148806014 | 148806799 | PPIAL4F | | | | | | |
| 1 | 148901844 | 148904054 | DRD5P2 | | | | | | |
| 1 | 148901844 | 148904054 | LOC101060524 | | | | | | |
| 1 | 148928312 | 148953054 | LOC645166 | | | | | | |
| 1 | 149287129 | 149291743 | LOC388692 | | | | | | |
| 1 | 149287450 | 149291743 | FAM231D | | | | | | |
| 1 | 149369293 | 149378303 | FCGR1CP | | | | | | |
| 1 | 149514089 | 149514256 | RNVU1-19 | | | | | | |
| 1 | 149553002 | 149553787 | PPIAL4A | | | | | | |
| 1 | 149553002 | 149553787 | PPIAL4C | | | | | | |
| 1 | 149575481 | 149619134 | LINC00623 | | | | | | |
| 1 | 149576160 | 149651107 | LINC00869 | | | | | | |
| 1 | 149576261 | 149672983 | LOC103091866 | | | | | | |
| 1 | 149576466 | 149619134 | LINC00623 | | | | | | |
| 1 | 149605916 | 149606079 | RNVU1-20 | | | | | | |
| 1 | 149754244 | 149783928 | HIST2H2BF | | | | | | |
| 1 | 149754249 | 149764074 | FCGR1A | | FCGR1A | | | | |
| 1 | 149783433 | 149783928 | HIST2H2BF | | | | | | |
| 1 | 149784779 | 149785236 | HIST2H3D | | | | | | |
| 1 | 149804220 | 149804616 | HIST2H4A | | | | | | |
| 1 | 149804220 | 149804616 | HIST2H4B | | | | | | |
| 1 | 149812258 | 149812765 | HIST2H3A | | | | | | |
| 1 | 149812258 | 149812765 | HIST2H3C | | | | | | |

| | | | | | | | | |
|---|---|---|---|---|---|---|---|---|
| 1 | 149813784 | 149814318 | HIST2H2AA3 | | | | | |
| 1 | 149813784 | 149814318 | HIST2H2AA4 | | | | | |
| 1 | 149821758 | 149822340 | HIST2H2BC | | | | | |
| 1 | 149822627 | 149823161 | HIST2H2AA3 | | | | | |
| 1 | 149822627 | 149823161 | HIST2H2AA4 | | | | | |
| 1 | 149824180 | 149824687 | HIST2H3A | | | | | |
| 1 | 149824180 | 149824687 | HIST2H3C | | | | | |
| 1 | 149832329 | 149832725 | HIST2H4A | | | | | |
| 1 | 149832329 | 149832725 | HIST2H4B | | | | | |
| 1 | 149856009 | 149858232 | HIST2H2BE | | | | | |
| 1 | 149858524 | 149858961 | HIST2H2AC | | | | | |
| 1 | 149859018 | 149859466 | HIST2H2AB | | | | | |
| 1 | 149871154 | 149872348 | BOLA1 | | | | | |
| 1 | 149874871 | 149889434 | SV2A | | | | | |
| 1 | 149895208 | 149900144 | SF3B4 | | | | | |
| 1 | 149900542 | 149908791 | MTMR11 | | | | | |
| 1 | 149912228 | 149982686 | OTUD7B | | | | | |
| 1 | 150039349 | 150117505 | VPS45 | | | VPS45 | | |
| 1 | 150121623 | 150132260 | PLEKHO1 | | | | | |
| 1 | 150132925 | 150153463 | LOC105371433 | | | | | |
| 1 | 150190716 | 150208504 | ANP32E | | | | | |
| 1 | 150230137 | 150237480 | CA14 | | | | | |
| 1 | 150237798 | 150241609 | APH1A | | | | | |
| 1 | 150244686 | 150253335 | C1orf54 | | | | | |
| 1 | 150254942 | 150259504 | CIART | | | | | |
| 1 | 150266261 | 150281414 | MRPS21 | | | | | |
| 1 | 150293927 | 150325704 | PRPF3 | | | | | |
| 1 | 150336586 | 150449041 | RPRD2 | | | | | |
| 1 | 150459839 | 150480085 | TARS2 | | | | | |
| 1 | 150464820 | 150464886 | MIR6878 | | | | | |
| 1 | 150480486 | 150486265 | ECM1 | | | | | |
| 1 | 150488232 | 150490508 | FALEC | | | | | |
| 1 | 150521844 | 150533412 | ADAMTSL4 | | | | | |
| 1 | 150524404 | 150524490 | MIR4257 | | | | | |
| 1 | 150533370 | 150547028 | ADAMTSL4 | | | | | |
| 1 | 150547026 | 150552214 | MCL1 | | | | | |
| 1 | 150594598 | 150602098 | ENSA | | | | | |
| 1 | 150618700 | 150669672 | GOLPH3L | | | | | |
| 1 | 150670534 | 150693364 | HORMAD1 | | | | | |
| 1 | 150702671 | 150738433 | CTSS | | | | | |
| 1 | 150768683 | 150780917 | CTSK | | | | | |
| 1 | 150782180 | 150849244 | ARNT | | | | | |
| 1 | 150898814 | 150937220 | SETDB1 | | | | | |
| 1 | 150937648 | 150947479 | CERS2 | | | | | |
| 1 | 150954498 | 150968114 | ANXA9 | | | | | |
| 1 | 150969300 | 150980854 | FAM63A | | | | | |
| 1 | 150980866 | 151008189 | PRUNE | | | | | |
| 1 | 151009028 | 151020076 | BNIPL | | | | | |
| 1 | 151020258 | 151023871 | C1orf56 | | | | | |
| 1 | 151023446 | 151032125 | CDC42SE1 | | | | | |
| 1 | 151032150 | 151040973 | MLLT11 | | | | | |
| 1 | 151043079 | 151091007 | GABPB2 | | | | | |
| 1 | 151104162 | 151119140 | SEMA6C | | | SEMA6C | | |
| 1 | 151129104 | 151132225 | TNFAIP8L2 | | | | | |
| 1 | 151129104 | 151142773 | TNFAIP8L2-SCNM1 | | | | | |
| 1 | 151132223 | 151138424 | LYSMD1 | | | | | |
| 1 | 151138497 | 151142773 | SCNM1 | | | | | |
| 1 | 151142462 | 151148547 | TMOD4 | | | | | |
| 1 | 151148775 | 151162689 | VPS72 | | | | | |
| 1 | 151171020 | 151222007 | PIP5K1A | | | | | |
| 1 | 151227196 | 151239954 | PSMD4 | | | PSMD4 | | |
| 1 | 151254030 | 151264381 | ZNF687 | | | | | |
| 1 | 151264272 | 151300191 | PI4KB | | | | | |
| 1 | 151313115 | 151319769 | RFX5 | | | | | |
| 1 | 151319485 | 151320367 | GBAT2 | | | | | |
| 1 | 151336777 | 151345210 | SELENBP1 | | | | | |

| | | | | | | | | |
|---|---|---|---|---|---|---|---|---|
| 1 | 151372040 | 151374412 | PSMB4 | | | | | |
| 1 | 151375199 | 151431941 | POGZ | | | | | |
| 1 | 151483861 | 151511167 | CGN | | | | | |
| 1 | 151512780 | 151556053 | TUFT1 | | | | | |
| 1 | 151518271 | 151518367 | MIR554 | | | | | |
| 1 | 151584661 | 151671559 | SNX27 | | | | | |
| 1 | 151672533 | 151689296 | CELF3 | | | | | |
| 1 | 151694012 | 151702082 | RIIAD1 | | | | | |
| 1 | 151732118 | 151736392 | MRPL9 | | | | | |
| 1 | 151735444 | 151743806 | OAZ3 | | | | | |
| 1 | 151744040 | 151763010 | TDRKH | | | TDRKH | | |
| 1 | 151772739 | 151777918 | LINGO4 | | | | | |
| 1 | 151778546 | 151804348 | RORC | | | | | |
| 1 | 151810338 | 151813033 | C2CD4D | | | | | |
| 1 | 151810944 | 151816641 | LOC100132111 | | | | | |
| 1 | 151819576 | 151826173 | THEM5 | | | | | |
| 1 | 151843342 | 151882361 | THEM4 | | | | | |
| 1 | 151955385 | 151966714 | S100A10 | | | | | |
| 1 | 151990753 | 151994985 | NBPF18P | | | | | |
| 1 | 152004981 | 152009511 | S100A11 | | | | | |
| 1 | 152051888 | 152053400 | LOC100131107 | | | | | |
| 1 | 152056619 | 152061540 | TCHHL1 | | | | | |
| 1 | 152078792 | 152087930 | TCHH | | | | | |
| 1 | 152126070 | 152131704 | RPTN | | | | | |
| 1 | 152184551 | 152196672 | HRNR | | | | | |
| 1 | 152274650 | 152339168 | FLG | | | | | |
| 1 | 152321212 | 152332482 | FLG2 | | | | | |
| 1 | 152381718 | 152386750 | CRNN | | | | | |
| 1 | 152483319 | 152484653 | LCE5A | | | | | |
| 1 | 152486977 | 152488481 | CRCT1 | | | | | |
| 1 | 152538174 | 152539229 | LCE3E | | | | | |
| 1 | 152551859 | 152552980 | LCE3D | | | | | |
| 1 | 152573137 | 152573562 | LCE3C | | | | | |
| 1 | 152586286 | 152586574 | LCE3B | | | | | |
| 1 | 152595309 | 152595579 | LCE3A | | | | | |
| 1 | 152635886 | 152637135 | LCE2D | | | | | |
| 1 | 152647790 | 152649049 | LCE2C | | | | | |
| 1 | 152658598 | 152659876 | LCE2B | | | | | |
| 1 | 152670839 | 152671918 | LCE2A | | | | | |
| 1 | 152681522 | 152681910 | LCE4A | | | | | |
| 1 | 152691997 | 152692905 | C1orf68 | | | | | |
| 1 | 152730505 | 152734529 | KPRP | | | | | |
| 1 | 152748847 | 152749445 | LCE1F | | | | | |
| 1 | 152758752 | 152760901 | LCE1E | | | | | |
| 1 | 152769226 | 152770657 | LCE1D | | | | | |
| 1 | 152777310 | 152779107 | LCE1C | | | | | |
| 1 | 152784446 | 152785585 | LCE1B | | | | | |
| 1 | 152799948 | 152800573 | LCE1A | | | | | |
| 1 | 152815329 | 152816459 | LCE6A | | | | | |
| 1 | 152850797 | 152857523 | SMCP | | | | | |
| 1 | 152881038 | 152884362 | IVL | | | | | |
| 1 | 152943128 | 152945049 | SPRR4 | | | | | |
| 1 | 152956563 | 152958290 | SPRR1A | | | | | |
| 1 | 152974222 | 152976332 | SPRR3 | | | | | |
| 1 | 153003678 | 153005376 | SPRR1B | | | | | |
| 1 | 153012200 | 153013594 | SPRR2D | | | | | |
| 1 | 153028595 | 153029988 | SPRR2A | | | | | |
| 1 | 153042703 | 153044084 | SPRR2B | | | | | |
| 1 | 153065610 | 153067004 | SPRR2E | | | | | |
| 1 | 153084599 | 153085991 | SPRR2F | | | | | |
| 1 | 153112593 | 153113969 | SPRR2C | | | | | |
| 1 | 153122057 | 153123427 | SPRR2G | | | | | |
| 1 | 153146993 | 153164152 | LOC101928009 | | | | | |
| 1 | 153175905 | 153177601 | LELP1 | | | | | |
| 1 | 153190059 | 153191793 | PRR9 | | | | | |
| 1 | 153232178 | 153234600 | LOR | | | | | |

| | | | | | | | | |
|---|---|---|---|---|---|---|---|---|
| 1 | 153270337 | 153283194 | PGLYRP3 | | | | | |
| 1 | 153302596 | 153321317 | PGLYRP4 | | | | | |
| 1 | 153330329 | 153333503 | S100A9 | | | | | |
| 1 | 153346183 | 153348075 | S100A12 | | | | | |
| 1 | 153362507 | 153395059 | S100A8 | | | | | |
| 1 | 153388999 | 153395701 | S100A7A | | | | | |
| 1 | 153409470 | 153412503 | S100A7L2 | | | | | |
| 1 | 153430219 | 153433137 | S100A7 | | | | | |
| 1 | 153507075 | 153508717 | S100A6 | | | | | |
| 1 | 153509622 | 153514241 | S100A5 | | | | | |
| 1 | 153516094 | 153518282 | S100A4 | | | | | |
| 1 | 153518217 | 153524245 | LOC101928034 | | | | | |
| 1 | 153519808 | 153521734 | S100A3 | | | | | |
| 1 | 153533584 | 153538306 | S100A2 | | | | | |
| 1 | 153579358 | 153585644 | S100A16 | | | | | |
| 1 | 153586731 | 153588808 | S100A14 | | | | | |
| 1 | 153591275 | 153606568 | S100A13 | | | | | |
| 1 | 153600872 | 153604513 | S100A1 | | | | | |
| 1 | 153606457 | 153618782 | CHTOP | | | | | |
| 1 | 153631129 | 153634328 | SNAPIN | | | SNAPIN | | |
| 1 | 153634263 | 153643504 | ILF2 | | | | | |
| 1 | 153651163 | 153666468 | NPR1 | | | | | |
| 1 | 153662180 | 153662269 | MIR8083 | | | | | |
| 1 | 153700566 | 153746555 | INTS3 | | | | | |
| 1 | 153747767 | 153752633 | SLC27A3 | | | | | |
| 1 | 153766422 | 153768778 | LOC343052 | | | | | |
| 1 | 153777202 | 153895451 | GATAD2B | | | | | |
| 1 | 153901976 | 153919154 | DENND4B | | | | | |
| 1 | 153920147 | 153931132 | CRTC2 | | | | | |
| 1 | 153931574 | 153940660 | SLC39A1 | | | | | |
| 1 | 153934826 | 153934896 | MIR6737 | | | | | |
| 1 | 153940314 | 153946840 | CREB3L4 | | | | | |
| 1 | 153946744 | 153950451 | JTB | | | | | |
| 1 | 153954092 | 153958853 | RAB13 | | | RAB13 | | |
| 1 | 153963238 | 153964631 | RPS27 | | | | | |
| 1 | 153965167 | 154127592 | NUP210L | | | | | |
| 1 | 154127779 | 154164543 | TPM3 | | TPM3 | | | |
| 1 | 154166140 | 154166219 | MIR190B | | | | | |
| 1 | 154171561 | 154178841 | C1orf189 | | | | | |
| 1 | 154179176 | 154193273 | C1orf43 | | | | | |
| 1 | 154192647 | 154243986 | UBAP2L | | | | | |
| 1 | 154245038 | 154248355 | HAX1 | | | HAX1 | | |
| 1 | 154293591 | 154297801 | AQP10 | | | | | |
| 1 | 154298035 | 154323780 | ATP8B2 | | | | | |
| 1 | 154377668 | 154441926 | IL6R | | | | | |
| 1 | 154451953 | 154474526 | SHE | | | | | |
| 1 | 154474694 | 154520623 | TDRD10 | | | | | |
| 1 | 154521050 | 154527504 | UBE2Q1 | | | | | |
| 1 | 154540256 | 154552353 | CHRNB2 | CHRNB2 | | | CHRNB2 | CHRNB2 |
| 1 | 154554533 | 154600456 | ADAR | | | ADAR | ADAR | |
| 1 | 154669941 | 154842754 | KCNN3 | | KCNN3 | | | |
| 1 | 154897207 | 154909484 | PMVK | | | | | |
| 1 | 154916558 | 154928639 | PBXIP1 | | | | | |
| 1 | 154929501 | 154934258 | PYGO2 | | | | | |
| 1 | 154934773 | 154946959 | SHC1 | | | SHC1 | | |
| 1 | 154947117 | 154951725 | CKS1B | | | | | |
| 1 | 154948168 | 154948259 | MIR4258 | | | | | |
| 1 | 154955769 | 154965587 | FLAD1 | | | | | |
| 1 | 154966061 | 154966791 | LENEP | | | | | |
| 1 | 154975105 | 154991001 | ZBTB7B | | | | | |
| 1 | 154991002 | 155006257 | DCST2 | | | | | |
| 1 | 155006281 | 155023406 | DCST1 | | | | | |
| 1 | 155017667 | 155036467 | LOC100505666 | | | | | |
| 1 | 155023747 | 155035252 | ADAM15 | | | | | |
| 1 | 155036212 | 155042029 | EFNA4 | | | | | |
| 1 | 155051347 | 155060014 | EFNA3 | | | | | |

| | | | | | | | | | |
|---|---|---|---|---|---|---|---|---|---|
| 1 | 155100348 | 155107386 | EFNA1 | | | | | | |
| 1 | 155107819 | 155111334 | SLC50A1 | | | | | | |
| 1 | 155112366 | 155112996 | DPM3 | | | | | | |
| 1 | 155141883 | 155145804 | KRTCAP2 | | | | | | |
| 1 | 155146262 | 155157447 | TRIM46 | | | | | | |
| 1 | 155158299 | 155162706 | MUC1 | | | | | | |
| 1 | 155164967 | 155165063 | MIR92B | | | | | | |
| 1 | 155165378 | 155177772 | THBS3 | | | | | | |
| 1 | 155178489 | 155183630 | MTX1 | | | | | | |
| 1 | 155183615 | 155197325 | GBAP1 | | | | | | |
| 1 | 155204238 | 155214653 | GBA | | | | | | |
| 1 | 155216995 | 155225274 | FAM189B | | | | | | |
| 1 | 155225769 | 155232176 | SCAMP3 | | | | | | |
| 1 | 155232658 | 155243320 | CLK2 | | | | | | |
| 1 | 155247217 | 155259639 | HCN3 | | | | | | |
| 1 | 155259083 | 155271225 | PKLR | | | PKLR | | | |
| 1 | 155278538 | 155290457 | FDPS | | | | | | |
| 1 | 155290250 | 155300909 | RUSC1 | | | | | | |
| 1 | 155305051 | 155532324 | ASH1L | | | | | | |
| 1 | 155316140 | 155316236 | MIR555 | | | | | | |
| 1 | 155402970 | 155404053 | POU5F1P4 | | | | | | |
| 1 | 155531771 | 155533735 | ASH1L | | | | | | |
| 1 | 155579960 | 155584758 | MSTO1 | | | | | | |
| 1 | 155579978 | 155584616 | MSTO2P | | | | | | |
| 1 | 155629232 | 155658823 | YY1AP1 | | | | | | |
| 1 | 155648898 | 155649046 | SCARNA26A | | | | | | |
| 1 | 155658881 | 155708800 | DAP3 | | | | | | |
| 1 | 155715558 | 155720479 | MSTO2P | | | | | | |
| 1 | 155719448 | 155827086 | GON4L | | | | | | |
| 1 | 155753474 | 155753623 | SCARNA26B | | | | | | |
| 1 | 155829259 | 155854990 | SYT11 | | | | | | |
| 1 | 155867598 | 155881193 | RIT1 | | | | RIT1 | | |
| 1 | 155882833 | 155904233 | KIAA0907 | | | | | | |
| 1 | 155889699 | 155889833 | SNORA80E | | | | | | |
| 1 | 155895748 | 155895877 | SCARNA4 | | | | | | |
| 1 | 155911479 | 155912625 | RXFP4 | | | | | | |
| 1 | 155916629 | 155948336 | ARHGEF2 | | | ARHGEF2 | | | |
| 1 | 155921063 | 155921127 | MIR6738 | | | | | | |
| 1 | 155978838 | 155990758 | SSR2 | | | | | | |
| 1 | 156005084 | 156023616 | UBQLN4 | | | | | | |
| 1 | 156024516 | 156028301 | LAMTOR2 | | | | | | |
| 1 | 156030939 | 156040305 | RAB25 | | | | | | |
| 1 | 156041803 | 156051789 | MEX3A | | | | | | |
| 1 | 156052336 | 156109880 | LMNA | | | LMNA | | | |
| 1 | 156119734 | 156147542 | SEMA4A | | | | | | |
| 1 | 156163722 | 156182587 | SLC25A44 | | | | | | |
| 1 | 156182778 | 156209868 | PMF1 | | | | | | |
| 1 | 156182778 | 156213123 | PMF1-BGLAP | | | | | | |
| 1 | 156211752 | 156213123 | BGLAP | | | | | | |
| 1 | 156213111 | 156217908 | PAQR6 | | | | | | |
| 1 | 156219014 | 156252620 | SMG5 | | | | | | |
| 1 | 156252703 | 156262234 | TMEM79 | | | | | | |
| 1 | 156262477 | 156265480 | GLMP | | | | | | |
| 1 | 156268414 | 156269428 | VHLL | | | | | | |
| 1 | 156278751 | 156308206 | CCT3 | | | | | | |
| 1 | 156307104 | 156316785 | TSACC | | | | | | |
| 1 | 156338979 | 156355013 | RHBG | | | | | | |
| 1 | 156374054 | 156399184 | C1orf61 | | | | | | |
| 1 | 156390132 | 156390221 | MIR9 | | | | | | |
| 1 | 156433512 | 156470634 | MEF2D | | | | | | |
| 1 | 156495196 | 156542396 | IQGAP3 | | | | | | |
| 1 | 156549518 | 156556562 | TTC24 | | | | | | |
| 1 | 156561557 | 156564091 | APOA1BP | | | | | | |
| 1 | 156564099 | 156571279 | GPATCH4 | | | | | | |
| 1 | 156584533 | 156591720 | LOC101928177 | | | | | | |
| 1 | 156589085 | 156595517 | HAPLN2 | | | | | | |

| | | | | | | | | |
|---|---|---|---|---|---|---|---|---|
| 1 | 156611739 | 156629324 | BCAN | | BCAN | | | BCAN |
| 1 | 156638555 | 156647189 | NES | | | NES | | |
| 1 | 156669399 | 156675608 | CRABP2 | | | | | |
| 1 | 156691682 | 156698231 | ISG20L2 | | | | | |
| 1 | 156698262 | 156706752 | RRNAD1 | | | | | |
| 1 | 156707093 | 156710923 | MRPL24 | | | | | |
| 1 | 156711898 | 156722240 | HDGF | | | | | |
| 1 | 156737273 | 156770609 | PRCC | | | | | |
| 1 | 156776034 | 156786640 | SH2D2A | | | | | |
| 1 | 156785541 | 156851642 | NTRK1 | | | NTRK1 | | |
| 1 | 156810664 | 156828712 | INSRR | | | | | |
| 1 | 156830670 | 156851642 | NTRK1 | | | NTRK1 | | |
| 1 | 156863522 | 156886226 | PEAR1 | | | | | |
| 1 | 156890423 | 156902880 | LRRC71 | | | | | |
| 1 | 156904631 | 157015162 | ARHGEF11 | | | | | |
| 1 | 156905922 | 156906036 | MIR765 | | | | | |
| 1 | 157061834 | 157069600 | ETV3L | | | | | |
| 1 | 157094458 | 157108383 | ETV3 | | | | | |
| 1 | 157098153 | 157098463 | CYCSP52 | | | | | |
| 1 | 157102975 | 157108177 | ETV3 | | | | | |
| 1 | 157483166 | 157522310 | FCRL5 | | | | | |
| 1 | 157543538 | 157567870 | FCRL4 | | | | | |
| 1 | 157647977 | 157670647 | FCRL3 | | | | | |
| 1 | 157715522 | 157746922 | FCRL2 | | | | | |
| 1 | 157764193 | 157789940 | FCRL1 | | | | | |
| 1 | 157800703 | 157811634 | CD5L | | | | | |
| 1 | 157963062 | 158070052 | KIRREL | | | | | |
| 1 | 158101833 | 158110430 | LOC646268 | | | | | |
| 1 | 158149736 | 158156216 | CD1D | | | | | |
| 1 | 158223926 | 158228058 | CD1A | | | | | |
| 1 | 158259562 | 158264564 | CD1C | | | | | |
| 1 | 158297739 | 158301321 | CD1B | | | | | |
| 1 | 158323485 | 158327343 | CD1E | | | | | |
| 1 | 158368311 | 158369256 | OR10T2 | | | | | |
| 1 | 158389717 | 158390656 | OR10K2 | | | | | |
| 1 | 158435351 | 158436293 | OR10K1 | | | | | |
| 1 | 158449667 | 158450675 | OR10R2 | | | | | |
| 1 | 158516917 | 158517895 | OR6Y1 | | | | | |
| 1 | 158532440 | 158533394 | OR6P1 | | | | | |
| 1 | 158548708 | 158549689 | OR10X1 | | | | | |
| 1 | 158576228 | 158577170 | OR10Z1 | | | | | |
| 1 | 158580495 | 158656506 | SPTA1 | | | SPTA1 | | |
| 1 | 158669467 | 158670442 | OR6K2 | | | | | |
| 1 | 158686957 | 158687905 | OR6K3 | | | | | |
| 1 | 158724605 | 158725637 | OR6K6 | | | | | |
| 1 | 158735533 | 158736472 | OR6N1 | | | | | |
| 1 | 158746471 | 158747425 | OR6N2 | | | | | |
| 1 | 158801167 | 158819270 | MNDA | | | | | |
| 1 | 158901336 | 158946849 | PYHIN1 | | | | | |
| 1 | 158969760 | 158970102 | POP3 | | | | | |
| 1 | 158979681 | 159024945 | IFI16 | | | | | |
| 1 | 159032274 | 159046647 | AIM2 | | | | | |
| 1 | 159141376 | 159172212 | CADM3 | | | | | |
| 1 | 159173802 | 159176290 | ACKR1 | | | | | |
| 1 | 159259503 | 159278014 | FCER1A | | | | | |
| 1 | 159283459 | 159284449 | OR10J3 | | | | | |
| 1 | 159409511 | 159410600 | OR10J1 | | | | | |
| 1 | 159504867 | 159505797 | OR10J5 | | | | | |
| 1 | 159557615 | 159558661 | APCS | | | | | |
| 1 | 159682078 | 159684379 | CRP | | | | | |
| 1 | 159750735 | 159752336 | DUSP23 | | | | | |
| 1 | 159770300 | 159786047 | FCRL6 | | | | | |
| 1 | 159796478 | 159807282 | SLAMF8 | | | | | |
| 1 | 159804263 | 159825137 | C1orf204 | | | | | |
| 1 | 159824105 | 159832447 | VSIG8 | | | | | |
| 1 | 159842153 | 159869906 | CFAP45 | | | | | |

| | | | | | | | | |
|---|---|---|---|---|---|---|---|---|
| 1 | 159869768 | 159869869 | MIR4259 | | | | | |
| 1 | 159887896 | 159895332 | TAGLN2 | | | | | |
| 1 | 159896828 | 159915386 | IGSF9 | | | | | |
| 1 | 159921281 | 159924044 | SLAMF9 | | | | | |
| 1 | 159931013 | 159948876 | LINC01133 | | | | | |
| 1 | 159997461 | 160001783 | PIGM | | | | | |
| 1 | 160007256 | 160040051 | KCNJ10 | | KCNJ10 | | | |
| 1 | 160051359 | 160059212 | KCNJ9 | | | KCNJ9 | | |
| 1 | 160061128 | 160068618 | IGSF8 | | IGSF8 | | | |
| 1 | 160085519 | 160113374 | ATP1A2 | ATP1A2 | ATP1A2 | | | ATP1A2 |
| 1 | 160121351 | 160156767 | ATP1A4 | | | ATP1A4 | | |
| 1 | 160160284 | 160171676 | CASQ1 | | | | | |
| 1 | 160171988 | 160178659 | LOC729867 | | | | | |
| 1 | 160175108 | 160185166 | PEA15 | | | | | |
| 1 | 160185504 | 160232318 | DCAF8 | | | | | |
| 1 | 160246598 | 160254941 | PEX19 | | | | | |
| 1 | 160258376 | 160313354 | COPA | | | | | |
| 1 | 160287054 | 160288260 | SUMO1P3 | | | | | |
| 1 | 160313062 | 160328742 | NCSTN | | | | | |
| 1 | 160336860 | 160342638 | NHLH1 | | | | | |
| 1 | 160370363 | 160398468 | VANGL2 | | | | | |
| 1 | 160454819 | 160493052 | SLAMF6 | | | SLAMF6 | | |
| 1 | 160510883 | 160549306 | CD84 | | | | | |
| 1 | 160579608 | 160617101 | SLAMF1 | | | | | |
| 1 | 160648535 | 160681641 | CD48 | | | | | |
| 1 | 160708846 | 160724608 | SLAMF7 | | | | | |
| 1 | 160765863 | 160798045 | LY9 | | | | | |
| 1 | 160799949 | 160832692 | CD244 | | | | | |
| 1 | 160846329 | 160854960 | ITLN1 | | | | | |
| 1 | 160902254 | 160919712 | LOC101928372 | | | | | |
| 1 | 160914815 | 160924589 | ITLN2 | | | | | |
| 1 | 160965000 | 160991133 | F11R | | | | | |
| 1 | 161007421 | 161008774 | TSTD1 | | | | | |
| 1 | 161009040 | 161015769 | USF1 | | | | | |
| 1 | 161016731 | 161039760 | ARHGAP30 | | | | | |
| 1 | 161040780 | 161059385 | PVRL4 | | | | | |
| 1 | 161068150 | 161070138 | KLHDC9 | | | | | |
| 1 | 161070345 | 161087866 | PFDN2 | | | | | |
| 1 | 161087861 | 161090984 | NIT1 | | | NIT1 | | |
| 1 | 161090768 | 161102478 | DEDD | | | | | |
| 1 | 161123533 | 161128646 | UFC1 | | | | | |
| 1 | 161129253 | 161135516 | USP21 | | | | | |
| 1 | 161136180 | 161141010 | PPOX | | | | | |
| 1 | 161141099 | 161147758 | B4GALT3 | | | | | |
| 1 | 161159537 | 161168845 | ADAMTS4 | | | | | |
| 1 | 161169104 | 161184184 | NDUFS2 | | | | | |
| 1 | 161185086 | 161189038 | FCER1G | | | | | |
| 1 | 161192082 | 161193418 | APOA2 | | | | | |
| 1 | 161195728 | 161200536 | TOMM40L | | | | | |
| 1 | 161196975 | 161197051 | MIR5187 | | | | | |
| 1 | 161199455 | 161208000 | NR1I3 | | | | | |
| 1 | 161228516 | 161255240 | PCP4L1 | | | | | |
| 1 | 161274524 | 161279762 | MPZ | | | | | |
| 1 | 161284165 | 161334535 | SDHC | | | | | |
| 1 | 161334520 | 161337673 | CFAP126 | | | | | |
| 1 | 161475204 | 161489360 | FCGR2A | | | | | |
| 1 | 161494329 | 161496687 | HSPA6 | | | | | |
| 1 | 161511550 | 161520413 | FCGR3A | | | | | |
| 1 | 161551128 | 161571010 | FCGR2C | | | | | |
| 1 | 161575848 | 161578341 | HSPA7 | | | | | |
| 1 | 161592987 | 161601753 | FCGR3B | | | | | |
| 1 | 161632904 | 161648444 | FCGR2B | | | | | |
| 1 | 161653494 | 161655042 | RPL31P11 | | | | | |
| 1 | 161676761 | 161684142 | FCRLA | | | | | |
| 1 | 161691333 | 161697933 | FCRLB | | | | | |
| 1 | 161719557 | 161726954 | DUSP12 | | | | | |

| | | | | | | | | |
|---|---|---|---|---|---|---|---|---|
| 1 | 161736033 | 161933860 | ATF6 | | | | | |
| 1 | 161952981 | 161994255 | OLFML2B | | | | | |
| 1 | 162039580 | 162339813 | NOS1AP | | NOS1AP | | | |
| 1 | 162126896 | 162126972 | MIR4654 | | | | | |
| 1 | 162312335 | 162312430 | MIR556 | | | | | |
| 1 | 162332772 | 162339813 | NOS1AP | | NOS1AP | | | |
| 1 | 162343514 | 162346644 | C1orf111 | | | | | |
| 1 | 162348695 | 162356608 | C1orf226 | | | | | |
| 1 | 162365055 | 162381928 | SH2D1B | | | | | |
| 1 | 162466963 | 162499419 | UHMK1 | | | | | |
| 1 | 162531295 | 162569633 | UAP1 | | | | | |
| 1 | 162602227 | 162750247 | DDR2 | | | | | |
| 1 | 162760491 | 162782608 | HSD17B7 | | | | | |
| 1 | 162824086 | 162838605 | CCDC190 | | | | | |
| 1 | 163038395 | 163046592 | RGS4 | | RGS4 | | | |
| 1 | 163112088 | 163291581 | RGS5 | | | | | |
| 1 | 163131464 | 163182813 | LOC101928404 | | | | | |
| 1 | 163273995 | 163291581 | RGS5 | | | | | |
| 1 | 163291722 | 163325553 | NUF2 | | | | | |
| 1 | 163390859 | 163392981 | LOC100422212 | | | | | |
| 1 | 164528596 | 164821060 | PBX1 | | | PBX1 | | |
| 1 | 164738352 | 164743878 | LOC100505795 | | | | | |
| 1 | 165171103 | 165325952 | LMX1A | | | | | |
| 1 | 165370158 | 165414592 | RXRG | | | | | |
| 1 | 165446078 | 165551392 | LOC400794 | | | | | |
| 1 | 165513477 | 165533185 | LRRC52 | | | | | |
| 1 | 165600109 | 165625372 | MGST3 | | | | | |
| 1 | 165631448 | 165667900 | ALDH9A1 | | | | | |
| 1 | 165667986 | 165679199 | LOC440700 | | | | | |
| 1 | 165693527 | 165738159 | TMCO1 | | | | | |
| 1 | 165738165 | 165744685 | LOC100147773 | | | | | |
| 1 | 165796731 | 165880855 | UCK2 | | | | | |
| 1 | 165877157 | 165877213 | MIR3658 | | | | | |
| 1 | 166039255 | 166135958 | FAM78B | | | | | |
| 1 | 166123979 | 166124035 | MIR921 | | | | | |
| 1 | 166573152 | 166594473 | FMO9P | | | | | |
| 1 | 166808640 | 166825581 | POGK | | | | | |
| 1 | 166825748 | 166845654 | TADA1 | | | | | |
| 1 | 166882440 | 166944561 | ILDR2 | | | | | |
| 1 | 166944818 | 166991449 | MAEL | | | | | |
| 1 | 167022072 | 167059868 | GPA33 | | | | | |
| 1 | 167064086 | 167098402 | DUSP27 | | | | | |
| 1 | 167144598 | 167165042 | LINC01363 | | | | | |
| 1 | 167190065 | 167396582 | POU2F1 | | | POU2F1 | | |
| 1 | 167399876 | 167487847 | CD247 | | | | | |
| 1 | 167510250 | 167523056 | CREG1 | | | | | |
| 1 | 167599473 | 167675486 | RCSD1 | | | | | |
| 1 | 167691186 | 167761156 | MPZL1 | | | | | |
| 1 | 167778624 | 167883464 | ADCY10 | | | | | |
| 1 | 167885912 | 167906307 | MPC2 | | | | | |
| 1 | 167905796 | 168045083 | DCAF6 | | | | | |
| 1 | 167967897 | 167967964 | MIR1255B2 | | | | | |
| 1 | 168048779 | 168106905 | GPR161 | | | | | |
| 1 | 168148082 | 168171351 | TIPRL | | | | | |
| 1 | 168195254 | 168212088 | SFT2D2 | | | | | |
| 1 | 168214818 | 168216668 | ANKRD36BP1 | | | | | |
| 1 | 168250277 | 168283664 | TBX19 | | | | | |
| 1 | 168344761 | 168344859 | MIR557 | | | | | |
| 1 | 168369426 | 168391894 | LOC100505918 | | | | | |
| 1 | 168433351 | 168464882 | LOC101928565 | | | | | |
| 1 | 168510002 | 168513235 | XCL2 | | | | | |
| 1 | 168545710 | 168551315 | XCL1 | | | | | |
| 1 | 168664694 | 168698442 | DPT | | | | | |
| 1 | 168756178 | 168762126 | LINC00626 | | | | | |
| 1 | 168873142 | 169056243 | LINC00970 | | | | | |
| 1 | 169075946 | 169101960 | ATP1B1 | | ATP1B1 | ATP1B1 | | |

| | | | | | | | | |
|---|---|---|---|---|---|---|---|---|
| 1 | 169101767 | 169337201 | NME7 | | | | | |
| 1 | 169337193 | 169365780 | BLZF1 | | | | | |
| 1 | 169364107 | 169429907 | CCDC181 | | | | | |
| 1 | 169433148 | 169455208 | SLC19A2 | | | | | |
| 1 | 169481191 | 169555769 | F5 | | | F5 | | |
| 1 | 169558087 | 169599377 | SELP | | | | | |
| 1 | 169659805 | 169680843 | SELL | | | | | |
| 1 | 169691780 | 169703220 | SELE | | | | | |
| 1 | 169761669 | 169764061 | METTL18 | | | | | |
| 1 | 169763870 | 169822230 | C1orf112 | | | | | |
| 1 | 169822214 | 169863100 | SCYL3 | | | | | |
| 1 | 169890469 | 170043879 | KIFAP3 | | KIFAP3 | | | |
| 1 | 170115187 | 170136923 | METTL11B | | | | | |
| 1 | 170120518 | 170120603 | MIR3119 | | | | | |
| 1 | 170240545 | 170253349 | LINC01142 | | | | | |
| 1 | 170430457 | 170501750 | LOC101928650 | | | | | |
| 1 | 170501262 | 170522974 | GORAB | | | | | |
| 1 | 170633312 | 170708541 | PRRX1 | | | | | |
| 1 | 170904611 | 170967946 | MROH9 | | | | | |
| 1 | 171060017 | 171086959 | FMO3 | | | FMO3 | | |
| 1 | 171070868 | 171070947 | MIR1295A | | | | | |
| 1 | 171070879 | 171070939 | MIR1295B | | | | | |
| 1 | 171106878 | 171130702 | FMO6P | | | | | |
| 1 | 171154346 | 171181822 | FMO2 | | | | | |
| 1 | 171217609 | 171255117 | FMO1 | | | | | |
| 1 | 171283321 | 171311223 | FMO4 | | | | | |
| 1 | 171308034 | 171310463 | TOP1P1 | | | | | |
| 1 | 171454665 | 171562650 | PRRC2C | | | | | |
| 1 | 171604556 | 171621773 | MYOC | | | | | |
| 1 | 171669295 | 171711379 | VAMP4 | | | | | |
| 1 | 171750760 | 171766856 | METTL13 | | | | | |
| 1 | 171810617 | 172381857 | DNM3 | | | DNM3 | | |
| 1 | 171833331 | 171833827 | DNM3-IT1 | | | | | |
| 1 | 172106018 | 172113975 | DNM3OS | | | | | |
| 1 | 172107937 | 172108047 | MIR214 | | | | | |
| 1 | 172107947 | 172108028 | MIR3120 | | | | | |
| 1 | 172113674 | 172113784 | MIR199A2 | | | | | |
| 1 | 172389827 | 172437969 | C1orf105 | | | | | |
| 1 | 172410596 | 172413230 | PIGC | | | | | |
| 1 | 172422032 | 172437969 | C1orf105 | | | | | |
| 1 | 172501488 | 172580975 | SUCO | | | | | |
| 1 | 172628147 | 172636012 | FASLG | | | | | |
| 1 | 173010359 | 173020103 | TNFSF18 | | | | | |
| 1 | 173152869 | 173176452 | TNFSF4 | | | | | |
| 1 | 173204198 | 173446294 | LOC100506023 | | | | | |
| 1 | 173386927 | 173430501 | LOC101928673 | | | | | |
| 1 | 173446485 | 173457946 | PRDX6 | | | | | |
| 1 | 173469603 | 173572233 | SLC9C2 | | | | | |
| 1 | 173577474 | 173639001 | ANKRD45 | | | | | |
| 1 | 173604660 | 173606272 | LOC730159 | | | | | |
| 1 | 173684079 | 173755840 | KLHL20 | | | | KLHL20 | |
| 1 | 173768687 | 173793777 | CENPL | | | | | |
| 1 | 173793796 | 173827682 | DARS2 | | | | | |
| 1 | 173832385 | 173837125 | GAS5 | | | | | |
| 1 | 173833312 | 173833355 | SNORD81 | | | | | |
| 1 | 173833506 | 173833583 | SNORD47 | | | | | |
| 1 | 173833970 | 173834041 | SNORD80 | | | | | |
| 1 | 173834487 | 173834568 | SNORD79 | | | | | |
| 1 | 173834770 | 173834824 | SNORD78 | | | | | |
| 1 | 173835105 | 173835166 | SNORD44 | | | | | |
| 1 | 173835342 | 173835428 | SNORA103 | | | | | |
| 1 | 173835448 | 173835509 | SNORD77 | | | | | |
| 1 | 173835772 | 173835853 | SNORD76 | | | | | |
| 1 | 173836016 | 173836076 | SNORD75 | | | | | |
| 1 | 173836811 | 173836883 | SNORD74 | | | | | |
| 1 | 173837492 | 173855774 | ZBTB37 | | | | | |

| | | | | | | | | |
|---|---|---|---|---|---|---|---|---|
| 1 | 173872941 | 173886516 | SERPINC1 | | | | | |
| 1 | 173900221 | 173962210 | RC3H1 | | | | | |
| 1 | 174090775 | 174128425 | LOC102724601 | | | | | |
| 1 | 174128551 | 174927327 | RABGAP1L | | | | | |
| 1 | 174417211 | 174418683 | GPR52 | | | | | |
| 1 | 174769034 | 174927327 | RABGAP1L | | | | | |
| 1 | 174904083 | 174923398 | LOC101928696 | | | | | |
| 1 | 174933904 | 174964445 | RABGAP1L | | | | | |
| 1 | 174968570 | 174981163 | CACYBP | | CACYBP | | | |
| 1 | 174982093 | 174992591 | MRPS14 | | | | | |
| 1 | 175036993 | 175117202 | TNN | | | | | |
| 1 | 175126122 | 175162229 | KIAA0040 | | | | | |
| 1 | 175291934 | 175712752 | TNR | | | | | |
| 1 | 175846478 | 175849600 | LOC101928751 | | | | | |
| 1 | 175913961 | 176176380 | RFWD2 | | | | | |
| 1 | 175937532 | 175937676 | SCARNA3 | | | | | |
| 1 | 176432306 | 176811970 | PAPPA2 | | | | | |
| 1 | 176826440 | 177134040 | ASTN1 | | | | | |
| 1 | 176998498 | 176998581 | MIR488 | | | | | |
| 1 | 177140523 | 177251558 | BRINP2 | | | | | |
| 1 | 177320721 | 177335594 | LOC102724661 | | | | | |
| 1 | 177669658 | 177679465 | LOC101928778 | | | | | |
| 1 | 177898241 | 177939050 | SEC16B | | | | | |
| 1 | 177975274 | 178007142 | LOC730102 | | | | | |
| 1 | 178060642 | 178448648 | RASAL2 | | | | | |
| 1 | 178482211 | 178492635 | TEX35 | | | | | |
| 1 | 178511930 | 178518024 | C1orf220 | | | | | |
| 1 | 178646883 | 178646969 | MIR4424 | | | | | |
| 1 | 178694281 | 178890977 | RALGPS2 | | | | | |
| 1 | 178818669 | 178840215 | ANGPTL1 | | | | | |
| 1 | 178995073 | 179045702 | FAM20B | | | | | |
| 1 | 179051111 | 179065129 | TOR3A | | | | | |
| 1 | 179068461 | 179198819 | ABL2 | | | | | |
| 1 | 179262848 | 179327814 | SOAT1 | | | | | |
| 1 | 179334854 | 179523870 | AXDND1 | | | | | |
| 1 | 179519673 | 179545087 | NPHS2 | | | | | |
| 1 | 179560747 | 179660407 | TDRD5 | | | | | |
| 1 | 179712297 | 179785333 | FAM163A | | | | | |
| 1 | 179809101 | 179846941 | TOR1AIP2 | | | | | |
| 1 | 179851176 | 179889212 | TOR1AIP1 | | | | | |
| 1 | 179923907 | 180084015 | CEP350 | | | | | |
| 1 | 180123967 | 180167169 | QSOX1 | | | | | |
| 1 | 180167143 | 180169859 | FLJ23867 | | | | | |
| 1 | 180199432 | 180243816 | LHX4 | | | LHX4 | | |
| 1 | 180257351 | 180472022 | ACBD6 | | | | | |
| 1 | 180407448 | 180407525 | MIR3121 | | | | | |
| 1 | 180528109 | 180535654 | OVAAL | | | | | |
| 1 | 180601145 | 180859415 | XPR1 | | | | | |
| 1 | 180882312 | 180915239 | KIAA1614 | | | | | |
| 1 | 180941849 | 180992257 | STX6 | | | | | |
| 1 | 181002560 | 181031074 | MR1 | | | | | |
| 1 | 181057637 | 181059979 | IER5 | | | | | |
| 1 | 181143619 | 181151342 | LOC101928973 | | | | | |
| 1 | 181205523 | 181207740 | GM140 | | | | | |
| 1 | 181452685 | 181775921 | CACNA1E | | CACNA1E | CACNA1E | CACNA1E | |
| 1 | 182023704 | 182030847 | ZNF648 | | | | | |
| 1 | 182173079 | 182283196 | LINC01344 | | | | | |
| 1 | 182347227 | 182361341 | GLUL | | GLUL | | | |
| 1 | 182367251 | 182369751 | TEDDM1 | | | | | |
| 1 | 182376755 | 182383948 | LINC00272 | | | | | |
| 1 | 182419255 | 182529732 | RGSL1 | | | | | |
| 1 | 182542768 | 182558394 | RNASEL | | | | | |
| 1 | 182567757 | 182573548 | RGS16 | | | | | |
| 1 | 182584274 | 182585764 | LOC284648 | | | | | |
| 1 | 182615791 | 182642067 | RGS8 | | | | | |
| 1 | 182758583 | 182799519 | NPL | | | | | |

| | | | | | | | | |
|---|---|---|---|---|---|---|---|---|
| 1 | 182808438 | 182857117 | DHX9 | | | | | |
| 1 | 182868999 | 182922553 | SHCBP1L | | | | | |
| 1 | 182992594 | 183114727 | LAMC1 | | | | | |
| 1 | 183155173 | 183214262 | LAMC2 | | | | | |
| 1 | 183217371 | 183387634 | NMNAT2 | | | | | |
| 1 | 183430010 | 183523328 | SMG7 | | | | | |
| 1 | 183524696 | 183560056 | NCF2 | | | | | |
| 1 | 183595327 | 183605076 | ARPC5 | | | | | |
| 1 | 183605181 | 183897685 | RGL1 | | | | | |
| 1 | 183615410 | 183622448 | APOBEC4 | | | | | |
| 1 | 183774214 | 183897685 | RGL1 | | | | | |
| 1 | 183898795 | 184006904 | COLGALT2 | | | | | |
| 1 | 184020784 | 184043344 | TSEN15 | | | | | |
| 1 | 184356149 | 184598155 | C1orf21 | | | | | |
| 1 | 184659624 | 184724041 | EDEM3 | | | | | |
| 1 | 184760158 | 184943718 | FAM129A | | | | | |
| 1 | 185014550 | 185071740 | RNF2 | | | | | |
| 1 | 185087217 | 185126230 | TRMT1L | | | | | |
| 1 | 185126191 | 185260913 | SWT1 | | | | | |
| 1 | 185265521 | 185286461 | IVNS1ABP | | | | | |
| 1 | 185292978 | 185304171 | GS1-279B7.1 | | | | | |
| 1 | 185527511 | 185597620 | LINC01350 | | | | | |
| 1 | 185703682 | 186160085 | HMCN1 | | | | | |
| 1 | 186029866 | 186446655 | MIR548F1 | | | | | |
| 1 | 186265404 | 186283694 | PRG4 | | | | | |
| 1 | 186280785 | 186344457 | TPR | | | | | |
| 1 | 186344889 | 186390503 | C1orf27 | | | | | |
| 1 | 186369703 | 186370587 | OCLM | | | | | |
| 1 | 186404292 | 186439423 | LOC102724919 | | | | | |
| 1 | 186412697 | 186430240 | PDC | | | | | |
| 1 | 186640943 | 186649559 | PTGS2 | | | | | |
| 1 | 186649785 | 186650578 | PACERR | | | | | |
| 1 | 186798031 | 186958113 | PLA2G4A | | | | | |
| 1 | 187061973 | 187329376 | LINC01036 | | | | | |
| 1 | 187412759 | 187446354 | LINC01037 | | | | | |
| 1 | 190066796 | 190447012 | BRINP3 | | | | | |
| 1 | 190447389 | 190450524 | LINC01351 | | | | | |
| 1 | 190594019 | 190770788 | LOC440704 | | | | | |
| 1 | 192127591 | 192154945 | RGS18 | | | | | |
| 1 | 192286121 | 192336414 | RGS21 | | | | | |
| 1 | 192544856 | 192549159 | RGS1 | | | | | |
| 1 | 192605267 | 192629440 | RGS13 | | | | | |
| 1 | 192685457 | 192685520 | MIR4426 | | | | | |
| 1 | 192778168 | 192781407 | RGS2 | | | RGS2 | | |
| 1 | 192904873 | 192917387 | LINC01032 | | | | | |
| 1 | 192981495 | 193029237 | UCHL5 | | | | | |
| 1 | 193028551 | 193060906 | TROVE2 | | | | | |
| 1 | 193065594 | 193074414 | GLRX2 | | | | | |
| 1 | 193091087 | 193223942 | CDC73 | | | | | |
| 1 | 193105632 | 193105713 | MIR1278 | | | | | |
| 1 | 193147859 | 193155743 | B3GALT2 | | | | | |
| 1 | 193273874 | 193335083 | LINC01031 | | | | | |
| 1 | 196194909 | 196577561 | KCNT2 | | | | | |
| 1 | 196551542 | 196551611 | MIR4735 | | | | | |
| 1 | 196621007 | 196716634 | CFH | | | | | |
| 1 | 196743929 | 196763203 | CFHR3 | | | | | |
| 1 | 196788860 | 196801319 | CFHR1 | | | | | |
| 1 | 196857143 | 196887843 | CFHR4 | | | | | |
| 1 | 196912897 | 196928356 | CFHR2 | | | | | |
| 1 | 196946666 | 196978803 | CFHR5 | | | | | |
| 1 | 197008320 | 197036397 | F13B | | | | | |
| 1 | 197053256 | 197115824 | ASPM | | | | | |
| 1 | 197122813 | 197169672 | ZBTB41 | | | | | |
| 1 | 197170591 | 197447585 | CRB1 | | CRB1 | CRB1 | | |
| 1 | 197473878 | 197744623 | DENND1B | | | | | |
| 1 | 197871681 | 197876497 | C1orf53 | | | | | |

| | | | | | | | | |
|---|---|---|---|---|---|---|---|---|
| 1 | 197881634 | 197899273 | LHX9 | | | | | |
| 1 | 198126107 | 198291548 | NEK7 | | | | | |
| 1 | 198492351 | 198510075 | ATP6V1G3 | | | | | |
| 1 | 198608097 | 198726605 | PTPRC | | | | | |
| 1 | 198777131 | 198906558 | MIR181A1HG | | | | | |
| 1 | 198828001 | 198828111 | MIR181B1 | | | | | |
| 1 | 198828172 | 198828282 | MIR181A1 | | | | | |
| 1 | 198961717 | 198988093 | LINC01222 | | | | | |
| 1 | 198985261 | 199045864 | LINC01221 | | | | | |
| 1 | 199996729 | 200146550 | NR5A2 | | | | | |
| 1 | 200311671 | 200342920 | LINC00862 | | | | | |
| 1 | 200374074 | 200379186 | ZNF281 | | | | | |
| 1 | 200520624 | 200589862 | KIF14 | | | | | |
| 1 | 200613164 | 200639126 | DDX59 | | | | | |
| 1 | 200638634 | 200663378 | LOC101929224 | | | | | |
| 1 | 200708685 | 200829835 | CAMSAP2 | | | | | |
| 1 | 200842082 | 200843306 | GPR25 | | | | | |
| 1 | 200860626 | 200884864 | C1orf106 | | | | | |
| 1 | 200938513 | 200992828 | KIF21B | | KIF21B | KIF21B | | |
| 1 | 201008639 | 201081694 | CACNA1S | | | | CACNA1S | |
| 1 | 201083080 | 201084500 | ASCL5 | | | | | |
| 1 | 201103898 | 201140710 | TMEM9 | | | | | |
| 1 | 201159952 | 201198080 | IGFN1 | | | | | |
| 1 | 201252579 | 201302121 | PKP1 | | | PKP1 | | |
| 1 | 201328135 | 201346828 | TNNT2 | | | TNNT2 | | |
| 1 | 201349965 | 201368669 | LAD1 | | | | | |
| 1 | 201372894 | 201390874 | TNNI1 | | | | | |
| 1 | 201433411 | 201438299 | PHLDA3 | | | | | |
| 1 | 201452657 | 201476387 | CSRP1 | | | | | |
| 1 | 201489031 | 201489720 | RPS10P7 | | | | | |
| 1 | 201617449 | 201796102 | NAV1 | | | | | |
| 1 | 201657383 | 201798687 | IPO9 | | | | | |
| 1 | 201688635 | 201688755 | MIR5191 | | | | | |
| 1 | 201708962 | 201796102 | NAV1 | | | | | |
| 1 | 201777738 | 201777830 | MIR1231 | | | | | |
| 1 | 201798287 | 201853422 | IPO9 | | | | | |
| 1 | 201832500 | 201832575 | MIR6739 | | | | | |
| 1 | 201857796 | 201861715 | SHISA4 | | | | | |
| 1 | 201865583 | 201915716 | LMOD1 | | | | | |
| 1 | 201924618 | 201939789 | TIMM17A | | | TIMM17A | | |
| 1 | 201951765 | 201975275 | RNPEP | | | | | |
| 1 | 201972251 | 201972364 | MIR6740 | | | | | |
| 1 | 201979689 | 201986315 | ELF3 | | | | | |
| 1 | 202092028 | 202098634 | GPR37L1 | | | | | |
| 1 | 202102531 | 202113871 | ARL8A | | | | | |
| 1 | 202116140 | 202130716 | PTPN7 | | | | | |
| 1 | 202137178 | 202158577 | PTPRVP | | | | | |
| 1 | 202163117 | 202288889 | LGR6 | | | | | |
| 1 | 202300784 | 202311094 | UBE2T | | | | | |
| 1 | 202317829 | 202557697 | PPP1R12B | | | | | |
| 1 | 202559724 | 202679551 | SYT2 | | | SYT2 | SYT2 | |
| 1 | 202694312 | 202778598 | KDM5B | | | | | |
| 1 | 202780073 | 202781041 | PCAT6 | | | | | |
| 1 | 202789384 | 202796341 | MGAT4EP | | | | | |
| 1 | 202830881 | 202844369 | LOC148709 | | | | | |
| 1 | 202847409 | 202858385 | RABIF | | | | | |
| 1 | 202860223 | 202897772 | KLHL12 | | | | | |
| 1 | 202909952 | 202927700 | ADIPOR1 | | | | | |
| 1 | 202931000 | 202936404 | CYB5R1 | | | | | |
| 1 | 202955579 | 202976393 | LOC100506747 | | | | | |
| 1 | 202976533 | 202993197 | TMEM183A | | | | | |
| 1 | 202976535 | 202992668 | TMEM183B | | | | | |
| 1 | 202995648 | 203047864 | PPFIA4 | | | | | |
| 1 | 203052256 | 203055166 | MYOG | | | | | |
| 1 | 203096835 | 203136533 | ADORA1 | | | | | |
| 1 | 203136938 | 203144942 | MYBPH | | | | | |

| | | | | | | | | |
|---|---|---|---|---|---|---|---|---|
| 1 | 203148058 | 203155922 | CHI3L1 | | CHI3L1 | | | |
| 1 | 203185206 | 203198860 | CHIT1 | | | | | |
| 1 | 203256279 | 203257924 | LINC01353 | | | | | |
| 1 | 203267885 | 203274453 | LINC01136 | | | | | |
| 1 | 203274663 | 203278729 | BTG2 | | | | | |
| 1 | 203309748 | 203320557 | FMOD | | | | | |
| 1 | 203444882 | 203460479 | PRELP | | | | | |
| 1 | 203463270 | 203478077 | OPTC | | | | | |
| 1 | 203595914 | 203713209 | ATP2B4 | | | ATP2B4 | | |
| 1 | 203698708 | 203698833 | SNORA77 | | | | | |
| 1 | 203699704 | 203700979 | LINC00260 | | | | | |
| 1 | 203734283 | 203745480 | LAX1 | | | | | |
| 1 | 203764664 | 203823256 | ZC3H11A | | | | | |
| 1 | 203766650 | 203769590 | ZBED6 | | | | | |
| 1 | 203830712 | 203840280 | SNRPE | | | | | |
| 1 | 204001574 | 204010392 | LINC00303 | | | | | |
| 1 | 204042245 | 204096871 | SOX13 | | | | | |
| 1 | 204100188 | 204121310 | ETNK2 | | | | | |
| 1 | 204110535 | 204112137 | LOC101929441 | | | | | |
| 1 | 204123943 | 204135465 | REN | | | | | |
| 1 | 204159468 | 204165619 | KISS1 | | | | | |
| 1 | 204167287 | 204183220 | GOLT1A | | | | | |
| 1 | 204187978 | 204329057 | PLEKHA6 | | | | | |
| 1 | 204337557 | 204338847 | LINC00628 | | | | | |
| 1 | 204372491 | 204380945 | PPP1R15B | | | | | |
| 1 | 204391757 | 204459474 | PIK3C2B | | | | | |
| 1 | 204485506 | 204527248 | MDM4 | | | | | |
| 1 | 204586302 | 204654597 | LRRN2 | | | LRRN2 | | |
| 1 | 204797781 | 204991950 | NFASC | | NFASC | NFASC | | |
| 1 | 205012339 | 205047171 | CNTN2 | | CNTN2 | | | |
| 1 | 205052256 | 205053588 | TMEM81 | | | | | |
| 1 | 205055269 | 205091150 | RBBP5 | | | | | |
| 1 | 205111630 | 205180727 | DSTYK | | DSTYK | | | |
| 1 | 205197037 | 205242471 | TMCC2 | | | | | |
| 1 | 205271190 | 205290919 | NUAK2 | | | | | |
| 1 | 205305192 | 205326218 | KLHDC8A | | | | | |
| 1 | 205342379 | 205391214 | LEMD1 | | | | | |
| 1 | 205404013 | 205425214 | BLACAT1 | | | | | |
| 1 | 205417429 | 205417526 | MIR135B | | | | | |
| 1 | 205473683 | 205501921 | CDK18 | | | | | |
| 1 | 205523400 | 205525763 | LOC284578 | | | | | |
| 1 | 205538111 | 205572046 | MFSD4A | | | | | |
| 1 | 205577070 | 205602000 | ELK4 | | | | | |
| 1 | 205626980 | 205649630 | SLC45A3 | | | | | |
| 1 | 205681946 | 205719372 | NUCKS1 | | NUCKS1 | | | |
| 1 | 205737113 | 205744610 | RAB29 | | | | | |
| 1 | 205758220 | 205782324 | SLC41A1 | | | | | |
| 1 | 205797149 | 205819276 | PM20D1 | | | | | |
| 1 | 205831206 | 205865215 | LOC284581 | | | | | |
| 1 | 205882176 | 205912588 | SLC26A9 | | | | | |
| 1 | 206137264 | 206155151 | FAM72A | | | | | |
| 1 | 206138439 | 206155074 | FAM72C | | | | | |
| 1 | 206138454 | 206155151 | FAM72A | | | | | |
| 1 | 206224282 | 206231482 | AVPR1B | | | AVPR1B | | |
| 1 | 206238871 | 206288647 | C1orf186 | | | | | |
| 1 | 206317458 | 206332104 | CTSE | | | | | |
| 1 | 206516199 | 206581301 | SRGAP2C | | | | | |
| 1 | 206516199 | 206637783 | SRGAP2 | | SRGAP2 | | | |
| 1 | 206557366 | 206581301 | SRGAP2D | | | | | |
| 1 | 206643585 | 206670223 | IKBKE | | | | | |
| 1 | 206648145 | 206648207 | MIR6769B | | | | | |
| 1 | 206680862 | 206762616 | RASSF5 | | | | | |
| 1 | 206764973 | 206785904 | EIF2D | | | | | |
| 1 | 206808880 | 206822542 | DYRK3 | | | | | |
| 1 | 206858364 | 206907630 | MAPKAPK2 | | | | | |
| 1 | 206940947 | 206945839 | IL10 | | | | | |

| | | | | | | | | |
|---|---|---|---|---|---|---|---|---|
| 1 | 206972214 | 207016326 | IL19 | | | | | |
| 1 | 207039153 | 207042568 | IL20 | | | | | |
| 1 | 207070787 | 207077484 | IL24 | | | | | |
| 1 | 207076630 | 207095378 | FCMR | | | | | |
| 1 | 207101866 | 207119811 | PIGR | | | | | |
| 1 | 207131311 | 207143970 | FCAMR | | | | | |
| 1 | 207191865 | 207206101 | C1orf116 | | | | | |
| 1 | 207217193 | 207226325 | YOD1 | | | | | |
| 1 | 207226619 | 207254368 | PFKFB2 | | | | | |
| 1 | 207262186 | 207273337 | C4BPB | | | | | |
| 1 | 207277606 | 207318317 | C4BPA | | | C4BPA | | |
| 1 | 207494816 | 207534311 | CD55 | | | | | |
| 1 | 207627644 | 207663240 | CR2 | | | | | |
| 1 | 207669472 | 207815110 | CR1 | | | | | |
| 1 | 207818457 | 207897036 | CR1L | | | | | |
| 1 | 207925382 | 207968861 | CD46 | | | | | |
| 1 | 207975196 | 207975284 | MIR29C | | | | | |
| 1 | 207975787 | 207975868 | MIR29B2 | | | | | |
| 1 | 207991723 | 207995941 | LOC148696 | | | | | |
| 1 | 208059882 | 208084683 | CD34 | | | | | |
| 1 | 208195587 | 208417665 | PLXNA2 | | | | | |
| 1 | 209498800 | 209501877 | LOC105372897 | | | | | |
| 1 | 209602167 | 209605892 | MIR205HG | | | | | |
| 1 | 209605477 | 209605587 | MIR205 | | | | | |
| 1 | 209757044 | 209787284 | CAMK1G | | | CAMK1G | | |
| 1 | 209788217 | 209825820 | LAMB3 | | | | | |
| 1 | 209796788 | 209796855 | MIR4260 | | | | | |
| 1 | 209834703 | 209915907 | LOC101930114 | | | | | |
| 1 | 209848669 | 209849735 | G0S2 | | | | | |
| 1 | 209859524 | 209908295 | HSD11B1 | | | | | |
| 1 | 209929376 | 209953002 | TRAF3IP3 | | | | | |
| 1 | 209952552 | 209958075 | C1orf74 | | | | | |
| 1 | 209958967 | 209979520 | IRF6 | | | IRF6 | | |
| 1 | 210001311 | 210030910 | DIEXF | | | | | |
| 1 | 210111518 | 210337633 | SYT14 | | SYT14 | | | |
| 1 | 210404803 | 210416440 | SERTAD4 | | | | | |
| 1 | 210501595 | 210849638 | HHAT | | | | | |
| 1 | 210851656 | 211307457 | KCNH1 | | | KCNH1 | | |
| 1 | 211432707 | 211489725 | RCOR3 | | | | | |
| 1 | 211499956 | 211548286 | TRAF5 | | | TRAF5 | | |
| 1 | 211556096 | 211605877 | LINC00467 | | | | | |
| 1 | 211649863 | 211666259 | RD3 | | | | | |
| 1 | 211748380 | 211752099 | SLC30A1 | | | | | |
| 1 | 211813149 | 211827963 | LOC105748977 | | | | | |
| 1 | 211831598 | 211848972 | NEK2 | | | | | |
| 1 | 211916798 | 212004114 | LPGAT1 | | | | | |
| 1 | 212113740 | 212209002 | INTS7 | | | | | |
| 1 | 212208894 | 212278348 | DTL | | | | | |
| 1 | 212250954 | 212251027 | MIR3122 | | | | | |
| 1 | 212386343 | 212458363 | LOC101929541 | | | | | |
| 1 | 212458878 | 212535205 | PPP2R5A | | | | | |
| 1 | 212526159 | 212526292 | SNORA16B | | | | | |
| 1 | 212537815 | 212588267 | TMEM206 | | | | | |
| 1 | 212606228 | 212619721 | NENF | | | | | |
| 1 | 212719035 | 212729408 | LOC101929565 | | | | | |
| 1 | 212738675 | 212794119 | ATF3 | | | | ATF3 | |
| 1 | 212797788 | 212800120 | FAM71A | | | | | |
| 1 | 212859758 | 212873327 | BATF3 | | | | | |
| 1 | 212899494 | 212965139 | NSL1 | | | | | |
| 1 | 212965169 | 212990167 | TATDN3 | | | | | |
| 1 | 213003484 | 213020991 | SPATA45 | | | | | |
| 1 | 213029945 | 213072705 | FLVCR1 | | | | | |
| 1 | 213123861 | 213164927 | VASH2 | | | | | |
| 1 | 213165523 | 213189217 | ANGEL2 | | | | | |
| 1 | 213224574 | 213446808 | RPS6KC1 | | | | | |
| 1 | 213992983 | 214159496 | PROX1 | | | | | |

| | | | | | | | | |
|---|---|---|---|---|---|---|---|---|
| 1 | 214098091 | 214099996 | LINC00538 | | | | | |
| 1 | 214161277 | 214214847 | PROX1 | | | | | |
| 1 | 214454564 | 214510477 | SMYD2 | | | | | |
| 1 | 214522038 | 214725024 | PTPN14 | | | | | |
| 1 | 214776531 | 214837914 | CENPF | | | CENPF | | |
| 1 | 215178884 | 215410436 | KCNK2 | | | | | |
| 1 | 215740721 | 215795163 | KCTD3 | | | | | |
| 1 | 215796235 | 216596738 | USH2A | | | | | |
| 1 | 216245806 | 216260259 | LOC102723833 | | | | | |
| 1 | 216347291 | 216596738 | USH2A | | | | | |
| 1 | 216676587 | 217311097 | ESRRG | | ESRRG | | | |
| 1 | 217600334 | 217804444 | GPATCH2 | | | | | |
| 1 | 217804665 | 217958462 | SPATA17 | | | | | |
| 1 | 218066241 | 218094146 | LINC00210 | | | | | |
| 1 | 218216846 | 218232482 | LOC101929631 | | | | | |
| 1 | 218458628 | 218511325 | RRP15 | | | | | |
| 1 | 218517537 | 218617961 | TGFB2 | | | | | |
| 1 | 218610974 | 219281452 | MIR548F3 | | | | | |
| 1 | 218615967 | 218617337 | TGFB2-OT1 | | | | | |
| 1 | 219254316 | 219386207 | LYPLAL1 | | | | | |
| 1 | 220046618 | 220292777 | RNU5F-1 | | | | | |
| 1 | 220087605 | 220101993 | SLC30A10 | | SLC30A10 | | | |
| 1 | 220141941 | 220220000 | EPRS | | | | | |
| 1 | 220230823 | 220263195 | BPNT1 | | | | | |
| 1 | 220267454 | 220321383 | IARS2 | | | | | |
| 1 | 220291194 | 220291304 | MIR215 | | | | | |
| 1 | 220291498 | 220291583 | MIR194 | | | | | |
| 1 | 220321609 | 220445843 | RAB3GAP2 | | | RAB3GAP2 | | |
| 1 | 220373883 | 220373961 | MIR664A | | | | | |
| 1 | 220373887 | 220374018 | SNORA36B | | | | | |
| 1 | 220439520 | 220441057 | AURKAPS1 | | | | | |
| 1 | 220701524 | 220837799 | MARK1 | | | MARK1 | | |
| 1 | 220863627 | 220872499 | C1orf115 | | | | | |
| 1 | 220921587 | 220958157 | MARC2 | | | | | |
| 1 | 220960038 | 220987741 | MARC1 | | | | | |
| 1 | 221002596 | 221005770 | LINC01352 | | | | | |
| 1 | 221006104 | 221058400 | HLX | | | | | |
| 1 | 221503269 | 221509638 | C1orf140 | | | | | |
| 1 | 221874761 | 221915518 | DUSP10 | | | | | |
| 1 | 222001007 | 222014008 | LOC101929771 | | | | | |
| 1 | 222695601 | 222721444 | HHIPL2 | | | | | |
| 1 | 222731243 | 222765975 | TAF1A | | | | | |
| 1 | 222791443 | 222841354 | MIA3 | | | | | |
| 1 | 222841354 | 222885864 | AIDA | | | | | |
| 1 | 222885894 | 222908538 | BROX | | | | | |
| 1 | 222910557 | 222924002 | FAM177B | | | | | |
| 1 | 222988430 | 223179337 | DISP1 | | | DISP1 | | |
| 1 | 223282747 | 223316624 | TLR5 | | | | | |
| 1 | 223394160 | 223537544 | SUSD4 | | | | | |
| 1 | 223566714 | 223568812 | CCDC185 | | | | | |
| 1 | 223714971 | 223853436 | CAPN8 | | | | | |
| 1 | 223889294 | 223963720 | CAPN2 | | | | | |
| 1 | 223967594 | 224033674 | TP53BP2 | | TP53BP2 | | | |
| 1 | 224138967 | 224180346 | GTF2IP20 | | | | | |
| 1 | 224301788 | 224349749 | FBXO28 | | | | | |
| 1 | 224370909 | 224381142 | DEGS1 | | | | | |
| 1 | 224396449 | 224400981 | LOC101927143 | | | | | |
| 1 | 224407314 | 224415745 | LOC101927164 | | | | | |
| 1 | 224415035 | 224517891 | NVL | | | | | |
| 1 | 224444705 | 224444843 | MIR320B2 | | | | | |
| 1 | 224544512 | 224567153 | CNIH4 | | | | | |
| 1 | 224572844 | 224622001 | WDR26 | | | | | |
| 1 | 224585928 | 224586013 | MIR4742 | | | | | |
| 1 | 224804178 | 224928249 | CNIH3 | | | | | |
| 1 | 225117355 | 225155626 | DNAH14 | | | | | |
| 1 | 225589203 | 225616557 | LBR | | | | | |

| | | | | | | | | | |
|---|---|---|---|---|---|---|---|---|---|
| 1 | 225674533 | 225840845 | ENAH | | | | | |
| 1 | 225965514 | 225978168 | SRP9 | | | | | |
| 1 | 225997775 | 226033262 | EPHX1 | | | EPHX1 | | |
| 1 | 226033232 | 226070420 | TMEM63A | | | | | |
| 1 | 226073981 | 226076846 | LEFTY1 | | | | | |
| 1 | 226107576 | 226112040 | PYCR2 | | | | | |
| 1 | 226109779 | 226109842 | MIR6741 | | | | | |
| 1 | 226124297 | 226129083 | LEFTY2 | | | | | |
| 1 | 226170402 | 226187066 | SDE2 | | | | | |
| 1 | 226250407 | 226259703 | H3F3A | | | | | |
| 1 | 226250427 | 226259703 | H3F3AP4 | | | | | |
| 1 | 226332379 | 226374423 | ACBD3 | | | ACBD3 | | |
| 1 | 226411318 | 226414756 | MIXL1 | | | | | |
| 1 | 226418849 | 226497449 | LIN9 | | | | | |
| 1 | 226548391 | 226595801 | PARP1 | | | | | |
| 1 | 226736500 | 226796915 | C1orf95 | | | | | |
| 1 | 226819390 | 226926876 | ITPKB | | | | | |
| 1 | 226843780 | 226862769 | ITPKB-IT1 | | | | | |
| 1 | 227058272 | 227083804 | PSEN2 | PSEN2 | | | | |
| 1 | 227127937 | 227175246 | ADCK3 | | | | | |
| 1 | 227177565 | 227505826 | CDC42BPA | | | | | |
| 1 | 227751219 | 227865144 | ZNF678 | | | | | |
| 1 | 227884732 | 227885408 | ZNF847P | | | | | |
| 1 | 227916239 | 227922055 | LOC100130093 | | | | | |
| 1 | 227918889 | 227923112 | JMJD4 | | | | | |
| 1 | 227922696 | 227968932 | SNAP47 | | | | | |
| 1 | 228003417 | 228034171 | PRSS38 | | | | | |
| 1 | 228109164 | 228135676 | WNT9A | | | | | |
| 1 | 228129290 | 228129384 | MIR5008 | | | | | |
| 1 | 228194722 | 228248972 | WNT3A | | | | | |
| 1 | 228270360 | 228286913 | ARF1 | | | | | |
| 1 | 228284963 | 228285042 | MIR3620 | | | | | |
| 1 | 228288427 | 228291163 | C1orf35 | | | | | |
| 1 | 228294379 | 228297013 | MRPL55 | | | | | |
| 1 | 228327784 | 228336655 | GUK1 | | | | | |
| 1 | 228337414 | 228347527 | GJC2 | | GJC2 | | | |
| 1 | 228351786 | 228369958 | IBA57 | | | | | |
| 1 | 228391206 | 228401365 | C1orf145 | | | | | |
| 1 | 228395830 | 228566575 | OBSCN | | | | | |
| 1 | 228581376 | 228594517 | TRIM11 | | | | | |
| 1 | 228584748 | 228584810 | MIR6742 | | | | | |
| 1 | 228595635 | 228604583 | TRIM17 | | | | | |
| 1 | 228612545 | 228613026 | HIST3H3 | | | | | |
| 1 | 228645064 | 228645560 | HIST3H2A | | | | | |
| 1 | 228645807 | 228646259 | HIST3H2BB | | | | | |
| 1 | 228649774 | 228649853 | MIR4666A | | | | | |
| 1 | 228675067 | 228683889 | RNF187 | | | | | |
| 1 | 228698059 | 228699989 | BTNL10 | | | | | |
| 1 | 228746044 | 228779635 | MIR7641 | | | | | |
| 1 | 228780393 | 228882416 | RHOU | | | | | |
| 1 | 228780656 | 228788159 | DUSP5P1 | | | | | |
| 1 | 228781816 | 228781866 | MIR7641 | | | | | |
| 1 | 228870823 | 228882416 | RHOU | | | | | |
| 1 | 229310429 | 229310548 | MIR4454 | | | | | |
| 1 | 229406808 | 229441640 | RAB4A | | | RAB4A | | |
| 1 | 229440128 | 229441250 | SPHAR | | | | | |
| 1 | 229456751 | 229478688 | CCSAP | | | | | |
| 1 | 229566992 | 229569843 | ACTA1 | | | ACTA1 | | |
| 1 | 229577043 | 229644088 | NUP133 | | | | | |
| 1 | 229644176 | 229649834 | LOC101927478 | | | | | |
| 1 | 229652328 | 229694442 | ABCB10 | | | | | |
| 1 | 229728865 | 229761794 | TAF5L | | | | | |
| 1 | 229761962 | 229795947 | URB2 | | | | | |
| 1 | 230138463 | 230142909 | LOC101927532 | | | | | |
| 1 | 230193535 | 230417876 | GALNT2 | | | | | |
| 1 | 230457391 | 230561674 | PGBD5 | | | | | |

| | | | | | | | | |
|---|---|---|---|---|---|---|---|---|
| 1 | 230778201 | 230829731 | COG2 | | | | | |
| 1 | 230838271 | 230850336 | AGT | | AGT | | | |
| 1 | 230883129 | 230937518 | CAPN9 | | | | | |
| 1 | 230972864 | 231005335 | C1orf198 | | | | | |
| 1 | 231010591 | 231014761 | LOC101927604 | | | | | |
| 1 | 231041986 | 231114618 | TTC13 | | | | | |
| 1 | 231114822 | 231136479 | ARV1 | | | | | |
| 1 | 231154703 | 231175995 | FAM89A | | | | | |
| 1 | 231155573 | 231155670 | MIR1182 | | | | | |
| 1 | 231298673 | 231357314 | TRIM67 | | | | | |
| 1 | 231319844 | 231323373 | LOC149373 | | | | | |
| 1 | 231359508 | 231376933 | C1orf131 | | | | | |
| 1 | 231376918 | 231413719 | GNPAT | | | | | |
| 1 | 231468476 | 231473618 | EXOC8 | | | | | |
| 1 | 231473681 | 231490773 | SPRTN | | | | | |
| 1 | 231499496 | 231560790 | EGLN1 | | | | | |
| 1 | 231611509 | 231612271 | SNRPD2P2 | | | | | |
| 1 | 231664398 | 231702269 | TSNAX | | | | | |
| 1 | 231664398 | 232177019 | TSNAX-DISC1 | | | | | |
| 1 | 231727037 | 231747836 | LINC00582 | | | | | |
| 1 | 231762560 | 232177019 | DISC1 | | | DISC1 | | |
| 1 | 231950371 | 231954263 | DISC2 | | | | | |
| 1 | 232061579 | 232080951 | DISC1-IT1 | | | | | |
| 1 | 232533711 | 232651354 | SIPA1L2 | | | | | |
| 1 | 232862999 | 232878461 | LOC101927683 | | | | | |
| 1 | 232940637 | 232946092 | MAP10 | | | | | |
| 1 | 233086369 | 233114219 | NTPCR | | | | | |
| 1 | 233119881 | 233431459 | PCNX2 | | | | | |
| 1 | 233463513 | 233520894 | KIAA1804 | | | | | |
| 1 | 233749749 | 233808258 | KCNK1 | | | KCNK1 | | |
| 1 | 233759897 | 233759965 | MIR4427 | | | | | |
| 1 | 234040457 | 234460264 | SLC35F3 | | | | | |
| 1 | 234442212 | 234442285 | MIR4671 | | | | | |
| 1 | 234508552 | 234509339 | LOC101927765 | | | | | |
| 1 | 234509182 | 234519795 | COA6 | | | | | |
| 1 | 234527058 | 234614849 | TARBP1 | | | | | |
| 1 | 234663636 | 234667525 | LINC01354 | | | | | |
| 1 | 234740014 | 234745271 | IRF2BP2 | | | | | |
| 1 | 234765056 | 234770526 | LINC00184 | | | | | |
| 1 | 234782034 | 234796670 | LOC101927787 | | | | | |
| 1 | 234859788 | 234867390 | LINC01132 | | | | | |
| 1 | 235093089 | 235099746 | LOC101927851 | | | | | |
| 1 | 235272657 | 235292256 | TOMM20 | | | | | |
| 1 | 235291117 | 235291252 | SNORA14B | | | | | |
| 1 | 235294497 | 235324571 | RBM34 | | | | | |
| 1 | 235330209 | 235491534 | ARID4B | | | | | |
| 1 | 235353348 | 235353431 | MIR4753 | | | | | |
| 1 | 235491752 | 235507844 | GGPS1 | | | | | |
| 1 | 235530674 | 235612283 | TBCE | | | | | |
| 1 | 235610504 | 235667781 | B3GALNT2 | | | | | |
| 1 | 235676124 | 235881413 | MIR5096 | | | | | |
| 1 | 235710984 | 235814054 | GNG4 | | | | | |
| 1 | 235824330 | 236047008 | LYST | | | LYST | | |
| 1 | 236016299 | 236016360 | MIR1537 | | | | | |
| 1 | 236139131 | 236228481 | NID1 | | | | | |
| 1 | 236305831 | 236372209 | GPR137B | | | | | |
| 1 | 236378421 | 236445339 | ERO1B | | | | | |
| 1 | 236557679 | 236648008 | EDARADD | | | | | |
| 1 | 236681513 | 236716279 | LGALS8 | | | | | |
| 1 | 236712304 | 236767841 | HEATR1 | | | | | |
| 1 | 236849753 | 236927927 | ACTN2 | | ACTN2 | | | |
| 1 | 236958580 | 237067281 | MTR | | | MTR | | |
| 1 | 237167402 | 237167718 | MT1HL1 | | | | | |
| 1 | 237205701 | 237997288 | RYR2 | | RYR2 | | | |
| 1 | 237634418 | 237634491 | MIR4428 | | | | | |
| 1 | 238025474 | 238091619 | LOC100130331 | | | | | |

| | | | | | | | | |
|---|---|---|---|---|---|---|---|---|
| 1 | 238041163 | 238054222 | ZP4 | | | | | |
| 1 | 238643683 | 238649317 | LINC01139 | | | | | |
| 1 | 239792372 | 240063172 | CHRM3 | | CHRM3 | CHRM3 | CHRM3 | CHRM3 |
| 1 | 240170823 | 240176560 | RPS7P5 | | | | | |
| 1 | 240255184 | 240638489 | FMN2 | | | | | |
| 1 | 240640408 | 240912217 | MIR1273E | | | | | |
| 1 | 240652872 | 240775462 | GREM2 | | | | | |
| 1 | 240938813 | 241520530 | RGS7 | | | RGS7 | | |
| 1 | 241295571 | 241295646 | MIR3123 | | | | | |
| 1 | 241660856 | 241683085 | FH | | | | | |
| 1 | 241695433 | 241758949 | KMO | | | | | |
| 1 | 241756451 | 241803701 | OPN3 | | | | | |
| 1 | 241792166 | 241799232 | CHML | | | | | |
| 1 | 241815579 | 241965434 | WDR64 | | | | | |
| 1 | 242011492 | 242053241 | EXO1 | | | | | |
| 1 | 242121068 | 242122364 | BECN2 | | | | | |
| 1 | 242158791 | 242162385 | MAP1LC3C | | | | | |
| 1 | 242251688 | 242687998 | PLD5 | | | | | |
| 1 | 243219615 | 243265046 | LINC01347 | | | | | |
| 1 | 243287729 | 243418708 | CEP170 | | | | | |
| 1 | 243419306 | 243663393 | SDCCAG8 | | | | | |
| 1 | 243509477 | 243509557 | MIR4677 | | | | | |
| 1 | 243651534 | 244006886 | AKT3 | | AKT3 | | | |
| 1 | 244080703 | 244210619 | LOC339529 | | | | | |
| 1 | 244212240 | 244220780 | ZBTB18 | | | | | |
| 1 | 244515936 | 244552388 | C1orf100 | | | | | |
| 1 | 244571793 | 244615436 | ADSS | | | | | |
| 1 | 244624672 | 244803662 | C1orf101 | | | | | |
| 1 | 244816349 | 244872336 | DESI2 | | | | | |
| 1 | 244998607 | 245008365 | COX20 | | | | | |
| 1 | 245013601 | 245027827 | HNRNPU | | | | | |
| 1 | 245017388 | 245017519 | SNORA100 | | | | | |
| 1 | 245128505 | 245134171 | LOC101928068 | | | | | |
| 1 | 245133170 | 245288530 | EFCAB2 | | | | | |
| 1 | 245318286 | 245866428 | KIF26B | | | | | |
| 1 | 245912641 | 246670644 | SMYD3 | | | | | |
| 1 | 246679340 | 246687589 | LOC255654 | | | | | |
| 1 | 246703862 | 246729565 | TFB2M | | | | | |
| 1 | 246729638 | 246831884 | CNST | | | | | |
| 1 | 246887377 | 246931440 | SCCPDH | | | | | |
| 1 | 246952918 | 246954788 | LINC01341 | | | | | |
| 1 | 247002401 | 247094726 | AHCTF1 | | | | | |
| 1 | 247108848 | 247171395 | ZNF695 | | | | | |
| 1 | 247108848 | 247242115 | ZNF670-ZNF695 | | | | | |
| 1 | 247148624 | 247171395 | ZNF695 | | | | | |
| 1 | 247197939 | 247242115 | ZNF670 | | | | | |
| 1 | 247263263 | 247267674 | ZNF669 | | | | | |
| 1 | 247273461 | 247275719 | C1orf229 | | | | | |
| 1 | 247285276 | 247335319 | ZNF124 | | | | | |
| 1 | 247365268 | 247365362 | MIR3916 | | | | | |
| 1 | 247419373 | 247420447 | VN1R5 | | | | | |
| 1 | 247463621 | 247495045 | ZNF496 | | | | | |
| 1 | 247579457 | 247612406 | NLRP3 | | | | | |
| 1 | 247614330 | 247615284 | OR2B11 | | | | | |
| 1 | 247654369 | 247655711 | OR2W5 | | | | | |
| 1 | 247670359 | 247694106 | GCSAML | | | | | |
| 1 | 247693433 | 247697141 | OR2C3 | | | | | |
| 1 | 247712346 | 247740992 | GCSAML | | | | | |
| 1 | 247751661 | 247752615 | OR2G2 | | | | | |
| 1 | 247768887 | 247769817 | OR2G3 | | | | | |
| 1 | 247835419 | 247836343 | OR13G1 | | | | | |
| 1 | 247875130 | 247876057 | OR6F1 | | | | | |
| 1 | 247920763 | 247921708 | OR1C1 | | | | | |
| 1 | 247978101 | 247979031 | OR14A16 | | | | | |
| 1 | 248004229 | 248005198 | OR11L1 | | | | | |
| 1 | 248020500 | 248043438 | TRIM58 | | | | | |

| | | | | | | | | |
|---|---|---|---|---|---|---|---|---|
| 1 | 248058888 | 248059833 | OR2W3 | | | OR2W3 | | |
| 1 | 248084319 | 248085258 | OR2T8 | | | | | |
| 1 | 248100330 | 248264224 | OR2L13 | | | | | |
| 1 | 248112159 | 248113098 | OR2L8 | | | | | |
| 1 | 248128633 | 248129641 | OR2AK2 | | | | | |
| 1 | 248153568 | 248154493 | OR2L1P | | | | | |
| 1 | 248185249 | 248186188 | OR2L5 | | | | | |
| 1 | 248201473 | 248202607 | OR2L2 | | | | | |
| 1 | 248223983 | 248224922 | OR2L3 | | | | | |
| 1 | 248285437 | 248286082 | OR2M1P | | | | | |
| 1 | 248308449 | 248309388 | OR2M5 | | | | | |
| 1 | 248343287 | 248344331 | OR2M2 | | | | | |
| 1 | 248366369 | 248367308 | OR2M3 | | | | | |
| 1 | 248402230 | 248403166 | OR2M4 | | | | | |
| 1 | 248436153 | 248437116 | OR2T33 | | | | | |
| 1 | 248457917 | 248458880 | OR2T12 | | | | | |
| 1 | 248486931 | 248487870 | OR2M7 | | | | | |
| 1 | 248512076 | 248513015 | OR14C36 | | | | | |
| 1 | 248524882 | 248525929 | OR2T4 | | | | | |
| 1 | 248550909 | 248551836 | OR2T6 | | | | | |
| 1 | 248569295 | 248570405 | OR2T1 | | | | | |
| 1 | 248616098 | 248617073 | OR2T2 | | | | | |
| 1 | 248636651 | 248637608 | OR2T3 | | | | | |
| 1 | 248651889 | 248652837 | OR2T5 | | | | | |
| 1 | 248684947 | 248685898 | OR2G6 | | | | | |
| 1 | 248721844 | 248722792 | OR2T29 | | | | | |
| 1 | 248737101 | 248738058 | OR2T34 | | | | | |
| 1 | 248756130 | 248757069 | OR2T10 | | | | | |
| 1 | 248789478 | 248790429 | OR2T11 | | | | | |
| 1 | 248801587 | 248802559 | OR2T35 | | | | | |
| 1 | 248813231 | 248814185 | OR2T27 | | | | | |
| 1 | 248844669 | 248845605 | OR14I1 | | | | | |
| 1 | 248860362 | 248862411 | LOC101928226 | | | | | |
| 1 | 248902716 | 248903151 | LYPD8 | | | | | |
| 1 | 249104650 | 249120154 | SH3BP5L | | | | | |
| 1 | 249120575 | 249120642 | MIR3124 | | | | | |
| 1 | 249132376 | 249143716 | ZNF672 | | | | | |
| 1 | 249144202 | 249153315 | ZNF692 | | | | | |
| 1 | 249200441 | 249213345 | PGBD2 | | | | | |
| 2 | 38813 | 46588 | FAM110C | | | | | |
| 2 | 218135 | 264866 | SH3YL1 | | | | | |
| 2 | 264868 | 278282 | ACP1 | | | | | |
| 2 | 279560 | 288308 | FAM150B | | | | | |
| 2 | 667972 | 677439 | TMEM18 | | | | | |
| 2 | 779836 | 864112 | LINC01115 | | | | | |
| 2 | 895901 | 901137 | LOC101060385 | | | | | |
| 2 | 946553 | 1371384 | SNTG2 | | | SNTG2 | SNTG2 | |
| 2 | 1417232 | 1546499 | TPO | | | TPO | | |
| 2 | 1635658 | 1748319 | PXDN | | | | | |
| 2 | 1792884 | 2330880 | MYT1L | | | | | |
| 2 | 2898819 | 3129798 | LINC01250 | | | | | |
| 2 | 3192740 | 3381653 | TSSC1 | | | | | |
| 2 | 3383445 | 3483342 | TRAPPC12 | | | | | |
| 2 | 3501689 | 3523350 | ADI1 | | | | | |
| 2 | 3592481 | 3609340 | RNASEH1 | | | | | |
| 2 | 3622852 | 3628509 | RPS7 | | | | | |
| 2 | 3642421 | 3692234 | COLEC11 | | | | | |
| 2 | 3705785 | 3750260 | ALLC | | | | | |
| 2 | 3751181 | 3895000 | DCDC2C | | | | | |
| 2 | 4005244 | 4021622 | LINC01304 | | | | | |
| 2 | 4675807 | 4703812 | LINC01249 | | | | | |
| 2 | 5774272 | 5831250 | LINC01248 | | | | | |
| 2 | 5832798 | 5841517 | SOX11 | | SOX11 | | | |
| 2 | 6072818 | 6120350 | LINC01105 | | | | | |
| 2 | 6114793 | 6114864 | MIR7158 | | | | | |
| 2 | 6122109 | 6128364 | LOC400940 | | | | | |

| | | | | | | | | |
|---|---|---|---|---|---|---|---|---|
| 2 | 6506141 | 6515554 | LINC01247 | | | | | |
| 2 | 6766240 | 6774838 | LINC01246 | | | | | |
| 2 | 6789238 | 6790651 | MIR7515HG | | | | | |
| 2 | 6790504 | 6790571 | MIR7515 | | | | | |
| 2 | 6869299 | 6910442 | LINC00487 | | | | | |
| 2 | 6968684 | 6980595 | NRIR | | | | | |
| 2 | 6980683 | 7005950 | CMPK2 | | | | | |
| 2 | 7017795 | 7038363 | RSAD2 | | | | | |
| 2 | 7052406 | 7184309 | RNF144A | | | | | |
| 2 | 7202857 | 7218011 | LOC101929452 | | | | | |
| 2 | 7561391 | 7590381 | LOC100506274 | | | | | |
| 2 | 8026897 | 8039786 | LOC101929551 | | | | | |
| 2 | 8062555 | 8116945 | LINC00298 | | | | | |
| 2 | 8147900 | 8468549 | LINC00299 | | | | | |
| 2 | 8699962 | 8723922 | LOC101929567 | | | | | |
| 2 | 8811113 | 8824583 | ID2 | | | | | |
| 2 | 8868986 | 8977755 | KIDINS220 | | | | | |
| 2 | 8996700 | 9143876 | MBOAT2 | | | | | |
| 2 | 9346893 | 9545812 | ASAP2 | | | | | |
| 2 | 9545809 | 9563698 | ITGB1BP1 | | | | | |
| 2 | 9563665 | 9613239 | CPSF3 | | | | | |
| 2 | 9614669 | 9628591 | IAH1 | | | | | |
| 2 | 9629410 | 9695917 | ADAM17 | | | | | |
| 2 | 9724095 | 9771184 | YWHAQ | | | YWHAQ | | |
| 2 | 9983570 | 10074546 | TAF1B | | | | | |
| 2 | 10091791 | 10142412 | GRHL1 | | | | | |
| 2 | 10183681 | 10194963 | KLF11 | | | | | |
| 2 | 10196925 | 10220538 | CYS1 | | | | | |
| 2 | 10262694 | 10271546 | RRM2 | | | | | |
| 2 | 10281508 | 10351856 | C2orf48 | | | | | |
| 2 | 10332739 | 10332797 | MIR4261 | | | | | |
| 2 | 10443029 | 10567743 | HPCAL1 | | | HPCAL1 | | |
| 2 | 10580496 | 10588680 | ODC1 | | | | | |
| 2 | 10586839 | 10586975 | SNORA80B | | | | | |
| 2 | 10589853 | 10595678 | LOC101929715 | | | | | |
| 2 | 10710891 | 10830113 | NOL10 | | | | | |
| 2 | 10861774 | 10925236 | ATP6V1C2 | | | | | |
| 2 | 10923516 | 10978103 | PDIA6 | | | | | |
| 2 | 11018392 | 11025244 | LOC101929733 | | | | | |
| 2 | 11052062 | 11054351 | KCNF1 | | KCNF1 | KCNF1 | | |
| 2 | 11239976 | 11272302 | FLJ33534 | | | | | |
| 2 | 11273178 | 11286916 | C2orf50 | | | | | |
| 2 | 11295497 | 11318998 | PQLC3 | | | | | |
| 2 | 11321777 | 11484711 | ROCK2 | | | | | |
| 2 | 11534106 | 11543203 | LINC00570 | | | | | |
| 2 | 11584500 | 11606303 | E2F6 | | | | | |
| 2 | 11674241 | 11728355 | GREB1 | | GREB1 | | | |
| 2 | 11680730 | 11680803 | MIR4429 | | | | | |
| 2 | 11682850 | 11732272 | GREB1 | | GREB1 | | | |
| 2 | 11798303 | 11810329 | NTSR2 | | | NTSR2 | | |
| 2 | 11817704 | 11967533 | LPIN1 | | | | | |
| 2 | 11907569 | 11907651 | MIR548S | | | | | |
| 2 | 11977058 | 11977112 | MIR4262 | | | | | |
| 2 | 12147241 | 12718474 | MIR3681HG | | | | | |
| 2 | 12339255 | 12339327 | MIR3681 | | | | | |
| 2 | 12856997 | 12882858 | TRIB2 | | | | | |
| 2 | 12877492 | 12877570 | MIR3125 | | | | | |
| 2 | 13106907 | 13147138 | LOC100506474 | | | | | |
| 2 | 14368997 | 14541082 | LINC00276 | | | | | |
| 2 | 14772809 | 14780168 | FAM84A | | | | | |
| 2 | 14775214 | 14790943 | LOC653602 | | | | | |
| 2 | 15307031 | 15701472 | NBAS | | | | | |
| 2 | 15731744 | 15771235 | DDX1 | | | DDX1 | | |
| 2 | 15830905 | 15859085 | LOC101926966 | | | | | |
| 2 | 16060520 | 16076140 | MYCNUT | | | | | |
| 2 | 16076386 | 16081845 | MYCNOS | | | | | |

| | | | | | | | | |
|---|---|---|---|---|---|---|---|---|
| 2 | 16080559 | 16087129 | MYCN | | MYCN | MYCN | | |
| 2 | 16190548 | 16225811 | GACAT3 | | | | | |
| 2 | 16730729 | 16847134 | FAM49A | | | | | |
| 2 | 17691985 | 17699706 | RAD51AP2 | | | | | |
| 2 | 17721806 | 17837706 | VSNL1 | | | | | |
| 2 | 17845078 | 17935096 | SMC6 | | | | | |
| 2 | 17935176 | 17966632 | GEN1 | | | | | |
| 2 | 17997785 | 17998367 | MSGN1 | | | | | |
| 2 | 18059113 | 18114225 | KCNS3 | | | KCNS3 | | |
| 2 | 18735988 | 18741959 | RDH14 | | | | | |
| 2 | 18735988 | 18770846 | NT5C1B-RDH14 | | | | | |
| 2 | 18744136 | 18770846 | NT5C1B | | | | | |
| 2 | 19548189 | 19548266 | MIR4757 | | | | | |
| 2 | 19551245 | 19558372 | OSR1 | | | | | |
| 2 | 20068614 | 20084808 | LINC00954 | | | | | |
| 2 | 20096391 | 20101747 | TTC32 | | | | | |
| 2 | 20110028 | 20189884 | WDR35 | | | | | |
| 2 | 20189964 | 20204567 | LOC101928222 | | | | | |
| 2 | 20191812 | 20212455 | MATN3 | | MATN3 | MATN3 | | |
| 2 | 20232410 | 20251789 | LAPTM4A | | | | | |
| 2 | 20400557 | 20425194 | SDC1 | | | | | |
| 2 | 20448452 | 20550611 | PUM2 | | | | | |
| 2 | 20646831 | 20649204 | RHOB | | | RHOB | | |
| 2 | 20790534 | 20792308 | HS1BP3-IT1 | | | | | |
| 2 | 20817563 | 20850864 | HS1BP3 | | | | | |
| 2 | 20866423 | 20871250 | GDF7 | | | | | |
| 2 | 20883773 | 21022890 | LDAH | | | | | |
| 2 | 21224300 | 21266945 | APOB | | | APOB | | |
| 2 | 21346857 | 21367238 | TDRD15 | | | | | |
| 2 | 21910305 | 21933524 | LOC645949 | | | | | |
| 2 | 22759350 | 22761159 | LOC102723362 | | | | | |
| 2 | 23608297 | 23931483 | KLHL29 | | | | | |
| 2 | 23971533 | 24149984 | ATAD2B | | | | | |
| 2 | 24163375 | 24223693 | UBXN2A | | | | | |
| 2 | 24232952 | 24247145 | MFSD2B | | | | | |
| 2 | 24252205 | 24270296 | C2orf44 | | | | | |
| 2 | 24272583 | 24286550 | FKBP1B | | | FKBP1B | | |
| 2 | 24290453 | 24299314 | SF3B6 | | | | | |
| 2 | 24299743 | 24392507 | FAM228B | | | | | |
| 2 | 24300302 | 24308085 | TP53I3 | | | | | |
| 2 | 24337678 | 24346347 | PFN4 | | | | | |
| 2 | 24346349 | 24392507 | FAM228B | | | | | |
| 2 | 24397911 | 24414567 | FAM228A | | | | | |
| 2 | 24425734 | 24583397 | ITSN2 | | | | ITSN2 | |
| 2 | 24807345 | 24993570 | NCOA1 | | | | | |
| 2 | 25013135 | 25016251 | PTRHD1 | | | | | |
| 2 | 25016174 | 25045245 | CENPO | | | | | |
| 2 | 25042038 | 25142055 | ADCY3 | | | | | |
| 2 | 25166504 | 25262563 | DNAJC27 | | | | | |
| 2 | 25264972 | 25382004 | EFR3B | | | | | |
| 2 | 25383721 | 25391720 | POMC | | | | POMC | |
| 2 | 25427181 | 25432071 | LINC01381 | | | | | |
| 2 | 25455829 | 25565459 | DNMT3A | | | | | |
| 2 | 25551508 | 25551590 | MIR1301 | | | | | |
| 2 | 25600066 | 25896516 | DTNB | | | DTNB | | |
| 2 | 25962252 | 26101312 | ASXL2 | | | | | |
| 2 | 26149454 | 26205443 | KIF3C | | | | | |
| 2 | 26256728 | 26360323 | RAB10 | | | RAB10 | | |
| 2 | 26395959 | 26412532 | GAREM2 | | | | | |
| 2 | 26413503 | 26467594 | HADHA | | | HADHA | | |
| 2 | 26467615 | 26513333 | HADHB | | | HADHB | | |
| 2 | 26531040 | 26541917 | ADGRF3 | | | | | |
| 2 | 26568953 | 26618759 | EPT1 | | | | | |
| 2 | 26624779 | 26679579 | DRC1 | | | | | |
| 2 | 26680070 | 26781566 | OTOF | | OTOF | | | |
| 2 | 26785480 | 26802395 | C2orf70 | | | | | |

| | | | | | | | | |
|---|---|---|---|---|---|---|---|---|
| 2 | 26804072 | 26864211 | CIB4 | | | | | |
| 2 | 26915580 | 26954066 | KCNK3 | | KCNK3 | | KCNK3 | |
| 2 | 26987141 | 27004099 | SLC35F6 | | | | | |
| 2 | 27008881 | 27017455 | CENPA | | | | | |
| 2 | 27070968 | 27173219 | DPYSL5 | | | DPYSL5 | | |
| 2 | 27193238 | 27250087 | MAPRE3 | | | | | |
| 2 | 27255773 | 27264565 | TMEM214 | | | | | |
| 2 | 27272550 | 27293490 | AGBL5 | | | | | |
| 2 | 27293341 | 27294567 | OST4 | | | | | |
| 2 | 27301434 | 27309265 | EMILIN1 | | | | | |
| 2 | 27309610 | 27323619 | KHK | | | | | |
| 2 | 27322220 | 27341995 | CGREF1 | | | | | |
| 2 | 27346656 | 27353680 | ABHD1 | | | | | |
| 2 | 27353624 | 27357542 | PREB | | | PREB | | |
| 2 | 27359714 | 27362332 | PRR30 | | | | | |
| 2 | 27371944 | 27375819 | TCF23 | | | TCF23 | | |
| 2 | 27422454 | 27435175 | SLC5A6 | | | | | |
| 2 | 27434898 | 27440046 | ATRAID | | | | | |
| 2 | 27440257 | 27466660 | CAD | | | | | |
| 2 | 27477439 | 27498685 | SLC30A3 | | | SLC30A3 | | |
| 2 | 27498288 | 27504298 | DNAJC5G | | | | | |
| 2 | 27505296 | 27530307 | TRIM54 | | | | | |
| 2 | 27530264 | 27531130 | UCN | UCN | UCN | | | |
| 2 | 27532359 | 27545969 | MPV17 | | | | | |
| 2 | 27548715 | 27560670 | GTF3C2 | | | | | |
| 2 | 27587218 | 27593324 | EIF2B4 | | | | | |
| 2 | 27593362 | 27600400 | SNX17 | | | SNX17 | | |
| 2 | 27600097 | 27603611 | ZNF513 | | | | | |
| 2 | 27604065 | 27632550 | PPM1G | | | | | |
| 2 | 27615489 | 27616443 | FTH1P3 | | | | | |
| 2 | 27651472 | 27665124 | NRBP1 | | | | | |
| 2 | 27665232 | 27669348 | KRTCAP3 | | | | | |
| 2 | 27667239 | 27712678 | IFT172 | | | | | |
| 2 | 27714749 | 27718126 | FNDC4 | | | | | |
| 2 | 27719705 | 27746550 | GCKR | | | | | |
| 2 | 27799388 | 27805589 | C2orf16 | | | | | |
| 2 | 27805835 | 27846082 | ZNF512 | | | | | |
| 2 | 27848505 | 27851898 | CCDC121 | | | | | |
| 2 | 27851514 | 27873713 | GPN1 | | | | | |
| 2 | 27873675 | 27886707 | SUPT7L | | | | | |
| 2 | 27886337 | 27917847 | SLC4A1AP | | | | | |
| 2 | 27994583 | 28002608 | MRPL33 | | | | | |
| 2 | 28004230 | 28113263 | RBKS | | | | | |
| 2 | 28112322 | 28561767 | BRE | | | | | |
| 2 | 28219233 | 28219316 | MIR4263 | | | | | |
| 2 | 28371423 | 28429828 | LOC100505736 | | | | | |
| 2 | 28530557 | 28533326 | LOC100505716 | | | | | |
| 2 | 28607275 | 28617539 | FLJ31356 | | | | | |
| 2 | 28615778 | 28637516 | FOSL2 | | | | | |
| 2 | 28718937 | 28866653 | PLB1 | | | | | |
| 2 | 28974613 | 29025806 | PPP1CB | | | PPP1CB | | |
| 2 | 29033699 | 29063675 | SPDYA | | | | | |
| 2 | 29072687 | 29093175 | TRMT61B | | | | | |
| 2 | 29117508 | 29171086 | WDR43 | | | | | |
| 2 | 29136527 | 29136616 | SNORD92 | | | | | |
| 2 | 29149932 | 29150008 | SNORD53 | | | | | |
| 2 | 29204163 | 29275096 | FAM179A | | | | | |
| 2 | 29284555 | 29297127 | C2orf71 | | | | | |
| 2 | 29320541 | 29406679 | CLIP4 | | | | | |
| 2 | 29415639 | 30144477 | ALK | | ALK | ALK | | |
| 2 | 30369749 | 30383399 | YPEL5 | | | | | |
| 2 | 30454396 | 30482899 | LBH | | | | | |
| 2 | 30569511 | 30573024 | LOC285043 | | | | | |
| 2 | 30670091 | 30867091 | LCLAT1 | | | | | |
| 2 | 30945637 | 31030311 | CAPN13 | | | | | |
| 2 | 31133330 | 31361592 | GALNT14 | | | | | |

| | | | | | | | | |
|---|---|---|---|---|---|---|---|---|
| 2 | 31395921 | 31440411 | CAPN14 | | | | | |
| 2 | 31456879 | 31491260 | EHD3 | | | | | |
| 2 | 31557187 | 31637611 | XDH | | | | | |
| 2 | 31749655 | 31806040 | SRD5A2 | | | SRD5A2 | | |
| 2 | 32092878 | 32236121 | MEMO1 | | | | | |
| 2 | 32248971 | 32264844 | DPY30 | | | | | |
| 2 | 32288679 | 32382706 | SPAST | | SPAST | SPAST | | |
| 2 | 32390909 | 32449181 | SLC30A6 | | | | | |
| 2 | 32449517 | 32490812 | NLRC4 | | | | | |
| 2 | 32502957 | 32531658 | YIPF4 | | | | | |
| 2 | 32582095 | 32843965 | BIRC6 | | | | | |
| 2 | 32757219 | 32757313 | MIR558 | | | | | |
| 2 | 32782587 | 32799885 | BIRC6 | | | | | |
| 2 | 32853086 | 33046118 | TTC27 | | | | | |
| 2 | 32860321 | 32860398 | MIR4765 | | | | | |
| 2 | 33050509 | 33151589 | LINC00486 | | | | | |
| 2 | 33152193 | 33171202 | LOC100271832 | | | | | |
| 2 | 33172368 | 33624575 | LTBP1 | | | | | |
| 2 | 33643582 | 33643631 | MIR4430 | | | | | |
| 2 | 33661415 | 33789798 | RASGRP3 | | | | | |
| 2 | 33808727 | 33824429 | FAM98A | | | | | |
| 2 | 33931952 | 34522813 | LINC01317 | | | | | |
| 2 | 33951127 | 33953284 | MYADML | | | | | |
| 2 | 34292292 | 34294617 | LINC01318 | | | | | |
| 2 | 34902623 | 34947630 | LINC01320 | | | | | |
| 2 | 36581891 | 36582713 | LOC100288911 | | | | | |
| 2 | 36583369 | 36778278 | CRIM1 | | | CRIM1 | | |
| 2 | 36779403 | 36825332 | FEZ2 | | | FEZ2 | | |
| 2 | 36923832 | 37041937 | VIT | | | | | |
| 2 | 37064840 | 37193615 | STRN | | | | | |
| 2 | 37208143 | 37311488 | HEATR5B | | | | | |
| 2 | 37311593 | 37326387 | GPATCH11 | | | | | |
| 2 | 37332283 | 37384190 | EIF2AK2 | | | | | |
| 2 | 37394962 | 37415690 | SULT6B1 | | | SULT6B1 | | |
| 2 | 37423634 | 37431886 | CEBPZOS | | | | | |
| 2 | 37428774 | 37458740 | CEBPZ | | | | | |
| 2 | 37458773 | 37476303 | NDUFAF7 | | | | | |
| 2 | 37477645 | 37544222 | PRKD3 | | | | | |
| 2 | 37571752 | 37600465 | QPCT | | | | | |
| 2 | 37869024 | 37899678 | CDC42EP3 | | | | | |
| 2 | 38053389 | 38103006 | LINC00211 | | | | | |
| 2 | 38152461 | 38294285 | RMDN2 | | | | | |
| 2 | 38294745 | 38408993 | CYP1B1 | | | | | |
| 2 | 38521098 | 38604432 | ATL2 | | | | | |
| 2 | 38685779 | 38742882 | LOC101929596 | | | | | |
| 2 | 38790327 | 38830178 | HNRNPLL | | | | | |
| 2 | 38893051 | 38961909 | GALM | | | | | |
| 2 | 38970740 | 38978636 | SRSF7 | | | | | |
| 2 | 39005326 | 39009106 | GEMIN6 | | | | | |
| 2 | 39024875 | 39103021 | DHX57 | | | | | |
| 2 | 39103102 | 39109850 | MORN2 | | | | | |
| 2 | 39146503 | 39202590 | ARHGEF33 | | | | | |
| 2 | 39186428 | 39187485 | LOC375196 | | | | | |
| 2 | 39208689 | 39347604 | SOS1 | | | SOS1 | | SOS1 |
| 2 | 39405687 | 39456673 | CDKL4 | | | | | |
| 2 | 39476406 | 39664453 | MAP4K3 | | | MAP4K3 | | |
| 2 | 39664556 | 39828484 | LOC728730 | | | | | |
| 2 | 39892637 | 39945104 | TMEM178A | | | | | |
| 2 | 39963199 | 40006416 | THUMPD2 | | | | | |
| 2 | 40144773 | 40739575 | SLC8A1 | | | | | |
| 2 | 42104694 | 42121186 | LOC388942 | | | | | |
| 2 | 42158738 | 42160863 | LOC101929723 | | | | | |
| 2 | 42162507 | 42180943 | C2orf91 | | | | | |
| 2 | 42275160 | 42285668 | PKDCC | | | | | |
| 2 | 42369574 | 42397371 | LOC102723824 | | | | | |
| 2 | 42396477 | 42559688 | EML4 | | | | | |

| | | | | | | | | |
|---|---|---|---|---|---|---|---|---|
| 2 | 42562700 | 42596097 | COX7A2L | | | | | |
| 2 | 42669156 | 42721237 | KCNG3 | | | KCNG3 | | |
| 2 | 42721708 | 42936686 | MTA3 | | | | | |
| 2 | 42989638 | 42991401 | OXER1 | | | | | |
| 2 | 42994228 | 43019751 | HAAO | | | | | |
| 2 | 43254991 | 43266682 | LOC102723854 | | | | | |
| 2 | 43449540 | 43453745 | ZFP36L2 | | | | | |
| 2 | 43454349 | 43455994 | LINC01126 | | | | | |
| 2 | 43457974 | 43823185 | THADA | | | | | |
| 2 | 43864438 | 43995126 | PLEKHH2 | | | | | |
| 2 | 43902291 | 43903461 | C1GALT1C1L | | | | | |
| 2 | 44001177 | 44037149 | DYNC2LI1 | | | | | |
| 2 | 44039610 | 44065958 | ABCG5 | | | | | |
| 2 | 44066102 | 44105605 | ABCG8 | | | ABCG8 | | |
| 2 | 44113362 | 44223144 | LRPPRC | | | | | |
| 2 | 44395941 | 44461742 | PPM1B | | | | | |
| 2 | 44502596 | 44547962 | SLC3A1 | | | SLC3A1 | | |
| 2 | 44544747 | 44589001 | PREPL | | PREPL | | | |
| 2 | 44589042 | 44999731 | CAMKMT | | | | | |
| 2 | 44776629 | 44776659 | MIR548AD | | | | | |
| 2 | 45167292 | 45173216 | SIX3 | | SIX3 | SIX3 | | |
| 2 | 45232323 | 45236542 | SIX2 | | | | | |
| 2 | 45401479 | 45482080 | LINC01121 | | | | | |
| 2 | 45615818 | 45838433 | SRBD1 | | | | | |
| 2 | 45879042 | 46415129 | PRKCE | | | PRKCE | | |
| 2 | 46524540 | 46613842 | EPAS1 | | | EPAS1 | | |
| 2 | 46656328 | 46668972 | LOC101805491 | | | | | |
| 2 | 46706703 | 46711564 | TMEM247 | | | | | |
| 2 | 46738973 | 46769696 | ATP6V1E2 | | | | | |
| 2 | 46769866 | 46811827 | RHOQ | | | | | |
| 2 | 46795395 | 46807377 | LOC100506142 | | | | | |
| 2 | 46808412 | 46844251 | PIGF | | | | | |
| 2 | 46844310 | 46857315 | CRIPT | | | | | CRIPT |
| 2 | 46926098 | 46989927 | SOCS5 | | | | | |
| 2 | 47043806 | 47049799 | LINC01118 | | | | | |
| 2 | 47055002 | 47086145 | LINC01119 | | | | | |
| 2 | 47129008 | 47168994 | MCFD2 | | | | | |
| 2 | 47143267 | 47303275 | TTC7A | | | | | |
| 2 | 47314129 | 47382517 | C2orf61 | | | | | |
| 2 | 47387220 | 47404075 | CALM2 | | CALM2 | | | |
| 2 | 47419543 | 47572213 | LOC101927043 | | | | | |
| 2 | 47596286 | 47614167 | EPCAM | | | | | |
| 2 | 47604813 | 47604909 | MIR559 | | | | | |
| 2 | 47630205 | 47710367 | MSH2 | | | | | |
| 2 | 47747914 | 47797470 | KCNK12 | | | | | |
| 2 | 47754675 | 47756303 | HCG2040054 | | | | | |
| 2 | 48010220 | 48034092 | MSH6 | | | | | |
| 2 | 48034058 | 48132932 | FBXO11 | | | | | |
| 2 | 48541794 | 48606434 | FOXN2 | | | | | |
| 2 | 48667907 | 48742531 | PPP1R21 | | | | | |
| 2 | 48757063 | 49003656 | STON1-GTF2A1L | | | | | |
| 2 | 48757307 | 48825654 | STON1 | | | | | |
| 2 | 48796158 | 48906748 | STON1-GTF2A1L | | | | | |
| 2 | 48844918 | 48906751 | GTF2A1L | | | GTF2A1L | | |
| 2 | 48913912 | 48982880 | LHCGR | | | LHCGR | | |
| 2 | 49189295 | 49381666 | FSHR | | | | | |
| 2 | 49286741 | 49286797 | MIR548BA | | | | | |
| 2 | 50145642 | 51259674 | NRXN1 | | NRXN1 | NRXN1 | NRXN1 | NRXN1 |
| 2 | 50923309 | 50923400 | MIR8485 | | | | | |
| 2 | 52929659 | 52929753 | MIR4431 | | | | | |
| 2 | 53897116 | 54014146 | ASB3 | | | | | |
| 2 | 53897449 | 54087170 | GPR75-ASB3 | | | | | |
| 2 | 53994928 | 54002287 | CHAC2 | | | | | |
| 2 | 54014067 | 54045956 | ERLEC1 | | | | | |
| 2 | 54076258 | 54076342 | MIR3682 | | | | | |
| 2 | 54080049 | 54087170 | GPR75 | | | GPR75 | | |

| | | | | | | | | |
|---|---|---|---|---|---|---|---|---|
| 2 | 54091203 | 54197977 | PSME4 | | | | | |
| 2 | 54342409 | 54532435 | ACYP2 | | | | | |
| 2 | 54480314 | 54483409 | TSPYL6 | | | | | |
| 2 | 54558070 | 54588714 | C2orf73 | | | | | |
| 2 | 54683453 | 54898583 | SPTBN1 | | | SPTBN1 | | |
| 2 | 54756358 | 54756978 | RPL23AP32 | | | | | |
| 2 | 54785530 | 54889445 | SPTBN1 | | | SPTBN1 | | |
| 2 | 54952148 | 55199156 | EML6 | | | | | |
| 2 | 55199326 | 55277734 | RTN4 | | RTN4 | RTN4 | | |
| 2 | 55399686 | 55459699 | CLHC1 | | | | | |
| 2 | 55459038 | 55462989 | RPS27A | | | | | |
| 2 | 55461299 | 55461985 | MIR4426 | | | | | |
| 2 | 55463755 | 55496384 | MTIF2 | | | | | |
| 2 | 55509454 | 55511607 | PRORSD1P | | | | | |
| 2 | 55514977 | 55647057 | CCDC88A | | CCDC88A | | | |
| 2 | 55746730 | 55772216 | CFAP36 | | | | | |
| 2 | 55774427 | 55844860 | PPP4R3B | | | | | |
| 2 | 55861197 | 55921045 | PNPT1 | | | | | |
| 2 | 56093096 | 56151298 | EFEMP1 | | | | | |
| 2 | 56190540 | 56274461 | MIR217HG | | | | | |
| 2 | 56210101 | 56210211 | MIR217 | | | | | |
| 2 | 56216084 | 56216194 | MIR216A | | | | | |
| 2 | 56227848 | 56227930 | MIR216B | | | | | |
| 2 | 56400668 | 56412905 | LOC100129434 | | | | | |
| 2 | 56411257 | 56613309 | CCDC85A | | | | | |
| 2 | 58134785 | 58387055 | VRK2 | | | | | |
| 2 | 58386377 | 58468515 | FANCL | | | FANCL | | |
| 2 | 58747887 | 59290901 | LINC01122 | | | | | |
| 2 | 59444842 | 59506535 | LOC101927285 | | | | | |
| 2 | 60586350 | 60618510 | MIR4432HG | | | | | |
| 2 | 60614496 | 60614580 | MIR4432 | | | | | |
| 2 | 60678301 | 60780633 | BCL11A | | | BCL11A | | |
| 2 | 60983364 | 61029221 | PAPOLG | | | | | |
| 2 | 61074894 | 61108449 | LINC01185 | | | | | |
| 2 | 61108629 | 61155291 | REL | | | | | |
| 2 | 61167547 | 61245365 | PUS10 | | | | | |
| 2 | 61244811 | 61279125 | PEX13 | | | | | |
| 2 | 61293005 | 61365169 | KIAA1841 | | | | | |
| 2 | 61368726 | 61372110 | LOC339803 | | | | | |
| 2 | 61372202 | 61391964 | C2orf74 | | | | | |
| 2 | 61404554 | 61414686 | AHSA2 | | | | | |
| 2 | 61414589 | 61697849 | USP34 | | | | | |
| 2 | 61644378 | 61644512 | SNORA70B | | | | | |
| 2 | 61705068 | 61765418 | XPO1 | | | | | |
| 2 | 62051982 | 62081278 | FAM161A | | | | | |
| 2 | 62095261 | 62115806 | CCT4 | | | | | |
| 2 | 62132802 | 62363205 | COMMD1 | | | | | |
| 2 | 62423261 | 62451866 | B3GNT2 | | | | | |
| 2 | 62432960 | 62433052 | MIR5192 | | | | | |
| 2 | 62442655 | 62451866 | B3GNT2 | | | | | |
| 2 | 62727355 | 62733604 | TMEM17 | | | | | |
| 2 | 62900985 | 63273621 | EHBP1 | | | | | |
| 2 | 63271099 | 63275656 | LOC100132215 | | | | | |
| 2 | 63277191 | 63284966 | OTX1 | | | | | |
| 2 | 63344985 | 63346677 | DBIL5P2 | | | | | |
| 2 | 63348534 | 63815867 | WDPCP | | | | | |
| 2 | 63815742 | 63834330 | MDH1 | | | | | |
| 2 | 64068097 | 64118696 | UGP2 | | | | | |
| 2 | 64119666 | 64246214 | VPS54 | | | VPS54 | | |
| 2 | 64319785 | 64371605 | PELI1 | | | | | |
| 2 | 64412212 | 64432619 | LINC00309 | | | | | |
| 2 | 64455534 | 64479665 | LOC100507006 | | | | | |
| 2 | 64567880 | 64567982 | MIR4433B | | | | | |
| 2 | 64567892 | 64567973 | MIR4433A | | | | | |
| 2 | 64681326 | 64688517 | LGALSL | | | | | |
| 2 | 64751438 | 64820138 | AFTPH | | | AFTPH | | |

| | | | | | | | | | |
|---|---|---|---|---|---|---|---|---|---|
| 2 | 64752646 | 64752699 | MIR4434 | | | | | | |
| 2 | 64834445 | 64843616 | LOC339807 | | | | | | |
| 2 | 64858754 | 64881046 | SERTAD2 | | | | | | |
| 2 | 65073263 | 65090765 | LOC101927438 | | | | | | |
| 2 | 65128973 | 65159581 | LOC400958 | | | | | | |
| 2 | 65215578 | 65251000 | SLC1A4 | | | | | | |
| 2 | 65283494 | 65314142 | CEP68 | | | | | | |
| 2 | 65313987 | 65357435 | RAB1A | | | RAB1A | | | |
| 2 | 65454828 | 65498390 | ACTR2 | | | | | | |
| 2 | 65537984 | 65659656 | SPRED2 | | | | | | |
| 2 | 66585380 | 66585460 | MIR4778 | | | | | | |
| 2 | 66610437 | 66619578 | LOC729348 | | | | | | |
| 2 | 66650474 | 66667462 | MEIS1 | | | | | | |
| 2 | 66801161 | 66922720 | LOC100507073 | | | | | | |
| 2 | 66924161 | 66930359 | LOC101927577 | | | | | | |
| 2 | 67131567 | 67198598 | LOC101060019 | | | | | | |
| 2 | 67148642 | 67149622 | LINC01628 | | | | | | |
| 2 | 67313577 | 67516376 | LOC102800447 | | | | | | |
| 2 | 67350488 | 67442451 | LOC644838 | | | | | | |
| 2 | 67440539 | 67471706 | LOC101927661 | | | | | | |
| 2 | 67624441 | 67637533 | ETAA1 | | | | | | |
| 2 | 68023185 | 68052694 | LOC101927701 | | | | | | |
| 2 | 68269331 | 68290159 | C1D | | | | | | |
| 2 | 68357280 | 68384692 | WDR92 | | | | | | |
| 2 | 68385004 | 68403094 | PNO1 | | | | | | |
| 2 | 68405988 | 68479651 | PPP3R1 | | | | | | |
| 2 | 68511302 | 68547183 | CNRIP1 | | | | | | |
| 2 | 68592321 | 68624585 | PLEK | | | | | | |
| 2 | 68689504 | 68694449 | FBXO48 | | | | | | |
| 2 | 68694690 | 68807294 | APLF | | | | | | |
| 2 | 68872953 | 68882708 | PROKR1 | | | PROKR1 | | | |
| 2 | 68961912 | 69053957 | ARHGAP25 | | | | | | |
| 2 | 69092612 | 69098649 | BMP10 | | | | | | |
| 2 | 69172363 | 69180102 | GKN2 | | | | | | |
| 2 | 69201704 | 69208112 | GKN1 | | | | | | |
| 2 | 69240275 | 69476459 | ANTXR1 | | | | | | |
| 2 | 69330813 | 69330887 | MIR3126 | | | | | | |
| 2 | 69546900 | 69614386 | GFPT1 | | | | | | |
| 2 | 69623244 | 69664760 | NFU1 | | | | | | |
| 2 | 69685126 | 69870977 | AAK1 | | | | | | |
| 2 | 69747176 | 69747303 | SNORA36C | | | | | | |
| 2 | 69969126 | 70053596 | ANXA4 | | | | | | |
| 2 | 70056817 | 70106727 | GMCL1 | | | | | | |
| 2 | 70121074 | 70132368 | SNRNP27 | | | | | | |
| 2 | 70142172 | 70170076 | MXD1 | | | | | | |
| 2 | 70187223 | 70189397 | ASPRV1 | | | | | | |
| 2 | 70189394 | 70316334 | PCBP1 | | | | | | |
| 2 | 70351167 | 70352448 | LOC100133985 | | | | | | |
| 2 | 70377016 | 70418151 | C2orf42 | | | | | | |
| 2 | 70436575 | 70475779 | TIA1 | | | | | | |
| 2 | 70485230 | 70508317 | PCYOX1 | | | PCYOX1 | | | |
| 2 | 70508493 | 70520903 | SNRPG | | | | | | |
| 2 | 70523107 | 70529220 | FAM136A | | | | | | |
| 2 | 70674411 | 70781147 | TGFA | | TGFA | | | | |
| 2 | 70694516 | 70708772 | TGFA-IT1 | | | | | | |
| 2 | 70889215 | 70995375 | ADD2 | | ADD2 | | | | |
| 2 | 71004441 | 71017775 | FIGLA | | | | | | |
| 2 | 71035775 | 71047732 | CLEC4F | | | | | | |
| 2 | 71057342 | 71062953 | CD207 | | | | | | |
| 2 | 71115000 | 71117114 | LINC01143 | | | | | | |
| 2 | 71127719 | 71160575 | VAX2 | | | | | | |
| 2 | 71162997 | 71175741 | ATP6V1B1 | | | ATP6V1B1 | | | |
| 2 | 71205574 | 71212629 | ANKRD53 | | | | | | |
| 2 | 71213067 | 71222001 | TEX261 | | | | | | |
| 2 | 71251204 | 71257060 | OR7E91P | | | | | | |
| 2 | 71295407 | 71305998 | NAGK | | | | | | |

| | | | | | | | | |
|---|---|---|---|---|---|---|---|---|
| 2 | 71336805 | 71357394 | MCEE | | | | | |
| 2 | 71357443 | 71377232 | MPHOSPH10 | | | | | |
| 2 | 71409867 | 71454233 | PAIP2B | | | | | |
| 2 | 71558884 | 71662191 | ZNF638 | | | ZNF638 | | |
| 2 | 71680752 | 71913893 | DYSF | | | | | |
| 2 | 72356366 | 72374991 | CYP26B1 | | | | | |
| 2 | 72406443 | 73053177 | EXOC6B | | | | | |
| 2 | 73114511 | 73119289 | SPR | | | | | |
| 2 | 73144603 | 73162020 | EMX1 | | | | | |
| 2 | 73169164 | 73298965 | SFXN5 | | | SFXN5 | | |
| 2 | 73300509 | 73340146 | RAB11FIP5 | | | | | |
| 2 | 73429385 | 73438340 | NOTO | | | | | |
| 2 | 73441365 | 73454355 | SMYD5 | | | | | |
| 2 | 73455133 | 73460356 | PRADC1 | | | | | |
| 2 | 73461363 | 73480150 | CCT7 | | | | | |
| 2 | 73481809 | 73496758 | FBXO41 | | FBXO41 | | | |
| 2 | 73518056 | 73520829 | EGR4 | | | | | |
| 2 | 73612885 | 73837046 | ALMS1 | | | | | |
| 2 | 73684229 | 73686604 | ALMS1-IT1 | | | | | |
| 2 | 73867849 | 73869537 | NAT8 | | | | | |
| 2 | 73872045 | 73912694 | ALMS1P1 | | | | | |
| 2 | 73927635 | 73928467 | NAT8B | | | | | |
| 2 | 73956956 | 73964517 | TPRKB | | | | | |
| 2 | 73989324 | 74007284 | DUSP11 | | | | | |
| 2 | 74011315 | 74044274 | C2orf78 | | | | | |
| 2 | 74056042 | 74094295 | STAMBP | | | STAMBP | | |
| 2 | 74120092 | 74146780 | ACTG2 | | | | | |
| 2 | 74153952 | 74208566 | DGUOK | | | | | |
| 2 | 74213530 | 74335302 | TET3 | | | | | |
| 2 | 74362527 | 74379516 | BOLA3 | | | | | |
| 2 | 74379724 | 74406094 | MOB1A | | | | | |
| 2 | 74425689 | 74442424 | MTHFD2 | | | | | |
| 2 | 74443368 | 74542152 | SLC4A5 | | | | | |
| 2 | 74588280 | 74621008 | DCTN1 | | DCTN1 | DCTN1 | | |
| 2 | 74641302 | 74648757 | C2orf81 | | | | | |
| 2 | 74648884 | 74652882 | WDR54 | | | | | |
| 2 | 74652987 | 74669060 | RTKN | | | | | |
| 2 | 74682149 | 74685087 | INO80B | | | | | |
| 2 | 74682149 | 74688018 | INO80B-WBP1 | | | | | |
| 2 | 74685526 | 74688018 | WBP1 | | | | | |
| 2 | 74688183 | 74692537 | MOGS | | | | | |
| 2 | 74699084 | 74699942 | MRPL53 | | | | | |
| 2 | 74699958 | 74710357 | CCDC142 | | | | | |
| 2 | 74710199 | 74721691 | TTC31 | | | | | |
| 2 | 74724643 | 74732192 | LBX2 | | | | | |
| 2 | 74732169 | 74734821 | PCGF1 | | | | | |
| 2 | 74741595 | 74744275 | TLX2 | | | | | |
| 2 | 74745257 | 74753408 | DQX1 | | | | | |
| 2 | 74753774 | 74757024 | AUP1 | | | | | |
| 2 | 74756531 | 74760683 | HTRA2 | | | | | |
| 2 | 74759385 | 74781091 | LOXL3 | | | | | |
| 2 | 74776146 | 74784678 | DOK1 | | | | | |
| 2 | 74785009 | 74875465 | M1AP | | | | | |
| 2 | 74881354 | 74910981 | SEMA4F | | | | | |
| 2 | 75059781 | 75120481 | HK2 | | | | | |
| 2 | 75136257 | 75165545 | LINC01291 | | | | | |
| 2 | 75185774 | 75196859 | POLE4 | | | | | |
| 2 | 75273589 | 75426645 | TACR1 | | TACR1 | TACR1 | TACR1 | |
| 2 | 75317938 | 75318041 | MIR5000 | | | | | |
| 2 | 75719443 | 75796848 | EVA1A | | | | | |
| 2 | 75751193 | 75769832 | LOC101927884 | | | | | |
| 2 | 75873908 | 75889334 | MRPL19 | | | | | |
| 2 | 75889831 | 75938111 | GCFC2 | | | | | |
| 2 | 76974849 | 77749502 | LRRTM4 | | | LRRTM4 | | |
| 2 | 77213090 | 77236914 | LOC101927907 | | | | | |
| 2 | 77743786 | 77749502 | LRRTM4 | | | LRRTM4 | | |

| | | | | | | | | |
|---|---|---|---|---|---|---|---|---|
| 2 | 77970821 | 78517857 | LOC101927967 | | | | | |
| 2 | 78143059 | 78145134 | LOC101927926 | | | | | |
| 2 | 78182032 | 78182152 | SNAR-H | | | | | |
| 2 | 78315855 | 78354932 | LOC101927948 | | | | | |
| 2 | 79252811 | 79255630 | REG3G | | | | | |
| 2 | 79312148 | 79315150 | REG1B | | | | | |
| 2 | 79347583 | 79350545 | REG1A | | | | | |
| 2 | 79362628 | 79365553 | REG1CP | | | | | |
| 2 | 79384131 | 79386880 | REG3A | | | | | |
| 2 | 79720841 | 79727970 | LOC101927987 | | | | | |
| 2 | 79740059 | 80875988 | CTNNA2 | | | | | |
| 2 | 79876419 | 79876485 | MIR4264 | | | | | |
| 2 | 80093620 | 80093709 | MIR8080 | | | | | |
| 2 | 80529002 | 80531487 | LRRTM1 | | | LRRTM1 | | |
| 2 | 80532229 | 80875988 | CTNNA2 | | | | | |
| 2 | 81688482 | 81694070 | LOC100507201 | | | | | |
| 2 | 83083926 | 83084893 | LOC1720 | | | | | |
| 2 | 84517805 | 84519324 | FUNDC2P2 | | | | | |
| 2 | 84650646 | 84686586 | SUCLG1 | | | SUCLG1 | | |
| 2 | 84743578 | 85046713 | DNAH6 | | | | | |
| 2 | 85048790 | 85108369 | TRABD2A | | | | | |
| 2 | 85132762 | 85133799 | TMSB10 | | | TMSB10 | | |
| 2 | 85198230 | 85286595 | KCMF1 | | | | | |
| 2 | 85360582 | 85537511 | TCF7L1 | | | | | |
| 2 | 85545140 | 85555419 | TGOLN2 | | | TGOLN2 | | |
| 2 | 85569077 | 85581821 | RETSAT | | | | | |
| 2 | 85581842 | 85618875 | ELMOD3 | | | | | |
| 2 | 85621870 | 85641197 | CAPG | | | CAPG | | |
| 2 | 85646053 | 85664152 | SH2D6 | | | | | |
| 2 | 85764589 | 85766009 | PARTICL | | | | | |
| 2 | 85766100 | 85772403 | MAT2A | | | | | |
| 2 | 85771842 | 85788632 | GGCX | | | | | |
| 2 | 85804613 | 85809156 | VAMP8 | | | VAMP8 | | |
| 2 | 85811530 | 85820511 | VAMP5 | | | | | |
| 2 | 85822836 | 85824831 | RNF181 | | | | | |
| 2 | 85825669 | 85829822 | TMEM150A | | | | | |
| 2 | 85829964 | 85876407 | USP39 | | | | | |
| 2 | 85832375 | 85839179 | C2orf68 | | | | | |
| 2 | 85839225 | 85876407 | USP39 | | | | | |
| 2 | 85884439 | 85895864 | SFTPB | | | | | |
| 2 | 85921413 | 85925974 | GNLY | | | | | |
| 2 | 85980908 | 86018506 | ATOH8 | | | | | |
| 2 | 86010722 | 86010800 | MIR6071 | | | | | |
| 2 | 86042252 | 86053911 | LOC284950 | | | | | |
| 2 | 86066270 | 86119375 | ST3GAL5 | | ST3GAL5 | ST3GAL5 | | |
| 2 | 86247338 | 86250991 | LOC90784 | | | | | |
| 2 | 86253450 | 86333278 | POLR1A | | | | | |
| 2 | 86333304 | 86369280 | PTCD3 | | | | | |
| 2 | 86362992 | 86363129 | SNORD94 | | | | | |
| 2 | 86371054 | 86422893 | IMMT | | | | | |
| 2 | 86420148 | 86420231 | MIR4779 | | | | | |
| 2 | 86426555 | 86440477 | MRPL35 | | | | | |
| 2 | 86441119 | 86565206 | REEP1 | | REEP1 | | | |
| 2 | 86668270 | 86719839 | KDM3A | | | | | |
| 2 | 86730552 | 86790620 | CHMP3 | | | | | |
| 2 | 86730552 | 86948245 | RNF103-CHMP3 | | | | | |
| 2 | 86830515 | 86851000 | RNF103 | | | | | |
| 2 | 86947413 | 87005164 | RMND5A | | | | | |
| 2 | 87011727 | 87035519 | CD8A | | | | | |
| 2 | 87042459 | 87089047 | CD8B | | | | | |
| 2 | 87088947 | 87122165 | ANAPC1P1 | | | | | |
| 2 | 87140934 | 87239861 | RGPD2 | | | RGPD2 | | |
| 2 | 87144737 | 87241099 | RGPD1 | | | | | |
| 2 | 87237587 | 87248969 | PLGLB1 | | | | | |
| 2 | 87237587 | 87248973 | PLGLB2 | | | | | |
| 2 | 87257797 | 87303536 | LOC285074 | | | | | |

| | | | | | | | | | |
|---|---|---|---|---|---|---|---|---|---|
| 2 | 87421908 | 87421982 | MIR4771 | | | | | | |
| 2 | 87754973 | 87821030 | LINC00152 | | | | | | |
| 2 | 87929273 | 87929352 | MIR4435 | | | | | | |
| 2 | 88047605 | 88058994 | PLGLB2 | | | | | | |
| 2 | 88047609 | 88058994 | PLGLB1 | | | | | | |
| 2 | 88055478 | 88285309 | RGPD1 | | | | | | |
| 2 | 88056717 | 88125286 | RGPD2 | | | RGPD2 | | | |
| 2 | 88326721 | 88355320 | KRCC1 | | | | | | |
| 2 | 88367298 | 88412902 | SMYD1 | | | | | | |
| 2 | 88382037 | 88382118 | MIR4780 | | | | | | |
| 2 | 88422507 | 88427650 | FABP1 | | | | | | |
| 2 | 88469813 | 88486156 | THNSL2 | | | | | | |
| 2 | 88747725 | 88752053 | FOXI3 | | | | | | |
| 2 | 88824168 | 88829103 | TEX37 | | | | | | |
| 2 | 88838237 | 88875128 | LOC101928371 | | | | | | |
| 2 | 88856257 | 88927094 | EIF2AK3 | | | EIF2AK3 | | | |
| 2 | 88991175 | 89050452 | RPIA | | | | | | |
| 2 | 89065418 | 89106109 | ANKRD36BP2 | | | | | | |
| 2 | 89111883 | 89111968 | MIR4436A | | | | | | |
| 2 | 91824708 | 91847975 | LOC654342 | | | | | | |
| 2 | 91963367 | 91970153 | GGT8P | | | | | | |
| 2 | 92129158 | 92130496 | ACTR3BP2 | | | | | | |
| 2 | 95421070 | 95426929 | FAM95A | | | | | | |
| 2 | 95426672 | 95522820 | ANKRD20A8P | | | | | | |
| 2 | 95534429 | 95613087 | LOC442028 | | | | | | |
| 2 | 95537177 | 95542574 | TEKT4 | | | | | | |
| 2 | 95691399 | 95719737 | MAL | | | | | | |
| 2 | 95752951 | 95787754 | MRPS5 | | | | | | |
| 2 | 95810744 | 95825330 | ZNF514 | | | | | | |
| 2 | 95831161 | 95850064 | ZNF2 | | | | | | |
| 2 | 95940200 | 95957055 | PROM2 | | | | | | |
| 2 | 95963071 | 96051825 | KCNIP3 | | | | | | |
| 2 | 96068447 | 96078879 | FAHD2A | | | | | | |
| 2 | 96142714 | 96150479 | TRIM43B | | | | | | |
| 2 | 96257765 | 96265469 | TRIM43 | | | | | | |
| 2 | 96472799 | 96492729 | LINC00342 | | | | | | |
| 2 | 96515295 | 96658068 | ANKRD36C | | | | | | |
| 2 | 96676298 | 96688884 | FAHD2CP | | | | | | |
| 2 | 96687693 | 96700727 | GPAT2 | | | | | | |
| 2 | 96778622 | 96781984 | ADRA2B | | | | | | |
| 2 | 96789588 | 96804175 | ASTL | | | | | | |
| 2 | 96808907 | 96811179 | DUSP2 | | | | | | |
| 2 | 96850602 | 96908362 | STARD7 | | | | | | |
| 2 | 96915945 | 96931751 | TMEM127 | | | | | | |
| 2 | 96931883 | 96939900 | CIAO1 | | | | | | |
| 2 | 96940073 | 96971307 | SNRNP200 | | | | | | |
| 2 | 96991061 | 96994091 | ITPRIPL1 | | | | | | |
| 2 | 97001478 | 97042833 | NCAPH | | | | | | |
| 2 | 97163379 | 97173846 | NEURL3 | | | | | | |
| 2 | 97202455 | 97218375 | ARID5A | | | | | | |
| 2 | 97258891 | 97304116 | KANSL3 | | | | | | |
| 2 | 97308473 | 97370622 | FER1L5 | | | | | | |
| 2 | 97371666 | 97405813 | LMAN2L | | | | | | |
| 2 | 97426638 | 97477628 | CNNM4 | | | | | | |
| 2 | 97464014 | 97464090 | MIR3127 | | | | | | |
| 2 | 97481990 | 97501121 | CNNM3 | | | | | | |
| 2 | 97503650 | 97509758 | ANKRD23 | | | | | | |
| 2 | 97513723 | 97523756 | ANKRD39 | | | | | | |
| 2 | 97525472 | 97535735 | SEMA4C | | | | | | |
| 2 | 97541618 | 97652301 | FAM178B | | | | | | |
| 2 | 97584842 | 97591574 | LOC101927053 | | | | | | |
| 2 | 97749322 | 97760582 | FAHD2B | | | | | | |
| 2 | 97779232 | 97930257 | ANKRD36 | | | | | | |
| 2 | 97944453 | 97957632 | LOC100506076 | | | | | | |
| 2 | 97949671 | 97957533 | LOC100506123 | | | | | | |
| 2 | 97949671 | 98094824 | LOC100506076 | | | | | | |

| | | | | | | | | |
|---|---|---|---|---|---|---|---|---|
| 2 | 98081774 | 98089620 | LOC100506123 | | | | | |
| 2 | 98121260 | 98206428 | ANKRD36B | | | | | |
| 2 | 98262520 | 98264657 | COX5B | | | | | |
| 2 | 98272401 | 98280561 | ACTR1B | | | | | |
| 2 | 98286205 | 98319529 | LINC01125 | | | | | |
| 2 | 98330030 | 98356323 | ZAP70 | | | | | |
| 2 | 98372800 | 98612354 | TMEM131 | | | | | |
| 2 | 98703594 | 98929410 | VWA3B | | | | | |
| 2 | 98962617 | 99015064 | CNGA3 | | | CNGA3 | | |
| 2 | 99061320 | 99207496 | INPP4A | | INPP4A | | | |
| 2 | 99215785 | 99224981 | COA5 | | | | | |
| 2 | 99225041 | 99234977 | UNC50 | | | | | |
| 2 | 99235568 | 99347589 | MGAT4A | | | | | |
| 2 | 99377525 | 99388361 | LOC101927070 | | | | | |
| 2 | 99410308 | 99552684 | KIAA1211L | | | | | |
| 2 | 99613723 | 99771187 | TSGA10 | | | | | |
| 2 | 99758075 | 99767928 | C2orf15 | | | | | |
| 2 | 99771417 | 99779613 | LIPT1 | | | LIPT1 | | |
| 2 | 99785725 | 99797492 | MITD1 | | | | | |
| 2 | 99797541 | 99816020 | MRPL30 | | | | | |
| 2 | 99858710 | 99871570 | LYG2 | | | | | |
| 2 | 99900700 | 99917639 | LYG1 | | | | | |
| 2 | 99935486 | 99952860 | TXNDC9 | | | | | |
| 2 | 99953833 | 100016728 | EIF5B | | | | | |
| 2 | 100016937 | 100106480 | REV1 | | | | | |
| 2 | 100163715 | 100759037 | AFF3 | | | AFF3 | | |
| 2 | 100824715 | 100867946 | LINC01104 | | | | | |
| 2 | 100889752 | 100939195 | LONRF2 | | | | | |
| 2 | 101008321 | 101034130 | CHST10 | CHST10 | CHST10 | | | |
| 2 | 101086943 | 101099742 | NMS | | | | | |
| 2 | 101179417 | 101193201 | PDCL3 | | | | | |
| 2 | 101436612 | 101613287 | NPAS2 | | | | | |
| 2 | 101589109 | 101593623 | LOC101927142 | | | | | |
| 2 | 101618690 | 101636155 | RPL31 | | | | | |
| 2 | 101623689 | 101767846 | TBC1D8 | | | | | |
| 2 | 101869344 | 101886778 | CNOT11 | | | | | |
| 2 | 101889397 | 101889511 | SNORD89 | | | | | |
| 2 | 101892062 | 101925178 | RNF149 | | | | | |
| 2 | 101925911 | 101925996 | MIR5696 | | | | | |
| 2 | 101964815 | 102003965 | CREG2 | | | | | |
| 2 | 102013822 | 102091165 | RFX8 | | | | | |
| 2 | 102314164 | 102511152 | MAP4K4 | | | MAP4K4 | | |
| 2 | 102599926 | 102602007 | LINC01127 | | | | | |
| 2 | 102608305 | 102644884 | IL1R2 | | | | | |
| 2 | 102686835 | 102796334 | IL1R1 | | | | | |
| 2 | 102803432 | 102855811 | IL1RL2 | | | | | |
| 2 | 102927961 | 102962560 | IL1RL1 | | | | | |
| 2 | 102972742 | 103015235 | IL18R1 | | | | | |
| 2 | 103035249 | 103069024 | IL18RAP | | | | | |
| 2 | 103048748 | 103048826 | MIR4772 | | | | | |
| 2 | 103089761 | 103150431 | SLC9A4 | | | | | |
| 2 | 103236147 | 103327809 | SLC9A2 | | | | | |
| 2 | 103333665 | 103353337 | MFSD9 | | | | | |
| 2 | 103378489 | 103434138 | TMEM182 | | | | | |
| 2 | 104995307 | 105024790 | LOC100287010 | | | | | |
| 2 | 105050804 | 105129215 | LINC01102 | | | | | |
| 2 | 105104913 | 105126967 | LINC01103 | | | | | |
| 2 | 105363094 | 105374177 | LINC01114 | | | | | |
| 2 | 105421882 | 105469641 | LINC01158 | | | | | |
| 2 | 105470525 | 105475031 | POU3F3 | | | | | |
| 2 | 105481954 | 105489053 | LINC01159 | | | | | |
| 2 | 105552698 | 105654954 | LOC102724691 | | | | | |
| 2 | 105654482 | 105716418 | MRPS9 | | | | | |
| 2 | 105713509 | 105719402 | UTAT33 | | | | | |
| 2 | 105858199 | 105859924 | GPR45 | | | | | |
| 2 | 105880846 | 105946171 | TGFBRAP1 | | | | | |

| | | | | | | | | |
|---|---|---|---|---|---|---|---|---|
| 2 | 105953815 | 105965271 | C2orf49 | | | | | |
| 2 | 105977282 | 106055230 | FHL2 | | | | | |
| 2 | 106209553 | 106227016 | LOC285000 | | | | | |
| 2 | 106361519 | 106510730 | NCK2 | | | | | |
| 2 | 106682112 | 106694609 | C2orf40 | | | | | |
| 2 | 106709758 | 106810795 | UXS1 | | | | | |
| 2 | 106998569 | 107007851 | PLGLA | | | | | |
| 2 | 107021135 | 107084801 | RGPD3 | | | | | |
| 2 | 107418055 | 107503563 | ST6GAL2 | | ST6GAL2 | | | |
| 2 | 107766120 | 107965981 | MIR548AU | | | | | |
| 2 | 108370567 | 108438585 | GACAT1 | | | | | |
| 2 | 108439519 | 108509000 | RGPD4 | | | | | |
| 2 | 108602969 | 108630443 | SLC5A7 | | | SLC5A7 | | |
| 2 | 108665655 | 108669211 | LINC01593 | | | | | |
| 2 | 108784203 | 108834297 | LINC01594 | | | | | |
| 2 | 108863650 | 108881807 | SULT1C3 | | | | | |
| 2 | 108905094 | 108926371 | SULT1C2 | | | | | |
| 2 | 108938693 | 108970254 | SULT1C2P1 | | | | | |
| 2 | 108994420 | 109004270 | SULT1C4 | | | | | |
| 2 | 109065576 | 109125854 | GCC2 | | | | | |
| 2 | 109150810 | 109303702 | LIMS1 | | | | | |
| 2 | 109335936 | 109402267 | RANBP2 | | | | | |
| 2 | 109403205 | 109493047 | CCDC138 | | | | | |
| 2 | 109510926 | 109605828 | EDAR | | | EDAR | | |
| 2 | 109743783 | 110262213 | SH3RF3 | | | | | |
| 2 | 109757945 | 109758044 | MIR4265 | | | | | |
| 2 | 109930026 | 109930081 | MIR4266 | | | | | |
| 2 | 110300373 | 110371783 | SEPT10 | | | | | |
| 2 | 110371910 | 110376564 | SOWAHC | | | | | |
| 2 | 110550334 | 110590596 | RGPD5 | | | | | |
| 2 | 110550334 | 110615282 | RGPD6 | | | | | |
| 2 | 110551965 | 110615263 | RGPD5 | | | | | |
| 2 | 110656008 | 110664033 | LIMS3 | | | | | |
| 2 | 110656008 | 110664033 | LIMS4 | | | | | |
| 2 | 110656008 | 110683202 | LIMS3 | | | | | |
| 2 | 110656008 | 110683202 | LIMS4 | | | | | |
| 2 | 110656008 | 110726149 | LIMS3-LOC440895 | | | | | |
| 2 | 110705266 | 110726149 | LOC100288570 | | | | | |
| 2 | 110705266 | 110726149 | LOC440895 | | | | | |
| 2 | 110744556 | 110752858 | LINC01123 | | | | | |
| 2 | 110827537 | 110827619 | MIR4267 | | | | | |
| 2 | 110841446 | 110874143 | MALL | | | MALL | | |
| 2 | 110844009 | 110844100 | MIR4436B1 | | | | | |
| 2 | 110844009 | 110844100 | MIR4436B2 | | | | | |
| 2 | 110880913 | 110962639 | NPHP1 | | | NPHP1 | | |
| 2 | 110969105 | 110980517 | LINC00116 | | | | | |
| 2 | 111003214 | 111024135 | LOC100507334 | | | | | |
| 2 | 111042429 | 111042520 | MIR4436B1 | | | | | |
| 2 | 111042429 | 111042520 | MIR4436B2 | | | | | |
| 2 | 111132685 | 111142113 | LINC01106 | | | | | |
| 2 | 111133764 | 111142102 | LINC01123 | | | | | |
| 2 | 111160495 | 111181393 | LOC100288570 | | | | | |
| 2 | 111160495 | 111181393 | LOC440895 | | | | | |
| 2 | 111160495 | 111230652 | LIMS3-LOC440895 | | | | | |
| 2 | 111203459 | 111230652 | LIMS3 | | | | | |
| 2 | 111203459 | 111230652 | LIMS4 | | | | | |
| 2 | 111222627 | 111230652 | LIMS3 | | | | | |
| 2 | 111222627 | 111230652 | LIMS4 | | | | | |
| 2 | 111271378 | 111336309 | RGPD6 | | | | | |
| 2 | 111271397 | 111336309 | RGPD5 | | | | | |
| 2 | 111296064 | 111336309 | RGPD6 | | | | | |
| 2 | 111395274 | 111435684 | BUB1 | | | | | |
| 2 | 111415726 | 111415826 | SNORD132 | | | | | |
| 2 | 111490149 | 111875799 | ACOXL | | | | | |
| 2 | 111855917 | 111873165 | LOC400997 | | | | | |
| 2 | 111878490 | 111926022 | BCL2L11 | | | | | |

| | | | | | | | | | |
|---|---|---|---|---|---|---|---|---|---|
| 2 | 112078588 | 112252692 | MIR4435 | | | | | | |
| 2 | 112525213 | 112641741 | ANAPC1 | | | | | | |
| 2 | 112528637 | 112528711 | MIR4771 | | | | | | |
| 2 | 112656190 | 112786945 | MERTK | | | | | | |
| 2 | 112812799 | 112876895 | TMEM87B | | | | | | |
| 2 | 112895961 | 112945791 | FBLN7 | | | | | | |
| 2 | 112973438 | 113012664 | ZC3H8 | | | | | | |
| 2 | 113033177 | 113097640 | ZC3H6 | | | | | | |
| 2 | 113125945 | 113191222 | RGPD8 | | | | | | |
| 2 | 113125964 | 113191107 | RGPD5 | | | | | | |
| 2 | 113239742 | 113290222 | TTL | | | | | | |
| 2 | 113299491 | 113334729 | POLR1B | | | | | | |
| 2 | 113342013 | 113346617 | CHCHD5 | | | | | | |
| 2 | 113399406 | 113401757 | FLJ42351 | | | | | | |
| 2 | 113403433 | 113421400 | SLC20A1 | | | | | | |
| 2 | 113493926 | 113522254 | CKAP2L | | | | | | |
| 2 | 113531491 | 113542975 | IL1A | | | | | | |
| 2 | 113587336 | 113594356 | IL1B | | | | | | |
| 2 | 113670547 | 113676458 | IL37 | | | | | | |
| 2 | 113735595 | 113743249 | IL36G | | | | | | |
| 2 | 113763037 | 113765621 | IL36A | | | | | | |
| 2 | 113779667 | 113810444 | IL36B | | | | | | |
| 2 | 113816214 | 113822320 | IL36RN | | | | | | |
| 2 | 113825546 | 113833427 | IL1F10 | | | | | | |
| 2 | 113875469 | 113891593 | IL1RN | | | | | | |
| 2 | 113931559 | 113960677 | PSD4 | | | | | | |
| 2 | 113973573 | 114024600 | PAX8 | | | | | | |
| 2 | 114195267 | 114253781 | CBWD2 | | | | | | |
| 2 | 114256660 | 114258727 | FOXD4L1 | | | | | | |
| 2 | 114285144 | 114300250 | PGM5P3 | | | | | | |
| 2 | 114286223 | 114300267 | PGM5P4 | | | | | | |
| 2 | 114334958 | 114336429 | FAM138B | | | | | | |
| 2 | 114340535 | 114340673 | MIR1302 | | | | | | |
| 2 | 114341229 | 114356613 | WASH2P | | | | | | |
| 2 | 114356604 | 114361294 | DDX11L2 | | | | | | |
| 2 | 114368815 | 114384715 | RPL23AP7 | | | | | | |
| 2 | 114384805 | 114399722 | RABL2A | | | | | | |
| 2 | 114470368 | 114514400 | SLC35F5 | | | | | | |
| 2 | 114478866 | 114478945 | MIR4782 | | | | | | |
| 2 | 114588759 | 114648569 | LOC101060091 | | | | | | |
| 2 | 114647510 | 114719129 | ACTR3 | | | | | | |
| 2 | 114735245 | 114737561 | LOC100499194 | | | | | | |
| 2 | 114737145 | 114764887 | LINC01191 | | | | | | |
| 2 | 115199898 | 116602326 | DPP10 | | | | | | |
| 2 | 118572254 | 118589953 | DDX18 | | | | | | |
| 2 | 118673053 | 118771739 | CCDC93 | | | | | | |
| 2 | 118846049 | 118867597 | INSIG2 | | | | | | |
| 2 | 119357358 | 119360542 | LOC101927709 | | | | | | |
| 2 | 119599746 | 119605759 | EN1 | | | | | | |
| 2 | 119699744 | 119752236 | MARCO | | | | | | |
| 2 | 119913818 | 119916471 | C1QL2 | | | | | | |
| 2 | 119981383 | 120006647 | STEAP3 | | | | | | |
| 2 | 120060019 | 120124258 | C2orf76 | | | | | | |
| 2 | 120124499 | 120130122 | DBI | | | | | | |
| 2 | 120189445 | 120196096 | TMEM37 | | | | | | |
| 2 | 120197418 | 120282028 | SCTR | | | | | | |
| 2 | 120302007 | 120414237 | CFAP221 | | | | | | |
| 2 | 120436742 | 120439694 | TMEM177 | | | | | | |
| 2 | 120517206 | 120742474 | PTPN4 | | | | | | |
| 2 | 120770603 | 120864492 | EPB41L5 | | | | | | |
| 2 | 120975046 | 120980984 | TMEM185B | | | | | | |
| 2 | 121010413 | 121052286 | RALB | | | | | | |
| 2 | 121103718 | 121109383 | INHBB | | | | | | |
| 2 | 121221910 | 121223925 | LINC01101 | | | | | | |
| 2 | 121554866 | 121750229 | GLI2 | | | GLI2 | | | |
| 2 | 121974163 | 122042778 | TFCP2L1 | | | | | | |

| | | | | | | | | |
|---|---|---|---|---|---|---|---|---|
| 2 | 122095351 | 122407052 | CLASP1 | | | | | |
| 2 | 122288455 | 122288585 | RNU4ATAC | | | | | |
| 2 | 122407229 | 122494503 | NIFK | | | | | |
| 2 | 122513120 | 122525428 | TSN | | | | | |
| 2 | 124782863 | 125672954 | CNTNAP5 | | | | | |
| 2 | 127413510 | 127454251 | GYPC | | | | | |
| 2 | 127805598 | 127864903 | BIN1 | | BIN1 | | BIN1 | |
| 2 | 127941411 | 127963343 | CYP27C1 | | | | | |
| 2 | 128014865 | 128051752 | ERCC3 | | | ERCC3 | | |
| 2 | 128056244 | 128100805 | MAP3K2 | | | | | |
| 2 | 128175995 | 128186822 | PROC | | | | | |
| 2 | 128181112 | 128181194 | MIR4783 | | | | | |
| 2 | 128238382 | 128284087 | IWS1 | | | | | |
| 2 | 128293377 | 128395303 | MYO7B | | | | | |
| 2 | 128317619 | 128328537 | LOC105373609 | | | | | |
| 2 | 128395995 | 128439360 | LIMS2 | | | | | |
| 2 | 128403438 | 128410213 | GPR17 | | | | | |
| 2 | 128458596 | 128461407 | SFT2D3 | | | | | |
| 2 | 128461807 | 128568761 | WDR33 | | | | | |
| 2 | 128603839 | 128615729 | POLR2D | | | | | |
| 2 | 128619206 | 128643514 | AMMECR1L | | | | | |
| 2 | 128698790 | 128784869 | SAP130 | | | | | |
| 2 | 128848753 | 128953249 | UGGT1 | | | | | |
| 2 | 129023053 | 129076171 | HS6ST1 | | | | | |
| 2 | 129622173 | 129626301 | LOC101927881 | | | | | |
| 2 | 129999745 | 130031464 | LOC151121 | | | | | |
| 2 | 130680434 | 130691890 | LOC389033 | | | | | |
| 2 | 130680749 | 130704276 | LOC101927924 | | | | | |
| 2 | 130724164 | 130740311 | RAB6C | | | RAB6C | | |
| 2 | 130783571 | 130808704 | FAR2P1 | | | | | |
| 2 | 130831107 | 130886795 | POTEF | | | | | |
| 2 | 130887195 | 130896986 | MED15P9 | | | | | |
| 2 | 130896855 | 130902707 | CCDC74B | | | | | |
| 2 | 130908964 | 130940323 | SMPD4 | | | | | |
| 2 | 130939500 | 130948300 | MZT2B | | | | | |
| 2 | 130949317 | 130956034 | TUBA3E | | | | | |
| 2 | 131095505 | 131100254 | CCDC115 | | | | | |
| 2 | 131100488 | 131104197 | IMP4 | | | | | |
| 2 | 131113579 | 131132982 | PTPN18 | | | | | |
| 2 | 131174325 | 131186119 | FAR2P2 | | | | | |
| 2 | 131193234 | 131199254 | CYP4F62P | | | | | |
| 2 | 131220388 | 131266808 | POTEI | | | | | |
| 2 | 131278666 | 131285565 | CFC1B | | | CFC1B | | |
| 2 | 131294142 | 131307471 | TISP43 | | | | | |
| 2 | 131294142 | 131341734 | LOC646743 | | | | | |
| 2 | 131328418 | 131341734 | TISP43 | | | | | |
| 2 | 131349737 | 131357148 | CFC1 | | | CFC1 | | |
| 2 | 131350352 | 131357251 | CFC1B | | | CFC1B | | |
| 2 | 131369105 | 131415610 | POTEJ | | | | | |
| 2 | 131437622 | 131443435 | CYP4F30P | | | | | |
| 2 | 131486642 | 131487909 | GPR148 | | | GPR148 | | |
| 2 | 131513076 | 131525707 | AMER3 | | | | | |
| 2 | 131674223 | 131804826 | ARHGEF4 | | | | | |
| 2 | 131805448 | 131851004 | FAM168B | | | | | |
| 2 | 131862419 | 131907425 | PLEKHB2 | | | | | |
| 2 | 131975923 | 132022416 | POTEE | | | | | |
| 2 | 132036862 | 132057392 | LOC440910 | | | | | |
| 2 | 132118064 | 132121731 | WTH3DI | | | | | |
| 2 | 132160473 | 132165801 | LINC01120 | | | | | |
| 2 | 132199733 | 132202467 | LOC401010 | | | | | |
| 2 | 132233579 | 132240507 | TUBA3D | | TUBA3D | | | |
| 2 | 132241532 | 132250064 | MZT2A | | | | | |
| 2 | 132248732 | 132248809 | MIR4784 | | | | | |
| 2 | 132250385 | 132279149 | LOC150776 | | | | | |
| 2 | 132285406 | 132291239 | CCDC74A | | | | | |
| 2 | 132354346 | 132366974 | POTEKP | | | | | |

| | | | | | | | | |
|---|---|---|---|---|---|---|---|---|
| 2 | 132394597 | 132407188 | LINC01087 | | | | | |
| 2 | 132480063 | 132524977 | C2orf27A | | | | | |
| 2 | 132552533 | 132559234 | C2orf27B | | | | | |
| 2 | 132905163 | 133015542 | ANKRD30BL | | | | | |
| 2 | 133014538 | 133014653 | MIR663B | | | | | |
| 2 | 133064716 | 133076320 | ZNF806 | | | | | |
| 2 | 133174146 | 133404169 | GPR39 | | | | | |
| 2 | 133402336 | 133429070 | LYPD1 | | | | | |
| 2 | 133429371 | 134326031 | NCKAP5 | | | | | |
| 2 | 133551020 | 134196212 | MIR7853 | | | | | |
| 2 | 134023766 | 134042330 | LOC101928161 | | | | | |
| 2 | 134884695 | 134884763 | MIR3679 | | | | | |
| 2 | 135011829 | 135212192 | MGAT5 | | | | | |
| 2 | 135213329 | 135476571 | TMEM163 | | | | | |
| 2 | 135596185 | 135659602 | ACMSD | | | | | |
| 2 | 135615389 | 135615443 | MIR5590 | | | | | |
| 2 | 135624202 | 135716915 | CCNT2 | | | | | |
| 2 | 135722060 | 135782248 | MAP3K19 | | | | | |
| 2 | 135809834 | 135928279 | RAB3GAP1 | | | RAB3GAP1 | | |
| 2 | 135954538 | 136288806 | ZRANB3 | | | | | |
| 2 | 136289024 | 136482840 | R3HDM1 | | | | | |
| 2 | 136422966 | 136423048 | MIR128 | | | | | |
| 2 | 136499188 | 136542633 | UBXN4 | | | | | |
| 2 | 136545409 | 136594750 | LCT | | | | | |
| 2 | 136577760 | 136580657 | LOC100507600 | | | | | |
| 2 | 136597195 | 136634047 | MCM6 | | | | | |
| 2 | 136663450 | 136765112 | DARS | | | | | |
| 2 | 136871918 | 136875725 | CXCR4 | | | CXCR4 | | |
| 2 | 137523131 | 138435282 | THSD7B | | | | | |
| 2 | 138636323 | 138685502 | LOC101928273 | | | | | |
| 2 | 138721807 | 138773934 | HNMT | | | | | |
| 2 | 139259349 | 139330805 | SPOPL | | | | | |
| 2 | 139426726 | 139537811 | NXPH2 | | | | | |
| 2 | 139654893 | 139656744 | YY1P2 | | | | | |
| 2 | 140988995 | 142889270 | LRP1B | | | | | |
| 2 | 141344194 | 141344254 | MIR7157 | | | | | |
| 2 | 143635194 | 143799885 | KYNU | | | | | |
| 2 | 143886898 | 144525921 | ARHGAP15 | | | | | |
| 2 | 144694633 | 144721722 | LOC101928386 | | | | | |
| 2 | 144703580 | 145090101 | GTDC1 | | | | | |
| 2 | 145141941 | 145278465 | ZEB2 | | ZEB2 | ZEB2 | | |
| 2 | 145279434 | 145337001 | LINC01412 | | | | | |
| 2 | 145425533 | 145834291 | TEX41 | | | | | |
| 2 | 147344624 | 147348558 | PABPC1P2 | | | | | |
| 2 | 148602085 | 148688396 | ACVR2A | | | | | |
| 2 | 148687965 | 148779173 | ORC4 | | | | | |
| 2 | 148778579 | 149271044 | MBD5 | | | | | |
| 2 | 149402559 | 149545136 | EPC2 | | | | | |
| 2 | 149632791 | 149883273 | KIF5C | | KIF5C | | | |
| 2 | 149894980 | 150071772 | LYPD6B | | | | | |
| 2 | 150186498 | 150330659 | LYPD6 | | | | | |
| 2 | 150426146 | 150444330 | MMADHC | | | | | |
| 2 | 150443871 | 150704748 | LOC101929231 | | | | | |
| 2 | 151324706 | 151344209 | RND3 | | | | | |
| 2 | 151409045 | 151428735 | LOC101929260 | | | | | |
| 2 | 151485410 | 151491871 | LOC101929282 | | | | | |
| 2 | 152104727 | 152118389 | RBM43 | | | | | |
| 2 | 152126981 | 152146430 | NMI | | | | | |
| 2 | 152194341 | 152235885 | LOC101929319 | | | | | |
| 2 | 152214105 | 152236562 | TNFAIP6 | | | | | |
| 2 | 152224847 | 152224925 | MIR4773 | | | | | |
| 2 | 152266396 | 152333860 | RIF1 | | | RIF1 | | |
| 2 | 152341852 | 152591001 | NEB | | | | | |
| 2 | 152657479 | 152685009 | ARL5A | | | | | |
| 2 | 152689285 | 152955593 | CACNB4 | | CACNB4 | CACNB4 | CACNB4 | |
| 2 | 152973314 | 153032506 | STAM2 | | | | | |

| | | | | | | | | |
|---|---|---|---|---|---|---|---|---|
| 2 | 153191750 | 153506348 | FMNL2 | | | | | |
| 2 | 153508106 | 153573975 | PRPF40A | | | | | |
| 2 | 153574406 | 153617767 | ARL6IP6 | | | | | |
| 2 | 154333851 | 154335322 | RPRM | | | | | |
| 2 | 154728410 | 155310489 | GALNT13 | | | | | |
| 2 | 155292364 | 155313950 | LOC100144595 | | | | | |
| 2 | 155555092 | 155714864 | KCNJ3 | | | KCNJ3 | | |
| 2 | 156877046 | 157111443 | LOC101929378 | | | | | |
| 2 | 157180943 | 157189287 | NR4A2 | | | | | |
| 2 | 157291964 | 157442915 | GPD2 | | | GPD2 | | |
| 2 | 158114339 | 158167913 | GALNT5 | | | | | |
| 2 | 158175124 | 158184225 | ERMN | | | | | |
| 2 | 158271130 | 158300604 | CYTIP | | | | | |
| 2 | 158383278 | 158485399 | ACVR1C | | | | | |
| 2 | 158592957 | 158732374 | ACVR1 | | | | | |
| 2 | 158851690 | 158992666 | UPP2 | | | UPP2 | | |
| 2 | 159023161 | 159313265 | CCDC148 | | | | | |
| 2 | 159313391 | 159537940 | PKP4 | | | | | |
| 2 | 159651828 | 159672496 | DAPL1 | | | | | |
| 2 | 159825145 | 160089170 | TANC1 | | | | | |
| 2 | 160043345 | 160043412 | MIR6888 | | | | | |
| 2 | 160092303 | 160143310 | WDSUB1 | | | | | |
| 2 | 160175489 | 160473112 | BAZ2B | | | BAZ2B | | |
| 2 | 160471804 | 160474553 | LOC643072 | | | | | |
| 2 | 160549706 | 160568946 | BAZ2B | | | BAZ2B | | |
| 2 | 160568967 | 160625094 | MARCH7 | | | | | |
| 2 | 160625138 | 160654766 | CD302 | | | | | |
| 2 | 160625138 | 160761267 | LY75-CD302 | | | | | |
| 2 | 160659867 | 160761267 | LY75 | | | | | |
| 2 | 160797259 | 160919126 | PLA2R1 | | | | | |
| 2 | 160956176 | 161056824 | ITGB6 | | | | | |
| 2 | 161114229 | 161128406 | LOC100505984 | | | | | |
| 2 | 161128661 | 161350318 | RBMS1 | | | | | |
| 2 | 161264320 | 161264393 | MIR4785 | | | | | |
| 2 | 161993465 | 162092683 | TANK | | | | | |
| 2 | 162079768 | 162111154 | LOC101929512 | | | | | |
| 2 | 162101249 | 162105561 | LOC100996579 | | | | | |
| 2 | 162164785 | 162268228 | PSMD14 | | | | | |
| 2 | 162272604 | 162282378 | TBR1 | | TBR1 | | | |
| 2 | 162355577 | 162364413 | AHCTF1P1 | | | | | |
| 2 | 162480844 | 162841786 | SLC4A10 | | SLC4A10 | SLC4A10 | | |
| 2 | 162848754 | 162931052 | DPP4 | | | | | |
| 2 | 162970950 | 163029243 | LOC101929532 | | | | | |
| 2 | 162999378 | 163008914 | GCG | | | GCG | | |
| 2 | 163027193 | 163100045 | FAP | | | | | |
| 2 | 163123588 | 163175218 | IFIH1 | | | | | |
| 2 | 163200582 | 163219148 | GCA | | | | | |
| 2 | 163227916 | 163695257 | KCNH7 | | | KCNH7 | | |
| 2 | 163625445 | 163654482 | LOC101929570 | | | | | |
| 2 | 164464117 | 164592513 | FIGN | | | | | |
| 2 | 165348915 | 165478360 | GRB14 | | | GRB14 | | |
| 2 | 165536694 | 165698678 | COBLL1 | | | | | |
| 2 | 165544152 | 165544287 | SNORA70F | | | | | |
| 2 | 165697258 | 165705906 | LOC101929633 | | | | | |
| 2 | 165754708 | 165812035 | SLC38A11 | | | | | |
| 2 | 165944029 | 166060577 | SCN3A | | SCN3A | SCN3A | SCN3A | |
| 2 | 166095911 | 166248820 | SCN2A | | SCN2A | SCN2A | SCN2A | |
| 2 | 166326156 | 166545917 | CSRNP3 | | | | | |
| 2 | 166604312 | 166650803 | GALNT3 | | | | | |
| 2 | 166713985 | 166728451 | LOC100506124 | | | | | |
| 2 | 166729871 | 166804831 | TTC21B | | | | | |
| 2 | 166813927 | 166892910 | LOC102724058 | | | | | |
| 2 | 166845669 | 167005642 | SCN1A | | SCN1A | SCN1A | SCN1A | |
| 2 | 166938040 | 167158293 | LOC101929680 | | | | | |
| 2 | 167051696 | 167232497 | SCN9A | | | SCN9A | SCN9A | |
| 2 | 167260082 | 167343481 | SCN7A | | | | | |

| | | | | | | | | |
|---|---|---|---|---|---|---|---|---|
| 2 | 167744996 | 168116261 | XIRP2 | | | | | |
| 2 | 168671283 | 168797654 | LOC105616981 | | | | | |
| 2 | 168675181 | 168727366 | B3GALT1 | | | B3GALT1 | | |
| 2 | 168810529 | 169104105 | STK39 | | | | | |
| 2 | 169312758 | 169631644 | CERS6 | | | | | |
| 2 | 169439452 | 169439528 | MIR4774 | | | | | |
| 2 | 169628460 | 169642939 | CERS6 | | | | | |
| 2 | 169643048 | 169721849 | NOSTRIN | | | | | |
| 2 | 169727400 | 169746944 | SPC25 | | | | | |
| 2 | 169757749 | 169766510 | G6PC2 | | | | | |
| 2 | 169779448 | 169887833 | ABCB11 | | | | | |
| 2 | 169923544 | 169952677 | DHRS9 | | | | | |
| 2 | 169983618 | 170219122 | LRP2 | | LRP2 | LRP2 | | |
| 2 | 170336005 | 170363165 | BBS5 | | BBS5 | | | |
| 2 | 170366211 | 170382772 | KLHL41 | | | | | |
| 2 | 170386261 | 170430431 | FASTKD1 | | | | | |
| 2 | 170440849 | 170494254 | PPIG | | | | | |
| 2 | 170501934 | 170550931 | CCDC173 | | | | | |
| 2 | 170550963 | 170558218 | PHOSPHO2 | | | | | |
| 2 | 170550963 | 170608396 | PHOSPHO2-KLHL23 | | | | | |
| 2 | 170590355 | 170608396 | KLHL23 | | | | | |
| 2 | 170655321 | 170668571 | SSB | | | | | |
| 2 | 170668266 | 170681441 | METTL5 | | | | | |
| 2 | 170684017 | 170940639 | UBR3 | | | | | |
| 2 | 171034654 | 171511674 | MYO3B | | | | | |
| 2 | 171197026 | 171207453 | LOC101929753 | | | | | |
| 2 | 171556877 | 171627276 | LOC101926913 | | | | | |
| 2 | 171568948 | 171571077 | LINC01124 | | | | | |
| 2 | 171571856 | 171574498 | SP5 | | | | | |
| 2 | 171627191 | 171655481 | ERICH2 | | | | | |
| 2 | 171673199 | 171717659 | GAD1 | | GAD1 | GAD1 | | GAD1 |
| 2 | 171784947 | 171823643 | GORASP2 | | | | | |
| 2 | 171847332 | 172087824 | TLK1 | | | | | |
| 2 | 172173912 | 172291312 | METTL8 | | | | | |
| 2 | 172290760 | 172341562 | DCAF17 | | | | | |
| 2 | 172378756 | 172414643 | CYBRD1 | | | | | |
| 2 | 172543918 | 172606668 | DYNC1I2 | | | | | |
| 2 | 172639914 | 172750816 | SLC25A12 | | | SLC25A12 | | |
| 2 | 172778934 | 172848600 | HAT1 | | | | | |
| 2 | 172864803 | 172945587 | METAP1D | | | | | |
| 2 | 172950207 | 172954401 | DLX1 | | DLX1 | DLX1 | | |
| 2 | 172964165 | 172974710 | DLX2 | | | DLX2 | | |
| 2 | 173292081 | 173371183 | ITGA6 | | | | | |
| 2 | 173420100 | 173463862 | PDK1 | | | PDK1 | | |
| 2 | 173587917 | 173917620 | RAPGEF4 | | | RAPGEF4 | | |
| 2 | 173940564 | 174132737 | ZAK | | | | | |
| 2 | 174062440 | 174146764 | MLK7 | | | | | |
| 2 | 174219560 | 174233718 | CDCA7 | | | | | |
| 2 | 174771186 | 174830430 | SP3 | | | | | |
| 2 | 174937174 | 175113365 | OLA1 | | | | | |
| 2 | 175190754 | 175195370 | LINC01305 | | | | | |
| 2 | 175199820 | 175202268 | SP9 | | | | | |
| 2 | 175212877 | 175260443 | CIR1 | | | | | |
| 2 | 175260456 | 175294303 | SCRN3 | | | | | |
| 2 | 175296299 | 175351816 | GPR155 | | | | | |
| 2 | 175422693 | 175422800 | RNU6-2 | | | | | |
| 2 | 175424301 | 175547627 | WIPF1 | | | WIPF1 | | |
| 2 | 175612322 | 175629200 | CHRNA1 | | | | CHRNA1 | CHRNA1 |
| 2 | 175664041 | 175870107 | CHN1 | | CHN1 | CHN1 | | |
| 2 | 175936977 | 176032934 | ATF2 | | | | | |
| 2 | 176032360 | 176032437 | MIR933 | | | | | |
| 2 | 176040985 | 176046490 | ATP5G3 | | | | | |
| 2 | 176788619 | 176867567 | KIAA1715 | | | | | |
| 2 | 176944834 | 176948690 | EVX2 | | | | | |
| 2 | 176957531 | 176960666 | HOXD13 | | | HOXD13 | | |
| 2 | 176964529 | 176965488 | HOXD12 | | | | | |

| | | | | | | | | |
|---|---|---|---|---|---|---|---|---|
| 2 | 176972083 | 176974316 | HOXD11 | | | | | |
| 2 | 176981491 | 176984670 | HOXD10 | | | | | |
| 2 | 176987412 | 176989645 | HOXD9 | | | | | |
| 2 | 176994421 | 176997423 | HOXD8 | | | | | |
| 2 | 176999568 | 177001826 | HOXD | | | | | |
| 2 | 177015030 | 177015140 | MIR10B | | | | | |
| 2 | 177016112 | 177017949 | HOXD4 | | | | | |
| 2 | 177028804 | 177037826 | HOXD3 | | | HOXD3 | | |
| 2 | 177037916 | 177053686 | HAGLR | | | | | |
| 2 | 177042893 | 177043737 | HAGLROS | | | | | |
| 2 | 177053306 | 177055635 | HOXD1 | | | HOXD1 | | |
| 2 | 177053570 | 177053629 | MIR7704 | | | | | |
| 2 | 177134122 | 177202753 | MTX2 | | | | | |
| 2 | 177465707 | 177465780 | MIR1246 | | | | | |
| 2 | 177494308 | 177502302 | LINC01116 | | | | | |
| 2 | 177502481 | 177520686 | LINC01117 | | | | | |
| 2 | 178077421 | 178088685 | HNRNPA3 | | | | | |
| 2 | 178077453 | 178077526 | MIR4444 | | | | | |
| 2 | 178095030 | 178129859 | NFE2L2 | | | | | |
| 2 | 178120672 | 178120738 | MIR3128 | | | | | |
| 2 | 178148235 | 178257419 | LOC100130691 | | | | | |
| 2 | 178178533 | 178178610 | MIR6512 | | | | | |
| 2 | 178257470 | 178408564 | AGPS | | | | | |
| 2 | 178414880 | 178417524 | TTC30B | | | | | |
| 2 | 178479025 | 178483694 | TTC30A | | | | | |
| 2 | 178487976 | 178787564 | PDE11A | | PDE11A | | | |
| 2 | 178977150 | 178994382 | RBM45 | | | | | |
| 2 | 179059207 | 179264160 | OSBPL6 | | | | | |
| 2 | 179246804 | 179541009 | MIR548N | | | | | |
| 2 | 179278385 | 179304970 | LOC101927027 | | | | | |
| 2 | 179296140 | 179315967 | PRKRA | | | | | |
| 2 | 179316162 | 179326110 | DFNB59 | | | | | |
| 2 | 179328390 | 179343355 | FKBP7 | | | | | |
| 2 | 179345198 | 179369782 | PLEKHA3 | | | | | |
| 2 | 179387553 | 179672150 | TTN | | | | | |
| 2 | 179641652 | 179644690 | LOC101927055 | | | | | |
| 2 | 179694483 | 179914786 | CCDC141 | | | | | |
| 2 | 179966418 | 180129350 | SESTD1 | | | | | |
| 2 | 180306710 | 180726232 | ZNF385B | | | | | |
| 2 | 180725562 | 180725635 | MIR1258 | | | | | |
| 2 | 180809603 | 180871780 | CWC22 | | | | | |
| 2 | 181556830 | 181781666 | SCHLAP1 | | | | | |
| 2 | 181845111 | 181928154 | UBE2E3 | | | | | |
| 2 | 181988563 | 182264280 | LOC101927156 | | | | | |
| 2 | 182170319 | 182170379 | MIR4437 | | | | | |
| 2 | 182321618 | 182403655 | ITGA4 | | | ITGA4 | | |
| 2 | 182401400 | 182521834 | CERKL | | | | | |
| 2 | 182540832 | 182545392 | NEUROD1 | | | | | |
| 2 | 182756442 | 182795464 | SSFA2 | | | | | |
| 2 | 182818967 | 182996109 | PPP1R1C | | | | | |
| 2 | 183004761 | 183387572 | PDE1A | | | | | |
| 2 | 183580767 | 183644750 | DNAJC10 | | | | | |
| 2 | 183698004 | 183731498 | FRZB | | | | | |
| 2 | 183789578 | 183903586 | NCKAP1 | | | | | |
| 2 | 183943286 | 183964722 | DUSP19 | | | | | |
| 2 | 183982217 | 184026412 | NUP35 | | | | | |
| 2 | 185243701 | 185243771 | MIR548AE1 | | | | | |
| 2 | 185463092 | 185804214 | ZNF804A | | ZNF804A | | | |
| 2 | 186584600 | 186605204 | LOC101927196 | | | | | |
| 2 | 186603621 | 186698016 | FSIP2 | | | | | |
| 2 | 186898260 | 186951044 | LINC01473 | | | | | |
| 2 | 187350884 | 187374087 | ZC3H15 | | | | | |
| 2 | 187454789 | 187545629 | ITGAV | | | | | |
| 2 | 187558788 | 187628512 | FAM171B | | | | | |
| 2 | 187692206 | 187713897 | ZSWIM2 | | | | | |
| 2 | 188206689 | 188313021 | CALCRL | | | | | |

| | | | | | | | | | |
|---|---|---|---|---|---|---|---|---|---|
| 2 | 188328957 | 188419219 | TFPI | | | | | | |
| 2 | 188900322 | 189152418 | LINC01090 | | | | | | |
| 2 | 189156395 | 189460652 | GULP1 | | | | | | |
| 2 | 189162218 | 189162315 | MIR561 | | | | | | |
| 2 | 189598464 | 189654831 | DIRC1 | | | | | | |
| 2 | 189839098 | 189877472 | COL3A1 | | | COL3A1 | | | |
| 2 | 189842817 | 189842886 | MIR1245A | | | | | | |
| 2 | 189842819 | 189842887 | MIR1245B | | | | | | |
| 2 | 189860355 | 189860418 | MIR3606 | | | | | | |
| 2 | 189896640 | 190044605 | COL5A2 | | | | | | |
| 2 | 189997761 | 189997837 | MIR3129 | | | | | | |
| 2 | 190306158 | 190340288 | WDR75 | | | | | | |
| 2 | 190425315 | 190445537 | SLC40A1 | | | | | | |
| 2 | 190526124 | 190535557 | ASNSD1 | | | | | | |
| 2 | 190540710 | 190611376 | ANKAR | | | | | | |
| 2 | 190611385 | 190630282 | OSGEPL1 | | | | | | |
| 2 | 190634992 | 190649097 | ORMDL1 | | | | | | |
| 2 | 190648810 | 190742355 | PMS1 | | | | | | |
| 2 | 190920425 | 190927455 | MSTN | | | | | | |
| 2 | 191002485 | 191068210 | C2orf88 | | | | | | |
| 2 | 191069359 | 191184771 | HIBCH | | | | | | |
| 2 | 191208195 | 191236391 | INPP1 | | | | | | |
| 2 | 191273080 | 191367041 | MFSD6 | | | | | | |
| 2 | 191371618 | 191399468 | NEMP2 | | | | | | |
| 2 | 191513847 | 191557492 | NAB1 | | | | | | |
| 2 | 191745546 | 191830270 | GLS | | | GLS | | | |
| 2 | 191833761 | 191878976 | STAT1 | | | STAT1 | | | |
| 2 | 191894301 | 192016322 | STAT4 | | | | | | |
| 2 | 192110106 | 192290115 | MYO1B | | | | | | |
| 2 | 192542797 | 192553248 | NABP1 | | | | | | |
| 2 | 192554906 | 192575195 | LOC105747689 | | | | | | |
| 2 | 192699031 | 192712006 | SDPR | | | | | | |
| 2 | 192813771 | 193059659 | TMEFF2 | | | TMEFF2 | | | |
| 2 | 193614570 | 193641625 | PCGEM1 | | | | | | |
| 2 | 195208992 | 195284355 | LOC101927406 | | | | | | |
| 2 | 195595318 | 195626159 | LOC101927431 | | | | | | |
| 2 | 196521531 | 196602426 | SLC39A10 | | | | | | |
| 2 | 196602426 | 196933536 | DNAH7 | | | | | | |
| 2 | 196998306 | 197036336 | STK17B | | | STK17B | | | |
| 2 | 197063974 | 197457335 | HECW2 | | | | | | |
| 2 | 197124747 | 197128928 | LOC101927482 | | | | | | |
| 2 | 197504355 | 197597530 | CCDC150 | | | | | | |
| 2 | 197565358 | 197577736 | LOC100130452 | | | | | | |
| 2 | 197627755 | 197664492 | GTF3C3 | | | | | | |
| 2 | 197669138 | 197675000 | C2orf66 | | | | | | |
| 2 | 197697727 | 197791454 | PGAP1 | | | | | | |
| 2 | 197851385 | 198175521 | ANKRD44 | | | | | | |
| 2 | 198115581 | 198167243 | ANKRD44-IT1 | | | | | | |
| 2 | 198256697 | 198299817 | SF3B1 | | | | | | |
| 2 | 198318230 | 198339851 | COQ10B | | | | | | |
| 2 | 198351307 | 198364998 | HSPD1 | | | HSPD1 | | | |
| 2 | 198351511 | 198351627 | SNORA105A | | | | | | |
| 2 | 198351511 | 198351627 | SNORA105B | | | | | | |
| 2 | 198364720 | 198368187 | HSPE1 | | | | | | |
| 2 | 198364720 | 198418423 | HSPE1-MOB4 | | | | | | |
| 2 | 198380294 | 198418423 | MOB4 | | | | | | |
| 2 | 198435526 | 198540584 | RFTN2 | | | | | | |
| 2 | 198570027 | 198573114 | MARS2 | | | | | | |
| 2 | 198591602 | 198651036 | BOLL | | | | | | |
| 2 | 198669425 | 199014608 | PLCL1 | | | | | | |
| 2 | 199164086 | 199239821 | LOC101927619 | | | | | | |
| 2 | 200134222 | 200337481 | SATB2 | | | SATB2 | | | |
| 2 | 200472790 | 200523855 | LOC101927641 | | | | | | |
| 2 | 200625258 | 200715896 | FTCDNL1 | | | | | | |
| 2 | 200775978 | 200792996 | C2orf69 | | | | | | |
| 2 | 200793633 | 200820459 | TYW5 | | | | | | |

| | | | | | | | | |
|---|---|---|---|---|---|---|---|---|
| 2 | 200820039 | 200828847 | C2orf47 | | | | | |
| 2 | 201170603 | 201346986 | SPATS2L | | | | | |
| 2 | 201353683 | 201374792 | KCTD18 | | | | | |
| 2 | 201390864 | 201448818 | SGOL2 | | | | | |
| 2 | 201450730 | 201536217 | AOX1 | | | | | |
| 2 | 201560445 | 201658941 | AOX2P | | | | | |
| 2 | 201560445 | 201659638 | AOX3P-AOX2P | | | | | |
| 2 | 201577027 | 201599900 | LOC100507140 | | | | | |
| 2 | 201645217 | 201676893 | LOC101927795 | | | | | |
| 2 | 201676268 | 201686966 | BZW1 | | | | | |
| 2 | 201717731 | 201729467 | CLK1 | | | | | |
| 2 | 201735678 | 201753849 | PPIL3 | | | | | |
| 2 | 201754049 | 201768655 | NIF3L1 | | | | | |
| 2 | 201774893 | 201828424 | ORC2 | | | | | |
| 2 | 201838440 | 201936392 | FAM126B | | | | | |
| 2 | 201936461 | 201950473 | NDUFB3 | | | | | |
| 2 | 201980826 | 202022515 | CFLAR | | | | | |
| 2 | 202047603 | 202094129 | CASP10 | | | | | |
| 2 | 202098165 | 202152434 | CASP8 | | | | | |
| 2 | 202152993 | 202222121 | ALS2CR12 | | | | | |
| 2 | 202241929 | 202316319 | TRAK2 | | | TRAK2 | | |
| 2 | 202316391 | 202345574 | STRADB | | | | | |
| 2 | 202352143 | 202483905 | ALS2CR11 | | | | | |
| 2 | 202484906 | 202508252 | TMEM237 | | | | | |
| 2 | 202509596 | 202563417 | MPP4 | | MPP4 | | | |
| 2 | 202564985 | 202645895 | ALS2 | | | ALS2 | | |
| 2 | 202671151 | 202760273 | CDK15 | | | | | |
| 2 | 202899309 | 202903160 | FZD7 | | | | | |
| 2 | 202937977 | 203061886 | KIAA2012 | | | | | |
| 2 | 203070902 | 203103322 | SUMO1 | | | | | |
| 2 | 203130438 | 203168384 | NOP58 | | | | | |
| 2 | 203141153 | 203141241 | SNORD70 | | | | | |
| 2 | 203156039 | 203156151 | SNORD11B | | | | | |
| 2 | 203157773 | 203157857 | SNORD11 | | | | | |
| 2 | 203241049 | 203432474 | BMPR2 | | | BMPR2 | | |
| 2 | 203499900 | 203634480 | FAM117B | | | | | |
| 2 | 203637872 | 203693957 | ICA1L | | | | | |
| 2 | 203745322 | 203776949 | WDR12 | | | | | |
| 2 | 203776940 | 203851208 | CARF | | | | | |
| 2 | 203879601 | 204082717 | NBEAL1 | | | | | |
| 2 | 204103163 | 204170563 | CYP20A1 | | | | | |
| 2 | 204192961 | 204296896 | ABI2 | | | ABI2 | | |
| 2 | 204298404 | 204400058 | RAPH1 | | | | | |
| 2 | 204571197 | 204603636 | CD28 | | | | | |
| 2 | 204732510 | 204738683 | CTLA4 | | | | | |
| 2 | 204801470 | 204826298 | ICOS | | | ICOS | | |
| 2 | 205410515 | 206484886 | PARD3B | | | | | |
| 2 | 206547223 | 206662857 | NRP2 | | | | | |
| 2 | 206858444 | 206950906 | INO80D | | | | | |
| 2 | 206980296 | 206981296 | GCSHP3 | | | | | |
| 2 | 206987802 | 207024243 | NDUFS1 | | | NDUFS1 | | |
| 2 | 207024317 | 207027653 | EEF1B2 | | | | | |
| 2 | 207026604 | 207026674 | SNORD51 | | | | | |
| 2 | 207026951 | 207027083 | SNORA41 | | | | | |
| 2 | 207040041 | 207130967 | GPR1 | | | | | |
| 2 | 207139364 | 207179150 | ZDBF2 | | ZDBF2 | | | |
| 2 | 207308367 | 207485854 | ADAM23 | | ADAM23 | | ADAM23 | |
| 2 | 207507141 | 207514173 | LOC200726 | | | | | |
| 2 | 207516344 | 207583120 | DYTN | | | | | |
| 2 | 207602488 | 207630273 | MDH1B | | | | | |
| 2 | 207630111 | 207660911 | FASTKD2 | | | | | |
| 2 | 207647957 | 207648032 | MIR3130 | | | | | |
| 2 | 207804277 | 207834198 | CPO | | | | | |
| 2 | 207938861 | 208031970 | KLF7 | | | | | |
| 2 | 207974710 | 207974797 | MIR2355 | | | | | |
| 2 | 208031123 | 208031222 | MIR7845 | | | | | |

| | | | | | | | | |
|---|---|---|---|---|---|---|---|---|
| 2 | 208051413 | 208119040 | LOC101927865 | | | | | |
| 2 | 208133998 | 208134148 | MIR1302 | | | | | |
| 2 | 208394615 | 208470284 | CREB1 | CREB1 | | CREB1 | CREB1 | |
| 2 | 208445354 | 208490055 | METTL21A | | | | | |
| 2 | 208576263 | 208620896 | CCNYL1 | | | | | |
| 2 | 208619530 | 208619605 | MIR4775 | | | | | |
| 2 | 208627309 | 208634143 | FZD5 | | | | | |
| 2 | 208686011 | 208890284 | PLEKHM3 | | | | | |
| 2 | 208983852 | 209021486 | LOC100507443 | | | | | |
| 2 | 208986330 | 208989313 | CRYGD | | | | | |
| 2 | 208992860 | 208994554 | CRYGC | | | | | |
| 2 | 209007296 | 209010877 | CRYGB | | | | | |
| 2 | 209025463 | 209028297 | CRYGA | | | | | |
| 2 | 209030070 | 209054773 | C2orf80 | | | | | |
| 2 | 209100950 | 209120918 | IDH1 | | | | | |
| 2 | 209130990 | 209223475 | PIKFYVE | | | | | |
| 2 | 209224568 | 209359231 | PTH2R | | | | | |
| 2 | 210288770 | 210598834 | MAP2 | | | MAP2 | | |
| 2 | 210636716 | 210864024 | UNC80 | | | | | |
| 2 | 210867288 | 210886984 | RPE | | | | | |
| 2 | 210886144 | 211036094 | KANSL1L | | | | | |
| 2 | 210894645 | 210929080 | LOC101928020 | | | | | |
| 2 | 211052715 | 211090215 | ACADL | | | ACADL | | |
| 2 | 211154867 | 211179895 | MYL1 | | | | | |
| 2 | 211189435 | 211341499 | LANCL1 | | | | | |
| 2 | 211342405 | 211543832 | CPS1 | | CPS1 | CPS1 | | |
| 2 | 211482294 | 211484599 | CPS1-IT1 | | | | | |
| 2 | 212240441 | 213403352 | ERBB4 | | ERBB4 | | | |
| 2 | 213290986 | 213291084 | MIR548F2 | | | | | |
| 2 | 213660054 | 213683987 | LOC102725079 | | | | | |
| 2 | 213790980 | 213791060 | MIR4776 | | | | | |
| 2 | 213864410 | 214016333 | IKZF2 | | | | | |
| 2 | 214141276 | 214148929 | LOC100130451 | | | | | |
| 2 | 214149102 | 215275225 | SPAG16 | | | SPAG16 | | |
| 2 | 214622790 | 214622883 | MIR4438 | | | | | |
| 2 | 215276460 | 215440653 | VWC2L | | | | | |
| 2 | 215374905 | 215401613 | VWC2L-IT1 | | | | | |
| 2 | 215590369 | 215674435 | BARD1 | | | | | |
| 2 | 215674952 | 215828329 | LOC101928103 | | | | | |
| 2 | 215796265 | 216003151 | ABCA12 | | | ABCA12 | | |
| 2 | 216176678 | 216214496 | ATIC | | | | | |
| 2 | 216225162 | 216300791 | FN1 | | | FN1 | | |
| 2 | 216318429 | 216328591 | LOC102724849 | | | | | |
| 2 | 216476285 | 216708259 | LINC00607 | | | | | |
| 2 | 216582765 | 216585669 | LINC01614 | | | | | |
| 2 | 216807313 | 216878346 | MREG | | | | | |
| 2 | 216903110 | 216946539 | PECR | | | | | |
| 2 | 216946588 | 216967506 | TMEM169 | | | | | |
| 2 | 216974019 | 217071016 | XRCC5 | | | | | |
| 2 | 217081611 | 217084915 | PKI55 | | | | | |
| 2 | 217122584 | 217236750 | MARCH4 | | | | | |
| 2 | 217277136 | 217347774 | SMARCAL1 | | | | | |
| 2 | 217363519 | 217366188 | RPL37A | | | | | |
| 2 | 217454715 | 217471661 | LINC01280 | | | | | |
| 2 | 217497550 | 217529158 | IGFBP2 | | | | | |
| 2 | 217536827 | 217560272 | IGFBP5 | | | | | |
| 2 | 217724181 | 217724782 | TNP1 | | | | | |
| 2 | 217735494 | 217736362 | LOC101928327 | | | | | |
| 2 | 218147455 | 218621316 | DIRC3 | | | | | |
| 2 | 218664511 | 218808796 | TNS1 | | | | | |
| 2 | 218765235 | 218765351 | MIR6809 | | | | | |
| 2 | 218899656 | 218955304 | RUFY4 | | | | | |
| 2 | 218923877 | 218926013 | CXCR2P1 | | | | | |
| 2 | 218933737 | 218955304 | RUFY4 | | | | | |
| 2 | 218990012 | 219001976 | CXCR2 | | | | | |
| 2 | 219027567 | 219031716 | CXCR1 | | | | | |

| | | | | | | | | |
|---|---|---|---|---|---|---|---|---|
| 2 | 219081816 | 219119071 | ARPC2 | | | | | |
| 2 | 219125737 | 219128582 | GPBAR1 | | | | | |
| 2 | 219128851 | 219134932 | AAMP | | | | | |
| 2 | 219135114 | 219211516 | PNKD | | | | | |
| 2 | 219138916 | 219157280 | TMBIM1 | | | | | |
| 2 | 219144847 | 219144911 | MIR6513 | | | | | |
| 2 | 219187901 | 219211516 | PNKD | | | | | |
| 2 | 219190963 | 219222689 | CATIP | | | | | |
| 2 | 219206633 | 219206703 | MIR6810 | | | | | |
| 2 | 219221578 | 219232625 | CATIP | | | | | |
| 2 | 219246751 | 219261617 | SLC11A1 | | | | | |
| 2 | 219263060 | 219270664 | CTDSP1 | | | | | |
| 2 | 219267368 | 219267445 | MIR26B | | | | | |
| 2 | 219283837 | 219314248 | VIL1 | | | | | |
| 2 | 219314973 | 219433084 | USP37 | | | | | |
| 2 | 219433302 | 219461158 | RQCD1 | | | | | |
| 2 | 219472487 | 219501909 | PLCD4 | | | | | |
| 2 | 219502639 | 219524355 | ZNF142 | | | ZNF142 | | |
| 2 | 219524378 | 219528166 | BCS1L | | | BCS1L | | |
| 2 | 219528586 | 219536781 | RNF25 | | | | | |
| 2 | 219536748 | 219567440 | STK36 | | | | | |
| 2 | 219575567 | 219620138 | TTLL4 | | | | | |
| 2 | 219646471 | 219680016 | CYP27A1 | | | | | |
| 2 | 219687105 | 219696512 | PRKAG3 | | | | | |
| 2 | 219687812 | 219687877 | MIR9500 | | | | | |
| 2 | 219724545 | 219738954 | WNT6 | | | | | |
| 2 | 219745254 | 219758651 | WNT10A | | | | | |
| 2 | 219765536 | 219795359 | LINC01494 | | | | | |
| 2 | 219824349 | 219826877 | CDK5R2 | | | | | |
| 2 | 219841005 | 219842644 | LINC00608 | | | | | |
| 2 | 219845808 | 219850379 | FEV | | | | | |
| 2 | 219854911 | 219858127 | CRYBA2 | | | | | |
| 2 | 219866366 | 219866430 | MIR375 | | | | | |
| 2 | 219866936 | 219880444 | LOC100129175 | | | | | |
| 2 | 219867567 | 219906273 | CFAP65 | | | | | |
| 2 | 219919141 | 219925238 | IHH | | | | | |
| 2 | 219923409 | 219923472 | MIR3131 | | | | | |
| 2 | 219940045 | 220025587 | NHEJ1 | | | | | |
| 2 | 220026180 | 220034817 | SLC23A3 | | | | | |
| 2 | 220036618 | 220041702 | CNPPD1 | | | | | |
| 2 | 220042938 | 220050197 | FAM134A | | | | | |
| 2 | 220071505 | 220074373 | ZFAND2B | | | | | |
| 2 | 220074487 | 220083712 | ABCB6 | | | ABCB6 | | |
| 2 | 220084101 | 220094410 | ATG9A | | | | | |
| 2 | 220094478 | 220101391 | ANKZF1 | | | | | |
| 2 | 220101319 | 220110151 | GLB1L | | | | | |
| 2 | 220110191 | 220115059 | STK16 | | | | | |
| 2 | 220114432 | 220119330 | TUBA4A | | TUBA4A | | | |
| 2 | 220117964 | 220136910 | TUBA4B | | | | | |
| 2 | 220144039 | 220151622 | DNAJB2 | | DNAJB2 | | | |
| 2 | 220154344 | 220174295 | PTPRN | | PTPRN | | | |
| 2 | 220158832 | 220158922 | MIR153 | | | | | |
| 2 | 220192130 | 220197899 | RESP18 | | | | | |
| 2 | 220236750 | 220253598 | DNPEP | | | | | |
| 2 | 220283098 | 220291461 | DES | | | DES | | DES |
| 2 | 220299699 | 220331584 | SPEG | | | | | |
| 2 | 220361076 | 220363009 | LOC100996693 | | | | | |
| 2 | 220363586 | 220371718 | GMPPA | | | | | |
| 2 | 220378891 | 220403494 | ASIC4 | | | | | |
| 2 | 220403668 | 220408487 | CHPF | | | | | |
| 2 | 220408384 | 220415317 | TMEM198 | | | | | |
| 2 | 220413794 | 220413869 | MIR3132 | | | | | |
| 2 | 220415449 | 220436268 | OBSL1 | | OBSL1 | | | |
| 2 | 220436953 | 220440435 | INHA | | | | | |
| 2 | 220462572 | 220481173 | STK11IP | | | | | |
| 2 | 220492291 | 220506702 | SLC4A3 | | SLC4A3 | | | |

| | | | | | | | | | |
|---|---|---|---|---|---|---|---|---|---|
| 2 | 220771222 | 220771286 | MIR4268 | | | | | | |
| 2 | 222282746 | 222438922 | EPHA4 | | | | | | |
| 2 | 223064605 | 223163715 | PAX3 | | | PAX3 | | | |
| 2 | 223162865 | 223169936 | CCDC140 | | | | | | |
| 2 | 223289321 | 223423617 | SGPP2 | | | | | | |
| 2 | 223436161 | 223521074 | FARSB | | | | | | |
| 2 | 223536456 | 223574649 | MOGAT1 | | | | | | |
| 2 | 223725731 | 223808119 | ACSL3 | | | | | | |
| 2 | 223916647 | 223920357 | KCNE4 | | | | | | |
| 2 | 224461657 | 224467217 | SCG2 | | SCG2 | | | | |
| 2 | 224620046 | 224702319 | AP1S3 | | | | | | |
| 2 | 224740059 | 224810104 | WDFY1 | | | WDFY1 | | | |
| 2 | 224822120 | 224832431 | MRPL44 | | | | | | |
| 2 | 224839764 | 224904036 | SERPINE2 | | SERPINE2 | | | | |
| 2 | 225243414 | 225266711 | FAM124B | | | | | | |
| 2 | 225334866 | 225450114 | CUL3 | | | | | | |
| 2 | 225629806 | 225907330 | DOCK10 | | DOCK10 | | | | |
| 2 | 225875177 | 225875257 | MIR4439 | | | | | | |
| 2 | 226265601 | 226518734 | NYAP2 | | | | | | |
| 2 | 227007509 | 227044778 | LOC646736 | | | | | | |
| 2 | 227523425 | 227523509 | MIR5702 | | | | | | |
| 2 | 227596032 | 227663506 | IRS1 | | | IRS1 | | | |
| 2 | 227700670 | 227863923 | RHBDD1 | | | | | | |
| 2 | 227867426 | 228029275 | COL4A4 | | | | | | |
| 2 | 228029280 | 228179508 | COL4A3 | | | | | | |
| 2 | 228085767 | 228189880 | LOC654841 | | | | | | |
| 2 | 228189866 | 228222552 | MFF | | | | | | |
| 2 | 228226873 | 228244022 | TM4SF20 | | | TM4SF20 | | | |
| 2 | 228336847 | 228336903 | MIR5703 | | | | | | |
| 2 | 228336887 | 228425938 | AGFG1 | | | | | | |
| 2 | 228474805 | 228498036 | C2orf83 | | | | | | |
| 2 | 228549925 | 228582745 | SLC19A3 | | | | | | |
| 2 | 228678557 | 228682280 | CCL20 | | | | | | |
| 2 | 228736326 | 228789026 | DAW1 | | | | | | |
| 2 | 228844669 | 229046361 | SPHKAP | | | | | | |
| 2 | 229888688 | 230136057 | PID1 | | | | | | |
| 2 | 230222344 | 230579286 | DNER | | | | | | |
| 2 | 230628552 | 230786725 | TRIP12 | | | | | | |
| 2 | 230787206 | 230877825 | FBXO36 | | | | | | |
| 2 | 230899689 | 230933715 | SLC16A14 | | | | | | |
| 2 | 231033644 | 231090444 | SP110 | | | | | | |
| 2 | 231090444 | 231177930 | SP140 | | | SP140 | | | |
| 2 | 231191893 | 231268445 | SP140L | | | | | | |
| 2 | 231280870 | 231335250 | SP100 | | | | | | |
| 2 | 231555635 | 231565244 | LOC151475 | | | | | | |
| 2 | 231577556 | 231685790 | CAB39 | | | | | | |
| 2 | 231729312 | 231743969 | ITM2C | | | | | | |
| 2 | 231751260 | 231769232 | LOC151484 | | | | | | |
| 2 | 231772042 | 231789941 | GPR55 | | | | | | |
| 2 | 231849082 | 231871999 | SPATA3 | | | | | | |
| 2 | 231902280 | 231914427 | C2orf72 | | | | | | |
| 2 | 231921577 | 232037540 | PSMD1 | | | | | | |
| 2 | 231972949 | 231989824 | HTR2B | | | HTR2B | | | |
| 2 | 232063259 | 232240590 | ARMC9 | | | | | | |
| 2 | 232227418 | 232227504 | MIR4777 | | | | | | |
| 2 | 232260334 | 232265875 | B3GNT7 | | | | | | |
| 2 | 232319458 | 232329205 | NCL | | | | | | |
| 2 | 232320510 | 232320647 | SNORA75 | | | | | | |
| 2 | 232321154 | 232321234 | SNORD20 | | | | | | |
| 2 | 232325078 | 232325153 | SNORD82 | | | | | | |
| 2 | 232373136 | 232379050 | LINC00471 | | | | | | |
| 2 | 232387870 | 232395182 | NMUR1 | | | | | | |
| 2 | 232457574 | 232458994 | C2orf57 | | | | | | |
| 2 | 232573234 | 232578250 | PTMA | | | | | | |
| 2 | 232578023 | 232578105 | MIR1244 | | | | | | |
| 2 | 232597134 | 232646037 | PDE6D | | | | | | |

| | | | | | | | | |
|---|---|---|---|---|---|---|---|---|
| 2 | 232646380 | 232673963 | COPS7B | | | | | |
| 2 | 232756951 | 232757008 | MIR1471 | | | | | |
| 2 | 232786803 | 232791113 | NPPC | | | | | |
| 2 | 232826292 | 233208678 | DIS3L2 | | | | | |
| 2 | 233243243 | 233247599 | ALPP | | | | | |
| 2 | 233250459 | 233251754 | ECEL1P2 | | | | | |
| 2 | 233271551 | 233275424 | ALPPL2 | | | | | |
| 2 | 233320821 | 233325455 | ALPI | | | | | |
| 2 | 233344536 | 233352569 | ECEL1 | | ECEL1 | ECEL1 | ECEL1 | |
| 2 | 233385172 | 233390425 | PRSS56 | | | | | |
| 2 | 233390869 | 233401375 | CHRND | | | CHRND | CHRND | |
| 2 | 233404436 | 233411038 | CHRNG | | | CHRNG | CHRNG | CHRNG |
| 2 | 233412679 | 233415300 | TIGD1 | | | | | |
| 2 | 233415183 | 233415283 | MIR5001 | | | | | |
| 2 | 233415296 | 233448355 | EIF4E2 | | | | | |
| 2 | 233470766 | 233547491 | EFHD1 | | | | | |
| 2 | 233562014 | 233725287 | GIGYF2 | | GIGYF2 | | | |
| 2 | 233630511 | 233641275 | KCNJ13 | | | | | |
| 2 | 233734993 | 233741107 | C2orf82 | | | | | |
| 2 | 233743395 | 233877951 | NGEF | | | | | |
| 2 | 233897381 | 233899767 | NEU2 | | | | | |
| 2 | 233924676 | 234116549 | INPP5D | | | | | |
| 2 | 234160216 | 234204320 | ATG16L1 | | | | | |
| 2 | 234184371 | 234184649 | SCARNA5 | | | | | |
| 2 | 234197321 | 234197587 | SCARNA6 | | | | | |
| 2 | 234216308 | 234255701 | SAG | | | | | |
| 2 | 234263152 | 234380743 | DGKD | | | | | |
| 2 | 234384164 | 234474236 | USP40 | | | | | |
| 2 | 234526290 | 234681945 | UGT1A8 | | | | | |
| 2 | 234545122 | 234681951 | UGT1A10 | | | | | |
| 2 | 234580543 | 234681951 | UGT1A9 | | | | | |
| 2 | 234590583 | 234681945 | UGT1A7 | | | | | |
| 2 | 234600320 | 234681951 | UGT1A6 | | | | | |
| 2 | 234621637 | 234681945 | UGT1A5 | | | | | |
| 2 | 234627437 | 234681945 | UGT1A4 | | | | | |
| 2 | 234637772 | 234681945 | UGT1A3 | | | | | |
| 2 | 234651395 | 234652661 | DNAJB3 | | | | | |
| 2 | 234662961 | 234663991 | LOC100286922 | | | | | |
| 2 | 234668918 | 234681945 | UGT1A1 | | | UGT1A1 | | |
| 2 | 234684324 | 234742069 | MROH2A | | | | | |
| 2 | 234745346 | 234763212 | HJURP | | | | | |
| 2 | 234774089 | 234777055 | MSL3P1 | | | | | |
| 2 | 234826042 | 234928166 | TRPM8 | | | | | |
| 2 | 234959345 | 234985776 | SPP2 | | | | | |
| 2 | 235401685 | 235405697 | ARL4C | | | | | |
| 2 | 235591311 | 235626408 | LINC01173 | | | | | |
| 2 | 235860627 | 235964358 | SH3BP4 | | | | | |
| 2 | 236402732 | 237040444 | AGAP1 | | AGAP1 | | | |
| 2 | 236414394 | 236416210 | AGAP1-IT1 | | | | | |
| 2 | 237073878 | 237076652 | GBX2 | | | | | |
| 2 | 237103514 | 237172988 | ASB18 | | | | | |
| 2 | 237232789 | 237416178 | IQCA1 | | | | | |
| 2 | 237478379 | 237490994 | ACKR3 | | | | | |
| 2 | 237994083 | 238007489 | COPS8 | | | COPS8 | | |
| 2 | 238232654 | 238322850 | COL6A3 | | | | | |
| 2 | 238395052 | 238463961 | MLPH | | | MLPH | | |
| 2 | 238419573 | 238419631 | MIR6811 | | | | | |
| 2 | 238475216 | 238475818 | PRLH | | | | | |
| 2 | 238482964 | 238499769 | RAB17 | | | RAB17 | | |
| 2 | 238536223 | 238690290 | LRRFIP1 | | | | | |
| 2 | 238707387 | 238751451 | RBM44 | | | | | |
| 2 | 238768186 | 238820759 | RAMP1 | | | | | |
| 2 | 238875586 | 238951423 | UBE2F | | | | | |
| 2 | 238875586 | 239008054 | UBE2F-SCLY | | | | | |
| 2 | 238877434 | 238951423 | UBE2F | | | | | |
| 2 | 238969564 | 239008054 | SCLY | | | | | |

| | | | | | | | | |
|---|---|---|---|---|---|---|---|---|
| 2 | 239008950 | 239041928 | ESPNL | | | | | |
| 2 | 239047362 | 239061547 | KLHL30 | | | | | |
| 2 | 239067648 | 239077532 | FAM132B | | | | | |
| 2 | 239079042 | 239112324 | ILKAP | | | | | |
| 2 | 239133753 | 239140318 | LOC151174 | | | | | |
| 2 | 239140326 | 239142985 | LOC643387 | | | | | |
| 2 | 239146907 | 239148765 | HES6 | | | HES6 | | |
| 2 | 239152678 | 239197207 | PER2 | | | | | |
| 2 | 239229184 | 239309541 | TRAF3IP1 | | | | | |
| 2 | 239335625 | 239360891 | ASB1 | | | | | |
| 2 | 239419330 | 239464140 | LINC01107 | | | | | |
| 2 | 239756672 | 239832244 | TWIST2 | | | | | |
| 2 | 239840997 | 239847965 | FLJ43879 | | | | | |
| 2 | 239969863 | 240322643 | HDAC4 | | | HDAC4 | | |
| 2 | 239990512 | 239990610 | MIR4440 | | | | | |
| 2 | 240007522 | 240007622 | MIR4441 | | | | | |
| 2 | 240115026 | 240117153 | MGC16025 | | | | | |
| 2 | 240227156 | 240227240 | MIR4269 | | | | | |
| 2 | 240273418 | 240273499 | MIR2467 | | | | | |
| 2 | 240684553 | 240722356 | LOC150935 | | | | | |
| 2 | 240882431 | 240882511 | MIR4786 | | | | | |
| 2 | 240896788 | 240964819 | NDUFA10 | | | | | |
| 2 | 240968907 | 240969846 | OR6B2 | | | | | |
| 2 | 240981229 | 240982399 | PRR21 | | | | | |
| 2 | 240984493 | 240985489 | OR6B3 | | | | | |
| 2 | 241065979 | 241075747 | MYEOV2 | | | | | |
| 2 | 241078445 | 241080073 | OTOS | | | | | |
| 2 | 241375114 | 241407495 | GPC1 | | | | | |
| 2 | 241388835 | 241396117 | PP14571 | | | | | |
| 2 | 241395417 | 241395506 | MIR149 | | | | | |
| 2 | 241418838 | 241500527 | ANKMY1 | | | | | |
| 2 | 241499470 | 241503431 | DUSP28 | | | | | |
| 2 | 241508003 | 241518149 | RNPEPL1 | | | | | |
| 2 | 241522166 | 241538526 | CAPN10 | | | CAPN10 | | |
| 2 | 241544824 | 241570676 | GPR35 | | | | | |
| 2 | 241615834 | 241622317 | AQP12B | | | | | |
| 2 | 241631261 | 241637900 | AQP12A | | | | | |
| 2 | 241653180 | 241759725 | KIF1A | | KIF1A | KIF1A | | |
| 2 | 241808161 | 241818536 | AGXT | | | | | |
| 2 | 241825464 | 241835573 | C2orf54 | | | | | |
| 2 | 241894035 | 241906868 | LOC200772 | | | | | |
| 2 | 241938254 | 242033643 | SNED1 | | | | | |
| 2 | 242026508 | 242041747 | MTERF4 | | | | | |
| 2 | 242045513 | 242088919 | PASK | | | | | |
| 2 | 242088987 | 242123065 | PPP1R7 | | | | | |
| 2 | 242127923 | 242164791 | ANO7 | | | | | |
| 2 | 242166681 | 242255115 | HDLBP | | | | | |
| 2 | 242254601 | 242293441 | SEPT2 | | | | | |
| 2 | 242295663 | 242434257 | FARP2 | | | | | |
| 2 | 242417319 | 242417397 | MIR3133 | | | | | |
| 2 | 242434121 | 242448987 | STK25 | | | | | |
| 2 | 242483800 | 242513553 | BOK | | BOK | | | |
| 2 | 242523819 | 242576725 | THAP4 | | | | | |
| 2 | 242577026 | 242613271 | ATG4B | | | | | |
| 2 | 242615156 | 242626383 | DTYMK | | | | | |
| 2 | 242641455 | 242668896 | ING5 | | | | | |
| 2 | 242673993 | 242708231 | D2HGDH | | | | | |
| 2 | 242716239 | 242743702 | GAL3ST2 | | | | | |
| 2 | 242750159 | 242758739 | NEU4 | | | | | |
| 2 | 242792032 | 242801058 | PDCD1 | | | | | |
| 2 | 242811885 | 242815482 | RTP5 | | | | | |
| 2 | 242823513 | 243020873 | LINC01237 | | | | | |
| 2 | 242912833 | 242919427 | LOC102723927 | | | | | |
| 2 | 243030783 | 243102476 | LOC728323 | | | | | |
| 3 | 65430 | 66175 | LOC102723448 | | | | | |
| 3 | 238278 | 427478 | CHL1 | | | | | |

| | | | | | | | | |
|---|---|---|---|---|---|---|---|---|
| 3 | 633787 | 887698 | LINC01266 | | | | | |
| 3 | 1134341 | 1445292 | CNTN6 | | CNTN6 | CNTN6 | | |
| 3 | 2140549 | 3102829 | CNTN4 | | CNTN4 | CNTN4 | | |
| 3 | 3108007 | 3152058 | IL5RA | | | | | |
| 3 | 3168599 | 3190706 | TRNT1 | | | TRNT1 | | |
| 3 | 3191316 | 3221401 | CRBN | | | CRBN | | |
| 3 | 3841120 | 3889387 | LRRN1 | | | | | |
| 3 | 4344987 | 4358949 | SETMAR | | | | | |
| 3 | 4402828 | 4508966 | SUMF1 | | | SUMF1 | | |
| 3 | 4532000 | 4889524 | ITPR1 | | | ITPR1 | ITPR1 | ITPR1 |
| 3 | 4790877 | 4793274 | EGOT | | | | | |
| 3 | 4938492 | 5026865 | BHLHE40 | | | | | |
| 3 | 5163929 | 5222601 | ARL8B | | | | | |
| 3 | 5229358 | 5261650 | EDEM1 | | | | | |
| 3 | 5291861 | 5291940 | MIR4790 | | | | | |
| 3 | 6674044 | 7576971 | GRM7 | GRM7 | GRM7 | GRM7 | GRM7 | GRM7 |
| 3 | 7994491 | 8057994 | LOC101927394 | | | | | |
| 3 | 8262833 | 8609811 | LMCD1 | | | | | |
| 3 | 8613467 | 8616354 | LINC00312 | | | | | |
| 3 | 8661085 | 8693764 | SSUH2 | | | | | |
| 3 | 8775485 | 8788451 | CAV3 | | | CAV3 | | |
| 3 | 8792094 | 8811300 | OXTR | | | | | |
| 3 | 8918879 | 9005159 | RAD18 | | | | | |
| 3 | 9022275 | 9291369 | SRGAP3 | | | | | |
| 3 | 9234176 | 9236137 | LOC101927416 | | | | | |
| 3 | 9258578 | 9261192 | SRGAP3 | | | | | |
| 3 | 9404716 | 9439178 | THUMPD3 | | | | | |
| 3 | 9439383 | 9519838 | SETD5 | | | | | |
| 3 | 9540044 | 9595486 | LHFPL4 | | | | | |
| 3 | 9691116 | 9744078 | MTMR14 | | | | | |
| 3 | 9745490 | 9771592 | CPNE9 | | | | | |
| 3 | 9773412 | 9789699 | BRPF1 | | | | | |
| 3 | 9791627 | 9808353 | OGG1 | | | | | |
| 3 | 9799028 | 9811668 | CAMK1 | | | CAMK1 | | |
| 3 | 9821647 | 9834695 | TADA3 | | | | | |
| 3 | 9834178 | 9848789 | ARPC4 | | | | | |
| 3 | 9834231 | 9878040 | ARPC4-TTLL3 | | | | | |
| 3 | 9834800 | 9848789 | ARPC4 | | | | | |
| 3 | 9851643 | 9878040 | TTLL3 | | | | | |
| 3 | 9879532 | 9885702 | RPUSD3 | | | | | |
| 3 | 9908393 | 9921938 | CIDEC | | | | | |
| 3 | 9932270 | 9936031 | JAGN1 | | | | | |
| 3 | 9944295 | 9958084 | IL17RE | | | | | |
| 3 | 9958757 | 9975305 | IL17RC | | | | | |
| 3 | 9975523 | 9987097 | CRELD1 | | | | | |
| 3 | 9987225 | 9994099 | PRRT3 | | | | | |
| 3 | 10005635 | 10048687 | EMC3 | | | | | |
| 3 | 10048101 | 10052779 | LOC401052 | | | | | |
| 3 | 10059236 | 10067820 | CIDECP | | | | | |
| 3 | 10068070 | 10143621 | FANCD2 | | | | | |
| 3 | 10123003 | 10149915 | FANCD2OS | | | | | |
| 3 | 10157332 | 10168874 | BRK1 | | | | | |
| 3 | 10183318 | 10195354 | VHL | | | | | |
| 3 | 10206562 | 10285427 | IRAK2 | | | | | |
| 3 | 10290176 | 10322906 | TATDN2 | | | | | |
| 3 | 10322635 | 10335133 | GHRLOS | | | | | |
| 3 | 10326102 | 10327430 | LINC00852 | | | | | |
| 3 | 10327433 | 10334631 | GHRL | | | | | |
| 3 | 10342612 | 10362872 | SEC13 | | | | | |
| 3 | 10365706 | 10547268 | ATP2B2 | | ATP2B2 | ATP2B2 | | |
| 3 | 10371912 | 10371969 | MIR378B | | | | | |
| 3 | 10436172 | 10436246 | MIR885 | | | | | |
| 3 | 10667700 | 10668492 | ATP2B2-IT2 | | | | | |
| 3 | 10801168 | 10805877 | LINC00606 | | | | | |
| 3 | 10857884 | 10982419 | SLC6A11 | | | SLC6A11 | | |
| 3 | 11034419 | 11060910 | SLC6A1 | | | | | |

| | | | | | | | | |
|---|---|---|---|---|---|---|---|---|
| 3 | 11178778 | 11304939 | HRH1 | | | HRH1 | | |
| 3 | 11314009 | 11599139 | ATG7 | | | | | |
| 3 | 11597540 | 11762242 | VGLL4 | | | | | |
| 3 | 11831915 | 11888393 | TAMM41 | | | | | |
| 3 | 12045833 | 12233532 | SYN2 | | SYN2 | | SYN2 | SYN2 |
| 3 | 12194567 | 12200851 | TIMP4 | | | | | |
| 3 | 12329348 | 12475855 | PPARG | | | | | |
| 3 | 12525930 | 12574820 | TSEN2 | | | | | |
| 3 | 12581279 | 12586963 | MKRN2OS | | | | | |
| 3 | 12598512 | 12625210 | MKRN2 | | | | | |
| 3 | 12625099 | 12705700 | RAF1 | | | RAF1 | | |
| 3 | 12775028 | 12810956 | TMEM40 | | | | | |
| 3 | 12838170 | 12876313 | CAND2 | | | | | |
| 3 | 12876443 | 12883081 | RPL32 | | | | | |
| 3 | 12881810 | 12881949 | SNORA7A | | | | | |
| 3 | 12938541 | 13114617 | IQSEC1 | | | | | |
| 3 | 13357729 | 13461819 | NUP210 | | | | | |
| 3 | 13518487 | 13547924 | HDAC11 | | | | | |
| 3 | 13590624 | 13679922 | FBLN2 | | | | | |
| 3 | 13659880 | 13660048 | SNORA93 | | | | | |
| 3 | 13692220 | 13788132 | LINC00620 | | | | | |
| 3 | 13860081 | 13921618 | WNT7A | | | | | |
| 3 | 13974552 | 13978444 | FGD5P1 | | | | | |
| 3 | 13978806 | 14107481 | TPRXL | | | | | |
| 3 | 14153576 | 14166371 | CHCHD4 | | | | | |
| 3 | 14166439 | 14185180 | TMEM43 | | | | | |
| 3 | 14186647 | 14220172 | XPC | | | | | |
| 3 | 14220227 | 14239869 | LSM3 | | | | | |
| 3 | 14389950 | 14394068 | LINC01267 | | | | | |
| 3 | 14444075 | 14530857 | SLC6A6 | | | SLC6A6 | | |
| 3 | 14530618 | 14581850 | GRIP2 | | | | | |
| 3 | 14693252 | 14714166 | CCDC174 | | | | | |
| 3 | 14716605 | 14814543 | C3orf20 | | | | | |
| 3 | 14860468 | 14989948 | FGD5 | | | | | |
| 3 | 14989090 | 15090786 | NR2C2 | | | | | |
| 3 | 15090018 | 15106816 | MRPS25 | | | | | |
| 3 | 15111575 | 15140664 | RBSN | | RBSN | | | |
| 3 | 15206868 | 15247466 | COL6A4P1 | | | | | |
| 3 | 15247732 | 15294423 | CAPN7 | | | | | |
| 3 | 15295690 | 15382901 | SH3BP5 | | | | | |
| 3 | 15451266 | 15469054 | METTL6 | | | | | |
| 3 | 15469063 | 15484120 | EAF1 | | | EAF1 | | |
| 3 | 15491639 | 15563258 | COLQ | | | COLQ | | COLQ |
| 3 | 15537745 | 15537815 | MIR4270 | | | | | |
| 3 | 15602210 | 15643359 | HACL1 | | | | | |
| 3 | 15642858 | 15687328 | BTD | | | BTD | | |
| 3 | 15708743 | 15901053 | ANKRD28 | | | | | |
| 3 | 15915277 | 15915356 | MIR563 | | | | | |
| 3 | 16215828 | 16271672 | GALNT15 | | | | | |
| 3 | 16298567 | 16306496 | DPH3 | | | | | |
| 3 | 16306666 | 16347594 | OXNAD1 | | | | | |
| 3 | 16357351 | 16555222 | RFTN1 | | | | | |
| 3 | 16577824 | 16582880 | LINC00690 | | | | | |
| 3 | 16628300 | 16647006 | DAZL | | | | | |
| 3 | 16926451 | 17132098 | PLCL2 | | | | | |
| 3 | 16974687 | 16974752 | MIR3714 | | | | | |
| 3 | 17198653 | 17782399 | TBC1D5 | | | | | |
| 3 | 18004063 | 18310410 | LOC339862 | | | | | |
| 3 | 18389132 | 18571606 | SATB1 | | | | | |
| 3 | 19190016 | 19577135 | KCNH8 | | | KCNH8 | | |
| 3 | 19356339 | 19356423 | MIR4791 | | | | | |
| 3 | 19920965 | 19975706 | EFHB | | | | | |
| 3 | 19988571 | 20026667 | RAB5A | | | RAB5A | | |
| 3 | 20021452 | 20053765 | PP2D1 | | | | | |
| 3 | 20081523 | 20195896 | KAT2B | | | | | |
| 3 | 20179056 | 20179133 | MIR3135A | | | | | |

| | | | | | | | | |
|---|---|---|---|---|---|---|---|---|
| 3 | 20202084 | 20227607 | SGOL1 | | | | | |
| 3 | 20383959 | 20392420 | LOC101927829 | | | | | |
| 3 | 21447217 | 21448177 | VENTXP7 | | | | | |
| 3 | 21462489 | 22021320 | ZNF385D | | | | | |
| 3 | 23244783 | 23632296 | UBE2E2 | | | | | |
| 3 | 23375625 | 23648882 | MIR548AC | | | | | |
| 3 | 23845514 | 23933131 | UBE2E1 | | | UBE2E1 | | |
| 3 | 23933571 | 23958537 | NKIRAS1 | | | | | |
| 3 | 23958294 | 23962347 | RPL15 | | | RPL15 | | |
| 3 | 23986750 | 24022109 | NR1D2 | | | | | |
| 3 | 24141464 | 24144738 | LINC00691 | | | | | |
| 3 | 24158644 | 24536313 | THRB | | | | | |
| 3 | 24192814 | 24231447 | LOC101927854 | | | | | |
| 3 | 24535577 | 24541502 | THRB | | | | | |
| 3 | 24562852 | 24562926 | MIR4792 | | | | | |
| 3 | 25215822 | 25639422 | RARB | | | | | |
| 3 | 25424595 | 25426418 | LOC105376997 | | | | | |
| 3 | 25469753 | 25639422 | RARB | | | | | |
| 3 | 25639395 | 25705863 | TOP2B | | | | | |
| 3 | 25706363 | 25706430 | MIR4442 | | | | | |
| 3 | 25760434 | 25831530 | NGLY1 | | | | | |
| 3 | 25831562 | 25836025 | OXSM | | | | | |
| 3 | 25900022 | 25915186 | LINC00692 | | | | | |
| 3 | 26664296 | 26752265 | LRRC3B | | | | | |
| 3 | 27152393 | 27410951 | NEK10 | | | | | |
| 3 | 27414211 | 27525911 | SLC4A7 | | | | | |
| 3 | 27757439 | 27764206 | EOMES | | | | | |
| 3 | 27872376 | 27875627 | LOC100996624 | | | | | |
| 3 | 28283123 | 28361263 | CMC1 | | | | | |
| 3 | 28363843 | 28390618 | AZI2 | | | | | |
| 3 | 28390639 | 28566632 | ZCWPW2 | | | | | |
| 3 | 28616768 | 28799828 | LINC00693 | | | | | |
| 3 | 29305684 | 29975647 | RBMS3 | | | | | |
| 3 | 30647993 | 30735633 | TGFBR2 | | | TGFBR2 | | |
| 3 | 30767691 | 30936153 | GADL1 | | | | | |
| 3 | 31203195 | 31203279 | MIR466 | | | | | |
| 3 | 31573992 | 31679114 | STT3B | | | | | |
| 3 | 31702316 | 31763078 | OSBPL10 | | | | | |
| 3 | 32023265 | 32033228 | ZNF860 | | | | | |
| 3 | 32148002 | 32210207 | GPD1L | | | | | |
| 3 | 32280170 | 32411813 | CMTM8 | | | | | |
| 3 | 32433162 | 32496333 | CMTM7 | | | | | |
| 3 | 32522803 | 32544403 | CMTM6 | | | | | |
| 3 | 32567462 | 32612366 | DYNC1LI1 | | | | | |
| 3 | 32726636 | 32815367 | CNOT10 | | | | | |
| 3 | 32859509 | 32939318 | TRIM71 | | | | | |
| 3 | 32993065 | 32996403 | CCR4 | | | CCR4 | | |
| 3 | 33038099 | 33138722 | GLB1 | | | GLB1 | | |
| 3 | 33131907 | 33138293 | TMPPE | | | | | |
| 3 | 33155449 | 33189265 | CRTAP | | | | | |
| 3 | 33191536 | 33260707 | SUSD5 | | | | | |
| 3 | 33318933 | 33428757 | FBXL2 | | | | | |
| 3 | 33429828 | 33481897 | UBP1 | | | | | |
| 3 | 33537738 | 33759705 | CLASP2 | | | CLASP2 | | |
| 3 | 33840062 | 33911199 | PDCD6IP | | | PDCD6IP | | |
| 3 | 34917288 | 35435515 | LOC101928135 | | | | | |
| 3 | 35680665 | 35727379 | ARPP21 | | ARPP21 | | | |
| 3 | 35785967 | 35786051 | MIR128 | | | | | |
| 3 | 36421978 | 36589498 | STAC | | | | | |
| 3 | 36753912 | 36781352 | DCLK3 | | | | | |
| 3 | 36868307 | 36986548 | TRANK1 | | | | | |
| 3 | 37027356 | 37034795 | EPM2AIP1 | | | | | |
| 3 | 37034840 | 37092337 | MLH1 | | | | | |
| 3 | 37094116 | 37217851 | LRRFIP2 | | | | | |
| 3 | 37284681 | 37408370 | GOLGA4 | | | | | |
| 3 | 37427759 | 37459865 | C3orf35 | | | | | |

| | | | | | | | | | |
|---|---|---|---|---|---|---|---|---|---|
| 3 | 37493812 | 37903271 | ITGA9 | | | | | | |
| 3 | 37903668 | 38025960 | CTDSPL | | | | | | |
| 3 | 38010894 | 38010971 | MIR26A1 | | | | | | |
| 3 | 38035077 | 38048676 | VILL | | | VILL | | | |
| 3 | 38048986 | 38071154 | PLCD1 | | | PLCD1 | | | |
| 3 | 38080695 | 38164228 | DLEC1 | | | | | | |
| 3 | 38164200 | 38178733 | ACAA1 | | | | | | |
| 3 | 38179968 | 38184512 | MYD88 | | | | | | |
| 3 | 38207025 | 38296979 | OXSR1 | | | OXSR1 | | | |
| 3 | 38307297 | 38319806 | SLC22A13 | | | | | | |
| 3 | 38323784 | 38360066 | SLC22A14 | | | | | | |
| 3 | 38388250 | 38456467 | XYLB | | | | | | |
| 3 | 38492517 | 38534633 | ACVR2B | | | | | | |
| 3 | 38537762 | 38567796 | EXOG | | | | | | |
| 3 | 38589552 | 38691164 | SCN5A | | | SCN5A | SCN5A | | |
| 3 | 38738836 | 38835501 | SCN10A | | | SCN10A | SCN10A | | |
| 3 | 38887259 | 38995142 | SCN11A | | | | SCN11A | | |
| 3 | 39093476 | 39138161 | WDR48 | | | | | | |
| 3 | 39138089 | 39149854 | GORASP1 | | | | | | |
| 3 | 39149151 | 39180394 | TTC21A | | | | | | |
| 3 | 39179696 | 39179757 | MIR6822 | | | | | | |
| 3 | 39183341 | 39195102 | CSRNP1 | | | | | | |
| 3 | 39224705 | 39234077 | XIRP1 | | | | | | |
| 3 | 39304984 | 39323226 | CX3CR1 | | CX3CR1 | | | | |
| 3 | 39371196 | 39375171 | CCR8 | | | | | | |
| 3 | 39424814 | 39438819 | SLC25A38 | | | | | | |
| 3 | 39448179 | 39454032 | RPSA | | | | | | |
| 3 | 39449881 | 39450030 | SNORA6 | | | | | | |
| 3 | 39452544 | 39452698 | SNORA62 | | | | | | |
| 3 | 39509063 | 39544517 | MOBP | | | | | | |
| 3 | 39850404 | 40301811 | MYRIP | | | MYRIP | | | |
| 3 | 40214637 | 40353915 | EIF1B | | | | | | |
| 3 | 40428646 | 40494799 | ENTPD3 | | ENTPD3 | | | | |
| 3 | 40498782 | 40503863 | RPL14 | | | | | | |
| 3 | 40518603 | 40530069 | ZNF619 | | | | | | |
| 3 | 40547529 | 40559712 | ZNF620 | | | | | | |
| 3 | 40566368 | 40581285 | ZNF621 | | | | | | |
| 3 | 41240941 | 41281939 | CTNNB1 | | | | | CTNNB1 | |
| 3 | 41288089 | 42003660 | ULK4 | | | | | | |
| 3 | 42132745 | 42254118 | TRAK1 | | | | | | |
| 3 | 42299315 | 42307687 | CCK | | | | | | |
| 3 | 42438569 | 42452094 | LYZL4 | | | | | | |
| 3 | 42530790 | 42574098 | VIPR1 | | | | | | |
| 3 | 42589458 | 42642572 | SEC22C | | | | | | |
| 3 | 42632297 | 42636490 | SS18L2 | | | | | | |
| 3 | 42642146 | 42690233 | NKTR | | | | | | |
| 3 | 42653801 | 42695880 | LOC101928323 | | | | | | |
| 3 | 42695175 | 42709072 | ZBTB47 | | | | | | |
| 3 | 42727010 | 42733938 | KLHL40 | | | | | | |
| 3 | 42734154 | 42744319 | HHATL | | | | | | |
| 3 | 42749873 | 42786650 | CCDC13 | | | | | | |
| 3 | 42812103 | 42815126 | LOC729083 | | | | | | |
| 3 | 42824399 | 42846027 | HIGD1A | | | | | | |
| 3 | 42850963 | 42908775 | ACKR2 | | | | | | |
| 3 | 42913683 | 42917633 | CYP8B1 | | | | | | |
| 3 | 42947401 | 42960825 | ZNF662 | | | | | | |
| 3 | 42975735 | 42984283 | KRBOX1 | | | | | | |
| 3 | 43020758 | 43099207 | FAM198A | | | | | | |
| 3 | 43120720 | 43147575 | POMGNT2 | | POMGNT2 | | | | |
| 3 | 43328003 | 43393454 | SNRK | | | | | | |
| 3 | 43407817 | 43663560 | ANO10 | | | | | | |
| 3 | 43732374 | 43764217 | ABHD5 | | | ABHD5 | | | |
| 3 | 44155703 | 44155802 | MIR138 | | | | | | |
| 3 | 44283377 | 44373590 | TOPAZ1 | | | | | | |
| 3 | 44379610 | 44450940 | TCAIM | | | | | | |
| 3 | 44462623 | 44465517 | LOC102724231 | | | | | | |

| | | | | | | | | |
|---|---|---|---|---|---|---|---|---|
| 3 | 44481261 | 44519162 | ZNF445 | | | | | |
| 3 | 44540461 | 44552132 | ZNF852 | | | | | |
| 3 | 44596666 | 44624956 | ZKSCAN7 | | | | | |
| 3 | 44626455 | 44637557 | ZNF660 | | | | | |
| 3 | 44658619 | 44689963 | ZNF197 | | | | | |
| 3 | 44690232 | 44702283 | ZNF35 | | | | | |
| 3 | 44754134 | 44765323 | ZNF502 | | | | | |
| 3 | 44771097 | 44778575 | ZNF501 | | | | | |
| 3 | 44790235 | 44803173 | KIAA1143 | | | | | |
| 3 | 44803208 | 44894748 | KIF15 | | | | | |
| 3 | 44903379 | 44903473 | MIR564 | | | | | |
| 3 | 44903400 | 44907159 | TMEM42 | | | | | |
| 3 | 44916097 | 44956088 | TGM4 | | | | | |
| 3 | 44956752 | 45017674 | ZDHHC3 | | | | | |
| 3 | 45017740 | 45054158 | EXOSC7 | | | | | |
| 3 | 45067758 | 45077565 | CLEC3B | | | | | |
| 3 | 45123765 | 45187914 | CDCP1 | | | | | |
| 3 | 45265955 | 45267814 | TMEM158 | | | | | |
| 3 | 45430074 | 45551037 | LARS2 | | LARS2 | LARS2 | | |
| 3 | 45636322 | 45730374 | LIMD1 | | | | | |
| 3 | 45730732 | 45786917 | SACM1L | | | | | |
| 3 | 45796940 | 45838035 | SLC6A20 | | | SLC6A20 | | |
| 3 | 45864809 | 45957216 | LZTFL1 | | | | | |
| 3 | 45927995 | 45944667 | CCR9 | | | | | |
| 3 | 45959390 | 46037316 | FYCO1 | | | | | |
| 3 | 45984972 | 45989845 | CXCR6 | | | | | |
| 3 | 46062290 | 46068979 | XCR1 | | | | | |
| 3 | 46243199 | 46249832 | CCR1 | | | | | |
| 3 | 46283871 | 46308197 | CCR3 | | | | | |
| 3 | 46395234 | 46402413 | CCR2 | | | | | |
| 3 | 46406150 | 46448550 | LOC102724297 | | | | | |
| 3 | 46411632 | 46417697 | CCR5 | | | CCR5 | | |
| 3 | 46448720 | 46451014 | CCRL2 | | | | | |
| 3 | 46477495 | 46506632 | LTF | | | | | |
| 3 | 46539484 | 46542439 | RTP3 | | | | | |
| 3 | 46556877 | 46601178 | LRRC2 | | | | | |
| 3 | 46616044 | 46623952 | TDGF1 | | TDGF1 | TDGF1 | | |
| 3 | 46653924 | 46668033 | LOC100132146 | | | | | |
| 3 | 46710484 | 46735194 | ALS2CL | | | | | |
| 3 | 46742822 | 46752413 | TMIE | | | | | |
| 3 | 46753605 | 46759373 | PRSS50 | | | | | |
| 3 | 46761072 | 46777921 | PRSS46 | | | | | |
| 3 | 46783580 | 46786245 | PRSS45 | | | | | |
| 3 | 46871893 | 46875585 | PRSS42 | | | | | |
| 3 | 46899356 | 46904973 | MYL3 | | | | | |
| 3 | 46919235 | 46945289 | PTH1R | | PTH1R | | | |
| 3 | 46963219 | 47023500 | CCDC12 | | | | | |
| 3 | 47021172 | 47051194 | NBEAL2 | | | | | |
| 3 | 47053031 | 47054957 | NRADDP | | | | | |
| 3 | 47057897 | 47205467 | SETD2 | | | | | |
| 3 | 47205859 | 47324337 | KIF9 | | | | | |
| 3 | 47324329 | 47388306 | KLHL18 | | | | | |
| 3 | 47422471 | 47454931 | PTPN23 | | | | | |
| 3 | 47455174 | 47517449 | SCAP | | | | | |
| 3 | 47537129 | 47555199 | ELP6 | | | | | |
| 3 | 47603727 | 47621730 | CSPG5 | | CSPG5 | CSPG5 | | |
| 3 | 47627377 | 47823405 | SMARCC1 | | | | | |
| 3 | 47844398 | 47891686 | DHX30 | | | | | |
| 3 | 47891044 | 47891119 | MIR1226 | | | | | |
| 3 | 47892179 | 48130769 | MAP4 | | | MAP4 | | |
| 3 | 48198667 | 48229801 | CDC25A | | | | | |
| 3 | 48238053 | 48238106 | MIR4443 | | | | | |
| 3 | 48264836 | 48266981 | CAMP | | | | | |
| 3 | 48282595 | 48312479 | ZNF589 | | | | | |
| 3 | 48331768 | 48333168 | FCF1P2 | | | | | |
| 3 | 48333806 | 48343175 | NME6 | | | | | |

| | | | | | | | | | |
|---|---|---|---|---|---|---|---|---|---|
| 3 | 48348335 | 48369831 | SPINK8 | | | | | |
| 3 | 48357849 | 48357949 | MIR2115 | | | | | |
| 3 | 48413708 | 48436190 | FBXW12 | | | | | |
| 3 | 48445260 | 48471460 | PLXNB1 | | | PLXNB1 | | |
| 3 | 48473579 | 48481529 | CCDC51 | | | | | |
| 3 | 48481685 | 48485537 | TMA7 | | | | | |
| 3 | 48488113 | 48507054 | ATRIP | | | | | |
| 3 | 48506918 | 48509044 | TREX1 | | | | | |
| 3 | 48509196 | 48542259 | SHISA5 | | | | | |
| 3 | 48555116 | 48598613 | PFKFB4 | | | | | |
| 3 | 48587393 | 48587454 | MIR6823 | | | | | |
| 3 | 48599150 | 48601201 | UCN2 | | | | | |
| 3 | 48601505 | 48632593 | COL7A1 | | | COL7A1 | | |
| 3 | 48616334 | 48616410 | MIR711 | | | | | |
| 3 | 48636431 | 48647098 | UQCRC1 | | | | | |
| 3 | 48642352 | 48642583 | SNORA94 | | | | | |
| 3 | 48658274 | 48659189 | TMEM89 | | | | | |
| 3 | 48663155 | 48672926 | SLC26A6 | | | | | |
| 3 | 48671068 | 48671131 | MIR6824 | | | | | |
| 3 | 48673895 | 48700348 | CELSR3 | | CELSR3 | | | |
| 3 | 48681626 | 48681713 | MIR4793 | | | | | |
| 3 | 48701200 | 48706603 | CELSR3 | | CELSR3 | | | |
| 3 | 48711271 | 48723366 | NCKIPSD | | | NCKIPSD | | |
| 3 | 48725435 | 48754711 | IP6K2 | | | | | |
| 3 | 48788092 | 48889415 | PRKAR2A | | | | | |
| 3 | 48894355 | 48936426 | SLC25A20 | | | SLC25A20 | | |
| 3 | 48955220 | 48956818 | ARIH2OS | | | | | |
| 3 | 48956252 | 49022971 | ARIH2 | | | | | |
| 3 | 49027340 | 49044581 | P4HTM | | | | | |
| 3 | 49044636 | 49053386 | WDR6 | | | | | |
| 3 | 49052920 | 49058504 | DALRD3 | | | | | |
| 3 | 49057580 | 49057667 | MIR425 | | | | | |
| 3 | 49057907 | 49060926 | NDUFAF3 | | | | | |
| 3 | 49058050 | 49058142 | MIR191 | | | | | |
| 3 | 49058617 | 49060926 | NDUFAF3 | | | | | |
| 3 | 49061761 | 49066875 | IMPDH2 | | | | | |
| 3 | 49067141 | 49131504 | QRICH1 | | | | | |
| 3 | 49133364 | 49142562 | QARS | | | | | |
| 3 | 49137286 | 49137347 | MIR6890 | | | | | |
| 3 | 49145478 | 49158371 | USP19 | | | | | |
| 3 | 49158546 | 49170599 | LAMB2 | | | | | |
| 3 | 49190291 | 49191834 | LAMB2P1 | | | | | |
| 3 | 49199967 | 49203785 | CCDC71 | | | | | |
| 3 | 49209017 | 49213919 | KLHDC8B | | | | | |
| 3 | 49215068 | 49229291 | C3orf84 | | | | | |
| 3 | 49235860 | 49295537 | CCDC36 | | | | | |
| 3 | 49306029 | 49314508 | C3orf62 | | | | | |
| 3 | 49311552 | 49311619 | MIR4271 | | | | | |
| 3 | 49314576 | 49377536 | USP4 | | | | | |
| 3 | 49394608 | 49395791 | GPX1 | | | | | |
| 3 | 49396568 | 49449530 | RHOA | | | | | |
| 3 | 49449638 | 49453909 | TCTA | | | | | |
| 3 | 49454210 | 49460111 | AMT | | | AMT | | |
| 3 | 49459765 | 49466757 | NICN1 | | NICN1 | | | |
| 3 | 49506135 | 49573051 | DAG1 | | | | | |
| 3 | 49586738 | 49708982 | BSN | | BSN | BSN | BSN | BSN |
| 3 | 49711434 | 49720934 | APEH | | | | | |
| 3 | 49721379 | 49726196 | MST1 | | | | | |
| 3 | 49726949 | 49758962 | RNF123 | | | | | |
| 3 | 49754266 | 49757238 | AMIGO3 | | | | | |
| 3 | 49758908 | 49761407 | GMPPB | | | | | |
| 3 | 49761727 | 49823973 | IP6K1 | | | | | |
| 3 | 49828166 | 49837268 | CDHR4 | | | | | |
| 3 | 49840686 | 49842463 | FAM212A | | | | | |
| 3 | 49842637 | 49851391 | UBA7 | | | | | |
| 3 | 49843569 | 49843678 | MIR5193 | | | | | |

| | | | | | | | | |
|---|---|---|---|---|---|---|---|---|
| 3 | 49866027 | 49893992 | TRAIP | | | | | |
| 3 | 49895421 | 49907369 | CAMKV | | | | | |
| 3 | 49924435 | 49941070 | MST1R | | | | | |
| 3 | 49946301 | 49967445 | MON1A | | | | | |
| 3 | 49977476 | 50114685 | RBM6 | | | | | |
| 3 | 50126340 | 50138421 | RBM5 | | | | | |
| 3 | 50192477 | 50226508 | SEMA3F | | | | | |
| 3 | 50210758 | 50210852 | MIR566 | | | | | |
| 3 | 50229042 | 50235129 | GNAT1 | | | | | |
| 3 | 50242678 | 50258411 | SLC38A3 | | | | | |
| 3 | 50264119 | 50296786 | GNAI2 | | | | | |
| 3 | 50264867 | 50264922 | MIR5787 | | | | | |
| 3 | 50268207 | 50296786 | GNAI2 | | | | | |
| 3 | 50304072 | 50314602 | SEMA3B | | SEMA3B | | | |
| 3 | 50310666 | 50310728 | MIR6872 | | | | | |
| 3 | 50316457 | 50325545 | LSMEM2 | | | | | |
| 3 | 50325162 | 50330026 | IFRD2 | | | | | |
| 3 | 50330258 | 50336899 | HYAL3 | | | | | |
| 3 | 50333832 | 50336852 | NAT6 | | | | | |
| 3 | 50337319 | 50349812 | HYAL1 | | | | | |
| 3 | 50355220 | 50360281 | HYAL2 | | | HYAL2 | | |
| 3 | 50362339 | 50365669 | TUSC2 | | | | | |
| 3 | 50367216 | 50375727 | RASSF1 | | | | | |
| 3 | 50378536 | 50383177 | ZMYND10 | | | | | |
| 3 | 50384918 | 50388486 | NPRL2 | | | | | |
| 3 | 50388125 | 50405628 | CYB561D2 | | | | | |
| 3 | 50392179 | 50396939 | TMEM115 | | | | | |
| 3 | 50400043 | 50541675 | CACNA2D2 | | CACNA2D2 | | | |
| 3 | 50402764 | 50405628 | CYB561D2 | | | | | |
| 3 | 50595455 | 50608458 | C3orf18 | | | | | |
| 3 | 50606582 | 50622421 | HEMK1 | | | | | |
| 3 | 50643884 | 50649262 | CISH | | | | | |
| 3 | 50649292 | 50686728 | MAPKAPK3 | | | | | |
| 3 | 50712510 | 50712594 | MIR4787 | | | | | |
| 3 | 50712671 | 51421629 | DOCK3 | | DOCK3 | | | |
| 3 | 51422667 | 51426828 | MANF | | | | | |
| 3 | 51428698 | 51435336 | RBM15B | | | | | |
| 3 | 51433297 | 51534018 | VPRBP | | | | | |
| 3 | 51575595 | 51697612 | RAD54L2 | | | | | |
| 3 | 51705190 | 51738339 | TEX264 | | | | | |
| 3 | 51741080 | 51752625 | GRM2 | | | GRM2 | | |
| 3 | 51812576 | 51813203 | IQCF6 | | | | | |
| 3 | 51851618 | 51853651 | IQCF4 | | | | | |
| 3 | 51860898 | 51864874 | IQCF3 | | | | | |
| 3 | 51895644 | 51897440 | IQCF2 | | | | | |
| 3 | 51907611 | 51909600 | IQCF5 | | | | | |
| 3 | 51928891 | 51937386 | IQCF1 | | | | | |
| 3 | 51967441 | 51975957 | RRP9 | | | | | |
| 3 | 51976320 | 51982883 | PARP3 | | | | | |
| 3 | 51989329 | 51991520 | GPR62 | | | | | |
| 3 | 51991469 | 52001482 | PCBP4 | | PCBP4 | | | |
| 3 | 52002525 | 52008646 | ABHD14B | | | | | |
| 3 | 52009041 | 52015216 | ABHD14A | | | | | |
| 3 | 52009041 | 52023218 | ABHD14A-ACY1 | | | | | |
| 3 | 52017299 | 52023218 | ACY1 | | | | | |
| 3 | 52027643 | 52029958 | RPL29 | | | | | |
| 3 | 52082936 | 52090461 | DUSP7 | | | | | |
| 3 | 52096109 | 52099128 | LINC00696 | | | | | |
| 3 | 52109248 | 52188706 | POC1A | | | | | |
| 3 | 52232098 | 52248343 | ALAS1 | | | | | |
| 3 | 52255095 | 52260179 | TLR9 | | | | | |
| 3 | 52262625 | 52273183 | TWF2 | | | | | |
| 3 | 52279808 | 52284615 | PPM1M | | | | | |
| 3 | 52288437 | 52312659 | WDR82 | | | | | |
| 3 | 52302293 | 52302377 | MIRLET7G | | | | | |
| 3 | 52321835 | 52323749 | GLYCTK | | | | | |

| | | | | | | | | |
|---|---|---|---|---|---|---|---|---|
| 3 | 52328234 | 52328324 | MIR135A1 | | | | | |
| 3 | 52350334 | 52434513 | DNAH1 | | | | | |
| 3 | 52435019 | 52444121 | BAP1 | | | | | |
| 3 | 52444576 | 52457657 | PHF7 | | | | | |
| 3 | 52467267 | 52479043 | SEMA3G | | | | | |
| 3 | 52485106 | 52488057 | TNNC1 | | | | | |
| 3 | 52489523 | 52527088 | NISCH | | | | | |
| 3 | 52529355 | 52558511 | STAB1 | | | STAB1 | | |
| 3 | 52558384 | 52569093 | NT5DC2 | | | | | |
| 3 | 52570620 | 52574586 | SMIM4 | | | | | |
| 3 | 52579367 | 52719866 | PBRM1 | | | | | |
| 3 | 52719935 | 52728510 | GNL3 | | | | | |
| 3 | 52722902 | 52723049 | SNORD136 | | | | | |
| 3 | 52723255 | 52723331 | SNORD19 | | | | | |
| 3 | 52724753 | 52724846 | SNORD19B | | | | | |
| 3 | 52726751 | 52726828 | SNORD69 | | | | | |
| 3 | 52728499 | 52740099 | GLT8D1 | | | | | |
| 3 | 52739856 | 52742197 | SPCS1 | | | | | |
| 3 | 52744795 | 52804965 | NEK4 | | | | | |
| 3 | 52811601 | 52826084 | ITIH1 | | | | | |
| 3 | 52828783 | 52843025 | ITIH3 | | | | | |
| 3 | 52847005 | 52859330 | ITIH4 | | | | | |
| 3 | 52867130 | 52869235 | MUSTN1 | | | | | |
| 3 | 52867130 | 52931597 | TMEM110-MUSTN1 | | | | | |
| 3 | 52870771 | 52931597 | TMEM110 | | | | | |
| 3 | 52880478 | 52880568 | MIR8064 | | | | | |
| 3 | 52937582 | 53080089 | SFMBT1 | | | | | |
| 3 | 53122500 | 53164470 | RFT1 | | | | | |
| 3 | 53195222 | 53226733 | PRKCD | | | PRKCD | | |
| 3 | 53258722 | 53290130 | TKT | | | | | |
| 3 | 53317444 | 53381654 | DCP1A | | | | | |
| 3 | 53529075 | 53846492 | CACNA1D | | CACNA1D | CACNA1D | CACNA1D | |
| 3 | 53850323 | 53880420 | CHDH | | | | | |
| 3 | 53880576 | 53899827 | IL17RB | | | | | |
| 3 | 53901093 | 53916229 | ACTR8 | | | | | |
| 3 | 53919225 | 53925989 | SELK | | | | | |
| 3 | 54156692 | 55108584 | CACNA2D3 | | | | | |
| 3 | 54666150 | 54673884 | ESRG | | | | | |
| 3 | 54908631 | 54935282 | CACNA2D3 | | | | | |
| 3 | 54952380 | 55001115 | LRTM1 | | | | | |
| 3 | 55499742 | 55521670 | WNT5A | | | | | |
| 3 | 55542338 | 56502391 | ERC2 | | | ERC2 | | |
| 3 | 55691242 | 55693497 | ERC2-IT1 | | | | | |
| 3 | 55886519 | 55886622 | MIR3938 | | | | | |
| 3 | 56591183 | 56655848 | CCDC66 | | | | | |
| 3 | 56654159 | 56717135 | FAM208A | | | | | |
| 3 | 56761445 | 56994880 | ARHGEF3 | | | | | |
| 3 | 57094468 | 57109460 | SPATA12 | | | | | |
| 3 | 57124009 | 57204345 | IL17RD | | | | | |
| 3 | 57231943 | 57234280 | HESX1 | | | | | |
| 3 | 57261764 | 57307498 | APPL1 | | | APPL1 | | |
| 3 | 57302378 | 57326710 | ASB14 | | | | | |
| 3 | 57327726 | 57530071 | DNAH12 | | | | | |
| 3 | 57541980 | 57547768 | PDE12 | | | | | |
| 3 | 57557089 | 57586642 | ARF4 | | | | | |
| 3 | 57611180 | 57678816 | DENND6A | | | | | |
| 3 | 57742982 | 57915741 | SLMAP | | | | | |
| 3 | 57994126 | 58157982 | FLNB | | | | | |
| 3 | 58178352 | 58196730 | DNASE1L3 | | | | | |
| 3 | 58223258 | 58280461 | ABHD6 | | | | | |
| 3 | 58291971 | 58305920 | RPP14 | | | | | |
| 3 | 58318616 | 58411854 | PXK | | | | | |
| 3 | 58413356 | 58419579 | PDHB | | | PDHB | | |
| 3 | 58477822 | 58488087 | KCTD6 | | | | | |
| 3 | 58490862 | 58522929 | ACOX2 | | | | | |
| 3 | 58549838 | 58613337 | FAM107A | | | | | |

| | | | | | | | | |
|---|---|---|---|---|---|---|---|---|
| 3 | 58592806 | 58652561 | FAM3D | | | | | |
| 3 | 58727736 | 59004819 | C3orf67 | | | | | |
| 3 | 59735035 | 61237133 | FHIT | | FHIT | FHIT | | |
| 3 | 60603537 | 60603603 | MIR548BB | | | | | |
| 3 | 61547242 | 62304622 | PTPRG | | | | | |
| 3 | 62304647 | 62321888 | C3orf14 | | | | | |
| 3 | 62355346 | 62359190 | FEZF2 | | | | | |
| 3 | 62384020 | 62861064 | CADPS | | CADPS | | | CADPS |
| 3 | 63088363 | 63110737 | LINC00698 | | | | | |
| 3 | 63263913 | 63602597 | SYNPR | | | SYNPR | | |
| 3 | 63638343 | 63650891 | SNTN | | | | | |
| 3 | 63805040 | 63834312 | C3orf49 | | | | | |
| 3 | 63819545 | 63847495 | THOC7 | | | | | |
| 3 | 63850232 | 63989136 | ATXN7 | | | ATXN7 | ATXN7 | |
| 3 | 63989697 | 64009686 | PSMD6 | | | | | |
| 3 | 64053639 | 64088807 | PRICKLE2 | | | | | |
| 3 | 64064036 | 64073039 | LINC00994 | | | | | |
| 3 | 64079525 | 64187236 | PRICKLE2 | | | | | |
| 3 | 64501330 | 64997143 | ADAMTS9 | | | ADAMTS9 | | |
| 3 | 64705682 | 64941858 | MIR548A2 | | | | | |
| 3 | 65339905 | 65910972 | MAGI1 | | | MAGI1 | | |
| 3 | 66119284 | 66429351 | SLC25A26 | | | | | |
| 3 | 66429220 | 66550845 | LRIG1 | | | | | |
| 3 | 67048726 | 67061632 | KBTBD8 | | | | | |
| 3 | 67275887 | 67275951 | MIR4272 | | | | | |
| 3 | 67410883 | 67998136 | SUCLG2 | | | SUCLG2 | | |
| 3 | 68040733 | 68594771 | FAM19A1 | | | | | |
| 3 | 68780914 | 68981761 | FAM19A4 | | | | | |
| 3 | 69024362 | 69063045 | EOGT | | | | | |
| 3 | 69068977 | 69101484 | TMF1 | | | | | |
| 3 | 69098108 | 69098186 | MIR3136 | | | | | |
| 3 | 69103880 | 69129524 | UBA3 | | | | | |
| 3 | 69134089 | 69155239 | ARL6IP5 | | | ARL6IP5 | | |
| 3 | 69157822 | 69171746 | LMOD3 | | | | | |
| 3 | 69217933 | 69435455 | FRMD4B | | | | | |
| 3 | 69788585 | 70017488 | MITF | | | MITF | | |
| 3 | 70048894 | 70064451 | LINC01212 | | | | | |
| 3 | 71003864 | 71355005 | FOXP1 | | | | | |
| 3 | 71591120 | 71591240 | MIR1284 | | | | | |
| 3 | 71728439 | 71803924 | EIF4E3 | | | | | |
| 3 | 71803200 | 71804328 | GPR27 | | | | | |
| 3 | 71820805 | 71834357 | PROK2 | | | | PROK2 | |
| 3 | 72084669 | 72149606 | LINC00877 | | | | | |
| 3 | 72200407 | 72223490 | LINC00870 | | | | | |
| 3 | 72423743 | 72495774 | RYBP | | | | | |
| 3 | 72798427 | 72897598 | SHQ1 | | | | | |
| 3 | 72937384 | 73024522 | GXYLT2 | | | | | |
| 3 | 73045893 | 73118352 | PPP4R2 | | | | | |
| 3 | 73110809 | 73112471 | EBLN2 | | | | | |
| 3 | 73431580 | 73674072 | PDZRN3 | | | | | |
| 3 | 73570472 | 73618925 | LOC101927296 | | | | | |
| 3 | 73672718 | 73677050 | PDZRN3 | | | | | |
| 3 | 74311719 | 74570291 | CNTN3 | | | | | |
| 3 | 75263626 | 75263699 | MIR4444 | | | | | |
| 3 | 75470702 | 75484266 | FAM86DP | | | | | |
| 3 | 75679913 | 75680009 | MIR1324 | | | | | |
| 3 | 75713480 | 75716368 | FRG2EP | | | | | |
| 3 | 75713486 | 75716368 | FRG2C | | | | | |
| 3 | 75721431 | 75728454 | LINC00960 | | | | | |
| 3 | 75779111 | 75834734 | ZNF717 | | | | | |
| 3 | 75787430 | 75787514 | MIR4273 | | | | | |
| 3 | 77089293 | 77699114 | ROBO2 | | | ROBO2 | | |
| 3 | 78646387 | 79817059 | ROBO1 | | ROBO1 | ROBO1 | | |
| 3 | 79460876 | 79484377 | LOC101927374 | | | | | |
| 3 | 79557036 | 79557119 | MIR3923 | | | | | |
| 3 | 81043018 | 81144798 | LOC728290 | | | | | |

| | | | | | | | | |
|---|---|---|---|---|---|---|---|---|
| 3 | 81538849 | 81810950 | GBE1 | | | | | |
| 3 | 84687555 | 84918726 | LINC00971 | | | | | |
| 3 | 85008132 | 86123579 | CADM2 | | | | | |
| 3 | 85111303 | 85111415 | SNORA95 | | | | | |
| 3 | 85434859 | 85434942 | MIR5688 | | | | | |
| 3 | 85554208 | 85877200 | CADM2 | | | | | |
| 3 | 86987122 | 87040257 | VGLL3 | | | | | |
| 3 | 87138429 | 87206219 | LINC00506 | | | | | |
| 3 | 87275338 | 87275427 | MIR4795 | | | | | |
| 3 | 87276412 | 87304698 | CHMP2B | | | CHMP2B | | |
| 3 | 87308782 | 87325737 | POU1F1 | | | POU1F1 | | |
| 3 | 88031725 | 88042919 | HTR1F | | | HTR1F | | |
| 3 | 88101099 | 88199016 | CGGBP1 | | | | | |
| 3 | 88188261 | 88193814 | ZNF654 | | | | | |
| 3 | 88198892 | 88207115 | C3orf38 | | | | | |
| 3 | 89156673 | 89531284 | EPHA3 | | | | | |
| 3 | 93591880 | 93692934 | PROS1 | | | PROS1 | | |
| 3 | 93698982 | 93774522 | ARL13B | | | | | |
| 3 | 93733214 | 93747454 | STX19 | | | | | |
| 3 | 93776765 | 93782067 | DHFRL1 | | | | | |
| 3 | 93781854 | 93845630 | NSUN3 | | | | | |
| 3 | 94309655 | 94309676 | MIR6730 | | | | | |
| 3 | 94657106 | 94710169 | LINC00879 | | | | | |
| 3 | 95373266 | 95402037 | MTHFD2P1 | | | | | |
| 3 | 96078807 | 96078883 | MIR8060 | | | | | |
| 3 | 96533424 | 97367421 | EPHA6 | | | | | |
| 3 | 97483364 | 97520086 | ARL6 | | ARL6 | | | |
| 3 | 97540883 | 97663830 | CRYBG3 | | | | | |
| 3 | 97660660 | 97691295 | MINA | | | | | |
| 3 | 97705526 | 97754148 | GABRR3 | | | | | |
| 3 | 97806016 | 97806946 | OR5AC2 | | | | | |
| 3 | 97851541 | 97852483 | OR5H1 | | | | | |
| 3 | 97868229 | 97869162 | OR5H14 | | | | | |
| 3 | 97887543 | 97888485 | OR5H15 | | | | | |
| 3 | 97983128 | 97984106 | OR5H6 | | | | | |
| 3 | 98001731 | 98002676 | OR5H2 | | | | | |
| 3 | 98072697 | 98073663 | OR5K4 | | | | | |
| 3 | 98109509 | 98110475 | OR5K3 | | | | | |
| 3 | 98188323 | 98189420 | OR5K1 | | | | | |
| 3 | 98216524 | 98217475 | OR5K2 | | | | | |
| 3 | 98234316 | 98241910 | CLDND1 | | | | | |
| 3 | 98250742 | 98251994 | GPR15 | | | | | |
| 3 | 98298289 | 98312455 | CPOX | | CPOX | CPOX | | |
| 3 | 98433176 | 98514689 | ST3GAL6 | | | | | |
| 3 | 98514813 | 98620533 | DCBLD2 | | | | | |
| 3 | 99273152 | 99717059 | MIR548G | | | | | |
| 3 | 99357439 | 99515577 | COL8A1 | | | | | |
| 3 | 99535475 | 99542709 | HP09053 | | | | | |
| 3 | 99536677 | 99897476 | CMSS1 | | | | | |
| 3 | 99551987 | 99833357 | FILIP1L | | | | | |
| 3 | 99683157 | 99683242 | MIR3921 | | | | | |
| 3 | 99833773 | 99897476 | CMSS1 | | | | | |
| 3 | 99904667 | 99913030 | TMEM30C | | | | | |
| 3 | 99979660 | 100044096 | TBC1D23 | | | | | |
| 3 | 100053561 | 100074478 | NIT2 | | | | | |
| 3 | 100082302 | 100120242 | TOMM70A | | | TOMM70A | | |
| 3 | 100120036 | 100175170 | LNP1 | | | | | |
| 3 | 100211462 | 100296296 | TMEM45A | | | | | |
| 3 | 100328432 | 100414325 | ADGRG7 | | | | | |
| 3 | 100428133 | 100467811 | TFG | | | | | |
| 3 | 100468178 | 100712334 | ABI3BP | | | ABI3BP | | |
| 3 | 100941389 | 101039419 | IMPG2 | | | | | |
| 3 | 101043032 | 101232085 | SENP7 | | | | | |
| 3 | 101237710 | 101242731 | FAM172BP | | | | | |
| 3 | 101280679 | 101285290 | TRMT10C | | | | | |
| 3 | 101293041 | 101313281 | PCNP | | | | | |

| | | | | | | | | | |
|---|---|---|---|---|---|---|---|---|---|
| 3 | 101368282 | 101398057 | ZBTB11 | | | | | | |
| 3 | 101399933 | 101405563 | RPL24 | | | | | | |
| 3 | 101431277 | 101432260 | PDCL3P4 | | | | | | |
| 3 | 101443436 | 101489406 | CEP97 | | | | | | |
| 3 | 101498028 | 101547075 | NXPE3 | | | | | | |
| 3 | 101546833 | 101579869 | NFKBIZ | | | | | | |
| 3 | 101659702 | 101716770 | LOC152225 | | | | | | |
| 3 | 102153858 | 102198685 | ZPLD1 | | | | | | |
| 3 | 103242876 | 103242960 | MIR548AB | | | | | | |
| 3 | 103903475 | 103946105 | MIR548A3 | | | | | | |
| 3 | 105085556 | 105295757 | ALCAM | | ALCAM | | | | |
| 3 | 105377108 | 105587887 | CBLB | | | | | | |
| 3 | 106828636 | 106959485 | LINC00882 | | | | | | |
| 3 | 106959538 | 107045811 | DUBR | | | | | | |
| 3 | 107096187 | 107097481 | CCDC54 | | | | | | |
| 3 | 107099322 | 107101006 | LOC101929579 | | | | | | |
| 3 | 107149776 | 107182759 | LOC101929607 | | | | | | |
| 3 | 107241782 | 107530176 | BBX | | | | | | |
| 3 | 107560508 | 107596915 | LINC00635 | | | | | | |
| 3 | 107602051 | 107647753 | LINC00636 | | | | | | |
| 3 | 107761940 | 107809935 | CD47 | | | CD47 | | | |
| 3 | 107843910 | 107857457 | LINC01215 | | | | | | |
| 3 | 107879658 | 107941417 | IFT57 | | | | | | |
| 3 | 108015336 | 108097131 | HHLA2 | | | | | | |
| 3 | 108099215 | 108248169 | MYH15 | | | | | | |
| 3 | 108268717 | 108308491 | KIAA1524 | | | | | | |
| 3 | 108308336 | 108413693 | DZIP3 | | | | | | |
| 3 | 108474485 | 108476130 | RETNLB | | | | | | |
| 3 | 108541544 | 108573852 | TRAT1 | | | | | | |
| 3 | 108626641 | 108672677 | GUCA1C | | | | | | |
| 3 | 108677086 | 108836993 | MORC1 | | | | | | |
| 3 | 108855560 | 108868951 | FLJ22763 | | | | | | |
| 3 | 108897011 | 108904108 | LINC00488 | | | | | | |
| 3 | 109012634 | 109035364 | DPPA2 | | | | | | |
| 3 | 109044987 | 109056419 | DPPA4 | | | | | | |
| 3 | 109128836 | 109214014 | LINC01205 | | | | | | |
| 3 | 109321674 | 109321744 | MIR4445 | | | | | | |
| 3 | 110764162 | 110913016 | PVRL3 | | | | | | |
| 3 | 111260925 | 111384838 | CD96 | | | | | | |
| 3 | 111311746 | 111314182 | ZBED2 | | | | | | |
| 3 | 111393522 | 111396280 | PLCXD2 | | | | | | |
| 3 | 111451326 | 111695364 | PHLDB2 | | | | | | |
| 3 | 111697722 | 111712215 | ABHD10 | | | | | | |
| 3 | 111717585 | 111732735 | TAGLN3 | | | | | | |
| 3 | 111758464 | 111800116 | TMPRSS7 | | | | | | |
| 3 | 111805181 | 111837073 | C3orf52 | | | | | | |
| 3 | 111831647 | 111831745 | MIR567 | | | | | | |
| 3 | 111839687 | 111852152 | GCSAM | | | | | | |
| 3 | 111859751 | 112013074 | SLC9C1 | | | | | | |
| 3 | 112051793 | 112081658 | CD200 | | | | | | |
| 3 | 112182812 | 112218408 | BTLA | | | | | | |
| 3 | 112251353 | 112280810 | ATG3 | | | | | | |
| 3 | 112280856 | 112303284 | SLC35A5 | | | | | | |
| 3 | 112315640 | 112320816 | LINC01279 | | | | | | |
| 3 | 112323232 | 112359990 | CCDC80 | | | | | | |
| 3 | 112455295 | 112468166 | LOC101929694 | | | | | | |
| 3 | 112534555 | 112564797 | CD200R1L | | | | | | |
| 3 | 112641531 | 112693937 | CD200R1 | | | | | | |
| 3 | 112709799 | 112720221 | GTPBP8 | | | | | | |
| 3 | 112721291 | 112738580 | C3orf17 | | | | | | |
| 3 | 112861196 | 112886578 | LOC101929717 | | | | | | |
| 3 | 112930311 | 113006310 | BOC | | | | | | |
| 3 | 113005776 | 113152839 | CFAP44 | | | | | | |
| 3 | 113150964 | 113151047 | MIR8076 | | | | | | |
| 3 | 113161564 | 113234034 | SPICE1 | | | | | | |
| 3 | 113251217 | 113348422 | SIDT1 | | | | | | |

| | | | | | | | | |
|---|---|---|---|---|---|---|---|---|
| 3 | 113313722 | 113313789 | MIR4446 | | | | | |
| 3 | 113367232 | 113415493 | USF3 | | | | | |
| 3 | 113435306 | 113465146 | NAA50 | | | | | |
| 3 | 113465865 | 113530905 | ATP6V1A | | ATP6V1A | | | |
| 3 | 113557680 | 113666021 | GRAMD1C | | | | | |
| 3 | 113666747 | 113681827 | ZDHHC23 | | | | | |
| 3 | 113682983 | 113775460 | CCDC191 | | | | | |
| 3 | 113775581 | 113807268 | QTRTD1 | | | | | |
| 3 | 113847498 | 113918254 | DRD3 | DRD3 | | DRD3 | DRD3 | |
| 3 | 113953479 | 113956425 | ZNF80 | | | | | |
| 3 | 114012832 | 114029135 | TIGIT | | | | | |
| 3 | 114033346 | 114866127 | ZBTB20 | | | | | |
| 3 | 114035321 | 114035416 | MIR568 | | | | | |
| 3 | 114070657 | 114107825 | ZBTB20 | | | | | |
| 3 | 114172439 | 114238979 | LOC101929754 | | | | | |
| 3 | 114462291 | 114462372 | MIR4796 | | | | | |
| 3 | 114591879 | 114821908 | ZBTB20 | | | | | |
| 3 | 115342150 | 115440334 | GAP43 | | | GAP43 | | |
| 3 | 115521209 | 116088932 | LSAMP | | | LSAMP | | |
| 3 | 116428634 | 116435887 | TUSC7 | | | | | |
| 3 | 116569123 | 116569214 | MIR4447 | | | | | |
| 3 | 116640277 | 116651085 | LINC00901 | | | | | |
| 3 | 118619478 | 118667088 | IGSF11 | | | | | |
| 3 | 118864996 | 118870302 | C3orf30 | | | | | |
| 3 | 118892424 | 118924000 | UPK1B | | | | | |
| 3 | 118930588 | 119009513 | B4GALT4 | | | B4GALT4 | | |
| 3 | 119013219 | 119041607 | ARHGAP31 | | | | | |
| 3 | 119147806 | 119182529 | TMEM39A | | | | | |
| 3 | 119187784 | 119213554 | POGLUT1 | | | | | |
| 3 | 119217367 | 119243125 | TIMMDC1 | | | | | |
| 3 | 119243139 | 119278481 | CD80 | | | | | |
| 3 | 119298279 | 119308792 | ADPRH | | | | | |
| 3 | 119316694 | 119348658 | PLA1A | | | | | |
| 3 | 119360898 | 119379437 | POPDC2 | | | | | |
| 3 | 119388371 | 119396243 | COX17 | | | | | |
| 3 | 119421868 | 119485949 | MAATS1 | | | | | |
| 3 | 119499330 | 119537332 | NR1I2 | | | | | |
| 3 | 119540801 | 119813264 | GSK3B | | GSK3B | | | |
| 3 | 119884327 | 119963142 | GPR156 | | | | | |
| 3 | 120043575 | 120068186 | LRRC58 | | | | | |
| 3 | 120113060 | 120169918 | FSTL1 | | | | | |
| 3 | 120114514 | 120114576 | MIR198 | | | | | |
| 3 | 120315127 | 120321258 | NDUFB4 | | | | | |
| 3 | 120347014 | 120401418 | HGD | | | | | |
| 3 | 120405527 | 120461384 | RABL3 | | | | | |
| 3 | 120461557 | 120501916 | GTF2E1 | | | | | |
| 3 | 120627049 | 121143608 | STXBP5L | | | | | |
| 3 | 120768486 | 120768562 | MIR5682 | | | | | |
| 3 | 121150272 | 121264853 | POLQ | | | | | |
| 3 | 121286777 | 121309469 | ARGFX | | | | | |
| 3 | 121312169 | 121349139 | FBXO40 | | | | | |
| 3 | 121350244 | 121379795 | HCLS1 | | | | | |
| 3 | 121382045 | 121468614 | GOLGB1 | | | | | |
| 3 | 121488607 | 121553926 | IQCB1 | | | | | |
| 3 | 121554026 | 121605373 | EAF2 | | | | | |
| 3 | 121613170 | 121663034 | SLC15A2 | | | | | |
| 3 | 121706169 | 121741127 | ILDR1 | | | | | |
| 3 | 121774208 | 121839988 | CD86 | | | | | |
| 3 | 121902529 | 122005344 | CASR | | | CASR | | |
| 3 | 122044010 | 122060815 | CSTA | | | | | |
| 3 | 122078437 | 122102078 | CCDC58 | | | | | |
| 3 | 122103022 | 122128961 | FAM162A | | | | | |
| 3 | 122130699 | 122134882 | WDR5B | | | | | |
| 3 | 122135052 | 122162094 | LOC102723582 | | | | | |
| 3 | 122140747 | 122233786 | KPNA1 | | | | | |
| 3 | 122246759 | 122283194 | PARP9 | | | | | |

| | | | | | | | | |
|---|---|---|---|---|---|---|---|---|
| 3 | 122283184 | 122294049 | DTX3L | | | | | |
| 3 | 122296448 | 122357894 | PARP15 | | | | | |
| 3 | 122399671 | 122449687 | PARP14 | | | | | |
| 3 | 122458843 | 122512666 | HSPBAP1 | | | | | |
| 3 | 122513900 | 122599986 | DIRC2 | | | | | |
| 3 | 122605359 | 122611263 | LOC100129550 | | | | | |
| 3 | 122628039 | 122747452 | SEMA5B | | | SEMA5B | | |
| 3 | 122785855 | 122880953 | PDIA5 | | | | | |
| 3 | 122880640 | 122880726 | MIR7110 | | | | | |
| 3 | 122920773 | 122992982 | SEC22A | | | | | |
| 3 | 123001142 | 123167392 | ADCY5 | | ADCY5 | | | |
| 3 | 123213362 | 123303924 | HACD2 | | | | | |
| 3 | 123304359 | 123411254 | MYLK | | | MYLK | | |
| 3 | 123632273 | 123680255 | CCDC14 | | | | | |
| 3 | 123687862 | 123711017 | ROPN1 | | | | | |
| 3 | 123813557 | 124440036 | KALRN | | KALRN | KALRN | KALRN | |
| 3 | 123851775 | 123851872 | MIR5002 | | | | | |
| 3 | 124093178 | 124093284 | MIR6083 | | | | | |
| 3 | 124303505 | 124440036 | KALRN | | KALRN | KALRN | KALRN | |
| 3 | 124449212 | 124468119 | UMPS | | | UMPS | | |
| 3 | 124451285 | 124451363 | MIR544B | | | | | |
| 3 | 124480794 | 124606152 | ITGB5 | | | | | |
| 3 | 124624288 | 124653595 | MUC13 | | | | | |
| 3 | 124684553 | 124774802 | HEG1 | | | | | |
| 3 | 124801479 | 124931609 | SLC12A8 | | | | | |
| 3 | 124870308 | 124870396 | MIR5092 | | | | | |
| 3 | 124944512 | 125094198 | ZNF148 | | | | | |
| 3 | 125165487 | 125239058 | SNX4 | | | | | |
| 3 | 125247701 | 125314381 | OSBPL11 | | | | | |
| 3 | 125509246 | 125509395 | MIR548I1 | | | | | |
| 3 | 125546079 | 125604920 | LOC101927056 | | | | | |
| 3 | 125635443 | 125648867 | FAM86JP | | | | | |
| 3 | 125648117 | 125655887 | ALG1L | | | | | |
| 3 | 125687986 | 125702296 | ROPN1B | | | | | |
| 3 | 125725199 | 125820391 | SLC41A3 | | | | | |
| 3 | 125822403 | 125929011 | ALDH1L1 | | | | | |
| 3 | 126061477 | 126076236 | KLF15 | | | | | |
| 3 | 126111874 | 126113641 | CCDC37 | | | | | |
| 3 | 126113781 | 126155398 | CFAP100 | | | | | |
| 3 | 126156443 | 126194762 | ZXDC | | | | | |
| 3 | 126200007 | 126236616 | UROC1 | | | | | |
| 3 | 126243130 | 126262134 | CHST13 | | | | | |
| 3 | 126245841 | 126277808 | C3orf22 | | | | | |
| 3 | 126290621 | 126327398 | TXNRD3NB | | | | | |
| 3 | 126325894 | 126373967 | TXNRD3 | | | | | |
| 3 | 126380923 | 126390782 | NUP210P1 | | | | | |
| 3 | 126423062 | 126679263 | CHCHD6 | | | | | |
| 3 | 126707436 | 126756235 | PLXNA1 | | | | | |
| 3 | 126911973 | 126917027 | C3orf56 | | | | | |
| 3 | 127041149 | 127109513 | LOC101927123 | | | | | |
| 3 | 127216443 | 127256623 | LINC01471 | | | | | |
| 3 | 127291906 | 127309602 | TPRA1 | | | | | |
| 3 | 127294108 | 127294174 | MIR6825 | | | | | |
| 3 | 127305953 | 127306019 | MIR7976 | | | | | |
| 3 | 127317199 | 127341278 | MCM2 | | | | | |
| 3 | 127348001 | 127391653 | PODXL2 | | | | | |
| 3 | 127391780 | 127399769 | ABTB1 | | | | | |
| 3 | 127407904 | 127542093 | MGLL | | | | | |
| 3 | 127641901 | 127706514 | KBTBD12 | | | | | |
| 3 | 127771211 | 127790526 | SEC61A1 | | | | | |
| 3 | 127783627 | 127842671 | RUVBL1 | | | | | |
| 3 | 127872299 | 128127489 | EEFSEC | | | | | |
| 3 | 128181274 | 128191160 | DNAJB8 | | | | | |
| 3 | 128198264 | 128222050 | GATA2 | | | | | |
| 3 | 128226677 | 128229429 | LOC90246 | | | | | |
| 3 | 128290842 | 128294929 | LINC01565 | | | | | |

| | | | | | | | | |
|---|---|---|---|---|---|---|---|---|
| 3 | 128338812 | 128369719 | RPN1 | | | | | |
| 3 | 128444978 | 128533641 | RAB7A | | | RAB7A | | |
| 3 | 128580350 | 128590384 | LOC653712 | | | | | |
| 3 | 128598332 | 128631957 | ACAD9 | | | | | |
| 3 | 128689781 | 128712986 | KIAA1257 | | | | | |
| 3 | 128720471 | 128759585 | EFCC1 | | | | | |
| 3 | 128779609 | 128781254 | GP9 | | | | | |
| 3 | 128806411 | 128840993 | RAB43 | | | | | |
| 3 | 128806411 | 128880073 | ISY1-RAB43 | | | | | |
| 3 | 128846258 | 128880073 | ISY1 | | | | | |
| 3 | 128886657 | 128902810 | CNBP | | | | | |
| 3 | 128968452 | 128996616 | COPG1 | | | | | |
| 3 | 128990988 | 128991086 | MIR6826 | | | | | |
| 3 | 128997683 | 129024135 | HMCES | | | | | |
| 3 | 129033613 | 129043412 | H1FX | | | | | |
| 3 | 129101676 | 129118282 | RPL32P3 | | | | | |
| 3 | 129116052 | 129116191 | SNORA7B | | | | | |
| 3 | 129120163 | 129147494 | EFCAB12 | | | | | |
| 3 | 129149786 | 129159022 | MBD4 | | | | | |
| 3 | 129158878 | 129239350 | IFT122 | | | | | |
| 3 | 129247481 | 129254187 | RHO | | | | | |
| 3 | 129262056 | 129270310 | H1FOO | | | | | |
| 3 | 129274055 | 129325582 | PLXND1 | | | | | |
| 3 | 129366634 | 129627755 | TMCC1 | | | | | |
| 3 | 129693235 | 129696781 | TRH | | | TRH | | |
| 3 | 129800673 | 129817233 | ALG1L2 | | | | | |
| 3 | 129816624 | 129830276 | FAM86HP | | | | | |
| 3 | 129931662 | 129992649 | COL6A4P2 | | | | | |
| 3 | 130064358 | 130203690 | COL6A5 | | | | | |
| 3 | 130279177 | 130395888 | COL6A6 | | | | | |
| 3 | 130397777 | 130465696 | PIK3R4 | | | | | |
| 3 | 130569368 | 130735555 | ATP2C1 | | | ATP2C1 | | |
| 3 | 130732716 | 130745698 | ASTE1 | | | | | |
| 3 | 130745693 | 131069309 | NEK11 | | | | | |
| 3 | 131043935 | 131100319 | LOC339874 | | | | | |
| 3 | 131080688 | 131083966 | NUDT16P1 | | | | | |
| 3 | 131100514 | 131107674 | NUDT16 | | | | | |
| 3 | 131181044 | 131221860 | MRPL3 | | | | | |
| 3 | 131197841 | 131198077 | SNORA58 | | | | | |
| 3 | 131252403 | 131758450 | CPNE4 | | | | | |
| 3 | 131704698 | 131704775 | MIR5704 | | | | | |
| 3 | 132036210 | 132087146 | ACPP | | | | | |
| 3 | 132136552 | 132257876 | DNAJC13 | | | | | |
| 3 | 132276981 | 132378975 | ACAD11 | | | | | |
| 3 | 132276981 | 132441303 | NPHP3-ACAD11 | | | | | |
| 3 | 132316080 | 132321485 | ACKR4 | | | | | |
| 3 | 132373289 | 132396944 | UBA5 | | | | | |
| 3 | 132399452 | 132593050 | NPHP3 | | | | | |
| 3 | 132757131 | 132975965 | TMEM108 | | | | | |
| 3 | 133118838 | 133194056 | BFSP2 | | | | | |
| 3 | 133292433 | 133309118 | CDV3 | | | | | |
| 3 | 133319448 | 133380737 | TOPBP1 | | | | | |
| 3 | 133464976 | 133497850 | TF | | TF | | | |
| 3 | 133502876 | 133540336 | SRPRB | | | | | |
| 3 | 133543079 | 133614691 | RAB6B | | | RAB6B | | |
| 3 | 133646989 | 133648656 | C3orf36 | | | | | |
| 3 | 133651539 | 133748920 | SLCO2A1 | | | | | |
| 3 | 133875977 | 133969586 | RYK | | | | | |
| 3 | 134074186 | 134094259 | AMOTL2 | | | AMOTL2 | | |
| 3 | 134086645 | 134086704 | MIR6827 | | | | | |
| 3 | 134156668 | 134156748 | MIR4788 | | | | | |
| 3 | 134196545 | 134204865 | ANAPC13 | | | | | |
| 3 | 134204574 | 134283870 | CEP63 | | | | | |
| 3 | 134318764 | 134369864 | KY | | | | | |
| 3 | 134514098 | 134979307 | EPHB1 | | | | | |
| 3 | 135684514 | 135866752 | PPP2R3A | | | | | |

| | | | | | | | | |
|---|---|---|---|---|---|---|---|---|
| 3 | 135867759 | 135914688 | MSL2 | | | | | |
| 3 | 135969166 | 136049013 | PCCB | | | PCCB | | |
| 3 | 136055998 | 136471245 | STAG1 | | | | | |
| 3 | 136537860 | 136574734 | SLC35G2 | | | | | |
| 3 | 136560817 | 136670452 | NCK1 | | | | | |
| 3 | 136676706 | 136729926 | IL20RB | | | | | |
| 3 | 137483133 | 137485172 | SOX14 | | | | | |
| 3 | 137490752 | 137499719 | LINC01210 | | | | | |
| 3 | 137717657 | 137752494 | CLDN18 | | | | | |
| 3 | 137780826 | 137832322 | DZIP1L | | | | | |
| 3 | 137842559 | 137851229 | A4GNT | | | | | |
| 3 | 137879829 | 137893791 | DBR1 | | | | | |
| 3 | 137906089 | 138017228 | ARMC8 | | | | | |
| 3 | 137980278 | 138048728 | NME9 | | | | | |
| 3 | 138066489 | 138124377 | MRAS | | | | | |
| 3 | 138153414 | 138197256 | ESYT3 | | | | | |
| 3 | 138213180 | 138313225 | CEP70 | | | | | |
| 3 | 138327541 | 138352213 | FAIM | | | | | |
| 3 | 138371539 | 138478201 | PIK3CB | | | | | |
| 3 | 138654030 | 138662862 | LINC01391 | | | | | |
| 3 | 138663065 | 138665982 | FOXL2 | | | FOXL2 | | |
| 3 | 138666075 | 138672830 | FOXL2NB | | | | | |
| 3 | 138722803 | 138725110 | PRR23A | | | | | |
| 3 | 138737872 | 138739768 | PRR23B | | | | | |
| 3 | 138760943 | 138763734 | PRR23C | | | | | |
| 3 | 138823026 | 138844005 | BPESC1 | | | | | |
| 3 | 138951833 | 138952364 | PISRT1 | | | | | |
| 3 | 139062860 | 139075887 | MRPS22 | | | MRPS22 | | |
| 3 | 139076432 | 139108522 | COPB2 | | | COPB2 | | |
| 3 | 139108644 | 139302161 | LOC100507291 | | | | | |
| 3 | 139171725 | 139195352 | RBP2 | | | | | |
| 3 | 139236275 | 139258671 | RBP1 | | | | | |
| 3 | 139279022 | 139396885 | NMNAT3 | | | | | |
| 3 | 139654026 | 140227631 | CLSTN2 | | | CLSTN2 | | |
| 3 | 140396865 | 140419992 | TRIM42 | | | | | |
| 3 | 140660661 | 140698785 | SLC25A36 | | | | | |
| 3 | 140770243 | 140867453 | SPSB4 | | | | | |
| 3 | 140950666 | 141013745 | PXYLP1 | | | | | |
| 3 | 141043054 | 141168632 | ZBTB38 | | | | | |
| 3 | 141205888 | 141334205 | RASA2 | | | | | |
| 3 | 141457050 | 141465645 | RNF7 | | | RNF7 | | |
| 3 | 141497042 | 141535892 | GRK7 | | | | | |
| 3 | 141595469 | 141645382 | ATP1B3 | | | | | |
| 3 | 141663269 | 141868386 | TFDP2 | | | | | |
| 3 | 141876368 | 141944449 | GK5 | | | | | |
| 3 | 142025448 | 142166904 | XRN1 | | | | | |
| 3 | 142168076 | 142297668 | ATR | | | | | |
| 3 | 142315228 | 142432505 | PLS1 | | | | | |
| 3 | 142443265 | 142526729 | TRPC1 | | TRPC1 | | | |
| 3 | 142536701 | 142608045 | PCOLCE2 | | | | | |
| 3 | 142645516 | 142661378 | LOC100507389 | | | | | |
| 3 | 142680073 | 142719932 | PAQR9 | | | | | |
| 3 | 142719686 | 142720309 | LOC100289361 | | | | | |
| 3 | 142720371 | 142779567 | U2SURP | | | | | |
| 3 | 142838617 | 142842856 | CHST2 | | CHST2 | | | |
| 3 | 142984063 | 143065913 | SLC9A9 | | | | | |
| 3 | 143690639 | 143711210 | C3orf58 | | | | | |
| 3 | 145787227 | 145879282 | PLOD2 | | | | | |
| 3 | 145910123 | 145968966 | PLSCR4 | | | | | |
| 3 | 146151074 | 146213778 | PLSCR2 | | | | | |
| 3 | 146232966 | 146262628 | PLSCR1 | | | PLSCR1 | | |
| 3 | 146303624 | 146324003 | PLSCR5 | | | | | |
| 3 | 147103834 | 147124596 | ZIC4 | | ZIC4 | ZIC4 | | |
| 3 | 147127180 | 147134506 | ZIC1 | | ZIC1 | ZIC1 | | |
| 3 | 147139113 | 147227697 | LOC440982 | | | | | |
| 3 | 147795945 | 147805816 | LOC100507461 | | | | | |

| | | | | | | | | |
|---|---|---|---|---|---|---|---|---|
| 3 | 148415657 | 148460790 | AGTR1 | | | | | |
| 3 | 148545587 | 148577972 | CPB1 | | | | | |
| 3 | 148583042 | 148614878 | CPA3 | | | | | |
| 3 | 148709194 | 148745456 | GYG1 | | | | | |
| 3 | 148747850 | 148820610 | HLTF | | | | | |
| 3 | 148847370 | 148890983 | HPS3 | | | HPS3 | | |
| 3 | 148880196 | 148939832 | CP | | | | | |
| 3 | 149036284 | 149051548 | TM4SF18 | | | TM4SF18 | | |
| 3 | 149086804 | 149104370 | TM4SF1 | | | TM4SF1 | | |
| 3 | 149192367 | 149221181 | TM4SF4 | | | TM4SF4 | | |
| 3 | 149235021 | 149379149 | WWTR1 | | | | | |
| 3 | 149456256 | 149470286 | COMMD2 | | | | | |
| 3 | 149479225 | 149510610 | ANKUB1 | | | | | |
| 3 | 149530474 | 149679925 | RNF13 | | | | | |
| 3 | 149682690 | 149688741 | PFN2 | | PFN2 | | | |
| 3 | 149689065 | 149691029 | LOC646903 | | | | | |
| 3 | 149956305 | 149957992 | LINC01213 | | | | | |
| 3 | 149983193 | 150041534 | LINC01214 | | | | | |
| 3 | 150126121 | 150177905 | TSC22D2 | | | | | |
| 3 | 150259779 | 150264428 | SERP1 | | | | | |
| 3 | 150264464 | 150303803 | EIF2A | | | | | |
| 3 | 150321065 | 150348234 | SELT | | | | | |
| 3 | 150377671 | 150437933 | ERICH6 | | | | | |
| 3 | 150452257 | 150456772 | LOC101928105 | | | | | |
| 3 | 150458909 | 150481263 | SIAH2 | | | SIAH2 | | |
| 3 | 150643949 | 150797617 | CLRN1 | | | | | |
| 3 | 150804584 | 151154465 | MED12L | | MED12L | | | |
| 3 | 150915618 | 150920988 | GPR171 | | | | | |
| 3 | 150929904 | 150996230 | P2RY14 | | | | | |
| 3 | 151011875 | 151034636 | GPR87 | | | | | |
| 3 | 151044095 | 151047337 | P2RY13 | | | | | |
| 3 | 151054630 | 151102600 | P2RY12 | | P2RY12 | | P2RY12 | |
| 3 | 151153777 | 151176497 | IGSF10 | | | | | |
| 3 | 151283663 | 151283783 | MIR5186 | | | | | |
| 3 | 151347319 | 151542394 | MIR548H2 | | | | | |
| 3 | 151451703 | 151645963 | AADACL2 | | | | | |
| 3 | 151488243 | 151502682 | AADACP1 | | | | | |
| 3 | 151531860 | 151546276 | AADAC | | | | | |
| 3 | 151591430 | 151599876 | SUCNR1 | | | | | |
| 3 | 151980404 | 152183569 | MBNL1 | | | | | |
| 3 | 152057486 | 152058779 | TMEM14EP | | | | | |
| 3 | 152552481 | 152559228 | P2RY1 | | | | | |
| 3 | 152880000 | 152888413 | RAP2B | | | | | |
| 3 | 153202283 | 153220486 | C3orf79 | | | | | |
| 3 | 153742189 | 153975616 | ARHGEF26 | | | | | |
| 3 | 153993456 | 154042286 | DHX36 | | | | | |
| 3 | 154055460 | 154147504 | GPR149 | | | | | |
| 3 | 154797435 | 154901518 | MME | | | | | |
| 3 | 154958733 | 154960912 | LINC01487 | | | | | |
| 3 | 155008020 | 155011489 | LOC100507537 | | | | | |
| 3 | 155197670 | 155421997 | PLCH1 | | | | | |
| 3 | 155480400 | 155524076 | C3orf33 | | | | | |
| 3 | 155544300 | 155572248 | SLC33A1 | | | | | |
| 3 | 155588324 | 155655520 | GMPS | | | | | |
| 3 | 155838336 | 156164725 | KCNAB1 | KCNAB1 | KCNAB1 | KCNAB1 | | |
| 3 | 156257341 | 156272989 | SSR3 | | | | | |
| 3 | 156390959 | 156424557 | TIPARP | | | | | |
| 3 | 156465131 | 156534851 | LINC00886 | | | | | |
| 3 | 156527059 | 156529810 | PA2G4P4 | | | | | |
| 3 | 156544095 | 156643045 | LEKR1 | | | | | |
| 3 | 156799455 | 156840791 | LINC00880 | | | | | |
| 3 | 156807669 | 156818924 | LINC00881 | | | | | |
| 3 | 156864290 | 156878549 | CCNL1 | | | | | |
| 3 | 156977531 | 157221136 | VEPH1 | | | | | |
| 3 | 157154579 | 157161417 | PTX3 | | | | | |
| 3 | 157177409 | 157221415 | VEPH1 | | | | | |

| | | | | | | | | |
|---|---|---|---|---|---|---|---|---|
| 3 | 157261132 | 157319021 | PQLC2L | | | | | |
| 3 | 157813799 | 157823952 | SHOX2 | | | SHOX2 | | |
| 3 | 157827840 | 158262624 | RSRC1 | | | | | |
| 3 | 158263008 | 158288855 | LOC100996447 | | | | | |
| 3 | 158288952 | 158324249 | MLF1 | | | | | |
| 3 | 158362316 | 158410360 | GFM1 | | | | | |
| 3 | 158384202 | 158390482 | LXN | | | | | |
| 3 | 158414896 | 158450275 | RARRES1 | | | | | |
| 3 | 158519714 | 158547508 | MFSD1 | | | | | |
| 3 | 158787040 | 158984096 | IQCJ | | | | | |
| 3 | 158787040 | 159615155 | IQCJ-SCHIP1 | | | | | |
| 3 | 158991035 | 159615155 | SCHIP1 | | | | | |
| 3 | 159000434 | 159000523 | MIR3919 | | | | | |
| 3 | 159483170 | 159486401 | IQCJ-SCHIP1-AS1 | | | | | |
| 3 | 159557649 | 159615155 | SCHIP1 | | | | | |
| 3 | 159631189 | 159713806 | IL12A | | | | | |
| 3 | 159738435 | 159749210 | LINC01100 | | | | | |
| 3 | 159943422 | 159946000 | C3orf80 | | | | | |
| 3 | 159974773 | 160117320 | IFT80 | | IFT80 | | | |
| 3 | 160117091 | 160152741 | SMC4 | | | | | |
| 3 | 160122375 | 160122473 | MIR15B | | | | | |
| 3 | 160122532 | 160122613 | MIR16 | | | | | |
| 3 | 160153290 | 160167626 | TRIM59 | | | | | |
| 3 | 160212782 | 160283376 | KPNA4 | | | | | |
| 3 | 160232694 | 160233024 | SCARNA7 | | | | | |
| 3 | 160394947 | 160396235 | ARL14 | | | | | |
| 3 | 160473991 | 160796695 | PPM1L | | | | | |
| 3 | 160801670 | 160823160 | B3GALNT1 | | B3GALNT1 | | | |
| 3 | 160939098 | 160969795 | NMD3 | | | | | |
| 3 | 161062579 | 161089871 | SPTSSB | | | | | |
| 3 | 161144214 | 161166030 | LOC101243545 | | | | | |
| 3 | 161214595 | 161221730 | OTOL1 | | | | | |
| 3 | 162895030 | 163021089 | LINC01192 | | | | | |
| 3 | 163889258 | 163889344 | MIR1263 | | | | | |
| 3 | 164431882 | 164549268 | LINC01324 | | | | | |
| 3 | 164696685 | 164796283 | SI | | | | | |
| 3 | 164904507 | 164914897 | SLITRK3 | | | | | |
| 3 | 164924747 | 165231516 | LINC01322 | | | | | |
| 3 | 165490691 | 165555253 | BCHE | BCHE | | BCHE | BCHE | |
| 3 | 166958076 | 167098085 | ZBBX | | | | | |
| 3 | 167110583 | 167126307 | LINC01327 | | | | | |
| 3 | 167159576 | 167191920 | SERPINI2 | | | | | |
| 3 | 167196472 | 167371289 | WDR49 | | | | | |
| 3 | 167401694 | 167452651 | PDCD10 | | | | | |
| 3 | 167453431 | 167543357 | SERPINI1 | | SERPINI1 | | | |
| 3 | 167613735 | 167641797 | LINC01330 | | | | | |
| 3 | 167726460 | 167813713 | GOLIM4 | | | | | |
| 3 | 167967309 | 168548374 | EGFEM1P | | | | | |
| 3 | 168269641 | 168269737 | MIR551B | | | | | |
| 3 | 168619732 | 168639781 | LOC100507661 | | | | | |
| 3 | 168801286 | 169381563 | MECOM | | | | | |
| 3 | 169165654 | 169194840 | LOC105374205 | | | | | |
| 3 | 169482397 | 169482848 | TERC | | | | | |
| 3 | 169484710 | 169487683 | ACTRT3 | | | | | |
| 3 | 169490852 | 169507504 | MYNN | | | | | |
| 3 | 169511215 | 169530574 | LRRC34 | | | | | |
| 3 | 169539709 | 169555560 | LRRIQ4 | | | | | |
| 3 | 169557028 | 169587723 | LRRC31 | | | | | |
| 3 | 169629359 | 169656956 | SAMD7 | | | | | |
| 3 | 169661771 | 169684522 | LOC100128164 | | | | | |
| 3 | 169684579 | 169716161 | SEC62 | | | | | |
| 3 | 169755734 | 169803183 | GPR160 | | | | | |
| 3 | 169805367 | 169899537 | PHC3 | | | | | |
| 3 | 169940219 | 170023770 | PRKCI | | | PRKCI | | |
| 3 | 170075472 | 170114637 | SKIL | | | | | |
| 3 | 170136652 | 170152479 | CLDN11 | | | CLDN11 | | |

| | | | | | | | | |
|---|---|---|---|---|---|---|---|---|
| 3 | 170140890 | 170140950 | MIR6828 | | | | | |
| 3 | 170177341 | 170303863 | SLC7A14 | | SLC7A14 | | | |
| 3 | 170582664 | 170588045 | RPL22L1 | | | | | |
| 3 | 170606203 | 170626426 | EIF5A2 | | | | | |
| 3 | 170714136 | 170744768 | SLC2A2 | | | | | |
| 3 | 170780291 | 171178197 | TNIK | | | TNIK | | |
| 3 | 170824452 | 170824548 | MIR569 | | | | | |
| 3 | 171064722 | 171178197 | TNIK | | | TNIK | | |
| 3 | 171318194 | 171528284 | PLD1 | | | PLD1 | | |
| 3 | 171561138 | 171618530 | TMEM212 | | | | | |
| 3 | 171757417 | 172118492 | FNDC3B | | | | | |
| 3 | 172161080 | 172166246 | GHSR | | | | | |
| 3 | 172223297 | 172241265 | TNFSF10 | | | | | |
| 3 | 172348434 | 172429008 | NCEH1 | | | | | |
| 3 | 172468474 | 172539264 | ECT2 | | | | | |
| 3 | 172607146 | 172859058 | SPATA16 | | | | | |
| 3 | 173116237 | 173638586 | NLGN1 | | NLGN1 | NLGN1 | NLGN1 | NLGN1 |
| 3 | 174577110 | 174988885 | NAALADL2 | | | | | |
| 3 | 175087328 | 175087410 | MIR4789 | | | | | |
| 3 | 175132985 | 175153256 | MIR548AY | | | | | |
| 3 | 175491365 | 175494121 | NAALADL2 | | | | | |
| 3 | 176232890 | 176232939 | MIR7977 | | | | | |
| 3 | 176321935 | 176353320 | LINC01208 | | | | | |
| 3 | 176531943 | 176534789 | LINC01209 | | | | | |
| 3 | 176738541 | 176915048 | TBL1XR1 | | | | | |
| 3 | 177012229 | 177041223 | LINC00501 | | | | | |
| 3 | 177159708 | 177470492 | LINC00578 | | | | | |
| 3 | 177534652 | 177617012 | KCCAT211 | | | | | |
| 3 | 178136988 | 178175093 | LINC01014 | | | | | |
| 3 | 178243254 | 178562217 | KCNMB2 | | | KCNMB2 | | |
| 3 | 178735010 | 178789656 | ZMAT3 | | ZMAT3 | | | |
| 3 | 178819158 | 178865761 | LOC101928739 | | | | | |
| 3 | 178866310 | 178952497 | PIK3CA | | | | | |
| 3 | 178957536 | 178984838 | KCNMB3 | | | KCNMB3 | | |
| 3 | 179040778 | 179053323 | ZNF639 | | | | | |
| 3 | 179065479 | 179111008 | MFN1 | | | MFN1 | | |
| 3 | 179113875 | 179169371 | GNB4 | | | | | |
| 3 | 179280667 | 179306193 | ACTL6A | | | | | |
| 3 | 179306254 | 179322434 | MRPL47 | | | | | |
| 3 | 179322574 | 179342288 | NDUFB5 | | | NDUFB5 | | |
| 3 | 179370932 | 179507189 | USP13 | | | | | |
| 3 | 179512746 | 179639687 | PEX5L | | | | | |
| 3 | 180131960 | 180140289 | LOC100505609 | | | | | |
| 3 | 180319917 | 180336135 | TTC14 | | | TTC14 | | |
| 3 | 180331795 | 180397283 | CCDC39 | | | | | |
| 3 | 180425377 | 180587966 | LOC101928882 | | | | | |
| 3 | 180630233 | 180700539 | FXR1 | | | | | |
| 3 | 180701497 | 180707562 | DNAJC19 | | | | | |
| 3 | 180774467 | 181460013 | SOX2-OT | | | | | |
| 3 | 180949524 | 180949631 | RNU6-2 | | | | | |
| 3 | 181138950 | 181160274 | LOC102724604 | | | | | |
| 3 | 181328121 | 181460013 | SOX2-OT | | | | | |
| 3 | 181429711 | 181432223 | SOX2 | | SOX2 | SOX2 | | |
| 3 | 181670151 | 181728464 | LINC01206 | | | | | |
| 3 | 182164757 | 182204150 | FLJ46066 | | | | | |
| 3 | 182216077 | 182224912 | LOC105374244 | | | | | |
| 3 | 182511290 | 182639421 | ATP11B | | | | | |
| 3 | 182655945 | 182703741 | DCUN1D1 | | | | | |
| 3 | 182733005 | 182817375 | MCCC1 | | | MCCC1 | | |
| 3 | 182840002 | 182880667 | LAMP3 | | | | | |
| 3 | 182895791 | 183146057 | MCF2L2 | | | | | |
| 3 | 182971031 | 182991179 | B3GNT5 | | | | | |
| 3 | 183165395 | 183173800 | LINC00888 | | | | | |
| 3 | 183205318 | 183270114 | KLHL6 | | | | | |
| 3 | 183353410 | 183402304 | KLHL24 | | | | | |
| 3 | 183415605 | 183530413 | YEATS2 | | | | | |

| | | | | | | | | |
|---|---|---|---|---|---|---|---|---|
| 3 | 183533663 | 183543393 | MAP6D1 | | | | | |
| 3 | 183547172 | 183602693 | PARL | | | PARL | | |
| 3 | 183637723 | 183729207 | ABCC5 | | | | | |
| 3 | 183749331 | 183757157 | HTR3D | | | | HTR3D | |
| 3 | 183770834 | 183778461 | HTR3C | | | | HTR3C | |
| 3 | 183812905 | 183824783 | HTR3E | | | | HTR3E | |
| 3 | 183852809 | 183863099 | EIF2B5 | | | | | |
| 3 | 183873283 | 183891314 | DVL3 | | | | | |
| 3 | 183892633 | 183901879 | AP2M1 | | | AP2M1 | | |
| 3 | 183903862 | 183911795 | ABCF3 | | | | | |
| 3 | 183948316 | 183960117 | VWA5B2 | | | | | |
| 3 | 183959192 | 183959277 | MIR1224 | | | | | |
| 3 | 183960116 | 183967313 | ALG3 | | | | | |
| 3 | 183967444 | 184010819 | ECE2 | | | | | |
| 3 | 183977002 | 183979251 | CAMK2N2 | | | | | |
| 3 | 183993798 | 184010819 | ECE2 | | | | | |
| 3 | 184016885 | 184026842 | PSMD2 | | | | | |
| 3 | 184032282 | 184053146 | EIF4G1 | | | | | |
| 3 | 184043483 | 184043559 | SNORD66 | | | | | |
| 3 | 184053716 | 184064063 | FAM131A | | | | | |
| 3 | 184063972 | 184079439 | CLCN2 | | CLCN2 | | | |
| 3 | 184079501 | 184086383 | POLR2H | | | | | |
| 3 | 184089722 | 184097476 | THPO | | | | | |
| 3 | 184097860 | 184107623 | CHRD | CHRD | | | | |
| 3 | 184264501 | 184274706 | EIF2B5 | | | | | |
| 3 | 184279586 | 184300196 | EPHB3 | | | | | |
| 3 | 184428154 | 184429836 | MAGEF1 | | | | | |
| 3 | 184474176 | 184490943 | LOC101928992 | | | | | |
| 3 | 184529930 | 184770402 | VPS8 | | | | | |
| 3 | 184795837 | 184870802 | C3orf70 | | | | | |
| 3 | 184880688 | 184971886 | EHHADH | | | EHHADH | | |
| 3 | 184970997 | 184971060 | MIR5588 | | | | | |
| 3 | 185000728 | 185206882 | MAP3K13 | | | MAP3K13 | | |
| 3 | 185207388 | 185216845 | TMEM41A | | | | | |
| 3 | 185225569 | 185270369 | LIPH | | | | | |
| 3 | 185304030 | 185348885 | SENP2 | | | | | |
| 3 | 185361526 | 185447575 | IGF2BP2 | | | | | |
| 3 | 185485634 | 185485692 | MIR548AQ | | | | | |
| 3 | 185632357 | 185655924 | TRA2B | | | | | |
| 3 | 185677757 | 185698665 | LOC344887 | | | | | |
| 3 | 185764105 | 185826901 | ETV5 | | | | | |
| 3 | 185864989 | 186080023 | DGKG | | | | | |
| 3 | 186172769 | 186211450 | LOC253573 | | | | | |
| 3 | 186256231 | 186262167 | CRYGS | | | | | |
| 3 | 186263855 | 186288332 | TBCCD1 | | | | | |
| 3 | 186288464 | 186303589 | DNAJB11 | | | | | |
| 3 | 186330849 | 186339107 | AHSG | | | AHSG | | |
| 3 | 186358148 | 186370797 | FETUB | | | | | |
| 3 | 186383740 | 186396029 | HRG | | | | | |
| 3 | 186435097 | 186462199 | KNG1 | | | | | |
| 3 | 186501360 | 186507685 | EIF4A2 | | | | | |
| 3 | 186502584 | 186502654 | SNORD2 | | | | | |
| 3 | 186504460 | 186504566 | MIR1248 | | | | | |
| 3 | 186504463 | 186504641 | SNORA81 | | | | | |
| 3 | 186505087 | 186505222 | SNORA63 | | | | | |
| 3 | 186505401 | 186505538 | SNORA4 | | | | | |
| 3 | 186507681 | 186524484 | RFC4 | | | | | |
| 3 | 186528668 | 186543310 | LOC102724699 | | | | | |
| 3 | 186560462 | 186573912 | ADIPOQ | | | | | |
| 3 | 186648314 | 186796341 | ST6GAL1 | | | | | |
| 3 | 186838740 | 186857263 | RPL39L | | | | | |
| 3 | 186914877 | 186925417 | LOC101929106 | | | | | |
| 3 | 186915273 | 186919253 | RTP1 | | | | | |
| 3 | 186933872 | 187009810 | MASP1 | | | | | |
| 3 | 187086167 | 187089369 | RTP4 | | | | | |
| 3 | 187386693 | 187388201 | SST | | | | | |

| | | | | | | | | |
|---|---|---|---|---|---|---|---|---|
| 3 | 187416046 | 187420345 | RTP2 | | | | | |
| 3 | 187420153 | 187450203 | LOC100131635 | | | | | |
| 3 | 187439164 | 187463513 | BCL6 | | | | | |
| 3 | 187868993 | 188608460 | LPP | | | | | |
| 3 | 187896330 | 187898596 | FLJ42393 | | | | | |
| 3 | 187930720 | 188286454 | LPP | | | | | |
| 3 | 188406568 | 188406654 | MIR28 | | | | | |
| 3 | 188659503 | 188958383 | TPRG1 | | | | | |
| 3 | 189349215 | 189615068 | TP63 | | | TP63 | | |
| 3 | 189547710 | 189547798 | MIR944 | | | | | |
| 3 | 189674516 | 189862635 | P3H2 | | | | | |
| 3 | 190023489 | 190040235 | CLDN1 | | | | | |
| 3 | 190105660 | 190129932 | CLDN16 | | | | | |
| 3 | 190146444 | 190167665 | TMEM207 | | | | | |
| 3 | 190231839 | 190374986 | IL1RAP | | | | | |
| 3 | 190570525 | 190580465 | GMNC | | | | | |
| 3 | 190595718 | 190595839 | SNAR-I | | | | | |
| 3 | 190930321 | 190952394 | OSTN | | | | | |
| 3 | 190984943 | 191048325 | UTS2B | | | | | |
| 3 | 191046873 | 191116459 | CCDC50 | | | | | |
| 3 | 191143312 | 191308145 | LINCR-0002 | | | | | |
| 3 | 191178951 | 191179245 | PYDC2 | | | | | |
| 3 | 191857181 | 192000884 | FGF12 | | | | | |
| 3 | 192514604 | 192635950 | MB21D2 | | | | | |
| 3 | 192958916 | 192988644 | HRASLS | | | | | |
| 3 | 192959567 | 192961761 | MGC2889 | | | | | |
| 3 | 192992830 | 193032310 | ATP13A5 | | | | | |
| 3 | 193116755 | 193273373 | ATP13A4 | | | | | |
| 3 | 193310932 | 193345121 | OPA1 | | OPA1 | OPA1 | | |
| 3 | 193675160 | 193721448 | LOC647323 | | | | | |
| 3 | 193710882 | 193712027 | DPPA2P3 | | | | | |
| 3 | 193853930 | 193856401 | HES1 | | | | | |
| 3 | 193920804 | 193967942 | LOC100505920 | | | | | |
| 3 | 193965436 | 193975561 | LOC101929337 | | | | | |
| 3 | 194018988 | 194030593 | LINC00887 | | | | | |
| 3 | 194060493 | 194072057 | CPN2 | | | | | |
| 3 | 194075975 | 194090472 | LRRC15 | | | | | |
| 3 | 194115549 | 194119995 | GP5 | | | | | |
| 3 | 194123402 | 194188968 | ATP13A3 | | | | | |
| 3 | 194207868 | 194209274 | LINC00884 | | | | | |
| 3 | 194304996 | 194354150 | TMEM44 | | | | | |
| 3 | 194361516 | 194393206 | LSG1 | | | | | |
| 3 | 194406621 | 194409766 | FAM43A | | | | | |
| 3 | 194429149 | 194502897 | LOC100507391 | | | | | |
| 3 | 194789007 | 194816786 | XXYLT1 | | | | | |
| 3 | 194855234 | 194855309 | MIR3137 | | | | | |
| 3 | 194868599 | 194873585 | XXYLT1 | | | | | |
| 3 | 194995464 | 195163817 | ACAP2 | | | | | |
| 3 | 195208644 | 195208667 | MIR5692C1 | | | | | |
| 3 | 195241217 | 195270224 | PPP1R2 | | | | | |
| 3 | 195295572 | 195311076 | APOD | | | APOD | | |
| 3 | 195384909 | 195415735 | SDHAP2 | | | | | |
| 3 | 195415368 | 195438746 | LINC00969 | | | | | |
| 3 | 195426271 | 195426368 | MIR570 | | | | | |
| 3 | 195447752 | 195460424 | MUC20 | | | | | |
| 3 | 195473637 | 195538844 | MUC4 | | | | | |
| 3 | 195563325 | 195587275 | LOC101929697 | | | | | |
| 3 | 195590234 | 195635880 | TNK2 | | TNK2 | | | |
| 3 | 195609199 | 195609266 | MIR6829 | | | | | |
| 3 | 195686791 | 195717150 | SDHAP1 | | | | | |
| 3 | 195776154 | 195809032 | TFRC | | | TFRC | | |
| 3 | 195869506 | 195887761 | LINC00885 | | | | | |
| 3 | 195924322 | 195938300 | ZDHHC19 | | | | | |
| 3 | 195943382 | 195960301 | SLC51A | | | | | |
| 3 | 195964615 | 196014623 | PCYT1A | | | | | |
| 3 | 196018089 | 196045165 | TCTEX1D2 | | | | | |

| | | | | | | | | |
|---|---|---|---|---|---|---|---|---|
| 3 | 196042955 | 196065291 | TM4SF19-TCTEX1D2 | | | | | |
| 3 | 196045202 | 196065291 | TM4SF19 | | | TM4SF19 | | |
| 3 | 196080368 | 196159345 | UBXN7 | | | | | |
| 3 | 196195656 | 196230639 | RNF168 | | | | | |
| 3 | 196233749 | 196242237 | SMCO1 | | | | | |
| 3 | 196281058 | 196295413 | WDR53 | | | | | |
| 3 | 196295724 | 196315930 | FBXO45 | | | | | |
| 3 | 196358374 | 196359458 | LINC01063 | | | | | |
| 3 | 196366566 | 196388874 | NRROS | | | | | |
| 3 | 196433147 | 196439165 | CEP19 | | | | | |
| 3 | 196439244 | 196462876 | PIGX | | | | | |
| 3 | 196466727 | 196559518 | PAK2 | | | PAK2 | | |
| 3 | 196594726 | 196661584 | SENP5 | | | | | |
| 3 | 196662272 | 196670884 | NCBP2 | | | | | |
| 3 | 196673213 | 196695742 | PIGZ | | | | | |
| 3 | 196728611 | 196756687 | MELTF | | | | | |
| 3 | 196729776 | 196731615 | MFI2 | | | | | |
| 3 | 196745823 | 196756686 | MELTF | | | | | |
| 3 | 196769430 | 197026143 | DLG1 | | DLG1 | DLG1 | DLG1 | DLG1 |
| 3 | 197020748 | 197020819 | MIR4797 | | | | | |
| 3 | 197025117 | 197030621 | DLG1 | | DLG1 | DLG1 | DLG1 | DLG1 |
| 3 | 197236653 | 197300194 | BDH1 | | | | | |
| 3 | 197340897 | 197354752 | LOC220729 | | | | | |
| 3 | 197398264 | 197476570 | RUBCN | | | | | |
| 3 | 197401366 | 197401447 | MIR922 | | | | | |
| 3 | 197476423 | 197511317 | FYTTD1 | | | | | |
| 3 | 197518096 | 197598456 | LRCH3 | | | | | |
| 3 | 197615945 | 197686886 | IQCG | | | | | |
| 3 | 197677051 | 197682721 | RPL35A | | | | | |
| 3 | 197687070 | 197766338 | LMLN | | LMLN | | | |
| 3 | 197784403 | 197807542 | ANKRD18DP | | | | | |
| 3 | 197879236 | 197907728 | FAM157A | | | | | |
| 4 | 53178 | 88099 | ZNF595 | | | | | |
| 4 | 53192 | 196095 | ZNF718 | | | | | |
| 4 | 206388 | 249773 | ZNF876P | | | | | |
| 4 | 264463 | 289944 | ZNF732 | | | | | |
| 4 | 331595 | 367691 | ZNF141 | | | | | |
| 4 | 343945 | 344041 | MIR571 | | | | | |
| 4 | 419223 | 467998 | ABCA11P | | | | | |
| 4 | 433772 | 492960 | ZNF721 | | | | | |
| 4 | 492988 | 533710 | PIGG | | | | | |
| 4 | 619362 | 664681 | PDE6B | | PDE6B | PDE6B | | |
| 4 | 666224 | 668127 | ATP5I | | | ATP5I | | |
| 4 | 671710 | 675817 | MYL5 | | | | | |
| 4 | 675612 | 683230 | MFSD7 | | | | | |
| 4 | 699529 | 764768 | PCGF3 | | | | | |
| 4 | 773936 | 775636 | LOC100129917 | | | | | |
| 4 | 778744 | 819945 | CPLX1 | | | | | |
| 4 | 843062 | 926178 | GAK | | | | | |
| 4 | 926174 | 952443 | TMEM175 | | | | | |
| 4 | 952671 | 967348 | DGKQ | | | | | |
| 4 | 972860 | 987228 | SLC26A1 | | | | | |
| 4 | 980784 | 998345 | IDUA | | | IDUA | | |
| 4 | 981444 | 987228 | SLC26A1 | | | | | |
| 4 | 1005609 | 1020686 | FGFRL1 | | | | | |
| 4 | 1065265 | 1107352 | RNF212 | | | | | |
| 4 | 1107426 | 1126763 | LOC105374344 | | | | | |
| 4 | 1108984 | 1116952 | TMED11P | | | | | |
| 4 | 1160720 | 1202750 | SPON2 | | | | | |
| 4 | 1189570 | 1202750 | LOC100130872 | | | | | |
| 4 | 1203907 | 1246795 | CTBP1 | | | | | |
| 4 | 1283638 | 1333936 | MAEA | | | | | |
| 4 | 1341053 | 1381837 | UVSSA | | | | | |
| 4 | 1385339 | 1389782 | CRIPAK | | | | | |
| 4 | 1396719 | 1400230 | NKX1-1 | | | | | |
| 4 | 1641607 | 1686040 | FAM53A | | | | | |

| | | | | | | | | |
|---|---|---|---|---|---|---|---|---|
| 4 | 1694457 | 1714468 | SLBP | | | | | |
| 4 | 1717678 | 1723084 | TMEM129 | | | | | |
| 4 | 1723216 | 1746905 | TACC3 | | | | | |
| 4 | 1795038 | 1810599 | FGFR3 | | FGFR3 | FGFR3 | | |
| 4 | 1813205 | 1857974 | LETM1 | | | LETM1 | | |
| 4 | 1873122 | 1950545 | WHSC1 | | | | | |
| 4 | 1976362 | 1976487 | SCARNA22 | | | | | |
| 4 | 1984440 | 2010962 | NELFA | | | | | |
| 4 | 1988110 | 1988204 | MIR943 | | | | | |
| 4 | 2043719 | 2045697 | C4orf48 | | | | | |
| 4 | 2061238 | 2070816 | NAT8L | | NAT8L | | | |
| 4 | 2073684 | 2243891 | POLN | | | | | |
| 4 | 2233562 | 2243891 | HAUS3 | | | | | |
| 4 | 2249159 | 2263739 | MXD4 | | | | | |
| 4 | 2251803 | 2251883 | MIR4800 | | | | | |
| 4 | 2271323 | 2420370 | ZFYVE28 | | | | | |
| 4 | 2420671 | 2464690 | CFAP99 | | | | | |
| 4 | 2470794 | 2517586 | RNF4 | | | | | |
| 4 | 2627158 | 2734302 | FAM193A | | | | | |
| 4 | 2743386 | 2758103 | TNIP2 | | | | | |
| 4 | 2794749 | 2842823 | SH3BP2 | | | | | |
| 4 | 2845583 | 2931802 | ADD1 | | | | | |
| 4 | 2932287 | 2936586 | MFSD10 | | | | | |
| 4 | 2937272 | 2965233 | NOP14 | | | | | |
| 4 | 2965342 | 3042474 | GRK4 | | GRK4 | | | |
| 4 | 3064972 | 3245687 | HTT | HTT | HTT | HTT | HTT | |
| 4 | 3250766 | 3258342 | MSANTD1 | | | | | |
| 4 | 3315873 | 3441640 | RGS12 | | | | | |
| 4 | 3443659 | 3451222 | HGFAC | | | | | |
| 4 | 3465032 | 3496209 | DOK7 | | | | | DOK7 |
| 4 | 3505323 | 3534224 | LRPAP1 | | | | | |
| 4 | 3578595 | 3592712 | LINC00955 | | | | | |
| 4 | 3675319 | 3679582 | LOC100133461 | | | | | |
| 4 | 3768295 | 3770253 | ADRA2C | | | ADRA2C | | |
| 4 | 3943486 | 3957155 | FAM86EP | | | | | |
| 4 | 4190529 | 4228621 | OTOP1 | | | | | |
| 4 | 4237268 | 4249969 | TMEM128 | | | | | |
| 4 | 4269428 | 4291896 | LYAR | | | | | |
| 4 | 4291923 | 4323513 | ZBTB49 | | | | | |
| 4 | 4387982 | 4420785 | NSG1 | | | | | |
| 4 | 4420695 | 4543775 | STX18 | | | STX18 | | |
| 4 | 4477847 | 4483427 | STX18-IT1 | | | | | |
| 4 | 4543857 | 4712664 | STX18 | | | STX18 | | |
| 4 | 4763486 | 4789101 | LOC101928279 | | | | | |
| 4 | 4846163 | 4852554 | LINC01396 | | | | | |
| 4 | 4861391 | 4865660 | MSX1 | | | | | |
| 4 | 4922534 | 4990398 | LOC101928306 | | | | | |
| 4 | 5016313 | 5021197 | CYTL1 | | | | | |
| 4 | 5053243 | 5502728 | STK32B | | | | | |
| 4 | 5526295 | 5529527 | LINC01587 | | | | | |
| 4 | 5564145 | 5711275 | EVC2 | | | | | |
| 4 | 5712923 | 5816031 | EVC | | | EVC | | |
| 4 | 5822490 | 5894810 | CRMP1 | | | | | |
| 4 | 5925001 | 5925055 | MIR378D1 | | | | | |
| 4 | 6027925 | 6202318 | JAKMIP1 | | | | | |
| 4 | 6202459 | 6235663 | LOC285484 | | | | | |
| 4 | 6271576 | 6304992 | WFS1 | | | | | |
| 4 | 6322304 | 6565327 | PPP2R2C | | PPP2R2C | | | |
| 4 | 6576900 | 6624131 | MAN2B2 | | | | | |
| 4 | 6641817 | 6644470 | MRFAP1 | | | | | |
| 4 | 6672454 | 6675608 | LOC105374366 | | | | | |
| 4 | 6675820 | 6677774 | LOC93622 | | | | | |
| 4 | 6695565 | 6698897 | S100P | | | | | |
| 4 | 6709428 | 6711606 | MRFAP1L1 | | | | | |
| 4 | 6717841 | 6719387 | BLOC1S4 | | | | | |
| 4 | 6784458 | 6885899 | KIAA0232 | | | | | |

| | | | | | | | | | |
|---|---|---|---|---|---|---|---|---|---|
| 4 | 6911170 | 7034845 | TBC1D14 | | | | | | |
| 4 | 7032280 | 7047958 | LOC100129931 | | | | | | |
| 4 | 7042575 | 7044728 | CCDC96 | | | | | | |
| 4 | 7045155 | 7059677 | TADA2B | | | | | | |
| 4 | 7060621 | 7069937 | GRPEL1 | | | GRPEL1 | | | |
| 4 | 7099150 | 7105103 | FLJ36777 | | | | | | |
| 4 | 7194373 | 7744564 | SORCS2 | | | | | | |
| 4 | 7312176 | 7312251 | MIR4798 | | | | | | |
| 4 | 7432020 | 7436700 | PSAPL1 | | | | | | |
| 4 | 7461754 | 7461845 | MIR4274 | | | | | | |
| 4 | 7755816 | 7941653 | AFAP1 | | | | | | |
| 4 | 7940727 | 7942023 | LOC389199 | | | | | | |
| 4 | 7967036 | 8160559 | ABLIM2 | | | | | | |
| 4 | 8007027 | 8007108 | MIR95 | | | | | | |
| 4 | 8008747 | 8160559 | ABLIM2 | | | | | | |
| 4 | 8200970 | 8242830 | SH3TC1 | | | | | | |
| 4 | 8271491 | 8308838 | HTRA3 | | | | | | |
| 4 | 8368008 | 8442452 | ACOX3 | | | | | | |
| 4 | 8442531 | 8478282 | TRMT44 | | | | | | |
| 4 | 8581963 | 8591750 | GPR78 | | | | | | |
| 4 | 8594386 | 8621488 | CPZ | | | | | | |
| 4 | 8847801 | 8873543 | HMX1 | | | | | | |
| 4 | 8951476 | 8952127 | LOC650293 | | | | | | |
| 4 | 9212382 | 9213975 | USP17L10 | | | | | | |
| 4 | 9217130 | 9218723 | USP17L11 | | | | | | |
| 4 | 9217130 | 9218723 | USP17L18 | | | | | | |
| 4 | 9217130 | 9218723 | USP17L20 | | | | | | |
| 4 | 9221877 | 9223470 | USP17L12 | | | | | | |
| 4 | 9221877 | 9223470 | USP17L21 | | | | | | |
| 4 | 9226621 | 9228214 | USP17L13 | | | | | | |
| 4 | 9236110 | 9238060 | USP17L15 | | | | | | |
| 4 | 9245604 | 9247197 | USP17L17 | | | | | | |
| 4 | 9250355 | 9251948 | USP17L11 | | | | | | |
| 4 | 9250355 | 9251948 | USP17L18 | | | | | | |
| 4 | 9255103 | 9256696 | USP17L19 | | | | | | |
| 4 | 9259849 | 9261442 | USP17L11 | | | | | | |
| 4 | 9259849 | 9261442 | USP17L20 | | | | | | |
| 4 | 9259849 | 9261442 | USP17L22 | | | | | | |
| 4 | 9264597 | 9266190 | USP17L12 | | | | | | |
| 4 | 9264597 | 9266190 | USP17L21 | | | | | | |
| 4 | 9269344 | 9270937 | USP17L20 | | | | | | |
| 4 | 9269344 | 9270937 | USP17L22 | | | | | | |
| 4 | 9326890 | 9328483 | USP17L24 | | | | | | |
| 4 | 9326890 | 9328483 | USP17L25 | | | | | | |
| 4 | 9326890 | 9328483 | USP17L26 | | | | | | |
| 4 | 9326890 | 9328483 | USP17L27 | | | | | | |
| 4 | 9326890 | 9328483 | USP17L28 | | | | | | |
| 4 | 9326890 | 9328483 | USP17L29 | | | | | | |
| 4 | 9326890 | 9328483 | USP17L30 | | | | | | |
| 4 | 9326890 | 9328483 | USP17L5 | | | | | | |
| 4 | 9331636 | 9333229 | USP17L24 | | | | | | |
| 4 | 9331636 | 9333229 | USP17L25 | | | | | | |
| 4 | 9331636 | 9333229 | USP17L26 | | | | | | |
| 4 | 9331636 | 9333229 | USP17L27 | | | | | | |
| 4 | 9331636 | 9333229 | USP17L28 | | | | | | |
| 4 | 9331636 | 9333229 | USP17L29 | | | | | | |
| 4 | 9331636 | 9333229 | USP17L30 | | | | | | |
| 4 | 9331636 | 9333229 | USP17L5 | | | | | | |
| 4 | 9336383 | 9337976 | USP17L24 | | | | | | |
| 4 | 9336383 | 9337976 | USP17L25 | | | | | | |
| 4 | 9336383 | 9337976 | USP17L26 | | | | | | |
| 4 | 9336383 | 9337976 | USP17L27 | | | | | | |
| 4 | 9336383 | 9337976 | USP17L28 | | | | | | |
| 4 | 9336383 | 9337976 | USP17L29 | | | | | | |
| 4 | 9336383 | 9337976 | USP17L30 | | | | | | |
| 4 | 9336383 | 9337976 | USP17L5 | | | | | | |

| | | | | | | | | |
|---|---|---|---|---|---|---|---|---|
| 4 | 9341128 | 9342721 | USP17L24 | | | | | |
| 4 | 9341128 | 9342721 | USP17L25 | | | | | |
| 4 | 9341128 | 9342721 | USP17L26 | | | | | |
| 4 | 9341128 | 9342721 | USP17L27 | | | | | |
| 4 | 9341128 | 9342721 | USP17L28 | | | | | |
| 4 | 9341128 | 9342721 | USP17L29 | | | | | |
| 4 | 9341128 | 9342721 | USP17L30 | | | | | |
| 4 | 9341128 | 9342721 | USP17L5 | | | | | |
| 4 | 9345873 | 9347466 | USP17L24 | | | | | |
| 4 | 9345873 | 9347466 | USP17L25 | | | | | |
| 4 | 9345873 | 9347466 | USP17L26 | | | | | |
| 4 | 9345873 | 9347466 | USP17L27 | | | | | |
| 4 | 9345873 | 9347466 | USP17L28 | | | | | |
| 4 | 9345873 | 9347466 | USP17L29 | | | | | |
| 4 | 9345873 | 9347466 | USP17L30 | | | | | |
| 4 | 9345873 | 9347466 | USP17L5 | | | | | |
| 4 | 9350618 | 9352211 | USP17L24 | | | | | |
| 4 | 9350618 | 9352211 | USP17L25 | | | | | |
| 4 | 9350618 | 9352211 | USP17L26 | | | | | |
| 4 | 9350618 | 9352211 | USP17L27 | | | | | |
| 4 | 9350618 | 9352211 | USP17L28 | | | | | |
| 4 | 9350618 | 9352211 | USP17L29 | | | | | |
| 4 | 9350618 | 9352211 | USP17L30 | | | | | |
| 4 | 9350618 | 9352211 | USP17L5 | | | | | |
| 4 | 9355363 | 9356956 | USP17L24 | | | | | |
| 4 | 9355363 | 9356956 | USP17L25 | | | | | |
| 4 | 9355363 | 9356956 | USP17L26 | | | | | |
| 4 | 9355363 | 9356956 | USP17L27 | | | | | |
| 4 | 9355363 | 9356956 | USP17L28 | | | | | |
| 4 | 9355363 | 9356956 | USP17L29 | | | | | |
| 4 | 9355363 | 9356956 | USP17L30 | | | | | |
| 4 | 9355363 | 9356956 | USP17L5 | | | | | |
| 4 | 9360107 | 9361700 | USP17L9P | | | | | |
| 4 | 9364854 | 9366447 | USP17L24 | | | | | |
| 4 | 9364854 | 9366447 | USP17L25 | | | | | |
| 4 | 9364854 | 9366447 | USP17L26 | | | | | |
| 4 | 9364854 | 9366447 | USP17L27 | | | | | |
| 4 | 9364854 | 9366447 | USP17L28 | | | | | |
| 4 | 9364854 | 9366447 | USP17L29 | | | | | |
| 4 | 9364854 | 9366447 | USP17L30 | | | | | |
| 4 | 9364854 | 9366447 | USP17L5 | | | | | |
| 4 | 9369599 | 9370796 | USP17L6P | | | | | |
| 4 | 9446259 | 9452240 | DEFB131 | | | | | |
| 4 | 9557788 | 9557937 | MIR548I2 | | | | | |
| 4 | 9783257 | 9785633 | DRD5 | DRD5 | | DRD5 | DRD5 | |
| 4 | 9827847 | 10041872 | SLC2A9 | | | | | |
| 4 | 10075962 | 10118573 | WDR1 | | | | | |
| 4 | 10080234 | 10080316 | MIR3138 | | | | | |
| 4 | 10441503 | 10459032 | ZNF518B | | | | | |
| 4 | 10491837 | 10686386 | CLNK | | | | | |
| 4 | 11370450 | 11370545 | MIR572 | | | | | |
| 4 | 11399987 | 11430537 | HS3ST1 | | | | | |
| 4 | 12225074 | 12252909 | LOC101929019 | | | | | |
| 4 | 13369346 | 13485989 | RAB28 | | | RAB28 | | |
| 4 | 13527942 | 13533041 | LINC01097 | | | | | |
| 4 | 13542453 | 13546114 | NKX3-2 | | | | | |
| 4 | 13547699 | 13549448 | LINC01096 | | | | | |
| 4 | 13570365 | 13629328 | BOD1L1 | | | | | |
| 4 | 13629488 | 13629581 | MIR5091 | | | | | |
| 4 | 13656802 | 13932852 | LINC01182 | | | | | |
| 4 | 14113591 | 14141676 | LINC01085 | | | | | |
| 4 | 14472089 | 14889793 | LINC00504 | | | | | |
| 4 | 14911584 | 15071777 | CPEB2 | | | | | |
| 4 | 15006565 | 15429538 | LOC101929095 | | | | | |
| 4 | 15341559 | 15447791 | C1QTNF7 | | | | | |
| 4 | 15471488 | 15603180 | CC2D2A | | | | | |

| | | | | | | | | |
|---|---|---|---|---|---|---|---|---|
| 4 | 15606006 | 15657035 | FBXL5 | | | | | |
| 4 | 15683351 | 15692070 | FAM200B | | | | | |
| 4 | 15704572 | 15733796 | BST1 | | | | | |
| 4 | 15779887 | 15854866 | CD38 | | | | | |
| 4 | 15937192 | 15940363 | FGFBP1 | | | | | |
| 4 | 15961862 | 15964859 | FGFBP2 | | | | | |
| 4 | 15969848 | 16085623 | PROM1 | | | | | |
| 4 | 16162127 | 16259810 | TAPT1 | | | | | |
| 4 | 16503156 | 16900432 | LDB2 | | | | | |
| 4 | 16595985 | 16623654 | MIR548AX | | | | | |
| 4 | 16752472 | 16900432 | LDB2 | | | | | |
| 4 | 17173379 | 17187680 | LOC101929123 | | | | | |
| 4 | 17488015 | 17513857 | QDPR | | QDPR | QDPR | | |
| 4 | 17516787 | 17528727 | CLRN2 | | | | | |
| 4 | 17578926 | 17609590 | LAP3 | | | | | |
| 4 | 17616250 | 17627251 | MED28 | | | | | |
| 4 | 17633708 | 17783135 | FAM184B | | | | | |
| 4 | 17802277 | 17812381 | DCAF16 | | | | | |
| 4 | 17812435 | 17846487 | NCAPG | | | | | |
| 4 | 17844838 | 18023483 | LCORL | | | | | |
| 4 | 20253527 | 20622184 | SLIT2 | | | SLIT2 | SLIT2 | |
| 4 | 20393811 | 20396479 | SLIT2-IT1 | | | | | |
| 4 | 20529897 | 20530007 | MIR218 | | | | | |
| 4 | 20697904 | 20729980 | PACRGL | | | | | |
| 4 | 20730238 | 21950374 | KCNIP4 | | | | | |
| 4 | 21466322 | 21466381 | MIR7978 | | | | | |
| 4 | 21583718 | 21615345 | LOC105374516 | | | | | |
| 4 | 21844963 | 21854811 | KCNIP4-IT1 | | | | | |
| 4 | 22328989 | 22341289 | LOC100505912 | | | | | |
| 4 | 22388996 | 22517677 | ADGRA3 | | | | | |
| 4 | 22694536 | 22821195 | GBA3 | | | | | |
| 4 | 23199716 | 23464726 | MIR548AJ2 | | | | | |
| 4 | 23793643 | 23891700 | PPARGC1A | | PPARGC1A | PPARGC1A | | |
| 4 | 24521814 | 24521913 | MIR573 | | | | | |
| 4 | 24529087 | 24586184 | DHX15 | | | | | |
| 4 | 24797084 | 24802467 | SOD3 | | | | | |
| 4 | 24807738 | 24981826 | CCDC149 | | | | | |
| 4 | 25000470 | 25032414 | LGI2 | | | | | |
| 4 | 25121626 | 25200127 | SEPSECS | | | | | |
| 4 | 25235652 | 25280831 | PI4K2B | | | | | |
| 4 | 25314395 | 25372005 | ZCCHC4 | | | | | |
| 4 | 25378847 | 25420120 | ANAPC4 | | | | | |
| 4 | 25532661 | 25570968 | LOC101929161 | | | | | |
| 4 | 25657434 | 25680368 | SLC34A2 | | | | | |
| 4 | 25749048 | 25865217 | SEL1L3 | | | | | |
| 4 | 25915813 | 25931501 | SMIM20 | | | | | |
| 4 | 26321331 | 26436752 | RBPJ | | | | | |
| 4 | 26483017 | 26492042 | CCKAR | | | | CCKAR | |
| 4 | 26585545 | 26757835 | TBC1D19 | | | | | |
| 4 | 26861245 | 26862221 | LOC105374546 | | | | | |
| 4 | 26862312 | 27027003 | STIM2 | | | | | |
| 4 | 27219100 | 27283847 | LOC101929199 | | | | | |
| 4 | 28821203 | 28821290 | MIR4275 | | | | | |
| 4 | 30722029 | 31148423 | PCDH7 | | | PCDH7 | | |
| 4 | 31172765 | 31213296 | LOC102723778 | | | | | |
| 4 | 31999000 | 32157028 | LOC102723828 | | | | | |
| 4 | 32007474 | 32024044 | LOC105377651 | | | | | |
| 4 | 32352659 | 32354842 | LOC101927363 | | | | | |
| 4 | 33897960 | 34041515 | LOC101928622 | | | | | |
| 4 | 36067619 | 36245979 | ARAP2 | | | | | |
| 4 | 36245737 | 36275837 | LOC439933 | | | | | |
| 4 | 36283236 | 36346407 | DTHD1 | | | | | |
| 4 | 36427987 | 36428050 | MIR1255B1 | | | | | |
| 4 | 37003400 | 37022328 | LOC100508631 | | | | | |
| 4 | 37243531 | 37243613 | MIR4801 | | | | | |
| 4 | 37246689 | 37451087 | NWD2 | | | | | |

| | | | | | | | | |
|---|---|---|---|---|---|---|---|---|
| 4 | 37455551 | 37595132 | C4orf19 | | | | | |
| 4 | 37592421 | 37687999 | RELL1 | | | | | |
| 4 | 37828281 | 37864559 | PGM2 | | | | | |
| 4 | 37892704 | 38140796 | TBC1D1 | | | | | |
| 4 | 37962055 | 37962631 | PTTG2 | | | | | |
| 4 | 38111801 | 38140796 | TBC1D1 | | | | | |
| 4 | 38422282 | 38524801 | LINC01258 | | | | | |
| 4 | 38614321 | 38703129 | KLF3 | | | | | |
| 4 | 38773859 | 38784611 | TLR10 | | | | | |
| 4 | 38797875 | 38806412 | TLR1 | | | | | |
| 4 | 38825328 | 38858438 | TLR6 | | | | | |
| 4 | 38869353 | 38947365 | FAM114A1 | | | | | |
| 4 | 38869652 | 38869748 | MIR574 | | | | | |
| 4 | 38968365 | 39034041 | TMEM156 | | | | | |
| 4 | 39046450 | 39127853 | KLHL5 | | | | | |
| 4 | 39105519 | 39584819 | MIR1273H | | | | | |
| 4 | 39184023 | 39287430 | WDR19 | | | | | |
| 4 | 39289068 | 39368001 | RFC1 | | | | | |
| 4 | 39408472 | 39453153 | KLB | | | | | |
| 4 | 39413529 | 39413594 | MIR5591 | | | | | |
| 4 | 39455744 | 39460568 | RPL9 | | | | | |
| 4 | 39460643 | 39479271 | LIAS | | | LIAS | | |
| 4 | 39481874 | 39483523 | LOC401127 | | | | | |
| 4 | 39500374 | 39596327 | UGDH | | | | | |
| 4 | 39548916 | 39640618 | SMIM14 | | | | | |
| 4 | 39699663 | 39784410 | UBE2K | | | | | |
| 4 | 39824482 | 39979576 | PDS5A | | | | | |
| 4 | 40044536 | 40058819 | LOC344967 | | | | | |
| 4 | 40058523 | 40159872 | N4BP2 | | | N4BP2 | | |
| 4 | 40192630 | 40246384 | RHOH | | | | | |
| 4 | 40309201 | 40332436 | LOC101060498 | | | | | |
| 4 | 40337345 | 40357234 | CHRNA9 | | | | | |
| 4 | 40425271 | 40631883 | RBM47 | | | | | |
| 4 | 40504056 | 40504136 | MIR4802 | | | | | |
| 4 | 40751913 | 40812002 | NSUN7 | | | | | |
| 4 | 40812043 | 41216635 | APBB2 | | | | APBB2 | APBB2 |
| 4 | 41222090 | 41270446 | UCHL1 | | UCHL1 | | | |
| 4 | 41361623 | 41702061 | LIMCH1 | | | | | |
| 4 | 41746098 | 41750987 | PHOX2B | | | PHOX2B | | |
| 4 | 41881538 | 41884628 | LINC00682 | | | | | |
| 4 | 41937136 | 41962824 | TMEM33 | | | | | |
| 4 | 41983712 | 41988484 | DCAF4L1 | | | | | |
| 4 | 41992522 | 42089551 | SLC30A9 | | | | | |
| 4 | 42112869 | 42154895 | BEND4 | | | | | |
| 4 | 42301028 | 42393285 | LOC105374428 | | | | | |
| 4 | 42399855 | 42404504 | SHISA3 | | | | | |
| 4 | 42410391 | 42659122 | ATP8A1 | | | ATP8A1 | | |
| 4 | 42895282 | 43032675 | GRXCR1 | | | | | |
| 4 | 44018877 | 44024078 | LVCAT1 | | | | | |
| 4 | 44175919 | 44450824 | KCTD8 | | | | | |
| 4 | 44624353 | 44653658 | YIPF7 | | | | | |
| 4 | 44680432 | 44702697 | GUF1 | | | | | |
| 4 | 44703811 | 44728651 | GNPDA2 | | | | | |
| 4 | 46037786 | 46126082 | GABRG1 | | GABRG1 | GABRG1 | GABRG1 | GABRG1 |
| 4 | 46251580 | 46392056 | GABRA2 | | GABRA2 | GABRA2 | GABRA2 | GABRA2 |
| 4 | 46736846 | 46911252 | COX7B2 | | | | | |
| 4 | 46920916 | 46996424 | GABRA4 | | GABRA4 | GABRA4 | GABRA4 | GABRA4 |
| 4 | 47033294 | 47428447 | GABRB1 | | | GABRB1 | | |
| 4 | 47452810 | 47465676 | COMMD8 | | | | | |
| 4 | 47487409 | 47595503 | ATP10D | | | | | |
| 4 | 47596014 | 47840123 | CORIN | | | CORIN | | |
| 4 | 47654685 | 47654758 | MIR8053 | | | | | |
| 4 | 47833361 | 47898865 | LOC101927179 | | | | | |
| 4 | 47849249 | 47916684 | NFXL1 | | | | | |
| 4 | 47916243 | 47992754 | LOC101927157 | | | | | |
| 4 | 47937993 | 48014961 | CNGA1 | | | | | |

| | | | | | | | | |
|---|---|---|---|---|---|---|---|---|
| 4 | 48018788 | 48042190 | NIPAL1 | | | | | |
| 4 | 48068409 | 48136273 | TXK | | | | | |
| 4 | 48137799 | 48271814 | TEC | | | | | |
| 4 | 48343612 | 48428215 | SLAIN2 | | | | | |
| 4 | 48485359 | 48491541 | SLC10A4 | | | | | |
| 4 | 48492268 | 48496406 | ZAR1 | | | | | |
| 4 | 48499379 | 48782316 | FRYL | | | | | |
| 4 | 48833059 | 48863834 | OCIAD1 | | | | | |
| 4 | 48887396 | 48908845 | OCIAD2 | | | | | |
| 4 | 48988264 | 49064095 | CWH43 | | | | | |
| 4 | 52709165 | 52783003 | DCUN1D4 | | | | | |
| 4 | 52859807 | 52883786 | LRRC66 | | | | | |
| 4 | 52886860 | 52904485 | SGCB | | SGCB | | | |
| 4 | 52917496 | 52963471 | SPATA18 | | | | | |
| 4 | 53457126 | 53527665 | USP46 | | | | | |
| 4 | 53578620 | 53580305 | DANCR | | | | | |
| 4 | 53578848 | 53578914 | MIR4449 | | | | | |
| 4 | 53579415 | 53579537 | SNORA26 | | | | | |
| 4 | 53609683 | 53617807 | ERVMER34-1 | | | | | |
| 4 | 53656160 | 53681631 | LINC01618 | | | | | |
| 4 | 53728494 | 53733002 | RASL11B | | | | | |
| 4 | 53739150 | 54232242 | SCFD2 | | | SCFD2 | | |
| 4 | 54243819 | 54326103 | FIP1L1 | | | | | |
| 4 | 54326436 | 54471548 | LNX1 | | | | | |
| 4 | 54562067 | 54600525 | LOC100506444 | | | | | |
| 4 | 54851665 | 54853449 | RPL21P44 | | | | | |
| 4 | 54875957 | 54930815 | CHIC2 | | | | | |
| 4 | 54966247 | 54968122 | GSX2 | | | | | |
| 4 | 55095263 | 55164412 | PDGFRA | | | | | |
| 4 | 55469377 | 55473298 | LOC339978 | | | | | |
| 4 | 55524094 | 55606881 | KIT | KIT | | | | |
| 4 | 55944425 | 55991762 | KDR | | | | | |
| 4 | 56212387 | 56251747 | SRD5A3 | | | | | |
| 4 | 56262079 | 56292342 | TMEM165 | | TMEM165 | | | |
| 4 | 56294067 | 56413076 | CLOCK | | | | | |
| 4 | 56422691 | 56458379 | PDCL2 | | | | | |
| 4 | 56461395 | 56502865 | NMU | | | | | |
| 4 | 56686236 | 56703430 | LOC644145 | | | | | |
| 4 | 56719815 | 56771244 | EXOC1 | | | | | |
| 4 | 56814973 | 56899529 | CEP135 | | | | | |
| 4 | 56963594 | 56963644 | MIR7641 | | | | | |
| 4 | 57036360 | 57196890 | KIAA1211 | | | | | |
| 4 | 57204450 | 57253674 | AASDH | | | | | |
| 4 | 57259528 | 57301802 | PPAT | | | | | |
| 4 | 57301914 | 57327534 | PAICS | | | | | |
| 4 | 57333761 | 57369847 | SRP72 | | | | | |
| 4 | 57371374 | 57390058 | ARL9 | | | | | |
| 4 | 57396774 | 57469489 | THEGL | | | | | |
| 4 | 57514153 | 57547872 | HOPX | | | | | |
| 4 | 57676025 | 57688035 | SPINK2 | | | | | |
| 4 | 57774041 | 57802010 | REST | | | | | |
| 4 | 57829509 | 57843826 | NOA1 | | | | | |
| 4 | 57844805 | 57897328 | POLR2B | | | | | |
| 4 | 57897236 | 58071465 | IGFBP7 | | | | | |
| 4 | 58292037 | 58332152 | LOC101928851 | | | | | |
| 4 | 59390679 | 59402493 | LOC105377671 | | | | | |
| 4 | 59849999 | 59912677 | LOC105377247 | | | | | |
| 4 | 59852905 | 59853872 | LOC105377245 | | | | | |
| 4 | 59879290 | 59912677 | LOC105377247 | | | | | |
| 4 | 61788336 | 61788402 | MIR548AG1 | | | | | |
| 4 | 62362838 | 63027483 | ADGRL3 | | ADGRL3 | | ADGRL3 | |
| 4 | 65144176 | 65275178 | TECRL | | | | | |
| 4 | 65779998 | 65870218 | LOC401134 | | | | | |
| 4 | 66185280 | 66559104 | EPHA5 | | | | | |
| 4 | 67142541 | 67142646 | MIR1269A | | | | | |
| 4 | 68283022 | 68287718 | LOC101927237 | | | | | |

| | | | | | | | | |
|---|---|---|---|---|---|---|---|---|
| 4 | 68337988 | 68411256 | CENPC | | | | | |
| 4 | 68424414 | 68473059 | STAP1 | | | | | |
| 4 | 68481478 | 68588222 | UBA6 | | | | | |
| 4 | 68603098 | 68621804 | GNRHR | | | | GNRHR | |
| 4 | 68686593 | 68749716 | TMPRSS11D | | | | | |
| 4 | 68776018 | 68829232 | TMPRSS11A | | | | | |
| 4 | 68857529 | 68863157 | TMPRSS11GP | | | | | |
| 4 | 68918915 | 68995587 | TMPRSS11F | | | | | |
| 4 | 68919225 | 68938951 | LOC550113 | | | | | |
| 4 | 68926327 | 68929015 | SYT14P1 | | | | | |
| 4 | 69048009 | 69078188 | FTLP10 | | | | | |
| 4 | 69049846 | 69083798 | TMPRSS11BNL | | | | | |
| 4 | 69092370 | 69111412 | TMPRSS11B | | | | | |
| 4 | 69176104 | 69215824 | YTHDC1 | | | | | |
| 4 | 69313166 | 69363322 | TMPRSS11E | | | | | |
| 4 | 69402902 | 69434245 | UGT2B17 | | | | | |
| 4 | 69512314 | 69536494 | UGT2B15 | | | | | |
| 4 | 69681710 | 69697735 | UGT2B10 | | | | | |
| 4 | 69794176 | 69817509 | UGT2A3 | | | | | |
| 4 | 69870294 | 69886115 | UGT2B10 | | | | | |
| 4 | 69962192 | 69978705 | UGT2B7 | | | | | |
| 4 | 70066050 | 70080449 | UGT2B11 | | | | | |
| 4 | 70146216 | 70160768 | UGT2B28 | | | | | |
| 4 | 70345882 | 70391732 | UGT2B4 | | | | | |
| 4 | 70454134 | 70505360 | UGT2A2 | | | | | |
| 4 | 70454134 | 70518967 | UGT2A1 | | | | | |
| 4 | 70592685 | 70626430 | SULT1B1 | | | SULT1B1 | | |
| 4 | 70706929 | 70725870 | SULT1E1 | | | | | |
| 4 | 70796798 | 70812288 | CSN1S1 | | | | | |
| 4 | 70820973 | 70826729 | CSN2 | | | | | |
| 4 | 70861647 | 70868173 | STATH | | | | | |
| 4 | 70894129 | 70902255 | HTN3 | | | | | |
| 4 | 70916131 | 70924565 | HTN1 | | | | | |
| 4 | 70933102 | 70950988 | CSN1S2AP | | | | | |
| 4 | 70999320 | 71012421 | CSN1S2BP | | | | | |
| 4 | 71019903 | 71032326 | PRR27 | | | | | |
| 4 | 71062243 | 71070293 | ODAM | | | | | |
| 4 | 71091787 | 71100968 | FDCSP | | | | | |
| 4 | 71108332 | 71117145 | CSN3 | | | | | |
| 4 | 71200670 | 71202833 | CABS1 | | | | | |
| 4 | 71226492 | 71232823 | SMR3A | | | | | |
| 4 | 71248794 | 71255961 | SMR3B | | | | | |
| 4 | 71263598 | 71275914 | PROL1 | | | | | |
| 4 | 71296208 | 71348714 | MUC7 | | | | | |
| 4 | 71384288 | 71398460 | AMTN | | | | | |
| 4 | 71457974 | 71473004 | AMBN | | | | | |
| 4 | 71494460 | 71512536 | ENAM | | | | | |
| 4 | 71521257 | 71532348 | JCHAIN | | | | | |
| 4 | 71554195 | 71556268 | UTP3 | | | | | |
| 4 | 71570653 | 71674339 | RUFY3 | | | | | |
| 4 | 71681498 | 71705627 | GRSF1 | | | | | |
| 4 | 71768056 | 71853891 | MOB1B | | | | | |
| 4 | 71859264 | 71896629 | DCK | | | | | |
| 4 | 72053002 | 72437804 | SLC4A4 | | SLC4A4 | | | |
| 4 | 72607410 | 72671237 | GC | | | | | |
| 4 | 72897520 | 73013918 | NPFFR2 | | | NPFFR2 | | |
| 4 | 73146685 | 73434516 | ADAMTS3 | | | ADAMTS3 | | |
| 4 | 73920412 | 73935476 | COX18 | | | | | |
| 4 | 73939682 | 74124515 | ANKRD17 | | | | | |
| 4 | 74269971 | 74287129 | ALB | | | ALB | | |
| 4 | 74301935 | 74321502 | AFP | | | | | |
| 4 | 74347461 | 74369718 | AFM | | | | | |
| 4 | 74374519 | 74394250 | LOC728040 | | | | | |
| 4 | 74437266 | 74486348 | RASSF6 | | | | | |
| 4 | 74606222 | 74609433 | CXCL8 | | | | | |
| 4 | 74702272 | 74704477 | CXCL6 | | | | | |

| | | | | | | | | |
|---|---|---|---|---|---|---|---|---|
| 4 | 74719012 | 74720201 | PF4V1 | | | | | |
| 4 | 74735108 | 74737019 | CXCL1 | | | | | |
| 4 | 74846541 | 74847841 | PF4 | | | | | |
| 4 | 74852155 | 74853907 | PPBP | | | | | |
| 4 | 74861358 | 74864446 | CXCL5 | | | | | |
| 4 | 74902311 | 74904490 | CXCL3 | | | | | |
| 4 | 74919754 | 74921116 | PPBPP2 | | | | | |
| 4 | 74962753 | 74964997 | CXCL2 | | | | | |
| 4 | 75023828 | 75168814 | MTHFD2L | | | | | |
| 4 | 75174186 | 75182520 | EPGN | | | | | |
| 4 | 75230859 | 75254477 | EREG | | | | | |
| 4 | 75310814 | 75490485 | AREG | | | | | |
| 4 | 75669969 | 75719896 | BTC | | | | | |
| 4 | 75858284 | 75975325 | PARM1 | | | | | |
| 4 | 75881183 | 75895572 | LOC100507388 | | | | | |
| 4 | 76279285 | 76287776 | LOC441025 | | | | | |
| 4 | 76404246 | 76439640 | RCHY1 | | | | | |
| 4 | 76439642 | 76472542 | THAP6 | | | | | |
| 4 | 76481257 | 76491103 | C4orf26 | | | | | |
| 4 | 76501703 | 76555721 | CDKL2 | | | | | |
| 4 | 76567952 | 76598667 | G3BP2 | | | | | |
| 4 | 76649705 | 76735442 | USO1 | | | | | |
| 4 | 76781025 | 76823681 | PPEF2 | | | | | |
| 4 | 76831807 | 76862166 | NAAA | | | | | |
| 4 | 76871058 | 76912115 | SDAD1 | | | | | |
| 4 | 76901942 | 76927094 | LOC101928809 | | | | | |
| 4 | 76922494 | 76928676 | CXCL9 | | | | | |
| 4 | 76932332 | 77033955 | ART3 | | | | | |
| 4 | 76942268 | 76944689 | CXCL10 | | | | | |
| 4 | 76954839 | 76957350 | CXCL11 | | | | | |
| 4 | 76995847 | 77033955 | ART3 | | | | | |
| 4 | 77035811 | 77069668 | NUP54 | | | | | |
| 4 | 77079891 | 77135052 | SCARB2 | | SCARB2 | | | |
| 4 | 77135192 | 77204936 | FAM47E | | | | | |
| 4 | 77172852 | 77232283 | FAM47E-STBD1 | | | | | |
| 4 | 77227178 | 77232283 | STBD1 | | | | | |
| 4 | 77234191 | 77328458 | CCDC158 | | | | | |
| 4 | 77356252 | 77704405 | SHROOM3 | | | | | |
| 4 | 77494720 | 77494785 | MIR4450 | | | | | |
| 4 | 77496703 | 77496779 | MIR548AH | | | | | |
| 4 | 77816081 | 77819002 | SOWAHB | | | | | |
| 4 | 77870866 | 77959768 | SEPT11 | | | | | |
| 4 | 77969176 | 77997125 | CCNI | | | | | |
| 4 | 78078356 | 78091213 | CCNG2 | | | | | |
| 4 | 78432906 | 78532988 | CXCL13 | | | | | |
| 4 | 78634540 | 78740544 | CNOT6L | | | | | |
| 4 | 78783804 | 78873944 | MRPL1 | | | | | |
| 4 | 78978723 | 79465423 | FRAS1 | | | | | |
| 4 | 79472741 | 79531605 | ANXA3 | | | | | |
| 4 | 79567147 | 79605655 | LINC01094 | | | | | |
| 4 | 79697531 | 79833341 | BMP2K | | | | | |
| 4 | 79741905 | 79741975 | MIR5096 | | | | | |
| 4 | 79839093 | 79860582 | PAQR3 | | | | | |
| 4 | 79892901 | 80229953 | LINC01088 | | | | | |
| 4 | 80238271 | 80247171 | NAA11 | | | | | |
| 4 | 80327506 | 80329372 | GK2 | | | | | |
| 4 | 80413746 | 80497614 | LINC00989 | | | | | |
| 4 | 80748624 | 80784401 | PCAT4 | | | | | |
| 4 | 80822770 | 80994477 | ANTXR2 | | | ANTXR2 | | |
| 4 | 81106423 | 81125482 | PRDM8 | | | PRDM8 | | |
| 4 | 81187741 | 81212171 | FGF5 | | | | | |
| 4 | 81256873 | 81884910 | C4orf22 | | | | | |
| 4 | 81952118 | 81978685 | BMP3 | | | | | |
| 4 | 82008523 | 82136271 | PRKG2 | | | | | |
| 4 | 82086093 | 82114549 | LOC101928942 | | | | | |
| 4 | 82347546 | 82393082 | RASGEF1B | | | | | |

| | | | | | | | | | |
|---|---|---|---|---|---|---|---|---|---|
| 4 | 83274466 | 83295149 | HNRNPD | | | | | |
| 4 | 83343716 | 83351378 | HNRNPDL | | | | | |
| 4 | 83351632 | 83382328 | ENOPH1 | | | | | |
| 4 | 83405603 | 83483126 | TMEM150C | | | | | |
| 4 | 83534265 | 83542590 | LINC00575 | | | | | |
| 4 | 83550689 | 83720010 | SCD5 | | | | | |
| 4 | 83674489 | 83674583 | MIR575 | | | | | |
| 4 | 83739661 | 83821724 | SEC31A | | | | | |
| 4 | 83814604 | 83822069 | THAP9 | | | | | |
| 4 | 83817005 | 83817081 | SNORD143 | | | | | |
| 4 | 83819292 | 83819378 | SNORD144 | | | | | |
| 4 | 83821802 | 83841122 | THAP9 | | | | | |
| 4 | 83845756 | 83934094 | LIN54 | | | | | |
| 4 | 83956238 | 83996971 | COPS4 | | | COPS4 | | |
| 4 | 84011210 | 84035911 | PLAC8 | | | | | |
| 4 | 84184976 | 84206067 | COQ2 | | | | | |
| 4 | 84213613 | 84256306 | HPSE | | | | | |
| 4 | 84328495 | 84377036 | HELQ | | | | | |
| 4 | 84377084 | 84382876 | MRPS18C | | | MRPS18C | | |
| 4 | 84382093 | 84406290 | FAM175A | | | | | |
| 4 | 84457066 | 84527027 | GPAT3 | | | | | |
| 4 | 84889234 | 85220322 | LOC101928978 | | | | | |
| 4 | 85414435 | 85419387 | NKX6-1 | | | | | |
| 4 | 85504056 | 85572493 | CDS1 | | | | | |
| 4 | 85590692 | 85928168 | WDFY3 | | WDFY3 | | | |
| 4 | 86396283 | 86923823 | ARHGAP24 | | | | | |
| 4 | 86643620 | 86643686 | MIR4451 | | | | | |
| 4 | 86699850 | 86923823 | ARHGAP24 | | | | | |
| 4 | 86933448 | 87374283 | MAPK10 | | MAPK10 | MAPK10 | | |
| 4 | 87040958 | 87141079 | LOC101929064 | | | | | |
| 4 | 87463634 | 87463705 | MIR4452 | | | | | |
| 4 | 87515467 | 87736328 | PTPN13 | | | | | |
| 4 | 87744620 | 87770416 | SLC10A6 | | | | | |
| 4 | 87797357 | 87813575 | C4orf36 | | | | | |
| 4 | 87846045 | 87856002 | LOC100506746 | | | | | |
| 4 | 87856153 | 88062206 | AFF1 | | | | | |
| 4 | 88081255 | 88141760 | KLHL8 | | | | | |
| 4 | 88221646 | 88221735 | MIR5705 | | | | | |
| 4 | 88224940 | 88244058 | HSD17B13 | | | | | |
| 4 | 88257676 | 88312455 | HSD17B11 | | | | | |
| 4 | 88343727 | 88380606 | NUDT9 | | | NUDT9 | | |
| 4 | 88394481 | 88450655 | SPARCL1 | | | | | |
| 4 | 88529680 | 88538025 | DSPP | | | | | |
| 4 | 88571453 | 88585512 | DMP1 | | | | | |
| 4 | 88720701 | 88733601 | IBSP | | | | | |
| 4 | 88742549 | 88767968 | MEPE | | | | | |
| 4 | 88896801 | 88904563 | SPP1 | | SPP1 | | | |
| 4 | 88928798 | 88998931 | PKD2 | | | | | |
| 4 | 89011415 | 89152474 | ABCG2 | | ABCG2 | | | |
| 4 | 89178760 | 89205983 | PPM1K | | PPM1K | | | |
| 4 | 89206081 | 89262809 | LOC105369192 | | | | | |
| 4 | 89299890 | 89364249 | HERC6 | | | | | |
| 4 | 89378267 | 89427319 | HERC5 | | | | | |
| 4 | 89442128 | 89444952 | PIGY | | | | | |
| 4 | 89442128 | 89444952 | PYURF | | | | | |
| 4 | 89444960 | 89446148 | LOC101929134 | | | | | |
| 4 | 89513573 | 89629693 | HERC3 | | | | | |
| 4 | 89617063 | 89619386 | NAP1L5 | | NAP1L5 | | | |
| 4 | 89630939 | 89978346 | FAM13A | | | | | |
| 4 | 90033967 | 90036052 | TIGD2 | | | | | |
| 4 | 90165428 | 90229161 | GPRIN3 | | | | | |
| 4 | 90645249 | 90763142 | SNCA | SNCA | SNCA | SNCA | SNCA | |
| 4 | 90816051 | 90875780 | MMRN1 | | | | | |
| 4 | 91048683 | 91703154 | CCSER1 | | | | | |
| 4 | 93183648 | 93198453 | LOC101929194 | | | | | |
| 4 | 93225549 | 94695706 | GRID2 | GRID2 | | GRID2 | GRID2 | GRID2 |

| | | | | | | | | |
|---|---|---|---|---|---|---|---|---|
| 4 | 94750077 | 94751142 | ATOH1 | | | | | |
| 4 | 95038970 | 95128707 | LOC101929210 | | | | | |
| 4 | 95128758 | 95212443 | SMARCAD1 | | | | | |
| 4 | 95219706 | 95264027 | HPGDS | | | | | |
| 4 | 95373007 | 95589378 | PDLIM5 | | | | | |
| 4 | 95664818 | 96079601 | BMPR1B | | | | | |
| 4 | 96083655 | 96470361 | UNC5C | | | UNC5C | | |
| 4 | 96761238 | 96762625 | PDHA2 | | | | | |
| 4 | 98288076 | 99064391 | STPG2 | | | | | |
| 4 | 99182526 | 99365012 | RAP1GDS1 | | | | | |
| 4 | 99391517 | 99579812 | TSPAN5 | | TSPAN5 | | | |
| 4 | 99799606 | 99851786 | EIF4E | | | | | |
| 4 | 99916787 | 99983960 | METAP1 | | | | | |
| 4 | 99918537 | 99918611 | MIR3684 | | | | | |
| 4 | 99992129 | 100009939 | ADH5 | | | | | |
| 4 | 100010007 | 100222513 | LOC100507053 | | | | | |
| 4 | 100044807 | 100065449 | ADH4 | | | | | |
| 4 | 100081750 | 100082804 | PCNAP1 | | | | | |
| 4 | 100123794 | 100140403 | ADH6 | | | | | |
| 4 | 100197522 | 100212185 | ADH1A | | | | | |
| 4 | 100227543 | 100242599 | ADH1B | | | | | |
| 4 | 100257648 | 100274202 | ADH1C | | | | | |
| 4 | 100333417 | 100356667 | ADH7 | | | | | |
| 4 | 100432160 | 100463460 | C4orf17 | | | | | |
| 4 | 100467863 | 100485214 | TRMT10A | | | | | |
| 4 | 100485234 | 100545154 | MTTP | | | | | |
| 4 | 100737980 | 100791346 | DAPP1 | | | | | |
| 4 | 100799494 | 100815703 | LAMTOR3 | | | | | |
| 4 | 100817406 | 100867883 | DNAJB14 | | | | | |
| 4 | 100869243 | 100871512 | H2AFZ | | | | | |
| 4 | 100871650 | 100958862 | LOC256880 | | | | | |
| 4 | 101107026 | 101111655 | DDIT4L | | | | | |
| 4 | 101111189 | 101136662 | LOC101929353 | | | | | |
| 4 | 101316497 | 101439250 | EMCN | | | | | |
| 4 | 101581435 | 101596270 | LINC01216 | | | | | |
| 4 | 101944586 | 102268628 | PPP3CA | | | | | |
| 4 | 102161951 | 102162029 | MIR8066 | | | | | |
| 4 | 102251458 | 102251571 | MIR1255A | | | | | |
| 4 | 102268933 | 102270040 | FLJ20021 | | | | | |
| 4 | 102711763 | 102995969 | BANK1 | | | | | |
| 4 | 103172197 | 103266655 | SLC39A8 | | | | | |
| 4 | 103422485 | 103538459 | NFKB1 | | | | | |
| 4 | 103552642 | 103682151 | MANBA | | | | | |
| 4 | 103698193 | 103720732 | LOC102723704 | | | | | |
| 4 | 103715539 | 103790050 | UBE2D3 | | | UBE2D3 | | |
| 4 | 103749211 | 103765263 | LOC105377348 | | | | | |
| 4 | 103790134 | 103813963 | CISD2 | | | | | |
| 4 | 103806204 | 103940896 | SLC9B1 | | | | | |
| 4 | 103946647 | 103998528 | SLC9B2 | | | | | |
| 4 | 103998781 | 104021024 | BDH2 | | | | | |
| 4 | 104026962 | 104119566 | CENPE | | | | | |
| 4 | 104346198 | 104360885 | LOC101929448 | | | | | |
| 4 | 104510624 | 104640973 | TACR3 | | | | TACR3 | |
| 4 | 105389462 | 105416058 | CXXC4 | | | | | |
| 4 | 105412121 | 105618749 | LOC101929468 | | | | | |
| 4 | 106067031 | 106274038 | TET2 | | | | | |
| 4 | 106290233 | 106395227 | PPA2 | | | PPA2 | | |
| 4 | 106473776 | 106602070 | ARHGEF38 | | | | | |
| 4 | 106482747 | 106491392 | ARHGEF38-IT1 | | | | | |
| 4 | 106603784 | 106629881 | INTS12 | | | | | |
| 4 | 106629940 | 106768882 | GSTCD | | | | | |
| 4 | 106736301 | 106748329 | LOC101929529 | | | | | |
| 4 | 106816596 | 106892828 | NPNT | | | | | |
| 4 | 106924473 | 106943635 | LOC101929577 | | | | | |
| 4 | 106965473 | 107237861 | TBCK | | | | | |
| 4 | 107236766 | 107270381 | AIMP1 | | | | | |

| | | | | | | | | |
|---|---|---|---|---|---|---|---|---|
| 4 | 107279339 | 107288592 | GIMD1 | | | | | |
| 4 | 107842958 | 107957453 | DKK2 | | | | | |
| 4 | 108534821 | 108641419 | PAPSS1 | | | | | |
| 4 | 108745720 | 108836204 | SGMS2 | | | | | |
| 4 | 108784634 | 108853275 | LOC101929595 | | | | | |
| 4 | 108814419 | 108836204 | SGMS2 | | | | | |
| 4 | 108852716 | 108874613 | CYP2U1 | | | | | |
| 4 | 108910869 | 108956331 | HADH | | | | | |
| 4 | 108968700 | 109097586 | LEF1 | | | | | |
| 4 | 109459345 | 109541613 | RPL34 | | | | | |
| 4 | 109478388 | 109481548 | LOC101929621 | | | | | |
| 4 | 109541713 | 109546526 | RPL34 | | | | | |
| 4 | 109571740 | 109588978 | OSTC | | | | | |
| 4 | 109663201 | 109684235 | ETNPPL | | | | | |
| 4 | 109731876 | 110223799 | COL25A1 | | | | | |
| 4 | 110351118 | 110461615 | SEC24B | | | | | |
| 4 | 110481354 | 110608872 | CCDC109B | | | | | |
| 4 | 110609784 | 110624629 | CASP6 | | | | | |
| 4 | 110631144 | 110651242 | PLA2G12A | | | | | |
| 4 | 110661847 | 110723381 | CFI | | | | | |
| 4 | 110736665 | 110745893 | GAR1 | | | | | |
| 4 | 110749149 | 110765861 | RRH | | | | | |
| 4 | 110769339 | 110793471 | LRIT3 | | | | | |
| 4 | 110834039 | 110934235 | EGF | EGF | | | | |
| 4 | 110970228 | 111119820 | ELOVL6 | | | | | |
| 4 | 111397228 | 111484493 | ENPEP | | | | | |
| 4 | 111538579 | 111563279 | PITX2 | | | PITX2 | | |
| 4 | 113066552 | 113110237 | C4orf32 | | | | | |
| 4 | 113152894 | 113191211 | AP1AR | | | | | |
| 4 | 113196781 | 113207059 | TIFA | | | | | |
| 4 | 113218498 | 113363764 | ALPK1 | | | | | |
| 4 | 113434671 | 113437328 | NEUROG2 | | | NEUROG2 | | |
| 4 | 113460488 | 113558151 | ZGRF1 | | | | | |
| 4 | 113558119 | 113578748 | LARP7 | | | | | |
| 4 | 113569029 | 113569097 | MIR367 | | | | | |
| 4 | 113569159 | 113569227 | MIR302D | | | | | |
| 4 | 113569338 | 113569407 | MIR302A | | | | | |
| 4 | 113569518 | 113569586 | MIR302C | | | | | |
| 4 | 113569640 | 113569713 | MIR302B | | | | | |
| 4 | 113739238 | 114304896 | ANK2 | | ANK2 | | | |
| 4 | 114028018 | 114028111 | MIR1243 | | | | | |
| 4 | 114073437 | 114073518 | MIR8082 | | | | | |
| 4 | 114372187 | 114683083 | CAMK2D | | | CAMK2D | | |
| 4 | 114821439 | 114900878 | ARSJ | | | | | |
| 4 | 115519610 | 115598202 | UGT8 | | | | | |
| 4 | 115577914 | 115578010 | MIR577 | | | | | |
| 4 | 115748928 | 116035032 | NDST4 | | | | | |
| 4 | 117220880 | 117220924 | MIR1973 | | | | | |
| 4 | 118004709 | 118006736 | TRAM1L1 | | | | | |
| 4 | 118349553 | 118610258 | LINC01378 | | | | | |
| 4 | 118955499 | 119179789 | NDST3 | | | | | |
| 4 | 119199916 | 119200978 | SNHG8 | | | | | |
| 4 | 119200344 | 119200475 | SNORA24 | | | | | |
| 4 | 119201192 | 119273922 | PRSS12 | | | PRSS12 | PRSS12 | |
| 4 | 119437494 | 119475359 | CEP170P1 | | | | | |
| 4 | 119512898 | 119556180 | LOC729218 | | | | | |
| 4 | 119585242 | 119606496 | LOC101929741 | | | | | |
| 4 | 119606524 | 119633425 | METTL14 | | | | | |
| 4 | 119643977 | 119757351 | SEC24D | | | | | |
| 4 | 119771842 | 119982402 | SYNPO2 | | | SYNPO2 | | |
| 4 | 120056938 | 120108944 | MYOZ2 | | | | | |
| 4 | 120113927 | 120133799 | LOC101929762 | | | | | |
| 4 | 120133781 | 120216673 | USP53 | | | | | |
| 4 | 120217573 | 120225600 | C4orf3 | | | | | |
| 4 | 120238404 | 120243316 | FABP2 | | | | | |
| 4 | 120326677 | 120331815 | LINC01061 | | | | | |

| | | | | | | | | | |
|---|---|---|---|---|---|---|---|---|---|
| 4 | 120370724 | 120375783 | GTF2IP12 | | | | | | |
| 4 | 120375937 | 120420747 | LOC645513 | | | | | | |
| 4 | 120415549 | 120549981 | PDE5A | | | | | | |
| 4 | 120720260 | 120725670 | LINC01365 | | | | | | |
| 4 | 120860694 | 120885448 | LOC100996694 | | | | | | |
| 4 | 120980578 | 120988013 | MAD2L1 | | | | | | |
| 4 | 121613067 | 121844021 | PRDM5 | | | | | | |
| 4 | 121956781 | 121993673 | NDNF | | | | | | |
| 4 | 122052563 | 122148621 | TNIP3 | | | | | | |
| 4 | 122249796 | 122302181 | QRFPR | | | | | | |
| 4 | 122589151 | 122618147 | ANXA5 | | | | | | |
| 4 | 122680087 | 122686555 | TMEM155 | | | | | | |
| 4 | 122685739 | 122687963 | PP12613 | | | | | | |
| 4 | 122722471 | 122738176 | EXOSC9 | | | | | | |
| 4 | 122737598 | 122745088 | CCNA2 | | | | | | |
| 4 | 122745483 | 122791652 | BBS7 | | BBS7 | | | | |
| 4 | 122800182 | 122872909 | TRPC3 | | TRPC3 | TRPC3 | | | |
| 4 | 123091757 | 123283914 | KIAA1109 | | | | | | |
| 4 | 123300120 | 123350947 | ADAD1 | | | | | | |
| 4 | 123372625 | 123377650 | IL2 | | | | | | |
| 4 | 123533782 | 123610311 | IL21 | | | | | | |
| 4 | 123651343 | 123653613 | CETN4P | | | | | | |
| 4 | 123653856 | 123666098 | BBS12 | | | | | | |
| 4 | 123747862 | 123819390 | FGF2 | | FGF2 | | | | |
| 4 | 123813798 | 123844159 | NUDT6 | | | | | | |
| 4 | 123844224 | 124240604 | SPATA5 | | | | | | |
| 4 | 124317949 | 124324915 | SPRY1 | | | | | | |
| 4 | 124573939 | 124786730 | LINC01091 | | | | | | |
| 4 | 125421096 | 125479589 | LOC101927087 | | | | | | |
| 4 | 125585203 | 125633887 | ANKRD50 | | | | | | |
| 4 | 126237566 | 126414087 | FAT4 | | | | | | |
| 4 | 126428413 | 126428462 | MIR2054 | | | | | | |
| 4 | 128554086 | 128637934 | INTU | | | | | | |
| 4 | 128651532 | 128695454 | SLC25A31 | | | | | | |
| 4 | 128702975 | 128755228 | HSPA4L | | | | | | |
| 4 | 128802015 | 128820377 | PLK4 | | | | | | |
| 4 | 128838959 | 128887139 | MFSD8 | | | | | | |
| 4 | 128886410 | 128952455 | ABHD18 | | | | | | |
| 4 | 128982420 | 129132298 | LARP1B | | | | | | |
| 4 | 129190391 | 129209984 | PGRMC2 | | | | | | |
| 4 | 129349170 | 129440551 | LOC100507487 | | | | | | |
| 4 | 129730777 | 129796379 | JADE1 | | | | | | |
| 4 | 129805147 | 130014762 | SCLT1 | | | | | | |
| 4 | 130014828 | 130033843 | C4orf33 | | | | | | |
| 4 | 130645325 | 130692633 | LOC101927282 | | | | | | |
| 4 | 132685992 | 132712637 | LOC101927305 | | | | | | |
| 4 | 133512243 | 133599655 | LINC01256 | | | | | | |
| 4 | 134015015 | 134070271 | LOC101927359 | | | | | | |
| 4 | 134070444 | 134115765 | PCDH10 | | | | | | |
| 4 | 135117488 | 135122903 | PABPC4L | | | | | | |
| 4 | 136788137 | 136834835 | LINC00613 | | | | | | |
| 4 | 138114909 | 138121102 | LOC729307 | | | | | | |
| 4 | 138440072 | 138453652 | PCDH18 | | | | | | |
| 4 | 138948576 | 139051839 | LINC00616 | | | | | | |
| 4 | 139010167 | 139163503 | SLC7A11 | | | | | | |
| 4 | 139230864 | 139345498 | LINC00499 | | | | | | |
| 4 | 139741110 | 139933800 | LOC105377448 | | | | | | |
| 4 | 139936912 | 139967093 | NOCT | | | | | | |
| 4 | 139978870 | 140060630 | ELF2 | | | ELF2 | | | |
| 4 | 140187316 | 140201492 | MGARP | | | | | | |
| 4 | 140211070 | 140223705 | NDUFC1 | | | | | | |
| 4 | 140222675 | 140311935 | NAA15 | | | | | | |
| 4 | 140374960 | 140397069 | RAB33B | | | | | | |
| 4 | 140417088 | 140477923 | SETD7 | | | | | | |
| 4 | 140586921 | 140661899 | MGST2 | | | | | | |
| 4 | 140637545 | 141075233 | MAML3 | | | | | | |

| | | | | | | | | |
|---|---|---|---|---|---|---|---|---|
| 4 | 141178439 | 141303710 | SCOC | | | | | |
| 4 | 141309606 | 141348815 | CLGN | | | | | |
| 4 | 141364528 | 141419531 | MGAT4D | | | | | |
| 4 | 141445311 | 141474924 | ELMOD2 | | | | | |
| 4 | 141481049 | 141489959 | UCP1 | | | | | |
| 4 | 141541935 | 141677471 | TBC1D9 | | | | | |
| 4 | 141562344 | 141564234 | TNRC18P1 | | | | | |
| 4 | 141786724 | 142054616 | RNF150 | | | | | |
| 4 | 142142040 | 142155850 | ZNF330 | | | | | |
| 4 | 142242277 | 142253771 | LOC100507639 | | | | | |
| 4 | 142557748 | 142655140 | IL15 | | | | | |
| 4 | 142949181 | 143767604 | INPP4B | | | | | |
| 4 | 144106069 | 144145027 | USP38 | | | | | |
| 4 | 144257982 | 144395718 | GAB1 | | | | | |
| 4 | 144264612 | 144264688 | MIR3139 | | | | | |
| 4 | 144434615 | 144435990 | SMARCA5 | | | | | |
| 4 | 144480624 | 144482613 | GUSBP5 | | | | | |
| 4 | 144498560 | 144621828 | FREM3 | | | | | |
| 4 | 144738946 | 144750501 | LOC105377458 | | | | | |
| 4 | 144792018 | 144826716 | GYPE | | | | | |
| 4 | 144833483 | 144903605 | LOC101927636 | | | | | |
| 4 | 144917256 | 144940498 | GYPB | | | | | |
| 4 | 145030455 | 145061939 | GYPA | | | | | |
| 4 | 145564067 | 145659881 | HHIP | | HHIP | | | |
| 4 | 145915726 | 146019693 | ANAPC10 | | | | | |
| 4 | 146019155 | 146050676 | ABCE1 | | | | | |
| 4 | 146054801 | 146095896 | OTUD4 | | | | | |
| 4 | 146402950 | 146438346 | SMAD1 | | | SMAD1 | | |
| 4 | 146540539 | 146581187 | MMAA | | | | | |
| 4 | 146601355 | 146653948 | C4orf51 | | | | | |
| 4 | 146678778 | 146860112 | ZNF827 | | | | | |
| 4 | 147030606 | 147043065 | LINC01095 | | | | | |
| 4 | 147096834 | 147111213 | LSM6 | | | | | |
| 4 | 147175139 | 147443123 | SLC10A7 | | | | | |
| 4 | 147329734 | 147329840 | MIR7849 | | | | | |
| 4 | 147362817 | 147443123 | SLC10A7 | | | | | |
| 4 | 147560044 | 147563623 | POU4F2 | | | POU4F2 | | |
| 4 | 147627789 | 147867038 | TTC29 | | | | | |
| 4 | 148402068 | 148466106 | EDNRA | | | EDNRA | | |
| 4 | 148538538 | 148556672 | TMEM184C | | | | | |
| 4 | 148558935 | 148605381 | PRMT9 | | | | | |
| 4 | 148653452 | 148993927 | ARHGAP10 | | | | | |
| 4 | 148703745 | 148703819 | MIR4799 | | | | | |
| 4 | 148999914 | 149363672 | NR3C2 | | | NR3C2 | | |
| 4 | 149862097 | 149863530 | LOC105377480 | | | | | |
| 4 | 150075446 | 150199274 | LOC101927849 | | | | | |
| 4 | 150999425 | 151178608 | DCLK2 | | | DCLK2 | | |
| 4 | 151185810 | 151936649 | LRBA | | | | | |
| 4 | 151503076 | 151505845 | MAB21L2 | | MAB21L2 | | | |
| 4 | 152020724 | 152025804 | RPS3A | | | | | |
| 4 | 152024978 | 152025043 | SNORD73A | | | | | |
| 4 | 152041432 | 152149182 | SH3D19 | | | | | |
| 4 | 152198324 | 152212605 | PRSS48 | | | | | |
| 4 | 152330397 | 152584784 | FAM160A1 | | | | | |
| 4 | 152591808 | 152682175 | GATB | | | | | |
| 4 | 153021904 | 153025868 | LOC100996286 | | | | | |
| 4 | 153242409 | 153456185 | FBXW7 | | | | | |
| 4 | 153258806 | 153259248 | DEAR | | | | | |
| 4 | 153332311 | 153456393 | FBXW7 | | | | | |
| 4 | 153410478 | 153410568 | MIR3140 | | | | | |
| 4 | 153457415 | 153460415 | DKFZP434I0714 | | | | | |
| 4 | 153457579 | 153457668 | MIR4453 | | | | | |
| 4 | 153547265 | 153601317 | TMEM154 | | | | | |
| 4 | 153690505 | 153700916 | TIGD4 | | | | | |
| 4 | 153701088 | 153833063 | ARFIP1 | | | | | |
| 4 | 153864134 | 153900848 | FHDC1 | | | | | |

| | | | | | | | | |
|---|---|---|---|---|---|---|---|---|
| 4 | 154074269 | 154197427 | TRIM2 | | TRIM2 | | | |
| 4 | 154228620 | 154229963 | ANXA2P1 | | | | | |
| 4 | 154265800 | 154336247 | MND1 | | | | | |
| 4 | 154387497 | 154557862 | KIAA0922 | | | | | |
| 4 | 154561319 | 154602332 | LOC100419170 | | | | | |
| 4 | 154605403 | 154627412 | TLR2 | | | | | |
| 4 | 154631311 | 154681387 | RNF175 | | RNF175 | | | |
| 4 | 154701741 | 154710228 | SFRP2 | | | | | |
| 4 | 155155526 | 155412877 | DCHS2 | | | | | |
| 4 | 155456148 | 155471585 | PLRG1 | | | | | |
| 4 | 155484131 | 155493915 | FGB | | | | | |
| 4 | 155504277 | 155511918 | FGA | | | FGA | | |
| 4 | 155525285 | 155533902 | FGG | | | | | |
| 4 | 155661992 | 155674271 | LRAT | | LRAT | | | |
| 4 | 155702423 | 155749965 | RBM46 | | | | | |
| 4 | 156129780 | 156138230 | NPY2R | | | NPY2R | | |
| 4 | 156263811 | 156298122 | MAP9 | | | | | |
| 4 | 156275367 | 156281613 | LOC102724776 | | | | | |
| 4 | 156587861 | 156658214 | GUCY1A3 | | | | | |
| 4 | 156680124 | 156728794 | GUCY1B3 | | | | | |
| 4 | 156750880 | 156787425 | ASIC5 | | | | | |
| 4 | 156824844 | 156841558 | TDO2 | | | | | |
| 4 | 156845269 | 156875048 | CTSO | | | | | |
| 4 | 157682762 | 157892546 | PDGFC | | | | | |
| 4 | 157997276 | 158093242 | GLRB | | GLRB | GLRB | GLRB | GLRB |
| 4 | 158141735 | 158287226 | GRIA2 | | | | | |
| 4 | 158493641 | 158497303 | LOC340017 | | | | | |
| 4 | 159045731 | 159094202 | FAM198B | | | | | |
| 4 | 159131400 | 159176439 | TMEM144 | | | | | |
| 4 | 159442865 | 159574521 | RXFP1 | | | | | |
| 4 | 159587826 | 159593407 | C4orf46 | | | | | |
| 4 | 159593252 | 159629865 | ETFDH | | | ETFDH | | |
| 4 | 159630278 | 159644552 | PPID | | | | | |
| 4 | 159690181 | 159827954 | FNIP2 | | | | | |
| 4 | 159814683 | 159956333 | C4orf45 | | | | | |
| 4 | 160049953 | 160050043 | MIR3688 | | | | | |
| 4 | 160188997 | 160281301 | RAPGEF2 | | | RAPGEF2 | | |
| 4 | 162305043 | 163085186 | FSTL5 | | | | | |
| 4 | 162943884 | 162968702 | LOC101928052 | | | | | |
| 4 | 164014725 | 164014780 | MIR4454 | | | | | |
| 4 | 164047859 | 164088073 | NAF1 | | | | | |
| 4 | 164245116 | 164253947 | NPY1R | | | NPY1R | | |
| 4 | 164264998 | 164273086 | NPY5R | | | NPY5R | | |
| 4 | 164392246 | 164395047 | TKTL2 | | | | | |
| 4 | 164415672 | 164441691 | TMA16 | | | | | |
| 4 | 164445449 | 165304407 | MARCH1 | | | | | |
| 4 | 165118158 | 165118863 | ANP32C | | | | | |
| 4 | 165426541 | 165630646 | MIR5684 | | | | | |
| 4 | 165675282 | 165724947 | LINC01207 | | | | | |
| 4 | 165798139 | 165818679 | APELA | | | | | |
| 4 | 165875597 | 165898818 | TRIM61 | | | | | |
| 4 | 165878099 | 165880273 | FAM218A | | | | | |
| 4 | 165953150 | 165962896 | TRIM60 | | | | | |
| 4 | 165997229 | 166034024 | TMEM192 | | | | | |
| 4 | 166128769 | 166244308 | KLHL2 | | | KLHL2 | | |
| 4 | 166198943 | 166201175 | GK3P | | | | | |
| 4 | 166248817 | 166264314 | MSMO1 | | | | | |
| 4 | 166300093 | 166419699 | CPE | | | | | |
| 4 | 166307393 | 166307489 | MIR578 | | | | | |
| 4 | 166605790 | 166683930 | LINC01179 | | | | | |
| 4 | 166651930 | 166664223 | LOC101928131 | | | | | |
| 4 | 166794409 | 167025609 | TLL1 | | | | | |
| 4 | 167654535 | 168155741 | SPOCK3 | | SPOCK3 | | | |
| 4 | 169013687 | 169108893 | ANXA10 | | | | | |
| 4 | 169137441 | 169239958 | DDX60 | | | | | |
| 4 | 169277885 | 169401665 | DDX60L | | | | | |

| | | | | | | | | |
|---|---|---|---|---|---|---|---|---|
| 4 | 169418216 | 169849608 | PALLD | | | | | |
| 4 | 169908741 | 169931468 | CBR4 | | | | | |
| 4 | 170015406 | 170192249 | SH3RF1 | | | | | |
| 4 | 170314420 | 170533778 | NEK1 | | NEK1 | | | |
| 4 | 170541671 | 170644338 | CLCN3 | | CLCN3 | CLCN3 | | |
| 4 | 170650618 | 170679093 | C4orf27 | | | | | |
| 4 | 170838911 | 170897053 | LOC100506085 | | | | | |
| 4 | 170907747 | 170954179 | MFAP3L | | | | | |
| 4 | 170981372 | 171011538 | AADAT | | | AADAT | | |
| 4 | 171147741 | 171204873 | LINC01612 | | | | | |
| 4 | 171663619 | 171664886 | LOC100506107 | | | | | |
| 4 | 171961752 | 171980311 | LOC100506122 | | | | | |
| 4 | 172107334 | 172107443 | MIR6082 | | | | | |
| 4 | 172733288 | 172734758 | LOC441052 | | | | | |
| 4 | 172734574 | 173961558 | GALNTL6 | | | | | |
| 4 | 173551082 | 173648029 | LOC101928314 | | | | | |
| 4 | 174053083 | 174090803 | LOC101930370 | | | | | |
| 4 | 174089903 | 174245118 | GALNT7 | | | | | |
| 4 | 174252526 | 174255595 | HMGB2 | | | | | |
| 4 | 174292092 | 174298683 | SAP30 | | | | | |
| 4 | 174309298 | 174320617 | SCRG1 | | | | | |
| 4 | 174447651 | 174462981 | HAND2 | | | | | |
| 4 | 175015810 | 175141549 | LOC101928509 | | | | | |
| 4 | 175157809 | 175205402 | FBXO8 | | | | | |
| 4 | 175204827 | 175254531 | CEP44 | | | | | |
| 4 | 175344945 | 175345015 | MIR4276 | | | | | |
| 4 | 175411327 | 175444049 | HPGD | | | | | |
| 4 | 175563161 | 175750466 | GLRA3 | | | GLRA3 | | |
| 4 | 175752878 | 175797115 | LOC101928551 | | | | | |
| 4 | 175839508 | 175899331 | ADAM29 | | | | | |
| 4 | 176554087 | 176923842 | GPM6A | | | GPM6A | | |
| 4 | 176711457 | 176733340 | LOC101928590 | | | | | |
| 4 | 176986984 | 177103979 | WDR17 | | | | | |
| 4 | 177105724 | 177116822 | SPATA4 | | | | | |
| 4 | 177134825 | 177190373 | ASB5 | | | | | |
| 4 | 177241089 | 177253396 | SPCS3 | | | | | |
| 4 | 177604684 | 177713899 | VEGFC | | | VEGFC | | |
| 4 | 178230990 | 178284092 | NEIL3 | | | | | |
| 4 | 178351928 | 178363657 | AGA | | | | | |
| 4 | 178649910 | 178911904 | LINC01098 | | | | | |
| 4 | 178733199 | 178829090 | LINC01099 | | | | | |
| 4 | 181985242 | 182080302 | LINC00290 | | | | | |
| 4 | 183059812 | 183065668 | LOC90768 | | | | | |
| 4 | 183090445 | 183090531 | MIR1305 | | | | | |
| 4 | 183164583 | 183724177 | TENM3 | | TENM3 | | | |
| 4 | 183811243 | 183838630 | DCTD | | | | | |
| 4 | 183958817 | 183961272 | FAM92A1P2 | | | | | |
| 4 | 184018173 | 184161787 | WWC2 | | | | | |
| 4 | 184239219 | 184241927 | CLDN22 | | | | | |
| 4 | 184242916 | 184243579 | CLDN24 | | | | | |
| 4 | 184365743 | 184370218 | CDKN2AIP | | | | | |
| 4 | 184415889 | 184425668 | LOC389247 | | | | | |
| 4 | 184426202 | 184433581 | ING2 | | | | | |
| 4 | 184560787 | 184580372 | RWDD4 | | | | | |
| 4 | 184580419 | 184634747 | TRAPPC11 | | | | | |
| 4 | 184719174 | 184944683 | STOX2 | | | | | |
| 4 | 185009858 | 185139114 | ENPP6 | | | | | |
| 4 | 185262183 | 185275130 | LOC728175 | | | | | |
| 4 | 185286713 | 185303460 | LOC102723766 | | | | | |
| 4 | 185308875 | 185395726 | IRF2 | | | | | |
| 4 | 185505246 | 185546026 | LVCAT8 | | | | | |
| 4 | 185548849 | 185570629 | CASP3 | | | | | |
| 4 | 185570766 | 185616113 | PRIMPOL | | | | | |
| 4 | 185615218 | 185655286 | CENPU | | | | | |
| 4 | 185676748 | 185747268 | ACSL1 | | | ACSL1 | | |
| 4 | 185719450 | 185720200 | SLED1 | | | | | |

| | | | | | | | | |
|---|---|---|---|---|---|---|---|---|
| 4 | 185764449 | 185776806 | MIR3945HG | | | | | |
| 4 | 185772166 | 185772264 | MIR3945 | | | | | |
| 4 | 185814153 | 185820615 | LINC01093 | | | | | |
| 4 | 185859536 | 185859594 | MIR4455 | | | | | |
| 4 | 185939994 | 185941958 | HELT | | | | | |
| 4 | 186064416 | 186071538 | SLC25A4 | | | SLC25A4 | | |
| 4 | 186080815 | 186125182 | CFAP97 | | | | | |
| 4 | 186125390 | 186285125 | SNX25 | | | | | |
| 4 | 186285031 | 186300152 | LRP2BP | | | | | |
| 4 | 186317839 | 186321399 | ANKRD37 | | | | | |
| 4 | 186320693 | 186347139 | UFSP2 | | | | | |
| 4 | 186350544 | 186370821 | C4orf47 | | | | | |
| 4 | 186366337 | 186392913 | CCDC110 | | | | | |
| 4 | 186392584 | 186393416 | LOC105377590 | | | | | |
| 4 | 186421814 | 186456712 | PDLIM3 | | | | | |
| 4 | 186506597 | 186877870 | SORBS2 | | | | | |
| 4 | 186990308 | 187006252 | TLR3 | | | | | |
| 4 | 187065994 | 187093817 | FAM149A | | | | | |
| 4 | 187110185 | 187112644 | FLJ38576 | | | | | |
| 4 | 187112673 | 187134617 | CYP4V2 | | | | | |
| 4 | 187148624 | 187179628 | KLKB1 | | KLKB1 | | | |
| 4 | 187187117 | 187422212 | F11 | | | | | |
| 4 | 187454808 | 187476721 | MTNR1A | | | MTNR1A | | |
| 4 | 187508936 | 187644987 | FAT1 | | | | | |
| 4 | 188225236 | 188426767 | LOC339975 | | | | | |
| 4 | 188454032 | 188593795 | LOC100506272 | | | | | |
| 4 | 188916924 | 188926203 | ZFP42 | | | | | |
| 4 | 189012425 | 189026741 | TRIML2 | | | | | |
| 4 | 189060597 | 189068649 | TRIML1 | | | | | |
| 4 | 189376731 | 189523062 | LINC01060 | | | | | |
| 4 | 190580759 | 190582640 | LINC01262 | | | | | |
| 4 | 190802669 | 190806022 | LINC01596 | | | | | |
| 4 | 190861973 | 190884359 | FRG1 | | | | | |
| 4 | 190945522 | 190948412 | FRG2 | | | | | |
| 4 | 190985656 | 190989019 | DBET | | | | | |
| 5 | 140372 | 190087 | PLEKHG4B | | | | | |
| 5 | 191625 | 195468 | LRRC14B | | | | | |
| 5 | 204874 | 218297 | CCDC127 | | | | | |
| 5 | 218337 | 257197 | SDHA | | | SDHA | | |
| 5 | 269670 | 271639 | HRAT5 | | | | | |
| 5 | 271735 | 315089 | PDCD6 | | | | | |
| 5 | 304290 | 438405 | AHRR | | | | | |
| 5 | 441950 | 467409 | EXOC3 | | | | | |
| 5 | 470624 | 473080 | PP7080 | | | | | |
| 5 | 473333 | 524549 | SLC9A3 | | | | | |
| 5 | 473350 | 481006 | LOC100288152 | | | | | |
| 5 | 535954 | 535997 | MIR4456 | | | | | |
| 5 | 602283 | 612325 | LOC100996325 | | | | | |
| 5 | 612404 | 653666 | CEP72 | | | | | |
| 5 | 659976 | 693510 | TPPP | | | | | |
| 5 | 795719 | 851101 | ZDHHC11 | | | | | |
| 5 | 863849 | 892939 | BRD9 | | | | | |
| 5 | 892968 | 918164 | TRIP13 | | | | | |
| 5 | 987291 | 997455 | LOC100506688 | | | | | |
| 5 | 1009076 | 1038927 | NKD2 | | | | | |
| 5 | 1050488 | 1112172 | SLC12A7 | | | SLC12A7 | | |
| 5 | 1063010 | 1063089 | MIR4635 | | | | | |
| 5 | 1173210 | 1178720 | CTD-3080P12.3 | | | | | |
| 5 | 1201709 | 1225230 | SLC6A19 | | | | | |
| 5 | 1225469 | 1246304 | SLC6A18 | | | SLC6A18 | | |
| 5 | 1253286 | 1295162 | TERT | | | TERT | | |
| 5 | 1309424 | 1309492 | MIR4457 | | | | | |
| 5 | 1317858 | 1345185 | CLPTM1L | | | | | |
| 5 | 1363696 | 1380188 | LINC01511 | | | | | |
| 5 | 1392904 | 1445543 | SLC6A3 | | SLC6A3 | | SLC6A3 | |
| 5 | 1461541 | 1524076 | LPCAT1 | | | | | |

| | | | | | | | | |
|---|---|---|---|---|---|---|---|---|
| 5 | 1510876 | 1510971 | MIR6075 | | | | | |
| 5 | 1572072 | 1594646 | SDHAP3 | | | | | |
| 5 | 1597671 | 1634120 | LOC728613 | | | | | |
| 5 | 1708899 | 1708983 | MIR4277 | | | | | |
| 5 | 1798498 | 1799956 | MRPL36 | | | | | |
| 5 | 1801495 | 1816167 | NDUFS6 | | | | | |
| 5 | 1856083 | 1856682 | LOC101929034 | | | | | |
| 5 | 1877540 | 1887293 | IRX4 | | | | | |
| 5 | 1887444 | 1900604 | CTD-2194D22.4 | | | | | |
| 5 | 2303833 | 2312315 | LOC100506858 | | | | | |
| 5 | 2746278 | 2751769 | IRX2 | | | | | |
| 5 | 2752244 | 2755511 | C5orf38 | | | | | |
| 5 | 3177948 | 3181346 | LINC01377 | | | | | |
| 5 | 3417265 | 3536208 | LINC01019 | | | | | |
| 5 | 3496485 | 3504118 | LINC01017 | | | | | |
| 5 | 3596167 | 3601517 | IRX1 | | | | | |
| 5 | 4773593 | 4774978 | LOC101929153 | | | | | |
| 5 | 5034471 | 5070115 | LINC01020 | | | | | |
| 5 | 5069304 | 5078424 | LOC105374631 | | | | | |
| 5 | 5132197 | 5140167 | CTD-2297D10.2 | | | | | |
| 5 | 5140442 | 5320412 | ADAMTS16 | | | | | |
| 5 | 5422785 | 5490347 | ICE1 | | | | | |
| 5 | 6310553 | 6337405 | FLJ33360 | | | | | |
| 5 | 6372038 | 6378639 | MED10 | | | | | |
| 5 | 6448735 | 6496834 | UBE2QL1 | | | | | |
| 5 | 6582248 | 6588613 | LINC01018 | | | | | |
| 5 | 6599351 | 6633473 | NSUN2 | | | | | |
| 5 | 6633499 | 6669675 | SRD5A1 | | | SRD5A1 | | |
| 5 | 6686437 | 6707824 | LOC100505625 | | | | | |
| 5 | 6714717 | 6757161 | PAPD7 | | | | | |
| 5 | 6827965 | 6828034 | MIR4278 | | | | | |
| 5 | 7269415 | 7269467 | MIR4454 | | | | | |
| 5 | 7301442 | 7306827 | LOC442132 | | | | | |
| 5 | 7347098 | 7348090 | LOC101929261 | | | | | |
| 5 | 7396342 | 7830194 | ADCY2 | | ADCY2 | | | |
| 5 | 7830490 | 7851603 | C5orf49 | | | | | |
| 5 | 7859271 | 7869150 | FASTKD3 | | | | | |
| 5 | 7869216 | 7901235 | MTRR | | | MTRR | | |
| 5 | 8333595 | 8457677 | LOC729506 | | | | | |
| 5 | 8457799 | 8463209 | MIR4458HG | | | | | |
| 5 | 8461037 | 8461112 | MIR4458 | | | | | |
| 5 | 8839843 | 8881636 | LOC101929284 | | | | | |
| 5 | 9035137 | 9546233 | SEMA5A | | | | | |
| 5 | 9053927 | 9054007 | MIR4636 | | | | | |
| 5 | 9511444 | 9518198 | CTD-2201E9.1 | | | | | |
| 5 | 9546311 | 9550409 | SNHG18 | | | | | |
| 5 | 9548938 | 9549026 | SNORD123 | | | | | |
| 5 | 9629108 | 9630463 | TAS2R1 | | | | | |
| 5 | 9641426 | 9903936 | LOC285692 | | | | | |
| 5 | 10225619 | 10250021 | FAM173B | | | | | |
| 5 | 10250032 | 10266524 | CCT5 | | | | | |
| 5 | 10277706 | 10308168 | CMBL | | | | | |
| 5 | 10353750 | 10440500 | MARCH6 | | | | | |
| 5 | 10440846 | 10465138 | ROPN1L | | | | | |
| 5 | 10478148 | 10478257 | MIR6131 | | | | | |
| 5 | 10493638 | 10502840 | LOC101929412 | | | | | |
| 5 | 10505101 | 10522196 | LOC389273 | | | | | |
| 5 | 10564434 | 10657928 | ANKRD33B | | | | | |
| 5 | 10679341 | 10761387 | DAP | | | | | |
| 5 | 10971953 | 11904155 | CTNND2 | CTNND2 | CTNND2 | | | |
| 5 | 12574968 | 12805295 | LINC01194 | | | | | |
| 5 | 12667970 | 12668022 | MIR4454 | | | | | |
| 5 | 13690436 | 13944589 | DNAH5 | | | DNAH5 | | |
| 5 | 14143810 | 14510313 | TRIO | | | TRIO | | |
| 5 | 14581890 | 14616287 | FAM105A | | | | | |
| 5 | 14652386 | 14652491 | SNORD141A | | | | | |

| | | | | | | | | |
|---|---|---|---|---|---|---|---|---|
| 5 | 14652386 | 14652491 | SNORD141B | | | | | |
| 5 | 14664782 | 14699842 | OTULIN | | | | | |
| 5 | 14704908 | 14871887 | ANKH | | ANKH | | | |
| 5 | 14712802 | 14716638 | LOC100130744 | | | | | |
| 5 | 14826037 | 14826121 | MIR4637 | | | | | |
| 5 | 15191754 | 15266669 | LOC101929454 | | | | | |
| 5 | 15500304 | 15939905 | FBXL7 | | | | | |
| 5 | 15602297 | 15615111 | CTD-2350J17.1 | | | | | |
| 5 | 15935290 | 15935369 | MIR887 | | | | | |
| 5 | 16067473 | 16179897 | MARCH11 | | | | | |
| 5 | 16373469 | 16441211 | LOC101929505 | | | | | |
| 5 | 16451627 | 16465894 | ZNF622 | | | | | |
| 5 | 16473146 | 16617167 | FAM134B | | FAM134B | | | |
| 5 | 16616034 | 16630078 | LOC101929524 | | | | | |
| 5 | 16662015 | 16936385 | MYO10 | | | | | |
| 5 | 17130136 | 17217531 | LOC285696 | | | | | |
| 5 | 17216931 | 17276954 | BASP1 | | | | | |
| 5 | 17379014 | 17387419 | LOC401177 | | | | | |
| 5 | 17404127 | 17441803 | LOC101929544 | | | | | |
| 5 | 17444118 | 17484055 | LOC102723526 | | | | | |
| 5 | 17807382 | 17930598 | LOC646241 | | | | | |
| 5 | 19473154 | 20575982 | CDH18 | | | | | |
| 5 | 21459588 | 21589481 | GUSBP1 | | | | | |
| 5 | 21750974 | 22853731 | CDH12 | | | CDH12 | | |
| 5 | 21884562 | 21884678 | SNORA105A | | | | | |
| 5 | 21884562 | 21884678 | SNORA105B | | | | | |
| 5 | 22142460 | 22152379 | PMCHL1 | | | | | |
| 5 | 23507717 | 23528193 | PRDM9 | | | | | |
| 5 | 23951456 | 24178374 | C5orf17 | | | | | |
| 5 | 24487208 | 24645087 | CDH10 | | | | | |
| 5 | 24835388 | 24840692 | LOC340107 | | | | | |
| 5 | 26880708 | 27038689 | CDH9 | | CDH9 | | | |
| 5 | 27472398 | 27496508 | LINC01021 | | | | | |
| 5 | 28286495 | 28287772 | LOC105374698 | | | | | |
| 5 | 28926976 | 28927420 | LSP1P3 | | | | | |
| 5 | 29065350 | 29073330 | LOC101929645 | | | | | |
| 5 | 29143609 | 29173082 | LOC101929660 | | | | | |
| 5 | 29380195 | 29396083 | LOC101929681 | | | | | |
| 5 | 29880666 | 29882210 | LOC105374704 | | | | | |
| 5 | 31193761 | 31329253 | CDH6 | | | | | |
| 5 | 31400601 | 31532282 | DROSHA | | | | | |
| 5 | 31532372 | 31555165 | C5orf22 | | | | | |
| 5 | 31639516 | 32111038 | PDZD2 | | PDZD2 | | | |
| 5 | 31936207 | 31936265 | MIR4279 | | | | | |
| 5 | 32124823 | 32174425 | GOLPH3 | | | | | |
| 5 | 32227112 | 32313114 | MTMR12 | | | | | |
| 5 | 32354455 | 32444844 | ZFR | | | | | |
| 5 | 32394483 | 32394581 | MIR579 | | | | | |
| 5 | 32585604 | 32604185 | SUB1 | | | | | |
| 5 | 32710742 | 32791830 | NPR3 | | | | | |
| 5 | 32947548 | 32962573 | LOC340113 | | | | | |
| 5 | 33440801 | 33468196 | TARS | | | | | |
| 5 | 33527286 | 33892124 | ADAMTS12 | | | | | |
| 5 | 33936490 | 33939023 | RXFP3 | | | | | |
| 5 | 33944720 | 33984780 | SLC45A2 | | | | | |
| 5 | 33987090 | 34008220 | AMACR | | | AMACR | | |
| 5 | 33987090 | 34124633 | C1QTNF3-AMACR | | | | | |
| 5 | 34017962 | 34043371 | C1QTNF3 | | | | | |
| 5 | 34656432 | 34832717 | RAI14 | | | | | |
| 5 | 34802422 | 34802472 | MIR7641 | | | | | |
| 5 | 34839268 | 34900606 | TTC23L | | | | | |
| 5 | 34905365 | 34915780 | RAD1 | | | | | |
| 5 | 34915819 | 34925787 | BRIX1 | | | | | |
| 5 | 34929697 | 34959069 | DNAJC21 | | | | | |
| 5 | 34998205 | 35048240 | AGXT2 | | | | | |
| 5 | 35048860 | 35118224 | PRLR | | | | | |

| | | | | | | | | |
|---|---|---|---|---|---|---|---|---|
| 5 | 35617988 | 35814713 | SPEF2 | | | | | |
| 5 | 35856976 | 35879705 | IL7R | | | IL7R | | |
| 5 | 35904397 | 35938881 | CAPSL | | | | | |
| 5 | 35938902 | 35940093 | LOC100506406 | | | | | |
| 5 | 35953190 | 36001130 | UGT3A1 | | | | | |
| 5 | 36035118 | 36067023 | UGT3A2 | | | | | |
| 5 | 36103413 | 36152015 | LMBRD2 | | | | | |
| 5 | 36147993 | 36148090 | MIR580 | | | | | |
| 5 | 36152144 | 36184142 | SKP2 | | | | | |
| 5 | 36192690 | 36242381 | NADK2 | | | | | |
| 5 | 36249103 | 36302011 | RANBP3L | | | | | |
| 5 | 36606456 | 36688436 | SLC1A3 | | SLC1A3 | SLC1A3 | SLC1A3 | SLC1A3 |
| 5 | 36871462 | 37065921 | NIPBL | | | NIPBL | | |
| 5 | 37106329 | 37249530 | C5orf42 | | | | | |
| 5 | 37249147 | 37250748 | LOC105374727 | | | | | |
| 5 | 37291734 | 37371228 | NUP155 | | | | | |
| 5 | 37379411 | 37752774 | WDR70 | | | | | |
| 5 | 37812778 | 37875900 | GDNF | | | GDNF | GDNF | |
| 5 | 37948592 | 37951156 | LOC105374729 | | | | | |
| 5 | 38148581 | 38153817 | LOC101929745 | | | | | |
| 5 | 38258510 | 38465582 | EGFLAM | | | | | |
| 5 | 38475064 | 38671318 | LIFR | | LIFR | | | |
| 5 | 38557603 | 38557663 | MIR3650 | | | | | |
| 5 | 38693314 | 38845931 | OSMR | | | | | |
| 5 | 38710468 | 38720375 | LINC01265 | | | | | |
| 5 | 38845959 | 38935743 | OSMR | | | | | |
| 5 | 38938022 | 39074510 | RICTOR | | | | | |
| 5 | 39105353 | 39270759 | FYB | | | | | |
| 5 | 39284377 | 39364655 | C9 | | | | | |
| 5 | 39371775 | 39425335 | DAB2 | | | | | |
| 5 | 39520532 | 39524810 | LOC101926940 | | | | | |
| 5 | 40052392 | 40053426 | LINC00603 | | | | | |
| 5 | 40680031 | 40693837 | PTGER4 | | | | | |
| 5 | 40711677 | 40756072 | TTC33 | | | | | |
| 5 | 40759480 | 40798297 | PRKAA1 | | | | | |
| 5 | 40825364 | 40829244 | LOC100506548 | | | | | |
| 5 | 40831429 | 40835387 | RPL37 | | | | | |
| 5 | 40832757 | 40832837 | SNORD72 | | | | | |
| 5 | 40841409 | 40855456 | CARD6 | | | | | |
| 5 | 40909598 | 40983042 | C7 | | | | | |
| 5 | 40998121 | 41071444 | MROH2B | | | | | |
| 5 | 41142247 | 41261540 | C6 | | | C6 | | |
| 5 | 41307047 | 41510730 | PLCXD3 | | | | | |
| 5 | 41730166 | 41872338 | OXCT1 | | | OXCT1 | | |
| 5 | 41904445 | 41921738 | C5orf51 | | | | | |
| 5 | 41925353 | 41941672 | FBXO4 | | | | | |
| 5 | 42155934 | 42175447 | LOC101926960 | | | | | |
| 5 | 42423876 | 42721980 | GHR | | | GHR | | |
| 5 | 42756919 | 42802539 | CCDC152 | | | | | |
| 5 | 42799981 | 42812024 | SEPP1 | | | | | |
| 5 | 42985500 | 42993435 | FLJ32255 | | | | | |
| 5 | 43014830 | 43018913 | LOC648987 | | | | | |
| 5 | 43039181 | 43040447 | ANXA2R | | | | | |
| 5 | 43042235 | 43045370 | LOC153684 | | | | | |
| 5 | 43065288 | 43067073 | LOC100132356 | | | | | |
| 5 | 43067124 | 43093660 | LOC100506639 | | | | | |
| 5 | 43121002 | 43176426 | ZNF131 | | | | | |
| 5 | 43192169 | 43280952 | NIM1K | | | | | |
| 5 | 43287571 | 43313614 | HMGCS1 | | | | | |
| 5 | 43376747 | 43412493 | CCL28 | | | | | |
| 5 | 43444353 | 43483992 | C5orf28 | | | | | |
| 5 | 43486802 | 43515273 | C5orf34 | | | | | |
| 5 | 43526369 | 43557521 | PAIP1 | | | | | |
| 5 | 43573286 | 43705668 | NNT | | | | | |
| 5 | 44305096 | 44414091 | FGF10 | | | | | |
| 5 | 44495245 | 44510384 | BRCAT107 | | | | | |

| | | | | | | | | | |
|---|---|---|---|---|---|---|---|---|---|
| 5 | 44744429 | 44808895 | BRCAT54 | | | | | | |
| 5 | 44809026 | 44815618 | MRPS30 | | | | | | |
| 5 | 45255051 | 45696220 | HCN1 | | HCN1 | HCN1 | | | |
| 5 | 49692030 | 49737234 | EMB | | | | | | |
| 5 | 49961732 | 50142356 | PARP8 | | | | | | |
| 5 | 50265050 | 50266021 | LOC100287592 | | | | | | |
| 5 | 50668570 | 50679166 | LOC642366 | | | | | | |
| 5 | 50678957 | 50690563 | ISL1 | | | | | | |
| 5 | 52083773 | 52098452 | PELO | | | | | | |
| 5 | 52084135 | 52249485 | ITGA1 | | | | | | |
| 5 | 52285155 | 52390609 | ITGA2 | | | | | | |
| 5 | 52391508 | 52405602 | MOCS2 | | | | | | |
| 5 | 52405671 | 52410956 | LOC257396 | | | | | | |
| 5 | 52776263 | 52782304 | FST | | | | | | |
| 5 | 52856462 | 52979171 | NDUFS4 | | | NDUFS4 | | | |
| 5 | 53180613 | 53606403 | ARL15 | | | | | | |
| 5 | 53247333 | 53247429 | MIR581 | | | | | | |
| 5 | 53371347 | 53371413 | MIR4459 | | | | | | |
| 5 | 53616773 | 53710955 | LINC01033 | | | | | | |
| 5 | 53751430 | 53752214 | HSPB3 | | | | | | |
| 5 | 53813588 | 53842416 | SNX18 | | | SNX18 | | | |
| 5 | 53955986 | 54040086 | LOC102467080 | | | | | | |
| 5 | 54273694 | 54281414 | ESM1 | | | | | | |
| 5 | 54317126 | 54319997 | LOC102467081 | | | | | | |
| 5 | 54320106 | 54329960 | GZMK | | | | | | |
| 5 | 54398473 | 54406080 | GZMA | | | | | | |
| 5 | 54408798 | 54469005 | CDC20B | | | | | | |
| 5 | 54455945 | 54463129 | GPX8 | | | | | | |
| 5 | 54466359 | 54466450 | MIR449A | | | | | | |
| 5 | 54466473 | 54466570 | MIR449B | | | | | | |
| 5 | 54468089 | 54468181 | MIR449C | | | | | | |
| 5 | 54515424 | 54523143 | MCIDAS | | | | | | |
| 5 | 54526980 | 54529545 | CCNO | | | | | | |
| 5 | 54552072 | 54603521 | DHX29 | | | | | | |
| 5 | 54603575 | 54721409 | SKIV2L2 | | | | | | |
| 5 | 54720669 | 54830906 | PLPP1 | | | | | | |
| 5 | 54804677 | 54804754 | MIR5687 | | | | | | |
| 5 | 54824669 | 54830370 | RNF138P1 | | | | | | |
| 5 | 54921672 | 55008163 | SLC38A9 | | | | | | |
| 5 | 55033844 | 55112974 | DDX4 | | | | | | |
| 5 | 55147206 | 55213165 | IL31RA | | | | | | |
| 5 | 55230924 | 55290821 | IL6ST | | | | | | |
| 5 | 55290994 | 55299475 | FLJ31104 | | | | | | |
| 5 | 55395506 | 55529186 | ANKRD55 | | | | | | |
| 5 | 55753621 | 55777596 | LOC102467147 | | | | | | |
| 5 | 55807220 | 55902059 | C5orf67 | | | | | | |
| 5 | 56110899 | 56191978 | MAP3K1 | | | | | | |
| 5 | 56205086 | 56221359 | SETD9 | | | | | | |
| 5 | 56215428 | 56247957 | MIER3 | | | | | | |
| 5 | 56469774 | 56560506 | GPBP1 | | | | | | |
| 5 | 56775842 | 56778636 | ACTBL2 | | | | | | |
| 5 | 56866168 | 56913254 | LINCR-0003 | | | | | | |
| 5 | 56946486 | 56966816 | LOC101928505 | | | | | | |
| 5 | 57186157 | 57194989 | LOC101928539 | | | | | | |
| 5 | 57403458 | 57418168 | LOC101928569 | | | | | | |
| 5 | 57749809 | 57755966 | PLK2 | | | | | | |
| 5 | 57787261 | 57792185 | GAPT | | | | | | |
| 5 | 57837393 | 57854070 | LOC101928600 | | | | | | |
| 5 | 57878014 | 58155222 | RAB3C | | | RAB3C | | | |
| 5 | 58264865 | 59783925 | PDE4D | | | | | | |
| 5 | 59783539 | 59822245 | PART1 | | | | | | |
| 5 | 59892738 | 59995993 | DEPDC1B | | | | | | |
| 5 | 60047615 | 60140101 | ELOVL7 | | | | | | |
| 5 | 60169658 | 60240905 | ERCC8 | | | ERCC8 | | | |
| 5 | 60240955 | 60448864 | NDUFAF2 | | | | | | |
| 5 | 60453535 | 60458302 | SMIM15 | | | | | | |

| | | | | | | | | |
|---|---|---|---|---|---|---|---|---|
| 5 | 60458142 | 60527907 | CTC-436P18.1 | | | | | |
| 5 | 60628099 | 60841999 | ZSWIM6 | | | | | |
| 5 | 60933607 | 61026824 | C5orf64 | | | | | |
| 5 | 60958984 | 60994259 | LOC101928651 | | | | | |
| 5 | 61028616 | 61031525 | LOC100506526 | | | | | |
| 5 | 61601988 | 61683011 | KIF2A | | KIF2A | KIF2A | | |
| 5 | 61684350 | 61699728 | DIMT1 | | | | | |
| 5 | 61708572 | 61924416 | IPO11 | | | | | |
| 5 | 61874561 | 61877275 | LRRC70 | | | | | |
| 5 | 61874561 | 61924416 | IPO11-LRRC70 | | | | | |
| 5 | 63255874 | 63258119 | HTR1A | HTR1A | | | HTR1A | |
| 5 | 63461670 | 63668696 | RNF180 | | | | | |
| 5 | 63801773 | 63908121 | RGS7BP | | | | | |
| 5 | 63986134 | 64014017 | FAM159B | | | | | |
| 5 | 64013977 | 64064496 | SREK1IP1 | | | | | |
| 5 | 64064744 | 64314590 | CWC27 | | | | | |
| 5 | 64444562 | 64777704 | ADAMTS6 | | | | | |
| 5 | 64813592 | 64858995 | CENPK | | | | | |
| 5 | 64859062 | 64883370 | PPWD1 | | | | | |
| 5 | 64885506 | 64920187 | TRIM23 | | | TRIM23 | | |
| 5 | 64920557 | 64961954 | TRAPPC13 | | | | | |
| 5 | 64961754 | 65017941 | SGTB | | | | | |
| 5 | 65018022 | 65125111 | NLN | | | NLN | | |
| 5 | 65222381 | 65376851 | ERBB2IP | | | | | |
| 5 | 65240632 | 65241401 | LOC100303749 | | | | | |
| 5 | 65440045 | 65479444 | SREK1 | | | | | |
| 5 | 65803372 | 65807432 | LOC101928769 | | | | | |
| 5 | 65892175 | 66465423 | MAST4 | | | | | |
| 5 | 66297445 | 66299982 | LOC101928794 | | | | | |
| 5 | 66300447 | 66465423 | MAST4 | | | | | |
| 5 | 66478103 | 66492617 | CD180 | | | | | |
| 5 | 67089040 | 67096886 | LOC101928858 | | | | | |
| 5 | 67485703 | 67494235 | LOC102467655 | | | | | |
| 5 | 67511583 | 67597649 | PIK3R1 | | | | | |
| 5 | 68263567 | 68325754 | LOC101928885 | | | | | |
| 5 | 68389775 | 68426899 | SLC30A5 | | | | | |
| 5 | 68462836 | 68474070 | CCNB1 | | | | | |
| 5 | 68485374 | 68506184 | CENPH | | | | | |
| 5 | 68513572 | 68525985 | MRPS36 | | | | | |
| 5 | 68530621 | 68573257 | CDK7 | | | | | |
| 5 | 68576518 | 68628620 | CCDC125 | | | | | |
| 5 | 68647552 | 68665840 | AK6 | | | | | |
| 5 | 68660569 | 68665840 | TAF9 | | | | | |
| 5 | 68665123 | 68710628 | RAD17 | | | RAD17 | | |
| 5 | 68710938 | 68737890 | MARVELD2 | | | | | |
| 5 | 68773299 | 68781439 | LOC101928924 | | | | | |
| 5 | 68788118 | 68853931 | OCLN | | | | | |
| 5 | 68856050 | 68888729 | GTF2H2C | | | | | |
| 5 | 68856073 | 68888352 | GTF2H2C_2 | | | | | |
| 5 | 68935289 | 69006354 | GUSBP3 | | | | | |
| 5 | 69321071 | 69338940 | SERF1A | | | | | |
| 5 | 69321077 | 69338934 | SERF1B | | | | | |
| 5 | 69345349 | 69373418 | SMN1 | | | SMN1 | | |
| 5 | 69345349 | 69373422 | SMN2 | | | | | |
| 5 | 69423288 | 69586004 | SMA4 | | | | | |
| 5 | 69515015 | 69881629 | GUSBP3 | | | | | |
| 5 | 69711196 | 69746189 | GTF2H2B | | | | | |
| 5 | 69776869 | 69881549 | SMA5 | | | | | |
| 5 | 69782327 | 69787614 | LOC441081 | | | | | |
| 5 | 69812078 | 69851150 | GUSBP9 | | | | | |
| 5 | 70196489 | 70214357 | SERF1A | | | | | |
| 5 | 70196495 | 70214351 | SERF1B | | | | | |
| 5 | 70220767 | 70248838 | SMN1 | | | SMN1 | | |
| 5 | 70220767 | 70248842 | SMN2 | | | | | |
| 5 | 70264309 | 70320941 | NAIP | | | NAIP | | |
| 5 | 70330950 | 70363497 | GTF2H2 | | | | | |

| | | | | | | | | |
|---|---|---|---|---|---|---|---|---|
| 5 | 70370029 | 70388897 | LOC647859 | | | | | |
| 5 | 70516052 | 70555122 | GUSBP9 | | | | | |
| 5 | 70616857 | 70742163 | LOC102724392 | | | | | |
| 5 | 70671611 | 70681820 | PMCHL2 | | | | | |
| 5 | 70751441 | 70863649 | BDP1 | | | | | |
| 5 | 70883114 | 70954530 | MCCC2 | | | MCCC2 | | |
| 5 | 71014989 | 71016875 | CARTPT | | CARTPT | | CARTPT | |
| 5 | 71403117 | 71505397 | MAP1B | | | MAP1B | | |
| 5 | 71465293 | 71465367 | MIR4803 | | | | | |
| 5 | 71515235 | 71616084 | MRPS27 | | | MRPS27 | | |
| 5 | 71616193 | 71655180 | PTCD2 | | | | | |
| 5 | 71735725 | 71803249 | ZNF366 | | | | | |
| 5 | 71852903 | 71867569 | LOC102503427 | | | | | |
| 5 | 71869901 | 71956496 | LOC102477328 | | | | | |
| 5 | 72112417 | 72210215 | TNPO1 | | | | | |
| 5 | 72174417 | 72174490 | MIR4804 | | | | | |
| 5 | 72251807 | 72386349 | FCHO2 | | | | | |
| 5 | 72416387 | 72427644 | TMEM171 | | | | | |
| 5 | 72427834 | 72446419 | LOC105379030 | | | | | |
| 5 | 72469022 | 72470970 | TMEM174 | | | | | |
| 5 | 72491116 | 72497788 | LOC340090 | | | | | |
| 5 | 72742084 | 72744352 | FOXD1 | | | | | |
| 5 | 72750014 | 72768791 | LINC01386 | | | | | |
| 5 | 72794249 | 72801448 | BTF3 | | | | | |
| 5 | 72848024 | 72861511 | ANKRA2 | | | | | |
| 5 | 72861565 | 72879202 | UTP15 | | | | | |
| 5 | 72921982 | 73237818 | ARHGEF28 | | | | | |
| 5 | 73602234 | 73604150 | LINC01335 | | | | | |
| 5 | 73618310 | 73624182 | LINC01333 | | | | | |
| 5 | 73665200 | 73832801 | LINC01331 | | | | | |
| 5 | 73923230 | 73937249 | ENC1 | | | ENC1 | | |
| 5 | 73935847 | 74017113 | HEXB | | | HEXB | | |
| 5 | 74017028 | 74063196 | GFM2 | | | | | |
| 5 | 74062815 | 74072737 | NSA2 | | | | | |
| 5 | 74073398 | 74162663 | FAM169A | | | | | |
| 5 | 74161717 | 74163100 | LOC441086 | | | | | |
| 5 | 74323288 | 74326724 | GCNT4 | | | | | |
| 5 | 74343543 | 74348468 | LINC01336 | | | | | |
| 5 | 74364121 | 74532703 | ANKRD31 | | | | | |
| 5 | 74632992 | 74657926 | HMGCR | HMGCR | | | | |
| 5 | 74666927 | 74807806 | COL4A3BP | | | | | |
| 5 | 74807656 | 74895646 | POLK | | | | | |
| 5 | 74907300 | 74967671 | ANKDD1B | | | | | |
| 5 | 74970023 | 75013313 | POC5 | | | | | |
| 5 | 75379238 | 75649764 | SV2C | | | | | |
| 5 | 75699079 | 76003957 | IQGAP2 | | | | | |
| 5 | 75902435 | 75904794 | LOC101929109 | | | | | |
| 5 | 75904919 | 76003957 | IQGAP2 | | | | | |
| 5 | 75911306 | 75919259 | F2RL2 | | | | | |
| 5 | 76008227 | 76008713 | NCRUPAR | | | | | |
| 5 | 76011867 | 76031605 | F2R | | | | | F2R |
| 5 | 76114832 | 76131140 | F2RL1 | | | | | |
| 5 | 76145825 | 76217056 | S100Z | | | | | |
| 5 | 76248679 | 76265299 | CRHBP | | | | | |
| 5 | 76326209 | 76361058 | AGGF1 | | | | | |
| 5 | 76372531 | 76383030 | ZBED3 | | | | | |
| 5 | 76376258 | 76376396 | SNORA47 | | | | | |
| 5 | 76382622 | 76444176 | ZBED3 | | | | | |
| 5 | 76506705 | 76724080 | PDE8B | | PDE8B | | | |
| 5 | 76726757 | 76788365 | WDR41 | | | | | |
| 5 | 76924536 | 76934522 | OTP | | | | | |
| 5 | 76986993 | 77072185 | TBCA | | | | | |
| 5 | 77180479 | 77254920 | LOC101929154 | | | | | |
| 5 | 77298149 | 77590579 | AP3B1 | | | AP3B1 | | |
| 5 | 77654587 | 77776562 | SCAMP1 | | | | | |
| 5 | 77781037 | 77944648 | LHFPL2 | | | | | |

| | | | | | | | | |
|---|---|---|---|---|---|---|---|---|
| 5 | 78073036 | 78281766 | ARSB | | | ARSB | | |
| 5 | 78293386 | 78365497 | DMGDH | | | DMGDH | | |
| 5 | 78365546 | 78385897 | BHMT2 | | | | | |
| 5 | 78407603 | 78428113 | BHMT | | | | | |
| 5 | 78531924 | 78623038 | JMY | | | | | |
| 5 | 78669646 | 78809659 | HOMER1 | | | HOMER1 | | |
| 5 | 78908242 | 78982471 | PAPD4 | | | | | |
| 5 | 78985658 | 79096049 | CMYA5 | | | | | |
| 5 | 79232401 | 79263449 | LINC01455 | | | | | |
| 5 | 79272538 | 79287088 | MTX3 | | | | | |
| 5 | 79287118 | 79379107 | THBS4 | | | | | THBS4 |
| 5 | 79362171 | 79379488 | CTD-2201I18.1 | | | | | |
| 5 | 79407049 | 79551901 | SERINC5 | | | | | |
| 5 | 79594916 | 79596297 | LOC644936 | | | | | |
| 5 | 79615789 | 79617660 | SPZ1 | | | | | |
| 5 | 79646423 | 79647785 | CRSP8P | | | | | |
| 5 | 79703831 | 79775688 | ZFYVE16 | | | ZFYVE16 | | |
| 5 | 79783799 | 79838382 | FAM151B | | | | | |
| 5 | 79852573 | 79866304 | ANKRD34B | | | | | |
| 5 | 79904439 | 79918343 | LINC01337 | | | | | |
| 5 | 79922044 | 79950800 | DHFR | | | | | |
| 5 | 79945818 | 79946854 | MTRNR2L2 | | | | | |
| 5 | 79950466 | 80172634 | MSH3 | | | | | |
| 5 | 80243511 | 80525981 | RASGRF2 | | RASGRF2 | | | |
| 5 | 80501461 | 80746718 | RNU5E-1 | | | | | |
| 5 | 80501462 | 80746718 | RNU5D-1 | | | | | |
| 5 | 80529138 | 80597388 | CKMT2 | | | CKMT2 | | |
| 5 | 80597401 | 80608965 | ZCCHC9 | | | | | |
| 5 | 80625946 | 80689988 | ACOT12 | | | | | |
| 5 | 80713178 | 81047072 | SSBP2 | | | | | |
| 5 | 81267843 | 81551216 | ATG10 | | | | | |
| 5 | 81569138 | 81574235 | RPS23 | | | | | |
| 5 | 81575280 | 81614465 | ATP6AP1L | | | | | |
| 5 | 82135973 | 82136043 | MIR3977 | | | | | |
| 5 | 82146683 | 82155655 | LINC01338 | | | | | |
| 5 | 82348664 | 82373272 | TMEM167A | | | | | |
| 5 | 82360022 | 82360156 | SCARNA18 | | | | | |
| 5 | 82373227 | 82649579 | XRCC4 | | | | | |
| 5 | 82767492 | 82878122 | VCAN | | | | | |
| 5 | 82934016 | 83016896 | HAPLN1 | | | | | |
| 5 | 83236413 | 83680685 | EDIL3 | | | | | |
| 5 | 85578261 | 85593365 | NBPF22P | | | | | |
| 5 | 85913783 | 85916583 | COX7C | | | | | |
| 5 | 85916313 | 85916392 | MIR3607 | | | | | |
| 5 | 86042634 | 86045589 | LOC100505878 | | | | | |
| 5 | 86410695 | 86410771 | MIR4280 | | | | | |
| 5 | 86415964 | 86543835 | LOC101929380 | | | | | |
| 5 | 86511551 | 86535018 | LOC55338 | | | | | |
| 5 | 86564069 | 86687743 | RASA1 | | | | | |
| 5 | 86614231 | 86619445 | LOC644285 | | | | | |
| 5 | 86690078 | 86708850 | CCNH | | | | | |
| 5 | 87485449 | 87732491 | TMEM161B | | | | | |
| 5 | 87704798 | 87734907 | LOC102546226 | | | | | |
| 5 | 87836596 | 87974262 | LINC00461 | | | | | |
| 5 | 87962670 | 87962757 | MIR9 | | | | | |
| 5 | 88014057 | 88263096 | MEF2C | | | | | |
| 5 | 89312437 | 89312537 | MIR3660 | | | | | |
| 5 | 89454155 | 89585888 | LINC01339 | | | | | |
| 5 | 89689151 | 89705603 | CETN3 | | | | | |
| 5 | 89705816 | 89706486 | LOC731157 | | | | | |
| 5 | 89754019 | 89770585 | MBLAC2 | | | | | |
| 5 | 89770680 | 89810369 | POLR3G | | | | | |
| 5 | 89811444 | 89825401 | LYSMD3 | | | | | |
| 5 | 89854616 | 90460033 | ADGRV1 | | | | | |
| 5 | 90598802 | 90610219 | LUCAT1 | | | | | |
| 5 | 90664540 | 90716532 | ARRDC3 | | | | | |

| | | | | | | | | |
|---|---|---|---|---|---|---|---|---|
| 5 | 92745061 | 92930315 | NR2F1 | | NR2F1 | | | |
| 5 | 92922830 | 92957593 | MIR548AO | | | | | |
| 5 | 92953430 | 93447404 | FAM172A | | | | | |
| 5 | 92956401 | 92956494 | MIR2277 | | | | | |
| 5 | 93076014 | 93077309 | POU5F2 | | | POU5F2 | | |
| 5 | 93486555 | 93954309 | KIAA0825 | | | | | |
| 5 | 93954390 | 94031573 | SLF1 | | | | | |
| 5 | 94041241 | 94620279 | MCTP1 | | | | | |
| 5 | 94727047 | 94786144 | FAM81B | | | | | |
| 5 | 94799598 | 94890709 | TTC37 | | | | | |
| 5 | 94890824 | 94940806 | ARSK | | | | | |
| 5 | 94955979 | 94957284 | GPR150 | | | | | |
| 5 | 94982480 | 94992849 | RFESD | | | | | |
| 5 | 94987884 | 95018714 | SPATA9 | | | | | |
| 5 | 95066849 | 95132071 | RHOBTB3 | | | | | |
| 5 | 95149552 | 95158577 | GLRX | | | | | |
| 5 | 95187935 | 95195836 | LINC01554 | | | | | |
| 5 | 95220801 | 95297775 | ELL2 | | | | | |
| 5 | 95297704 | 95966789 | LOC101929710 | | | | | |
| 5 | 95414841 | 95414916 | MIR583 | | | | | |
| 5 | 95726039 | 95768985 | PCSK1 | | PCSK1 | PCSK1 | | |
| 5 | 95997740 | 96109116 | CAST | | | | | |
| 5 | 96096513 | 96149848 | ERAP1 | | | | | |
| 5 | 96211643 | 96255406 | ERAP2 | | | | | |
| 5 | 96271345 | 96365115 | LNPEP | | | LNPEP | | |
| 5 | 96427573 | 96478520 | LIX1 | | | | | |
| 5 | 96496570 | 96519005 | RIOK2 | | | | | |
| 5 | 96840399 | 97006755 | LINC01340 | | | | | |
| 5 | 98104998 | 98108788 | RGMB | | | | | |
| 5 | 98190907 | 98262238 | CHD1 | | | | | |
| 5 | 98264837 | 98266713 | LOC100289230 | | | | | |
| 5 | 98869693 | 98913658 | CTD-2151A2.1 | | | | | |
| 5 | 99715208 | 99723958 | LOC100133050 | | | | | |
| 5 | 99871008 | 99922444 | FAM174A | | | | | |
| 5 | 100142638 | 100238989 | ST8SIA4 | | | | | |
| 5 | 100152185 | 100152269 | MIR548P | | | | | |
| 5 | 100221014 | 100238989 | ST8SIA4 | | | | | |
| 5 | 101569691 | 101632253 | SLCO4C1 | | | | | |
| 5 | 101707485 | 101834720 | SLCO6A1 | | | | | |
| 5 | 101917071 | 101953291 | LINC00492 | | | | | |
| 5 | 101944195 | 102007168 | LINC00491 | | | | | |
| 5 | 102090486 | 102366808 | PAM | | | | | |
| 5 | 102421703 | 102455842 | GIN1 | | | | | |
| 5 | 102455957 | 102539224 | PPIP5K2 | | | | | |
| 5 | 102594402 | 102614361 | C5orf30 | | | | | |
| 5 | 102864134 | 102877686 | LOC102467212 | | | | | |
| 5 | 102884555 | 102898502 | NUDT12 | | | | | |
| 5 | 104435174 | 104435799 | RAB9BP1 | | | | | |
| 5 | 106150897 | 106346715 | LOC102467213 | | | | | |
| 5 | 106712589 | 107006596 | EFNA5 | | | | | |
| 5 | 107194733 | 107717799 | FBXL17 | | | | | |
| 5 | 108063525 | 108063962 | LINC01023 | | | | | |
| 5 | 108083522 | 108532541 | FER | | | | | |
| 5 | 108670409 | 108745675 | PJA2 | | | | | |
| 5 | 109025066 | 109205326 | MAN2A1 | | | | | |
| 5 | 109218882 | 109221200 | LOC100289673 | | | | | |
| 5 | 109755197 | 110062450 | TMEM232 | | | | | |
| 5 | 109849529 | 109849616 | MIR548F3 | | | | | |
| 5 | 110073836 | 110100861 | SLC25A46 | | | | | |
| 5 | 110405777 | 110413722 | TSLP | | | | | |
| 5 | 110427869 | 110466200 | WDR36 | | | | | |
| 5 | 110559946 | 110820748 | CAMK4 | | | CAMK4 | | |
| 5 | 110831731 | 111075423 | STARD4 | | | | | |
| 5 | 111064999 | 111353003 | NREP | | | | | |
| 5 | 111496222 | 111498198 | EPB41L4A | | | | | |
| 5 | 111497181 | 111497314 | SNORA13 | | | | | |

| | | | | | | | | |
|---|---|---|---|---|---|---|---|---|
| 5 | 111498314 | 111755010 | EPB41L4A | | | | | |
| 5 | 111563979 | 111593006 | LOC101927023 | | | | | |
| 5 | 111755279 | 111756677 | EPB41L4A | | | | | |
| 5 | 111964132 | 111966618 | LOC102467214 | | | | | |
| 5 | 111992124 | 112018585 | LOC102467216 | | | | | |
| 5 | 112043201 | 112181936 | APC | | APC | APC | | |
| 5 | 112196884 | 112228776 | SRP19 | | | | | |
| 5 | 112212080 | 112258031 | REEP5 | | | | | |
| 5 | 112312406 | 112357892 | DCP2 | | | | | |
| 5 | 112357795 | 112824527 | MCC | | | | | |
| 5 | 112768250 | 112770728 | TSSK1B | | | | | |
| 5 | 112849390 | 112930984 | YTHDC2 | | | | | |
| 5 | 113698015 | 113832197 | KCNN2 | | | | | |
| 5 | 113783114 | 114109110 | LOC101927078 | | | | | |
| 5 | 113911737 | 113915667 | LOC101927059 | | | | | |
| 5 | 114460458 | 114516243 | TRIM36 | | | TRIM36 | | |
| 5 | 114546526 | 114598569 | PGGT1B | | | | | |
| 5 | 114602884 | 114632458 | CCDC112 | | | | | |
| 5 | 114856607 | 114880591 | FEM1C | | | | | |
| 5 | 114914192 | 114938176 | TICAM2 | | | | | |
| 5 | 114914192 | 114961876 | TMED7-TICAM2 | | | | | |
| 5 | 114937851 | 114956116 | LOC101927100 | | | | | |
| 5 | 114948904 | 114961876 | TMED7 | | | | | |
| 5 | 114977821 | 115008349 | LOC102467217 | | | | | |
| 5 | 115140429 | 115152405 | CDO1 | | | | | |
| 5 | 115163893 | 115177548 | ATG12 | | | | | |
| 5 | 115177302 | 115249778 | AP3S1 | | | AP3S1 | | |
| 5 | 115298150 | 115363299 | LVRN | | | | | |
| 5 | 115387162 | 115394827 | ARL14EPL | | | | | |
| 5 | 115420672 | 115628987 | COMMD10 | | | | | |
| 5 | 115703112 | 115710267 | LOC101927190 | | | | | |
| 5 | 115779250 | 115807369 | SEMA6A | | | SEMA6A | | |
| 5 | 116078997 | 116097905 | LOC102467223 | | | | | |
| 5 | 116751207 | 116915439 | LINC00992 | | | | | |
| 5 | 117066055 | 117601757 | LOC102467224 | | | | | |
| 5 | 117618268 | 117620472 | HNCAT21 | | | | | |
| 5 | 117686666 | 117897812 | HRAT56 | | | | | |
| 5 | 117931882 | 117963787 | LOC102467225 | | | | | |
| 5 | 118171768 | 118324300 | DTWD2 | | | | | |
| 5 | 118310280 | 118310362 | MIR1244 | | | | | |
| 5 | 118332006 | 118406585 | LOC105379143 | | | | | |
| 5 | 118406781 | 118584824 | DMXL1 | | | | | |
| 5 | 118490331 | 118490411 | MIR5706 | | | | | |
| 5 | 118604386 | 118730299 | TNFAIP8 | | | | | |
| 5 | 118788137 | 118878030 | HSD17B4 | | | | | |
| 5 | 118965253 | 118971517 | FAM170A | | | | | |
| 5 | 119799972 | 120023025 | PRR16 | | | | | |
| 5 | 120658244 | 120661532 | LOC102467226 | | | | | |
| 5 | 121187649 | 121188523 | FTMT | | | FTMT | | |
| 5 | 121297655 | 121364295 | SRFBP1 | | | | | |
| 5 | 121398889 | 121414196 | LOX | | | | | |
| 5 | 121465207 | 121489266 | ZNF474 | | | | | |
| 5 | 121495870 | 121518358 | LOC100505841 | | | | | |
| 5 | 121647385 | 121799914 | SNCAIP | | | | | SNCAIP |
| 5 | 121772191 | 121814782 | MGC32805 | | | | | |
| 5 | 121917193 | 121920295 | LOC101927357 | | | | | |
| 5 | 121964646 | 122066255 | LOC101927379 | | | | | |
| 5 | 122110690 | 122170234 | SNX2 | | | | | |
| 5 | 122181159 | 122344902 | SNX24 | | | | | |
| 5 | 122359077 | 122372425 | PPIC | | | | | |
| 5 | 122424840 | 122523745 | PRDM6 | | | | | |
| 5 | 122680578 | 122759286 | CEP120 | | | | | |
| 5 | 122847792 | 122952738 | CSNK1G3 | | | | | |
| 5 | 123395486 | 123774213 | LINC01170 | | | | | |
| 5 | 123972609 | 124080805 | ZNF608 | | | | | |
| 5 | 124372523 | 124703427 | LOC101927421 | | | | | |

| | | | | | | | | |
|---|---|---|---|---|---|---|---|---|
| 5 | 124828953 | 124937920 | LOC101927460 | | | | | |
| 5 | 125515269 | 125529543 | LOC102546228 | | | | | |
| 5 | 125608209 | 125620867 | LOC101927488 | | | | | |
| 5 | 125695787 | 125829853 | GRAMD3 | | | | | |
| 5 | 125877532 | 125931082 | ALDH7A1 | | | | | |
| 5 | 125936606 | 125962944 | PHAX | | | | | |
| 5 | 125967413 | 125971974 | TEX43 | | | | | |
| 5 | 126087654 | 126112178 | LOC102723557 | | | | | |
| 5 | 126112314 | 126172712 | LMNB1 | | | LMNB1 | | |
| 5 | 126203405 | 126366500 | MARCH3 | | | | | |
| 5 | 126378249 | 126409184 | C5orf63 | | | | | |
| 5 | 126626455 | 126796910 | MEGF10 | | MEGF10 | | | |
| 5 | 126853300 | 126890780 | PRRC1 | | | | | |
| 5 | 126984712 | 126994322 | CTXN3 | | | | | |
| 5 | 127039080 | 127277208 | CCDC192 | | | | | |
| 5 | 127357243 | 127418766 | LINC01184 | | | | | |
| 5 | 127419482 | 127525380 | SLC12A2 | | SLC12A2 | | | |
| 5 | 127593600 | 127873735 | FBN2 | | | | | |
| 5 | 128300819 | 128369335 | SLC27A6 | | | | | |
| 5 | 128430441 | 128449719 | ISOC1 | | | | | |
| 5 | 128433380 | 128433459 | MIR4633 | | | | | |
| 5 | 128732754 | 128732840 | MIR4460 | | | | | |
| 5 | 128795251 | 129074376 | ADAMTS19 | | | | | |
| 5 | 129083883 | 129100756 | KIAA1024L | | | | | |
| 5 | 129240522 | 129522327 | CHSY3 | | | CHSY3 | | |
| 5 | 130494975 | 130501041 | HINT1 | | | | | |
| 5 | 130506606 | 130541119 | LYRM7 | | | | | |
| 5 | 130599701 | 130730382 | CDC42SE2 | | | | | |
| 5 | 130759613 | 130970929 | RAPGEF6 | | | RAPGEF6 | | |
| 5 | 130977406 | 131132756 | FNIP1 | | | | | |
| 5 | 131142683 | 131281391 | MEIKIN | | | | | |
| 5 | 131285666 | 131347761 | ACSL6 | | ACSL6 | ACSL6 | | |
| 5 | 131396346 | 131398896 | IL3 | | | | | |
| 5 | 131409484 | 131411863 | CSF2 | | | | | |
| 5 | 131520568 | 131563556 | P4HA2 | | | | | |
| 5 | 131553541 | 131553611 | MIR6830 | | | | | |
| 5 | 131593350 | 131609147 | PDLIM4 | | | | | |
| 5 | 131630144 | 131679899 | SLC22A4 | | | | | |
| 5 | 131646968 | 131705608 | LOC553103 | | | | | |
| 5 | 131701181 | 131701291 | MIR3936 | | | | | |
| 5 | 131705400 | 131731306 | SLC22A5 | | | SLC22A5 | | |
| 5 | 131746464 | 131811736 | C5orf56 | | | | | |
| 5 | 131817300 | 131826465 | IRF1 | | | | | |
| 5 | 131877135 | 131879214 | IL5 | | IL5 | | | |
| 5 | 131892615 | 131980313 | RAD50 | | | | | |
| 5 | 131966280 | 131991846 | TH2LCRR | | | | | |
| 5 | 131993864 | 131996801 | IL13 | | | | | |
| 5 | 132009677 | 132018370 | IL4 | | | | | |
| 5 | 132014776 | 132018321 | LOC105379176 | | | | | |
| 5 | 132028318 | 132073270 | KIF3A | | | | | |
| 5 | 132083136 | 132090095 | CCNI2 | | | | | |
| 5 | 132086508 | 132113561 | SEPT8 | | | | | |
| 5 | 132149015 | 132152489 | SOWAHA | | | | | |
| 5 | 132157832 | 132166590 | SHROOM1 | | | | | |
| 5 | 132196873 | 132202576 | GDF9 | | | | | |
| 5 | 132202318 | 132204536 | UQCRQ | | | | | |
| 5 | 132209357 | 132210582 | LEAP2 | | | | | |
| 5 | 132211070 | 132299354 | AFF4 | | | AFF4 | | |
| 5 | 132332676 | 132362296 | ZCCHC10 | | | | | |
| 5 | 132387661 | 132440709 | HSPA4 | | | | | |
| 5 | 132532151 | 132948223 | FSTL4 | | | | | |
| 5 | 132763287 | 132763398 | MIR1289 | | | | | |
| 5 | 133249367 | 133252960 | WSPAR | | | | | |
| 5 | 133291197 | 133304406 | C5orf15 | | | | | |
| 5 | 133304379 | 133305914 | LOC105379183 | | | | | |
| 5 | 133307565 | 133340824 | VDAC1 | VDAC1 | | VDAC1 | | |

| | | | | | | | | |
|---|---|---|---|---|---|---|---|---|
| 5 | 133450401 | 133483920 | TCF7 | | | | | |
| 5 | 133492081 | 133512724 | SKP1 | | | | | |
| 5 | 133532147 | 133561950 | PPP2CA | | | | | |
| 5 | 133561447 | 133561543 | MIR3661 | | | | | |
| 5 | 133634114 | 133702765 | CDKL3 | | CDKL3 | | | |
| 5 | 133706866 | 133727799 | UBE2B | | | | | |
| 5 | 133737755 | 133747598 | CDKN2AIPNL | | | | | |
| 5 | 133764741 | 133770738 | LOC102546229 | | | | | |
| 5 | 133842242 | 133844921 | LOC101927934 | | | | | |
| 5 | 133860065 | 133918920 | JADE2 | | | | | |
| 5 | 133936838 | 133968533 | SAR1B | | | | | |
| 5 | 133984474 | 134063601 | SEC24A | | | | | |
| 5 | 134074169 | 134087850 | CAMLG | | | | | |
| 5 | 134094423 | 134166826 | DDX46 | | | | | |
| 5 | 134181369 | 134195425 | C5orf24 | | | | | |
| 5 | 134209459 | 134237323 | TXNDC15 | | | | | |
| 5 | 134240809 | 134298336 | PCBD2 | | | | | |
| 5 | 134263728 | 134263802 | MIR4461 | | | | | |
| 5 | 134303595 | 134347397 | CATSPER3 | | | | | |
| 5 | 134363423 | 134369964 | PITX1 | | | | | |
| 5 | 134368969 | 134583867 | C5orf66 | | | | | |
| 5 | 134670070 | 134735577 | H2AFY | | | | | |
| 5 | 134779903 | 134783038 | DCANP1 | | | | | |
| 5 | 134784557 | 134788089 | TIFAB | | | | | |
| 5 | 134869971 | 134871639 | NEUROG1 | | | | | |
| 5 | 134906370 | 134914969 | CXCL14 | | | CXCL14 | | |
| 5 | 134984371 | 134989624 | LOC340074 | | | | | |
| 5 | 135170364 | 135224326 | SLC25A48 | | | | | |
| 5 | 135227934 | 135231516 | IL9 | | | | | |
| 5 | 135266005 | 135277367 | FBXL21 | | | | | |
| 5 | 135282599 | 135290723 | LECT2 | | | | | |
| 5 | 135364583 | 135399507 | TGFBI | | | | | |
| 5 | 135416179 | 135416287 | VTRNA2-1 | | | | | |
| 5 | 135465202 | 135518422 | SMAD5 | | | | | |
| 5 | 135527155 | 135528851 | LOC389332 | | | | | |
| 5 | 135548998 | 135651788 | TRPC7 | | | | | |
| 5 | 135637492 | 135637544 | MIR4454 | | | | | |
| 5 | 136310986 | 136835018 | SPOCK1 | | SPOCK1 | | | |
| 5 | 136463885 | 136466658 | LOC105379192 | | | | | |
| 5 | 136953188 | 137071779 | KLHL3 | | KLHL3 | | KLHL3 | |
| 5 | 136983260 | 136983338 | MIR874 | | | | | |
| 5 | 137087072 | 137090039 | HNRNPA0 | | | | | |
| 5 | 137136881 | 137146439 | NPY6R | | | | | |
| 5 | 137203544 | 137223540 | MYOT | | | | | |
| 5 | 137225124 | 137278434 | PKD2L2 | | | | | |
| 5 | 137273606 | 137368802 | FAM13B | | | | | |
| 5 | 137368462 | 137374622 | LOC100130172 | | | | | |
| 5 | 137419580 | 137428054 | WNT8A | | | | | |
| 5 | 137450860 | 137475132 | NME5 | | | | | |
| 5 | 137475458 | 137514358 | BRD8 | | | | | |
| 5 | 137514416 | 137523404 | KIF20A | | | KIF20A | | |
| 5 | 137523336 | 137549032 | CDC23 | | | | | |
| 5 | 137588068 | 137610253 | GFRA3 | | | | | |
| 5 | 137620953 | 137674044 | CDC25C | | | | | |
| 5 | 137673223 | 137685418 | FAM53C | | | | | |
| 5 | 137688284 | 137772716 | KDM3B | | | | | |
| 5 | 137774689 | 137782658 | REEP2 | | REEP2 | | | |
| 5 | 137801180 | 137805004 | EGR1 | EGR1 | | | | |
| 5 | 137841781 | 137878989 | ETF1 | | | ETF1 | | |
| 5 | 137890570 | 137911318 | HSPA9 | | | | | |
| 5 | 137896731 | 137896799 | SNORD63 | | | | | |
| 5 | 138080118 | 138089613 | LOC105379194 | | | | | |
| 5 | 138089084 | 138270723 | CTNNA1 | | | | | |
| 5 | 138205078 | 138211057 | LRRTM2 | | | LRRTM2 | | |
| 5 | 138210918 | 138270723 | CTNNA1 | | | | | |
| 5 | 138282409 | 138534065 | SIL1 | | | | | |

| | | | | | | | | | |
|---|---|---|---|---|---|---|---|---|---|
| 5 | 138609440 | 138618873 | SNHG4 | | | | | | |
| 5 | 138609440 | 138667366 | MATR3 | | | | | | |
| 5 | 138614468 | 138614668 | SNORA74A | | | | | | |
| 5 | 138629332 | 138667366 | MATR3 | | | | | | |
| 5 | 138677520 | 138705409 | PAIP2 | | | | | | |
| 5 | 138702884 | 138719039 | SLC23A1 | | | SLC23A1 | | | |
| 5 | 138723256 | 138725605 | MZB1 | | | | | | |
| 5 | 138727634 | 138730885 | PROB1 | | | | | | |
| 5 | 138732455 | 138739776 | SPATA24 | | | | | | |
| 5 | 138745891 | 138775214 | DNAJC18 | | | | | | |
| 5 | 138784242 | 138842328 | ECSCR | | | | | | |
| 5 | 138855112 | 138862375 | TMEM173 | | | | | | |
| 5 | 138940750 | 139008018 | UBE2D2 | | | | | | |
| 5 | 139026883 | 139063470 | CXXC5 | | | | | | |
| 5 | 139120931 | 139125808 | LOC101929696 | | | | | | |
| 5 | 139175405 | 139224048 | PSD2 | | | | | | |
| 5 | 139226363 | 139422884 | NRG2 | | | NRG2 | | | |
| 5 | 139482506 | 139487597 | LINC01024 | | | | | | |
| 5 | 139493707 | 139499001 | PURA | | PURA | | | | |
| 5 | 139505520 | 139508391 | IGIP | | | | | | |
| 5 | 139536903 | 139548370 | LOC101929719 | | | | | | |
| 5 | 139554652 | 139623374 | CYSTM1 | | | | | | |
| 5 | 139624634 | 139682689 | PFDN1 | | | | | | |
| 5 | 139712427 | 139726188 | HBEGF | | | | | | |
| 5 | 139739786 | 139754722 | SLC4A9 | | | | | | |
| 5 | 139781398 | 139919441 | ANKHD1 | | | | | | |
| 5 | 139781398 | 139929163 | ANKHD1-EIF4EBP3 | | | | | | |
| 5 | 139927250 | 139929163 | EIF4EBP3 | | | | | | |
| 5 | 139929651 | 139937678 | SRA1 | | | | | | |
| 5 | 139937852 | 139944189 | APBB3 | | | | | | |
| 5 | 139943255 | 139943336 | MIR6831 | | | | | | |
| 5 | 139944149 | 139948689 | SLC35A4 | | | | | | |
| 5 | 140011312 | 140013286 | CD14 | | | | | | |
| 5 | 140019011 | 140024993 | TMCO6 | | | | | | |
| 5 | 140024947 | 140027370 | NDUFA2 | | | | | | |
| 5 | 140027383 | 140042065 | IK | | | | | | |
| 5 | 140027428 | 140027511 | MIR3655 | | | | | | |
| 5 | 140044383 | 140050553 | WDR55 | | | | | | |
| 5 | 140050380 | 140053171 | DND1 | | | | | | |
| 5 | 140053488 | 140071312 | HARS | | | HARS | | | |
| 5 | 140071010 | 140078903 | HARS2 | | | HARS2 | | | |
| 5 | 140079944 | 140086266 | ZMAT2 | | | | | | |
| 5 | 140090860 | 140090958 | VTRNA1-1 | | | | | | |
| 5 | 140098510 | 140098598 | VTRNA1-2 | | | | | | |
| 5 | 140105743 | 140105831 | VTRNA1-3 | | | | | | |
| 5 | 140165720 | 140391929 | PCDHA1 | | | PCDHA1 | | | |
| 5 | 140174443 | 140391929 | PCDHA2 | | | | | | |
| 5 | 140180782 | 140391929 | PCDHA3 | | | | | | |
| 5 | 140186658 | 140391929 | PCDHA4 | | | | | | |
| 5 | 140201360 | 140391929 | PCDHA5 | | | | | | |
| 5 | 140207506 | 140391929 | PCDHA6 | | | | | | |
| 5 | 140213968 | 140391929 | PCDHA7 | | | | | | |
| 5 | 140220906 | 140391929 | PCDHA8 | | | | | | |
| 5 | 140227356 | 140391929 | PCDHA9 | | | | | | |
| 5 | 140235467 | 140391929 | PCDHA10 | | PCDHA10 | | | | |
| 5 | 140247097 | 140391929 | PCDHA11 | | | | | | |
| 5 | 140254886 | 140391929 | PCDHA12 | | | | | | |
| 5 | 140261853 | 140391929 | PCDHA13 | | | | | | |
| 5 | 140306301 | 140391929 | PCDHAC1 | | | | | | |
| 5 | 140345746 | 140349006 | PCDHAC2 | | | | | | |
| 5 | 140425789 | 140457609 | LOC101926905 | | | | | | |
| 5 | 140430960 | 140433547 | PCDHB1 | | | | | | |
| 5 | 140474190 | 140476964 | PCDHB2 | | | | | | |
| 5 | 140479825 | 140483407 | PCDHB3 | | | | | | |
| 5 | 140501331 | 140505203 | PCDHB4 | | PCDHB4 | | | | |
| 5 | 140514787 | 140517709 | PCDHB5 | | | | | | |

| | | | | | | | | |
|---|---|---|---|---|---|---|---|---|
| 5 | 140529602 | 140532868 | PCDHB6 | | | | | |
| 5 | 140535579 | 140537990 | PCDHB17P | | | | | |
| 5 | 140552201 | 140555957 | PCDHB7 | | | | | |
| 5 | 140557370 | 140560081 | PCDHB8 | | | | | |
| 5 | 140561264 | 140565796 | PCDHB16 | | | | | |
| 5 | 140566700 | 140571111 | PCDHB9 | | | | | |
| 5 | 140571951 | 140575213 | PCDHB10 | | | | | |
| 5 | 140579347 | 140582618 | PCDHB11 | | | | | |
| 5 | 140588290 | 140591698 | PCDHB12 | | | | | |
| 5 | 140593490 | 140596993 | PCDHB13 | | | | | |
| 5 | 140601897 | 140605860 | PCDHB14 | | | PCDHB14 | | |
| 5 | 140613904 | 140617101 | PCDHB18P | | | | | |
| 5 | 140619688 | 140624312 | PCDHB19P | | | | | |
| 5 | 140624916 | 140627801 | PCDHB15 | | | | | |
| 5 | 140682195 | 140683630 | SLC25A2 | | | | | |
| 5 | 140698056 | 140700351 | TAF7 | | | | | |
| 5 | 140710251 | 140892546 | PCDHGA1 | | | | | |
| 5 | 140718326 | 140892546 | PCDHGA2 | | | | | |
| 5 | 140723600 | 140892546 | PCDHGA3 | | | | | |
| 5 | 140729827 | 140892546 | PCDHGB1 | | | | | |
| 5 | 140734591 | 140892546 | PCDHGA4 | | | | | |
| 5 | 140739702 | 140892546 | PCDHGB2 | | | | | |
| 5 | 140743897 | 140892546 | PCDHGA5 | | | | | |
| 5 | 140749830 | 140892546 | PCDHGB3 | | | | | |
| 5 | 140753650 | 140892546 | PCDHGA6 | | | | | |
| 5 | 140762305 | 140892546 | PCDHGA7 | | | | | |
| 5 | 140767451 | 140892546 | PCDHGB4 | | | | | |
| 5 | 140771482 | 140892546 | PCDHGA8 | | | | | |
| 5 | 140777694 | 140892546 | PCDHGB5 | | | | | |
| 5 | 140782519 | 140892546 | PCDHGA9 | | | | | |
| 5 | 140787769 | 140892546 | PCDHGB6 | | | | | |
| 5 | 140792742 | 140892546 | PCDHGA10 | | | | | |
| 5 | 140797213 | 140892548 | PCDHGB7 | | | | | |
| 5 | 140800536 | 140892546 | PCDHGA11 | | | | | |
| 5 | 140805852 | 140807825 | PCDHGB8P | | | | | |
| 5 | 140810157 | 140892546 | PCDHGA12 | | | | | |
| 5 | 140855568 | 140892544 | PCDHGC3 | | | | | |
| 5 | 140864740 | 140892546 | PCDHGC4 | | | | | |
| 5 | 140868807 | 140892546 | PCDHGC5 | | | | | |
| 5 | 140894587 | 140998622 | DIAPH1 | | | | | |
| 5 | 140937877 | 140944827 | LOC100505658 | | | | | |
| 5 | 141000442 | 141016423 | HDAC3 | | | | | |
| 5 | 141016516 | 141020631 | RELL2 | | | | | |
| 5 | 141018868 | 141030986 | FCHSD1 | | | | | |
| 5 | 141032967 | 141061800 | ARAP3 | | | | | |
| 5 | 141232675 | 141257975 | PCDH1 | | | | | |
| 5 | 141275190 | 141276260 | LOC729080 | | | | | |
| 5 | 141303384 | 141321612 | KIAA0141 | | | | | |
| 5 | 141323149 | 141338627 | PCDH12 | | | | | |
| 5 | 141346401 | 141369856 | RNF14 | | | | | |
| 5 | 141380233 | 141392620 | GNPDA1 | | | | | |
| 5 | 141488323 | 141534008 | NDFIP1 | | | | | |
| 5 | 141689991 | 141704620 | SPRY4 | | | | | |
| 5 | 141697198 | 141697887 | SPRY4-IT1 | | | | | |
| 5 | 141704857 | 141843619 | LOC101926941 | | | | | |
| 5 | 141971742 | 142077635 | FGF1 | | | | | |
| 5 | 142125164 | 142140558 | LOC101926975 | | | | | |
| 5 | 142150291 | 142248475 | ARHGAP26 | | | | | |
| 5 | 142572064 | 142573731 | ARHGAP26-IT1 | | | | | |
| 5 | 142657495 | 142783254 | NR3C1 | | | NR3C1 | | |
| 5 | 143059424 | 143059536 | MIR5197 | | | | | |
| 5 | 143191725 | 143200284 | HMHB1 | | | | | |
| 5 | 143537722 | 143550278 | YIPF5 | | | | | |
| 5 | 143550436 | 143856944 | KCTD16 | | | | | |
| 5 | 145135906 | 145214932 | PRELID2 | | | | | |
| 5 | 145239295 | 145252531 | GRXCR2 | | | | | |

| | | | | | | | | |
|---|---|---|---|---|---|---|---|---|
| 5 | 145316125 | 145442879 | SH3RF2 | | | | | |
| 5 | 145463875 | 145483946 | PLAC8L1 | | | | | |
| 5 | 145492588 | 145562350 | LARS | | | | | |
| 5 | 145583162 | 145668784 | RBM27 | | | | | |
| 5 | 145718586 | 145720083 | POU4F3 | | | POU4F3 | | |
| 5 | 145826872 | 145891069 | TCERG1 | | | | | |
| 5 | 145894416 | 145895676 | GPR151 | | | | | |
| 5 | 145969066 | 146461033 | PPP2R2B | | PPP2R2B | | | |
| 5 | 146293769 | 146299069 | PPP2R2B-IT1 | | | | | |
| 5 | 146614578 | 146767418 | STK32A | | | | | |
| 5 | 146770370 | 146889619 | DPYSL3 | | | DPYSL3 | | |
| 5 | 146939556 | 147162411 | JAKMIP2 | | | | | |
| 5 | 147204142 | 147218794 | SPINK1 | | | SPINK1 | | |
| 5 | 147258273 | 147261756 | SCGB3A2 | | | | | |
| 5 | 147272270 | 147286101 | C5orf46 | | | | | |
| 5 | 147443534 | 147516925 | SPINK5 | | | SPINK5 | | |
| 5 | 147548108 | 147555182 | SPINK14 | | | | | |
| 5 | 147582356 | 147594700 | SPINK6 | | | | | |
| 5 | 147647869 | 147763413 | LOC102546294 | | | | | |
| 5 | 147648422 | 147665773 | SPINK13 | | | | | |
| 5 | 147691989 | 147695481 | SPINK7 | | | | | |
| 5 | 147715121 | 147719412 | SPINK9 | | | | | |
| 5 | 147763497 | 147822399 | FBXO38 | | | | | |
| 5 | 147830594 | 148034090 | HTR4 | | | HTR4 | | |
| 5 | 148206155 | 148208197 | ADRB2 | | | | ADRB2 | |
| 5 | 148361712 | 148442737 | SH3TC2 | | SH3TC2 | SH3TC2 | | |
| 5 | 148442879 | 148489350 | LOC255187 | | | | | |
| 5 | 148521045 | 148640001 | ABLIM3 | | | | | |
| 5 | 148651400 | 148721367 | AFAP1L1 | | | | | |
| 5 | 148724976 | 148737205 | GRPEL2 | | | | | |
| 5 | 148737569 | 148749221 | PCYOX1L | | | | | |
| 5 | 148753829 | 148783765 | IL17B | | | | | |
| 5 | 148786407 | 148812399 | CARMN | | | | | |
| 5 | 148808480 | 148808586 | MIR143 | | | | | |
| 5 | 148810208 | 148810296 | MIR145 | | | | | |
| 5 | 148875456 | 148931115 | CSNK1A1 | | | CSNK1A1 | | |
| 5 | 148961134 | 149014527 | ARHGEF37 | | | | | |
| 5 | 149109814 | 149234585 | PPARGC1B | | | | | |
| 5 | 149112387 | 149112453 | MIR378A | | | | | |
| 5 | 149151502 | 149234585 | PPARGC1B | | | | | |
| 5 | 149237518 | 149324356 | PDE6A | | | | | |
| 5 | 149310926 | 149312540 | LOC644762 | | | | | |
| 5 | 149340299 | 149366963 | SLC26A2 | | | | | |
| 5 | 149372685 | 149380730 | TIGD6 | | | | | |
| 5 | 149380168 | 149432706 | HMGXB3 | | | | | |
| 5 | 149432853 | 149492935 | CSF1R | | CSF1R | | | |
| 5 | 149493401 | 149535422 | PDGFRB | | | | | |
| 5 | 149546343 | 149564121 | CDX1 | | | | | |
| 5 | 149569519 | 149590635 | SLC6A7 | | | | | |
| 5 | 149599053 | 149669403 | CAMK2A | | CAMK2A | CAMK2A | | |
| 5 | 149675908 | 149682525 | ARSI | | | ARSI | | |
| 5 | 149737201 | 149779871 | TCOF1 | | | TCOF1 | | |
| 5 | 149781199 | 149792499 | CD74 | | | | | |
| 5 | 149823791 | 149829319 | RPS14 | | | | | |
| 5 | 149855078 | 149865555 | LOC102546298 | | | | | |
| 5 | 149877339 | 149937773 | NDST1 | | NDST1 | | | |
| 5 | 149980641 | 150038792 | SYNPO | | | | | |
| 5 | 150040402 | 150058930 | MYOZ3 | | | | | |
| 5 | 150070351 | 150080669 | RBM22 | | | | | |
| 5 | 150088308 | 150138657 | DCTN4 | | | | | |
| 5 | 150157507 | 150176298 | SMIM3 | | | | | |
| 5 | 150226084 | 150228231 | IRGM | | | | | |
| 5 | 150273953 | 150284545 | ZNF300 | | | | | |
| 5 | 150309997 | 150326146 | ZNF300P1 | | | | | |
| 5 | 150399998 | 150408554 | GPX3 | | | | | |
| 5 | 150409503 | 150467221 | TNIP1 | | | | | |

| | | | | | | | | |
|---|---|---|---|---|---|---|---|---|
| 5 | 150480266 | 150537443 | ANXA6 | | | | | |
| 5 | 150560612 | 150603654 | CCDC69 | | | | | |
| 5 | 150632612 | 150649953 | GM2A | GM2A | | | | |
| 5 | 150655925 | 150683334 | SLC36A3 | | | | | |
| 5 | 150694538 | 150727151 | SLC36A2 | | | | | |
| 5 | 150816552 | 150871942 | SLC36A1 | | | | | |
| 5 | 150883652 | 150948505 | FAT2 | | | FAT2 | | |
| 5 | 150901647 | 150901709 | MIR6499 | | | | | |
| 5 | 151040656 | 151066615 | SPARC | | | | | |
| 5 | 151056505 | 151067468 | CTB-113P19.1 | | | | | |
| 5 | 151122382 | 151138210 | ATOX1 | | | | | |
| 5 | 151149353 | 151151737 | LOC100652758 | | | | | |
| 5 | 151151475 | 151184915 | G3BP1 | | | | | |
| 5 | 151202073 | 151304397 | GLRA1 | | | GLRA1 | GLRA1 | GLRA1 |
| 5 | 151338458 | 151650010 | CTB-12O2.1 | | | | | |
| 5 | 151771101 | 151784840 | NMUR2 | | | | | |
| 5 | 151998524 | 152351909 | LINC01470 | | | | | |
| 5 | 152870083 | 153193429 | GRIA1 | | GRIA1 | GRIA1 | GRIA1 | GRIA1 |
| 5 | 153369690 | 153418498 | FAM114A2 | | | | | |
| 5 | 153418518 | 153437014 | MFAP3 | | | | | |
| 5 | 153570294 | 153800543 | GALNT10 | | | | | |
| 5 | 153726665 | 153726807 | MIR1294 | | | | | |
| 5 | 153769328 | 153840613 | SAP30L | | | | | |
| 5 | 153854531 | 153857824 | HAND1 | | | | | |
| 5 | 153975571 | 153975632 | MIR3141 | | | | | |
| 5 | 154065335 | 154065421 | MIR1303 | | | | | |
| 5 | 154092461 | 154197167 | LARP1 | | | | | |
| 5 | 154198051 | 154230245 | FAXDC2 | | | | | |
| 5 | 154209017 | 154209100 | MIR378H | | | | | |
| 5 | 154237808 | 154256352 | CNOT8 | | | | | |
| 5 | 154266975 | 154317776 | GEMIN5 | | | | | |
| 5 | 154320632 | 154348971 | MRPL22 | | | | | |
| 5 | 154393259 | 154397685 | KIF4B | | | | | |
| 5 | 155753766 | 156194798 | SGCD | | | | | |
| 5 | 156277548 | 156279539 | PPP1R2P3 | | | | | |
| 5 | 156346292 | 156390266 | TIMD4 | | | | | |
| 5 | 156456423 | 156486130 | HAVCR1 | | | | | |
| 5 | 156512842 | 156536248 | HAVCR2 | | HAVCR2 | | | |
| 5 | 156565450 | 156569921 | MED7 | | | | | |
| 5 | 156589343 | 156593279 | FAM71B | | | | | |
| 5 | 156607906 | 156682109 | ITK | | | | | |
| 5 | 156693089 | 156822606 | CYFIP2 | | | | | |
| 5 | 156768606 | 156772729 | FNDC9 | | | | | |
| 5 | 156887026 | 156901730 | NIPAL4 | | NIPAL4 | | | |
| 5 | 156904311 | 157002831 | ADAM19 | | | ADAM19 | | |
| 5 | 157052686 | 157098488 | SOX30 | | | | | |
| 5 | 157098560 | 157107162 | C5orf52 | | | | | |
| 5 | 157158396 | 157168455 | THG1L | | | | | |
| 5 | 157170702 | 157187717 | LSM11 | | | | | |
| 5 | 157212750 | 157286183 | CLINT1 | | | | | |
| 5 | 157747711 | 157836781 | LOC101927697 | | | | | |
| 5 | 158122922 | 158526788 | EBF1 | | | | | |
| 5 | 158527490 | 158544486 | LOC101927740 | | | | | |
| 5 | 158584416 | 158637061 | RNF145 | | | | | |
| 5 | 158654722 | 158672135 | LOC105377682 | | | | | |
| 5 | 158690088 | 158713048 | UBLCP1 | | | | | |
| 5 | 158741790 | 158757481 | IL12B | | | IL12B | | |
| 5 | 158758525 | 158789842 | LOC285626 | | | | | |
| 5 | 158875563 | 158893284 | LOC285627 | | | | | |
| 5 | 159203781 | 159298391 | LOC101927766 | | | | | |
| 5 | 159343739 | 159400017 | ADRA1B | | | ADRA1B | | |
| 5 | 159436106 | 159492552 | TTC1 | | | | | |
| 5 | 159502891 | 159546452 | PWWP2A | | | | | |
| 5 | 159614373 | 159665729 | FABP6 | | FABP6 | | | |
| 5 | 159678658 | 159766599 | CCNJL | | | | | |
| 5 | 159774774 | 159797648 | C1QTNF2 | | | | | |

| | | | | | | | | | |
|---|---|---|---|---|---|---|---|---|---|
| 5 | 159820154 | 159827104 | ZBED8 | | | | | | |
| 5 | 159828647 | 159846168 | SLU7 | | | | | | |
| 5 | 159848813 | 159855751 | PTTG1 | | | | | | |
| 5 | 159895257 | 159914433 | LOC285628 | | | | | | |
| 5 | 159901408 | 159901490 | MIR3142 | | | | | | |
| 5 | 159912358 | 159912457 | MIR146A | | | | | | |
| 5 | 159990126 | 160279219 | ATP10B | | | | | | |
| 5 | 160358785 | 160365633 | LOC285629 | | | | | | |
| 5 | 160715435 | 160975130 | GABRB2 | | GABRB2 | GABRB2 | GABRB2 | GABRB2 | |
| 5 | 161112657 | 161129598 | GABRA6 | | | | | | |
| 5 | 161274196 | 161326965 | GABRA1 | | GABRA1 | GABRA1 | GABRA1 | GABRA1 | |
| 5 | 161337467 | 161428202 | LINC01202 | | | | | | |
| 5 | 161494647 | 161582545 | GABRG2 | | GABRG2 | | GABRG2 | GABRG2 | |
| 5 | 162864576 | 162872022 | CCNG1 | | | | | | |
| 5 | 162880585 | 162887143 | NUDCD2 | | | | | | |
| 5 | 162887516 | 162921064 | HMMR | | | | | | |
| 5 | 162930069 | 162946359 | MAT2B | | | | | | |
| 5 | 163875427 | 163894408 | LOC101927835 | | | | | | |
| 5 | 163897284 | 163969989 | LOC102546299 | | | | | | |
| 5 | 166332226 | 166353375 | CTB-7E3.1 | | | | | | |
| 5 | 166591938 | 166595406 | LOC101927908 | | | | | | |
| 5 | 166711842 | 167691162 | TENM2 | | | | | | |
| 5 | 167656587 | 167659377 | CTB-178M22.2 | | | | | | |
| 5 | 167719064 | 167899308 | WWC1 | | | | | | |
| 5 | 167913462 | 167946309 | RARS | | | | | | |
| 5 | 167956581 | 167957639 | FBLL1 | | | | | | |
| 5 | 167982627 | 168006614 | PANK3 | | | | | | |
| 5 | 167987900 | 167987978 | MIR103A1 | | | | | | |
| 5 | 167987908 | 167987970 | MIR103B1 | | | | | | |
| 5 | 168088737 | 168728133 | SLIT3 | | | SLIT3 | SLIT3 | | |
| 5 | 168133931 | 168147889 | LOC101927969 | | | | | | |
| 5 | 168195150 | 168195260 | MIR218 | | | | | | |
| 5 | 168440231 | 168465003 | LOC728095 | | | | | | |
| 5 | 168690604 | 168690698 | MIR585 | | | | | | |
| 5 | 169010637 | 169031781 | SPDL1 | | | | | | |
| 5 | 169064250 | 169510386 | DOCK2 | | | | | | |
| 5 | 169290718 | 169407744 | FAM196B | | | | | | |
| 5 | 169455491 | 169455570 | MIR378E | | | | | | |
| 5 | 169532916 | 169536729 | FOXI1 | | | | | | |
| 5 | 169618650 | 169626145 | LINC01187 | | | | | | |
| 5 | 169659920 | 169673235 | C5orf58 | | | | | | |
| 5 | 169675087 | 169724822 | LCP2 | | | | | | |
| 5 | 169758396 | 169762104 | LINC01366 | | | | | | |
| 5 | 169780490 | 170163637 | KCNIP1 | | KCNIP1 | KCNIP1 | | | |
| 5 | 169805164 | 169816681 | KCNMB1 | | | KCNMB1 | KCNMB1 | | |
| 5 | 169816496 | 169849848 | CTD-2270F17.1 | | | | | | |
| 5 | 169931039 | 170163637 | KCNIP1 | | KCNIP1 | KCNIP1 | | | |
| 5 | 170210722 | 170241050 | GABRP | | | GABRP | | | |
| 5 | 170288885 | 170727019 | RANBP17 | | | | | | |
| 5 | 170736287 | 170739138 | TLX3 | | | | | | |
| 5 | 170813659 | 170813764 | MIR3912 | | | | | | |
| 5 | 170814707 | 170837888 | NPM1 | | | | | | |
| 5 | 170846666 | 170884630 | FGF18 | | | | | | |
| 5 | 171212875 | 171218092 | SMIM23 | | | | | | |
| 5 | 171288555 | 171433877 | FBXW11 | | FBXW11 | FBXW11 | | | |
| 5 | 171469073 | 171615346 | STK10 | | | | | | |
| 5 | 171621175 | 171630458 | EFCAB9 | | | | | | |
| 5 | 171636649 | 171710795 | UBTD2 | | | | | | |
| 5 | 171712600 | 171725380 | LOC100288254 | | | | | | |
| 5 | 171752186 | 171881527 | SH3PXD2B | | | | | | |
| 5 | 172068268 | 172118533 | NEURL1B | | | | | | |
| 5 | 172182505 | 172189565 | LOC101928093 | | | | | | |
| 5 | 172195092 | 172198203 | DUSP1 | | | | | | |
| 5 | 172261222 | 172379688 | ERGIC1 | | | | | | |
| 5 | 172381785 | 172386371 | LOC100268168 | | | | | | |
| 5 | 172385731 | 172396774 | RPL26L1 | | | | | | |

| | | | | | | | | |
|---|---|---|---|---|---|---|---|---|
| 5 | 172410762 | 172461900 | ATP6V0E1 | | | | | |
| 5 | 172447728 | 172447932 | SNORA74B | | | | | |
| 5 | 172483354 | 172566291 | CREBRF | | | | | |
| 5 | 172571444 | 172591390 | BNIP1 | | | | | |
| 5 | 172659106 | 172662315 | NKX2-5 | | | NKX2-5 | | |
| 5 | 172741725 | 172756506 | STC2 | | | | | |
| 5 | 172774457 | 172774539 | MIR8056 | | | | | |
| 5 | 173006636 | 173012071 | LOC285593 | | | | | |
| 5 | 173034147 | 173043666 | BOD1 | | | | | |
| 5 | 173134601 | 173173212 | LINC01484 | | | | | |
| 5 | 173213792 | 173217945 | LINC01485 | | | | | |
| 5 | 173315330 | 173387994 | CPEB4 | | | | | |
| 5 | 173416161 | 173433143 | C5orf47 | | | | | |
| 5 | 173472606 | 173536182 | HMP19 | | | HMP19 | | |
| 5 | 173763356 | 173954142 | LINC01411 | | | | | |
| 5 | 174151574 | 174157902 | MSX2 | | | | | |
| 5 | 174178736 | 174178790 | MIR4634 | | | | | |
| 5 | 174346084 | 174422734 | FLJ16171 | | | | | |
| 5 | 174867674 | 174871163 | DRD1 | DRD1 | DRD1 | DRD1 | DRD1 | |
| 5 | 174905513 | 174955621 | SFXN1 | | | | | |
| 5 | 175085039 | 175111558 | HRH2 | | | | | |
| 5 | 175223609 | 175311023 | CPLX2 | | | CPLX2 | | |
| 5 | 175386533 | 175395318 | THOC3 | | | | | |
| 5 | 175476680 | 175489058 | LOC100996385 | | | | | |
| 5 | 175490711 | 175541801 | FAM153B | | | | | |
| 5 | 175546551 | 175552168 | LOC100507387 | | | | | |
| 5 | 175570087 | 175626298 | LOC643201 | | | | | |
| 5 | 175665361 | 175772994 | SIMC1 | | | | | |
| 5 | 175773063 | 175788810 | KIAA1191 | | | | | |
| 5 | 175792470 | 175800503 | ARL10 | | | | | |
| 5 | 175794948 | 175795034 | MIR1271 | | | | | |
| 5 | 175810939 | 175815928 | NOP16 | | | | | |
| 5 | 175815783 | 175816751 | HIGD2A | | | | | |
| 5 | 175819455 | 175843570 | CLTB | | | | | |
| 5 | 175875355 | 175937075 | FAF2 | | | | | |
| 5 | 175953699 | 175964421 | RNF44 | | | | | |
| 5 | 175969511 | 176022769 | CDHR2 | | | | | |
| 5 | 176022802 | 176037131 | GPRIN1 | | | | | |
| 5 | 176047084 | 176057562 | SNCB | | SNCB | SNCB | SNCB | |
| 5 | 176056439 | 176056501 | MIR4281 | | | | | |
| 5 | 176057682 | 176073642 | EIF4E1B | | | | | |
| 5 | 176074387 | 176086059 | TSPAN17 | | | | | |
| 5 | 176170205 | 176170922 | LINC01574 | | | | | |
| 5 | 176237559 | 176307899 | UNC5A | | | | | |
| 5 | 176307869 | 176326333 | HK3 | | | | | |
| 5 | 176332005 | 176433795 | UIMC1 | | | | | |
| 5 | 176449680 | 176508190 | ZNF346 | | | | | |
| 5 | 176513872 | 176525143 | FGFR4 | | | | | |
| 5 | 176560079 | 176727214 | NSD1 | | | NSD1 | | |
| 5 | 176728190 | 176730745 | RAB24 | | | RAB24 | | |
| 5 | 176730762 | 176733960 | PRELID1 | | | | | |
| 5 | 176732500 | 176739292 | MXD3 | | | | | |
| 5 | 176758562 | 176778885 | LMAN2 | | | | | |
| 5 | 176784843 | 176799599 | RGS14 | | | | | |
| 5 | 176811431 | 176825849 | SLC34A1 | | | | | |
| 5 | 176827107 | 176827637 | PFN3 | | | | | |
| 5 | 176829138 | 176836577 | F12 | | | | | |
| 5 | 176853686 | 176869850 | GRK6 | | | | | |
| 5 | 176864889 | 176883283 | PRR7 | | | | | |
| 5 | 176883613 | 176900694 | DBN1 | | | DBN1 | | |
| 5 | 176910394 | 176924606 | PDLIM7 | | | | | |
| 5 | 176928905 | 176937427 | DOK3 | | | | | |
| 5 | 176938577 | 176943967 | DDX41 | | | | | |
| 5 | 176946789 | 176981586 | FAM193B | | | | | |
| 5 | 177019212 | 177023099 | TMED9 | | | | | |
| 5 | 177027118 | 177037346 | B4GALT7 | | | | | |

| | | | | | | | | |
|---|---|---|---|---|---|---|---|---|
| 5 | 177045500 | 177099278 | LOC202181 | | | | | |
| 5 | 177150364 | 177207505 | FAM153A | | | | | |
| 5 | 177302261 | 177311269 | LOC728554 | | | | | |
| 5 | 177419235 | 177423243 | PROP1 | | | | | |
| 5 | 177433966 | 177476088 | FAM153C | | | | | |
| 5 | 177540555 | 177553107 | N4BP3 | | | | | |
| 5 | 177557961 | 177575571 | RMND5B | | | | | |
| 5 | 177576464 | 177580961 | NHP2 | | | | | |
| 5 | 177611510 | 177614433 | GMCL1P1 | | | | | |
| 5 | 177631507 | 177638184 | HNRNPAB | | | | | |
| 5 | 177635474 | 177659823 | PHYKPL | | | | | |
| 5 | 177664616 | 178017556 | COL23A1 | | | | | |
| 5 | 178029664 | 178054054 | CLK4 | | | CLK4 | | |
| 5 | 178138521 | 178157703 | ZNF354A | | | | | |
| 5 | 178191861 | 178245436 | AACSP1 | | | | | |
| 5 | 178286953 | 178311424 | ZNF354B | | | | | |
| 5 | 178322894 | 178360212 | ZFP2 | | | | | |
| 5 | 178368193 | 178393218 | ZNF454 | | | | | |
| 5 | 178405329 | 178422124 | GRM6 | | | GRM6 | GRM6 | |
| 5 | 178450775 | 178461388 | ZNF879 | | | | | |
| 5 | 178487415 | 178510978 | ZNF354C | | | | | |
| 5 | 178537851 | 178772431 | ADAMTS2 | | | | | |
| 5 | 178977561 | 179037027 | RUFY1 | | | | | |
| 5 | 179022904 | 179030742 | LOC101928445 | | | | | |
| 5 | 179041178 | 179050722 | HNRNPH1 | | | | | |
| 5 | 179068544 | 179072047 | C5orf60 | | | | | |
| 5 | 179084762 | 179101296 | LOC105377763 | | | | | |
| 5 | 179105558 | 179107975 | CBY3 | | | | | |
| 5 | 179125929 | 179158639 | CANX | | | | | |
| 5 | 179159850 | 179204287 | MAML1 | | | | | |
| 5 | 179220985 | 179223513 | LTC4S | | | LTC4S | | |
| 5 | 179224597 | 179233952 | MGAT4B | | | | | |
| 5 | 179225277 | 179225346 | MIR1229 | | | | | |
| 5 | 179233387 | 179265077 | SQSTM1 | | | | | |
| 5 | 179264265 | 179285840 | C5orf45 | | | | | |
| 5 | 179286002 | 179288282 | LOC100996419 | | | | | |
| 5 | 179289070 | 179334856 | TBC1D9B | | | | | |
| 5 | 179382066 | 179499118 | RNF130 | | | RNF130 | | |
| 5 | 179442302 | 179442397 | MIR340 | | | | | |
| 5 | 179527794 | 179636130 | RASGEF1C | | | | | |
| 5 | 179660594 | 179719071 | MAPK9 | | MAPK9 | | | |
| 5 | 179727689 | 179780387 | GFPT2 | | | | | |
| 5 | 179921398 | 180005405 | CNOT6 | | | | | |
| 5 | 180017104 | 180018487 | SCGB3A1 | | | | | |
| 5 | 180028505 | 180076624 | FLT4 | | | FLT4 | | |
| 5 | 180166122 | 180167058 | OR2Y1 | | | | | |
| 5 | 180217540 | 180237137 | MGAT1 | | | | | |
| 5 | 180256953 | 180258618 | HEIH | | | | | |
| 5 | 180257956 | 180262726 | LINC00847 | | | | | |
| 5 | 180274610 | 180288286 | ZFP62 | | | | | |
| 5 | 180326076 | 180377906 | BTNL8 | | | | | |
| 5 | 180415844 | 180433727 | BTNL3 | | | | | |
| 5 | 180467224 | 180488523 | BTNL9 | | | | | |
| 5 | 180470402 | 180470484 | MIR8089 | | | | | |
| 5 | 180551356 | 180552304 | OR2V1 | | | | | |
| 5 | 180581942 | 180582890 | OR2V2 | | | | | |
| 5 | 180618045 | 180618908 | LOC102577426 | | | | | |
| 5 | 180620923 | 180632293 | TRIM7 | | | | | |
| 5 | 180649565 | 180649633 | MIR4638 | | | | | |
| 5 | 180650262 | 180662808 | TRIM41 | | | | | |
| 5 | 180663927 | 180670906 | GNB2L1 | | | GNB2L1 | | |
| 5 | 180668817 | 180668889 | SNORD96A | | | | | |
| 5 | 180670313 | 180670376 | SNORD95 | | | | | |
| 5 | 180673540 | 180684587 | CTC-338M12.4 | | | | | |
| 5 | 180683385 | 180699308 | TRIM52 | | | | | |
| 5 | 180750506 | 180755196 | LOC100132062 | | | | | |

| | | | | | | | | |
|---|---|---|---|---|---|---|---|---|
| 5 | 180750506 | 180755196 | LOC100132287 | | | | | |
| 5 | 180750506 | 180755196 | LOC100133331 | | | | | |
| 5 | 180794287 | 180795226 | OR4F16 | | | | | |
| 5 | 180794287 | 180795226 | OR4F29 | | | | | |
| 5 | 180794287 | 180795226 | OR4F3 | | | | | |
| 6 | 140263 | 148159 | LINC00266-3 | | | | | |
| 6 | 181465 | 205484 | LOC285766 | | | | | |
| 6 | 292056 | 351355 | DUSP22 | | | | | |
| 6 | 391738 | 411443 | IRF4 | | | | | |
| 6 | 485137 | 693141 | EXOC2 | | | | | |
| 6 | 655938 | 656964 | HUS1B | | | | | |
| 6 | 868497 | 876613 | LOC101927691 | | | | | |
| 6 | 961240 | 1101567 | LINC01622 | | | | | |
| 6 | 1312674 | 1314993 | FOXQ1 | | | | | |
| 6 | 1390068 | 1395832 | FOXF2 | | | | | |
| 6 | 1390548 | 1390646 | MIR6720 | | | | | |
| 6 | 1605765 | 1607591 | FOXCUT | | | | | |
| 6 | 1610680 | 1614129 | FOXC1 | | | FOXC1 | | |
| 6 | 1624034 | 2413825 | GMDS | | | | | |
| 6 | 2622146 | 2634837 | LINC01600 | | | | | |
| 6 | 2663862 | 2751154 | MYLK4 | | | | | |
| 6 | 2765665 | 2785979 | WRNIP1 | | | | | |
| 6 | 2832565 | 2842283 | SERPINB1 | | | | | |
| 6 | 2854264 | 2854341 | MIR4645 | | | | | |
| 6 | 2854890 | 2876744 | SERPINB9P1 | | | | | |
| 6 | 2884221 | 2900910 | LOC101927730 | | | | | |
| 6 | 2887499 | 2903546 | SERPINB9 | | | | | |
| 6 | 2948392 | 2972399 | SERPINB6 | | | | | |
| 6 | 2988200 | 2991405 | LINC01011 | | | | | |
| 6 | 3000049 | 3020110 | NQO2 | | | | | |
| 6 | 3020389 | 3025005 | HTATSF1P2 | | | | | |
| 6 | 3025024 | 3027658 | LOC101927759 | | | | | |
| 6 | 3064121 | 3115421 | RIPK1 | | | RIPK1 | | |
| 6 | 3118609 | 3153432 | BPHL | | | | | |
| 6 | 3153900 | 3157783 | TUBB2A | | TUBB2A | | | |
| 6 | 3183051 | 3196001 | LOC100507194 | | | | | |
| 6 | 3224494 | 3227968 | TUBB2B | | TUBB2B | | | |
| 6 | 3259161 | 3268300 | PSMG4 | | | | | |
| 6 | 3269206 | 3456793 | SLC22A23 | | | | | |
| 6 | 3722835 | 3752246 | PXDC1 | | | | | |
| 6 | 3849599 | 3851554 | FAM50B | | | | | |
| 6 | 4021568 | 4065217 | PRPF4B | | | | | |
| 6 | 4068592 | 4079457 | FAM217A | | | | | |
| 6 | 4079439 | 4130999 | C6orf201 | | | | | |
| 6 | 4115926 | 4135831 | ECI2 | | | | | |
| 6 | 4136305 | 4157619 | LOC100507506 | | | | | |
| 6 | 4186553 | 4190263 | LOC102724096 | | | | | |
| 6 | 4428235 | 4428285 | MIR7641 | | | | | |
| 6 | 4610798 | 4612153 | KU-MEL-3 | | | | | |
| 6 | 4706392 | 4955778 | CDYL | | | | | |
| 6 | 4995279 | 5004297 | RPP40 | | | | | |
| 6 | 5004049 | 5261183 | LYRM4 | | | | | |
| 6 | 5085719 | 5087455 | PPP1R3G | | | | | |
| 6 | 5108652 | 5261183 | LYRM4 | | | | | |
| 6 | 5148466 | 5148556 | MIR3691 | | | | | |
| 6 | 5186833 | 5261183 | LYRM4 | | | | | |
| 6 | 5261250 | 5771825 | FARS2 | | | | | |
| 6 | 5452700 | 5458308 | LOC101927972 | | | | | |
| 6 | 5665219 | 5695505 | LOC101927950 | | | | | |
| 6 | 5998232 | 6007838 | NRN1 | | | | | |
| 6 | 6144310 | 6320924 | F13A1 | | | | | |
| 6 | 6169566 | 6169642 | MIR5683 | | | | | |
| 6 | 6346697 | 6655216 | LY86 | | | | | |
| 6 | 7107829 | 7252213 | RREB1 | | | | | |
| 6 | 7281375 | 7313547 | SSR1 | | | | | |
| 6 | 7326886 | 7389942 | CAGE1 | | | | | |

| | | | | | | | | |
|---|---|---|---|---|---|---|---|---|
| 6 | 7390061 | 7418270 | RIOK1 | | | RIOK1 | | |
| 6 | 7541807 | 7586950 | DSP | | | | | |
| 6 | 7590431 | 7612200 | SNRNP48 | | | | | |
| 6 | 7727010 | 7881961 | BMP6 | | | | | |
| 6 | 7881482 | 7911047 | TXNDC5 | | | | | |
| 6 | 7881482 | 8064647 | BLOC1S5-TXNDC5 | | | | | |
| 6 | 7986334 | 7990577 | PIP5K1P1 | | | | | |
| 6 | 8013799 | 8064647 | BLOC1S5 | | | | | |
| 6 | 8013799 | 8102828 | EEF1E1-BLOC1S5 | | | | | |
| 6 | 8073592 | 8102828 | EEF1E1 | | | | | |
| 6 | 8086640 | 8086766 | SCARNA27 | | | | | |
| 6 | 8413299 | 8435800 | SLC35B3 | | | | | |
| 6 | 8435855 | 8785678 | LOC100506207 | | | | | |
| 6 | 8652441 | 8654080 | HULC | | | | | |
| 6 | 10396915 | 10416402 | TFAP2A | | | TFAP2A | | |
| 6 | 10428017 | 10435055 | LINC00518 | | | | | |
| 6 | 10434561 | 10457012 | MIR5689HG | | | | | |
| 6 | 10439949 | 10440027 | MIR5689 | | | | | |
| 6 | 10521567 | 10629601 | GCNT2 | | | | | |
| 6 | 10671650 | 10695030 | C6orf52 | | | | | |
| 6 | 10695187 | 10709970 | PAK1IP1 | | | PAK1IP1 | | |
| 6 | 10723147 | 10731362 | TMEM14C | | | | | |
| 6 | 10747991 | 10760006 | TMEM14B | | | | | |
| 6 | 10762955 | 10838788 | MAK | | | | | |
| 6 | 10873455 | 10882098 | GCM2 | | | | | |
| 6 | 10887063 | 10974541 | SYCP2L | | | | | |
| 6 | 10932549 | 10954322 | LOC101928191 | | | | | |
| 6 | 10980992 | 11079377 | ELOVL2 | | | | | |
| 6 | 11094265 | 11138969 | SMIM13 | | | | | |
| 6 | 11102721 | 11112071 | ERVFRD-1 | | | | | |
| 6 | 11183530 | 11232915 | NEDD9 | | | NEDD9 | | |
| 6 | 11538459 | 11583757 | TMEM170B | | | | | |
| 6 | 11713887 | 11779280 | ADTRP | | | | | |
| 6 | 11990575 | 12001477 | LOC101928253 | | | | | |
| 6 | 12012723 | 12165232 | HIVEP1 | | | | | |
| 6 | 12290528 | 12297427 | EDN1 | | | | | |
| 6 | 12717036 | 13288075 | PHACTR1 | | | | | |
| 6 | 13266773 | 13328815 | TBC1D7-LOC100130357 | | | | | |
| 6 | 13273325 | 13283674 | LOC100130357 | | | | | |
| 6 | 13275080 | 13328815 | TBC1D7-LOC100130357 | | | | | |
| 6 | 13279526 | 13295818 | LOC100130357 | | | | | |
| 6 | 13305183 | 13328815 | TBC1D7 | | TBC1D7 | | | |
| 6 | 13363586 | 13487869 | GFOD1 | | | | | |
| 6 | 13574760 | 13615390 | SIRT5 | | | | | |
| 6 | 13615558 | 13632702 | NOL7 | | | | | |
| 6 | 13621729 | 13711796 | RANBP9 | | | RANBP9 | | |
| 6 | 13786780 | 13814792 | MCUR1 | | | | | |
| 6 | 13924676 | 13980240 | RNF182 | | | | | |
| 6 | 14117486 | 14137148 | CD83 | | | | | |
| 6 | 14280357 | 14285685 | LINC01108 | | | | | |
| 6 | 15246205 | 15522273 | JARID2 | | | | | |
| 6 | 15523031 | 15663289 | DTNBP1 | | DTNBP1 | DTNBP1 | | |
| 6 | 16129316 | 16148478 | MYLIP | | | | | |
| 6 | 16141786 | 16141855 | MIR4639 | | | | | |
| 6 | 16238810 | 16295780 | GMPR | | | | | |
| 6 | 16299342 | 16761721 | ATXN1 | ATXN1 | | ATXN1 | | |
| 6 | 17102488 | 17131603 | STMND1 | | | | | |
| 6 | 17281808 | 17294099 | RBM24 | | | | | |
| 6 | 17393735 | 17558023 | CAP2 | | | | | |
| 6 | 17501595 | 17511496 | LOC101928491 | | | | | |
| 6 | 17600517 | 17611950 | FAM8A1 | | | | | |
| 6 | 17615265 | 17707065 | NUP153 | | | | | |
| 6 | 17706360 | 17711258 | LOC105374952 | | | | | |
| 6 | 17759413 | 17987854 | KIF13A | | | KIF13A | | |
| 6 | 18120717 | 18122851 | NHLRC1 | | | NHLRC1 | | |
| 6 | 18128544 | 18155396 | TPMT | | | | | |

| | | | | | | | | |
|---|---|---|---|---|---|---|---|---|
| 6 | 18155618 | 18224084 | KDM1B | | | | | |
| 6 | 18224399 | 18264799 | DEK | | | | | |
| 6 | 18387580 | 18469105 | RNF144B | | | | | |
| 6 | 18572014 | 18572111 | MIR548A1 | | | | | |
| 6 | 19068773 | 19180711 | LOC101928519 | | | | | |
| 6 | 19450051 | 19459579 | LOC105374960 | | | | | |
| 6 | 19729651 | 19804990 | LOC100506885 | | | | | |
| 6 | 19837600 | 19842431 | ID4 | | | | | |
| 6 | 20099915 | 20212695 | MBOAT1 | | | | | |
| 6 | 20402136 | 20493945 | E2F3 | | | | | |
| 6 | 20534687 | 21232634 | CDKAL1 | | | | | |
| 6 | 21486291 | 21512123 | LINC00581 | | | | | |
| 6 | 21593971 | 21598849 | SOX4 | | | | | |
| 6 | 21666674 | 22194616 | CASC15 | | | | | |
| 6 | 22134830 | 22147422 | NBAT1 | | | | | |
| 6 | 22287472 | 22303082 | PRL | | PRL | | | |
| 6 | 22569677 | 22570750 | HDGFL1 | | | | | |
| 6 | 22643738 | 22718153 | LOC105374972 | | | | | |
| 6 | 24126413 | 24147757 | NRSN1 | | | | | |
| 6 | 24171982 | 24383520 | DCDC2 | | | | | |
| 6 | 24357130 | 24358512 | KAAG1 | | | | | |
| 6 | 24403135 | 24426422 | MRS2 | | | | | |
| 6 | 24426061 | 24489850 | GPLD1 | | GPLD1 | | | |
| 6 | 24495196 | 24537435 | ALDH5A1 | | ALDH5A1 | ALDH5A1 | | |
| 6 | 24544331 | 24646383 | KIAA0319 | | KIAA0319 | | | |
| 6 | 24650204 | 24667115 | TDP2 | | | | | |
| 6 | 24667262 | 24705295 | ACOT13 | | | | | |
| 6 | 24705089 | 24719403 | C6orf62 | | | | | |
| 6 | 24775158 | 24786325 | GMNN | | | | | |
| 6 | 24797548 | 24799124 | C6orf229 | | | | | |
| 6 | 24804508 | 25042396 | FAM65B | | | | | |
| 6 | 25081294 | 25138620 | CMAHP | | | | | |
| 6 | 25245145 | 25261635 | LOC101928663 | | | | | |
| 6 | 25279655 | 25620758 | LRRC16A | | | | | |
| 6 | 25652428 | 25702008 | SCGN | | | | | |
| 6 | 25726290 | 25726790 | HIST1H2AA | | | | | |
| 6 | 25727136 | 25727573 | HIST1H2BA | | | | | |
| 6 | 25732660 | 25733089 | HIST1H2APS1 | | | | | |
| 6 | 25754926 | 25781403 | SLC17A4 | | | | | |
| 6 | 25783125 | 25832287 | SLC17A1 | | | | | |
| 6 | 25845327 | 25874471 | SLC17A3 | | | | | |
| 6 | 25912983 | 25930954 | SLC17A2 | | | | | |
| 6 | 25962916 | 25987557 | TRIM38 | | | | | |
| 6 | 26017259 | 26018040 | HIST1H1A | | | | | |
| 6 | 26020717 | 26021186 | HIST1H3A | | | | | |
| 6 | 26021906 | 26022278 | HIST1H4A | | | | | |
| 6 | 26027123 | 26027480 | HIST1H4B | | | | | |
| 6 | 26031816 | 26032288 | HIST1H3B | | | | | |
| 6 | 26033319 | 26033796 | HIST1H2AB | | | | | |
| 6 | 26043454 | 26043885 | HIST1H2BB | | | | | |
| 6 | 26045638 | 26046097 | HIST1H3C | | | | | |
| 6 | 26055967 | 26056699 | HIST1H1C | | | | | |
| 6 | 26087508 | 26095469 | HFE | | | | | |
| 6 | 26104175 | 26104565 | HIST1H4C | | | | | |
| 6 | 26107639 | 26108364 | HIST1H1T | | | | | |
| 6 | 26123694 | 26124132 | HIST1H2BC | | | | | |
| 6 | 26124372 | 26124918 | HIST1H2AC | | | | | |
| 6 | 26156558 | 26157343 | HIST1H1E | | | | | |
| 6 | 26158348 | 26171576 | HIST1H2BD | | | | | |
| 6 | 26184023 | 26184458 | HIST1H2BE | | | | | |
| 6 | 26188937 | 26189304 | HIST1H4D | | | | | |
| 6 | 26197011 | 26199521 | HIST1H3D | | | | | |
| 6 | 26199011 | 26199521 | HIST1H2AD | | | | | |
| 6 | 26199786 | 26200216 | HIST1H2BF | | | | | |
| 6 | 26204872 | 26205249 | HIST1H4E | | | | | |
| 6 | 26216427 | 26216872 | HIST1H2BG | | | | | |

| | | | | | | | | | |
|---|---|---|---|---|---|---|---|---|---|
| 6 | 26217147 | 26217711 | HIST1H2AE | | | | | | |
| 6 | 26225382 | 26225844 | HIST1H3E | | | | | | |
| 6 | 26234439 | 26235216 | HIST1H1D | | | | | | |
| 6 | 26240653 | 26241021 | HIST1H4F | | | | | | |
| 6 | 26246838 | 26247205 | HIST1H4G | | | | | | |
| 6 | 26250369 | 26250835 | HIST1H3F | | | | | | |
| 6 | 26251878 | 26252303 | HIST1H2BH | | | | | | |
| 6 | 26271145 | 26271612 | HIST1H3G | | | | | | |
| 6 | 26273203 | 26273640 | HIST1H2BI | | | | | | |
| 6 | 26285353 | 26285727 | HIST1H4H | | | | | | |
| 6 | 26365386 | 26378548 | BTN3A2 | | | | | | |
| 6 | 26383323 | 26395100 | BTN2A2 | | | | | | |
| 6 | 26402464 | 26415444 | BTN3A1 | | | | | | |
| 6 | 26421618 | 26430816 | BTN2A3P | | | | | | |
| 6 | 26440699 | 26453643 | BTN3A3 | | | | | | |
| 6 | 26458131 | 26476849 | BTN2A1 | | | | | | |
| 6 | 26472171 | 26482737 | LOC285819 | | | | | | |
| 6 | 26501494 | 26510652 | BTN1A1 | | | | | | |
| 6 | 26521933 | 26527612 | HCG11 | | | | | | |
| 6 | 26538571 | 26547164 | HMGN4 | | | | | | |
| 6 | 26569551 | 26574926 | LOC105374988 | | | | | | |
| 6 | 26597170 | 26600277 | ABT1 | | | | | | |
| 6 | 26634610 | 26659980 | ZNF322 | | | | | | |
| 6 | 26839265 | 26924333 | GUSBP2 | | | | | | |
| 6 | 26924771 | 26991753 | LINC00240 | | | | | | |
| 6 | 26987144 | 26988085 | LOC100270746 | | | | | | |
| 6 | 27100094 | 27100575 | HIST1H2BJ | | | | | | |
| 6 | 27100816 | 27101314 | HIST1H2AG | | | | | | |
| 6 | 27106071 | 27114637 | HIST1H2BK | | | | | | |
| 6 | 27107087 | 27107457 | HIST1H4I | | | | | | |
| 6 | 27114139 | 27114637 | HIST1H2BK | | | | | | |
| 6 | 27114860 | 27115341 | HIST1H2AH | | | | | | |
| 6 | 27115404 | 27115467 | MIR3143 | | | | | | |
| 6 | 27215501 | 27224399 | PRSS16 | | | | | | |
| 6 | 27276841 | 27280011 | POM121L2 | | | | | | |
| 6 | 27292539 | 27293741 | VN1R10P | | | | | | |
| 6 | 27325601 | 27343153 | ZNF204P | | | | | | |
| 6 | 27356523 | 27369227 | ZNF391 | | | | | | |
| 6 | 27418520 | 27440897 | ZNF184 | | | | | | |
| 6 | 27661813 | 27676936 | LINC01012 | | | | | | |
| 6 | 27729522 | 27730966 | LOC100131289 | | | | | | |
| 6 | 27775256 | 27775709 | HIST1H2BL | | | | | | |
| 6 | 27775976 | 27776445 | HIST1H2AI | | | | | | |
| 6 | 27777841 | 27778314 | HIST1H3H | | | | | | |
| 6 | 27782079 | 27782518 | HIST1H2AJ | | | | | | |
| 6 | 27782821 | 27783267 | HIST1H2BM | | | | | | |
| 6 | 27791902 | 27792258 | HIST1H4J | | | | | | |
| 6 | 27798951 | 27799305 | HIST1H4K | | | | | | |
| 6 | 27805657 | 27806117 | HIST1H2AK | | | | | | |
| 6 | 27806439 | 27806888 | HIST1H2BN | | | | | | |
| 6 | 27833106 | 27833576 | HIST1H2AL | | | | | | |
| 6 | 27834569 | 27835359 | HIST1H1B | | | | | | |
| 6 | 27839622 | 27840099 | HIST1H3I | | | | | | |
| 6 | 27840925 | 27841289 | HIST1H4L | | | | | | |
| 6 | 27858092 | 27858570 | HIST1H3J | | | | | | |
| 6 | 27860476 | 27860963 | HIST1H2AM | | | | | | |
| 6 | 27861202 | 27861669 | HIST1H2BO | | | | | | |
| 6 | 27878962 | 27880174 | OR2B2 | | | | | | |
| 6 | 27925018 | 27925960 | OR2B6 | | | | | | |
| 6 | 28048481 | 28057340 | ZNF165 | | | | | | |
| 6 | 28058584 | 28063493 | ZSCAN12P1 | | | | | | |
| 6 | 28089572 | 28097856 | ZSCAN16 | | | | | | |
| 6 | 28109687 | 28127250 | ZKSCAN8 | | | | | | |
| 6 | 28129538 | 28137373 | ZNF192P1 | | | | | | |
| 6 | 28183115 | 28186707 | TOB2P1 | | | | | | |
| 6 | 28193028 | 28201264 | ZSCAN9 | | | | | | |

| | | | | | | | | |
|---|---|---|---|---|---|---|---|---|
| 6 | 28209482 | 28227030 | ZKSCAN4 | | | | | |
| 6 | 28227074 | 28228736 | NKAPL | | | | | |
| 6 | 28234787 | 28246000 | ZSCAN26 | | | | | |
| 6 | 28249313 | 28270326 | PGBD1 | | | | | |
| 6 | 28292514 | 28324048 | ZSCAN31 | | | | | |
| 6 | 28317690 | 28336954 | ZKSCAN3 | | | | | |
| 6 | 28346597 | 28367544 | ZSCAN12 | | | | | |
| 6 | 28400431 | 28411279 | ZSCAN23 | | | | | |
| 6 | 28471072 | 28483570 | GPX6 | | | | | |
| 6 | 28493788 | 28502728 | GPX5 | | | | | |
| 6 | 28539406 | 28555112 | ZBED9 | | | | | |
| 6 | 28827401 | 28831454 | LINC01623 | | | | | |
| 6 | 28864306 | 28865097 | HCG14 | | | | | |
| 6 | 28870778 | 28891768 | TRIM27 | | | | | |
| 6 | 28911560 | 28912315 | LINC01556 | | | | | |
| 6 | 28962561 | 28971842 | ZNF311 | | | | | |
| 6 | 29003797 | 29044517 | LOC100129636 | | | | | |
| 6 | 29011989 | 29012952 | OR2W1 | | | OR2W1 | | |
| 6 | 29053984 | 29055090 | OR2B3 | | | | | |
| 6 | 29079586 | 29080661 | OR2J3 | | | | | |
| 6 | 29141310 | 29142351 | OR2J2 | | | | | |
| 6 | 29191749 | 29258217 | LOC101929006 | | | | | |
| 6 | 29274466 | 29275432 | OR14J1 | | | | | |
| 6 | 29323006 | 29324054 | OR5V1 | | | | | |
| 6 | 29341199 | 29343068 | OR12D3 | | | | | |
| 6 | 29364415 | 29365448 | OR12D2 | | | | | |
| 6 | 29393280 | 29395509 | OR11A1 | | | | | |
| 6 | 29407715 | 29408754 | OR10C1 | | | | | |
| 6 | 29424931 | 29432099 | OR2H1 | | | | | |
| 6 | 29454542 | 29455679 | MAS1L | | | | | |
| 6 | 29497182 | 29501345 | LINC01015 | | | | | |
| 6 | 29523388 | 29527702 | UBD | | | | | |
| 6 | 29550028 | 29550105 | SNORD32B | | | | | |
| 6 | 29555682 | 29556745 | OR2H2 | | | | | |
| 6 | 29570004 | 29600912 | GABBR1 | GABBR1 | GABBR1 | GABBR1 | GABBR1 | GABBR1 |
| 6 | 29624757 | 29640149 | MOG | | MOG | MOG | | |
| 6 | 29640168 | 29644931 | ZFP57 | | | | | |
| 6 | 29691116 | 29695073 | HLA-F | | | | | |
| 6 | 29694377 | 29716826 | HLA-F-AS1 | | | | | |
| 6 | 29718583 | 29718925 | IFITM4P | | | | | |
| 6 | 29758807 | 29760850 | HCG4 | | | | | |
| 6 | 29759682 | 29765584 | LOC554223 | | | | | |
| 6 | 29794755 | 29798899 | HLA-G | | | | | |
| 6 | 29855536 | 29858857 | HLA-H | | | | | |
| 6 | 29892368 | 29894992 | HCG4B | | | | | |
| 6 | 29910246 | 29913661 | HLA-A | | | | | |
| 6 | 29942891 | 29946177 | HCG9 | | | | | |
| 6 | 29968787 | 30028961 | ZNRD1 | | | | | |
| 6 | 29973747 | 29977733 | HLA-J | | | | | |
| 6 | 29979877 | 29981699 | HCG8 | | | | | |
| 6 | 30029016 | 30032686 | ZNRD1 | | | | | |
| 6 | 30034931 | 30038108 | PPP1R11 | | | | | |
| 6 | 30038042 | 30043628 | RNF39 | | | | | |
| 6 | 30070673 | 30082501 | TRIM31 | | | | | |
| 6 | 30103884 | 30116512 | TRIM40 | | | | | |
| 6 | 30119722 | 30128711 | TRIM10 | | | TRIM10 | | |
| 6 | 30130982 | 30140473 | TRIM15 | | | | | |
| 6 | 30152231 | 30181271 | TRIM26 | | | | | |
| 6 | 30201815 | 30293911 | HCG17 | | | | | |
| 6 | 30227338 | 30234728 | HLA-L | | | | | |
| 6 | 30229276 | 30229317 | MIR6891 | | | | | |
| 6 | 30255173 | 30294933 | HCG18 | | | | | |
| 6 | 30294620 | 30311506 | TRIM39 | | | | | |
| 6 | 30297087 | 30314635 | TRIM39-RPP21 | | | | | |
| 6 | 30312905 | 30314635 | RPP21 | | | | | |
| 6 | 30457182 | 30461982 | HLA-E | | | | | |

| | | | | | | | | |
|---|---|---|---|---|---|---|---|---|
| 6 | 30509154 | 30525371 | GNL1 | | | | | |
| 6 | 30524485 | 30532473 | PRR3 | | | | | |
| 6 | 30539169 | 30559309 | ABCF1 | | | | | |
| 6 | 30552108 | 30552194 | MIR877 | | | | | |
| 6 | 30568176 | 30585084 | PPP1R10 | | | | | |
| 6 | 30585485 | 30594174 | MRPS18B | | | | | |
| 6 | 30594618 | 30614600 | ATAT1 | | | | | |
| 6 | 30614815 | 30620987 | C6orf136 | | | | | |
| 6 | 30620895 | 30640830 | DHX16 | | | | | |
| 6 | 30644165 | 30655672 | PPP1R18 | | | | | |
| 6 | 30655823 | 30659197 | NRM | | | | | |
| 6 | 30667583 | 30680961 | MDC1 | | | MDC1 | | |
| 6 | 30687977 | 30693203 | TUBB | | | TUBB | | |
| 6 | 30695485 | 30710628 | FLOT1 | | | | | |
| 6 | 30710975 | 30712327 | IER3 | | | | | |
| 6 | 30780642 | 30798436 | LINC00243 | | | | | |
| 6 | 30807305 | 30815936 | LOC105375014 | | | | | |
| 6 | 30850389 | 30867933 | DDR1 | | DDR1 | | | |
| 6 | 30858659 | 30858749 | MIR4640 | | | | | |
| 6 | 30875976 | 30881880 | GTF2H4 | | | | | |
| 6 | 30881984 | 30894235 | VARS2 | | | | | |
| 6 | 30899126 | 30899952 | SFTA2 | | | | | |
| 6 | 30908776 | 30921998 | DPCR1 | | | | | |
| 6 | 30951484 | 30956414 | MUC21 | | | | | |
| 6 | 30973728 | 31003179 | MUC22 | | | | | |
| 6 | 31021226 | 31027655 | HCG22 | | | | | |
| 6 | 31078999 | 31080332 | C6orf15 | | | | | |
| 6 | 31082607 | 31107869 | PSORS1C1 | | | | | |
| 6 | 31082864 | 31088252 | CDSN | | | | | |
| 6 | 31105310 | 31107127 | PSORS1C2 | | | | | |
| 6 | 31110215 | 31126015 | CCHCR1 | | | | | |
| 6 | 31126300 | 31131992 | TCF19 | | | | | |
| 6 | 31132113 | 31138470 | POU5F1 | | | POU5F1 | | |
| 6 | 31141511 | 31145676 | PSORS1C3 | | | | | |
| 6 | 31165536 | 31171745 | HCG27 | | | | | |
| 6 | 31236525 | 31239913 | HLA-C | | | | | |
| 6 | 31321642 | 31325022 | HLA-B | | | | | |
| 6 | 31323000 | 31323093 | MIR6891 | | | | | |
| 6 | 31367560 | 31383092 | MICA | | | | | |
| 6 | 31430956 | 31433586 | HCP5 | | | | | |
| 6 | 31439005 | 31440185 | HCG26 | | | | | |
| 6 | 31462657 | 31478901 | MICB | | | | | |
| 6 | 31496738 | 31498008 | MCCD1 | | | | | |
| 6 | 31497995 | 31510252 | DDX39B | | | | | |
| 6 | 31497995 | 31514625 | ATP6V1G2-DDX39B | | | | | |
| 6 | 31504150 | 31504226 | SNORD117 | | | | | |
| 6 | 31508877 | 31508955 | SNORD84 | | | | | |
| 6 | 31510080 | 31510915 | DDX39B | | | | | |
| 6 | 31512227 | 31514625 | ATP6V1G2 | | | | | |
| 6 | 31514627 | 31526606 | NFKBIL1 | | | | | |
| 6 | 31539875 | 31542100 | LTA | | | | | |
| 6 | 31543343 | 31546112 | TNF | | | TNF | | |
| 6 | 31548335 | 31550202 | LTB | | | | | |
| 6 | 31553955 | 31556686 | LST1 | | | | | |
| 6 | 31556659 | 31560762 | NCR3 | | | | | |
| 6 | 31582985 | 31584802 | AIF1 | | | | | |
| 6 | 31588449 | 31605554 | PRRC2A | | | | | |
| 6 | 31590855 | 31590987 | SNORA38 | | | | | |
| 6 | 31601563 | 31601635 | MIR6832 | | | | | |
| 6 | 31606804 | 31620477 | BAG6 | | | | | |
| 6 | 31620186 | 31625987 | APOM | | | | | |
| 6 | 31626074 | 31628549 | C6orf47 | | | | | |
| 6 | 31629005 | 31634060 | GPANK1 | | | | | |
| 6 | 31633656 | 31637847 | CSNK2B | | | | | |
| 6 | 31638727 | 31640227 | LY6G5B | | | | | |
| 6 | 31644460 | 31648150 | LY6G5C | | | | | |

| | | | | | | | | |
|---|---|---|---|---|---|---|---|---|
| 6 | 31654725 | 31671137 | ABHD16A | | | | | |
| 6 | 31668805 | 31668868 | MIR4646 | | | | | |
| 6 | 31674683 | 31678372 | LY6G6F | | | | | |
| 6 | 31679752 | 31681842 | LY6G6E | | | | | |
| 6 | 31683132 | 31685581 | LY6G6D | | | | | |
| 6 | 31686424 | 31689511 | LY6G6C | | | | | |
| 6 | 31691120 | 31694485 | C6orf25 | | | | | |
| 6 | 31694813 | 31698042 | DDAH2 | | | | | |
| 6 | 31698357 | 31705095 | CLIC1 | | | | | |
| 6 | 31707724 | 31730455 | MSH5 | | | | | |
| 6 | 31707724 | 31732624 | MSH5-SAPCD1 | | | | | |
| 6 | 31730772 | 31733365 | SAPCD1 | | | | | |
| 6 | 31733370 | 31745108 | VWA7 | | | | | |
| 6 | 31745296 | 31763712 | VARS | | | | | |
| 6 | 31765168 | 31774761 | LSM2 | | | | | |
| 6 | 31777395 | 31782835 | HSPA1L | | | | | |
| 6 | 31783290 | 31785719 | HSPA1A | | | | | |
| 6 | 31795511 | 31798031 | HSPA1B | | | | | |
| 6 | 31802691 | 31807543 | C6orf48 | | | | | |
| 6 | 31803039 | 31803103 | SNORD48 | | | | | |
| 6 | 31804852 | 31804916 | SNORD52 | | | | | |
| 6 | 31826828 | 31830709 | NEU1 | | | | | |
| 6 | 31830969 | 31846823 | SLC44A4 | | | | | |
| 6 | 31847535 | 31865484 | EHMT2 | | | | | |
| 6 | 31865561 | 31913451 | C2 | | | | | |
| 6 | 31867393 | 31869769 | ZBTB12 | | | | | |
| 6 | 31868775 | 31909501 | C2 | | | | | |
| 6 | 31913720 | 31919861 | CFB | | | | | |
| 6 | 31919863 | 31926864 | NELFE | | | | | |
| 6 | 31924615 | 31924717 | MIR1236 | | | | | |
| 6 | 31926580 | 31937532 | SKIV2L | | | | | |
| 6 | 31937587 | 31940032 | DXO | | | | | |
| 6 | 31938951 | 31949223 | STK19 | | | | | |
| 6 | 31949833 | 31970457 | C4A | | | | | |
| 6 | 31949833 | 31970458 | C4B | | | | | |
| 6 | 31949833 | 31970458 | C4B_2 | | | | | |
| 6 | 31973358 | 31976712 | CYP21A2 | | | CYP21A2 | | |
| 6 | 31973412 | 31976686 | CYP21A1P | | | | | |
| 6 | 31976196 | 31980800 | TNXA | | | | | |
| 6 | 31976196 | 31981050 | TNXB | | | TNXB | | |
| 6 | 31981517 | 31981961 | STK19 | | | | | |
| 6 | 31982571 | 32003195 | C4B | | | | | |
| 6 | 31982571 | 32003195 | C4B_2 | | | | | |
| 6 | 32006092 | 32009447 | CYP21A2 | | | CYP21A2 | | |
| 6 | 32008931 | 32077151 | TNXB | | | TNXB | | |
| 6 | 32083044 | 32096017 | ATF6B | | | | | |
| 6 | 32096483 | 32098067 | FKBPL | | | | | |
| 6 | 32116139 | 32119720 | PRRT1 | | | | | |
| 6 | 32120578 | 32122142 | LOC100507547 | | | | | |
| 6 | 32121228 | 32131458 | PPT2 | | | | | |
| 6 | 32121775 | 32136062 | PPT2-EGFL8 | | | | | |
| 6 | 32132381 | 32136062 | EGFL8 | | | | | |
| 6 | 32135982 | 32145888 | AGPAT1 | | | | | |
| 6 | 32137806 | 32137893 | MIR6721 | | | | | |
| 6 | 32146161 | 32148570 | RNF5 | | | | | |
| 6 | 32146231 | 32148567 | RNF5P1 | | | | | |
| 6 | 32147592 | 32147653 | MIR6833 | | | | | |
| 6 | 32148744 | 32152099 | AGER | | | | | |
| 6 | 32152509 | 32157963 | PBX2 | | | | | |
| 6 | 32158542 | 32163300 | GPSM3 | | | | | |
| 6 | 32162619 | 32191844 | NOTCH4 | | | | | |
| 6 | 32260474 | 32339689 | C6orf10 | | | | | |
| 6 | 32358286 | 32361468 | HCG23 | | | | | |
| 6 | 32361115 | 32374907 | BTNL2 | | | | | |
| 6 | 32407618 | 32412826 | HLA-DRA | | | | | |
| 6 | 32485153 | 32498006 | HLA-DRB5 | | | | | |

| | | | | | | | | |
|---|---|---|---|---|---|---|---|---|
| 6 | 32520489 | 32527779 | HLA-DRB6 | | | | | |
| 6 | 32546546 | 32557613 | HLA-DRB1 | | | | | |
| 6 | 32605182 | 32611429 | HLA-DQA1 | | | | | |
| 6 | 32627240 | 32634466 | HLA-DQB1 | | | | | |
| 6 | 32627656 | 32628506 | HLA-DQB1-AS1 | | | | | |
| 6 | 32709162 | 32714664 | HLA-DQA2 | | | | | |
| 6 | 32717688 | 32717756 | MIR3135B | | | | | |
| 6 | 32723874 | 32731330 | HLA-DQB2 | | | | | |
| 6 | 32780539 | 32784825 | HLA-DOB | | | | | |
| 6 | 32789609 | 32806600 | TAP2 | | | | | |
| 6 | 32808493 | 32814277 | PSMB8 | | | | | |
| 6 | 32812985 | 32821748 | TAP1 | | | | | |
| 6 | 32821937 | 32827628 | PSMB9 | | | | | |
| 6 | 32861952 | 32871535 | LOC100294145 | | | | | |
| 6 | 32902405 | 32908847 | HLA-DMB | | | | | |
| 6 | 32916390 | 32920899 | HLA-DMA | | | | | |
| 6 | 32936436 | 32949282 | BRD2 | | | BRD2 | | |
| 6 | 32971959 | 32977389 | HLA-DOA | | | | | |
| 6 | 33032345 | 33048555 | HLA-DPA1 | | | | | |
| 6 | 33043702 | 33057473 | HLA-DPB1 | | | | | |
| 6 | 33080292 | 33096890 | HLA-DPB2 | | | | | |
| 6 | 33130468 | 33160245 | COL11A2 | | | COL11A2 | | |
| 6 | 33161361 | 33168630 | RXRB | | | | | |
| 6 | 33168602 | 33172214 | SLC39A7 | | | | | |
| 6 | 33172413 | 33174608 | HSD17B8 | | | | | |
| 6 | 33175611 | 33175721 | MIR219A1 | | | | | |
| 6 | 33176285 | 33180499 | RING1 | | | | | |
| 6 | 33217312 | 33222667 | HCG25 | | | | | |
| 6 | 33218048 | 33239742 | VPS52 | | | | | |
| 6 | 33239851 | 33244281 | RPS18 | | | | | |
| 6 | 33244916 | 33246602 | B3GALT4 | | | | | |
| 6 | 33246879 | 33257304 | WDR46 | | | | | |
| 6 | 33255003 | 33255066 | MIR6873 | | | | | |
| 6 | 33257373 | 33258711 | PFDN6 | | | | | |
| 6 | 33258021 | 33258102 | MIR6834 | | | | | |
| 6 | 33259430 | 33267165 | RGL2 | | | | | |
| 6 | 33267471 | 33282164 | TAPBP | | | TAPBP | | |
| 6 | 33282181 | 33285719 | ZBTB22 | | | | | |
| 6 | 33285441 | 33285461 | MIR1234 | | | | | |
| 6 | 33286334 | 33290793 | DAXX | | | | | |
| 6 | 33359312 | 33377699 | KIFC1 | | | | | |
| 6 | 33378772 | 33384230 | PHF1 | | | | | |
| 6 | 33384318 | 33386065 | CUTA | | | | | |
| 6 | 33387846 | 33421466 | SYNGAP1 | SYNGAP1 | | SYNGAP1 | | |
| 6 | 33406107 | 33406214 | MIR5004 | | | | | |
| 6 | 33422355 | 33425320 | ZBTB9 | | | | | |
| 6 | 33540322 | 33548070 | BAK1 | | | | | |
| 6 | 33551475 | 33556803 | GGNBP1 | | | | | |
| 6 | 33553882 | 33561115 | LINC00336 | | | | | |
| 6 | 33589155 | 33664348 | ITPR3 | | | | ITPR3 | |
| 6 | 33599020 | 33601522 | LOC101929188 | | | | | |
| 6 | 33664537 | 33679528 | UQCC2 | | | | | |
| 6 | 33665904 | 33666011 | MIR3934 | | | | | |
| 6 | 33689442 | 33714762 | IP6K3 | | | | | |
| 6 | 33738989 | 33756906 | LEMD2 | | | LEMD2 | | |
| 6 | 33762448 | 33771793 | MLN | | | | | |
| 6 | 33857287 | 33864684 | LINC01016 | | | | | |
| 6 | 33866911 | 33866977 | MIR7159 | | | | | |
| 6 | 33967748 | 33967828 | MIR1275 | | | | | |
| 6 | 33986419 | 34123399 | GRM4 | GRM4 | | GRM4 | GRM4 | |
| 6 | 34204576 | 34214008 | HMGA1 | | | | | |
| 6 | 34208449 | 34208513 | MIR6835 | | | | | |
| 6 | 34214156 | 34216885 | C6orf1 | | | | | |
| 6 | 34254972 | 34360457 | NUDT3 | | | | | |
| 6 | 34254972 | 34393902 | RPS10-NUDT3 | | | | | |
| 6 | 34385230 | 34393902 | RPS10 | | | | | |

| | | | | | | | | |
|---|---|---|---|---|---|---|---|---|
| 6 | 34433837 | 34503000 | PACSIN1 | | | PACSIN1 | | |
| 6 | 34505578 | 34524110 | SPDEF | | | | | |
| 6 | 34555056 | 34664627 | C6orf106 | | | | | |
| 6 | 34663930 | 34665248 | LOC101929243 | | | | | |
| 6 | 34724870 | 34741634 | SNRPC | | | | | |
| 6 | 34759793 | 34845291 | UHRF1BP1 | | | | | |
| 6 | 34845554 | 34855848 | TAF11 | | | | | |
| 6 | 34857037 | 35059190 | ANKS1A | | | ANKS1A | | |
| 6 | 35085848 | 35109187 | TCP11 | | | | | |
| 6 | 35181838 | 35220856 | SCUBE3 | | | | | |
| 6 | 35227490 | 35263764 | ZNF76 | | | | | |
| 6 | 35265594 | 35289548 | DEF6 | | | | | |
| 6 | 35310334 | 35395968 | PPARD | | | | | |
| 6 | 35420137 | 35434881 | FANCE | | | FANCE | | |
| 6 | 35436177 | 35438558 | RPL10A | | | | | |
| 6 | 35438284 | 35438356 | MIR7111 | | | | | |
| 6 | 35441373 | 35464861 | TEAD3 | | | | | |
| 6 | 35465650 | 35480679 | TULP1 | | | | | |
| 6 | 35541361 | 35656719 | FKBP5 | | | | FKBP5 | |
| 6 | 35632493 | 35632566 | MIR5690 | | | | | |
| 6 | 35694538 | 35704724 | LOC285847 | | | | | |
| 6 | 35704808 | 35716690 | ARMC12 | | | | | |
| 6 | 35744370 | 35747329 | CLPSL2 | | | | | |
| 6 | 35748830 | 35755841 | CLPSL1 | | | | | |
| 6 | 35762758 | 35765121 | CLPS | | | | | |
| 6 | 35773070 | 35791852 | LHFPL5 | | | | | |
| 6 | 35800810 | 35888957 | SRPK1 | | | | | |
| 6 | 35911290 | 35992413 | SLC26A8 | | SLC26A8 | | | |
| 6 | 35995453 | 36079013 | MAPK14 | | | MAPK14 | | |
| 6 | 36098260 | 36112301 | MAPK13 | | | | | |
| 6 | 36164549 | 36200567 | BRPF3 | | | | | |
| 6 | 36210944 | 36276372 | PNPLA1 | | | | | |
| 6 | 36283534 | 36304662 | C6orf222 | | | | | |
| 6 | 36321997 | 36355577 | ETV7 | | | | | |
| 6 | 36358328 | 36410666 | PXT1 | | | | | |
| 6 | 36410543 | 36458920 | KCTD20 | | | | | |
| 6 | 36461659 | 36515293 | STK38 | | | STK38 | | |
| 6 | 36562089 | 36572244 | SRSF3 | | | | | |
| 6 | 36590212 | 36590289 | MIR3925 | | | | | |
| 6 | 36641397 | 36642903 | PANDAR | | | | | |
| 6 | 36644236 | 36655116 | CDKN1A | | | | | |
| 6 | 36665627 | 36700960 | RAB44 | | | | | |
| 6 | 36708554 | 36807220 | CPNE5 | | | | | |
| 6 | 36822605 | 36842800 | PPIL1 | | | | | |
| 6 | 36839645 | 36896740 | C6orf89 | | | | | |
| 6 | 36916038 | 36932613 | PI16 | | | | | |
| 6 | 36935910 | 36954327 | MTCH1 | | | | | |
| 6 | 36973422 | 36996845 | FGD2 | | | | | |
| 6 | 37137921 | 37143204 | PIM1 | | | | | |
| 6 | 37179953 | 37225931 | TMEM217 | | | | | |
| 6 | 37225479 | 37300746 | TBC1D22B | | | | | |
| 6 | 37321747 | 37362514 | RNF8 | | | | | |
| 6 | 37400906 | 37449284 | CMTR1 | | | | | |
| 6 | 37450696 | 37467700 | CCDC167 | | | | | |
| 6 | 37475123 | 37504056 | LOC100505530 | | | | | |
| 6 | 37523140 | 37523198 | MIR4462 | | | | | |
| 6 | 37600283 | 37665766 | MDGA1 | | | | | |
| 6 | 37787306 | 38122399 | ZFAND3 | | | | | |
| 6 | 38136226 | 38607924 | BTBD9 | | | | | |
| 6 | 38643701 | 38670952 | GLO1 | | | | | |
| 6 | 38683116 | 38998574 | DNAH8 | | | | | |
| 6 | 38874270 | 38920883 | LOC100131047 | | | | | |
| 6 | 39016556 | 39055520 | GLP1R | | | | | |
| 6 | 39071838 | 39082962 | SAYSD1 | | | | | |
| 6 | 39156746 | 39197251 | KCNK5 | | | | | |
| 6 | 39266776 | 39282237 | KCNK17 | | | | | |

| | | | | | | | | |
|---|---|---|---|---|---|---|---|---|
| 6 | 39282473 | 39290330 | KCNK16 | | | | | |
| 6 | 39302875 | 39693181 | KIF6 | | | | | |
| 6 | 39760158 | 39872653 | DAAM2 | | | DAAM2 | | |
| 6 | 39856565 | 39865156 | LOC100505635 | | | | | |
| 6 | 39872033 | 39902290 | MOCS1 | | | | | |
| 6 | 40312083 | 40323745 | LINC00951 | | | | | |
| 6 | 40346162 | 40347631 | TDRG1 | | | | | |
| 6 | 40359372 | 40555203 | LRFN2 | | | | | |
| 6 | 40846624 | 40991019 | LOC101929555 | | | | | |
| 6 | 40994639 | 41006938 | UNC5CL | | | | | |
| 6 | 41010236 | 41012076 | TSPO2 | | | | | |
| 6 | 41020939 | 41032630 | APOBEC2 | | | | | |
| 6 | 41034530 | 41040188 | OARD1 | | | | | |
| 6 | 41040706 | 41070146 | NFYA | | | | | |
| 6 | 41068772 | 41108573 | ADCY10P1 | | | | | |
| 6 | 41116998 | 41122087 | TREML1 | | | | | |
| 6 | 41126243 | 41130924 | TREM2 | | TREM2 | | | |
| 6 | 41157486 | 41168925 | TREML2 | | | | | |
| 6 | 41176291 | 41185685 | TREML3P | | | | | |
| 6 | 41196061 | 41206120 | TREML4 | | | | | |
| 6 | 41217114 | 41217327 | TREML5P | | | | | |
| 6 | 41242998 | 41254457 | TREM1 | | | | | |
| 6 | 41303527 | 41318625 | NCR2 | | | | | |
| 6 | 41470181 | 41487590 | LINC01276 | | | | | |
| 6 | 41491632 | 41570122 | FOXP4 | | | | | |
| 6 | 41566460 | 41566526 | MIR4641 | | | | | |
| 6 | 41604914 | 41621982 | MDFI | | | | | |
| 6 | 41651715 | 41703997 | TFEB | | | | | |
| 6 | 41704448 | 41715139 | PGC | | | | | |
| 6 | 41737913 | 41747643 | FRS3 | | | FRS3 | | |
| 6 | 41748499 | 41755110 | PRICKLE4 | | | | | |
| 6 | 41755180 | 41757634 | TOMM6 | | | | | |
| 6 | 41757633 | 41863099 | USP49 | | | | | |
| 6 | 41873090 | 41888885 | MED20 | | | | | |
| 6 | 41888964 | 41900784 | BYSL | | | | | |
| 6 | 41902670 | 42016632 | CCND3 | | | | | |
| 6 | 42018250 | 42048644 | TAF8 | | | | | |
| 6 | 42068856 | 42110715 | C6orf132 | | | | | |
| 6 | 42123114 | 42147821 | GUCA1A | | | | | |
| 6 | 42151021 | 42162694 | GUCA1B | | | | | |
| 6 | 42174538 | 42185633 | MRPS10 | | | MRPS10 | | |
| 6 | 42192670 | 42419789 | TRERF1 | | | | | |
| 6 | 42531759 | 42661243 | UBR2 | | | | | |
| 6 | 42664332 | 42690358 | PRPH2 | | | | | |
| 6 | 42695313 | 42695932 | ATP6V0CP3 | | | | | |
| 6 | 42712233 | 42713884 | TBCC | | | | | |
| 6 | 42714695 | 42836298 | GLTSCR1L | | | | | |
| 6 | 42751233 | 42759061 | LOC401261 | | | | | |
| 6 | 42788793 | 42836298 | GLTSCR1L | | | | | |
| 6 | 42847353 | 42857634 | RPL7L1 | | | | | |
| 6 | 42858002 | 42858554 | C6orf226 | | | | | |
| 6 | 42883726 | 42893575 | PTCRA | | | | | |
| 6 | 42895739 | 42907025 | CNPY3 | | | | | |
| 6 | 42896867 | 42931618 | CNPY3-GNMT | | | | | |
| 6 | 42928491 | 42931618 | GNMT | | | | | |
| 6 | 42931610 | 42946981 | PEX6 | | | | | |
| 6 | 42952236 | 42980083 | PPP2R5D | | | | | |
| 6 | 42979750 | 42984624 | MEA1 | | | | | |
| 6 | 42981840 | 42989036 | KLHDC3 | | | | | |
| 6 | 42989384 | 42997337 | RRP36 | | | | | |
| 6 | 43005354 | 43021683 | CUL7 | | | CUL7 | | |
| 6 | 43021766 | 43027283 | MRPL2 | | | | | |
| 6 | 43027331 | 43042837 | KLC4 | | | | | |
| 6 | 43044005 | 43129458 | PTK7 | | | | | |
| 6 | 43139032 | 43149244 | SRF | | | | | |
| 6 | 43149921 | 43192325 | CUL9 | | | | | |

| | | | | | | | | |
|---|---|---|---|---|---|---|---|---|
| 6 | 43193366 | 43197211 | DNPH1 | | | | | |
| 6 | 43211221 | 43255997 | TTBK1 | | | | | |
| 6 | 43265997 | 43273276 | SLC22A7 | | | | | |
| 6 | 43273210 | 43276530 | CRIP3 | | | | | |
| 6 | 43303807 | 43337181 | ZNF318 | | | | | |
| 6 | 43395291 | 43418163 | ABCC10 | | | | | |
| 6 | 43402279 | 43402358 | MIR6780B | | | | | |
| 6 | 43418089 | 43424370 | DLK2 | | | | | |
| 6 | 43445260 | 43474294 | TJAP1 | | | | | |
| 6 | 43474702 | 43478081 | LRRC73 | | | | | |
| 6 | 43479564 | 43484728 | YIPF3 | | | | | |
| 6 | 43484761 | 43497114 | POLR1C | | | | | |
| 6 | 43490067 | 43543812 | XPO5 | | | | | |
| 6 | 43543877 | 43588260 | POLH | | | | | |
| 6 | 43588217 | 43596983 | GTPBP2 | | | | | |
| 6 | 43597278 | 43608688 | MAD2L1BP | | | | | |
| 6 | 43612766 | 43638748 | RSPH9 | | | | | |
| 6 | 43638933 | 43655549 | MRPS18A | | | | | |
| 6 | 43737945 | 43754223 | VEGFA | | | VEGFA | | |
| 6 | 43858764 | 43905944 | LINC01512 | | | | | |
| 6 | 43963460 | 44042389 | LOC101929705 | | | | | |
| 6 | 43968336 | 43973694 | C6orf223 | | | | | |
| 6 | 44081190 | 44095228 | MRPL14 | | | | | |
| 6 | 44094650 | 44123256 | TMEM63B | | | | | |
| 6 | 44126547 | 44152139 | CAPN11 | | | | | |
| 6 | 44184675 | 44185973 | LOC101929726 | | | | | |
| 6 | 44187241 | 44201888 | SLC29A1 | | | | | |
| 6 | 44213902 | 44221625 | HSP90AB1 | | | | | |
| 6 | 44221837 | 44225627 | SLC35B2 | | | SLC35B2 | | |
| 6 | 44221942 | 44222022 | MIR4647 | | | | | |
| 6 | 44225902 | 44233525 | NFKBIE | | | | | |
| 6 | 44238479 | 44247182 | TMEM151B | | | | | |
| 6 | 44247896 | 44265458 | TCTE1 | | | | | |
| 6 | 44266462 | 44281063 | AARS2 | | | | | |
| 6 | 44310396 | 44344904 | SPATS1 | | | | | |
| 6 | 44355250 | 44418161 | CDC5L | | | | | |
| 6 | 44403377 | 44403459 | MIR4642 | | | | | |
| 6 | 44513897 | 44519618 | LOC105375075 | | | | | |
| 6 | 44794466 | 45345788 | SUPT3H | | | | | |
| 6 | 45165410 | 45165507 | MIR586 | | | | | |
| 6 | 45296053 | 45518819 | RUNX2 | | | RUNX2 | | |
| 6 | 45866189 | 45983626 | CLIC5 | | | CLIC5 | | |
| 6 | 46097700 | 46114436 | ENPP4 | | | | | |
| 6 | 46126918 | 46138747 | ENPP5 | | ENPP5 | | | |
| 6 | 46188466 | 46459804 | RCAN2 | | RCAN2 | | | |
| 6 | 46244060 | 46333242 | LOC101926915 | | | | | |
| 6 | 46459788 | 46501217 | LOC101926898 | | | | | |
| 6 | 46517316 | 46620567 | CYP39A1 | | | | | |
| 6 | 46620651 | 46645927 | SLC25A27 | | | SLC25A27 | | |
| 6 | 46638180 | 46655902 | LOC101926934 | | | | | |
| 6 | 46655611 | 46672056 | TDRD6 | | | | | |
| 6 | 46672052 | 46703430 | PLA2G7 | | | | | |
| 6 | 46714653 | 46726954 | ANKRD66 | | | | | |
| 6 | 46761093 | 46807519 | MEP1A | | | | | |
| 6 | 46820241 | 46922675 | ADGRF5 | | | | | |
| 6 | 46871207 | 46877764 | LOC101926962 | | | | | |
| 6 | 46965439 | 47010099 | ADGRF1 | | | | | |
| 6 | 47199262 | 47277683 | TNFRSF21 | | | | | |
| 6 | 47445524 | 47594996 | CD2AP | | | | | |
| 6 | 47624325 | 47665533 | ADGRF2 | | | | | |
| 6 | 47666288 | 47689757 | ADGRF4 | | | | | |
| 6 | 47749774 | 47794116 | OPN5 | | | | | |
| 6 | 47845763 | 48036425 | PTCHD4 | | | | | |
| 6 | 49398072 | 49431041 | MUT | | | MUT | | |
| 6 | 49431095 | 49460820 | CENPQ | | | | | |
| 6 | 49467670 | 49495777 | GLYATL3 | | | | | |

| | | | | | | | | |
|---|---|---|---|---|---|---|---|---|
| 6 | 49518112 | 49519808 | C6orf141 | | | | | |
| 6 | 49572889 | 49604587 | RHAG | | | | | |
| 6 | 49660070 | 49681303 | CRISP2 | | | | | |
| 6 | 49695091 | 49712168 | CRISP3 | | | | | |
| 6 | 49753363 | 49755053 | PGK2 | | | PGK2 | | |
| 6 | 49779252 | 49788216 | LOC101927020 | | | | | |
| 6 | 49783205 | 49792479 | LOC101927048 | | | | | |
| 6 | 49801978 | 49844809 | CRISP1 | | | | | |
| 6 | 49913813 | 49917157 | DEFB133 | | | | | |
| 6 | 49928004 | 49931818 | DEFB114 | | | | | |
| 6 | 49936389 | 49937338 | DEFB113 | | | | | |
| 6 | 49976850 | 49989694 | DEFB110 | | | | | |
| 6 | 50011287 | 50016364 | DEFB112 | | | | | |
| 6 | 50681256 | 50740746 | TFAP2D | | | | | |
| 6 | 50786438 | 50815326 | TFAP2B | | | TFAP2B | | |
| 6 | 51480144 | 51952423 | PKHD1 | | | | | |
| 6 | 51486667 | 51487375 | LOC101927082 | | | | | |
| 6 | 51585646 | 51952423 | PKHD1 | | | | | |
| 6 | 52009146 | 52009232 | MIR206 | | | | | |
| 6 | 52011611 | 52015821 | LINCMD1 | | | | | |
| 6 | 52013720 | 52013839 | MIR133B | | | | | |
| 6 | 52051184 | 52055436 | IL17A | | | | | |
| 6 | 52101483 | 52109298 | IL17F | | | | | |
| 6 | 52128811 | 52149679 | MCM3 | | | | | |
| 6 | 52226925 | 52272575 | PAQR8 | | | | | |
| 6 | 52284993 | 52360583 | EFHC1 | | | | | |
| 6 | 52362199 | 52448791 | TRAM2 | | | | | |
| 6 | 52529198 | 52533951 | LOC730101 | | | | | |
| 6 | 52535883 | 52551385 | TMEM14A | | | | | |
| 6 | 52604260 | 52609957 | GSTA7P | | | | | |
| 6 | 52614884 | 52628361 | GSTA2 | | | | | |
| 6 | 52656177 | 52668765 | GSTA1 | | | | | |
| 6 | 52696540 | 52710893 | GSTA5 | | | | | |
| 6 | 52761438 | 52774496 | GSTA3 | | | | | |
| 6 | 52842745 | 52860178 | GSTA4 | | | | | |
| 6 | 52866097 | 52926600 | ICK | | | ICK | | |
| 6 | 52929795 | 52965670 | FBXO9 | | | | | |
| 6 | 52991759 | 53013624 | GCM1 | | | | | |
| 6 | 53132195 | 53213977 | ELOVL5 | | | | | |
| 6 | 53141790 | 53141869 | MIR5685 | | | | | |
| 6 | 53158887 | 53213977 | ELOVL5 | | | | | |
| 6 | 53199257 | 53202415 | RPS16P5 | | | | | |
| 6 | 53362139 | 53409927 | GCLC | | | | | |
| 6 | 53426086 | 53438073 | LOC101927136 | | | | | |
| 6 | 53493177 | 53496192 | LINC01564 | | | | | |
| 6 | 53512698 | 53530506 | KLHL31 | | | | | |
| 6 | 53659777 | 53788919 | LRRC1 | | | | | |
| 6 | 53794779 | 53862465 | LOC101927189 | | | | | |
| 6 | 53863687 | 53871950 | MLIP-IT1 | | | | | |
| 6 | 53883713 | 54131078 | MLIP | | | | | |
| 6 | 54173202 | 54254950 | TINAG | | | | | |
| 6 | 54711568 | 54809897 | FAM83B | | | | | |
| 6 | 55039070 | 55147418 | HCRTR2 | | HCRTR2 | HCRTR2 | | |
| 6 | 55192266 | 55267291 | GFRAL | | | | | |
| 6 | 55299170 | 55444012 | HMGCLL1 | | | | | |
| 6 | 55620237 | 55740375 | BMP5 | | | | | |
| 6 | 55921387 | 56258926 | COL21A1 | | | | | |
| 6 | 56322784 | 56507694 | DST | | DST | | | |
| 6 | 56708783 | 56729136 | LOC101930010 | | | | | |
| 6 | 56819772 | 56892142 | BEND6 | | | | | |
| 6 | 56911329 | 56920037 | KIAA1586 | | | | | |
| 6 | 56954807 | 57035098 | ZNF451 | | | | | |
| 6 | 56979708 | 57037011 | LOC101927211 | | | | | |
| 6 | 57037103 | 57050012 | BAG2 | | | | | |
| 6 | 57051790 | 57087112 | RAB23 | | | RAB23 | | |
| 6 | 57125321 | 57128144 | LOC100506188 | | | | | |

| | | | | | | | | |
|---|---|---|---|---|---|---|---|---|
| 6 | 57179602 | 57513376 | PRIM2 | | | | | |
| 6 | 57254929 | 57255010 | MIR548U | | | | | |
| 6 | 58234830 | 58263207 | GUSBP4 | | | | | |
| 6 | 58246158 | 58287724 | LINC00680-GUSBP4 | | | | | |
| 6 | 58272111 | 58287724 | LINC00680 | | | | | |
| 6 | 62284007 | 62284534 | MTRNR2L9 | | | | | |
| 6 | 62389864 | 62996132 | KHDRBS2 | | | | | |
| 6 | 63985855 | 64029882 | LGSN | | | | | |
| 6 | 64281916 | 64293493 | PTP4A1 | | | | | |
| 6 | 64345706 | 64425418 | PHF3 | | | | | |
| 6 | 64429875 | 66417118 | EYS | | | | | |
| 6 | 66011310 | 66015504 | LOC441155 | | | | | |
| 6 | 66039168 | 66289697 | EYS | | | | | |
| 6 | 66497771 | 66499376 | SLC25A51P1 | | | | | |
| 6 | 68765242 | 68770394 | LOC102723883 | | | | | |
| 6 | 68936861 | 69039791 | LOC101928280 | | | | | |
| 6 | 69342554 | 69344216 | LOC101928307 | | | | | |
| 6 | 69345631 | 70099403 | ADGRB3 | | | | | |
| 6 | 70385640 | 70507049 | LMBRD1 | | | | | |
| 6 | 70576447 | 70922157 | COL19A1 | | | | | |
| 6 | 70925742 | 71012786 | COL9A1 | | COL9A1 | COL9A1 | | |
| 6 | 71103904 | 71109120 | EVADR | | | | | |
| 6 | 71123106 | 71270877 | FAM135A | | | | | |
| 6 | 71276624 | 71298606 | SDHAF4 | | | | | |
| 6 | 71377473 | 71571716 | SMAP1 | | | | | |
| 6 | 71571068 | 71666788 | B3GAT2 | | | | | |
| 6 | 71998476 | 72011973 | OGFRL1 | | | | | |
| 6 | 72086662 | 72086734 | MIR30C2 | | | | | |
| 6 | 72113253 | 72113324 | MIR30A | | | | | |
| 6 | 72117566 | 72130448 | LINC00472 | | | | | |
| 6 | 72130729 | 72168575 | LINC01626 | | | | | |
| 6 | 72596405 | 73112845 | RIMS1 | | RIMS1 | RIMS1 | RIMS1 | RIMS1 |
| 6 | 73331570 | 73908573 | KCNQ5 | | | KCNQ5 | KCNQ5 | |
| 6 | 73340222 | 73388282 | KCNQ5-IT1 | | | | | |
| 6 | 73677409 | 73677476 | MIR4282 | | | | | |
| 6 | 73844525 | 73853237 | KCNQ5 | | | KCNQ5 | KCNQ5 | |
| 6 | 73933267 | 73935175 | KHDC1L | | | | | |
| 6 | 73951036 | 74020088 | KHDC1 | | | | | |
| 6 | 74062784 | 74063999 | DPPA5 | | | | | |
| 6 | 74072399 | 74073898 | KHDC3L | | | | | |
| 6 | 74078279 | 74079515 | OOEP | | | | | |
| 6 | 74104284 | 74127289 | DDX43 | | | | | |
| 6 | 74134855 | 74162043 | MB21D1 | | | | | |
| 6 | 74171453 | 74211179 | MTO1 | | | | | |
| 6 | 74225472 | 74230755 | EEF1A1 | | | | | |
| 6 | 74227967 | 74228161 | SNORD141A | | | | | |
| 6 | 74227967 | 74228161 | SNORD141B | | | | | |
| 6 | 74303101 | 74363737 | SLC17A5 | | | SLC17A5 | | |
| 6 | 74403625 | 74405854 | LOC101928489 | | | | | |
| 6 | 74405807 | 74538041 | CD109 | | | | | |
| 6 | 74779166 | 75400443 | LOC101928516 | | | | | |
| 6 | 75794041 | 75915623 | COL12A1 | | | COL12A1 | | |
| 6 | 75947390 | 75953644 | COX7A2 | | | | | |
| 6 | 75962637 | 75994632 | TMEM30A | | | | | |
| 6 | 75994729 | 76001580 | LOC100506804 | | | | | |
| 6 | 76005582 | 76203545 | FILIP1 | | | | | |
| 6 | 76092901 | 76168615 | LOC101928540 | | | | | |
| 6 | 76138122 | 76138189 | MIR4463 | | | | | |
| 6 | 76166105 | 76203545 | FILIP1 | | | | | |
| 6 | 76311224 | 76428001 | SENP6 | | | | | |
| 6 | 76458892 | 76629254 | MYO6 | | MYO6 | | | |
| 6 | 76630830 | 76782395 | IMPG1 | | | | | |
| 6 | 78170564 | 78173739 | HTR1B | | | HTR1B | | |
| 6 | 78400372 | 78634476 | MEI4 | | | | | |
| 6 | 79577260 | 79610683 | IRAK1BP1 | | | | | |
| 6 | 79644135 | 79788011 | PHIP | | | | | |

| | | | | | | | | |
|---|---|---|---|---|---|---|---|---|
| 6 | 79910961 | 79946517 | HMGN3 | | | | | |
| 6 | 80017385 | 80023101 | LCAL1 | | | | | |
| 6 | 80194707 | 80247147 | LCA5 | | | | | |
| 6 | 80340821 | 80413387 | SH3BGRL2 | | | | | |
| 6 | 80451635 | 80451669 | RNY4 | | | | | |
| 6 | 80513299 | 80521963 | LINC01621 | | | | | |
| 6 | 80624528 | 80657315 | ELOVL4 | | ELOVL4 | | | |
| 6 | 80714321 | 80752244 | TTK | | | TTK | | |
| 6 | 80816326 | 81055987 | BCKDHB | | | BCKDHB | | |
| 6 | 82455446 | 82462428 | FAM46A | | | | | |
| 6 | 82523002 | 82523909 | LINC01526 | | | | | |
| 6 | 82879703 | 82957471 | IBTK | | | | | |
| 6 | 83072922 | 83077133 | TPBG | | | | | |
| 6 | 83602116 | 83775560 | UBE3D | | | | | |
| 6 | 83777384 | 83878190 | DOPEY1 | | | | | |
| 6 | 83874592 | 83903012 | PGM3 | | | | | |
| 6 | 83903031 | 83906256 | RWDD2A | | | | | |
| 6 | 83920109 | 84140938 | ME1 | | | | | |
| 6 | 84222193 | 84235421 | PRSS35 | | | | | |
| 6 | 84262604 | 84419127 | SNAP91 | | | SNAP91 | | |
| 6 | 84562984 | 84567234 | RIPPLY2 | | | | | |
| 6 | 84569369 | 84670146 | CYB5R4 | | | | | |
| 6 | 84743419 | 84800605 | MRAP2 | | | | | |
| 6 | 84833959 | 84937353 | CEP162 | | | | | |
| 6 | 85130750 | 85184647 | LINC01611 | | | | | |
| 6 | 85399175 | 85473954 | TBX18 | | | | | |
| 6 | 86096936 | 86114520 | LOC101928820 | | | | | |
| 6 | 86159301 | 86205509 | NT5E | | | | | |
| 6 | 86215214 | 86303874 | SNX14 | | | SNX14 | | |
| 6 | 86317501 | 86353043 | SYNCRIP | | | | | |
| 6 | 86386724 | 86388451 | SNHG5 | | | | | |
| 6 | 86387011 | 86387086 | SNORD50A | | | | | |
| 6 | 86387306 | 86387377 | SNORD50B | | | | | |
| 6 | 87647023 | 87726397 | HTR1E | | | HTR1E | | |
| 6 | 87795215 | 87804865 | CGA | | | | | |
| 6 | 87865268 | 87973406 | ZNF292 | | | | | |
| 6 | 87992696 | 88038996 | GJB7 | | | | | |
| 6 | 88032305 | 88052046 | SMIM8 | | | | | |
| 6 | 88054570 | 88075181 | C6orf163 | | | | | |
| 6 | 88106841 | 88109459 | LINC01590 | | | | | |
| 6 | 88117689 | 88174191 | CFAP206 | | | | | |
| 6 | 88182642 | 88222057 | SLC35A1 | | | | | |
| 6 | 88223652 | 88299750 | RARS2 | | | | | |
| 6 | 88299784 | 88377172 | ORC3 | | | | | |
| 6 | 88384577 | 88411985 | AKIRIN2 | | | | | |
| 6 | 88494577 | 88621449 | LOC101928911 | | | | | |
| 6 | 88757506 | 88776550 | SPACA1 | | | | | |
| 6 | 88849584 | 88875767 | CNR1 | | | CNR1 | | |
| 6 | 89235642 | 89249088 | LOC101928936 | | | | | |
| 6 | 89319615 | 89673348 | RNGTT | | | | | |
| 6 | 89790428 | 89794879 | PNRC1 | | | | | |
| 6 | 89805677 | 89827800 | SRSF12 | | | | | |
| 6 | 89855768 | 89875288 | PM20D2 | | | | | |
| 6 | 89887222 | 89941007 | GABRR1 | | | GABRR1 | | |
| 6 | 89966839 | 90025018 | GABRR2 | | | | GABRR2 | GABRR2 |
| 6 | 90036343 | 90062619 | UBE2J1 | | | | | |
| 6 | 90074334 | 90121995 | RRAGD | | | | | |
| 6 | 90142896 | 90343553 | ANKRD6 | | | | | |
| 6 | 90341942 | 90348474 | LYRM2 | | | | | |
| 6 | 90348250 | 90399200 | LOC101929057 | | | | | |
| 6 | 90352493 | 90529513 | MDN1 | | | | | |
| 6 | 90539618 | 90584155 | CASP8AP2 | | | | | |
| 6 | 90604187 | 90605819 | GJA10 | | | | | |
| 6 | 90636246 | 91006627 | BACH2 | | | | | |
| 6 | 91022460 | 91022552 | MIR4464 | | | | | |
| 6 | 91223291 | 91297020 | MAP3K7 | | | | | |

| | | | | | | | | |
|---|---|---|---|---|---|---|---|---|
| 6 | 92231377 | 92231455 | MIR4643 | | | | | |
| 6 | 92338757 | 92400146 | CASC6 | | | | | |
| 6 | 93949739 | 94129300 | EPHA7 | | | EPHA7 | | |
| 6 | 94416800 | 94486199 | TSG1 | | | | | |
| 6 | 96007971 | 96057328 | MANEA | | | | | |
| 6 | 96463844 | 96663488 | FUT9 | | | | | |
| 6 | 96969701 | 97003151 | UFL1 | | | | | |
| 6 | 97010423 | 97064512 | FHL5 | | | | | |
| 6 | 97241997 | 97285353 | GPR63 | | | | | |
| 6 | 97337186 | 97345767 | NDUFAF4 | | | | | |
| 6 | 97372495 | 97588630 | KLHL32 | | | | | |
| 6 | 97537842 | 97862283 | MIR548H3 | | | | | |
| 6 | 97590036 | 97731052 | MMS22L | | | | | |
| 6 | 97753461 | 98156793 | LOC101927314 | | | | | |
| 6 | 98472406 | 98472495 | MIR2113 | | | | | |
| 6 | 99282579 | 99286666 | POU3F2 | | | POU3F2 | | |
| 6 | 99321600 | 99395882 | FBXL4 | | | | | |
| 6 | 99720792 | 99797531 | FAXC | | | | | |
| 6 | 99817347 | 99842082 | COQ3 | | | COQ3 | | |
| 6 | 99847840 | 99873207 | PNISR | | | | | |
| 6 | 99872746 | 99879190 | LOC101927365 | | | | | |
| 6 | 99880183 | 99963252 | USP45 | | | | | |
| 6 | 99968869 | 99981059 | TSTD3 | | | | | |
| 6 | 99990262 | 100016690 | CCNC | | | CCNC | | |
| 6 | 100054649 | 100063454 | PRDM13 | | | | | |
| 6 | 100367785 | 100524295 | MCHR2 | | | | | |
| 6 | 100836749 | 100911551 | SIM1 | | | SIM1 | | |
| 6 | 100956070 | 101329248 | ASCC3 | | | | | |
| 6 | 101846860 | 102517958 | GRIK2 | | GRIK2 | GRIK2 | GRIK2 | GRIK2 |
| 6 | 105175967 | 105307794 | HACE1 | | HACE1 | | | |
| 6 | 105384168 | 105388402 | LINC00577 | | | | | |
| 6 | 105404922 | 105531207 | LIN28B | | | | | |
| 6 | 105544698 | 105617819 | BVES | | | | | |
| 6 | 105605774 | 105627858 | POPDC3 | | | | | |
| 6 | 105725441 | 105850999 | PREP | | | | | |
| 6 | 106534194 | 106557814 | PRDM1 | | | | | |
| 6 | 106632351 | 106773695 | ATG5 | | | | | |
| 6 | 106899370 | 106905123 | LOC105377924 | | | | | |
| 6 | 106959729 | 107018324 | AIM1 | | | | | |
| 6 | 107018645 | 107078366 | RTN4IP1 | | | RTN4IP1 | | |
| 6 | 107077440 | 107116292 | QRSL1 | | | | | |
| 6 | 107165326 | 107235300 | LOC100422737 | | | | | |
| 6 | 107231999 | 107232095 | MIR587 | | | | | |
| 6 | 107349375 | 107372790 | C6orf203 | | | | | |
| 6 | 107386384 | 107435636 | BEND3 | | | | | |
| 6 | 107473760 | 107780779 | PDSS2 | | | | | |
| 6 | 107811316 | 107982513 | SOBP | | SOBP | | | |
| 6 | 108023360 | 108145521 | SCML4 | | | | | |
| 6 | 108188959 | 108279482 | SEC63 | | | | | |
| 6 | 108362612 | 108395941 | OSTM1 | | | | | |
| 6 | 108487261 | 108510013 | NR2E1 | | NR2E1 | | | |
| 6 | 108532420 | 108582464 | SNX3 | | | | | |
| 6 | 108616097 | 108844251 | LACE1 | | | | | |
| 6 | 108881025 | 109005971 | FOXO3 | | | FOXO3 | | |
| 6 | 109072856 | 109091145 | LINC00222 | | | | | |
| 6 | 109169618 | 109245306 | ARMC2 | | | | | |
| 6 | 109307639 | 109415708 | SESN1 | | | | | |
| 6 | 109416355 | 109485115 | CEP57L1 | | | | | |
| 6 | 109557816 | 109591717 | LOC100996634 | | | | | |
| 6 | 109615505 | 109629423 | CCDC162P | | | | | |
| 6 | 109687716 | 109703762 | CD164 | | | | | |
| 6 | 109711417 | 109762374 | PPIL6 | | | | | |
| 6 | 109761930 | 109765122 | SMPD2 | | | | | |
| 6 | 109765265 | 109787171 | MICAL1 | | | MICAL1 | | |
| 6 | 109783718 | 109804440 | ZBTB24 | | | | | |
| 6 | 109814058 | 110012415 | AK9 | | | | | |

| | | | | | | | | |
|---|---|---|---|---|---|---|---|---|
| 6 | 110012423 | 110146634 | FIG4 | | FIG4 | | | |
| 6 | 110074369 | 110074419 | MIR7641 | | | | | |
| 6 | 110299458 | 110301923 | GPR6 | | | | | |
| 6 | 110421021 | 110501207 | WASF1 | | | | | |
| 6 | 110501623 | 110553422 | CDC40 | | | | | |
| 6 | 110567148 | 110679475 | METTL24 | | | | | |
| 6 | 110713382 | 110736753 | DDO | | | DDO | | |
| 6 | 110745891 | 110797844 | SLC22A16 | | | | | |
| 6 | 110931180 | 111137088 | CDK19 | | | | | |
| 6 | 111135823 | 111216915 | AMD1 | | | | | |
| 6 | 111279762 | 111289091 | GTF3C6 | | | | | |
| 6 | 111303219 | 111349466 | RPF2 | | | | | |
| 6 | 111367621 | 111368757 | GSTM2P1 | | | | | |
| 6 | 111408780 | 111544606 | SLC16A10 | | | | | |
| 6 | 111580481 | 111590261 | MFSD4B | | | | | |
| 6 | 111620233 | 111804918 | REV3L | | | | | |
| 6 | 111804674 | 111927154 | TRAF3IP2 | | | | | |
| 6 | 111981534 | 112194655 | FYN | | | FYN | | |
| 6 | 112375277 | 112390887 | WISP3 | | | | | |
| 6 | 112391859 | 112408751 | TUBE1 | | | TUBE1 | | |
| 6 | 112408673 | 112423993 | FAM229B | | | | | |
| 6 | 112429133 | 112575917 | LAMA4 | | | | | |
| 6 | 112557796 | 112627885 | LOC101927640 | | | | | |
| 6 | 112574983 | 112575917 | LAMA4 | | | | | |
| 6 | 112668531 | 112672498 | RFPL4B | | | | | |
| 6 | 113944736 | 113971277 | LOC101927686 | | | | | |
| 6 | 114178513 | 114184652 | MARCKS | | | MARCKS | | |
| 6 | 114189178 | 114194512 | LINC01268 | | | | | |
| 6 | 114225550 | 114242806 | FLJ34503 | | | | | |
| 6 | 114257319 | 114292359 | HDAC2 | | | | | |
| 6 | 114290864 | 114661893 | LOC101927768 | | | | | |
| 6 | 114376749 | 114384041 | HS3ST5 | | | | | |
| 6 | 115954705 | 115964999 | LOC105377962 | | | | | |
| 6 | 116262692 | 116381921 | FRK | | | | | |
| 6 | 116359893 | 116361107 | TPI1P3 | | | | | |
| 6 | 116421998 | 116566853 | NT5DC1 | | | | | |
| 6 | 116440084 | 116447296 | COL10A1 | | | COL10A1 | | |
| 6 | 116571130 | 116575261 | TSPYL4 | | | | | |
| 6 | 116596021 | 116601280 | TSPYL1 | | | | | |
| 6 | 116601282 | 116759442 | DSE | | | | | |
| 6 | 116782532 | 116784934 | FAM26F | | | | | |
| 6 | 116817650 | 116866773 | TRAPPC3L | | | | | |
| 6 | 116832807 | 116839709 | FAM26E | | | | | |
| 6 | 116850175 | 116880031 | FAM26D | | | | | |
| 6 | 116892529 | 116914764 | RWDD1 | | | | | |
| 6 | 116937641 | 116954148 | RSPH4A | | | | | |
| 6 | 116956780 | 116989973 | ZUFSP | | | | | |
| 6 | 117002366 | 117063030 | KPNA5 | | | | | |
| 6 | 117073359 | 117086886 | FAM162B | | | | | |
| 6 | 117113247 | 117150220 | GPRC6A | | | | | |
| 6 | 117198375 | 117253326 | RFX6 | | | | | |
| 6 | 117586720 | 117594728 | VGLL2 | | | | | |
| 6 | 117609529 | 117747018 | ROS1 | | | | | |
| 6 | 117803766 | 117891020 | DCBLD1 | | | | | |
| 6 | 117881432 | 117923705 | GOPC | | | | | |
| 6 | 117993975 | 117996447 | LOC101927919 | | | | | |
| 6 | 117996616 | 118031886 | NUS1 | | | | | |
| 6 | 118228688 | 118638839 | SLC35F1 | | | | | |
| 6 | 118407065 | 118416635 | LOC105377967 | | | | | |
| 6 | 118781934 | 118973020 | CEP85L | | | | | |
| 6 | 118822535 | 118824996 | BRD7P3 | | | | | |
| 6 | 118869441 | 118881587 | PLN | | | | | |
| 6 | 119103870 | 119104581 | LOC100287632 | | | | | |
| 6 | 119134611 | 119252903 | MCM9 | | | | | |
| 6 | 119215240 | 119230335 | ASF1A | | | | | |
| 6 | 119231761 | 119256327 | MCM9 | | | | | |

| | | | | | | | | |
|---|---|---|---|---|---|---|---|---|
| 6 | 119280993 | 119470358 | FAM184A | | | | | |
| 6 | 119390211 | 119390308 | MIR548B | | | | | |
| 6 | 119498365 | 119670931 | MAN1A1 | | | | | |
| 6 | 119773711 | 119812467 | LOC285762 | | | | | |
| 6 | 119870972 | 120166230 | LOC105377975 | | | | | |
| 6 | 120336324 | 120336403 | MIR3144 | | | | | |
| 6 | 121400639 | 121655646 | TBC1D32 | | | | | |
| 6 | 121756722 | 121770890 | GJA1 | | GJA1 | GJA1 | | |
| 6 | 122720695 | 122754264 | HSF2 | | | | | |
| 6 | 122764492 | 122793026 | SERINC1 | | | | | |
| 6 | 122793061 | 123047518 | PKIB | | | | | |
| 6 | 123070345 | 123105222 | FABP7 | | FABP7 | | | |
| 6 | 123110193 | 123130864 | SMPDL3A | | | | | |
| 6 | 123317115 | 123394064 | CLVS2 | | | | | |
| 6 | 123537483 | 123958238 | TRDN | | | | | |
| 6 | 123760748 | 123792901 | HRAT13 | | | | | |
| 6 | 123785395 | 123958238 | TRDN | | | | | |
| 6 | 124124990 | 125146786 | NKAIN2 | | | | | |
| 6 | 125229391 | 125407484 | RNF217 | | | | | |
| 6 | 125474874 | 125585552 | TPD52L1 | | | | | |
| 6 | 125596495 | 125623282 | HDDC2 | | | | | |
| 6 | 125995498 | 126041364 | LOC643623 | | | | | |
| 6 | 126070731 | 126082415 | HEY2 | | | | | |
| 6 | 126102306 | 126253176 | NCOA7 | | | | | |
| 6 | 126277860 | 126301389 | HINT3 | | | | | |
| 6 | 126307575 | 126360420 | TRMT11 | | | | | |
| 6 | 126433836 | 126443762 | MIR5695 | | | | | |
| 6 | 126660934 | 126804466 | CENPW | | | | | |
| 6 | 126805776 | 126805859 | MIR588 | | | | | |
| 6 | 127440047 | 127520626 | RSPO3 | | | | | |
| 6 | 127587826 | 127609705 | RNF146 | | | | | |
| 6 | 127609856 | 127664754 | ECHDC1 | | | | | |
| 6 | 127759550 | 127780535 | KIAA0408 | | | | | |
| 6 | 127759550 | 127840500 | SOGA3 | | | | | |
| 6 | 127898318 | 127912963 | C6orf58 | | | | | |
| 6 | 128029338 | 128239776 | THEMIS | | | | | |
| 6 | 128289923 | 128841819 | PTPRK | | | | | |
| 6 | 128349009 | 128407435 | LOC101928140 | | | | | |
| 6 | 129204285 | 129837710 | LAMA2 | | | | | |
| 6 | 129898239 | 130031370 | ARHGAP18 | | | | | |
| 6 | 130152388 | 130182416 | TMEM244 | | | | | |
| 6 | 130339727 | 130462594 | L3MBTL3 | | | | | |
| 6 | 130465446 | 130686570 | SAMD3 | | | | | |
| 6 | 130687425 | 130764210 | TMEM200A | | | | | |
| 6 | 131148544 | 131158276 | SMLR1 | | | | | |
| 6 | 131160487 | 131384462 | EPB41L2 | | | | | |
| 6 | 131456825 | 131604675 | AKAP7 | | | AKAP7 | | |
| 6 | 131894343 | 131905472 | ARG1 | | | ARG1 | | |
| 6 | 131895105 | 131949379 | MED23 | | | | | |
| 6 | 131958374 | 132068550 | ENPP3 | | | | | |
| 6 | 132020783 | 132022541 | OR2A4 | | | | | |
| 6 | 132029580 | 132032157 | CTAGE9 | | | | | |
| 6 | 132113311 | 132113371 | MIR548H5 | | | | | |
| 6 | 132129155 | 132216295 | ENPP1 | | | ENPP1 | | |
| 6 | 132269316 | 132272518 | CTGF | | | | | |
| 6 | 132436331 | 132436403 | MIR548AJ1 | | | | | |
| 6 | 132455117 | 132490514 | LINC01013 | | | | | |
| 6 | 132617193 | 132722673 | MOXD1 | | | | | |
| 6 | 132778662 | 132834337 | STX7 | | STX7 | STX7 | | |
| 6 | 132859426 | 132860475 | TAAR9 | | | | | |
| 6 | 132873831 | 132874860 | TAAR8 | | | | | |
| 6 | 132891460 | 132892498 | TAAR6 | | | | TAAR6 | |
| 6 | 132909730 | 132910877 | TAAR5 | | | | | |
| 6 | 132929363 | 132930441 | TAAR3 | | | | | |
| 6 | 132938288 | 132945414 | TAAR2 | | | TAAR2 | | |
| 6 | 132966036 | 132967165 | TAAR1 | | | | | |

| | | | | | | | | |
|---|---|---|---|---|---|---|---|---|
| 6 | 133001996 | 133035194 | VNN1 | | | | | |
| 6 | 133043925 | 133055904 | VNN3 | | | | | |
| 6 | 133065008 | 133084598 | VNN2 | | | | | |
| 6 | 133090506 | 133119747 | SLC18B1 | | | | | |
| 6 | 133135707 | 133138703 | RPS12 | | | | | |
| 6 | 133136445 | 133136518 | SNORD101 | | | | | |
| 6 | 133137940 | 133138016 | SNORD100 | | | | | |
| 6 | 133138357 | 133138490 | SNORA33 | | | | | |
| 6 | 133409218 | 133427717 | LINC00326 | | | | | |
| 6 | 133562494 | 133853258 | EYA4 | | | EYA4 | | |
| 6 | 133823389 | 134210144 | TARID | | | | | |
| 6 | 134142284 | 134175130 | LINC01312 | | | | | |
| 6 | 134210258 | 134213393 | TCF21 | | | TCF21 | | |
| 6 | 134273307 | 134308638 | TBPL1 | | | | | |
| 6 | 134308718 | 134373789 | SLC2A12 | | | | | |
| 6 | 134435110 | 134438818 | HMGA1P7 | | | | | |
| 6 | 134490383 | 134639196 | SGK1 | | | SGK1 | | |
| 6 | 134750167 | 134800035 | LOC101928231 | | | | | |
| 6 | 134758853 | 134825158 | LINC01010 | | | | | |
| 6 | 134846455 | 134861143 | LOC101928304 | | | | | |
| 6 | 135238527 | 135271260 | ALDH8A1 | | ALDH8A1 | | | |
| 6 | 135281516 | 135376036 | HBS1L | | HBS1L | | | |
| 6 | 135300475 | 135300570 | MIR3662 | | | | | |
| 6 | 135356994 | 135376036 | HBS1L | | HBS1L | | | |
| 6 | 135502452 | 135540311 | MYB | | | | | |
| 6 | 135605109 | 135818903 | AHI1 | | AHI1 | AHI1 | | |
| 6 | 135818938 | 136011976 | LINC00271 | | | | | |
| 6 | 136172833 | 136516709 | PDE7B | | PDE7B | | | |
| 6 | 136552167 | 136571473 | MTFR2 | | | | | |
| 6 | 136578000 | 136610989 | BCLAF1 | | | | | |
| 6 | 136663418 | 136871957 | MAP7 | | | | | |
| 6 | 136878186 | 137113656 | MAP3K5 | | MAP3K5 | MAP3K5 | | |
| 6 | 136950251 | 136969336 | LOC101928461 | | | | | |
| 6 | 137105184 | 137107192 | LOC101928429 | | | | | |
| 6 | 137143701 | 137235072 | PEX7 | | | PEX7 | | |
| 6 | 137243378 | 137246798 | SLC35D3 | | | | | |
| 6 | 137303295 | 137314368 | NHEG1 | | | | | |
| 6 | 137321107 | 137366317 | IL20RA | | | | | |
| 6 | 137464956 | 137494785 | IL22RA2 | | | IL22RA2 | | |
| 6 | 137518620 | 137540567 | IFNGR1 | | | | | |
| 6 | 137813335 | 137815531 | OLIG3 | | | OLIG3 | | |
| 6 | 137986782 | 137995691 | LOC102723649 | | | | | |
| 6 | 138051306 | 138060174 | LOC100507406 | | | | | |
| 6 | 138144806 | 138189370 | LOC100130476 | | | | | |
| 6 | 138188324 | 138204451 | TNFAIP3 | | | | | |
| 6 | 138409641 | 138428660 | PERP | | | | | |
| 6 | 138483052 | 138665800 | ARFGEF3 | | | | | |
| 6 | 138537126 | 138539627 | PBOV1 | | | | | |
| 6 | 138725335 | 138734582 | HEBP2 | | | | | |
| 6 | 138743180 | 138893668 | NHSL1 | | | | | |
| 6 | 138756349 | 138756431 | MIR3145 | | | | | |
| 6 | 139012804 | 139018425 | FLJ46906 | | | | | |
| 6 | 139046347 | 139094816 | GVQW2 | | | | | |
| 6 | 139094656 | 139114456 | CCDC28A | | | | | |
| 6 | 139117247 | 139225207 | ECT2L | | | | | |
| 6 | 139225151 | 139309398 | REPS1 | | | | REPS1 | |
| 6 | 139349818 | 139364439 | ABRACL | | | | | |
| 6 | 139456248 | 139501946 | HECA | | | | | |
| 6 | 139561198 | 139613208 | TXLNB | | | TXLNB | | |
| 6 | 139693391 | 139695787 | CITED2 | | | | | |
| 6 | 139790131 | 139795733 | LINC01625 | | | | | |
| 6 | 140092209 | 140181608 | LOC100132735 | | | | | |
| 6 | 140297455 | 140414858 | LOC100507477 | | | | | |
| 6 | 140299486 | 140312398 | LOC103352541 | | | | | |
| 6 | 140526388 | 140526463 | MIR3668 | | | | | |
| 6 | 141004950 | 141005020 | MIR4465 | | | | | |

| | | | | | | | | |
|---|---|---|---|---|---|---|---|---|
| 6 | 142396744 | 142409936 | NMBR | | | NMBR | | |
| 6 | 142468298 | 142542085 | VTA1 | | | | | |
| 6 | 142623055 | 142767403 | ADGRG6 | | | | | |
| 6 | 142847591 | 142959026 | LOC153910 | | | | | |
| 6 | 143072603 | 143266338 | HIVEP2 | | | | | |
| 6 | 143287558 | 143358719 | LINC01277 | | | | | |
| 6 | 143380499 | 143661480 | AIG1 | | | | | |
| 6 | 143743968 | 143771841 | ADAT2 | | | | | |
| 6 | 143771917 | 143811751 | PEX3 | | | | | |
| 6 | 143815948 | 143833020 | FUCA2 | | | FUCA2 | | |
| 6 | 143875251 | 144152322 | PHACTR2 | | | | | |
| 6 | 144164507 | 144184943 | LTV1 | | | | | |
| 6 | 144185572 | 144259483 | ZC2HC1B | | | | | |
| 6 | 144261436 | 144385736 | PLAGL1 | | | PLAGL1 | | |
| 6 | 144326052 | 144329867 | HYMAI | | | HYMAI | | |
| 6 | 144416017 | 144416754 | SF3B5 | | | | | |
| 6 | 144471653 | 144513076 | STX11 | | | STX11 | | |
| 6 | 144612872 | 145174170 | UTRN | | | | | UTRN |
| 6 | 144726790 | 144726903 | SNORA98 | | | | | |
| 6 | 145946439 | 146056991 | EPM2A | | | | | |
| 6 | 146056004 | 146207721 | LOC100507557 | | | | | |
| 6 | 146119271 | 146135921 | FBXO30 | | | | | |
| 6 | 146136011 | 146207721 | LOC100507557 | | | | | |
| 6 | 146205944 | 146285233 | SHPRH | | | | | |
| 6 | 146348917 | 146758734 | GRM1 | | GRM1 | GRM1 | GRM1 | GRM1 |
| 6 | 146864827 | 146876092 | RAB32 | | | RAB32 | | |
| 6 | 146915651 | 146920067 | LOC101928661 | | | | | |
| 6 | 146920135 | 147136597 | ADGB | | | | | |
| 6 | 147122804 | 147124960 | KATNBL1P6 | | | | | |
| 6 | 147162524 | 147525750 | STXBP5 | | | | | |
| 6 | 147480060 | 147502128 | LUADT1 | | | | | |
| 6 | 147525493 | 147711612 | STXBP5 | | | | | |
| 6 | 147829827 | 147891157 | SAMD5 | | | | | |
| 6 | 148663728 | 148873184 | SASH1 | | | | | |
| 6 | 149068270 | 149285820 | UST | | | | | |
| 6 | 149348364 | 149353797 | LOC105378047 | | | | | |
| 6 | 149539059 | 149732749 | TAB2 | | | | | |
| 6 | 149721494 | 149722182 | SUMO4 | | | | | |
| 6 | 149768765 | 149806148 | ZC3H12D | | | | | |
| 6 | 149825630 | 149867238 | PPIL4 | | | | | |
| 6 | 149887527 | 149912067 | GINM1 | | | | | |
| 6 | 149914751 | 149915720 | RPS18P9 | | | | | |
| 6 | 149916010 | 149969940 | KATNA1 | | | KATNA1 | | |
| 6 | 149979288 | 150039392 | LATS1 | | | | | |
| 6 | 150038756 | 150040031 | LOC645967 | | | | | |
| 6 | 150045451 | 150067708 | NUP43 | | | | | |
| 6 | 150070830 | 150132557 | PCMT1 | | PCMT1 | | | |
| 6 | 150139893 | 150185480 | LRP11 | | | | | |
| 6 | 150184633 | 150240644 | RAET1E | | | | | |
| 6 | 150238013 | 150244288 | RAET1G | | | | | |
| 6 | 150255648 | 150257455 | LOC105378052 | | | | | |
| 6 | 150263073 | 150270368 | ULBP2 | | | | | |
| 6 | 150285109 | 150294849 | ULBP1 | | | | | |
| 6 | 150319154 | 150326280 | RAET1K | | | | | |
| 6 | 150341265 | 150346668 | RAET1L | | | | | |
| 6 | 150382181 | 150390283 | ULBP3 | | | | | |
| 6 | 150464187 | 150571528 | PPP1R14C | | | | | |
| 6 | 150690027 | 150725765 | IYD | | | | | |
| 6 | 150920998 | 151164799 | PLEKHG1 | | | | | |
| 6 | 151186814 | 151423023 | MTHFD1L | | | | | |
| 6 | 151517807 | 151549592 | LOC102723831 | | | | | |
| 6 | 151561133 | 151679694 | AKAP12 | | | AKAP12 | | |
| 6 | 151685249 | 151712835 | ZBTB2 | | | | | |
| 6 | 151725896 | 151773316 | RMND1 | | | | | |
| 6 | 151773392 | 151791234 | ARMT1 | | | | | |
| 6 | 151815174 | 151942328 | CCDC170 | | | | | |

| | | | | | | | | |
|---|---|---|---|---|---|---|---|---|
| 6 | 152011630 | 152424408 | ESR1 | | | ESR1 | ESR1 | |
| 6 | 152442818 | 152958534 | SYNE1 | | | SYNE1 | | SYNE1 |
| 6 | 152469761 | 152546267 | MIR3163 | | | | | |
| 6 | 152701664 | 152702847 | SYNE1 | | | SYNE1 | | SYNE1 |
| 6 | 153019029 | 153045715 | MYCT1 | | | | | |
| 6 | 153071931 | 153080902 | VIP | | | | | |
| 6 | 153291657 | 153304740 | FBXO5 | | | | | |
| 6 | 153308500 | 153323950 | MTRF1L | | | | | |
| 6 | 153332031 | 153452389 | RGS17 | | | | | |
| 6 | 153741611 | 153741661 | MIR7641 | | | | | |
| 6 | 154331630 | 154413073 | OPRM1 | | | OPRM1 | OPRM1 | |
| 6 | 154475617 | 154677900 | IPCEF1 | | | | | |
| 6 | 154726432 | 154831753 | CNKSR3 | | | | | |
| 6 | 155054511 | 155155378 | SCAF8 | | | | | |
| 6 | 155174493 | 155174570 | MIR1273C | | | | | |
| 6 | 155411422 | 155578857 | TIAM2 | | | | | |
| 6 | 155577263 | 155635617 | TFB1M | | | | | |
| 6 | 155585146 | 155597682 | CLDN20 | | | | | |
| 6 | 155716501 | 155777037 | NOX3 | | | | | |
| 6 | 155835977 | 155847152 | LOC105378068 | | | | | |
| 6 | 156267930 | 156268013 | MIR1202 | | | | | |
| 6 | 157099063 | 157531913 | ARID1B | | | | | |
| 6 | 157100811 | 157100865 | MIR4466 | | | | | |
| 6 | 157710053 | 157745291 | TMEM242 | | | | | |
| 6 | 157802556 | 158094977 | ZDHHC14 | | | | | |
| 6 | 157950163 | 157950232 | MIR3692 | | | | | |
| 6 | 158244202 | 158366109 | SNX9 | | | SNX9 | | |
| 6 | 158402887 | 158520207 | SYNJ2 | | SYNJ2 | | | SYNJ2 |
| 6 | 158422138 | 158423415 | SYNJ2-IT1 | | | | | |
| 6 | 158438079 | 158520207 | SYNJ2 | | SYNJ2 | | | SYNJ2 |
| 6 | 158530535 | 158589312 | SERAC1 | | | | | |
| 6 | 158589378 | 158620376 | GTF2H5 | | | | | |
| 6 | 158733691 | 158932856 | TULP4 | | | | | |
| 6 | 158957467 | 159056467 | TMEM181 | | | | | |
| 6 | 159030738 | 159030822 | MIR7161 | | | | | |
| 6 | 159057506 | 159065818 | DYNLT1 | | | DYNLT1 | | |
| 6 | 159071045 | 159185908 | SYTL3 | | | | | |
| 6 | 159185692 | 159185785 | MIR3918 | | | | | |
| 6 | 159186772 | 159243272 | EZR | | | | | |
| 6 | 159262157 | 159278664 | OSTCP1 | | | | | |
| 6 | 159290898 | 159331385 | C6orf99 | | | | | |
| 6 | 159398265 | 159421198 | RSPH3 | | | | | |
| 6 | 159455500 | 159466184 | TAGAP | | | | | |
| 6 | 159586930 | 159591039 | LOC101929122 | | | | | |
| 6 | 159590428 | 159693140 | FNDC1 | | | | | |
| 6 | 159804562 | 159817473 | LOC102724053 | | | | | |
| 6 | 160100148 | 160114353 | SOD2 | | | | | |
| 6 | 160148029 | 160177352 | WTAP | | | | | |
| 6 | 160181290 | 160183364 | LOC100129518 | | | | | |
| 6 | 160182988 | 160200144 | ACAT2 | | | | | |
| 6 | 160199529 | 160210735 | TCP1 | | | | | |
| 6 | 160201281 | 160201413 | SNORA20 | | | | | |
| 6 | 160206625 | 160206765 | SNORA29 | | | | | |
| 6 | 160210843 | 160219461 | MRPL18 | | | MRPL18 | | |
| 6 | 160221280 | 160241736 | PNLDC1 | | | | | |
| 6 | 160327973 | 160329107 | MAS1 | | | | | |
| 6 | 160390130 | 160532534 | IGF2R | | | | | |
| 6 | 160424322 | 160428696 | AIRN | | | | | |
| 6 | 160514113 | 160517244 | LOC729603 | | | | | |
| 6 | 160542862 | 160579750 | SLC22A1 | | | | | |
| 6 | 160637793 | 160679963 | SLC22A2 | | | | | |
| 6 | 160769404 | 160873611 | SLC22A3 | | | SLC22A3 | | |
| 6 | 160887586 | 160932156 | LPAL2 | | | | | |
| 6 | 160952514 | 161087407 | LPA | | | | | |
| 6 | 161123224 | 161175085 | PLG | | | | | |
| 6 | 161412758 | 161538417 | MAP3K4 | | | MAP3K4 | | |

| | | | | | | | | |
|---|---|---|---|---|---|---|---|---|
| 6 | 161551056 | 161695107 | AGPAT4 | | | | | |
| 6 | 161581163 | 161583014 | AGPAT4-IT1 | | | | | |
| 6 | 161768589 | 163148834 | PARK2 | PARK2 | | PARK2 | | |
| 6 | 161892008 | 161905077 | LOC105378098 | | | | | |
| 6 | 163148163 | 163745505 | PACRG | | | | | |
| 6 | 163759373 | 163768065 | DKFZp451B082 | | | | | |
| 6 | 163834096 | 163834982 | CAHM | | | | | |
| 6 | 163835674 | 163999628 | QKI | | QKI | | | |
| 6 | 164085397 | 164092516 | LOC102724152 | | | | | |
| 6 | 165206865 | 165235552 | MEAT6 | | | | | |
| 6 | 165693152 | 165723111 | C6orf118 | | | | | |
| 6 | 165740775 | 166075588 | PDE10A | | | | | |
| 6 | 165823089 | 165823139 | MIR7641 | | | | | |
| 6 | 166337535 | 166401527 | LINC00473 | | | | | |
| 6 | 166401038 | 166403103 | LINC00602 | | | | | |
| 6 | 166571145 | 166582157 | T | | | | | |
| 6 | 166650456 | 166670432 | LOC101929297 | | | | | |
| 6 | 166719167 | 166721871 | PRR18 | | | | | |
| 6 | 166733215 | 166756094 | SFT2D1 | | | | | |
| 6 | 166756118 | 166764957 | LOC100289495 | | | | | |
| 6 | 166778407 | 166796501 | MPC1 | | | | | |
| 6 | 166822851 | 167276039 | RPS6KA2 | | | | | |
| 6 | 166874150 | 166878871 | RPS6KA2-IT1 | | | | | |
| 6 | 166922841 | 166922921 | MIR1913 | | | | | |
| 6 | 167317185 | 167318557 | RPS6KA2 | | | | | |
| 6 | 167343003 | 167370077 | RNASET2 | | | | | |
| 6 | 167411294 | 167411400 | MIR3939 | | | | | |
| 6 | 167412804 | 167455906 | FGFR1OP | | | | | |
| 6 | 167525294 | 167552629 | CCR6 | | | CCR6 | | |
| 6 | 167570359 | 167571319 | GPR31 | | | | | |
| 6 | 167578836 | 167583468 | LOC105378123 | | | | | |
| 6 | 167584080 | 167596396 | TCP10L2 | | | | | |
| 6 | 167704802 | 167729502 | UNC93A | | | | | |
| 6 | 167738573 | 167756177 | TTLL2 | | | | | |
| 6 | 167786576 | 167797998 | TCP10 | | | | | |
| 6 | 167871476 | 167885013 | LOC105378127 | | | | | |
| 6 | 168067521 | 168079903 | LOC401286 | | | | | |
| 6 | 168080305 | 168084467 | LOC441178 | | | | | |
| 6 | 168185218 | 168197539 | LINC01558 | | | | | |
| 6 | 168224569 | 168372700 | MLLT4 | | | | | |
| 6 | 168376603 | 168377619 | HGC6.3 | | | | | |
| 6 | 168394866 | 168445769 | KIF25 | | | | | |
| 6 | 168456463 | 168482237 | FRMD1 | | | | | |
| 6 | 168625960 | 168627197 | LOC105378137 | | | | | |
| 6 | 168643617 | 168663258 | LOC101929420 | | | | | |
| 6 | 168693509 | 168720434 | DACT2 | | | | | |
| 6 | 168841830 | 169068674 | SMOC2 | | | | | |
| 6 | 169434265 | 169435738 | LOC101929460 | | | | | |
| 6 | 169467880 | 169469519 | LOC102724357 | | | | | |
| 6 | 169558186 | 169563084 | LINC01615 | | | | | |
| 6 | 169575398 | 169582835 | LOC101929504 | | | | | |
| 6 | 169613306 | 169639657 | LOC101929523 | | | | | |
| 6 | 169615874 | 169654209 | THBS2 | | | | | |
| 6 | 169857302 | 170102159 | WDR27 | | | | | |
| 6 | 170102207 | 170106453 | C6orf120 | | | | | |
| 6 | 170104001 | 170124106 | PHF10 | | | | | |
| 6 | 170140214 | 170151638 | TCTE3 | | | | | |
| 6 | 170151717 | 170181680 | ERMARD | | | | | |
| 6 | 170188885 | 170198921 | LINC00242 | | | | | |
| 6 | 170190168 | 170202969 | LINC00574 | | | | | |
| 6 | 170475865 | 170478251 | LOC102724511 | | | | | |
| 6 | 170563421 | 170571657 | LOC154449 | | | | | |
| 6 | 170575756 | 170585850 | LOC285804 | | | | | |
| 6 | 170581561 | 170588554 | LINC01624 | | | | | |
| 6 | 170591293 | 170599697 | DLL1 | | | DLL1 | | |
| 6 | 170599790 | 170716159 | FAM120B | | | | | |

| | | | | | | | | |
|---|---|---|---|---|---|---|---|---|
| 6 | 170639848 | 170639932 | MIR4644 | | | | | |
| 6 | 170844203 | 170862417 | PSMB1 | | | | | |
| 6 | 170863420 | 170881958 | TBP | | | | | |
| 6 | 170884659 | 170893780 | PDCD2 | | | | | |
| 7 | 144444 | 149438 | LOC102723672 | | | | | |
| 7 | 149717 | 155461 | LOC100507642 | | | | | |
| 7 | 174919 | 176013 | LOC105375115 | | | | | |
| 7 | 192968 | 300740 | FAM20C | | | | | |
| 7 | 330135 | 331454 | WI2-2373I1.2 | | | | | |
| 7 | 419390 | 422845 | LOC442497 | | | | | |
| 7 | 536896 | 559481 | PDGFA | | PDGFA | | | |
| 7 | 560027 | 564869 | HRAT92 | | | | | |
| 7 | 588833 | 767313 | PRKAR1B | PRKAR1B | PRKAR1B | | | |
| 7 | 601594 | 605256 | LOC101927000 | | | | | |
| 7 | 642481 | 648139 | LOC101926963 | | | | | |
| 7 | 766337 | 826116 | DNAAF5 | | | | | |
| 7 | 855193 | 914557 | SUN1 | | | | | |
| 7 | 916190 | 936071 | GET4 | | | | | |
| 7 | 937536 | 995043 | ADAP1 | | | | | |
| 7 | 1004485 | 1015235 | COX19 | | | | | |
| 7 | 1022834 | 1029276 | CYP2W1 | | | | | |
| 7 | 1036621 | 1177893 | C7orf50 | | | | | |
| 7 | 1062568 | 1062662 | MIR339 | | | | | |
| 7 | 1064133 | 1068068 | C7orf50 | | | | | |
| 7 | 1084208 | 1098905 | GPR146 | | | | | |
| 7 | 1126442 | 1133451 | GPER1 | | | | | |
| 7 | 1192542 | 1199855 | ZFAND2A | | | | | |
| 7 | 1200009 | 1205594 | LOC101927021 | | | | | |
| 7 | 1272653 | 1276613 | UNCX | | | | | |
| 7 | 1473994 | 1499109 | MICALL2 | | | | | |
| 7 | 1509912 | 1544018 | INTS1 | | | | | |
| 7 | 1570367 | 1582679 | MAFK | | | | | |
| 7 | 1581870 | 1596066 | TMEM184A | | | | | |
| 7 | 1606969 | 1629261 | PSMG3 | | | | | |
| 7 | 1654105 | 1656328 | TFAMP1 | | | | | |
| 7 | 1748797 | 1781946 | ELFN1 | | | | | |
| 7 | 1855427 | 2272583 | MAD1L1 | | | | | |
| 7 | 1883815 | 1883889 | MIR4655 | | | | | |
| 7 | 2273925 | 2281833 | FTSJ2 | | | | | |
| 7 | 2281856 | 2290780 | NUDT1 | | | | | |
| 7 | 2291404 | 2354110 | SNX8 | | | SNX8 | | |
| 7 | 2297149 | 2297212 | MIR6836 | | | | | |
| 7 | 2394473 | 2420377 | EIF3B | | | | | |
| 7 | 2443194 | 2474216 | CHST12 | | | | | |
| 7 | 2477397 | 2487485 | LOC101927181 | | | | | |
| 7 | 2514771 | 2516017 | GRIFIN | | | | | |
| 7 | 2552162 | 2568810 | LFNG | | | | | |
| 7 | 2566707 | 2566779 | MIR4648 | | | | | |
| 7 | 2577443 | 2595392 | BRAT1 | | | | | |
| 7 | 2598605 | 2654368 | IQCE | | | | | |
| 7 | 2671602 | 2704436 | TTYH3 | | | | | |
| 7 | 2719155 | 2755070 | AMZ1 | | | | | |
| 7 | 2767740 | 2883959 | GNA12 | | | | | |
| 7 | 2945709 | 3083579 | CARD11 | | | | | |
| 7 | 3180564 | 3198206 | LOC100129603 | | | | | |
| 7 | 3341079 | 4308631 | SDK1 | | | | | |
| 7 | 4721929 | 4811074 | FOXK1 | | | | | |
| 7 | 4815261 | 4834026 | AP5Z1 | | | | | |
| 7 | 4828195 | 4828270 | MIR4656 | | | | | |
| 7 | 4838739 | 4923335 | RADIL | | | | | |
| 7 | 4897368 | 4901625 | PAPOLB | | | | | |
| 7 | 4931875 | 4998844 | MMD2 | | | | | |
| 7 | 5013615 | 5037800 | RNF216P1 | | | | | |
| 7 | 5085451 | 5109119 | RBAK | | | | | |
| 7 | 5085451 | 5112854 | RBAK-RBAKDN | | | | | |
| 7 | 5085552 | 5109119 | RBAK | | | | | |

| | | | | | | | | |
|---|---|---|---|---|---|---|---|---|
| 7 | 5111690 | 5112854 | RBAKDN | | | | | |
| 7 | 5160940 | 5184177 | ZNF890P | | | | | |
| 7 | 5229834 | 5273486 | WIPI2 | | | | | |
| 7 | 5322560 | 5343704 | SLC29A4 | | | | | |
| 7 | 5346422 | 5463177 | TNRC18 | | | | | |
| 7 | 5515427 | 5553399 | FBXL18 | | | FBXL18 | | |
| 7 | 5535449 | 5535548 | MIR589 | | | | | |
| 7 | 5553484 | 5565176 | LOC221946 | | | | | |
| 7 | 5566778 | 5570232 | ACTB | | | | | |
| 7 | 5632435 | 5646287 | FSCN1 | | FSCN1 | FSCN1 | | |
| 7 | 5659671 | 5821361 | RNF216 | | | | | |
| 7 | 5702062 | 5720092 | RNF216-IT1 | | | | | |
| 7 | 5751470 | 5751541 | MIR6874 | | | | | |
| 7 | 5862790 | 5894066 | ZNF815P | | | | | |
| 7 | 5920428 | 5925994 | OCM | | | | | |
| 7 | 5938340 | 5965603 | CCZ1 | | | | | |
| 7 | 5965776 | 6010314 | RSPH10B | | | | | |
| 7 | 5965776 | 6010314 | RSPH10B2 | | | | | |
| 7 | 6012869 | 6048737 | PMS2 | | | | | |
| 7 | 6048881 | 6063465 | AIMP2 | | | | | |
| 7 | 6061877 | 6098860 | EIF2AK1 | | | | | |
| 7 | 6071006 | 6076183 | ANKRD61 | | | | | |
| 7 | 6144549 | 6201195 | USP42 | | | | | |
| 7 | 6201411 | 6312242 | CYTH3 | | | | | |
| 7 | 6369039 | 6388590 | FAM220A | | | | | |
| 7 | 6414125 | 6443598 | RAC1 | | | RAC1 | | |
| 7 | 6448746 | 6487643 | DAGLB | | | | | |
| 7 | 6500711 | 6523849 | KDELR2 | | | | | |
| 7 | 6536408 | 6591067 | GRID2IP | | | | | |
| 7 | 6617064 | 6628610 | ZDHHC4 | | | | | |
| 7 | 6629651 | 6648357 | C7orf26 | | | | | |
| 7 | 6655526 | 6663921 | ZNF853 | | | | | |
| 7 | 6676952 | 6697910 | ZNF316 | | | | | |
| 7 | 6728063 | 6746566 | ZNF12 | | | | | |
| 7 | 6774935 | 6791232 | PMS2CL | | | | | |
| 7 | 6793739 | 6838396 | RSPH10B | | | | | |
| 7 | 6793739 | 6838396 | RSPH10B2 | | | | | |
| 7 | 6838565 | 6865926 | CCZ1B | | | | | |
| 7 | 7106594 | 7106676 | MIR3683 | | | | | |
| 7 | 7115400 | 7136417 | LOC100131257 | | | | | |
| 7 | 7222245 | 7288280 | C1GALT1 | | | | | |
| 7 | 7294784 | 7317700 | LOC101927354 | | | | | |
| 7 | 7398243 | 7575460 | COL28A1 | | | | | |
| 7 | 7589734 | 7605696 | LOC101927391 | | | | | |
| 7 | 7606615 | 7647110 | MIOS | | | | | |
| 7 | 7676193 | 7758238 | RPA3 | | | | | |
| 7 | 7680266 | 7918851 | UMAD1 | | | | | |
| 7 | 7989469 | 8008383 | LOC100505921 | | | | | |
| 7 | 8008373 | 8128709 | GLCCI1 | | | | | |
| 7 | 8152814 | 8302242 | ICA1 | | | | | |
| 7 | 8301854 | 8382911 | LOC100505938 | | | | | |
| 7 | 8473584 | 8792593 | NXPH1 | | | | | |
| 7 | 9673899 | 9675447 | PER4 | | | | | |
| 7 | 10971579 | 10979813 | NDUFA4 | | NDUFA4 | | | |
| 7 | 11013498 | 11209250 | PHF14 | | | | | |
| 7 | 11410061 | 11871824 | THSD7A | | THSD7A | | | |
| 7 | 12250847 | 12276890 | TMEM106B | | | | | |
| 7 | 12370508 | 12443852 | VWDE | | | | | |
| 7 | 12610202 | 12693228 | SCIN | | | SCIN | | |
| 7 | 12726451 | 12730558 | ARL4A | | | | | |
| 7 | 13930855 | 14031050 | ETV1 | | ETV1 | | | |
| 7 | 14184673 | 14881075 | DGKB | | DGKB | | | |
| 7 | 15239942 | 15601640 | AGMO | | | | | |
| 7 | 15650836 | 15726308 | MEOX2 | | | | | |
| 7 | 15707571 | 15721604 | LOC105375166 | | | | | |
| 7 | 15728002 | 15736517 | MEOX2 | | | | | |

| | | | | | | | | |
|---|---|---|---|---|---|---|---|---|
| 7 | 16127151 | 16310229 | ISPD | | | | | |
| 7 | 16501105 | 16505474 | SOSTDC1 | | | | | |
| 7 | 16566504 | 16621114 | LRRC72 | | | | | |
| 7 | 16639400 | 16685442 | ANKMY2 | | | | | |
| 7 | 16685758 | 16746148 | BZW2 | | | | | |
| 7 | 16793350 | 16824161 | TSPAN13 | | | | | |
| 7 | 16832263 | 16844738 | AGR2 | | | | | |
| 7 | 16899029 | 16921613 | AGR3 | | | | | |
| 7 | 17338275 | 17385775 | AHR | | | | | |
| 7 | 17414540 | 17506880 | KCCAT333 | | | | | |
| 7 | 17503068 | 17598533 | LOC101927630 | | | | | |
| 7 | 17830384 | 17980131 | SNX13 | | | | | |
| 7 | 18066399 | 18067486 | PRPS1L1 | | | | | |
| 7 | 18126571 | 18708466 | HDAC9 | | | | | |
| 7 | 18166842 | 18166932 | MIR1302 | | | | | |
| 7 | 18535368 | 18708466 | HDAC9 | | | | | |
| 7 | 19155090 | 19157295 | TWIST1 | | | TWIST1 | | |
| 7 | 19184404 | 19185044 | FERD3L | | | | | |
| 7 | 19735084 | 19748660 | TWISTNB | | | | | |
| 7 | 19744980 | 19745059 | MIR3146 | | | | | |
| 7 | 19758937 | 19812404 | TMEM196 | | | | | |
| 7 | 19958603 | 20180049 | LOC101927668 | | | | | |
| 7 | 20174278 | 20193154 | MACC1 | | | | | |
| 7 | 20257199 | 20261322 | LOC100506098 | | | | | |
| 7 | 20336330 | 20351335 | LOC101927769 | | | | | |
| 7 | 20367923 | 20371385 | LOC101927811 | | | | | |
| 7 | 20370724 | 20455382 | ITGB8 | | | | | |
| 7 | 20655244 | 20796637 | ABCB5 | | | | | |
| 7 | 20821893 | 20826508 | SP8 | | | | | |
| 7 | 20866916 | 20867439 | RPL23P8 | | | | | |
| 7 | 20875049 | 21061771 | LINC01162 | | | | | |
| 7 | 21467688 | 21554151 | SP4 | | | | | |
| 7 | 21510675 | 21510764 | MIR1183 | | | | | |
| 7 | 21582832 | 21941186 | DNAH11 | | | | | |
| 7 | 21940516 | 21985542 | CDCA7L | | | | | |
| 7 | 22157907 | 22396533 | RAPGEF5 | | | | | |
| 7 | 22459062 | 22539901 | STEAP1B | | | | | |
| 7 | 22602955 | 22613617 | LOC100506178 | | | | | |
| 7 | 22689578 | 22705152 | LOC401312 | | | | | |
| 7 | 22765013 | 22767239 | LOC541472 | | | | | |
| 7 | 22766760 | 22771621 | IL6 | | | | | |
| 7 | 22852250 | 22862471 | TOMM7 | | | TOMM7 | | |
| 7 | 22896231 | 22896305 | SNORD93 | | | | | |
| 7 | 22980877 | 23053770 | FAM126A | | | | | |
| 7 | 23140846 | 23215038 | KLHL7 | | | | | |
| 7 | 23221445 | 23240630 | NUPL2 | | | | | |
| 7 | 23286315 | 23314729 | GPNMB | | | | | |
| 7 | 23338939 | 23349180 | MALSU1 | | | | | |
| 7 | 23349827 | 23509995 | IGF2BP3 | | | | | |
| 7 | 23530006 | 23531031 | RPS2P32 | | | | | |
| 7 | 23544400 | 23571660 | TRA2A | | | | | |
| 7 | 23624334 | 23626146 | CLK2P1 | | | | | |
| 7 | 23636997 | 23684327 | CCDC126 | | | | | |
| 7 | 23719732 | 23742269 | FAM221A | | | | | |
| 7 | 23749785 | 23872130 | STK31 | | | | | |
| 7 | 24323806 | 24331484 | NPY | | NPY | | | |
| 7 | 24612964 | 24733322 | MPP6 | | | | | |
| 7 | 24737973 | 24797639 | DFNA5 | | | | | |
| 7 | 24836155 | 25019831 | OSBPL3 | | | | | |
| 7 | 25158269 | 25164980 | CYCS | | | CYCS | | |
| 7 | 25174315 | 25219817 | C7orf31 | | | | | |
| 7 | 25264190 | 25268105 | NPVF | | | | | |
| 7 | 25989538 | 25989606 | MIR148A | | | | | |
| 7 | 26191846 | 26226756 | NFE2L3 | | | | | |
| 7 | 26229555 | 26240413 | HNRNPA2B1 | | | | | |
| 7 | 26240830 | 26253227 | CBX3 | | | | | |

| | | | | | | | | |
|---|---|---|---|---|---|---|---|---|
| 7 | 26331514 | 26413949 | SNX10 | | SNX10 | SNX10 | | |
| 7 | 26443107 | 26535986 | LOC441204 | | | | | |
| 7 | 26572739 | 26578444 | KIAA0087 | | | | | |
| 7 | 26677489 | 26686889 | C7orf71 | | | | | |
| 7 | 26706680 | 26904362 | SKAP2 | | | | | |
| 7 | 27132613 | 27135625 | HOXA1 | | | | | |
| 7 | 27135712 | 27139877 | HOTAIRM1 | | | | | |
| 7 | 27139972 | 27142394 | HOXA2 | | | | | |
| 7 | 27145808 | 27166639 | HOXA3 | | | | | |
| 7 | 27161537 | 27168379 | HOXA | | | | | |
| 7 | 27168125 | 27170399 | HOXA4 | | | HOXA4 | | |
| 7 | 27179982 | 27195547 | HOXA | | | | | |
| 7 | 27180670 | 27183287 | HOXA5 | | | | | |
| 7 | 27185201 | 27187393 | HOXA6 | | | | | |
| 7 | 27186781 | 27195547 | HOXA | | | | | |
| 7 | 27193337 | 27196296 | HOXA7 | | | | | |
| 7 | 27202056 | 27205149 | HOXA9 | | | | | |
| 7 | 27202056 | 27219880 | HOXA10-HOXA9 | | | | | |
| 7 | 27208517 | 27211534 | HOXA10 | | | | | |
| 7 | 27209098 | 27209182 | MIR196B | | | | | |
| 7 | 27210209 | 27219880 | HOXA10 | | | | | |
| 7 | 27220775 | 27228912 | HOXA11 | | | | | |
| 7 | 27236498 | 27239725 | HOXA13 | | | | | |
| 7 | 27240039 | 27246878 | HOTTIP | | | | | |
| 7 | 27281047 | 27287438 | EVX1 | | | EVX1 | | |
| 7 | 27565058 | 27702620 | HIBADH | | | | | |
| 7 | 27655592 | 27667111 | TSL | | | | | |
| 7 | 27778991 | 27869386 | TAX1BP1 | | | | | |
| 7 | 27870192 | 28280996 | JAZF1 | | | | | |
| 7 | 28338939 | 28865511 | CREB5 | | | | | |
| 7 | 28992973 | 28998029 | TRIL | | | | | |
| 7 | 29019582 | 29052985 | LOC100506497 | | | | | |
| 7 | 29035246 | 29186153 | CPVL | | | | | |
| 7 | 29164648 | 29167788 | LOC101928168 | | | | | |
| 7 | 29186162 | 29553951 | CHN2 | | CHN2 | | | |
| 7 | 29238655 | 29248586 | LOC102724484 | | | | | |
| 7 | 29519327 | 29553951 | CHN2 | | CHN2 | | | |
| 7 | 29603426 | 29606911 | PRR15 | | | | | |
| 7 | 29685537 | 29724754 | LOC646762 | | | | | |
| 7 | 29720349 | 29720444 | MIR550A3 | | | | | |
| 7 | 29724387 | 29725437 | ZNRF2P2 | | | | | |
| 7 | 29724769 | 29727859 | DPY19L2P3 | | | | | |
| 7 | 29846169 | 29956682 | WIPF3 | | | | | |
| 7 | 29959718 | 30029905 | SCRN1 | | SCRN1 | | | |
| 7 | 30050198 | 30066417 | FKBP14 | | | | | |
| 7 | 30067976 | 30157961 | PLEKHA8 | | | | | |
| 7 | 30174551 | 30202381 | MTURN | | | | | |
| 7 | 30323922 | 30407308 | ZNRF2 | | | | | |
| 7 | 30329409 | 30329506 | MIR550A1 | | | | | |
| 7 | 30329409 | 30329506 | MIR550B1 | | | | | |
| 7 | 30409665 | 30412410 | DKFZP586I1420 | | | | | |
| 7 | 30430500 | 30443670 | LINC01176 | | | | | |
| 7 | 30464142 | 30518393 | NOD1 | | | | | |
| 7 | 30536236 | 30544457 | GGCT | | | | | |
| 7 | 30587972 | 30617395 | LOC401320 | | | | | |
| 7 | 30634350 | 30673648 | GARS | | | GARS | | |
| 7 | 30691558 | 30739719 | CRHR2 | | | | CRHR2 | |
| 7 | 30791750 | 30797218 | INMT | | | | | |
| 7 | 30791750 | 30932002 | INMT-FAM188B | | | | | |
| 7 | 30811032 | 30932002 | FAM188B | | | | | |
| 7 | 30951414 | 30965131 | AQP1 | | | | | |
| 7 | 31003635 | 31019146 | GHRHR | | | | | |
| 7 | 31092075 | 31151093 | ADCYAP1R1 | | ADCYAP1R1 | | | |
| 7 | 31377074 | 31380538 | NEUROD6 | | | | | |
| 7 | 31553684 | 31693303 | CCDC129 | | | | | |
| 7 | 31726630 | 31748069 | PPP1R17 | | PPP1R17 | | | |

| | | | | | | | | |
|---|---|---|---|---|---|---|---|---|
| 7 | 31790792 | 32111045 | PDE1C | | | | | |
| 7 | 32496509 | 32498106 | LOC100130673 | | | | | |
| 7 | 32524944 | 32534870 | LSM5 | | | | | |
| 7 | 32535037 | 32628353 | AVL9 | | | | | |
| 7 | 32620552 | 32758780 | DPY19L1P1 | | | | | |
| 7 | 32767561 | 32769595 | ZNRF2P1 | | | | | |
| 7 | 32772592 | 32772689 | MIR550A2 | | | | | |
| 7 | 32772592 | 32772689 | MIR550B2 | | | | | |
| 7 | 32797897 | 32802536 | LINC00997 | | | | | |
| 7 | 32865913 | 32878356 | DPY19L1P2 | | | | | |
| 7 | 32907777 | 32931468 | KBTBD2 | | | | | |
| 7 | 32956426 | 32982782 | RP9P | | | | | |
| 7 | 32997015 | 33046543 | FKBP9 | | | | | |
| 7 | 33053724 | 33102409 | NT5C3A | | | | | |
| 7 | 33134409 | 33149002 | RP9 | | | | | |
| 7 | 33169151 | 33645680 | BBS9 | | | | | |
| 7 | 33944522 | 34195484 | BMPER | | | | | |
| 7 | 34386123 | 34873943 | NPSR1 | | | | | |
| 7 | 34968492 | 35077653 | DPY19L1 | | | | | |
| 7 | 35120898 | 35225774 | DPY19L2P1 | | | | | |
| 7 | 35242041 | 35293711 | TBX20 | | | | | |
| 7 | 35353465 | 35416086 | LOC401324 | | | | | |
| 7 | 35672269 | 35734772 | HERPUD2 | | | | | |
| 7 | 35756046 | 35759322 | LOC100506725 | | | | | |
| 7 | 35794573 | 35946715 | SEPT7 | | | | | |
| 7 | 36134919 | 36140262 | LOC101928618 | | | | | |
| 7 | 36192835 | 36341152 | EEPD1 | | | | | |
| 7 | 36363758 | 36429734 | KIAA0895 | | | | | |
| 7 | 36429411 | 36493401 | ANLN | | ANLN | | | |
| 7 | 36552548 | 36764154 | AOAH | | | | | |
| 7 | 36637439 | 36639726 | AOAH-IT1 | | | | | |
| 7 | 36892510 | 37488929 | ELMO1 | | | | | |
| 7 | 36958961 | 36959037 | MIR1200 | | | | | |
| 7 | 37037400 | 37053235 | ELMO1 | | | | | |
| 7 | 37779995 | 37780913 | GPR141 | | | | | |
| 7 | 37888198 | 37940002 | NME8 | | | | | |
| 7 | 37945534 | 37956525 | SFRP4 | | | | | |
| 7 | 37960162 | 37991542 | EPDR1 | | | | | |
| 7 | 38217807 | 38270272 | STARD3NL | | STARD3NL | STARD3NL | | |
| 7 | 38299243 | 38313248 | TARP | | | | | |
| 7 | 38381177 | 38418238 | TRG | | | | | |
| 7 | 38423296 | 38671167 | AMPH | | | AMPH | | |
| 7 | 38724945 | 38726689 | FAM183BP | | | | | |
| 7 | 38763542 | 38948800 | VPS41 | | | | | |
| 7 | 39017608 | 39445945 | POU6F2 | | POU6F2 | POU6F2 | | |
| 7 | 39605974 | 39651688 | YAE1D1 | | | | | |
| 7 | 39663151 | 39747723 | RALA | | | | | |
| 7 | 39773166 | 39834222 | LINC00265 | | | | | |
| 7 | 39989958 | 40136733 | CDK13 | | | | | |
| 7 | 40172341 | 40174251 | MPLKIP | | | | | |
| 7 | 40174574 | 40900366 | SUGCT | | | | | |
| 7 | 41004276 | 41019537 | LINC01450 | | | | | |
| 7 | 41141201 | 41173099 | LINC01449 | | | | | |
| 7 | 41728600 | 41752792 | INHBA | | | | | |
| 7 | 42000547 | 42276618 | GLI3 | | | GLI3 | | |
| 7 | 42701325 | 42746046 | LINC01448 | | | | | |
| 7 | 42948871 | 42951689 | C7orf25 | | | | | |
| 7 | 42956461 | 42971805 | PSMA2 | | | | | |
| 7 | 42971938 | 42977453 | MRPL32 | | | | | |
| 7 | 43152197 | 43602938 | HECW1 | | | | | |
| 7 | 43190493 | 43190593 | MIR3943 | | | | | |
| 7 | 43548326 | 43562141 | LOC100506895 | | | | | |
| 7 | 43622691 | 43666978 | STK17A | | | | | |
| 7 | 43670750 | 43769140 | COA1 | | | | | |
| 7 | 43798271 | 43846941 | BLVRA | | | | | |
| 7 | 43906156 | 43909145 | MRPS24 | | | | | |

| | | | | | | | | |
|---|---|---|---|---|---|---|---|---|
| 7 | 43906156 | 43946231 | URGCP-MRPS24 | | | | | |
| 7 | 43915492 | 43966010 | URGCP | | | | | |
| 7 | 43966034 | 43995735 | UBE2D4 | | | | | |
| 7 | 43980493 | 44058793 | POLR2J4 | | | | | |
| 7 | 44040488 | 44049723 | SPDYE1 | | | | | |
| 7 | 44068485 | 44080222 | RASA4CP | | | | | |
| 7 | 44078647 | 44083895 | LINC00957 | | | | | |
| 7 | 44084238 | 44101316 | DBNL | | | | | |
| 7 | 44091364 | 44091428 | MIR6837 | | | | | |
| 7 | 44102325 | 44105186 | PGAM2 | | | | | |
| 7 | 44111660 | 44122139 | POLM | | | | | |
| 7 | 44112976 | 44113032 | MIR6838 | | | | | |
| 7 | 44143959 | 44154164 | AEBP1 | | | | | |
| 7 | 44150447 | 44150511 | MIR4649 | | | | | |
| 7 | 44154278 | 44163169 | POLD2 | | | | | |
| 7 | 44178462 | 44180916 | MYL7 | | | | | |
| 7 | 44183869 | 44229022 | GCK | | GCK | GCK | | |
| 7 | 44240577 | 44253893 | YKT6 | | | | | |
| 7 | 44256748 | 44365230 | CAMK2B | | CAMK2B | CAMK2B | | |
| 7 | 44421964 | 44530385 | NUDCD3 | | | | | |
| 7 | 44552134 | 44580914 | NPC1L1 | | | | | |
| 7 | 44605015 | 44614137 | DDX56 | | | | | |
| 7 | 44617493 | 44621894 | TMED4 | | | | | |
| 7 | 44646120 | 44748669 | OGDH | | | OGDH | | |
| 7 | 44788164 | 44809479 | ZMIZ2 | | | | | |
| 7 | 44836234 | 44842722 | PPIA | | | | | |
| 7 | 44866487 | 44887725 | H2AFV | | | | | |
| 7 | 44915891 | 44924984 | PURB | | | | | |
| 7 | 44921346 | 44921399 | MIR4657 | | | | | |
| 7 | 45002259 | 45018704 | MYO1G | | | | | |
| 7 | 45022626 | 45026259 | SNHG15 | | | | | |
| 7 | 45024976 | 45025109 | SNORA9 | | | | | |
| 7 | 45039344 | 45116069 | CCM2 | | CCM2 | | | |
| 7 | 45120035 | 45128493 | NACAD | | | | | |
| 7 | 45139698 | 45151346 | TBRG4 | | | | | |
| 7 | 45143947 | 45144081 | SNORA5A | | | | | |
| 7 | 45144504 | 45144641 | SNORA5C | | | | | |
| 7 | 45145566 | 45145698 | SNORA5B | | | | | |
| 7 | 45197366 | 45223850 | RAMP3 | | | | | |
| 7 | 45613738 | 45762714 | ADCY1 | | ADCY1 | ADCY1 | | |
| 7 | 45763385 | 45808617 | SEPT7P2 | | | | | |
| 7 | 45927958 | 45933267 | IGFBP1 | | | | | |
| 7 | 45951843 | 45960871 | IGFBP3 | | | | | |
| 7 | 46727476 | 46736720 | LOC730338 | | | | | |
| 7 | 47314751 | 47621742 | TNS3 | | | | | |
| 7 | 47661536 | 47669491 | LINC01447 | | | | | |
| 7 | 47694841 | 47701246 | C7orf65 | | | | | |
| 7 | 47801073 | 47806370 | LINC00525 | | | | | |
| 7 | 47814249 | 47988071 | PKD1L1 | | | | | |
| 7 | 47834888 | 47859444 | C7orf69 | | | | | |
| 7 | 48002884 | 48019222 | HUS1 | | | | | |
| 7 | 48026745 | 48068716 | SUN3 | | | | | |
| 7 | 48075107 | 48100894 | C7orf57 | | | | | |
| 7 | 48128199 | 48148330 | UPP1 | | | | | |
| 7 | 48211056 | 48687091 | ABCA13 | | | | | |
| 7 | 48964156 | 48967049 | CDC14C | | | | | |
| 7 | 49813256 | 49952138 | VWC2 | | | | | |
| 7 | 49977023 | 50132860 | ZPBP | | | | | |
| 7 | 50135681 | 50198852 | C7orf72 | | | | | |
| 7 | 50343678 | 50472798 | IKZF1 | | | | | |
| 7 | 50511826 | 50518088 | FIGNL1 | | | | | |
| 7 | 50526133 | 50611161 | DDC | | | DDC | | |
| 7 | 50657759 | 50861159 | GRB10 | | GRB10 | GRB10 | | |
| 7 | 51083908 | 51384515 | COBL | | | | | |
| 7 | 53103348 | 53104618 | POM121L12 | | | | | |
| 7 | 53723201 | 53879624 | LINC01446 | | | | | |

| | | | | | | | | |
|---|---|---|---|---|---|---|---|---|
| 7 | 54268916 | 54270114 | HPVC1 | | | | | |
| 7 | 54398389 | 54417538 | LINC01445 | | | | | |
| 7 | 54610017 | 54638773 | VSTM2A | | | | | |
| 7 | 54624662 | 54639419 | VSTM2A-OT1 | | | | | |
| 7 | 54819939 | 54826939 | SEC61G | | | | | |
| 7 | 54827005 | 54872656 | LOC100996654 | | | | | |
| 7 | 55086724 | 55256642 | EGFR | | | EGFR | | |
| 7 | 55306128 | 55323328 | ELDR | | | | | |
| 7 | 55433140 | 55501435 | LANCL2 | | | | | |
| 7 | 55538300 | 55640200 | VOPP1 | | | | | |
| 7 | 55748766 | 55772260 | FKBP9P1 | | | | | |
| 7 | 55861236 | 55930482 | SEPT14 | | | | | |
| 7 | 55954969 | 56009918 | ZNF713 | | | | | |
| 7 | 56019610 | 56023033 | MRPS17 | | | | | |
| 7 | 56032269 | 56067875 | GBAS | | | | | |
| 7 | 56078743 | 56119268 | PSPH | | PSPH | | | |
| 7 | 56119377 | 56131682 | CCT6A | | | | | |
| 7 | 56128162 | 56128295 | SNORA15 | | | | | |
| 7 | 56131916 | 56148365 | SUMF2 | | | | | |
| 7 | 56147975 | 56160689 | PHKG1 | | | PHKG1 | | |
| 7 | 56169265 | 56174187 | CHCHD2 | | | | | |
| 7 | 56182373 | 56184090 | NUPR2 | | | | | |
| 7 | 56491396 | 56516068 | LOC650226 | | | | | |
| 7 | 56549797 | 56551012 | LOC100240728 | | | | | |
| 7 | 56563915 | 56564977 | DKFZp434L192 | | | | | |
| 7 | 56601922 | 56605823 | LOC101928401 | | | | | |
| 7 | 56876816 | 56880429 | LOC401357 | | | | | |
| 7 | 56943077 | 56949839 | LOC100130849 | | | | | |
| 7 | 57023491 | 57023571 | MIR4283 | | | | | |
| 7 | 57187325 | 57207571 | ZNF479 | | | | | |
| 7 | 57233376 | 57247863 | GUSBP10 | | | | | |
| 7 | 57472730 | 57472796 | MIR3147 | | | | | |
| 7 | 57509882 | 57533265 | ZNF716 | | | | | |
| 7 | 62751669 | 62764434 | ZNF733P | | | | | |
| 7 | 62809447 | 62812152 | LOC100287704 | | | | | |
| 7 | 62809447 | 62859419 | LOC100287834 | | | | | |
| 7 | 63081467 | 63081547 | MIR4283 | | | | | |
| 7 | 63484795 | 63490480 | LINC01005 | | | | | |
| 7 | 63505820 | 63538927 | ZNF727 | | | | | |
| 7 | 63667580 | 63680668 | ZNF735 | | | | | |
| 7 | 63688851 | 63727309 | ZNF679 | | | | | |
| 7 | 63774250 | 63817012 | ZNF736 | | | | | |
| 7 | 63894070 | 63895895 | YWHAEP1 | | | | | |
| 7 | 63980254 | 64023505 | ZNF680 | | | | | |
| 7 | 64035072 | 64078967 | LOC100128885 | | | | | |
| 7 | 64042987 | 64044129 | LOC641746 | | | | | |
| 7 | 64126460 | 64171960 | ZNF107 | | | | | |
| 7 | 64139441 | 64139554 | MIR6839 | | | | | |
| 7 | 64254765 | 64314178 | ZNF138 | | | | | |
| 7 | 64363619 | 64391955 | ZNF273 | | | | | |
| 7 | 64434829 | 64451414 | ZNF117 | | | | | |
| 7 | 64450732 | 64467124 | ERV3-1 | | | | | |
| 7 | 64498731 | 64535091 | CCT6P3 | | | | | |
| 7 | 64838711 | 64866048 | ZNF92 | | | | | |
| 7 | 65111923 | 65235797 | LOC441242 | | | | | |
| 7 | 65112766 | 65183667 | INTS4P2 | | | | | |
| 7 | 65179585 | 65215911 | LOC441242 | | | | | |
| 7 | 65216091 | 65228662 | CCT6P1 | | | | | |
| 7 | 65220512 | 65220646 | SNORA22 | | | | | |
| 7 | 65338256 | 65424550 | VKORC1L1 | | | | | |
| 7 | 65425671 | 65447301 | GUSB | GUSB | | GUSB | | |
| 7 | 65540775 | 65558329 | ASL | | | ASL | | |
| 7 | 65579804 | 65619553 | CRCP | | | | | |
| 7 | 65670258 | 65825438 | TPST1 | | | | | |
| 7 | 65841030 | 65865395 | LINC00174 | | | | | |
| 7 | 65958618 | 65960461 | GS1-124K5.4 | | | | | |

| | | | | | | | | |
|---|---|---|---|---|---|---|---|---|
| 7 | 65995291 | 66057394 | GS1-124K5.11 | | | | | |
| 7 | 66093867 | 66108216 | KCTD7 | | KCTD7 | | | |
| 7 | 66119505 | 66134589 | LOC100996437 | | | | | |
| 7 | 66147077 | 66276448 | RABGEF1 | | | | | |
| 7 | 66274979 | 66309813 | GTF2IRD1P1 | | | | | |
| 7 | 66386202 | 66423538 | TMEM248 | | | | | |
| 7 | 66452689 | 66460588 | SBDS | SBDS | | | | |
| 7 | 66461791 | 66704507 | TYW1 | | | | | |
| 7 | 66579308 | 66579384 | MIR4650 | | | | | |
| 7 | 66741117 | 66767429 | PMS2P4 | | | | | |
| 7 | 66767624 | 66786513 | STAG3L4 | | | | | |
| 7 | 66800962 | 66805012 | LINC01372 | | | | | |
| 7 | 67485239 | 67497677 | LOC102723427 | | | | | |
| 7 | 69061123 | 69062481 | LOC100507468 | | | | | |
| 7 | 69063904 | 70258054 | AUTS2 | | | AUTS2 | | |
| 7 | 70597522 | 71178586 | WBSCR17 | | | | | |
| 7 | 70772657 | 70772754 | MIR3914 | | | | | |
| 7 | 71244475 | 71877360 | CALN1 | | | | | |
| 7 | 72039491 | 72298813 | TYW1B | | | | | |
| 7 | 72162873 | 72162949 | MIR4650 | | | | | |
| 7 | 72299951 | 72307978 | SBDSP1 | | | | | |
| 7 | 72333320 | 72339656 | SPDYE7P | | | | | |
| 7 | 72349905 | 72418843 | POM121 | | | | | |
| 7 | 72418831 | 72425302 | NSUN5P2 | | | | | |
| 7 | 72430015 | 72439997 | TRIM74 | | | | | |
| 7 | 72440164 | 72443674 | LOC541473 | | | | | |
| 7 | 72440212 | 72443674 | LOC100101148 | | | | | |
| 7 | 72467817 | 72476448 | STAG3L1 | | | | | |
| 7 | 72467817 | 72476466 | STAG3L3 | | | | | |
| 7 | 72476602 | 72486904 | PMS2P7 | | | | | |
| 7 | 72476618 | 72520245 | PMS2P5 | | | | | |
| 7 | 72476643 | 72491707 | PMS2P2 | | | | | |
| 7 | 72490259 | 72500309 | SPDYE8P | | | | | |
| 7 | 72569011 | 72621336 | GTF2IP4 | | | | | |
| 7 | 72569016 | 72621336 | GTF2IP1 | | | | | |
| 7 | 72634673 | 72649979 | NCF1B | | | | | |
| 7 | 72716512 | 72722864 | NSUN5 | | | | | |
| 7 | 72726531 | 72742085 | TRIM50 | | | | | |
| 7 | 72742154 | 72772646 | FKBP6 | | | | | |
| 7 | 72848108 | 72850450 | FZD9 | FZD9 | | | | |
| 7 | 72854727 | 72936615 | BAZ1B | | | | | |
| 7 | 72950682 | 72972065 | BCL7B | | | | | |
| 7 | 72983276 | 72993013 | TBL2 | | | | | |
| 7 | 73007523 | 73038873 | MLXIPL | | | | | |
| 7 | 73082173 | 73086440 | VPS37D | | | | | |
| 7 | 73095247 | 73097781 | DNAJC30 | | | | | |
| 7 | 73097897 | 73112551 | WBSCR22 | | | | | |
| 7 | 73113534 | 73134017 | STX1A | | STX1A | STX1A | STX1A | |
| 7 | 73125646 | 73125727 | MIR4284 | | | | | |
| 7 | 73149398 | 73153190 | ABHD11 | | | | | |
| 7 | 73183326 | 73184600 | CLDN3 | | | | | |
| 7 | 73245192 | 73247023 | CLDN4 | | | | | |
| 7 | 73248920 | 73256855 | WBSCR27 | | | | | |
| 7 | 73275488 | 73280223 | WBSCR28 | | | | | |
| 7 | 73442118 | 73484236 | ELN | | | ELN | | |
| 7 | 73498106 | 73536855 | LIMK1 | LIMK1 | LIMK1 | LIMK1 | | |
| 7 | 73588705 | 73611429 | EIF4H | | | | | |
| 7 | 73605527 | 73605624 | MIR590 | | | | | |
| 7 | 73624086 | 73644164 | LAT2 | | | | | |
| 7 | 73645831 | 73668788 | RFC2 | | | | | |
| 7 | 73703804 | 73820273 | CLIP2 | | CLIP2 | | | |
| 7 | 73868119 | 74016920 | GTF2IRD1 | | | | | |
| 7 | 74071990 | 74175022 | GTF2I | | | GTF2I | | |
| 7 | 74103270 | 74143256 | LOC101926943 | | | | | |
| 7 | 74188308 | 74203720 | NCF1 | | | NCF1 | | |
| 7 | 74210482 | 74267872 | GTF2IRD2 | | | | | |

| | | | | | | | | |
|---|---|---|---|---|---|---|---|---|
| 7 | 74298091 | 74306731 | STAG3L2 | | | | | |
| 7 | 74306886 | 74336166 | PMS2P5 | | | | | |
| 7 | 74441223 | 74489717 | WBSCR16 | | | | | |
| 7 | 74508346 | 74565623 | GTF2IRD2 | | | | | |
| 7 | 74508346 | 74565623 | GTF2IRD2B | | | | | |
| 7 | 74572383 | 74587802 | NCF1C | | | | | |
| 7 | 74601103 | 74653459 | GTF2IP4 | | | | | |
| 7 | 74601103 | 74867641 | GTF2IP1 | | | | | |
| 7 | 74702376 | 74988276 | PMS2P5 | | | | | |
| 7 | 74800771 | 74867509 | GATSL2 | | | | | |
| 7 | 74936532 | 74974606 | SPDYE8P | | | | | |
| 7 | 74977963 | 74988292 | PMS2P7 | | | | | |
| 7 | 74988446 | 74997085 | STAG3L1 | | | | | |
| 7 | 75024902 | 75034896 | TRIM73 | | | | | |
| 7 | 75039604 | 75046071 | NSUN5P1 | | | | | |
| 7 | 75046059 | 75115565 | POM121C | | | | | |
| 7 | 75122946 | 75133628 | SPDYE5 | | | | | |
| 7 | 75137068 | 75157453 | PMS2P3 | | | | | |
| 7 | 75162618 | 75368290 | HIP1 | | HIP1 | HIP1 | | |
| 7 | 75398841 | 75419064 | CCL26 | | | | | |
| 7 | 75441113 | 75443033 | CCL24 | | | | | |
| 7 | 75508316 | 75518244 | RHBDD2 | | | | | |
| 7 | 75544419 | 75616173 | POR | | | | | |
| 7 | 75544514 | 75544587 | MIR4651 | | | | | |
| 7 | 75573100 | 75573234 | SNORA14A | | | | | |
| 7 | 75616154 | 75623992 | TMEM120A | | | | | |
| 7 | 75625654 | 75677321 | STYXL1 | | | | | |
| 7 | 75677336 | 75696827 | MDH2 | | | | | |
| 7 | 75721380 | 75738080 | GTF2IP7 | | | | | |
| 7 | 75831210 | 75916609 | SRRM3 | | | | | |
| 7 | 75931874 | 75933614 | HSPB1 | | | HSPB1 | | |
| 7 | 75956107 | 75988342 | YWHAG | | | YWHAG | | |
| 7 | 76018645 | 76039012 | SSC4D | | | | | |
| 7 | 76026840 | 76071388 | ZP3 | | | | | |
| 7 | 76090971 | 76135312 | DTX2 | | | | | |
| 7 | 76099336 | 76104317 | FDPSP2 | | | | | |
| 7 | 76109826 | 76135312 | DTX2 | | | | | |
| 7 | 76139739 | 76157199 | UPK3B | | | | | |
| 7 | 76178657 | 76257299 | LOC100133091 | | | | | |
| 7 | 76239302 | 76256620 | POMZP3 | | | | | |
| 7 | 76610138 | 76653076 | DTX2P1-UPK3BP1-PMS2P11 | | | | | |
| 7 | 76668796 | 76682355 | PMS2P9 | | | | | |
| 7 | 76751933 | 76924521 | CCDC146 | | | | | |
| 7 | 76822687 | 76829150 | FGL2 | | | | | |
| 7 | 76940067 | 77045717 | GSAP | | | | | |
| 7 | 77044514 | 77054760 | LOC101927243 | | | | | |
| 7 | 77166772 | 77269388 | PTPN12 | | | | | |
| 7 | 77286976 | 77326662 | APTR | | | | | |
| 7 | 77325742 | 77409120 | RSBN1L | | | | | |
| 7 | 77423044 | 77427747 | TMEM60 | | | | | |
| 7 | 77428108 | 77586821 | PHTF2 | | | | | |
| 7 | 77646373 | 79082890 | MAGI2 | | MAGI2 | | MAGI2 | |
| 7 | 77976558 | 77988770 | RPL13AP17 | | | | | |
| 7 | 78638303 | 79100524 | MAGI2 | | MAGI2 | | MAGI2 | |
| 7 | 79400571 | 79400606 | MIR548M | | | | | |
| 7 | 79764139 | 79848725 | GNAI1 | | | | | |
| 7 | 80000832 | 80003755 | LOC101927269 | | | | | |
| 7 | 80087950 | 80141325 | GNAT3 | | | | | |
| 7 | 80231503 | 80303734 | CD36 | | | | | |
| 7 | 80371853 | 80548667 | SEMA3C | | | | | |
| 7 | 81205701 | 81320722 | LOC100128317 | | | | | |
| 7 | 81328323 | 81399514 | HGF | | | | | |
| 7 | 81575759 | 82073122 | CACNA2D1 | | CACNA2D1 | | | |
| 7 | 81638492 | 81659271 | LOC101927356 | | | | | |
| 7 | 81679651 | 82073122 | CACNA2D1 | | CACNA2D1 | | | |
| 7 | 82383320 | 82792197 | PCLO | | PCLO | | PCLO | PCLO |

| | | | | | | | | |
|---|---|---|---|---|---|---|---|---|
| 7 | 82993221 | 83278479 | SEMA3E | | SEMA3E | | | |
| 7 | 83587658 | 83824217 | SEMA3A | | | | | |
| 7 | 84161791 | 84213634 | LOC101927378 | | | | | |
| 7 | 84624871 | 84751247 | SEMA3D | | | | | |
| 7 | 85050437 | 85118654 | LINC00972 | | | | | |
| 7 | 86273229 | 86494192 | GRM3 | | | GRM3 | | |
| 7 | 86506221 | 86689014 | KIAA1324L | | | | | |
| 7 | 86781676 | 86825648 | DMTF1 | | | | | |
| 7 | 86825477 | 86849031 | TMEM243 | | | | | |
| 7 | 86954663 | 86974808 | TP53TG1 | | | | | |
| 7 | 86974950 | 87029112 | CROT | | | CROT | | |
| 7 | 87031360 | 87105019 | ABCB4 | | | | | |
| 7 | 87133178 | 87342639 | ABCB1 | | ABCB1 | ABCB1 | | |
| 7 | 87257728 | 87461613 | RUNDC3B | | | | | |
| 7 | 87463813 | 87505692 | SLC25A40 | | | | | |
| 7 | 87505543 | 87538856 | DBF4 | | | | | |
| 7 | 87563565 | 87832204 | ADAM22 | | | | | |
| 7 | 87834431 | 87856308 | SRI | | | | | |
| 7 | 87845974 | 87848539 | LOC102723885 | | | | | |
| 7 | 87905743 | 87936228 | STEAP4 | | | | | |
| 7 | 88388681 | 88966371 | ZNF804B | | | | | |
| 7 | 88423419 | 88425031 | C7orf62 | | | | | |
| 7 | 89511666 | 89840949 | STEAP2 | | | | | |
| 7 | 89748713 | 89754914 | DPY19L2P4 | | | | | |
| 7 | 89783688 | 89794141 | STEAP1 | | | | | |
| 7 | 89840999 | 89866992 | STEAP2 | | | | | |
| 7 | 89874487 | 89940377 | CFAP69 | | | | | |
| 7 | 89964536 | 89973408 | LOC101927446 | | | | | |
| 7 | 89975978 | 90020769 | GTPBP10 | | | | | |
| 7 | 90025605 | 90027550 | LOC101409256 | | | | | |
| 7 | 90032647 | 90045268 | CLDN12 | | | | | |
| 7 | 90225675 | 90839905 | CDK14 | | | | | |
| 7 | 90893782 | 90898132 | FZD1 | | | | | |
| 7 | 91502190 | 91510034 | MTERF1 | | | | | |
| 7 | 91570188 | 91739987 | AKAP9 | | | AKAP9 | | AKAP9 |
| 7 | 91741462 | 91810039 | CYP51A1 | | CYP51A1 | | | |
| 7 | 91774197 | 91794590 | LRRD1 | | | | | |
| 7 | 91828282 | 91875414 | KRIT1 | | | | | |
| 7 | 91875547 | 92030698 | ANKIB1 | | | | | |
| 7 | 92041863 | 92066628 | LOC105375396 | | | | | |
| 7 | 92076761 | 92089381 | GATAD1 | | | | | |
| 7 | 92116336 | 92157845 | PEX1 | | | PEX1 | | |
| 7 | 92158086 | 92169795 | RBM48 | | | | | |
| 7 | 92190071 | 92219708 | FAM133B | | | | | |
| 7 | 92190367 | 92219696 | FAM133DP | | | | | |
| 7 | 92234234 | 92465941 | CDK6 | | | | | |
| 7 | 92465796 | 92546501 | LOC101927497 | | | | | |
| 7 | 92728825 | 92747336 | SAMD9 | | | | | |
| 7 | 92759366 | 92777701 | SAMD9L | | | | | |
| 7 | 92817898 | 92855832 | HEPACAM2 | | | | | |
| 7 | 92861652 | 92990435 | VPS50 | | | | | |
| 7 | 93053798 | 93204042 | CALCR | | | | CALCR | |
| 7 | 93112071 | 93112167 | MIR653 | | | | | |
| 7 | 93113247 | 93113331 | MIR489 | | | | | |
| 7 | 93346239 | 93346317 | MIR4652 | | | | | |
| 7 | 93514708 | 93520303 | TFPI2 | | | | | |
| 7 | 93520205 | 93522897 | LOC105375401 | | | | | |
| 7 | 93535819 | 93540485 | GNGT1 | | | | | |
| 7 | 93551015 | 93555826 | GNG11 | | | | | |
| 7 | 93592081 | 93633694 | BET1 | | | | | |
| 7 | 94023872 | 94060544 | COL1A2 | | | COL1A2 | | |
| 7 | 94139169 | 94186328 | CASD1 | | | | | |
| 7 | 94214535 | 94285521 | SGCE | | | | | |
| 7 | 94285636 | 94299006 | PEG10 | | PEG10 | | | |
| 7 | 94536948 | 94925727 | PPP1R9A | | PPP1R9A | | | |
| 7 | 94927668 | 94953884 | PON1 | | | | | |

| | | | | | | | | |
|---|---|---|---|---|---|---|---|---|
| 7 | 94989183 | 95025687 | PON3 | | | | | |
| 7 | 95034173 | 95064384 | PON2 | | PON2 | | | |
| 7 | 95115212 | 95169543 | ASB4 | | | | | |
| 7 | 95212808 | 95225925 | PDK4 | | | PDK4 | | |
| 7 | 95401817 | 95739634 | DYNC1I1 | | | | | |
| 7 | 95749531 | 95951459 | SLC25A13 | | | SLC25A13 | | |
| 7 | 95848973 | 95849068 | MIR591 | | | | | |
| 7 | 96110937 | 96132835 | C7orf76 | | | | | |
| 7 | 96250968 | 96293650 | LOC100506136 | | | | | |
| 7 | 96318078 | 96339203 | SHFM1 | | | SHFM1 | | |
| 7 | 96597826 | 96640352 | DLX6 | | | | | |
| 7 | 96649701 | 96654143 | DLX5 | | | | | |
| 7 | 96745904 | 96811075 | SDHAF3 | | | | | |
| 7 | 97361270 | 97369784 | TAC1 | | | TAC1 | | |
| 7 | 97481428 | 97501854 | ASNS | | ASNS | | | |
| 7 | 97592969 | 97593038 | MIR5692A1 | | | | | |
| 7 | 97592974 | 97593033 | MIR5692A2 | | | | | |
| 7 | 97595907 | 97601638 | MGC72080 | | | | | |
| 7 | 97614012 | 97619416 | OCM2 | | | | | |
| 7 | 97736196 | 97838944 | LMTK2 | | | | | |
| 7 | 97841565 | 97842271 | BHLHA15 | | | | | |
| 7 | 97844754 | 97881563 | TECPR1 | | | | | |
| 7 | 97910978 | 97922275 | BRI3 | | | | | |
| 7 | 97920961 | 98030427 | BAIAP2L1 | | | | | |
| 7 | 98246596 | 98259181 | NPTX2 | | NPTX2 | NPTX2 | | |
| 7 | 98444110 | 98467673 | TMEM130 | | | | | |
| 7 | 98476112 | 98610866 | TRRAP | | | | | |
| 7 | 98479272 | 98479352 | MIR3609 | | | | | |
| 7 | 98479319 | 98479513 | SCARNA28 | | | | | |
| 7 | 98610787 | 98634102 | LOC101927550 | | | | | |
| 7 | 98625057 | 98741743 | SMURF1 | | | | | |
| 7 | 98771196 | 98805089 | KPNA7 | | | | | |
| 7 | 98870923 | 98895594 | MYH16 | | | | | |
| 7 | 98923495 | 98963885 | ARPC1A | | | | | |
| 7 | 98972297 | 98992404 | ARPC1B | | | | | |
| 7 | 98992297 | 99006305 | PDAP1 | | | | | |
| 7 | 99006600 | 99017239 | BUD31 | | | | | |
| 7 | 99014361 | 99036462 | PTCD1 | | | | | |
| 7 | 99014361 | 99063824 | ATP5J2-PTCD1 | | | | | |
| 7 | 99036562 | 99055000 | CPSF4 | | | | | |
| 7 | 99055783 | 99063824 | ATP5J2 | | | | | |
| 7 | 99070514 | 99085217 | ZNF789 | | | | | |
| 7 | 99090853 | 99097877 | ZNF394 | | | | | |
| 7 | 99102266 | 99131445 | ZKSCAN5 | | | | | |
| 7 | 99143922 | 99149757 | FAM200A | | | | | |
| 7 | 99156044 | 99174076 | ZNF655 | | | | | |
| 7 | 99195901 | 99208456 | GS1-259H13.2 | | | | | |
| 7 | 99214570 | 99230030 | ZSCAN25 | | | | | |
| 7 | 99245811 | 99277649 | CYP3A5 | | | | | |
| 7 | 99282301 | 99332823 | CYP3A7-CYP3A51P | | | | | |
| 7 | 99302659 | 99332823 | CYP3A7 | | | | | |
| 7 | 99354582 | 99381811 | CYP3A4 | | | | | |
| 7 | 99425635 | 99463727 | CYP3A43 | | | | | |
| 7 | 99473684 | 99474656 | OR2AE1 | | | | | |
| 7 | 99488029 | 99517223 | TRIM4 | | | | | |
| 7 | 99520891 | 99527243 | GJC3 | | | | | |
| 7 | 99564349 | 99573735 | AZGP1 | | | | | |
| 7 | 99578384 | 99581860 | AZGP1P1 | | | | | |
| 7 | 99613194 | 99639312 | ZKSCAN1 | | | | | |
| 7 | 99647416 | 99662663 | ZSCAN21 | | | | | |
| 7 | 99661469 | 99680171 | ZNF3 | | | | | |
| 7 | 99686582 | 99689822 | COPS6 | | | COPS6 | | |
| 7 | 99690350 | 99699563 | MCM7 | | | | | |
| 7 | 99691182 | 99691266 | MIR25 | | | | | |
| 7 | 99691390 | 99691470 | MIR93 | | | | | |
| 7 | 99691615 | 99691697 | MIR106B | | | | | |

| | | | | | | | | |
|---|---|---|---|---|---|---|---|---|
| 7 | 99699129 | 99704803 | AP4M1 | | | | | |
| 7 | 99704692 | 99717481 | TAF6 | | | | | |
| 7 | 99717264 | 99723128 | CNPY4 | | | | | |
| 7 | 99724316 | 99726121 | MBLAC1 | | | | | |
| 7 | 99746521 | 99751835 | LAMTOR4 | | | | | |
| 7 | 99752042 | 99756344 | C7orf43 | | | | | |
| 7 | 99754227 | 99754292 | MIR4658 | | | | | |
| 7 | 99756864 | 99766373 | GAL3ST4 | | | | | |
| 7 | 99767228 | 99775049 | GPC2 | | | | | |
| 7 | 99775346 | 99812010 | STAG3 | | | STAG3 | | |
| 7 | 99798277 | 99869855 | GATS | | | | | |
| 7 | 99816870 | 99819111 | PVRIG | | | | | |
| 7 | 99905324 | 99919819 | SPDYE3 | | | | | |
| 7 | 99918262 | 99933930 | PMS2P1 | | | | | |
| 7 | 99933687 | 99965454 | STAG3L5P-PVRIG2P-PILRB | | | | | |
| 7 | 99933701 | 99938951 | STAG3L5P | | | | | |
| 7 | 99949940 | 99951559 | PVRIG2P | | | | | |
| 7 | 99954273 | 99954344 | MIR6840 | | | | | |
| 7 | 99955625 | 99965454 | PILRB | | | | | |
| 7 | 99971067 | 99997722 | PILRA | | | | | |
| 7 | 99998494 | 100026431 | ZCWPW1 | | | | | |
| 7 | 100026412 | 100031749 | MEPCE | | | | | |
| 7 | 100032911 | 100034094 | PPP1R35 | | | | | |
| 7 | 100054237 | 100061894 | C7orf61 | | | | | |
| 7 | 100064141 | 100076902 | TSC22D4 | | | | | |
| 7 | 100081549 | 100092424 | NYAP1 | | | | | |
| 7 | 100136833 | 100165843 | AGFG2 | | | | | |
| 7 | 100169852 | 100171270 | SAP25 | | | | | |
| 7 | 100171633 | 100183811 | LRCH4 | | | | | |
| 7 | 100171657 | 100172356 | ZASP | | | | | |
| 7 | 100183955 | 100198740 | FBXO24 | | | | | |
| 7 | 100187023 | 100201661 | PCOLCE | | | | | |
| 7 | 100187195 | 100198740 | FBXO24 | | | | | |
| 7 | 100199881 | 100205798 | PCOLCE | | | | | |
| 7 | 100209724 | 100213000 | MOSPD3 | | | | | |
| 7 | 100218038 | 100239173 | TFR2 | | TFR2 | | | |
| 7 | 100240725 | 100254084 | ACTL6B | | | | | |
| 7 | 100254183 | 100264049 | LOC105375429 | | | | | |
| 7 | 100271362 | 100276792 | GNB2 | | | | | |
| 7 | 100277129 | 100286870 | GIGYF1 | | | | | |
| 7 | 100303675 | 100305123 | POP7 | | | | | |
| 7 | 100318422 | 100321323 | EPO | | | | | |
| 7 | 100331248 | 100395419 | ZAN | | | | | |
| 7 | 100400186 | 100425143 | EPHB4 | | | | | |
| 7 | 100450340 | 100464634 | SLC12A9 | | | | | |
| 7 | 100464949 | 100471076 | TRIP6 | | | | | |
| 7 | 100465657 | 100465729 | MIR6875 | | | | | |
| 7 | 100472700 | 100486285 | SRRT | | | | | |
| 7 | 100486343 | 100487339 | UFSP1 | | | | | |
| 7 | 100487614 | 100493592 | ACHE | | ACHE | ACHE | ACHE | ACHE |
| 7 | 100547051 | 100611619 | MUC3A | | | | | |
| 7 | 100612903 | 100662230 | MUC12 | | | | | |
| 7 | 100657600 | 100660889 | LOC102724094 | | | | | |
| 7 | 100663363 | 100702140 | MUC17 | | | | | |
| 7 | 100728719 | 100735019 | TRIM56 | | | | | |
| 7 | 100770369 | 100782547 | SERPINE1 | | | | | |
| 7 | 100797685 | 100804557 | AP1S1 | | AP1S1 | AP1S1 | | |
| 7 | 100802753 | 100802836 | MIR4653 | | | | | |
| 7 | 100805789 | 100808852 | VGF | | | | | |
| 7 | 100813773 | 100823557 | NAT16 | | | | | |
| 7 | 100839011 | 100844302 | MOGAT3 | | | | | |
| 7 | 100849257 | 100861011 | PLOD3 | | | | | |
| 7 | 100860984 | 100867471 | ZNHIT1 | | | | | |
| 7 | 100875372 | 100882101 | CLDN15 | | | | | |
| 7 | 100882892 | 100888371 | FIS1 | | | | | |
| 7 | 100951620 | 100964425 | LOC101927746 | | | | | |

| | | | | | | | | | |
|---|---|---|---|---|---|---|---|---|---|
| 7 | 100956647 | 100965104 | IFT22 | | | | | | |
| 7 | 101006100 | 101202304 | COL26A1 | | COL26A1 | | | | |
| 7 | 101206034 | 101212286 | LINC01007 | | | | | | |
| 7 | 101256604 | 101272576 | MYL10 | | | | | | |
| 7 | 101459183 | 101901513 | CUX1 | | | CUX1 | | | |
| 7 | 101928352 | 101962178 | SH2B2 | | | | | | |
| 7 | 101936368 | 101936453 | MIR4285 | | | | | | |
| 7 | 101986191 | 101996889 | SPDYE6 | | | | | | |
| 7 | 102004307 | 102021080 | LOC100289561 | | | | | | |
| 7 | 102004307 | 102067129 | LOC100630923 | | | | | | |
| 7 | 102036803 | 102067129 | PRKRIP1 | | | | | | |
| 7 | 102046188 | 102046302 | MIR548O | | | | | | |
| 7 | 102073976 | 102097268 | ORAI2 | | | | | | |
| 7 | 102096666 | 102105321 | ALKBH4 | | | | | | |
| 7 | 102105329 | 102113615 | LRWD1 | | | | | | |
| 7 | 102106188 | 102106273 | MIR5090 | | | | | | |
| 7 | 102111915 | 102111978 | MIR4467 | | | | | | |
| 7 | 102113547 | 102119381 | POLR2J | | | | | | |
| 7 | 102123585 | 102158224 | RASA4B | | | | | | |
| 7 | 102178365 | 102213068 | POLR2J3 | | | | | | |
| 7 | 102191678 | 102202757 | SPDYE2 | | | | | | |
| 7 | 102191678 | 102202757 | SPDYE2B | | | | | | |
| 7 | 102220092 | 102257205 | RASA4 | | | | | | |
| 7 | 102222764 | 102257203 | RASA4B | | | | | | |
| 7 | 102277194 | 102312176 | POLR2J2 | | | | | | |
| 7 | 102277471 | 102283238 | UPK3BL | | | | | | |
| 7 | 102290771 | 102301847 | SPDYE2 | | | | | | |
| 7 | 102290771 | 102301847 | SPDYE2B | | | | | | |
| 7 | 102389398 | 102449672 | FAM185A | | | | | | |
| 7 | 102453307 | 102715288 | FBXL13 | | | | | | |
| 7 | 102553343 | 102585556 | LRRC17 | | | | | | |
| 7 | 102715327 | 102740210 | ARMC10 | | | | | | |
| 7 | 102740022 | 102789569 | NAPEPLD | | | | | | |
| 7 | 102781716 | 102782850 | RPL19P12 | | | | | | |
| 7 | 102815461 | 102920913 | DPY19L2P2 | | | | | | |
| 7 | 102937872 | 102955133 | PMPCB | | | | | | |
| 7 | 102952920 | 102985320 | DNAJC2 | | | | | | |
| 7 | 102987970 | 103009842 | PSMC2 | | | | | | |
| 7 | 102993176 | 103086624 | SLC26A5 | | | | | | |
| 7 | 103085653 | 103154454 | LOC101927870 | | | | | | |
| 7 | 103112230 | 103629963 | RELN | | RELN | RELN | | | |
| 7 | 103766787 | 103848495 | ORC5 | | | | | | |
| 7 | 103969103 | 104567092 | LHFPL3 | | | | | | |
| 7 | 104622193 | 104631612 | LINC01004 | | | | | | |
| 7 | 104650988 | 104754532 | KMT2E | | | | | | |
| 7 | 104756820 | 105029377 | SRPK2 | | | | | | |
| 7 | 105096947 | 105162705 | PUS7 | | | | | | |
| 7 | 105172531 | 105208124 | RINT1 | | | RINT1 | | | |
| 7 | 105205579 | 105221976 | EFCAB10 | | | | | | |
| 7 | 105245220 | 105517031 | ATXN7L1 | | | | | | |
| 7 | 105603656 | 105676877 | CDHR3 | | | | | | |
| 7 | 105730813 | 105753093 | SYPL1 | | | | | | |
| 7 | 105888731 | 105925638 | NAMPT | | | | | | |
| 7 | 106297210 | 106301634 | CCDC71L | | | | | | |
| 7 | 106505722 | 106549423 | PIK3CG | | | | | | |
| 7 | 106685177 | 106802256 | PRKAR2B | | | | | | |
| 7 | 106809405 | 106842974 | HBP1 | | | | | | |
| 7 | 106842188 | 107204959 | COG5 | | | | | | |
| 7 | 107110501 | 107116125 | GPR22 | | | | | | |
| 7 | 107204401 | 107218968 | DUS4L | | | | | | |
| 7 | 107220421 | 107263762 | BCAP29 | | | | | | |
| 7 | 107296960 | 107358252 | SLC26A4 | | | | | | |
| 7 | 107384141 | 107402112 | CBLL1 | | | | | | |
| 7 | 107405911 | 107443678 | SLC26A3 | | | | | | |
| 7 | 107531551 | 107561643 | DLD | | | DLD | | | |
| 7 | 107564245 | 107643804 | LAMB1 | | | | | | |

| | | | | | | | | |
|---|---|---|---|---|---|---|---|---|
| 7 | 107663995 | 107770801 | LAMB4 | | | | | |
| 7 | 107788070 | 108096841 | NRCAM | | NRCAM | NRCAM | | |
| 7 | 108110865 | 108168605 | PNPLA8 | | PNPLA8 | | | |
| 7 | 108202587 | 108210212 | THAP5 | | | | | |
| 7 | 108210188 | 108215294 | DNAJB9 | | | | | |
| 7 | 108524031 | 108524644 | C7orf66 | | | | | |
| 7 | 109599283 | 109600270 | EIF3IP1 | | | | | |
| 7 | 110303105 | 111202573 | IMMP2L | | | | | |
| 7 | 110731061 | 110765509 | LRRN3 | | | | | |
| 7 | 111366163 | 111461835 | DOCK4 | | DOCK4 | | | |
| 7 | 111846642 | 111983989 | ZNF277 | | | | | |
| 7 | 112063198 | 112117258 | IFRD1 | | | | | |
| 7 | 112120907 | 112130943 | LSMEM1 | | | | | |
| 7 | 112258119 | 112260704 | LOC100996249 | | | | | |
| 7 | 112262435 | 112348122 | LOC101928012 | | | | | |
| 7 | 112402436 | 112430478 | TMEM168 | | | | | |
| 7 | 112459201 | 112579932 | C7orf60 | | | | | |
| 7 | 112594689 | 112635698 | HRAT17 | | | | | |
| 7 | 112720467 | 112727833 | GPR85 | | | | | |
| 7 | 112756772 | 112758637 | LINC00998 | | | | | |
| 7 | 113516881 | 113559082 | PPP1R3A | | | PPP1R3A | | |
| 7 | 113726364 | 114294198 | FOXP2 | | | FOXP2 | | |
| 7 | 114293399 | 114293510 | MIR3666 | | | | | |
| 7 | 114562208 | 114659970 | MDFIC | | | | | |
| 7 | 114719011 | 114766368 | LINC01393 | | | | | |
| 7 | 114763652 | 114871409 | LINC01392 | | | | | |
| 7 | 115575201 | 115670867 | TFEC | | | | | |
| 7 | 115850546 | 115898837 | TES | | | | | |
| 7 | 115915659 | 115926781 | LOC102724434 | | | | | |
| 7 | 116139654 | 116148595 | CAV2 | | | | | |
| 7 | 116164838 | 116201239 | CAV1 | | | CAV1 | | |
| 7 | 116203647 | 116254874 | LINC01510 | | | | | |
| 7 | 116312458 | 116438440 | MET | | | MET | | |
| 7 | 116502562 | 116559313 | CAPZA2 | | | | | |
| 7 | 116592500 | 116870075 | ST7 | | | | | |
| 7 | 116593952 | 116599867 | ST7-OT4 | | | | | |
| 7 | 116660264 | 116660373 | MIR6132 | | | | | |
| 7 | 116712125 | 116785646 | ST7 | | | | | |
| 7 | 116822734 | 116849991 | ST7-OT3 | | | | | |
| 7 | 116916685 | 116963343 | WNT2 | | | | | |
| 7 | 117003275 | 117067577 | ASZ1 | | | | | |
| 7 | 117120016 | 117308718 | CFTR | | | CFTR | | |
| 7 | 117350705 | 117513561 | CTTNBP2 | | CTTNBP2 | | | |
| 7 | 117824085 | 117844093 | LSM8 | | | | | |
| 7 | 117864711 | 117882784 | ANKRD7 | | | | | |
| 7 | 119259483 | 119547429 | LVCAT5 | | | | | |
| 7 | 119913721 | 120390387 | KCND2 | | KCND2 | KCND2 | | |
| 7 | 120427373 | 120498177 | TSPAN12 | | | | | |
| 7 | 120590816 | 120615711 | ING3 | | | | | |
| 7 | 120628750 | 120901972 | CPED1 | | | | | |
| 7 | 120965420 | 120981158 | WNT16 | | | | | |
| 7 | 120988904 | 121036422 | FAM3C | | | | | |
| 7 | 121513158 | 121702090 | PTPRZ1 | | PTPRZ1 | | | |
| 7 | 121713597 | 121784344 | AASS | | | | | |
| 7 | 121941362 | 121950131 | FEZF1 | | | | | |
| 7 | 121958477 | 122526813 | CADPS2 | | | CADPS2 | | CADPS2 |
| 7 | 122337765 | 122339208 | RNF133 | | | | | |
| 7 | 122341719 | 122343021 | RNF148 | | | | | |
| 7 | 122634758 | 122635754 | TAS2R16 | | | | | |
| 7 | 122753587 | 122840025 | SLC13A1 | | | | | |
| 7 | 123092235 | 123174718 | IQUB | | | | | |
| 7 | 123177051 | 123241705 | NDUFA5 | | | NDUFA5 | | |
| 7 | 123241920 | 123277934 | ASB15 | | | | | |
| 7 | 123254214 | 123265011 | LOC102724555 | | | | | |
| 7 | 123295860 | 123304147 | LMOD2 | | | | | |
| 7 | 123321980 | 123389125 | WASL | | | | | |

| | | | | | | | | |
|---|---|---|---|---|---|---|---|---|
| 7 | 123430660 | 123430765 | RNU6-2 | | | | | |
| 7 | 123454192 | 123459484 | HYALP1 | | | | | |
| 7 | 123485222 | 123517531 | HYAL4 | | | | | |
| 7 | 123565285 | 123600100 | SPAM1 | | | | | |
| 7 | 123634675 | 123667700 | LOC105375483 | | | | | |
| 7 | 123670969 | 123673523 | TMEM229A | | | | | |
| 7 | 123977433 | 123992150 | LOC101928211 | | | | | |
| 7 | 124385654 | 124406079 | GPR37 | | | | | |
| 7 | 124417345 | 124430864 | C7orf77 | | | | | |
| 7 | 124462439 | 124785287 | POT1 | | | | | |
| 7 | 124824622 | 125019375 | LOC101928283 | | | | | |
| 7 | 124869632 | 124904345 | LOC101928254 | | | | | |
| 7 | 126078651 | 126892428 | GRM8 | | | GRM8 | GRM8 | |
| 7 | 126698141 | 126698238 | MIR592 | | | | | |
| 7 | 126855180 | 126869975 | LOC101928333 | | | | | |
| 7 | 127010096 | 127032778 | ZNF800 | | | | | |
| 7 | 127116936 | 127125858 | LOC100506682 | | | | | |
| 7 | 127220681 | 127225654 | GCC1 | | | GCC1 | | |
| 7 | 127228405 | 127231759 | ARF5 | | | | | |
| 7 | 127233688 | 127241851 | FSCN3 | | | | | |
| 7 | 127250345 | 127255780 | PAX4 | | | | | |
| 7 | 127292201 | 127732659 | SND1 | | | | | |
| 7 | 127637561 | 127640130 | SND1-IT1 | | | | | |
| 7 | 127667123 | 127671002 | LRRC4 | | | | | |
| 7 | 127721912 | 127722012 | MIR593 | | | | | |
| 7 | 127847924 | 127847996 | MIR129 | | | | | |
| 7 | 127881330 | 127897682 | LEP | | | | | |
| 7 | 127937737 | 127947816 | MGC27345 | | | | | |
| 7 | 127950435 | 127983962 | RBM28 | | | | | |
| 7 | 127990378 | 128001739 | PRRT4 | | | | | |
| 7 | 128032330 | 128050041 | IMPDH1 | | | | | |
| 7 | 128095883 | 128098472 | HILPDA | | | | | |
| 7 | 128116782 | 128142978 | METTL2B | | | | | |
| 7 | 128281294 | 128301052 | LINC01000 | | | | | |
| 7 | 128312319 | 128327926 | FAM71F2 | | | | | |
| 7 | 128349114 | 128371797 | FAM71F1 | | | | | |
| 7 | 128379345 | 128413477 | CALU | | | | | |
| 7 | 128412542 | 128415844 | OPN1SW | | | | | |
| 7 | 128431463 | 128462187 | CCDC136 | | | | | |
| 7 | 128470482 | 128499328 | FLNC | | | FLNC | | |
| 7 | 128502856 | 128505903 | ATP6V1F | | | | | |
| 7 | 128506463 | 128512101 | LOC100130705 | | | | | |
| 7 | 128516918 | 128550773 | KCP | | | | | |
| 7 | 128577990 | 128590096 | IRF5 | | | | | |
| 7 | 128594233 | 128695227 | TNPO3 | | | TNPO3 | | |
| 7 | 128695276 | 128697293 | TPI1P2 | | | | | |
| 7 | 128766324 | 128768050 | LOC407835 | | | | | |
| 7 | 128784711 | 128809535 | TSPAN33 | | | | | |
| 7 | 128828712 | 128853385 | SMO | | | | | |
| 7 | 128864854 | 129070052 | AHCYL2 | | AHCYL2 | | | |
| 7 | 129074273 | 129128239 | STRIP2 | | | | | |
| 7 | 129142319 | 129152773 | SMKR1 | | | | | |
| 7 | 129251542 | 129396922 | NRF1 | | | | | |
| 7 | 129410222 | 129410332 | MIR182 | | | | | |
| 7 | 129414532 | 129414609 | MIR96 | | | | | |
| 7 | 129414744 | 129414854 | MIR183 | | | | | |
| 7 | 129470572 | 129592800 | UBE2H | | | | | |
| 7 | 129658125 | 129691291 | ZC3HC1 | | | | | |
| 7 | 129710348 | 129775560 | KLHDC10 | | | | | |
| 7 | 129804552 | 129845338 | TMEM209 | | | | | |
| 7 | 129847703 | 129856684 | SSMEM1 | | | | | |
| 7 | 129906702 | 129929637 | CPA2 | | | | | |
| 7 | 129932973 | 129964020 | CPA4 | | | | | |
| 7 | 129984629 | 130008571 | CPA5 | | | | | |
| 7 | 130005490 | 130006968 | LOC105375504 | | | | | |
| 7 | 130020211 | 130027949 | CPA1 | | | | | |

| | | | | | | | | |
|---|---|---|---|---|---|---|---|---|
| 7 | 130033611 | 130081051 | CEP41 | | CEP41 | | | |
| 7 | 130126015 | 130146138 | MEST | | | | | |
| 7 | 130126897 | 130131013 | MESTIT1 | | | | | |
| 7 | 130131169 | 130146138 | MEST | | | | | |
| 7 | 130135951 | 130136045 | MIR335 | | | | | |
| 7 | 130146079 | 130353598 | COPG2 | | | | | |
| 7 | 130353485 | 130372268 | TSGA13 | | | | | |
| 7 | 130417381 | 130418860 | KLF14 | | | | | |
| 7 | 130561505 | 130561569 | MIR29A | | | | | |
| 7 | 130562217 | 130562298 | MIR29B1 | | | | | |
| 7 | 130562320 | 130598069 | LINC-PINT | | | | | |
| 7 | 130598222 | 130606705 | LOC100506860 | | | | | |
| 7 | 130626518 | 130794675 | LINC-PINT | | | | | |
| 7 | 130794854 | 131181398 | MKLN1 | | | | | |
| 7 | 131185020 | 131241376 | PODXL | | | | | |
| 7 | 131594978 | 131633712 | LOC101928782 | | | | | |
| 7 | 131808090 | 132261323 | PLXNA4 | | | | | |
| 7 | 131945619 | 131949048 | LOC101928807 | | | | | |
| 7 | 132068246 | 132333447 | PLXNA4 | | | | | |
| 7 | 132333552 | 132413528 | FLJ40288 | | | | | |
| 7 | 132443729 | 132445392 | LOC100506937 | | | | | |
| 7 | 132469622 | 132766918 | CHCHD3 | | | | | |
| 7 | 132719619 | 132719675 | MIR3654 | | | | | |
| 7 | 132783830 | 132793271 | LOC105375512 | | | | | |
| 7 | 132937822 | 133750513 | EXOC4 | | | | | |
| 7 | 132975634 | 132975742 | MIR6133 | | | | | |
| 7 | 133485245 | 133509641 | LOC101928861 | | | | | |
| 7 | 133812104 | 133948933 | LRGUK | | | | | |
| 7 | 133974089 | 134001827 | SLC35B4 | | | | | |
| 7 | 134127106 | 134143888 | AKR1B1 | | | AKR1B1 | | |
| 7 | 134212343 | 134226166 | AKR1B10 | | | | | |
| 7 | 134233848 | 134264592 | AKR1B15 | | | | | |
| 7 | 134331530 | 134364567 | BPGM | | | | | |
| 7 | 134464163 | 134655480 | CALD1 | | | | | |
| 7 | 134671258 | 134820530 | AGBL3 | | | | | |
| 7 | 134777060 | 134855578 | C7orf49 | | | | | |
| 7 | 134832765 | 134850967 | TMEM140 | | | | | |
| 7 | 134850531 | 134855578 | C7orf49 | | | | | |
| 7 | 134868589 | 134896316 | WDR91 | | | | | |
| 7 | 134891746 | 134891830 | MIR6509 | | | | | |
| 7 | 134916730 | 134943244 | STRA8 | | | | | |
| 7 | 135046546 | 135194875 | CNOT4 | | | | | |
| 7 | 135242661 | 135333499 | NUP205 | | | NUP205 | | |
| 7 | 135347220 | 135361160 | C7orf73 | | | | | |
| 7 | 135365986 | 135412952 | SLC13A4 | | | | | |
| 7 | 135414345 | 135433594 | FAM180A | | | | | |
| 7 | 135611502 | 135662204 | LUZP6 | | | | | |
| 7 | 135611502 | 135662204 | MTPN | | | | | |
| 7 | 136553398 | 136701771 | CHRM2 | | | | CHRM2 | CHRM2 |
| 7 | 136583519 | 136849088 | LOC349160 | | | | | |
| 7 | 136587913 | 136588041 | MIR490 | | | | | |
| 7 | 136912091 | 137028546 | PTN | | | | | |
| 7 | 137074384 | 137531609 | DGKI | | | | | |
| 7 | 137559724 | 137686847 | CREB3L2 | | | | | |
| 7 | 137638093 | 137642712 | LOC100130880 | | | | | |
| 7 | 137761177 | 137803050 | AKR1D1 | | | | | |
| 7 | 137808503 | 137808567 | MIR4468 | | | | | |
| 7 | 138145078 | 138270332 | TRIM24 | | | | | |
| 7 | 138279029 | 138363790 | SVOPL | | | | | |
| 7 | 138391038 | 138482941 | ATP6V0A4 | | | ATP6V0A4 | | |
| 7 | 138482738 | 138490769 | TMEM213 | | | | | |
| 7 | 138516126 | 138666064 | KIAA1549 | | | | | |
| 7 | 138710451 | 138720775 | ZC3HAV1L | | | | | |
| 7 | 138728265 | 138794465 | ZC3HAV1 | | | | | |
| 7 | 138818489 | 138876732 | TTC26 | | | | | |
| 7 | 138916230 | 138992982 | UBN2 | | | | | |

| | | | | | | | | |
|---|---|---|---|---|---|---|---|---|
| 7 | 139025104 | 139108203 | LUC7L2 | | | | | |
| 7 | 139025195 | 139031065 | C7orf55 | | | | | |
| 7 | 139025877 | 139108203 | C7orf55-LUC7L2 | | | | | |
| 7 | 139044591 | 139108203 | LUC7L2 | | | | | |
| 7 | 139102208 | 139112272 | LOC100129148 | | | | | |
| 7 | 139138087 | 139168457 | KLRG2 | | | | | |
| 7 | 139208673 | 139229731 | CLEC2L | | | | | |
| 7 | 139246315 | 139477693 | HIPK2 | | | | | |
| 7 | 139478046 | 139720125 | TBXAS1 | | | | | |
| 7 | 139723543 | 139763521 | PARP12 | | | | | |
| 7 | 139784545 | 139876741 | KDM7A | | | | | |
| 7 | 139877060 | 139879440 | JHDM1D | | | | | |
| 7 | 140033551 | 140098350 | SLC37A3 | | | | | |
| 7 | 140103842 | 140126050 | RAB19 | | | | | |
| 7 | 140152839 | 140179369 | MKRN1 | | | | | |
| 7 | 140218218 | 140341286 | DENND2A | | | | | |
| 7 | 140372952 | 140394908 | ADCK2 | | | | | |
| 7 | 140395135 | 140406446 | NDUFB2 | | | | | |
| 7 | 140433812 | 140624564 | BRAF | | | BRAF | | |
| 7 | 140705960 | 140714781 | MRPS33 | | | | | |
| 7 | 140774031 | 141180179 | TMEM178B | | | | | |
| 7 | 141251077 | 141354209 | AGK | | | | | |
| 7 | 141356527 | 141401953 | KIAA1147 | | | | | |
| 7 | 141404137 | 141431071 | WEE2 | | | | | |
| 7 | 141438120 | 141450288 | SSBP1 | | | | | |
| 7 | 141463896 | 141464997 | TAS2R3 | | | | | |
| 7 | 141478288 | 141479188 | TAS2R4 | | | | | |
| 7 | 141490016 | 141491166 | TAS2R5 | | | | | |
| 7 | 141536077 | 141541221 | PRSS37 | | | | | |
| 7 | 141618675 | 141619620 | OR9A4 | | | | | |
| 7 | 141627156 | 141646807 | CLEC5A | | | | | |
| 7 | 141672430 | 141673573 | TAS2R38 | | | | | |
| 7 | 141695678 | 141806547 | MGAM | | | | | |
| 7 | 141811548 | 141922124 | MGAM2 | | | | | |
| 7 | 141940555 | 141946886 | MOXD2P | | | | | |
| 7 | 141951957 | 141957878 | PRSS58 | | | | | |
| 7 | 141968100 | 141972068 | TRY2P | | | | | |
| 7 | 142374130 | 142375525 | MTRNR2L6 | | | | | |
| 7 | 142457318 | 142460927 | PRSS1 | | | | | |
| 7 | 142478756 | 142482399 | PRSS3P2 | | | | | |
| 7 | 142552775 | 142568847 | EPHB6 | | | | | |
| 7 | 142568955 | 142583490 | TRPV6 | | | | | |
| 7 | 142605266 | 142630820 | TRPV5 | | | | | |
| 7 | 142636602 | 142637957 | C7orf34 | | | | | |
| 7 | 142638200 | 142659503 | KEL | | | | | |
| 7 | 142723286 | 142724219 | OR9A2 | | | OR9A2 | | |
| 7 | 142749437 | 142750379 | OR6V1 | | | | | |
| 7 | 142759380 | 142760882 | OR6W1P | | | | | |
| 7 | 142829173 | 142836834 | PIP | | | | | |
| 7 | 142880511 | 142881528 | TAS2R39 | | | | | |
| 7 | 142919171 | 142920143 | TAS2R40 | | | | | |
| 7 | 142952391 | 142983427 | LOC105375545 | | | | | |
| 7 | 142960521 | 142966222 | GSTK1 | | | | | |
| 7 | 142981991 | 142985142 | TMEM139 | | | | | |
| 7 | 142985307 | 143004789 | CASP2 | | | | | |
| 7 | 143013218 | 143049097 | CLCN1 | | | | | |
| 7 | 143050492 | 143059840 | FAM131B | | | | | |
| 7 | 143078359 | 143088206 | ZYX | | | | | |
| 7 | 143079778 | 143079893 | MIR6892 | | | | | |
| 7 | 143088204 | 143220540 | EPHA1 | | | | | |
| 7 | 143140545 | 143141502 | TAS2R60 | | | | | |
| 7 | 143174965 | 143175889 | TAS2R41 | | | | | |
| 7 | 143268893 | 143271480 | CTAGE15 | | | | | |
| 7 | 143318044 | 143422176 | TCAF2 | | | | | |
| 7 | 143339346 | 143344739 | TCAF2P1 | | | | | |
| 7 | 143412938 | 143427173 | TCAF2 | | | | | |

| | | | | | | | | |
|---|---|---|---|---|---|---|---|---|
| 7 | 143452181 | 143454843 | CTAGE6 | | | | | |
| 7 | 143507082 | 143512482 | TCAF2P1 | | | | | |
| 7 | 143509060 | 143533810 | LOC154761 | | | | | |
| 7 | 143548460 | 143599278 | TCAF1 | | | | | |
| 7 | 143632325 | 143633279 | OR2F2 | | | | | |
| 7 | 143657019 | 143658108 | OR2F1 | | | OR2F1 | | |
| 7 | 143701089 | 143702025 | OR6B1 | | | | | |
| 7 | 143747494 | 143748430 | OR2A5 | | | | | |
| 7 | 143771312 | 143772245 | OR2A25 | | | | | |
| 7 | 143792200 | 143793133 | OR2A12 | | | | | |
| 7 | 143806675 | 143807632 | OR2A2 | | | | | |
| 7 | 143826205 | 143827138 | OR2A14 | | | | | |
| 7 | 143880547 | 143883173 | CTAGE4 | | | | | |
| 7 | 143883175 | 143892791 | ARHGEF35 | | | | | |
| 7 | 143892413 | 143996682 | LOC101928605 | | | | | |
| 7 | 143892413 | 143929936 | OR2A1 | | | | | |
| 7 | 143929003 | 143929936 | OR2A42 | | | | | |
| 7 | 143935665 | 144052811 | OR2A1 | | | | | |
| 7 | 143947563 | 143948548 | OR2A9P | | | | | |
| 7 | 143947766 | 143948696 | OR2A20P | | | | | |
| 7 | 143955788 | 143956721 | OR2A7 | | | | | |
| 7 | 143956088 | 143983096 | ARHGEF34P | | | | | |
| 7 | 143963766 | 143966392 | CTAGE4 | | | | | |
| 7 | 143963766 | 143966877 | CTAGE8 | | | | | |
| 7 | 143996465 | 143997395 | OR2A20P | | | | | |
| 7 | 143996613 | 143997598 | OR2A9P | | | | | |
| 7 | 144015217 | 144016150 | OR2A1 | | | | | |
| 7 | 144015217 | 144016150 | OR2A42 | | | | | |
| 7 | 144052488 | 144077725 | ARHGEF5 | | | | | |
| 7 | 144094332 | 144107320 | NOBOX | | | | | |
| 7 | 144149033 | 144533146 | TPK1 | | | | | |
| 7 | 145813452 | 148118088 | CNTNAP2 | | CNTNAP2 | CNTNAP2 | CNTNAP2 | |
| 7 | 146778029 | 146794701 | LOC101928700 | | | | | |
| 7 | 146991341 | 147254962 | MIR548I4 | | | | | |
| 7 | 147075108 | 147075213 | MIR548F4 | | | | | |
| 7 | 147368770 | 147370236 | LOC105375556 | | | | | |
| 7 | 147626684 | 148043927 | MIR548T | | | | | |
| 7 | 148287656 | 148312952 | C7orf33 | | | | | |
| 7 | 148395932 | 148498202 | CUL1 | | | | | |
| 7 | 148504463 | 148581441 | EZH2 | | | | | |
| 7 | 148684618 | 148686516 | GHET1 | | | | | |
| 7 | 148700153 | 148725782 | PDIA4 | | | | | |
| 7 | 148766732 | 148787869 | ZNF786 | | | | | |
| 7 | 148799877 | 148823438 | ZNF425 | | | | | |
| 7 | 148823507 | 148880118 | ZNF398 | | | | | |
| 7 | 148892553 | 148923339 | ZNF282 | | | | | |
| 7 | 148936741 | 148952700 | ZNF212 | | | ZNF212 | | |
| 7 | 148959261 | 148982085 | ZNF783 | | | | | |
| 7 | 148982371 | 148994403 | LOC155060 | | | | | |
| 7 | 149128453 | 149158053 | ZNF777 | | | | | |
| 7 | 149169883 | 149194898 | ZNF746 | | | | | |
| 7 | 149244244 | 149321881 | ZNF767P | | | | | |
| 7 | 149412101 | 149431664 | KRBA1 | | | | | |
| 7 | 149461452 | 149470295 | ZNF467 | | | | | |
| 7 | 149473130 | 149531053 | SSPO | | | | | |
| 7 | 149535508 | 149564568 | ZNF862 | | | | | |
| 7 | 149564782 | 149577801 | ATP6V0E2 | | | | | |
| 7 | 149944300 | 150020758 | ACTR3C | | | | | |
| 7 | 150020295 | 150035245 | LRRC61 | | | | | |
| 7 | 150026937 | 150029811 | ZBED6CL | | | | | |
| 7 | 150035406 | 150038763 | RARRES2 | | | | | |
| 7 | 150065878 | 150071133 | REPIN1 | | | | | |
| 7 | 150076405 | 150095719 | ZNF775 | | | | | |
| 7 | 150102839 | 150109558 | LOC728743 | | | | | |
| 7 | 150130741 | 150145228 | LINC00996 | | | | | |
| 7 | 150147717 | 150176483 | GIMAP8 | | | | | |

| | | | | | | | | |
|---|---|---|---|---|---|---|---|---|
| 7 | 150211944 | 150218161 | GIMAP7 | | | | | |
| 7 | 150264457 | 150271041 | GIMAP4 | | | | | |
| 7 | 150322463 | 150329736 | GIMAP6 | | | | | |
| 7 | 150382793 | 150390728 | GIMAP2 | | | | | |
| 7 | 150413644 | 150421368 | GIMAP1 | | | | | |
| 7 | 150413644 | 150440737 | GIMAP1-GIMAP5 | | | | | |
| 7 | 150434435 | 150440737 | GIMAP5 | | | | | |
| 7 | 150488375 | 150498448 | TMEM176B | | | | | |
| 7 | 150497853 | 150502208 | TMEM176A | | | | | |
| 7 | 150549564 | 150558379 | AOC1 | | | | | |
| 7 | 150642043 | 150675402 | KCNH2 | | KCNH2 | KCNH2 | | |
| 7 | 150688143 | 150701023 | NOS3 | NOS3 | | | | |
| 7 | 150709296 | 150721586 | ATG9B | | | | | |
| 7 | 150725508 | 150744869 | ABCB8 | | | | | |
| 7 | 150745378 | 150749843 | ASIC3 | | | | | |
| 7 | 150750898 | 150755052 | CDK5 | CDK5 | CDK5 | | | CDK5 |
| 7 | 150755298 | 150773614 | SLC4A2 | | | | | |
| 7 | 150773707 | 150777970 | FASTK | | | | | |
| 7 | 150778171 | 150780620 | TMUB1 | | | | | |
| 7 | 150782917 | 150820725 | AGAP3 | | | | | |
| 7 | 150845675 | 150864635 | GBX1 | | | | | |
| 7 | 150872784 | 150884919 | ASB10 | | ASB10 | | | |
| 7 | 150887960 | 150902582 | IQCA1L | | | | | |
| 7 | 150904922 | 150924460 | ABCF2 | | | | | |
| 7 | 150929574 | 150935913 | CHPF2 | | | | | |
| 7 | 150935506 | 150935624 | MIR671 | | | | | |
| 7 | 150936058 | 150974231 | SMARCD3 | | SMARCD3 | | | |
| 7 | 151038846 | 151075548 | NUB1 | | | | | |
| 7 | 151078206 | 151110440 | WDR86 | | | | | |
| 7 | 151125917 | 151137899 | CRYGN | | | | | |
| 7 | 151130574 | 151130725 | MIR3907 | | | | | |
| 7 | 151163097 | 151217010 | RHEB | | | | | |
| 7 | 151253200 | 151576308 | PRKAG2 | | | | | |
| 7 | 151653463 | 151717019 | GALNTL5 | | | | | |
| 7 | 151722758 | 151819432 | GALNT11 | | | | | |
| 7 | 151832009 | 152133090 | KMT2C | | | | | |
| 7 | 152133979 | 152140100 | FABP5P3 | | | | | |
| 7 | 152161208 | 152162630 | LINC01003 | | | | | |
| 7 | 152343586 | 152373250 | XRCC2 | | | | | |
| 7 | 152456833 | 152552464 | ACTR3B | | | | | |
| 7 | 153097003 | 153109319 | LINC01287 | | | | | |
| 7 | 153584181 | 154686000 | DPP6 | | DPP6 | | | |
| 7 | 154720226 | 154797413 | PAXIP1 | | | | | |
| 7 | 154858778 | 154879102 | HTR5A | | HTR5A | HTR5A | HTR5A | |
| 7 | 155089485 | 155101945 | INSIG1 | | | | | |
| 7 | 155149682 | 155160628 | BLACE | | | | | |
| 7 | 155250823 | 155257526 | EN2 | | | EN2 | | |
| 7 | 155293952 | 155326539 | CNPY1 | | | | | |
| 7 | 155434609 | 155437100 | LOC100506302 | | | | | |
| 7 | 155437202 | 155574179 | RBM33 | | | | | |
| 7 | 155592677 | 155604967 | SHH | | | SHH | | |
| 7 | 155755325 | 155759037 | LOC389602 | | | | | |
| 7 | 156230482 | 156238282 | LOC285889 | | | | | |
| 7 | 156264792 | 156395889 | LINC01006 | | | | | |
| 7 | 156333184 | 156333795 | LINC00244 | | | | | |
| 7 | 156431059 | 156433348 | C7orf13 | | | | | |
| 7 | 156433352 | 156469820 | RNF32 | | | | | |
| 7 | 156473569 | 156685902 | LMBR1 | | LMBR1 | | | |
| 7 | 156742416 | 156765876 | NOM1 | | | | | |
| 7 | 156797546 | 156809118 | MNX1 | | | MNX1 | | |
| 7 | 156931654 | 157062066 | UBE3C | | | | | |
| 7 | 157129709 | 157210133 | DNAJB6 | | DNAJB6 | | | |
| 7 | 157258924 | 157292410 | LOC101927914 | | | | | |
| 7 | 157331745 | 158380494 | PTPRN2 | | PTPRN2 | | | |
| 7 | 157367027 | 157367114 | MIR153 | | | | | |
| 7 | 157647276 | 157658782 | LOC100506585 | | | | | |

| | | | | | | | | |
|---|---|---|---|---|---|---|---|---|
| 7 | 158325409 | 158325505 | MIR595 | | | | | |
| 7 | 158383320 | 158384625 | LINC01022 | | | | | |
| 7 | 158384307 | 158384388 | MIR5707 | | | | | |
| 7 | 158423860 | 158497522 | NCAPG2 | | | | | |
| 7 | 158523688 | 158622319 | ESYT2 | | | | | |
| 7 | 158649268 | 158738883 | WDR60 | | | | | |
| 7 | 158801044 | 158818929 | LINC00689 | | | | | |
| 7 | 158820865 | 158937649 | VIPR2 | | | | | |
| 8 | 116085 | 117024 | OR4F21 | | | | | |
| 8 | 158344 | 182318 | RPL23AP53 | | | | | |
| 8 | 182136 | 197340 | ZNF596 | | | | | |
| 8 | 325930 | 333174 | FAM87A | | | | | |
| 8 | 356807 | 419875 | FBXO25 | | | | | |
| 8 | 439789 | 495781 | TDRP | | | | | |
| 8 | 564736 | 1087777 | ERICH1 | | | | | |
| 8 | 688547 | 690374 | LOC401442 | | | | | |
| 8 | 1244293 | 1250823 | LOC286083 | | | | | |
| 8 | 1449531 | 1569830 | DLGAP2 | | DLGAP2 | DLGAP2 | DLGAP2 | DLGAP2 |
| 8 | 1710128 | 1712750 | LOC101927752 | | | | | |
| 8 | 1711869 | 1734736 | CLN8 | CLN8 | CLN8 | | CLN8 | |
| 8 | 1749290 | 1749358 | MIR3674 | | | | | |
| 8 | 1765396 | 1765473 | MIR596 | | | | | |
| 8 | 1772141 | 1906807 | ARHGEF10 | | | | | |
| 8 | 1919562 | 1924610 | KBTBD11-OT1 | | | | | |
| 8 | 1922043 | 1955109 | KBTBD11 | | | | | |
| 8 | 1993157 | 2093380 | MYOM2 | | | | | |
| 8 | 2024668 | 2024720 | MIR7160 | | | | | |
| 8 | 2387218 | 2585991 | LOC101927815 | | | | | |
| 8 | 2792874 | 4852328 | CSMD1 | | | | | |
| 8 | 6261076 | 6264069 | LOC100287015 | | | | | |
| 8 | 6264112 | 6501140 | MCPH1 | | | | | |
| 8 | 6357174 | 6420784 | ANGPT2 | | | | | |
| 8 | 6473123 | 6565730 | MCPH1 | | | | | |
| 8 | 6479644 | 6479741 | MIR8055 | | | | | |
| 8 | 6565877 | 6619021 | AGPAT5 | | | | | |
| 8 | 6602684 | 6602765 | MIR4659A | | | | | |
| 8 | 6602688 | 6602761 | MIR4659B | | | | | |
| 8 | 6666040 | 6693166 | XKR5 | | | | | |
| 8 | 6693075 | 6699975 | GS1-24F4.2 | | | | | |
| 8 | 6728096 | 6735529 | DEFB1 | | | | | |
| 8 | 6782215 | 6783598 | DEFA6 | | | | | |
| 8 | 6793341 | 6795860 | DEFA4 | | | | | |
| 8 | 6808247 | 6809121 | DEFA8P | | | | | |
| 8 | 6816810 | 6817683 | DEFA9P | | | | | |
| 8 | 6825662 | 6826635 | DEFA10P | | | | | |
| 8 | 6835170 | 6837614 | DEFA1 | | | | | |
| 8 | 6835170 | 6837614 | DEFA1B | | | | | |
| 8 | 6844699 | 6847243 | DEFT1P | | | | | |
| 8 | 6844699 | 6847243 | DEFT1P2 | | | | | |
| 8 | 6854287 | 6856724 | DEFA1 | | | | | |
| 8 | 6854287 | 6856724 | DEFA1B | | | | | |
| 8 | 6863802 | 6866346 | DEFT1P | | | | | |
| 8 | 6863802 | 6866346 | DEFT1P2 | | | | | |
| 8 | 6873390 | 6875816 | DEFA3 | | | | | |
| 8 | 6873390 | 6875823 | DEFA1 | | | | | |
| 8 | 6873390 | 6875823 | DEFA1B | | | | | |
| 8 | 6886122 | 6887011 | DEFA11P | | | | | |
| 8 | 6912821 | 6914261 | DEFA5 | | | | | |
| 8 | 7118140 | 7143880 | LINC00965 | | | | | |
| 8 | 7159132 | 7212876 | FAM66B | | | | | |
| 8 | 7170367 | 7177473 | DEFB109P1B | | | | | |
| 8 | 7189908 | 7191501 | USP17L1 | | | | | |
| 8 | 7194636 | 7196229 | USP17L4 | | | | | |
| 8 | 7215497 | 7220490 | ZNF705G | | | | | |
| 8 | 7272384 | 7274354 | DEFB4B | | | | | |
| 8 | 7286415 | 7287870 | DEFB103A | | | | | |

| | | | | | | | | |
|---|---|---|---|---|---|---|---|---|
| 8 | 7286415 | 7287870 | DEFB103B | | | | | |
| 8 | 7305275 | 7321192 | SPAG11B | | | | | |
| 8 | 7327829 | 7332604 | DEFB104A | | | | | |
| 8 | 7327829 | 7332604 | DEFB104B | | | | | |
| 8 | 7340025 | 7343909 | DEFB106A | | | | | |
| 8 | 7340025 | 7343909 | DEFB106B | | | | | |
| 8 | 7345190 | 7347115 | DEFB105A | | | | | |
| 8 | 7345190 | 7347115 | DEFB105B | | | | | |
| 8 | 7353367 | 7366833 | DEFB107A | | | | | |
| 8 | 7353367 | 7366833 | DEFB107B | | | | | |
| 8 | 7397148 | 7399972 | PRR23D1 | | | | | |
| 8 | 7397149 | 7399972 | PRR23D2 | | | | | |
| 8 | 7413659 | 7431920 | FAM90A7P | | | | | |
| 8 | 7627105 | 7628835 | FAM90A10P | | | | | |
| 8 | 7636112 | 7638935 | PRR23D2 | | | | | |
| 8 | 7636112 | 7638936 | PRR23D1 | | | | | |
| 8 | 7669241 | 7673238 | DEFB107A | | | | | |
| 8 | 7669241 | 7673238 | DEFB107B | | | | | |
| 8 | 7679487 | 7681412 | DEFB105A | | | | | |
| 8 | 7679487 | 7681412 | DEFB105B | | | | | |
| 8 | 7682693 | 7686575 | DEFB106A | | | | | |
| 8 | 7682693 | 7686575 | DEFB106B | | | | | |
| 8 | 7693992 | 7698764 | DEFB104A | | | | | |
| 8 | 7693992 | 7698764 | DEFB104B | | | | | |
| 8 | 7705401 | 7707807 | SPAG11B | | | | | |
| 8 | 7705401 | 7721319 | SPAG11A | | | | | |
| 8 | 7738725 | 7740180 | DEFB103A | | | | | |
| 8 | 7738725 | 7740180 | DEFB103B | | | | | |
| 8 | 7752086 | 7754238 | DEFB4A | | | | | |
| 8 | 7783858 | 7809935 | ZNF705B | | | | | |
| 8 | 7812534 | 7866277 | FAM66E | | | | | |
| 8 | 7829182 | 7830775 | USP17L8 | | | | | |
| 8 | 7833914 | 7835507 | USP17L3 | | | | | |
| 8 | 7847936 | 7855043 | DEFB109P1B | | | | | |
| 8 | 7946462 | 7946611 | MIR548I3 | | | | | |
| 8 | 8086091 | 8102387 | FAM86B3P | | | | | |
| 8 | 8175257 | 8239344 | SGK223 | | | | | |
| 8 | 8559665 | 8561617 | CLDN23 | | | | | |
| 8 | 8641998 | 8751131 | MFHAS1 | | | | | |
| 8 | 8860313 | 8890849 | ERI1 | | | | | |
| 8 | 8905954 | 8906028 | MIR4660 | | | | | |
| 8 | 8993763 | 9009152 | PPP1R3B | | | | | |
| 8 | 9046508 | 9060366 | LOC101929128 | | | | | |
| 8 | 9182560 | 9192590 | LOC157273 | | | | | |
| 8 | 9413444 | 9639856 | TNKS | | | TNKS | | |
| 8 | 9599181 | 9599278 | MIR597 | | | | | |
| 8 | 9757573 | 9760839 | LINC00599 | | | | | |
| 8 | 9760897 | 9760982 | MIR124 | | | | | |
| 8 | 9911778 | 10286401 | MSRA | | | | | |
| 8 | 10332074 | 10339484 | LINCR-0001 | | | | | |
| 8 | 10383055 | 10411676 | PRSS55 | | | | | |
| 8 | 10463859 | 10512617 | RP1L1 | | | | | |
| 8 | 10524487 | 10524580 | MIR4286 | | | | | |
| 8 | 10530146 | 10558103 | C8orf74 | | | | | |
| 8 | 10581277 | 10588084 | SOX7 | | | | | |
| 8 | 10622470 | 10697409 | PINX1 | | | | | |
| 8 | 10682882 | 10682953 | MIR1322 | | | | | |
| 8 | 10697535 | 10704019 | LOC101929229 | | | | | |
| 8 | 10753656 | 11058875 | XKR6 | | | | | |
| 8 | 10892715 | 10892812 | MIR598 | | | | | |
| 8 | 10920161 | 10924599 | LOC101929269 | | | | | |
| 8 | 11141999 | 11185654 | MTMR9 | | | | | |
| 8 | 11188494 | 11189695 | SLC35G5 | | | | | |
| 8 | 11197145 | 11225961 | TDH | | | | | |
| 8 | 11225910 | 11324276 | FAM167A | | | | | |
| 8 | 11351520 | 11422108 | BLK | | | BLK | | |

| | | | | | | | | |
|---|---|---|---|---|---|---|---|---|
| 8 | 11434043 | 11438850 | LINC00208 | | | | | |
| 8 | 11534427 | 11617511 | GATA4 | | | GATA4 | | |
| 8 | 11562870 | 11563067 | SNORA99 | | | | | |
| 8 | 11565364 | 11617511 | GATA4 | | | GATA4 | | |
| 8 | 11618764 | 11620732 | C8orf49 | | | | | |
| 8 | 11627171 | 11644854 | NEIL2 | | | NEIL2 | | |
| 8 | 11653081 | 11696818 | FDFT1 | | | | | |
| 8 | 11700033 | 11725659 | CTSB | | | | | |
| 8 | 11831445 | 11832108 | DEFB136 | | | | | |
| 8 | 11839829 | 11842099 | DEFB135 | | | | | |
| 8 | 11850685 | 11858261 | DEFB134 | | | | | |
| 8 | 11921897 | 11929256 | DEFB130 | | | | | |
| 8 | 11921897 | 11929256 | LOC100133267 | | | | | |
| 8 | 11946846 | 11973025 | ZNF705D | | | | | |
| 8 | 11973290 | 12008698 | FAM66D | | | | | |
| 8 | 11985366 | 11986806 | LOC392196 | | | | | |
| 8 | 11989925 | 11991518 | USP17L7 | | | | | |
| 8 | 11994676 | 11996269 | USP17L2 | | | | | |
| 8 | 12030962 | 12034776 | FAM90A2P | | | | | |
| 8 | 12039612 | 12051624 | FAM86B1 | | | | | |
| 8 | 12168470 | 12175825 | DEFB130 | | | | | |
| 8 | 12168470 | 12175825 | LOC100133267 | | | | | |
| 8 | 12219527 | 12268510 | FAM66A | | | | | |
| 8 | 12236775 | 12237298 | LOC649352 | | | | | |
| 8 | 12250792 | 12257874 | DEFB109P1 | | | | | |
| 8 | 12272029 | 12275541 | FAM90A25P | | | | | |
| 8 | 12283123 | 12293852 | FAM86B2 | | | | | |
| 8 | 12294521 | 12424354 | LOC100506990 | | | | | |
| 8 | 12394587 | 12523120 | LOC729732 | | | | | |
| 8 | 12576635 | 12576704 | MIR5692A1 | | | | | |
| 8 | 12576640 | 12576699 | MIR5692A2 | | | | | |
| 8 | 12579405 | 12612992 | LONRF1 | | | | | |
| 8 | 12584740 | 12584808 | MIR3926 | | | | | |
| 8 | 12623570 | 12668910 | LOC340357 | | | | | |
| 8 | 12651751 | 12675800 | LINC00681 | | | | | |
| 8 | 12803182 | 12887284 | KIAA1456 | | | | | |
| 8 | 12940871 | 13372429 | DLC1 | | | | | |
| 8 | 13424351 | 13425797 | C8orf48 | | | | | |
| 8 | 13487198 | 13490085 | LOC102725080 | | | | | |
| 8 | 13947372 | 15095792 | SGCZ | | | | | |
| 8 | 14710946 | 14711019 | MIR383 | | | | | |
| 8 | 15397595 | 15624158 | TUSC3 | | | | | |
| 8 | 15965386 | 16050300 | MSR1 | | | MSR1 | | |
| 8 | 16850333 | 16859674 | FGF20 | | FGF20 | | | |
| 8 | 16884746 | 16980148 | MICU3 | | | | | |
| 8 | 17013835 | 17080241 | ZDHHC2 | | | | | |
| 8 | 17086739 | 17104387 | CNOT7 | | | | | |
| 8 | 17104400 | 17155533 | VPS37A | | | | | |
| 8 | 17154305 | 17271040 | MTMR7 | | | | | |
| 8 | 17354596 | 17428077 | SLC7A2 | | | | | |
| 8 | 17433941 | 17500642 | PDGFRL | | | | | |
| 8 | 17501302 | 17658426 | MTUS1 | | MTUS1 | | | |
| 8 | 17539086 | 17539166 | MIR548V | | | | | |
| 8 | 17721899 | 17753047 | FGL1 | | | | | |
| 8 | 17780365 | 17887457 | PCM1 | | | | | |
| 8 | 17913807 | 17942507 | ASAH1 | | | | | |
| 8 | 17942376 | 17955160 | LOC101929066 | | | | | |
| 8 | 18027970 | 18081198 | NAT1 | | | | | |
| 8 | 18248754 | 18258723 | NAT2 | | NAT2 | | | |
| 8 | 18384812 | 18871196 | PSD3 | | | | | |
| 8 | 19041185 | 19103032 | LOC100128993 | | | | | |
| 8 | 19171080 | 19253729 | SH2D4A | | | | | |
| 8 | 19261671 | 19540261 | CSGALNACT1 | | | | | |
| 8 | 19674917 | 19709586 | INTS10 | | | | | |
| 8 | 19796581 | 19824770 | LPL | | | LPL | | |
| 8 | 20002365 | 20040717 | SLC18A1 | | | SLC18A1 | | |

| | | | | | | | | |
|---|---|---|---|---|---|---|---|---|
| 8 | 20054703 | 20079207 | ATP6V1B2 | | ATP6V1B2 | | | |
| 8 | 20103675 | 20147969 | LZTS1 | | LZTS1 | LZTS1 | | LZTS1 |
| 8 | 20811143 | 20826449 | LOC102467222 | | | | | |
| 8 | 20831496 | 20852630 | LOC286114 | | | | | |
| 8 | 21155749 | 21166960 | LOC101929172 | | | | | |
| 8 | 21549529 | 21646346 | GFRA2 | | | GFRA2 | | |
| 8 | 21766383 | 21771219 | DOK2 | | | | | |
| 8 | 21777179 | 21864096 | XPO7 | | | | | |
| 8 | 21881620 | 21894408 | NPM2 | | | | | |
| 8 | 21900263 | 21906319 | FGF17 | | | | | |
| 8 | 21911065 | 21940038 | DMTN | | | | | |
| 8 | 21946713 | 21961891 | FAM160B2 | | | | | |
| 8 | 21964382 | 21966932 | NUDT18 | | | | | |
| 8 | 21971931 | 21988565 | HR | | | | | |
| 8 | 21995526 | 21999464 | REEP4 | | | | | |
| 8 | 22004342 | 22014344 | LGI3 | | | | | |
| 8 | 22019183 | 22021991 | SFTPC | | SFTPC | | | |
| 8 | 22022652 | 22069840 | BMP1 | | | | | |
| 8 | 22077215 | 22089851 | PHYHIP | | | | | |
| 8 | 22102474 | 22102556 | MIR320A | | | | | |
| 8 | 22102618 | 22108680 | POLR3D | | | | | |
| 8 | 22112088 | 22132675 | LOC100507071 | | | | | |
| 8 | 22132809 | 22213584 | PIWIL2 | | | | | |
| 8 | 22224761 | 22280249 | SLC39A14 | | | | | |
| 8 | 22298482 | 22398657 | PPP3CC | | | | | |
| 8 | 22409250 | 22433008 | SORBS3 | | | SORBS3 | | |
| 8 | 22436253 | 22455538 | PDLIM2 | | | | | |
| 8 | 22457113 | 22461662 | C8orf58 | | | | | |
| 8 | 22462144 | 22477983 | CCAR2 | | | | | |
| 8 | 22477946 | 22526661 | BIN3 | | | BIN3 | | |
| 8 | 22497883 | 22499722 | BIN3-IT1 | | | | | |
| 8 | 22545173 | 22550815 | EGR3 | | | | | |
| 8 | 22570764 | 22785421 | PEBP4 | | | | | |
| 8 | 22735484 | 22745535 | LOC101929237 | | | | | |
| 8 | 22844929 | 22877710 | RHOBTB2 | | RHOBTB2 | | | |
| 8 | 22877647 | 22926700 | TNFRSF10B | | | | | |
| 8 | 22925741 | 22941132 | LOC286059 | | | | | |
| 8 | 22941867 | 22961070 | LOC254896 | | | | | |
| 8 | 22960326 | 22974950 | TNFRSF10C | | | | | |
| 8 | 22993100 | 23021543 | TNFRSF10D | | | | | |
| 8 | 23048969 | 23082680 | TNFRSF10A | | | | | |
| 8 | 23082733 | 23088439 | LOC389641 | | | | | |
| 8 | 23101149 | 23119513 | CHMP7 | | | | | |
| 8 | 23145604 | 23153792 | R3HCC1 | | | | | |
| 8 | 23154409 | 23261722 | LOXL2 | | | | | |
| 8 | 23193720 | 23223638 | LOC100507156 | | | | | |
| 8 | 23286664 | 23315244 | ENTPD4 | | | | | |
| 8 | 23386307 | 23432976 | SLC25A37 | | | | | |
| 8 | 23536205 | 23540450 | NKX3-1 | | | | | |
| 8 | 23559963 | 23564111 | NKX2-6 | | | | | |
| 8 | 23699433 | 23712320 | STC1 | | | | | |
| 8 | 24151552 | 24216531 | ADAM28 | | | | | |
| 8 | 24153326 | 24406131 | LOC101929294 | | | | | |
| 8 | 24241797 | 24263526 | ADAMDEC1 | | | | | |
| 8 | 24298508 | 24367077 | ADAM7 | | | | | |
| 8 | 24347824 | 24372381 | LOC101929315 | | | | | |
| 8 | 24771273 | 24776606 | NEFM | | NEFM | NEFM | | |
| 8 | 24808468 | 24814383 | NEFL | | NEFL | | | |
| 8 | 24811309 | 24811381 | MIR6841 | | | | | |
| 8 | 25042286 | 25270619 | DOCK5 | | | | | |
| 8 | 25202917 | 25202990 | MIR6876 | | | | | |
| 8 | 25276773 | 25282556 | GNRH1 | | | | | |
| 8 | 25285363 | 25315984 | KCTD9 | | | | | |
| 8 | 25316446 | 25365433 | CDCA2 | | | | | |
| 8 | 25699245 | 25902640 | EBF2 | | | | | |
| 8 | 26149006 | 26230195 | PPP2R2A | | | PPP2R2A | | |

| | | | | | | | | |
|---|---|---|---|---|---|---|---|---|
| 8 | 26240522 | 26270644 | BNIP3L | | | | | |
| 8 | 26362195 | 26371483 | PNMA2 | | | PNMA2 | | |
| 8 | 26371708 | 26515693 | DPYSL2 | | DPYSL2 | | | |
| 8 | 26605666 | 26722922 | ADRA1A | | ADRA1A | | | |
| 8 | 27092839 | 27115956 | STMN4 | | | | | |
| 8 | 27142402 | 27168836 | TRIM35 | | | | | |
| 8 | 27168998 | 27316908 | PTK2B | | | PTK2B | | |
| 8 | 27290886 | 27290951 | MIR6842 | | | | | |
| 8 | 27317277 | 27336813 | CHRNA2 | | | CHRNA2 | CHRNA2 | CHRNA2 |
| 8 | 27348518 | 27402439 | EPHX2 | | | EPHX2 | | |
| 8 | 27454433 | 27472328 | CLU | | CLU | | | |
| 8 | 27468117 | 27468268 | MIR6843 | | | | | |
| 8 | 27491576 | 27534286 | SCARA3 | | | | | |
| 8 | 27559189 | 27559284 | MIR3622B | | | | | |
| 8 | 27559193 | 27559276 | MIR3622A | | | | | |
| 8 | 27590832 | 27630170 | CCDC25 | | | | | |
| 8 | 27632057 | 27662424 | ESCO2 | | | | | |
| 8 | 27667137 | 27695612 | PBK | | | | | |
| 8 | 27727398 | 27850369 | SCARA5 | | | | | |
| 8 | 27743555 | 27743633 | MIR4287 | | | | | |
| 8 | 27879480 | 27941388 | NUGGC | | | | | |
| 8 | 27947747 | 28048669 | ELP3 | | | | | |
| 8 | 28174648 | 28200871 | PNOC | | | PNOC | | |
| 8 | 28203101 | 28243977 | ZNF395 | | | | | |
| 8 | 28285925 | 28347835 | FBXO16 | | | | | |
| 8 | 28351721 | 28431785 | FZD3 | | FZD3 | | | |
| 8 | 28362632 | 28362699 | MIR4288 | | | | | |
| 8 | 28387966 | 28388016 | MIR7641 | | | | | |
| 8 | 28555919 | 28611207 | EXTL3 | | | | | |
| 8 | 28625174 | 28747698 | INTS9 | | | | | |
| 8 | 28747910 | 28910242 | HMBOX1 | | | | | |
| 8 | 28924794 | 29120610 | KIF13B | | | KIF13B | | |
| 8 | 29190578 | 29208267 | DUSP4 | | | | | |
| 8 | 29578775 | 29605625 | LINC00589 | | | | | |
| 8 | 29605824 | 29656008 | LOC101929450 | | | | | |
| 8 | 29672519 | 29686020 | LOC101929470 | | | | | |
| 8 | 29779028 | 29811123 | FAM183CP | | | | | |
| 8 | 29814787 | 29814864 | MIR3148 | | | | | |
| 8 | 29920257 | 30108213 | MIR548O2 | | | | | |
| 8 | 29920527 | 29940724 | SARAF | | | | | |
| 8 | 29952921 | 29995222 | LEPROTL1 | | | | | |
| 8 | 29989186 | 30002200 | MBOAT4 | | | | | |
| 8 | 30013812 | 30041155 | DCTN6 | | | | | |
| 8 | 30239634 | 30429778 | RBPMS | | | | | |
| 8 | 30436030 | 30515738 | GTF2E2 | | | | | |
| 8 | 30496116 | 30503469 | SMIM18 | | | | | |
| 8 | 30535579 | 30585486 | GSR | | | GSR | | |
| 8 | 30601681 | 30624520 | UBXN8 | | | | | |
| 8 | 30643125 | 30670352 | PPP2CB | | | | | |
| 8 | 30689059 | 30706533 | TEX15 | | | | | |
| 8 | 30853317 | 30890317 | PURG | | | | | |
| 8 | 30890777 | 31031277 | WRN | | | WRN | | |
| 8 | 31497267 | 32600770 | NRG1 | NRG1 | NRG1 | NRG1 | | NRG1 |
| 8 | 31883251 | 31996993 | NRG1-IT1 | | | | | |
| 8 | 32298261 | 32300241 | NRG1-IT3 | | | | | |
| 8 | 32405727 | 32622558 | NRG1 | NRG1 | NRG1 | NRG1 | | NRG1 |
| 8 | 33228343 | 33330664 | FUT10 | | | | | |
| 8 | 33342684 | 33358778 | MAK16 | | | | | |
| 8 | 33356026 | 33370703 | TTI2 | | | | | |
| 8 | 33405271 | 33424646 | RNF122 | | | | | |
| 8 | 33448847 | 33457624 | DUSP26 | | | | | |
| 8 | 34641438 | 34722316 | LINC01288 | | | | | |
| 8 | 35092974 | 35652181 | UNC5D | | | | | |
| 8 | 35529680 | 35567838 | LOC101929550 | | | | | |
| 8 | 36641841 | 36793643 | KCNU1 | | | | | |
| 8 | 36979470 | 37258543 | MIR1268A | | | | | |

| | | | | | | | | |
|---|---|---|---|---|---|---|---|---|
| 8 | 37263981 | 37378904 | LINC01605 | | | | | |
| 8 | 37553268 | 37557539 | ZNF703 | | | | | |
| 8 | 37575096 | 37591011 | LOC101929622 | | | | | |
| 8 | 37592278 | 37594944 | LOC102723701 | | | | | |
| 8 | 37594096 | 37604071 | ERLIN2 | | | | | |
| 8 | 37604073 | 37605564 | LOC728024 | | | | | |
| 8 | 37620100 | 37637286 | PROSC | | | | | |
| 8 | 37654400 | 37701504 | ADGRA2 | | | | | |
| 8 | 37701397 | 37707431 | BRF2 | | | | | |
| 8 | 37716464 | 37757015 | RAB11FIP1 | | | RAB11FIP1 | | |
| 8 | 37791799 | 37797647 | GOT1L1 | | | | | |
| 8 | 37820513 | 37824184 | ADRB3 | | | | ADRB3 | |
| 8 | 37888019 | 37917883 | EIF4EBP1 | | | | | |
| 8 | 37963010 | 37997598 | ASH2L | | | | | |
| 8 | 38000217 | 38008600 | STAR | | STAR | | | |
| 8 | 38020838 | 38034248 | LSM1 | | | | | |
| 8 | 38034105 | 38070819 | BAG4 | | | | | |
| 8 | 38089008 | 38120287 | DDHD2 | | DDHD2 | | | |
| 8 | 38120649 | 38126738 | PLPP5 | | | | | |
| 8 | 38132560 | 38239790 | WHSC1L1 | | | | | |
| 8 | 38243958 | 38267042 | LETM2 | | | | | |
| 8 | 38268655 | 38326352 | FGFR1 | | | | | |
| 8 | 38368351 | 38386180 | C8orf86 | | | | | |
| 8 | 38457692 | 38458775 | RNF5P1 | | | | | |
| 8 | 38585703 | 38710546 | TACC1 | | | TACC1 | | |
| 8 | 38758752 | 38831430 | PLEKHA2 | | | | | |
| 8 | 38831667 | 38846181 | HTRA4 | | | | | |
| 8 | 38846326 | 38854041 | TM2D2 | | | | | |
| 8 | 38854504 | 38962779 | ADAM9 | | | | | |
| 8 | 38965047 | 39142436 | ADAM32 | | | | | |
| 8 | 39172181 | 39260375 | ADAM5 | | | | | |
| 8 | 39308563 | 39380508 | ADAM3A | | | | | |
| 8 | 39420630 | 39437653 | LOC100130964 | | | | | |
| 8 | 39442086 | 39587583 | ADAM18 | | | | | |
| 8 | 39601254 | 39695808 | ADAM2 | | | | | |
| 8 | 39771327 | 39786309 | IDO1 | | | | | |
| 8 | 39792473 | 39873910 | IDO2 | | | | | |
| 8 | 40010986 | 40012827 | C8orf4 | | | | | |
| 8 | 40388110 | 40755343 | ZMAT4 | | | | | |
| 8 | 41119475 | 41166990 | SFRP1 | | | | | |
| 8 | 41348080 | 41368499 | GOLGA7 | | | | | |
| 8 | 41386724 | 41402565 | GINS4 | | | | | |
| 8 | 41391823 | 41435887 | LOC102723729 | | | | | |
| 8 | 41435706 | 41482520 | GPAT4 | | | | | |
| 8 | 41503828 | 41504878 | NKX6-3 | | | | | |
| 8 | 41510743 | 41754280 | ANK1 | | ANK1 | ANK1 | | |
| 8 | 41517958 | 41518025 | MIR486 | | | | | |
| 8 | 41786996 | 41909544 | KAT6A | | | | | |
| 8 | 42010463 | 42028701 | AP3M2 | | | AP3M2 | | |
| 8 | 42032235 | 42065242 | PLAT | PLAT | | PLAT | | PLAT |
| 8 | 42112840 | 42128479 | LOC101929897 | | | | | |
| 8 | 42128819 | 42190171 | IKBKB | | | | | |
| 8 | 42195972 | 42229331 | POLB | | | | | |
| 8 | 42231585 | 42234674 | DKK4 | | | | | |
| 8 | 42249278 | 42263455 | VDAC3 | | | | | |
| 8 | 42273979 | 42397356 | SLC20A2 | | | | | |
| 8 | 42396297 | 42408140 | SMIM19 | | | | | |
| 8 | 42552561 | 42592209 | CHRNB3 | | | CHRNB3 | | |
| 8 | 42607779 | 42623929 | CHRNA6 | | | CHRNA6 | | |
| 8 | 42691816 | 42698474 | THAP1 | | | | | |
| 8 | 42704779 | 42751866 | RNF170 | | RNF170 | | | |
| 8 | 42751339 | 42751418 | MIR4469 | | | | | |
| 8 | 42752032 | 42885682 | HOOK3 | | | | | |
| 8 | 42911441 | 42940932 | FNTA | | | | | |
| 8 | 42948648 | 42978323 | POMK | | | | | |
| 8 | 42995591 | 43057970 | HGSNAT | | | | | |

| | | | | | | | | |
|---|---|---|---|---|---|---|---|---|
| 8 | 43147584 | 43218328 | POTEA | | | | | |
| 8 | 47752507 | 47767407 | LINC00293 | | | | | |
| 8 | 48100929 | 48104439 | LOC100287846 | | | | | |
| 8 | 48173469 | 48648563 | SPIDR | | | | | |
| 8 | 48649475 | 48650726 | CEBPD | | | | | |
| 8 | 48685668 | 48872743 | PRKDC | | | | | |
| 8 | 48872762 | 48890719 | MCM4 | | | | | |
| 8 | 48920994 | 48974454 | UBE2V2 | | | | | |
| 8 | 49464126 | 49611069 | LOC101929268 | | | | | |
| 8 | 49532962 | 49536455 | LOC101929217 | | | | | |
| 8 | 49627473 | 49647870 | EFCAB1 | | | | | |
| 8 | 49830238 | 49833999 | SNAI2 | | | | | |
| 8 | 49966894 | 49988642 | C8orf22 | | | | | |
| 8 | 50409321 | 50424739 | LOC100507464 | | | | | |
| 8 | 50822348 | 51706678 | SNTG1 | | | SNTG1 | | |
| 8 | 52232136 | 52722005 | PXDNL | | | | | |
| 8 | 52730134 | 52811746 | PCMTD1 | | | | | |
| 8 | 53023391 | 53322439 | ST18 | | | | | |
| 8 | 53106921 | 53112112 | LOC101929341 | | | | | |
| 8 | 53446596 | 53478021 | FAM150A | | | | | |
| 8 | 53535017 | 53627026 | RB1CC1 | | | | | |
| 8 | 53852467 | 53853454 | NPBWR1 | | | | | |
| 8 | 54138275 | 54164257 | OPRK1 | | | OPRK1 | | |
| 8 | 54628102 | 54755871 | ATP6V1H | | | ATP6V1H | | |
| 8 | 54764367 | 54871864 | RGS20 | | | | | |
| 8 | 54879113 | 54935016 | TCEA1 | | | | | |
| 8 | 54958926 | 55014577 | LYPLA1 | | | LYPLA1 | | |
| 8 | 55047780 | 55061074 | MRPL15 | | | | | |
| 8 | 55370494 | 55373456 | SOX17 | | | | | |
| 8 | 55528626 | 55543394 | RP1 | | | | | |
| 8 | 56015016 | 56438710 | XKR4 | | | | | |
| 8 | 56361756 | 56367881 | SBF1P1 | | | | | |
| 8 | 56429746 | 56433537 | LOC105375843 | | | | | |
| 8 | 56651302 | 56685966 | TMEM68 | | | | | |
| 8 | 56685790 | 56739004 | TGS1 | | | | | |
| 8 | 56792385 | 56925006 | LYN | | | | | |
| 8 | 56980738 | 56987140 | RPS20 | | | | | |
| 8 | 56986397 | 56986460 | SNORD54 | | | | | |
| 8 | 57025500 | 57026541 | MOS | | | | | |
| 8 | 57073467 | 57123859 | PLAG1 | | | | | |
| 8 | 57124196 | 57131357 | CHCHD7 | | | | | |
| 8 | 57212206 | 57233335 | SDR16C5 | | | | | |
| 8 | 57287276 | 57303269 | SDR16C6P | | | | | |
| 8 | 57353512 | 57359293 | PENK | | | PENK | | |
| 8 | 57358365 | 57464626 | LOC101929415 | | | | | |
| 8 | 57430877 | 57472382 | LINC00968 | | | | | |
| 8 | 57870487 | 57906430 | IMPAD1 | | | | | |
| 8 | 58130834 | 58145513 | LINC01606 | | | | | |
| 8 | 58173784 | 58179170 | LOC286177 | | | | | |
| 8 | 58192101 | 58197290 | LINC00588 | | | | | |
| 8 | 58256345 | 58277415 | LOC101929488 | | | | | |
| 8 | 58658707 | 58662756 | LOC286178 | | | | | |
| 8 | 58890916 | 58896685 | LINC01602 | | | | | |
| 8 | 58907112 | 59062277 | FAM110B | | | | | |
| 8 | 59168329 | 59184660 | LOC101929528 | | | | | |
| 8 | 59323822 | 59364060 | UBXN2B | | | | | |
| 8 | 59402736 | 59412720 | CYP7A1 | | | | | |
| 8 | 59465727 | 59495419 | SDCBP | | | | | |
| 8 | 59496063 | 59572404 | NSMAF | | | | | |
| 8 | 59717976 | 60031767 | TOX | | | | | |
| 8 | 61101422 | 61193954 | CA8 | | | | | |
| 8 | 61314729 | 61326377 | LINC01301 | | | | | |
| 8 | 61429468 | 61536203 | RAB2A | | | RAB2A | | |
| 8 | 61591323 | 61780586 | CHD7 | | CHD7 | CHD7 | | |
| 8 | 61878679 | 61880307 | LOC100130298 | | | | | |
| 8 | 62200524 | 62414204 | CLVS1 | | | | | |

| | | | | | | | | |
|---|---|---|---|---|---|---|---|---|
| 8 | 62413114 | 62627199 | ASPH | | | | | |
| 8 | 62627346 | 62627418 | MIR4470 | | | | | |
| 8 | 63161500 | 63912211 | NKAIN3 | | | | | |
| 8 | 63890419 | 63897460 | UG0898H09 | | | | | |
| 8 | 63927638 | 63951610 | GGH | | | | | |
| 8 | 63972047 | 63998612 | TTPA | | | | | |
| 8 | 64080283 | 64125346 | YTHDF3 | | | | | |
| 8 | 64378406 | 64388040 | LOC102724612 | | | | | |
| 8 | 64681987 | 64698054 | LINC01289 | | | | | |
| 8 | 64769499 | 65281115 | LOC102724623 | | | | | |
| 8 | 65285774 | 65291814 | MIR124 | | | | | |
| 8 | 65486865 | 65489820 | LOC401463 | | | | | |
| 8 | 65492794 | 65496191 | BHLHE22 | | | | | |
| 8 | 65508528 | 65711348 | CYP7B1 | | CYP7B1 | | | |
| 8 | 66073379 | 66092575 | LINC00251 | | | | | |
| 8 | 66439242 | 66474901 | LINC01299 | | | | | |
| 8 | 66514690 | 66546452 | ARMC1 | | | | | |
| 8 | 66556887 | 66622798 | MTFR1 | | | | | |
| 8 | 66626568 | 66753969 | PDE7A | | | | | |
| 8 | 66933790 | 67012755 | DNAJC5B | | | | | |
| 8 | 67039277 | 67087718 | TRIM55 | | | | | |
| 8 | 67088611 | 67090880 | CRH | CRH | CRH | | CRH | |
| 8 | 67104348 | 67109554 | LINC00967 | | | | | |
| 8 | 67331821 | 67342968 | RRS1 | | | | | |
| 8 | 67344717 | 67381044 | ADHFE1 | | | | | |
| 8 | 67405490 | 67430759 | C8orf46 | | | | | |
| 8 | 67474409 | 67525484 | MYBL1 | | | | | |
| 8 | 67542487 | 67579452 | VCPIP1 | | | | | |
| 8 | 67579786 | 67593377 | C8orf44 | | | | | |
| 8 | 67579786 | 67774257 | C8orf44-SGK3 | | | | | |
| 8 | 67624652 | 67774257 | SGK3 | | | SGK3 | | |
| 8 | 67679631 | 67680240 | PTTG3P | | | | | |
| 8 | 67687415 | 67774257 | SGK3 | | | SGK3 | | |
| 8 | 67782983 | 67817599 | MCMDC2 | | | | | |
| 8 | 67834164 | 67837777 | SNHG6 | | | | | |
| 8 | 67834708 | 67834784 | SNORD87 | | | | | |
| 8 | 67858735 | 67874825 | TCF24 | | | | | |
| 8 | 67900366 | 67940786 | PPP1R42 | | | | | |
| 8 | 67955314 | 67974562 | COPS5 | | | COPS5 | | |
| 8 | 67976602 | 68108849 | CSPP1 | | | | | |
| 8 | 68109883 | 68255912 | ARFGEF1 | | | | | |
| 8 | 68334404 | 68658620 | CPA6 | | | CPA6 | | |
| 8 | 68864602 | 69143897 | PREX2 | | | | | |
| 8 | 69215702 | 69731258 | C8orf34 | | | | | |
| 8 | 69824037 | 70016425 | LINC01592 | | | | | |
| 8 | 70337105 | 70360479 | LINC01603 | | | | | |
| 8 | 70378858 | 70573147 | SULF1 | | | | | |
| 8 | 70584567 | 70747299 | SLCO5A1 | | | | | |
| 8 | 70963885 | 70983562 | PRDM14 | | | | | |
| 8 | 71024266 | 71316020 | NCOA2 | | | | | |
| 8 | 71383368 | 71397922 | LOC101926892 | | | | | |
| 8 | 71485452 | 71520694 | TRAM1 | | | | | |
| 8 | 71520811 | 71581447 | LACTB2 | | | | | |
| 8 | 71581599 | 71648177 | XKR9 | | | | | |
| 8 | 72109667 | 72274467 | EYA1 | | EYA1 | EYA1 | | |
| 8 | 72753776 | 72968547 | MSC | | | | | |
| 8 | 72933485 | 72987819 | TRPA1 | | | | | |
| 8 | 73114986 | 73163869 | LOC392232 | | | | | |
| 8 | 73449625 | 73850584 | KCNB2 | | | KCNB2 | | |
| 8 | 73644279 | 73664078 | LOC101926908 | | | | | |
| 8 | 73921096 | 73959987 | TERF1 | | | | | |
| 8 | 73976777 | 74005507 | SBSPON | | | | | |
| 8 | 74153658 | 74171737 | C8orf89 | | | | | |
| 8 | 74202873 | 74205869 | RPL7 | | | | | |
| 8 | 74206836 | 74268696 | RDH10 | | | | | |
| 8 | 74332308 | 74659943 | STAU2 | | | STAU2 | | |

| | | | | | | | | | |
|---|---|---|---|---|---|---|---|---|---|
| 8 | 74692331 | 74791145 | UBE2W | | | | | | |
| 8 | 74857372 | 74884522 | TCEB1 | | | | | | |
| 8 | 74888376 | 74895018 | TMEM70 | | | | | | |
| 8 | 74903563 | 74941307 | LY96 | | | | | | |
| 8 | 75146934 | 75233596 | JPH1 | | | | | | |
| 8 | 75262617 | 75279335 | GDAP1 | | GDAP1 | GDAP1 | | | |
| 8 | 75460777 | 75460852 | MIR5681A | | | | | | |
| 8 | 75460784 | 75460844 | MIR5681B | | | | | | |
| 8 | 75512100 | 75670587 | MIR2052HG | | | | | | |
| 8 | 75617927 | 75617982 | MIR2052 | | | | | | |
| 8 | 75736771 | 75767264 | PI15 | | | | | | |
| 8 | 75896707 | 75946793 | CRISPLD1 | | | | | | |
| 8 | 76135351 | 76191124 | CASC9 | | | | | | |
| 8 | 76452202 | 76479061 | HNF4G | | | | | | |
| 8 | 77318888 | 77436567 | LINC01111 | | | | | | |
| 8 | 77523113 | 77779521 | ZFHX4 | | | | | | |
| 8 | 77879003 | 77879086 | MIR3149 | | | | | | |
| 8 | 77892493 | 77913280 | PEX2 | | | | | | |
| 8 | 78362932 | 78388567 | LOC102724874 | | | | | | |
| 8 | 79428335 | 79470738 | PKIA | | | PKIA | | | |
| 8 | 79578281 | 79631997 | ZC2HC1A | | | | | | |
| 8 | 79635082 | 79636609 | LOC101241902 | | | | | | |
| 8 | 79645006 | 79717758 | IL7 | | | | | | |
| 8 | 80523048 | 80578410 | STMN2 | | | | | | |
| 8 | 80676244 | 80680098 | HEY1 | | | | | | |
| 8 | 80680376 | 80715077 | LINC01607 | | | | | | |
| 8 | 80681614 | 80783972 | LOC101927040 | | | | | | |
| 8 | 80831094 | 80942506 | MRPS28 | | | | | | |
| 8 | 80947102 | 81083894 | TPD52 | | | | | | |
| 8 | 81153623 | 81153708 | MIR5708 | | | | | | |
| 8 | 81397853 | 81438500 | ZBTB10 | | | | | | |
| 8 | 81540685 | 81787016 | ZNF704 | | | | | | |
| 8 | 81880045 | 82024303 | PAG1 | | | | | | |
| 8 | 82192717 | 82197012 | FABP5 | | | | | | |
| 8 | 82352563 | 82359719 | PMP2 | | | | | | |
| 8 | 82370617 | 82373758 | FABP9 | | | | | | |
| 8 | 82390731 | 82395473 | FABP4 | | | | | | |
| 8 | 82437280 | 82443550 | FABP12 | | | | | | |
| 8 | 82569150 | 82598589 | IMPA1 | | IMPA1 | IMPA1 | | | |
| 8 | 82605890 | 82607207 | SLC10A5 | | | | | | |
| 8 | 82613565 | 82633539 | ZFAND1 | | | | | | |
| 8 | 82644687 | 82671748 | CHMP4C | | | | | | |
| 8 | 82711817 | 82754521 | SNX16 | | | | | | |
| 8 | 83824338 | 83874159 | LOC101927141 | | | | | | |
| 8 | 84315992 | 84321132 | LINC01419 | | | | | | |
| 8 | 85095452 | 85834078 | RALYL | | | | | | |
| 8 | 86019322 | 86058314 | LRRCC1 | | | | | | |
| 8 | 86084311 | 86089276 | LOC102723322 | | | | | | |
| 8 | 86089618 | 86126753 | E2F5 | | | | | | |
| 8 | 86126287 | 86132651 | C8orf59 | | | | | | |
| 8 | 86157715 | 86196302 | CA13 | | | | | | |
| 8 | 86240457 | 86290383 | CA1 | | | | | | |
| 8 | 86351055 | 86375283 | CA3 | | | | | | |
| 8 | 86376130 | 86393721 | CA2 | | CA2 | | | | |
| 8 | 86566827 | 86840171 | REXO1L2P | | | | | | |
| 8 | 87060690 | 87081851 | PSKH2 | | | | | | |
| 8 | 87111138 | 87166454 | ATP6V0D2 | | | | | | |
| 8 | 87226287 | 87242604 | SLC7A13 | | | | | | |
| 8 | 87354993 | 87480178 | WWP1 | | | | | | |
| 8 | 87484577 | 87526577 | RMDN1 | | | | | | |
| 8 | 87526655 | 87573726 | CPNE3 | | | | | | |
| 8 | 87586162 | 87755903 | CNGB3 | | | CNGB3 | | | |
| 8 | 87878675 | 88394955 | CNBD1 | | | | | | |
| 8 | 88882970 | 88886296 | DCAF4L2 | | | | | | |
| 8 | 89049459 | 89339717 | MMP16 | | | | | | |
| 8 | 90729626 | 90769939 | LOC101929709 | | | | | | |

| | | | | | | | | | |
|---|---|---|---|---|---|---|---|---|---|
| 8 | 90769974 | 90803292 | RIPK2 | | | RIPK2 | | | |
| 8 | 90914095 | 90940115 | OSGIN2 | | | | | | |
| 8 | 90945563 | 90996952 | NBN | | | | | | |
| 8 | 91013579 | 91064227 | DECR1 | | | DECR1 | | | |
| 8 | 91070835 | 91095109 | CALB1 | | | | | | |
| 8 | 91233715 | 91400187 | LINC00534 | | | | | | |
| 8 | 91605002 | 91618291 | LINC01030 | | | | | | |
| 8 | 91634222 | 91658133 | TMEM64 | | | | | | |
| 8 | 91803920 | 91971630 | NECAB1 | | | | | | |
| 8 | 91970705 | 91997485 | C8orf88 | | | | | | |
| 8 | 92006501 | 92053203 | TMEM55A | | | | | | |
| 8 | 92072133 | 92099323 | OTUD6B | | | | | | |
| 8 | 92114846 | 92231464 | LRRC69 | | | | | | |
| 8 | 92217712 | 92217786 | MIR4661 | | | | | | |
| 8 | 92221721 | 92410382 | SLC26A7 | | | | | | |
| 8 | 92967194 | 93115454 | RUNX1T1 | | | | | | |
| 8 | 93179689 | 93179739 | MIR7641 | | | | | | |
| 8 | 93577670 | 93667722 | LOC102724710 | | | | | | |
| 8 | 93725189 | 93798288 | FLJ46284 | | | | | | |
| 8 | 93895757 | 93978372 | TRIQK | | | | | | |
| 8 | 94041978 | 94042067 | MIR8084 | | | | | | |
| 8 | 94146323 | 94179079 | C8orf87 | | | | | | |
| 8 | 94358694 | 94712661 | LINC00535 | | | | | | |
| 8 | 94712734 | 94741478 | FAM92A1 | | | | | | |
| 8 | 94743730 | 94753047 | RBM12B | | | | | | |
| 8 | 94767071 | 94831460 | TMEM67 | | | | | | |
| 8 | 94928249 | 94928347 | MIR378D2 | | | | | | |
| 8 | 94929082 | 94938296 | PDP1 | | | | | | |
| 8 | 95139393 | 95229531 | CDH17 | | | | | | |
| 8 | 95261484 | 95274547 | GEM | | | | | | |
| 8 | 95384187 | 95487343 | RAD54B | | | | | | |
| 8 | 95439939 | 95449180 | FSBP | | | | | | |
| 8 | 95439939 | 95487343 | RAD54B | | | | | | |
| 8 | 95500004 | 95565746 | KIAA1429 | | | | | | |
| 8 | 95649512 | 95651695 | LOC100288748 | | | | | | |
| 8 | 95653363 | 95719694 | ESRP1 | | | | | | |
| 8 | 95732102 | 95806076 | DPY19L4 | | | | | | |
| 8 | 95835517 | 95892721 | INTS8 | | | | | | |
| 8 | 95892452 | 95907482 | CCNE2 | | CCNE2 | | | | |
| 8 | 95938199 | 95961615 | TP53INP1 | | | | | | |
| 8 | 96037213 | 96070944 | NDUFAF6 | | | | | | |
| 8 | 96079035 | 96085410 | LOC105375650 | | | | | | |
| 8 | 96085138 | 96085224 | MIR3150B | | | | | | |
| 8 | 96085141 | 96085221 | MIR3150A | | | | | | |
| 8 | 96145948 | 96168913 | PLEKHF2 | | | | | | |
| 8 | 96219234 | 96228602 | LINC01298 | | | | | | |
| 8 | 96257140 | 96822371 | C8orf37 | | | | | | |
| 8 | 96959212 | 96960576 | LOC100500773 | | | | | | |
| 8 | 97154557 | 97173020 | GDF6 | | | | | | |
| 8 | 97238903 | 97247862 | UQCRB | | | | | | |
| 8 | 97251625 | 97273841 | MTERF3 | | | | | | |
| 8 | 97274113 | 97346779 | PTDSS1 | | | | | | |
| 8 | 97384153 | 97399666 | LOC102724804 | | | | | | |
| 8 | 97505881 | 97624037 | SDC2 | | | | | | |
| 8 | 97657454 | 98155731 | CPQ | | | | | | |
| 8 | 97964091 | 98458725 | LOC101927066 | | | | | | |
| 8 | 98285713 | 98290176 | TSPYL5 | | | | | | |
| 8 | 98656406 | 98742488 | MTDH | | | | | | |
| 8 | 98787808 | 98864830 | LAPTM4B | | | | | | |
| 8 | 98881248 | 99048946 | MATN2 | | | MATN2 | | | |
| 8 | 99053937 | 99057818 | RPL30 | | | | | | |
| 8 | 99054313 | 99054445 | SNORA72 | | | | | | |
| 8 | 99076749 | 99105838 | ERICH5 | | | | | | |
| 8 | 99114566 | 99129418 | RIDA | | | | | | |
| 8 | 99129520 | 99172069 | POP1 | | | | | | |
| 8 | 99204386 | 99306621 | NIPAL2 | | | | | | |

| | | | | | | | | |
|---|---|---|---|---|---|---|---|---|
| 8 | 99439249 | 99443023 | KCNS2 | | | KCNS2 | | |
| 8 | 99466858 | 99954799 | STK3 | | | STK3 | | |
| 8 | 99956630 | 99964332 | OSR2 | | | | | |
| 8 | 100025493 | 100889814 | VPS13B | | | VPS13B | | |
| 8 | 100548863 | 100548958 | MIR599 | | | | | |
| 8 | 100549013 | 100549089 | MIR875 | | | | | |
| 8 | 100890222 | 100906242 | COX6C | | | | | |
| 8 | 100973165 | 101118344 | RGS22 | | | | | |
| 8 | 101036209 | 101036312 | MIR1273A | | | | | |
| 8 | 101145587 | 101158099 | FBXO43 | | | | | |
| 8 | 101162838 | 101166230 | POLR2K | | | | | |
| 8 | 101170262 | 101254132 | SPAG1 | | | | | |
| 8 | 101269286 | 101348446 | RNF19A | | | RNF19A | | |
| 8 | 101394990 | 101395073 | MIR4471 | | | | | |
| 8 | 101521979 | 101572014 | ANKRD46 | | | | | |
| 8 | 101585111 | 101661893 | SNX31 | | | | | |
| 8 | 101715143 | 101734315 | PABPC1 | | | | | |
| 8 | 101715195 | 101715252 | MIR7705 | | | | | |
| 8 | 101930803 | 101965623 | YWHAZ | | | YWHAZ | | |
| 8 | 102064281 | 102088479 | FLJ42969 | | | | | |
| 8 | 102209265 | 102218292 | ZNF706 | | | | | |
| 8 | 102381120 | 102381823 | NACAP1 | | | | | |
| 8 | 102504667 | 102681952 | GRHL2 | | | | | |
| 8 | 102698769 | 103137135 | NCALD | | | NCALD | | |
| 8 | 102996659 | 103007078 | LOC104054148 | | | | | |
| 8 | 103137659 | 103137743 | MIR5680 | | | | | |
| 8 | 103216728 | 103251346 | RRM2B | | | | | |
| 8 | 103251678 | 103424917 | UBR5 | | | | | |
| 8 | 103563847 | 103573245 | ODF1 | | | | | |
| 8 | 103661004 | 103668130 | KLF10 | | | | | |
| 8 | 103838529 | 103990104 | AZIN1 | | | | | |
| 8 | 104033247 | 104085285 | ATP6V1C1 | | | | | |
| 8 | 104145190 | 104242533 | BAALC | | | | | |
| 8 | 104166841 | 104166917 | MIR3151 | | | | | |
| 8 | 104169217 | 104184368 | BAALC | | | | | |
| 8 | 104310660 | 104345094 | FZD6 | | | | | |
| 8 | 104383742 | 104395232 | CTHRC1 | | | | | |
| 8 | 104410865 | 104427563 | SLC25A32 | | | | | |
| 8 | 104426941 | 104455680 | DCAF13 | | | | | |
| 8 | 104512975 | 105266653 | RIMS2 | | | RIMS2 | | |
| 8 | 105352023 | 105368917 | DCSTAMP | | | | | |
| 8 | 105391651 | 105479277 | DPYS | | DPYS | | | |
| 8 | 105501458 | 105601252 | LRP12 | | | LRP12 | | |
| 8 | 106331146 | 107072731 | ZFPM2 | | | ZFPM2 | | |
| 8 | 107282405 | 107764921 | OXR1 | | | | | |
| 8 | 107771710 | 107782472 | ABRA | | | | | |
| 8 | 108261709 | 108510283 | ANGPT1 | | | | | |
| 8 | 108911543 | 109095913 | RSPO2 | | | | | |
| 8 | 109213971 | 109260959 | EIF3E | | | | | |
| 8 | 109455852 | 109499136 | EMC2 | | | | | |
| 8 | 109795345 | 109799770 | TMEM74 | | | | | |
| 8 | 110099652 | 110131812 | TRHR | | | TRHR | | |
| 8 | 110253147 | 110346350 | NUDCD1 | | | | | |
| 8 | 110346551 | 110358189 | ENY2 | | | | | |
| 8 | 110374705 | 110543500 | PKHD1L1 | | | | | |
| 8 | 110551928 | 110578225 | EBAG9 | | | | | |
| 8 | 110586404 | 110704020 | SYBU | | | | | |
| 8 | 110656343 | 110660313 | LOC100132813 | | | | | |
| 8 | 110979232 | 110986959 | KCNV1 | | | | | |
| 8 | 111949918 | 112039662 | LINC01608 | | | | | |
| 8 | 112111189 | 112248432 | LINC01609 | | | | | |
| 8 | 113235158 | 114449242 | CSMD3 | | | CSMD3 | | |
| 8 | 113655721 | 113655812 | MIR2053 | | | | | |
| 8 | 116420723 | 116681255 | TRPS1 | | | TRPS1 | | |
| 8 | 116962735 | 117337297 | LINC00536 | | | | | |
| 8 | 117657054 | 117768062 | EIF3H | | | | | |

| | | | | | | | | |
|---|---|---|---|---|---|---|---|---|
| 8 | 117778741 | 117786921 | UTP23 | | | | | |
| 8 | 117858172 | 117889107 | RAD21 | | | | | |
| 8 | 117886966 | 117887039 | MIR3610 | | | | | |
| 8 | 117950463 | 117956239 | AARD | | | | | |
| 8 | 117962511 | 118188953 | SLC30A8 | | | | | |
| 8 | 118532951 | 118552501 | MED30 | | | | | |
| 8 | 118811601 | 119124058 | EXT1 | | | EXT1 | | |
| 8 | 119201694 | 119738306 | SAMD12 | | | | | |
| 8 | 119935795 | 119964383 | TNFRSF11B | | | | | |
| 8 | 120075181 | 120081021 | LOC101927513 | | | | | |
| 8 | 120079423 | 120119202 | COLEC10 | | | | | |
| 8 | 120220609 | 120257914 | MAL2 | | | | | |
| 8 | 120428551 | 120436678 | NOV | | | | | |
| 8 | 120569316 | 120651106 | ENPP2 | | ENPP2 | | | |
| 8 | 120743013 | 120845074 | TAF2 | | | | | |
| 8 | 120846180 | 120868170 | DSCC1 | | | | | |
| 8 | 120885894 | 121063157 | DEPTOR | | | | | |
| 8 | 121137346 | 121384273 | COL14A1 | | | | | |
| 8 | 121408082 | 121457647 | MRPL13 | | | MRPL13 | | |
| 8 | 121457637 | 121535875 | MTBP | | | | | |
| 8 | 121547984 | 121824309 | SNTB1 | | | SNTB1 | | |
| 8 | 121773492 | 121789072 | LOC101927543 | | | | | |
| 8 | 122625270 | 122657564 | HAS2 | | | | | |
| 8 | 123426565 | 123580883 | LOC105375734 | | | | | |
| 8 | 123682623 | 123706345 | LINC01151 | | | | | |
| 8 | 123793900 | 123986755 | ZHX2 | | | ZHX2 | | |
| 8 | 124025403 | 124054663 | DERL1 | | | | | |
| 8 | 124084919 | 124164392 | TBC1D31 | | | | | |
| 8 | 124191286 | 124214983 | FAM83A | | | | | |
| 8 | 124228027 | 124228103 | MIR4663 | | | | | |
| 8 | 124232195 | 124253638 | C8orf76 | | | | | |
| 8 | 124238428 | 124286727 | ZHX1-C8orf76 | | | | | |
| 8 | 124260689 | 124287781 | ZHX1 | | | | | |
| 8 | 124332090 | 124408705 | ATAD2 | | | | | |
| 8 | 124428964 | 124460330 | WDYHV1 | | | | | |
| 8 | 124510126 | 124553493 | FBXO32 | | | | | |
| 8 | 124657914 | 124665190 | KLHL38 | | | | | |
| 8 | 124693033 | 124749666 | ANXA13 | | | | | |
| 8 | 124780678 | 124827690 | FAM91A1 | | | | | |
| 8 | 124864226 | 125183763 | FER1L6 | | | | | |
| 8 | 125204931 | 125259639 | LOC101927588 | | | | | |
| 8 | 125323158 | 125384940 | TMEM65 | | | | | |
| 8 | 125463047 | 125465266 | TRMT12 | | | | | |
| 8 | 125474737 | 125500859 | RNF139 | | | | | |
| 8 | 125500734 | 125551329 | TATDN1 | | | | | |
| 8 | 125520755 | 125520817 | MIR6844 | | | | | |
| 8 | 125551342 | 125562227 | NDUFB9 | | | NDUFB9 | | |
| 8 | 125563010 | 125740748 | MTSS1 | | | MTSS1 | | |
| 8 | 125834219 | 125834300 | MIR4662B | | | | | |
| 8 | 125834226 | 125834293 | MIR4662A | | | | | |
| 8 | 125954249 | 125963337 | LINC00964 | | | | | |
| 8 | 125985538 | 125991630 | ZNF572 | | | | | |
| 8 | 126009230 | 126010440 | LOC105375744 | | | | | |
| 8 | 126010719 | 126034525 | SQLE | | | | | |
| 8 | 126036502 | 126104061 | KIAA0196 | | | | | |
| 8 | 126104082 | 126379367 | NSMCE2 | | | | | |
| 8 | 126442562 | 126450647 | TRIB1 | | | | | |
| 8 | 126934766 | 126963441 | LINC00861 | | | | | |
| 8 | 127337739 | 127341778 | LOC101927657 | | | | | |
| 8 | 127564682 | 127570711 | FAM84B | | | | | |
| 8 | 128025398 | 128033259 | PCAT1 | | | | | |
| 8 | 128084938 | 128094466 | PCAT2 | | | | | |
| 8 | 128092118 | 128104840 | PRNCR1 | | | | | |
| 8 | 128200030 | 128209872 | CASC19 | | | | | |
| 8 | 128219626 | 128231513 | CCAT1 | | | | | |
| 8 | 128256881 | 128404876 | CASC21 | | | | | |

| | | | | | | | | |
|---|---|---|---|---|---|---|---|---|
| 8 | 128301920 | 128494384 | CASC8 | | | | | |
| 8 | 128412643 | 128414395 | CCAT2 | | | | | |
| 8 | 128427856 | 128429441 | POU5F1B | | | | | |
| 8 | 128455594 | 128494384 | CASC8 | | | | | |
| 8 | 128712852 | 128746213 | CASC11 | | | | | |
| 8 | 128748314 | 128753680 | MYC | | | | | |
| 8 | 128806778 | 129113499 | PVT1 | | | | | |
| 8 | 128808207 | 128808274 | MIR1204 | | | | | |
| 8 | 128958804 | 128960969 | TMEM75 | | | | | |
| 8 | 128972878 | 128972941 | MIR1205 | | | | | |
| 8 | 129021143 | 129021202 | MIR1206 | | | | | |
| 8 | 129061397 | 129061484 | MIR1207 | | | | | |
| 8 | 129162361 | 129162434 | MIR1208 | | | | | |
| 8 | 129417515 | 129576925 | LINC00824 | | | | | |
| 8 | 130228712 | 130253486 | LINC00977 | | | | | |
| 8 | 130363939 | 130692485 | CCDC26 | | | | | |
| 8 | 130496302 | 130496388 | MIR3686 | | | | | |
| 8 | 130760441 | 130799134 | GSDMC | | | | | |
| 8 | 130851838 | 131028897 | FAM49B | | | | | |
| 8 | 131020579 | 131020699 | MIR5194 | | | | | |
| 8 | 131064350 | 131455906 | ASAP1 | | | | | |
| 8 | 131094983 | 131097014 | ASAP1-IT2 | | | | | |
| 8 | 131307600 | 131308779 | ASAP1-IT1 | | | | | |
| 8 | 131792546 | 132052835 | ADCY8 | | | | | |
| 8 | 132916355 | 133025886 | EFR3A | | | | | |
| 8 | 133036466 | 133071627 | OC90 | | | | | |
| 8 | 133073732 | 133117512 | HHLA1 | | | | | |
| 8 | 133133104 | 133493004 | KCNQ3 | | KCNQ3 | KCNQ3 | KCNQ3 | |
| 8 | 133572744 | 133573726 | HPYR1 | | | | | |
| 8 | 133584200 | 133687863 | LRRC6 | | | | | |
| 8 | 133722191 | 133772914 | TMEM71 | | | | | |
| 8 | 133787603 | 133861052 | PHF20L1 | | | | | |
| 8 | 133879204 | 134147143 | TG | | | | | |
| 8 | 134048972 | 134115310 | SLA | | | SLA | | |
| 8 | 134058725 | 134058826 | MIR7848 | | | | | |
| 8 | 134203281 | 134243932 | WISP1 | | | | | |
| 8 | 134249413 | 134309547 | NDRG1 | | | | | |
| 8 | 134467090 | 134584183 | ST3GAL1 | | | | | |
| 8 | 134676003 | 134696309 | LOC105375773 | | | | | |
| 8 | 134787793 | 134789595 | LOC101927798 | | | | | |
| 8 | 134898738 | 134914650 | LOC101927822 | | | | | |
| 8 | 135490030 | 135612932 | ZFAT | | | | | |
| 8 | 135812762 | 135812850 | MIR30B | | | | | |
| 8 | 135817118 | 135817188 | MIR30D | | | | | |
| 8 | 135850311 | 135854880 | NCRNA00250 | | | | | |
| 8 | 135862177 | 135894142 | LOC101927845 | | | | | |
| 8 | 136246373 | 136311962 | LINC01591 | | | | | |
| 8 | 136469707 | 136659852 | KHDRBS3 | | | | | |
| 8 | 138418343 | 138425831 | LOC101927915 | | | | | |
| 8 | 139142265 | 139509065 | FAM135B | | | | | |
| 8 | 139600477 | 139926249 | COL22A1 | | | | | |
| 8 | 140613080 | 140715299 | KCNK9 | | KCNK9 | | | |
| 8 | 140742585 | 141468678 | TRAPPC9 | | | | | |
| 8 | 141521396 | 141527252 | CHRAC1 | | | | | |
| 8 | 141541263 | 141645646 | AGO2 | | | | | |
| 8 | 141668480 | 142011412 | PTK2 | | PTK2 | | | |
| 8 | 142138719 | 142205900 | DENND3 | | | | | |
| 8 | 142217264 | 142238673 | SLC45A4 | | | | | |
| 8 | 142264665 | 142266916 | LOC105375787 | | | | | |
| 8 | 142350647 | 142354720 | LINC01300 | | | | | |
| 8 | 142366586 | 142377365 | GPR20 | | | | | |
| 8 | 142431487 | 142442554 | PTP4A3 | | | | | |
| 8 | 142443928 | 142517330 | MROH5 | | | | | |
| 8 | 142867602 | 142867674 | MIR1302 | | | | | |
| 8 | 143079636 | 143079657 | MIR4539 | | | | | |
| 8 | 143257699 | 143257779 | MIR4472 | | | | | |

| | | | | | | | | | |
|---|---|---|---|---|---|---|---|---|---|
| 8 | 143279716 | 143290364 | LINC00051 | | | | | | |
| 8 | 143293440 | 143484610 | TSNARE1 | | | TSNARE1 | | | |
| 8 | 143545376 | 143626368 | ADGRB1 | | | | | | |
| 8 | 143692404 | 143695833 | ARC | | | | | | |
| 8 | 143719948 | 143722024 | LOC101928087 | | | | | | |
| 8 | 143738873 | 143751412 | JRK | | | | | | |
| 8 | 143751725 | 143764145 | PSCA | | | | | | |
| 8 | 143781528 | 143785584 | LY6K | | | | | | |
| 8 | 143783669 | 143808391 | LOC100288181 | | | | | | |
| 8 | 143808620 | 143818350 | THEM6 | | | | | | |
| 8 | 143822361 | 143823829 | SLURP1 | | | | | | |
| 8 | 143831627 | 143833952 | LYPD2 | | | | | | |
| 8 | 143845751 | 143859264 | LYNX1 | | | | | | |
| 8 | 143866297 | 143868008 | LY6D | | | | | | |
| 8 | 143916216 | 143928262 | GML | | | | | | |
| 8 | 143953772 | 143961236 | CYP11B1 | | | | | | |
| 8 | 143991974 | 143999259 | CYP11B2 | | | | | | |
| 8 | 144063447 | 144099807 | LOC100133669 | | | | | | |
| 8 | 144077244 | 144079080 | CDC42P3 | | | | | | |
| 8 | 144099901 | 144103827 | LY6E | | | | | | |
| 8 | 144120625 | 144135720 | C8orf31 | | | | | | |
| 8 | 144239330 | 144242053 | LY6H | | | | | | |
| 8 | 144295067 | 144299044 | GPIHBP1 | | GPIHBP1 | | | | |
| 8 | 144328990 | 144344875 | ZFP41 | | | | | | |
| 8 | 144349606 | 144359101 | GLI4 | | | | | | |
| 8 | 144362324 | 144363870 | MINCR | | | | | | |
| 8 | 144373558 | 144382120 | ZNF696 | | | | | | |
| 8 | 144391496 | 144442147 | TOP1MT | | | TOP1MT | | | |
| 8 | 144448792 | 144466390 | RHPN1 | | | | | | |
| 8 | 144499848 | 144512602 | MAFA | | | | | | |
| 8 | 144519824 | 144623620 | ZC3H3 | | | | | | |
| 8 | 144635556 | 144645231 | GSDMD | | | | | | |
| 8 | 144648362 | 144654928 | MROH6 | | | | | | |
| 8 | 144656954 | 144660521 | NAPRT | | | | | | |
| 8 | 144661866 | 144679845 | EEF1D | | | | | | |
| 8 | 144680073 | 144682485 | TIGD5 | | | | | | |
| 8 | 144686082 | 144691784 | PYCRL | | | | | | |
| 8 | 144694787 | 144699732 | TSTA3 | | | | | | |
| 8 | 144718182 | 144735900 | ZNF623 | | | | | | |
| 8 | 144766621 | 144777555 | ZNF707 | | | | | | |
| 8 | 144779284 | 144780583 | BREA2 | | | | | | |
| 8 | 144788863 | 144790279 | CCDC166 | | | | | | |
| 8 | 144790220 | 144796068 | LOC101928160 | | | | | | |
| 8 | 144798506 | 144804633 | MAPK15 | | | | | | |
| 8 | 144806102 | 144815914 | FAM83H | | | | | | |
| 8 | 144815252 | 144815323 | MIR4664 | | | | | | |
| 8 | 144816309 | 144828507 | FAM83H | | | | | | |
| 8 | 144873089 | 144897549 | SCRIB | | | | | | |
| 8 | 144895126 | 144895212 | MIR937 | | | | | | |
| 8 | 144898513 | 144911556 | PUF60 | | | | | | |
| 8 | 144915754 | 144923146 | NRBP2 | | | | | | |
| 8 | 144919927 | 144919988 | MIR6845 | | | | | | |
| 8 | 144939491 | 144952632 | EPPK1 | | | | | | |
| 8 | 144989314 | 145050913 | PLEC | | | | | | |
| 8 | 145019358 | 145019447 | MIR661 | | | | | | |
| 8 | 145051319 | 145060639 | PARP10 | | | | | | |
| 8 | 145064225 | 145067583 | GRINA | | | | | | |
| 8 | 145086581 | 145102015 | SPATC1 | | | | | | |
| 8 | 145106166 | 145115606 | OPLAH | | | | | | |
| 8 | 145112223 | 145112283 | MIR6846 | | | | | | |
| 8 | 145133521 | 145135551 | EXOSC4 | | | | | | |
| 8 | 145134776 | 145134845 | MIR6847 | | | | | | |
| 8 | 145137523 | 145141119 | GPAA1 | | | | | | |
| 8 | 145149937 | 145152430 | CYC1 | | | CYC1 | | | |
| 8 | 145153535 | 145159138 | SHARPIN | | | | | | |
| 8 | 145159304 | 145162515 | MAF1 | | | | | | |

| | | | | | | | | |
|---|---|---|---|---|---|---|---|---|
| 8 | 145162594 | 145171286 | WDR97 | | | | | |
| 8 | 145192671 | 145195746 | HGH1 | | | | | |
| 8 | 145202918 | 145316843 | MROH1 | | | | | |
| 8 | 145317575 | 145317640 | MIR7112 | | | | | |
| 8 | 145321460 | 145323386 | SCX | | | | | |
| 8 | 145437879 | 145440954 | HGH1 | | | | | |
| 8 | 145486001 | 145515120 | BOP1 | | | | | |
| 8 | 145486628 | 145486693 | MIR7112 | | | | | |
| 8 | 145490546 | 145492472 | SCX | | | | | |
| 8 | 145515269 | 145538385 | HSF1 | | | | | |
| 8 | 145538245 | 145550582 | DGAT1 | | | | | |
| 8 | 145540908 | 145540978 | MIR6848 | | | | | |
| 8 | 145554227 | 145559943 | SCRT1 | | | | | |
| 8 | 145576885 | 145579269 | TMEM249 | | | | | |
| 8 | 145579087 | 145582183 | FBXL6 | | | | | |
| 8 | 145582216 | 145584948 | SLC52A2 | | | | | |
| 8 | 145592595 | 145596648 | LOC101928902 | | | | | |
| 8 | 145597703 | 145618453 | ADCK5 | | | | | |
| 8 | 145618445 | 145634733 | CPSF1 | | | | | |
| 8 | 145619363 | 145619445 | MIR939 | | | | | |
| 8 | 145625670 | 145625739 | MIR6849 | | | | | |
| 8 | 145637797 | 145642279 | SLC39A4 | | | | | |
| 8 | 145648983 | 145653946 | VPS28 | | | | | |
| 8 | 145654162 | 145665276 | TONSL | | | | | |
| 8 | 145660933 | 145661002 | MIR6893 | | | | | |
| 8 | 145675314 | 145691031 | CYHR1 | | | | | |
| 8 | 145691719 | 145699499 | KIFC2 | | | | | |
| 8 | 145699114 | 145701718 | FOXH1 | | | | | |
| 8 | 145722108 | 145727504 | PPP1R16A | | | | | |
| 8 | 145729464 | 145732555 | GPT | | | | | |
| 8 | 145734418 | 145736611 | MFSD3 | | | | | |
| 8 | 145736666 | 145743210 | RECQL4 | | | | | |
| 8 | 145743348 | 145750559 | LRRC14 | | | | | |
| 8 | 145747760 | 145752416 | LRRC24 | | | | | |
| 8 | 145751602 | 145754458 | C8orf82 | | | | | |
| 8 | 145754562 | 145911197 | ARHGAP39 | | | | | |
| 8 | 145946293 | 145980970 | ZNF251 | | | | | |
| 8 | 145997608 | 146012730 | ZNF34 | | | | | |
| 8 | 146015149 | 146017831 | RPL8 | | | | | |
| 8 | 146017315 | 146017376 | MIR6850 | | | | | |
| 8 | 146024260 | 146036139 | ZNF517 | | | | | |
| 8 | 146052902 | 146072893 | ZNF7 | | | | | |
| 8 | 146075550 | 146078963 | COMMD5 | | | | | |
| 8 | 146102335 | 146126846 | ZNF250 | | | | | |
| 8 | 146155743 | 146176274 | ZNF16 | | | | | |
| 8 | 146198974 | 146228285 | ZNF252P | | | | | |
| 8 | 146220250 | 146224283 | TMED10P1 | | | | | |
| 8 | 146228196 | 146231432 | ZNF252P | | | | | |
| 8 | 146277823 | 146281416 | C8orf33 | | | | | |
| 9 | 11986 | 14525 | DDX11L5 | | | | | |
| 9 | 14510 | 29739 | WASH1 | | | | | |
| 9 | 30143 | 30281 | MIR1302 | | | | | |
| 9 | 34393 | 35864 | FAM138C | | | | | |
| 9 | 72689 | 88826 | PGM5P3 | | | | | |
| 9 | 116230 | 118417 | FOXD4 | | | | | |
| 9 | 121037 | 179075 | CBWD1 | | | | | |
| 9 | 213107 | 215893 | C9orf66 | | | | | |
| 9 | 214864 | 465259 | DOCK8 | | | | | |
| 9 | 470293 | 746106 | KANK1 | | | | | |
| 9 | 841689 | 969090 | DMRT1 | | | | | |
| 9 | 976967 | 991732 | DMRT3 | | | | | |
| 9 | 1048142 | 1048640 | LINC01230 | | | | | |
| 9 | 1050345 | 1057554 | DMRT2 | | | | | |
| 9 | 2015218 | 2193623 | SMARCA2 | | | | | |
| 9 | 2535654 | 2654485 | VLDLR | | | VLDLR | | |
| 9 | 2717525 | 2730037 | KCNV2 | | | | | |

| | | | | | | | | |
|---|---|---|---|---|---|---|---|---|
| 9 | 2804154 | 2844130 | PUM3 | | | | | |
| 9 | 3181588 | 3200500 | LINC01231 | | | | | |
| 9 | 3218296 | 3691756 | RFX3 | | | RFX3 | | |
| 9 | 3824127 | 3901248 | GLIS3 | | | | | |
| 9 | 4490426 | 4587469 | SLC1A1 | | SLC1A1 | SLC1A1 | SLC1A1 | |
| 9 | 4598315 | 4666674 | SPATA6L | | | | | |
| 9 | 4662293 | 4665272 | PLPP6 | | | | | |
| 9 | 4676600 | 4708398 | CDC37L1 | | | | | |
| 9 | 4709556 | 4742043 | AK3 | | | | | |
| 9 | 4792833 | 4861077 | RCL1 | | | | | |
| 9 | 4850296 | 4850375 | MIR101 | | | | | |
| 9 | 4985244 | 5128183 | JAK2 | | | | | |
| 9 | 5163862 | 5185618 | INSL6 | | | | | |
| 9 | 5231418 | 5233967 | INSL4 | | | | | |
| 9 | 5299865 | 5304611 | RLN2 | | | | | |
| 9 | 5334931 | 5339873 | RLN1 | | | | | |
| 9 | 5357966 | 5437937 | PLGRKT | | | | | |
| 9 | 5450502 | 5470567 | CD274 | | | | | |
| 9 | 5510544 | 5571282 | PDCD1LG2 | | | | | |
| 9 | 5629118 | 5776556 | RIC1 | | | | | |
| 9 | 5784571 | 5833081 | ERMP1 | | | | | |
| 9 | 5890908 | 5909822 | MLANA | | | | | |
| 9 | 5919007 | 6008003 | KIAA2026 | | | | | |
| 9 | 6007825 | 6007904 | MIR4665 | | | | | |
| 9 | 6011018 | 6015640 | RANBP6 | | | | | |
| 9 | 6215148 | 6257983 | IL33 | | | | | |
| 9 | 6328348 | 6331900 | TPD52L3 | | | | | |
| 9 | 6413150 | 6507051 | UHRF2 | | | | | |
| 9 | 6532463 | 6645692 | GLDC | | GLDC | GLDC | | |
| 9 | 6720862 | 7175648 | KDM4C | | | | | |
| 9 | 7796490 | 7799806 | TMEM261 | | | | | |
| 9 | 8314245 | 10620420 | PTPRD | PTPRD | PTPRD | | | |
| 9 | 12693385 | 12710266 | TYRP1 | | | | | |
| 9 | 12699998 | 12823059 | LURAP1L | | | | | |
| 9 | 12972555 | 12972632 | SNORD137 | | | | | |
| 9 | 13105702 | 13279563 | MPDZ | | MPDZ | MPDZ | | MPDZ |
| 9 | 13406378 | 13431328 | FLJ41200 | | | | | |
| 9 | 13927969 | 13945606 | LINC00583 | | | | | |
| 9 | 14081841 | 14398982 | NFIB | | | | | |
| 9 | 14611068 | 14693480 | ZDHHC21 | | | | | |
| 9 | 14719731 | 14722715 | CER1 | | | | | |
| 9 | 14734663 | 14910993 | FREM1 | | | | | |
| 9 | 14993324 | 15019722 | LOC389705 | | | | | |
| 9 | 15170841 | 15307358 | TTC39B | | | | | |
| 9 | 15422781 | 15461627 | SNAPC3 | | | | | |
| 9 | 15464064 | 15511017 | PSIP1 | | | | | |
| 9 | 15553096 | 15971897 | CCDC171 | | | | | |
| 9 | 16203932 | 16276311 | C9orf92 | | | | | |
| 9 | 16409500 | 16870786 | BNC2 | | | | | |
| 9 | 17134988 | 17503921 | CNTLN | | | | | |
| 9 | 17578951 | 17797126 | SH3GL2 | | | SH3GL2 | | |
| 9 | 18474078 | 18910947 | ADAMTSL1 | | | | | |
| 9 | 18573303 | 18573377 | MIR3152 | | | | | |
| 9 | 18927657 | 19049352 | SAXO1 | | | | | |
| 9 | 19049371 | 19051021 | RRAGA | | | | | |
| 9 | 19053134 | 19102940 | HAUS6 | | | | | |
| 9 | 19063653 | 19063784 | SCARNA8 | | | | | |
| 9 | 19115758 | 19127604 | PLIN2 | | | | | |
| 9 | 19230762 | 19374266 | DENND4C | | | | | |
| 9 | 19376253 | 19380235 | RPS6 | | | | | |
| 9 | 19408924 | 19452500 | ACER2 | | | | | |
| 9 | 19507449 | 19787017 | SLC24A2 | | SLC24A2 | | | |
| 9 | 20341662 | 20622542 | MLLT3 | | | | | |
| 9 | 20411145 | 20411236 | MIR4473 | | | | | |
| 9 | 20502262 | 20502340 | MIR4474 | | | | | |
| 9 | 20658307 | 20684749 | FOCAD | | | | | |

| | | | | | | | | |
|---|---|---|---|---|---|---|---|---|
| 9 | 20716103 | 20716187 | MIR491 | | | | | |
| 9 | 21006364 | 21031635 | HACD4 | | | | | |
| 9 | 21077103 | 21077962 | IFNB1 | | | | | |
| 9 | 21140630 | 21141900 | IFNW1 | | | | | |
| 9 | 21165635 | 21166659 | IFNA21 | | | | | |
| 9 | 21186617 | 21187598 | IFNA4 | | | | | |
| 9 | 21201467 | 21202204 | IFNA7 | | | | | |
| 9 | 21206179 | 21207142 | IFNA10 | | | | | |
| 9 | 21216371 | 21217310 | IFNA16 | | | | | |
| 9 | 21227241 | 21228221 | IFNA17 | | | | | |
| 9 | 21239200 | 21239978 | IFNA14 | | | | | |
| 9 | 21277686 | 21278562 | IFNA22P | | | | | |
| 9 | 21304612 | 21305312 | IFNA5 | | | | | |
| 9 | 21331017 | 21335429 | KLHL9 | | | | | |
| 9 | 21350316 | 21350886 | IFNA6 | | | | | |
| 9 | 21367370 | 21368075 | IFNA13 | | | | | |
| 9 | 21384253 | 21385396 | IFNA2 | | | | | |
| 9 | 21409145 | 21410184 | IFNA8 | | | | | |
| 9 | 21440452 | 21441315 | IFNA1 | | | | | |
| 9 | 21454266 | 21559697 | MIR31HG | | | | | |
| 9 | 21480838 | 21482312 | IFNE | | | | | |
| 9 | 21512113 | 21512184 | MIR31 | | | | | |
| 9 | 21802634 | 21865969 | MTAP | | | | | |
| 9 | 21967137 | 21994490 | CDKN2A | | | CDKN2A | | |
| 9 | 21994789 | 22009312 | CDKN2B | | | | | |
| 9 | 22446839 | 22452472 | DMRTA1 | | | | | |
| 9 | 22646198 | 22824212 | LINC01239 | | | | | |
| 9 | 23500688 | 23672397 | LOC101929563 | | | | | |
| 9 | 23690102 | 23826063 | ELAVL2 | | ELAVL2 | | | |
| 9 | 24543212 | 24545674 | IZUMO3 | | | | | |
| 9 | 25676386 | 25678856 | TUSC1 | | | | | |
| 9 | 25780053 | 25812966 | LINC01241 | | | | | |
| 9 | 26066672 | 26118406 | LOC100506422 | | | | | |
| 9 | 26840682 | 26892826 | CAAP1 | | | | | |
| 9 | 26903367 | 26947468 | PLAA | | | | | |
| 9 | 26947036 | 27062931 | IFT74 | | | | | |
| 9 | 26993134 | 27005691 | LRRC19 | | | | | |
| 9 | 27109138 | 27230176 | TEK | | | | | |
| 9 | 27245681 | 27282791 | LINC00032 | | | | | |
| 9 | 27284653 | 27297137 | EQTN | | | | | |
| 9 | 27325206 | 27529850 | MOB3B | | | | | |
| 9 | 27524311 | 27526496 | IFNK | | | | | |
| 9 | 27546543 | 27573864 | C9orf72 | | | | | |
| 9 | 27948083 | 29212998 | LINGO2 | | | | | |
| 9 | 28863623 | 28863704 | MIR876 | | | | | |
| 9 | 28888876 | 28888953 | MIR873 | | | | | |
| 9 | 30388932 | 30408452 | LINC01242 | | | | | |
| 9 | 31371608 | 31381488 | LINC01243 | | | | | |
| 9 | 32384600 | 32450832 | ACO1 | | | | | |
| 9 | 32455299 | 32526322 | DDX58 | | | | | |
| 9 | 32540541 | 32566858 | TOPORS | | | | | |
| 9 | 32553523 | 32573182 | NDUFB6 | | | NDUFB6 | | |
| 9 | 32629451 | 32635667 | TAF1L | | | | | |
| 9 | 32783496 | 32789199 | TMEM215 | | | | | |
| 9 | 32972603 | 33001639 | APTX | | | | | |
| 9 | 33025200 | 33039906 | DNAJA1 | | | | | |
| 9 | 33041849 | 33076714 | SMU1 | | | | | |
| 9 | 33110638 | 33179981 | B4GALT1 | | | | | |
| 9 | 33240195 | 33248565 | SPINK4 | | | | | |
| 9 | 33252469 | 33264759 | BAG1 | | | | | |
| 9 | 33264876 | 33282067 | CHMP5 | | | CHMP5 | | |
| 9 | 33290414 | 33365168 | NFX1 | | | | | |
| 9 | 33384947 | 33402680 | AQP7 | | | | | |
| 9 | 33441151 | 33447631 | AQP3 | | | | | |
| 9 | 33461350 | 33473941 | NOL6 | | | | | |
| 9 | 33467866 | 33467933 | MIR6851 | | | | | |

| | | | | | | | | | |
|---|---|---|---|---|---|---|---|---|---|
| 9 | 33504534 | 33511164 | SUGT1P1 | | | | | | |
| 9 | 33524410 | 33573001 | ANKRD18B | | | | | | |
| 9 | 33624222 | 33625532 | ANXA2P2 | | | | | | |
| 9 | 33673501 | 33688008 | PTENP1 | | | | | | |
| 9 | 33732972 | 33738414 | LINC01251 | | | | | | |
| 9 | 33750463 | 33799229 | PRSS3 | | | | | | |
| 9 | 33817181 | 33920401 | UBE2R2 | | | | | | |
| 9 | 33921690 | 34048947 | UBAP2 | | | | | | |
| 9 | 33934294 | 33934373 | SNORD121B | | | | | | |
| 9 | 33952761 | 33952852 | SNORD121A | | | | | | |
| 9 | 34086380 | 34126771 | DCAF12 | | | | | | |
| 9 | 34179002 | 34252521 | UBAP1 | | | | | | |
| 9 | 34252377 | 34329198 | KIF24 | | | | | | |
| 9 | 34329503 | 34343711 | NUDT2 | | | | | | |
| 9 | 34366663 | 34376894 | KIAA1161 | | | | | | |
| 9 | 34379016 | 34397849 | C9orf24 | | | | | | |
| 9 | 34398181 | 34458568 | FAM219A | | | | | | |
| 9 | 34458749 | 34520987 | DNAI1 | | | | | | |
| 9 | 34521039 | 34523037 | ENHO | | | | | | |
| 9 | 34551429 | 34583070 | CNTFR | | | | CNTFR | | |
| 9 | 34610481 | 34612110 | RPP25L | | | | | | |
| 9 | 34613541 | 34620520 | DCTN3 | | | | | | |
| 9 | 34621454 | 34628011 | ARID3C | | | | | | |
| 9 | 34634718 | 34637823 | SIGMAR1 | | | | | | |
| 9 | 34646585 | 34650595 | GALT | | | | | | |
| 9 | 34652181 | 34661898 | IL11RA | | | | | | |
| 9 | 34661879 | 34662689 | CCL27 | | | | | | |
| 9 | 34664159 | 34666053 | LOC730098 | | | | | | |
| 9 | 34689566 | 34691274 | CCL19 | | | | | | |
| 9 | 34709001 | 34710164 | CCL21 | | | | | | |
| 9 | 34723049 | 34729535 | FAM205A | | | | | | |
| 9 | 34830263 | 34838583 | FAM205BP | | | | | | |
| 9 | 34889060 | 34895775 | FAM205C | | | | | | |
| 9 | 34957483 | 34982541 | PHF24 | | | | | | |
| 9 | 34985408 | 34998430 | DNAJB5 | | | | | | |
| 9 | 35041091 | 35045988 | C9orf131 | | | | | | |
| 9 | 35056064 | 35072739 | VCP | | | | | | |
| 9 | 35073834 | 35080013 | FANCG | | | | | | |
| 9 | 35088684 | 35096598 | PIGO | | | | | | |
| 9 | 35099772 | 35103192 | STOML2 | | | | | | |
| 9 | 35104117 | 35115969 | FAM214B | | | | | | |
| 9 | 35161988 | 35405332 | UNC13B | | | | | | UNC13B |
| 9 | 35406751 | 35483026 | ATP8B5P | | | | | | |
| 9 | 35489948 | 35561895 | RUSC2 | | | | | | |
| 9 | 35561826 | 35563896 | FAM166B | | | | | | |
| 9 | 35605280 | 35610038 | TESK1 | | | | | | |
| 9 | 35608090 | 35608156 | MIR4667 | | | | | | |
| 9 | 35609975 | 35618424 | CD72 | | | | | | |
| 9 | 35646266 | 35647472 | LOC101926948 | | | | | | |
| 9 | 35649296 | 35650947 | SIT1 | | | | | | |
| 9 | 35657747 | 35658015 | RMRP | | | | | | |
| 9 | 35658286 | 35661500 | CCDC107 | | | | | | |
| 9 | 35659340 | 35665278 | ARHGEF39 | | | | | | |
| 9 | 35673914 | 35681154 | CA9 | | | | | | |
| 9 | 35681989 | 35690053 | TPM2 | | | | | | |
| 9 | 35697333 | 35732392 | TLN1 | | | | | | |
| 9 | 35710672 | 35710738 | MIR6852 | | | | | | |
| 9 | 35732316 | 35737005 | CREB3 | | | | CREB3 | | |
| 9 | 35732918 | 35732992 | MIR6853 | | | | | | |
| 9 | 35736862 | 35749225 | GBA2 | | | | | | |
| 9 | 35749276 | 35753264 | RGP1 | | | | | | |
| 9 | 35752987 | 35754274 | MSMP | | | | | | |
| 9 | 35792405 | 35809728 | NPR2 | | | | | | |
| 9 | 35807781 | 35812259 | SPAG8 | | | | | | |
| 9 | 35812956 | 35815042 | HINT2 | | | | | | |
| 9 | 35817013 | 35828744 | FAM221B | | | | | | |

| | | | | | | | | |
|---|---|---|---|---|---|---|---|---|
| 9 | 35829221 | 35854844 | TMEM8B | | | | | |
| 9 | 35860270 | 35865515 | LINC00950 | | | | | |
| 9 | 35869459 | 35870398 | OR13J1 | | | | | |
| 9 | 35906188 | 35907138 | HRCT1 | | | | | |
| 9 | 35909479 | 35911617 | LINC00961 | | | | | |
| 9 | 35957104 | 35958151 | OR2S2 | | | OR2S2 | | |
| 9 | 36036909 | 36124452 | RECK | | | | | |
| 9 | 36136532 | 36163910 | GLIPR2 | | | | | |
| 9 | 36169388 | 36171331 | CCIN | | | | | |
| 9 | 36190852 | 36212059 | CLTA | | | | | |
| 9 | 36214438 | 36277053 | GNE | | | GNE | | |
| 9 | 36336398 | 36401195 | RNF38 | | | | | |
| 9 | 36572858 | 36677680 | MELK | | | | | |
| 9 | 36823535 | 36823596 | MIR4475 | | | | | |
| 9 | 36833271 | 37034476 | PAX5 | | | | | |
| 9 | 36864250 | 36864305 | MIR4540 | | | | | |
| 9 | 36893458 | 36893528 | MIR4476 | | | | | |
| 9 | 37079892 | 37090398 | EBLN3 | | | | | |
| 9 | 37120468 | 37358145 | ZCCHC7 | | | | | |
| 9 | 37422706 | 37436986 | GRHPR | | | | | |
| 9 | 37438099 | 37465407 | ZBTB5 | | | | | |
| 9 | 37485931 | 37503694 | POLR1E | | | | | |
| 9 | 37510888 | 37576250 | FBXO10 | | | | | |
| 9 | 37588411 | 37592636 | TOMM5 | | | | | |
| 9 | 37651051 | 37746901 | FRMPD1 | | | | | |
| 9 | 37753799 | 37778969 | TRMT10B | | | | | |
| 9 | 37779710 | 37785089 | EXOSC3 | | | | | |
| 9 | 37800550 | 37867665 | DCAF10 | | | | | |
| 9 | 37877571 | 37904350 | SLC25A51 | | | | | |
| 9 | 37915894 | 38069210 | SHB | | | | | |
| 9 | 38392660 | 38398662 | ALDH1B1 | | | ALDH1B1 | | |
| 9 | 38406524 | 38424444 | IGFBPL1 | | | | | |
| 9 | 38540563 | 38545369 | FAM95C | | | | | |
| 9 | 38571360 | 38620360 | ANKRD18A | | | | | |
| 9 | 38621084 | 38623277 | FAM201A | | | | | |
| 9 | 39072763 | 39288300 | CNTNAP3 | | | CNTNAP3 | | |
| 9 | 39355665 | 39361959 | SPATA31A1 | | | | | |
| 9 | 39371061 | 39376913 | FAM74A1 | | | | | |
| 9 | 39443813 | 39464526 | ZNF658B | | | | | |
| 9 | 39884941 | 39891210 | SPATA31A1 | | | | | |
| 9 | 39900345 | 39906219 | FAM74A1 | | | | | |
| 9 | 40683818 | 40685916 | LOC105379450 | | | | | |
| 9 | 40700290 | 40706537 | SPATA31A3 | | | | | |
| 9 | 40715523 | 40722679 | FAM74A3 | | | | | |
| 9 | 40760699 | 40792116 | ZNF658 | | | | | |
| 9 | 41306132 | 41311985 | FAM74A1 | | | | | |
| 9 | 41321106 | 41327364 | SPATA31A5 | | | | | |
| 9 | 41321109 | 41506922 | SPATA31A7 | | | | | |
| 9 | 41500678 | 41506925 | SPATA31A5 | | | | | |
| 9 | 41516053 | 41521999 | FAM74A1 | | | | | |
| 9 | 41516053 | 41521999 | FAM74A6 | | | | | |
| 9 | 41588832 | 41609544 | ZNF658B | | | | | |
| 9 | 41948513 | 41955177 | GLIDR | | | | | |
| 9 | 41958801 | 42019584 | KGFLP2 | | | | | |
| 9 | 42018870 | 42020413 | LOC102724238 | | | | | |
| 9 | 42018874 | 42020413 | LOC554249 | | | | | |
| 9 | 42368302 | 42411410 | ANKRD20A2 | | | | | |
| 9 | 42368336 | 42411863 | ANKRD20A3 | | | | | |
| 9 | 42468588 | 42474238 | FAM95B1 | | | | | |
| 9 | 42493516 | 42498676 | GXYLT1P3 | | | | | |
| 9 | 42718065 | 42719316 | FOXD4L4 | | | | | |
| 9 | 42771707 | 42782614 | LOC101928381 | | | | | |
| 9 | 42844369 | 42859085 | LOC286297 | | | | | |
| 9 | 42858151 | 42893137 | AQP7P3 | | | | | |
| 9 | 43003238 | 43008397 | GXYLT1P3 | | | | | |
| 9 | 43027662 | 43033312 | FAM95B1 | | | | | |

| | | | | | | | | | |
|---|---|---|---|---|---|---|---|---|---|
| 9 | 43089971 | 43133510 | ANKRD20A3 | | | | | | |
| 9 | 43090424 | 43133544 | ANKRD20A2 | | | | | | |
| 9 | 43140536 | 43145484 | LOC642929 | | | | | | |
| 9 | 43608372 | 43615527 | FAM74A7 | | | | | | |
| 9 | 43624501 | 43630730 | SPATA31A6 | | | | | | |
| 9 | 43684884 | 43922473 | CNTNAP3B | | | | | | |
| 9 | 43685195 | 43921493 | CNTNAP3P2 | | | | | | |
| 9 | 44181510 | 44182719 | XLOC_007697 | | | | | | |
| 9 | 44384584 | 44391314 | LOC101927827 | | | | | | |
| 9 | 44401769 | 44403260 | LOC103908605 | | | | | | |
| 9 | 44990235 | 44991492 | FAM27C | | | | | | |
| 9 | 45003233 | 45005290 | LOC102723709 | | | | | | |
| 9 | 46116942 | 46387373 | FAM27E2 | | | | | | |
| 9 | 46686628 | 46688167 | LOC554249 | | | | | | |
| 9 | 46686628 | 46688171 | LOC102724238 | | | | | | |
| 9 | 46687556 | 46746820 | KGFLP1 | | | | | | |
| 9 | 46763790 | 46833319 | LINC01189 | | | | | | |
| 9 | 46843668 | 46845159 | LOC103908605 | | | | | | |
| 9 | 65487272 | 65494386 | FAM74A4 | | | | | | |
| 9 | 65488295 | 65494240 | FAM74A1 | | | | | | |
| 9 | 65488295 | 65494240 | FAM74A6 | | | | | | |
| 9 | 65503362 | 65509610 | SPATA31A5 | | | | | | |
| 9 | 65503365 | 65509610 | SPATA31A7 | | | | | | |
| 9 | 66457288 | 66469310 | LINC01410 | | | | | | |
| 9 | 66490071 | 66494170 | LOC100132249 | | | | | | |
| 9 | 66494268 | 66503030 | PTGER4P2-CDK2AP2P2 | | | | | | |
| 9 | 66513679 | 66553911 | LOC403323 | | | | | | |
| 9 | 66553199 | 66555601 | LOC728673 | | | | | | |
| 9 | 66922966 | 66933829 | LOC101928381 | | | | | | |
| 9 | 67017374 | 67032072 | LOC286297 | | | | | | |
| 9 | 67270214 | 67289492 | AQP7P1 | | | | | | |
| 9 | 67779122 | 67781179 | LOC102723709 | | | | | | |
| 9 | 67784943 | 67786625 | FAM27E3 | | | | | | |
| 9 | 67792928 | 67794189 | FAM27B | | | | | | |
| 9 | 67926760 | 67969840 | ANKRD20A1 | | | | | | |
| 9 | 67926794 | 67970293 | ANKRD20A3 | | | | | | |
| 9 | 68415307 | 68415388 | MIR4477A | | | | | | |
| 9 | 68415307 | 68415388 | MIR4477B | | | | | | |
| 9 | 68427783 | 68455368 | FRG1JP | | | | | | |
| 9 | 68726540 | 68748376 | FRG1HP | | | | | | |
| 9 | 69002238 | 69002321 | MIR1299 | | | | | | |
| 9 | 69065565 | 69087368 | FRG1HP | | | | | | |
| 9 | 69080243 | 69147854 | PGM5P2 | | | | | | |
| 9 | 69174213 | 69181041 | LOC440896 | | | | | | |
| 9 | 69199479 | 69202204 | FOXD4L6 | | | | | | |
| 9 | 69204537 | 69262593 | CBWD6 | | | | | | |
| 9 | 69256633 | 69262630 | CBWD5 | | | | | | |
| 9 | 69381980 | 69425109 | ANKRD20A4 | | | | | | |
| 9 | 69651360 | 69664949 | LOC100133920 | | | | | | |
| 9 | 69746916 | 69757784 | LOC101928381 | | | | | | |
| 9 | 70175706 | 70178815 | FOXD4L5 | | | | | | |
| 9 | 70364017 | 70374924 | LOC101928381 | | | | | | |
| 9 | 70427648 | 70428899 | FOXD4L4 | | | | | | |
| 9 | 70432003 | 70862784 | CBWD5 | | | | | | |
| 9 | 70856860 | 70914951 | CBWD3 | | | | | | |
| 9 | 70917782 | 70920000 | FOXD4L3 | | | | | | |
| 9 | 70970079 | 71145977 | PGM5 | | | | | | |
| 9 | 71151497 | 71155783 | TMEM252 | | | | | | |
| 9 | 71158456 | 71161518 | LINC01506 | | | | | | |
| 9 | 71320329 | 71624092 | PIP5K1B | | | | | | |
| 9 | 71394921 | 71398795 | FAM122A | | | | | | |
| 9 | 71568003 | 71590757 | LOC101927069 | | | | | | |
| 9 | 71627448 | 71629039 | PRKACG | | | PRKACG | | | |
| 9 | 71650478 | 71715094 | FXN | | | FXN | | | |
| 9 | 71736179 | 71870124 | TJP2 | | | TJP2 | | | |
| 9 | 71911596 | 71921972 | BANCR | | | | | | |

| | | | | | | | | |
|---|---|---|---|---|---|---|---|---|
| 9 | 71939487 | 72007370 | FAM189A2 | | | | | |
| 9 | 72042448 | 72287275 | APBA1 | | | | | |
| 9 | 72324437 | 72374876 | PTAR1 | | | | | |
| 9 | 72434320 | 72521151 | C9orf135 | | | | | |
| 9 | 72658496 | 72790804 | MAMDC2 | | | | | |
| 9 | 72830974 | 72969789 | SMC5 | | | | | |
| 9 | 72999512 | 73029573 | KLF9 | | | | | |
| 9 | 73149965 | 73483974 | TRPM3 | | TRPM3 | | | |
| 9 | 73424890 | 73425000 | MIR204 | | | | | |
| 9 | 74298281 | 74383800 | TMEM2 | | | | | |
| 9 | 74477367 | 74526161 | ABHD17B | | | | | |
| 9 | 74526422 | 74588371 | C9orf85 | | | | | |
| 9 | 74666296 | 74675521 | C9orf57 | | | | | |
| 9 | 74729510 | 74867140 | GDA | | | GDA | | |
| 9 | 74920351 | 74958126 | LINC01504 | | | | | |
| 9 | 74966340 | 74980163 | ZFAND5 | | | | | |
| 9 | 75136716 | 75451267 | TMC1 | | | | | |
| 9 | 75486644 | 75489025 | LINC01474 | | | | | |
| 9 | 75515577 | 75568233 | ALDH1A1 | | | | | |
| 9 | 75766646 | 75785310 | ANXA1 | | | ANXA1 | | |
| 9 | 76368039 | 77204569 | MIR6130 | | | | | |
| 9 | 76986446 | 76998446 | LOC101927358 | | | | | |
| 9 | 77088350 | 77302117 | RORB | | RORB | | | |
| 9 | 77337410 | 77503010 | TRPM6 | | TRPM6 | TRPM6 | | |
| 9 | 77561498 | 77567802 | C9orf40 | | | | | |
| 9 | 77567880 | 77612296 | C9orf41 | | | | | |
| 9 | 77597872 | 77643310 | CARNMT1 | | | | | |
| 9 | 77676115 | 77703133 | NMRK1 | | | | | |
| 9 | 77703397 | 77762114 | OSTF1 | | | | | |
| 9 | 78118511 | 78338744 | MIR548H3 | | | | | |
| 9 | 78505559 | 78977255 | PCSK5 | | | | | |
| 9 | 79000432 | 79009444 | RFK | | | | | |
| 9 | 79013514 | 79014954 | RPSAP9 | | | | | |
| 9 | 79056581 | 79122332 | GCNT1 | | | | | |
| 9 | 79226291 | 79521003 | PRUNE2 | | | | | |
| 9 | 79379351 | 79402485 | PCA3 | | | | | |
| 9 | 79634570 | 79635869 | FOXB2 | | | | | |
| 9 | 79791671 | 80032399 | VPS13A | | | VPS13A | | |
| 9 | 80037994 | 80141678 | GNA14 | | | | | |
| 9 | 80331189 | 80646365 | GNAQ | | GNAQ | | | |
| 9 | 80850990 | 80881983 | CEP78 | | | | | |
| 9 | 80911990 | 80945009 | PSAT1 | | PSAT1 | | | |
| 9 | 81750337 | 81760581 | LOC101927450 | | | | | |
| 9 | 82186687 | 82341796 | TLE4 | | | | | |
| 9 | 82439444 | 82649470 | LINC01507 | | | | | |
| 9 | 84198597 | 84304450 | TLE1 | | | | | |
| 9 | 84304627 | 84391814 | LOC101927502 | | | | | |
| 9 | 84528351 | 84534842 | SPATA31D5P | | | | | |
| 9 | 84543342 | 84549913 | SPATA31D4 | | | | | |
| 9 | 84558414 | 84565009 | SPATA31D3 | | | | | |
| 9 | 84603686 | 84610171 | SPATA31D1 | | | | | |
| 9 | 85594499 | 85678043 | RASEF | | | | | |
| 9 | 85857904 | 86153348 | FRMD3 | | | | | |
| 9 | 86237963 | 86259045 | IDNK | | | | | |
| 9 | 86274877 | 86323168 | UBQLN1 | | | | | |
| 9 | 86354335 | 86432752 | GKAP1 | | | | | |
| 9 | 86451614 | 86536380 | KIF27 | | | | | |
| 9 | 86553226 | 86571901 | C9orf64 | | | | | |
| 9 | 86582997 | 86595697 | HNRNPK | | | | | |
| 9 | 86584662 | 86584772 | MIR7 | | | | | |
| 9 | 86595636 | 86618987 | RMI1 | | | | | |
| 9 | 86678357 | 86709494 | LOC101927575 | | | | | |
| 9 | 86890764 | 86983413 | SLC28A3 | | | | | |
| 9 | 87283372 | 87641985 | NTRK2 | NTRK2 | NTRK2 | NTRK2 | | |
| 9 | 88161453 | 88356944 | AGTPBP1 | | | | | |
| 9 | 88420916 | 88457794 | LOC389765 | | | | | |

| | | | | | | | | |
|---|---|---|---|---|---|---|---|---|
| 9 | 88556056 | 88637217 | NAA35 | | | | | |
| 9 | 88641057 | 88715116 | GOLM1 | | | | | |
| 9 | 88742454 | 88771987 | LOC101927623 | | | | | |
| 9 | 88835179 | 88874572 | C9orf153 | | | | | |
| 9 | 88879462 | 88897490 | ISCA1 | | | | | |
| 9 | 88902647 | 88969402 | ZCCHC6 | | | | | |
| 9 | 89559276 | 89562104 | GAS1 | | | | | |
| 9 | 89563612 | 89616948 | GAS1RR | | | | | |
| 9 | 89623365 | 89657041 | LOC440173 | | | | | |
| 9 | 89698800 | 89700140 | LOC494127 | | | | | |
| 9 | 89763558 | 89774641 | C9orf170 | | | | | |
| 9 | 90112142 | 90323549 | DAPK1 | | | | | |
| 9 | 90340973 | 90346384 | CTSL | | | | | |
| 9 | 90387829 | 90401799 | CTSL3P | | | | | |
| 9 | 90459659 | 90462339 | CTSLP8 | | | | | |
| 9 | 90472957 | 90473971 | LOC392364 | | | | | |
| 9 | 90497771 | 90503814 | SPATA31E1 | | | | | |
| 9 | 90532876 | 90538572 | SPATA31C1 | | | | | |
| 9 | 90581358 | 90589695 | CDK20 | | | | | |
| 9 | 90744219 | 90749900 | SPATA31C2 | | | | | |
| 9 | 91003296 | 91093622 | SPIN1 | | | | | |
| 9 | 91150015 | 91190704 | NXNL2 | | | | | |
| 9 | 91262093 | 91267075 | LOC286238 | | | | | |
| 9 | 91360750 | 91360820 | MIR4289 | | | | | |
| 9 | 91605777 | 91611057 | C9orf47 | | | | | |
| 9 | 91606323 | 91620069 | S1PR3 | | | | | |
| 9 | 91620685 | 91793682 | SHC3 | | | SHC3 | | |
| 9 | 91926109 | 91931618 | CKS2 | | | | | |
| 9 | 91927139 | 91927221 | MIR3153 | | | | | |
| 9 | 91933387 | 91974578 | SECISBP2 | | | | | |
| 9 | 91975705 | 92094611 | SEMA4D | | | | | |
| 9 | 92219926 | 92221469 | GADD45G | | | | | |
| 9 | 92254697 | 92334674 | UNQ6494 | | | | | |
| 9 | 92731697 | 92749768 | LOC101927847 | | | | | |
| 9 | 92782935 | 92803781 | MIR4290HG | | | | | |
| 9 | 92785722 | 92785817 | MIR4290 | | | | | |
| 9 | 93063177 | 93195771 | LINC01508 | | | | | |
| 9 | 93224713 | 93345028 | LINC01501 | | | | | |
| 9 | 93372113 | 93405387 | DIRAS2 | | | | | |
| 9 | 93563961 | 93660842 | SYK | | | | | |
| 9 | 93825575 | 93837414 | LOC100129316 | | | | | |
| 9 | 93976096 | 94124251 | AUH | | AUH | AUH | | |
| 9 | 94171326 | 94186908 | NFIL3 | | | | | |
| 9 | 94398532 | 94398627 | MIR3910 | | | | | |
| 9 | 94484877 | 94712444 | ROR2 | | | ROR2 | | |
| 9 | 94793415 | 94877756 | SPTLC1 | | | | | |
| 9 | 94895115 | 94900911 | LOC100128076 | | | | | |
| 9 | 94903748 | 94921890 | LINC00475 | | | | | |
| 9 | 94972489 | 95056038 | IARS | | | | | |
| 9 | 95054739 | 95054829 | MIR3651 | | | | | |
| 9 | 95054742 | 95054875 | SNORA84 | | | | | |
| 9 | 95059639 | 95087876 | NOL8 | | | | | |
| 9 | 95087749 | 95377437 | CENPP | | | | | |
| 9 | 95145552 | 95166981 | OGN | | | | | |
| 9 | 95176526 | 95186836 | OMD | | | | | |
| 9 | 95218488 | 95244844 | ASPN | | | | | |
| 9 | 95255828 | 95298374 | ECM2 | | | | | |
| 9 | 95290265 | 95290340 | MIR4670 | | | | | |
| 9 | 95362538 | 95377437 | CENPP | | | | | |
| 9 | 95375465 | 95432547 | IPPK | | | | | |
| 9 | 95380331 | 95382817 | LOC100128361 | | | | | |
| 9 | 95473644 | 95527083 | BICD2 | | | BICD2 | | |
| 9 | 95571892 | 95600739 | ANKRD19P | | | | | |
| 9 | 95607312 | 95640320 | ZNF484 | | | | | |
| 9 | 95644848 | 95651837 | LOC642943 | | | | | |
| 9 | 95709600 | 95798518 | FGD3 | | | | | |

| | | | | | | | | | |
|---|---|---|---|---|---|---|---|---|---|
| 9 | 95770396 | 95777226 | LOC101927954 | | | | | | |
| 9 | 95820969 | 95847418 | SUSD3 | | | | | | |
| 9 | 95858404 | 95875577 | CARD19 | | | | | | |
| 9 | 95883770 | 95896570 | NINJ1 | | | | | | |
| 9 | 95947211 | 96082854 | WNK2 | | | | | | |
| 9 | 96080480 | 96108696 | C9orf129 | | | | | | |
| 9 | 96208781 | 96215874 | FAM120AOS | | | | | | |
| 9 | 96213977 | 96328397 | FAM120A | | | | | | |
| 9 | 96338908 | 96441869 | PHF2 | | | | | | |
| 9 | 96357122 | 96357176 | MIR548AU | | | | | | |
| 9 | 96581638 | 96581703 | MIR4291 | | | | | | |
| 9 | 96713908 | 96717608 | BARX1 | | | | | | |
| 9 | 96793075 | 96872138 | PTPDC1 | | | | | | |
| 9 | 96938238 | 96938318 | MIRLET7A1 | | | | | | |
| 9 | 96938628 | 96938715 | MIRLET7F1 | | | | | | |
| 9 | 96938851 | 96966848 | MIRLET7DHG | | | | | | |
| 9 | 96941115 | 96941202 | MIRLET7D | | | | | | |
| 9 | 97021547 | 97064111 | ZNF169 | | | | | | |
| 9 | 97080477 | 97090926 | NUTM2F | | | | | | |
| 9 | 97094757 | 97123230 | LOC100132077 | | | | | | |
| 9 | 97136832 | 97223202 | MFSD14B | | | | | | |
| 9 | 97317326 | 97330411 | PCAT7 | | | | | | |
| 9 | 97320995 | 97356114 | FBP2 | | | | | | |
| 9 | 97365420 | 97402531 | FBP1 | | | FBP1 | | | |
| 9 | 97488950 | 97849500 | C9orf3 | | | C9orf3 | | | |
| 9 | 97572243 | 97572339 | MIR2278 | | | | | | |
| 9 | 97827631 | 97827728 | MIR6081 | | | | | | |
| 9 | 97847489 | 97847586 | MIR23B | | | | | | |
| 9 | 97847726 | 97847823 | MIR27B | | | | | | |
| 9 | 97848295 | 97848376 | MIR3074 | | | | | | |
| 9 | 97848302 | 97848370 | MIR24 | | | | | | |
| 9 | 97861335 | 98079991 | FANCC | | | | | | |
| 9 | 98205263 | 98279247 | PTCH1 | | | PTCH1 | | | |
| 9 | 98225890 | 98232301 | LOC100507346 | | | | | | |
| 9 | 98568369 | 98638259 | LINC00476 | | | | | | |
| 9 | 98637899 | 98780735 | ERCC6L2 | | | | | | |
| 9 | 98782013 | 98784037 | LINC00092 | | | | | | |
| 9 | 98828120 | 98864194 | LOC158435 | | | | | | |
| 9 | 98868942 | 98878693 | LOC158434 | | | | | | |
| 9 | 98997588 | 99064434 | HSD17B3 | | HSD17B3 | | | | |
| 9 | 99075718 | 99145992 | SLC35D2 | | | | | | |
| 9 | 99148224 | 99180669 | ZNF367 | | | | | | |
| 9 | 99212436 | 99253618 | HABP4 | | | | | | |
| 9 | 99262394 | 99382112 | CDC14B | | | | | | |
| 9 | 99403532 | 99417599 | AAED1 | | | | | | |
| 9 | 99488102 | 99489749 | LOC441455 | | | | | | |
| 9 | 99516830 | 99540411 | ZNF510 | | | | | | |
| 9 | 99579272 | 99616389 | ZNF782 | | | | | | |
| 9 | 99631181 | 99634510 | LOC100132781 | | | | | | |
| 9 | 99671356 | 99672737 | LOC441454 | | | | | | |
| 9 | 99691285 | 99704572 | NUTM2G | | | | | | |
| 9 | 99708326 | 99775862 | MFSD14C | | | | | | |
| 9 | 99791958 | 99801925 | CTSV | | | | | | |
| 9 | 99837952 | 99844227 | GAS2L1P2 | | | | | | |
| 9 | 100000707 | 100059596 | LOC100499484 | | | | | | |
| 9 | 100000707 | 100139577 | LOC100499484-C9ORF174 | | | | | | |
| 9 | 100069909 | 100139577 | CCDC180 | | | | | | |
| 9 | 100125835 | 100125963 | MIR1302 | | | | | | |
| 9 | 100153118 | 100158973 | LOC286359 | | | | | | |
| 9 | 100174301 | 100258405 | TDRD7 | | | | | | |
| 9 | 100263461 | 100364025 | TMOD1 | | | | | | |
| 9 | 100362361 | 100395962 | TSTD2 | | | | | | |
| 9 | 100395704 | 100436029 | NCBP1 | | | | | | |
| 9 | 100437190 | 100459691 | XPA | | | XPA | | | |
| 9 | 100615536 | 100618997 | FOXE1 | | | | | | |
| 9 | 100666770 | 100684852 | TRMO | | | | | | |

| | | | | | | | | |
|---|---|---|---|---|---|---|---|---|
| 9 | 100689072 | 100707197 | HEMGN | | | | | |
| 9 | 100745488 | 100778224 | ANP32B | | | | | |
| 9 | 100818958 | 100845365 | NANS | | | | | |
| 9 | 100846634 | 100881635 | TRIM14 | | | | | |
| 9 | 100883256 | 100954956 | CORO2A | | | | | |
| 9 | 100961279 | 101018003 | TBC1D2 | | | | | |
| 9 | 100991430 | 100991499 | MIR6854 | | | | | |
| 9 | 101050363 | 101471479 | GABBR2 | | GABBR2 | GABBR2 | GABBR2 | GABBR2 |
| 9 | 101494290 | 101558824 | ANKS6 | | | | | |
| 9 | 101569980 | 101612363 | GALNT12 | | | | | |
| 9 | 101705994 | 101833074 | COL15A1 | | | | | |
| 9 | 101867370 | 101916473 | TGFBR1 | | | TGFBR1 | | |
| 9 | 101978706 | 101984246 | ALG2 | | | | | |
| 9 | 101984569 | 101992901 | SEC61B | | | | | |
| 9 | 102117621 | 102137539 | NAMA | | | | | |
| 9 | 102348067 | 102582171 | LOC101928438 | | | | | |
| 9 | 102584136 | 102629173 | NR4A3 | | | | | |
| 9 | 102648599 | 102736818 | STX17 | | | STX17 | | |
| 9 | 102741462 | 102861334 | ERP44 | | | | | |
| 9 | 102861466 | 103064457 | INVS | | | | | |
| 9 | 103064356 | 103115259 | TEX10 | | | | | |
| 9 | 103189494 | 103214016 | MSANTD3 | | | | | |
| 9 | 103204187 | 103339918 | MSANTD3-TMEFF1 | | | | | |
| 9 | 103235519 | 103339918 | TMEFF1 | | | | | |
| 9 | 103340360 | 103350669 | MURC | | | | | |
| 9 | 103791030 | 104087417 | PLPPR1 | | | | | |
| 9 | 104122698 | 104147287 | BAAT | | | BAAT | | |
| 9 | 104152248 | 104160919 | MRPL50 | | | | | |
| 9 | 104161135 | 104172942 | ZNF189 | | | | | |
| 9 | 104182841 | 104198062 | ALDOB | | | | | |
| 9 | 104230720 | 104249503 | TMEM246 | | | | | |
| 9 | 104296130 | 104325626 | RNF20 | | | | | |
| 9 | 104331633 | 104500862 | GRIN3A | | | GRIN3A | | |
| 9 | 104353896 | 104357283 | PPP3R2 | | | | | |
| 9 | 105281918 | 105419791 | LINC00587 | | | | | |
| 9 | 105757592 | 105780770 | CYLC2 | | | | | |
| 9 | 105902809 | 106087315 | LINC01492 | | | | | |
| 9 | 106761989 | 106762763 | LOC101928523 | | | | | |
| 9 | 106842304 | 106903700 | SMC2 | | | | | |
| 9 | 107266543 | 107267503 | OR13F1 | | | | | |
| 9 | 107288533 | 107289490 | OR13C4 | | | | | |
| 9 | 107298050 | 107299094 | OR13C3 | | | | | |
| 9 | 107331448 | 107332411 | OR13C8 | | | | | |
| 9 | 107360737 | 107361694 | OR13C5 | | | | | |
| 9 | 107366951 | 107367908 | OR13C2 | | | | | |
| 9 | 107379528 | 107380485 | OR13C9 | | | | | |
| 9 | 107456702 | 107457743 | OR13D1 | | | | | |
| 9 | 107509968 | 107522403 | NIPSNAP3A | | | | | |
| 9 | 107526021 | 107540045 | NIPSNAP3B | | | | | |
| 9 | 107543283 | 107690527 | ABCA1 | | | ABCA1 | | |
| 9 | 108006893 | 108159628 | SLC44A1 | | | | | |
| 9 | 108210314 | 108314714 | FSD1L | | | | | |
| 9 | 108320410 | 108403399 | FKTN | | FKTN | | | |
| 9 | 108424737 | 108425385 | TAL2 | | | | | |
| 9 | 108456805 | 108538892 | TMEM38B | | | | | |
| 9 | 109363208 | 109363303 | MIR8081 | | | | | |
| 9 | 109378338 | 109442081 | LINC01505 | | | | | |
| 9 | 109625377 | 109773796 | ZNF462 | | | | | |
| 9 | 109653504 | 109848716 | MIR548Q | | | | | |
| 9 | 109737113 | 109865269 | LOC340512 | | | | | |
| 9 | 110045516 | 110094475 | RAD23B | | | | | |
| 9 | 110182564 | 110228866 | LINC01509 | | | | | |
| 9 | 110247132 | 110252001 | KLF4 | | | | | |
| 9 | 111616868 | 111618275 | ACTL7B | | | | | |
| 9 | 111624507 | 111626035 | ACTL7A | | | | | |
| 9 | 111629799 | 111696612 | IKBKAP | | | IKBKAP | | |

| | | | | | | | | |
|---|---|---|---|---|---|---|---|---|
| 9 | 111696672 | 111703237 | FAM206A | | | | | |
| 9 | 111704848 | 111775874 | CTNNAL1 | | | | | |
| 9 | 111777414 | 111882225 | TMEM245 | | | | | |
| 9 | 111808508 | 111808578 | MIR32 | | | | | |
| 9 | 111899580 | 111929571 | FRRS1L | | | | | |
| 9 | 111934253 | 112083244 | EPB41L4B | | | | | |
| 9 | 112137973 | 112260593 | PTPN3 | | | | | |
| 9 | 112273754 | 112273825 | MIR3927 | | | | | |
| 9 | 112403067 | 112713756 | PALM2 | | | | | |
| 9 | 112542576 | 112934791 | PALM2-AKAP2 | | | | | |
| 9 | 112810877 | 112934791 | AKAP2 | | | AKAP2 | | |
| 9 | 112961845 | 112970413 | C9orf152 | | | | | |
| 9 | 113006091 | 113018920 | TXN | | | | | |
| 9 | 113065866 | 113100164 | TXNDC8 | | | | | |
| 9 | 113127528 | 113342160 | SVEP1 | | | | | |
| 9 | 113431050 | 113563278 | MUSK | | | MUSK | | |
| 9 | 113636053 | 113800365 | LPAR1 | | | LPAR1 | | |
| 9 | 114033435 | 114033494 | MIR7702 | | | | | |
| 9 | 114089762 | 114090713 | OR2K2 | | | | | |
| 9 | 114122972 | 114247025 | KIAA0368 | | | | | |
| 9 | 114287438 | 114340124 | ZNF483 | | | | | |
| 9 | 114312001 | 114362135 | PTGR1 | | | | | |
| 9 | 114365110 | 114375833 | LRRC37A5P | | | | | |
| 9 | 114393631 | 114416631 | DNAJC25 | | | | | |
| 9 | 114393631 | 114432526 | DNAJC25-GNG10 | | | | | |
| 9 | 114423850 | 114432526 | GNG10 | | | | | |
| 9 | 114448798 | 114557288 | C9orf84 | | | | | |
| 9 | 114659045 | 114697654 | UGCG | | | | | |
| 9 | 114694379 | 114694449 | MIR4668 | | | | | |
| 9 | 114758831 | 115112545 | MIR3134 | | | | | |
| 9 | 114803060 | 114937577 | SUSD1 | | | | | |
| 9 | 114979994 | 115095944 | PTBP3 | | | | | |
| 9 | 115142188 | 115234685 | HSDL2 | | | | | |
| 9 | 115249247 | 115427591 | KIAA1958 | | | | | |
| 9 | 115448790 | 115480387 | INIP | | | | | |
| 9 | 115513133 | 115637267 | SNX30 | | | | | |
| 9 | 115641199 | 115653193 | SLC46A2 | | | | | |
| 9 | 115759399 | 115774472 | ZNF883 | | | | | |
| 9 | 115804094 | 115819071 | ZFP37 | | | | | |
| 9 | 115867002 | 115873957 | FAM225B | | | | | |
| 9 | 115875175 | 115882126 | FAM225A | | | | | |
| 9 | 115913237 | 115926422 | SLC31A2 | | | | | |
| 9 | 115927799 | 115983641 | FKBP15 | | | | | |
| 9 | 115983807 | 116026772 | SLC31A1 | | | | | |
| 9 | 116029289 | 116037869 | CDC26 | | | | | |
| 9 | 116037913 | 116055185 | PRPF4 | | | | | |
| 9 | 116059372 | 116061320 | RNF183 | | | | | |
| 9 | 116075501 | 116102620 | WDR31 | | | | | |
| 9 | 116111811 | 116133513 | BSPRY | | | | | |
| 9 | 116135695 | 116139289 | HDHD3 | | | | | |
| 9 | 116148591 | 116163618 | ALAD | | ALAD | | | |
| 9 | 116169517 | 116173029 | POLE3 | | | | | |
| 9 | 116172923 | 116191964 | C9orf43 | | | | | |
| 9 | 116207008 | 116360023 | RGS3 | | | RGS3 | | |
| 9 | 116638561 | 116818875 | ZNF618 | | | | | |
| 9 | 116822407 | 116840752 | AMBP | | | | | |
| 9 | 116853917 | 116861337 | KIF12 | | | | | |
| 9 | 116917824 | 117074796 | COL27A1 | | | | | |
| 9 | 116971713 | 116971809 | MIR455 | | | | | |
| 9 | 117085302 | 117088759 | ORM1 | | | | | |
| 9 | 117092068 | 117095536 | ORM2 | | | | | |
| 9 | 117098406 | 117156685 | AKNA | | | | | |
| 9 | 117164359 | 117267736 | DFNB31 | | | | | |
| 9 | 117349993 | 117361152 | ATP6V1G1 | | | | | |
| 9 | 117373705 | 117408703 | C9orf91 | | | | | |
| 9 | 117428713 | 117444369 | LOC100505478 | | | | | |

| | | | | | | | | |
|---|---|---|---|---|---|---|---|---|
| 9 | 117546914 | 117568408 | TNFSF15 | | | | | |
| 9 | 117655622 | 117692875 | TNFSF8 | | | | | |
| 9 | 117781853 | 117880536 | TNC | | | TNC | | |
| 9 | 117881818 | 117900688 | LOC101928748 | | | | | |
| 9 | 117904096 | 118164923 | DEC1 | | | | | |
| 9 | 118501948 | 118506518 | LOC101928775 | | | | | |
| 9 | 118650543 | 118687377 | LINC00474 | | | | | |
| 9 | 118916070 | 119162885 | PAPPA | | | | | |
| 9 | 119187503 | 119449542 | ASTN2 | | ASTN2 | | | |
| 9 | 119449580 | 119463579 | TRIM32 | | | | | |
| 9 | 119943344 | 119943480 | SNORA70C | | | | | |
| 9 | 120410883 | 120419304 | LOC101928797 | | | | | |
| 9 | 120466452 | 120479769 | TLR4 | | | | | |
| 9 | 121928907 | 122131739 | BRINP1 | | | | | |
| 9 | 122697337 | 122700110 | LINC01613 | | | | | |
| 9 | 123007256 | 123007328 | MIR147A | | | | | |
| 9 | 123151146 | 123342448 | CDK5RAP2 | | | CDK5RAP2 | | |
| 9 | 123363195 | 123476765 | MEGF9 | | | | | |
| 9 | 123519253 | 123555740 | FBXW2 | | | | | |
| 9 | 123555774 | 123561009 | LOC100288842 | | | | | |
| 9 | 123578331 | 123616651 | PSMD5 | | | PSMD5 | | |
| 9 | 123617928 | 123639606 | PHF19 | | | | | |
| 9 | 123664670 | 123691451 | TRAF1 | | | | | |
| 9 | 123714613 | 123812551 | C5 | | | C5 | | |
| 9 | 123850573 | 123939886 | CNTRL | | | | | |
| 9 | 123940414 | 123964365 | RAB14 | | | RAB14 | | |
| 9 | 124030379 | 124095120 | GSN | | | GSN | | |
| 9 | 124101265 | 124132582 | STOM | | | | | |
| 9 | 124217318 | 124262306 | GGTA1P | | | | | |
| 9 | 124329380 | 124545154 | DAB2IP | | | | | |
| 9 | 124584203 | 124855885 | TTLL11 | | | | | |
| 9 | 124882360 | 124882446 | MIR4478 | | | | | |
| 9 | 124894744 | 124922098 | NDUFA8 | | | | | |
| 9 | 124922186 | 124962367 | MORN5 | | | | | |
| 9 | 124964855 | 124991091 | LHX6 | | | | | |
| 9 | 125001833 | 125027143 | RBM18 | | | | | |
| 9 | 125026881 | 125085742 | MRRF | | | | | |
| 9 | 125132808 | 125157982 | PTGS1 | | | | | |
| 9 | 125239236 | 125240205 | OR1J1 | | | | | |
| 9 | 125273080 | 125274022 | OR1J2 | | | OR1J2 | | |
| 9 | 125281419 | 125282361 | OR1J4 | | | | | |
| 9 | 125288636 | 125289572 | OR1N1 | | | | | |
| 9 | 125315448 | 125316441 | OR1N2 | | | | | |
| 9 | 125329826 | 125330756 | OR1L8 | | | | | |
| 9 | 125377016 | 125377961 | OR1Q1 | | | | | |
| 9 | 125390857 | 125391814 | OR1B1 | | | | | |
| 9 | 125423994 | 125424927 | OR1L1 | | | | | |
| 9 | 125437408 | 125438383 | OR1L3 | | | | | |
| 9 | 125486268 | 125487204 | OR1L4 | | | | | |
| 9 | 125512126 | 125513062 | OR1L6 | | | | | |
| 9 | 125551211 | 125552174 | OR5C1 | | | | | |
| 9 | 125562401 | 125563352 | OR1K1 | | | | | |
| 9 | 125580375 | 125590935 | PDCL | | | | | |
| 9 | 125611731 | 125667562 | RC3H2 | | | | | |
| 9 | 125642491 | 125642598 | SNORD90 | | | | | |
| 9 | 125670334 | 125675607 | ZBTB6 | | | | | |
| 9 | 125680307 | 125693830 | ZBTB26 | | | | | |
| 9 | 125703287 | 125867147 | RABGAP1 | | | | | |
| 9 | 125795921 | 125797944 | GPR21 | | | | | |
| 9 | 125871772 | 125877756 | MIR600HG | | | | | |
| 9 | 125873824 | 125873922 | MIR600 | | | | | |
| 9 | 125883907 | 126030855 | STRBP | | | | | |
| 9 | 126118445 | 126142614 | CRB2 | | | CRB2 | | |
| 9 | 126141932 | 126692417 | DENND1A | | | | | |
| 9 | 126164803 | 126164882 | MIR601 | | | | | |
| 9 | 126247807 | 126247901 | MIR7150 | | | | | |

| | | | | | | | | | |
|---|---|---|---|---|---|---|---|---|---|
| 9 | 126768555 | 126771675 | LOC100505588 | | | | | | |
| 9 | 126773888 | 126795442 | LHX2 | | | | | | |
| 9 | 127019884 | 127114719 | NEK6 | | | | | | |
| 9 | 127115743 | 127177752 | PSMB7 | | | | | | |
| 9 | 127115751 | 127121463 | LOC100129034 | | | | | | |
| 9 | 127243514 | 127269699 | NR5A1 | | | NR5A1 | | | |
| 9 | 127279553 | 127533589 | NR6A1 | | | | | | |
| 9 | 127420714 | 127460907 | MIR181A2HG | | | | | | |
| 9 | 127454720 | 127454830 | MIR181A2 | | | | | | |
| 9 | 127455988 | 127456077 | MIR181B2 | | | | | | |
| 9 | 127539436 | 127577170 | OLFML2A | | | | | | |
| 9 | 127615695 | 127620170 | WDR38 | | | | | | |
| 9 | 127620157 | 127624240 | RPL35 | | | | | | |
| 9 | 127624396 | 127640012 | ARPC5L | | | | | | |
| 9 | 127640572 | 127703386 | GOLGA1 | | | GOLGA1 | | | |
| 9 | 127704887 | 127905838 | SCAI | | | | | | |
| 9 | 127908851 | 127952218 | PPP6C | | | | | | |
| 9 | 127962820 | 127996438 | RABEPK | | | | | | |
| 9 | 127997126 | 128003666 | HSPA5 | | | HSPA5 | | | |
| 9 | 128024072 | 128127290 | GAPVD1 | | | | | | |
| 9 | 128199672 | 128469513 | MAPKAP1 | | | | | | |
| 9 | 128506033 | 128508208 | LOC51145 | | | | | | |
| 9 | 128509616 | 128729655 | PBX3 | | | | | | |
| 9 | 129032402 | 129038194 | LOC101929116 | | | | | | |
| 9 | 129089122 | 129269320 | MVB12B | | | | | | |
| 9 | 129170053 | 129172783 | NRON | | | | | | |
| 9 | 129376721 | 129463311 | LMX1B | | | LMX1B | | | |
| 9 | 129567284 | 129600487 | ZBTB43 | | | | | | |
| 9 | 129622943 | 129648156 | ZBTB34 | | | | | | |
| 9 | 129677052 | 129979724 | RALGPS1 | | | | | | |
| 9 | 129849627 | 129885044 | ANGPTL2 | | | ANGPTL2 | | | |
| 9 | 130025940 | 130155828 | GARNL3 | | | | | | |
| 9 | 130159416 | 130170170 | SLC2A8 | | | | | | |
| 9 | 130186652 | 130207651 | ZNF79 | | | | | | |
| 9 | 130209952 | 130213711 | RPL12 | | | | | | |
| 9 | 130210780 | 130210916 | SNORA65 | | | | | | |
| 9 | 130213764 | 130265780 | LRSAM1 | | | LRSAM1 | | | |
| 9 | 130267616 | 130341286 | FAM129B | | | | | | |
| 9 | 130374485 | 130454995 | STXBP1 | | STXBP1 | | STXBP1 | | |
| 9 | 130452965 | 130453074 | MIR3911 | | | | | | |
| 9 | 130469270 | 130476303 | CFAP157 | | | | | | |
| 9 | 130476226 | 130477936 | PTRH1 | | | | | | |
| 9 | 130478342 | 130493879 | TTC16 | | | | | | |
| 9 | 130493802 | 130497628 | TOR2A | | | | | | |
| 9 | 130500595 | 130541048 | SH2D3C | | | | | | |
| 9 | 130548111 | 130548202 | MIR3960 | | | | | | |
| 9 | 130548196 | 130548286 | MIR2861 | | | | | | |
| 9 | 130548304 | 130553052 | CDK9 | | | | | | |
| 9 | 130565136 | 130576360 | FPGS | | | | | | |
| 9 | 130577290 | 130617052 | ENG | | | | | | |
| 9 | 130628758 | 130641900 | AK1 | | | | | | |
| 9 | 130631693 | 130631774 | MIR4672 | | | | | | |
| 9 | 130647599 | 130667627 | ST6GALNAC6 | | | | | | |
| 9 | 130670164 | 130679305 | ST6GALNAC4 | | | | | | |
| 9 | 130683807 | 130693076 | PIP5KL1 | | | | | | |
| 9 | 130697373 | 130700763 | DPM2 | | | | | | |
| 9 | 130702860 | 130742812 | FAM102A | | | | | | |
| 9 | 130823511 | 130829599 | NAIF1 | | | | | | |
| 9 | 130830478 | 130881013 | SLC25A25 | | | | | | |
| 9 | 130882971 | 130892913 | PTGES2 | | | | | | |
| 9 | 130911708 | 130915734 | LCN2 | | | | | | |
| 9 | 130922538 | 130926207 | C9orf16 | | | | | | |
| 9 | 130928343 | 130966662 | CIZ1 | | | | | | |
| 9 | 130965633 | 131017527 | DNM1 | | DNM1 | | | DNM1 | |
| 9 | 131006999 | 131007109 | MIR199B | | | | | | |
| 9 | 131007225 | 131007309 | MIR3154 | | | | | | |

| | | | | | | | | |
|---|---|---|---|---|---|---|---|---|
| 9 | 131018107 | 131038268 | GOLGA2 | | | GOLGA2 | | |
| 9 | 131037657 | 131051268 | SWI5 | | | | | |
| 9 | 131071395 | 131084697 | TRUB2 | | | | | |
| 9 | 131084786 | 131096351 | COQ4 | | | | | |
| 9 | 131102838 | 131123749 | SLC27A4 | | SLC27A4 | | | |
| 9 | 131114325 | 131424415 | MIR1268A | | | | | |
| 9 | 131133597 | 131154295 | URM1 | | | | | |
| 9 | 131154896 | 131154993 | MIR219A2 | | | | | |
| 9 | 131154899 | 131154987 | MIR219B | | | | | |
| 9 | 131181438 | 131199630 | CERCAM | | | | | |
| 9 | 131217433 | 131257119 | ODF2 | | | | | |
| 9 | 131266970 | 131304580 | GLE1 | | | | | |
| 9 | 131314836 | 131395944 | SPTAN1 | | SPTAN1 | SPTAN1 | | |
| 9 | 131395939 | 131419129 | WDR34 | | | | | |
| 9 | 131445933 | 131458675 | SET | | | | | |
| 9 | 131464782 | 131483199 | PKN3 | | | | | |
| 9 | 131483148 | 131486408 | ZDHHC12 | | | | | |
| 9 | 131486740 | 131495522 | LOC100506100 | | | | | |
| 9 | 131492066 | 131534213 | ZER1 | | | | | |
| 9 | 131549485 | 131572711 | TBC1D13 | | | | | |
| 9 | 131580778 | 131584955 | ENDOG | | | ENDOG | | |
| 9 | 131581929 | 131592100 | C9orf114 | | | | | |
| 9 | 131595220 | 131644354 | CCBL1 | | | | | |
| 9 | 131644390 | 131680317 | LRRC8A | | LRRC8A | | | |
| 9 | 131683173 | 131704320 | PHYHD1 | | | | | |
| 9 | 131707808 | 131710012 | DOLK | | | | | |
| 9 | 131709971 | 131769375 | NUP188 | | | | | |
| 9 | 131770071 | 131790632 | SH3GLB2 | | | SH3GLB2 | | |
| 9 | 131799252 | 131834351 | FAM73B | | | | | |
| 9 | 131843382 | 131852717 | DOLPP1 | | | | | |
| 9 | 131857072 | 131873070 | CRAT | | | | | |
| 9 | 131873227 | 131911225 | PPP2R4 | | | | | |
| 9 | 131937830 | 131940540 | IER5L | | | | | |
| 9 | 132044694 | 132047998 | LOC101929331 | | | | | |
| 9 | 132083294 | 132084882 | C9orf106 | | | | | |
| 9 | 132099201 | 132109756 | LINC01503 | | | | | |
| 9 | 132250938 | 132275965 | LINC00963 | | | | | |
| 9 | 132371162 | 132398410 | NTMT1 | | | | | |
| 9 | 132374503 | 132383055 | C9orf50 | | | | | |
| 9 | 132382491 | 132398410 | NTMT1 | | | | | |
| 9 | 132396882 | 132404448 | ASB6 | | | | | |
| 9 | 132427919 | 132480985 | PRRX2 | | | | | |
| 9 | 132500614 | 132515344 | PTGES | | | | | |
| 9 | 132565421 | 132573568 | TOR1B | | | | | |
| 9 | 132575220 | 132586441 | TOR1A | | | TOR1A | | |
| 9 | 132589563 | 132597572 | C9orf78 | | | | | |
| 9 | 132597695 | 132644117 | USP20 | | | | | |
| 9 | 132631883 | 132631950 | MIR6855 | | | | | |
| 9 | 132649465 | 132805473 | FNBP1 | | | | | |
| 9 | 132815984 | 132902448 | GPR107 | | | | | |
| 9 | 132902888 | 132906498 | LOC401554 | | | | | |
| 9 | 132934856 | 132999583 | NCS1 | | | | | |
| 9 | 133028157 | 133309510 | HMCN2 | | | | | |
| 9 | 133320093 | 133376661 | ASS1 | | | | | |
| 9 | 133452736 | 133454881 | LOC100272217 | | | | | |
| 9 | 133454959 | 133513739 | FUBP3 | | | | | |
| 9 | 133501683 | 133501750 | MIR6856 | | | | | |
| 9 | 133539980 | 133558384 | PRDM12 | | | | | |
| 9 | 133569146 | 133580281 | EXOSC2 | | | | | |
| 9 | 133589267 | 133763062 | ABL1 | | | ABL1 | | |
| 9 | 133768814 | 133772199 | QRFP | | | | | |
| 9 | 133777824 | 133814455 | FIBCD1 | | | | | |
| 9 | 133884503 | 133968446 | LAMC3 | | | | | |
| 9 | 133971862 | 133998539 | AIF1L | | | | | |
| 9 | 134000947 | 134110057 | NUP214 | | | | | |
| 9 | 134133464 | 134151906 | FAM78A | | | | | |

| | | | | | | | | |
|---|---|---|---|---|---|---|---|---|
| 9 | 134165080 | 134184649 | PLPP7 | | | | | |
| 9 | 134305476 | 134375575 | PRRC2B | | | | | |
| 9 | 134361051 | 134361137 | SNORD62A | | | | | |
| 9 | 134361051 | 134361137 | SNORD62B | | | | | |
| 9 | 134365872 | 134365958 | SNORD62A | | | | | |
| 9 | 134365872 | 134365958 | SNORD62B | | | | | |
| 9 | 134378288 | 134399193 | POMT1 | | | POMT1 | | |
| 9 | 134399182 | 134406662 | UCK1 | | | UCK1 | | |
| 9 | 134452156 | 134615331 | RAPGEF1 | | | | | |
| 9 | 134735496 | 134955274 | MED27 | | | | | |
| 9 | 135037333 | 135118220 | NTNG2 | | | | | |
| 9 | 135136826 | 135230372 | SETX | | | SETX | | |
| 9 | 135250934 | 135282238 | TTF1 | | | | | |
| 9 | 135285582 | 135448706 | CFAP77 | | | | | |
| 9 | 135457992 | 135465640 | BARHL1 | | | | | |
| 9 | 135469675 | 135545788 | DDX31 | | | | | |
| 9 | 135545421 | 135570342 | GTF3C4 | | | | | |
| 9 | 135600964 | 135754198 | AK8 | | | | | |
| 9 | 135753706 | 135765419 | C9orf9 | | | | | |
| 9 | 135766734 | 135820020 | TSC1 | | | TSC1 | | |
| 9 | 135820931 | 135867084 | GFI1B | | | | | |
| 9 | 135821093 | 135821158 | MIR548AW | | | | | |
| 9 | 135854097 | 135867084 | GFI1B | | | | | |
| 9 | 135895816 | 135895921 | SNORD141A | | | | | |
| 9 | 135895816 | 135895921 | SNORD141B | | | | | |
| 9 | 135903538 | 135905809 | LOC105376306 | | | | | |
| 9 | 135906061 | 135933890 | GTF3C5 | | | | | |
| 9 | 135927382 | 135927446 | MIR6877 | | | | | |
| 9 | 135937364 | 135947250 | CEL | | | | | |
| 9 | 135957925 | 135962478 | CELP | | | | | |
| 9 | 135973106 | 136024607 | RALGDS | | | | | |
| 9 | 136028334 | 136039332 | GBGT1 | | | | | |
| 9 | 136080665 | 136084637 | OBP2B | | | | | |
| 9 | 136130562 | 136150630 | ABO | | | | | |
| 9 | 136197542 | 136203047 | SURF6 | | | | | |
| 9 | 136207750 | 136215011 | MED22 | | | | | |
| 9 | 136215068 | 136218280 | RPL7A | | | | | |
| 9 | 136216250 | 136216325 | SNORD24 | | | | | |
| 9 | 136216948 | 136217019 | SNORD36B | | | | | |
| 9 | 136217310 | 136217382 | SNORD36A | | | | | |
| 9 | 136217700 | 136217767 | SNORD36C | | | | | |
| 9 | 136218659 | 136223361 | SURF1 | | | SURF1 | | |
| 9 | 136223420 | 136228041 | SURF2 | | | | | |
| 9 | 136228324 | 136244820 | SURF4 | | | | | |
| 9 | 136243283 | 136271220 | STKLD1 | | | | | |
| 9 | 136271181 | 136283292 | REXO4 | | | | | |
| 9 | 136279458 | 136324525 | ADAMTS13 | | ADAMTS13 | | | |
| 9 | 136325086 | 136335909 | CACFD1 | | | | | |
| 9 | 136336215 | 136344276 | SLC2A6 | | | | | |
| 9 | 136379707 | 136390068 | TMEM8C | | | | | |
| 9 | 136397285 | 136440641 | ADAMTSL2 | | ADAMTSL2 | | | |
| 9 | 136443536 | 136445368 | FAM163B | | | | | |
| 9 | 136501484 | 136522530 | DBH | DBH | | DBH | DBH | |
| 9 | 136528683 | 136605077 | SARDH | | SARDH | | | |
| 9 | 136627015 | 136857446 | VAV2 | | | | | |
| 9 | 136890560 | 136896719 | LINC00094 | | | | | |
| 9 | 136895445 | 136933141 | BRD3 | | | | | |
| 9 | 136919411 | 136923927 | LOC100130548 | | | | | |
| 9 | 137001209 | 137025094 | WDR5 | | | | | |
| 9 | 137029561 | 137029686 | RNU6ATAC | | | | | |
| 9 | 137218308 | 137332432 | RXRA | | | | | |
| 9 | 137271256 | 137271318 | MIR4669 | | | | | |
| 9 | 137298427 | 137332432 | RXRA | | | | | |
| 9 | 137533650 | 137736688 | COL5A1 | | | COL5A1 | | |
| 9 | 137711260 | 137764464 | LOC101448202 | | | | | |
| 9 | 137741020 | 137742490 | MIR3689A | | | | | |

| | | | | | | | | |
|---|---|---|---|---|---|---|---|---|
| 9 | 137741143 | 137741215 | MIR3689C | | | | | |
| 9 | 137741454 | 137741528 | MIR3689D1 | | | | | |
| 9 | 137741970 | 137742118 | MIR3689B | | | | | |
| 9 | 137742122 | 137742202 | MIR3689D2 | | | | | |
| 9 | 137742415 | 137742487 | MIR3689E | | | | | |
| 9 | 137742587 | 137742653 | MIR3689F | | | | | |
| 9 | 137772657 | 137779366 | FCN2 | | | | | |
| 9 | 137801430 | 137809806 | FCN1 | | | | | |
| 9 | 137967088 | 138013030 | OLFM1 | | | | | |
| 9 | 138073454 | 138079152 | LOC401557 | | | | | |
| 9 | 138235094 | 138238404 | C9orf62 | | | | | |
| 9 | 138354564 | 138380739 | PPP1R26 | | | | | |
| 9 | 138387025 | 138391761 | C9orf116 | | | | | |
| 9 | 138392476 | 138396519 | MRPS2 | | | MRPS2 | | |
| 9 | 138395118 | 138398293 | LOC101928525 | | | | | |
| 9 | 138413283 | 138418386 | LCN1 | | | | | |
| 9 | 138437984 | 138441815 | OBP2A | | | | | |
| 9 | 138453601 | 138458622 | PAEP | | | | | |
| 9 | 138466770 | 138478958 | LINC01502 | | | | | |
| 9 | 138515501 | 138531386 | GLT6D1 | | | | | |
| 9 | 138555167 | 138557755 | LCN9 | | | | | |
| 9 | 138585254 | 138591374 | SOHLH1 | | SOHLH1 | | | |
| 9 | 138594030 | 138684993 | KCNT1 | | KCNT1 | KCNT1 | | |
| 9 | 138700332 | 138799005 | CAMSAP1 | | | | | |
| 9 | 138824814 | 138853226 | UBAC1 | | | | | |
| 9 | 138898382 | 138987131 | NACC2 | | | | | |
| 9 | 139006426 | 139010731 | C9orf69 | | | | | |
| 9 | 139088095 | 139096975 | LHX3 | | | | | |
| 9 | 139098181 | 139137687 | QSOX2 | | | | | |
| 9 | 139216997 | 139221779 | DKFZP434A062 | | | | | |
| 9 | 139221931 | 139254057 | GPSM1 | | GPSM1 | | | |
| 9 | 139256351 | 139258263 | DNLZ | | | | | |
| 9 | 139258407 | 139268133 | CARD9 | | | | | |
| 9 | 139270028 | 139292889 | SNAPC4 | | | | | |
| 9 | 139296373 | 139305054 | SDCCAG3 | | | | | |
| 9 | 139305024 | 139318213 | PMPCA | | | PMPCA | | |
| 9 | 139323066 | 139334274 | INPP5E | | | | | |
| 9 | 139334547 | 139378211 | SEC16A | | | | | |
| 9 | 139377946 | 139380519 | C9orf163 | | | | | |
| 9 | 139388884 | 139440238 | NOTCH1 | | | | | |
| 9 | 139414019 | 139414078 | MIR4673 | | | | | |
| 9 | 139440624 | 139440711 | MIR4674 | | | | | |
| 9 | 139442293 | 139444196 | NALT1 | | | | | |
| 9 | 139543061 | 139554873 | HSPC324 | | | | | |
| 9 | 139553307 | 139567130 | EGFL7 | | | | | |
| 9 | 139565053 | 139565138 | MIR126 | | | | | |
| 9 | 139567594 | 139581911 | AGPAT2 | | | | | |
| 9 | 139607023 | 139619170 | FAM69B | | | | | |
| 9 | 139619045 | 139622636 | SNHG7 | | | | | |
| 9 | 139620555 | 139620689 | SNORA17B | | | | | |
| 9 | 139621198 | 139621331 | SNORA17A | | | | | |
| 9 | 139632618 | 139637411 | LCN10 | | | | | |
| 9 | 139638468 | 139642980 | LCN6 | | | | | |
| 9 | 139640612 | 139644363 | LOC100128593 | | | | | |
| 9 | 139641344 | 139641422 | MIR6722 | | | | | |
| 9 | 139648839 | 139652731 | LCN8 | | | | | |
| 9 | 139654085 | 139658965 | LCN15 | | | | | |
| 9 | 139685776 | 139687769 | TMEM141 | | | | | |
| 9 | 139690789 | 139703300 | CCDC183 | | | | | |
| 9 | 139702373 | 139735639 | RABL6 | | RABL6 | | | |
| 9 | 139725408 | 139725475 | MIR4292 | | | | | |
| 9 | 139738866 | 139741797 | C9orf172 | | | | | |
| 9 | 139743267 | 139745495 | PHPT1 | | | | | |
| 9 | 139746818 | 139755251 | MAMDC4 | | | | | |
| 9 | 139756565 | 139760788 | EDF1 | | | EDF1 | | |
| 9 | 139780964 | 139821067 | TRAF2 | | | TRAF2 | | |

| | | | | | | | | |
|---|---|---|---|---|---|---|---|---|
| 9 | 139781184 | 139781255 | MIR4479 | | | | | |
| 9 | 139834884 | 139839206 | FBXW5 | | | | | |
| 9 | 139839697 | 139841426 | C8G | | | | | |
| 9 | 139846767 | 139849949 | LCN12 | | | | | |
| 9 | 139871955 | 139876194 | PTGDS | | | | | |
| 9 | 139877444 | 139880210 | LCNL1 | | | | | |
| 9 | 139886869 | 139888428 | C9orf142 | | | | | |
| 9 | 139889059 | 139891024 | CLIC3 | | | | | |
| 9 | 139901685 | 139923374 | ABCA2 | | ABCA2 | ABCA2 | | |
| 9 | 139921915 | 139931234 | C9orf139 | | | | | |
| 9 | 139924625 | 139927292 | FUT7 | | | | | |
| 9 | 139933908 | 139940676 | NPDC1 | | | | | |
| 9 | 139942550 | 139948503 | ENTPD2 | | ENTPD2 | | | |
| 9 | 139956578 | 139965028 | SAPCD2 | | | | | |
| 9 | 139971952 | 139978990 | UAP1L1 | | | | | |
| 9 | 139979397 | 140003639 | MAN1B1 | | | | | |
| 9 | 140004991 | 140009195 | DPP7 | | | | | |
| 9 | 140033608 | 140063214 | GRIN1 | GRIN1 | GRIN1 | GRIN1 | GRIN1 | GRIN1 |
| 9 | 140063211 | 140064491 | LRRC26 | | | | | |
| 9 | 140063637 | 140063722 | MIR3621 | | | | | |
| 9 | 140065379 | 140066498 | TMEM210 | | | | | |
| 9 | 140069235 | 140083057 | ANAPC2 | | | | | |
| 9 | 140083053 | 140084822 | SSNA1 | | | | | |
| 9 | 140086068 | 140095163 | TPRN | | | | | |
| 9 | 140098534 | 140100090 | TMEM203 | | | | | |
| 9 | 140100118 | 140113813 | NDOR1 | | | | | |
| 9 | 140114698 | 140115775 | RNF208 | | | | | |
| 9 | 140119086 | 140120763 | CYSRT1 | | | | | |
| 9 | 140122017 | 140124090 | RNF224 | | | | | |
| 9 | 140125208 | 140131006 | SLC34A3 | | | | | |
| 9 | 140135710 | 140138159 | TUBB4B | | | | | |
| 9 | 140138034 | 140142244 | FAM166A | | | | | |
| 9 | 140144670 | 140147935 | C9orf173 | | | | | |
| 9 | 140149751 | 140168000 | NELFB | | | | | |
| 9 | 140172279 | 140177093 | TOR4A | | | | | |
| 9 | 140194082 | 140196703 | NRARP | | | | | |
| 9 | 140201345 | 140317714 | EXD3 | | | | | |
| 9 | 140317846 | 140328858 | NOXA1 | | | | | |
| 9 | 140328815 | 140335901 | ENTPD8 | | | | | |
| 9 | 140342022 | 140353786 | NSMF | | | | | |
| 9 | 140344477 | 140344538 | MIR7114 | | | | | |
| 9 | 140354403 | 140445021 | PNPLA7 | | | | | |
| 9 | 140446308 | 140447007 | MRPL41 | | | | | |
| 9 | 140449358 | 140473387 | DPH7 | | | | | |
| 9 | 140476530 | 140484937 | ZMYND19 | | | | | |
| 9 | 140500091 | 140513347 | ARRDC1 | | | | | |
| 9 | 140513443 | 140730578 | EHMT1 | | | EHMT1 | | |
| 9 | 140657473 | 140659224 | EHMT1-IT1 | | | | | |
| 9 | 140732870 | 140732968 | MIR602 | | | | | |
| 9 | 140762376 | 140787022 | LOC100133077 | | | | | |
| 9 | 140772240 | 141019076 | CACNA1B | | CACNA1B | CACNA1B | CACNA1B | |
| 9 | 140838081 | 140841512 | LOC105376331 | | | | | |
| 9 | 140934954 | 140939523 | LOC101928786 | | | | | |
| 9 | 141044564 | 141071885 | TUBBP5 | | | | | |
| 9 | 141090384 | 141093903 | LOC101928932 | | | | | |
| 9 | 141106636 | 141134172 | FAM157B | | | | | |
| 10 | 92827 | 95178 | TUBB8 | | | | | |
| 10 | 180404 | 295729 | ZMYND11 | | | | | |
| 10 | 320129 | 735608 | DIP2C | | | | | |
| 10 | 327976 | 328026 | MIR7641 | | | | | |
| 10 | 687628 | 687718 | MIR5699 | | | | | |
| 10 | 695887 | 711109 | PRR26 | | | | | |
| 10 | 852853 | 977645 | LARP4B | | | | | |
| 10 | 978965 | 988683 | LOC101927762 | | | | | |
| 10 | 1034348 | 1063708 | GTPBP4 | | | | | |
| 10 | 1064846 | 1090141 | IDI2 | | | | | |

| | | | | | | | | |
|---|---|---|---|---|---|---|---|---|
| 10 | 1085359 | 1102656 | IDI1 | | | | | |
| 10 | 1102775 | 1178237 | WDR37 | | | | | |
| 10 | 1205707 | 1210612 | LINC00200 | | | | | |
| 10 | 1223252 | 1599179 | ADARB2 | | | | | |
| 10 | 2047666 | 2056542 | LINC00700 | | | | | |
| 10 | 2118212 | 2118283 | MIR6072 | | | | | |
| 10 | 2342512 | 2357268 | LINC00701 | | | | | |
| 10 | 3109711 | 3178997 | PFKP | | | | | |
| 10 | 3176295 | 3176490 | SNORD142 | | | | | |
| 10 | 3179918 | 3190821 | PITRM1 | | | | | |
| 10 | 3360886 | 3792955 | LOC105376360 | | | | | |
| 10 | 3818187 | 3827473 | KLF6 | | | | | |
| 10 | 3876141 | 3876920 | LOC105376365 | | | | | |
| 10 | 4033351 | 4033451 | MIR6078 | | | | | |
| 10 | 4093917 | 4131205 | LOC101927964 | | | | | |
| 10 | 4249067 | 4286104 | LINC00702 | | | | | |
| 10 | 4426437 | 4452804 | LINC00703 | | | | | |
| 10 | 4692376 | 4720262 | LINC00704 | | | | | |
| 10 | 4698347 | 4704606 | LINC00705 | | | | | |
| 10 | 4868368 | 4890251 | AKR1E2 | | | | | |
| 10 | 4913858 | 4958465 | AKR1C6P | | | | | |
| 10 | 5005453 | 5020158 | AKR1C1 | | | | | |
| 10 | 5029967 | 5046206 | AKR1C2 | | | | | |
| 10 | 5090957 | 5149878 | AKR1C3 | | | AKR1C3 | | |
| 10 | 5196654 | 5227150 | AKR1C8P | | | | | |
| 10 | 5238797 | 5260910 | AKR1C4 | | | | | |
| 10 | 5406975 | 5416169 | UCN3 | | | | | |
| 10 | 5435060 | 5446793 | TUBAL3 | | | | | |
| 10 | 5454513 | 5501019 | NET1 | | | | | |
| 10 | 5540657 | 5541533 | CALML5 | | | | | |
| 10 | 5556206 | 5568231 | CALML3 | | | | | |
| 10 | 5636947 | 5638081 | LOC105376382 | | | | | |
| 10 | 5680819 | 5708558 | ASB13 | | | | | |
| 10 | 5726800 | 5805703 | FAM208B | | | | | |
| 10 | 5807185 | 5855512 | GDI2 | | | GDI2 | | |
| 10 | 5903688 | 5931860 | ANKRD16 | | | | | |
| 10 | 5931534 | 5979558 | FBXO18 | | | | | |
| 10 | 5994333 | 6020150 | IL15RA | | | | | |
| 10 | 6052656 | 6104333 | IL2RA | | | | | |
| 10 | 6130948 | 6159422 | RBM17 | | | | | |
| 10 | 6186840 | 6277507 | PFKFB3 | | | | | |
| 10 | 6194158 | 6194240 | MIR3155A | | | | | |
| 10 | 6194169 | 6194225 | MIR3155B | | | | | |
| 10 | 6244839 | 6277507 | PFKFB3 | | | | | |
| 10 | 6368506 | 6377943 | LOC399715 | | | | | |
| 10 | 6469104 | 6627323 | PRKCQ | | | | | |
| 10 | 6660677 | 6681743 | LOC101928150 | | | | | |
| 10 | 6677031 | 6677083 | MIR4454 | | | | | |
| 10 | 6816264 | 6821142 | LINC00706 | | | | | |
| 10 | 6821559 | 6884868 | LINC00707 | | | | | |
| 10 | 7200585 | 7453448 | SFMBT2 | | | | | |
| 10 | 7228641 | 7228746 | SNORD129 | | | | | |
| 10 | 7601231 | 7708961 | ITIH5 | | | | | |
| 10 | 7745235 | 7791483 | ITIH2 | | | | | |
| 10 | 7792924 | 7829990 | KIN | | | | | |
| 10 | 7830092 | 7849762 | ATP5C1 | | | ATP5C1 | | |
| 10 | 7860466 | 8057016 | TAF3 | | | | | |
| 10 | 8092412 | 8117164 | GATA3 | | | GATA3 | | |
| 10 | 8301294 | 8310268 | LINC00708 | | | | | |
| 10 | 8614709 | 8636354 | LOC105376398 | | | | | |
| 10 | 8939951 | 8956559 | LOC105755953 | | | | | |
| 10 | 9239328 | 9337556 | LOC101928272 | | | | | |
| 10 | 9317575 | 9329019 | LINC00709 | | | | | |
| 10 | 10100684 | 10105465 | LOC101928298 | | | | | |
| 10 | 10461500 | 10504312 | LOC101928322 | | | | | |
| 10 | 10826401 | 10836877 | SFTA1P | | | | | |

| | | | | | | | | |
|---|---|---|---|---|---|---|---|---|
| 10 | 10976902 | 10994126 | LINC00710 | | | | | |
| 10 | 11047258 | 11386673 | CELF2 | | | | | |
| 10 | 11502508 | 11653788 | USP6NL | | | | | |
| 10 | 11784355 | 11806065 | ECHDC3 | | | | | |
| 10 | 11865396 | 11936709 | PROSER2 | | | | | |
| 10 | 11962020 | 12085169 | UPF2 | | | | | |
| 10 | 12110915 | 12165227 | DHTKD1 | | | | | |
| 10 | 12171639 | 12211957 | SEC61A2 | | | | | |
| 10 | 12172758 | 12172815 | MIR548AK | | | | | |
| 10 | 12209572 | 12238143 | NUDT5 | | | | | |
| 10 | 12237960 | 12292589 | CDC123 | | | | | |
| 10 | 12391541 | 12877545 | CAMK1D | | | | | |
| 10 | 12620751 | 12620822 | MIR4480 | | | | | |
| 10 | 12695136 | 12695196 | MIR4481 | | | | | |
| 10 | 12938624 | 13141773 | CCDC3 | | | | | |
| 10 | 13142081 | 13180276 | OPTN | | | | | |
| 10 | 13203553 | 13253104 | MCM10 | | | | | |
| 10 | 13259268 | 13259375 | RNU6-2 | | | | | |
| 10 | 13263765 | 13276328 | UCMA | | | | | |
| 10 | 13319795 | 13342130 | PHYH | | | PHYH | | |
| 10 | 13359437 | 13390298 | SEPHS1 | | | | | |
| 10 | 13480483 | 13544976 | BEND7 | | | | | |
| 10 | 13628938 | 13672868 | PRPF18 | | | | | |
| 10 | 13685705 | 14372883 | FRMD4A | | | | | |
| 10 | 14116282 | 14129878 | LOC101928453 | | | | | |
| 10 | 14124945 | 14372883 | FRMD4A | | | | | |
| 10 | 14425198 | 14425276 | MIR4293 | | | | | |
| 10 | 14478574 | 14478660 | MIR1265 | | | | | |
| 10 | 14560555 | 14816896 | FAM107B | | | | | |
| 10 | 14861250 | 14879983 | CDNF | | | | | |
| 10 | 14880158 | 14913740 | HSPA14 | | | | | |
| 10 | 14920781 | 14946304 | SUV39H2 | | | | | |
| 10 | 14946609 | 14996431 | DCLRE1C | | | DCLRE1C | | |
| 10 | 15001437 | 15014850 | MEIG1 | | | | | |
| 10 | 15014562 | 15030046 | LOC105376430 | | | | | |
| 10 | 15085894 | 15115851 | OLAH | | | | | |
| 10 | 15117473 | 15130775 | ACBD7 | | | | | |
| 10 | 15137383 | 15139318 | C10orf111 | | | | | |
| 10 | 15139178 | 15146256 | RPP38 | | | | | |
| 10 | 15147770 | 15210709 | NMT2 | | | | | |
| 10 | 15196720 | 15197346 | PPIAP30 | | | | | |
| 10 | 15253643 | 15413058 | FAM171A1 | | | | | |
| 10 | 15555947 | 15762334 | ITGA8 | | | | | |
| 10 | 15820167 | 15902532 | FAM188A | | | | | |
| 10 | 16478941 | 16555744 | PTER | | | | | |
| 10 | 16555741 | 16564004 | C1QL3 | | | | | |
| 10 | 16632616 | 16859453 | RSU1 | | | | | |
| 10 | 16865964 | 17171816 | CUBN | | | | | |
| 10 | 17184981 | 17244070 | TRDMT1 | | | | | |
| 10 | 17256237 | 17279592 | VIM | | | | | |
| 10 | 17362675 | 17450285 | ST8SIA6 | | | | | |
| 10 | 17631957 | 17659373 | HACD1 | | | | | |
| 10 | 17683282 | 17758821 | STAM | | | | | |
| 10 | 17794259 | 17842866 | TMEM236 | | | | | |
| 10 | 17851341 | 18200091 | MRC1 | | | | | |
| 10 | 17887106 | 17887193 | MIR511 | | | | | |
| 10 | 18041226 | 18089854 | TMEM236 | | | | | |
| 10 | 18134035 | 18134122 | MIR511 | | | | | |
| 10 | 18240767 | 18299491 | SLC39A12 | | | | | |
| 10 | 18429605 | 18830688 | CACNB2 | | | | CACNB2 | |
| 10 | 18834135 | 18940566 | NSUN6 | | | | | |
| 10 | 18948312 | 18966940 | ARL5B | | | | | |
| 10 | 19337699 | 20023407 | MALRD1 | | | | | |
| 10 | 19999263 | 20017479 | LOC101928834 | | | | | |
| 10 | 20105371 | 20578784 | PLXDC2 | | | | | |
| 10 | 20840898 | 20840975 | MIR4675 | | | | | |

| | | | | | | | | |
|---|---|---|---|---|---|---|---|---|
| 10 | 21068902 | 21463116 | NEBL | | NEBL | NEBL | | |
| 10 | 21414691 | 21435488 | C10orf113 | | | | | |
| 10 | 21462918 | 21463852 | NEBL | | NEBL | NEBL | | |
| 10 | 21783420 | 21786213 | CASC10 | | | | | |
| 10 | 21785490 | 21785570 | MIR1915 | | | | | |
| 10 | 21802408 | 21814611 | SKIDA1 | | | | | |
| 10 | 21823100 | 22032559 | MLLT10 | | | | | |
| 10 | 22045476 | 22292650 | DNAJC1 | | | | | |
| 10 | 22497742 | 22498912 | EBLN1 | | | | | |
| 10 | 22541000 | 22547477 | LOC100130992 | | | | | |
| 10 | 22605311 | 22609246 | COMMD3 | | | | | |
| 10 | 22605311 | 22620414 | COMMD3-BMI1 | | | | | |
| 10 | 22610138 | 22620414 | BMI1 | | | | | |
| 10 | 22634373 | 22706539 | SPAG6 | | | | | |
| 10 | 22724353 | 22726858 | LOC100499489 | | | | | |
| 10 | 22823765 | 23003503 | PIP4K2A | | | PIP4K2A | | |
| 10 | 23216952 | 23327452 | ARMC3 | | | | | |
| 10 | 23384426 | 23410942 | MSRB2 | | | | | |
| 10 | 23481459 | 23483181 | PTF1A | | | | | |
| 10 | 23605519 | 23633772 | C10orf67 | | | | | |
| 10 | 23682333 | 23682396 | MIR1254 | | | | | |
| 10 | 23728197 | 23731310 | OTUD1 | | | | | |
| 10 | 23983674 | 24836777 | KIAA1217 | | | | | |
| 10 | 24564613 | 24564710 | MIR603 | | | | | |
| 10 | 24738361 | 24834245 | KIAA1217 | | | | | |
| 10 | 24872537 | 25012597 | ARHGAP21 | | | | | |
| 10 | 25137535 | 25241573 | PRTFDC1 | | | | | |
| 10 | 25270907 | 25351208 | ENKUR | | | | | |
| 10 | 25305507 | 25315593 | THNSL1 | | | THNSL1 | | |
| 10 | 25401986 | 25450162 | LINC01516 | | | | | |
| 10 | 25447000 | 25891157 | GPR158 | | | | | |
| 10 | 25940640 | 26021864 | LINC00836 | | | | | |
| 10 | 26213306 | 26222639 | LOC101929073 | | | | | |
| 10 | 26223001 | 26501465 | MYO3A | | | MYO3A | | |
| 10 | 26505235 | 26593491 | GAD2 | | | | | |
| 10 | 26727265 | 26856732 | APBB1IP | | | | | |
| 10 | 26866614 | 26868236 | LOC101929117 | | | | | |
| 10 | 26878793 | 26883251 | LINC00264 | | | | | |
| 10 | 26932036 | 26942383 | LINC00202-2 | | | | | |
| 10 | 26986594 | 27035726 | PDSS1 | | | | | |
| 10 | 27035524 | 27150016 | ABI1 | | | ABI1 | | |
| 10 | 27220134 | 27230930 | LINC00202-1 | | | | | |
| 10 | 27293044 | 27389427 | ANKRD26 | | ANKRD26 | | | |
| 10 | 27399039 | 27443349 | YME1L1 | | | YME1L1 | | |
| 10 | 27443752 | 27475848 | MASTL | | | MASTL | | |
| 10 | 27484142 | 27531068 | ACBD5 | | ACBD5 | | | |
| 10 | 27534781 | 27541235 | LRRC37A6P | | | | | |
| 10 | 27687116 | 27703297 | PTCHD3 | | | | | |
| 10 | 27793102 | 27831166 | RAB18 | | | RAB18 | | |
| 10 | 27961802 | 28056728 | MKX | | | | | |
| 10 | 28101092 | 28287979 | ARMC4 | | | | | |
| 10 | 28339922 | 28591995 | MPP7 | | | | | |
| 10 | 28362165 | 28362307 | SNORD130 | | | | | |
| 10 | 28578186 | 28578279 | MIR8086 | | | | | |
| 10 | 28721936 | 28784742 | LOC105376468 | | | | | |
| 10 | 28808845 | 28912041 | WAC | | | | | |
| 10 | 28835324 | 28835346 | MIR5586 | | | | | |
| 10 | 28966423 | 28971868 | BAMBI | | | | | |
| 10 | 29032578 | 29097148 | LINC01517 | | | | | |
| 10 | 29078239 | 29084979 | LINC00837 | | | | | |
| 10 | 29135336 | 29170826 | C10orf126 | | | | | |
| 10 | 29577989 | 29600158 | LYZL1 | | | | | |
| 10 | 29698462 | 30024730 | SVIL | | | | | |
| 10 | 29833932 | 29834026 | MIR604 | | | | | |
| 10 | 29891192 | 29891275 | MIR938 | | | | | |
| 10 | 30301728 | 30348488 | KIAA1462 | | | | | |

| | | | | | | | | |
|---|---|---|---|---|---|---|---|---|
| 10 | 30500780 | 30521619 | LOC101929279 | | | | | |
| 10 | 30598729 | 30638267 | MTPAP | | | | | |
| 10 | 30653255 | 30660836 | GOLGA2P6 | | | | | |
| 10 | 30657525 | 30657594 | MIR7162 | | | | | |
| 10 | 30722949 | 30750762 | MAP3K8 | | | MAP3K8 | | |
| 10 | 30900707 | 30918647 | LYZL2 | | | | | |
| 10 | 30981202 | 31005942 | SVILP1 | | | | | |
| 10 | 31012147 | 31012669 | LOC105376480 | | | | | |
| 10 | 31133564 | 31320866 | ZNF438 | | | | | |
| 10 | 31476811 | 31550481 | LOC101929352 | | | | | |
| 10 | 31605456 | 31818742 | ZEB1 | | | | | |
| 10 | 32094325 | 32217804 | ARHGAP12 | | | | | |
| 10 | 32297937 | 32345371 | KIF5B | | | KIF5B | | |
| 10 | 32556643 | 32636146 | EPC1 | | | EPC1 | | |
| 10 | 32636291 | 32663416 | LOC102031319 | | | | | |
| 10 | 32723296 | 32734984 | LOC101929431 | | | | | |
| 10 | 32735009 | 33171792 | CCDC7 | | | | | |
| 10 | 33189245 | 33247293 | ITGB1 | | | | | |
| 10 | 33190261 | 33190400 | SNORA86 | | | | | |
| 10 | 33466418 | 33623833 | NRP1 | | | | | |
| 10 | 34048640 | 34061608 | LINC00838 | | | | | |
| 10 | 34398487 | 35105350 | PARD3 | | | PARD3 | | |
| 10 | 35297478 | 35379570 | CUL2 | | | | | |
| 10 | 35368525 | 35368608 | MIR3611 | | | | | |
| 10 | 35415768 | 35501886 | CREM | | | | CREM | |
| 10 | 35535908 | 35861598 | CCNY | | | | | |
| 10 | 35894337 | 35897863 | GJD4 | | | | | |
| 10 | 35927176 | 35930362 | FZD8 | | | FZD8 | | |
| 10 | 35930099 | 35930180 | MIR4683 | | | | | |
| 10 | 36067229 | 36089848 | PCAT5 | | | | | |
| 10 | 37414784 | 37521495 | ANKRD30A | | | | | |
| 10 | 37598113 | 37635956 | LINC00993 | | | | | |
| 10 | 37890365 | 37891859 | MTRNR2L7 | | | | | |
| 10 | 38065453 | 38146564 | ZNF248 | | | | | |
| 10 | 38082865 | 38084103 | ZNF33BP1 | | | | | |
| 10 | 38117692 | 38147012 | ZNF248 | | | | | |
| 10 | 38238794 | 38265453 | ZNF25 | | | ZNF25 | | |
| 10 | 38299577 | 38348995 | ZNF33A | | | | | |
| 10 | 38383263 | 38412278 | ZNF37A | | | | | |
| 10 | 38464598 | 38503273 | LOC100129055 | | | | | |
| 10 | 38645307 | 38667433 | HSD17B7P2 | | | | | |
| 10 | 38671997 | 38691780 | SEPT7P9 | | | | | |
| 10 | 38717073 | 38741081 | LINC00999 | | | | | |
| 10 | 38989726 | 38991371 | ACTR3BP5 | | | | | |
| 10 | 42827313 | 42863493 | LOC441666 | | | | | |
| 10 | 42903621 | 42967688 | CCNYL2 | | | | | |
| 10 | 42970938 | 42990785 | LINC00839 | | | | | |
| 10 | 43008960 | 43048318 | ZNF37BP | | | | | |
| 10 | 43069630 | 43134285 | ZNF33B | | | | | |
| 10 | 43168460 | 43187207 | LINC01518 | | | | | |
| 10 | 43250589 | 43265069 | LOC105378269 | | | | | |
| 10 | 43277953 | 43330385 | BMS1 | | | | | |
| 10 | 43366968 | 43369508 | LOC101929445 | | | | | |
| 10 | 43474464 | 43476955 | LINC01264 | | | | | |
| 10 | 43493010 | 43493129 | MIR5100 | | | | | |
| 10 | 43572516 | 43625797 | RET | | RET | RET | | |
| 10 | 43633892 | 43680754 | CSGALNACT2 | | | | | |
| 10 | 43689980 | 43762367 | RASGEF1A | | | | | |
| 10 | 43867091 | 43871783 | FXYD4 | | | | | |
| 10 | 43881064 | 43904696 | HNRNPF | | | | | |
| 10 | 43932573 | 43978007 | ZNF487 | | | | | |
| 10 | 44051792 | 44070066 | ZNF239 | | | | | |
| 10 | 44101854 | 44113352 | ZNF485 | | | | | |
| 10 | 44124264 | 44143467 | ZNF32 | | | | | |
| 10 | 44273014 | 44274350 | LOC102724264 | | | | | |
| 10 | 44282859 | 44285865 | HNRNPA3P1 | | | | | |

| | | | | | | | | |
|---|---|---|---|---|---|---|---|---|
| 10 | 44340753 | 44346070 | LINC00619 | | | | | |
| 10 | 44354858 | 44390881 | LINC00840 | | | | | |
| 10 | 44404751 | 44465355 | LINC00841 | | | | | |
| 10 | 44788197 | 44790097 | C10orf142 | | | | | |
| 10 | 44865600 | 44880545 | CXCL12 | | | | | |
| 10 | 45306471 | 45432458 | TMEM72 | | | | | |
| 10 | 45455218 | 45490172 | RASSF4 | | | | | |
| 10 | 45471708 | 45474330 | C10orf10 | | | | | |
| 10 | 45489379 | 45496358 | C10orf25 | | | | | |
| 10 | 45496272 | 45500777 | ZNF22 | | | | | |
| 10 | 45594923 | 45650044 | RSU1P2 | | | | | |
| 10 | 45650109 | 45681489 | ANKRD30BP3 | | | | | |
| 10 | 45659461 | 45659536 | MIR3156 | | | | | |
| 10 | 45798101 | 45811056 | OR13A1 | | | | | |
| 10 | 45869623 | 45941567 | ALOX5 | | | | | |
| 10 | 45940015 | 45948569 | LOC102724323 | | | | | |
| 10 | 45950032 | 46090354 | MARCH8 | | | | | |
| 10 | 46110948 | 46168261 | ZFAND4 | | | | | |
| 10 | 46222647 | 46288412 | FAM21C | | | | | |
| 10 | 46321039 | 46349325 | AGAP4 | | | | | |
| 10 | 46550122 | 46641003 | PTPN20 | | | | | |
| 10 | 46737612 | 46762892 | BMS1P5 | | | | | |
| 10 | 46897640 | 46939145 | FAM35BP | | | | | |
| 10 | 46951471 | 46966835 | LOC102724593 | | | | | |
| 10 | 46955443 | 46970601 | SYT15 | | | | | |
| 10 | 46993545 | 47000568 | GPRIN2 | | | | | |
| 10 | 47083533 | 47088320 | NPY4R | | | | | |
| 10 | 47086724 | 47088173 | CH17-360D5.1 | | | | | |
| 10 | 47096453 | 47151400 | LINC00842 | | | | | |
| 10 | 47133294 | 47133836 | HNRNPA1P33 | | | | | |
| 10 | 47157983 | 47174143 | ANXA8 | | | | | |
| 10 | 47177203 | 47181688 | FAM25C | | | | | |
| 10 | 47177221 | 47181688 | FAM25G | | | | | |
| 10 | 47191843 | 47213626 | AGAP9 | | | | | |
| 10 | 47225570 | 47243511 | BMS1P6 | | | | | |
| 10 | 47379719 | 47421238 | FAM35DP | | | | | |
| 10 | 47590260 | 47640844 | ANTXRLP1 | | | | | |
| 10 | 47658232 | 47701443 | ANTXRL | | | | | |
| 10 | 47740303 | 47744822 | FAM25BP | | | | | |
| 10 | 47746849 | 47763040 | ANXA8L1 | | | | | |
| 10 | 48155942 | 48158691 | CTSLP2 | | | | | |
| 10 | 48185838 | 48203780 | BMS1P6 | | | | | |
| 10 | 48215723 | 48237508 | AGAP9 | | | | | |
| 10 | 48247661 | 48252125 | FAM25G | | | | | |
| 10 | 48247661 | 48252143 | FAM25C | | | | | |
| 10 | 48255203 | 48271368 | ANXA8 | | | | | |
| 10 | 48324787 | 48332197 | LOC107001062 | | | | | |
| 10 | 48324886 | 48325973 | LOC105378292 | | | | | |
| 10 | 48355088 | 48373866 | ZNF488 | | | | | |
| 10 | 48381486 | 48390991 | RBP3 | | | | | |
| 10 | 48413091 | 48416872 | GDF2 | | | | | |
| 10 | 48425787 | 48439138 | GDF10 | | | | | |
| 10 | 48737041 | 48827924 | PTPN20 | | | | | |
| 10 | 48844035 | 48862688 | FRMPD2B | | | | | |
| 10 | 48927373 | 48952629 | BMS1P5 | | | | | |
| 10 | 48952591 | 48980339 | GLUD1P7 | | | | | |
| 10 | 49203354 | 49207825 | FAM25C | | | | | |
| 10 | 49203372 | 49207825 | FAM25G | | | | | |
| 10 | 49218158 | 49239658 | AGAP12P | | | | | |
| 10 | 49364600 | 49383240 | FRMPD2B | | | | | |
| 10 | 49364601 | 49482941 | FRMPD2 | | | | | |
| 10 | 49514681 | 49647402 | MAPK8 | | | | | |
| 10 | 49654067 | 49864310 | ARHGAP22 | | | | | |
| 10 | 49893517 | 50191001 | WDFY4 | | | | | |
| 10 | 50117528 | 50122280 | LRRC18 | | | | | |
| 10 | 50193556 | 50193632 | MIR4294 | | | | | |

| | | | | | | | | |
|---|---|---|---|---|---|---|---|---|
| 10 | 50222289 | 50323577 | VSTM4 | | | | | |
| 10 | 50329883 | 50342065 | FAM170B | | | | | |
| 10 | 50362768 | 50396445 | C10orf128 | | | | | |
| 10 | 50504327 | 50535537 | C10orf71 | | | | | |
| 10 | 50574160 | 50604062 | DRGX | | | | | |
| 10 | 50662525 | 50747169 | ERCC6 | | | ERCC6 | | |
| 10 | 50723150 | 50732327 | PGBD3 | | | | | |
| 10 | 50723150 | 50747584 | ERCC6-PGBD3 | | | | | |
| 10 | 50817140 | 50873150 | CHAT | | | | | |
| 10 | 50818346 | 50820766 | SLC18A3 | | | SLC18A3 | | |
| 10 | 50821153 | 50873150 | CHAT | | | | | |
| 10 | 50887683 | 50916956 | C10orf53 | | | | | |
| 10 | 50942686 | 50970425 | OGDHL | | | | | |
| 10 | 51026320 | 51371371 | PARG | | | PARG | | |
| 10 | 51253907 | 51371344 | PARGP1 | | | | | |
| 10 | 51371378 | 51734610 | TIMM23B | | | | | |
| 10 | 51464143 | 51486327 | AGAP7P | | | | | |
| 10 | 51549552 | 51562518 | MSMB | | | | | |
| 10 | 51565107 | 51590734 | NCOA4 | | | | | |
| 10 | 51592080 | 51623386 | TIMM23 | | | | | |
| 10 | 51748077 | 51770259 | AGAP6 | | | | | |
| 10 | 51780941 | 51827563 | FAM21EP | | | | | |
| 10 | 51827647 | 51893269 | FAM21A | | | | | |
| 10 | 51946999 | 52008370 | ASAH2 | | | | | |
| 10 | 52065344 | 52391396 | SGMS1 | | | | | |
| 10 | 52499687 | 52514569 | ASAH2B | | | | | |
| 10 | 52559168 | 52645435 | A1CF | | | | | |
| 10 | 52750910 | 54058110 | PRKG1 | | | PRKG1 | PRKG1 | |
| 10 | 52822338 | 52828312 | LOC102724719 | | | | | |
| 10 | 52834233 | 54058110 | PRKG1 | | | PRKG1 | PRKG1 | |
| 10 | 53059332 | 53059415 | MIR605 | | | | | |
| 10 | 53455245 | 53459355 | CSTF2T | | | | | |
| 10 | 54056607 | 54073888 | PRKG1 | | | PRKG1 | PRKG1 | |
| 10 | 54074040 | 54077417 | DKK1 | | | | | |
| 10 | 54210636 | 54230293 | LINC01468 | | | | | |
| 10 | 54525139 | 54531460 | MBL2 | | | | | |
| 10 | 55562532 | 56561051 | PCDH15 | | | PCDH15 | | |
| 10 | 56245989 | 56415811 | LOC105378311 | | | | | |
| 10 | 57358749 | 57360487 | MTRNR2L5 | | | | | |
| 10 | 58117198 | 58121034 | ZWINT | | | | | |
| 10 | 59064238 | 59064319 | MIR3924 | | | | | |
| 10 | 59951277 | 60027694 | IPMK | | | | | |
| 10 | 60028861 | 60049019 | CISD1 | | | | | |
| 10 | 60094738 | 60130513 | UBE2D1 | | | | | |
| 10 | 60144902 | 60158990 | TFAM | | | | | |
| 10 | 60272903 | 60591197 | BICC1 | | | | | |
| 10 | 60474774 | 60477293 | FAM133CP | | | | | |
| 10 | 60759277 | 60761377 | LINC00844 | | | | | |
| 10 | 60933722 | 60936224 | CCEPR | | | | | |
| 10 | 60936347 | 61007534 | PHYHIPL | | | | | |
| 10 | 61005888 | 61122661 | FAM13C | | | | | |
| 10 | 61410521 | 61469649 | SLC16A9 | | | | | |
| 10 | 61496747 | 61513203 | MRLN | | | | | |
| 10 | 61548505 | 61666414 | CCDC6 | | | | | |
| 10 | 61717974 | 61720671 | LINC01553 | | | | | |
| 10 | 61786055 | 62493284 | ANK3 | | ANK3 | ANK3 | | ANK3 |
| 10 | 62538088 | 62554610 | CDK1 | | | | | |
| 10 | 62629197 | 62761198 | RHOBTB1 | | | | | |
| 10 | 62776032 | 62786175 | LINC00845 | | | | | |
| 10 | 63166399 | 63253189 | TMEM26 | | | | | |
| 10 | 63422718 | 63526091 | C10orf107 | | | | | |
| 10 | 63661012 | 63856707 | ARID5B | | | ARID5B | | |
| 10 | 63736060 | 63736088 | MIR548AV | | | | | |
| 10 | 63808969 | 63856707 | ARID5B | | | ARID5B | | |
| 10 | 63952844 | 64028622 | RTKN2 | | | | | |
| 10 | 64099346 | 64134886 | LOC283045 | | | | | |

| | | | | | | | | |
|---|---|---|---|---|---|---|---|---|
| 10 | 64133915 | 64431771 | ZNF365 | | ZNF365 | | | |
| 10 | 64564515 | 64568239 | ADO | | | | | |
| 10 | 64571755 | 64578927 | EGR2 | | | EGR2 | | |
| 10 | 64893006 | 64914791 | NRBF2 | | | | | |
| 10 | 64926980 | 65281835 | JMJD1C | | | | | |
| 10 | 65132716 | 65132808 | MIR1296 | | | | | |
| 10 | 65190334 | 65190962 | LOC105378330 | | | | | |
| 10 | 65224988 | 65226322 | JMJD1C | | | | | |
| 10 | 65281122 | 65384883 | REEP3 | | | | | |
| 10 | 66585284 | 66586634 | ANXA2P3 | | | | | |
| 10 | 66660893 | 66684276 | LOC101928887 | | | | | |
| 10 | 67331182 | 67526299 | LINC01515 | | | | | |
| 10 | 67672275 | 69455949 | CTNNA3 | | CTNNA3 | | | |
| 10 | 68653867 | 68771911 | LOC101928961 | | | | | |
| 10 | 68685791 | 68861309 | LRRTM3 | | | LRRTM3 | | |
| 10 | 68935242 | 69425443 | CTNNA3 | | CTNNA3 | | | |
| 10 | 69163108 | 69163168 | MIR7151 | | | | | |
| 10 | 69556426 | 69597937 | DNAJC12 | | | | | |
| 10 | 69644426 | 69678147 | SIRT1 | | | | | |
| 10 | 69681655 | 69835103 | HERC4 | | | | | |
| 10 | 69769703 | 69770640 | POU5F1P5 | | | | | |
| 10 | 69828758 | 69835103 | HERC4 | | | | | |
| 10 | 69865873 | 69971773 | MYPN | | | | | |
| 10 | 69990351 | 69991870 | ATOH7 | | ATOH7 | | | |
| 10 | 70042416 | 70092684 | PBLD | | | | | |
| 10 | 70091767 | 70102953 | HNRNPH3 | | | | | |
| 10 | 70100863 | 70167051 | RUFY2 | | | | | |
| 10 | 70173820 | 70231878 | DNA2 | | | | | |
| 10 | 70242089 | 70287280 | SLC25A16 | | | | | |
| 10 | 70320116 | 70454239 | TET1 | | | | | |
| 10 | 70480900 | 70552134 | CCAR1 | | | | | |
| 10 | 70514928 | 70514995 | SNORD98 | | | | | |
| 10 | 70519074 | 70519171 | MIR1254 | | | | | |
| 10 | 70587293 | 70655209 | STOX1 | | STOX1 | | | |
| 10 | 70661033 | 70706603 | DDX50 | | | | | |
| 10 | 70715878 | 70744825 | DDX21 | | | | | |
| 10 | 70748476 | 70776739 | KIAA1279 | | | | | |
| 10 | 70847827 | 70864567 | SRGN | | | | | |
| 10 | 70883908 | 70934172 | VPS26A | | | VPS26A | | |
| 10 | 70939959 | 70968854 | SUPV3L1 | | | | | |
| 10 | 70975088 | 70992246 | LOC101928994 | | | | | |
| 10 | 70980058 | 71027315 | HKDC1 | | | | | |
| 10 | 71029755 | 71161637 | HK1 | | | HK1 | | |
| 10 | 71163957 | 71176674 | TACR2 | | | | | |
| 10 | 71211225 | 71267423 | TSPAN15 | | | | | |
| 10 | 71331790 | 71333210 | NEUROG3 | | | | | |
| 10 | 71390002 | 71393355 | C10orf35 | | | | | |
| 10 | 71561643 | 71718904 | COL13A1 | | | | | |
| 10 | 71812356 | 71872040 | H2AFY2 | | | | | |
| 10 | 71872022 | 71892690 | AIFM2 | | | | | |
| 10 | 71897736 | 71906496 | TYSND1 | | | | | |
| 10 | 71909960 | 71930285 | SAR1A | | | | | |
| 10 | 71962585 | 71993190 | PPA1 | | | | | |
| 10 | 72014712 | 72043450 | NPFFR1 | | | | | |
| 10 | 72058725 | 72142406 | LRRC20 | | | | | |
| 10 | 72163860 | 72188374 | EIF4EBP2 | | | | | |
| 10 | 72191691 | 72201465 | NODAL | | | | | |
| 10 | 72238563 | 72328206 | PALD1 | | | | | |
| 10 | 72357103 | 72362531 | PRF1 | | | | | |
| 10 | 72432558 | 72522195 | ADAMTS14 | | ADAMTS14 | | | |
| 10 | 72530993 | 72545175 | TBATA | | | | | |
| 10 | 72575703 | 72640932 | SGPL1 | | | | | |
| 10 | 72643264 | 72648543 | PCBD1 | | | | | |
| 10 | 72698809 | 72715398 | LOC105378349 | | | | | |
| 10 | 72972291 | 72977985 | UNC5B | | | | | |
| 10 | 73079009 | 73123147 | SLC29A3 | | | | | |

| | | | | | | | | |
|---|---|---|---|---|---|---|---|---|
| 10 | 73156690 | 73575704 | CDH23 | | CDH23 | | | CDH23 |
| 10 | 73267909 | 73271677 | LOC102723377 | | | | | |
| 10 | 73471457 | 73497581 | C10orf105 | | | | | |
| 10 | 73507313 | 73533337 | C10orf54 | | | | | |
| 10 | 73550503 | 73550557 | MIR7152 | | | | | |
| 10 | 73555527 | 73575704 | CDH23 | | CDH23 | | | CDH23 |
| 10 | 73576054 | 73611082 | PSAP | | | PSAP | | |
| 10 | 73724119 | 73773322 | CHST3 | | | | | |
| 10 | 73818791 | 73848790 | SPOCK2 | | | | | |
| 10 | 73855789 | 73976892 | ASCC1 | | | | | |
| 10 | 73975757 | 73995618 | ANAPC16 | | | | | |
| 10 | 74033676 | 74035797 | DDIT4 | | | | | |
| 10 | 74092587 | 74114907 | DNAJB12 | | | | | |
| 10 | 74127083 | 74385949 | MICU1 | | | | | |
| 10 | 74451888 | 74647452 | MCU | | | | | |
| 10 | 74480786 | 74480858 | MIR4676 | | | | | |
| 10 | 74653313 | 74692794 | OIT3 | | | | | |
| 10 | 74694519 | 74714577 | PLA2G12B | | | | | |
| 10 | 74766979 | 74856732 | P4HA1 | | | | | |
| 10 | 74870132 | 74891586 | NUDT13 | | | | | |
| 10 | 74894281 | 74927853 | ECD | | | | | |
| 10 | 74927876 | 75001939 | FAM149B1 | | | | | |
| 10 | 75001713 | 75036742 | DNAJC9 | | | | | |
| 10 | 75008600 | 75012451 | MRPS16 | | | | | |
| 10 | 75012498 | 75036742 | DNAJC9 | | | | | |
| 10 | 75013515 | 75118617 | CFAP70 | | | | | |
| 10 | 75135188 | 75173841 | ANXA7 | | | | | |
| 10 | 75183336 | 75193319 | MSS51 | | | | | |
| 10 | 75196185 | 75267067 | PPP3CB | | | | | |
| 10 | 75257295 | 75335433 | USP54 | | | | | |
| 10 | 75391369 | 75401515 | MYOZ1 | | | | | |
| 10 | 75404638 | 75415863 | SYNPO2L | | | SYNPO2L | | |
| 10 | 75434032 | 75457554 | AGAP5 | | | | | |
| 10 | 75458908 | 75490272 | BMS1P4 | | | | | |
| 10 | 75490319 | 75491529 | GLUD1P3 | | | | | |
| 10 | 75504130 | 75531933 | SEC24C | | | | | |
| 10 | 75532048 | 75535976 | FUT11 | | | | | |
| 10 | 75541807 | 75543406 | CHCHD1 | | | | | |
| 10 | 75545381 | 75561157 | ZSWIM8 | | | | | |
| 10 | 75561668 | 75571589 | NDST2 | | | | | |
| 10 | 75572258 | 75634349 | CAMK2G | | | | | |
| 10 | 75669726 | 75682535 | C10orf55 | | | | | |
| 10 | 75670858 | 75677258 | PLAU | PLAU | | | | |
| 10 | 75757871 | 75879914 | VCL | | | | | |
| 10 | 75880014 | 75910826 | AP3M1 | | | | | |
| 10 | 75910942 | 76469061 | ADK | | | | | |
| 10 | 76266255 | 76289082 | LOC102723439 | | | | | |
| 10 | 76586170 | 76792380 | KAT6B | | | | | |
| 10 | 76672543 | 76696160 | LOC101929165 | | | | | |
| 10 | 76797593 | 76818272 | DUPD1 | | | | | |
| 10 | 76854189 | 76859248 | DUSP13 | | | | | |
| 10 | 76871392 | 76941881 | SAMD8 | | | | | |
| 10 | 76969911 | 76991207 | VDAC2 | | | VDAC2 | | |
| 10 | 76993728 | 76995770 | COMTD1 | | | | | |
| 10 | 77039483 | 77168740 | ZNF503 | | | | | |
| 10 | 77168914 | 77171082 | LOC101929234 | | | | | |
| 10 | 77191403 | 78317133 | C10orf11 | | | | | |
| 10 | 77312215 | 77312311 | MIR606 | | | | | |
| 10 | 77407266 | 78317133 | C10orf11 | | | | | |
| 10 | 77472212 | 77503606 | LOC105378367 | | | | | |
| 10 | 77542518 | 78317133 | C10orf11 | | | | | |
| 10 | 78629358 | 79397577 | KCNMA1 | | | KCNMA1 | KCNMA1 | KCNMA1 |
| 10 | 79550548 | 79689583 | DLG5 | | | | | |
| 10 | 79734906 | 79789298 | POLR3A | | | | | |
| 10 | 79793517 | 79816571 | RPS24 | | | | | |
| 10 | 80008381 | 80012402 | LINC00856 | | | | | |

| | | | | | | | | |
|---|---|---|---|---|---|---|---|---|
| 10 | 80027084 | 80039970 | LINC00595 | | | | | |
| 10 | 80703082 | 81076285 | ZMIZ1 | | | | | |
| 10 | 81107219 | 81115089 | PPIF | | | | | |
| 10 | 81142082 | 81205383 | ZCCHC24 | | | | | |
| 10 | 81272356 | 81276192 | EIF5AL1 | | | | | |
| 10 | 81315607 | 81320163 | SFTPA2 | | | | | |
| 10 | 81370694 | 81375199 | SFTPA1 | | | | | |
| 10 | 81388513 | 81422941 | LOC105378385 | | | | | |
| 10 | 81442730 | 81448650 | BEND3P3 | | | | | |
| 10 | 81451255 | 81586350 | NUTM2B | | | | | |
| 10 | 81585657 | 81587358 | LOC642361 | | | | | |
| 10 | 81664653 | 81691557 | BMS1P21 | | | | | |
| 10 | 81679933 | 81682875 | MBL1P | | | | | |
| 10 | 81697495 | 81708861 | SFTPD | | | | | |
| 10 | 81805988 | 81852307 | TMEM254 | | | | | |
| 10 | 81892257 | 81904784 | PLAC9 | | | | | |
| 10 | 81914879 | 81965433 | ANXA11 | | | | | |
| 10 | 81967465 | 81979413 | LINC00857 | | | | | |
| 10 | 82031575 | 82049434 | MAT1A | | | MAT1A | | |
| 10 | 82095860 | 82116514 | DYDC1 | | | | | |
| 10 | 82104500 | 82127829 | DYDC2 | | | | | |
| 10 | 82167584 | 82192753 | FAM213A | | | | | |
| 10 | 82214037 | 82282391 | TSPAN14 | | | | | |
| 10 | 82289352 | 82295698 | LOC101929574 | | | | | |
| 10 | 82291005 | 82292879 | LOC102723703 | | | | | |
| 10 | 82297657 | 82406316 | SH2D4B | | | | | |
| 10 | 83635069 | 83992676 | NRG3 | | | NRG3 | | |
| 10 | 85432161 | 85436845 | LOC105378397 | | | | | |
| 10 | 85899184 | 85913311 | GHITM | | | | | |
| 10 | 85926983 | 85931832 | HOST2 | | | | | |
| 10 | 85933553 | 85945050 | C10orf99 | | | | | |
| 10 | 85954390 | 85979376 | CDHR1 | | CDHR1 | | | |
| 10 | 85980248 | 85985345 | LRIT2 | | | | | |
| 10 | 85991275 | 86001217 | LRIT1 | | | | | |
| 10 | 86004808 | 86018944 | RGR | | | | | |
| 10 | 86039735 | 86054415 | LINC00858 | | | | | |
| 10 | 86088344 | 86278277 | CCSER2 | | | | | |
| 10 | 86953176 | 86958694 | LINC01519 | | | | | |
| 10 | 87191698 | 87192528 | LOC101929646 | | | | | |
| 10 | 87192618 | 87208713 | LOC101929662 | | | | | |
| 10 | 87209348 | 87251758 | LINC01520 | | | | | |
| 10 | 87337487 | 88126250 | GRID1 | | GRID1 | GRID1 | GRID1 | GRID1 |
| 10 | 88024450 | 88024545 | MIR346 | | | | | |
| 10 | 88195012 | 88281541 | WAPL | | | | | |
| 10 | 88414313 | 88426216 | OPN4 | | | | | |
| 10 | 88428205 | 88495824 | LDB3 | | | | | |
| 10 | 88516395 | 88684945 | BMPR1A | | | BMPR1A | | |
| 10 | 88695297 | 88717425 | MMRN2 | | | | | |
| 10 | 88718287 | 88723017 | SNCG | | SNCG | SNCG | | |
| 10 | 88728187 | 88730666 | ADIRF | | | | | |
| 10 | 88730497 | 88769960 | AGAP11 | | | | | |
| 10 | 88780045 | 88784487 | FAM25A | | | | | |
| 10 | 88809958 | 88854804 | GLUD1 | | GLUD1 | GLUD1 | | |
| 10 | 88854952 | 88951222 | FAM35A | | | | | |
| 10 | 88985204 | 89102315 | NUTM2A | | | | | |
| 10 | 89102167 | 89103331 | LOC439994 | | | | | |
| 10 | 89117476 | 89130452 | NUTM2D | | | | | |
| 10 | 89156330 | 89167457 | LINC00864 | | | | | |
| 10 | 89263637 | 89263711 | MIR4678 | | | | | |
| 10 | 89264222 | 89313218 | MINPP1 | | | | | |
| 10 | 89419475 | 89507462 | PAPSS2 | | | | | |
| 10 | 89512874 | 89577917 | ATAD1 | | | | | |
| 10 | 89578069 | 89605369 | CFL1P1 | | | | | |
| 10 | 89618917 | 89623194 | KLLN | | | | | |
| 10 | 89623194 | 89731687 | PTEN | | | PTEN | | |
| 10 | 90033620 | 90343082 | RNLS | | | | | |

| | | | | | | | | |
|---|---|---|---|---|---|---|---|---|
| 10 | 90346518 | 90366733 | LIPJ | | | | | |
| 10 | 90424145 | 90438572 | LIPF | | | | | |
| 10 | 90484300 | 90512513 | LIPK | | | | | |
| 10 | 90521162 | 90537999 | LIPN | | | | | |
| 10 | 90562486 | 90580303 | LIPM | | | | | |
| 10 | 90579658 | 90611732 | ANKRD22 | | | | | |
| 10 | 90639943 | 90683259 | STAMBPL1 | | | | | |
| 10 | 90692440 | 90751147 | ACTA2 | | | ACTA2 | | |
| 10 | 90750287 | 90752732 | FAS | | | | | |
| 10 | 90823091 | 90823167 | MIR4679 | | | | | |
| 10 | 90965693 | 90967071 | CH25H | | | | | |
| 10 | 90973325 | 91011796 | LIPA | | LIPA | LIPA | | |
| 10 | 91061705 | 91069033 | IFIT2 | | | | | |
| 10 | 91087575 | 91100725 | IFIT3 | | | | | |
| 10 | 91137812 | 91144962 | IFIT1B | | | | | |
| 10 | 91152302 | 91166244 | IFIT1 | | | | | |
| 10 | 91174324 | 91180759 | IFIT5 | | | | | |
| 10 | 91190050 | 91227908 | SLC16A12 | | | SLC16A12 | | |
| 10 | 91339253 | 91405329 | PANK1 | | | PANK1 | | |
| 10 | 91352503 | 91352584 | MIR107 | | | | | |
| 10 | 91451056 | 91457685 | FLJ37201 | | | | | |
| 10 | 91461346 | 91534700 | KIF20B | | | | | |
| 10 | 91589249 | 91600618 | LINC00865 | | | | | |
| 10 | 91675245 | 91717130 | LINC01375 | | | | | |
| 10 | 92162277 | 92300562 | LOC101926942 | | | | | |
| 10 | 92500575 | 92617671 | HTR7 | | | HTR7 | HTR7 | |
| 10 | 92631708 | 92668312 | RPP30 | | | | | |
| 10 | 92671856 | 92681032 | ANKRD1 | | | | | |
| 10 | 92707056 | 92751889 | XLOC_008559 | | | | | |
| 10 | 92792922 | 92801012 | LOC105378430 | | | | | |
| 10 | 92805564 | 92821916 | LINC00502 | | | | | |
| 10 | 92911760 | 92912837 | NUDT9P1 | | | | | |
| 10 | 92922768 | 93044088 | PCGF5 | | | | | |
| 10 | 93066718 | 93274585 | HECTD2 | | | | | |
| 10 | 93388196 | 93392858 | PPP1R3C | | | | | |
| 10 | 93542595 | 93625232 | TNKS2 | | | TNKS2 | | |
| 10 | 93666344 | 93669258 | FGFBP3 | | | | | |
| 10 | 93683735 | 93790080 | BTAF1 | | | | | |
| 10 | 93808396 | 94050875 | CPEB3 | | | | | |
| 10 | 94050919 | 94113721 | MARCH5 | | | | | |
| 10 | 94178417 | 94179363 | MARK2P9 | | | | | |
| 10 | 94211440 | 94333852 | IDE | | | IDE | | |
| 10 | 94352824 | 94415152 | KIF11 | | | KIF11 | | |
| 10 | 94449680 | 94455408 | HHEX | | | | | |
| 10 | 94586587 | 94819251 | EXOC6 | | | | | |
| 10 | 94821020 | 94828454 | CYP26C1 | | | | | |
| 10 | 94833231 | 94837641 | CYP26A1 | | | | | |
| 10 | 95066185 | 95242074 | MYOF | | | | | |
| 10 | 95256368 | 95288849 | CEP55 | | | | | |
| 10 | 95326421 | 95349829 | FFAR4 | | | | | |
| 10 | 95351592 | 95360993 | RBP4 | | | | | |
| 10 | 95372344 | 95425429 | PDE6C | | | | | |
| 10 | 95427639 | 95462329 | FRA10AC1 | | FRA10AC1 | | | |
| 10 | 95517565 | 95557931 | LGI1 | | LGI1 | | | |
| 10 | 95653729 | 95662491 | SLC35G1 | | | | | |
| 10 | 95717896 | 95721672 | PIPSL | | | | | |
| 10 | 95753745 | 96046827 | PLCE1 | | | PLCE1 | | |
| 10 | 96092982 | 96122733 | NOC3L | | | | | |
| 10 | 96162185 | 96296089 | TBC1D12 | | | | | |
| 10 | 96305523 | 96361856 | HELLS | | | | | |
| 10 | 96443250 | 96495947 | CYP2C18 | | | | | |
| 10 | 96522437 | 96612962 | CYP2C19 | | | | | |
| 10 | 96698414 | 96749148 | CYP2C9 | | | | | |
| 10 | 96796528 | 96829254 | CYP2C8 | | CYP2C8 | | | |
| 10 | 96953956 | 96988686 | ACSM6 | | | | | |
| 10 | 96997324 | 97050905 | PDLIM1 | | | | | |

| | | | | | | | | |
|---|---|---|---|---|---|---|---|---|
| 10 | 97071529 | 97321177 | SORBS1 | | | SORBS1 | | |
| 10 | 97365685 | 97416567 | ALDH18A1 | | | ALDH18A1 | | |
| 10 | 97423152 | 97453900 | TCTN3 | | | | | |
| 10 | 97471535 | 97637023 | ENTPD1 | | | | | |
| 10 | 97667721 | 97698415 | C10orf131 | | | | | |
| 10 | 97759847 | 97792441 | CC2D2B | | | | | |
| 10 | 97780956 | 97849992 | ENTPD1 | | | | | |
| 10 | 97803158 | 97820625 | CCNJ | | | | | |
| 10 | 97824071 | 97824156 | MIR3157 | | | | | |
| 10 | 97889471 | 97923517 | ZNF518A | | | | | |
| 10 | 97951454 | 98031333 | BLNK | | | BLNK | | |
| 10 | 98064084 | 98098321 | DNTT | | | | | |
| 10 | 98102974 | 98119122 | OPALIN | | | OPALIN | | |
| 10 | 98124362 | 98273683 | TLL2 | | | | | |
| 10 | 98277866 | 98346809 | TM9SF3 | | | | | |
| 10 | 98353068 | 98480279 | PIK3AP1 | | | | | |
| 10 | 98588425 | 98588521 | MIR607 | | | | | |
| 10 | 98592016 | 98724198 | LCOR | | | | | |
| 10 | 98741040 | 98745585 | C10orf12 | | | | | |
| 10 | 98757794 | 98863504 | SLIT1 | | | SLIT1 | | |
| 10 | 98912798 | 99052430 | ARHGAP19-SLIT1 | | | | | |
| 10 | 98981929 | 99052430 | ARHGAP19 | | | | | |
| 10 | 99079021 | 99081672 | FRAT1 | | | | | |
| 10 | 99092253 | 99094458 | FRAT2 | | | | | |
| 10 | 99116457 | 99161127 | RRP12 | | | | | |
| 10 | 99185881 | 99193201 | PGAM1 | | | | | |
| 10 | 99195665 | 99205774 | EXOSC1 | | | | | |
| 10 | 99205887 | 99217127 | ZDHHC16 | | | | | |
| 10 | 99218080 | 99258551 | MMS19 | | | | | |
| 10 | 99258638 | 99330967 | UBTD1 | | | | | |
| 10 | 99332197 | 99343641 | ANKRD2 | | | | | |
| 10 | 99344101 | 99372555 | HOGA1 | | HOGA1 | | | |
| 10 | 99349449 | 99350691 | C10orf62 | | | | | |
| 10 | 99354974 | 99354995 | MIR5692C2 | | | | | |
| 10 | 99374309 | 99393913 | MORN4 | | | | | |
| 10 | 99400442 | 99436189 | PI4K2A | | | | | |
| 10 | 99437180 | 99447015 | AVPI1 | | | | | |
| 10 | 99473464 | 99477909 | MARVELD1 | | | | | |
| 10 | 99496877 | 99520664 | ZFYVE27 | | | | | |
| 10 | 99526507 | 99531756 | SFRP5 | | | | | |
| 10 | 99588234 | 99609555 | LINC00866 | | | | | |
| 10 | 99609994 | 99631335 | GOLGA7B | | | | | |
| 10 | 99624756 | 99790585 | CRTAC1 | | | | | |
| 10 | 99894380 | 100004654 | R3HCC1L | | | | | |
| 10 | 100007442 | 100028007 | LOXL4 | | | | | |
| 10 | 100143321 | 100174978 | PYROXD2 | | | | | |
| 10 | 100154974 | 100155064 | MIR1287 | | | | | |
| 10 | 100175954 | 100206720 | HPS1 | | | HPS1 | | |
| 10 | 100191048 | 100191117 | MIR4685 | | | | | |
| 10 | 100206077 | 100213562 | LOC101927278 | | | | | |
| 10 | 100216833 | 100995632 | HPSE2 | | HPSE2 | | | |
| 10 | 100684255 | 100684325 | MIR6507 | | | | | |
| 10 | 101088855 | 101154087 | CNNM1 | | | | | |
| 10 | 101156626 | 101190530 | GOT1 | | GOT1 | GOT1 | | |
| 10 | 101286106 | 101290934 | LINC01475 | | | | | |
| 10 | 101292689 | 101296280 | NKX2-3 | | | | | |
| 10 | 101370274 | 101380221 | SLC25A28 | | | | | |
| 10 | 101419262 | 101471002 | ENTPD7 | | | | | |
| 10 | 101468504 | 101492423 | COX15 | | | | | |
| 10 | 101491957 | 101515894 | CUTC | | | | | |
| 10 | 101542354 | 101611949 | ABCC2 | | | ABCC2 | | |
| 10 | 101635327 | 101718755 | DNMBP | | | DNMBP | | |
| 10 | 101802064 | 101841642 | CPN1 | | | | | |
| 10 | 101909846 | 101945814 | ERLIN1 | | | | | |
| 10 | 101948123 | 101989344 | CHUK | | | | | |
| 10 | 101992052 | 102027438 | CWF19L1 | | | | | |

| | | | | | | | | |
|---|---|---|---|---|---|---|---|---|
| 10 | 101996912 | 101997059 | SNORA12 | | | | | |
| 10 | 102033034 | 102046469 | BLOC1S2 | | | | | |
| 10 | 102047902 | 102090243 | PKD2L1 | | | | | |
| 10 | 102106771 | 102124588 | SCD | | | SCD | | |
| 10 | 102133332 | 102148111 | OLMALINC | | | | | |
| 10 | 102222811 | 102243399 | WNT8B | | | | | |
| 10 | 102246402 | 102279595 | SEC31B | | | | | |
| 10 | 102283485 | 102289680 | NDUFB8 | | | NDUFB8 | | |
| 10 | 102295640 | 102313681 | HIF1AN | | | | | |
| 10 | 102495465 | 102589698 | PAX2 | | | | | |
| 10 | 102672325 | 102724891 | SLF2 | | | | | |
| 10 | 102732285 | 102745373 | SEMA4G | | | | | |
| 10 | 102734741 | 102734841 | MIR608 | | | | | |
| 10 | 102737578 | 102747272 | MRPL43 | | | MRPL43 | | |
| 10 | 102747292 | 102754158 | C10orf2 | | | | | |
| 10 | 102756822 | 102767593 | LZTS2 | | | | | |
| 10 | 102767439 | 102790914 | PDZD7 | | PDZD7 | | | |
| 10 | 102790995 | 102800998 | SFXN3 | | | | | |
| 10 | 102820998 | 102827898 | KAZALD1 | | | | | |
| 10 | 102849077 | 102901023 | TLX1NB | | | | | |
| 10 | 102891060 | 102897546 | TLX1 | | | | | |
| 10 | 102936078 | 102953904 | LINC01514 | | | | | |
| 10 | 102986732 | 102998616 | LBX1 | | | | | |
| 10 | 103012577 | 103023307 | LOC101927419 | | | | | |
| 10 | 103113789 | 103317078 | BTRC | | BTRC | | | |
| 10 | 103338638 | 103348027 | POLL | | | POLL | | |
| 10 | 103348088 | 103369410 | DPCD | | | | | |
| 10 | 103361173 | 103361254 | MIR3158 | | | | | |
| 10 | 103370420 | 103454743 | FBXW4 | | | FBXW4 | | |
| 10 | 103529886 | 103540126 | FGF8 | | | | | |
| 10 | 103541081 | 103543170 | NPM3 | | | | | |
| 10 | 103544199 | 103578222 | MGEA5 | | | | | |
| 10 | 103578824 | 103603677 | KCNIP2 | | KCNIP2 | | | |
| 10 | 103605355 | 103815932 | C10orf76 | | | | | |
| 10 | 103825123 | 103827795 | HPS6 | | | | | |
| 10 | 103867324 | 103880210 | LDB1 | | | | | |
| 10 | 103892750 | 103910090 | PPRC1 | | | | | |
| 10 | 103911932 | 103923628 | NOLC1 | | | | | |
| 10 | 103986034 | 103989344 | ELOVL3 | | | | | |
| 10 | 103989945 | 104001231 | PITX3 | | | | | |
| 10 | 104005254 | 104142656 | GBF1 | | | | | |
| 10 | 104153866 | 104162286 | NFKB2 | | | | | |
| 10 | 104162373 | 104179691 | PSD | | | | | |
| 10 | 104179570 | 104182893 | FBXL15 | | | | | |
| 10 | 104183001 | 104192423 | CUEDC2 | | | | | |
| 10 | 104196268 | 104196341 | MIR146B | | | | | |
| 10 | 104209573 | 104216050 | RPARP | | | | | |
| 10 | 104209593 | 104211300 | C10orf95 | | | | | |
| 10 | 104221169 | 104236802 | MFSD13A | | | | | |
| 10 | 104238985 | 104262512 | ACTR1A | | ACTR1A | | | |
| 10 | 104263718 | 104393214 | SUFU | | | | | |
| 10 | 104404251 | 104418076 | TRIM8 | | | | | |
| 10 | 104433483 | 104474190 | ARL3 | | | | | |
| 10 | 104474297 | 104498946 | SFXN2 | | | SFXN2 | | |
| 10 | 104503726 | 104576021 | WBP1L | | | | | |
| 10 | 104590287 | 104597290 | CYP17A1 | | | | | |
| 10 | 104613966 | 104624718 | BORCS7 | | | | | |
| 10 | 104613966 | 104661655 | BORCS7-ASMT | | | | | |
| 10 | 104629209 | 104661655 | AS3MT | | | | | |
| 10 | 104678074 | 104838344 | CNNM2 | | CNNM2 | | | |
| 10 | 104847773 | 104953063 | NT5C2 | | | | | |
| 10 | 105005643 | 105007773 | RPEL1 | | | | | |
| 10 | 105036919 | 105050108 | INA | | INA | INA | | |
| 10 | 105062552 | 105110891 | PCGF6 | | | | | |
| 10 | 105127709 | 105148822 | TAF5 | | | | | |
| 10 | 105148808 | 105156270 | USMG5 | | | | | |

| | | | | | | | | |
|---|---|---|---|---|---|---|---|---|
| 10 | 105154009 | 105154158 | MIR1307 | | | | | |
| 10 | 105156411 | 105206019 | PDCD11 | | | | | |
| 10 | 105206542 | 105212162 | CALHM2 | | | | | |
| 10 | 105213143 | 105218648 | CALHM1 | | | | | |
| 10 | 105232560 | 105238997 | CALHM3 | | | | | |
| 10 | 105239359 | 105277199 | NEURL1 | | | | | |
| 10 | 105353783 | 105515167 | SH3PXD2A | | | | | |
| 10 | 105637317 | 105678045 | OBFC1 | | | | | |
| 10 | 105726942 | 105788991 | SLK | | | | | |
| 10 | 105791045 | 105845638 | COL17A1 | | | | | |
| 10 | 105807846 | 105807944 | MIR936 | | | | | |
| 10 | 105881815 | 105886143 | SFR1 | | | | | |
| 10 | 105889645 | 105992120 | CFAP43 | | | | | |
| 10 | 105978546 | 105978641 | MIR609 | | | | | |
| 10 | 106013951 | 106027222 | GSTO1 | | | | | |
| 10 | 106028093 | 106028163 | MIR4482 | | | | | |
| 10 | 106028630 | 106059176 | GSTO2 | | | | | |
| 10 | 106069453 | 106098251 | ITPRIP | | | | | |
| 10 | 106083121 | 106086503 | LOC101927472 | | | | | |
| 10 | 106111058 | 106214848 | CFAP58 | | | | | |
| 10 | 106234697 | 106240033 | LOC101927523 | | | | | |
| 10 | 106400858 | 106425974 | SORCS3 | | | | | |
| 10 | 107433367 | 107580091 | LOC101927549 | | | | | |
| 10 | 107899922 | 107948480 | LOC105378470 | | | | | |
| 10 | 108333420 | 108924466 | SORCS1 | | SORCS1 | | SORCS1 | |
| 10 | 109631334 | 109829051 | LINC01435 | | | | | |
| 10 | 111624523 | 111683311 | XPNPEP1 | | | | | |
| 10 | 111705316 | 111895323 | ADD3 | | | | | |
| 10 | 111967362 | 112047123 | MXI1 | | MXI1 | | | |
| 10 | 112052797 | 112064707 | SMNDC1 | | | | | |
| 10 | 112257624 | 112271302 | DUSP5 | | | | | |
| 10 | 112327448 | 112364392 | SMC3 | | | SMC3 | | |
| 10 | 112404154 | 112599229 | RBM20 | | | | | |
| 10 | 112628647 | 112659764 | PDCD4 | | | | | |
| 10 | 112657847 | 112657913 | MIR4680 | | | | | |
| 10 | 112658487 | 112679124 | BBIP1 | | | | | |
| 10 | 112679300 | 112773425 | SHOC2 | | | | | |
| 10 | 112696360 | 112697013 | RPL13AP6 | | | | | |
| 10 | 112723882 | 112773425 | SHOC2 | | | | | |
| 10 | 112836789 | 112840662 | ADRA2A | | | | | |
| 10 | 113909621 | 113943537 | GPAM | | | GPAM | | |
| 10 | 114043413 | 114064792 | TECTB | | | | | |
| 10 | 114059369 | 114059446 | MIR6715B | | | | | |
| 10 | 114059369 | 114059448 | MIR6715A | | | | | |
| 10 | 114067935 | 114116353 | GUCY2GP | | | | | |
| 10 | 114133915 | 114188138 | ACSL5 | | | ACSL5 | | |
| 10 | 114190047 | 114206717 | ZDHHC6 | | | | | |
| 10 | 114206745 | 114578503 | VTI1A | | | | | |
| 10 | 114393928 | 114394013 | MIR4295 | | | | | |
| 10 | 114583254 | 114587491 | LOC103344931 | | | | | |
| 10 | 114710008 | 114927436 | TCF7L2 | | | TCF7L2 | | |
| 10 | 114805113 | 114805335 | SNORA87 | | | | | |
| 10 | 115310589 | 115349360 | HABP2 | | | | | |
| 10 | 115348582 | 115423829 | NRAP | | | | | |
| 10 | 115438920 | 115490668 | CASP7 | | | | | |
| 10 | 115511212 | 115537489 | PLEKHS1 | | | | | |
| 10 | 115537751 | 115537813 | MIR4483 | | | | | |
| 10 | 115594482 | 115614163 | DCLRE1A | | | | | |
| 10 | 115614390 | 115672265 | NHLRC2 | | | | | |
| 10 | 115803805 | 115806667 | ADRB1 | | ADRB1 | | ADRB1 | |
| 10 | 115881973 | 115934364 | CCDC186 | | | | | |
| 10 | 115933863 | 115933938 | MIR2110 | | | | | |
| 10 | 115939028 | 115992063 | TDRD1 | | | | | |
| 10 | 115999012 | 116054259 | VWA2 | | | | | |
| 10 | 116054582 | 116164537 | AFAP1L2 | | | | | |
| 10 | 116190868 | 116444414 | ABLIM1 | | | | | |

| | | | | | | | | |
|---|---|---|---|---|---|---|---|---|
| 10 | 116524546 | 116539662 | LOC101927692 | | | | | |
| 10 | 116581502 | 116659586 | FAM160B1 | | | | | |
| 10 | 116697951 | 116737439 | TRUB1 | | | | | |
| 10 | 116754415 | 116756352 | LOC102724589 | | | | | |
| 10 | 116853123 | 117708509 | ATRNL1 | | | | | |
| 10 | 117816441 | 118033157 | GFRA1 | | GFRA1 | | | |
| 10 | 118083939 | 118139541 | CCDC172 | | | | | |
| 10 | 118187423 | 118237468 | PNLIPRP3 | | | | | |
| 10 | 118305427 | 118327367 | PNLIP | | | | | |
| 10 | 118350470 | 118368687 | PNLIPRP1 | | | | | |
| 10 | 118380464 | 118404654 | PNLIPRP2 | | | | | |
| 10 | 118423206 | 118429481 | C10orf82 | | | | | |
| 10 | 118430702 | 118502085 | HSPA12A | | | | | |
| 10 | 118609022 | 118642112 | ENO4 | | | | | |
| 10 | 118642887 | 118886097 | SHTN1 | | | | | |
| 10 | 118888031 | 118897812 | VAX1 | | | | | |
| 10 | 118913031 | 118928566 | MIR3663HG | | | | | |
| 10 | 118927188 | 118927285 | MIR3663 | | | | | |
| 10 | 118956999 | 118969810 | KCNK18 | | | | | |
| 10 | 119000583 | 119038941 | SLC18A2 | | | SLC18A2 | | |
| 10 | 119039999 | 119134978 | PDZD8 | | | | | |
| 10 | 119243803 | 119304579 | EMX2OS | | | | | |
| 10 | 119301955 | 119309057 | EMX2 | | | EMX2 | | |
| 10 | 119764426 | 119806114 | RAB11FIP2 | | | RAB11FIP2 | | |
| 10 | 119806331 | 119969665 | CASC2 | | | | | |
| 10 | 120068571 | 120101839 | FAM204A | | | | | |
| 10 | 120116619 | 120118715 | LINC00867 | | | | | |
| 10 | 120352915 | 120355160 | PRLHR | | | | | |
| 10 | 120440493 | 120514758 | CACUL1 | | | | | |
| 10 | 120789227 | 120793244 | NANOS1 | | | | | |
| 10 | 120794540 | 120840334 | EIF3A | | | | | |
| 10 | 120819524 | 120819650 | SNORA19 | | | | | |
| 10 | 120863576 | 120897496 | FAM45A | | | | | |
| 10 | 120863628 | 120897376 | FAM45BP | | | | | |
| 10 | 120900424 | 120925204 | SFXN4 | | | | | |
| 10 | 120927210 | 120938377 | PRDX3 | | | | | |
| 10 | 120967196 | 121215131 | GRK5 | | | GRK5 | | |
| 10 | 121137483 | 121137555 | MIR4681 | | | | | |
| 10 | 121259338 | 121302222 | RGS10 | | | | | |
| 10 | 121332977 | 121356541 | TIAL1 | | | | | |
| 10 | 121410881 | 121437329 | BAG3 | | | | | |
| 10 | 121485558 | 121588662 | INPP5F | | INPP5F | | | |
| 10 | 121588915 | 121633140 | MCMBP | | | | | |
| 10 | 121652084 | 121704170 | SEC23IP | | | | | |
| 10 | 121718024 | 121718104 | MIR4682 | | | | | |
| 10 | 122216465 | 122349376 | PLPP4 | | | | | |
| 10 | 122344590 | 122806858 | MIR5694 | | | | | |
| 10 | 122357460 | 122359635 | LINC01561 | | | | | |
| 10 | 122521323 | 122669038 | WDR11 | | | | | |
| 10 | 123237843 | 123357972 | FGFR2 | | FGFR2 | FGFR2 | | |
| 10 | 123499935 | 123711480 | ATE1 | | | | | |
| 10 | 123716602 | 123734743 | NSMCE4A | | | | | |
| 10 | 123748688 | 124014060 | TACC2 | | | TACC2 | | |
| 10 | 124030810 | 124097695 | BTBD16 | | | | | |
| 10 | 124134093 | 124191871 | PLEKHA1 | | | | | |
| 10 | 124176480 | 124176583 | MIR3941 | | | | | |
| 10 | 124214178 | 124216868 | ARMS2 | | | | | |
| 10 | 124221040 | 124274424 | HTRA1 | | HTRA1 | | | |
| 10 | 124320180 | 124403252 | DMBT1 | | | | | |
| 10 | 124457224 | 124459338 | C10orf120 | | | | | |
| 10 | 124516209 | 124557161 | DMBT1P1 | | | | | |
| 10 | 124591670 | 124605691 | CUZD1 | | | | | |
| 10 | 124591670 | 124639157 | FAM24B-CUZD1 | | | | | |
| 10 | 124608609 | 124639157 | FAM24B | | | | | |
| 10 | 124639148 | 124658230 | LOC399815 | | | | | |
| 10 | 124670216 | 124672627 | FAM24A | | | | | |

| | | | | | | | | |
|---|---|---|---|---|---|---|---|---|
| 10 | 124690418 | 124713919 | C10orf88 | | | | | |
| 10 | 124739555 | 124749907 | PSTK | | | | | |
| 10 | 124750321 | 124768366 | IKZF5 | | | | | |
| 10 | 124768428 | 124817806 | ACADSB | | | ACADSB | | |
| 10 | 124895566 | 124897247 | HMX3 | | | | | |
| 10 | 124907637 | 124910188 | HMX2 | | | | | |
| 10 | 124913759 | 124924886 | BUB3 | | | | | |
| 10 | 125425870 | 125456913 | GPR26 | | | | | |
| 10 | 125505151 | 125651500 | CPXM2 | | | | | |
| 10 | 125767181 | 125853123 | CHST15 | | | | | |
| 10 | 126085871 | 126107545 | OAT | | | | | |
| 10 | 126135997 | 126138550 | NKX1-2 | | | | | |
| 10 | 126150340 | 126302710 | LHPP | | | | | |
| 10 | 126307862 | 126403329 | FAM53B | | | | | |
| 10 | 126446399 | 126480510 | METTL10 | | | | | |
| 10 | 126490353 | 126525239 | FAM175B | | | | | |
| 10 | 126630691 | 126676005 | ZRANB1 | | | ZRANB1 | | |
| 10 | 126676417 | 126849624 | CTBP2 | | | | | |
| 10 | 126721351 | 126721439 | MIR4296 | | | | | |
| 10 | 127262939 | 127371713 | TEX36 | | | | | |
| 10 | 127371811 | 127398246 | LOC283038 | | | | | |
| 10 | 127393858 | 127408062 | FLJ37035 | | | | | |
| 10 | 127408083 | 127440629 | EDRF1 | | | | | |
| 10 | 127455026 | 127464390 | MMP21 | | | | | |
| 10 | 127477146 | 127511837 | UROS | | UROS | | | |
| 10 | 127508308 | 127508391 | MIR4484 | | | | | |
| 10 | 127512103 | 127542264 | BCCIP | | | | | |
| 10 | 127524908 | 127569884 | DHX32 | | | | | |
| 10 | 127585107 | 127661842 | FANK1 | | | | | |
| 10 | 127700953 | 128077127 | ADAM12 | | | | | |
| 10 | 128102437 | 128110448 | LINC00601 | | | | | |
| 10 | 128113573 | 128210010 | C10orf90 | | | | | |
| 10 | 128593977 | 129250781 | DOCK1 | | | | | |
| 10 | 128933689 | 128994422 | FAM196A | | | | | |
| 10 | 129347612 | 129350935 | NPS | | | | | |
| 10 | 129535537 | 129539450 | FOXI2 | | | | | |
| 10 | 129676113 | 129691211 | CLRN3 | | | | | |
| 10 | 129705324 | 129884119 | PTPRE | | | | | |
| 10 | 129894924 | 129924468 | MKI67 | | | | | |
| 10 | 130084213 | 130115990 | LINC01163 | | | | | |
| 10 | 131265447 | 131565884 | MGMT | | | | | |
| 10 | 131633495 | 131762091 | EBF3 | | | | | |
| 10 | 131641562 | 131641638 | MIR4297 | | | | | |
| 10 | 131862161 | 131909081 | LINC00959 | | | | | |
| 10 | 131904272 | 131907101 | CTAGE7P | | | | | |
| 10 | 131934638 | 131978646 | GLRX3 | | | | | |
| 10 | 132760850 | 132760931 | MIR378C | | | | | |
| 10 | 132890654 | 132894040 | TCERG1L | | | | | |
| 10 | 133604733 | 133622535 | LINC01164 | | | | | |
| 10 | 133747954 | 133773338 | PPP2R2D | | | | | |
| 10 | 133781186 | 133795517 | BNIP3 | | | BNIP3 | | |
| 10 | 133918312 | 133998313 | JAKMIP3 | | | | | |
| 10 | 134000413 | 134019280 | DPYSL4 | | | | | |
| 10 | 134020985 | 134145379 | STK32C | | | | | |
| 10 | 134145613 | 134180509 | LRRC27 | | | | | |
| 10 | 134210701 | 134231358 | PWWP2B | | | | | |
| 10 | 134258713 | 134261825 | C10orf91 | | | | | |
| 10 | 134351352 | 134596984 | INPP5A | | | | | |
| 10 | 134598319 | 134599537 | NKX6-2 | | | | | |
| 10 | 134621895 | 134756089 | CFAP46 | | | | | |
| 10 | 134757470 | 134778793 | LINC01166 | | | | | |
| 10 | 134774843 | 134775741 | LINC01167 | | | | | |
| 10 | 134779037 | 134790001 | LINC01168 | | | | | |
| 10 | 134898752 | 134945179 | ADGRA1 | | ADGRA1 | | | |
| 10 | 134973970 | 135039916 | KNDC1 | | | | | |
| 10 | 135043777 | 135045062 | UTF1 | | | | | |

| | | | | | | | | |
|---|---|---|---|---|---|---|---|---|
| 10 | 135051407 | 135055434 | VENTX | | | | | |
| 10 | 135059982 | 135061389 | MIR202HG | | | | | |
| 10 | 135061014 | 135061124 | MIR202 | | | | | |
| 10 | 135075919 | 135090407 | ADAM8 | | | | | |
| 10 | 135092133 | 135125841 | TUBGCP2 | | | | | |
| 10 | 135122392 | 135126666 | ZNF511 | | | | | |
| 10 | 135138927 | 135150475 | CALY | | | CALY | | |
| 10 | 135160843 | 135166187 | PRAP1 | | | | | |
| 10 | 135168657 | 135171529 | FUOM | | | | | |
| 10 | 135175986 | 135186908 | ECHS1 | | | | | |
| 10 | 135185059 | 135185167 | MIR3944 | | | | | |
| 10 | 135192740 | 135205200 | PAOX | | | | | |
| 10 | 135207620 | 135234174 | MTG1 | | | | | |
| 10 | 135234169 | 135238121 | SPRN | | | | | |
| 10 | 135267431 | 135281949 | SCART1 | | | | | |
| 10 | 135340866 | 135352620 | CYP2E1 | | | | | |
| 10 | 135367403 | 135379138 | SYCE1 | | | | | |
| 10 | 135380437 | 135383462 | SPRNP1 | | | | | |
| 10 | 135438602 | 135440299 | FRG2B | | | | | |
| 11 | 126986 | 131920 | LINC01001 | | | | | |
| 11 | 193079 | 194500 | SCGB1C2 | | | | | |
| 11 | 193079 | 194573 | SCGB1C1 | | | | | |
| 11 | 196760 | 200258 | ODF3 | | | | | |
| 11 | 202923 | 207422 | BET1L | | | | | |
| 11 | 207510 | 215175 | RIC8A | | | | | |
| 11 | 209335 | 209406 | MIR6743 | | | | | |
| 11 | 215029 | 236362 | SIRT3 | | | | | |
| 11 | 236807 | 252984 | PSMD13 | | | | | |
| 11 | 278569 | 285388 | NLRP6 | | | | | |
| 11 | 289137 | 295688 | ATHL1 | | | | | |
| 11 | 298200 | 299526 | IFITM5 | | | | | |
| 11 | 308106 | 309410 | IFITM2 | | | | | |
| 11 | 313990 | 315272 | IFITM1 | | | | | |
| 11 | 319672 | 320914 | IFITM3 | | | | | |
| 11 | 369794 | 382117 | B4GALNT4 | | | | | |
| 11 | 392598 | 404908 | PKP3 | | | | | |
| 11 | 405715 | 417397 | SIGIRR | | | | | |
| 11 | 417929 | 442011 | ANO9 | | | | | |
| 11 | 450279 | 491387 | PTDSS2 | | | | | |
| 11 | 494511 | 507283 | RNH1 | | | | | |
| 11 | 532241 | 535567 | HRAS | | HRAS | | | |
| 11 | 537521 | 554916 | LRRC56 | | | | | |
| 11 | 554849 | 560779 | LMNTD2 | | | | | |
| 11 | 560970 | 564025 | RASSF7 | | | | | |
| 11 | 565656 | 568457 | MIR210HG | | | | | |
| 11 | 568088 | 568198 | MIR210 | | | | | |
| 11 | 573807 | 575885 | LOC143666 | | | | | |
| 11 | 576445 | 612222 | PHRF1 | | | | | |
| 11 | 612554 | 615999 | IRF7 | | | | | |
| 11 | 616564 | 625067 | CDHR5 | | | | | |
| 11 | 626312 | 627173 | SCT | | | | | |
| 11 | 637304 | 640705 | DRD4 | DRD4 | | DRD4 | DRD4 | |
| 11 | 644219 | 695754 | DEAF1 | | DEAF1 | | | |
| 11 | 695590 | 705028 | TMEM80 | | | | | |
| 11 | 706119 | 727727 | EPS8L2 | | | | | |
| 11 | 747431 | 765024 | TALDO1 | | | | | |
| 11 | 767222 | 777502 | PDDC1 | | | | | |
| 11 | 777577 | 784284 | LOC171391 | | | | | |
| 11 | 787109 | 790126 | CEND1 | | | | | |
| 11 | 790474 | 798269 | SLC25A22 | | SLC25A22 | SLC25A22 | | |
| 11 | 797510 | 799185 | PANO1 | | | | | |
| 11 | 799178 | 805250 | PIDD1 | | | | | |
| 11 | 809935 | 812876 | RPLP2 | | | | | |
| 11 | 811680 | 811814 | SNORA52 | | | | | |
| 11 | 818900 | 825571 | PNPLA2 | | | | | |
| 11 | 828259 | 831991 | CRACR2B | | | | | |

| | | | | | | | | |
|---|---|---|---|---|---|---|---|---|
| 11 | 832951 | 838835 | CD151 | | | | | |
| 11 | 839720 | 842529 | POLR2L | | | | | |
| 11 | 842823 | 867116 | TSPAN4 | | | | | |
| 11 | 867858 | 915058 | CHID1 | | | | | |
| 11 | 925808 | 1012245 | AP2A2 | | | | | |
| 11 | 1012823 | 1036706 | MUC6 | | | | | |
| 11 | 1074874 | 1104416 | MUC2 | | | | | |
| 11 | 1151579 | 1222366 | MUC5AC | | | | | |
| 11 | 1244294 | 1283406 | MUC5B | | | | | |
| 11 | 1277834 | 1277900 | MIR6744 | | | | | |
| 11 | 1295597 | 1331937 | TOLLIP | | | | | |
| 11 | 1411128 | 1483919 | BRSK2 | | | | | |
| 11 | 1490677 | 1508009 | MOB2 | | | | | |
| 11 | 1575280 | 1593150 | DUSP8 | | | | | |
| 11 | 1593970 | 1620414 | KRTAP5 | | | | | |
| 11 | 1605571 | 1606513 | KRTAP5-1 | | | | | |
| 11 | 1618406 | 1619524 | KRTAP5-2 | | | | | |
| 11 | 1628794 | 1629693 | KRTAP5-3 | | | | | |
| 11 | 1642187 | 1643368 | KRTAP5-4 | | | | | |
| 11 | 1651032 | 1652159 | KRTAP5-5 | | | | | |
| 11 | 1686828 | 1689086 | FAM99A | | | | | |
| 11 | 1704499 | 1706859 | FAM99B | | | | | |
| 11 | 1718424 | 1718985 | KRTAP5-6 | | | | | |
| 11 | 1753639 | 1771824 | IFITM10 | | | | | |
| 11 | 1773981 | 1785222 | CTSD | | | | | |
| 11 | 1855539 | 1858751 | SYT8 | | | SYT8 | | |
| 11 | 1860232 | 1862910 | TNNI2 | | | | | |
| 11 | 1874199 | 1913493 | LSP1 | | | | | |
| 11 | 1880693 | 1880766 | MIR4298 | | | | | |
| 11 | 1886394 | 1913493 | LSP1 | | | | | |
| 11 | 1901274 | 1901377 | MIR7847 | | | | | |
| 11 | 1917988 | 1929589 | LINC01150 | | | | | |
| 11 | 1940798 | 1959936 | TNNT3 | | | | | |
| 11 | 1968501 | 1977839 | MRPL23 | | | | | |
| 11 | 1970560 | 1970706 | SNORD131 | | | | | |
| 11 | 2004438 | 2011150 | MRPL23 | | | | | |
| 11 | 2012325 | 2014699 | LINC01219 | | | | | |
| 11 | 2013637 | 2017524 | HOTS | | | | | |
| 11 | 2016405 | 2022696 | H19 | | | | | |
| 11 | 2017988 | 2018061 | MIR675 | | | | | |
| 11 | 2150341 | 2170833 | IGF2 | | | IGF2 | | |
| 11 | 2150341 | 2182439 | INS-IGF2 | | | | | |
| 11 | 2155363 | 2155439 | MIR483 | | | | | |
| 11 | 2161741 | 2169896 | IGF2 | | | IGF2 | | |
| 11 | 2167852 | 2182439 | INS-IGF2 | | | | | |
| 11 | 2181008 | 2182439 | INS | | | | | |
| 11 | 2185158 | 2193035 | TH | TH | TH | TH | | |
| 11 | 2194292 | 2194368 | MIR4686 | | | | | |
| 11 | 2289727 | 2292182 | ASCL2 | | | | | |
| 11 | 2317506 | 2323143 | C11orf21 | | | | | |
| 11 | 2323242 | 2339430 | TSPAN32 | | | | | |
| 11 | 2349978 | 2418649 | CD81 | | | CD81 | | |
| 11 | 2421717 | 2425108 | TSSC4 | | | | | |
| 11 | 2425745 | 2444275 | TRPM5 | | | | | |
| 11 | 2466220 | 2870340 | KCNQ1 | | | KCNQ1 | | |
| 11 | 2629557 | 2721228 | KCNQ1OT1 | | | | | |
| 11 | 2861364 | 2882799 | KCNQ1 | | | KCNQ1 | | |
| 11 | 2891262 | 2893336 | KCNQ1DN | | | | | |
| 11 | 2904447 | 2906995 | CDKN1C | | CDKN1C | | | |
| 11 | 2908574 | 2925175 | SLC22A18AS | | | | | |
| 11 | 2920950 | 2946476 | SLC22A18 | | | | | |
| 11 | 2949502 | 2950650 | PHLDA2 | | | | | |
| 11 | 2965659 | 3013607 | NAP1L4 | | | | | |
| 11 | 2985000 | 2985123 | SNORA54 | | | | | |
| 11 | 3022151 | 3078681 | CARS | | | | | |
| 11 | 3108345 | 3186582 | OSBPL5 | | | | | |

| | | | | | | | | |
|---|---|---|---|---|---|---|---|---|
| 11 | 3239173 | 3244361 | MRGPRG | | | | | |
| 11 | 3249040 | 3253616 | MRGPRE | | | | | |
| 11 | 3379156 | 3400452 | ZNF195 | | | | | |
| 11 | 3402190 | 3430378 | TSSC2 | | | | | |
| 11 | 3411936 | 3413145 | OR7E12P | | | | | |
| 11 | 3529209 | 3593217 | LOC101927708 | | | | | |
| 11 | 3647689 | 3658789 | TRPC2 | | | | | |
| 11 | 3659735 | 3663546 | ART5 | | | | | |
| 11 | 3666360 | 3685646 | ART1 | | | | | |
| 11 | 3686816 | 3692614 | CHRNA10 | | | CHRNA10 | | |
| 11 | 3696239 | 3819022 | NUP98 | | | | | |
| 11 | 3818953 | 3847601 | PGAP2 | | | | | |
| 11 | 3848207 | 3862213 | RHOG | | | | | |
| 11 | 3876932 | 4114440 | STIM1 | | | | | |
| 11 | 3877291 | 3877371 | MIR4687 | | | | | |
| 11 | 4115914 | 4160155 | RRM1 | | | | | |
| 11 | 4208381 | 4223883 | LOC100506082 | | | | | |
| 11 | 4388492 | 4389616 | OR52B4 | | | | | |
| 11 | 4406126 | 4414926 | TRIM21 | | | | | |
| 11 | 4470524 | 4471591 | OR52K2 | | | | | |
| 11 | 4510108 | 4511138 | OR52K1 | | | | | |
| 11 | 4566420 | 4567374 | OR52M1 | | | | | |
| 11 | 4592652 | 4599050 | C11orf40 | | | | | |
| 11 | 4608020 | 4609135 | OR52I2 | | | | | |
| 11 | 4615268 | 4616243 | OR52I1 | | | | | |
| 11 | 4619901 | 4629489 | TRIM68 | | | | | |
| 11 | 4660944 | 4662068 | OR51D1 | | | | | |
| 11 | 4665155 | 4676716 | OR51E1 | | | | | |
| 11 | 4701400 | 4719076 | OR51E2 | | | | | |
| 11 | 4790208 | 4791147 | OR51F1 | | | | | |
| 11 | 4824662 | 4825610 | OR52R1 | | | | | |
| 11 | 4842615 | 4843644 | OR51F2 | | | | | |
| 11 | 4869466 | 4870438 | OR51S1 | | | | | |
| 11 | 4903048 | 4904113 | OR51T1 | | | | | |
| 11 | 4928599 | 4929538 | OR51A7 | | | | | |
| 11 | 4935948 | 4936893 | OR51G2 | | | | | |
| 11 | 4944603 | 4945569 | OR51G1 | | | | | |
| 11 | 4967388 | 4968330 | OR51A4 | | | | | |
| 11 | 4976001 | 4976943 | OR51A2 | | | | | |
| 11 | 5009423 | 5013659 | MMP26 | | | | | |
| 11 | 5020212 | 5021160 | OR51L1 | | | | | |
| 11 | 5067755 | 5068691 | OR52J3 | | | | | |
| 11 | 5079879 | 5080857 | OR52E2 | | | | | |
| 11 | 5152921 | 5153872 | OR52A5 | | | | | |
| 11 | 5172660 | 5173599 | OR52A1 | | | | | |
| 11 | 5220964 | 5221930 | OR51V1 | | | | | |
| 11 | 5246695 | 5248301 | HBB | | | HBB | | |
| 11 | 5254058 | 5255858 | HBD | | | | | |
| 11 | 5263184 | 5264822 | HBBP1 | | | | | |
| 11 | 5265783 | 5266776 | BGLT3 | | | | | |
| 11 | 5269501 | 5271087 | HBG1 | | | | | |
| 11 | 5274420 | 5276011 | HBG2 | | | | | |
| 11 | 5289579 | 5291373 | HBE1 | | | | | |
| 11 | 5322243 | 5323176 | OR51B4 | | | | | |
| 11 | 5344527 | 5345582 | OR51B2 | | | | | |
| 11 | 5362112 | 5526882 | OR51B5 | | | | | |
| 11 | 5372737 | 5373676 | OR51B6 | | | | | |
| 11 | 5410606 | 5411664 | OR51M1 | | | | | |
| 11 | 5443340 | 5444436 | OR51Q1 | | | | | |
| 11 | 5461771 | 5462783 | OR51I1 | | | | | |
| 11 | 5474637 | 5475707 | OR51I2 | | | | | |
| 11 | 5509914 | 5510978 | OR52D1 | | | | | |
| 11 | 5528529 | 5531153 | UBQLN3 | | | | | |
| 11 | 5535622 | 5537956 | UBQLNL | | | | | |
| 11 | 5539539 | 5543015 | OLFM5P | | | | | |
| 11 | 5565790 | 5566753 | OR52H1 | | | | | |

| | | | | | | | | |
|---|---|---|---|---|---|---|---|---|
| 11 | 5602106 | 5603114 | OR52B6 | | | | | |
| 11 | 5617330 | 5634188 | TRIM6 | | | | | |
| 11 | 5617864 | 5665625 | TRIM6-TRIM34 | | | | | |
| 11 | 5641173 | 5662526 | TRIM34 | | | | | |
| 11 | 5684424 | 5706339 | TRIM5 | | | | | |
| 11 | 5710816 | 5732093 | TRIM22 | | | | | |
| 11 | 5757677 | 5758768 | OR56B1 | | | | | |
| 11 | 5775922 | 5776959 | OR52N4 | | | | | |
| 11 | 5798863 | 5799897 | OR52N5 | | | | | |
| 11 | 5809083 | 5810046 | OR52N1 | | | | | |
| 11 | 5841565 | 5842531 | OR52N2 | | | | | |
| 11 | 5862185 | 5863127 | OR52E6 | | | | | |
| 11 | 5877978 | 5878932 | OR52E8 | | | | | |
| 11 | 5905522 | 5906461 | OR52E4 | | | | | |
| 11 | 5968576 | 5969524 | OR56A3 | | | | | |
| 11 | 5988782 | 5989724 | OR56A5 | | | | | |
| 11 | 6007121 | 6008215 | OR52L1 | | | | | |
| 11 | 6023280 | 6024378 | OR56A4 | | | | | |
| 11 | 6047900 | 6048971 | OR56A1 | | | | | |
| 11 | 6128913 | 6130065 | OR56B4 | | | | | |
| 11 | 6190584 | 6191556 | OR52B2 | | | | | |
| 11 | 6220453 | 6221416 | OR52W1 | | | | | |
| 11 | 6226797 | 6232365 | C11orf42 | | | | | |
| 11 | 6232563 | 6255941 | FAM160A2 | | | | | |
| 11 | 6260321 | 6265707 | CNGA4 | | | | | |
| 11 | 6280903 | 6293357 | CCKBR | | CCKBR | | CCKBR | |
| 11 | 6340175 | 6341740 | PRKCDBP | | | | | |
| 11 | 6411643 | 6416228 | SMPD1 | | | | | |
| 11 | 6416353 | 6440702 | APBB1 | APBB1 | APBB1 | | APBB1 | APBB1 |
| 11 | 6452267 | 6462254 | HPX | | | | | |
| 11 | 6469842 | 6495689 | TRIM3 | | | | | |
| 11 | 6495912 | 6502709 | ARFIP2 | | | | | |
| 11 | 6502676 | 6505911 | TIMM10B | | | | | |
| 11 | 6518525 | 6593254 | DNHD1 | | | | | |
| 11 | 6621143 | 6624880 | RRP8 | | | | | |
| 11 | 6624937 | 6632105 | ILK | | | | | |
| 11 | 6632047 | 6633475 | TAF10 | | | | | |
| 11 | 6633996 | 6640692 | TPP1 | | | TPP1 | | |
| 11 | 6642554 | 6677080 | DCHS1 | | | | | |
| 11 | 6701615 | 6704632 | MRPL17 | | | | | |
| 11 | 6734380 | 6743110 | GVINP1 | | | | | |
| 11 | 6789237 | 6790188 | OR2AG2 | | | | | |
| 11 | 6806247 | 6807245 | OR2AG1 | | | | | |
| 11 | 6815755 | 6817139 | OR6A2 | | | | | |
| 11 | 6866913 | 6867867 | OR10A5 | | | | | |
| 11 | 6890985 | 6891897 | OR10A2 | | | | | |
| 11 | 6897855 | 6898850 | OR10A4 | | | | | |
| 11 | 6912804 | 6913731 | OR2D2 | | | | | |
| 11 | 6942232 | 6943225 | OR2D3 | | | | | |
| 11 | 6947653 | 6979278 | ZNF215 | | | | | |
| 11 | 7020548 | 7041586 | ZNF214 | | | | | |
| 11 | 7041699 | 7092757 | NLRP14 | | | | | |
| 11 | 7110164 | 7112379 | RBMXL2 | | | | | |
| 11 | 7255996 | 7256068 | MIR302E | | | | | |
| 11 | 7273180 | 7490276 | SYT9 | | | | | |
| 11 | 7458867 | 7487066 | LOC100506258 | | | | | |
| 11 | 7506599 | 7532606 | OLFML1 | | | | | |
| 11 | 7534995 | 7674996 | PPFIBP2 | | | | | |
| 11 | 7686325 | 7695474 | CYB5R2 | | | | | |
| 11 | 7711154 | 7727941 | OVCH2 | | | | | |
| 11 | 7817520 | 7818489 | OR5P2 | | | | | |
| 11 | 7846583 | 7847519 | OR5P3 | | | | | |
| 11 | 7870597 | 7871118 | OR5E1P | | | | | |
| 11 | 7872297 | 7927502 | LOC283299 | | | | | |
| 11 | 7949264 | 7950209 | OR10A6 | | | | | |
| 11 | 7960122 | 7961067 | OR10A3 | | | | | |

| | | | | | | | | |
|---|---|---|---|---|---|---|---|---|
| 11 | 7981155 | 7985059 | NLRP10 | | | | | |
| 11 | 8008866 | 8017719 | EIF3F | | | | | |
| 11 | 8032824 | 8038056 | CASC23 | | | | | |
| 11 | 8060179 | 8127654 | TUB | | TUB | | | |
| 11 | 8127596 | 8190590 | RIC3 | | | | | |
| 11 | 8245850 | 8290182 | LMO1 | | | | | |
| 11 | 8413412 | 8615836 | STK33 | | | | | |
| 11 | 8633583 | 8680383 | TRIM66 | | | | | |
| 11 | 8703994 | 8711419 | RPL27A | | | | | |
| 11 | 8705773 | 8705903 | SNORA3A | | | | | |
| 11 | 8706985 | 8707116 | SNORA3B | | | | | |
| 11 | 8714898 | 8932498 | ST5 | | | | | |
| 11 | 8790324 | 8831823 | LOC102724784 | | | | | |
| 11 | 8932700 | 8941626 | AKIP1 | | | | | |
| 11 | 8941622 | 8954553 | C11orf16 | | | | | |
| 11 | 8959118 | 8964580 | ASCL3 | | | | | |
| 11 | 8968747 | 8997830 | TMEM9B | | | | | |
| 11 | 9002122 | 9025596 | NRIP3 | | | | | |
| 11 | 9041046 | 9113150 | SCUBE2 | | | | | |
| 11 | 9111858 | 9111926 | MIR5691 | | | | | |
| 11 | 9115909 | 9117737 | KRT8P41 | | | | | |
| 11 | 9160374 | 9286873 | DENND5A | | | | | |
| 11 | 9302200 | 9336315 | TMEM41B | | | | | |
| 11 | 9406168 | 9469674 | IPO7 | | | | | |
| 11 | 9450312 | 9450501 | SNORA23 | | | | | |
| 11 | 9481102 | 9482245 | LOC644656 | | | | | |
| 11 | 9482511 | 9550071 | ZNF143 | | | | | |
| 11 | 9595227 | 9611313 | WEE1 | | | | | |
| 11 | 9685623 | 9774538 | SWAP70 | | | | | |
| 11 | 9776316 | 9781080 | LOC440028 | | | | | |
| 11 | 9779839 | 10315754 | SBF2 | | | | | |
| 11 | 9860688 | 9950808 | LOC101928008 | | | | | |
| 11 | 10326526 | 10328949 | ADM | | | ADM | | |
| 11 | 10329859 | 10452220 | CAND1.11 | | | | | |
| 11 | 10471867 | 10529126 | AMPD3 | | | | | |
| 11 | 10529433 | 10530723 | MTRNR2L8 | | | | | |
| 11 | 10529816 | 10529873 | MIR4485 | | | | | |
| 11 | 10533224 | 10562774 | RNF141 | | | | | |
| 11 | 10562782 | 10621479 | MRVI1 | | | | | |
| 11 | 10579412 | 10590365 | LYVE1 | | | | | |
| 11 | 10594637 | 10715535 | MRVI1 | | | | | |
| 11 | 10772533 | 10801302 | CTR9 | | | | | |
| 11 | 10818592 | 10830582 | EIF4G2 | | | EIF4G2 | | |
| 11 | 10823013 | 10823155 | SNORD97 | | | | | |
| 11 | 10830843 | 10844478 | LOC101928053 | | | | | |
| 11 | 10874250 | 10900823 | ZBED5 | | | | | |
| 11 | 11292420 | 11643561 | GALNT18 | | | | | |
| 11 | 11373488 | 11374904 | CSNK2A3 | | | | | |
| 11 | 11678197 | 11678269 | MIR4299 | | | | | |
| 11 | 11804681 | 11804769 | MIR8070 | | | | | |
| 11 | 11862969 | 11980872 | USP47 | | | | | |
| 11 | 11984542 | 12030917 | DKK3 | | | | | |
| 11 | 12052421 | 12083332 | LOC105376554 | | | | | |
| 11 | 12132122 | 12285337 | MICAL2 | | | | | |
| 11 | 12185229 | 12185314 | MIR6124 | | | | | |
| 11 | 12308446 | 12380691 | MICALCL | | | | | |
| 11 | 12399025 | 12556903 | PARVA | | | | | |
| 11 | 12695968 | 12966284 | TEAD1 | | | | | |
| 11 | 13001080 | 13011095 | LINC00958 | | | | | |
| 11 | 13030969 | 13033653 | RASSF10 | | | | | |
| 11 | 13299273 | 13408812 | ARNTL | | | | | |
| 11 | 13409542 | 13484844 | BTBD10 | | | | | |
| 11 | 13513591 | 13517722 | PTH | | | | | |
| 11 | 13690205 | 13753893 | FAR1 | | | | | |
| 11 | 13942993 | 13946410 | LOC101928132 | | | | | |
| 11 | 13984183 | 14289679 | SPON1 | | | | | |

| | | | | | | | | |
|---|---|---|---|---|---|---|---|---|
| 11 | 14299465 | 14386052 | RRAS2 | | | | RRAS2 | |
| 11 | 14479048 | 14521441 | COPB1 | | | COPB1 | | |
| 11 | 14526421 | 14541991 | PSMA1 | | | | | |
| 11 | 14665268 | 14893604 | PDE3B | | | | | |
| 11 | 14899555 | 14913751 | CYP2R1 | | | | | |
| 11 | 14988214 | 14993832 | CALCA | | | CALCA | | |
| 11 | 15095145 | 15100177 | CALCB | | | CALCB | | |
| 11 | 15133969 | 15268756 | INSC | | | | | |
| 11 | 15665430 | 15726914 | LOC102724957 | | | | | |
| 11 | 15987994 | 16497935 | SOX6 | | | SOX6 | | |
| 11 | 15991078 | 15991167 | MIR6073 | | | | | |
| 11 | 16760147 | 16779901 | C11orf58 | | | | | |
| 11 | 16809206 | 17035963 | PLEKHA7 | | | | | |
| 11 | 17073432 | 17074591 | OR7E14P | | | | | |
| 11 | 17095938 | 17099220 | RPS13 | | | | | |
| 11 | 17108123 | 17191354 | PIK3C2A | | | | | |
| 11 | 17298285 | 17353070 | NUCB2 | | | | | |
| 11 | 17353588 | 17371520 | LOC105376575 | | | | | |
| 11 | 17373308 | 17398868 | NCR3LG1 | | | | | |
| 11 | 17406795 | 17410878 | KCNJ11 | | KCNJ11 | KCNJ11 | | |
| 11 | 17414431 | 17498392 | ABCC8 | | ABCC8 | ABCC8 | | |
| 11 | 17515441 | 17565963 | USH1C | | USH1C | USH1C | | USH1C |
| 11 | 17568919 | 17667491 | OTOG | | | | | |
| 11 | 17716813 | 17718486 | LOC102723330 | | | | | |
| 11 | 17741109 | 17743678 | MYOD1 | | | | | |
| 11 | 17757494 | 17804602 | KCNC1 | | KCNC1 | KCNC1 | KCNC1 | |
| 11 | 17809595 | 18034709 | SERGEF | | | | | |
| 11 | 18042083 | 18062335 | TPH1 | | | | | |
| 11 | 18101889 | 18127638 | SAAL1 | | | | | |
| 11 | 18134018 | 18137679 | SAA3P | | | | | |
| 11 | 18142501 | 18160027 | MRGPRX3 | | | | | |
| 11 | 18194383 | 18195827 | MRGPRX4 | | | | | |
| 11 | 18230684 | 18235111 | LOC494141 | | | | | |
| 11 | 18252901 | 18258384 | SAA4 | | | | | |
| 11 | 18252901 | 18270221 | SAA2-SAA4 | | | | | |
| 11 | 18259779 | 18270221 | SAA2 | | | | | |
| 11 | 18287807 | 18291523 | SAA1 | | | | | |
| 11 | 18300216 | 18343721 | HPS5 | | | | | |
| 11 | 18343815 | 18388590 | GTF2H1 | | | | | |
| 11 | 18415935 | 18429765 | LDHA | | | | | |
| 11 | 18433852 | 18472793 | LDHC | | | | | |
| 11 | 18477373 | 18501147 | LDHAL6A | | | | | |
| 11 | 18501857 | 18548503 | TSG101 | | | TSG101 | | |
| 11 | 18552949 | 18610293 | UEVLD | | | | | |
| 11 | 18621350 | 18656020 | SPTY2D1 | | | | | |
| 11 | 18720281 | 18726332 | TMEM86A | | | | | |
| 11 | 18725851 | 18747777 | IGSF22 | | | | | |
| 11 | 18749474 | 18814268 | PTPN5 | | | | | |
| 11 | 18955359 | 18956549 | MRGPRX1 | | | | | |
| 11 | 19076002 | 19082228 | MRGPRX2 | | | | | |
| 11 | 19138691 | 19197967 | ZDHHC13 | | | ZDHHC13 | | |
| 11 | 19203576 | 19232118 | CSRP3 | | | | | |
| 11 | 19245609 | 19263202 | E2F8 | | | | | |
| 11 | 19372270 | 19545625 | NAV2 | | NAV2 | | | |
| 11 | 19596856 | 19596919 | MIR4486 | | | | | |
| 11 | 19732479 | 19736218 | LOC100126784 | | | | | |
| 11 | 19734880 | 20143147 | NAV2 | | NAV2 | | | |
| 11 | 19781549 | 19781629 | MIR4694 | | | | | |
| 11 | 20044101 | 20070849 | NAV2 | | NAV2 | | | |
| 11 | 20177759 | 20181870 | DBX1 | | | | | |
| 11 | 20385230 | 20389200 | HTATIP2 | | | | | |
| 11 | 20409075 | 20530879 | PRMT3 | | | | | |
| 11 | 20620945 | 20676610 | SLC6A5 | | | SLC6A5 | SLC6A5 | |
| 11 | 20691096 | 21597229 | NELL1 | | NELL1 | NELL1 | | |
| 11 | 22214721 | 22304913 | ANO5 | | ANO5 | ANO5 | | |
| 11 | 22359666 | 22401046 | SLC17A6 | | | | | |

| | | | | | | | | |
|---|---|---|---|---|---|---|---|---|
| 11 | 22467217 | 22513565 | LINC01495 | | | | | |
| 11 | 22644078 | 22647387 | FANCF | | FANCF | | | |
| 11 | 22688159 | 22834547 | GAS2 | | | | | |
| 11 | 22843597 | 22851382 | SVIP | | | | | |
| 11 | 22868467 | 22881972 | CCDC179 | | | | | |
| 11 | 23440650 | 23440736 | MIR8054 | | | | | |
| 11 | 24518515 | 25104186 | LUZP2 | | | | | |
| 11 | 26210669 | 26684836 | ANO3 | | ANO3 | ANO3 | | |
| 11 | 26307131 | 26309554 | LOC105376599 | | | | | |
| 11 | 26353677 | 26684836 | ANO3 | | ANO3 | ANO3 | | |
| 11 | 26580578 | 26593815 | MUC15 | | | | | |
| 11 | 26688565 | 26743574 | SLC5A12 | | | | | |
| 11 | 27015627 | 27018632 | FIBIN | | | | | |
| 11 | 27062508 | 27241660 | BBOX1 | | | | | |
| 11 | 27360060 | 27384795 | CCDC34 | | | | | |
| 11 | 27387507 | 27494334 | LGR4 | | | | | |
| 11 | 27493277 | 27503980 | LOC105376671 | | | | | |
| 11 | 27515964 | 27528326 | LIN7C | | | LIN7C | | |
| 11 | 27528398 | 27719718 | BDNF | BDNF | BDNF | | BDNF | |
| 11 | 27536516 | 27536594 | MIR8087 | | | | | |
| 11 | 27639172 | 27656174 | LINC00678 | | | | | |
| 11 | 27676441 | 27743605 | BDNF | BDNF | BDNF | | BDNF | |
| 11 | 28042162 | 28129746 | KIF18A | | | | | |
| 11 | 28078361 | 28078457 | MIR610 | | | | | |
| 11 | 28129797 | 28355054 | METTL15 | | | | | |
| 11 | 28499027 | 28499095 | MIR8068 | | | | | |
| 11 | 30001667 | 30003940 | LINC01616 | | | | | |
| 11 | 30031287 | 30038577 | KCNA4 | | KCNA4 | KCNA4 | KCNA4 | |
| 11 | 30252562 | 30256824 | FSHB | | FSHB | | | |
| 11 | 30344645 | 30359770 | ARL14EP | | | | | |
| 11 | 30406039 | 30602040 | MPPED2 | | | | | |
| 11 | 30885149 | 31014233 | DCDC5 | | | | | |
| 11 | 31284170 | 31391357 | DCDC1 | | | | | |
| 11 | 31391376 | 31454382 | DNAJC24 | | | | | |
| 11 | 31453945 | 31531175 | IMMP1L | | | | | |
| 11 | 31531275 | 31806073 | ELP4 | | | | | |
| 11 | 31806339 | 31828473 | PAX6 | | PAX6 | PAX6 | | |
| 11 | 31838113 | 31908587 | DKFZp686K1684 | | | | | |
| 11 | 31848113 | 31850850 | PAUPAR | | | | | |
| 11 | 32057524 | 32062864 | LOC100506675 | | | | | |
| 11 | 32112476 | 32127272 | RCN1 | | | | | |
| 11 | 32165287 | 32165458 | SNORA88 | | | | | |
| 11 | 32409321 | 32461003 | WT1 | | | WT1 | | |
| 11 | 32605312 | 32624419 | EIF3M | | | | | |
| 11 | 32623625 | 32816204 | CCDC73 | | | | | |
| 11 | 32851480 | 32879669 | PRRG4 | | | | | |
| 11 | 32914723 | 33001814 | QSER1 | | | | | |
| 11 | 33037409 | 33055128 | DEPDC7 | | | | | |
| 11 | 33060962 | 33095109 | TCP11L1 | | | | | |
| 11 | 33097695 | 33101000 | LINC00294 | | | | | |
| 11 | 33106129 | 33213142 | CSTF3 | | | | | |
| 11 | 33278217 | 33378568 | HIPK3 | | | | | |
| 11 | 33563876 | 33695646 | KIAA1549L | | | | | |
| 11 | 33719653 | 33722286 | C11orf91 | | | | | |
| 11 | 33724555 | 33758025 | CD59 | | | CD59 | | |
| 11 | 33762489 | 33797216 | FBXO3 | | | | | |
| 11 | 33880122 | 33913836 | LMO2 | | | | | |
| 11 | 34073229 | 34124157 | CAPRIN1 | | | | | |
| 11 | 34127110 | 34168458 | NAT10 | | | | | |
| 11 | 34172533 | 34379555 | ABTB2 | | | | | |
| 11 | 34460471 | 34493607 | CAT | | | | | |
| 11 | 34500341 | 34535347 | ELF5 | | | | | |
| 11 | 34642587 | 34684834 | EHF | | | | | |
| 11 | 34903842 | 34937958 | APIP | | | | | |
| 11 | 34937676 | 35017675 | PDHX | | | PDHX | | |
| 11 | 34963383 | 34963467 | MIR1343 | | | | | |

| | | | | | | | | |
|---|---|---|---|---|---|---|---|---|
| 11 | 35154196 | 35159579 | LOC100507144 | | | | | |
| 11 | 35160416 | 35253949 | CD44 | | | | | |
| 11 | 35272751 | 35441610 | SLC1A2 | | SLC1A2 | SLC1A2 | SLC1A2 | SLC1A2 |
| 11 | 35453375 | 35551848 | PAMR1 | | | | | |
| 11 | 35639734 | 35642421 | FJX1 | | | | | |
| 11 | 35684299 | 35832603 | TRIM44 | | | | | |
| 11 | 35965530 | 36253686 | LDLRAD3 | | | | | |
| 11 | 36031647 | 36031754 | MIR3973 | | | | | |
| 11 | 36084833 | 36099451 | LOC101928510 | | | | | |
| 11 | 36293841 | 36310999 | COMMD9 | | | | | |
| 11 | 36317724 | 36486754 | PRR5L | | | | | |
| 11 | 36505316 | 36531863 | TRAF6 | | | | | |
| 11 | 36589562 | 36601310 | RAG1 | | | | | |
| 11 | 36613492 | 36619829 | RAG2 | | | | | |
| 11 | 36616042 | 36680841 | C11orf74 | | | | | |
| 11 | 37960150 | 37976213 | LOC105376633 | | | | | |
| 11 | 38639813 | 38667906 | LOC103312105 | | | | | |
| 11 | 38670407 | 38676799 | LINC01493 | | | | | |
| 11 | 40135750 | 41481186 | LRRC4C | | | | | |
| 11 | 41736117 | 41857992 | LINC01499 | | | | | |
| 11 | 42023271 | 42107008 | LOC101928591 | | | | | |
| 11 | 42209292 | 42275240 | LOC100507205 | | | | | |
| 11 | 43283053 | 43290919 | HNRNPKP3 | | | | | |
| 11 | 43333504 | 43366080 | API5 | | | API5 | | |
| 11 | 43380434 | 43516483 | TTC17 | | | | | |
| 11 | 43581205 | 43581303 | MIR670 | | | | | |
| 11 | 43590855 | 43591950 | MIR670HG | | | | | |
| 11 | 43602943 | 43603033 | MIR129 | | | | | |
| 11 | 43702142 | 43878169 | HSD17B12 | | | | | |
| 11 | 43902356 | 43941825 | ALKBH3 | | | | | |
| 11 | 43918852 | 43921424 | SEC14L1P1 | | | | | |
| 11 | 43930838 | 43942494 | ALKBH3 | | | | | |
| 11 | 43964105 | 43965433 | C11orf96 | | | | | |
| 11 | 44069530 | 44081527 | ACCSL | | | | | |
| 11 | 44087728 | 44105569 | ACCS | | | | | |
| 11 | 44117098 | 44266980 | EXT2 | | | EXT2 | | |
| 11 | 44282277 | 44331716 | ALX4 | | | ALX4 | | |
| 11 | 44587140 | 44641315 | CD82 | | | | | |
| 11 | 44785975 | 44953977 | TSPAN18 | | | | | |
| 11 | 44953898 | 44972857 | TP53I11 | | | | | |
| 11 | 44995452 | 44999578 | LOC221122 | | | | | |
| 11 | 45115563 | 45256675 | PRDM11 | | | | | |
| 11 | 45261852 | 45307884 | SYT13 | | | | | |
| 11 | 45376921 | 45378208 | LOC101928812 | | | | | |
| 11 | 45392947 | 45410059 | LOC399886 | | | | | |
| 11 | 45669238 | 45687206 | CHST1 | | CHST1 | | | |
| 11 | 45713253 | 45713326 | MIR7154 | | | | | |
| 11 | 45743857 | 45746106 | LOC100507384 | | | | | |
| 11 | 45792982 | 45793909 | DKFZp779M0652 | | | | | |
| 11 | 45825622 | 45834567 | SLC35C1 | | | | | |
| 11 | 45868668 | 45904799 | CRY2 | | | | | |
| 11 | 45907046 | 45928016 | MAPK8IP1 | | MAPK8IP1 | MAPK8IP1 | | |
| 11 | 45928085 | 45928833 | C11orf94 | | | | | |
| 11 | 45931219 | 45939674 | PEX16 | | | | | |
| 11 | 45943171 | 45950647 | GYLTL1B | | | | | |
| 11 | 45950869 | 46142985 | PHF21A | | | | | |
| 11 | 46193505 | 46198656 | LOC101928894 | | | | | |
| 11 | 46299188 | 46342972 | CREB3L1 | | | | | |
| 11 | 46354454 | 46402104 | DGKZ | | | | | |
| 11 | 46397951 | 46398034 | MIR4688 | | | | | |
| 11 | 46402333 | 46405387 | MDK | | | | | |
| 11 | 46406341 | 46408158 | CHRM4 | | | CHRM4 | | |
| 11 | 46417961 | 46615619 | AMBRA1 | | | | | |
| 11 | 46473354 | 46473437 | MIR3160 | | | | | |
| 11 | 46624855 | 46638777 | HARBI1 | | | | | |
| 11 | 46638825 | 46697568 | ATG13 | | | | | |

| | | | | | | | | |
|---|---|---|---|---|---|---|---|---|
| 11 | 46698624 | 46722215 | ARHGAP1 | | | | | |
| 11 | 46722316 | 46727466 | ZNF408 | | | | | |
| 11 | 46740715 | 46761058 | F2 | | | | | |
| 11 | 46765083 | 46867859 | CKAP5 | | CKAP5 | | | |
| 11 | 46774674 | 46774742 | MIR5582 | | | | | |
| 11 | 46783938 | 46784049 | SNORD67 | | | | | |
| 11 | 46867961 | 46940173 | LRP4 | | LRP4 | | | |
| 11 | 46958239 | 47185931 | C11orf49 | | | | | |
| 11 | 47185848 | 47198676 | ARFGAP2 | | | | | |
| 11 | 47199072 | 47208010 | PACSIN3 | | | | | |
| 11 | 47201161 | 47201288 | MIR6745 | | | | | |
| 11 | 47236492 | 47260769 | DDB2 | | | | | |
| 11 | 47260852 | 47270398 | ACP2 | | | | | |
| 11 | 47269850 | 47290584 | NR1H3 | | | | | |
| 11 | 47290926 | 47351582 | MADD | | MADD | | MADD | |
| 11 | 47292207 | 47293661 | LOC101928943 | | | | | |
| 11 | 47352956 | 47374253 | MYBPC3 | | | MYBPC3 | | |
| 11 | 47376408 | 47400127 | SPI1 | | | | | |
| 11 | 47422520 | 47422593 | MIR4487 | | | | | |
| 11 | 47428826 | 47438051 | SLC39A13 | | | | | |
| 11 | 47440319 | 47448024 | PSMC3 | | | | | |
| 11 | 47459307 | 47470730 | RAPSN | | | RAPSN | RAPSN | RAPSN |
| 11 | 47487488 | 47574792 | CELF1 | | | | | |
| 11 | 47586981 | 47595013 | PTPMT1 | | | | | |
| 11 | 47593748 | 47600567 | KBTBD4 | | | | | |
| 11 | 47600561 | 47606115 | NDUFS3 | | | NDUFS3 | | |
| 11 | 47608229 | 47610746 | FAM180B | | | | | |
| 11 | 47611215 | 47615961 | C1QTNF4 | | | | | |
| 11 | 47638857 | 47664206 | MTCH2 | | | | | |
| 11 | 47681142 | 47736928 | AGBL2 | | | | | |
| 11 | 47738061 | 47789030 | FNBP4 | | | | | |
| 11 | 47799634 | 47870096 | NUP160 | | | | | |
| 11 | 48002109 | 48192394 | PTPRJ | | | | | |
| 11 | 48118333 | 48118410 | MIR3161 | | | | | |
| 11 | 48238361 | 48239291 | OR4B1 | | | | | |
| 11 | 48266655 | 48267567 | OR4X2 | | | | | |
| 11 | 48285412 | 48286330 | OR4X1 | | | | | |
| 11 | 48327774 | 48328704 | OR4S1 | | | | | |
| 11 | 48346492 | 48347482 | OR4C3 | | | | | |
| 11 | 48366899 | 48373999 | OR4C45 | | | | | |
| 11 | 48510344 | 48511274 | OR4A47 | | | | | |
| 11 | 49053151 | 49059529 | TRIM49B | | | | | |
| 11 | 49075265 | 49080664 | TRIM64C | | | | | |
| 11 | 49168186 | 49230222 | FOLH1 | | FOLH1 | | | |
| 11 | 49580079 | 49831969 | LOC440040 | | | | | |
| 11 | 49973942 | 49974971 | OR4C13 | | | | | |
| 11 | 50003008 | 50004071 | OR4C12 | | | | | |
| 11 | 50238998 | 50257633 | LOC441601 | | | | | |
| 11 | 50368317 | 50379802 | LOC646813 | | | | | |
| 11 | 51411377 | 51412448 | OR4A5 | | | | | |
| 11 | 51515281 | 51516211 | OR4C46 | | | | | |
| 11 | 55029657 | 55038595 | TRIM48 | | | | | |
| 11 | 55061178 | 55065708 | TRIM51HP | | | | | |
| 11 | 55110676 | 55111663 | OR4A16 | | | | | |
| 11 | 55135359 | 55136394 | OR4A15 | | | | | |
| 11 | 55321782 | 55322895 | OR4C15 | | | | | |
| 11 | 55339603 | 55340536 | OR4C16 | | | | | |
| 11 | 55370829 | 55371874 | OR4C11 | | | | | |
| 11 | 55405833 | 55406772 | OR4P4 | | | | | |
| 11 | 55418379 | 55419315 | OR4S2 | | | | | |
| 11 | 55432642 | 55433572 | OR4C6 | | | | | |
| 11 | 55540913 | 55541858 | OR5D13 | | | | | |
| 11 | 55563031 | 55563976 | OR5D14 | | | | | |
| 11 | 55578942 | 55579878 | OR5L1 | | | | | |
| 11 | 55587105 | 55588047 | OR5D18 | | | | | |
| 11 | 55594694 | 55595630 | OR5L2 | | | | | |

| | | | | | | | | | |
|---|---|---|---|---|---|---|---|---|---|
| 11 | 55606227 | 55607214 | OR5D16 | | | | | | |
| 11 | 55650772 | 55659284 | TRIM51 | | | | | | |
| 11 | 55681125 | 55682058 | OR5W2 | | | | | | |
| 11 | 55702931 | 55703876 | OR5I1 | | | | | | |
| 11 | 55735033 | 55735939 | OR10AG1 | | | | | | |
| 11 | 55746178 | 55753881 | OR7E5P | | | | | | |
| 11 | 55761156 | 55762101 | OR5F1 | | | | | | |
| 11 | 55797894 | 55798869 | OR5AS1 | | | | | | |
| 11 | 55860783 | 55861716 | OR8I2 | | | | | | |
| 11 | 55872518 | 55873457 | OR8H2 | | | | | | |
| 11 | 55889848 | 55890787 | OR8H3 | | | | | | |
| 11 | 55904246 | 55905194 | OR8J3 | | | | | | |
| 11 | 55926869 | 55927793 | OR8K5 | | | | | | |
| 11 | 55944093 | 55945032 | OR5J2 | | | | | | |
| 11 | 55999581 | 56000661 | OR5T2 | | | | | | |
| 11 | 56019675 | 56020698 | OR5T3 | | | | | | |
| 11 | 56043114 | 56044095 | OR5T1 | | | | | | |
| 11 | 56057602 | 56058538 | OR8H1 | | | | | | |
| 11 | 56085782 | 56086721 | OR8K3 | | | | | | |
| 11 | 56113514 | 56114474 | OR8K1 | | | | | | |
| 11 | 56127690 | 56128764 | OR8J1 | | | | | | |
| 11 | 56143099 | 56143975 | OR8U8 | | | | | | |
| 11 | 56143099 | 56144029 | OR8U1 | | | | | | |
| 11 | 56184733 | 56185708 | OR5R1 | | | | | | |
| 11 | 56229944 | 56230877 | OR5M9 | | | | | | |
| 11 | 56236963 | 56238014 | OR5M3 | | | | | | |
| 11 | 56257910 | 56258846 | OR5M8 | | | | | | |
| 11 | 56309815 | 56310733 | OR5M11 | | | | | | |
| 11 | 56344249 | 56345197 | OR5M10 | | | | | | |
| 11 | 56380030 | 56380978 | OR5M1 | | | OR5M1 | | | |
| 11 | 56408964 | 56409915 | OR5AP2 | | | | | | |
| 11 | 56431161 | 56432094 | OR5AR1 | | | | | | |
| 11 | 56467863 | 56468781 | OR9G1 | | | | | | |
| 11 | 56467863 | 56468781 | OR9G9 | | | | | | |
| 11 | 56510303 | 56511287 | OR9G4 | | | | | | |
| 11 | 56511348 | 56511457 | MIR6128 | | | | | | |
| 11 | 56615953 | 56645554 | LOC101927120 | | | | | | |
| 11 | 56756388 | 56757318 | OR5AK2 | | | | | | |
| 11 | 56805008 | 56805935 | OR5AK4P | | | | | | |
| 11 | 56949220 | 56959188 | LRRC55 | | | | | | |
| 11 | 57001051 | 57004927 | APLNR | | | | | | |
| 11 | 57067102 | 57092413 | TNKS1BP1 | | | | | | |
| 11 | 57093458 | 57103351 | SSRP1 | | | | | | |
| 11 | 57105840 | 57137549 | P2RX3 | | | | | | |
| 11 | 57144241 | 57148623 | PRG3 | | | | | | |
| 11 | 57154259 | 57158130 | PRG2 | | | | | | |
| 11 | 57174426 | 57195053 | SLC43A3 | | | | | | |
| 11 | 57228009 | 57245012 | RTN4RL2 | | | | | | |
| 11 | 57252003 | 57283192 | SLC43A1 | | | | | | |
| 11 | 57295935 | 57298232 | TIMM10 | | | TIMM10 | | | |
| 11 | 57310113 | 57317747 | SMTNL1 | | | | | | |
| 11 | 57319127 | 57335803 | UBE2L6 | | | | | | |
| 11 | 57365026 | 57382326 | SERPING1 | | | | | | |
| 11 | 57408670 | 57408759 | MIR130A | | | | | | |
| 11 | 57412559 | 57417417 | YPEL4 | | | | | | |
| 11 | 57425215 | 57429337 | CLP1 | | | | | | |
| 11 | 57435473 | 57468659 | ZDHHC5 | | | | | | |
| 11 | 57471180 | 57479795 | MED19 | | | | | | |
| 11 | 57479994 | 57508445 | TMX2 | | | | | | |
| 11 | 57479994 | 57586652 | TMX2-CTNND1 | | | | | | |
| 11 | 57508721 | 57510883 | C11orf31 | | | | | | |
| 11 | 57510985 | 57519253 | BTBD18 | | | | | | |
| 11 | 57529233 | 57586652 | CTNND1 | | | | | | |
| 11 | 57791352 | 57949038 | OR9Q1 | | | | | | |
| 11 | 57798424 | 57799378 | OR6Q1 | | | | | | |
| 11 | 57885971 | 57886916 | OR9I1 | | | | | | |

| | | | | | | | | | |
|---|---|---|---|---|---|---|---|---|---|
| 11 | 57957905 | 57958990 | OR9Q2 | | | | | | |
| 11 | 57970675 | 57971653 | OR1S2 | | | | | | |
| 11 | 57982216 | 57983194 | OR1S1 | | | | | | |
| 11 | 57995353 | 57996390 | OR10Q1 | | | | | | |
| 11 | 58034263 | 58035732 | OR10W1 | | | | | | |
| 11 | 58125597 | 58126542 | OR5B17 | | | | | | |
| 11 | 58169937 | 58170882 | OR5B3 | | | | | | |
| 11 | 58189737 | 58190786 | OR5B2 | | | | | | |
| 11 | 58206592 | 58207646 | OR5B12 | | | | | | |
| 11 | 58274648 | 58275578 | OR5B21 | | | | | | |
| 11 | 58294343 | 58345712 | LPXN | | | | | | |
| 11 | 58346586 | 58389023 | ZFP91 | | | | | | |
| 11 | 58346586 | 58393205 | ZFP91-CNTF | | | | | | |
| 11 | 58390145 | 58393205 | CNTF | | | | | | |
| 11 | 58476229 | 58499447 | GLYAT | | | | | | |
| 11 | 58601539 | 58611997 | GLYATL2 | | | | | | |
| 11 | 58695101 | 58724547 | GLYATL1 | | | | | | |
| 11 | 58701115 | 58825925 | LOC283194 | | | | | | |
| 11 | 58710721 | 58724547 | GLYATL1 | | | | | | |
| 11 | 58874657 | 58894888 | FAM111B | | | | | | |
| 11 | 58901445 | 58910281 | LOC101927204 | | | | | | |
| 11 | 58910218 | 58922511 | FAM111A | | | | | | |
| 11 | 58938902 | 58976060 | DTX4 | | | | | | |
| 11 | 58975982 | 58980494 | MPEG1 | | | | | | |
| 11 | 59131931 | 59132867 | OR5AN1 | | | | | | |
| 11 | 59189451 | 59190426 | OR5A2 | | | | | | |
| 11 | 59210641 | 59211589 | OR5A1 | | | OR5A1 | | | |
| 11 | 59224433 | 59225378 | OR4D6 | | | | | | |
| 11 | 59244902 | 59245838 | OR4D10 | | | | | | |
| 11 | 59271048 | 59271984 | OR4D11 | | | | | | |
| 11 | 59282385 | 59283330 | OR4D9 | | | | | | |
| 11 | 59341870 | 59383617 | OSBP | | | | | | |
| 11 | 59362549 | 59362631 | MIR3162 | | | | | | |
| 11 | 59404191 | 59436511 | PATL1 | | | | | | |
| 11 | 59480388 | 59481318 | OR10V1 | | | | | | |
| 11 | 59516284 | 59517054 | OR10V2P | | | | | | |
| 11 | 59522531 | 59573355 | STX3 | | | STX3 | STX3 | | |
| 11 | 59573607 | 59578345 | MRPL16 | | | | | | |
| 11 | 59596745 | 59612974 | GIF | | | | | | |
| 11 | 59620280 | 59634041 | TCN1 | | | | | | |
| 11 | 59710351 | 59763318 | OOSP1 | | | | | | |
| 11 | 59807747 | 59815516 | OOSP2 | | | | | | |
| 11 | 59824100 | 59838588 | MS4A3 | | | | | | |
| 11 | 59856136 | 59865940 | MS4A2 | | | | | | |
| 11 | 59939079 | 59952139 | MS4A6A | | | | | | |
| 11 | 60048013 | 60076445 | MS4A4A | | | | | | |
| 11 | 60073143 | 60073172 | MIR6503 | | | | | | |
| 11 | 60102354 | 60108441 | MS4A6E | | | | | | |
| 11 | 60145957 | 60163426 | MS4A7 | | | | | | |
| 11 | 60163486 | 60185228 | MS4A14 | | | | | | |
| 11 | 60197061 | 60215265 | MS4A5 | | | | | | |
| 11 | 60223281 | 60238225 | MS4A1 | | | | | | |
| 11 | 60260250 | 60274901 | MS4A12 | | | | | | |
| 11 | 60282885 | 60310191 | MS4A13 | | | | | | |
| 11 | 60383223 | 60454621 | LINC00301 | | | | | | |
| 11 | 60467046 | 60483285 | MS4A8 | | | | | | |
| 11 | 60491481 | 60511686 | MS4A18 | | | | | | |
| 11 | 60524339 | 60544204 | MS4A15 | | | | | | |
| 11 | 60552820 | 60568778 | MS4A10 | | | | | | |
| 11 | 60609428 | 60618561 | CCDC86 | | | | | | |
| 11 | 60618397 | 60623444 | PTGDR2 | | PTGDR2 | | | | |
| 11 | 60635014 | 60643164 | ZP1 | | | | | | |
| 11 | 60658019 | 60674061 | PRPF19 | | | | | | |
| 11 | 60681370 | 60690915 | TMEM109 | | | | | | |
| 11 | 60691912 | 60704631 | TMEM132A | | | | | | |
| 11 | 60704554 | 60719257 | SLC15A3 | | | | | | |

| | | | | | | | | |
|---|---|---|---|---|---|---|---|---|
| 11 | 60739112 | 60787848 | CD6 | | | CD6 | | |
| 11 | 60869929 | 60895323 | CD5 | | | | | |
| 11 | 60897727 | 60928916 | VPS37C | | | | | |
| 11 | 60970984 | 60980341 | PGA3 | | | | | |
| 11 | 60989820 | 60999167 | PGA4 | | | | | |
| 11 | 61008647 | 61018915 | PGA5 | | | | | |
| 11 | 61025757 | 61062788 | VWCE | | | | | |
| 11 | 61066918 | 61100684 | DDB1 | | | | | |
| 11 | 61100653 | 61116231 | TKFC | | | | | |
| 11 | 61116219 | 61129755 | CYB561A3 | | | | | |
| 11 | 61129472 | 61136975 | TMEM138 | | | | | |
| 11 | 61159831 | 61166335 | TMEM216 | | | | | |
| 11 | 61170119 | 61197464 | CPSF7 | | | | | |
| 11 | 61197596 | 61214239 | SDHAF2 | | | | | |
| 11 | 61248584 | 61258400 | PPP1R32 | | | | | |
| 11 | 61276067 | 61276129 | MIR4488 | | | | | |
| 11 | 61276271 | 61278490 | LRRC10B | | | | | |
| 11 | 61281187 | 61348344 | SYT7 | | | SYT7 | | |
| 11 | 61355965 | 61375018 | LOC101927495 | | | | | |
| 11 | 61382507 | 61406921 | RPLP0P2 | | | | | |
| 11 | 61447904 | 61514474 | DAGLA | | | | | |
| 11 | 61520108 | 61555990 | MYRF | | MYRF | | | |
| 11 | 61521500 | 61525136 | DKFZP434K028 | | | | | |
| 11 | 61522860 | 61555990 | MYRF | | MYRF | | | |
| 11 | 61556601 | 61560085 | TMEM258 | | | | | |
| 11 | 61559966 | 61560033 | MIR611 | | | | | |
| 11 | 61560108 | 61564714 | FEN1 | | | | | |
| 11 | 61567096 | 61584529 | FADS1 | | | | | |
| 11 | 61582632 | 61582712 | MIR1908 | | | | | |
| 11 | 61583674 | 61634826 | FADS2 | | | | | |
| 11 | 61640994 | 61659017 | FADS3 | | | | | |
| 11 | 61645687 | 61645750 | MIR6746 | | | | | |
| 11 | 61664767 | 61687741 | RAB3IL1 | | | | | |
| 11 | 61717355 | 61731935 | BEST1 | | BEST1 | | | |
| 11 | 61731756 | 61735132 | FTH1 | | | | | |
| 11 | 61891444 | 61920635 | INCENP | | | | | |
| 11 | 61957709 | 61961009 | SCGB1D1 | | | | | |
| 11 | 61976139 | 61981411 | SCGB2A1 | | | | | |
| 11 | 62009723 | 62012280 | SCGB1D2 | | | | | |
| 11 | 62037626 | 62040629 | SCGB2A2 | | | | | |
| 11 | 62063753 | 62066536 | SCGB1D4 | | | | | |
| 11 | 62104773 | 62160887 | ASRGL1 | | | | | |
| 11 | 62186506 | 62190678 | SCGB1A1 | | | | | |
| 11 | 62201013 | 62314332 | AHNAK | | | | | |
| 11 | 62327072 | 62341460 | EEF1G | | | | | |
| 11 | 62327634 | 62327778 | MIR3654 | | | | | |
| 11 | 62334482 | 62334543 | MIR6747 | | | | | |
| 11 | 62342516 | 62359109 | TUT1 | | | | | |
| 11 | 62360674 | 62369312 | MTA2 | | | MTA2 | | |
| 11 | 62369690 | 62380237 | EML3 | | | | | |
| 11 | 62380212 | 62382592 | ROM1 | | ROM1 | ROM1 | | |
| 11 | 62382767 | 62389647 | B3GAT3 | | | | | |
| 11 | 62392297 | 62414198 | GANAB | | | | | |
| 11 | 62414319 | 62420774 | INTS5 | | | | | |
| 11 | 62430288 | 62432688 | C11orf98 | | | | | |
| 11 | 62430288 | 62439241 | LBHD1 | | | | | |
| 11 | 62432778 | 62434923 | METTL12 | | | | | |
| 11 | 62432893 | 62433042 | SNORA57 | | | | | |
| 11 | 62439125 | 62441162 | UQCC3 | | | | | |
| 11 | 62443969 | 62446589 | UBXN1 | | | | | |
| 11 | 62453873 | 62457371 | LRRN4CL | | | | | |
| 11 | 62457733 | 62477091 | BSCL2 | | BSCL2 | | | |
| 11 | 62457733 | 62494856 | HNRNPUL2-BSCL2 | | | | | |
| 11 | 62475065 | 62476678 | GNG3 | | | | | |
| 11 | 62480096 | 62494857 | HNRNPUL2 | | | | | |
| 11 | 62495544 | 62506108 | TTC9C | | | | | |

| | | | | | | | | | |
|---|---|---|---|---|---|---|---|---|---|
| 11 | 62518434 | 62521656 | ZBTB3 | | | | | | |
| 11 | 62529010 | 62534187 | POLR2G | | | | | | |
| 11 | 62538774 | 62554813 | TAF6L | | | | | | |
| 11 | 62554873 | 62557872 | TMEM179B | | | | | | |
| 11 | 62557286 | 62557357 | MIR6748 | | | | | | |
| 11 | 62557786 | 62559486 | TMEM223 | | | | | | |
| 11 | 62559597 | 62572964 | NXF1 | | | | | | |
| 11 | 62560173 | 62560243 | MIR6514 | | | | | | |
| 11 | 62574331 | 62599563 | STX5 | | | | | | |
| 11 | 62599705 | 62601513 | LOC105369332 | | | | | | |
| 11 | 62600376 | 62607628 | WDR74 | | | | | | |
| 11 | 62619459 | 62623360 | SNHG1 | | | | | | |
| 11 | 62620381 | 62620507 | SNORD22 | | | | | | |
| 11 | 62620797 | 62620865 | SNORD31 | | | | | | |
| 11 | 62621134 | 62621204 | SNORD30 | | | | | | |
| 11 | 62621375 | 62621440 | SNORD29 | | | | | | |
| 11 | 62622092 | 62622167 | SNORD28 | | | | | | |
| 11 | 62622483 | 62622555 | SNORD27 | | | | | | |
| 11 | 62622763 | 62622838 | SNORD26 | | | | | | |
| 11 | 62623036 | 62623103 | SNORD25 | | | | | | |
| 11 | 62623483 | 62656355 | SLC3A2 | | | | | | |
| 11 | 62676150 | 62689012 | CHRM1 | | | | | | |
| 11 | 62744068 | 62752495 | SLC22A6 | | | | | | |
| 11 | 62760295 | 62783317 | SLC22A8 | | | | | | |
| 11 | 62847411 | 62911693 | SLC22A24 | | | | | | |
| 11 | 62931295 | 62997124 | SLC22A25 | | | | | | |
| 11 | 63057418 | 63079246 | SLC22A10 | | | | | | |
| 11 | 63137260 | 63177712 | SLC22A9 | | | | | | |
| 11 | 63228875 | 63258680 | HRASLS5 | | | | | | |
| 11 | 63273523 | 63284246 | LGALS12 | | | | | | |
| 11 | 63304272 | 63313930 | RARRES3 | | | | | | |
| 11 | 63320241 | 63330855 | HRASLS2 | | | | | | |
| 11 | 63341943 | 63381941 | PLA2G16 | | | | | | |
| 11 | 63391553 | 63439446 | ATL3 | | | | | | |
| 11 | 63448921 | 63527363 | RTN3 | | | | | | |
| 11 | 63527363 | 63536113 | C11orf95 | | | | | | |
| 11 | 63580845 | 63595190 | C11orf84 | | | | | | |
| 11 | 63606399 | 63678492 | MARK2 | | | MARK2 | | | |
| 11 | 63678692 | 63684316 | RCOR2 | | | | | | |
| 11 | 63706441 | 63724791 | NAA40 | | | | | | |
| 11 | 63742078 | 63744015 | COX8A | | | COX8A | | | |
| 11 | 63753324 | 63765892 | OTUB1 | | | | | | |
| 11 | 63766029 | 63933585 | MACROD1 | | | | | | |
| 11 | 63871361 | 63886645 | FLRT1 | | | | | | |
| 11 | 63952743 | 63972020 | STIP1 | | | | | | |
| 11 | 63974151 | 63991363 | FERMT3 | | | | | | |
| 11 | 63991270 | 63993726 | TRPT1 | | | | | | |
| 11 | 63993729 | 63997488 | NUDT22 | | | | | | |
| 11 | 63997752 | 64001753 | DNAJC4 | | | | | | |
| 11 | 64002055 | 64006736 | VEGFB | | | VEGFB | | | |
| 11 | 64008412 | 64011607 | FKBP2 | | | | | | |
| 11 | 64011950 | 64014413 | PPP1R14B | | | | | | |
| 11 | 64013337 | 64015686 | LOC105369340 | | | | | | |
| 11 | 64018994 | 64036924 | PLCB3 | | | | | | |
| 11 | 64037299 | 64052176 | BAD | | | | | | |
| 11 | 64051810 | 64056972 | GPR137 | | | | | | |
| 11 | 64058773 | 64067503 | KCNK4 | | | | | | |
| 11 | 64059193 | 64072241 | KCNK4-TEX40 | | | | | | |
| 11 | 64059203 | 64067503 | KCNK4 | | | | | | |
| 11 | 64067862 | 64072239 | TEX40 | | | | | | |
| 11 | 64072999 | 64084212 | ESRRA | | | | | | |
| 11 | 64083963 | 64085556 | TRMT112 | | | | | | |
| 11 | 64085559 | 64089295 | PRDX5 | | | | | | |
| 11 | 64107689 | 64125006 | CCDC88B | | | | | | |
| 11 | 64109320 | 64109376 | MIR7155 | | | | | | |
| 11 | 64126624 | 64139687 | RPS6KA4 | | | | | | |

| | | | | | | | | |
|---|---|---|---|---|---|---|---|---|
| 11 | 64136073 | 64136175 | MIR1237 | | | | | |
| 11 | 64216528 | 64219128 | LOC100996455 | | | | | |
| 11 | 64323072 | 64340347 | SLC22A11 | | | | | |
| 11 | 64358281 | 64369825 | SLC22A12 | | | | | |
| 11 | 64373645 | 64490660 | NRXN2 | | NRXN2 | NRXN2 | NRXN2 | NRXN2 |
| 11 | 64494382 | 64512928 | RASGRP2 | | | | RASGRP2 | |
| 11 | 64513860 | 64528187 | PYGM | | | | | |
| 11 | 64532075 | 64546316 | SF1 | | | SF1 | | |
| 11 | 64556572 | 64570726 | MAP4K2 | | | | | |
| 11 | 64570985 | 64578766 | MEN1 | | | | | |
| 11 | 64591661 | 64612041 | CDC42BPG | | | | | |
| 11 | 64620198 | 64647185 | EHD1 | | | | | |
| 11 | 64655929 | 64660921 | MIR194 | | | | | |
| 11 | 64658608 | 64658718 | MIR192 | | | | | |
| 11 | 64658826 | 64658911 | MIR194 | | | | | |
| 11 | 64662003 | 64684722 | ATG2A | | | | | |
| 11 | 64665834 | 64665909 | MIR6750 | | | | | |
| 11 | 64669858 | 64669927 | MIR6749 | | | | | |
| 11 | 64692142 | 64701950 | PPP2R5B | | PPP2R5B | | | |
| 11 | 64701942 | 64703360 | GPHA2 | | | | | |
| 11 | 64705701 | 64739563 | C11orf85 | | | | | |
| 11 | 64755416 | 64764517 | BATF2 | | | | | |
| 11 | 64781584 | 64789657 | ARL2 | | | | | |
| 11 | 64781584 | 64808044 | ARL2-SNX15 | | | | | |
| 11 | 64785976 | 64786042 | MIR6879 | | | | | |
| 11 | 64794879 | 64808044 | SNX15 | | | SNX15 | | |
| 11 | 64808375 | 64812300 | SAC3D1 | | | | | |
| 11 | 64812294 | 64826009 | NAALADL1 | | | | | |
| 11 | 64844926 | 64851615 | CDCA5 | | | | | |
| 11 | 64851693 | 64855874 | ZFPL1 | | | | | |
| 11 | 64855843 | 64856581 | TMEM262 | | | | | |
| 11 | 64863586 | 64879332 | VPS51 | | | | | |
| 11 | 64879325 | 64883707 | TM7SF2 | | | | | |
| 11 | 64883874 | 64885210 | ZNHIT2 | | | | | |
| 11 | 64888098 | 64889672 | FAU | | | | | |
| 11 | 64889654 | 64894841 | MRPL49 | | | | | |
| 11 | 64894750 | 64902003 | SYVN1 | | | | | |
| 11 | 64897387 | 64897450 | MIR6751 | | | | | |
| 11 | 64937706 | 64940688 | SPDYC | | | | | |
| 11 | 64948685 | 64979477 | CAPN1 | | | | | |
| 11 | 64981310 | 65010228 | SLC22A20 | | | | | |
| 11 | 65029322 | 65066156 | POLA2 | | | | | |
| 11 | 65082288 | 65089900 | CDC42EP2 | | | | | |
| 11 | 65101224 | 65120451 | DPF2 | | | | | |
| 11 | 65122281 | 65125082 | TIGD3 | | | | | |
| 11 | 65142662 | 65151172 | SLC25A45 | | | | | |
| 11 | 65154040 | 65180995 | FRMD8 | | | | | |
| 11 | 65190268 | 65213009 | NEAT1 | | | | | |
| 11 | 65211928 | 65212028 | MIR612 | | | | | |
| 11 | 65265232 | 65273983 | MALAT1 | | | | | |
| 11 | 65292547 | 65306182 | SCYL1 | | | | | |
| 11 | 65306029 | 65325699 | LTBP3 | | | | | |
| 11 | 65336690 | 65339359 | SSSCA1 | | | | | |
| 11 | 65339819 | 65341669 | FAM89B | | | | | |
| 11 | 65343508 | 65360116 | EHBP1L1 | | | | | |
| 11 | 65360325 | 65363467 | KCNK7 | | | | | |
| 11 | 65365225 | 65381720 | MAP3K11 | | | MAP3K11 | | |
| 11 | 65383782 | 65404910 | PCNX3 | | | | | |
| 11 | 65403780 | 65403840 | MIR4690 | | | | | |
| 11 | 65405577 | 65418391 | SIPA1 | | | | | |
| 11 | 65416662 | 65416724 | MIR4489 | | | | | |
| 11 | 65421066 | 65430443 | RELA | | | | | |
| 11 | 65479472 | 65487077 | KAT5 | | | | | |
| 11 | 65485143 | 65488409 | RNASEH2C | | | | | |
| 11 | 65541368 | 65548062 | AP5B1 | | | | | |
| 11 | 65554464 | 65554484 | MIR1234 | | | | | |

| | | | | | | | | |
|---|---|---|---|---|---|---|---|---|
| 11 | 65554504 | 65558339 | OVOL1 | | | | | |
| 11 | 65601409 | 65621172 | SNX32 | | | | | |
| 11 | 65622284 | 65625804 | CFL1 | | | | | |
| 11 | 65627871 | 65633914 | MUS81 | | | | | |
| 11 | 65633911 | 65640405 | EFEMP2 | | | | | |
| 11 | 65647283 | 65651212 | CTSW | | | | | |
| 11 | 65651210 | 65656010 | FIBP | | | FIBP | | |
| 11 | 65657874 | 65659106 | CCDC85B | | | | | |
| 11 | 65659606 | 65667997 | FOSL1 | | | | | |
| 11 | 65684282 | 65686531 | C11orf68 | | | | | |
| 11 | 65686727 | 65689048 | DRAP1 | | | | | |
| 11 | 65713114 | 65727434 | TSGA10IP | | | | | |
| 11 | 65729159 | 65747607 | SART1 | | | | | |
| 11 | 65764015 | 65769637 | EIF1AD | | | | | |
| 11 | 65769549 | 65771617 | BANF1 | | | | | |
| 11 | 65779461 | 65780976 | CST6 | | | | | |
| 11 | 65784222 | 65793988 | CATSPER1 | | | | | |
| 11 | 65808235 | 65816651 | GAL3ST3 | | | | | |
| 11 | 65819815 | 65836382 | SF3B2 | | | | | |
| 11 | 65837823 | 66012218 | PACS1 | | | PACS1 | | |
| 11 | 66024764 | 66035332 | KLC2 | | KLC2 | | | |
| 11 | 66036055 | 66044963 | RAB1B | | | | | |
| 11 | 66045671 | 66051685 | CNIH2 | | | | | |
| 11 | 66052050 | 66056638 | YIF1A | | | | | |
| 11 | 66059372 | 66064135 | TMEM151A | | | | | |
| 11 | 66081957 | 66084515 | CD248 | | | | | |
| 11 | 66099541 | 66104000 | RIN1 | | | RIN1 | | |
| 11 | 66104803 | 66112582 | BRMS1 | | | | | |
| 11 | 66112842 | 66115161 | B4GAT1 | | B4GAT1 | | | |
| 11 | 66129991 | 66139947 | SLC29A2 | | | | | |
| 11 | 66188474 | 66194177 | NPAS4 | | | | | |
| 11 | 66202545 | 66206319 | MRPL11 | | | | | |
| 11 | 66233797 | 66244808 | PELI3 | | PELI3 | | | |
| 11 | 66240960 | 66247720 | LOC101928069 | | | | | |
| 11 | 66247483 | 66277130 | DPP3 | | | | | |
| 11 | 66278118 | 66301084 | BBS1 | | | | | |
| 11 | 66306734 | 66313671 | ZDHHC24 | | | | | |
| 11 | 66313865 | 66330800 | ACTN3 | | | | | |
| 11 | 66330934 | 66336047 | CTSF | | | | | |
| 11 | 66357639 | 66360554 | CCDC87 | | | | | |
| 11 | 66360689 | 66373490 | CCS | | | | | |
| 11 | 66384052 | 66397397 | RBM14 | | | RBM14 | | |
| 11 | 66384052 | 66413944 | RBM14-RBM4 | | | | | |
| 11 | 66406087 | 66435858 | RBM4 | | | | | |
| 11 | 66432464 | 66445392 | RBM4B | | | | | |
| 11 | 66452719 | 66488870 | SPTBN2 | | SPTBN2 | SPTBN2 | | |
| 11 | 66512206 | 66610987 | C11orf80 | | | | | |
| 11 | 66610882 | 66614003 | RCE1 | | | | | |
| 11 | 66615996 | 66725847 | PC | | PC | | | |
| 11 | 66624875 | 66627946 | LRFN4 | | | | | |
| 11 | 66701904 | 66701977 | MIR3163 | | | | | |
| 11 | 66742753 | 66744479 | C11orf86 | | | | | |
| 11 | 66790189 | 66818334 | SYT12 | | | SYT12 | | |
| 11 | 66813113 | 66813179 | MIR6860 | | | | | |
| 11 | 66824288 | 66839488 | RHOD | | | | | |
| 11 | 66886739 | 67025550 | KDM2A | | | | | |
| 11 | 67033904 | 67054029 | ADRBK1 | | | | | |
| 11 | 67056761 | 67069955 | ANKRD13D | | | | | |
| 11 | 67070918 | 67080078 | SSH3 | | | | | |
| 11 | 67085309 | 67159158 | LOC100130987 | | | | | |
| 11 | 67118235 | 67121067 | POLD4 | | | | | |
| 11 | 67131638 | 67141648 | CLCF1 | | | | | |
| 11 | 67159422 | 67165883 | RAD9A | | | | | |
| 11 | 67165651 | 67169376 | PPP1CA | | | PPP1CA | | |
| 11 | 67171383 | 67177561 | TBC1D10C | | | | | |
| 11 | 67183148 | 67193078 | CARNS1 | | | | | |

| | | | | | | | | |
|---|---|---|---|---|---|---|---|---|
| 11 | 67195934 | 67202879 | RPS6KB2 | | | | | |
| 11 | 67202980 | 67205153 | PTPRCAP | | | | | |
| 11 | 67205517 | 67211292 | CORO1B | | | | | |
| 11 | 67218771 | 67220200 | GPR152 | | | | | |
| 11 | 67219885 | 67229246 | CABP4 | | | CABP4 | | |
| 11 | 67231818 | 67236748 | TMEM134 | | | | | |
| 11 | 67250499 | 67258579 | AIP | | | | | |
| 11 | 67257715 | 67257786 | MIR6752 | | | | | |
| 11 | 67259238 | 67272843 | PITPNM1 | | | | | |
| 11 | 67273960 | 67276199 | CDK2AP2 | | | | | |
| 11 | 67286417 | 67290899 | CABP2 | | | | | |
| 11 | 67351065 | 67354124 | GSTP1 | | | | | |
| 11 | 67370350 | 67374177 | C11orf72 | | | | | |
| 11 | 67374322 | 67380012 | NDUFV1 | | | | | |
| 11 | 67380994 | 67383135 | DOC2GP | | | | | |
| 11 | 67395408 | 67397408 | NUDT8 | | | | | |
| 11 | 67398773 | 67407031 | TBX10 | | | | | |
| 11 | 67410025 | 67418130 | ACY3 | | | | | |
| 11 | 67429632 | 67448685 | ALDH3B2 | | | | | |
| 11 | 67559237 | 67572807 | FAM86C2P | | | | | |
| 11 | 67758570 | 67771595 | UNC93B1 | | | | | |
| 11 | 67776016 | 67796749 | ALDH3B1 | | | | | |
| 11 | 67798083 | 67804114 | NDUFS8 | | | NDUFS8 | | |
| 11 | 67800330 | 67800389 | MIR7113 | | | | | |
| 11 | 67801363 | 67801448 | MIR4691 | | | | | |
| 11 | 67806461 | 67818366 | TCIRG1 | | | | | |
| 11 | 67812260 | 67812424 | MIR6753 | | | | | |
| 11 | 67820325 | 67888858 | CHKA | | | | | |
| 11 | 67922329 | 67981239 | KMT5B | | | | | |
| 11 | 68028802 | 68039469 | C11orf24 | | | | | |
| 11 | 68080076 | 68216743 | LRP5 | | | LRP5 | | |
| 11 | 68228185 | 68382801 | PPP6R3 | | | | | |
| 11 | 68451942 | 68458643 | GAL | | | | | |
| 11 | 68474907 | 68518988 | MTL5 | | | | | |
| 11 | 68522087 | 68609399 | CPT1A | | | CPT1A | | |
| 11 | 68658745 | 68671303 | MRPL21 | | | | | |
| 11 | 68671318 | 68708069 | IGHMBP2 | | | IGHMBP2 | | |
| 11 | 68747489 | 68748455 | MRGPRD | | | | | |
| 11 | 68771861 | 68785916 | MRGPRF | | | | | |
| 11 | 68816349 | 68858072 | TPCN2 | | | | | |
| 11 | 68850643 | 68850726 | MIR3164 | | | | | |
| 11 | 68914695 | 68939030 | LOC338694 | | | | | |
| 11 | 69061604 | 69064754 | MYEOV | | | | | |
| 11 | 69240438 | 69244389 | LOC102724265 | | | | | |
| 11 | 69300328 | 69308311 | LINC01488 | | | | | |
| 11 | 69455872 | 69469242 | CCND1 | | | | | |
| 11 | 69480331 | 69490165 | ORAOV1 | | | | | |
| 11 | 69513005 | 69519106 | FGF19 | | | | | |
| 11 | 69587796 | 69590171 | FGF4 | | | | | |
| 11 | 69624735 | 69634192 | FGF3 | | | | | |
| 11 | 69902335 | 69911477 | LOC101928443 | | | | | |
| 11 | 69918539 | 70035652 | ANO1 | | | | | |
| 11 | 70049268 | 70053508 | FADD | | | | | |
| 11 | 70116805 | 70230607 | PPFIA1 | | | | | |
| 11 | 70130060 | 70130176 | MIR548K | | | | | |
| 11 | 70244611 | 70282690 | CTTN | | | | | |
| 11 | 70313960 | 70711220 | SHANK2 | | SHANK2 | SHANK2 | | SHANK2 |
| 11 | 70718374 | 70718473 | MIR3664 | | | | | |
| 11 | 71116791 | 71134400 | FLJ42102 | | | | | |
| 11 | 71145456 | 71159477 | DHCR7 | | | DHCR7 | | |
| 11 | 71164216 | 71212581 | NADSYN1 | | | | | |
| 11 | 71184548 | 71184614 | MIR6754 | | | | | |
| 11 | 71238312 | 71239210 | KRTAP5-7 | | | | | |
| 11 | 71249070 | 71250253 | KRTAP5-8 | | | | | |
| 11 | 71259465 | 71260653 | KRTAP5-9 | | | | | |
| 11 | 71276608 | 71277666 | KRTAP5-10 | | | KRTAP5-10 | | |

| | | | | | | | | |
|---|---|---|---|---|---|---|---|---|
| 11 | 71292900 | 71293921 | KRTAP5-11 | | | | | |
| 11 | 71498556 | 71512280 | FAM86C1 | | | | | |
| 11 | 71505408 | 71524905 | ALG1L9P | | | | | |
| 11 | 71525090 | 71532593 | ZNF705E | | | | | |
| 11 | 71544245 | 71548608 | DEFB108B | | | | | |
| 11 | 71576554 | 71639493 | LOC100133315 | | | | | |
| 11 | 71589498 | 71595607 | LOC100129216 | | | | | |
| 11 | 71639767 | 71708643 | RNF121 | | | | | |
| 11 | 71709957 | 71713574 | IL18BP | | | | | |
| 11 | 71713909 | 71791739 | NUMA1 | | | | | |
| 11 | 71725336 | 71731956 | LOC100128494 | | | | | |
| 11 | 71783273 | 71783348 | MIR3165 | | | | | |
| 11 | 71791376 | 71821828 | LRTOMT | | LRTOMT | | | |
| 11 | 71808337 | 71814433 | LAMTOR1 | | | | | |
| 11 | 71820632 | 71823822 | ANAPC15 | | | | | |
| 11 | 71846755 | 71850936 | FOLR3 | | | | | |
| 11 | 71900601 | 71907367 | FOLR1 | | | | | |
| 11 | 71927818 | 71932994 | FOLR2 | | | | | |
| 11 | 71935881 | 71950188 | INPPL1 | | | | | |
| 11 | 71950120 | 71955220 | PHOX2A | | | | | |
| 11 | 72003469 | 72145728 | CLPB | | | | | |
| 11 | 72281699 | 72284266 | LINC01537 | | | | | |
| 11 | 72287183 | 72385497 | PDE2A | | | | | |
| 11 | 72326106 | 72326174 | MIR139 | | | | | |
| 11 | 72396113 | 72463434 | ARAP1 | | | | | |
| 11 | 72465773 | 72504750 | STARD10 | | | | | |
| 11 | 72494574 | 72494637 | MIR4692 | | | | | |
| 11 | 72525450 | 72540680 | ATG16L2 | | | | | |
| 11 | 72547789 | 72853143 | FCHSD2 | | | | | |
| 11 | 72590964 | 72604767 | MIR4459 | | | | | |
| 11 | 72929342 | 72953472 | P2RY2 | | | | | |
| 11 | 72975549 | 73009670 | P2RY6 | | | | | |
| 11 | 73019662 | 73080425 | ARHGEF17 | | | | | |
| 11 | 73087404 | 73108519 | RELT | | | | | |
| 11 | 73111522 | 73309234 | FAM168A | | | | | |
| 11 | 73357222 | 73373864 | PLEKHB1 | | | | | |
| 11 | 73386682 | 73472201 | RAB6A | | RAB6A | RAB6A | | |
| 11 | 73498916 | 73575656 | MRPL48 | | | | | |
| 11 | 73583712 | 73587890 | COA4 | | | | | |
| 11 | 73587743 | 73638781 | PAAF1 | | | | | |
| 11 | 73661363 | 73681332 | DNAJB13 | | | | | |
| 11 | 73685715 | 73693889 | UCP2 | | | | | |
| 11 | 73711325 | 73720282 | UCP3 | | | | | |
| 11 | 73723758 | 73882064 | C2CD3 | | | | | |
| 11 | 73882107 | 73965748 | PPME1 | | | | | |
| 11 | 73977693 | 74022721 | P4HA3 | | | | | |
| 11 | 74022406 | 74035750 | LOC101928580 | | | | | |
| 11 | 74041356 | 74109510 | PGM2L1 | | | | | |
| 11 | 74165885 | 74178600 | KCNE3 | | | | | |
| 11 | 74202922 | 74204755 | LIPT2 | | | | | |
| 11 | 74204429 | 74209578 | LOC100287896 | | | | | |
| 11 | 74303574 | 74354105 | POLD3 | | | | | |
| 11 | 74407472 | 74442430 | CHRDL2 | | | | | |
| 11 | 74431312 | 74431382 | MIR4696 | | | | | |
| 11 | 74459912 | 74553458 | RNF169 | | | | | |
| 11 | 74551954 | 74660232 | XRRA1 | | | | | |
| 11 | 74660291 | 74690076 | SPCS2 | | | | | |
| 11 | 74699949 | 74718743 | NEU3 | | | | | |
| 11 | 74799795 | 74800758 | OR2AT4 | | | | | |
| 11 | 74862031 | 74917445 | SLCO2B1 | | SLCO2B1 | | | |
| 11 | 74951949 | 74954749 | TPBGL | | | | | |
| 11 | 74971165 | 75062875 | ARRB1 | | | | | ARRB1 |
| 11 | 75046135 | 75046230 | MIR326 | | | | | |
| 11 | 75110534 | 75133345 | RPS3 | | | | | |
| 11 | 75111434 | 75111582 | SNORD15A | | | | | |
| 11 | 75115464 | 75115610 | SNORD15B | | | | | |

| | | | | | | | | |
|---|---|---|---|---|---|---|---|---|
| 11 | 75133437 | 75141674 | KLHL35 | | | | | |
| 11 | 75145684 | 75236599 | GDPD5 | | | | | |
| 11 | 75273100 | 75283849 | SERPINH1 | | | | | |
| 11 | 75297962 | 75379479 | MAP6 | | | | | |
| 11 | 75428863 | 75444003 | MOGAT2 | | | | | |
| 11 | 75469499 | 75479692 | LOC283214 | | | | | |
| 11 | 75479777 | 75512581 | DGAT2 | | | | | |
| 11 | 75526211 | 75855282 | UVRAG | | | | | |
| 11 | 75897369 | 75917574 | WNT11 | | | | | |
| 11 | 76061000 | 76092009 | PRKRIR | | | | | |
| 11 | 76092356 | 76125665 | LOC100506127 | | | | | |
| 11 | 76156068 | 76263943 | EMSY | | | | | |
| 11 | 76368567 | 76381791 | LRRC32 | | | | | |
| 11 | 76391209 | 76432833 | GUCY2EP | | | | | |
| 11 | 76493356 | 76509198 | TSKU | | | | | |
| 11 | 76493624 | 76494106 | LOC101928837 | | | | | |
| 11 | 76494284 | 76509198 | TSKU | | | | | |
| 11 | 76571916 | 76737841 | ACER3 | | | | | |
| 11 | 76745384 | 76753005 | B3GNT6 | | | | | |
| 11 | 76777991 | 76837198 | CAPN5 | | | CAPN5 | | |
| 11 | 76813885 | 76814377 | OMP | | | | | |
| 11 | 76839309 | 76926286 | MYO7A | | | | | MYO7A |
| 11 | 76927602 | 76998463 | GDPD4 | | | | | |
| 11 | 77033059 | 77185108 | PAK1 | | | | | |
| 11 | 77282133 | 77286707 | LOC646029 | | | | | |
| 11 | 77300679 | 77321401 | AQP11 | | | | | |
| 11 | 77327195 | 77348851 | CLNS1A | | | CLNS1A | | |
| 11 | 77377273 | 77531880 | RSF1 | | | | | |
| 11 | 77532159 | 77611831 | AAMDC | | | | | |
| 11 | 77589765 | 77705717 | INTS4 | | | | | |
| 11 | 77726760 | 77757237 | KCTD14 | | | | | |
| 11 | 77726760 | 77791265 | NDUFC2-KCTD14 | | | | | |
| 11 | 77774906 | 77779403 | THRSP | | | | | |
| 11 | 77779392 | 77791265 | NDUFC2 | | | NDUFC2 | | |
| 11 | 77811987 | 77850699 | ALG8 | | | | | |
| 11 | 77850838 | 77899675 | KCTD21 | | | | | |
| 11 | 77899857 | 77925757 | USP35 | | | | | |
| 11 | 77926335 | 78128868 | GAB2 | | GAB2 | | | |
| 11 | 78135028 | 78140882 | LOC101928865 | | | | | |
| 11 | 78147006 | 78285909 | NARS2 | | | | | |
| 11 | 78244221 | 78269611 | LOC101928896 | | | | | |
| 11 | 78364327 | 79151695 | TENM4 | | | | | |
| 11 | 79113065 | 79113153 | MIR708 | | | | | |
| 11 | 79133212 | 79133270 | MIR5579 | | | | | |
| 11 | 80462243 | 80473846 | LOC101928944 | | | | | |
| 11 | 81590892 | 82114955 | MIR4300HG | | | | | |
| 11 | 81601782 | 81601878 | MIR4300 | | | | | |
| 11 | 82443045 | 82444906 | FAM181B | | | | | |
| 11 | 82533982 | 82612782 | PRCP | | | | | |
| 11 | 82612736 | 82645699 | DDIAS | | | | | |
| 11 | 82684174 | 82782965 | RAB30 | | | RAB30 | | |
| 11 | 82752505 | 82752640 | SNORA70E | | | | | |
| 11 | 82783107 | 82784754 | RAB30 | | | RAB30 | | |
| 11 | 82868136 | 82898526 | PCF11 | | | | | |
| 11 | 82904780 | 82967141 | ANKRD42 | | | | | |
| 11 | 82970134 | 82997450 | CCDC90B | | | | | |
| 11 | 83166055 | 85338314 | DLG2 | | DLG2 | DLG2 | DLG2 | DLG2 |
| 11 | 85339616 | 85347583 | TMEM126B | | | | | |
| 11 | 85358962 | 85367597 | TMEM126A | | | | | |
| 11 | 85368607 | 85376182 | CREBZF | | | | | |
| 11 | 85394886 | 85397320 | CCDC89 | | | | | |
| 11 | 85405264 | 85522202 | SYTL2 | | | SYTL2 | | |
| 11 | 85566143 | 85631063 | CCDC83 | | | | | |
| 11 | 85668213 | 85780923 | PICALM | | | PICALM | | |
| 11 | 85955425 | 85989852 | EED | | | | | |
| 11 | 85989374 | 85989440 | MIR6755 | | | | | |

| | | | | | | | | |
|---|---|---|---|---|---|---|---|---|
| 11 | 86013252 | 86056985 | C11orf73 | | | | | |
| 11 | 86085777 | 86134151 | CCDC81 | | | | | |
| 11 | 86152149 | 86383678 | ME3 | | | | | |
| 11 | 86502100 | 86663945 | PRSS23 | | | | | |
| 11 | 86567972 | 86568996 | OR7E2P | | | | | |
| 11 | 86656716 | 86666440 | FZD4 | | | | | |
| 11 | 86666662 | 86711989 | LOC100506368 | | | | | |
| 11 | 86748885 | 87039876 | TMEM135 | | | | | |
| 11 | 87429245 | 87430303 | LOC105369423 | | | | | |
| 11 | 87846414 | 87908635 | RAB38 | | | RAB38 | | |
| 11 | 87909669 | 87909761 | MIR3166 | | | | | |
| 11 | 88026759 | 88070955 | CTSC | | | | | |
| 11 | 88237743 | 88796846 | GRM5 | | | GRM5 | | |
| 11 | 88911039 | 89028927 | TYR | | | | | |
| 11 | 89057521 | 89224733 | NOX4 | | | | | |
| 11 | 89392464 | 89431886 | FOLH1B | | | | | |
| 11 | 89443466 | 89451040 | TRIM77 | | | | | |
| 11 | 89530822 | 89541743 | TRIM49 | | | | | |
| 11 | 89575164 | 89584136 | TRIM53AP | | | | | |
| 11 | 89603605 | 89609185 | TRIM64B | | | | | |
| 11 | 89644578 | 89653576 | TRIM49D2 | | | | | |
| 11 | 89644639 | 89650878 | TRIM49D1 | | | | | |
| 11 | 89657231 | 89666229 | TRIM49D2 | | | | | |
| 11 | 89659929 | 89666168 | TRIM49D1 | | | | | |
| 11 | 89701671 | 89707240 | TRIM64 | | | | | |
| 11 | 89726708 | 89735676 | TRIM53AP | | | | | |
| 11 | 89764273 | 89775193 | TRIM49C | | | | | |
| 11 | 89819117 | 89820299 | UBTFL1 | | | | | |
| 11 | 89867817 | 89925779 | NAALAD2 | | | NAALAD2 | | |
| 11 | 89933596 | 89956532 | CHORDC1 | | | | | |
| 11 | 89984399 | 90648220 | DISC1FP1 | | | | | |
| 11 | 90288941 | 90289025 | MIR4490 | | | | | |
| 11 | 90602288 | 90602370 | MIR1261 | | | | | |
| 11 | 92085261 | 92629635 | FAT3 | | | | | |
| 11 | 92646404 | 92648794 | LOC105369431 | | | | | |
| 11 | 92702788 | 92715948 | MTNR1B | | | MTNR1B | MTNR1B | |
| 11 | 92877336 | 92931141 | SLC36A4 | | | | | |
| 11 | 93063882 | 93171636 | CCDC67 | | | | | |
| 11 | 93211638 | 93276546 | SMCO4 | | | | | |
| 11 | 93394815 | 93463522 | CEP295 | | | | | |
| 11 | 93454679 | 93455032 | SCARNA9 | | | | | |
| 11 | 93463680 | 93463812 | SNORA25 | | | | | |
| 11 | 93464144 | 93464265 | SNORA32 | | | | | |
| 11 | 93464668 | 93464739 | SNORD6 | | | | | |
| 11 | 93465169 | 93465299 | SNORA1 | | | | | |
| 11 | 93465526 | 93465665 | SNORA8 | | | | | |
| 11 | 93466393 | 93466466 | SNORD5 | | | | | |
| 11 | 93466631 | 93466763 | SNORA18 | | | | | |
| 11 | 93466839 | 93466930 | MIR1304 | | | | | |
| 11 | 93468275 | 93468402 | SNORA40 | | | | | |
| 11 | 93469095 | 93474703 | TAF1D | | | | | |
| 11 | 93474759 | 93496268 | C11orf54 | | | | | |
| 11 | 93517404 | 93546496 | MED17 | | | | | |
| 11 | 93553734 | 93583668 | VSTM5 | | | | | |
| 11 | 93754377 | 93847374 | HEPHL1 | | | | | |
| 11 | 93862093 | 93915137 | PANX1 | | | | | |
| 11 | 94038802 | 94040858 | IZUMO1R | | | | | |
| 11 | 94110476 | 94134585 | GPR83 | | | GPR83 | | |
| 11 | 94150468 | 94227040 | MRE11A | | | MRE11A | | |
| 11 | 94199660 | 94199746 | MIR548L | | | | | |
| 11 | 94227152 | 94232744 | ANKRD49 | | | | | |
| 11 | 94245694 | 94265289 | C11orf97 | | | | | |
| 11 | 94277016 | 94283064 | FUT4 | | | | | |
| 11 | 94278497 | 94473521 | LOC105369438 | | | | | |
| 11 | 94300473 | 94354587 | PIWIL4 | | | | | |
| 11 | 94306937 | 94473521 | LOC105369438 | | | | | |

| | | | | | | | | |
|---|---|---|---|---|---|---|---|---|
| 11 | 94501507 | 94609918 | AMOTL1 | | | | | |
| 11 | 94695786 | 94706776 | CWC15 | | | | | |
| 11 | 94706844 | 94732676 | KDM4D | | | | | |
| 11 | 94758421 | 94760760 | KDM4E | | | | | |
| 11 | 94800040 | 94804387 | SRSF8 | | | | | |
| 11 | 94822973 | 94865815 | ENDOD1 | | | | | |
| 11 | 94883702 | 94892312 | LOC101929295 | | | | | |
| 11 | 94898676 | 94965705 | SESN3 | | | | | |
| 11 | 94963349 | 94967568 | LOC100129203 | | | | | |
| 11 | 95502105 | 95522954 | FAM76B | | | | | |
| 11 | 95523624 | 95565857 | CEP57 | | | CEP57 | | |
| 11 | 95566043 | 95657371 | MTMR2 | | | | | |
| 11 | 95709756 | 96076344 | MAML2 | | | | | |
| 11 | 96074601 | 96074690 | MIR1260B | | | | | |
| 11 | 96085928 | 96123083 | CCDC82 | | | | | |
| 11 | 96123157 | 96240041 | JRKL | | | | | |
| 11 | 96241588 | 96247914 | LOC105369443 | | | | | |
| 11 | 98891705 | 100229616 | CNTN5 | | CNTN5 | CNTN5 | | |
| 11 | 100554891 | 100558686 | LOC100128386 | | | | | |
| 11 | 100558406 | 100861656 | ARHGAP42 | | | | | |
| 11 | 100862810 | 100864666 | TMEM133 | | | | | |
| 11 | 100900354 | 101000544 | PGR | | | | PGR | |
| 11 | 100999807 | 101030001 | LOC101054525 | | | | | |
| 11 | 101322294 | 101454659 | TRPC6 | | | | | |
| 11 | 101390550 | 101390636 | MIR3920 | | | | | |
| 11 | 101761404 | 101787253 | ANGPTL5 | | | | | |
| 11 | 101785745 | 101871796 | CEP126 | | | | | |
| 11 | 101918168 | 101955291 | C11orf70 | | | | | |
| 11 | 101981191 | 102104154 | YAP1 | | | | | |
| 11 | 102188180 | 102210135 | BIRC3 | | | | | |
| 11 | 102217912 | 102249401 | BIRC2 | | | | | |
| 11 | 102267055 | 102323775 | TMEM123 | | | | | |
| 11 | 102323649 | 102332769 | LOC101928424 | | | | | |
| 11 | 102337985 | 102369530 | LOC102723838 | | | | | |
| 11 | 102391238 | 102401484 | MMP7 | | | | | |
| 11 | 102447565 | 102496063 | MMP20 | | | | | |
| 11 | 102562414 | 102576468 | MMP27 | | | | | |
| 11 | 102582525 | 102595685 | MMP8 | | | | | |
| 11 | 102641232 | 102651359 | MMP10 | | | | | |
| 11 | 102654406 | 102707497 | WTAPP1 | | | | | |
| 11 | 102660640 | 102668966 | MMP1 | | | | | |
| 11 | 102706527 | 102714420 | MMP3 | | | | | |
| 11 | 102733459 | 102745764 | MMP12 | | | | | |
| 11 | 102813720 | 102826463 | MMP13 | | | | | |
| 11 | 102921412 | 102962944 | DCUN1D5 | | | | | |
| 11 | 102980159 | 103350591 | DYNC2H1 | | | | | |
| 11 | 103720633 | 103720708 | MIR4693 | | | | | |
| 11 | 103777913 | 104035027 | PDGFD | | | | | |
| 11 | 103907307 | 103909922 | DDI1 | | | | | |
| 11 | 104123318 | 104123379 | MIR7641 | | | | | |
| 11 | 104439216 | 104480030 | LOC102723895 | | | | | |
| 11 | 104756444 | 104769397 | CASP12 | | | | | |
| 11 | 104772275 | 104788902 | LOC643733 | | | | | |
| 11 | 104813593 | 104839325 | CASP4 | | | | | |
| 11 | 104864966 | 104893895 | CASP5 | | | | | |
| 11 | 104896236 | 104905884 | CASP1 | | | CASP1 | | |
| 11 | 104912052 | 104916051 | CARD16 | | | | | |
| 11 | 104933328 | 104942257 | CASP1P2 | | | | | |
| 11 | 104963195 | 104972158 | CARD17 | | | | | |
| 11 | 105008447 | 105010461 | CARD18 | | | | | |
| 11 | 105480799 | 105852819 | GRIA4 | | | | | |
| 11 | 105878647 | 105893014 | MSANTD4 | | | | | |
| 11 | 105921824 | 105948465 | KBTBD3 | | | | | |
| 11 | 105948291 | 105969419 | AASDHPPT | | | | | |
| 11 | 105983185 | 106002843 | LOC105369473 | | | | | |
| 11 | 106120745 | 106135632 | LOC101928535 | | | | | |

| | | | | | | | | |
|---|---|---|---|---|---|---|---|---|
| 11 | 106544737 | 106889171 | GUCY1A2 | | | | | |
| 11 | 107197071 | 107328572 | CWF19L2 | | | | | |
| 11 | 107373452 | 107436461 | ALKBH8 | | | | | |
| 11 | 107461816 | 107537505 | ELMOD1 | | | | | |
| 11 | 107462470 | 107463949 | LOC643923 | | | | | |
| 11 | 107578100 | 107582787 | SLN | | | | | |
| 11 | 107661716 | 107729914 | SLC35F2 | | | | | |
| 11 | 107799200 | 107834208 | RAB39A | | | | | |
| 11 | 107879407 | 107978488 | CUL5 | | | | | |
| 11 | 107992257 | 108018891 | ACAT1 | | | ACAT1 | | |
| 11 | 108028118 | 108093365 | NPAT | | | | | |
| 11 | 108093558 | 108239826 | ATM | ATM | | | | |
| 11 | 108253726 | 108338258 | C11orf65 | | | | | |
| 11 | 108342832 | 108369159 | KDELC2 | | | | | |
| 11 | 108376157 | 108464465 | EXPH5 | | | EXPH5 | | |
| 11 | 108535751 | 108811657 | DDX10 | | | | | |
| 11 | 109292845 | 109299893 | C11orf87 | | | | | |
| 11 | 109964086 | 110042566 | ZC3H12C | | | | | |
| 11 | 110045604 | 110167437 | RDX | | | RDX | | |
| 11 | 110225838 | 110276993 | LOC105369486 | | | | | |
| 11 | 110300660 | 110335608 | FDX1 | | | FDX1 | | |
| 11 | 110447758 | 110583912 | ARHGAP20 | | | | | |
| 11 | 111126706 | 111157129 | C11orf53 | | | | | |
| 11 | 111164113 | 111175773 | COLCA1 | | | | | |
| 11 | 111169270 | 111179460 | COLCA2 | | | | | |
| 11 | 111218481 | 111218547 | MIR4491 | | | | | |
| 11 | 111222980 | 111250157 | POU2AF1 | | | | | |
| 11 | 111284966 | 111288911 | LOC100132078 | | | | | |
| 11 | 111338255 | 111383079 | BTG4 | | | | | |
| 11 | 111383662 | 111383746 | MIR34B | | | | | |
| 11 | 111384163 | 111384240 | MIR34C | | | | | |
| 11 | 111385509 | 111407756 | C11orf88 | | | | | |
| 11 | 111411004 | 111432470 | LAYN | | | | | |
| 11 | 111473114 | 111597641 | SIK2 | | | | | |
| 11 | 111597631 | 111637169 | PPP2R1B | | | PPP2R1B | | |
| 11 | 111652918 | 111742305 | ALG9 | | | | | |
| 11 | 111744779 | 111750181 | FDXACB1 | | | | | |
| 11 | 111749947 | 111754797 | C11orf1 | | | | | |
| 11 | 111779343 | 111783937 | CRYAB | | CRYAB | | | |
| 11 | 111783459 | 111784817 | HSPB2 | | | | | |
| 11 | 111783459 | 111797595 | HSPB2-C11orf52 | | | | | |
| 11 | 111789600 | 111797595 | C11orf52 | | | | | |
| 11 | 111797867 | 111893374 | DIXDC1 | | DIXDC1 | | | |
| 11 | 111895537 | 111935002 | DLAT | | | | | |
| 11 | 111934733 | 111944895 | PIH1D2 | | | | | |
| 11 | 111944967 | 111955874 | C11orf57 | | | | | |
| 11 | 111955538 | 111957522 | TIMM8B | | | | | |
| 11 | 111957547 | 111966525 | SDHD | | | SDHD | | |
| 11 | 112013973 | 112034840 | IL18 | IL18 | | | | |
| 11 | 112038094 | 112043279 | TEX12 | | | | | |
| 11 | 112046207 | 112089649 | BCO2 | | | | | |
| 11 | 112097087 | 112104695 | PTS | | | PTS | | |
| 11 | 112118875 | 112131583 | PLET1 | | | | | |
| 11 | 112130987 | 112132119 | LOC100132686 | | | | | |
| 11 | 112141471 | 112233257 | LOC283140 | | | | | |
| 11 | 112352954 | 112358559 | LOC101928823 | | | | | |
| 11 | 112404944 | 112426525 | LOC387810 | | | | | |
| 11 | 112830002 | 112834182 | LOC101928847 | | | | | |
| 11 | 112831968 | 113144623 | NCAM1 | | NCAM1 | | NCAM1 | |
| 11 | 113185250 | 113237114 | TTC12 | | | | | |
| 11 | 113258512 | 113271140 | ANKK1 | | | | | |
| 11 | 113280316 | 113346001 | DRD2 | DRD2 | DRD2 | DRD2 | DRD2 | |
| 11 | 113320744 | 113320810 | MIR4301 | | | | | |
| 11 | 113558267 | 113577095 | TMPRSS5 | | | TMPRSS5 | | |
| 11 | 113603904 | 113644485 | ZW10 | | | | | |
| 11 | 113650517 | 113651207 | CLDN25 | | | | | |

| | | | | | | | | |
|---|---|---|---|---|---|---|---|---|
| 11 | 113668596 | 113746292 | USP28 | | | | | |
| 11 | 113775517 | 113817283 | HTR3B | | | | HTR3B | HTR3B |
| 11 | 113845796 | 113861034 | HTR3A | | | | HTR3A | HTR3A |
| 11 | 113930430 | 114121397 | ZBTB16 | | | ZBTB16 | | |
| 11 | 114166534 | 114183238 | NNMT | | | | | |
| 11 | 114231356 | 114250761 | LOC101928940 | | | | | |
| 11 | 114262169 | 114271272 | C11orf71 | | | | | |
| 11 | 114271250 | 114281332 | RBM7 | | | | | |
| 11 | 114310107 | 114321000 | REXO2 | | | | | |
| 11 | 114392436 | 114430617 | NXPE1 | | | | | |
| 11 | 114441312 | 114466484 | NXPE4 | | | | | |
| 11 | 114549199 | 114577652 | NXPE2 | | | | | |
| 11 | 115044344 | 115375241 | CADM1 | | | | | |
| 11 | 115204295 | 115211158 | LOC105369509 | | | | | |
| 11 | 115267473 | 115268376 | LOC105369507 | | | | | |
| 11 | 115602662 | 115605480 | LOC101928985 | | | | | |
| 11 | 115626050 | 115630918 | LINC00900 | | | | | |
| 11 | 116510138 | 116528969 | LOC101929011 | | | | | |
| 11 | 116618885 | 116643714 | BUD13 | | | | | |
| 11 | 116648904 | 116658754 | ZPR1 | | | | | |
| 11 | 116660085 | 116663136 | APOA5 | | | | | |
| 11 | 116691417 | 116694011 | APOA4 | | | | | |
| 11 | 116700623 | 116703787 | APOC3 | | | | | |
| 11 | 116706466 | 116726445 | APOA1 | | | APOA1 | | |
| 11 | 116714117 | 116969131 | SIK3 | | | | | |
| 11 | 117014999 | 117048889 | PAFAH1B2 | | | PAFAH1B2 | | |
| 11 | 117049938 | 117068161 | SIDT2 | | | | | |
| 11 | 117066328 | 117072630 | LOC100652768 | | | | | |
| 11 | 117070039 | 117075508 | TAGLN | | | | | |
| 11 | 117075786 | 117103241 | PCSK7 | | | | | |
| 11 | 117103340 | 117156404 | RNF214 | | | | | |
| 11 | 117156401 | 117162886 | BACE1 | BACE1 | BACE1 | BACE1 | | |
| 11 | 117192493 | 117283982 | CEP164 | | | | | |
| 11 | 117298487 | 117667976 | DSCAML1 | | | DSCAML1 | | |
| 11 | 117690789 | 117698807 | FXYD2 | | | | | |
| 11 | 117690789 | 117747746 | FXYD6-FXYD2 | | | | | |
| 11 | 117707690 | 117748201 | FXYD6 | | FXYD6 | | | |
| 11 | 117771355 | 117800168 | TMPRSS13 | | | | | |
| 11 | 117857105 | 117872199 | IL10RA | | | | | |
| 11 | 117886486 | 117990556 | TMPRSS4 | | | TMPRSS4 | | |
| 11 | 118004091 | 118023630 | SCN4B | | SCN4B | SCN4B | | |
| 11 | 118033518 | 118047337 | SCN2B | | SCN2B | | | |
| 11 | 118064441 | 118095809 | JAML | | | | | |
| 11 | 118097404 | 118123083 | MPZL3 | | | | | |
| 11 | 118124130 | 118135251 | MPZL2 | | | | | |
| 11 | 118175294 | 118186890 | CD3E | | | | | |
| 11 | 118209788 | 118213459 | CD3D | | | | | |
| 11 | 118215058 | 118224497 | CD3G | | | | | |
| 11 | 118230295 | 118269926 | UBE4A | | | | | |
| 11 | 118251934 | 118289530 | LOC100131626 | | | | | |
| 11 | 118272103 | 118280562 | ATP5L | | | | | |
| 11 | 118307204 | 118397539 | KMT2A | | | | | |
| 11 | 118382632 | 118401809 | LOC101929089 | | | | | |
| 11 | 118398209 | 118401740 | TTC36 | | | | | |
| 11 | 118401802 | 118417313 | TMEM25 | | | | | |
| 11 | 118415242 | 118436791 | IFT46 | | | | | |
| 11 | 118443101 | 118473747 | ARCN1 | | | | | |
| 11 | 118477212 | 118528748 | PHLDB1 | | | | | |
| 11 | 118514717 | 118514797 | MIR6716 | | | | | |
| 11 | 118528941 | 118550381 | TREH | | | | | |
| 11 | 118618472 | 118661972 | DDX6 | | | | | |
| 11 | 118754474 | 118766980 | CXCR5 | | | | | |
| 11 | 118766850 | 118781613 | BCL9L | | | | | |
| 11 | 118781416 | 118781496 | MIR4492 | | | | | |
| 11 | 118827007 | 118829269 | UPK2 | | | | | |
| 11 | 118842416 | 118851995 | FOXR1 | | | | | |

| | | | | | | | | |
|---|---|---|---|---|---|---|---|---|
| 11 | 118868842 | 118886502 | CCDC84 | | | | | |
| 11 | 118873670 | 118874303 | RPL23AP64 | | | | | |
| 11 | 118886421 | 118889057 | RPS25 | | | | | |
| 11 | 118889178 | 118894384 | TRAPPC4 | | | TRAPPC4 | | |
| 11 | 118889653 | 118889722 | MIR3656 | | | | | |
| 11 | 118895060 | 118901616 | SLC37A4 | | | | | |
| 11 | 118914895 | 118927957 | HYOU1 | | | | | |
| 11 | 118938462 | 118952688 | VPS11 | | | | | |
| 11 | 118955586 | 118964259 | HMBS | | | | | |
| 11 | 118964584 | 118966177 | H2AFX | | | | | |
| 11 | 118967212 | 118972785 | DPAGT1 | | | | | |
| 11 | 118978059 | 118987834 | C2CD2L | | | | | |
| 11 | 118992232 | 119005765 | HINFP | | | | | |
| 11 | 119019749 | 119033374 | ABCG4 | | ABCG4 | | | |
| 11 | 119039042 | 119054726 | NLRX1 | | | | | |
| 11 | 119056165 | 119060932 | PDZD3 | | | | | |
| 11 | 119060962 | 119066584 | CCDC153 | | | | | |
| 11 | 119076985 | 119178859 | CBL | | | | | |
| 11 | 119179233 | 119187840 | MCAM | | | | | |
| 11 | 119183659 | 119183722 | MIR6756 | | | | | |
| 11 | 119205209 | 119208024 | RNF26 | | | | | |
| 11 | 119209643 | 119217383 | C1QTNF5 | | | | | |
| 11 | 119209643 | 119217383 | MFRP | | | | | |
| 11 | 119225924 | 119369944 | USP2 | | USP2 | | | |
| 11 | 119288650 | 119295695 | THY1 | | | THY1 | | |
| 11 | 119508807 | 119599435 | PVRL1 | | | | | |
| 11 | 119600292 | 119610333 | LOC102724301 | | | | | |
| 11 | 119981993 | 120008863 | TRIM29 | | | TRIM29 | | |
| 11 | 120081746 | 120100650 | OAF | | | | | |
| 11 | 120107348 | 120190653 | POU2F3 | | | POU2F3 | | |
| 11 | 120120467 | 120136641 | LOC649133 | | | | | |
| 11 | 120195837 | 120204388 | TMEM136 | | | | | |
| 11 | 120207617 | 120360645 | ARHGEF12 | | | | | |
| 11 | 120382454 | 120859513 | GRIK4 | | GRIK4 | GRIK4 | GRIK4 | GRIK4 |
| 11 | 120682053 | 120689824 | LOC105369532 | | | | | |
| 11 | 120738641 | 120765506 | LOC101929227 | | | | | |
| 11 | 120811263 | 120828748 | LOC101929208 | | | | | |
| 11 | 120894802 | 120960354 | TBCEL | | | | | |
| 11 | 120973374 | 121061515 | TECTA | | TECTA | | | |
| 11 | 121163387 | 121184119 | SC5D | | SC5D | | | |
| 11 | 121322911 | 121504471 | SORL1 | | SORL1 | | | |
| 11 | 121959810 | 122073770 | MIR100HG | | | | | |
| 11 | 121970464 | 121970552 | MIR125B1 | | | | | |
| 11 | 121986061 | 121986923 | BLID | | | | | |
| 11 | 122017229 | 122017301 | MIRLET7A2 | | | | | |
| 11 | 122022936 | 122023016 | MIR100 | | | | | |
| 11 | 122526397 | 122685187 | UBASH3B | | | | | |
| 11 | 122709205 | 122743347 | CRTAM | | | | | |
| 11 | 122753235 | 122830430 | C11orf63 | | | | | |
| 11 | 122848356 | 122852379 | BSX | | | | | |
| 11 | 122888273 | 122890319 | LOC341056 | | | | | |
| 11 | 122928199 | 122933043 | HSPA8 | | | | | |
| 11 | 122942713 | 123066013 | CLMP | | | | | |
| 11 | 123252147 | 123252220 | MIR4493 | | | | | |
| 11 | 123396343 | 123498479 | GRAMD1B | | | | | |
| 11 | 123499894 | 123525315 | SCN3B | | SCN3B | SCN3B | SCN3B | |
| 11 | 123594634 | 123612391 | ZNF202 | | | | | |
| 11 | 123624287 | 123625226 | OR6X1 | | | | | |
| 11 | 123676115 | 123677057 | OR6M1 | | | | | |
| 11 | 123753632 | 123756349 | TMEM225 | | | | | |
| 11 | 123777138 | 123778083 | OR8D4 | | | | | |
| 11 | 123810323 | 123811280 | OR4D5 | | | | | |
| 11 | 123813573 | 123814545 | OR6T1 | | | | | |
| 11 | 123847402 | 123848398 | OR10S1 | | | | | |
| 11 | 123886281 | 123887217 | OR10G4 | | | | | |
| 11 | 123893719 | 123894655 | OR10G9 | | | | | |

| | | | | | | | | |
|---|---|---|---|---|---|---|---|---|
| 11 | 123900329 | 123901265 | OR10G8 | | | OR10G8 | | |
| 11 | 123908772 | 123909708 | OR10G7 | | | | | |
| 11 | 123986110 | 124017618 | VWA5A | | | | | |
| 11 | 124095343 | 124096368 | OR8G2 | | | | | |
| 11 | 124120422 | 124135756 | OR8G1 | | | | | |
| 11 | 124134722 | 124135763 | OR8G5 | | | | | |
| 11 | 124179735 | 124180662 | OR8D1 | | | | | |
| 11 | 124189157 | 124190093 | OR8D2 | | | | | |
| 11 | 124252297 | 124253239 | OR8B2 | | | | | |
| 11 | 124266305 | 124267247 | OR8B3 | | | | | |
| 11 | 124293837 | 124294767 | OR8B4 | | | | | |
| 11 | 124310045 | 124310981 | OR8B8 | | | | | |
| 11 | 124412617 | 124413550 | OR8B12 | | | | | |
| 11 | 124439964 | 124440945 | OR8A1 | | | | | |
| 11 | 124481452 | 124490251 | PANX3 | | | | | |
| 11 | 124492741 | 124505822 | TBRG1 | | | | | |
| 11 | 124505684 | 124546199 | SIAE | | SIAE | | | |
| 11 | 124506393 | 124506443 | MIR7641 | | | | | |
| 11 | 124543739 | 124564687 | SPA17 | | | | | |
| 11 | 124609828 | 124617102 | NRGN | | | NRGN | | |
| 11 | 124617369 | 124622109 | VSIG2 | | | | | |
| 11 | 124623018 | 124632223 | ESAM | | | | | |
| 11 | 124629029 | 124635816 | LOC101929340 | | | | | |
| 11 | 124636393 | 124670300 | MSANTD2 | | | | | |
| 11 | 124670323 | 124704383 | LOC100507283 | | | | | |
| 11 | 124735304 | 124751370 | ROBO3 | | ROBO3 | | | |
| 11 | 124754113 | 124767831 | ROBO4 | | | | | |
| 11 | 124789145 | 124790573 | HEPN1 | | | | | |
| 11 | 124789145 | 124806308 | HEPACAM | | HEPACAM | | | |
| 11 | 124824016 | 124911385 | CCDC15 | | | | | |
| 11 | 124933012 | 124960412 | SLC37A2 | | | | | |
| 11 | 124964265 | 124981659 | TMEM218 | | | | | |
| 11 | 125028356 | 125303285 | PKNOX2 | | | | | |
| 11 | 125315640 | 125366206 | FEZ1 | | | FEZ1 | | |
| 11 | 125365109 | 125369422 | LOC403312 | | | | | |
| 11 | 125439282 | 125454584 | EI24 | | | | | |
| 11 | 125440185 | 125492654 | STT3A | | | | | |
| 11 | 125495030 | 125527042 | CHEK1 | | | | | |
| 11 | 125542228 | 125550793 | ACRV1 | | | | | |
| 11 | 125616187 | 125619743 | PATE1 | | | | | |
| 11 | 125646027 | 125648714 | PATE2 | | | | | |
| 11 | 125658005 | 125661495 | PATE3 | | | | | |
| 11 | 125703210 | 125709967 | PATE4 | | | | | |
| 11 | 125753508 | 125770541 | HYLS1 | | | | | |
| 11 | 125763379 | 125773116 | PUS3 | | | | | |
| 11 | 125774260 | 125793006 | DDX25 | | | | | |
| 11 | 125826712 | 125933187 | CDON | | | | | |
| 11 | 126071988 | 126081587 | RPUSD4 | | | | | |
| 11 | 126081618 | 126132879 | FAM118B | | | | | |
| 11 | 126132813 | 126138877 | SRPRA | | | | | |
| 11 | 126138934 | 126148027 | FOXRED1 | | | | | |
| 11 | 126152981 | 126164828 | TIRAP | | | | | |
| 11 | 126173646 | 126215648 | DCPS | | | DCPS | | |
| 11 | 126211611 | 126284536 | ST3GAL4 | | | | | |
| 11 | 126293395 | 126870766 | KIRREL3 | | KIRREL3 | | | |
| 11 | 126522672 | 126551999 | LOC101929427 | | | | | |
| 11 | 126810641 | 126814986 | KIRREL3 | | KIRREL3 | | | |
| 11 | 126858353 | 126858438 | MIR3167 | | | | | |
| 11 | 126872804 | 126875953 | KIRREL3 | | KIRREL3 | | | |
| 11 | 126936896 | 126971201 | LOC101929473 | | | | | |
| 11 | 127140964 | 127206928 | LOC101929497 | | | | | |
| 11 | 128328655 | 128457453 | ETS1 | | | | | |
| 11 | 128392284 | 128392344 | MIR6090 | | | | | |
| 11 | 128396036 | 128400278 | LOC101929517 | | | | | |
| 11 | 128551134 | 128556817 | LOC101929538 | | | | | |
| 11 | 128556429 | 128683162 | FLI1 | | | | | |

| | | | | | | | | | |
|---|---|---|---|---|---|---|---|---|---|
| 11 | 128561566 | 128565918 | SENCR | | | | | | |
| 11 | 128563810 | 128683162 | FLI1 | | | | | | |
| 11 | 128707908 | 128737268 | KCNJ1 | | | KCNJ1 | | | |
| 11 | 128761312 | 128787951 | KCNJ5 | KCNJ5 | | KCNJ5 | | | |
| 11 | 128769459 | 128776126 | C11orf45 | | | | | | |
| 11 | 128804626 | 128813294 | TP53AIP1 | | | | | | |
| 11 | 128834954 | 129062093 | ARHGAP32 | | | | | | |
| 11 | 129245880 | 129322174 | BARX2 | | | BARX2 | | | |
| 11 | 129482015 | 129487164 | LINC01395 | | | | | | |
| 11 | 129685740 | 129729898 | TMEM45B | | | | | | |
| 11 | 129733669 | 129765490 | NFRKB | | | | | | |
| 11 | 129769600 | 129872730 | PRDM10 | | | | | | |
| 11 | 129872518 | 129875381 | LINC00167 | | | | | | |
| 11 | 129939715 | 130014706 | APLP2 | | | | | | |
| 11 | 130029681 | 130080257 | ST14 | | | | | | |
| 11 | 130096573 | 130184607 | ZBTB44 | | | | | | |
| 11 | 130274817 | 130298539 | ADAMTS8 | | | | | | |
| 11 | 130318868 | 130346539 | ADAMTS15 | | | | | | |
| 11 | 130536629 | 130536698 | MIR8052 | | | | | | |
| 11 | 130542850 | 130587247 | C11orf44 | | | | | | |
| 11 | 130714087 | 130732005 | LOC100507431 | | | | | | |
| 11 | 130736143 | 130740142 | LOC103611081 | | | | | | |
| 11 | 130745765 | 130786382 | SNX19 | | | SNX19 | | | |
| 11 | 131240370 | 132206716 | NTM | | | | | | |
| 11 | 131403750 | 131410761 | LOC101929653 | | | | | | |
| 11 | 131532022 | 132206716 | NTM | | | | | | |
| 11 | 132154195 | 132158353 | NTM-IT | | | | | | |
| 11 | 132284558 | 133402507 | OPCML | | OPCML | | | | |
| 11 | 133653565 | 133680271 | LOC646522 | | | | | | |
| 11 | 133710516 | 133715433 | SPATA19 | | | | | | |
| 11 | 133766329 | 133771635 | MIR4697HG | | | | | | |
| 11 | 133768398 | 133768476 | MIR4697 | | | | | | |
| 11 | 133778519 | 133826649 | IGSF9B | | | | | | |
| 11 | 133902166 | 133911236 | LOC100128239 | | | | | | |
| 11 | 133938819 | 134021652 | JAM3 | | JAM3 | | | | |
| 11 | 134022336 | 134094426 | NCAPD3 | | | | | | |
| 11 | 134094498 | 134117686 | VPS26B | | | | | | |
| 11 | 134118172 | 134123260 | THYN1 | | | | | | |
| 11 | 134123433 | 134135746 | ACAD8 | | | ACAD8 | | | |
| 11 | 134146274 | 134189458 | GLB1L3 | | | | | | |
| 11 | 134201767 | 134246218 | GLB1L2 | | | | | | |
| 11 | 134248397 | 134281812 | B3GAT1 | | | | | | |
| 11 | 134306375 | 134375555 | LOC283177 | | | | | | |
| 11 | 134855245 | 134856693 | LOC100507548 | | | | | | |
| 12 | 67606 | 69079 | FAM138D | | | | | | |
| 12 | 73724 | 91263 | LOC100288778 | | | | | | |
| 12 | 176048 | 280494 | IQSEC3 | | | | | | |
| 12 | 246576 | 258332 | LOC574538 | | | | | | |
| 12 | 299242 | 323371 | SLC6A12 | | | SLC6A12 | | | |
| 12 | 312810 | 314259 | LOC101929384 | | | | | | |
| 12 | 329786 | 372039 | SLC6A13 | | | | | | |
| 12 | 362607 | 366465 | LOC102723544 | | | | | | |
| 12 | 368154 | 372039 | SLC6A13 | | | | | | |
| 12 | 389222 | 498621 | KDM5A | | | | | | |
| 12 | 498515 | 551806 | CCDC77 | | | | | | |
| 12 | 569542 | 671058 | B4GALNT3 | | | | | | |
| 12 | 673461 | 772907 | NINJ2 | | | | | | |
| 12 | 694864 | 695652 | LOC105369595 | | | | | | |
| 12 | 740056 | 755044 | LOC100049716 | | | | | | |
| 12 | 862088 | 1020618 | WNK1 | | WNK1 | WNK1 | | | |
| 12 | 1020901 | 1042242 | RAD52 | | | | | | |
| 12 | 1100373 | 1605099 | ERC1 | | | ERC1 | | | |
| 12 | 1609656 | 1613590 | LINC00942 | | | | | | |
| 12 | 1675158 | 1703331 | FBXL14 | | | | | | |
| 12 | 1726221 | 1756378 | WNT5B | | | | | | |
| 12 | 1769480 | 1769546 | MIR3649 | | | | | | |

| | | | | | | | | |
|---|---|---|---|---|---|---|---|---|
| 12 | 1800246 | 1897845 | ADIPOR2 | | | | | |
| 12 | 1901122 | 2027870 | CACNA2D4 | | | | CACNA2D4 | |
| 12 | 1929432 | 1945918 | LRTM2 | | | | | |
| 12 | 2038367 | 2045742 | LINC00940 | | | | | |
| 12 | 2050756 | 2113701 | DCP1B | | | | | |
| 12 | 2157517 | 2158628 | CACNA1C-IT2 | | | | | |
| 12 | 2162415 | 2332647 | CACNA1C | CACNA1C | | CACNA1C | CACNA1C | |
| 12 | 2378941 | 2397911 | CACNA1C-IT3 | | | | | |
| 12 | 2777665 | 2800323 | CACNA1C | CACNA1C | | CACNA1C | CACNA1C | |
| 12 | 2870365 | 2880835 | LOC283440 | | | | | |
| 12 | 2904107 | 2914587 | FKBP4 | | | | | |
| 12 | 2921786 | 2939770 | ITFG2 | | | | | |
| 12 | 2934513 | 2944221 | NRIP2 | | | | | |
| 12 | 2945981 | 2968961 | LOC100507424 | | | | | |
| 12 | 2966846 | 2986321 | FOXM1 | | | | | |
| 12 | 2985423 | 2998691 | RHNO1 | | | | | |
| 12 | 3000032 | 3050306 | TULP3 | | | | | |
| 12 | 3068477 | 3149842 | TEAD4 | | | | | |
| 12 | 3186520 | 3395730 | TSPAN9 | | | | | |
| 12 | 3490514 | 3703138 | PRMT8 | | | | | |
| 12 | 3592877 | 3601985 | THCAT155 | | | | | |
| 12 | 3600363 | 3703138 | PRMT8 | | | | | |
| 12 | 3724493 | 3862366 | CRACR2A | | | | | |
| 12 | 3918026 | 3982614 | PARP11 | | | | | |
| 12 | 4357932 | 4414522 | CCND2 | | | | | |
| 12 | 4430358 | 4469190 | TIGAR | | | | | |
| 12 | 4477392 | 4488894 | FGF23 | | | FGF23 | FGF23 | |
| 12 | 4543307 | 4554780 | FGF6 | | | | | |
| 12 | 4596895 | 4647674 | C12orf4 | | | | | |
| 12 | 4647949 | 4669213 | RAD51AP1 | | | | | |
| 12 | 4699237 | 4723054 | DYRK4 | | | | | |
| 12 | 4724673 | 4758213 | AKAP3 | | | | | |
| 12 | 4758263 | 4796720 | NDUFA9 | | | | | |
| 12 | 4809582 | 4829268 | LOC101929549 | | | | | |
| 12 | 4829751 | 4881892 | GALNT8 | | | | | |
| 12 | 4918341 | 4960278 | KCNA6 | | | KCNA6 | | |
| 12 | 5019072 | 5027422 | KCNA1 | | KCNA1 | KCNA1 | KCNA1 | |
| 12 | 5153084 | 5155954 | KCNA5 | | | | KCNA5 | |
| 12 | 5343161 | 5352317 | LOC101929584 | | | | | |
| 12 | 5541279 | 5604465 | NTF3 | NTF3 | | | | |
| 12 | 5671816 | 6055398 | ANO2 | | | | | |
| 12 | 6058039 | 6233836 | VWF | | | VWF | | |
| 12 | 6309481 | 6347437 | CD9 | | | | | |
| 12 | 6419601 | 6437672 | PLEKHG6 | | | | | |
| 12 | 6437922 | 6451283 | TNFRSF1A | | | | | |
| 12 | 6456008 | 6486523 | SCNN1A | | | SCNN1A | | |
| 12 | 6484533 | 6500737 | LTBR | | | | | |
| 12 | 6548166 | 6560884 | CD27 | | | CD27 | | |
| 12 | 6561176 | 6571488 | TAPBPL | | | | | |
| 12 | 6571403 | 6580065 | VAMP1 | | VAMP1 | | | VAMP1 |
| 12 | 6601315 | 6602471 | MRPL51 | | | | | |
| 12 | 6603297 | 6641132 | NCAPD2 | | | | | |
| 12 | 6619387 | 6619717 | SCARNA10 | | | | | |
| 12 | 6643570 | 6647541 | GAPDH | | | | | |
| 12 | 6648693 | 6665249 | IFFO1 | | | | | |
| 12 | 6666035 | 6677498 | NOP2 | | | | | |
| 12 | 6679247 | 6716599 | CHD4 | | | | | |
| 12 | 6690638 | 6690775 | SCARNA11 | | | | | |
| 12 | 6728000 | 6745297 | LPAR5 | | | | | |
| 12 | 6747241 | 6756580 | ACRBP | | | | | |
| 12 | 6759703 | 6772308 | ING4 | | | | | |
| 12 | 6775642 | 6798738 | ZNF384 | | | | | |
| 12 | 6802956 | 6810009 | PIANP | | | | | |
| 12 | 6833149 | 6841041 | COPS7A | | | | | |
| 12 | 6857157 | 6862636 | MLF2 | | | | | |
| 12 | 6875540 | 6880118 | PTMS | | | | | |

| | | | | | | | | |
|---|---|---|---|---|---|---|---|---|
| 12 | 6881669 | 6887621 | LAG3 | | | | | |
| 12 | 6898637 | 6929976 | CD4 | | | | | |
| 12 | 6930962 | 6936583 | GPR162 | | | | | |
| 12 | 6937537 | 6949018 | P3H3 | | | | | |
| 12 | 6950017 | 6956559 | GNB3 | | | | | |
| 12 | 6955888 | 6961230 | CDCA3 | | | | | |
| 12 | 6961284 | 6975795 | USP5 | | | | | |
| 12 | 6976583 | 6980110 | TPI1 | | | TPI1 | | |
| 12 | 6980099 | 6982521 | SPSB2 | | | | | |
| 12 | 6984928 | 6998522 | LOC105369632 | | | | | |
| 12 | 6993144 | 6993768 | RPL13P5 | | | | | |
| 12 | 6993845 | 6994950 | DSTNP2 | | | | | |
| 12 | 7013896 | 7023406 | LRRC23 | | | | | |
| 12 | 7023613 | 7032859 | ENO2 | | | | | |
| 12 | 7033625 | 7051484 | ATN1 | | | | | |
| 12 | 7052600 | 7055166 | C12orf57 | | | | | |
| 12 | 7055739 | 7070479 | PTPN6 | | | | | |
| 12 | 7072407 | 7073610 | LOC105369635 | | | | | |
| 12 | 7072861 | 7072929 | MIR200C | | | | | |
| 12 | 7073259 | 7073354 | MIR141 | | | | | |
| 12 | 7074514 | 7079916 | PHB2 | | | | | |
| 12 | 7076499 | 7076769 | SCARNA12 | | | | | |
| 12 | 7079943 | 7105520 | EMG1 | | | | | |
| 12 | 7085346 | 7125842 | LPCAT3 | | | | | |
| 12 | 7167979 | 7178335 | C1S | | | C1S | | |
| 12 | 7187514 | 7245043 | C1R | | | | | |
| 12 | 7247145 | 7274447 | C1RL | | | | | |
| 12 | 7276279 | 7281466 | RBP5 | | | | | |
| 12 | 7282966 | 7311530 | CLSTN3 | | | | | |
| 12 | 7341758 | 7364079 | PEX5 | | PEX5 | | | |
| 12 | 7456927 | 7480969 | ACSM4 | | | | | |
| 12 | 7507555 | 7596781 | CD163L1 | | | | | |
| 12 | 7623411 | 7656414 | CD163 | | | | | |
| 12 | 7801995 | 7823195 | APOBEC1 | | | | | |
| 12 | 7842380 | 7848360 | GDF3 | | | | | |
| 12 | 7864049 | 7870152 | DPPA3 | | | | | |
| 12 | 7882010 | 7902069 | CLEC4C | | | | | |
| 12 | 7917811 | 7926717 | NANOGNB | | | | | |
| 12 | 7941991 | 7948657 | NANOG | | | | | |
| 12 | 7965107 | 8043792 | SLC2A14 | | | | | |
| 12 | 8071823 | 8088892 | SLC2A3 | | | | | |
| 12 | 8185358 | 8208118 | FOXJ2 | | | | | |
| 12 | 8210918 | 8218955 | C3AR1 | | | | | |
| 12 | 8234806 | 8250373 | NECAP1 | | NECAP1 | NECAP1 | | |
| 12 | 8276227 | 8291203 | CLEC4A | | | | | |
| 12 | 8286364 | 8287448 | POU5F1P3 | | | | | |
| 12 | 8325149 | 8332642 | ZNF705A | | | | | |
| 12 | 8332804 | 8353596 | FAM66C | | | | | |
| 12 | 8373855 | 8380214 | FAM90A1 | | | | | |
| 12 | 8383644 | 8395542 | FAM86FP | | | | | |
| 12 | 8388010 | 8395160 | LOC101927905 | | | | | |
| 12 | 8509559 | 8543348 | LINC00937 | | | | | |
| 12 | 8608590 | 8630926 | CLEC6A | | | | | |
| 12 | 8666135 | 8674960 | CLEC4D | | | | | |
| 12 | 8685900 | 8693558 | CLEC4E | | | | | |
| 12 | 8754761 | 8765442 | AICDA | | | | | |
| 12 | 8798538 | 8815484 | MFAP5 | | | | | |
| 12 | 8834272 | 8929787 | RIMKLB | | | | | |
| 12 | 8975067 | 9029377 | A2ML1 | | | | | |
| 12 | 9067315 | 9094060 | PHC1 | | | | | |
| 12 | 9092956 | 9102357 | M6PR | | | M6PR | | |
| 12 | 9142220 | 9163340 | KLRG1 | | | | | |
| 12 | 9208184 | 9217666 | LINC00612 | | | | | |
| 12 | 9217772 | 9268558 | A2M | | | | | |
| 12 | 9301435 | 9360966 | PZP | | | | | |
| 12 | 9381128 | 9386803 | A2MP1 | | | | | |

| | | | | | | | | |
|---|---|---|---|---|---|---|---|---|
| 12 | 9392065 | 9392147 | MIR1244 | | | | | |
| 12 | 9392598 | 9395645 | LINC00987 | | | | | |
| 12 | 9436252 | 9466684 | LOC642846 | | | | | |
| 12 | 9520059 | 9550213 | LOC101930452 | | | | | |
| 12 | 9524377 | 9534018 | LOC101928030 | | | | | |
| 12 | 9570286 | 9600768 | DDX12P | | | | | |
| 12 | 9747869 | 9760497 | KLRB1 | | | | | |
| 12 | 9769879 | 9811010 | LOC374443 | | | | | |
| 12 | 9822303 | 9852151 | CLEC2D | | | | | |
| 12 | 9868455 | 9885895 | CLECL1 | | | | | |
| 12 | 9905081 | 9913497 | CD69 | | | | | |
| 12 | 9980076 | 9997603 | KLRF1 | | | KLRF1 | | |
| 12 | 10004967 | 10022458 | CLEC2B | | | | | |
| 12 | 10034087 | 10048432 | KLRF2 | | | | | |
| 12 | 10051271 | 10084980 | CLEC2A | | | | | |
| 12 | 10089177 | 10096094 | LOC100506159 | | | | | |
| 12 | 10100735 | 10105936 | LOC400002 | | | | | |
| 12 | 10103914 | 10138194 | CLEC12A | | | | | |
| 12 | 10145661 | 10151899 | CLEC1B | | | | | |
| 12 | 10163225 | 10171399 | CLEC12B | | | | | |
| 12 | 10167950 | 10183205 | LOC102724020 | | | | | |
| 12 | 10183275 | 10218629 | CLEC9A | | | | | |
| 12 | 10222152 | 10251664 | CLEC1A | | | | | |
| 12 | 10269375 | 10282868 | CLEC7A | | | | | |
| 12 | 10310898 | 10324790 | OLR1 | | OLR1 | | | |
| 12 | 10323197 | 10344403 | TMEM52B | | | | | |
| 12 | 10365488 | 10375724 | GABARAPL1 | | | GABARAPL1 | | |
| 12 | 10457049 | 10469850 | KLRD1 | | | | | |
| 12 | 10516367 | 10551105 | LOC101928100 | | | | | |
| 12 | 10524951 | 10542653 | KLRK1 | | | | | |
| 12 | 10524951 | 10562745 | KLRC4-KLRK1 | | | | | |
| 12 | 10559982 | 10562356 | KLRC4 | | | | | |
| 12 | 10564913 | 10573194 | KLRC3 | | KLRC3 | | | |
| 12 | 10583205 | 10588592 | KLRC2 | | | | | |
| 12 | 10594862 | 10607215 | KLRC1 | | | | | |
| 12 | 10741076 | 10752434 | KLRA1P | | | | | |
| 12 | 10756788 | 10766208 | MAGOHB | | | | | |
| 12 | 10771537 | 10826891 | STYK1 | | | | | |
| 12 | 10851675 | 10875953 | YBX3 | | | | | |
| 12 | 10902832 | 10930050 | LOC101928162 | | | | | |
| 12 | 10954130 | 10955226 | TAS2R7 | | | | | |
| 12 | 10958649 | 10959579 | TAS2R8 | | | | | |
| 12 | 10961692 | 10962767 | TAS2R9 | | | | | |
| 12 | 10977944 | 10978868 | TAS2R10 | | | | | |
| 12 | 10998447 | 11002075 | PRR4 | | | | | |
| 12 | 10998447 | 11324224 | PRH1-PRR4 | | | | | |
| 12 | 11033559 | 11324222 | PRH1 | | | | | |
| 12 | 11060524 | 11062161 | TAS2R13 | | | TAS2R13 | | |
| 12 | 11081834 | 11087444 | PRH2 | | | | | |
| 12 | 11090004 | 11324222 | PRH1-TAS2R14 | | | | | |
| 12 | 11090852 | 11091806 | TAS2R14 | | | | | |
| 12 | 11116402 | 11324222 | PRH1 | | | | | |
| 12 | 11138511 | 11139511 | TAS2R50 | | | | | |
| 12 | 11148560 | 11150474 | TAS2R20 | | | | | |
| 12 | 11174217 | 11175219 | TAS2R19 | | | | | |
| 12 | 11182985 | 11184006 | TAS2R31 | | | | | |
| 12 | 11213963 | 11214893 | TAS2R46 | | | | | |
| 12 | 11243885 | 11244912 | TAS2R43 | | | | | |
| 12 | 11285883 | 11286843 | TAS2R30 | | | | | |
| 12 | 11323779 | 11328619 | SMIM10L1 | | | | | |
| 12 | 11338598 | 11339543 | TAS2R42 | | | | | |
| 12 | 11418846 | 11422641 | PRB3 | | | | | |
| 12 | 11460014 | 11463369 | PRB4 | | | | | |
| 12 | 11504756 | 11508524 | PRB1 | | | | | |
| 12 | 11544473 | 11548498 | PRB2 | | | | | |
| 12 | 11700963 | 11717335 | LINC01252 | | | | | |

| | | | | | | | | |
|---|---|---|---|---|---|---|---|---|
| 12 | 11802787 | 12048325 | ETV6 | | | | | |
| 12 | 12223877 | 12252627 | BCL2L14 | | | | | |
| 12 | 12264885 | 12264967 | MIR1244 | | | | | |
| 12 | 12268960 | 12419811 | LRP6 | | | | | |
| 12 | 12482217 | 12503169 | MANSC1 | | | | | |
| 12 | 12508341 | 12510001 | LOH12CR2 | | | | | |
| 12 | 12510012 | 12619838 | BORCS5 | | | | | |
| 12 | 12626215 | 12715448 | DUSP16 | | | | | |
| 12 | 12764766 | 12798042 | CREBL2 | | | | | |
| 12 | 12813994 | 12849121 | GPR19 | | | | | |
| 12 | 12870203 | 12875316 | CDKN1B | | | | | |
| 12 | 12878850 | 12944399 | APOLD1 | | | | | |
| 12 | 12917582 | 12917677 | MIR613 | | | | | |
| 12 | 12938540 | 12944399 | APOLD1 | | | | | |
| 12 | 12966279 | 12982915 | DDX47 | | | | | |
| 12 | 13028410 | 13029070 | RPL13AP20 | | | | | |
| 12 | 13043955 | 13066600 | GPRC5A | | | | | |
| 12 | 13068762 | 13068852 | MIR614 | | | | | |
| 12 | 13093708 | 13103318 | GPRC5D | | | | | |
| 12 | 13127798 | 13153243 | HEBP1 | | | | | |
| 12 | 13132770 | 13137576 | LOC100506314 | | | | | |
| 12 | 13153375 | 13157764 | HTR7P1 | | | | | |
| 12 | 13197314 | 13236383 | FAM234B | | | | | |
| 12 | 13199124 | 13199174 | MIR7641 | | | | | |
| 12 | 13236470 | 13256630 | GSG1 | | | | | |
| 12 | 13349601 | 13369708 | EMP1 | | | | | |
| 12 | 13523604 | 13529679 | LINC01559 | | | | | |
| 12 | 13714409 | 14133022 | GRIN2B | GRIN2B | GRIN2B | GRIN2B | GRIN2B | GRIN2B |
| 12 | 14518565 | 14655869 | ATF7IP | | | | | |
| 12 | 14656596 | 14772689 | PLBD1 | | | | | |
| 12 | 14765567 | 14849519 | GUCY2C | | | | | |
| 12 | 14923653 | 14924065 | HIST4H4 | | | | | |
| 12 | 14927269 | 14930936 | H2AFJ | | | | | |
| 12 | 14939411 | 14956401 | WBP11 | | | WBP11 | | |
| 12 | 14956505 | 14976791 | C12orf60 | | | | | |
| 12 | 14957583 | 14967116 | SMCO3 | | | | | |
| 12 | 14982244 | 14996413 | ART4 | | | | | |
| 12 | 15034114 | 15038853 | MGP | | | | | |
| 12 | 15066960 | 15091483 | ERP27 | | | | | |
| 12 | 15094949 | 15114562 | ARHGDIB | | | | | |
| 12 | 15125955 | 15134799 | PDE6H | | PDE6H | | | |
| 12 | 15154766 | 15159617 | LINC01489 | | | | | |
| 12 | 15260715 | 15308215 | RERG | | | | | |
| 12 | 15475190 | 15751265 | PTPRO | | PTPRO | | | |
| 12 | 15773074 | 15942510 | EPS8 | | | | | |
| 12 | 16035287 | 16056410 | STRAP | | | | | |
| 12 | 16064105 | 16190315 | DERA | | | | | |
| 12 | 16341418 | 16430619 | SLC15A5 | | | | | |
| 12 | 16500075 | 16517344 | MGST1 | | | MGST1 | | |
| 12 | 16701305 | 16761148 | LMO3 | | LMO3 | | | |
| 12 | 17141680 | 17143562 | SKP1P2 | | | | | |
| 12 | 17826232 | 17826328 | MIR3974 | | | | | |
| 12 | 18233802 | 18243127 | RERGL | | | | | |
| 12 | 18414473 | 18801352 | PIK3C2G | | | | | |
| 12 | 18836109 | 18890993 | PLCZ1 | | | | | |
| 12 | 18891044 | 18892122 | CAPZA3 | | | | | |
| 12 | 19282625 | 19529333 | PLEKHA5 | | | | | |
| 12 | 19592607 | 19675173 | AEBP2 | | | | | |
| 12 | 20167618 | 20251802 | LOC100506393 | | | | | |
| 12 | 20522178 | 20837041 | PDE3A | | | | | |
| 12 | 20848288 | 20906320 | SLCO1C1 | | | | | |
| 12 | 20963637 | 21069843 | SLCO1B3 | | | | | |
| 12 | 21168629 | 21243040 | SLCO1B7 | | | | | |
| 12 | 21284127 | 21392730 | SLCO1B1 | | | | | |
| 12 | 21417533 | 21548371 | SLCO1A2 | | | | | |
| 12 | 21525801 | 21532914 | IAPP | | | IAPP | | |

| | | | | | | | | |
|---|---|---|---|---|---|---|---|---|
| 12 | 21590537 | 21624182 | PYROXD1 | | | | | |
| 12 | 21621843 | 21654603 | RECQL | | | | | |
| 12 | 21654698 | 21671337 | GOLT1B | | | | | |
| 12 | 21679255 | 21684210 | SPX | | | | | |
| 12 | 21689122 | 21757781 | GYS2 | | | | | |
| 12 | 21788275 | 21810789 | LDHB | | LDHB | | | |
| 12 | 21917888 | 21927755 | KCNJ8 | | | | | |
| 12 | 21950323 | 22089628 | ABCC9 | | | | | |
| 12 | 22199107 | 22218606 | CMAS | | | | | |
| 12 | 22346324 | 22487648 | ST8SIA1 | | | | | |
| 12 | 22601479 | 22697480 | C2CD5 | | | | | |
| 12 | 22741940 | 22777980 | LOC105369691 | | | | | |
| 12 | 22778075 | 22843608 | ETNK1 | | | | | |
| 12 | 23328569 | 23344521 | LOC101928441 | | | | | |
| 12 | 23685230 | 24715383 | SOX5 | | | | | |
| 12 | 24365354 | 24365429 | MIR920 | | | | | |
| 12 | 24376204 | 24390899 | LOC101928471 | | | | | |
| 12 | 24719897 | 24737102 | LINC00477 | | | | | |
| 12 | 24962957 | 25102393 | BCAT1 | | BCAT1 | | | |
| 12 | 25146364 | 25150373 | C12orf77 | | | | | |
| 12 | 25205180 | 25261269 | LRMP | | | | | |
| 12 | 25261222 | 25348096 | CASC1 | | | | | |
| 12 | 25348149 | 25357949 | LYRM5 | | | | | |
| 12 | 25357722 | 25403865 | KRAS | KRAS | | | | |
| 12 | 25629015 | 25706217 | LMNTD1 | | | | | |
| 12 | 26026952 | 26027012 | MIR4302 | | | | | |
| 12 | 26107587 | 26225807 | RASSF8 | | | | | |
| 12 | 26272958 | 26278003 | BHLHE41 | | BHLHE41 | | | |
| 12 | 26348268 | 26387708 | SSPN | | | SSPN | | |
| 12 | 26488269 | 26986131 | ITPR2 | | | | ITPR2 | |
| 12 | 27058111 | 27091254 | ASUN | | | | | |
| 12 | 27091304 | 27119581 | FGFR1OP2 | | | | | |
| 12 | 27124505 | 27167339 | TM7SF3 | | | | | |
| 12 | 27175454 | 27183606 | MED21 | | | | | |
| 12 | 27233989 | 27235455 | C12orf71 | | | | | |
| 12 | 27397077 | 27478890 | STK38L | | | STK38L | | |
| 12 | 27485786 | 27599567 | ARNTL2 | | | | | |
| 12 | 27619742 | 27655118 | SMCO2 | | | | | |
| 12 | 27677044 | 27848497 | PPFIBP1 | | | | | |
| 12 | 27849427 | 27850566 | REP15 | | | | | |
| 12 | 27863705 | 27909237 | MRPS35 | | | | | |
| 12 | 27915598 | 27924209 | MANSC4 | | | | | |
| 12 | 27933186 | 27955973 | KLHL42 | | | | | |
| 12 | 28111016 | 28124916 | PTHLH | | | | | |
| 12 | 28410132 | 28703099 | CCDC91 | | | | | |
| 12 | 29301935 | 29488549 | FAR2 | | | | | |
| 12 | 29433348 | 29470781 | LOC100506606 | | | | | |
| 12 | 29493578 | 29534143 | ERGIC2 | | | | | |
| 12 | 29542226 | 29650619 | OVCH1 | | | | | |
| 12 | 29653745 | 29937692 | TMTC1 | | | | | |
| 12 | 30781914 | 30848929 | IPO8 | | | | | |
| 12 | 30862485 | 30907884 | CAPRIN2 | | CAPRIN2 | | | |
| 12 | 30908007 | 30933673 | LOC645485 | | | | | |
| 12 | 30948614 | 30955645 | LINC00941 | | | | | |
| 12 | 31079837 | 31149537 | TSPAN11 | | | | | |
| 12 | 31173696 | 31257725 | DDX11 | | | | | |
| 12 | 31433519 | 31479159 | FAM60A | | | | | |
| 12 | 31477249 | 31478879 | FLJ13224 | | | | | |
| 12 | 31535156 | 31768285 | DENND5B | | | | | |
| 12 | 31800093 | 31822016 | ETFBKMT | | | | | |
| 12 | 31824070 | 31882108 | AMN1 | | | | | |
| 12 | 31944118 | 31945175 | H3F3C | | | | | |
| 12 | 32029902 | 32040137 | LOC105369723 | | | | | |
| 12 | 32112352 | 32146043 | KIAA1551 | | | | | |
| 12 | 32260184 | 32531141 | BICD1 | | | BICD1 | | |
| 12 | 32638905 | 32798984 | FGD4 | | | | | |

| | | | | | | | | | |
|---|---|---|---|---|---|---|---|---|---|
| 12 | 32832133 | 32898584 | DNM1L | | DNM1L | DNM1L | | | |
| 12 | 32899477 | 32908887 | YARS2 | | | | | | |
| 12 | 32943679 | 33049780 | PKP2 | | | | | | |
| 12 | 33528347 | 33592754 | SYT10 | | | SYT10 | | | |
| 12 | 34175215 | 34181236 | ALG10 | | | | | | |
| 12 | 38710556 | 38723528 | ALG10B | | | | | | |
| 12 | 39046001 | 39299420 | CPNE8 | | | | | | |
| 12 | 39687029 | 39837192 | KIF21A | | KIF21A | KIF21A | | | |
| 12 | 39945021 | 40013843 | ABCD2 | | | | | | |
| 12 | 40019968 | 40302102 | C12orf40 | | | | | | |
| 12 | 40148822 | 40499661 | SLC2A13 | | | | | | |
| 12 | 40618812 | 40763086 | LRRK2 | | | | | | |
| 12 | 40787195 | 40964557 | MUC19 | | | | | | |
| 12 | 41086243 | 41466213 | CNTN1 | | CNTN1 | | | | |
| 12 | 41582249 | 41968392 | PDZRN4 | | | | | | |
| 12 | 42157990 | 42159383 | LOC101927038 | | | | | | |
| 12 | 42475647 | 42538673 | GXYLT1 | | | | | | |
| 12 | 42550906 | 42632151 | YAF2 | | | | | | |
| 12 | 42705887 | 42719932 | ZCRB1 | | | | | | |
| 12 | 42717501 | 42717661 | MIR7851 | | | | | | |
| 12 | 42719946 | 42842422 | PPHLN1 | | | | | | |
| 12 | 42852139 | 42983572 | PRICKLE1 | | | | | | |
| 12 | 43009304 | 43040300 | LOC101927058 | | | | | | |
| 12 | 43040384 | 43080503 | LOC105369738 | | | | | | |
| 12 | 43086017 | 43110921 | LOC105369739 | | | | | | |
| 12 | 43748011 | 43945724 | ADAMTS20 | | | | | | |
| 12 | 44122409 | 44152561 | PUS7L | | | | | | |
| 12 | 44152746 | 44183346 | IRAK4 | | | | | | |
| 12 | 44187525 | 44200178 | TWF1 | | | TWF1 | | | |
| 12 | 44229663 | 44783545 | TMEM117 | | | | | | |
| 12 | 44902057 | 45307711 | NELL2 | | | NELL2 | | | |
| 12 | 45408538 | 45444882 | DBX2 | | | | | | |
| 12 | 45456400 | 45459194 | RACGAP1P | | | | | | |
| 12 | 45566816 | 45609789 | PLEKHA8P1 | | | | | | |
| 12 | 45580873 | 45581252 | RNY5 | | | | | | |
| 12 | 45609769 | 45826134 | ANO6 | | | ANO6 | | | |
| 12 | 46119502 | 46121704 | LINC00938 | | | | | | |
| 12 | 46123619 | 46301819 | ARID2 | | | | | | |
| 12 | 46312913 | 46384401 | SCAF11 | | | | | | |
| 12 | 46576839 | 46661770 | SLC38A1 | | | | | | |
| 12 | 46751970 | 46766645 | SLC38A2 | | | | | | |
| 12 | 46777458 | 47046362 | LOC100288798 | | | | | | |
| 12 | 47158543 | 47219780 | SLC38A4 | | | | | | |
| 12 | 47469489 | 47473734 | AMIGO2 | | | | | | |
| 12 | 47473385 | 47630446 | PCED1B | | | | | | |
| 12 | 47581594 | 47581674 | MIR4698 | | | | | | |
| 12 | 47602202 | 47610226 | PCED1B | | | | | | |
| 12 | 47699699 | 47701182 | LOC105369747 | | | | | | |
| 12 | 47757968 | 47758052 | MIR4494 | | | | | | |
| 12 | 48055714 | 48099844 | RPAP3 | | | | | | |
| 12 | 48103517 | 48119355 | ENDOU | | | | | | |
| 12 | 48128452 | 48152889 | RAPGEF3 | | | RAPGEF3 | | | |
| 12 | 48166966 | 48176536 | SLC48A1 | | | | | | |
| 12 | 48176493 | 48213763 | HDAC7 | | | | | | |
| 12 | 48235319 | 48298814 | VDR | | | | | | |
| 12 | 48357329 | 48362661 | TMEM106C | | | | | | |
| 12 | 48366747 | 48398285 | COL2A1 | | | COL2A1 | | | |
| 12 | 48436680 | 48500091 | SENP1 | | | | | | |
| 12 | 48499655 | 48540187 | PFKM | | PFKM | | | | |
| 12 | 48526579 | 48526650 | MIR6505 | | | | | | |
| 12 | 48541570 | 48551378 | ASB8 | | | | | | |
| 12 | 48577365 | 48579709 | CCDC184 | | | | | | |
| 12 | 48596121 | 48597075 | OR10AD1 | | | | | | |
| 12 | 48722762 | 48724062 | H1FNT | | | | | | |
| 12 | 48733792 | 48745029 | ZNF641 | | | | | | |
| 12 | 48866447 | 48866843 | ANP32D | | | | | | |

| | | | | | | | | |
|---|---|---|---|---|---|---|---|---|
| 12 | 48876285 | 48890297 | C12orf54 | | | | | |
| 12 | 48919414 | 48921886 | OR8S1 | | | | | |
| 12 | 48961466 | 48963829 | LALBA | | | | | |
| 12 | 49046994 | 49076035 | KANSL2 | | | | | |
| 12 | 49048164 | 49048301 | SNORA2C | | | | | |
| 12 | 49048226 | 49048313 | MIR1291 | | | | | |
| 12 | 49050430 | 49050565 | SNORA2A | | | | | |
| 12 | 49061239 | 49061376 | SNORA2B | | | | | |
| 12 | 49082240 | 49110781 | CCNT1 | | | | | |
| 12 | 49121217 | 49159569 | LINC00935 | | | | | |
| 12 | 49159973 | 49182879 | ADCY6 | | | | | |
| 12 | 49165757 | 49165820 | MIR4701 | | | | | |
| 12 | 49182929 | 49184318 | LOC100506125 | | | | | |
| 12 | 49208214 | 49222726 | CACNB3 | | | | CACNB3 | |
| 12 | 49223538 | 49245957 | DDX23 | | | | | |
| 12 | 49250915 | 49259653 | RND1 | | | | | |
| 12 | 49297892 | 49315359 | CCDC65 | | | | | |
| 12 | 49315741 | 49319330 | FKBP11 | | | | | |
| 12 | 49329991 | 49351252 | ARF3 | | | | | |
| 12 | 49359122 | 49365641 | WNT10B | | WNT10B | | | |
| 12 | 49372235 | 49376396 | WNT1 | | | WNT1 | | |
| 12 | 49388932 | 49393088 | DDN | | | | | |
| 12 | 49396054 | 49412629 | PRKAG1 | | | PRKAG1 | | |
| 12 | 49412757 | 49449107 | KMT2D | | | | | |
| 12 | 49458458 | 49463808 | RHEBL1 | | | | | |
| 12 | 49483205 | 49488602 | DHH | | | | | |
| 12 | 49490922 | 49504683 | LMBR1L | | | | | |
| 12 | 49521566 | 49525304 | TUBA1B | | | | | |
| 12 | 49578577 | 49583107 | TUBA1A | | TUBA1A | | | |
| 12 | 49621708 | 49667121 | TUBA1C | | | | | |
| 12 | 49686413 | 49718359 | LOC101927267 | | | | | |
| 12 | 49688908 | 49692481 | PRPH | | | PRPH | | |
| 12 | 49716970 | 49725514 | TROAP | | | | | |
| 12 | 49726199 | 49730971 | C1QL4 | | | | | |
| 12 | 49740699 | 49747467 | DNAJC22 | | | | | |
| 12 | 49760687 | 49921209 | SPATS2 | | | | | |
| 12 | 49782956 | 49786116 | LOC100335030 | | | | | |
| 12 | 49932939 | 49952095 | KCNH3 | | | KCNH3 | | |
| 12 | 49952076 | 49960222 | MCRS1 | | | | | |
| 12 | 49976657 | 49999433 | FAM186B | | | | | |
| 12 | 50017196 | 50038452 | PRPF40B | | | | | |
| 12 | 50031723 | 50101197 | FMNL3 | | | | | |
| 12 | 50135292 | 50158717 | TMBIM6 | | | | | |
| 12 | 50184928 | 50222208 | NCKAP5L | | | | | |
| 12 | 50222325 | 50236912 | BCDIN3D | | | | | |
| 12 | 50260678 | 50297760 | FAIM2 | | | FAIM2 | | |
| 12 | 50302665 | 50305646 | LOC283332 | | | | | |
| 12 | 50305735 | 50320122 | LOC101927292 | | | | | |
| 12 | 50344523 | 50352664 | AQP2 | | | | | |
| 12 | 50345294 | 50356707 | LOC101927318 | | | | | |
| 12 | 50355278 | 50359465 | AQP5 | | | | | |
| 12 | 50366619 | 50370922 | AQP6 | | | | | |
| 12 | 50382944 | 50419340 | RACGAP1 | | | | | |
| 12 | 50451419 | 50477405 | ASIC1 | | | | | |
| 12 | 50478982 | 50494494 | SMARCD1 | | | | | |
| 12 | 50497601 | 50505103 | GPD1 | | | | | |
| 12 | 50505763 | 50514240 | COX14 | | | | | |
| 12 | 50523088 | 50561316 | CERS5 | | | | | |
| 12 | 50569562 | 50677353 | LIMA1 | | | | | |
| 12 | 50627924 | 50627995 | MIR1293 | | | | | |
| 12 | 50721094 | 50790405 | FAM186A | | | | | |
| 12 | 50794591 | 50873788 | LARP4 | | | | | |
| 12 | 50850353 | 50850569 | SNORD133 | | | | | |
| 12 | 50898767 | 51142450 | DIP2B | | DIP2B | | | |
| 12 | 51157788 | 51214943 | ATF1 | | | | | |
| 12 | 51236700 | 51281663 | TMPRSS12 | | | | | |

| | | | | | | | | |
|---|---|---|---|---|---|---|---|---|
| 12 | 51318533 | 51326300 | METTL7A | | | | | |
| 12 | 51347781 | 51364289 | HIGD1C | | | | | |
| 12 | 51373565 | 51422058 | SLC11A2 | | | | | |
| 12 | 51442081 | 51454207 | LETMD1 | | | | | |
| 12 | 51454987 | 51477454 | CSRNP2 | | | | | |
| 12 | 51487538 | 51566926 | TFCP2 | | | | | |
| 12 | 51580718 | 51611477 | POU6F1 | | | POU6F1 | | |
| 12 | 51632507 | 51640501 | DAZAP2 | | | | | |
| 12 | 51639132 | 51664202 | SMAGP | | | | | |
| 12 | 51674821 | 51718464 | BIN2 | | | | | |
| 12 | 51722226 | 51740463 | CELA1 | | | | | |
| 12 | 51745832 | 51785200 | GALNT6 | | | | | |
| 12 | 51785100 | 51874391 | SLC4A8 | | SLC4A8 | | | |
| 12 | 51985019 | 52206648 | SCN8A | | | SCN8A | SCN8A | |
| 12 | 52211675 | 52225701 | FIGNL2 | | | | | |
| 12 | 52281792 | 52285505 | ANKRD33 | | | | | |
| 12 | 52301201 | 52317145 | ACVRL1 | | | ACVRL1 | | |
| 12 | 52345450 | 52390863 | ACVR1B | | | | | |
| 12 | 52400728 | 52409673 | GRASP | | | | | |
| 12 | 52416615 | 52453291 | NR4A1 | | | | | |
| 12 | 52463757 | 52471279 | ATG101 | | | | | |
| 12 | 52473479 | 52502103 | OR7E47P | | | | | |
| 12 | 52562779 | 52585784 | KRT80 | | | | | |
| 12 | 52599364 | 52604639 | C12orf80 | | | | | |
| 12 | 52604713 | 52617597 | LINC00592 | | | | | |
| 12 | 52626953 | 52642709 | KRT7 | | | | | |
| 12 | 52679696 | 52685299 | KRT81 | | | KRT81 | | |
| 12 | 52695648 | 52702947 | KRT86 | | | | | |
| 12 | 52708084 | 52715182 | KRT83 | | | | | |
| 12 | 52753789 | 52761309 | KRT85 | | | | | |
| 12 | 52771595 | 52779417 | KRT84 | | | KRT84 | | |
| 12 | 52787734 | 52800176 | KRT82 | | | | | |
| 12 | 52817853 | 52828110 | KRT75 | | | | | |
| 12 | 52840434 | 52845910 | KRT6B | | | | | |
| 12 | 52862299 | 52867569 | KRT6C | | | | | |
| 12 | 52880957 | 52887181 | KRT6A | | | | | |
| 12 | 52908358 | 52914243 | KRT5 | | | | | |
| 12 | 52937692 | 52946931 | KRT71 | | | | | |
| 12 | 52959602 | 52967609 | KRT74 | | | | | |
| 12 | 52979372 | 52995322 | KRT72 | | | | | |
| 12 | 53001353 | 53009089 | KRT73 | | | | | |
| 12 | 53038341 | 53045959 | KRT2 | | | | | |
| 12 | 53068519 | 53074191 | KRT1 | | | | | |
| 12 | 53083410 | 53097247 | KRT77 | | | | | |
| 12 | 53161938 | 53171129 | KRT76 | | | | | |
| 12 | 53183468 | 53189892 | KRT3 | | | | | |
| 12 | 53200326 | 53207900 | KRT4 | | | | | |
| 12 | 53215230 | 53228077 | KRT79 | | | | | |
| 12 | 53231587 | 53242778 | KRT78 | | | | | |
| 12 | 53290970 | 53343650 | KRT8 | | | KRT8 | | |
| 12 | 53342654 | 53346685 | KRT18 | | | KRT18 | | |
| 12 | 53400041 | 53435993 | EIF4B | | | | | |
| 12 | 53436972 | 53448222 | LOC283335 | | | | | |
| 12 | 53440809 | 53458162 | TNS2 | | | | | |
| 12 | 53450727 | 53450796 | MIR6757 | | | | | |
| 12 | 53458099 | 53473204 | SPRYD3 | | | | | |
| 12 | 53491435 | 53496128 | IGFBP6 | | | | | |
| 12 | 53497273 | 53518323 | SOAT2 | | | | | |
| 12 | 53551446 | 53574693 | CSAD | | | | | |
| 12 | 53574534 | 53584654 | ZNF740 | | | | | |
| 12 | 53585101 | 53601091 | ITGB7 | | | | | |
| 12 | 53604349 | 53626040 | RARG | | | | | |
| 12 | 53645369 | 53648190 | MFSD5 | | | | | |
| 12 | 53662082 | 53687427 | ESPL1 | | | | | |
| 12 | 53689234 | 53693234 | PFDN5 | | | | | |
| 12 | 53693469 | 53700965 | C12orf10 | | | | | |

| | | | | | | | | |
|---|---|---|---|---|---|---|---|---|
| 12 | 53701239 | 53715412 | AAAS | AAAS | | AAAS | | |
| 12 | 53720359 | 53738577 | SP7 | | | | | |
| 12 | 53773978 | 53810226 | SP1 | | | | | |
| 12 | 53817638 | 53825318 | AMHR2 | | | | | |
| 12 | 53835432 | 53840427 | PRR13 | | | | | |
| 12 | 53845885 | 53874946 | PCBP2 | | | | | |
| 12 | 53858251 | 53858841 | PCBP2-OT1 | | | | | |
| 12 | 53874275 | 53893444 | MAP3K12 | | | MAP3K12 | | |
| 12 | 53894704 | 53900215 | TARBP2 | | | | | |
| 12 | 53900473 | 53901422 | NPFF | | | | | |
| 12 | 53901639 | 54020199 | ATF7 | | | | | |
| 12 | 53907767 | 53911392 | LOC100652999 | | | | | |
| 12 | 53936668 | 54020199 | ATF7 | | | | | |
| 12 | 54058943 | 54070512 | ATP5G2 | | | | | |
| 12 | 54104901 | 54121307 | CALCOCO1 | | | | | |
| 12 | 54144230 | 54150818 | CISTR | | | | | |
| 12 | 54329111 | 54340328 | HOXC13 | | | | | |
| 12 | 54348713 | 54350350 | HOXC12 | | | | | |
| 12 | 54356091 | 54368740 | HOTAIR | | | | | |
| 12 | 54366909 | 54370203 | HOXC11 | | | | | |
| 12 | 54377734 | 54379303 | HOXC | | | | | |
| 12 | 54378945 | 54384062 | HOXC10 | | | | | |
| 12 | 54385521 | 54385631 | MIR196A2 | | | | | |
| 12 | 54388991 | 54393794 | HOXC | | | | | |
| 12 | 54393876 | 54397120 | HOXC9 | | | | | |
| 12 | 54402889 | 54406545 | HOXC8 | | | | | |
| 12 | 54410635 | 54424607 | HOXC6 | | | | | |
| 12 | 54410635 | 54429144 | HOXC5 | | | | | |
| 12 | 54410635 | 54449814 | HOXC4 | | | | | |
| 12 | 54422193 | 54424607 | HOXC6 | | | | | |
| 12 | 54426831 | 54429144 | HOXC5 | | | | | |
| 12 | 54427733 | 54427829 | MIR615 | | | | | |
| 12 | 54447660 | 54449814 | HOXC4 | | | | | |
| 12 | 54452037 | 54516018 | FLJ12825 | | | | | |
| 12 | 54472622 | 54475607 | LOC100240735 | | | | | |
| 12 | 54495992 | 54496483 | LOC100240734 | | | | | |
| 12 | 54519854 | 54526626 | LOC400043 | | | | | |
| 12 | 54574141 | 54582778 | SMUG1 | | | | | |
| 12 | 54624730 | 54673915 | CBX5 | | | | | |
| 12 | 54625180 | 54625260 | MIR3198 | | | | | |
| 12 | 54674487 | 54679030 | HNRNPA1 | | | | | |
| 12 | 54674532 | 54678663 | HNRNPA1P10 | | | | | |
| 12 | 54685890 | 54694821 | NFE2 | | | | | |
| 12 | 54718873 | 54745635 | COPZ1 | | | COPZ1 | | |
| 12 | 54730999 | 54731098 | MIR148B | | | | | |
| 12 | 54747474 | 54860814 | LOC102724050 | | | | | |
| 12 | 54756228 | 54758270 | GPR84 | | | | | |
| 12 | 54762912 | 54785089 | ZNF385A | | | | | |
| 12 | 54789044 | 54813244 | ITGA5 | | | | | |
| 12 | 54813568 | 54830891 | LOC102724050 | | | | | |
| 12 | 54849735 | 54867386 | GTSF1 | | | | | |
| 12 | 54891494 | 54936899 | NCKAP1L | | | | | |
| 12 | 54943176 | 54973023 | PDE1B | PDE1B | PDE1B | | | |
| 12 | 54973023 | 54982443 | PPP1R1A | | | PPP1R1A | | |
| 12 | 55001970 | 55004246 | GLYCAM1 | | | | | |
| 12 | 55024622 | 55028663 | LACRT | | | | | |
| 12 | 55038374 | 55042277 | DCD | | | | | |
| 12 | 55248298 | 55252174 | MUCL1 | | | | | |
| 12 | 55342086 | 55378530 | TESPA1 | | | | | |
| 12 | 55413728 | 55423801 | NEUROD4 | | | | | |
| 12 | 55523552 | 55524560 | OR9K2 | | | | | |
| 12 | 55614808 | 55615759 | OR10A7 | | | | | |
| 12 | 55641071 | 55642010 | OR6C74 | | | | | |
| 12 | 55688071 | 55689016 | OR6C6 | | | | | |
| 12 | 55714383 | 55715322 | OR6C1 | | | | | |
| 12 | 55725484 | 55726420 | OR6C3 | | | | | |

| | | | | | | | | |
|---|---|---|---|---|---|---|---|---|
| 12 | 55758894 | 55759833 | OR6C75 | | | | | |
| 12 | 55794312 | 55795251 | OR6C65 | | | | | |
| 12 | 55820037 | 55820976 | OR6C76 | | | | | |
| 12 | 55845997 | 55846936 | OR6C2 | | | | | |
| 12 | 55862983 | 55863922 | OR6C70 | | | | | |
| 12 | 55886161 | 55887100 | OR6C68 | | | | | |
| 12 | 55945010 | 55945940 | OR6C4 | | | | | |
| 12 | 55968198 | 55969128 | OR2AP1 | | | | | |
| 12 | 56030675 | 56031617 | OR10P1 | | | | | |
| 12 | 56075329 | 56078394 | METTL7B | | | | | |
| 12 | 56078353 | 56106089 | ITGA7 | | | ITGA7 | | |
| 12 | 56109817 | 56113491 | BLOC1S1 | | | BLOC1S1 | | |
| 12 | 56109817 | 56118526 | BLOC1S1-RDH5 | | | | | |
| 12 | 56110075 | 56113491 | BLOC1S1 | | | BLOC1S1 | | |
| 12 | 56114150 | 56118526 | RDH5 | | | | | |
| 12 | 56119226 | 56123457 | CD63 | | | | | |
| 12 | 56137063 | 56146665 | GDF11 | | | | | |
| 12 | 56146246 | 56211540 | SARNP | | | | | |
| 12 | 56211805 | 56214959 | ORMDL2 | | | | | |
| 12 | 56214743 | 56223420 | DNAJC14 | | | | | |
| 12 | 56223391 | 56230030 | TMEM198B | | | | | |
| 12 | 56229213 | 56236767 | MMP19 | | | | | |
| 12 | 56295196 | 56321697 | PYM1 | | | | | |
| 12 | 56324945 | 56347807 | DGKA | | | | | |
| 12 | 56347888 | 56360496 | PMEL | | | | | |
| 12 | 56360552 | 56366573 | CDK2 | | | | | |
| 12 | 56367696 | 56390467 | RAB5B | | | | | |
| 12 | 56391042 | 56399309 | SUOX | | | | | |
| 12 | 56403875 | 56418875 | LOC105369781 | | | | | |
| 12 | 56414688 | 56432219 | IKZF4 | | | | | |
| 12 | 56435685 | 56438007 | RPS26 | | | | | |
| 12 | 56473808 | 56479401 | ERBB3 | | ERBB3 | | | |
| 12 | 56498102 | 56507694 | PA2G4 | | | | | |
| 12 | 56510373 | 56511616 | RPL41 | | | | | |
| 12 | 56512003 | 56516280 | ZC3H10 | | | | | |
| 12 | 56521985 | 56538460 | ESYT1 | | | | | |
| 12 | 56546203 | 56551771 | MYL6B | | | | | |
| 12 | 56552044 | 56555366 | MYL6 | | | | | |
| 12 | 56555635 | 56583351 | SMARCC2 | | | SMARCC2 | | |
| 12 | 56596287 | 56615753 | RNF41 | | | | | |
| 12 | 56618124 | 56623638 | NABP2 | | | | | |
| 12 | 56623819 | 56631629 | SLC39A5 | | | | | |
| 12 | 56631590 | 56652143 | ANKRD52 | | | | | |
| 12 | 56660641 | 56664750 | COQ10A | | | | | |
| 12 | 56665482 | 56694175 | CS | | | | | |
| 12 | 56704212 | 56710128 | CNPY2 | | | | | |
| 12 | 56710006 | 56727837 | PAN2 | | | | | |
| 12 | 56732662 | 56734194 | IL23A | | | | | |
| 12 | 56735381 | 56754037 | STAT2 | | | | | |
| 12 | 56754354 | 56756583 | APOF | | | | | |
| 12 | 56810156 | 56843200 | TIMELESS | | | | | |
| 12 | 56843285 | 56848435 | MIP | | | | | |
| 12 | 56862300 | 56864767 | SPRYD4 | | | | | |
| 12 | 56864727 | 56882198 | GLS2 | | | | | |
| 12 | 56906648 | 56906764 | SNORA105C | | | | | |
| 12 | 56915608 | 56989980 | RBMS2 | | | | | |
| 12 | 56989379 | 57030163 | BAZ2A | | | | | |
| 12 | 57031958 | 57039852 | ATP5B | | | | | |
| 12 | 57037463 | 57037538 | SNORD59B | | | | | |
| 12 | 57038810 | 57038885 | SNORD59A | | | | | |
| 12 | 57057124 | 57082192 | PTGES3 | | | | | |
| 12 | 57106210 | 57119326 | NACA | | | | | |
| 12 | 57125363 | 57146146 | PRIM1 | | | | | |
| 12 | 57157001 | 57181574 | HSD17B6 | | | | | |
| 12 | 57316937 | 57328189 | SDR9C7 | | | | | |
| 12 | 57345215 | 57351418 | RDH16 | | | | | |

| | | | | | | | | |
|---|---|---|---|---|---|---|---|---|
| 12 | 57388354 | 57390469 | GPR182 | | | | | |
| 12 | 57392616 | 57400297 | ZBTB39 | | | | | |
| 12 | 57403780 | 57410344 | TAC3 | | | | | |
| 12 | 57422300 | 57444549 | MYO1A | | | MYO1A | | |
| 12 | 57449425 | 57472574 | NEMP1 | | | | | |
| 12 | 57482676 | 57489259 | NAB2 | | | | | |
| 12 | 57489186 | 57505196 | STAT6 | | | | | |
| 12 | 57522281 | 57541402 | LRP1 | | | | | |
| 12 | 57588286 | 57588359 | MIR1228 | | | | | |
| 12 | 57610577 | 57620232 | NXPH4 | | | | | |
| 12 | 57623355 | 57628718 | SHMT2 | | | | | |
| 12 | 57628685 | 57634475 | NDUFA4L2 | | | | | |
| 12 | 57637237 | 57644976 | STAC3 | | | | | |
| 12 | 57647547 | 57704246 | R3HDM2 | | | | | |
| 12 | 57828467 | 57845845 | INHBC | | | | | |
| 12 | 57849095 | 57851791 | INHBE | | | | | |
| 12 | 57853917 | 57866047 | GLI1 | | | | | |
| 12 | 57866037 | 57882597 | ARHGAP9 | | | | | |
| 12 | 57881735 | 57910438 | MARS | | | | | |
| 12 | 57906470 | 57906533 | MIR6758 | | | | | |
| 12 | 57910370 | 57914300 | DDIT3 | | | | | |
| 12 | 57912947 | 57913042 | MIR616 | | | | | |
| 12 | 57916658 | 57923931 | MBD6 | | | | | |
| 12 | 57923832 | 57941114 | DCTN2 | | | DCTN2 | DCTN2 | |
| 12 | 57943846 | 57978554 | KIF5A | | KIF5A | | | |
| 12 | 57984941 | 57997211 | PIP4K2C | | | | | |
| 12 | 57998404 | 58003587 | DTX3 | | | | | |
| 12 | 58003962 | 58011028 | ARHGEF25 | | | | | |
| 12 | 58012187 | 58015686 | LOC101927583 | | | | | |
| 12 | 58013692 | 58019934 | SLC26A10 | | | | | |
| 12 | 58019677 | 58027022 | B4GALNT1 | | B4GALNT1 | | | |
| 12 | 58087737 | 58115340 | OS9 | | | | | |
| 12 | 58118075 | 58122139 | AGAP2 | | AGAP2 | | | |
| 12 | 58138783 | 58142026 | TSPAN31 | | | | | |
| 12 | 58141509 | 58146230 | CDK4 | | | | | |
| 12 | 58142400 | 58142465 | MIR6759 | | | | | |
| 12 | 58148880 | 58154193 | MARCH9 | | | | | |
| 12 | 58156116 | 58160976 | CYP27B1 | | | | | |
| 12 | 58162350 | 58165914 | METTL1 | | | | | |
| 12 | 58166382 | 58176324 | METTL21B | | | | | |
| 12 | 58176527 | 58196639 | TSFM | | | TSFM | | |
| 12 | 58191159 | 58209852 | AVIL | | | AVIL | | |
| 12 | 58213709 | 58240747 | CTDSP2 | | | | | |
| 12 | 58218391 | 58218475 | MIR26A2 | | | | | |
| 12 | 58325231 | 58329947 | LOC100506844 | | | | | |
| 12 | 58335444 | 58351052 | XRCC6BP1 | | | | | |
| 12 | 58481674 | 58487143 | LOC105369785 | | | | | |
| 12 | 58959741 | 59175498 | LOC101927653 | | | | | |
| 12 | 58985483 | 59206450 | LOC100506869 | | | | | |
| 12 | 59265936 | 59314319 | LRIG3 | | | | | |
| 12 | 59989820 | 60183635 | SLC16A7 | | | | | |
| 12 | 62102028 | 62586620 | FAM19A2 | | | | | |
| 12 | 62654120 | 62803501 | USP15 | | | | | |
| 12 | 62654139 | 62654235 | MIR6125 | | | | | |
| 12 | 62860596 | 62991363 | MON2 | | | | | |
| 12 | 62995530 | 62997214 | LINC01465 | | | | | |
| 12 | 62997465 | 62997549 | MIRLET7I | | | | | |
| 12 | 63037762 | 63328665 | PPM1H | | | | | |
| 12 | 63536538 | 63546590 | AVPR1A | | | AVPR1A | | |
| 12 | 63952692 | 64062354 | DPY19L2 | | | | | |
| 12 | 64173582 | 64215936 | TMEM5 | | | | | |
| 12 | 64238540 | 64541613 | SRGAP1 | | | SRGAP1 | | |
| 12 | 64580091 | 64616076 | C12orf66 | | | | | |
| 12 | 64660762 | 64784345 | C12orf56 | | | | | |
| 12 | 64798152 | 64842463 | XPOT | | | | | |
| 12 | 64845839 | 64895899 | TBK1 | | | | | |

| | | | | | | | | |
|---|---|---|---|---|---|---|---|---|
| 12 | 65004292 | 65091347 | RASSF3 | | | | | |
| 12 | 65016288 | 65016385 | MIR548C | | | | | |
| 12 | 65016288 | 65016385 | MIR548Z | | | | | |
| 12 | 65107221 | 65153226 | GNS | | | | | |
| 12 | 65218351 | 65274798 | TBC1D30 | | | | | |
| 12 | 65277553 | 65371302 | FLJ41278 | | | | | |
| 12 | 65444403 | 65515346 | WIF1 | | | | | |
| 12 | 65563350 | 65642141 | LEMD3 | | | LEMD3 | | |
| 12 | 65672422 | 65860687 | MSRB3 | | | | | |
| 12 | 65860598 | 66036152 | LOC100507065 | | | | | |
| 12 | 65938017 | 65951091 | LOC105369187 | | | | | |
| 12 | 65951678 | 66036152 | LOC100507065 | | | | | |
| 12 | 66151799 | 66220754 | RPSAP52 | | | | | |
| 12 | 66218239 | 66360071 | HMGA2 | | | | | |
| 12 | 66245006 | 66275358 | LOC100129940 | | | | | |
| 12 | 66417399 | 66417506 | MIR6074 | | | | | |
| 12 | 66516848 | 66528229 | LLPH | | | | | |
| 12 | 66530715 | 66563852 | TMBIM4 | | | | | |
| 12 | 66582977 | 66648394 | IRAK3 | | | | | |
| 12 | 66644861 | 66644937 | MIR6502 | | | | | |
| 12 | 66696324 | 66737423 | HELB | | | | | |
| 12 | 66741210 | 67072925 | GRIP1 | | GRIP1 | GRIP1 | | GRIP1 |
| 12 | 67471405 | 67490064 | LOC102724421 | | | | | |
| 12 | 67663060 | 67708472 | CAND1 | | | | | |
| 12 | 67913861 | 67960911 | LOC100507175 | | | | | |
| 12 | 68042511 | 68056444 | DYRK2 | | | | | |
| 12 | 68102826 | 68123255 | LOC101927901 | | | | | |
| 12 | 68323014 | 68363797 | LINC01479 | | | | | |
| 12 | 68383224 | 68553521 | IFNG | | | | | |
| 12 | 68595128 | 68619571 | IL26 | | | | | |
| 12 | 68642024 | 68647281 | IL22 | | | | | |
| 12 | 68688345 | 68726161 | MDM1 | | | | | |
| 12 | 68825621 | 68845264 | LOC100507195 | | | | | |
| 12 | 69004618 | 69054385 | RAP1B | | | RAP1B | | |
| 12 | 69021013 | 69021155 | SNORA70G | | | | | |
| 12 | 69068150 | 69080639 | LOC100507250 | | | | | |
| 12 | 69080730 | 69136473 | NUP107 | | | | | |
| 12 | 69139935 | 69159853 | SLC35E3 | | | | | |
| 12 | 69198216 | 69199274 | LOC100130075 | | | | | |
| 12 | 69201951 | 69239324 | MDM2 | | | | | |
| 12 | 69244955 | 69357020 | CPM | | | | | |
| 12 | 69633316 | 69668138 | CPSF6 | | | | | |
| 12 | 69666936 | 69666998 | MIR1279 | | | | | |
| 12 | 69742133 | 69748013 | LYZ | | | | | |
| 12 | 69753489 | 69784650 | YEATS4 | | | | | |
| 12 | 69864128 | 69973571 | FRS2 | | | | | |
| 12 | 69978501 | 69978602 | MIR3913 | | | | | |
| 12 | 69979207 | 69995357 | CCT2 | | | | | |
| 12 | 70002344 | 70004942 | LRRC10 | | | | | |
| 12 | 70037287 | 70093196 | BEST3 | | | BEST3 | | |
| 12 | 70107412 | 70132348 | LOC101928002 | | | | | |
| 12 | 70132465 | 70216984 | RAB3IP | | | RAB3IP | | |
| 12 | 70219102 | 70352505 | MYRFL | | | | | |
| 12 | 70615969 | 70637140 | LINC01481 | | | | | |
| 12 | 70636773 | 70748773 | CNOT2 | | | | | |
| 12 | 70760061 | 70828072 | KCNMB4 | | | KCNMB4 | | |
| 12 | 70910631 | 71031220 | PTPRB | | | PTPRB | | |
| 12 | 71031852 | 71314584 | PTPRR | | | | | |
| 12 | 71518876 | 71551779 | TSPAN8 | | | | | |
| 12 | 71833549 | 71980088 | LGR5 | | | | | |
| 12 | 72003378 | 72057749 | ZFC3H1 | | | | | |
| 12 | 72057676 | 72074428 | THAP2 | | | | | |
| 12 | 72079877 | 72097839 | TMEM19 | | | | | |
| 12 | 72148642 | 72187256 | RAB21 | | | RAB21 | | |
| 12 | 72233486 | 72320629 | TBC1D15 | | | | | |
| 12 | 72242073 | 72244763 | MRS2P2 | | | | | |

| | | | | | | | | |
|---|---|---|---|---|---|---|---|---|
| 12 | 72332625 | 72426221 | TPH2 | | TPH2 | | | |
| 12 | 72647286 | 73059422 | TRHDE | | | | | |
| 12 | 73552969 | 73602097 | LOC101928137 | | | | | |
| 12 | 74526955 | 74686411 | LOC100507377 | | | | | |
| 12 | 74931550 | 74935232 | ATXN7L3B | | | | | |
| 12 | 75433857 | 75603528 | KCNC2 | | | | | |
| 12 | 75669758 | 75784702 | CAPS2 | | | | | |
| 12 | 75728418 | 75764169 | GLIPR1L1 | | | | | |
| 12 | 75784849 | 75826177 | GLIPR1L2 | | | | | |
| 12 | 75874512 | 75895716 | GLIPR1 | | | | | |
| 12 | 75891418 | 75905418 | KRR1 | | | | | |
| 12 | 76419226 | 76425556 | PHLDA1 | | | | | |
| 12 | 76439162 | 76478813 | NAP1L1 | | | | | |
| 12 | 76738265 | 76742222 | BBS10 | | | | | |
| 12 | 76745577 | 76953589 | OSBPL8 | | | | | |
| 12 | 77157853 | 77247474 | ZDHHC17 | | | | | |
| 12 | 77252494 | 77272820 | CSRP2 | | | | | |
| 12 | 77415025 | 77459360 | E2F7 | | | | | |
| 12 | 78225068 | 78606792 | NAV3 | | | | | |
| 12 | 78720459 | 78753526 | LOC105369860 | | | | | |
| 12 | 79257772 | 79845788 | SYT1 | | | SYT1 | | |
| 12 | 79813036 | 79813101 | MIR1252 | | | | | |
| 12 | 79850650 | 79852440 | MIR5692B | | | | | |
| 12 | 79985744 | 80084790 | PAWR | | | PAWR | | |
| 12 | 80167342 | 80329235 | PPP1R12A | | | | | |
| 12 | 80603232 | 80772870 | OTOGL | | | | | |
| 12 | 80838125 | 81073968 | PTPRQ | | | | | |
| 12 | 81101407 | 81103256 | MYF6 | | | | | |
| 12 | 81110707 | 81113447 | MYF5 | | | | | |
| 12 | 81156932 | 81164494 | LINC01490 | | | | | |
| 12 | 81191170 | 81331694 | LIN7A | | | LIN7A | LIN7A | LIN7A |
| 12 | 81226311 | 81226408 | MIR617 | | | | | |
| 12 | 81329514 | 81329612 | MIR618 | | | | | |
| 12 | 81471808 | 81649582 | ACSS3 | | | | | |
| 12 | 81552166 | 81552240 | MIR4699 | | | | | |
| 12 | 81651753 | 82153109 | PPFIA2 | | | | | |
| 12 | 81672967 | 81706201 | LOC102724663 | | | | | |
| 12 | 82347497 | 82386912 | LOC101928449 | | | | | |
| 12 | 82746082 | 82752584 | CCDC59 | | | | | |
| 12 | 82752275 | 82873016 | METTL25 | | | | | |
| 12 | 83080933 | 83528067 | TMTC2 | | | | | |
| 12 | 85253266 | 85306608 | SLC6A15 | | | SLC6A15 | | |
| 12 | 85408093 | 85430055 | TSPAN19 | | | | | |
| 12 | 85430098 | 85638883 | LRRIQ1 | | | | | |
| 12 | 85674035 | 85695561 | ALX1 | | | | | |
| 12 | 86198330 | 86230318 | RASSF9 | | | | | |
| 12 | 86268072 | 86276770 | NTS | | | NTS | | |
| 12 | 86373036 | 87232681 | MGAT4C | | | | | |
| 12 | 86946651 | 86946729 | MIR548AL | | | | | |
| 12 | 87724515 | 87726098 | LOC105369879 | | | | | |
| 12 | 88176662 | 88178488 | MKRN9P | | | | | |
| 12 | 88373815 | 88423176 | C12orf50 | | | | | |
| 12 | 88429267 | 88443937 | C12orf29 | | | | | |
| 12 | 88442789 | 88535993 | CEP290 | | | CEP290 | | |
| 12 | 88536072 | 88593664 | TMTC3 | | | | | |
| 12 | 88886569 | 88974250 | KITLG | | | | | |
| 12 | 89404902 | 89413469 | LOC728084 | | | | | |
| 12 | 89741601 | 89746636 | DUSP6 | | | | | |
| 12 | 89813497 | 89920039 | POC1B | | | | | |
| 12 | 89913189 | 89918583 | GALNT4 | | | | | |
| 12 | 89913189 | 89920039 | POC1B-GALNT4 | | | | | |
| 12 | 89981825 | 90049844 | ATP2B1 | | ATP2B1 | ATP2B1 | | |
| 12 | 90102731 | 90105729 | LINC00936 | | | | | |
| 12 | 90341469 | 90343503 | LOC105369891 | | | | | |
| 12 | 90687094 | 90694475 | LOC105369893 | | | | | |
| 12 | 91311799 | 91342446 | LINC00615 | | | | | |

| | | | | | | | | |
|---|---|---|---|---|---|---|---|---|
| 12 | 91345991 | 91348953 | CCER1 | | | | | |
| 12 | 91357455 | 91398803 | EPYC | | | | | |
| 12 | 91444270 | 91452131 | KERA | | | SLC25A3 | | |
| 12 | 91497231 | 91505542 | LUM | | | | | |
| 12 | 91537053 | 91576901 | DCN | | | | | |
| 12 | 92378751 | 92536607 | LINC01619 | | | | | |
| 12 | 92534053 | 92539673 | BTG1 | | | | | |
| 12 | 92539860 | 92579560 | LOC101928617 | | | | | |
| 12 | 92813869 | 92821924 | CLLU1OS | | | | | |
| 12 | 92815306 | 92824778 | CLLU1 | | | | | |
| 12 | 93096618 | 93102325 | C12orf74 | | | | | |
| 12 | 93130264 | 93165868 | PLEKHG7 | | | | | |
| 12 | 93166284 | 93323107 | EEA1 | | | | | |
| 12 | 93397533 | 93771512 | LOC643339 | | | | | |
| 12 | 93568141 | 93575608 | LOC102724933 | | | | | |
| 12 | 93771698 | 93797024 | NUDT4 | | | | | |
| 12 | 93771701 | 93796045 | NUDT4P2 | | | | | |
| 12 | 93771745 | 93796052 | NUDT4P1 | | | | | |
| 12 | 93772325 | 93797024 | NUDT4 | | | | | |
| 12 | 93802087 | 93836026 | UBE2N | | | | | |
| 12 | 93861265 | 93897548 | MRPL42 | | | | | |
| 12 | 93959403 | 93970521 | SOCS2 | | | | | |
| 12 | 94071150 | 94244531 | CRADD | | | | | |
| 12 | 94101566 | 94131599 | LOC101928731 | | | | | |
| 12 | 94288740 | 94337382 | LOC105369911 | | | | | |
| 12 | 94542498 | 94701451 | PLXNC1 | | | | | |
| 12 | 94702055 | 94856344 | CEP83 | | | | | |
| 12 | 94955564 | 94955635 | MIR5700 | | | | | |
| 12 | 94960899 | 95044338 | TMCC3 | | | | | |
| 12 | 94965006 | 94965128 | MIR7844 | | | | | |
| 12 | 95228173 | 95228289 | MIR492 | | | | | |
| 12 | 95228229 | 95228804 | KRT19P2 | | | | | |
| 12 | 95365103 | 95397489 | NDUFA12 | | | | | |
| 12 | 95414004 | 95467404 | NR2C1 | | | | | |
| 12 | 95470524 | 95611240 | FGD6 | | | | | |
| 12 | 95611521 | 95696566 | VEZT | | | | | |
| 12 | 95702195 | 95702289 | MIR331 | | | | | |
| 12 | 95703698 | 95703760 | MIR3685 | | | | | |
| 12 | 95867821 | 95909613 | METAP2 | | | | | |
| 12 | 95910335 | 95945266 | USP44 | | | | | |
| 12 | 96043030 | 96067770 | PGAM1P5 | | | | | |
| 12 | 96051582 | 96184536 | NTN4 | | | | | |
| 12 | 96189122 | 96252617 | LOC105369921 | | | | | |
| 12 | 96196874 | 96217092 | LOC105369920 | | | | | |
| 12 | 96252014 | 96252617 | LOC105369921 | | | | | |
| 12 | 96252708 | 96260238 | SNRPF | | | | | |
| 12 | 96260825 | 96336428 | CCDC38 | | | | | |
| 12 | 96337070 | 96362370 | AMDHD1 | | | | | |
| 12 | 96366439 | 96390143 | HAL | | | | | |
| 12 | 96394530 | 96437298 | LTA4H | | | | | |
| 12 | 96588159 | 96663613 | ELK3 | | | | | |
| 12 | 96672038 | 96794366 | CDK17 | | | | | |
| 12 | 96883348 | 97269333 | CFAP54 | | | | | |
| 12 | 97301000 | 97347469 | NEDD1 | | | | | |
| 12 | 97858798 | 97927544 | RMST | | | | | |
| 12 | 97885686 | 97885756 | MIR1251 | | | | | |
| 12 | 97957589 | 97957689 | MIR135A2 | | | | | |
| 12 | 98107189 | 98150295 | LOC643711 | | | | | |
| 12 | 98332833 | 98332899 | MIR4495 | | | | | |
| 12 | 98389160 | 98389226 | MIR4303 | | | | | |
| 12 | 98847618 | 98850923 | SLC9A7P1 | | | | | |
| 12 | 98879321 | 98897633 | LOC643770 | | | | | |
| 12 | 98906750 | 98944157 | TMPO | | | | | |
| 12 | 98987402 | 98995778 | SLC25A3 | | | SLC25A3 | | |
| 12 | 98993412 | 98993662 | SNORA53 | | | | | |
| 12 | 99007181 | 99038829 | IKBIP | | | | | |

| | | | | | | | | |
|---|---|---|---|---|---|---|---|---|
| 12 | 99039077 | 99129211 | APAF1 | | | | | |
| 12 | 99128568 | 99548868 | ANKS1B | | ANKS1B | | | |
| 12 | 99487136 | 99498789 | LOC101928937 | | | | | |
| 12 | 100041527 | 100043892 | FAM71C | | | | | |
| 12 | 100430862 | 100536642 | UHRF1BP1L | | | | | |
| 12 | 100550174 | 100567121 | GOLGA2P5 | | | | | |
| 12 | 100583661 | 100583727 | MIR1827 | | | | | |
| 12 | 100593864 | 100618202 | ACTR6 | | | | | |
| 12 | 100597446 | 100660857 | DEPDC4 | | | | | |
| 12 | 100660917 | 100735502 | SCYL2 | | | | | |
| 12 | 100750856 | 100815837 | SLC17A8 | | SLC17A8 | | | |
| 12 | 100867550 | 100957645 | NR1H4 | | | | | |
| 12 | 100967438 | 101022066 | GAS2L3 | | | | | |
| 12 | 101188373 | 101522419 | ANO4 | | | | | |
| 12 | 101549993 | 101604016 | SLC5A8 | | | | | |
| 12 | 101673904 | 101780397 | UTP20 | | | | | |
| 12 | 101786897 | 101801598 | ARL1 | | | | | |
| 12 | 101871334 | 101880775 | SPIC | | | | | |
| 12 | 101988708 | 102079658 | MYBPC1 | | MYBPC1 | | | |
| 12 | 102091416 | 102122846 | CHPT1 | | | | | |
| 12 | 102122425 | 102133250 | SYCP3 | | | | | |
| 12 | 102139274 | 102224645 | GNPTAB | | | GNPTAB | | |
| 12 | 102271104 | 102317401 | DRAM1 | | | | | |
| 12 | 102406617 | 102455902 | CCDC53 | | | | | |
| 12 | 102467966 | 102513909 | NUP37 | | | | | |
| 12 | 102513948 | 102591298 | PARPBP | | | | | |
| 12 | 102590236 | 102591623 | PMCH | | | PMCH | | |
| 12 | 102789644 | 102874423 | IGF1 | | | | | |
| 12 | 103203060 | 103218177 | LINC00485 | | | | | |
| 12 | 103232103 | 103311381 | PAH | | PAH | PAH | | |
| 12 | 103351451 | 103354294 | ASCL1 | | ASCL1 | | ASCL1 | |
| 12 | 103545619 | 103562086 | LOC101929058 | | | | | |
| 12 | 103631368 | 103889788 | C12orf42 | | | | | |
| 12 | 103889817 | 103891473 | LOC105369945 | | | | | |
| 12 | 103941571 | 103953593 | LOC101929084 | | | | | |
| 12 | 103981068 | 104160502 | STAB2 | | | STAB2 | | |
| 12 | 104166080 | 104234975 | NT5DC3 | | | | | |
| 12 | 104237526 | 104323989 | TTC41P | | | | | |
| 12 | 104324111 | 104341708 | HSP90B1 | | | | | |
| 12 | 104324202 | 104324333 | MIR3652 | | | | | |
| 12 | 104343980 | 104350993 | C12orf73 | | | | | |
| 12 | 104359592 | 104382656 | TDG | | | | | |
| 12 | 104382760 | 104457955 | GLT8D2 | | | | | |
| 12 | 104458235 | 104500304 | HCFC2 | | | | | |
| 12 | 104510857 | 104532040 | NFYB | | | | | |
| 12 | 104519052 | 104519102 | MIR7641 | | | | | |
| 12 | 104609556 | 104744085 | TXNRD1 | | | | | |
| 12 | 104697509 | 104698982 | EID3 | | | | | |
| 12 | 104850691 | 105155792 | CHST11 | | CHST11 | | | |
| 12 | 104985410 | 104985494 | MIR3922 | | | | | |
| 12 | 105197274 | 105322472 | SLC41A2 | | | | | |
| 12 | 105380097 | 105388505 | C12orf45 | | | | | |
| 12 | 105413561 | 105478341 | ALDH1L2 | | | | | |
| 12 | 105496249 | 105501420 | LOC414300 | | | | | |
| 12 | 105501491 | 105562912 | KIAA1033 | | | | | |
| 12 | 105567074 | 105630008 | APPL2 | | | APPL2 | | |
| 12 | 105698644 | 105720795 | KCCAT198 | | | | | |
| 12 | 105724413 | 105765296 | C12orf75 | | | | | |
| 12 | 106097980 | 106137841 | CASC18 | | | | | |
| 12 | 106457124 | 106533811 | NUAK1 | | | NUAK1 | | |
| 12 | 106631658 | 106641713 | CKAP4 | | | | | |
| 12 | 106696568 | 106740792 | TCP11L2 | | | | | |
| 12 | 106751435 | 106903976 | POLR3B | | | | | |
| 12 | 106890187 | 107168609 | LOC100287944 | | | | | |
| 12 | 106976684 | 107156582 | RFX4 | | RFX4 | | | |
| 12 | 107074535 | 107078478 | LOC100505978 | | | | | |

| | | | | | | | | |
|---|---|---|---|---|---|---|---|---|
| 12 | 107078476 | 107156582 | RFX4 | | RFX4 | | | |
| 12 | 107168398 | 107283094 | RIC8B | | | | | |
| 12 | 107349499 | 107367815 | TMEM263 | | | | | |
| 12 | 107371068 | 107380944 | MTERF2 | | | | | |
| 12 | 107385142 | 107487635 | CRY1 | | | | | |
| 12 | 107712196 | 108053419 | BTBD11 | | | | | |
| 12 | 108079508 | 108106939 | PWP1 | | | | | |
| 12 | 108126642 | 108154914 | PRDM4 | | | | | |
| 12 | 108130331 | 108153745 | LOC101929162 | | | | | |
| 12 | 108168161 | 108170421 | ASCL4 | | | | | |
| 12 | 108296926 | 108297548 | LOC728739 | | | | | |
| 12 | 108523247 | 108644313 | WSCD2 | | | | | |
| 12 | 108681820 | 108733094 | CMKLR1 | | | | | |
| 12 | 108853201 | 108867466 | LINC01498 | | | | | |
| 12 | 108909050 | 108913380 | FICD | | | | | |
| 12 | 108915990 | 108955165 | SART3 | | | | | |
| 12 | 108955238 | 108963160 | ISCU | | | ISCU | | |
| 12 | 108983621 | 108991894 | TMEM119 | | | | | |
| 12 | 109015679 | 109027670 | SELPLG | | | | | |
| 12 | 109029585 | 109029646 | MIR4496 | | | | | |
| 12 | 109038884 | 109125326 | CORO1C | | | | | |
| 12 | 109176465 | 109251359 | SSH1 | | | | | |
| 12 | 109230683 | 109230782 | MIR619 | | | | | |
| 12 | 109273856 | 109294710 | DAO | | DAO | | | |
| 12 | 109301904 | 109459045 | SVOP | | | | | |
| 12 | 109460893 | 109525831 | USP30 | | | | | |
| 12 | 109525992 | 109531293 | ALKBH2 | | | | | |
| 12 | 109535398 | 109548798 | UNG | UNG | | UNG | | |
| 12 | 109577201 | 109706030 | ACACB | | | | | |
| 12 | 109715782 | 109747025 | FOXN4 | | | | | |
| 12 | 109791991 | 109797293 | LINC01486 | | | | | |
| 12 | 109826523 | 109886176 | MYO1H | | | | | |
| 12 | 109886459 | 109915349 | KCTD10 | | | | | |
| 12 | 109915427 | 109974510 | UBE3B | | | | | |
| 12 | 109991520 | 110011358 | MMAB | | | | | |
| 12 | 110011499 | 110035075 | MVK | | | MVK | | |
| 12 | 110152186 | 110211292 | FAM222A | | | | | |
| 12 | 110220891 | 110271212 | TRPV4 | | | | | |
| 12 | 110271152 | 110271241 | MIR4497 | | | | | |
| 12 | 110288747 | 110318293 | GLTP | | | | | |
| 12 | 110338078 | 110355874 | TCHP | | | | | |
| 12 | 110367606 | 110434194 | GIT2 | | | | | |
| 12 | 110437234 | 110477235 | ANKRD13A | | | | | |
| 12 | 110478981 | 110505500 | C12orf76 | | | | | |
| 12 | 110562139 | 110656600 | IFT81 | | | | | |
| 12 | 110719031 | 110788897 | ATP2A2 | | ATP2A2 | ATP2A2 | | |
| 12 | 110810704 | 110841535 | ANAPC7 | | | | | |
| 12 | 110872694 | 110888216 | ARPC3 | | | | | |
| 12 | 110890290 | 110906526 | GPN3 | | | | | |
| 12 | 110906231 | 110928192 | FAM216A | | | | | |
| 12 | 110929327 | 110939945 | VPS29 | | | | | |
| 12 | 110940004 | 110969891 | RAD9B | | | | | |
| 12 | 110972236 | 111021064 | PPTC7 | | | | | |
| 12 | 111051831 | 111086935 | TCTN1 | | | | | |
| 12 | 111086490 | 111127617 | HVCN1 | | | | | |
| 12 | 111157612 | 111180783 | PPP1CC | | | PPP1CC | | PPP1CC |
| 12 | 111284763 | 111345339 | CCDC63 | | | | | |
| 12 | 111348623 | 111358404 | MYL2 | | | | | |
| 12 | 111374405 | 111375250 | LINC01405 | | | | | |
| 12 | 111389486 | 111395622 | LOC105369980 | | | | | |
| 12 | 111471827 | 111788358 | CUX2 | | CUX2 | | | |
| 12 | 111741945 | 111742013 | MIR6760 | | | | | |
| 12 | 111798454 | 111806925 | FAM109A | | | | | |
| 12 | 111843751 | 111889427 | SH2B3 | | | | | |
| 12 | 111890017 | 112037480 | ATXN2 | | | ATXN2 | ATXN2 | |
| 12 | 112079949 | 112123800 | BRAP | | | BRAP | | |

| | | | | | | | | |
|---|---|---|---|---|---|---|---|---|
| 12 | 112123856 | 112194911 | ACAD10 | | | | | |
| 12 | 112204690 | 112247789 | ALDH2 | | | ALDH2 | | |
| 12 | 112237637 | 112237709 | MIR6761 | | | | | |
| 12 | 112277572 | 112331228 | MAPKAPK5 | | | | | |
| 12 | 112336866 | 112339706 | ADAM1A | | | | | |
| 12 | 112369086 | 112451023 | TMEM116 | | | | | |
| 12 | 112451151 | 112461024 | ERP29 | | | | | |
| 12 | 112464492 | 112546635 | NAA25 | | | | | |
| 12 | 112475402 | 112475519 | MIR3657 | | | | | |
| 12 | 112563348 | 112591408 | TRAFD1 | | | | | |
| 12 | 112597991 | 112819896 | HECTD4 | | | | | |
| 12 | 112601061 | 112601125 | MIR6861 | | | | | |
| 12 | 112842993 | 112847443 | RPL6 | | | | | |
| 12 | 112856535 | 112947717 | PTPN11 | | PTPN11 | PTPN11 | | |
| 12 | 113132838 | 113132981 | MIR1302 | | | | | |
| 12 | 113229548 | 113336684 | RPH3A | | | RPH3A | | |
| 12 | 113344738 | 113357712 | OAS1 | | | | | |
| 12 | 113376237 | 113411055 | OAS3 | | | | | |
| 12 | 113416273 | 113449528 | OAS2 | | | | | |
| 12 | 113495661 | 113535833 | DTX1 | | | | | |
| 12 | 113536623 | 113574044 | RASAL1 | | | | | |
| 12 | 113587662 | 113597081 | CFAP73 | | | | | |
| 12 | 113594977 | 113623284 | DDX54 | | | | | |
| 12 | 113596917 | 113596982 | MIR7106 | | | | | |
| 12 | 113623330 | 113630173 | RITA1 | | | | | |
| 12 | 113633245 | 113658899 | IQCD | | | | | |
| 12 | 113659242 | 113736389 | TPCN1 | | | | | |
| 12 | 113729327 | 113729413 | MIR6762 | | | | | |
| 12 | 113736570 | 113772925 | SLC8B1 | | | | | |
| 12 | 113796370 | 113827458 | PLBD2 | | | | | |
| 12 | 113830250 | 113841692 | SDS | | | | | |
| 12 | 113860194 | 113876081 | SDSL | | | | | |
| 12 | 113900693 | 113918286 | LHX5 | | | LHX5 | | |
| 12 | 114182381 | 114211488 | LINC01234 | | | | | |
| 12 | 114254542 | 114404176 | RBM19 | | | | | |
| 12 | 114791734 | 114850637 | TBX5 | | | TBX5 | | |
| 12 | 115108058 | 115121969 | TBX3 | | | TBX3 | | |
| 12 | 116396380 | 116714991 | MED13L | | | | | |
| 12 | 116586364 | 116586459 | MIR620 | | | | | |
| 12 | 116866056 | 116866123 | MIR4472 | | | | | |
| 12 | 116971226 | 116974318 | LINC00173 | | | | | |
| 12 | 116997185 | 117014425 | MAP1LC3B2 | | | | | |
| 12 | 117151124 | 117175875 | C12orf49 | | | | | |
| 12 | 117176095 | 117291436 | RNFT2 | | | | | |
| 12 | 117298224 | 117319232 | HRK | | | | | |
| 12 | 117348760 | 117468953 | FBXW8 | | | | | |
| 12 | 117415244 | 117425142 | LOC100506551 | | | | | |
| 12 | 117476727 | 117579294 | TESC | | | | | |
| 12 | 117581584 | 117628300 | FBXO21 | | | | | |
| 12 | 117645946 | 117799607 | NOS1 | | NOS1 | NOS1 | | NOS1 |
| 12 | 117890816 | 118406028 | KSR2 | | | | | |
| 12 | 118454505 | 118470042 | RFC5 | | | | | |
| 12 | 118470496 | 118499979 | WSB2 | | | | | |
| 12 | 118501397 | 118541810 | VSIG10 | | | | | |
| 12 | 118573688 | 118583393 | PEBP1 | | | PEBP1 | | |
| 12 | 118587605 | 118810750 | TAOK3 | | | | | |
| 12 | 118814357 | 118855840 | SUDS3 | | | | | |
| 12 | 119196558 | 119199484 | LOC105370014 | | | | | |
| 12 | 119256611 | 119271341 | LOC105370016 | | | | | |
| 12 | 119419299 | 119600856 | SRRM4 | | | | | |
| 12 | 119611870 | 119614289 | LOC105370024 | | | | | |
| 12 | 119616594 | 119632551 | HSPB8 | | | HSPB8 | | |
| 12 | 119721629 | 119741185 | LINC00934 | | | | | |
| 12 | 119772516 | 119978852 | CCDC60 | | | | | |
| 12 | 120031263 | 120079363 | TMEM233 | | | | | |
| 12 | 120105760 | 120119429 | PRKAB1 | | | | | |

| | | | | | | | | |
|---|---|---|---|---|---|---|---|---|
| 12 | 120123594 | 120315095 | CIT | | CIT | CIT | | |
| 12 | 120151438 | 120151529 | MIR1178 | | | | | |
| 12 | 120427647 | 120532299 | CCDC64 | | | | | |
| 12 | 120532898 | 120554643 | RAB35 | | | | | |
| 12 | 120565013 | 120632513 | GCN1 | | | | | |
| 12 | 120593237 | 120593303 | MIR4498 | | | | | |
| 12 | 120634502 | 120639014 | RPLP0 | | | | | |
| 12 | 120639093 | 120703574 | PXN | | | PXN | | |
| 12 | 120740123 | 120751045 | SIRT4 | | | | | |
| 12 | 120759913 | 120765592 | PLA2G1B | | | | | |
| 12 | 120779132 | 120806983 | MSI1 | | | | | |
| 12 | 120875892 | 120878545 | COX6A1 | | COX6A1 | COX6A1 | | |
| 12 | 120881763 | 120884215 | TRIAP1 | | | | | |
| 12 | 120884240 | 120901556 | GATC | | | | | |
| 12 | 120899470 | 120907558 | SRSF9 | | | | | |
| 12 | 120907659 | 120936298 | DYNLL1 | | | | | |
| 12 | 120928140 | 120933749 | NRAV | | | | | |
| 12 | 120933858 | 120936298 | DYNLL1 | | | | | |
| 12 | 120941081 | 120966964 | COQ5 | | | | | |
| 12 | 120972131 | 121015397 | RNF10 | | | | | |
| 12 | 121016847 | 121019201 | POP5 | | | | | |
| 12 | 121078421 | 121105127 | CABP1 | | CABP1 | | | |
| 12 | 121124926 | 121139667 | MLEC | | | | | |
| 12 | 121148237 | 121161443 | UNC119B | | | | | |
| 12 | 121160995 | 121161069 | MIR4700 | | | | | |
| 12 | 121163540 | 121177811 | ACADS | | | ACADS | | |
| 12 | 121200312 | 121342155 | SPPL3 | | | | | |
| 12 | 121342497 | 121345877 | XLOC_009911 | | | | | |
| 12 | 121407640 | 121440314 | HNF1A | | | | | |
| 12 | 121440224 | 121454305 | C12orf43 | | | | | |
| 12 | 121458094 | 121477045 | OASL | | | | | |
| 12 | 121570621 | 121624354 | P2RX7 | | P2RX7 | | P2RX7 | |
| 12 | 121647663 | 121671909 | P2RX4 | | | | | |
| 12 | 121675494 | 121736111 | CAMKK2 | | | CAMKK2 | CAMKK2 | |
| 12 | 121746047 | 121792012 | ANAPC5 | | | | | |
| 12 | 121837885 | 121862155 | RNF34 | | | RNF34 | | |
| 12 | 121866899 | 122018920 | KDM2B | | | | | |
| 12 | 121882075 | 121882155 | MIR7107 | | | | | |
| 12 | 122034433 | 122034505 | MIR548AQ | | | | | |
| 12 | 122064454 | 122079946 | ORAI1 | | | | | |
| 12 | 122089292 | 122107560 | MORN3 | | | | | |
| 12 | 122150657 | 122219974 | TMEM120B | | | | | |
| 12 | 122215659 | 122231594 | RHOF | | | | | |
| 12 | 122233172 | 122241390 | LINC01089 | | | | | |
| 12 | 122242637 | 122270562 | SETD1B | | | | | |
| 12 | 122277432 | 122326517 | HPD | | | HPD | | |
| 12 | 122326636 | 122355771 | PSMD9 | | | | | |
| 12 | 122356462 | 122441832 | WDR66 | | | | | |
| 12 | 122459791 | 122499950 | BCL7A | | | | | |
| 12 | 122501195 | 122506466 | LOC100506691 | | | | | |
| 12 | 122516633 | 122631894 | MLXIP | | | | | |
| 12 | 122652265 | 122688018 | LRRC43 | | | | | |
| 12 | 122656576 | 122658746 | IL31 | | | | | |
| 12 | 122667666 | 122688018 | LRRC43 | | | | | |
| 12 | 122688227 | 122692084 | B3GNT4 | | | | | |
| 12 | 122692208 | 122712081 | DIABLO | | | DIABLO | | |
| 12 | 122710988 | 122713339 | LOC101593348 | | | | | |
| 12 | 122716093 | 122751068 | VPS33A | | | | | |
| 12 | 122755980 | 122884491 | CLIP1 | | | | | |
| 12 | 122956145 | 122985620 | ZCCHC8 | | | | | |
| 12 | 122989189 | 123011560 | RSRC2 | | | | | |
| 12 | 123011808 | 123110947 | KNTC1 | | | KNTC1 | | |
| 12 | 123185839 | 123187904 | HCAR2 | | | | | |
| 12 | 123199302 | 123201439 | HCAR3 | | | | | |
| 12 | 123212152 | 123215129 | HCAR1 | | | | | |
| 12 | 123237367 | 123255611 | DENR | | | | | |

| | | | | | | | | |
|---|---|---|---|---|---|---|---|---|
| 12 | 123259055 | 123311927 | CCDC62 | | | | | |
| 12 | 123319261 | 123347507 | HIP1R | | | | | |
| 12 | 123349874 | 123380712 | VPS37B | | | | | |
| 12 | 123405497 | 123451056 | ABCB9 | | | | | |
| 12 | 123459249 | 123464588 | OGFOD2 | | | | | |
| 12 | 123464606 | 123467460 | ARL6IP4 | | | | | |
| 12 | 123468026 | 123595036 | PITPNM2 | | | | | |
| 12 | 123495213 | 123495275 | MIR4304 | | | | | |
| 12 | 123565930 | 123569291 | LOC100507091 | | | | | |
| 12 | 123640942 | 123717785 | MPHOSPH9 | | | | | |
| 12 | 123717843 | 123742506 | C12orf65 | | | | | |
| 12 | 123745516 | 123756863 | CDK2AP1 | | | | | |
| 12 | 123773655 | 123834988 | SBNO1 | | | | | |
| 12 | 123849310 | 123849390 | MIR8072 | | | | | |
| 12 | 123868703 | 123893900 | KMT5A | | | | | |
| 12 | 123899585 | 123921280 | RILPL2 | | | | | |
| 12 | 123942650 | 123950941 | SNRNP35 | | | | | |
| 12 | 123955634 | 124018265 | RILPL1 | | | | | |
| 12 | 124020955 | 124021081 | MIR3908 | | | | | |
| 12 | 124065612 | 124068874 | LOC101927415 | | | | | |
| 12 | 124069075 | 124082688 | TMED2 | | | | | |
| 12 | 124086623 | 124105490 | DDX55 | | | | | |
| 12 | 124105569 | 124118323 | EIF2B1 | | | | | |
| 12 | 124118285 | 124147151 | GTF2H3 | | | | | |
| 12 | 124155659 | 124192950 | TCTN2 | | | | | |
| 12 | 124196864 | 124246301 | ATP6V0A2 | | | | | |
| 12 | 124247041 | 124420267 | DNAH10 | | | | | |
| 12 | 124420955 | 124457532 | CCDC92 | | | | | |
| 12 | 124457761 | 124499986 | ZNF664 | | | | | |
| 12 | 124457761 | 124800570 | ZNF664-FAM101A | | | | | |
| 12 | 124773709 | 124800570 | FAM101A | | | | | |
| 12 | 124808956 | 125052010 | NCOR2 | | | | | |
| 12 | 124821726 | 124821788 | MIR6880 | | | | | |
| 12 | 125262173 | 125348519 | SCARB1 | | | | | |
| 12 | 125396190 | 125399587 | UBC | | | | | |
| 12 | 125400092 | 125400205 | MIR5188 | | | | | |
| 12 | 125431369 | 125473667 | DHX37 | | | | | |
| 12 | 125478193 | 125510349 | BRI3BP | | | | | |
| 12 | 125509988 | 125511969 | THRIL | | | | | |
| 12 | 125549912 | 125627879 | AACS | | | | | |
| 12 | 125811161 | 126146923 | TMEM132B | | | | | |
| 12 | 126443233 | 126467920 | LINC00939 | | | | | |
| 12 | 126580537 | 126588478 | LOC101927464 | | | | | |
| 12 | 126927026 | 126957331 | LOC100128554 | | | | | |
| 12 | 127137491 | 127174864 | LOC100996671 | | | | | |
| 12 | 127215246 | 127256808 | LINC00944 | | | | | |
| 12 | 127221552 | 127230800 | LINC00943 | | | | | |
| 12 | 127354039 | 127359236 | LOC440117 | | | | | |
| 12 | 127399773 | 127544942 | LOC101927592 | | | | | |
| 12 | 127808699 | 127824617 | LOC101927616 | | | | | |
| 12 | 128115813 | 128121012 | LOC101927637 | | | | | |
| 12 | 128133440 | 128140133 | LOC105370068 | | | | | |
| 12 | 128366161 | 128383184 | FLJ37505 | | | | | |
| 12 | 128368534 | 128468436 | LINC00508 | | | | | |
| 12 | 128399954 | 128436097 | LINC00507 | | | | | |
| 12 | 128508332 | 128511711 | CRAT8 | | | | | |
| 12 | 128571527 | 128602686 | LOC100996679 | | | | | |
| 12 | 128602761 | 128606497 | LOC101927694 | | | | | |
| 12 | 128729050 | 128729118 | MIR4419B | | | | | |
| 12 | 128751947 | 129192460 | TMEM132C | | | | | |
| 12 | 128778636 | 128778723 | MIR3612 | | | | | |
| 12 | 129277738 | 129308541 | SLC15A4 | | | | | |
| 12 | 129337978 | 129469509 | GLT1D1 | | | | | |
| 12 | 129556270 | 130388212 | TMEM132D | | | | | |
| 12 | 129594234 | 129597843 | LOC283352 | | | | | |
| 12 | 129693145 | 129697205 | LOC101927735 | | | | | |

| | | | | | | | | |
|---|---|---|---|---|---|---|---|---|
| 12 | 130518356 | 130526887 | LOC100190940 | | | | | |
| 12 | 130636137 | 130650285 | FZD10 | | | | | |
| 12 | 130822432 | 130856877 | PIWIL1 | | | | | |
| 12 | 130880680 | 131002410 | RIMBP2 | | | | | |
| 12 | 131274144 | 131323819 | STX2 | | | STX2 | | |
| 12 | 131356538 | 131362223 | RAN | | | RAN | | |
| 12 | 131438451 | 131626008 | ADGRD1 | | | | | |
| 12 | 131475170 | 131478521 | LACAT8 | | | | | |
| 12 | 131649555 | 131697476 | LINC01257 | | | | | |
| 12 | 131780657 | 131782517 | LOC107161159 | | | | | |
| 12 | 131832014 | 131852100 | LOC338797 | | | | | |
| 12 | 132195631 | 132284283 | SFSWAP | | | | | |
| 12 | 132312937 | 132336316 | MMP17 | | | | | |
| 12 | 132379278 | 132407707 | ULK1 | | | ULK1 | | |
| 12 | 132413744 | 132428406 | PUS1 | | | | | |
| 12 | 132434464 | 132565011 | EP400 | | | EP400 | | |
| 12 | 132515768 | 132515905 | SNORA49 | | | | | |
| 12 | 132568827 | 132610885 | EP400NL | | | | | |
| 12 | 132621139 | 132628880 | DDX51 | | | | | |
| 12 | 132628971 | 132637018 | NOC4L | | | | | |
| 12 | 132680916 | 132905905 | GALNT9 | | | | | |
| 12 | 132851976 | 132857486 | LOC100130238 | | | | | |
| 12 | 132906435 | 132908161 | LOC101928416 | | | | | |
| 12 | 133067156 | 133161773 | FBRSL1 | | | | | |
| 12 | 133158582 | 133158647 | MIR6763 | | | | | |
| 12 | 133179735 | 133187037 | LRCOL1 | | | | | |
| 12 | 133195365 | 133198972 | P2RX2 | | | | P2RX2 | |
| 12 | 133200347 | 133264110 | POLE | | | POLE | | |
| 12 | 133264191 | 133281577 | PXMP2 | | | | | |
| 12 | 133287392 | 133299323 | PGAM5 | | | | | |
| 12 | 133302252 | 133338474 | ANKLE2 | | | | | |
| 12 | 133345494 | 133405426 | GOLGA3 | | | | | |
| 12 | 133416937 | 133464204 | CHFR | | | | | |
| 12 | 133464427 | 133465157 | LOC101928530 | | | | | |
| 12 | 133498018 | 133532892 | ZNF605 | | | | | |
| 12 | 133562933 | 133589154 | ZNF26 | | | | | |
| 12 | 133609244 | 133613808 | LOC101928597 | | | | | |
| 12 | 133613871 | 133639890 | ZNF84 | | | | | |
| 12 | 133656788 | 133684258 | ZNF140 | | | | | |
| 12 | 133696554 | 133707059 | ZNF891 | | | | | |
| 12 | 133707213 | 133736049 | ZNF10 | | | | | |
| 12 | 133757994 | 133783697 | ZNF268 | | | | | |
| 12 | 133794897 | 133812422 | ANHX | | | | | |
| 13 | 19312239 | 19314239 | LINC00417 | | | | | |
| 13 | 19408542 | 19446109 | ANKRD20A9P | | | | | |
| 13 | 19479578 | 19500881 | LINC00408 | | | | | |
| 13 | 19582398 | 19586774 | LINC00442 | | | | | |
| 13 | 19747909 | 19755992 | TUBA3C | | | | | |
| 13 | 19756172 | 19761605 | LOC101928697 | | | | | |
| 13 | 19836939 | 19919113 | ANKRD26P3 | | | | | |
| 13 | 19919188 | 19920889 | LINC00421 | | | | | |
| 13 | 19997018 | 20135714 | TPTE2 | | | | | |
| 13 | 20161257 | 20162630 | LINC00350 | | | | | |
| 13 | 20207787 | 20247599 | MPHOSPH8 | | | | | |
| 13 | 20248891 | 20357159 | PSPC1 | | | | | |
| 13 | 20397623 | 20437776 | ZMYM5 | | | | | |
| 13 | 20532809 | 20665984 | ZMYM2 | | | ZMYM2 | | |
| 13 | 20676839 | 20677369 | LINC01072 | | | | | |
| 13 | 20712394 | 20735183 | GJA3 | | | GJA3 | | |
| 13 | 20761603 | 20767114 | GJB2 | | | GJB2 | | |
| 13 | 20796100 | 20806534 | GJB6 | | GJB6 | | | |
| 13 | 20977805 | 21100012 | CRYL1 | | | | | |
| 13 | 21007916 | 21007985 | MIR4499 | | | | | |
| 13 | 21140954 | 21265576 | IFT88 | | | | | |
| 13 | 21277481 | 21297237 | IL17D | | | | | |
| 13 | 21303069 | 21348097 | EEF1AKMT1 | | | | | |

| | | | | | | | | |
|---|---|---|---|---|---|---|---|---|
| 13 | 21351467 | 21476913 | XPO4 | | | | | |
| 13 | 21512702 | 21523567 | LINC00367 | | | | | |
| 13 | 21547175 | 21635722 | LATS2 | | | LATS2 | | |
| 13 | 21714652 | 21723224 | SAP18 | | | | | |
| 13 | 21727733 | 21750741 | SKA3 | | | | | |
| 13 | 21750371 | 21753220 | MRPL57 | | | | | |
| 13 | 21808706 | 21809343 | LINC01046 | | | | | |
| 13 | 21872263 | 21967061 | MIPEPP3 | | | | | |
| 13 | 21877650 | 21918998 | LINC00539 | | | | | |
| 13 | 21946709 | 22033509 | ZDHHC20 | | | | | |
| 13 | 22066827 | 22178355 | MICU2 | | | | | |
| 13 | 22245214 | 22278640 | FGF9 | | FGF9 | | | |
| 13 | 22446934 | 22452299 | LINC00424 | | | | | |
| 13 | 22784423 | 22850659 | LINC00540 | | | | | |
| 13 | 23471168 | 23472320 | BASP1P1 | | | | | |
| 13 | 23755059 | 23899304 | SGCG | | | | | |
| 13 | 23902961 | 24003008 | SACS | | SACS | | | |
| 13 | 24043650 | 24061603 | LINC00327 | | | | | |
| 13 | 24144508 | 24250244 | TNFRSF19 | | | | | |
| 13 | 24304327 | 24463587 | MIPEP | | | MIPEP | | |
| 13 | 24463027 | 24471641 | C1QTNF9B | | | | | |
| 13 | 24481422 | 24523454 | ANKRD20A19P | | | | | |
| 13 | 24553764 | 24881212 | SPATA13 | | | | | |
| 13 | 24736554 | 24736643 | MIR2276 | | | | | |
| 13 | 24826886 | 24881212 | SPATA13 | | | | | |
| 13 | 24881303 | 24896669 | C1QTNF9 | | | | | |
| 13 | 24905587 | 24911258 | LINC00566 | | | | | |
| 13 | 24995068 | 25086948 | PARP4 | | | | | |
| 13 | 25154345 | 25171812 | TPTE2P6 | | | | | |
| 13 | 25254548 | 25285923 | ATP12A | | | | | |
| 13 | 25338300 | 25454058 | RNF17 | | | | | |
| 13 | 25456411 | 25497027 | CENPJ | | | | | |
| 13 | 25502892 | 25542607 | TPTE2P1 | | | | | |
| 13 | 25670275 | 25672704 | PABPC3 | | | | | |
| 13 | 25735816 | 25746421 | AMER2 | | | | | |
| 13 | 25746961 | 25754139 | LINC00463 | | | | | |
| 13 | 25755469 | 25764795 | LINC01053 | | | | | |
| 13 | 25820340 | 25861704 | MTMR6 | | | | | |
| 13 | 25875665 | 25916561 | NUP58 | | | | | |
| 13 | 25946148 | 26599989 | ATP8A2 | | ATP8A2 | | | |
| 13 | 26618734 | 26625198 | SHISA2 | | | | | |
| 13 | 26706252 | 26796508 | RNF6 | | | | | |
| 13 | 26828240 | 26979373 | CDK8 | | | | | |
| 13 | 27131839 | 27263082 | WASF3 | | | WASF3 | | |
| 13 | 27329338 | 27334922 | GPR12 | | | | | |
| 13 | 27640286 | 27757640 | USP12 | | | | | |
| 13 | 27810418 | 27811394 | LINC00412 | | | | | |
| 13 | 27825691 | 27830702 | RPL21 | | | | | |
| 13 | 27825692 | 27830699 | RPL21P28 | | | | | |
| 13 | 27829200 | 27829272 | SNORD102 | | | | | |
| 13 | 27829537 | 27829663 | SNORA27 | | | | | |
| 13 | 27844463 | 27847827 | RASL11A | | | | | |
| 13 | 27998680 | 28009846 | GTF3A | | | | | |
| 13 | 28009775 | 28024739 | MTIF3 | | | | | |
| 13 | 28120049 | 28194720 | LNX2 | | | | | |
| 13 | 28194879 | 28241559 | POLR1D | | | | | |
| 13 | 28366779 | 28368089 | GSX1 | | | | | |
| 13 | 28403895 | 28500451 | PDX1 | | | PDX1 | | |
| 13 | 28519342 | 28519710 | ATP5EP2 | | | | | |
| 13 | 28536204 | 28543505 | CDX2 | | | | | |
| 13 | 28552242 | 28562774 | URAD | | | | | |
| 13 | 28577410 | 28674729 | FLT3 | | FLT3 | | | |
| 13 | 28710979 | 28869475 | PAN3 | | | | | |
| 13 | 28874482 | 29069265 | FLT1 | | | FLT1 | | |
| 13 | 29233140 | 29253093 | POMP | | | | | |
| 13 | 29274217 | 29293150 | SLC46A3 | | | | | |

| | | | | | | | | |
|---|---|---|---|---|---|---|---|---|
| 13 | 29598747 | 30061887 | MTUS2 | | | | | |
| 13 | 30083550 | 30169825 | SLC7A1 | | | | | |
| 13 | 30338544 | 30424820 | UBL3 | | | | | |
| 13 | 30446749 | 30462542 | LINC00297 | | | | | |
| 13 | 30492783 | 30500788 | LINC00572 | | | | | |
| 13 | 30510667 | 30524625 | LINC00544 | | | | | |
| 13 | 30677314 | 30683012 | LINC00365 | | | | | |
| 13 | 30776766 | 30881624 | KATNAL1 | | | | | |
| 13 | 30914402 | 30948051 | LINC00426 | | | | | |
| 13 | 30993575 | 30996381 | LINC01058 | | | | | |
| 13 | 31032052 | 31191734 | HMGB1 | | | | | |
| 13 | 31191829 | 31233686 | USPL1 | | | | | |
| 13 | 31287614 | 31338565 | ALOX5AP | | | | | |
| 13 | 31377342 | 31384782 | LINC00398 | | | | | |
| 13 | 31456698 | 31457532 | LINC00545 | | | | | |
| 13 | 31456971 | 31506745 | TEX26 | | | | | |
| 13 | 31480311 | 31499709 | MEDAG | | | | | |
| 13 | 31504808 | 31549153 | TEX26 | | | | | |
| 13 | 31709110 | 31736525 | HSPH1 | | | | | |
| 13 | 31774111 | 31906411 | B3GLCT | | | | | |
| 13 | 32313678 | 32377009 | RXFP2 | | | | | |
| 13 | 32420919 | 32533721 | EEF1DP3 | | | | | |
| 13 | 32598195 | 32870776 | FRY | | | | | |
| 13 | 32877907 | 32886091 | ZAR1L | | | | | |
| 13 | 32889616 | 32973809 | BRCA2 | | | BRCA2 | | |
| 13 | 32974859 | 33002315 | N4BP2L1 | | | | | |
| 13 | 33006624 | 33113022 | N4BP2L2 | | | | | |
| 13 | 33070350 | 33072629 | MINOS1P1 | | | | | |
| 13 | 33078642 | 33083532 | N4BP2L2-IT2 | | | | | |
| 13 | 33091029 | 33112932 | N4BP2L2 | | | | | |
| 13 | 33160563 | 33352158 | PDS5B | | | | | |
| 13 | 33383327 | 33485790 | LINC00423 | | | | | |
| 13 | 33590570 | 33640282 | KL | | | | | |
| 13 | 33677271 | 33855471 | STARD13 | | | | | |
| 13 | 34392205 | 34540695 | RFC3 | | | | | |
| 13 | 35009590 | 35214822 | LINC00457 | | | | | |
| 13 | 35516423 | 36246873 | NBEA | | NBEA | | | NBEA |
| 13 | 36047925 | 36050832 | MAB21L1 | | | | | |
| 13 | 36048405 | 36515382 | MIR548F5 | | | | | |
| 13 | 36050885 | 36246874 | NBEA | | NBEA | | | NBEA |
| 13 | 36271660 | 36273392 | LINC00445 | | | | | |
| 13 | 36342788 | 36429998 | DCLK1 | | | DCLK1 | | |
| 13 | 36742344 | 36788752 | SOHLH2 | | | | | |
| 13 | 36742344 | 36871992 | CCDC169-SOHLH2 | | | | | |
| 13 | 36756128 | 36788752 | SOHLH2 | | | | | |
| 13 | 36801178 | 36871992 | CCDC169 | | | | | |
| 13 | 36875774 | 36943872 | SPG20 | | | SPG20 | | |
| 13 | 37005966 | 37017019 | CCNA1 | | | | | |
| 13 | 37248048 | 37271975 | SERTM1 | | | | | |
| 13 | 37393338 | 37403740 | RFXAP | | | | | |
| 13 | 37418967 | 37494409 | SMAD9 | | | | | |
| 13 | 37523907 | 37573504 | ALG5 | | | | | |
| 13 | 37574677 | 37583751 | EXOSC8 | | | | | |
| 13 | 37583450 | 37633850 | SUPT20H | | | | | |
| 13 | 37677396 | 37679801 | CSNK1A1L | | | CSNK1A1L | | |
| 13 | 38055600 | 38058921 | LINC01048 | | | | | |
| 13 | 38109076 | 38125673 | LINC00547 | | | | | |
| 13 | 38136718 | 38172981 | POSTN | | | | | |
| 13 | 38210772 | 38443939 | TRPC4 | | | | | |
| 13 | 38624953 | 38717369 | LINC00571 | | | | | |
| 13 | 38923907 | 38937144 | UFM1 | | | | | |
| 13 | 39106136 | 39260812 | LINC00437 | | | | | |
| 13 | 39141860 | 39153653 | LINC00366 | | | | | |
| 13 | 39261172 | 39461267 | FREM2 | | | | | |
| 13 | 39540061 | 39564996 | STOML3 | | | | | |
| 13 | 39584001 | 39612213 | PROSER1 | | | | | |

| | | | | | | | | |
|---|---|---|---|---|---|---|---|---|
| 13 | 39612447 | 39624244 | NHLRC3 | | | | | |
| 13 | 39917028 | 40177356 | LHFP | | | | | |
| 13 | 40229763 | 40365802 | COG6 | | | | | |
| 13 | 40238170 | 40238272 | MIR4305 | | | | | |
| 13 | 40755945 | 40763167 | LINC00332 | | | | | |
| 13 | 40768645 | 40794639 | LINC00548 | | | | | |
| 13 | 40921270 | 41055143 | LINC00598 | | | | | |
| 13 | 41129800 | 41240734 | FOXO1 | | | | | |
| 13 | 41301963 | 41302011 | MIR320D1 | | | | | |
| 13 | 41303431 | 41345347 | MRPS31 | | | | | |
| 13 | 41363546 | 41386596 | SLC25A15 | | | SLC25A15 | | |
| 13 | 41371120 | 41495886 | TPTE2P5 | | | | | |
| 13 | 41384901 | 41384997 | MIR621 | | | | | |
| 13 | 41486024 | 41495910 | SUGT1P3 | | | | | |
| 13 | 41506054 | 41593508 | ELF1 | | | | | |
| 13 | 41635696 | 41658139 | WBP4 | | | | | |
| 13 | 41675154 | 41675236 | MIR3168 | | | | | |
| 13 | 41701708 | 41706936 | KBTBD6 | | | | | |
| 13 | 41707063 | 41810822 | LOC101929140 | | | | | |
| 13 | 41765710 | 41768702 | KBTBD7 | | | | | |
| 13 | 41790515 | 41837713 | MTRF1 | | | MTRF1 | | |
| 13 | 41885340 | 41951166 | NAA16 | | | | | |
| 13 | 42016699 | 42017902 | OR7E37P | | | | | |
| 13 | 42031541 | 42045013 | RGCC | | | | | |
| 13 | 42140960 | 42535221 | VWA8 | | | | | |
| 13 | 42142421 | 42142531 | MIR5006 | | | | | |
| 13 | 42293472 | 42555701 | VWA8 | | | | | |
| 13 | 42614171 | 42830716 | DGKH | | | | | |
| 13 | 42846288 | 42897403 | AKAP11 | | | AKAP11 | | |
| 13 | 43136871 | 43182149 | TNFSF11 | | | | | |
| 13 | 43355685 | 43365686 | FAM216B | | | | | |
| 13 | 43384501 | 43386698 | LINC01050 | | | | | |
| 13 | 43416547 | 43446880 | LINC00428 | | | | | |
| 13 | 43460523 | 43566407 | EPSTI1 | | | | | |
| 13 | 43597361 | 43683306 | DNAJC15 | | | | | |
| 13 | 43687300 | 43733602 | LINC00400 | | | | | |
| 13 | 43787665 | 44033624 | ENOX1 | | | | | |
| 13 | 44410488 | 44453826 | CCDC122 | | | | | |
| 13 | 44453419 | 44468068 | LACC1 | | | | | |
| 13 | 44596470 | 44604599 | LINC00284 | | | | | |
| 13 | 44680226 | 44721871 | LINC00390 | | | | | |
| 13 | 44684684 | 44735393 | SMIM2 | | | | | |
| 13 | 44720605 | 44732358 | SMIM2-IT1 | | | | | |
| 13 | 44770264 | 44770336 | MIR8079 | | | | | |
| 13 | 44947977 | 44971850 | SERP2 | | | | | |
| 13 | 44974385 | 44980120 | TUSC8 | | | | | |
| 13 | 45006278 | 45154568 | TSC22D1 | | | | | |
| 13 | 45373639 | 45383766 | LINC00330 | | | | | |
| 13 | 45513383 | 45563613 | NUFIP1 | | NUFIP1 | | | NUFIP1 |
| 13 | 45563664 | 45607743 | GPALPP1 | | | | | |
| 13 | 45680413 | 45687341 | LOC101929259 | | | | | |
| 13 | 45694630 | 45858239 | GTF2F2 | | | | | |
| 13 | 45766987 | 45775175 | KCTD4 | | | | | |
| 13 | 45907605 | 45915419 | TPT1 | | | | | |
| 13 | 45911614 | 45911744 | SNORA31 | | | | | |
| 13 | 45915479 | 45965618 | TPT1 | | | | | |
| 13 | 45967453 | 45994506 | SLC25A30 | | | | | |
| 13 | 46039029 | 46110833 | COG3 | | | COG3 | | |
| 13 | 46115431 | 46189874 | ERICH6B | | | | | |
| 13 | 46255333 | 46275319 | LINC01055 | | | | | |
| 13 | 46276438 | 46288694 | SPERT | | | | | |
| 13 | 46354415 | 46425846 | SIAH3 | | | | | |
| 13 | 46536313 | 46626896 | ZC3H13 | | | ZC3H13 | | |
| 13 | 46626982 | 46679211 | CPB2 | | | | | |
| 13 | 46700057 | 46756459 | LCP1 | | | | | |
| 13 | 46786077 | 46850938 | LRRC63 | | | | | |

| | | | | | | | | |
|---|---|---|---|---|---|---|---|---|
| 13 | 46870579 | 46871979 | LINC00563 | | | | | |
| 13 | 46916134 | 46964177 | KIAA0226L | | | | | |
| 13 | 47029338 | 47040911 | LINC01198 | | | | | |
| 13 | 47127295 | 47327175 | LRCH1 | | | | | |
| 13 | 47345390 | 47371367 | ESD | | | | | |
| 13 | 47405676 | 47430434 | HTR2A | | HTR2A | HTR2A | HTR2A | |
| 13 | 48504287 | 48506757 | LINC00562 | | | | | |
| 13 | 48516790 | 48575462 | SUCLA2 | | SUCLA2 | | | |
| 13 | 48611702 | 48621358 | NUDT15 | | | | | |
| 13 | 48649863 | 48654129 | MED4 | | | | | |
| 13 | 48807273 | 48836232 | ITM2B | | | | | |
| 13 | 48870648 | 48877797 | LINC00441 | | | | | |
| 13 | 48877882 | 49056026 | RB1 | | | RB1 | | |
| 13 | 48985181 | 49018840 | LPAR6 | | | | | |
| 13 | 49063098 | 49107392 | RCBTB2 | | | | | |
| 13 | 49151109 | 49155037 | LINC00462 | | | | | |
| 13 | 49227846 | 49283498 | CYSLTR2 | | | | | |
| 13 | 49550047 | 49783915 | FNDC3A | | | | | |
| 13 | 49794473 | 49796513 | MLNR | | | | | |
| 13 | 49822046 | 49867622 | CDADC1 | | | | | |
| 13 | 49882785 | 50018262 | CAB39L | | | | | |
| 13 | 50018428 | 50069139 | SETDB2 | | | | | |
| 13 | 50069800 | 50103117 | PHF11 | | | | | |
| 13 | 50106081 | 50159719 | RCBTB1 | | RCBTB1 | | | |
| 13 | 50202434 | 50208008 | ARL11 | | | | | |
| 13 | 50234811 | 50265623 | EBPL | | | | | |
| 13 | 50273442 | 50367057 | KPNA3 | | | | | |
| 13 | 50464544 | 50467516 | CTAGE10P | | | | | |
| 13 | 50486837 | 50510637 | SPRYD7 | | | | | |
| 13 | 50556687 | 50699677 | DLEU2 | | | | | |
| 13 | 50570550 | 50570637 | MIR3613 | | | | | |
| 13 | 50571142 | 50592603 | TRIM13 | | | TRIM13 | | |
| 13 | 50589389 | 50595058 | KCNRG | | | | | |
| 13 | 50623108 | 50623197 | MIR16 | | | | | |
| 13 | 50623254 | 50623337 | MIR15A | | | | | |
| 13 | 50656304 | 51102779 | DLEU1 | | | | | |
| 13 | 50746153 | 50747751 | ST13P4 | | | | | |
| 13 | 51095068 | 51101585 | DLEU1 | | | | | |
| 13 | 51285161 | 51418075 | DLEU7 | | | | | |
| 13 | 51456514 | 51544596 | RNASEH2B | | | | | |
| 13 | 51568646 | 51640293 | GUCY1B2 | | | | | |
| 13 | 51656983 | 51746524 | LINC00371 | | | | | |
| 13 | 51796469 | 51858377 | FAM124A | | | | | |
| 13 | 51915167 | 51936239 | SERPINE3 | | | | | |
| 13 | 51922702 | 51922775 | MIR5693 | | | | | |
| 13 | 51935700 | 52028851 | INTS6 | | | | | |
| 13 | 52126724 | 52126803 | MIR4703 | | | | | |
| 13 | 52158483 | 52336171 | WDFY2 | | | | | |
| 13 | 52342128 | 52378298 | DHRS12 | | | | | |
| 13 | 52387482 | 52419286 | LINC00282 | | | | | |
| 13 | 52436116 | 52440372 | CCDC70 | | | | | |
| 13 | 52506805 | 52585630 | ATP7B | | | ATP7B | | |
| 13 | 52586522 | 52603780 | ALG11 | | | | | |
| 13 | 52598826 | 52607736 | UTP14C | | | | | |
| 13 | 52638899 | 52703228 | NEK5 | | | | | |
| 13 | 52703030 | 52706859 | LOC101929657 | | | | | |
| 13 | 52706778 | 52733996 | NEK3 | | | | | |
| 13 | 52741844 | 52768602 | MRPS31P5 | | | | | |
| 13 | 52908429 | 52916018 | LOC103191607 | | | | | |
| 13 | 52951302 | 52980629 | THSD1 | | | | | |
| 13 | 52986736 | 53024813 | VPS36 | | | VPS36 | | |
| 13 | 53029494 | 53050766 | CKAP2 | | | | | |
| 13 | 53063127 | 53161225 | TPTE2P3 | | | | | |
| 13 | 53191604 | 53217919 | HNRNPA1L2 | | | | | |
| 13 | 53226830 | 53262433 | SUGT1 | | | | | |
| 13 | 53277399 | 53313947 | LECT1 | | | | | |

| | | | | | | | | |
|---|---|---|---|---|---|---|---|---|
| 13 | 53384184 | 53384275 | MIR759 | | | | | |
| 13 | 53418108 | 53422775 | PCDH8 | | | | | |
| 13 | 53602875 | 53626196 | OLFM4 | | | | | |
| 13 | 53705880 | 53726035 | LINC01065 | | | | | |
| 13 | 54389553 | 54450254 | LINC00558 | | | | | |
| 13 | 54698458 | 54707006 | LINC00458 | | | | | |
| 13 | 54886106 | 54886183 | MIR1297 | | | | | |
| 13 | 55748588 | 55748683 | MIR5007 | | | | | |
| 13 | 57715051 | 57718073 | PRR20A | | | | | |
| 13 | 57715051 | 57718073 | PRR20B | | | | | |
| 13 | 57715051 | 57718073 | PRR20C | | | | | |
| 13 | 57715051 | 57718073 | PRR20D | | | | | |
| 13 | 57715051 | 57718073 | PRR20E | | | | | |
| 13 | 57721621 | 57724643 | PRR20A | | | | | |
| 13 | 57721621 | 57724643 | PRR20B | | | | | |
| 13 | 57721621 | 57724643 | PRR20C | | | | | |
| 13 | 57721621 | 57724643 | PRR20D | | | | | |
| 13 | 57721621 | 57724643 | PRR20E | | | | | |
| 13 | 57728194 | 57731216 | PRR20A | | | | | |
| 13 | 57728194 | 57731216 | PRR20B | | | | | |
| 13 | 57728194 | 57731216 | PRR20C | | | | | |
| 13 | 57728194 | 57731216 | PRR20D | | | | | |
| 13 | 57728194 | 57731216 | PRR20E | | | | | |
| 13 | 57734765 | 57737787 | PRR20A | | | | | |
| 13 | 57734765 | 57737787 | PRR20B | | | | | |
| 13 | 57734765 | 57737787 | PRR20C | | | | | |
| 13 | 57734765 | 57737787 | PRR20D | | | | | |
| 13 | 57734765 | 57737787 | PRR20E | | | | | |
| 13 | 57741330 | 57744352 | PRR20A | | | | | |
| 13 | 57741330 | 57744352 | PRR20B | | | | | |
| 13 | 57741330 | 57744352 | PRR20C | | | | | |
| 13 | 57741330 | 57744352 | PRR20D | | | | | |
| 13 | 57741330 | 57744352 | PRR20E | | | | | |
| 13 | 58205788 | 58303065 | PCDH17 | | | | | |
| 13 | 58739960 | 58783617 | LOC101926897 | | | | | |
| 13 | 58785830 | 58807251 | LINC00374 | | | | | |
| 13 | 60239720 | 60727639 | DIAPH3 | | | | | |
| 13 | 60788480 | 60842238 | LINC00434 | | | | | |
| 13 | 60970590 | 61148013 | TDRD3 | | | | | |
| 13 | 61247088 | 61269934 | LINC00378 | | | | | |
| 13 | 61773931 | 61774014 | MIR3169 | | | | | |
| 13 | 61983818 | 61989655 | PCDH20 | | | | | |
| 13 | 61998821 | 62002079 | LOC101926951 | | | | | |
| 13 | 62577657 | 62603681 | LINC00358 | | | | | |
| 13 | 62786414 | 62824084 | LINC01075 | | | | | |
| 13 | 63246417 | 63381492 | LINC00448 | | | | | |
| 13 | 63757234 | 63902227 | LINC00376 | | | | | |
| 13 | 64241814 | 64312151 | LINC00395 | | | | | |
| 13 | 64311567 | 64316701 | OR7E156P | | | | | |
| 13 | 64402972 | 64418258 | LOC102723968 | | | | | |
| 13 | 66440178 | 66452351 | LINC01052 | | | | | |
| 13 | 66540461 | 66540561 | MIR548X2 | | | | | |
| 13 | 66792381 | 66792456 | MIR4704 | | | | | |
| 13 | 66876965 | 67804468 | PCDH9 | | PCDH9 | PCDH9 | | |
| 13 | 67946518 | 67954126 | LINC00364 | | | | | |
| 13 | 69435416 | 69459457 | LINC00550 | | | | | |
| 13 | 69796477 | 69896231 | LINC00383 | | | | | |
| 13 | 70274724 | 70682625 | KLHL1 | | | | | |
| 13 | 70681344 | 70713885 | ATXN8OS | | | | | |
| 13 | 71589272 | 71742549 | LINC00348 | | | | | |
| 13 | 72012097 | 72441330 | DACH1 | | | DACH1 | | |
| 13 | 73282494 | 73301938 | MZT1 | | | | | |
| 13 | 73301886 | 73330336 | BORA | | | | | |
| 13 | 73329539 | 73356266 | DIS3 | | | | | |
| 13 | 73356229 | 73590591 | PIBF1 | | | | | |
| 13 | 73629113 | 73651680 | KLF5 | | | | | |

| | | | | | | | | |
|---|---|---|---|---|---|---|---|---|
| 13 | 74138380 | 74162016 | LINC00392 | | | | | |
| 13 | 74260148 | 74708066 | KLF12 | | | KLF12 | | |
| 13 | 74993309 | 75009296 | LINC00381 | | | | | |
| 13 | 75126979 | 75131257 | LINC00347 | | | | | |
| 13 | 75811888 | 75814517 | CTAGE11P | | | | | |
| 13 | 75814723 | 75826148 | LINC01078 | | | | | |
| 13 | 75858799 | 76056304 | TBC1D4 | | | | | |
| 13 | 76099349 | 76123575 | COMMD6 | | | | | |
| 13 | 76123615 | 76180156 | UCHL3 | | | | | |
| 13 | 76178835 | 76434006 | LMO7 | | | | | |
| 13 | 76445173 | 76457948 | LMO7DN | | | | | |
| 13 | 76451021 | 76455261 | LMO7DN-IT1 | | | | | |
| 13 | 77454303 | 77460540 | KCTD12 | | | | | |
| 13 | 77502584 | 77503224 | BTF3P11 | | | | | |
| 13 | 77526623 | 77532776 | IRG1 | | | | | |
| 13 | 77566058 | 77576652 | CLN5 | | | | | |
| 13 | 77579388 | 77601331 | FBXL3 | | | | | |
| 13 | 77618791 | 77661911 | MYCBP2 | | | MYCBP2 | | |
| 13 | 78109808 | 78180686 | SCEL | | | | | |
| 13 | 78235508 | 78236938 | LOC100129307 | | | | | |
| 13 | 78271988 | 78338377 | SLAIN1 | | | | | |
| 13 | 78272146 | 78272251 | MIR3665 | | | | | |
| 13 | 78273015 | 78338377 | SLAIN1 | | | | | |
| 13 | 78393071 | 78549664 | EDNRB | | EDNRB | EDNRB | | |
| 13 | 78553900 | 78568908 | LINC01069 | | | | | |
| 13 | 78587017 | 78627730 | LINC00446 | | | | | |
| 13 | 78628989 | 79191460 | RNF219 | | | | | |
| 13 | 79173229 | 79177695 | POU4F1 | | | POU4F1 | | |
| 13 | 79188420 | 79233314 | RNF219 | | | | | |
| 13 | 79361453 | 79414185 | LINC00331 | | | | | |
| 13 | 79893002 | 79998468 | RBM26 | | | | | |
| 13 | 80051498 | 80130212 | NDFIP2 | | | NDFIP2 | | |
| 13 | 80140861 | 80145581 | LINC01068 | | | | | |
| 13 | 80378520 | 80379817 | LINC01038 | | | | | |
| 13 | 80446720 | 80492171 | LINC00382 | | | | | |
| 13 | 80585211 | 80600798 | LINC01080 | | | | | |
| 13 | 80910110 | 80915250 | SPRY2 | | | SPRY2 | | |
| 13 | 81592525 | 81618577 | LINC00377 | | | | | |
| 13 | 81800000 | 81801118 | LINC00564 | | | | | |
| 13 | 84451339 | 84456528 | SLITRK1 | | SLITRK1 | | | |
| 13 | 84714736 | 85180903 | LINC00333 | | | | | |
| 13 | 85357788 | 85357913 | SNORA107 | | | | | |
| 13 | 85639221 | 85722192 | LINC00375 | | | | | |
| 13 | 85937737 | 86118797 | LINC00351 | | | | | |
| 13 | 86366921 | 86373483 | SLITRK6 | | | | | |
| 13 | 87564174 | 87589062 | LINC00430 | | | | | |
| 13 | 88096241 | 88323218 | MIR4500HG | | | | | |
| 13 | 88270919 | 88270995 | MIR4500 | | | | | |
| 13 | 88324869 | 88331870 | SLITRK5 | | | | | |
| 13 | 88453297 | 88462775 | LINC00397 | | | | | |
| 13 | 88795121 | 88888337 | LOC105370306 | | | | | |
| 13 | 89193083 | 89197764 | LINC00433 | | | | | |
| 13 | 89867101 | 89889144 | LINC01047 | | | | | |
| 13 | 89887544 | 89932494 | LINC00440 | | | | | |
| 13 | 90129860 | 90152763 | LINC01040 | | | | | |
| 13 | 90201047 | 90216667 | LINC00353 | | | | | |
| 13 | 90712500 | 90771971 | LINC00559 | | | | | |
| 13 | 90883435 | 90883531 | MIR622 | | | | | |
| 13 | 91145541 | 91187595 | LINC01049 | | | | | |
| 13 | 91543207 | 91578851 | LINC00410 | | | | | |
| 13 | 91739508 | 91743693 | LINC00380 | | | | | |
| 13 | 91779866 | 91863952 | LINC00379 | | | | | |
| 13 | 92000073 | 92006829 | MIR17HG | | | | | |
| 13 | 92002858 | 92002942 | MIR17 | | | | | |
| 13 | 92003004 | 92003075 | MIR18A | | | | | |
| 13 | 92003144 | 92003226 | MIR19A | | | | | |

| | | | | | | | | |
|---|---|---|---|---|---|---|---|---|
| 13 | 92003318 | 92003389 | MIR20A | | | | | |
| 13 | 92003445 | 92003532 | MIR19B1 | | | | | |
| 13 | 92003567 | 92003645 | MIR92A1 | | | | | |
| 13 | 92050872 | 93372227 | GPC5 | | GPC5 | GPC5 | | |
| 13 | 93697593 | 93710179 | LINC00363 | | | | | |
| 13 | 93879077 | 94840245 | GPC6 | | | GPC6 | | |
| 13 | 95091840 | 95131936 | DCT | | | | | |
| 13 | 95226307 | 95248529 | TGDS | | | | | |
| 13 | 95254103 | 95286899 | GPR180 | | | | | |
| 13 | 95356997 | 95360740 | LOC101927248 | | | | | |
| 13 | 95361878 | 95368199 | SOX21 | | | | | |
| 13 | 95413194 | 95587664 | LOC101927284 | | | | | |
| 13 | 95612294 | 95613573 | LINC00557 | | | | | |
| 13 | 95672082 | 95953700 | ABCC4 | | | | | |
| 13 | 96085852 | 96232010 | CLDN10 | | CLDN10 | | | |
| 13 | 96230455 | 96296960 | DZIP1 | | | | | |
| 13 | 96325091 | 96447243 | DNAJC3 | | | | | |
| 13 | 96453835 | 96705736 | UGGT2 | | | | | |
| 13 | 96743092 | 97491816 | HS6ST3 | | | | | |
| 13 | 97079482 | 97079546 | MIR4501 | | | | | |
| 13 | 97593534 | 97636426 | LINC00359 | | | | | |
| 13 | 97637972 | 97646604 | OXGR1 | | | | | |
| 13 | 97824635 | 97831876 | LINC00456 | | | | | |
| 13 | 97874542 | 98046374 | MBNL2 | | | | | |
| 13 | 98086474 | 98120252 | RAP2A | | | | | |
| 13 | 98605928 | 98676550 | IPO5 | | | | | |
| 13 | 98794815 | 99102027 | FARP1 | | | | | |
| 13 | 98828038 | 98829521 | RNF113B | | | | | |
| 13 | 98860777 | 98860854 | MIR3170 | | | | | |
| 13 | 99102452 | 99229405 | STK24 | | | STK24 | | |
| 13 | 99336054 | 99404929 | SLC15A1 | | | | | |
| 13 | 99445740 | 99740879 | DOCK9 | | | | | |
| 13 | 99848627 | 100038753 | UBAC2 | | | | | |
| 13 | 99868261 | 100058554 | MIR548AN | | | | | |
| 13 | 99906966 | 99910682 | GPR18 | | | | | |
| 13 | 99946788 | 99959749 | GPR183 | | | | | |
| 13 | 100003673 | 100004281 | FKSG29 | | | | | |
| 13 | 100008384 | 100008482 | MIR623 | | | | | |
| 13 | 100139215 | 100151560 | LINC01232 | | | | | |
| 13 | 100151976 | 100153317 | LINC00449 | | | | | |
| 13 | 100153627 | 100216302 | TM9SF2 | | | TM9SF2 | | |
| 13 | 100229365 | 100238233 | LINC01039 | | | | | |
| 13 | 100258917 | 100561713 | CLYBL | | | | | |
| 13 | 100295312 | 100295403 | MIR4306 | | | | | |
| 13 | 100342351 | 100381255 | CLYBL | | | | | |
| 13 | 100393123 | 100609419 | LOC101927437 | | | | | |
| 13 | 100615274 | 100624178 | ZIC5 | | | | | |
| 13 | 100634025 | 100639019 | ZIC2 | | ZIC2 | ZIC2 | | |
| 13 | 100647153 | 100650163 | LINC00554 | | | | | |
| 13 | 100738284 | 100741197 | LOC105370333 | | | | | |
| 13 | 100741268 | 101133269 | PCCA | | | PCCA | | |
| 13 | 101182417 | 101241046 | GGACT | | | | | |
| 13 | 101256089 | 101327189 | TMTC4 | | | | | |
| 13 | 101360578 | 101711638 | NALCN | | | | | |
| 13 | 101591774 | 101596618 | LINC00411 | | | | | |
| 13 | 101706129 | 102068813 | NALCN | | | | | |
| 13 | 102104943 | 102368796 | ITGBL1 | | | | | |
| 13 | 102373204 | 103054124 | FGF14 | | FGF14 | | | |
| 13 | 102619991 | 102620096 | MIR2681 | | | | | |
| 13 | 102698283 | 102698354 | MIR4705 | | | | | |
| 13 | 102944669 | 103046869 | FGF14-IT1 | | | | | |
| 13 | 103019879 | 103048055 | FGF14 | | FGF14 | | | |
| 13 | 103249285 | 103331523 | TPP2 | | | | | |
| 13 | 103338096 | 103346871 | METTL21C | | | | | |
| 13 | 103381716 | 103411422 | CCDC168 | | | | | |
| 13 | 103418237 | 103426171 | TEX30 | | | | | |

| | | | | | | | | |
|---|---|---|---|---|---|---|---|---|
| 13 | 103436630 | 103451404 | KDELC1 | | | | | |
| 13 | 103451398 | 103493888 | BIVM | | | | | |
| 13 | 103459495 | 103528351 | BIVM-ERCC5 | | | | | |
| 13 | 103498190 | 103528351 | ERCC5 | | | ERCC5 | | |
| 13 | 103532448 | 103548383 | METTL21EP | | | | | |
| 13 | 103696347 | 103719196 | SLC10A2 | | | | | |
| 13 | 103934828 | 103934848 | MIR548AS | | | | | |
| 13 | 104077549 | 104080035 | LINC01309 | | | | | |
| 13 | 106111405 | 106143383 | DAOA | | | | | |
| 13 | 106359178 | 106414145 | LINC00343 | | | | | |
| 13 | 107028910 | 107030142 | LINC00460 | | | | | |
| 13 | 107142078 | 107187388 | EFNB2 | | | | | |
| 13 | 107195661 | 107220514 | ARGLU1 | | | | | |
| 13 | 107270157 | 107284006 | LINC00551 | | | | | |
| 13 | 107306227 | 107324528 | LINC00443 | | | | | |
| 13 | 107820878 | 108519460 | FAM155A | | | | | |
| 13 | 108183518 | 108183596 | MIR1267 | | | | | |
| 13 | 108439708 | 108487806 | FAM155A-IT1 | | | | | |
| 13 | 108859791 | 108870716 | LIG4 | | LIG4 | LIG4 | | |
| 13 | 108870762 | 108886603 | ABHD13 | | | | | |
| 13 | 108921976 | 108960832 | TNFSF13B | | | | | |
| 13 | 109248499 | 109819651 | MYO16 | | | | | |
| 13 | 110053042 | 110053986 | LINC00399 | | | | | |
| 13 | 110380620 | 110382381 | LINC00676 | | | | | |
| 13 | 110406183 | 110438914 | IRS2 | | | | | |
| 13 | 110705631 | 110707299 | LINC00396 | | | | | |
| 13 | 110801304 | 110959504 | COL4A1 | | | | | |
| 13 | 110959630 | 111165373 | COL4A2 | | | | | |
| 13 | 110993304 | 110993376 | MIR8073 | | | | | |
| 13 | 111154922 | 111160526 | COL4A2 | | | | | |
| 13 | 111175412 | 111214084 | RAB20 | | | RAB20 | | |
| 13 | 111267806 | 111292342 | CARKD | | | | | |
| 13 | 111293756 | 111358527 | CARS2 | | | | | |
| 13 | 111364969 | 111373421 | ING1 | | | | | |
| 13 | 111516333 | 111522655 | LINC00346 | | | | | |
| 13 | 111530886 | 111567454 | ANKRD10 | | | | | |
| 13 | 111618373 | 111642949 | LINC00431 | | | | | |
| 13 | 111748184 | 111758237 | LINC00368 | | | | | |
| 13 | 111766158 | 111800912 | ARHGEF7 | | | | | |
| 13 | 111800543 | 111805924 | LOC101060553 | | | | | |
| 13 | 111806060 | 111958081 | ARHGEF7 | | | | | |
| 13 | 111968531 | 111996594 | TEX29 | | | | | |
| 13 | 112547691 | 112555490 | LINC00354 | | | | | |
| 13 | 112626623 | 112762329 | LINC00403 | | | | | |
| 13 | 112721912 | 112726020 | SOX1 | | | | | |
| 13 | 112761432 | 112762329 | LINC00403 | | | | | |
| 13 | 112809470 | 112815024 | LOC100506016 | | | | | |
| 13 | 112851646 | 112855316 | LINC01070 | | | | | |
| 13 | 112911039 | 112927314 | LOC101928730 | | | | | |
| 13 | 112976880 | 112985539 | LINC01044 | | | | | |
| 13 | 113030650 | 113089009 | SPACA7 | | | | | |
| 13 | 113139318 | 113242499 | TUBGCP3 | | | TUBGCP3 | | |
| 13 | 113301357 | 113338811 | ATP11AUN | | | | | |
| 13 | 113344642 | 113409045 | ATP11A | | | | | |
| 13 | 113621797 | 113754053 | MCF2L | | | MCF2L | | |
| 13 | 113760101 | 113774995 | F7 | | | | | |
| 13 | 113777112 | 113784360 | F10 | | | | | |
| 13 | 113812967 | 113826698 | PROZ | | | | | |
| 13 | 113831852 | 113863029 | PCID2 | | | | | |
| 13 | 113862506 | 113919392 | CUL4A | | | | | |
| 13 | 113917233 | 113917313 | MIR8075 | | | | | |
| 13 | 113951468 | 113977741 | LAMP1 | | | | | |
| 13 | 113978478 | 114016182 | GRTP1 | | | | | |
| 13 | 114053924 | 114068708 | LOC101928841 | | | | | |
| 13 | 114076254 | 114107839 | ADPRHL1 | | | | | |
| 13 | 114110133 | 114145023 | DCUN1D2 | | | | | |

| | | | | | | | | |
|---|---|---|---|---|---|---|---|---|
| 13 | 114145307 | 114204544 | TMCO3 | | | | | |
| 13 | 114239002 | 114295788 | TFDP1 | | | | | |
| 13 | 114303121 | 114312513 | ATP4B | | | | | |
| 13 | 114321596 | 114438637 | GRK1 | | | | | |
| 13 | 114451483 | 114454062 | LINC00552 | | | | | |
| 13 | 114462215 | 114514899 | TMEM255B | | | | | |
| 13 | 114518582 | 114569805 | GAS6 | | | GAS6 | | |
| 13 | 114598075 | 114624290 | LINC00452 | | | | | |
| 13 | 114629486 | 114631964 | LINC00565 | | | | | |
| 13 | 114747193 | 114898095 | RASA3 | | | | | |
| 13 | 115000361 | 115038150 | CDC16 | | | | | |
| 13 | 115009979 | 115010036 | MIR548AR | | | | | |
| 13 | 115039302 | 115039383 | MIR4502 | | | | | |
| 13 | 115047058 | 115071291 | UPF3A | | | | | |
| 13 | 115079964 | 115092803 | CHAMP1 | | | | | |
| 13 | 115095231 | 115099423 | LINC01054 | | | | | |
| 14 | 19377593 | 19378574 | OR11H12 | | | | | |
| 14 | 19407014 | 19410111 | LOC642426 | | | | | |
| 14 | 19553364 | 19584942 | POTEG | | | | | |
| 14 | 19563121 | 19566680 | LOC101929572 | | | | | |
| 14 | 19563711 | 19566680 | POTEH | | | | | |
| 14 | 19650031 | 19692916 | DUXAP10 | | | | | |
| 14 | 19650036 | 19684290 | LINC01296 | | | | | |
| 14 | 19670791 | 19681016 | BMS1P17 | | | | | |
| 14 | 19670791 | 19681016 | BMS1P18 | | | | | |
| 14 | 19670791 | 19686002 | BMS1P22 | | | | | |
| 14 | 19880208 | 19925329 | LINC01296 | | | | | |
| 14 | 19882467 | 19925334 | DUXAP10 | | | | | |
| 14 | 19889385 | 19904572 | BMS1P22 | | | | | |
| 14 | 19891096 | 19925329 | LINC01296 | | | | | |
| 14 | 19894368 | 19904572 | BMS1P17 | | | | | |
| 14 | 19894368 | 19904572 | BMS1P18 | | | | | |
| 14 | 19983953 | 20020272 | POTEM | | | | | |
| 14 | 20006912 | 20010956 | LOC100508046 | | | | | |
| 14 | 20181062 | 20182491 | OR11H2 | | | | | |
| 14 | 20215586 | 20216528 | OR4Q3 | | | | | |
| 14 | 20248481 | 20249423 | OR4M1 | | | | | |
| 14 | 20271928 | 20296531 | OR4N2 | | | | | |
| 14 | 20344426 | 20345371 | OR4K2 | | | OR4K2 | | |
| 14 | 20388765 | 20389737 | OR4K5 | | | | | |
| 14 | 20403766 | 20404842 | OR4K1 | | | | | |
| 14 | 20443677 | 20444724 | OR4K15 | | | | | |
| 14 | 20482419 | 20483352 | OR4K14 | | | | | |
| 14 | 20502002 | 20502917 | OR4K13 | | | | | |
| 14 | 20528203 | 20529142 | OR4L1 | | | | | |
| 14 | 20585565 | 20586597 | OR4K17 | | | | | |
| 14 | 20611894 | 20612821 | OR4N5 | | | | | |
| 14 | 20665494 | 20666532 | OR11G2 | | | | | |
| 14 | 20691868 | 20692861 | OR11H6 | | | | | |
| 14 | 20710950 | 20711925 | OR11H4 | | | | | |
| 14 | 20757300 | 20774153 | TTC5 | | | | | |
| 14 | 20779526 | 20801471 | CCNB1IP1 | | | CCNB1IP1 | | |
| 14 | 20794599 | 20794698 | SNORD126 | | | | | |
| 14 | 20811229 | 20811570 | RPPH1 | | | | | |
| 14 | 20811772 | 20826063 | PARP2 | | | | | |
| 14 | 20833825 | 20881579 | TEP1 | | | | | |
| 14 | 20896969 | 20903801 | KLHL33 | | | | | |
| 14 | 20915206 | 20923267 | OSGEP | | | | | |
| 14 | 20923289 | 20925931 | APEX1 | | | APEX1 | | |
| 14 | 20926011 | 20929771 | TMEM55B | | | | | |
| 14 | 20937537 | 20946165 | PNP | | | | | |
| 14 | 20978630 | 20979281 | RNASE10 | | | | | |
| 14 | 21024251 | 21029090 | RNASE9 | | | | | |
| 14 | 21051051 | 21058417 | RNASE11 | | | | | |
| 14 | 21056382 | 21075380 | LOC254028 | | | | | |
| 14 | 21058239 | 21058982 | RNASE12 | | | | | |

| | | | | | | | | |
|---|---|---|---|---|---|---|---|---|
| 14 | 21108854 | 21109850 | OR6S1 | | | | | |
| 14 | 21152335 | 21162345 | ANG | | | | | |
| 14 | 21152371 | 21168761 | RNASE4 | | | | | |
| 14 | 21156931 | 21162345 | ANG | | | | | |
| 14 | 21156935 | 21168761 | RNASE4 | | | | | |
| 14 | 21214098 | 21216539 | EDDM3A | | | | | |
| 14 | 21236585 | 21239107 | EDDM3B | | | | | |
| 14 | 21249209 | 21250626 | RNASE6 | | | | | |
| 14 | 21269514 | 21271036 | RNASE1 | | | | | |
| 14 | 21359561 | 21360507 | RNASE3 | | | | | |
| 14 | 21387499 | 21388335 | ECRP | | | | | |
| 14 | 21423629 | 21424594 | RNASE2 | | | | | |
| 14 | 21457964 | 21465194 | METTL17 | | | | | |
| 14 | 21463995 | 21467322 | LOC101929718 | | | | | |
| 14 | 21467413 | 21470034 | SLC39A2 | | | | | |
| 14 | 21484921 | 21539031 | NDRG2 | | | | | |
| 14 | 21491472 | 21491545 | MIR6717 | | | | | |
| 14 | 21498344 | 21500332 | TPPP2 | | | | | |
| 14 | 21500978 | 21502944 | RNASE13 | | | | | |
| 14 | 21510384 | 21512392 | RNASE7 | | | | | |
| 14 | 21525980 | 21526614 | RNASE8 | | | | | |
| 14 | 21538418 | 21558406 | ARHGEF40 | | | | | |
| 14 | 21558204 | 21572863 | ZNF219 | | | ZNF219 | | |
| 14 | 21567095 | 21571883 | TMEM253 | | | | | |
| 14 | 21623095 | 21624184 | OR5AU1 | | | | | |
| 14 | 21668237 | 21675059 | LINC00641 | | | | | |
| 14 | 21677295 | 21737638 | HNRNPC | | | | | |
| 14 | 21756135 | 21819460 | RPGRIP1 | | | | | |
| 14 | 21819630 | 21852425 | SUPT16H | | | SUPT16H | | |
| 14 | 21853352 | 21905457 | CHD8 | | | CHD8 | | |
| 14 | 21860309 | 21860412 | SNORD9 | | | | | |
| 14 | 21865451 | 21865560 | SNORD8 | | | | | |
| 14 | 21927178 | 21945132 | RAB2B | | | | | |
| 14 | 21945334 | 21967321 | TOX4 | | | | | |
| 14 | 21966274 | 21979517 | METTL3 | | | | | |
| 14 | 21989230 | 22005350 | SALL2 | | | | | |
| 14 | 22037933 | 22038875 | OR10G3 | | | | | |
| 14 | 22101991 | 22103096 | OR10G2 | | | | | |
| 14 | 22133296 | 22134238 | OR4E2 | | | | | |
| 14 | 22138124 | 22139232 | OR4E1 | | | | | |
| 14 | 23033806 | 23058143 | DAD1 | | | | | |
| 14 | 23067146 | 23081265 | ABHD4 | | | | | |
| 14 | 23235730 | 23240998 | OXA1L | | | | | |
| 14 | 23242431 | 23289020 | SLC7A7 | | | SLC7A7 | | |
| 14 | 23299091 | 23304246 | MRPL52 | | | | | |
| 14 | 23305741 | 23316808 | MMP14 | | | | | |
| 14 | 23340959 | 23347291 | LRP10 | | | | | |
| 14 | 23352431 | 23356889 | REM2 | | | | | |
| 14 | 23369853 | 23388396 | RBM23 | | | RBM23 | | |
| 14 | 23388664 | 23398794 | PRMT5 | | | | | |
| 14 | 23398817 | 23423901 | LOC101926933 | | | | | |
| 14 | 23415436 | 23426351 | HAUS4 | | | | | |
| 14 | 23426158 | 23426238 | MIR4707 | | | | | |
| 14 | 23440382 | 23451851 | AJUBA | | | | | |
| 14 | 23456109 | 23479410 | C14orf93 | | | C14orf93 | | |
| 14 | 23495059 | 23504429 | PSMB5 | | | | | |
| 14 | 23511375 | 23513269 | PSMB11 | | | | | |
| 14 | 23516269 | 23526747 | CDH24 | | | | | |
| 14 | 23527773 | 23564823 | ACIN1 | | | | | |
| 14 | 23564682 | 23569665 | C14orf119 | | | | | |
| 14 | 23586514 | 23588820 | CEBPE | | | | | |
| 14 | 23594503 | 23652869 | SLC7A8 | | | | | |
| 14 | 23707126 | 23743494 | RNF212B | | | | | |
| 14 | 23742843 | 23755309 | HOMEZ | | | | | |
| 14 | 23765129 | 23772057 | PPP1R3E | | | | | |
| 14 | 23775970 | 23780968 | BCL2L2 | | | BCL2L2 | | |

| | | | | | | | | |
|---|---|---|---|---|---|---|---|---|
| 14 | 23775970 | 23795394 | BCL2L2-PABPN1 | | | | | |
| 14 | 23776068 | 23780968 | BCL2L2 | | | BCL2L2 | | |
| 14 | 23789396 | 23795394 | PABPN1 | | | | | |
| 14 | 23815519 | 23822121 | SLC22A17 | | | | | |
| 14 | 23825608 | 23834842 | EFS | | | | | |
| 14 | 23842017 | 23845612 | IL25 | | | | | |
| 14 | 23846016 | 23848981 | CMTM5 | | | | | |
| 14 | 23851198 | 23877486 | MYH6 | | | | | |
| 14 | 23857804 | 23857875 | MIR208A | | | | | |
| 14 | 23881946 | 23904895 | MYH7 | | | MYH7 | | |
| 14 | 23884658 | 23886804 | MHRT | | | | | |
| 14 | 23887195 | 23887272 | MIR208B | | | | | |
| 14 | 23938897 | 23947402 | NGDN | | | | | |
| 14 | 23980968 | 24028790 | THTPA | | | | | |
| 14 | 23990063 | 24020858 | ZFHX2 | | | | | |
| 14 | 24025190 | 24028790 | THTPA | | | | | |
| 14 | 24028771 | 24037045 | AP1G2 | | | | | |
| 14 | 24030305 | 24037284 | LOC102724814 | | | | | |
| 14 | 24037243 | 24048009 | JPH4 | | | | | |
| 14 | 24099323 | 24114848 | DHRS2 | | | | | |
| 14 | 24407939 | 24438488 | DHRS4 | | | | | |
| 14 | 24439082 | 24475617 | DHRS4L2 | | | | | |
| 14 | 24476217 | 24520586 | DHRS4L1 | | | | | |
| 14 | 24521205 | 24538937 | LRRC16B | | | | | |
| 14 | 24540045 | 24547309 | CPNE6 | | | CPNE6 | | |
| 14 | 24549315 | 24553832 | NRL | | | | | |
| 14 | 24563339 | 24573341 | PCK2 | | | | | |
| 14 | 24583905 | 24594451 | DCAF11 | | | | | |
| 14 | 24600674 | 24602058 | FITM1 | | | | | |
| 14 | 24605366 | 24608176 | PSME1 | | | | | |
| 14 | 24608173 | 24610797 | EMC9 | | | | | |
| 14 | 24612573 | 24615855 | PSME2 | | | | | |
| 14 | 24612697 | 24612774 | MIR7703 | | | | | |
| 14 | 24616083 | 24629870 | RNF31 | | | | | |
| 14 | 24630421 | 24635774 | IRF9 | | | | | |
| 14 | 24641233 | 24649463 | REC8 | | | | | |
| 14 | 24649424 | 24658124 | IPO4 | | | | | |
| 14 | 24658348 | 24664942 | TM9SF1 | | | | | |
| 14 | 24674856 | 24677454 | TSSK4 | | | | | |
| 14 | 24678786 | 24683036 | CHMP4A | | | CHMP4A | | |
| 14 | 24683142 | 24685276 | MDP1 | | | | | |
| 14 | 24683142 | 24701576 | NEDD8-MDP1 | | | | | |
| 14 | 24686056 | 24701576 | NEDD8 | | | | | |
| 14 | 24701627 | 24708447 | GMPR2 | | | | | |
| 14 | 24708850 | 24711880 | TINF2 | | | | | |
| 14 | 24718319 | 24732416 | TGM1 | | | | | |
| 14 | 24734743 | 24740833 | RABGGTA | | | | | |
| 14 | 24759803 | 24769039 | DHRS1 | | | | | |
| 14 | 24769059 | 24778332 | NOP9 | | | | | |
| 14 | 24774392 | 24780583 | CIDEB | | | | | |
| 14 | 24779356 | 24781259 | LTB4R2 | | | | | |
| 14 | 24780704 | 24787242 | LTB4R | | | | | |
| 14 | 24787554 | 24804277 | ADCY4 | | | | | |
| 14 | 24805226 | 24809242 | RIPK3 | | | | | |
| 14 | 24836116 | 24848811 | NFATC4 | | | | | |
| 14 | 24867991 | 24888494 | NYNRIN | | | | | |
| 14 | 24895739 | 24898731 | CBLN3 | | | | | |
| 14 | 24898491 | 24910547 | KHNYN | | | | | |
| 14 | 24908971 | 24912111 | SDR39U1 | | | | | |
| 14 | 24912158 | 24971403 | LOC101927045 | | | | | |
| 14 | 24974560 | 24977471 | CMA1 | | | | | |
| 14 | 25042723 | 25045466 | CTSG | | | | | |
| 14 | 25075685 | 25078926 | GZMH | | | | | |
| 14 | 25100160 | 25103432 | GZMB | | | | | |
| 14 | 25278860 | 25519503 | STXBP6 | | | STXBP6 | | |
| 14 | 26915088 | 27066960 | NOVA1 | | | NOVA1 | | |

| | | | | | | | | |
|---|---|---|---|---|---|---|---|---|
| 14 | 27244700 | 27291326 | LOC101927062 | | | | | |
| 14 | 27278553 | 27289909 | LOC102724890 | | | | | |
| 14 | 27342338 | 27383949 | MIR4307HG | | | | | |
| 14 | 27377847 | 27377931 | MIR4307 | | | | | |
| 14 | 28081793 | 28108842 | LINC00645 | | | | | |
| 14 | 28102410 | 28102484 | MIR3171 | | | | | |
| 14 | 29194447 | 29239483 | FOXG1 | | FOXG1 | FOXG1 | | |
| 14 | 29241909 | 29264000 | LINC01551 | | | | | |
| 14 | 29896116 | 30174608 | MIR548AI | | | | | |
| 14 | 30045686 | 30396899 | PRKD1 | | | PRKD1 | | |
| 14 | 31028328 | 31089267 | G2E3 | | | | | |
| 14 | 31091459 | 31205033 | SCFD1 | | | SCFD1 | | |
| 14 | 31343740 | 31359822 | COCH | | | | | |
| 14 | 31345384 | 31359014 | LOC100506071 | | | | | |
| 14 | 31363004 | 31495607 | STRN3 | | | | | |
| 14 | 31483851 | 31483948 | MIR624 | | | | | |
| 14 | 31494311 | 31565656 | AP4S1 | | AP4S1 | | | |
| 14 | 31569320 | 31676729 | HECTD1 | | | | | |
| 14 | 31760993 | 31889788 | HEATR5A | | | | | |
| 14 | 31889963 | 31922089 | LOC101927124 | | | | | |
| 14 | 31915242 | 31926680 | DTD2 | | | | | |
| 14 | 31952149 | 31957151 | GPR33 | | | | | |
| 14 | 32030590 | 32330429 | NUBPL | | | | | |
| 14 | 32544624 | 32628934 | ARHGAP5 | | | | | |
| 14 | 32671250 | 32672475 | RNU6-2 | | | | | |
| 14 | 32798478 | 33302268 | AKAP6 | | | AKAP6 | | |
| 14 | 33408458 | 34273382 | NPAS3 | | NPAS3 | | | |
| 14 | 34178142 | 34178319 | SNORA89 | | | | | |
| 14 | 34393420 | 34420284 | EGLN3 | | | | | |
| 14 | 34902143 | 34931468 | SPTSSA | | | | | |
| 14 | 34985134 | 35008943 | EAPP | | | | | |
| 14 | 35030617 | 35099366 | SNX6 | | | SNX6 | | |
| 14 | 35179587 | 35184029 | CFL2 | | CFL2 | | | |
| 14 | 35221936 | 35344853 | BAZ1A | | | | | |
| 14 | 35409127 | 35409702 | IGBP1P1 | | | | | |
| 14 | 35452103 | 35498773 | SRP54 | | | | | |
| 14 | 35514112 | 35552589 | FAM177A1 | | | | | |
| 14 | 35550286 | 35582336 | LOC101927178 | | | | | |
| 14 | 35554673 | 35591749 | PPP2R3C | | | | | |
| 14 | 35591526 | 35743284 | KIAA0391 | | | | | |
| 14 | 35747763 | 35786685 | PSMA6 | | | | | |
| 14 | 35870715 | 35873960 | NFKBIA | | | | | |
| 14 | 36003247 | 36006260 | INSM2 | | | | | |
| 14 | 36007557 | 36278432 | RALGAPA1 | | | | | |
| 14 | 36007960 | 36278350 | RALGAPA1P1 | | | | | |
| 14 | 36017713 | 36278432 | RALGAPA1 | | | | | |
| 14 | 36295596 | 36341169 | BRMS1L | | | | | |
| 14 | 36539632 | 36634494 | LINC00609 | | | | | |
| 14 | 36604915 | 36645857 | PTCSC3 | | | | | |
| 14 | 36767763 | 36789882 | MBIP | | | | | |
| 14 | 36942493 | 36982990 | SFTA3 | | | | | |
| 14 | 36985603 | 36989430 | NKX2-1 | | | | | |
| 14 | 36988482 | 36992221 | NKX2-1-AS1 | | | | | |
| 14 | 37049215 | 37051819 | NKX2-8 | | | | | |
| 14 | 37126772 | 37147011 | PAX9 | | | | | |
| 14 | 37147125 | 37641865 | SLC25A21 | | | | | |
| 14 | 37421513 | 37421596 | MIR4503 | | | | | |
| 14 | 37641230 | 37642998 | SLC25A21 | | | | | |
| 14 | 37667117 | 38020464 | MIPOL1 | | | | | |
| 14 | 38058756 | 38064325 | FOXA1 | | | | | |
| 14 | 38064403 | 38311830 | TTC6 | | | | | |
| 14 | 38677203 | 38682268 | SSTR1 | | | SSTR1 | | |
| 14 | 38723204 | 38725575 | CLEC14A | | | | | |
| 14 | 39218542 | 39386086 | LINC00639 | | | | | |
| 14 | 39501122 | 39572437 | SEC23A | | | | | |
| 14 | 39583487 | 39606177 | GEMIN2 | | | | | |

| | | | | | | | | |
|---|---|---|---|---|---|---|---|---|
| 14 | 39617014 | 39639634 | TRAPPC6B | | | | | |
| 14 | 39644386 | 39652422 | PNN | | | PNN | | |
| 14 | 39703124 | 39722575 | MIA2 | | | | | |
| 14 | 39734475 | 39820397 | CTAGE5 | | | | | |
| 14 | 39734906 | 39736265 | LOC100288846 | | | | | |
| 14 | 39735501 | 39820397 | CTAGE5 | | | | | |
| 14 | 39865576 | 39901704 | FBXO33 | | | | | |
| 14 | 41423915 | 41610251 | LOC644919 | | | | | |
| 14 | 42076763 | 42373752 | LRFN5 | | | | | |
| 14 | 44973353 | 44976499 | FSCB | | | | | |
| 14 | 45366506 | 45376460 | C14orf28 | | | | | |
| 14 | 45368110 | 45381120 | LOC101927418 | | | | | |
| 14 | 45393512 | 45431179 | KLHL28 | | | | | |
| 14 | 45431392 | 45543634 | FAM179B | | | | | |
| 14 | 45553301 | 45584804 | PRPF39 | | | | | |
| 14 | 45580077 | 45580176 | SNORD127 | | | | | |
| 14 | 45584801 | 45603732 | FKBP3 | | | | | |
| 14 | 45605141 | 45670093 | FANCM | | | FANCM | | |
| 14 | 45672392 | 45722605 | MIS18BP1 | | | | | |
| 14 | 46533361 | 46971104 | LINC00871 | | | | | |
| 14 | 47120219 | 47121028 | RPL10L | | | | | |
| 14 | 47308825 | 47812449 | MDGA2 | | | MDGA2 | | |
| 14 | 48230197 | 48230307 | MIR548Y | | | | | |
| 14 | 48234155 | 48264217 | LINC00648 | | | | | |
| 14 | 50043389 | 50053134 | RPS29 | | | | | |
| 14 | 50065414 | 50081390 | LRR1 | | | | | |
| 14 | 50085405 | 50087403 | RPL36AL | | | | | |
| 14 | 50087488 | 50090199 | MGAT2 | | | | | |
| 14 | 50091891 | 50101948 | DNAAF2 | | | | | |
| 14 | 50110269 | 50155098 | POLE2 | | | | | |
| 14 | 50159822 | 50219870 | KLHDC1 | | | | | |
| 14 | 50234786 | 50249856 | KLHDC2 | | | KLHDC2 | | |
| 14 | 50250531 | 50319791 | NEMF | | | | | |
| 14 | 50359735 | 50363772 | ARF6 | | | | | |
| 14 | 50433116 | 50433229 | MIR6076 | | | | | |
| 14 | 50448386 | 50473381 | LINC01588 | | | | | |
| 14 | 50474030 | 50571761 | LINC01599 | | | | | |
| 14 | 50575349 | 50583297 | VCPKMT | | | | | |
| 14 | 50583845 | 50698099 | SOS2 | | | | | |
| 14 | 50709151 | 50778947 | L2HGDH | | | | | |
| 14 | 50766572 | 50766664 | MIR4504 | | | | | |
| 14 | 50779046 | 50792946 | ATP5S | | | ATP5S | | |
| 14 | 50796308 | 50864122 | CDKL1 | | | | | |
| 14 | 50885210 | 50999376 | MAP4K5 | | | MAP4K5 | | |
| 14 | 50999799 | 51099784 | ATL1 | | ATL1 | | | |
| 14 | 51100297 | 51135071 | SAV1 | | | | | |
| 14 | 51186480 | 51297839 | NIN | | | | | |
| 14 | 51338877 | 51371688 | ABHD12B | | | | | |
| 14 | 51371934 | 51411248 | PYGL | | | | | |
| 14 | 51441980 | 51562422 | TRIM9 | | | TRIM9 | | |
| 14 | 51706885 | 51724372 | TMX1 | | | | | |
| 14 | 51800110 | 51832275 | LINC00640 | | | | | |
| 14 | 51921229 | 52197444 | FRMD6 | | | | | |
| 14 | 52327021 | 52436518 | GNG2 | | | | | |
| 14 | 52382862 | 52385289 | LOC102723604 | | | | | |
| 14 | 52456227 | 52471420 | C14orf166 | | | | | |
| 14 | 52471519 | 52535946 | NID2 | | | | | |
| 14 | 52734430 | 52743442 | PTGDR | | | PTGDR | | |
| 14 | 52781015 | 52795322 | PTGER2 | | | | | |
| 14 | 52897307 | 53019301 | TXNDC16 | | | | | |
| 14 | 53019865 | 53104431 | GPR137C | | | | | |
| 14 | 53106632 | 53162649 | ERO1A | | | | | |
| 14 | 53173895 | 53194716 | PSMC6 | | | | | |
| 14 | 53196882 | 53241705 | STYX | | | | | |
| 14 | 53241910 | 53258386 | GNPNAT1 | | | | | |
| 14 | 53323988 | 53417815 | FERMT2 | | | | | |

| | | | | | | | | |
|---|---|---|---|---|---|---|---|---|
| 14 | 53503457 | 53620046 | DDHD1 | | DDHD1 | | | |
| 14 | 53620071 | 53624246 | LOC101927620 | | | | | |
| 14 | 54415144 | 54415202 | MIR5580 | | | | | |
| 14 | 54416454 | 54423580 | BMP4 | | | | | |
| 14 | 54863672 | 54886934 | CDKN3 | | | | | |
| 14 | 54893646 | 54908148 | CNIH1 | | | | | |
| 14 | 54941208 | 54955744 | GMFB | | | GMFB | | |
| 14 | 54976586 | 55005334 | CGRRF1 | | | | | |
| 14 | 55034329 | 55260033 | SAMD4A | | | | | |
| 14 | 55308723 | 55369542 | GCH1 | | | GCH1 | | |
| 14 | 55344830 | 55344911 | MIR4308 | | | | | |
| 14 | 55405655 | 55493819 | WDHD1 | | | | | |
| 14 | 55493843 | 55516206 | SOCS4 | | | | | |
| 14 | 55518361 | 55536912 | MAPK1IP1L | | | | | |
| 14 | 55595934 | 55612148 | LGALS3 | | | | | |
| 14 | 55614833 | 55658396 | DLGAP5 | | | | | |
| 14 | 55738020 | 55819801 | FBXO34 | | | FBXO34 | | |
| 14 | 55833108 | 55878576 | ATG14 | | | | | |
| 14 | 55880929 | 55907263 | TBPL2 | | | | | |
| 14 | 56042874 | 56151302 | KTN1 | | | KTN1 | | |
| 14 | 56232962 | 56234435 | RPL13AP3 | | | | | |
| 14 | 56247852 | 56263392 | LINC00520 | | | | | |
| 14 | 56585092 | 56768031 | PELI2 | | | | | |
| 14 | 57046510 | 57116232 | TMEM260 | | | | | |
| 14 | 57267424 | 57397550 | OTX2 | | OTX2 | | | |
| 14 | 57669193 | 57735617 | EXOC5 | | | | | |
| 14 | 57735605 | 57756797 | AP5M1 | | | | | |
| 14 | 57857270 | 57879466 | NAA30 | | | | | |
| 14 | 57936017 | 57960581 | C14orf105 | | | | | |
| 14 | 58030639 | 58332592 | SLC35F4 | | | | | |
| 14 | 58470807 | 58618847 | C14orf37 | | | | | |
| 14 | 58666832 | 58702353 | ACTR10 | | | | | |
| 14 | 58711522 | 58764855 | PSMA3 | | | | | |
| 14 | 58765221 | 58840451 | ARID4A | | | | | |
| 14 | 58862643 | 58875419 | TOMM20L | | | | | |
| 14 | 58875211 | 58894332 | TIMM9 | | | | | |
| 14 | 58894102 | 59003931 | KIAA0586 | | | | | |
| 14 | 59100785 | 59115038 | DACT1 | | | | | |
| 14 | 59295005 | 59484046 | LINC01500 | | | | | |
| 14 | 59655380 | 59838123 | DAAM1 | | | | | |
| 14 | 59930239 | 59932059 | GPR135 | | | | | |
| 14 | 59939405 | 59951073 | L3HYPDH | | | | | |
| 14 | 59951160 | 59972124 | JKAMP | | | | | |
| 14 | 59971784 | 60043549 | CCDC175 | | | | | |
| 14 | 60062693 | 60337557 | RTN1 | | RTN1 | | | |
| 14 | 60386430 | 60530277 | LRRC9 | | | | | |
| 14 | 60558628 | 60601532 | PCNX4 | | | | | |
| 14 | 60611499 | 60632211 | DHRS7 | | | | | |
| 14 | 60712469 | 60765805 | PPM1A | | | | | |
| 14 | 60902673 | 60952764 | C14orf39 | | | | | |
| 14 | 60975937 | 60978525 | SIX6 | | | SIX6 | | |
| 14 | 61105933 | 61107134 | SALRNA1 | | | | | |
| 14 | 61111416 | 61116155 | SIX1 | | | SIX1 | | |
| 14 | 61176255 | 61190852 | SIX4 | | | | | |
| 14 | 61201458 | 61435398 | MNAT1 | | | | | |
| 14 | 61438166 | 61447797 | TRMT5 | | | | | |
| 14 | 61447831 | 61550451 | SLC38A6 | | | | | |
| 14 | 61744088 | 61748530 | TMEM30B | | | | | |
| 14 | 61788160 | 62017698 | PRKCH | | | PRKCH | | |
| 14 | 62022989 | 62037371 | LOC101927780 | | | | | |
| 14 | 62037257 | 62121431 | FLJ22447 | | | | | |
| 14 | 62147758 | 62215807 | HIF1A | | | HIF1A | | |
| 14 | 62229074 | 62263146 | SNAPC1 | | | | | |
| 14 | 62462540 | 62568427 | SYT16 | | | | | |
| 14 | 62584074 | 62600903 | LINC00643 | | | | | |
| 14 | 62600867 | 62606691 | LINC00644 | | | | | |

| | | | | | | | | |
|---|---|---|---|---|---|---|---|---|
| 14 | 63173290 | 63511956 | KCNH5 | | KCNH5 | KCNH5 | | |
| 14 | 63671101 | 63760230 | RHOJ | | | | | |
| 14 | 63779548 | 63785593 | GPHB5 | | | | | |
| 14 | 63840609 | 64010079 | PPP2R5E | | | | | |
| 14 | 64063756 | 64108641 | WDR89 | | | | | |
| 14 | 64150934 | 64194813 | SGPP1 | | | | | |
| 14 | 64319682 | 64693167 | SYNE2 | | | SYNE2 | | |
| 14 | 64419067 | 64927439 | MIR548AZ | | | | | |
| 14 | 64561741 | 64561843 | MIR548H1 | | | | | |
| 14 | 64680858 | 64693167 | SYNE2 | | | SYNE2 | | |
| 14 | 64693429 | 64761128 | ESR2 | ESR2 | | | ESR2 | |
| 14 | 64812190 | 64814329 | TEX21P | | | | | |
| 14 | 64854758 | 64926725 | MTHFD1 | | | MTHFD1 | | |
| 14 | 64915823 | 64971931 | ZBTB25 | | | | | |
| 14 | 64932216 | 64941221 | AKAP5 | | AKAP5 | | | |
| 14 | 64952955 | 64970563 | ZBTB25 | | | | | |
| 14 | 64971291 | 65000408 | ZBTB1 | | | | | |
| 14 | 64980868 | 65007086 | LOC102723809 | | | | | |
| 14 | 65007185 | 65009954 | HSPA2 | | | | | |
| 14 | 65016619 | 65056097 | PPP1R36 | | | | | |
| 14 | 65171125 | 65211064 | PLEKHG3 | | | | | |
| 14 | 65213000 | 65289866 | SPTB | | SPTB | | | |
| 14 | 65252343 | 65252404 | MIR7855 | | | | | |
| 14 | 65381078 | 65402084 | CHURC1 | | | | | |
| 14 | 65381078 | 65529373 | CHURC1-FNTB | | | | | |
| 14 | 65405869 | 65409623 | GPX2 | | | | | |
| 14 | 65412531 | 65438875 | RAB15 | | | | | |
| 14 | 65453506 | 65529370 | FNTB | | | | | |
| 14 | 65472818 | 65569262 | MAX | | | | | |
| 14 | 65511405 | 65511487 | MIR4706 | | | | | |
| 14 | 65541841 | 65569413 | MAX | | | | | |
| 14 | 65556635 | 65561029 | LOC100506321 | | | | | |
| 14 | 65679610 | 65689065 | LOC100128233 | | | | | |
| 14 | 65801835 | 65801901 | MIR4708 | | | | | |
| 14 | 65877309 | 66210839 | FUT8 | | | | | |
| 14 | 65937819 | 65937904 | MIR625 | | | | | |
| 14 | 66953088 | 66965271 | LINC00238 | | | | | |
| 14 | 66974124 | 67648525 | GPHN | | GPHN | GPHN | | |
| 14 | 67656109 | 67695267 | FAM71D | | | | | |
| 14 | 67708011 | 67802778 | MPP5 | | | MPP5 | | |
| 14 | 67804580 | 67826720 | ATP6V1D | | | | | |
| 14 | 67827033 | 67853233 | EIF2S1 | | | | | |
| 14 | 67853695 | 67878917 | PLEK2 | | | | | |
| 14 | 67936982 | 67982021 | TMEM229B | | | | | |
| 14 | 68000007 | 68056255 | PLEKHH1 | | | | | |
| 14 | 68056022 | 68067017 | PIGH | | | | | |
| 14 | 68086578 | 68118436 | ARG2 | | ARG2 | | | |
| 14 | 68117866 | 68141602 | VTI1B | | | VTI1B | | |
| 14 | 68143518 | 68162510 | RDH11 | | | | | |
| 14 | 68168602 | 68201168 | RDH12 | | | | | |
| 14 | 68213236 | 68283306 | ZFYVE26 | | | | | |
| 14 | 68286495 | 69062738 | RAD51B | | | | | |
| 14 | 69254371 | 69262960 | ZFP36L1 | | | | | |
| 14 | 69340839 | 69454180 | ACTN1 | | | | | |
| 14 | 69517636 | 69619914 | DCAF5 | | | | | |
| 14 | 69658193 | 69710737 | EXD2 | | | | | |
| 14 | 69726680 | 69821190 | GALNT16 | | | | | |
| 14 | 69846839 | 69865021 | ERH | | | | | |
| 14 | 69865095 | 69929107 | SLC39A9 | | | | | |
| 14 | 69951470 | 69995215 | PLEKHD1 | | | | | |
| 14 | 70036530 | 70041600 | CCDC177 | | | | | |
| 14 | 70078309 | 70181861 | SUSD6 | | | | | |
| 14 | 70232999 | 70234430 | LOC100289511 | | | | | |
| 14 | 70233828 | 70238722 | SRSF5 | | | | | |
| 14 | 70242551 | 70264006 | SLC10A1 | | | | | |
| 14 | 70346113 | 70499083 | SMOC1 | | SMOC1 | | | |

| | | | | | | | | |
|---|---|---|---|---|---|---|---|---|
| 14 | 70510933 | 70655787 | SLC8A3 | | SLC8A3 | | | |
| 14 | 70712469 | 70714518 | ADAM21P1 | | | | | |
| 14 | 70791797 | 70826448 | COX16 | | | | | |
| 14 | 70791797 | 70883807 | SYNJ2BP-COX16 | | | | | |
| 14 | 70833212 | 70883807 | SYNJ2BP | | | SYNJ2BP | | |
| 14 | 70918873 | 70926622 | ADAM21 | | | | | |
| 14 | 70935595 | 70950473 | ADAM20P1 | | | | | |
| 14 | 70989077 | 71001732 | ADAM20 | | | | | |
| 14 | 71049937 | 71067407 | MED6 | | | | | |
| 14 | 71075514 | 71108015 | LOC101928075 | | | | | |
| 14 | 71108503 | 71142077 | TTC9 | | | | | |
| 14 | 71165414 | 71178870 | LINC01269 | | | | | |
| 14 | 71189242 | 71275888 | MAP3K9 | | | | | |
| 14 | 71374121 | 71582099 | PCNX1 | | | | | |
| 14 | 71865053 | 71865124 | SNORD56B | | | | | |
| 14 | 71954577 | 71956420 | LOC145474 | | | | | |
| 14 | 71996028 | 72207946 | SIPA1L1 | | | | | |
| 14 | 72398816 | 73033238 | RGS6 | | RGS6 | | | |
| 14 | 72983527 | 72983606 | MIR7843 | | | | | |
| 14 | 73086003 | 73360824 | DPF3 | | | | | |
| 14 | 73393039 | 73426357 | DCAF4 | | | | | |
| 14 | 73436152 | 73493920 | ZFYVE1 | | | ZFYVE1 | | |
| 14 | 73525220 | 73588076 | RBM25 | | | | | |
| 14 | 73603142 | 73690399 | PSEN1 | PSEN1 | PSEN1 | PSEN1 | | |
| 14 | 73704204 | 73741347 | PAPLN | | | | | |
| 14 | 73741917 | 73925286 | NUMB | | | | | |
| 14 | 73945188 | 74025651 | HEATR4 | | | | | |
| 14 | 73957638 | 73960105 | C14orf169 | | | | | |
| 14 | 74003927 | 74010498 | ACOT1 | | | | | |
| 14 | 74034323 | 74042362 | ACOT2 | | | | | |
| 14 | 74058409 | 74062470 | ACOT4 | | | | | |
| 14 | 74083547 | 74086592 | ACOT6 | | | | | |
| 14 | 74111577 | 74170431 | DNAL1 | | DNAL1 | | | |
| 14 | 74178485 | 74181128 | PNMA1 | | | | | |
| 14 | 74181824 | 74253961 | ELMSAN1 | | | | | |
| 14 | 74225449 | 74225522 | MIR4505 | | | | | |
| 14 | 74254062 | 74270331 | LOC100506476 | | | | | |
| 14 | 74318533 | 74352168 | PTGR2 | | | | | |
| 14 | 74353317 | 74398991 | ZNF410 | | | | | |
| 14 | 74399694 | 74417117 | FAM161B | | | FAM161B | | |
| 14 | 74416636 | 74429813 | COQ6 | | | | | |
| 14 | 74433180 | 74486026 | ENTPD5 | | | | | |
| 14 | 74486058 | 74532796 | BBOF1 | | | | | |
| 14 | 74524367 | 74551196 | ALDH6A1 | | ALDH6A1 | | | |
| 14 | 74551655 | 74667117 | LIN52 | | | | | |
| 14 | 74706174 | 74729441 | VSX2 | | | | | |
| 14 | 74751979 | 74769767 | ABCD4 | | | | | |
| 14 | 74815165 | 74826711 | VRTN | | | | | |
| 14 | 74872595 | 74892805 | SYNDIG1L | | | | | |
| 14 | 74946642 | 74960084 | NPC2 | | | NPC2 | | |
| 14 | 74946835 | 74946907 | MIR4709 | | | | | |
| 14 | 74960422 | 74962271 | ISCA2 | | | | | |
| 14 | 74964885 | 75079034 | LTBP2 | | | | | |
| 14 | 75127954 | 75179807 | AREL1 | | | | | |
| 14 | 75179849 | 75203390 | FCF1 | | | | | |
| 14 | 75230068 | 75304013 | YLPM1 | | | | | |
| 14 | 75319735 | 75330537 | PROX2 | | | | | |
| 14 | 75348593 | 75370450 | DLST | | | DLST | | |
| 14 | 75370656 | 75389145 | RPS6KL1 | | RPS6KL1 | | | |
| 14 | 75408532 | 75422467 | PGF | | | | | |
| 14 | 75469611 | 75476294 | EIF2B2 | | | | | |
| 14 | 75480466 | 75518235 | MLH3 | | | | | |
| 14 | 75519927 | 75536186 | ACYP1 | | | | | |
| 14 | 75536279 | 75546690 | ZC2HC1C | | | | | |
| 14 | 75548817 | 75593778 | NEK9 | | | | | |
| 14 | 75598170 | 75643349 | TMED10 | | | | | |

| | | | | | | | | | |
|---|---|---|---|---|---|---|---|---|---|
| 14 | 75745480 | 75748937 | FOS | | | | | | |
| 14 | 75761106 | 75763111 | LINC01220 | | | | | | |
| 14 | 75894508 | 75939404 | JDP2 | | | | | | |
| 14 | 75988783 | 76013334 | BATF | | | | | | |
| 14 | 76041246 | 76045931 | LOC102724153 | | | | | | |
| 14 | 76044939 | 76114512 | FLVCR2 | | | | | | |
| 14 | 76070551 | 76070604 | MIR7641 | | | | | | |
| 14 | 76071804 | 76114512 | FLVCR2 | | | | | | |
| 14 | 76117232 | 76127538 | C14orf1 | | | | | | |
| 14 | 76127550 | 76421425 | TTLL5 | | | | | | |
| 14 | 76424439 | 76448365 | TGFB3 | | | | | | |
| 14 | 76452095 | 76550416 | IFT43 | | | | | | |
| 14 | 76618227 | 76679146 | GPATCH2L | | | | | | |
| 14 | 76837689 | 76968180 | ESRRB | | | | | | |
| 14 | 77228234 | 77249363 | VASH1 | | | | | | |
| 14 | 77248075 | 77253067 | LOC100506603 | | | | | | |
| 14 | 77253585 | 77279283 | ANGEL1 | | | | | | |
| 14 | 77292724 | 77336645 | LRRC74A | | | | | | |
| 14 | 77425980 | 77432145 | LINC01629 | | | | | | |
| 14 | 77490885 | 77495042 | IRF2BPL | | | | | | |
| 14 | 77507391 | 77535846 | LOC283575 | | | | | | |
| 14 | 77535522 | 77542534 | LOC102724190 | | | | | | |
| 14 | 77564577 | 77583630 | CIPC | | | | | | |
| 14 | 77597612 | 77608134 | ZDHHC22 | | | | | | |
| 14 | 77648101 | 77725838 | TMEM63C | | | | | | |
| 14 | 77731833 | 77737655 | NGB | | | | | | |
| 14 | 77732560 | 77732633 | MIR1260A | | | | | | |
| 14 | 77741298 | 77787225 | POMT2 | | POMT2 | POMT2 | | | |
| 14 | 77787229 | 77797940 | GSTZ1 | | | | | | |
| 14 | 77808113 | 77843396 | TMED8 | | | | | | |
| 14 | 77843761 | 77857587 | SAMD15 | | | | | | |
| 14 | 77860364 | 77889399 | NOXRED1 | | | | | | |
| 14 | 77893017 | 77923983 | VIPAS39 | | | | | | |
| 14 | 77924372 | 77935815 | AHSA1 | | | | | | |
| 14 | 77940737 | 77965210 | ISM2 | | | | | | |
| 14 | 77972339 | 78083110 | SPTLC2 | | | | | | |
| 14 | 78138748 | 78174356 | ALKBH1 | | | | | | |
| 14 | 78174413 | 78183941 | SLIRP | | | | | | |
| 14 | 78183943 | 78227497 | SNW1 | | | | | | |
| 14 | 78227172 | 78236085 | C14orf178 | | | | | | |
| 14 | 78266425 | 78400297 | ADCK1 | | | | | | |
| 14 | 78636715 | 80334633 | NRXN3 | | | NRXN3 | | | |
| 14 | 80663867 | 80921810 | DIO2 | | | | | | |
| 14 | 80962820 | 81405884 | CEP128 | | | | | | |
| 14 | 81421868 | 81612646 | TSHR | | | | | | |
| 14 | 81641795 | 81687575 | GTF2A1 | | | | | | |
| 14 | 81669038 | 81669178 | SNORA79 | | | | | | |
| 14 | 81726993 | 81893748 | STON2 | | | | | | |
| 14 | 81908330 | 81916501 | LOC100506700 | | | | | | |
| 14 | 81937890 | 82000205 | SEL1L | | | | | | |
| 14 | 82071690 | 82089405 | LINC01467 | | | | | | |
| 14 | 85860222 | 85886418 | LINC00911 | | | | | | |
| 14 | 85996487 | 86094270 | FLRT2 | | | | | | |
| 14 | 86401021 | 86596121 | LOC101928767 | | | | | | |
| 14 | 87372121 | 87389099 | LOC283585 | | | | | | |
| 14 | 88399357 | 88460009 | GALC | | GALC | GALC | | | |
| 14 | 88471467 | 88481155 | GPR65 | | | | | | |
| 14 | 88484948 | 88492656 | LOC101928791 | | | | | | |
| 14 | 88490893 | 88553688 | LINC01146 | | | | | | |
| 14 | 88646451 | 88793256 | KCNK10 | | | | | | |
| 14 | 88851987 | 88904804 | SPATA7 | | SPATA7 | | | | |
| 14 | 88932121 | 89021123 | PTPN21 | | | | | | |
| 14 | 89029252 | 89079853 | ZC3H14 | | | | | | |
| 14 | 89081173 | 89259096 | EML5 | | | | | | |
| 14 | 89290496 | 89344340 | TTC8 | | | | | | |
| 14 | 89622515 | 90043820 | FOXN3 | | | | | | |

| | | | | | | | | |
|---|---|---|---|---|---|---|---|---|
| 14 | 90261335 | 90421121 | EFCAB11 | | | | | |
| 14 | 90422245 | 90511108 | TDP1 | | | TDP1 | | |
| 14 | 90528108 | 90652201 | KCNK13 | | | | | |
| 14 | 90722893 | 90738966 | PSMC1 | | | | | |
| 14 | 90744397 | 90798481 | NRDE2 | | | | | |
| 14 | 90863326 | 90874619 | CALM1 | | CALM1 | CALM1 | | |
| 14 | 90921573 | 90925249 | LINC00642 | | | | | |
| 14 | 91006931 | 91282761 | TTC7B | | | | | |
| 14 | 91108972 | 91115232 | LOC101928909 | | | | | |
| 14 | 91337166 | 91526993 | RPS6KA5 | | | RPS6KA5 | | |
| 14 | 91580356 | 91691740 | C14orf159 | | | C14orf159 | | |
| 14 | 91592769 | 91592896 | SNORA11B | | | | | |
| 14 | 91698875 | 91720224 | GPR68 | | | | | |
| 14 | 91737666 | 91884188 | CCDC88C | | | | | |
| 14 | 91923824 | 91976824 | PPP4R3A | | | | | |
| 14 | 92047117 | 92198430 | CATSPERB | | | | | |
| 14 | 92246095 | 92333880 | TC2N | | | TC2N | | |
| 14 | 92335754 | 92414046 | FBLN5 | | | | | |
| 14 | 92434242 | 92506403 | TRIP11 | | | TRIP11 | | |
| 14 | 92524895 | 92572965 | ATXN3 | | | | | |
| 14 | 92582467 | 92588153 | NDUFB1 | | | | | |
| 14 | 92588297 | 92630543 | CPSF2 | | | | | |
| 14 | 92788924 | 92967825 | SLC24A4 | | SLC24A4 | | | |
| 14 | 92980124 | 93155339 | RIN3 | | | | | |
| 14 | 93170151 | 93215047 | LGMN | | | LGMN | | |
| 14 | 93260575 | 93306306 | GOLGA5 | | | GOLGA5 | | |
| 14 | 93389444 | 93401641 | CHGA | | | | | |
| 14 | 93403258 | 93538478 | ITPK1 | | | | | |
| 14 | 93648540 | 93651249 | MOAP1 | | | | | |
| 14 | 93651295 | 93653425 | TMEM251 | | | | | |
| 14 | 93669236 | 93673459 | C14orf142 | | | | | |
| 14 | 93673400 | 93695561 | UBR7 | | | | | |
| 14 | 93703895 | 93799438 | BTBD7 | | | | | |
| 14 | 93799564 | 94173689 | UNC79 | | | | | |
| 14 | 93813536 | 93814700 | COX8C | | | COX8C | | |
| 14 | 94184643 | 94254766 | PRIMA1 | | PRIMA1 | PRIMA1 | | PRIMA1 |
| 14 | 94371075 | 94395954 | FAM181A | | | | | |
| 14 | 94400498 | 94443076 | ASB2 | | | | | |
| 14 | 94414571 | 94414648 | MIR4506 | | | | | |
| 14 | 94463615 | 94478041 | LINC00521 | | | | | |
| 14 | 94492723 | 94515276 | OTUB2 | | | | | |
| 14 | 94517267 | 94547558 | DDX24 | | | | | |
| 14 | 94547638 | 94569060 | IFI27L1 | | | | | |
| 14 | 94577078 | 94583036 | IFI27 | | | | | |
| 14 | 94594117 | 94595957 | IFI27L2 | | | | | |
| 14 | 94640648 | 94746072 | PPP4R4 | | | | | |
| 14 | 94749649 | 94759608 | SERPINA10 | | | | | |
| 14 | 94770584 | 94789688 | SERPINA6 | | | | | |
| 14 | 94829974 | 94833039 | SERPINA2 | | | | | |
| 14 | 94843083 | 94857029 | SERPINA1 | | | | | |
| 14 | 94908800 | 94919122 | SERPINA11 | | | | | |
| 14 | 94929057 | 94942670 | SERPINA9 | | | | | |
| 14 | 94953610 | 94984181 | SERPINA12 | | | | | |
| 14 | 95027756 | 95036250 | SERPINA4 | | | | | |
| 14 | 95047705 | 95059457 | SERPINA5 | | | | | |
| 14 | 95078713 | 95090390 | SERPINA3 | | | SERPINA3 | | |
| 14 | 95107061 | 95113331 | SERPINA13P | | | | | |
| 14 | 95234559 | 95236499 | GSC | | | | | |
| 14 | 95552564 | 95624347 | DICER1 | | | DICER1 | | |
| 14 | 95604255 | 95604323 | MIR3173 | | | | | |
| 14 | 95624024 | 95646270 | DICER1 | | | DICER1 | | |
| 14 | 95648275 | 95786245 | CLMN | | | | | |
| 14 | 95795920 | 95801841 | LOC101929080 | | | | | |
| 14 | 95873603 | 95876427 | LINC00341 | | | | | |
| 14 | 95883830 | 95942173 | SYNE3 | | | | | |
| 14 | 95999248 | 96001209 | SNHG10 | | | | | |

| | | | | | | | | |
|---|---|---|---|---|---|---|---|---|
| 14 | 95999691 | 95999966 | SCARNA13 | | | | | |
| 14 | 96001322 | 96011055 | GLRX5 | | | | | |
| 14 | 96117514 | 96139789 | TCL6 | | | | | |
| 14 | 96152753 | 96158980 | TCL1B | | | | | |
| 14 | 96176303 | 96180533 | TCL1A | | | | | |
| 14 | 96342728 | 96391908 | TUNAR | | | | | |
| 14 | 96505660 | 96560308 | C14orf132 | | | | | |
| 14 | 96671134 | 96710666 | BDKRB2 | | | | | |
| 14 | 96722546 | 96731100 | BDKRB1 | | | BDKRB1 | | |
| 14 | 96747594 | 96829678 | ATG2B | | | | | |
| 14 | 96829788 | 96853627 | GSKIP | | | | | |
| 14 | 96858447 | 96955764 | AK7 | | | | | |
| 14 | 96967984 | 96968663 | LOC730202 | | | | | |
| 14 | 96968712 | 97033453 | PAPOLA | | | | | |
| 14 | 97263683 | 97347951 | VRK1 | | | | | |
| 14 | 97409915 | 97411731 | LINC00618 | | | | | |
| 14 | 97925152 | 98047720 | LOC101929241 | | | | | |
| 14 | 98098983 | 98152995 | LOC100129345 | | | | | |
| 14 | 98391946 | 98444461 | LINC01550 | | | | | |
| 14 | 99177949 | 99184103 | C14orf177 | | | | | |
| 14 | 99635624 | 99738050 | BCL11B | | | | | |
| 14 | 99864082 | 99947226 | SETD3 | | | | | |
| 14 | 99947738 | 99977852 | CCNK | | | | | |
| 14 | 99977602 | 100070727 | CCDC85C | | | | | |
| 14 | 100111479 | 100143011 | HHIPL1 | | | | | |
| 14 | 100140218 | 100140241 | MIR5698 | | | | | |
| 14 | 100150754 | 100193638 | CYP46A1 | | CYP46A1 | | | |
| 14 | 100259744 | 100408395 | EML1 | | | | | |
| 14 | 100531750 | 100610573 | EVL | | | | | |
| 14 | 100575755 | 100575851 | MIR151B | | | | | |
| 14 | 100575991 | 100576090 | MIR342 | | | | | |
| 14 | 100612752 | 100626012 | DEGS2 | | | | | |
| 14 | 100705101 | 100745371 | YY1 | | | | | |
| 14 | 100743693 | 100743754 | MIR6764 | | | | | |
| 14 | 100757447 | 100772884 | SLC25A29 | | | SLC25A29 | | |
| 14 | 100774195 | 100774293 | MIR345 | | | | | |
| 14 | 100789678 | 100796715 | SLC25A47 | | | | | |
| 14 | 100800124 | 100842680 | WARS | | | | | |
| 14 | 100842754 | 100996640 | WDR25 | | | | | |
| 14 | 101003483 | 101036131 | BEGAIN | | | | | |
| 14 | 101123604 | 101139081 | LINC00523 | | | | | |
| 14 | 101193201 | 101204561 | DLK1 | | | | | |
| 14 | 101280827 | 101280911 | MIR2392 | | | | | |
| 14 | 101292444 | 101327360 | MEG3 | | | | | |
| 14 | 101318726 | 101318824 | MIR770 | | | | | |
| 14 | 101335396 | 101335485 | MIR493 | | | | | |
| 14 | 101340829 | 101340922 | MIR337 | | | | | |
| 14 | 101341369 | 101341441 | MIR665 | | | | | |
| 14 | 101346991 | 101351184 | RTL1 | | | | | |
| 14 | 101347343 | 101347457 | MIR431 | | | | | |
| 14 | 101348222 | 101348315 | MIR433 | | | | | |
| 14 | 101349315 | 101349412 | MIR127 | | | | | |
| 14 | 101350819 | 101350913 | MIR432 | | | | | |
| 14 | 101351038 | 101351120 | MIR136 | | | | | |
| 14 | 101361106 | 101373305 | MEG8 | | | | | |
| 14 | 101377475 | 101377550 | MIR370 | | | | | |
| 14 | 101391157 | 101391227 | SNORD113-1 | | | | | |
| 14 | 101393678 | 101393749 | SNORD113-2 | | | | | |
| 14 | 101402827 | 101402901 | SNORD113-4 | | | | | |
| 14 | 101404523 | 101404600 | SNORD113-5 | | | | | |
| 14 | 101405892 | 101405966 | SNORD113-6 | | | | | |
| 14 | 101407462 | 101407538 | SNORD113-7 | | | | | |
| 14 | 101411985 | 101412056 | SNORD113-9 | | | | | |
| 14 | 101416169 | 101416240 | SNORD114-1 | | | | | |
| 14 | 101418192 | 101418269 | SNORD114-2 | | | | | |
| 14 | 101419685 | 101419759 | SNORD114-3 | | | | | |

| | | | | | | | | | |
|---|---|---|---|---|---|---|---|---|---|
| 14 | 101420680 | 101420784 | SNORD114-4 | | | | | | |
| 14 | 101421706 | 101421775 | SNORD114-5 | | | | | | |
| 14 | 101423502 | 101423573 | SNORD114-6 | | | | | | |
| 14 | 101429390 | 101429466 | SNORD114-7 | | | | | | |
| 14 | 101431117 | 101431188 | SNORD114-8 | | | | | | |
| 14 | 101432365 | 101432436 | SNORD114-9 | | | | | | |
| 14 | 101433388 | 101433459 | SNORD114-10 | | | | | | |
| 14 | 101433988 | 101452987 | SNHG24 | | | | | | |
| 14 | 101434447 | 101434521 | SNORD114-11 | | | | | | |
| 14 | 101435284 | 101435358 | SNORD114-12 | | | | | | |
| 14 | 101436215 | 101436288 | SNORD114-13 | | | | | | |
| 14 | 101438439 | 101438513 | SNORD114-14 | | | | | | |
| 14 | 101439006 | 101439077 | SNORD114-15 | | | | | | |
| 14 | 101439931 | 101440000 | SNORD114-16 | | | | | | |
| 14 | 101441142 | 101441216 | SNORD114-17 | | | | | | |
| 14 | 101442161 | 101442232 | SNORD114-18 | | | | | | |
| 14 | 101442813 | 101442887 | SNORD114-19 | | | | | | |
| 14 | 101447340 | 101447411 | SNORD114-20 | | | | | | |
| 14 | 101448311 | 101448382 | SNORD114-21 | | | | | | |
| 14 | 101449262 | 101449333 | SNORD114-22 | | | | | | |
| 14 | 101450212 | 101450283 | SNORD114-23 | | | | | | |
| 14 | 101451113 | 101451184 | SNORD114-24 | | | | | | |
| 14 | 101452393 | 101452464 | SNORD114-25 | | | | | | |
| 14 | 101453382 | 101453453 | SNORD114-26 | | | | | | |
| 14 | 101454497 | 101454566 | SNORD114-27 | | | | | | |
| 14 | 101455466 | 101455537 | SNORD114-28 | | | | | | |
| 14 | 101456427 | 101456496 | SNORD114-29 | | | | | | |
| 14 | 101458255 | 101458326 | SNORD114-30 | | | | | | |
| 14 | 101459572 | 101459646 | SNORD114-31 | | | | | | |
| 14 | 101488402 | 101488469 | MIR379 | | | | | | |
| 14 | 101489661 | 101489757 | MIR411 | | | | | | |
| 14 | 101490130 | 101490193 | MIR299 | | | | | | |
| 14 | 101491353 | 101491414 | MIR380 | | | | | | |
| 14 | 101491900 | 101491988 | MIR1197 | | | | | | |
| 14 | 101492068 | 101492154 | MIR323A | | | | | | |
| 14 | 101492356 | 101492444 | MIR758 | | | | | | |
| 14 | 101493121 | 101493520 | MIR329 | | | | | | |
| 14 | 101495970 | 101496051 | MIR494 | | | | | | |
| 14 | 101496388 | 101496466 | MIR1193 | | | | | | |
| 14 | 101498323 | 101498401 | MIR543 | | | | | | |
| 14 | 101500091 | 101500173 | MIR495 | | | | | | |
| 14 | 101506026 | 101506092 | MIR376C | | | | | | |
| 14 | 101506405 | 101506485 | MIR376A2 | | | | | | |
| 14 | 101506555 | 101506636 | MIR654 | | | | | | |
| 14 | 101506772 | 101506872 | MIR376B | | | | | | |
| 14 | 101507118 | 101507186 | MIR376A1 | | | | | | |
| 14 | 101507699 | 101507781 | MIR300 | | | | | | |
| 14 | 101509313 | 101510619 | MIR1185 | | | | | | |
| 14 | 101511493 | 101518132 | MIR381HG | | | | | | |
| 14 | 101512256 | 101512331 | MIR381 | | | | | | |
| 14 | 101512791 | 101512875 | MIR487B | | | | | | |
| 14 | 101513657 | 101513735 | MIR539 | | | | | | |
| 14 | 101514237 | 101514316 | MIR889 | | | | | | |
| 14 | 101514994 | 101515085 | MIR544A | | | | | | |
| 14 | 101515886 | 101515983 | MIR655 | | | | | | |
| 14 | 101518782 | 101518862 | MIR487A | | | | | | |
| 14 | 101520642 | 101520718 | MIR382 | | | | | | |
| 14 | 101521023 | 101521096 | MIR134 | | | | | | |
| 14 | 101521594 | 101521660 | MIR668 | | | | | | |
| 14 | 101521755 | 101521828 | MIR485 | | | | | | |
| 14 | 101522555 | 101522637 | MIR323B | | | | | | |
| 14 | 101526091 | 101526175 | MIR154 | | | | | | |
| 14 | 101526909 | 101527011 | MIR496 | | | | | | |
| 14 | 101528386 | 101528455 | MIR377 | | | | | | |
| 14 | 101530831 | 101530915 | MIR541 | | | | | | |
| 14 | 101531636 | 101531715 | MIR409 | | | | | | |

| | | | | | | | | | |
|---|---|---|---|---|---|---|---|---|---|
| 14 | 101531783 | 101531874 | MIR412 | | | | | | |
| 14 | 101531934 | 101532004 | MIR369 | | | | | | |
| 14 | 101532248 | 101532328 | MIR410 | | | | | | |
| 14 | 101533060 | 101533138 | MIR656 | | | | | | |
| 14 | 101536247 | 101539273 | MEG9 | | | | | | |
| 14 | 101872323 | 101874259 | LINC00524 | | | | | | |
| 14 | 102018559 | 102022013 | DIO3OS | | | | | | |
| 14 | 102026623 | 102026759 | MIR1247 | | | | | | |
| 14 | 102027687 | 102029789 | DIO3 | | | | | | |
| 14 | 102196773 | 102198862 | LINC00239 | | | | | | |
| 14 | 102228134 | 102394328 | PPP2R5C | | | | | | |
| 14 | 102430864 | 102517135 | DYNC1H1 | | DYNC1H1 | DYNC1H1 | | | |
| 14 | 102547074 | 102606086 | HSP90AA1 | | | HSP90AA1 | | | |
| 14 | 102606188 | 102690010 | WDR20 | | | | | | |
| 14 | 102690833 | 102771537 | MOK | | | | | | |
| 14 | 102783713 | 102809511 | ZNF839 | | | | | | |
| 14 | 102814618 | 102829253 | CINP | | | | | | |
| 14 | 102829248 | 102968818 | TECPR2 | | TECPR2 | | | | |
| 14 | 102973197 | 102976128 | ANKRD9 | | | | | | |
| 14 | 103005980 | 103006063 | MIR4309 | | | | | | |
| 14 | 103058995 | 103196913 | RCOR1 | | | | | | |
| 14 | 103243815 | 103377837 | TRAF3 | | | | | | |
| 14 | 103388992 | 103397179 | AMN | | | | | | |
| 14 | 103398715 | 103523742 | CDC42BPB | | | | | | |
| 14 | 103566480 | 103576894 | EXOC3L4 | | | | | | |
| 14 | 103592663 | 103603776 | TNFAIP2 | | | | | | |
| 14 | 103653557 | 103655365 | LINC00605 | | | | | | |
| 14 | 103800338 | 103811361 | EIF5 | | | | | | |
| 14 | 103804185 | 103804311 | SNORA28 | | | | | | |
| 14 | 103851700 | 103970166 | MARK3 | | | MARK3 | | | |
| 14 | 103985994 | 103989196 | CKB | | | | | | |
| 14 | 103995508 | 104003410 | TRMT61A | | | | | | |
| 14 | 104022880 | 104029151 | BAG5 | | | | | | |
| 14 | 104029293 | 104058510 | APOPT1 | | | | | | |
| 14 | 104095524 | 104167888 | KLC1 | | | | | | |
| 14 | 104163953 | 104181823 | XRCC3 | | | | | | |
| 14 | 104182080 | 104200005 | ZFYVE21 | | | | | | |
| 14 | 104200087 | 104313927 | PPP1R13B | | | PPP1R13B | | | |
| 14 | 104314057 | 104324386 | LINC00637 | | | | | | |
| 14 | 104378624 | 104387903 | C14orf2 | | | | | | |
| 14 | 104394816 | 104519004 | TDRD9 | | | | | | |
| 14 | 104406762 | 104408645 | RD3L | | | | | | |
| 14 | 104552022 | 104579046 | ASPG | | | | | | |
| 14 | 104583741 | 104583851 | MIR203A | | | | | | |
| 14 | 104583754 | 104583840 | MIR203B | | | | | | |
| 14 | 104605059 | 104647235 | KIF26A | | | | | | |
| 14 | 105046020 | 105056184 | C14orf180 | | | | | | |
| 14 | 105057200 | 105071097 | TMEM179 | | | | | | |
| 14 | 105144030 | 105144086 | MIR4710 | | | | | | |
| 14 | 105155942 | 105185947 | INF2 | | INF2 | | | | |
| 14 | 105190533 | 105213647 | ADSSL1 | | | | | | |
| 14 | 105219469 | 105225996 | SIVA1 | | | | | | |
| 14 | 105235686 | 105262080 | AKT1 | | | AKT1 | | | |
| 14 | 105267517 | 105271048 | ZBTB42 | | | | | | |
| 14 | 105287537 | 105290055 | LINC00638 | | | | | | |
| 14 | 105331649 | 105363107 | CEP170B | | | | | | |
| 14 | 105391152 | 105399573 | PLD4 | | | | | | |
| 14 | 105403590 | 105444694 | AHNAK2 | | | | | | |
| 14 | 105452615 | 105461855 | C14orf79 | | | | | | |
| 14 | 105475909 | 105487425 | CDCA4 | | | | | | |
| 14 | 105515725 | 105531887 | GPR132 | | | | | | |
| 14 | 105560483 | 105565833 | LOC102723354 | | | | | | |
| 14 | 105607317 | 105635161 | JAG2 | | | | | | |
| 14 | 105617114 | 105617201 | MIR6765 | | | | | | |
| 14 | 105639273 | 105647660 | NUDT14 | | | | | | |
| 14 | 105675622 | 105781914 | BRF1 | | BRF1 | | | | |

| | | | | | | | | |
|---|---|---|---|---|---|---|---|---|
| 14 | 105714878 | 105717430 | BTBD6 | | | | | |
| 14 | 105722522 | 105767329 | BRF1 | | BRF1 | | | |
| 14 | 105767147 | 105864484 | PACS2 | | | | | |
| 14 | 105864919 | 105880196 | TEX22 | | | | | |
| 14 | 105883917 | 105886076 | LOC100507437 | | | | | |
| 14 | 105886185 | 105937057 | MTA1 | | | | | |
| 14 | 105939274 | 105946507 | CRIP2 | | | | | |
| 14 | 105953256 | 105955124 | CRIP1 | | | | | |
| 14 | 105956191 | 105965585 | C14orf80 | | | | | |
| 14 | 105992952 | 105996539 | TMEM121 | | | | | |
| 14 | 106087452 | 106106569 | MIR8071 | | | | | |
| 14 | 106135913 | 106139144 | ELK2AP | | | | | |
| 14 | 106324292 | 106324344 | MIR4507 | | | | | |
| 14 | 106324333 | 106324411 | MIR4538 | | | | | |
| 14 | 106325211 | 106325722 | MIR4537 | | | | | |
| 14 | 106326563 | 106326584 | MIR4539 | | | | | |
| 14 | 106383837 | 106398502 | KIAA0125 | | | | | |
| 14 | 106435817 | 106438358 | ADAM6 | | | | | |
| 14 | 106744268 | 106744966 | LINC00226 | | | | | |
| 14 | 106938444 | 106951529 | LINC00221 | | | | | |
| 14 | 107092715 | 107092765 | MIR7641 | | | | | |
| 14 | 107259099 | 107259214 | MIR5195 | | | | | |
| 15 | 20487996 | 20496811 | CHEK2P2 | | | | | |
| 15 | 20613649 | 20711433 | HERC2P3 | | | | | |
| 15 | 20737093 | 20747114 | GOLGA6L6 | | | | | |
| 15 | 20767673 | 20781026 | GOLGA8CP | | | | | |
| 15 | 20874796 | 20961480 | NBEAP1 | | | | | |
| 15 | 21038123 | 21038198 | MIR3118 | | | | | |
| 15 | 21040700 | 21071643 | POTEB | | | | | |
| 15 | 21040700 | 21071643 | POTEB2 | | | | | |
| 15 | 21040700 | 21071981 | POTEB3 | | | | | |
| 15 | 21122020 | 21134625 | NF1P2 | | | | | |
| 15 | 21145580 | 21145662 | MIR5701 | | | | | |
| 15 | 21145766 | 21198633 | LINC01193 | | | | | |
| 15 | 21932513 | 21940739 | LOC646214 | | | | | |
| 15 | 22014419 | 22016878 | CXADRP2 | | | | | |
| 15 | 22049273 | 22049348 | MIR3118 | | | | | |
| 15 | 22051852 | 22082803 | POTEB | | | | | |
| 15 | 22051852 | 22082803 | POTEB2 | | | | | |
| 15 | 22051852 | 22083141 | POTEB3 | | | | | |
| 15 | 22133180 | 22145802 | NF1P2 | | | | | |
| 15 | 22156765 | 22156847 | MIR5701 | | | | | |
| 15 | 22278030 | 22383564 | LOC101927079 | | | | | |
| 15 | 22278031 | 22371088 | LOC727924 | | | | | |
| 15 | 22303211 | 22383564 | LOC101927079 | | | | | |
| 15 | 22368477 | 22369561 | OR4M2 | | | | | |
| 15 | 22382381 | 22383815 | OR4N4 | | | | | |
| 15 | 22413461 | 22414395 | OR4N3P | | | | | |
| 15 | 22513228 | 22513280 | MIR1268A | | | | | |
| 15 | 22546564 | 22570831 | REREP3 | | | | | |
| 15 | 22675147 | 22675241 | MIR4509 | | | | | |
| 15 | 22702284 | 22715728 | GOLGA8DP | | | | | |
| 15 | 22736245 | 22746002 | GOLGA6L1 | | | | | |
| 15 | 22736267 | 22746002 | GOLGA6L22 | | | | | |
| 15 | 22833394 | 22873891 | TUBGCP5 | | | TUBGCP5 | | |
| 15 | 22892648 | 23003603 | CYFIP1 | | | | | |
| 15 | 23004683 | 23034427 | NIPA2 | | | | | |
| 15 | 23043278 | 23086843 | NIPA1 | | NIPA1 | NIPA1 | | |
| 15 | 23094330 | 23115254 | LOC283683 | | | | | |
| 15 | 23187728 | 23208357 | WHAMMP3 | | | | | |
| 15 | 23255241 | 23262743 | GOLGA8IP | | | | | |
| 15 | 23282264 | 23378259 | HERC2P2 | | | | | |
| 15 | 23390721 | 23393943 | HERC2P7 | | | | | |
| 15 | 23403865 | 23579357 | GOLGA6L22 | | | | | |
| 15 | 23435069 | 23448423 | GOLGA8EP | | | | | |
| 15 | 23599894 | 23613471 | GOLGA8S | | | | | |

| | | | | | | | | |
|---|---|---|---|---|---|---|---|---|
| 15 | 23684532 | 23692390 | GOLGA6L2 | | | | | |
| 15 | 23807208 | 23807278 | MIR4508 | | | | | |
| 15 | 23810453 | 23813166 | MKRN3 | | MKRN3 | | | |
| 15 | 23888695 | 23892993 | MAGEL2 | | MAGEL2 | | | |
| 15 | 23930553 | 23932450 | NDN | | | NDN | | |
| 15 | 24220293 | 24333301 | PWRN4 | | | | | |
| 15 | 24409925 | 24415053 | PWRN2 | | | | | |
| 15 | 24686273 | 24693114 | PWRN3 | | | | | |
| 15 | 24803303 | 24832926 | PWRN1 | | | | | |
| 15 | 24920540 | 24928593 | NPAP1 | | | | | |
| 15 | 25068793 | 25223729 | SNRPN | | SNRPN | SNRPN | | |
| 15 | 25200069 | 25213976 | SNURF | | SNURF | | | |
| 15 | 25200134 | 25223729 | SNRPN | | SNRPN | SNRPN | | |
| 15 | 25200134 | 25223729 | SNURF | | SNURF | | | |
| 15 | 25227140 | 25227215 | SNORD107 | | | | | |
| 15 | 25227140 | 25228937 | PWARSN | | | | | |
| 15 | 25230006 | 25233379 | PWAR5 | | | | | |
| 15 | 25230246 | 25230313 | SNORD64 | | | | | |
| 15 | 25232071 | 25232140 | SNORD108 | | | | | |
| 15 | 25287120 | 25287187 | SNORD109A | | | | | |
| 15 | 25287120 | 25287187 | SNORD109B | | | | | |
| 15 | 25296622 | 25296719 | SNORD116-1 | | | | | |
| 15 | 25299355 | 25299452 | SNORD116-2 | | | | | |
| 15 | 25302005 | 25302102 | SNORD116-3 | | | | | |
| 15 | 25302005 | 25302102 | SNORD116-9 | | | | | |
| 15 | 25304683 | 25304781 | SNORD116-4 | | | | | |
| 15 | 25307478 | 25307575 | SNORD116-5 | | | | | |
| 15 | 25307478 | 25307575 | SNORD116-7 | | | | | |
| 15 | 25310171 | 25310269 | SNORD116-2 | | | | | |
| 15 | 25310171 | 25310269 | SNORD116-6 | | | | | |
| 15 | 25312933 | 25313030 | SNORD116-5 | | | | | |
| 15 | 25312933 | 25313030 | SNORD116-7 | | | | | |
| 15 | 25315577 | 25315674 | SNORD116-8 | | | | | |
| 15 | 25318252 | 25318349 | SNORD116-3 | | | | | |
| 15 | 25318252 | 25318349 | SNORD116-9 | | | | | |
| 15 | 25319259 | 25319363 | SNORD116-10 | | | | | |
| 15 | 25321074 | 25321168 | SNORD116-11 | | | | | |
| 15 | 25322196 | 25322290 | SNORD116-12 | | | | | |
| 15 | 25324203 | 25324297 | SNORD116-13 | | | | | |
| 15 | 25325287 | 25325381 | SNORD116-14 | | | | | |
| 15 | 25326432 | 25326526 | SNORD116-15 | | | | | |
| 15 | 25327913 | 25328007 | SNORD116-16 | | | | | |
| 15 | 25328733 | 25328827 | SNORD116-17 | | | | | |
| 15 | 25328733 | 25328827 | SNORD116-19 | | | | | |
| 15 | 25330530 | 25330624 | SNORD116-18 | | | | | |
| 15 | 25331672 | 25331766 | SNORD116-17 | | | | | |
| 15 | 25331672 | 25331766 | SNORD116-19 | | | | | |
| 15 | 25332807 | 25332901 | SNORD116-20 | | | | | |
| 15 | 25333949 | 25334043 | SNORD116-21 | | | | | |
| 15 | 25335068 | 25335162 | SNORD116-22 | | | | | |
| 15 | 25336931 | 25337025 | SNORD116-23 | | | | | |
| 15 | 25339182 | 25339276 | SNORD116-24 | | | | | |
| 15 | 25342808 | 25342902 | SNORD116-25 | | | | | |
| 15 | 25344644 | 25344742 | SNORD116-26 | | | | | |
| 15 | 25346720 | 25346814 | SNORD116-27 | | | | | |
| 15 | 25349787 | 25349880 | SNORD116-28 | | | | | |
| 15 | 25351666 | 25351751 | SNORD116-29 | | | | | |
| 15 | 25353414 | 25353499 | SNORD116-30 | | | | | |
| 15 | 25361691 | 25367623 | IPW | | | | | |
| 15 | 25380788 | 25383200 | PWAR1 | | | | | |
| 15 | 25415869 | 25415951 | SNORD115-1 | | | | | |
| 15 | 25417781 | 25417863 | SNORD115-2 | | | | | |
| 15 | 25420073 | 25420155 | SNORD115-3 | | | | | |
| 15 | 25421978 | 25422060 | SNORD115-4 | | | | | |
| 15 | 25423884 | 25423966 | SNORD115-5 | | | | | |
| 15 | 25423887 | 25423966 | SNORD115-10 | | | | | |

| | | | | | | | | |
|---|---|---|---|---|---|---|---|---|
| 15 | 25423887 | 25423966 | SNORD115-12 | | | | | |
| 15 | 25423887 | 25423966 | SNORD115-9 | | | | | |
| 15 | 25425643 | 25425725 | SNORD115-6 | | | | | |
| 15 | 25427531 | 25427613 | SNORD115-7 | | | | | |
| 15 | 25429452 | 25429534 | SNORD115-8 | | | | | |
| 15 | 25430777 | 25430859 | SNORD115-10 | | | | | |
| 15 | 25430777 | 25430859 | SNORD115-12 | | | | | |
| 15 | 25430777 | 25430859 | SNORD115-9 | | | | | |
| 15 | 25430780 | 25430859 | SNORD115-5 | | | | | |
| 15 | 25432682 | 25432763 | SNORD115-10 | | | | | |
| 15 | 25434560 | 25434642 | SNORD115-11 | | | | | |
| 15 | 25434560 | 25434642 | SNORD115-29 | | | | | |
| 15 | 25434560 | 25434642 | SNORD115-36 | | | | | |
| 15 | 25434560 | 25434642 | SNORD115-43 | | | | | |
| 15 | 25436562 | 25436644 | SNORD115-10 | | | | | |
| 15 | 25436562 | 25436644 | SNORD115-12 | | | | | |
| 15 | 25436562 | 25436644 | SNORD115-9 | | | | | |
| 15 | 25436565 | 25436644 | SNORD115-5 | | | | | |
| 15 | 25438467 | 25438549 | SNORD115-13 | | | | | |
| 15 | 25440067 | 25440148 | SNORD115-14 | | | | | |
| 15 | 25444594 | 25444676 | SNORD115-16 | | | | | |
| 15 | 25446469 | 25446551 | SNORD115-17 | | | | | |
| 15 | 25446469 | 25446551 | SNORD115-18 | | | | | |
| 15 | 25446469 | 25446551 | SNORD115-19 | | | | | |
| 15 | 25448373 | 25448455 | SNORD115-17 | | | | | |
| 15 | 25448373 | 25448455 | SNORD115-18 | | | | | |
| 15 | 25448373 | 25448455 | SNORD115-19 | | | | | |
| 15 | 25449503 | 25449585 | SNORD115-17 | | | | | |
| 15 | 25449503 | 25449585 | SNORD115-18 | | | | | |
| 15 | 25449503 | 25449585 | SNORD115-19 | | | | | |
| 15 | 25451408 | 25451490 | SNORD115-20 | | | | | |
| 15 | 25451408 | 25477615 | SNORD115-15 | | | | | |
| 15 | 25451408 | 25477615 | SNORD115-21 | | | | | |
| 15 | 25455064 | 25455146 | SNORD115-22 | | | | | |
| 15 | 25456838 | 25457180 | PWAR4 | | | | | |
| 15 | 25456942 | 25457024 | SNORD115-23 | | | | | |
| 15 | 25458805 | 25458876 | SNORD115-24 | | | | | |
| 15 | 25460687 | 25460769 | SNORD115-25 | | | | | |
| 15 | 25463763 | 25463845 | SNORD115-26 | | | | | |
| 15 | 25465649 | 25465725 | SNORD115-27 | | | | | |
| 15 | 25467500 | 25467574 | SNORD115-28 | | | | | |
| 15 | 25468392 | 25468474 | SNORD115-11 | | | | | |
| 15 | 25468392 | 25468474 | SNORD115-29 | | | | | |
| 15 | 25468392 | 25468474 | SNORD115-36 | | | | | |
| 15 | 25468392 | 25468474 | SNORD115-43 | | | | | |
| 15 | 25470349 | 25470431 | SNORD115-30 | | | | | |
| 15 | 25472255 | 25472337 | SNORD115-31 | | | | | |
| 15 | 25474113 | 25474195 | SNORD115-32 | | | | | |
| 15 | 25475984 | 25476066 | SNORD115-33 | | | | | |
| 15 | 25477533 | 25477615 | SNORD115-34 | | | | | |
| 15 | 25479393 | 25479475 | SNORD115-35 | | | | | |
| 15 | 25481231 | 25481313 | SNORD115-11 | | | | | |
| 15 | 25481231 | 25481313 | SNORD115-29 | | | | | |
| 15 | 25481231 | 25481313 | SNORD115-36 | | | | | |
| 15 | 25481231 | 25481313 | SNORD115-43 | | | | | |
| 15 | 25483132 | 25483214 | SNORD115-37 | | | | | |
| 15 | 25484984 | 25485066 | SNORD115-38 | | | | | |
| 15 | 25486892 | 25486974 | SNORD115-39 | | | | | |
| 15 | 25488760 | 25488842 | SNORD115-40 | | | | | |
| 15 | 25490624 | 25490706 | SNORD115-41 | | | | | |
| 15 | 25492491 | 25492573 | SNORD115-10 | | | | | |
| 15 | 25492491 | 25492573 | SNORD115-42 | | | | | |
| 15 | 25494344 | 25494426 | SNORD115-11 | | | | | |
| 15 | 25494344 | 25494426 | SNORD115-29 | | | | | |
| 15 | 25494344 | 25494426 | SNORD115-36 | | | | | |
| 15 | 25494344 | 25494426 | SNORD115-43 | | | | | |

| | | | | | | | | |
|---|---|---|---|---|---|---|---|---|
| 15 | 25496005 | 25496087 | SNORD115-44 | | | | | |
| 15 | 25509673 | 25509726 | SNORD115-45 | | | | | |
| 15 | 25511737 | 25511808 | SNORD115-46 | | | | | |
| 15 | 25513663 | 25513696 | SNORD115-47 | | | | | |
| 15 | 25514929 | 25515005 | SNORD115-48 | | | | | |
| 15 | 25523489 | 25523556 | SNORD109A | | | | | |
| 15 | 25523489 | 25523556 | SNORD109B | | | | | |
| 15 | 25582395 | 25684175 | UBE3A | | | UBE3A | | |
| 15 | 25923859 | 26108349 | ATP10A | | ATP10A | ATP10A | | |
| 15 | 26093893 | 26093972 | MIR4715 | | | | | |
| 15 | 26147506 | 26298267 | LOC100128714 | | | | | |
| 15 | 26360959 | 26378184 | LINC00929 | | | | | |
| 15 | 26788693 | 26874325 | GABRB3 | GABRB3 | GABRB3 | GABRB3 | GABRB3 | GABRB3 |
| 15 | 27111865 | 27194357 | GABRA5 | GABRA5 | GABRA5 | GABRA5 | GABRA5 | GABRA5 |
| 15 | 27216428 | 27406466 | GABRG3 | | GABRG3 | GABRG3 | GABRG3 | GABRG3 |
| 15 | 28000020 | 28344458 | OCA2 | | | OCA2 | | |
| 15 | 28356182 | 28567298 | HERC2 | | | | | |
| 15 | 28623783 | 28636412 | GOLGA8F | | | | | |
| 15 | 28623783 | 28637171 | GOLGA8G | | | | | |
| 15 | 28671636 | 28735991 | MIR4509 | | | | | |
| 15 | 28764756 | 28778143 | GOLGA8G | | | | | |
| 15 | 28765515 | 28778143 | GOLGA8F | | | | | |
| 15 | 28899587 | 28930410 | HERC2P9 | | | | | |
| 15 | 28943840 | 28957567 | GOLGA8M | | | | | |
| 15 | 28982728 | 29003508 | WHAMMP2 | | | | | |
| 15 | 29033388 | 29034538 | LOC100289656 | | | | | |
| 15 | 29034979 | 29101720 | PDCD6IPP2 | | | | | |
| 15 | 29090106 | 29093821 | GOLGA6L7P | | | | | |
| 15 | 29213839 | 29410516 | APBA2 | | | APBA2 | | |
| 15 | 29412454 | 29862927 | FAM189A1 | | | | | |
| 15 | 29560352 | 29562020 | NSMCE3 | | | | | |
| 15 | 29991570 | 30261002 | TJP1 | | | | | |
| 15 | 30375157 | 30388904 | GOLGA8J | | | | | |
| 15 | 30395934 | 30423948 | ULK4P3 | | | | | |
| 15 | 30427989 | 30439395 | GOLGA8T | | | | | |
| 15 | 30488238 | 30506743 | DKFZP434L187 | | | | | |
| 15 | 30653442 | 30685864 | CHRFAM7A | | | | | |
| 15 | 30692764 | 30706463 | GOLGA8R | | | | | |
| 15 | 30864757 | 30892911 | ULK4P1 | | | | | |
| 15 | 30864757 | 30892911 | ULK4P2 | | | | | |
| 15 | 30896232 | 30910029 | GOLGA8H | | | | | |
| 15 | 30918878 | 30931013 | ARHGAP11B | | | | | |
| 15 | 30938317 | 31065209 | LOC100288637 | | | | | |
| 15 | 31110238 | 31119986 | HERC2P10 | | | | | |
| 15 | 31196054 | 31235310 | FAN1 | | | | | |
| 15 | 31231143 | 31283807 | MTMR10 | | | | | |
| 15 | 31293263 | 31453476 | TRPM1 | | TRPM1 | | | |
| 15 | 31357234 | 31357344 | MIR211 | | | | | |
| 15 | 31361592 | 31393929 | TRPM1 | | TRPM1 | | | |
| 15 | 31514970 | 31523050 | LOC283710 | | | | | |
| 15 | 31619057 | 31727868 | KLF13 | | | | | |
| 15 | 31775328 | 31947542 | OTUD7A | | | | | |
| 15 | 32322685 | 32462384 | CHRNA7 | CHRNA7 | | CHRNA7 | | CHRNA7 |
| 15 | 32681785 | 32695493 | GOLGA8K | | | | | |
| 15 | 32698800 | 32727065 | ULK4P3 | | | | | |
| 15 | 32698811 | 32727250 | ULK4P1 | | | | | |
| 15 | 32698811 | 32727250 | ULK4P2 | | | | | |
| 15 | 32734114 | 32747835 | GOLGA8O | | | | | |
| 15 | 32812048 | 32825942 | WHAMMP1 | | | | | |
| 15 | 32828959 | 32872810 | LOC100996255 | | | | | |
| 15 | 32885656 | 32899511 | GOLGA8N | | | | | |
| 15 | 32907344 | 32932150 | ARHGAP11A | | | | | |
| 15 | 32933869 | 32989298 | SCG5 | | | | | |
| 15 | 33010204 | 33026870 | GREM1 | | | | | |
| 15 | 33010301 | 33011066 | LOC100131315 | | | | | |
| 15 | 33057744 | 33486934 | FMN1 | | | | | |

| | | | | | | | | |
|---|---|---|---|---|---|---|---|---|
| 15 | 33528676 | 33539756 | TMCO5B | | | | | |
| 15 | 33595857 | 33602860 | LOC101928134 | | | | | |
| 15 | 33603162 | 34158304 | RYR3 | | RYR3 | RYR3 | RYR3 | |
| 15 | 34158427 | 34331303 | AVEN | | | | | |
| 15 | 34261088 | 34357287 | CHRM5 | | | CHRM5 | | |
| 15 | 34376223 | 34394053 | EMC7 | | | | | |
| 15 | 34394273 | 34396591 | PGBD4 | | | | | |
| 15 | 34432874 | 34502297 | KATNBL1 | | | | | |
| 15 | 34517197 | 34522366 | EMC4 | | | | | |
| 15 | 34522196 | 34630265 | SLC12A6 | | | SLC12A6 | | |
| 15 | 34633916 | 34635362 | NOP10 | | | | | |
| 15 | 34635515 | 34649938 | NUTM1 | | | | | |
| 15 | 34651088 | 34659395 | LPCAT4 | | | | | |
| 15 | 34671269 | 34729667 | GOLGA8A | | | | | |
| 15 | 34674269 | 34674351 | MIR1233 | | | | | |
| 15 | 34817483 | 34875771 | GOLGA8B | | | | | |
| 15 | 34820490 | 34820572 | MIR1233 | | | | | |
| 15 | 35044641 | 35046782 | GJD2 | | | | | |
| 15 | 35047284 | 35105124 | LOC101928174 | | | | | |
| 15 | 35080296 | 35087927 | ACTC1 | | | | | |
| 15 | 35148551 | 35261995 | AQR | | | | | |
| 15 | 35270541 | 35280497 | ZNF770 | | | | | |
| 15 | 35529526 | 35530264 | ANP32AP1 | | | | | |
| 15 | 35663169 | 35838404 | DPH6 | | | | | |
| 15 | 35664456 | 35664565 | MIR3942 | | | | | |
| 15 | 35812473 | 36151202 | DPH6 | | | | | |
| 15 | 36219056 | 36219124 | MIR4510 | | | | | |
| 15 | 36871803 | 37102461 | C15orf41 | | | | | |
| 15 | 37091300 | 37110707 | CSNK1A1P1 | | | | | |
| 15 | 37156643 | 37178734 | LOC145845 | | | | | |
| 15 | 37183221 | 37393500 | MEIS2 | | | | | |
| 15 | 37265021 | 37265102 | MIR8063 | | | | | |
| 15 | 38226807 | 38243623 | TMCO5A | | | | | |
| 15 | 38332593 | 38354541 | LOC101928227 | | | | | |
| 15 | 38545051 | 38649450 | SPRED1 | | SPRED1 | | | |
| 15 | 38746327 | 38779911 | FAM98B | | | | | |
| 15 | 38780298 | 38857007 | RASGRP1 | | | | | |
| 15 | 38988798 | 38992239 | C15orf53 | | | | | |
| 15 | 39542869 | 39547043 | C15orf54 | | | | | |
| 15 | 39873279 | 39891121 | THBS1 | | | | | |
| 15 | 39892231 | 40075039 | FSIP1 | | | | | |
| 15 | 40091222 | 40213093 | GPR176 | | | | | |
| 15 | 40226324 | 40327797 | EIF2AK4 | | | | | |
| 15 | 40327890 | 40359710 | SRP14 | | | | | |
| 15 | 40380090 | 40401075 | BMF | | | | | |
| 15 | 40453209 | 40513337 | BUB1B | | | | | |
| 15 | 40509628 | 40569688 | BUB1B-PAK6 | | | | | |
| 15 | 40531291 | 40569688 | PAK6 | | | | | |
| 15 | 40542865 | 40545110 | C15orf56 | | | | | |
| 15 | 40545378 | 40569688 | PAK6 | | | | | |
| 15 | 40570572 | 40574787 | ANKRD63 | | | | | |
| 15 | 40580097 | 40600174 | PLCB2 | | | PLCB2 | | |
| 15 | 40616256 | 40618916 | INAFM2 | | | | | |
| 15 | 40623652 | 40633168 | C15orf52 | | | | | |
| 15 | 40643233 | 40648634 | PHGR1 | | | | | |
| 15 | 40650435 | 40663257 | DISP2 | | | | | |
| 15 | 40674921 | 40686489 | KNSTRN | | | | | |
| 15 | 40697685 | 40713512 | IVD | | | IVD | | |
| 15 | 40731919 | 40760441 | BAHD1 | | | | | |
| 15 | 40763159 | 40765357 | CHST14 | | | | | |
| 15 | 40823539 | 40857256 | C15orf57 | | | | | |
| 15 | 40824082 | 40824749 | MRPL42P5 | | | | | |
| 15 | 40845111 | 40857256 | C15orf57 | | | | | |
| 15 | 40861491 | 40866893 | RPUSD2 | | | | | |
| 15 | 40886446 | 40954881 | CASC5 | | | CASC5 | | |
| 15 | 40985952 | 41024356 | RAD51 | | | | | |

| | | | | | | | | |
|---|---|---|---|---|---|---|---|---|
| 15 | 41028081 | 41047534 | RMDN3 | | | | | |
| 15 | 41056284 | 41059911 | GCHFR | | | | | |
| 15 | 41060066 | 41099676 | DNAJC17 | | | | | |
| 15 | 41062158 | 41064648 | C15orf62 | | | | | |
| 15 | 41099273 | 41106767 | ZFYVE19 | | | | | |
| 15 | 41107642 | 41120907 | PPP1R14D | | | | | |
| 15 | 41136245 | 41149853 | SPINT1 | | | | | |
| 15 | 41164411 | 41166487 | RHOV | | | | | |
| 15 | 41186627 | 41196173 | VPS18 | | | | | |
| 15 | 41221530 | 41231258 | DLL4 | | | | | |
| 15 | 41245635 | 41248717 | CHAC1 | | | | | |
| 15 | 41271078 | 41408444 | INO80 | | | | | |
| 15 | 41474930 | 41522955 | EXD1 | | | | | |
| 15 | 41523436 | 41574083 | CHP1 | | | | | |
| 15 | 41576200 | 41624819 | OIP5 | | | | | |
| 15 | 41624891 | 41673248 | NUSAP1 | | | | | |
| 15 | 41679546 | 41694658 | NDUFAF1 | | | | | |
| 15 | 41709301 | 41775761 | RTF1 | | | | | |
| 15 | 41786055 | 41795757 | ITPKA | | | | | |
| 15 | 41795839 | 41806085 | LTK | | | | | |
| 15 | 41809374 | 41836475 | RPAP1 | | | | | |
| 15 | 41851219 | 41871536 | TYRO3 | | TYRO3 | TYRO3 | | |
| 15 | 41952609 | 42062141 | MGA | | | | | |
| 15 | 41983782 | 41983876 | MIR626 | | | | | |
| 15 | 42066631 | 42120053 | MAPKBP1 | | | | | |
| 15 | 42120282 | 42129785 | JMJD7 | | | | | |
| 15 | 42120282 | 42140346 | JMJD7-PLA2G4B | | | | | |
| 15 | 42131010 | 42140346 | PLA2G4B | | | | | |
| 15 | 42140343 | 42186275 | SPTBN5 | | | SPTBN5 | | |
| 15 | 42158692 | 42158749 | MIR4310 | | | | | |
| 15 | 42191638 | 42221484 | EHD4 | | | | | |
| 15 | 42264960 | 42342901 | PLA2G4E | | | | | |
| 15 | 42359880 | 42386752 | PLA2G4D | | | | | |
| 15 | 42433331 | 42448839 | PLA2G4F | | | | | |
| 15 | 42450898 | 42500524 | VPS39 | | | VPS39 | | |
| 15 | 42491767 | 42491864 | MIR627 | | | | | |
| 15 | 42502649 | 42565782 | TMEM87A | | | | | |
| 15 | 42565855 | 42645864 | GANC | | | | | |
| 15 | 42651697 | 42704515 | CAPN3 | | CAPN3 | CAPN3 | | |
| 15 | 42704634 | 42783395 | ZNF106 | | | | | |
| 15 | 42787503 | 42825259 | SNAP23 | | | SNAP23 | | |
| 15 | 42834719 | 42841002 | LRRC57 | | | | | |
| 15 | 42841010 | 42862190 | HAUS2 | | | | | |
| 15 | 42867856 | 43013196 | STARD9 | | | | | |
| 15 | 43015759 | 43029417 | CDAN1 | | | | | |
| 15 | 43036541 | 43213007 | TTBK2 | | TTBK2 | | | |
| 15 | 43235097 | 43398286 | UBR1 | | | | | |
| 15 | 43425721 | 43477341 | TMEM62 | | | | | |
| 15 | 43477465 | 43489375 | CCNDBP1 | | | | | |
| 15 | 43489425 | 43513323 | EPB42 | | | EPB42 | | |
| 15 | 43524792 | 43559055 | TGM5 | | | | | |
| 15 | 43568478 | 43594453 | TGM7 | | | | | |
| 15 | 43619973 | 43622820 | LCMT2 | | | | | |
| 15 | 43622553 | 43646753 | ADAL | | | | | |
| 15 | 43650369 | 43662258 | ZSCAN29 | | | | | |
| 15 | 43663256 | 43699296 | TUBGCP4 | | TUBGCP4 | | | |
| 15 | 43699411 | 43802707 | TP53BP1 | | | | | |
| 15 | 43809805 | 43823818 | MAP1A | | | | | |
| 15 | 43825659 | 43882451 | PPIP5K1 | | PPIP5K1 | | | |
| 15 | 43885251 | 43891604 | CKMT1B | | | CKMT1B | | |
| 15 | 43891760 | 43910998 | STRC | | | | | |
| 15 | 43922759 | 43941043 | CATSPER2 | | | | | |
| 15 | 43985083 | 43991420 | CKMT1A | | | | | |
| 15 | 44028145 | 44038496 | CATSPER2P1 | | | | | |
| 15 | 44038589 | 44064804 | PDIA3 | | | | | |
| 15 | 44064797 | 44069502 | ELL3 | | | | | |

| | | | | | | | | |
|---|---|---|---|---|---|---|---|---|
| 15 | 44069293 | 44088287 | SERF2 | | | | | |
| 15 | 44084173 | 44094769 | SERF2-C15ORF63 | | | | | |
| 15 | 44084574 | 44088287 | SERF2 | | | | | |
| 15 | 44085856 | 44085957 | MIR1282 | | | | | |
| 15 | 44086371 | 44092295 | SERINC4 | | | | | |
| 15 | 44092618 | 44094769 | HYPK | | | | | |
| 15 | 44096732 | 44116951 | MFAP1 | | | | | |
| 15 | 44119111 | 44160617 | WDR76 | | | | | |
| 15 | 44162958 | 44487492 | FRMD5 | | | | | |
| 15 | 44168023 | 44170369 | PIN4P1 | | | | | |
| 15 | 44580908 | 44707959 | CASC4 | | | | | |
| 15 | 44719578 | 44819429 | CTDSPL2 | | | | | |
| 15 | 44826702 | 44855001 | EIF3J | | | | | |
| 15 | 44854893 | 44955876 | SPG11 | | | | | |
| 15 | 44957929 | 44969086 | PATL2 | | | | | |
| 15 | 45003684 | 45010357 | B2M | | | | | |
| 15 | 45021185 | 45025049 | LOC100419583 | | | | | |
| 15 | 45028559 | 45060027 | TRIM69 | | | | | |
| 15 | 45248899 | 45271421 | C15orf43 | | | | | |
| 15 | 45315301 | 45367287 | SORD | | | | | |
| 15 | 45384851 | 45406359 | DUOX2 | | | | | |
| 15 | 45406522 | 45410301 | DUOXA2 | | | | | |
| 15 | 45409563 | 45415241 | DUOXA1 | | | | | |
| 15 | 45422191 | 45457776 | DUOX1 | | | | | |
| 15 | 45459411 | 45493373 | SHF | | | | | |
| 15 | 45543778 | 45571420 | LOC101928414 | | | | | |
| 15 | 45544427 | 45568132 | SLC28A2 | | | | | |
| 15 | 45653321 | 45670980 | GATM | | GATM | | | |
| 15 | 45694518 | 45713616 | SPATA5L1 | | | | | |
| 15 | 45722726 | 45725647 | C15orf48 | | | | | |
| 15 | 45725247 | 45725327 | MIR147B | | | | | |
| 15 | 45774679 | 45815002 | SLC30A4 | | | SLC30A4 | | |
| 15 | 45803333 | 45848928 | HMGN2P46 | | | | | |
| 15 | 45879320 | 45901914 | BLOC1S6 | | | | | |
| 15 | 45923345 | 45983492 | SQRDL | | | | | |
| 15 | 47476402 | 48066420 | SEMA6D | | SEMA6D | | | |
| 15 | 48095580 | 48138433 | LINC01491 | | | | | |
| 15 | 48413168 | 48434589 | SLC24A5 | | SLC24A5 | | | |
| 15 | 48431624 | 48470558 | MYEF2 | | | | | |
| 15 | 48483866 | 48495951 | CTXN2 | | | | | |
| 15 | 48498497 | 48596275 | SLC12A1 | | SLC12A1 | SLC12A1 | | |
| 15 | 48623620 | 48635570 | DUT | | | | | |
| 15 | 48700502 | 48937985 | FBN1 | | | FBN1 | | |
| 15 | 49030134 | 49103343 | CEP152 | | | | | |
| 15 | 49115933 | 49255641 | SHC4 | | | | | |
| 15 | 49170289 | 49172380 | EID1 | | | | | |
| 15 | 49280834 | 49338760 | SECISBP2L | | | | | |
| 15 | 49417470 | 49447854 | COPS2 | | | COPS2 | | |
| 15 | 49447955 | 49622002 | GALK2 | | | | | |
| 15 | 49448494 | 49450822 | NDUFAF4P1 | | | | | |
| 15 | 49461266 | 49461350 | MIR4716 | | | | | |
| 15 | 49462212 | 49622002 | GALK2 | | | | | |
| 15 | 49620591 | 49913118 | FAM227B | | | | | |
| 15 | 49715374 | 49779523 | FGF7 | | | | | |
| 15 | 49913225 | 49937333 | DTWD1 | | | | | |
| 15 | 50150434 | 50411419 | ATP8B4 | | | | | |
| 15 | 50474392 | 50528589 | SLC27A2 | | | | | |
| 15 | 50534145 | 50558162 | HDC | | | | | |
| 15 | 50569388 | 50647605 | GABPB1 | | | | | |
| 15 | 50641134 | 50647076 | FLJ10038 | | | | | |
| 15 | 50646370 | 50650503 | GABPB1 | | | | | |
| 15 | 50652525 | 50652607 | MIR4712 | | | | | |
| 15 | 50716573 | 50793280 | USP8 | | | | | |
| 15 | 50792758 | 50838902 | USP50 | | | | | |
| 15 | 50849355 | 50979012 | TRPM7 | | | | | |
| 15 | 50999736 | 51057910 | SPPL2A | | | | | |

| | | | | | | | | |
|---|---|---|---|---|---|---|---|---|
| 15 | 51200868 | 51298097 | AP4E1 | | | AP4E1 | | |
| 15 | 51236325 | 51238762 | DCAF13P3 | | | | | |
| 15 | 51348798 | 51397473 | TNFAIP8L3 | | | | | |
| 15 | 51500253 | 51630795 | CYP19A1 | | | | | |
| 15 | 51534386 | 51534461 | MIR4713 | | | | | |
| 15 | 51606228 | 51606306 | MIR7973 | | | | | |
| 15 | 51633712 | 51700209 | GLDN | | | | | |
| 15 | 51739920 | 51914967 | DMXL2 | | DMXL2 | | | |
| 15 | 51973549 | 52013223 | SCG3 | | SCG3 | | | |
| 15 | 52015260 | 52043650 | LYSMD2 | | | | | |
| 15 | 52043757 | 52108558 | TMOD2 | | | | | |
| 15 | 52121824 | 52204331 | TMOD3 | | | | | |
| 15 | 52211159 | 52216317 | LOC100422556 | | | | | |
| 15 | 52230221 | 52263998 | LEO1 | | | | | |
| 15 | 52311410 | 52358462 | MAPK6 | | | MAPK6 | | |
| 15 | 52401465 | 52404972 | BCL2L10 | | | | | |
| 15 | 52413122 | 52483565 | GNB5 | | | | | |
| 15 | 52472222 | 52498076 | LOC100129973 | | | | | |
| 15 | 52484514 | 52587995 | MYO5C | | | | | |
| 15 | 52569313 | 52569397 | MIR1266 | | | | | |
| 15 | 52599479 | 52821247 | MYO5A | | MYO5A | MYO5A | | |
| 15 | 52839241 | 52861643 | ARPP19 | | | | | |
| 15 | 52873517 | 52970831 | FAM214A | | | | | |
| 15 | 53049159 | 53082209 | ONECUT1 | | | | | |
| 15 | 53805937 | 54055075 | WDR72 | | | | | |
| 15 | 54305100 | 54920806 | UNC13C | | | | | |
| 15 | 55473511 | 55489231 | RSL24D1 | | | | | |
| 15 | 55495163 | 55582013 | RAB27A | | | RAB27A | | |
| 15 | 55609381 | 55611392 | PIGBOS1 | | | | | |
| 15 | 55611132 | 55647846 | PIGB | | | | | |
| 15 | 55647420 | 55700708 | CCPG1 | | | | | |
| 15 | 55647420 | 55790782 | DYX1C1-CCPG1 | | | | | |
| 15 | 55665137 | 55665232 | MIR628 | | | | | |
| 15 | 55700722 | 55710910 | C15orf65 | | | | | |
| 15 | 55709953 | 55800432 | DYX1C1 | | | | | |
| 15 | 55831084 | 55881050 | PYGO1 | | | | | |
| 15 | 55903738 | 56035317 | PRTG | | | | | |
| 15 | 56119116 | 56285944 | NEDD4 | | | | | |
| 15 | 56382730 | 56535483 | RFX7 | | | | | |
| 15 | 56657621 | 56738195 | TEX9 | | | | | |
| 15 | 56720928 | 56757335 | MNS1 | | | | | |
| 15 | 56922373 | 57026284 | ZNF280D | | | | | |
| 15 | 57178367 | 57210697 | LOC145783 | | | | | |
| 15 | 57210832 | 57580714 | TCF12 | | | | | |
| 15 | 57592562 | 57599967 | LINC00926 | | | | | |
| 15 | 57611335 | 57617237 | LINC01413 | | | | | |
| 15 | 57668702 | 57842925 | CGNL1 | | | | | |
| 15 | 57884101 | 57977562 | MYZAP | | | | | |
| 15 | 57884101 | 58009755 | GCOM1 | | | | | |
| 15 | 57998718 | 58009755 | POLR2M | | | | | |
| 15 | 58245621 | 58358121 | ALDH1A2 | | | | | |
| 15 | 58430407 | 58478110 | AQP9 | | | | | |
| 15 | 58724174 | 58861073 | LIPC | | | | | |
| 15 | 58727099 | 58790934 | LOC101928694 | | | | | |
| 15 | 58887402 | 59042177 | ADAM10 | | | | | |
| 15 | 58982671 | 58985806 | HSP90AB4P | | | | | |
| 15 | 59063392 | 59149734 | FAM63B | | | | | |
| 15 | 59171243 | 59225875 | SLTM | | | | | |
| 15 | 59279864 | 59389618 | RNF111 | | | | | |
| 15 | 59397283 | 59417244 | CCNB2 | | | | | |
| 15 | 59428167 | 59665071 | MYO1E | | | | | |
| 15 | 59463381 | 59463461 | MIR2116 | | | | | |
| 15 | 59499014 | 59500785 | LDHAL6B | | | | | |
| 15 | 59730371 | 59815751 | FAM81A | | | | | |
| 15 | 59903981 | 59912210 | GCNT3 | | | | | |
| 15 | 59930260 | 59949737 | GTF2A2 | | | | | |

| | | | | | | | | |
|---|---|---|---|---|---|---|---|---|
| 15 | 59955061 | 59981642 | BNIP2 | | | | | |
| 15 | 60296420 | 60298142 | FOXB1 | | | | | |
| 15 | 60639349 | 60690185 | ANXA2 | | | ANXA2 | | |
| 15 | 60711807 | 60771359 | ICE2 | | | | | |
| 15 | 60771376 | 60979448 | RORA | | | | | |
| 15 | 62144589 | 62352664 | VPS13C | | | | | |
| 15 | 62359175 | 62363116 | C2CD4A | | | | | |
| 15 | 62455736 | 62457482 | C2CD4B | | | | | |
| 15 | 62596856 | 62596933 | MIR8067 | | | | | |
| 15 | 62635227 | 62635337 | MIR6085 | | | | | |
| 15 | 62929370 | 62937380 | MGC15885 | | | | | |
| 15 | 62939509 | 63136829 | TLN2 | | TLN2 | TLN2 | | TLN2 |
| 15 | 63116155 | 63116240 | MIR190A | | | | | |
| 15 | 63334837 | 63364113 | TPM1 | | | | | |
| 15 | 63413998 | 63434264 | LACTB | | | | | |
| 15 | 63445538 | 63449741 | RPS27L | | | | | |
| 15 | 63481727 | 63559973 | RAB8B | | | | | |
| 15 | 63569748 | 63601325 | APH1B | | | | | |
| 15 | 63615729 | 63674309 | CA12 | | | | | |
| 15 | 63682428 | 63729735 | LOC102723344 | | | | | |
| 15 | 63796709 | 63893026 | USP3 | | | | | |
| 15 | 63889551 | 63894620 | FBXL22 | | | | | |
| 15 | 63900816 | 64126147 | HERC1 | | | | | |
| 15 | 64163128 | 64163218 | MIR422A | | | | | |
| 15 | 64199234 | 64338521 | DAPK2 | | | | | |
| 15 | 64220472 | 64228152 | LOC101928988 | | | | | |
| 15 | 64364757 | 64386207 | FAM96A | | | | | |
| 15 | 64388082 | 64436433 | SNX1 | | | | | |
| 15 | 64443915 | 64449680 | SNX22 | | SNX22 | | | |
| 15 | 64448013 | 64455354 | PPIB | | | | | |
| 15 | 64457715 | 64648442 | CSNK1G1 | | | | | |
| 15 | 64657210 | 64679886 | KIAA0101 | | | | | |
| 15 | 64680002 | 64747502 | TRIP4 | | | | | |
| 15 | 64791618 | 64978266 | ZNF609 | | | | | |
| 15 | 64979772 | 64995480 | OAZ2 | | | | | |
| 15 | 65032094 | 65067770 | RBPMS2 | | | | | |
| 15 | 65054585 | 65054714 | MIR1272 | | | | | |
| 15 | 65107828 | 65117867 | PIF1 | | | | | |
| 15 | 65134081 | 65160201 | PLEKHO2 | | | | | |
| 15 | 65204100 | 65251041 | ANKDD1A | | | | | |
| 15 | 65255361 | 65282284 | SPG21 | | | | | |
| 15 | 65293849 | 65321977 | MTFMT | | | MTFMT | | |
| 15 | 65337707 | 65345734 | SLC51B | | | | | |
| 15 | 65345674 | 65360450 | RASL12 | | | | | |
| 15 | 65369153 | 65372276 | KBTBD13 | | | | | |
| 15 | 65385341 | 65398697 | UBAP1L | | | | | |
| 15 | 65409716 | 65426174 | PDCD7 | | | | | |
| 15 | 65440557 | 65477758 | CLPX | | | | | |
| 15 | 65488336 | 65503840 | CILP | | | | | |
| 15 | 65550431 | 65579181 | PARP16 | | | | | |
| 15 | 65619464 | 65670378 | IGDCC3 | | | | | |
| 15 | 65673824 | 65715410 | IGDCC4 | | | | | |
| 15 | 65737997 | 65810035 | DPP8 | | | | | |
| 15 | 65822826 | 65870693 | HACD3 | | | | | |
| 15 | 65871095 | 65903627 | VWA9 | | | | | |
| 15 | 65903742 | 65948598 | SLC24A1 | | | | | |
| 15 | 65952956 | 66084631 | DENND4A | | | | | |
| 15 | 66011583 | 66011670 | MIR4511 | | | | | |
| 15 | 66161796 | 66184329 | RAB11A | | | RAB11A | | |
| 15 | 66187633 | 66546075 | MEGF11 | | | | | |
| 15 | 66332570 | 66332670 | MIR4311 | | | | | |
| 15 | 66585632 | 66626236 | DIS3L | | | | | |
| 15 | 66629007 | 66649054 | TIPIN | | | | | |
| 15 | 66639543 | 66639680 | SCARNA14 | | | | | |
| 15 | 66679210 | 66783882 | MAP2K1 | | | MAP2K1 | | |
| 15 | 66782665 | 66790146 | SNAPC5 | | | | | |

| | | | | | | | | | |
|---|---|---|---|---|---|---|---|---|---|
| 15 | 66789295 | 66789372 | MIR4512 | | | | | | |
| 15 | 66791652 | 66797193 | RPL4 | | | | | | |
| 15 | 66793589 | 66793656 | SNORD18C | | | | | | |
| 15 | 66794359 | 66794429 | SNORD18B | | | | | | |
| 15 | 66795148 | 66795248 | SNORD16 | | | | | | |
| 15 | 66795582 | 66795652 | SNORD18A | | | | | | |
| 15 | 66797420 | 66841822 | ZWILCH | | | | | | |
| 15 | 66839805 | 66858317 | LCTL | | | | | | |
| 15 | 66874527 | 66978136 | LINC01169 | | | | | | |
| 15 | 66994673 | 67074337 | SMAD6 | | | | | | |
| 15 | 67358194 | 67487533 | SMAD3 | | | SMAD3 | | | |
| 15 | 67493012 | 67547536 | AAGAB | | | | | | |
| 15 | 67547137 | 67814182 | IQCH | | | | | | |
| 15 | 67813521 | 67819641 | C15orf61 | | | | | | |
| 15 | 67835020 | 68099455 | MAP2K5 | | | MAP2K5 | | | |
| 15 | 68112041 | 68126174 | SKOR1 | | | | | | |
| 15 | 68126645 | 68131217 | LOC101929076 | | | | | | |
| 15 | 68132276 | 68132383 | RNU6-2 | | | | | | |
| 15 | 68346571 | 68480404 | PIAS1 | | | PIAS1 | | | |
| 15 | 68483042 | 68498448 | CALML4 | | | | | | |
| 15 | 68499329 | 68522080 | CLN6 | | | | | | |
| 15 | 68570140 | 68588203 | FEM1B | | | | | | |
| 15 | 68594041 | 68724492 | ITGA11 | | | | | | |
| 15 | 68871307 | 69020144 | CORO2B | | | | | | |
| 15 | 69070874 | 69113261 | ANP32A | | | | | | |
| 15 | 69094188 | 69094264 | MIR4312 | | | | | | |
| 15 | 69096159 | 69099440 | ANP32A-IT1 | | | | | | |
| 15 | 69116302 | 69489862 | MIR548H4 | | | | | | |
| 15 | 69222838 | 69239150 | SPESP1 | | | | | | |
| 15 | 69222838 | 69349501 | NOX5 | | | | | | |
| 15 | 69373189 | 69388163 | EWSAT1 | | | | | | |
| 15 | 69452972 | 69564544 | GLCE | | | | | | |
| 15 | 69591293 | 69699976 | PAQR5 | | | | | | |
| 15 | 69689242 | 69707368 | LOC145694 | | | | | | |
| 15 | 69706584 | 69740766 | KIF23 | | | | | | |
| 15 | 69745158 | 69747884 | RPLP1 | | | | | | |
| 15 | 69854058 | 69863779 | DRAIC | | | | | | |
| 15 | 69884538 | 69988091 | PCAT29 | | | | | | |
| 15 | 70127572 | 70135306 | LINC00593 | | | | | | |
| 15 | 70340129 | 70390256 | TLE3 | | | | | | |
| 15 | 70371710 | 70371807 | MIR629 | | | | | | |
| 15 | 70907885 | 70908906 | SALRNA3 | | | | | | |
| 15 | 70927587 | 70929653 | SALRNA2 | | | | | | |
| 15 | 70946892 | 71055850 | UACA | | | | | | |
| 15 | 71123862 | 71146604 | LARP6 | | | | | | |
| 15 | 71145577 | 71342436 | LRRC49 | | | | | | |
| 15 | 71173680 | 71184772 | THAP10 | | | | | | |
| 15 | 71184781 | 71342436 | LRRC49 | | | | | | |
| 15 | 71402582 | 71407839 | CT62 | | | | | | |
| 15 | 71433787 | 71983878 | THSD4 | | | | | | |
| 15 | 72102887 | 72110597 | NR2E3 | | | | | | |
| 15 | 72118360 | 72410440 | MYO9A | | | | | | |
| 15 | 72406598 | 72433311 | SENP8 | | | | | | |
| 15 | 72452146 | 72490136 | GRAMD2 | | | | | | |
| 15 | 72491369 | 72523965 | PKM | | | | | | |
| 15 | 72533521 | 72563628 | PARP6 | | | | | | |
| 15 | 72577067 | 72612525 | CELF6 | | | | | | |
| 15 | 72635775 | 72671129 | HEXA | | | HEXA | | | |
| 15 | 72690667 | 72700708 | TMEM202 | | | | | | |
| 15 | 72766666 | 72878896 | ARIH1 | | | | | | |
| 15 | 72879557 | 72879654 | MIR630 | | | | | | |
| 15 | 72947037 | 72959738 | GOLGA6B | | | | | | |
| 15 | 72968122 | 72978490 | HIGD2B | | | | | | |
| 15 | 72978519 | 73030817 | BBS4 | | | | | | |
| 15 | 73043707 | 73090540 | ADPGK | | | | | | |
| 15 | 73344824 | 73597547 | NEO1 | | NEO1 | NEO1 | | | |

| | | | | | | | | |
|---|---|---|---|---|---|---|---|---|
| 15 | 73612199 | 73661605 | HCN4 | | | HCN4 | | |
| 15 | 73735498 | 73852353 | REC114 | | | | | |
| 15 | 73852343 | 73925753 | NPTN | | | | | |
| 15 | 73859278 | 73861884 | NPTN-IT1 | | | | | |
| 15 | 73976621 | 74006859 | CD276 | | | | | |
| 15 | 74027771 | 74062143 | C15orf59 | | | | | |
| 15 | 74165926 | 74181556 | TBC1D21 | | | | | |
| 15 | 74209808 | 74244482 | LOXL1 | | | | | |
| 15 | 74275558 | 74286963 | STOML1 | | | | | |
| 15 | 74287013 | 74340155 | PML | | | | | |
| 15 | 74362197 | 74374891 | GOLGA6A | | | | | |
| 15 | 74418713 | 74421619 | LOC283731 | | | | | |
| 15 | 74421714 | 74429143 | ISLR2 | | | | | |
| 15 | 74466086 | 74469212 | ISLR | | | | | |
| 15 | 74471807 | 74501371 | STRA6 | | | | | |
| 15 | 74528629 | 74628482 | CCDC33 | | | | | |
| 15 | 74630102 | 74660081 | CYP11A1 | | | | | |
| 15 | 74653892 | 74654382 | LOC729739 | | | | | |
| 15 | 74701629 | 74726299 | SEMA7A | | | | | |
| 15 | 74703697 | 74703773 | MIR6881 | | | | | |
| 15 | 74738317 | 74773633 | UBL7 | | | | | |
| 15 | 74833517 | 74890472 | ARID3B | | | | | |
| 15 | 74900712 | 74922542 | CLK3 | | | CLK3 | | |
| 15 | 74922898 | 74988386 | EDC3 | | | | | |
| 15 | 75011882 | 75017951 | CYP1A1 | | | | | |
| 15 | 75041183 | 75048941 | CYP1A2 | | | | | |
| 15 | 75074424 | 75095539 | CSK | | | | | |
| 15 | 75081012 | 75081098 | MIR4513 | | | | | |
| 15 | 75105193 | 75118099 | LMAN1L | | | | | |
| 15 | 75118950 | 75124136 | CPLX3 | | | | | |
| 15 | 75128456 | 75135552 | ULK3 | | | | | |
| 15 | 75132982 | 75133048 | MIR6882 | | | | | |
| 15 | 75137196 | 75165670 | SCAMP2 | | | | | |
| 15 | 75182351 | 75191798 | MPI | | MPI | | | |
| 15 | 75192327 | 75199462 | FAM219B | | | | | |
| 15 | 75212616 | 75230495 | COX5A | | | | | |
| 15 | 75247442 | 75249775 | RPP25 | | | | | |
| 15 | 75287875 | 75313836 | SCAMP5 | | | SCAMP5 | | |
| 15 | 75315895 | 75343067 | PPCDC | | | | | |
| 15 | 75494220 | 75504510 | C15orf39 | | | | | |
| 15 | 75550898 | 75565796 | GOLGA6C | | | | | |
| 15 | 75575181 | 75588148 | GOLGA6D | | | | | |
| 15 | 75628336 | 75632618 | COMMD4 | | | | | |
| 15 | 75639330 | 75647592 | NEIL1 | | | | | |
| 15 | 75645951 | 75646026 | MIR631 | | | | | |
| 15 | 75648132 | 75660968 | MAN2C1 | | | | | |
| 15 | 75661719 | 75748124 | SIN3A | | | | | |
| 15 | 75759461 | 75871632 | PTPN9 | | | | | |
| 15 | 75890423 | 75918719 | SNUPN | | | | | |
| 15 | 75931425 | 75932664 | IMP3 | | | | | |
| 15 | 75941347 | 75950968 | SNX33 | | | | | |
| 15 | 75966662 | 76005189 | CSPG4 | | | CSPG4 | | |
| 15 | 76016318 | 76020027 | ODF3L1 | | | | | |
| 15 | 76020010 | 76032418 | DNM1P35 | | | | | |
| 15 | 76054555 | 76054656 | MIR4313 | | | | | |
| 15 | 76135626 | 76193388 | UBE2Q2 | | | | | |
| 15 | 76196199 | 76225471 | FBXO22 | | | | | |
| 15 | 76234327 | 76304785 | NRG4 | | | | | |
| 15 | 76352298 | 76497304 | TMEM266 | | | | | |
| 15 | 76467231 | 76473827 | LOC101929439 | | | | | |
| 15 | 76508628 | 76603810 | ETFA | | | ETFA | | |
| 15 | 76551629 | 76552493 | TYRO3P | | | | | |
| 15 | 76629064 | 76634816 | ISL2 | | | ISL2 | | |
| 15 | 76640526 | 77176217 | SCAPER | | | | | |
| 15 | 76878987 | 76879032 | MIR3713 | | | | | |
| 15 | 77223961 | 77242601 | RCN2 | | | | | |

| | | | | | | | | |
|---|---|---|---|---|---|---|---|---|
| 15 | 77287464 | 77329671 | PSTPIP1 | | | | | |
| 15 | 77336359 | 77363570 | TSPAN3 | | | | | |
| 15 | 77400497 | 77712446 | PEAK1 | | | | | |
| 15 | 77516249 | 77517746 | LINC00597 | | | | | |
| 15 | 77712992 | 77777946 | HMG20A | | | | | |
| 15 | 77905365 | 77960416 | LINGO1 | | | | | |
| 15 | 78206558 | 78219188 | LOC645752 | | | | | |
| 15 | 78285574 | 78286567 | LOC91450 | | | | | |
| 15 | 78287326 | 78369994 | TBC1D2B | | | | | |
| 15 | 78375874 | 78375901 | MIR5003 | | | | | |
| 15 | 78384926 | 78396393 | SH2D7 | | | | | |
| 15 | 78396947 | 78423877 | CIB2 | | | | | |
| 15 | 78441718 | 78462884 | IDH3A | | | IDH3A | | |
| 15 | 78463186 | 78527049 | ACSBG1 | | | | | |
| 15 | 78556486 | 78574538 | DNAJA4 | | | | | |
| 15 | 78575577 | 78592068 | WDR61 | | | | | |
| 15 | 78632665 | 78640572 | CRABP1 | | | | | |
| 15 | 78730517 | 78793798 | IREB2 | | | | | |
| 15 | 78799905 | 78829715 | HYKK | | | | | |
| 15 | 78832746 | 78841563 | PSMA4 | | | | | |
| 15 | 78857861 | 78887611 | CHRNA5 | | | CHRNA5 | CHRNA5 | CHRNA5 |
| 15 | 78885394 | 78913637 | CHRNA3 | | CHRNA3 | | CHRNA3 | CHRNA3 |
| 15 | 78916635 | 78933587 | CHRNB4 | | | | | |
| 15 | 79044378 | 79045734 | LOC646938 | | | | | |
| 15 | 79051544 | 79103773 | ADAMTS7 | | | | | |
| 15 | 79165122 | 79190081 | MORF4L1 | | | | | |
| 15 | 79214091 | 79237440 | CTSH | | | | | |
| 15 | 79252288 | 79383215 | RASGRF1 | | | RASGRF1 | | |
| 15 | 79484048 | 79576287 | ANKRD34C | | | | | |
| 15 | 79502129 | 79502213 | MIR184 | | | | | |
| 15 | 79575145 | 79590581 | ANKRD34C | | | | | |
| 15 | 79603403 | 79677061 | TMED3 | | | | | |
| 15 | 79724857 | 79764642 | KIAA1024 | | | | | |
| 15 | 80135888 | 80189627 | MTHFS | | | | | |
| 15 | 80135888 | 80215448 | ST20-MTHFS | | | | | |
| 15 | 80191181 | 80217196 | ST20 | | | | | |
| 15 | 80253231 | 80263643 | BCL2A1 | | | | | |
| 15 | 80351909 | 80430735 | ZFAND6 | | | | | |
| 15 | 80445232 | 80478924 | FAH | | | FAH | | |
| 15 | 80487820 | 80544603 | LINC01314 | | | | | |
| 15 | 80555409 | 80634147 | LINC00927 | | | | | |
| 15 | 80696691 | 80890277 | ARNT2 | | | | | |
| 15 | 80846950 | 80855285 | LOC101929586 | | | | | |
| 15 | 80873443 | 80873580 | MIR5572 | | | | | |
| 15 | 80987651 | 81047962 | ABHD17C | | | | | |
| 15 | 81071683 | 81244003 | CEMIP | | | | | |
| 15 | 81134318 | 81134414 | MIR549A | | | | | |
| 15 | 81239667 | 81282219 | MESDC2 | | | | | |
| 15 | 81289757 | 81289814 | MIR4514 | | | | | |
| 15 | 81293294 | 81296345 | MESDC1 | | | | | |
| 15 | 81426643 | 81441516 | CFAP161 | | | | | |
| 15 | 81489218 | 81605104 | IL16 | | | | | |
| 15 | 81605006 | 81616524 | STARD5 | | | | | |
| 15 | 81616673 | 81666418 | TMC3 | | | | | |
| 15 | 82334118 | 82338484 | MEX3B | | | | | |
| 15 | 82380934 | 82390034 | LINC01583 | | | | | |
| 15 | 82422560 | 82555104 | EFL1 | | | | | |
| 15 | 82555151 | 82577267 | SAXO2 | | | | | |
| 15 | 82585620 | 82626915 | ADAMTS7P1 | | | | | |
| 15 | 82632349 | 83018198 | GOLGA6L10 | | | | | |
| 15 | 82647353 | 82707816 | UBE2Q2P2 | | | | | |
| 15 | 82722184 | 82731586 | GOLGA6L9 | | | | | |
| 15 | 82763612 | 82798399 | GOLGA2P10 | | | | | |
| 15 | 82804002 | 83195272 | GOLGA6L17P | | | | | |
| 15 | 82804768 | 83108111 | GOLGA6L9 | | | | | |
| 15 | 82821157 | 82824952 | RPS17 | | | | | |

| | | | | | | | | |
|---|---|---|---|---|---|---|---|---|
| 15 | 82882945 | 82924242 | ADAMTS7P1 | | | | | |
| 15 | 82944745 | 82976263 | GOLGA2P10 | | | | | |
| 15 | 82944749 | 82976258 | LOC727751 | | | | | |
| 15 | 83002903 | 83004442 | LOC102724034 | | | | | |
| 15 | 83023840 | 83084341 | UBE2Q2P2 | | | | | |
| 15 | 83098709 | 83108111 | GOLGA6L9 | | | | | |
| 15 | 83140198 | 83182977 | GOLGA2P10 | | | | | |
| 15 | 83140203 | 83182973 | LOC727751 | | | | | |
| 15 | 83205500 | 83209295 | RPS17 | | | | | |
| 15 | 83211950 | 83361572 | CPEB1 | | | | | |
| 15 | 83328032 | 83378660 | AP3B2 | | | | | |
| 15 | 83379222 | 83382745 | LOC338963 | | | | | |
| 15 | 83394649 | 83408532 | ACTG1P17 | | | | | |
| 15 | 83419315 | 83425958 | SNHG21 | | | | | |
| 15 | 83424116 | 83474822 | FSD2 | | | | | |
| 15 | 83424696 | 83424823 | SCARNA15 | | | | | |
| 15 | 83478379 | 83503613 | WHAMM | | | | | |
| 15 | 83517728 | 83621476 | HOMER2 | | | HOMER2 | HOMER2 | |
| 15 | 83654954 | 83659809 | FAM103A1 | | | | | |
| 15 | 83657714 | 83680393 | C15orf40 | | | | | |
| 15 | 83685180 | 83736106 | BTBD1 | | | | | |
| 15 | 83736086 | 83736167 | MIR4515 | | | | | |
| 15 | 83776323 | 83806111 | TM6SF1 | | | | | |
| 15 | 83806803 | 83876770 | HDGFRP3 | | | | | |
| 15 | 83924654 | 83953468 | BNC1 | | | BNC1 | | |
| 15 | 84115979 | 84287493 | SH3GL3 | | | SH3GL3 | | |
| 15 | 84322837 | 84708593 | ADAMTSL3 | | | | | |
| 15 | 84748938 | 84795353 | EFTUD1P1 | | | | | |
| 15 | 84841241 | 84850985 | UBE2Q2L | | | | | |
| 15 | 84860599 | 84878025 | LOC440300 | | | | | |
| 15 | 84867599 | 84898920 | GOLGA2P7 | | | | | |
| 15 | 84868829 | 84869441 | LOC642423 | | | | | |
| 15 | 84904524 | 84914120 | GOLGA6L4 | | | | | |
| 15 | 84977216 | 84980581 | LOC103171574 | | | | | |
| 15 | 84977315 | 84978856 | LOC102724034 | | | | | |
| 15 | 85045805 | 85050249 | DNM1P41 | | | | | |
| 15 | 85049398 | 85060078 | GOLGA6L5P | | | | | |
| 15 | 85070426 | 85114026 | UBE2Q2P1 | | | | | |
| 15 | 85113879 | 85123412 | LINC00933 | | | | | |
| 15 | 85144248 | 85166947 | ZSCAN2 | | | | | |
| 15 | 85174690 | 85185694 | SCAND2P | | | | | |
| 15 | 85184725 | 85197574 | WDR73 | | | | | |
| 15 | 85198359 | 85201802 | NMB | | | | | |
| 15 | 85212767 | 85259691 | SEC11A | | | | | |
| 15 | 85291817 | 85349663 | ZNF592 | | | | | |
| 15 | 85359910 | 85416713 | ALPK3 | | | | | |
| 15 | 85427891 | 85489027 | SLC28A1 | | | | | |
| 15 | 85523743 | 85682372 | PDE8A | | PDE8A | | | |
| 15 | 85747906 | 85748518 | LOC642423 | | | | | |
| 15 | 85783640 | 85790418 | GOLGA6L3 | | | | | |
| 15 | 85923826 | 85923893 | MIR7706 | | | | | |
| 15 | 85923846 | 86292589 | AKAP13 | | | | | |
| 15 | 86298171 | 86301956 | LOC101929679 | | | | | |
| 15 | 86302558 | 86338189 | KLHL25 | | | | | |
| 15 | 86313726 | 86313809 | MIR1276 | | | | | |
| 15 | 86626575 | 86659948 | LINC01584 | | | | | |
| 15 | 86685241 | 86860199 | AGBL1 | | | | | |
| 15 | 88120159 | 88122917 | LINC00052 | | | | | |
| 15 | 88419987 | 88814297 | NTRK3 | | NTRK3 | NTRK3 | | |
| 15 | 89002708 | 89010633 | MRPL46 | | | MRPL46 | | |
| 15 | 89010683 | 89021861 | MRPS11 | | | | | |
| 15 | 89055713 | 89089912 | DET1 | | | | | |
| 15 | 89128801 | 89148341 | LINC01586 | | | | | |
| 15 | 89151337 | 89151428 | MIR1179 | | | | | |
| 15 | 89155055 | 89155165 | MIR7 | | | | | |
| 15 | 89155077 | 89155155 | MIR3529 | | | | | |

| | | | | | | | | | |
|---|---|---|---|---|---|---|---|---|---|
| 15 | 89164526 | 89175512 | AEN | | | | | | |
| 15 | 89178867 | 89199575 | ISG20 | | | | | | |
| 15 | 89346673 | 89418585 | ACAN | | | | | | |
| 15 | 89420515 | 89438857 | HAPLN3 | | | | | | |
| 15 | 89441913 | 89456700 | MFGE8 | | | | | | |
| 15 | 89631380 | 89745591 | ABHD2 | | | | | | |
| 15 | 89753097 | 89764922 | RLBP1 | | | | | | |
| 15 | 89787193 | 89860362 | FANCI | | | | | | |
| 15 | 89859535 | 89878026 | POLG | | | | POLG | | |
| 15 | 89869969 | 89870041 | MIR6766 | | | | | | |
| 15 | 89904809 | 89941718 | MIR9 | | | | | | |
| 15 | 90014639 | 90039844 | RHCG | | | | | | |
| 15 | 90048160 | 90067265 | LINC00928 | | | | | | |
| 15 | 90118817 | 90171253 | TICRR | | | | | | |
| 15 | 90171200 | 90198682 | KIF7 | | | | | | |
| 15 | 90207599 | 90222648 | PLIN1 | | | | | | |
| 15 | 90224761 | 90234015 | PEX11A | | | | | | |
| 15 | 90234027 | 90286869 | WDR93 | | | | | | |
| 15 | 90293097 | 90294540 | MESP1 | | | | | | |
| 15 | 90319588 | 90321982 | MESP2 | | | | | | |
| 15 | 90328125 | 90358072 | ANPEP | | | | | | |
| 15 | 90373830 | 90437617 | AP3S2 | | | | AP3S2 | | |
| 15 | 90373830 | 90456222 | C15orf38-AP3S2 | | | | | | |
| 15 | 90393868 | 90393953 | MIR5094 | | | | | | |
| 15 | 90439763 | 90456222 | ARPIN | | | | | | |
| 15 | 90544722 | 90625432 | ZNF710 | | | | | | |
| 15 | 90549986 | 90550073 | MIR3174 | | | | | | |
| 15 | 90627210 | 90645786 | IDH2 | | | | | | |
| 15 | 90728151 | 90772892 | SEMA4B | | | | | | |
| 15 | 90773476 | 90808991 | CIB1 | | | | | | |
| 15 | 90777486 | 90785312 | GDPGP1 | | | | | | |
| 15 | 90792763 | 90802320 | TTLL13P | | | | | | |
| 15 | 90808894 | 90815443 | NGRN | | | | | | |
| 15 | 90889762 | 90892679 | GABARAPL3 | | | | | | |
| 15 | 90895476 | 90904715 | ZNF774 | | | | | | |
| 15 | 90931472 | 91045475 | IQGAP1 | | | | IQGAP1 | | |
| 15 | 91073117 | 91260371 | CRTC3 | | | | | | |
| 15 | 91203464 | 91208185 | LINC01585 | | | | | | |
| 15 | 91260557 | 91358692 | BLM | | | | | | |
| 15 | 91411821 | 91426688 | FURIN | | | | | | |
| 15 | 91427664 | 91439006 | FES | | | | | | |
| 15 | 91447419 | 91465815 | MAN2A2 | | | | | | |
| 15 | 91473409 | 91497323 | UNC45A | | | | | | |
| 15 | 91474147 | 91475799 | HDDC3 | | | | | | |
| 15 | 91478214 | 91497323 | UNC45A | | | | | | |
| 15 | 91498105 | 91506355 | RCCD1 | | | | | | |
| 15 | 91509267 | 91531854 | PRC1 | | | | | | |
| 15 | 91541645 | 91565851 | VPS33B | | | | VPS33B | | |
| 15 | 91565851 | 91574369 | LOC101926911 | | | | | | |
| 15 | 91643181 | 91844539 | SV2B | | | | | | |
| 15 | 92006568 | 92038080 | CRAT37 | | | | | | |
| 15 | 92396937 | 92715665 | SLCO3A1 | | | | | | |
| 15 | 92937139 | 93011958 | ST8SIA2 | | | | | | |
| 15 | 93013463 | 93014776 | LOC104613533 | | | | | | |
| 15 | 93014906 | 93044347 | C15orf32 | | | | | | |
| 15 | 93111047 | 93115493 | LINC00930 | | | | | | |
| 15 | 93160678 | 93199031 | FAM174B | | | | | | |
| 15 | 93338713 | 93340338 | ASB9P1 | | | | | | |
| 15 | 93426072 | 93441977 | LINC01578 | | | | | | |
| 15 | 93443550 | 93571237 | CHD2 | | | | CHD2 | | |
| 15 | 93447628 | 93447705 | MIR3175 | | | | | | |
| 15 | 93586635 | 93632443 | RGMA | | | | | | |
| 15 | 94399788 | 94421585 | LOC101927153 | | | | | | |
| 15 | 94443929 | 94527379 | LINC01580 | | | | | | |
| 15 | 94448633 | 94651167 | LINC01581 | | | | | | |
| 15 | 94841429 | 94946494 | MCTP2 | | | | | | |

| | | | | | | | | |
|---|---|---|---|---|---|---|---|---|
| 15 | 95398591 | 95400293 | LOC440311 | | | | | |
| 15 | 95822518 | 95870329 | LINC01197 | | | | | |
| 15 | 95976321 | 96051076 | LINC00924 | | | | | |
| 15 | 96670588 | 96883492 | NR2F2 | | | NR2F2 | | |
| 15 | 96876489 | 96876536 | MIR1469 | | | | | |
| 15 | 96876568 | 96883492 | NR2F2 | | | NR2F2 | | |
| 15 | 97315234 | 97328845 | SPATA8 | | | | | |
| 15 | 97913600 | 98065041 | LOC101927286 | | | | | |
| 15 | 98096513 | 98103928 | LOC101927310 | | | | | |
| 15 | 98285845 | 98417659 | LINC00923 | | | | | |
| 15 | 98503932 | 98517068 | ARRDC4 | | | | | |
| 15 | 98626207 | 98631982 | LINC01582 | | | | | |
| 15 | 98980390 | 99057611 | FAM169B | | | | | |
| 15 | 99189094 | 99194450 | IRAIN | | | | | |
| 15 | 99191767 | 99507759 | IGF1R | | | IGF1R | | |
| 15 | 99327654 | 99327731 | MIR4714 | | | | | |
| 15 | 99511458 | 99551024 | PGPEP1L | | | | | |
| 15 | 99557932 | 99574275 | LUNAR1 | | | | | |
| 15 | 99645285 | 99675800 | SYNM | | | | | |
| 15 | 99676527 | 99791431 | TTC23 | | | | | |
| 15 | 99791566 | 99926514 | LRRC28 | | | | | |
| 15 | 99797729 | 99800481 | HSP90B2P | | | | | |
| 15 | 100106132 | 100256693 | MEF2A | | | | | |
| 15 | 100267605 | 100273649 | LYSMD4 | | | | | |
| 15 | 100330360 | 100347132 | DNM1P46 | | | | | |
| 15 | 100511642 | 100882183 | ADAMTS17 | | | | | |
| 15 | 100884661 | 100890923 | SPATA41 | | | | | |
| 15 | 100913143 | 101084925 | CERS3 | | | | | |
| 15 | 101087956 | 101099488 | PRKXP1 | | | | | |
| 15 | 101109427 | 101142445 | LINS1 | | | | | |
| 15 | 101142754 | 101191904 | ASB7 | | | | | |
| 15 | 101419896 | 101456830 | ALDH1A3 | | | | | |
| 15 | 101459419 | 101610317 | LRRK1 | | | | | |
| 15 | 101715927 | 101792137 | CHSY1 | | | | | |
| 15 | 101811113 | 101817725 | VIMP | | | | | |
| 15 | 101821714 | 101835460 | SNRPA1 | | | | | |
| 15 | 101844132 | 102030187 | PCSK6 | | PCSK6 | | | |
| 15 | 101847455 | 101849509 | LOC100507472 | | | | | |
| 15 | 101874641 | 102030187 | PCSK6 | | PCSK6 | | | |
| 15 | 102173179 | 102192594 | TM2D3 | | | | | |
| 15 | 102193954 | 102264645 | TARSL2 | | | | | |
| 15 | 102345922 | 102346861 | OR4F6 | | | | | |
| 15 | 102358389 | 102359328 | OR4F15 | | | | | |
| 15 | 102382321 | 102390527 | OR4F13P | | | | | |
| 15 | 102462344 | 102463262 | OR4F4 | | | | | |
| 15 | 102495087 | 102496558 | FAM138E | | | | | |
| 15 | 102500661 | 102500799 | MIR1302 | | | | | |
| 15 | 102501015 | 102516808 | WASH3P | | | | | |
| 15 | 102513726 | 102513794 | MIR6859 | | | | | |
| 15 | 102516760 | 102519296 | DDX11L9 | | | | | |
| 16 | 61554 | 64090 | DDX11L10 | | | | | |
| 16 | 67051 | 67119 | MIR6859 | | | | | |
| 16 | 72915 | 75112 | WASIR2 | | | | | |
| 16 | 96978 | 103632 | POLR3K | | | | | |
| 16 | 103828 | 107669 | SNRNP25 | | SNRNP25 | | | |
| 16 | 108057 | 122629 | RHBDF1 | | | | | |
| 16 | 127017 | 135850 | MPG | | | | | |
| 16 | 135803 | 188697 | NPRL3 | | | | | |
| 16 | 202853 | 204504 | HBZ | | | | | |
| 16 | 215972 | 216767 | HBM | | | | | |
| 16 | 222845 | 223709 | HBA2 | | | HBA2 | | |
| 16 | 226649 | 227521 | HBA1 | | | HBA1 | | |
| 16 | 230332 | 231178 | HBQ1 | | | | | |
| 16 | 238969 | 279449 | LUC7L | | | | | |
| 16 | 284544 | 319942 | FAM234A | | | | | |
| 16 | 318299 | 325943 | RGS11 | | | | | |

| | | | | | | | | |
|---|---|---|---|---|---|---|---|---|
| 16 | 330605 | 333003 | ARHGDIG | | | | | |
| 16 | 333117 | 337209 | PDIA2 | | | | | |
| 16 | 337439 | 402676 | AXIN1 | | | | | AXIN1 |
| 16 | 417383 | 420569 | MRPL28 | | | MRPL28 | | |
| 16 | 420775 | 431950 | TMEM8A | | | | | |
| 16 | 432240 | 442960 | LOC100134368 | | | | | |
| 16 | 446754 | 450754 | NME4 | | | NME4 | | |
| 16 | 451857 | 462487 | DECR2 | | | | | |
| 16 | 475667 | 572481 | RAB11FIP3 | | | | | |
| 16 | 576846 | 577407 | LINC00235 | | | | | |
| 16 | 577855 | 604636 | CAPN15 | | | | | |
| 16 | 585315 | 585368 | MIR5587 | | | | | |
| 16 | 593276 | 593366 | MIR3176 | | | | | |
| 16 | 610421 | 615529 | PRR35 | | | | | |
| 16 | 616994 | 619495 | NHLRC4 | | | | | |
| 16 | 619967 | 634136 | PIGQ | | | | | |
| 16 | 639356 | 679273 | RAB40C | | | RAB40C | | |
| 16 | 681011 | 684116 | WFIKKN1 | | | | | |
| 16 | 684426 | 686366 | C16orf13 | | | | | |
| 16 | 691848 | 698474 | FAM195A | | | | | |
| 16 | 699362 | 717829 | WDR90 | | | | | |
| 16 | 718082 | 724174 | RHOT2 | | | | | |
| 16 | 725665 | 728268 | RHBDL1 | | | | | |
| 16 | 730110 | 732768 | STUB1 | | | | | |
| 16 | 731666 | 734439 | JMJD8 | | | | | |
| 16 | 734621 | 740424 | WDR24 | | | | | |
| 16 | 742499 | 755825 | FBXL16 | | | | | |
| 16 | 765172 | 767480 | METRN | | | | | |
| 16 | 771141 | 772590 | FAM173A | | | | | |
| 16 | 772581 | 776473 | CCDC78 | | | | | |
| 16 | 776935 | 779715 | HAGHL | | | | | |
| 16 | 779754 | 791038 | NARFL | | | | | |
| 16 | 810764 | 818865 | MSLN | | | | | |
| 16 | 820182 | 820277 | MIR662 | | | | | |
| 16 | 834973 | 838383 | RPUSD1 | | | | | |
| 16 | 838621 | 848074 | CHTF18 | | | | | |
| 16 | 848040 | 850733 | GNG13 | | | | | |
| 16 | 855442 | 863861 | PRR25 | | | | | |
| 16 | 903634 | 1031318 | LMF1 | | | | | |
| 16 | 1031807 | 1036979 | SOX8 | | SOX8 | SOX8 | | |
| 16 | 1114081 | 1131454 | SSTR5 | | | | | |
| 16 | 1138225 | 1146244 | C1QTNF8 | | | | | |
| 16 | 1203240 | 1271772 | CACNA1H | | | CACNA1H | CACNA1H | |
| 16 | 1271650 | 1275254 | TPSG1 | | | | | |
| 16 | 1278335 | 1280185 | TPSB2 | | | TPSB2 | | |
| 16 | 1290677 | 1292555 | TPSAB1 | | | TPSAB1 | | |
| 16 | 1306272 | 1308494 | TPSD1 | | | | | |
| 16 | 1359153 | 1377019 | UBE2I | | | | | |
| 16 | 1383605 | 1399442 | BAIAP3 | | BAIAP3 | | | |
| 16 | 1399240 | 1401873 | TSR3 | | | | | |
| 16 | 1401899 | 1413352 | GNPTG | | | GNPTG | | |
| 16 | 1413205 | 1464721 | UNKL | | | | | |
| 16 | 1469744 | 1470801 | C16orf91 | | | | | |
| 16 | 1484388 | 1494490 | CCDC154 | | | | | |
| 16 | 1494933 | 1525085 | CLCN7 | | | CLCN7 | | |
| 16 | 1535939 | 1538468 | PTX4 | | | | | |
| 16 | 1543351 | 1560460 | TELO2 | | | | | |
| 16 | 1560427 | 1662109 | IFT140 | | | | | |
| 16 | 1578741 | 1605581 | TMEM204 | | | | | |
| 16 | 1664640 | 1727909 | CRAMP1 | | | | | |
| 16 | 1728277 | 1752073 | HN1L | | | | | |
| 16 | 1756183 | 1820318 | MAPK8IP3 | | | MAPK8IP3 | | |
| 16 | 1784985 | 1785067 | MIR3177 | | | | | |
| 16 | 1820320 | 1821710 | NME3 | | | | | |
| 16 | 1821895 | 1823140 | MRPS34 | | | | | |
| 16 | 1823228 | 1826239 | EME2 | | | | | |

| | | | | | | | | |
|---|---|---|---|---|---|---|---|---|
| 16 | 1826712 | 1832581 | SPSB3 | | | | | |
| 16 | 1832923 | 1839192 | NUBP2 | | | | | |
| 16 | 1840413 | 1844909 | IGFALS | | | | | |
| 16 | 1859103 | 1877195 | HAGH | | | | | |
| 16 | 1877224 | 1890203 | FAHD1 | | | | | |
| 16 | 1883983 | 1922102 | MEIOB | | | | | |
| 16 | 1928285 | 1934232 | LINC00254 | | | | | |
| 16 | 1961464 | 1968324 | HS3ST6 | | | | | |
| 16 | 1988233 | 1993294 | MSRB1 | | | | | |
| 16 | 1994579 | 2004679 | RPL3L | | | | | |
| 16 | 2009516 | 2011976 | NDUFB10 | | | | | |
| 16 | 2012061 | 2014827 | RPS2 | | | | | |
| 16 | 2012334 | 2012467 | SNORA10 | | | | | |
| 16 | 2012973 | 2013107 | SNORA64 | | | | | |
| 16 | 2014996 | 2015505 | SNHG9 | | | | | |
| 16 | 2015184 | 2015311 | SNORA78 | | | | | |
| 16 | 2016874 | 2018976 | RNF151 | | | | | |
| 16 | 2022063 | 2028751 | TBL3 | | | | | |
| 16 | 2028917 | 2031550 | NOXO1 | | | | | |
| 16 | 2034149 | 2037750 | GFER | | | | | |
| 16 | 2039945 | 2044276 | SYNGR3 | | | | | |
| 16 | 2047652 | 2059822 | ZNF598 | | | | | |
| 16 | 2069520 | 2070756 | NPW | | | | | |
| 16 | 2076868 | 2089027 | SLC9A3R2 | | | SLC9A3R2 | | |
| 16 | 2089815 | 2097870 | NTHL1 | | | | | |
| 16 | 2097895 | 2138721 | TSC2 | | | TSC2 | | |
| 16 | 2138710 | 2185899 | PKD1 | | | PKD1 | | |
| 16 | 2140195 | 2140285 | MIR1225 | | | | | |
| 16 | 2156669 | 2156754 | MIR6511B1 | | | | | |
| 16 | 2156678 | 2156749 | MIR6511B2 | | | | | |
| 16 | 2183119 | 2183205 | MIR4516 | | | | | |
| 16 | 2185977 | 2186130 | MIR3180 | | | | | |
| 16 | 2198144 | 2204141 | RAB26 | | | | | |
| 16 | 2204782 | 2205359 | SNHG19 | | | | | |
| 16 | 2205023 | 2205106 | SNORD60 | | | | | |
| 16 | 2205798 | 2228130 | TRAF7 | | | | | |
| 16 | 2227183 | 2246465 | CASKIN1 | | | CASKIN1 | | |
| 16 | 2255177 | 2259418 | MLST8 | | | | | |
| 16 | 2259253 | 2261069 | BRICD5 | | | | | |
| 16 | 2261602 | 2264822 | PGP | | | | | |
| 16 | 2273488 | 2285743 | E4F1 | | | | | |
| 16 | 2286467 | 2288712 | DNASE1L2 | | | | | |
| 16 | 2289872 | 2301602 | ECI1 | | | | | |
| 16 | 2303116 | 2318413 | RNPS1 | | | | | |
| 16 | 2318663 | 2323073 | LOC106660606 | | | | | |
| 16 | 2320713 | 2320773 | MIR3677 | | | | | |
| 16 | 2321747 | 2321841 | MIR940 | | | | | |
| 16 | 2324620 | 2324692 | MIR4717 | | | | | |
| 16 | 2325878 | 2390747 | ABCA3 | | ABCA3 | | | |
| 16 | 2390922 | 2476700 | ABCA17P | | | | | |
| 16 | 2479394 | 2508859 | CCNF | | | | | |
| 16 | 2495392 | 2495458 | MIR6767 | | | | | |
| 16 | 2510114 | 2514964 | C16orf59 | | | | | |
| 16 | 2513967 | 2514039 | MIR6768 | | | | | |
| 16 | 2521499 | 2524146 | NTN3 | | | | | |
| 16 | 2525146 | 2555734 | TBC1D24 | | TBC1D24 | | | |
| 16 | 2563726 | 2570224 | ATP6V0C | | | | | |
| 16 | 2570362 | 2580955 | AMDHD2 | | | | | |
| 16 | 2580035 | 2581409 | CEMP1 | | | | | |
| 16 | 2581922 | 2582006 | MIR3178 | | | | | |
| 16 | 2587964 | 2653191 | PDPK1 | | | | | |
| 16 | 2653384 | 2680495 | LOC652276 | | | | | |
| 16 | 2688982 | 2696130 | FLJ42627 | | | | | |
| 16 | 2708389 | 2723440 | ERVK13-1 | | | | | |
| 16 | 2732494 | 2759031 | KCTD5 | | | | | |
| 16 | 2762418 | 2770552 | PRSS27 | | | | | |

| | | | | | | | | | |
|---|---|---|---|---|---|---|---|---|---|
| 16 | 2787076 | 2821413 | SRRM2 | | | | | | |
| 16 | 2821414 | 2827297 | TCEB2 | | | | | | |
| 16 | 2833953 | 2836708 | PRSS33 | | | | | | |
| 16 | 2848485 | 2855133 | PRSS41 | | | | | | |
| 16 | 2867163 | 2871723 | PRSS21 | | | | | | |
| 16 | 2880172 | 2882285 | ZG16B | | | | | | |
| 16 | 2889573 | 2892752 | PRSS30P | | | | | | |
| 16 | 2902727 | 2908171 | PRSS22 | | | | | | |
| 16 | 2933195 | 2949383 | FLYWCH2 | | | | | | |
| 16 | 2961979 | 3001209 | FLYWCH1 | | | | | | |
| 16 | 3014216 | 3018384 | KREMEN2 | | | | | | |
| 16 | 3019245 | 3023490 | PAQR4 | | | | | | |
| 16 | 3022791 | 3030540 | PKMYT1 | | | PKMYT1 | | | |
| 16 | 3039054 | 3044510 | LINC00514 | | | | | | |
| 16 | 3048955 | 3050814 | LOC101929613 | | | | | | |
| 16 | 3062456 | 3064506 | CLDN9 | | | | | | |
| 16 | 3064712 | 3068188 | CLDN6 | | | | | | |
| 16 | 3070312 | 3072383 | TNFRSF12A | | | | | | |
| 16 | 3072620 | 3074287 | HCFC1R1 | | | | | | |
| 16 | 3074031 | 3077756 | THOC6 | | | | | | |
| 16 | 3077867 | 3085542 | CCDC64B | | | | | | |
| 16 | 3082481 | 3089133 | LOC100128770 | | | | | | |
| 16 | 3096681 | 3109371 | MMP25 | | | MMP25 | | | |
| 16 | 3115312 | 3119668 | IL32 | | | | | | |
| 16 | 3138890 | 3149318 | ZSCAN10 | | | | | | |
| 16 | 3160460 | 3170518 | ZNF205 | | | | | | |
| 16 | 3175529 | 3192805 | ZNF213 | | | | | | |
| 16 | 3194291 | 3199947 | CASP16P | | | | | | |
| 16 | 3254246 | 3255185 | OR1F1 | | | | | | |
| 16 | 3265561 | 3266546 | OR1F2P | | | | | | |
| 16 | 3272324 | 3285457 | ZNF200 | | | | | | |
| 16 | 3292027 | 3306627 | MEFV | | | MEFV | | | |
| 16 | 3313767 | 3317566 | LINC00921 | | | | | | |
| 16 | 3333486 | 3341459 | ZNF263 | | | | | | |
| 16 | 3348807 | 3355439 | TIGD7 | | | | | | |
| 16 | 3355405 | 3368576 | ZNF75A | | | | | | |
| 16 | 3405888 | 3406924 | OR2C1 | | | | | | |
| 16 | 3421052 | 3422283 | MTRNR2L4 | | | | | | |
| 16 | 3432080 | 3451065 | ZSCAN32 | | | | | | |
| 16 | 3451189 | 3459364 | ZNF174 | | | | | | |
| 16 | 3482421 | 3493537 | ZNF597 | | | | | | |
| 16 | 3493610 | 3536963 | NAA60 | | | | | | |
| 16 | 3535380 | 3535469 | MIR6126 | | | | | | |
| 16 | 3543483 | 3545421 | C16orf90 | | | | | | |
| 16 | 3550944 | 3589051 | CLUAP1 | | | | | | |
| 16 | 3589035 | 3627405 | NLRC3 | | | | | | |
| 16 | 3631183 | 3661585 | SLX4 | | | | | | |
| 16 | 3702939 | 3708096 | DNASE1 | | | DNASE1 | | | |
| 16 | 3708037 | 3767598 | TRAP1 | | | | | | |
| 16 | 3775055 | 3930121 | CREBBP | | | CREBBP | | | |
| 16 | 3997625 | 4000445 | LOC102724927 | | | | | | |
| 16 | 4012649 | 4166186 | ADCY9 | | | | | | |
| 16 | 4239374 | 4292081 | SRL | | | | | | |
| 16 | 4295825 | 4303790 | LINC01569 | | | | | | |
| 16 | 4307186 | 4323001 | TFAP4 | | | TFAP4 | | | |
| 16 | 4364761 | 4389598 | GLIS2 | | | | | | |
| 16 | 4390251 | 4401373 | PAM16 | | | | | | |
| 16 | 4390251 | 4466962 | CORO7-PAM16 | | | | | | |
| 16 | 4404542 | 4466962 | CORO7 | | | | | | |
| 16 | 4421848 | 4433529 | VASN | | | | | | |
| 16 | 4475805 | 4506775 | DNAJA3 | | | DNAJA3 | | | |
| 16 | 4511677 | 4524896 | NMRAL1 | | | | | | |
| 16 | 4524703 | 4560348 | HMOX2 | | | | | | |
| 16 | 4560676 | 4588816 | CDIP1 | | | | | | |
| 16 | 4606490 | 4650318 | C16orf96 | | | | | | |
| 16 | 4658883 | 4664927 | UBALD1 | | | | | | |

| | | | | | | | | |
|---|---|---|---|---|---|---|---|---|
| 16 | 4674824 | 4740975 | MGRN1 | | | | | |
| 16 | 4721318 | 4721391 | MIR6769A | | | | | |
| 16 | 4743693 | 4745860 | NUDT16L1 | | | | | |
| 16 | 4746510 | 4784378 | ANKS3 | | | | | |
| 16 | 4784288 | 4799397 | C16orf71 | | | | | |
| 16 | 4800814 | 4817219 | ZNF500 | | | | | |
| 16 | 4827614 | 4838522 | SEPT12 | | SEPT12 | | | |
| 16 | 4838397 | 4846492 | SMIM22 | | | | | |
| 16 | 4846962 | 4852951 | ROGDI | | ROGDI | | | |
| 16 | 4853203 | 4897303 | GLYR1 | | | | | |
| 16 | 4897631 | 4932402 | UBN1 | | | | | |
| 16 | 4932507 | 4987136 | PPL | | | PPL | | |
| 16 | 5008317 | 5069156 | SEC14L5 | | | | | |
| 16 | 5074844 | 5092972 | NAGPA | | | | | |
| 16 | 5094122 | 5116146 | C16orf89 | | | | | |
| 16 | 5121809 | 5137380 | ALG1 | | | | | |
| 16 | 5134300 | 5147821 | EEF2KMT | | | | | |
| 16 | 5651169 | 5666251 | LINC01570 | | | | | |
| 16 | 5682467 | 5682567 | MIR8065 | | | | | |
| 16 | 6069131 | 7763340 | RBFOX1 | | RBFOX1 | | | |
| 16 | 8576550 | 8622226 | TMEM114 | | | | | |
| 16 | 8715496 | 8740079 | METTL22 | | | | | |
| 16 | 8768443 | 8878432 | ABAT | | ABAT | ABAT | | |
| 16 | 8889036 | 8891505 | TMEM186 | | | | | |
| 16 | 8891669 | 8943194 | PMM2 | | | PMM2 | | |
| 16 | 8946798 | 8962869 | CARHSP1 | | | | | |
| 16 | 8985950 | 9057341 | USP7 | | | | | |
| 16 | 9185536 | 9213555 | C16orf72 | | | | | |
| 16 | 9328773 | 9328803 | MIR548X | | | | | |
| 16 | 9535150 | 9538842 | LINC01177 | | | | | |
| 16 | 9539869 | 9549362 | LINC01195 | | | | | |
| 16 | 9847261 | 10276611 | GRIN2A | GRIN2A | GRIN2A | GRIN2A | GRIN2A | GRIN2A |
| 16 | 10479911 | 10577495 | ATF7IP2 | | | | | |
| 16 | 10622278 | 10674573 | EMP2 | | | | EMP2 | |
| 16 | 10721360 | 10788802 | TEKT5 | | | | | |
| 16 | 10837642 | 10863208 | NUBP1 | | | | | |
| 16 | 10854775 | 10912651 | TVP23A | | | | | |
| 16 | 10971054 | 11018840 | CIITA | | | | | |
| 16 | 11022747 | 11036257 | DEXI | | | | | |
| 16 | 11038344 | 11276046 | CLEC16A | | | | | |
| 16 | 11348273 | 11350039 | SOCS1 | | | SOCS1 | | |
| 16 | 11361713 | 11363160 | TNP2 | | | | | |
| 16 | 11367055 | 11367452 | PRM3 | | | | | |
| 16 | 11369492 | 11370337 | PRM2 | | | | | |
| 16 | 11374692 | 11375192 | PRM1 | | | | | |
| 16 | 11439294 | 11445620 | RMI2 | | | | | |
| 16 | 11559115 | 11567030 | LOC101927131 | | | | | |
| 16 | 11641577 | 11681322 | LITAF | | | | | |
| 16 | 11762288 | 11773015 | SNN | | | | | |
| 16 | 11772935 | 11836734 | TXNDC11 | | | | | |
| 16 | 11844441 | 11891114 | ZC3H7A | | | | | |
| 16 | 11913690 | 11922689 | BCAR4 | | | | | |
| 16 | 11928054 | 11945442 | RSL1D1 | | | | | |
| 16 | 11961984 | 12010519 | GSPT1 | | | | | |
| 16 | 12058963 | 12061925 | TNFRSF17 | | | | | |
| 16 | 12070590 | 12668146 | SNX29 | | | | | |
| 16 | 12753655 | 12897744 | CPPED1 | | | | | |
| 16 | 12814177 | 12814228 | MIR4718 | | | | | |
| 16 | 12995476 | 13334273 | SHISA9 | | | | | |
| 16 | 14014013 | 14046205 | ERCC4 | | | ERCC4 | | |
| 16 | 14103132 | 14109875 | LOC101927311 | | | | | |
| 16 | 14112736 | 14114934 | LOC101927348 | | | | | |
| 16 | 14165169 | 14360636 | MKL2 | | | | | |
| 16 | 14396144 | 14420374 | MIR193BHG | | | | | |
| 16 | 14397823 | 14397906 | MIR193B | | | | | |
| 16 | 14403141 | 14403228 | MIR365A | | | | | |

| | | | | | | | | |
|---|---|---|---|---|---|---|---|---|
| 16 | 14456965 | 14464123 | LOC105447648 | | | | | |
| 16 | 14529556 | 14724128 | PARN | | | | | |
| 16 | 14726667 | 14763093 | BFAR | | | | | |
| 16 | 14766404 | 14788430 | PLA2G10 | | | | | |
| 16 | 14805545 | 14820185 | NPIPA2 | | | | | |
| 16 | 14805545 | 14820195 | NPIPA3 | | | | | |
| 16 | 14844669 | 14859305 | NPIPA2 | | | | | |
| 16 | 14844669 | 14859315 | NPIPA3 | | | | | |
| 16 | 14916288 | 14918559 | ABCC6P2 | | | | | |
| 16 | 14927642 | 14990014 | NOMO1 | | | | | |
| 16 | 14995364 | 14995448 | MIR3179 | | | | | |
| 16 | 15001573 | 15001638 | MIR3670 | | | | | |
| 16 | 15005076 | 15005168 | MIR3180 | | | | | |
| 16 | 15019606 | 15021140 | LOC100288162 | | | | | |
| 16 | 15019793 | 15019860 | MIR6511A1 | | | | | |
| 16 | 15019793 | 15019860 | MIR6511A2 | | | | | |
| 16 | 15019793 | 15019860 | MIR6511A3 | | | | | |
| 16 | 15019793 | 15019860 | MIR6511A4 | | | | | |
| 16 | 15024676 | 15024736 | MIR6770 | | | | | |
| 16 | 15031299 | 15045931 | NPIPA1 | | | | | |
| 16 | 15068591 | 15233206 | PDXDC1 | | | | | |
| 16 | 15131709 | 15149936 | NTAN1 | | | NTAN1 | | |
| 16 | 15153878 | 15188192 | RRN3 | | | | | |
| 16 | 15188267 | 15203054 | LOC100505915 | | | | | |
| 16 | 15198158 | 15225458 | PKD1P6-NPIPP1 | | | | | |
| 16 | 15227922 | 15228007 | MIR6511B1 | | | | | |
| 16 | 15227931 | 15228002 | MIR6511B2 | | | | | |
| 16 | 15248706 | 15248859 | MIR3180 | | | | | |
| 16 | 15457480 | 15472151 | NPIPA5 | | | | | |
| 16 | 15489610 | 15503543 | MPV17L | | | | | |
| 16 | 15528324 | 15682116 | C16orf45 | | | | | |
| 16 | 15688225 | 15737023 | KIAA0430 | | | | | |
| 16 | 15704886 | 15704952 | MIR6506 | | | | | |
| 16 | 15737123 | 15820208 | NDE1 | | | NDE1 | | |
| 16 | 15737150 | 15737229 | MIR484 | | | | | |
| 16 | 15744082 | 15820208 | NDE1 | | | NDE1 | | |
| 16 | 15796991 | 15950887 | MYH11 | | | | | |
| 16 | 15959575 | 15982506 | FOPNL | | | | | |
| 16 | 16043433 | 16236930 | ABCC1 | | | | | |
| 16 | 16243421 | 16317328 | ABCC6 | | | | | |
| 16 | 16326388 | 16388668 | NOMO3 | | | | | |
| 16 | 16394015 | 16394099 | MIR3179 | | | | | |
| 16 | 16400226 | 16400291 | MIR3670 | | | | | |
| 16 | 16403731 | 16403823 | MIR3180 | | | | | |
| 16 | 16411465 | 16444465 | PKD1P1 | | | | | |
| 16 | 16418257 | 16419791 | LOC100288162 | | | | | |
| 16 | 16418444 | 16418511 | MIR6511A1 | | | | | |
| 16 | 16418444 | 16418511 | MIR6511A2 | | | | | |
| 16 | 16418444 | 16418511 | MIR6511A3 | | | | | |
| 16 | 16418444 | 16418511 | MIR6511A4 | | | | | |
| 16 | 16423161 | 16423221 | MIR6770 | | | | | |
| 16 | 16462732 | 16462799 | MIR6511A1 | | | | | |
| 16 | 16462732 | 16462799 | MIR6511A2 | | | | | |
| 16 | 16462732 | 16462799 | MIR6511A3 | | | | | |
| 16 | 16462732 | 16462799 | MIR6511A4 | | | | | |
| 16 | 16473101 | 16487829 | NPIPA7 | | | | | |
| 16 | 16473101 | 16487829 | NPIPA8 | | | | | |
| 16 | 17196180 | 17564738 | XYLT1 | | | | | |
| 16 | 18411775 | 18426519 | NPIPA7 | | | | | |
| 16 | 18411775 | 18426519 | NPIPA8 | | | | | |
| 16 | 18436589 | 18438123 | LOC100288162 | | | | | |
| 16 | 18437869 | 18437936 | MIR6511A1 | | | | | |
| 16 | 18437869 | 18437936 | MIR6511A2 | | | | | |
| 16 | 18437869 | 18437936 | MIR6511A3 | | | | | |
| 16 | 18437869 | 18437936 | MIR6511A4 | | | | | |
| 16 | 18473207 | 18473267 | MIR6770 | | | | | |

| | | | | | | | | |
|---|---|---|---|---|---|---|---|---|
| 16 | 18496034 | 18496124 | MIR3180 | | | | | |
| 16 | 18499554 | 18499619 | MIR3670 | | | | | |
| 16 | 18505750 | 18505834 | MIR3179 | | | | | |
| 16 | 18511181 | 18573434 | NOMO2 | | | | | |
| 16 | 18582569 | 18609607 | ABCC6P1 | | | | | |
| 16 | 18794276 | 18801656 | RPS15A | | | | | |
| 16 | 18802988 | 18813000 | ARL6IP1 | | ARL6IP1 | | | |
| 16 | 18816174 | 18937726 | SMG1 | | | | | |
| 16 | 18995255 | 19075262 | TMC7 | | | | | |
| 16 | 19074075 | 19079013 | LOC102723385 | | | | | |
| 16 | 19078916 | 19091417 | COQ7 | | | | | |
| 16 | 19125253 | 19132952 | ITPRIPL2 | | | | | |
| 16 | 19179534 | 19279656 | SYT17 | | | | | |
| 16 | 19297104 | 19322269 | CLEC19A | | | | | |
| 16 | 19421860 | 19510434 | TMC5 | | | | | |
| 16 | 19513014 | 19533450 | GDE1 | | | | | |
| 16 | 19535178 | 19564728 | CCP110 | | | | | |
| 16 | 19566736 | 19712485 | C16orf62 | | | | | |
| 16 | 19717673 | 19729492 | KNOP1 | | | | | |
| 16 | 19727777 | 19869789 | IQCK | | | | | |
| 16 | 19870292 | 19896956 | GPRC5B | | | | | |
| 16 | 20042806 | 20085100 | GPR139 | | | | | |
| 16 | 20320895 | 20338942 | GP2 | | | | | |
| 16 | 20344372 | 20364200 | UMOD | | | | | |
| 16 | 20370491 | 20416033 | PDILT | | | | | |
| 16 | 20420855 | 20452281 | ACSM5 | | | | | |
| 16 | 20462782 | 20498991 | ACSM2A | | | | | |
| 16 | 20548082 | 20587695 | ACSM2B | | | | | |
| 16 | 20634558 | 20709110 | ACSM1 | | | | | |
| 16 | 20744985 | 20753286 | THUMPD1 | | | | | |
| 16 | 20775311 | 20808479 | ACSM3 | | | | | |
| 16 | 20791514 | 20817795 | ERI2 | | | | | |
| 16 | 20817766 | 20860990 | LOC81691 | | | | | |
| 16 | 20869395 | 20911561 | DCUN1D3 | | | | | |
| 16 | 20911189 | 20936331 | LYRM1 | | | | | |
| 16 | 20944432 | 21170762 | DNAH3 | | | | | |
| 16 | 21169697 | 21191937 | TMEM159 | | | | | |
| 16 | 21208772 | 21225831 | ZP2 | | | | | |
| 16 | 21245015 | 21263750 | ANKS4B | | | | | |
| 16 | 21269838 | 21329912 | CRYM | | | | | |
| 16 | 21360683 | 21397254 | SNX29P1 | | | | | |
| 16 | 21413454 | 21436658 | NPIPB3 | | | | | |
| 16 | 21443344 | 21445776 | LOC100190986 | | | | | |
| 16 | 21458003 | 21513602 | SMG1P3 | | | | | |
| 16 | 21517369 | 21517456 | MIR3680 | | | | | |
| 16 | 21529229 | 21531765 | SLC7A5P2 | | | | | |
| 16 | 21601018 | 21610706 | LOC101927814 | | | | | |
| 16 | 21608541 | 21668794 | METTL9 | | | | | |
| 16 | 21652604 | 21663987 | IGSF6 | | | | | |
| 16 | 21689834 | 21772050 | OTOA | | | | | |
| 16 | 21807950 | 21830495 | RRN3P1 | | | | | |
| 16 | 21845883 | 21869077 | NPIPB4 | | | | | |
| 16 | 21964384 | 21994981 | UQCRC2 | | | | | |
| 16 | 21995185 | 22012431 | PDZD9 | | | | | |
| 16 | 22019455 | 22095972 | C16orf52 | | | | | |
| 16 | 22103858 | 22168287 | VWA3A | | | | | |
| 16 | 22217591 | 22300066 | EEF2K | | | | | |
| 16 | 22308695 | 22346424 | POLR3E | | | | | |
| 16 | 22357256 | 22385938 | CDR2 | | | | | |
| 16 | 22430866 | 22449036 | RRN3P3 | | | | | |
| 16 | 22448328 | 22503541 | SMG1P1 | | | | | |
| 16 | 22524843 | 22547841 | NPIPB5 | | | | | |
| 16 | 22557018 | 22588186 | LOC653786 | | | | | |
| 16 | 22729602 | 22803362 | MIR548D2 | | | | | |
| 16 | 22729602 | 22803363 | MIR548AA2 | | | | | |
| 16 | 22825859 | 22927659 | HS3ST2 | | | | | |

| | | | | | | | | | |
|---|---|---|---|---|---|---|---|---|---|
| 16 | 23072727 | 23160591 | USP31 | | | | | | |
| 16 | 23194039 | 23228200 | SCNN1G | | | SCNN1G | | | |
| 16 | 23313590 | 23392620 | SCNN1B | | | | | | |
| 16 | 23399813 | 23464512 | COG7 | | | | | | |
| 16 | 23474862 | 23521815 | GGA2 | | | GGA2 | | | |
| 16 | 23533333 | 23568696 | EARS2 | | | | | | |
| 16 | 23568861 | 23585710 | UBFD1 | | | | | | |
| 16 | 23592334 | 23607639 | NDUFAB1 | | | | | | |
| 16 | 23614482 | 23652678 | PALB2 | | | | | | |
| 16 | 23652686 | 23685068 | DCTN5 | | | | | | |
| 16 | 23690092 | 23701688 | PLK1 | | | | | | |
| 16 | 23701625 | 23724821 | ERN2 | | | | | | |
| 16 | 23765947 | 23770272 | CHP2 | | | | | | |
| 16 | 23847299 | 24231932 | PRKCB | | PRKCB | PRKCB | | | |
| 16 | 24266873 | 24373737 | CACNG3 | | | | | | |
| 16 | 24550907 | 24584183 | RBBP6 | | | | | | |
| 16 | 24672742 | 24682383 | LINC01567 | | | | | | |
| 16 | 24741048 | 24837547 | TNRC6A | | | | | | |
| 16 | 24857183 | 24922949 | SLC5A11 | | | | | | |
| 16 | 24930704 | 25026699 | ARHGAP17 | | | | | | |
| 16 | 25043061 | 25044098 | LOC554206 | | | | | | |
| 16 | 25111884 | 25160353 | LCMT1 | | | | | | |
| 16 | 25228284 | 25240253 | AQP8 | | | | | | |
| 16 | 25247321 | 25268855 | ZKSCAN2 | | | | | | |
| 16 | 25703346 | 26149009 | HS3ST4 | | | | | | |
| 16 | 27078218 | 27080487 | C16orf82 | | | | | | |
| 16 | 27214806 | 27233089 | KDM8 | | | | | | |
| 16 | 27236314 | 27280113 | NSMCE1 | | | | | | |
| 16 | 27279525 | 27301789 | FLJ21408 | | | | | | |
| 16 | 27325229 | 27376099 | IL4R | | | | | | |
| 16 | 27413482 | 27464714 | IL21R | | | | | | |
| 16 | 27471933 | 27561251 | GTF3C1 | | | | | | |
| 16 | 27561467 | 27791692 | KIAA0556 | | | | | | |
| 16 | 27798849 | 28074830 | GSG1L | | | | | | |
| 16 | 28109297 | 28223239 | XPO6 | | | | | | |
| 16 | 28303839 | 28335170 | SBK1 | | | | | | |
| 16 | 28353838 | 28374181 | NPIPB6 | | | | | | |
| 16 | 28390902 | 28415206 | EIF3CL | | | | | | |
| 16 | 28390902 | 28437775 | EIF3C | | | | | | |
| 16 | 28402302 | 28402372 | MIR6862 | | | | | | |
| 16 | 28477973 | 28503623 | CLN3 | CLN3 | | | CLN3 | | |
| 16 | 28505969 | 28510291 | APOBR | | | | | | |
| 16 | 28510682 | 28518155 | IL27 | | | | | | |
| 16 | 28548661 | 28550495 | NUPR1 | | | | | | |
| 16 | 28565248 | 28603111 | SGF29 | | | | | | |
| 16 | 28603263 | 28608391 | SULT1A2 | | | SULT1A2 | | | |
| 16 | 28616907 | 28634907 | SULT1A1 | | | | | | |
| 16 | 28649681 | 28669951 | NPIPB8 | | | | | | |
| 16 | 28699878 | 28747050 | EIF3C | | | | | | |
| 16 | 28722740 | 28747050 | EIF3CL | | | | | | |
| 16 | 28735572 | 28735642 | MIR6862 | | | | | | |
| 16 | 28763755 | 28784144 | NPIPB9 | | | | | | |
| 16 | 28834368 | 28848558 | ATXN2L | | | | | | |
| 16 | 28853731 | 28857729 | TUFM | | | | | | |
| 16 | 28855239 | 28855328 | MIR4721 | | | | | | |
| 16 | 28857920 | 28885534 | SH2B1 | | | | | | |
| 16 | 28889808 | 28915830 | ATP2A1 | | | ATP2A1 | | | |
| 16 | 28915741 | 28936532 | RABEP2 | | | RABEP2 | | | |
| 16 | 28943259 | 28950668 | CD19 | | | | | | |
| 16 | 28962317 | 28977767 | NFATC2IP | | | | | | |
| 16 | 28969903 | 28969982 | MIR4517 | | | | | | |
| 16 | 28986095 | 28995869 | SPNS1 | | | | | | |
| 16 | 28996146 | 29002104 | LAT | | | | | | |
| 16 | 29086162 | 29128038 | RRN3P2 | | | | | | |
| 16 | 29313607 | 29376380 | SNX29P2 | | | | | | |
| 16 | 29392638 | 29415123 | NPIPB11 | | | | | | |

| | | | | | | | | |
|---|---|---|---|---|---|---|---|---|
| 16 | 29460665 | 29461236 | LOC606724 | | | | | |
| 16 | 29464913 | 29466285 | BOLA2 | | | | | |
| 16 | 29464913 | 29466285 | BOLA2B | | | | | |
| 16 | 29465821 | 29469545 | SLX1A | | | | | |
| 16 | 29465821 | 29469545 | SLX1B | | | | | |
| 16 | 29466411 | 29476301 | SLX1A-SULT1A3 | | | | | |
| 16 | 29466411 | 29476301 | SLX1B-SULT1A4 | | | | | |
| 16 | 29471206 | 29476301 | SULT1A3 | | | SULT1A3 | | |
| 16 | 29471206 | 29476301 | SULT1A4 | | | | | |
| 16 | 29476288 | 29478899 | LOC388242 | | | | | |
| 16 | 29476288 | 29478899 | LOC613038 | | | | | |
| 16 | 29538928 | 29577540 | SMG1P2 | | | | | |
| 16 | 29610499 | 29610586 | MIR3680 | | | | | |
| 16 | 29624423 | 29625038 | SLC7A5P1 | | | | | |
| 16 | 29674270 | 29681823 | SPN | | | | | |
| 16 | 29690328 | 29710021 | QPRT | | | QPRT | | |
| 16 | 29753785 | 29757340 | C16orf54 | | | | | |
| 16 | 29789560 | 29792969 | ZG16 | | | | | |
| 16 | 29802033 | 29816706 | KIF22 | | | KIF22 | | |
| 16 | 29817416 | 29822504 | MAZ | | | MAZ | | |
| 16 | 29823408 | 29827202 | PRRT2 | | | | | |
| 16 | 29827527 | 29833816 | PAGR1 | | | | | |
| 16 | 29831714 | 29859360 | MVP | | | | | |
| 16 | 29869676 | 29879374 | CDIPT | | | CDIPT | | |
| 16 | 29882479 | 29910585 | SEZ6L2 | | SEZ6L2 | SEZ6L2 | | |
| 16 | 29912146 | 29917377 | ASPHD1 | | | ASPHD1 | | |
| 16 | 29917656 | 29937553 | KCTD13 | | | KCTD13 | | |
| 16 | 29973350 | 29984373 | TMEM219 | | | | | |
| 16 | 29985187 | 30003582 | TAOK2 | | | TAOK2 | | |
| 16 | 30003641 | 30007417 | HIRIP3 | | | HIRIP3 | | |
| 16 | 30007529 | 30017115 | INO80E | | | | | |
| 16 | 30016834 | 30024917 | DOC2A | | | DOC2A | | |
| 16 | 30034654 | 30036023 | C16orf92 | | | | | |
| 16 | 30035743 | 30042186 | FAM57B | | | FAM57B | | |
| 16 | 30064410 | 30081741 | ALDOA | | | ALDOA | | |
| 16 | 30087296 | 30096698 | PPP4C | | | | | |
| 16 | 30097114 | 30103205 | TBX6 | | | TBX6 | | |
| 16 | 30103634 | 30107537 | YPEL3 | | | | | |
| 16 | 30116130 | 30124878 | GDPD3 | | | | | |
| 16 | 30125425 | 30134630 | MAPK3 | | MAPK3 | MAPK3 | | |
| 16 | 30194730 | 30200397 | CORO1A | | | CORO1A | | |
| 16 | 30200004 | 30200575 | LOC606724 | | | | | |
| 16 | 30204255 | 30205627 | BOLA2 | | | | | |
| 16 | 30204255 | 30205627 | BOLA2B | | | | | |
| 16 | 30205163 | 30208887 | SLX1A | | | | | |
| 16 | 30205163 | 30208887 | SLX1B | | | | | |
| 16 | 30205753 | 30215650 | SLX1A-SULT1A3 | | | | | |
| 16 | 30205753 | 30215650 | SLX1B-SULT1A4 | | | | | |
| 16 | 30210548 | 30215650 | SULT1A3 | | | SULT1A3 | | |
| 16 | 30210548 | 30215650 | SULT1A4 | | | | | |
| 16 | 30215637 | 30218248 | LOC388242 | | | | | |
| 16 | 30215637 | 30218248 | LOC613038 | | | | | |
| 16 | 30234349 | 30256932 | LOC613037 | | | | | |
| 16 | 30278913 | 30317244 | SMG1P2 | | | | | |
| 16 | 30278913 | 30346695 | SMG1P5 | | | | | |
| 16 | 30362086 | 30366682 | CD2BP2 | | | | | |
| 16 | 30368421 | 30381522 | TBC1D10B | | | | | |
| 16 | 30386122 | 30389310 | MYLPF | | | | | |
| 16 | 30389453 | 30394171 | SEPT1 | | | | | |
| 16 | 30389632 | 30411429 | ZNF48 | | | | | |
| 16 | 30418734 | 30429916 | ZNF771 | | | | | |
| 16 | 30435018 | 30441373 | DCTPP1 | | | | | |
| 16 | 30454945 | 30457296 | SEPHS2 | | | | | |
| 16 | 30483982 | 30534506 | ITGAL | | | | | |
| 16 | 30515239 | 30515322 | MIR4518 | | | | | |
| 16 | 30535321 | 30537910 | ZNF768 | | | | | |

| | | | | | | | | |
|---|---|---|---|---|---|---|---|---|
| 16 | 30541687 | 30546291 | ZNF747 | | | | | |
| 16 | 30565084 | 30569642 | ZNF764 | | | | | |
| 16 | 30581018 | 30583728 | ZNF688 | | | | | |
| 16 | 30591993 | 30597092 | ZNF785 | | | | | |
| 16 | 30613878 | 30622096 | ZNF689 | | | | | |
| 16 | 30662240 | 30667734 | PRR14 | | | | | |
| 16 | 30670839 | 30682131 | FBRS | | | | | |
| 16 | 30709024 | 30709810 | LOC730183 | | | | | |
| 16 | 30710461 | 30751450 | SRCAP | | | | | |
| 16 | 30721857 | 30721986 | SNORA30 | | | | | |
| 16 | 30751511 | 30756517 | TMEM265 | | | | | |
| 16 | 30759619 | 30772497 | PHKG2 | | PHKG2 | | | |
| 16 | 30768743 | 30773565 | CCDC189 | | | | | |
| 16 | 30772932 | 30787628 | RNF40 | | | | | |
| 16 | 30789769 | 30798523 | ZNF629 | | | | | |
| 16 | 30845361 | 30905623 | BCL7C | | | | | |
| 16 | 30886586 | 30886644 | MIR4519 | | | | | |
| 16 | 30886780 | 30906539 | MIR762HG | | | | | |
| 16 | 30899115 | 30905623 | BCL7C | | | | | |
| 16 | 30905223 | 30905306 | MIR762 | | | | | |
| 16 | 30907927 | 30914881 | CTF1 | | | | | |
| 16 | 30930639 | 30960104 | FBXL19 | | | | | |
| 16 | 30960404 | 30966259 | ORAI3 | | | | | |
| 16 | 30968614 | 30995983 | SETD1A | | | | | |
| 16 | 30996518 | 31000473 | HSD3B7 | | | | | |
| 16 | 31000576 | 31021959 | STX1B | | STX1B | STX1B | STX1B | |
| 16 | 31044415 | 31051488 | STX4 | | | STX4 | | |
| 16 | 31072163 | 31084823 | ZNF668 | | | | | |
| 16 | 31085742 | 31094833 | ZNF646 | | | | | |
| 16 | 31094744 | 31100130 | PRSS53 | | | | | |
| 16 | 31102162 | 31106320 | VKORC1 | | | | | |
| 16 | 31119614 | 31124112 | BCKDK | | | | | |
| 16 | 31128984 | 31142714 | KAT8 | | | | | |
| 16 | 31142753 | 31147083 | PRSS8 | | | | | |
| 16 | 31150246 | 31161415 | PRSS36 | | | | | |
| 16 | 31191430 | 31206192 | FUS | | | | FUS | |
| 16 | 31212806 | 31214773 | PYCARD | | | | | |
| 16 | 31225341 | 31236510 | TRIM72 | | | | | |
| 16 | 31227282 | 31228414 | PYDC1 | | | | | |
| 16 | 31271287 | 31344213 | ITGAM | | | | | |
| 16 | 31366454 | 31394318 | ITGAX | | | | | |
| 16 | 31404632 | 31437826 | ITGAD | | | | | |
| 16 | 31439051 | 31439749 | COX6A2 | | | | | |
| 16 | 31446884 | 31454348 | ZNF843 | | | | | |
| 16 | 31469593 | 31478488 | ARMC5 | | | | | |
| 16 | 31483475 | 31489281 | TGFB1I1 | | | | | |
| 16 | 31494438 | 31502091 | SLC5A2 | | | | | |
| 16 | 31500795 | 31519740 | C16orf58 | | | | | |
| 16 | 31539172 | 31540125 | AHSP | | | | | |
| 16 | 31574542 | 31579036 | FRG2KP | | | | | |
| 16 | 31579087 | 31580845 | YBX3P1 | | | | | |
| 16 | 31711933 | 31718745 | CLUHP3 | | | | | |
| 16 | 31724549 | 31772886 | ZNF720 | | | | | |
| 16 | 31885078 | 31928629 | ZNF267 | | | | | |
| 16 | 32181363 | 32199434 | HERC2P4 | | | | | |
| 16 | 32264644 | 32267249 | TP53TG3D | | | | | |
| 16 | 32300867 | 32301302 | LOC390705 | | | | | |
| 16 | 32684848 | 32687450 | TP53TG3 | | | | | |
| 16 | 32684848 | 32687450 | TP53TG3B | | | | | |
| 16 | 32685408 | 32687448 | TP53TG3C | | | | | |
| 16 | 32888796 | 32896463 | SLC6A10P | | | | | |
| 16 | 33205582 | 33208179 | TP53TG3 | | | | | |
| 16 | 33205582 | 33208179 | TP53TG3B | | | | | |
| 16 | 33205584 | 33207624 | TP53TG3C | | | | | |
| 16 | 33262117 | 33264719 | TP53TG3 | | | | | |
| 16 | 33262117 | 33264719 | TP53TG3B | | | | | |

| | | | | | | | | |
|---|---|---|---|---|---|---|---|---|
| 16 | 33262119 | 33264159 | TP53TG3C | | | | | |
| 16 | 33298267 | 33298702 | LOC390705 | | | | | |
| 16 | 33571843 | 33586742 | ENPP7P13 | | | | | |
| 16 | 33961051 | 33962503 | LINC00273 | | | | | |
| 16 | 34403801 | 34404762 | UBE2MP1 | | | | | |
| 16 | 34597786 | 34624955 | LINC01566 | | | | | |
| 16 | 34711784 | 34714967 | FRG2DP | | | | | |
| 16 | 34739458 | 34741541 | TP53TG3HP | | | | | |
| 16 | 34980922 | 34990995 | FLJ26245 | | | | | |
| 16 | 46503248 | 46603009 | ANKRD26P1 | | | | | |
| 16 | 46614467 | 46655311 | SHCBP1 | | | | | |
| 16 | 46693588 | 46723144 | VPS35 | | VPS35 | VPS35 | | |
| 16 | 46723557 | 46732306 | ORC6 | | | | | |
| 16 | 46736193 | 46797158 | MYLK3 | | | | | |
| 16 | 46835958 | 46865074 | C16orf87 | | | | | |
| 16 | 46918291 | 46965209 | GPT2 | | | | | |
| 16 | 46989273 | 47007625 | DNAJA2 | | | | | |
| 16 | 47115430 | 47177936 | NETO2 | | NETO2 | NETO2 | | |
| 16 | 47177978 | 47495185 | ITFG1 | | | | | |
| 16 | 47495209 | 47735434 | PHKB | | | | | |
| 16 | 47883224 | 47921041 | LOC100507534 | | | | | |
| 16 | 47892491 | 47942342 | LOC101927132 | | | | | |
| 16 | 48116883 | 48180681 | ABCC12 | | | | | |
| 16 | 48200821 | 48269088 | ABCC11 | | | | | |
| 16 | 48274602 | 48420559 | MIR548AE2 | | | | | |
| 16 | 48278077 | 48387890 | LONP2 | | | | | |
| 16 | 48344528 | 48658037 | MIR5095 | | | | | |
| 16 | 48389595 | 48396910 | LOC100507577 | | | | | |
| 16 | 48394445 | 48419229 | SIAH1 | | | SIAH1 | | |
| 16 | 48572636 | 48644120 | N4BP1 | | | | | |
| 16 | 49311828 | 49315742 | CBLN1 | | | CBLN1 | | |
| 16 | 49407718 | 49433342 | C16orf78 | | | | | |
| 16 | 49524514 | 49891830 | ZNF423 | | | | | |
| 16 | 50059116 | 50070999 | CNEP1R1 | | | | | |
| 16 | 50099880 | 50139375 | HEATR3 | | | | | |
| 16 | 50186828 | 50269219 | PAPD5 | | | | | |
| 16 | 50300450 | 50352045 | ADCY7 | | | | | |
| 16 | 50326526 | 50326586 | MIR6771 | | | | | |
| 16 | 50352928 | 50402845 | BRD7 | | | | | |
| 16 | 50582240 | 50674771 | NKD1 | | | | | |
| 16 | 50700210 | 50715264 | SNX20 | | | | | |
| 16 | 50702765 | 50705550 | LOC101927272 | | | | | |
| 16 | 50706885 | 50715264 | SNX20 | | | | | |
| 16 | 50731049 | 50766988 | NOD2 | | | | | |
| 16 | 50775960 | 50835846 | CYLD | | | | | |
| 16 | 50776215 | 50776288 | MIR3181 | | | | | |
| 16 | 51051668 | 51069688 | LOC101927334 | | | | | |
| 16 | 51169885 | 51185183 | SALL1 | | SALL1 | SALL1 | | |
| 16 | 51796429 | 51806557 | LINC01571 | | | | | |
| 16 | 52060263 | 52107847 | C16orf97 | | | | | |
| 16 | 52116976 | 52119021 | LINC00919 | | | | | |
| 16 | 52119192 | 52132979 | LOC102467079 | | | | | |
| 16 | 52471917 | 52581714 | TOX3 | | TOX3 | | | |
| 16 | 52585998 | 52640887 | CASC16 | | | | | |
| 16 | 53088944 | 53361414 | CHD9 | | | | | |
| 16 | 53403273 | 53405041 | LOC643802 | | | | | |
| 16 | 53407404 | 53418171 | LOC102723373 | | | | | |
| 16 | 53468350 | 53525560 | RBL2 | | | | | |
| 16 | 53524951 | 53537216 | AKTIP | | | | | |
| 16 | 53633150 | 53737846 | RPGRIP1L | | | | | |
| 16 | 53737874 | 54148379 | FTO | | | | | |
| 16 | 54073304 | 54074285 | FTO-IT1 | | | | | |
| 16 | 54279456 | 54304791 | LOC100996338 | | | | | |
| 16 | 54317211 | 54320378 | IRX3 | | | | | |
| 16 | 54399918 | 54404611 | LOC100996345 | | | | | |
| 16 | 54881161 | 54882145 | LOC101927480 | | | | | |

| | | | | | | | | |
|---|---|---|---|---|---|---|---|---|
| 16 | 54952776 | 54963101 | CRNDE | | | | | |
| 16 | 54965110 | 54968395 | IRX5 | | | | | |
| 16 | 55358470 | 55364672 | IRX6 | | | | | |
| 16 | 55512741 | 55540603 | MMP2 | | | MMP2 | | |
| 16 | 55542912 | 55620582 | LPCAT2 | | | | | |
| 16 | 55600583 | 55601592 | CAPNS2 | | | | | |
| 16 | 55689541 | 55737700 | SLC6A2 | | | SLC6A2 | SLC6A2 | |
| 16 | 55758836 | 55784123 | CES1P2 | | | | | |
| 16 | 55794510 | 55808826 | CES1P1 | | | | | |
| 16 | 55836763 | 55867075 | CES1 | | | CES1 | | |
| 16 | 55880065 | 55989943 | CES5A | | | | | |
| 16 | 56126898 | 56225006 | LOC283856 | | | | | |
| 16 | 56225250 | 56391356 | GNAO1 | | GNAO1 | | | |
| 16 | 56226528 | 56228437 | DKFZP434H168 | | | | | |
| 16 | 56279431 | 56279535 | MIR3935 | | | | | |
| 16 | 56395363 | 56459450 | AMFR | | | | | |
| 16 | 56463047 | 56485261 | NUDT21 | | | | | |
| 16 | 56485423 | 56511407 | OGFOD1 | | | | | |
| 16 | 56518258 | 56554008 | BBS2 | | BBS2 | | | |
| 16 | 56598960 | 56602869 | MT4 | | | | | |
| 16 | 56623266 | 56625000 | MT3 | | | MT3 | | |
| 16 | 56642477 | 56643409 | MT2A | | | | | |
| 16 | 56651372 | 56652730 | MT1L | | | | | |
| 16 | 56659584 | 56661024 | MT1E | | | | | |
| 16 | 56666533 | 56667898 | MT1M | | | | | |
| 16 | 56669650 | 56670998 | MT1JP | | | | | |
| 16 | 56672577 | 56673999 | MT1A | | | | | |
| 16 | 56677598 | 56678853 | MT1DP | | | | | |
| 16 | 56685810 | 56687116 | MT1B | | | | | |
| 16 | 56691854 | 56693215 | MT1F | | | | | |
| 16 | 56700646 | 56701977 | MT1G | | | | | |
| 16 | 56703725 | 56705041 | MT1H | | | | | |
| 16 | 56710027 | 56711789 | MT1IP | | | | | |
| 16 | 56716381 | 56718108 | MT1X | | | | | |
| 16 | 56764016 | 56878861 | NUP93 | | | NUP93 | | |
| 16 | 56892429 | 56892513 | MIR138 | | | | | |
| 16 | 56899118 | 56949762 | SLC12A3 | | | SLC12A3 | | |
| 16 | 56938175 | 56938265 | MIR6863 | | | | | |
| 16 | 56966001 | 56977793 | HERPUD1 | | | | | |
| 16 | 56995834 | 57017756 | CETP | | | | | |
| 16 | 57050985 | 57117436 | NLRC5 | | | | | |
| 16 | 57126454 | 57181878 | CPNE2 | | | | | |
| 16 | 57186377 | 57219976 | FAM192A | | | | | |
| 16 | 57220195 | 57274387 | RSPRY1 | | | | | |
| 16 | 57279037 | 57287545 | ARL2BP | | | | | |
| 16 | 57290008 | 57318584 | PLLP | | | | | |
| 16 | 57392694 | 57400102 | CCL22 | | | CCL22 | | |
| 16 | 57406372 | 57418960 | CX3CL1 | | | | | |
| 16 | 57438678 | 57449974 | CCL17 | | | | | |
| 16 | 57462080 | 57481440 | CIAPIN1 | | | | | |
| 16 | 57481336 | 57495187 | COQ9 | | | | | |
| 16 | 57496550 | 57505921 | POLR2C | | | | | |
| 16 | 57505869 | 57520385 | DOK4 | | | | | |
| 16 | 57546089 | 57570477 | CCDC102A | | | | | |
| 16 | 57576548 | 57611100 | ADGRG5 | | | | | |
| 16 | 57653604 | 57698951 | ADGRG1 | | | | | |
| 16 | 57702156 | 57723290 | ADGRG3 | | | | | |
| 16 | 57728712 | 57765367 | DRC7 | | | | | |
| 16 | 57769659 | 57791162 | KATNB1 | | KATNB1 | KATNB1 | | |
| 16 | 57792128 | 57836936 | KIFC3 | | | KIFC3 | | |
| 16 | 57806200 | 57806264 | MIR6772 | | | | | |
| 16 | 57844548 | 57850831 | LOC388282 | | | | | |
| 16 | 57916243 | 58005020 | CNGB1 | | CNGB1 | | | |
| 16 | 58010338 | 58022017 | TEPP | | | | | |
| 16 | 58028571 | 58033762 | ZNF319 | | | | | |
| 16 | 58035276 | 58055527 | USB1 | | | | | |

| | | | | | | | | |
|---|---|---|---|---|---|---|---|---|
| 16 | 58059469 | 58080804 | MMP15 | | | | | |
| 16 | 58147496 | 58163296 | CFAP20 | | | | | |
| 16 | 58191811 | 58231782 | CSNK2A2 | | | | | |
| 16 | 58283839 | 58317734 | CCDC113 | | | | | |
| 16 | 58313900 | 58328951 | PRSS54 | | | | | |
| 16 | 58426297 | 58440048 | GINS3 | | | | | |
| 16 | 58497548 | 58547523 | NDRG4 | | NDRG4 | | | |
| 16 | 58549382 | 58554436 | SETD6 | | | | | |
| 16 | 58553849 | 58663790 | CNOT1 | | | | | |
| 16 | 58582402 | 58582537 | SNORA46 | | | | | |
| 16 | 58593699 | 58593835 | SNORA50A | | | | | |
| 16 | 58699012 | 58718681 | SLC38A7 | | | | | |
| 16 | 58741034 | 58768261 | GOT2 | | | | | |
| 16 | 59788044 | 59789095 | APOOP5 | | | | | |
| 16 | 59889256 | 60087875 | LOC101927580 | | | | | |
| 16 | 60392358 | 60393667 | LOC729159 | | | | | |
| 16 | 61089610 | 61089670 | MIR4426 | | | | | |
| 16 | 61685914 | 62070739 | CDH8 | | | | | |
| 16 | 64977656 | 65156040 | CDH11 | | | | | |
| 16 | 65175658 | 65210476 | LOC101927650 | | | | | |
| 16 | 65318401 | 65610203 | LINC00922 | | | | | |
| 16 | 66400509 | 66438689 | CDH5 | | | | | |
| 16 | 66442426 | 66444803 | LINC00920 | | | | | |
| 16 | 66461199 | 66510305 | BEAN1 | | | | | |
| 16 | 66541905 | 66584315 | TK2 | | | TK2 | | |
| 16 | 66586465 | 66600190 | CKLF | | | | | |
| 16 | 66586465 | 66613038 | CKLF-CMTM1 | | | | | |
| 16 | 66600293 | 66613038 | CMTM1 | | | | | |
| 16 | 66613350 | 66622177 | CMTM2 | | | | | |
| 16 | 66637934 | 66647795 | CMTM3 | | | | | |
| 16 | 66648652 | 66730610 | CMTM4 | | | | | |
| 16 | 66754798 | 66785525 | DYNC1LI2 | | | | | |
| 16 | 66785654 | 66788645 | LOC106699570 | | | | | |
| 16 | 66788878 | 66835523 | CCDC79 | | | | | |
| 16 | 66836780 | 66864879 | NAE1 | | | | | |
| 16 | 66878281 | 66888049 | CA7 | | | | | |
| 16 | 66914382 | 66925002 | PDP2 | | | PDP2 | | |
| 16 | 66942024 | 66952887 | CDH16 | | | | | |
| 16 | 66955581 | 66959439 | RRAD | | | RRAD | | |
| 16 | 66965957 | 66968326 | FAM96B | | | | | |
| 16 | 66968346 | 66978994 | CES2 | | | | | |
| 16 | 66995131 | 67009052 | CES3 | | | | | |
| 16 | 67022491 | 67043659 | CES4A | | | | | |
| 16 | 67063049 | 67134958 | CBFB | | | | | |
| 16 | 67143914 | 67182442 | C16orf70 | | | | | |
| 16 | 67182004 | 67184902 | B3GNT9 | | | | | |
| 16 | 67188088 | 67193812 | TRADD | | | | | |
| 16 | 67193890 | 67198077 | FBXL8 | | | | | |
| 16 | 67197287 | 67203848 | HSF4 | | | | | |
| 16 | 67204399 | 67209640 | NOL3 | | | | | |
| 16 | 67209504 | 67217883 | KIAA0895L | | | | | |
| 16 | 67218281 | 67224107 | EXOC3L1 | | | | | |
| 16 | 67226067 | 67232821 | E2F4 | | | | | |
| 16 | 67233027 | 67237927 | ELMO3 | | | | | |
| 16 | 67236223 | 67236298 | MIR328 | | | | | |
| 16 | 67241041 | 67260901 | LRRC29 | | | | | |
| 16 | 67261015 | 67263182 | TMEM208 | | | | | |
| 16 | 67263291 | 67281425 | FHOD1 | | | | | |
| 16 | 67282854 | 67306094 | SLC9A5 | | | | | |
| 16 | 67312059 | 67323403 | PLEKHG4 | | | | | |
| 16 | 67323392 | 67360661 | KCTD19 | | | | | |
| 16 | 67360746 | 67419109 | LRRC36 | | | | | |
| 16 | 67423709 | 67427438 | TPPP3 | | | | | |
| 16 | 67428321 | 67450339 | ZDHHC1 | | | | | |
| 16 | 67465035 | 67471454 | HSD11B2 | | | | | |
| 16 | 67471916 | 67515089 | ATP6V0D1 | | ATP6V0D1 | | | |

| | | | | | | | | |
|---|---|---|---|---|---|---|---|---|
| 16 | 67516473 | 67517716 | AGRP | | | | | |
| 16 | 67551608 | 67562648 | LOC100505942 | | | | | |
| 16 | 67562716 | 67580691 | FAM65A | | | | | |
| 16 | 67596309 | 67673088 | CTCF | | | CTCF | | |
| 16 | 67679029 | 67691472 | RLTPR | | | | | |
| 16 | 67691414 | 67694718 | ACD | | | | | |
| 16 | 67694850 | 67696681 | PARD6A | | | | | |
| 16 | 67696849 | 67700628 | ENKD1 | | | | | |
| 16 | 67700716 | 67702661 | C16orf86 | | | | | |
| 16 | 67708435 | 67753273 | GFOD2 | | | | | |
| 16 | 67757004 | 67840555 | RANBP10 | | | | | |
| 16 | 67840780 | 67861971 | TSNAXIP1 | | | | | |
| 16 | 67862059 | 67881361 | CENPT | | | | | |
| 16 | 67876212 | 67878098 | THAP11 | | | | | |
| 16 | 67880818 | 67905219 | NUTF2 | | | | | |
| 16 | 67906925 | 67918417 | EDC4 | | | | | |
| 16 | 67918780 | 67920271 | NRN1L | | | | | |
| 16 | 67927174 | 67963581 | PSKH1 | | | | | |
| 16 | 67963472 | 67965778 | CTRL | | | | | |
| 16 | 67968406 | 67970780 | PSMB10 | | | | | |
| 16 | 67973786 | 67978015 | LCAT | | | LCAT | | |
| 16 | 67977376 | 68002597 | SLC12A4 | | | | | |
| 16 | 68009565 | 68014452 | DPEP3 | | | | | |
| 16 | 68021292 | 68033421 | DPEP2 | | | | | |
| 16 | 68047338 | 68049814 | LOC100131303 | | | | | |
| 16 | 68055176 | 68057770 | DDX28 | | | | | |
| 16 | 68056846 | 68113226 | DUS2 | | | | | |
| 16 | 68119268 | 68263162 | NFATC3 | | | | | |
| 16 | 68262449 | 68270136 | ESRP2 | | | | | |
| 16 | 68267328 | 68267402 | MIR6773 | | | | | |
| 16 | 68279246 | 68294961 | PLA2G15 | | | | | |
| 16 | 68298418 | 68335726 | SLC7A6 | | | | | |
| 16 | 68334517 | 68344868 | SLC7A6OS | | | | | |
| 16 | 68344876 | 68391169 | PRMT7 | | | | | |
| 16 | 68392229 | 68482409 | SMPD3 | | | | | |
| 16 | 68573115 | 68609975 | ZFP90 | | | | | |
| 16 | 68678738 | 68732970 | CDH3 | | | | | |
| 16 | 68771192 | 68869445 | CDH1 | | | CDH1 | | |
| 16 | 68776418 | 68776474 | MIR7641 | | | | | |
| 16 | 68877508 | 69119085 | TANGO6 | | | | | |
| 16 | 69139466 | 69151570 | HAS3 | | | | | |
| 16 | 69151911 | 69166493 | CHTF8 | | | | | |
| 16 | 69166498 | 69202937 | UTP4 | | | | | |
| 16 | 69221049 | 69342955 | SNTB2 | | | SNTB2 | | |
| 16 | 69345286 | 69358946 | VPS4A | | | | | |
| 16 | 69362523 | 69364498 | PDF | | | | | |
| 16 | 69362523 | 69373526 | COG8 | | | | | |
| 16 | 69373414 | 69377013 | NIP7 | | | | | |
| 16 | 69377148 | 69385712 | TMED6 | | | | | |
| 16 | 69389463 | 69419891 | TERF2 | | | | | |
| 16 | 69458497 | 69500167 | CYB5B | | | | | |
| 16 | 69599710 | 69599771 | MIR1538 | | | | | |
| 16 | 69599868 | 69738569 | NFAT5 | | | | | |
| 16 | 69743303 | 69760571 | NQO1 | | | | | |
| 16 | 69775756 | 69788871 | NOB1 | | | | | |
| 16 | 69796186 | 69975644 | WWP2 | | | | | |
| 16 | 69966983 | 69967083 | MIR140 | | | | | |
| 16 | 69984607 | 69998250 | CLEC18A | | | | | |
| 16 | 70010201 | 70099851 | PDXDC2P | | | | | |
| 16 | 70064248 | 70064325 | MIR1972 | | | | | |
| 16 | 70147528 | 70195184 | PDPR | | | | | |
| 16 | 70190242 | 70207351 | LOC400541 | | | | | |
| 16 | 70208075 | 70220798 | CLEC18C | | | | | |
| 16 | 70253483 | 70259936 | SMG1P7 | | | | | |
| 16 | 70284133 | 70285833 | EXOSC6 | | | | | |
| 16 | 70286296 | 70323412 | AARS | | AARS | | | |

| | | | | | | | | |
|---|---|---|---|---|---|---|---|---|
| 16 | 70323669 | 70367735 | DDX19B | | | | | |
| 16 | 70349542 | 70380650 | LOC100506083 | | | | | |
| 16 | 70380823 | 70407281 | DDX19A | | | | | |
| 16 | 70413337 | 70472991 | ST3GAL2 | | | | | |
| 16 | 70488497 | 70514177 | FUK | | | | | |
| 16 | 70514471 | 70557457 | COG4 | | | | | |
| 16 | 70557690 | 70611571 | SF3B3 | | | | | |
| 16 | 70563401 | 70563502 | SNORD111B | | | | | |
| 16 | 70571907 | 70572001 | SNORD111 | | | | | |
| 16 | 70613797 | 70694585 | IL34 | | | | | |
| 16 | 70695106 | 70719954 | MTSS1L | | | | | |
| 16 | 70721341 | 70807154 | VAC14 | | | | | |
| 16 | 70841286 | 71264625 | HYDIN | | | | | |
| 16 | 71316202 | 71323509 | CMTR2 | | | | | |
| 16 | 71392615 | 71424342 | CALB2 | | | | | |
| 16 | 71481502 | 71496155 | ZNF23 | | | | | |
| 16 | 71507975 | 71523254 | ZNF19 | | | | | |
| 16 | 71560022 | 71572493 | CHST4 | | | | | |
| 16 | 71598918 | 71610998 | TAT | | | | | |
| 16 | 71660055 | 71675868 | MARVELD3 | | | | | |
| 16 | 71678828 | 71758604 | PHLPP2 | | | | | |
| 16 | 71732469 | 71732604 | SNORA70D | | | | | |
| 16 | 71762904 | 71842976 | AP1G1 | | | AP1G1 | | |
| 16 | 71792304 | 71792390 | SNORD71 | | | | | |
| 16 | 71879893 | 71891236 | ATXN1L | | | | | |
| 16 | 71893582 | 71918093 | ZNF821 | | | | | |
| 16 | 71928310 | 71964540 | IST1 | | | | | |
| 16 | 71963440 | 72033877 | PKD1L3 | | | | | |
| 16 | 72042642 | 72059316 | DHODH | | | | | |
| 16 | 72088490 | 72094955 | HP | | | | | |
| 16 | 72097124 | 72111145 | HPR | | | | | |
| 16 | 72118755 | 72128215 | TXNL4B | | | | | |
| 16 | 72127614 | 72146811 | DHX38 | | | | | |
| 16 | 72152995 | 72206349 | PMFBP1 | | | | | |
| 16 | 72317199 | 72698908 | LINC01572 | | | | | |
| 16 | 72816785 | 73092534 | ZFHX3 | | | | | |
| 16 | 73126247 | 73127672 | HCCAT5 | | | | | |
| 16 | 73160536 | 73178346 | C16orf47 | | | | | |
| 16 | 73420703 | 73455295 | LINC01568 | | | | | |
| 16 | 74226290 | 74249420 | LOC101928035 | | | | | |
| 16 | 74330672 | 74340186 | PSMD7 | | | | | |
| 16 | 74366303 | 74402153 | LOC283922 | | | | | |
| 16 | 74411849 | 74426012 | NPIPB15 | | | | | |
| 16 | 74442528 | 74455368 | CLEC18B | | | | | |
| 16 | 74481325 | 74641042 | GLG1 | | | | | |
| 16 | 74655296 | 74700779 | RFWD3 | | | | | |
| 16 | 74705752 | 74735046 | MLKL | | | | | |
| 16 | 74746855 | 74808729 | FA2H | | FA2H | | | |
| 16 | 74907470 | 75019017 | WDR59 | | | | | |
| 16 | 75032914 | 75144892 | ZNRF1 | | | | | |
| 16 | 75145757 | 75150670 | LDHD | | | | | |
| 16 | 75182338 | 75206132 | ZFP1 | | | | | |
| 16 | 75237993 | 75241072 | CTRB2 | | | | | |
| 16 | 75252883 | 75258822 | CTRB1 | | | | | |
| 16 | 75260280 | 75262101 | LOC100506281 | | | | | |
| 16 | 75262927 | 75301951 | BCAR1 | | | | | |
| 16 | 75327607 | 75467387 | CFDP1 | | | | | |
| 16 | 75477141 | 75498635 | TMEM170A | | | | | |
| 16 | 75507021 | 75528926 | CHST6 | | | CHST6 | | |
| 16 | 75562427 | 75569068 | CHST5 | | | | | |
| 16 | 75572014 | 75590184 | TMEM231 | | | | | |
| 16 | 75600248 | 75611779 | GABARAPL2 | | | GABARAPL2 | | |
| 16 | 75632246 | 75657221 | ADAT1 | | | | | |
| 16 | 75661621 | 75681585 | KARS | | | | | |
| 16 | 75681634 | 75691341 | TERF2IP | | | | | |
| 16 | 76311175 | 76593135 | CNTNAP4 | | | CNTNAP4 | | |

| | | | | | | | | | |
|---|---|---|---|---|---|---|---|---|---|
| 16 | 76668894 | 76692375 | LOC101928203 | | | | | | |
| 16 | 76902832 | 76902916 | MIR4719 | | | | | | |
| 16 | 77224815 | 77233543 | MON1B | | | | | | |
| 16 | 77233348 | 77246976 | SYCE1L | | | | | | |
| 16 | 77316024 | 77469011 | ADAMTS18 | | ADAMTS18 | ADAMTS18 | | | |
| 16 | 77756388 | 77776157 | NUDT7 | | | | | | |
| 16 | 77822460 | 78014004 | VAT1L | | | | | | |
| 16 | 78056442 | 78066001 | CLEC3A | | | | | | |
| 16 | 78133309 | 79246564 | WWOX | | | | | | |
| 16 | 79627744 | 79634622 | MAF | | | | | | |
| 16 | 79755208 | 79804429 | MAFTRR | | | | | | |
| 16 | 79804367 | 79832987 | LINC01229 | | | | | | |
| 16 | 80189854 | 80597032 | LOC102724084 | | | | | | |
| 16 | 80574630 | 80584706 | DYNLRB2 | | | DYNLRB2 | | | |
| 16 | 80600999 | 80603507 | LINC01227 | | | | | | |
| 16 | 80637675 | 80838175 | CDYL2 | | | | | | |
| 16 | 80862631 | 80926493 | PRCAT47 | | | | | | |
| 16 | 81009698 | 81040502 | CMC2 | | | | | | |
| 16 | 81040102 | 81066709 | CENPN | | | | | | |
| 16 | 81069457 | 81080951 | ATMIN | | | | | | |
| 16 | 81087101 | 81110872 | C16orf46 | | | | | | |
| 16 | 81115551 | 81129980 | GCSH | | GCSH | GCSH | | | |
| 16 | 81134483 | 81253975 | PKD1L2 | | | | | | |
| 16 | 81272295 | 81324747 | BCO1 | | | | | | |
| 16 | 81348570 | 81413803 | GAN | | | | | | |
| 16 | 81418622 | 81418698 | MIR4720 | | | | | | |
| 16 | 81478774 | 81745367 | CMIP | | | | | | |
| 16 | 81567506 | 81567571 | MIR7854 | | | | | | |
| 16 | 81644952 | 81645013 | MIR6504 | | | | | | |
| 16 | 81698958 | 81700879 | LOC100129617 | | | | | | |
| 16 | 81812862 | 81996298 | PLCG2 | | | PLCG2 | | | |
| 16 | 82031250 | 82045093 | SDR42E1 | | | | | | |
| 16 | 82068857 | 82132139 | HSD17B2 | | | | | | |
| 16 | 82181766 | 82203829 | MPHOSPH6 | | | | | | |
| 16 | 82660398 | 83830215 | CDH13 | | | CDH13 | | | |
| 16 | 82722535 | 82722624 | MIR8058 | | | | | | |
| 16 | 82806923 | 82863243 | LOC101928446 | | | | | | |
| 16 | 82986834 | 83023903 | LOC101928417 | | | | | | |
| 16 | 83541950 | 83542013 | MIR3182 | | | | | | |
| 16 | 83830840 | 83837581 | LOC102724163 | | | | | | |
| 16 | 83841507 | 83846607 | HSBP1 | | | | | | |
| 16 | 83932729 | 83949787 | MLYCD | | | MLYCD | | | |
| 16 | 83986826 | 83999937 | OSGIN1 | | | | | | |
| 16 | 84002236 | 84036379 | NECAB2 | | | | | | |
| 16 | 84043271 | 84075762 | SLC38A8 | | | | | | |
| 16 | 84087365 | 84150548 | MBTPS1 | | | | | | |
| 16 | 84155743 | 84178800 | HSDL1 | | | | | | |
| 16 | 84178864 | 84211526 | DNAAF1 | | DNAAF1 | | | | |
| 16 | 84211452 | 84220676 | TAF1C | | | TAF1C | | | |
| 16 | 84224722 | 84230772 | ADAD2 | | | | | | |
| 16 | 84254740 | 84273356 | KCNG4 | | | | | | |
| 16 | 84328311 | 84363457 | WFDC1 | | | | | | |
| 16 | 84402128 | 84497793 | ATP2C2 | | | | | | |
| 16 | 84509965 | 84538288 | TLDC1 | | | | | | |
| 16 | 84599203 | 84651702 | COTL1 | | | | | | |
| 16 | 84682116 | 84701292 | KLHL36 | | | | | | |
| 16 | 84733554 | 84813527 | USP10 | | | | | | |
| 16 | 84853586 | 84943116 | CRISPLD2 | | | | | | |
| 16 | 85008066 | 85045141 | ZDHHC7 | | | | | | |
| 16 | 85061356 | 85127828 | KIAA0513 | | | | | | |
| 16 | 85131957 | 85146114 | FAM92B | | | | | | |
| 16 | 85170755 | 85183049 | LOC400548 | | | | | | |
| 16 | 85316563 | 85321685 | LINC00311 | | | | | | |
| 16 | 85339831 | 85339931 | MIR5093 | | | | | | |
| 16 | 85645028 | 85709812 | GSE1 | | | | | | |
| 16 | 85711279 | 85722588 | GINS2 | | | | | | |

| | | | | | | | | | |
|---|---|---|---|---|---|---|---|---|---|
| 16 | 85741123 | 85784689 | C16orf74 | | | | | | |
| 16 | 85775226 | 85775306 | MIR1910 | | | | | | |
| 16 | 85812230 | 85833148 | EMC8 | | | | | | |
| 16 | 85833172 | 85840609 | COX4I1 | | | COX4I1 | | | |
| 16 | 85932773 | 85956211 | IRF8 | | | | | | |
| 16 | 85951952 | 85952022 | MIR6774 | | | | | | |
| 16 | 86229786 | 86233324 | LINC01082 | | | | | | |
| 16 | 86259185 | 86319853 | LINC01081 | | | | | | |
| 16 | 86320036 | 86326995 | LOC146513 | | | | | | |
| 16 | 86365455 | 86379285 | LINC00917 | | | | | | |
| 16 | 86508130 | 86542466 | FENDRR | | | | | | |
| 16 | 86544132 | 86548070 | FOXF1 | | | | | | |
| 16 | 86563781 | 86588841 | MTHFSD | | | | | | |
| 16 | 86588925 | 86590905 | FLJ30679 | | | | | | |
| 16 | 86598750 | 86602537 | FOXC2 | | | | | | |
| 16 | 86612114 | 86615304 | FOXL1 | | | | | | |
| 16 | 87091389 | 87097597 | LOC440390 | | | | | | |
| 16 | 87245720 | 87260035 | LOC101928708 | | | | | | |
| 16 | 87305511 | 87326058 | LOC101928682 | | | | | | |
| 16 | 87336403 | 87351026 | C16orf95 | | | | | | |
| 16 | 87360592 | 87425713 | FBXO31 | | | | | | |
| 16 | 87425800 | 87438380 | MAP1LC3B | | MAP1LC3B | MAP1LC3B | | | |
| 16 | 87439851 | 87525460 | ZCCHC14 | | | | | | |
| 16 | 87527792 | 87549236 | LOC101928737 | | | | | | |
| 16 | 87635440 | 87731761 | JPH3 | | JPH3 | | | | |
| 16 | 87741417 | 87799598 | KLHDC4 | | | | | | |
| 16 | 87799936 | 87812754 | LOC102724467 | | | | | | |
| 16 | 87863628 | 87903100 | SLC7A5 | | | | | | |
| 16 | 87868197 | 87868266 | MIR6775 | | | | | | |
| 16 | 87921624 | 87970112 | CA5A | | CA5A | | | | |
| 16 | 87985037 | 88110924 | BANP | | | | | | |
| 16 | 88133014 | 88134591 | LOC400553 | | | | | | |
| 16 | 88227886 | 88228823 | LOC101928880 | | | | | | |
| 16 | 88493878 | 88507165 | ZNF469 | | ZNF469 | | | | |
| 16 | 88520013 | 88601574 | ZFPM1 | | | ZFPM1 | | | |
| 16 | 88535325 | 88535439 | MIR5189 | | | | | | |
| 16 | 88636788 | 88698372 | ZC3H18 | | | | | | |
| 16 | 88705000 | 88706882 | IL17C | | | | | | |
| 16 | 88709696 | 88717492 | CYBA | | | | | | |
| 16 | 88718347 | 88729557 | MVD | | | | | | |
| 16 | 88729780 | 88752882 | SNAI3 | | | | | | |
| 16 | 88762902 | 88772829 | RNF166 | | | | | | |
| 16 | 88772870 | 88781786 | CTU2 | | | | | | |
| 16 | 88781745 | 88851628 | PIEZO1 | | | | | | |
| 16 | 88782685 | 88782745 | MIR4722 | | | | | | |
| 16 | 88797587 | 88807833 | LOC100289580 | | | | | | |
| 16 | 88809174 | 88812156 | LOC339059 | | | | | | |
| 16 | 88870185 | 88875666 | CDT1 | | | | | | |
| 16 | 88875876 | 88878342 | APRT | | | | | | |
| 16 | 88880141 | 88923374 | GALNS | | | | | | |
| 16 | 88922627 | 88929094 | TRAPPC2L | | | | | | |
| 16 | 88929747 | 88933068 | PABPN1L | | | | | | |
| 16 | 88941262 | 89043504 | CBFA2T3 | | | | | | |
| 16 | 89006446 | 89017932 | LOC100129697 | | | | | | |
| 16 | 89160216 | 89222254 | ACSF3 | | | | | | |
| 16 | 89225627 | 89230083 | LINC00304 | | | | | | |
| 16 | 89232790 | 89235555 | LOC400558 | | | | | | |
| 16 | 89238162 | 89261900 | CDH15 | | | | | | |
| 16 | 89262168 | 89266529 | SLC22A31 | | | | | | |
| 16 | 89284110 | 89295965 | ZNF778 | | | | | | |
| 16 | 89334028 | 89556969 | ANKRD11 | | | | | | |
| 16 | 89387540 | 89391518 | LOC100287036 | | | | | | |
| 16 | 89497325 | 89520902 | LOC101927817 | | | | | | |
| 16 | 89574795 | 89624176 | SPG7 | | | SPG7 | | | |
| 16 | 89627064 | 89633237 | RPL13 | | | | | | |
| 16 | 89627837 | 89627909 | SNORD68 | | | | | | |

| | | | | | | | | |
|---|---|---|---|---|---|---|---|---|
| 16 | 89642175 | 89663654 | CPNE7 | | | | | |
| 16 | 89679715 | 89704839 | DPEP1 | | | | | |
| 16 | 89710838 | 89724193 | CHMP1A | | | CHMP1A | | |
| 16 | 89724151 | 89736866 | SPATA33 | | | | | |
| 16 | 89753075 | 89762772 | CDK10 | | | | | |
| 16 | 89762764 | 89768121 | SPATA2L | | | | | |
| 16 | 89773540 | 89784573 | VPS9D1 | | | | | |
| 16 | 89786775 | 89807332 | ZNF276 | | | | | |
| 16 | 89803958 | 89883065 | FANCA | | | FANCA | | |
| 16 | 89894906 | 89937727 | SPIRE2 | | | | | |
| 16 | 89939993 | 89977792 | TCF25 | | | TCF25 | | |
| 16 | 89984286 | 89987385 | MC1R | | | MC1R | | |
| 16 | 89988416 | 90002505 | TUBB3 | | TUBB3 | | | |
| 16 | 90015138 | 90034468 | DEF8 | | | | | |
| 16 | 90036182 | 90039240 | CENPBD1 | | | | | |
| 16 | 90038987 | 90067195 | AFG3L1P | | | | | |
| 16 | 90071272 | 90086539 | DBNDD1 | | | | | |
| 16 | 90086036 | 90096309 | GAS8 | | | | | |
| 16 | 90106168 | 90114191 | URAHP | | | | | |
| 16 | 90122973 | 90142338 | PRDM7 | | | | | |
| 16 | 90168671 | 90244014 | FAM157C | | | | | |
| 17 | 6010 | 31421 | DOC2B | | | | DOC2B | |
| 17 | 33614 | 37042 | LOC100506371 | | | | | |
| 17 | 62179 | 202633 | RPH3AL | | | | RPH3AL | |
| 17 | 180995 | 183279 | LOC100506388 | | | | | |
| 17 | 260117 | 264457 | C17orf97 | | | | | |
| 17 | 289770 | 295731 | FAM101B | | | | | |
| 17 | 411907 | 618096 | VPS53 | | | | | |
| 17 | 635785 | 646208 | FAM57A | | | | | |
| 17 | 647660 | 655501 | GEMIN4 | | | | | |
| 17 | 655572 | 658576 | DBIL5P | | | | | |
| 17 | 662548 | 685571 | GLOD4 | | | | | |
| 17 | 685568 | 695749 | RNMTL1 | | | | | |
| 17 | 702552 | 882998 | NXN | | | | | |
| 17 | 900356 | 905390 | TIMM22 | | | | | |
| 17 | 906757 | 1090616 | ABR | | ABR | | | |
| 17 | 925715 | 925799 | MIR3183 | | | | | |
| 17 | 1173857 | 1174565 | BHLHA9 | | | | | |
| 17 | 1182956 | 1204281 | TUSC5 | | | | | |
| 17 | 1247833 | 1303556 | YWHAE | | YWHAE | YWHAE | | |
| 17 | 1324646 | 1359561 | CRK | | | | | |
| 17 | 1367479 | 1396001 | MYO1C | | | | | |
| 17 | 1397870 | 1420182 | INPP5K | | | | | |
| 17 | 1420212 | 1466110 | PITPNA | | | | | |
| 17 | 1472547 | 1532180 | SLC43A2 | | | | | |
| 17 | 1537151 | 1549083 | SCARF1 | | | | | |
| 17 | 1549444 | 1553392 | RILP | | | | | |
| 17 | 1553922 | 1588176 | PRPF8 | | | | | |
| 17 | 1606083 | 1613662 | TLCD2 | | | | | |
| 17 | 1614797 | 1619566 | MIR22HG | | | | | |
| 17 | 1617196 | 1617281 | MIR22 | | | | | |
| 17 | 1619816 | 1641893 | WDR81 | | | | | |
| 17 | 1646129 | 1658559 | SERPINF2 | | | | | |
| 17 | 1665258 | 1680859 | SERPINF1 | | | SERPINF1 | | |
| 17 | 1682828 | 1733175 | SMYD4 | | | | | |
| 17 | 1733272 | 1802848 | RPA1 | | | | | |
| 17 | 1837970 | 1928639 | RTN4RL1 | | | | | |
| 17 | 1933430 | 1946725 | DPH1 | | | | | |
| 17 | 1945276 | 1946725 | OVCA2 | | | | | |
| 17 | 1953201 | 1953302 | MIR132 | | | | | |
| 17 | 1953564 | 1953674 | MIR212 | | | | | |
| 17 | 1958392 | 1962981 | HIC1 | | | | | |
| 17 | 1963132 | 2207069 | SMG6 | | | SMG6 | | |
| 17 | 2135971 | 2136904 | LOC101927839 | | | | | |
| 17 | 2207236 | 2228558 | SRR | | | | | |
| 17 | 2225981 | 2240678 | TSR1 | | | | | |

| | | | | | | | | | |
|---|---|---|---|---|---|---|---|---|---|
| 17 | 2232418 | 2232504 | SNORD91B | | | | | | |
| 17 | 2233572 | 2233664 | SNORD91A | | | | | | |
| 17 | 2240805 | 2284348 | SGSM2 | | | | | | |
| 17 | 2287353 | 2304258 | MNT | | | | | | |
| 17 | 2310274 | 2318730 | LOC284009 | | | | | | |
| 17 | 2319347 | 2415200 | METTL16 | | | | | | |
| 17 | 2496922 | 2588909 | PAFAH1B1 | PAFAH1B1 | PAFAH1B1 | PAFAH1B1 | | | |
| 17 | 2592679 | 2614927 | CLUH | | | | | | |
| 17 | 2596154 | 2596213 | MIR6776 | | | | | | |
| 17 | 2651371 | 2651476 | MIR1253 | | | | | | |
| 17 | 2699731 | 2941035 | RAP1GAP2 | | | | | | |
| 17 | 2865540 | 2869189 | LOC101927911 | | | | | | |
| 17 | 2965962 | 2966901 | OR1D5 | | | OR1D5 | | | |
| 17 | 2995351 | 2996290 | OR1D2 | | | | | | |
| 17 | 3029903 | 3030845 | OR1G1 | | | OR1G1 | | | |
| 17 | 3100810 | 3101742 | OR1A2 | | | | | | |
| 17 | 3118914 | 3119844 | OR1A1 | | | | | | |
| 17 | 3143969 | 3144559 | OR1D4 | | | | | | |
| 17 | 3181183 | 3182268 | OR3A2 | | | | | | |
| 17 | 3194928 | 3195876 | OR3A1 | | | | | | |
| 17 | 3213538 | 3214740 | OR3A4P | | | | | | |
| 17 | 3300759 | 3301704 | OR1E1 | | | | | | |
| 17 | 3323861 | 3324827 | OR3A3 | | | OR3A3 | | | |
| 17 | 3336163 | 3337135 | OR1E2 | | | | | | |
| 17 | 3343305 | 3375142 | SPATA22 | | | | | | |
| 17 | 3377403 | 3402700 | ASPA | | ASPA | | | | |
| 17 | 3413795 | 3461289 | TRPV3 | | | | | | |
| 17 | 3468739 | 3512705 | TRPV1 | | | | | | |
| 17 | 3511555 | 3539616 | SHPK | | | | | | |
| 17 | 3539761 | 3566397 | CTNS | | | CTNS | | | |
| 17 | 3566186 | 3571973 | TAX1BP3 | | | | | | |
| 17 | 3566186 | 3599698 | P2RX5-TAX1BP3 | | | | | | |
| 17 | 3572016 | 3572963 | EMC6 | | | | | | |
| 17 | 3576521 | 3599698 | P2RX5 | | | | | | |
| 17 | 3617918 | 3704537 | ITGAE | | | | | | |
| 17 | 3627196 | 3629992 | GSG2 | | | | | | |
| 17 | 3710044 | 3749545 | NCBP3 | | | | | | |
| 17 | 3763616 | 3796337 | CAMKK1 | | | CAMKK1 | | | |
| 17 | 3799884 | 3819960 | P2RX1 | | | | P2RX1 | | |
| 17 | 3827162 | 3867758 | ATP2A3 | | | | | | |
| 17 | 3907738 | 4046253 | ZZEF1 | | | | | | |
| 17 | 4046461 | 4060995 | CYB5D2 | | | | | | |
| 17 | 4066664 | 4167274 | ANKFY1 | | | ANKFY1 | | | |
| 17 | 4172511 | 4269969 | UBE2G1 | | | | | | |
| 17 | 4174725 | 4175189 | LOC103021295 | | | | | | |
| 17 | 4337218 | 4391499 | SPNS3 | | | | | | |
| 17 | 4402128 | 4443228 | SPNS2 | | | | | | |
| 17 | 4442190 | 4458681 | MYBBP1A | | | | | | |
| 17 | 4460221 | 4463891 | GGT6 | | | | | | |
| 17 | 4487275 | 4511614 | SMTNL2 | | | | | | |
| 17 | 4534213 | 4544960 | ALOX15 | | | | | | |
| 17 | 4574678 | 4607632 | PELP1 | | | | | | |
| 17 | 4607524 | 4608824 | LOC101559451 | | | | | | |
| 17 | 4613788 | 4624795 | ARRB2 | | | | | | |
| 17 | 4634702 | 4636905 | MED11 | | | | | | |
| 17 | 4636823 | 4643223 | CXCL16 | | | CXCL16 | | | |
| 17 | 4643309 | 4649414 | ZMYND15 | | | | | | |
| 17 | 4675186 | 4686506 | TM4SF5 | | | TM4SF5 | | | |
| 17 | 4688579 | 4689729 | VMO1 | | | | | | |
| 17 | 4692253 | 4693884 | GLTPD2 | | | | | | |
| 17 | 4699438 | 4701798 | PSMB6 | | | | | | |
| 17 | 4710395 | 4726727 | PLD2 | | | PLD2 | | | |
| 17 | 4736634 | 4801356 | MINK1 | | MINK1 | | | | |
| 17 | 4801063 | 4806369 | CHRNE | | | | CHRNE | CHRNE | |
| 17 | 4802947 | 4806227 | C17orf107 | | | | | | |
| 17 | 4835569 | 4838325 | GP1BA | | | | | | |

| | | | | | | | | |
|---|---|---|---|---|---|---|---|---|
| 17 | 4840425 | 4843462 | SLC25A11 | | | | | |
| 17 | 4843629 | 4848517 | RNF167 | | | | | |
| 17 | 4848944 | 4852381 | PFN1 | | | | | |
| 17 | 4854383 | 4860426 | ENO3 | | | | | |
| 17 | 4862520 | 4871132 | SPAG7 | | | | | |
| 17 | 4871286 | 4890960 | CAMTA2 | | | | | |
| 17 | 4872996 | 4873066 | MIR6864 | | | | | |
| 17 | 4873380 | 4873445 | MIR6865 | | | | | |
| 17 | 4891424 | 4900905 | INCA1 | | | | | |
| 17 | 4901242 | 4931694 | KIF1C | | | | | |
| 17 | 4922509 | 4923388 | LOC102724009 | | | | | |
| 17 | 4935896 | 4938727 | SLC52A1 | | | | | |
| 17 | 4981753 | 4999669 | ZFP3 | | | | | |
| 17 | 5009030 | 5026397 | ZNF232 | | | | | |
| 17 | 5019732 | 5078326 | USP6 | | | | | |
| 17 | 5082830 | 5095178 | ZNF594 | | | | | |
| 17 | 5095378 | 5138931 | LOC100130950 | | | | | |
| 17 | 5112215 | 5138155 | SCIMP | | | | | |
| 17 | 5185557 | 5289659 | RABEP1 | | | RABEP1 | | |
| 17 | 5289345 | 5323059 | NUP88 | | | | | |
| 17 | 5322960 | 5336340 | RPAIN | | | | | |
| 17 | 5336098 | 5342471 | C1QBP | | | C1QBP | | |
| 17 | 5344231 | 5372380 | DHX33 | | | | | |
| 17 | 5374570 | 5389550 | DERL2 | | | | | |
| 17 | 5389693 | 5394130 | MIS12 | | | | | |
| 17 | 5402746 | 5404319 | LOC728392 | | | | | |
| 17 | 5404718 | 5487832 | NLRP1 | | NLRP1 | | | |
| 17 | 5675553 | 5834016 | LOC339166 | | | | | |
| 17 | 5973933 | 6027747 | WSCD1 | | | | | |
| 17 | 6327056 | 6338519 | AIPL1 | | | | | |
| 17 | 6347734 | 6354385 | FAM64A | | | | | |
| 17 | 6354582 | 6459877 | PITPNM3 | | | | | |
| 17 | 6481644 | 6544247 | KIAA0753 | | | | | |
| 17 | 6544221 | 6547861 | TXNDC17 | | | | | |
| 17 | 6546632 | 6554954 | MED31 | | | | | |
| 17 | 6555058 | 6556617 | C17orf100 | | | | | |
| 17 | 6558758 | 6558821 | MIR4520 | | | | | |
| 17 | 6561037 | 6564701 | ALOX15P1 | | | | | |
| 17 | 6588031 | 6616740 | SLC13A5 | | SLC13A5 | | | |
| 17 | 6659155 | 6678964 | XAF1 | | | | | |
| 17 | 6679551 | 6690965 | FBXO39 | | | FBXO39 | | |
| 17 | 6703299 | 6735060 | TEKT1 | | | TEKT1 | | |
| 17 | 6756894 | 6803668 | ALOX12P2 | | | | | |
| 17 | 6888441 | 6914055 | ALOX12 | | | | | |
| 17 | 6915735 | 6917852 | RNASEK | | | | | |
| 17 | 6915735 | 6920843 | RNASEK-C17orf49 | | | | | |
| 17 | 6918055 | 6920843 | C17orf49 | | | | | |
| 17 | 6919136 | 6922973 | MIR497HG | | | | | |
| 17 | 6920933 | 6921020 | MIR195 | | | | | |
| 17 | 6921229 | 6921341 | MIR497 | | | | | |
| 17 | 6926368 | 6932961 | BCL6B | | | | | |
| 17 | 6939393 | 6943440 | SLC16A13 | | | | | |
| 17 | 6944948 | 6947242 | SLC16A11 | | | | | |
| 17 | 6977855 | 6983600 | CLEC10A | | | | | |
| 17 | 7004640 | 7018130 | ASGR2 | | | | | |
| 17 | 7076750 | 7082883 | ASGR1 | | | | | |
| 17 | 7093209 | 7123369 | DLG4 | DLG4 | DLG4 | DLG4 | DLG4 | DLG4 |
| 17 | 7120443 | 7128586 | ACADVL | | | ACADVL | | |
| 17 | 7126615 | 7126698 | MIR324 | | | | | |
| 17 | 7128660 | 7137863 | DVL2 | | | | | |
| 17 | 7138346 | 7142825 | PHF23 | | | | | |
| 17 | 7143737 | 7145753 | GABARAP | | | | | |
| 17 | 7146905 | 7155259 | CTDNEP1 | | | | | |
| 17 | 7155371 | 7163259 | ELP5 | | | | | |
| 17 | 7163221 | 7166512 | CLDN7 | | | | | |
| 17 | 7185053 | 7191367 | SLC2A4 | | | | | |

| | | | | | | | | |
|---|---|---|---|---|---|---|---|---|
| 17 | 7191570 | 7197876 | YBX2 | | | | | |
| 17 | 7210317 | 7215782 | EIF5A | | | | | |
| 17 | 7215977 | 7218658 | GPS2 | | | | | |
| 17 | 7218950 | 7232638 | NEURL4 | | | | | |
| 17 | 7239847 | 7254793 | ACAP1 | | | | | |
| 17 | 7255207 | 7258262 | KCTD11 | | | | | |
| 17 | 7258496 | 7260538 | TMEM95 | | | | | |
| 17 | 7284292 | 7293092 | TNK1 | | | | | |
| 17 | 7293046 | 7298162 | PLSCR3 | | | | | |
| 17 | 7293046 | 7307450 | TMEM256-PLSCR3 | | | | | |
| 17 | 7306292 | 7307450 | TMEM256 | | | | | |
| 17 | 7311501 | 7323183 | NLGN2 | | | NLGN2 | | |
| 17 | 7323678 | 7324951 | SPEM1 | | | | | |
| 17 | 7328934 | 7330886 | C17orf74 | | | | | |
| 17 | 7338761 | 7340998 | TMEM102 | | | | | |
| 17 | 7341591 | 7348251 | FGF11 | | | | | |
| 17 | 7348405 | 7360932 | CHRNB1 | | | | CHRNB1 | CHRNB1 |
| 17 | 7362684 | 7387568 | ZBTB4 | | | | | |
| 17 | 7384720 | 7386383 | SLC35G6 | | | | | |
| 17 | 7387697 | 7417935 | POLR2A | | | | | |
| 17 | 7452374 | 7461207 | TNFSF12 | | | | | |
| 17 | 7452374 | 7464925 | TNFSF12-TNFSF13 | | | | | |
| 17 | 7461608 | 7464925 | TNFSF13 | | | | | |
| 17 | 7465308 | 7475287 | SENP3 | | | | | |
| 17 | 7465308 | 7482324 | SENP3-EIF4A1 | | | | | |
| 17 | 7476023 | 7482324 | EIF4A1 | | | | | |
| 17 | 7478030 | 7478165 | SNORA48 | | | | | |
| 17 | 7480128 | 7480276 | SNORD10 | | | | | |
| 17 | 7481272 | 7481409 | SNORA67 | | | | | |
| 17 | 7482804 | 7485429 | CD68 | | | | | |
| 17 | 7486964 | 7491527 | MPDU1 | | | | | |
| 17 | 7491497 | 7493488 | SOX15 | | | | | |
| 17 | 7494547 | 7518215 | FXR2 | | FXR2 | | | |
| 17 | 7517381 | 7536701 | SHBG | | | | | |
| 17 | 7529555 | 7531194 | SAT2 | | | | | |
| 17 | 7531286 | 7536701 | SHBG | | | | | |
| 17 | 7549944 | 7561089 | ATP1B2 | | | | | |
| 17 | 7571719 | 7590868 | TP53 | | | TP53 | | |
| 17 | 7589388 | 7606820 | WRAP53 | | | | | |
| 17 | 7608519 | 7614693 | EFNB3 | | | | | |
| 17 | 7620671 | 7647219 | DNAH2 | | | | | |
| 17 | 7657637 | 7658286 | RPL29P2 | | | | | |
| 17 | 7743234 | 7758118 | KDM6B | | | | | |
| 17 | 7758383 | 7759417 | TMEM88 | | | | | |
| 17 | 7760002 | 7761172 | NAA38 | | | | | |
| 17 | 7761063 | 7765600 | CYB5D1 | | | | | |
| 17 | 7788122 | 7816075 | CHD3 | | | | | |
| 17 | 7809440 | 7809578 | SCARNA21 | | | | | |
| 17 | 7816639 | 7819265 | LOC284023 | | | | | |
| 17 | 7825176 | 7832753 | KCNAB3 | | | | | |
| 17 | 7833662 | 7835317 | TRAPPC1 | | | TRAPPC1 | | |
| 17 | 7835441 | 7853237 | CNTROB | | | | | |
| 17 | 7905987 | 7923658 | GUCY2D | | | GUCY2D | | |
| 17 | 7942357 | 7952451 | ALOX15B | | | | | |
| 17 | 7975953 | 7991021 | ALOX12B | | | ALOX12B | | |
| 17 | 7991373 | 7991465 | MIR4314 | | | | | |
| 17 | 7999217 | 8022234 | ALOXE3 | | | | | |
| 17 | 8023907 | 8027410 | HES7 | | | | | |
| 17 | 8043787 | 8055753 | PER1 | | | | | |
| 17 | 8048311 | 8048389 | MIR6883 | | | | | |
| 17 | 8062464 | 8066293 | VAMP2 | | | | | |
| 17 | 8076296 | 8079714 | TMEM107 | | | | | |
| 17 | 8090262 | 8090322 | MIR4521 | | | | | |
| 17 | 8091650 | 8093564 | BORCS6 | | | | | |
| 17 | 8108048 | 8113944 | AURKB | | | | | |
| 17 | 8123947 | 8127361 | LINC00324 | | | | | |

| | | | | | | | | |
|---|---|---|---|---|---|---|---|---|
| 17 | 8128138 | 8151413 | CTC1 | | | | | |
| 17 | 8152595 | 8173809 | PFAS | | | | | |
| 17 | 8191081 | 8198170 | SLC25A35 | | | | | |
| 17 | 8191968 | 8193409 | RANGRF | | | | | |
| 17 | 8213555 | 8225834 | ARHGEF15 | | | ARHGEF15 | | |
| 17 | 8243157 | 8249363 | ODF4 | | | | | |
| 17 | 8261730 | 8263859 | LOC100128288 | | | | | |
| 17 | 8271970 | 8280029 | KRBA2 | | | | | |
| 17 | 8280833 | 8286565 | RPL26 | | | | | |
| 17 | 8294022 | 8301144 | RNF222 | | | | | |
| 17 | 8339169 | 8371495 | NDEL1 | | | | | |
| 17 | 8377522 | 8534079 | MYH10 | | | | | |
| 17 | 8633245 | 8648154 | CCDC42 | | | | | |
| 17 | 8656423 | 8661877 | SPDYE4 | | | | | |
| 17 | 8700427 | 8702667 | MFSD6L | | | | | |
| 17 | 8706040 | 8770994 | PIK3R6 | | | | | |
| 17 | 8782232 | 8869029 | PIK3R5 | | | | | |
| 17 | 8924858 | 9147317 | NTN1 | | | | | |
| 17 | 9074384 | 9082435 | LOC101928266 | | | | | |
| 17 | 9153787 | 9479275 | STX8 | | | STX8 | STX8 | |
| 17 | 9479943 | 9546776 | CFAP52 | | | | | |
| 17 | 9548853 | 9633003 | USP43 | | | | | |
| 17 | 9674750 | 9694614 | DHRS7C | | | | | |
| 17 | 9705702 | 9725388 | GSG1L2 | | | | | |
| 17 | 9728867 | 9795420 | GLP2R | | | | | |
| 17 | 9801026 | 9808684 | RCVRN | | | | | |
| 17 | 9813925 | 10101868 | GAS7 | | GAS7 | | | |
| 17 | 10204182 | 10276322 | MYH13 | | | MYH13 | | |
| 17 | 10286448 | 10528857 | MYHAS | | | | | |
| 17 | 10293641 | 10325267 | MYH8 | | | | | |
| 17 | 10346607 | 10372876 | MYH4 | | | | | |
| 17 | 10395626 | 10421859 | MYH1 | | | | | |
| 17 | 10424464 | 10453017 | MYH2 | | | | | |
| 17 | 10531842 | 10560626 | MYH3 | | | | | |
| 17 | 10583648 | 10600885 | SCO1 | | | | | |
| 17 | 10600926 | 10614875 | ADPRM | | | | | |
| 17 | 10616638 | 10633646 | TMEM220 | | | | | |
| 17 | 10619259 | 10619899 | MAGOH2P | | | | | |
| 17 | 10633093 | 10718481 | TMEM220 | | | | | |
| 17 | 10698229 | 10707416 | LINC00675 | | | | | |
| 17 | 10725791 | 10741418 | PIRT | | | | | |
| 17 | 11144739 | 11467380 | SHISA6 | | | | | |
| 17 | 11501747 | 11873065 | DNAH9 | | DNAH9 | DNAH9 | | |
| 17 | 11880755 | 11900827 | ZNF18 | | | | | |
| 17 | 11924134 | 12047148 | MAP2K4 | | MAP2K4 | | | |
| 17 | 11985215 | 11985313 | MIR744 | | | | | |
| 17 | 12453284 | 12540504 | LINC00670 | | | | | |
| 17 | 12569206 | 12672265 | MYOCD | | | | | |
| 17 | 12575178 | 12609452 | LOC101928418 | | | | | |
| 17 | 12663450 | 12693601 | LOC100128006 | | | | | |
| 17 | 12692828 | 12894960 | ARHGAP44 | | | | | |
| 17 | 12820584 | 12820659 | MIR1269B | | | | | |
| 17 | 12894928 | 12921381 | ELAC2 | | | | | |
| 17 | 13397348 | 13505259 | HS3ST3A1 | | | | | |
| 17 | 13927814 | 13928915 | CDRT15P1 | | | | | |
| 17 | 13932608 | 14111996 | COX10 | | | COX10 | | |
| 17 | 14139172 | 14140150 | CDRT15 | | | | | |
| 17 | 14204366 | 14252721 | HS3ST3B1 | | | | | |
| 17 | 14207056 | 14209062 | MGC12916 | | | | | |
| 17 | 14934291 | 14935274 | CDRT7 | | | | | |
| 17 | 15008301 | 15009504 | CDRT8 | | | | | |
| 17 | 15133093 | 15168674 | PMP22 | | | PMP22 | PMP22 | |
| 17 | 15154943 | 15155013 | MIR4731 | | | | | |
| 17 | 15207128 | 15244958 | TEKT3 | | | TEKT3 | | |
| 17 | 15339331 | 15370925 | CDRT4 | | | | | |
| 17 | 15339331 | 15466945 | TVP23C-CDRT4 | | | | | |

| | | | | | | | | |
|---|---|---|---|---|---|---|---|---|
| 17 | 15405577 | 15466945 | TVP23C | | | | | |
| 17 | 15468795 | 15523018 | CDRT1 | | | | | |
| 17 | 15531279 | 15586193 | TRIM16 | | | | | |
| 17 | 15602890 | 15624100 | ZNF286A | | | | | |
| 17 | 15635590 | 15648098 | TBC1D26 | | | | | |
| 17 | 15668016 | 15669003 | CDRT15P2 | | | | | |
| 17 | 15690163 | 15693019 | MEIS3P1 | | | | | |
| 17 | 15709555 | 15721056 | LOC101928567 | | | | | |
| 17 | 15848230 | 15879210 | ADORA2B | | | ADORA2B | | |
| 17 | 15879874 | 15903006 | ZSWIM7 | | | | | |
| 17 | 15902693 | 15932723 | TTC19 | | | | | |
| 17 | 15933407 | 16097953 | NCOR1 | | | | | |
| 17 | 16120508 | 16229573 | PIGL | | | | | |
| 17 | 16185327 | 16185402 | MIR1288 | | | | | |
| 17 | 16245847 | 16256812 | CENPV | | | | | |
| 17 | 16284106 | 16286059 | UBB | | | | | |
| 17 | 16318855 | 16340317 | TRPV2 | | | TRPV2 | | |
| 17 | 16342300 | 16373962 | LRRC75A | | | | | |
| 17 | 16342822 | 16342870 | SNORD49B | | | | | |
| 17 | 16343349 | 16343420 | SNORD49A | | | | | |
| 17 | 16344539 | 16344612 | SNORD65 | | | | | |
| 17 | 16345318 | 16395505 | LRRC75A | | | | | |
| 17 | 16453630 | 16472520 | ZNF287 | | | | | |
| 17 | 16524047 | 16557167 | ZNF624 | | | | | |
| 17 | 16593638 | 16678313 | CCDC144A | | | | | |
| 17 | 16690205 | 16707819 | USP32P1 | | | | | |
| 17 | 16692056 | 16693815 | FAM106CP | | | | | |
| 17 | 16733796 | 16736147 | KRT16P2 | | | | | |
| 17 | 16842397 | 16875402 | TNFRSF13B | | | | | |
| 17 | 16946073 | 17095962 | MPRIP | | | | | |
| 17 | 17104308 | 17109646 | PLD6 | | | | | |
| 17 | 17115526 | 17140502 | FLCN | | | FLCN | | |
| 17 | 17149937 | 17184617 | COPS3 | | | COPS3 | | |
| 17 | 17206679 | 17250977 | NT5M | | | NT5M | | |
| 17 | 17380299 | 17396534 | MED9 | | | | | |
| 17 | 17397752 | 17399709 | RASD1 | | | | | |
| 17 | 17408876 | 17495017 | PEMT | | | | | |
| 17 | 17577340 | 17581002 | SMCR2 | | | | | |
| 17 | 17584786 | 17669616 | RAI1 | | | RAI1 | | |
| 17 | 17679999 | 17682843 | SMCR5 | | | | | |
| 17 | 17714662 | 17740325 | SREBF1 | | | SREBF1 | | |
| 17 | 17716793 | 17716859 | MIR6777 | | | | | |
| 17 | 17717149 | 17717245 | MIR33B | | | | | |
| 17 | 17746821 | 17875784 | TOM1L2 | | | | | |
| 17 | 17876126 | 17920189 | DRC3 | | | | | |
| 17 | 17921333 | 17942480 | ATPAF2 | | | ATPAF2 | | |
| 17 | 17942610 | 17971718 | GID4 | | | | | |
| 17 | 17991180 | 18011299 | DRG2 | | | | | |
| 17 | 18012019 | 18083116 | MYO15A | | | | | |
| 17 | 18086866 | 18113267 | ALKBH5 | | | | | |
| 17 | 18128935 | 18148188 | LLGL1 | | | | | |
| 17 | 18148130 | 18162230 | FLII | | | | | |
| 17 | 18163847 | 18169095 | MIEF2 | | | | | |
| 17 | 18177234 | 18218321 | TOP3A | | | | | |
| 17 | 18218593 | 18231370 | SMCR8 | | | | | |
| 17 | 18231173 | 18266877 | SHMT1 | | | | | |
| 17 | 18244127 | 18244200 | MIR6778 | | | | | |
| 17 | 18281078 | 18292960 | EVPLL | | | | | |
| 17 | 18314472 | 18317702 | FLJ35934 | | | | | |
| 17 | 18325494 | 18328647 | KRT17P5 | | | | | |
| 17 | 18343221 | 18346208 | KRT16P1 | | | | | |
| 17 | 18380098 | 18398259 | LGALS9C | | | | | |
| 17 | 18414575 | 18424566 | USP32P2 | | | | | |
| 17 | 18427879 | 18430160 | FAM106A | | | | | |
| 17 | 18441114 | 18528930 | CCDC144B | | | | | |
| 17 | 18538841 | 18547740 | TBC1D28 | | | | | |

| | | | | | | | | |
|---|---|---|---|---|---|---|---|---|
| 17 | 18561741 | 18585572 | ZNF286B | | | | | |
| 17 | 18569235 | 18576494 | FOXO3B | | | | | |
| 17 | 18625401 | 18639431 | TRIM16L | | | | | |
| 17 | 18647325 | 18682662 | FBXW10 | | | | | |
| 17 | 18684307 | 18710026 | TVP23B | | | | | |
| 17 | 18759611 | 18834599 | PRPSAP2 | | | | | |
| 17 | 18853988 | 18924004 | SLC5A10 | | | | | |
| 17 | 18874380 | 18908060 | FAM83G | | | | | |
| 17 | 18923989 | 18950336 | GRAP | | | | | |
| 17 | 18996525 | 19000876 | LOC388436 | | | | | |
| 17 | 18996525 | 19000876 | LOC79999 | | | | | |
| 17 | 19030781 | 19062148 | GRAPL | | | | | |
| 17 | 19057836 | 19062185 | LOC388436 | | | | | |
| 17 | 19057836 | 19062185 | LOC79999 | | | | | |
| 17 | 19122694 | 19127043 | LOC388436 | | | | | |
| 17 | 19122694 | 19127043 | LOC79999 | | | | | |
| 17 | 19140689 | 19240028 | EPN2 | | | EPN2 | | |
| 17 | 19174705 | 19177701 | EPN2-IT1 | | | | | |
| 17 | 19199908 | 19209574 | EPN2 | | | EPN2 | | |
| 17 | 19240866 | 19266046 | B9D1 | | | | | |
| 17 | 19247818 | 19247887 | MIR1180 | | | | | |
| 17 | 19281033 | 19286857 | MAPK7 | | | MAPK7 | | |
| 17 | 19286754 | 19290532 | MFAP4 | | | | | |
| 17 | 19314490 | 19320589 | RNF112 | | | | | |
| 17 | 19437166 | 19482346 | SLC47A1 | | | | | |
| 17 | 19460872 | 19461024 | SNORA59A | | | | | |
| 17 | 19460872 | 19461024 | SNORA59B | | | | | |
| 17 | 19552063 | 19580908 | ALDH3A2 | | | | | |
| 17 | 19581627 | 19620043 | SLC47A2 | | | | | |
| 17 | 19641297 | 19651746 | ALDH3A1 | | | | | |
| 17 | 19674142 | 19771239 | ULK2 | | ULK2 | | | |
| 17 | 19807764 | 19881169 | AKAP10 | | | AKAP10 | | |
| 17 | 19912648 | 20218072 | SPECC1 | | | | | |
| 17 | 20224486 | 20305504 | CCDC144CP | | | | | |
| 17 | 20320604 | 20322363 | FAM106B | | | | | |
| 17 | 20353175 | 20370848 | LGALS9B | | | | | |
| 17 | 20404826 | 20407811 | KRT16P3 | | | | | |
| 17 | 20483036 | 20484224 | CDRT15L2 | | | | | |
| 17 | 20615758 | 20641178 | LOC100287072 | | | | | |
| 17 | 20766707 | 20808543 | CCDC144NL | | | | | |
| 17 | 20841832 | 20885670 | LOC339260 | | | | | |
| 17 | 20902905 | 20946352 | USP22 | | | | | |
| 17 | 20978868 | 20993926 | LINC01563 | | | | | |
| 17 | 21030240 | 21095285 | DHRS7B | | | | | |
| 17 | 21101262 | 21117908 | TMEM11 | | | | | |
| 17 | 21142183 | 21156578 | NATD1 | | | | | |
| 17 | 21187967 | 21218551 | MAP2K3 | | | | | |
| 17 | 21279698 | 21323179 | KCNJ12 | | | KCNJ12 | KCNJ12 | |
| 17 | 21308447 | 21320482 | KCNJ18 | | | | | |
| 17 | 21431570 | 21454941 | C17orf51 | | | | | |
| 17 | 21825369 | 21826499 | FAM27E5 | | | | | |
| 17 | 21904061 | 21913070 | FLJ36000 | | | | | |
| 17 | 22022436 | 22023991 | MTRNR2L1 | | | | | |
| 17 | 25620935 | 25621022 | MIR4522 | | | | | |
| 17 | 25621105 | 25640645 | WSB1 | | | | | |
| 17 | 25745030 | 25758649 | TBC1D3P5 | | | | | |
| 17 | 25799035 | 25950718 | KSR1 | | | KSR1 | | |
| 17 | 25958173 | 25976586 | LGALS9 | | | | | |
| 17 | 26083791 | 26127555 | NOS2 | | NOS2 | | | |
| 17 | 26205339 | 26220409 | LYRM9 | | | | | |
| 17 | 26369687 | 26523404 | NLK | | | NLK | | |
| 17 | 26553588 | 26555085 | PYY2 | | | | | |
| 17 | 26574469 | 26575315 | PPY2P | | | | | |
| 17 | 26603011 | 26634408 | KRT18P55 | | | | | |
| 17 | 26646120 | 26655711 | TMEM97 | | | | | |
| 17 | 26655350 | 26662515 | IFT20 | | | | | |

| | | | | | | | | |
|---|---|---|---|---|---|---|---|---|
| 17 | 26662547 | 26674035 | TNFAIP1 | | | | | |
| 17 | 26673653 | 26684612 | POLDIP2 | | | | | |
| 17 | 26684603 | 26690705 | TMEM199 | | | | | |
| 17 | 26687676 | 26687757 | MIR4723 | | | | | |
| 17 | 26691289 | 26692173 | SEBOX | | | | | |
| 17 | 26694298 | 26697373 | VTN | | | | | |
| 17 | 26698682 | 26728055 | SARM1 | | | | | |
| 17 | 26721660 | 26733230 | SLC46A1 | | | | | |
| 17 | 26800663 | 26824798 | SLC13A2 | | | | | |
| 17 | 26850958 | 26865175 | FOXN1 | | | FOXN1 | | |
| 17 | 26873724 | 26879646 | UNC119 | | | UNC119 | UNC119 | |
| 17 | 26880405 | 26898887 | PIGS | | | | | |
| 17 | 26900132 | 26903951 | ALDOC | | | | | |
| 17 | 26904582 | 26944395 | SPAG5 | | | SPAG5 | | |
| 17 | 26934981 | 26941211 | SGK494 | | | | | |
| 17 | 26941457 | 26972177 | KIAA0100 | | | | | |
| 17 | 26975373 | 26989207 | SDF2 | | | | | |
| 17 | 26989301 | 27029249 | SUPT6H | | | | | |
| 17 | 27030214 | 27038925 | PROCA1 | | | | | |
| 17 | 27041298 | 27045286 | RAB34 | | | | | |
| 17 | 27043010 | 27044908 | NARR | | | | | |
| 17 | 27046999 | 27051374 | RPL23A | | | | | |
| 17 | 27047567 | 27047634 | SNORD42B | | | | | |
| 17 | 27049599 | 27049671 | SNORD4A | | | | | |
| 17 | 27050446 | 27050509 | SNORD42A | | | | | |
| 17 | 27050698 | 27050772 | SNORD4B | | | | | |
| 17 | 27051365 | 27053949 | TLCD1 | | | | | |
| 17 | 27055831 | 27069784 | NEK8 | | | | | |
| 17 | 27071022 | 27077976 | TRAF4 | | | | | |
| 17 | 27082995 | 27169857 | FAM222B | | | | | |
| 17 | 27181974 | 27188085 | ERAL1 | | | | | |
| 17 | 27188386 | 27188458 | MIR451A | | | | | |
| 17 | 27188388 | 27188456 | MIR451B | | | | | |
| 17 | 27188550 | 27188636 | MIR144 | | | | | |
| 17 | 27188672 | 27188748 | MIR4732 | | | | | |
| 17 | 27206356 | 27224715 | FLOT2 | | | | | |
| 17 | 27224798 | 27230089 | DHRS13 | | | | | |
| 17 | 27232270 | 27278508 | PHF12 | | | | | |
| 17 | 27253292 | 27271766 | LOC101927018 | | | | | |
| 17 | 27281946 | 27333458 | SEZ6 | | | | | |
| 17 | 27369917 | 27384236 | PIPOX | | | | | |
| 17 | 27400527 | 27507407 | MYO18A | | | | | |
| 17 | 27400538 | 27402627 | TIAF1 | | | | | |
| 17 | 27573874 | 27581502 | CRYBA1 | | | | | |
| 17 | 27582853 | 27621166 | NUFIP2 | | | | | |
| 17 | 27717679 | 27717748 | MIR4523 | | | | | |
| 17 | 27717942 | 27878921 | TAOK1 | | | | | |
| 17 | 27887688 | 27894042 | ABHD15 | | | | | |
| 17 | 27895738 | 27900175 | TP53I13 | | | | | |
| 17 | 27900486 | 27916610 | GIT1 | | | GIT1 | | |
| 17 | 27920526 | 27941779 | ANKRD13B | | | | | |
| 17 | 27941773 | 27948441 | CORO6 | | | | | |
| 17 | 27952956 | 28257246 | SSH2 | | | | | |
| 17 | 28256873 | 28435470 | EFCAB5 | | | | | |
| 17 | 28443824 | 28513493 | NSRP1 | | | | | |
| 17 | 28444096 | 28444190 | MIR423 | | | | | |
| 17 | 28444103 | 28444178 | MIR3184 | | | | | |
| 17 | 28521336 | 28562986 | SLC6A4 | | | SLC6A4 | SLC6A4 | |
| 17 | 28575212 | 28619184 | BLMH | | | | | |
| 17 | 28643365 | 28661077 | TMIGD1 | | | | | |
| 17 | 28705941 | 28796675 | CPD | | | | | |
| 17 | 28804425 | 28853832 | GOSR1 | | | GOSR1 | | |
| 17 | 28886583 | 28890509 | TBC1D29 | | | | | |
| 17 | 28903482 | 28964484 | LOC107133515 | | | | | |
| 17 | 28951335 | 28953825 | SH3GL1P2 | | | | | |
| 17 | 29036625 | 29097068 | SUZ12P1 | | | | | |

| | | | | | | | | |
|---|---|---|---|---|---|---|---|---|
| 17 | 29109701 | 29151778 | CRLF3 | | | | | |
| 17 | 29158987 | 29222883 | ATAD5 | | | | | |
| 17 | 29226000 | 29233286 | TEFM | | | | | |
| 17 | 29248753 | 29286211 | ADAP2 | | | | | |
| 17 | 29297955 | 29326929 | RNF135 | | | | | |
| 17 | 29302352 | 29303017 | DPRXP4 | | | | | |
| 17 | 29421367 | 29421443 | MIR4733 | | | | | |
| 17 | 29421944 | 29704695 | NF1 | NF1 | | NF1 | | |
| 17 | 29621667 | 29624380 | OMG | | | | | |
| 17 | 29630787 | 29641130 | EVI2B | | | | | |
| 17 | 29643427 | 29648767 | EVI2A | | | | | |
| 17 | 29718641 | 29865236 | RAB11FIP4 | | | RAB11FIP4 | | |
| 17 | 29861900 | 29861989 | MIR4724 | | | | | |
| 17 | 29887014 | 29887102 | MIR193A | | | | | |
| 17 | 29902287 | 29902377 | MIR4725 | | | | | |
| 17 | 29902429 | 29902540 | MIR365B | | | | | |
| 17 | 30178883 | 30186326 | COPRS | | | | | |
| 17 | 30190189 | 30228729 | UTP6 | | | | | |
| 17 | 30264043 | 30328057 | SUZ12 | | | | | |
| 17 | 30348154 | 30380519 | LRRC37B | | | | | |
| 17 | 30367354 | 30369851 | SH3GL1P1 | | | | | |
| 17 | 30469472 | 30552790 | RHOT1 | | | | | |
| 17 | 30477386 | 30478590 | ARGFXP2 | | | | | |
| 17 | 30593194 | 30651680 | RHBDL3 | | | | | |
| 17 | 30655459 | 30669228 | C17orf75 | | | | | |
| 17 | 30677127 | 30677221 | MIR632 | | | | | |
| 17 | 30677156 | 30697468 | ZNF207 | | | | | |
| 17 | 30771480 | 30810337 | PSMD11 | | | | | |
| 17 | 30814104 | 30818271 | CDK5R1 | | | | | |
| 17 | 30819539 | 31204191 | MYO1D | | | | | |
| 17 | 31254927 | 31268667 | TMEM98 | | | | | |
| 17 | 31318881 | 31324895 | SPACA3 | | | | | |
| 17 | 31340105 | 32483825 | ASIC2 | | ASIC2 | | | |
| 17 | 31856805 | 31860779 | AA06 | | | | | |
| 17 | 32496394 | 32510651 | LOC101927239 | | | | | |
| 17 | 32582295 | 32584220 | CCL2 | | | | | |
| 17 | 32597234 | 32599261 | CCL7 | | | | | |
| 17 | 32612686 | 32615199 | CCL11 | | | | | |
| 17 | 32646065 | 32648421 | CCL8 | | | | | |
| 17 | 32683470 | 32685629 | CCL13 | | | | | |
| 17 | 32687346 | 32690252 | CCL1 | | | | | |
| 17 | 32901141 | 32906388 | C17orf102 | | | | | |
| 17 | 32907767 | 32966337 | TMEM132E | | | | | |
| 17 | 33254877 | 33288528 | CCT6B | | | | | |
| 17 | 33288548 | 33290205 | ZNF830 | | | | | |
| 17 | 33307516 | 33332088 | LIG3 | | | | | |
| 17 | 33336130 | 33416348 | RFFL | | | | | |
| 17 | 33336130 | 33448541 | RAD51L3-RFFL | | | | | |
| 17 | 33426810 | 33446888 | RAD51D | | | | | |
| 17 | 33448630 | 33457751 | FNDC8 | | | | | |
| 17 | 33458340 | 33469334 | NLE1 | | | | | |
| 17 | 33474835 | 33516364 | UNC45B | | | | | |
| 17 | 33519538 | 33521412 | SLC35G3 | | | | | |
| 17 | 33570085 | 33594761 | SLFN5 | | | | | |
| 17 | 33677328 | 33700720 | SLFN11 | | | | | |
| 17 | 33737940 | 33760195 | SLFN12 | | | | | |
| 17 | 33762114 | 33775856 | SLFN13 | | | | | |
| 17 | 33801941 | 33814758 | SLFN12L | | | | | |
| 17 | 33875143 | 33885110 | SLFN14 | | | | | |
| 17 | 33900675 | 33900772 | SNORD7 | | | | | |
| 17 | 33901813 | 33905656 | PEX12 | | | | | |
| 17 | 33914281 | 34053436 | AP2B1 | | | AP2B1 | | |
| 17 | 34058678 | 34070540 | RASL10B | | | | | |
| 17 | 34071529 | 34079897 | GAS2L2 | | | | | |
| 17 | 34083267 | 34122711 | MMP28 | | | | | |
| 17 | 34087915 | 34092098 | C17orf50 | | | | | |

| | | | | | | | | |
|---|---|---|---|---|---|---|---|---|
| 17 | 34092875 | 34122711 | MMP28 | | | | | |
| 17 | 34136458 | 34174246 | TAF15 | | | | TAF15 | |
| 17 | 34181959 | 34195895 | HEATR9 | | | | | |
| 17 | 34198495 | 34207377 | CCL5 | | | | | |
| 17 | 34231223 | 34238027 | LRRC37A8P | | | | | |
| 17 | 34245084 | 34257780 | RDM1 | | | | | |
| 17 | 34261521 | 34270714 | LYZL6 | | | | | |
| 17 | 34303528 | 34308532 | CCL16 | | | | | |
| 17 | 34310691 | 34313764 | CCL14 | | | | | |
| 17 | 34310691 | 34329084 | CCL15-CCL14 | | | | | |
| 17 | 34324617 | 34329084 | CCL15 | | | | | |
| 17 | 34340095 | 34345005 | CCL23 | | | | | |
| 17 | 34391631 | 34398841 | CCL18 | | | | | |
| 17 | 34415602 | 34417506 | CCL3 | | | | | |
| 17 | 34431219 | 34433014 | CCL4 | | | | | |
| 17 | 34522267 | 34524147 | CCL3L3 | | | | | |
| 17 | 34522268 | 34524157 | CCL3L1 | | | | | |
| 17 | 34538309 | 34540280 | CCL4L1 | | | | | |
| 17 | 34538464 | 34540280 | CCL4L2 | | | | | |
| 17 | 34581028 | 34591996 | TBC1D3H | | | | | |
| 17 | 34581084 | 34591982 | TBC1D3G | | | | | |
| 17 | 34581084 | 34591996 | TBC1D3F | | | | | |
| 17 | 34623841 | 34625721 | CCL3L3 | | | | | |
| 17 | 34623841 | 34625731 | CCL3L1 | | | | | |
| 17 | 34639875 | 34641846 | CCL4L1 | | | | | |
| 17 | 34640030 | 34641846 | CCL4L2 | | | | | |
| 17 | 34746062 | 34757007 | TBC1D3H | | | | | |
| 17 | 34746118 | 34756993 | TBC1D3G | | | | | |
| 17 | 34746118 | 34808060 | TBC1D3F | | | | | |
| 17 | 34797171 | 34808092 | TBC1D3B | | | | | |
| 17 | 34842470 | 34855154 | ZNHIT3 | | | | | |
| 17 | 34851598 | 34891305 | MYO19 | | | | | |
| 17 | 34891402 | 34895150 | PIGW | | | | | |
| 17 | 34900736 | 34946276 | GGNBP2 | | | | | |
| 17 | 34948225 | 34957233 | DHRS11 | | | | | |
| 17 | 34958024 | 34965407 | MRM1 | | | | | |
| 17 | 35294771 | 35301915 | LHX1 | | | | | |
| 17 | 35306174 | 35414171 | AATF | | | | | |
| 17 | 35391041 | 35391110 | MIR2909 | | | | | |
| 17 | 35441926 | 35766902 | ACACA | | ACACA | ACACA | | |
| 17 | 35601716 | 35601891 | SNORA90 | | | | | |
| 17 | 35732984 | 35749662 | C17orf78 | | | | | |
| 17 | 35766976 | 35837226 | TADA2A | | | | | |
| 17 | 35849950 | 35873588 | DUSP14 | | | | | |
| 17 | 35874899 | 35969486 | SYNRG | | | | | |
| 17 | 35969789 | 36003493 | DDX52 | | | | | |
| 17 | 35974975 | 35975084 | MIR378J | | | | | |
| 17 | 36046433 | 36105069 | HNF1B | | | HNF1B | | |
| 17 | 36202572 | 36244363 | YWHAEP7 | | | | | |
| 17 | 36283976 | 36294915 | TBC1D3C | | | | | |
| 17 | 36284002 | 36294915 | TBC1D3F | | | | | |
| 17 | 36284002 | 36294915 | TBC1D3L | | | | | |
| 17 | 36284002 | 36294971 | TBC1D3H | | | | | |
| 17 | 36284004 | 36295098 | TBC1D3 | | | | | |
| 17 | 36284016 | 36294915 | LOC101060389 | | | | | |
| 17 | 36284016 | 36294915 | TBC1D3G | | | | | |
| 17 | 36284016 | 36294915 | TBC1D3I | | | | | |
| 17 | 36284016 | 36294915 | TBC1D3K | | | | | |
| 17 | 36284018 | 36294915 | TBC1D3E | | | | | |
| 17 | 36337527 | 36348621 | TBC1D3 | | | | | |
| 17 | 36337654 | 36348623 | TBC1D3H | | | | | |
| 17 | 36337710 | 36348607 | TBC1D3E | | | | | |
| 17 | 36337710 | 36348609 | LOC101060389 | | | | | |
| 17 | 36337710 | 36348609 | TBC1D3G | | | | | |
| 17 | 36337710 | 36348609 | TBC1D3I | | | | | |
| 17 | 36337710 | 36348609 | TBC1D3K | | | | | |

| | | | | | | | | |
|---|---|---|---|---|---|---|---|---|
| 17 | 36337710 | 36348623 | TBC1D3F | | | | | |
| 17 | 36337710 | 36348623 | TBC1D3L | | | | | |
| 17 | 36337710 | 36348649 | TBC1D3C | | | | | |
| 17 | 36351795 | 36413256 | LOC440434 | | | | | |
| 17 | 36452988 | 36479101 | MRPL45 | | | | | |
| 17 | 36481492 | 36499693 | GPR179 | | | | | |
| 17 | 36508006 | 36561846 | SOCS7 | | | | | |
| 17 | 36584719 | 36668628 | ARHGAP23 | | | | | |
| 17 | 36686258 | 36762183 | SRCIN1 | | | | | |
| 17 | 36827958 | 36831187 | C17orf96 | | | | | |
| 17 | 36858514 | 36858584 | MIR4734 | | | | | |
| 17 | 36861872 | 36886056 | MLLT6 | | | | | |
| 17 | 36875943 | 36876001 | MIR4726 | | | | | |
| 17 | 36886509 | 36891858 | CISD3 | | | | | |
| 17 | 36890149 | 36904558 | PCGF2 | | | | | |
| 17 | 36908965 | 36920484 | PSMB3 | | | | | |
| 17 | 36921943 | 36956158 | PIP4K2B | | | | | |
| 17 | 36956686 | 36981603 | CWC25 | | | | | |
| 17 | 36982090 | 36982145 | MIR4727 | | | | | |
| 17 | 36991340 | 36997642 | C17orf98 | | | | | |
| 17 | 37006320 | 37010053 | RPL23 | | | | | |
| 17 | 37009115 | 37009248 | SNORA21 | | | | | |
| 17 | 37026111 | 37078023 | LASP1 | | | | | |
| 17 | 37071231 | 37071295 | MIR6779 | | | | | |
| 17 | 37081420 | 37085637 | LINC00672 | | | | | |
| 17 | 37092684 | 37123655 | FBXO47 | | | | | |
| 17 | 37186158 | 37209458 | LRRC37A11P | | | | | |
| 17 | 37213271 | 37237704 | LOC100131347 | | | | | |
| 17 | 37219555 | 37307902 | PLXDC1 | | | | | |
| 17 | 37313146 | 37322414 | ARL5C | | | | | |
| 17 | 37329708 | 37353956 | CACNB1 | | | CACNB1 | | |
| 17 | 37356535 | 37360980 | RPL19 | | | | | |
| 17 | 37366788 | 37382040 | STAC2 | | | | | |
| 17 | 37408896 | 37557909 | FBXL20 | | | | | |
| 17 | 37560537 | 37607527 | MED1 | | | | | |
| 17 | 37617763 | 37690818 | CDK12 | | | | | |
| 17 | 37760020 | 37764175 | NEUROD2 | | | | | |
| 17 | 37783176 | 37792878 | PPP1R1B | | PPP1R1B | PPP1R1B | PPP1R1B | |
| 17 | 37793332 | 37820454 | STARD3 | | | | | |
| 17 | 37821598 | 37822807 | TCAP | | | | | |
| 17 | 37824233 | 37826728 | PNMT | | | | | |
| 17 | 37827374 | 37844323 | PGAP3 | | | | | |
| 17 | 37844336 | 37884915 | ERBB2 | | | | | |
| 17 | 37882747 | 37882814 | MIR4728 | | | | | |
| 17 | 37885408 | 37886788 | MIEN1 | | | | | |
| 17 | 37894161 | 37903538 | GRB7 | | | | | |
| 17 | 37913967 | 38020441 | IKZF3 | | | | | |
| 17 | 38024454 | 38034149 | ZPBP2 | | | | | |
| 17 | 38060847 | 38074903 | GSDMB | | | | | |
| 17 | 38077295 | 38083884 | ORMDL3 | | | | | |
| 17 | 38097726 | 38100987 | LRRC3C | | | | | |
| 17 | 38119225 | 38134019 | GSDMA | | | | | |
| 17 | 38137020 | 38154213 | PSMD3 | | | | | |
| 17 | 38171613 | 38174066 | CSF3 | | | | | |
| 17 | 38175349 | 38210889 | MED24 | | | | | |
| 17 | 38182584 | 38182662 | MIR6884 | | | | | |
| 17 | 38183795 | 38183898 | SNORD124 | | | | | |
| 17 | 38218445 | 38250120 | THRA | | THRA | | | |
| 17 | 38249036 | 38256978 | NR1D1 | | | | | |
| 17 | 38278789 | 38293045 | MSL1 | | | | | |
| 17 | 38296506 | 38328431 | CASC3 | | | | | |
| 17 | 38318185 | 38318254 | MIR6866 | | | | | |
| 17 | 38333262 | 38351908 | RAPGEFL1 | | | | | |
| 17 | 38349848 | 38349915 | MIR6867 | | | | | |
| 17 | 38375573 | 38438439 | WIPF2 | | | | | |
| 17 | 38444145 | 38459413 | CDC6 | | | | | |

| | | | | | | | | | |
|---|---|---|---|---|---|---|---|---|---|
| 17 | 38465422 | 38513895 | RARA | | | | | | |
| 17 | 38516904 | 38520945 | GJD3 | | | | | | |
| 17 | 38544772 | 38574202 | TOP2A | | | | | | |
| 17 | 38599675 | 38613982 | IGFBP4 | | | | | | |
| 17 | 38632079 | 38657854 | TNS4 | | | | | | |
| 17 | 38710021 | 38721736 | CCR7 | | | | | | |
| 17 | 38783975 | 38804103 | SMARCE1 | | | | | | |
| 17 | 38811871 | 38821416 | KRT222 | | | | | | |
| 17 | 38854242 | 38860002 | KRT24 | | | | | | |
| 17 | 38904272 | 38911584 | KRT25 | | | | | | |
| 17 | 38922489 | 38928411 | KRT26 | | | | | | |
| 17 | 38933059 | 38938786 | KRT27 | | | | | | |
| 17 | 38948447 | 38956211 | KRT28 | | | | | | |
| 17 | 38974368 | 38978863 | KRT10 | | | | | | |
| 17 | 38975343 | 38992526 | TMEM99 | | | | | | |
| 17 | 39017429 | 39023462 | KRT12 | | | | | | |
| 17 | 39032140 | 39041495 | KRT20 | | | | | | |
| 17 | 39078947 | 39093895 | KRT23 | | | | | | |
| 17 | 39114668 | 39123144 | KRT39 | | | | | | |
| 17 | 39133967 | 39143387 | KRT40 | | | | | | |
| 17 | 39149681 | 39150385 | KRTAP3-3 | | | | | | |
| 17 | 39155444 | 39156138 | KRTAP3-2 | | | | | | |
| 17 | 39164773 | 39165366 | KRTAP3-1 | | | | | | |
| 17 | 39182278 | 39183454 | KRTAP1-5 | | | | | | |
| 17 | 39185928 | 39186377 | KRTAP1-4 | | | | | | |
| 17 | 39190136 | 39191107 | KRTAP1-3 | | | | | | |
| 17 | 39196810 | 39197713 | KRTAP1-1 | | | | | | |
| 17 | 39202795 | 39203568 | KRTAP2-1 | | | | | | |
| 17 | 39210750 | 39211463 | KRTAP2-2 | | | | | | |
| 17 | 39215492 | 39216344 | KRTAP2-3 | | | | | | |
| 17 | 39221367 | 39222131 | KRTAP2-4 | | | | | | |
| 17 | 39240458 | 39241396 | KRTAP4-7 | | | | | | |
| 17 | 39253233 | 39254375 | KRTAP4-8 | | | | | | |
| 17 | 39261640 | 39262740 | KRTAP4-9 | | | | | | |
| 17 | 39273433 | 39274606 | KRTAP4-11 | | | | | | |
| 17 | 39279344 | 39280419 | KRTAP4-12 | | | | | | |
| 17 | 39295684 | 39296739 | KRTAP4-6 | | | | | | |
| 17 | 39305175 | 39306054 | KRTAP4-5 | | | | | | |
| 17 | 39315905 | 39316983 | KRTAP4-4 | | | | | | |
| 17 | 39323482 | 39324424 | KRTAP4-3 | | | | | | |
| 17 | 39333697 | 39334460 | KRTAP4-2 | | | | | | |
| 17 | 39340351 | 39341147 | KRTAP4-1 | | | | | | |
| 17 | 39346138 | 39346891 | KRTAP9-1 | | | | | | |
| 17 | 39382899 | 39383904 | KRTAP9-2 | | | | | | |
| 17 | 39388714 | 39389706 | KRTAP9-3 | | | | | | |
| 17 | 39388751 | 39412616 | KRTAP9-9 | | | | | | |
| 17 | 39394269 | 39395256 | KRTAP9-8 | | | | | | |
| 17 | 39405938 | 39406905 | KRTAP9-4 | | | | | | |
| 17 | 39411635 | 39412616 | KRTAP9-9 | | | | | | |
| 17 | 39421629 | 39422112 | KRTAP9-6 | | | | | | |
| 17 | 39431949 | 39432459 | KRTAP9-7 | | | | | | |
| 17 | 39458077 | 39459103 | KRTAP29-1 | | | | | | |
| 17 | 39463951 | 39465505 | KRTAP16-1 | | | | | | |
| 17 | 39471168 | 39471947 | KRTAP17-1 | | | | | | |
| 17 | 39502344 | 39507064 | KRT33A | | | | | | |
| 17 | 39519745 | 39526052 | KRT33B | | | | | | |
| 17 | 39533920 | 39538636 | KRT34 | | | | | | |
| 17 | 39549976 | 39553844 | KRT31 | | | | | | |
| 17 | 39558667 | 39568840 | LOC100505782 | | | | | | |
| 17 | 39576808 | 39580822 | KRT37 | | | | | | |
| 17 | 39592620 | 39597596 | KRT38 | | | | | | |
| 17 | 39615764 | 39623638 | KRT32 | | | | | | |
| 17 | 39632940 | 39637392 | KRT35 | | | | | | |
| 17 | 39642387 | 39646116 | KRT36 | | | | | | |
| 17 | 39657232 | 39661865 | KRT13 | | | | | | |
| 17 | 39669996 | 39675270 | KRT15 | | | | | | |

| | | | | | | | | |
|---|---|---|---|---|---|---|---|---|
| 17 | 39673415 | 39673469 | MIR6510 | | | | | |
| 17 | 39679868 | 39684641 | KRT19 | | | | | |
| 17 | 39705857 | 39710747 | LINC00974 | | | | | |
| 17 | 39722093 | 39728310 | KRT9 | | | | | |
| 17 | 39738530 | 39743147 | KRT14 | | | KRT14 | | |
| 17 | 39766030 | 39769079 | KRT16 | | | | | |
| 17 | 39775691 | 39780882 | KRT17 | | | | | |
| 17 | 39782578 | 39796451 | KRT42P | | | | | |
| 17 | 39845126 | 39847898 | EIF1 | | | | | |
| 17 | 39868577 | 39872221 | GAST | | | | | |
| 17 | 39878890 | 39890898 | HAP1 | | | | | |
| 17 | 39910858 | 39942964 | JUP | | | | | |
| 17 | 39958204 | 39968451 | P3H4 | | | | | |
| 17 | 39968961 | 39979469 | FKBP10 | | | | | |
| 17 | 39981333 | 39992523 | NT5C3B | | | | | |
| 17 | 39994042 | 40004599 | KLHL10 | | | | | |
| 17 | 40004770 | 40021684 | KLHL11 | | | | | |
| 17 | 40023169 | 40075272 | ACLY | | | | | |
| 17 | 40086875 | 40117668 | TTC25 | | | | | |
| 17 | 40118758 | 40129754 | CNP | | | | | |
| 17 | 40128438 | 40169715 | DNAJC7 | | | | | |
| 17 | 40169593 | 40177656 | NKIRAS2 | | | | | |
| 17 | 40177593 | 40190044 | ZNF385C | | | | | |
| 17 | 40253421 | 40264751 | DHX58 | | | | | |
| 17 | 40265128 | 40273382 | KAT2A | | | | | |
| 17 | 40274755 | 40275371 | HSPB9 | | | | | |
| 17 | 40276993 | 40307062 | RAB5C | | | RAB5C | | |
| 17 | 40308909 | 40333296 | KCNH4 | | | | | |
| 17 | 40336077 | 40337470 | HCRT | | | | HCRT | |
| 17 | 40341104 | 40346550 | GHDC | | | | | |
| 17 | 40351194 | 40428424 | STAT5B | | | | | |
| 17 | 40439564 | 40463960 | STAT5A | | | STAT5A | | |
| 17 | 40465342 | 40540513 | STAT3 | | | | | |
| 17 | 40554466 | 40575338 | PTRF | | | | | |
| 17 | 40610861 | 40674597 | ATP6V0A1 | | ATP6V0A1 | ATP6V0A1 | | |
| 17 | 40646790 | 40646848 | MIR548AT | | | | | |
| 17 | 40666205 | 40666325 | MIR5010 | | | | | |
| 17 | 40687950 | 40696466 | NAGLU | | | | | |
| 17 | 40703983 | 40707232 | HSD17B1 | | | | | |
| 17 | 40714091 | 40718299 | COASY | | | | | |
| 17 | 40719077 | 40725221 | MLX | | | | | |
| 17 | 40724327 | 40729849 | PSMC3IP | | | | | |
| 17 | 40731525 | 40761445 | FAM134C | | | | | |
| 17 | 40761357 | 40767256 | TUBG1 | | | | | |
| 17 | 40811265 | 40819024 | TUBG2 | | | | | |
| 17 | 40819931 | 40829048 | PLEKHH3 | | | | | |
| 17 | 40831419 | 40833845 | CCR10 | | | | | |
| 17 | 40834631 | 40852011 | CNTNAP1 | | CNTNAP1 | | CNTNAP1 | |
| 17 | 40852292 | 40897071 | EZH1 | | | EZH1 | | |
| 17 | 40860101 | 40860169 | MIR6780A | | | | | |
| 17 | 40905946 | 40915060 | RAMP2 | | | | | |
| 17 | 40925453 | 40931618 | VPS25 | | | | | |
| 17 | 40932648 | 40949084 | WNK4 | | | | | |
| 17 | 40949635 | 40950743 | COA3 | | | | | |
| 17 | 40950853 | 40963605 | CNTD1 | | | | | |
| 17 | 40962149 | 40976333 | BECN1 | | | BECN1 | | |
| 17 | 40975897 | 40975961 | MIR6781 | | | | | |
| 17 | 40985158 | 40995777 | PSME3 | | | | | |
| 17 | 40996608 | 41002724 | AOC2 | | | AOC2 | | |
| 17 | 41003200 | 41010147 | AOC3 | | | | | |
| 17 | 41019161 | 41021234 | AOC4P | | | | | |
| 17 | 41026690 | 41050751 | LINC00671 | | | | | |
| 17 | 41052813 | 41066450 | G6PC | | | G6PC | | |
| 17 | 41102542 | 41116515 | AARSD1 | | | | | |
| 17 | 41102542 | 41132545 | PTGES3L-AARSD1 | | | | | |
| 17 | 41120104 | 41132545 | PTGES3L | | | | | |

| | | | | | | | | |
|---|---|---|---|---|---|---|---|---|
| 17 | 41132581 | 41145707 | RUNDC1 | | | | | |
| 17 | 41150445 | 41154971 | RPL27 | | | | | |
| 17 | 41158741 | 41166476 | IFI35 | | | | | |
| 17 | 41166621 | 41174459 | VAT1 | | | | | |
| 17 | 41177257 | 41184058 | RND2 | | | | | |
| 17 | 41196311 | 41277500 | BRCA1 | | | | | |
| 17 | 41277599 | 41297125 | NBR2 | | | | | |
| 17 | 41300383 | 41322420 | LOC101929767 | | | | | |
| 17 | 41322487 | 41363708 | NBR1 | | | | | |
| 17 | 41363845 | 41372057 | TMEM106A | | | | | |
| 17 | 41373436 | 41381062 | LINC00854 | | | | | |
| 17 | 41447212 | 41466266 | LINC00910 | | | | | |
| 17 | 41476352 | 41478504 | ARL4D | | | | | |
| 17 | 41522173 | 41522253 | MIR2117 | | | | | |
| 17 | 41561284 | 41604164 | DHX8 | | | | | |
| 17 | 41605210 | 41623800 | ETV4 | | | | | |
| 17 | 41717757 | 41739262 | MEOX1 | | | | | |
| 17 | 41831098 | 41836156 | SOST | | | | | |
| 17 | 41843488 | 41856368 | DUSP3 | | | | | |
| 17 | 41857802 | 41862054 | C17orf105 | | | | | |
| 17 | 41878166 | 41910547 | MPP3 | | | | | |
| 17 | 41924515 | 41940997 | CD300LG | | | | | |
| 17 | 41952726 | 41987079 | MPP2 | | | | | |
| 17 | 41994575 | 41995355 | FAM215A | | | | | |
| 17 | 42018169 | 42019835 | PPY | | | | | |
| 17 | 42030106 | 42081837 | PYY | | | | | |
| 17 | 42082031 | 42086436 | NAGS | | | NAGS | | |
| 17 | 42088555 | 42100519 | TMEM101 | | | | | |
| 17 | 42112002 | 42144987 | LSM12 | | | | | |
| 17 | 42148097 | 42153712 | G6PC3 | | | | | |
| 17 | 42154120 | 42201014 | HDAC5 | | | HDAC5 | | |
| 17 | 42219273 | 42239844 | C17orf53 | | | | | |
| 17 | 42248073 | 42264085 | ASB16 | | | | | |
| 17 | 42264353 | 42269099 | TMUB2 | | | | | |
| 17 | 42269172 | 42275529 | ATXN7L3 | | | | | |
| 17 | 42282400 | 42298994 | UBTF | | | | | |
| 17 | 42285138 | 42285207 | MIR6782 | | | | | |
| 17 | 42325757 | 42345502 | SLC4A1 | | | SLC4A1 | | |
| 17 | 42376941 | 42396038 | RUNDC3A | | | | | |
| 17 | 42396992 | 42402217 | SLC25A39 | | | | | |
| 17 | 42422453 | 42430474 | GRN | | | | | |
| 17 | 42431100 | 42441235 | FAM171A2 | | | | | |
| 17 | 42449548 | 42466969 | ITGA2B | | | | | |
| 17 | 42472644 | 42580970 | GPATCH8 | | | | | |
| 17 | 42634811 | 42638630 | FZD2 | | | | | |
| 17 | 42723731 | 42727717 | LINC01180 | | | | | |
| 17 | 42733761 | 42753165 | MEIOC | | | | | |
| 17 | 42754804 | 42767165 | CCDC43 | | | | | |
| 17 | 42785975 | 42829636 | DBF4B | | | | | |
| 17 | 42836546 | 42859214 | ADAM11 | | | | | |
| 17 | 42875815 | 42908179 | GJC1 | | | GJC1 | | |
| 17 | 42923720 | 42927848 | HIGD1B | | | | | |
| 17 | 42927654 | 42976993 | EFTUD2 | | | | | |
| 17 | 42977079 | 42981047 | CCDC103 | | | | | |
| 17 | 42982993 | 42992920 | GFAP | | GFAP | | | |
| 17 | 43002078 | 43025079 | KIF18B | | | | | |
| 17 | 43011985 | 43012049 | MIR6783 | | | | | |
| 17 | 43037060 | 43045644 | C1QL1 | | | | | |
| 17 | 43100705 | 43138477 | DCAKD | | | | | |
| 17 | 43138666 | 43186382 | NMT1 | | | | | |
| 17 | 43189007 | 43209900 | PLCD3 | | | | | |
| 17 | 43191734 | 43191801 | MIR6784 | | | | | |
| 17 | 43209966 | 43221543 | ACBD4 | | | | | |
| 17 | 43224683 | 43229468 | HEXIM1 | | | | | |
| 17 | 43237540 | 43247407 | HEXIM2 | | | | | |
| 17 | 43299291 | 43324683 | FMNL1 | | | | | |

| | | | | | | | | |
|---|---|---|---|---|---|---|---|---|
| 17 | 43325291 | 43345997 | MAP3K14 | | | | | |
| 17 | 43331759 | 43339479 | SPATA32 | | | | | |
| 17 | 43339446 | 43394430 | MAP3K14 | | | | | |
| 17 | 43471267 | 43510282 | ARHGAP27 | | | | | |
| 17 | 43513265 | 43568146 | PLEKHM1 | | | | | |
| 17 | 43552728 | 43552801 | MIR4315 | | | | | |
| 17 | 43583248 | 43597889 | LRRC37A4P | | | | | |
| 17 | 43677468 | 43679228 | LOC644172 | | | | | |
| 17 | 43697694 | 43715329 | MGC57346 | | | | | |
| 17 | 43697694 | 43913194 | MGC57346-CRHR1 | | | | | |
| 17 | 43697980 | 43715329 | MGC57346 | | | | | |
| 17 | 43699273 | 43913194 | MGC57346-CRHR1 | | | | | |
| 17 | 43716340 | 43723595 | CRHR1-IT1 | | | | | |
| 17 | 43861645 | 43913194 | CRHR1 | CRHR1 | CRHR1 | CRHR1 | CRHR1 | |
| 17 | 43920721 | 43972879 | MAPT | | MAPT | MAPT | | |
| 17 | 43922255 | 43924438 | SPPL2C | | | | | |
| 17 | 43971747 | 44105699 | MAPT | | MAPT | MAPT | | |
| 17 | 43973148 | 43976164 | MAPT-IT1 | | | | | |
| 17 | 44076615 | 44077060 | STH | | | | | |
| 17 | 44107281 | 44274089 | KANSL1 | | | | | |
| 17 | 44372496 | 44415160 | LRRC37A | | | | | |
| 17 | 44376499 | 44439163 | ARL17A | | | | | |
| 17 | 44376912 | 44439134 | ARL17B | | | | | |
| 17 | 44412267 | 44439163 | ARL17A | | | | | |
| 17 | 44416763 | 44439134 | ARL17B | | | | | |
| 17 | 44450178 | 44500463 | NSFP1 | | | | | |
| 17 | 44586046 | 44657088 | ARL17A | | | | | |
| 17 | 44590075 | 44633014 | LRRC37A2 | | | | | |
| 17 | 44594067 | 44657088 | ARL17A | | | | | |
| 17 | 44668034 | 44834828 | NSF | | | | | |
| 17 | 44668099 | 44718040 | NSFP1 | | | | | |
| 17 | 44839871 | 44896126 | WNT3 | | WNT3 | | | |
| 17 | 44928967 | 44954437 | WNT9B | | | | | |
| 17 | 45000485 | 45018733 | GOSR2 | | | GOSR2 | | |
| 17 | 45050382 | 45050466 | MIR5089 | | | | | |
| 17 | 45055521 | 45056614 | RPRML | | | | | |
| 17 | 45108951 | 45120079 | ARL17A | | | | | |
| 17 | 45195062 | 45266678 | CDC27 | | | | | |
| 17 | 45286427 | 45301045 | MYL4 | | | | | |
| 17 | 45331207 | 45390077 | ITGB3 | | | ITGB3 | | |
| 17 | 45380811 | 45401267 | THCAT158 | | | | | |
| 17 | 45401326 | 45518677 | EFCAB13 | | | | | |
| 17 | 45527592 | 45569986 | MRPL45P2 | | | | | |
| 17 | 45608443 | 45700642 | NPEPPS | | | | | |
| 17 | 45727203 | 45761004 | KPNB1 | | | KPNB1 | | |
| 17 | 45772629 | 45789429 | TBKBP1 | | | | | |
| 17 | 45810609 | 45823485 | TBX21 | | | | | |
| 17 | 45884732 | 45899147 | OSBPL7 | | | | | |
| 17 | 45900637 | 45908907 | MRPL10 | | | | | |
| 17 | 45908992 | 45915079 | LRRC46 | | | | | |
| 17 | 45915046 | 45918699 | SCRN2 | | | | | |
| 17 | 45922279 | 45933240 | SP6 | | | | | |
| 17 | 45973515 | 46018770 | SP2 | | | | | |
| 17 | 46018888 | 46026674 | PNPO | | PNPO | | | |
| 17 | 46029333 | 46035243 | PRR15L | | | | | |
| 17 | 46047893 | 46059152 | CDK5RAP3 | | | | | |
| 17 | 46103532 | 46115168 | COPZ2 | | | | | |
| 17 | 46114526 | 46114613 | MIR152 | | | | | |
| 17 | 46125685 | 46138907 | NFE2L1 | | | | | |
| 17 | 46147413 | 46178883 | CBX1 | | | | | |
| 17 | 46184919 | 46200105 | SNX11 | | | SNX11 | | |
| 17 | 46210801 | 46507594 | SKAP1 | | | | | |
| 17 | 46233788 | 46233873 | MIR1203 | | | | | |
| 17 | 46371708 | 46385191 | THRA1/BTR | | | | | |
| 17 | 46522194 | 46543402 | LOC101927166 | | | | | |
| 17 | 46606806 | 46608272 | HOXB1 | | | | | |

| | | | | | | | | |
|---|---|---|---|---|---|---|---|---|
| 17 | 46620018 | 46622393 | HOXB2 | | | | | |
| 17 | 46621712 | 46628603 | HOXB | | | | | |
| 17 | 46626231 | 46651810 | HOXB3 | | | | | |
| 17 | 46652868 | 46655743 | HOXB4 | | | | | |
| 17 | 46657199 | 46657309 | MIR10A | | | | | |
| 17 | 46667781 | 46683774 | HOXB | | | | | |
| 17 | 46668618 | 46671103 | HOXB5 | | | | | |
| 17 | 46669653 | 46683774 | HOXB | | | | | |
| 17 | 46673098 | 46682334 | HOXB6 | | | | | |
| 17 | 46673319 | 46679703 | HOXB | | | | | |
| 17 | 46684594 | 46688383 | HOXB7 | | | | | |
| 17 | 46689707 | 46692301 | HOXB8 | | | | | |
| 17 | 46698518 | 46703835 | HOXB9 | | | | | |
| 17 | 46709851 | 46709921 | MIR196A1 | | | | | |
| 17 | 46799081 | 46799882 | PRAC1 | | | | | |
| 17 | 46800529 | 46802120 | PRAC2 | | | | | |
| 17 | 46801769 | 46801837 | MIR3185 | | | | | |
| 17 | 46802126 | 46806111 | HOXB13 | | | | | |
| 17 | 46839592 | 46894469 | TTLL6 | | | | | |
| 17 | 46908349 | 46942607 | CALCOCO2 | | | | | |
| 17 | 46970147 | 46973232 | ATP5G1 | | | ATP5G1 | | |
| 17 | 46985730 | 47006422 | UBE2Z | | | | | |
| 17 | 47007458 | 47022204 | SNF8 | | | | | |
| 17 | 47035917 | 47045955 | GIP | | | | | |
| 17 | 47074773 | 47133507 | IGF2BP1 | | | | | |
| 17 | 47209821 | 47247351 | B4GALNT2 | | | | | |
| 17 | 47283595 | 47287936 | GNGT2 | | | | | |
| 17 | 47287588 | 47300587 | ABI3 | | | | | |
| 17 | 47300731 | 47308128 | PHOSPHO1 | | | | | |
| 17 | 47325604 | 47336027 | FLJ40194 | | | | | |
| 17 | 47365707 | 47365816 | MIR6129 | | | | | |
| 17 | 47366567 | 47439835 | ZNF652 | | | | | |
| 17 | 47438526 | 47443477 | LOC102724596 | | | | | |
| 17 | 47481409 | 47492267 | PHB | | | | | |
| 17 | 47535222 | 47539111 | LOC101927207 | | | | | |
| 17 | 47572654 | 47592382 | NGFR | | | NGFR | | |
| 17 | 47583018 | 47651426 | LOC100288866 | | | | | |
| 17 | 47588178 | 47588262 | MIR6165 | | | | | |
| 17 | 47653297 | 47661171 | NXPH3 | | | | | |
| 17 | 47676245 | 47755525 | SPOP | | | | | |
| 17 | 47778295 | 47785313 | SLC35B1 | | | | | |
| 17 | 47787686 | 47841518 | FAM117A | | | | | |
| 17 | 47865980 | 47906458 | KAT7 | | | | | |
| 17 | 47915670 | 47925379 | TAC4 | | | | | |
| 17 | 47923271 | 47926199 | FLJ45513 | | | | | |
| 17 | 48046561 | 48052323 | DLX4 | | | | | |
| 17 | 48067368 | 48072588 | DLX3 | | | | | |
| 17 | 48127712 | 48133103 | LOC284080 | | | | | |
| 17 | 48133331 | 48167849 | ITGA3 | | | | | |
| 17 | 48172100 | 48188733 | PDK2 | | | PDK2 | | |
| 17 | 48188672 | 48207246 | SAMD14 | | | | | |
| 17 | 48211098 | 48227878 | PPP1R9B | | PPP1R9B | | | |
| 17 | 48243365 | 48253293 | SGCA | | | | | |
| 17 | 48248788 | 48249837 | HILS1 | | | | | |
| 17 | 48261456 | 48279000 | COL1A1 | | | COL1A1 | | |
| 17 | 48292054 | 48292781 | LOC101927230 | | | | | |
| 17 | 48348766 | 48365216 | TMEM92 | | | | | |
| 17 | 48423392 | 48438546 | XYLT2 | | | | | |
| 17 | 48445227 | 48450562 | MRPL27 | | | | | |
| 17 | 48450580 | 48458820 | EME1 | | | | | |
| 17 | 48458593 | 48474914 | LRRC59 | | | | | |
| 17 | 48503518 | 48552206 | ACSF2 | | | | | |
| 17 | 48541852 | 48546227 | CHAD | | | | | |
| 17 | 48545689 | 48552206 | ACSF2 | | | | | |
| 17 | 48556160 | 48563341 | RSAD1 | | | | | |
| 17 | 48585744 | 48608862 | MYCBPAP | | | | | |

| | | | | | | | | |
|---|---|---|---|---|---|---|---|---|
| 17 | 48610047 | 48621111 | EPN3 | | | EPN3 | | |
| 17 | 48624449 | 48633213 | SPATA20 | | | | | |
| 17 | 48633567 | 48704832 | CACNA1G | | CACNA1G | | CACNA1G | |
| 17 | 48712217 | 48769063 | ABCC3 | | | | | |
| 17 | 48770550 | 48785270 | ANKRD40 | | | | | |
| 17 | 48796925 | 48830072 | LUC7L3 | | | | | |
| 17 | 48838394 | 48844876 | LINC00483 | | | | | |
| 17 | 48846010 | 48846091 | MIR8059 | | | | | |
| 17 | 48912604 | 48919709 | WFIKKN2 | | | | | |
| 17 | 48939586 | 48945732 | TOB1 | | | | | |
| 17 | 49039534 | 49198226 | SPAG9 | | SPAG9 | | | |
| 17 | 49230896 | 49249105 | NME1-NME2 | | | | | |
| 17 | 49230919 | 49239450 | NME1 | | NME1 | | | |
| 17 | 49242795 | 49249105 | NME2 | | | | | |
| 17 | 49254785 | 49337427 | MBTD1 | | | | | |
| 17 | 49337896 | 49375292 | UTP18 | | | | | |
| 17 | 49389969 | 49412523 | LOC101927274 | | | | | |
| 17 | 49414075 | 49419932 | LOC440446 | | | | | |
| 17 | 49707673 | 50237377 | CA10 | | | | | |
| 17 | 51062879 | 51065012 | C17orf112 | | | | | |
| 17 | 51900238 | 51902573 | KIF2B | | | | | |
| 17 | 52978051 | 53039328 | TOM1L1 | | | | | |
| 17 | 53029258 | 53046064 | COX11 | | | COX11 | | |
| 17 | 53046125 | 53241449 | STXBP4 | | | | | |
| 17 | 53342320 | 53402426 | HLF | | | | | |
| 17 | 53469973 | 53499341 | MMD | | | MMD | | |
| 17 | 53796987 | 53809482 | TMEM100 | | | | | |
| 17 | 53828339 | 53854748 | PCTP | | | | | |
| 17 | 54230835 | 54560007 | ANKFN1 | | | | | |
| 17 | 54671059 | 54672951 | NOG | | NOG | | | |
| 17 | 54869273 | 54911256 | C17orf67 | | | | | |
| 17 | 54911459 | 54946036 | DGKE | | | | | |
| 17 | 54961462 | 54962274 | MTVR2 | | | | | |
| 17 | 54965269 | 54991409 | TRIM25 | | | | | |
| 17 | 54968630 | 54968716 | MIR3614 | | | | | |
| 17 | 55015560 | 55038411 | COIL | | | | | |
| 17 | 55055467 | 55084129 | SCPEP1 | | | | | |
| 17 | 55122838 | 55124156 | RNF126P1 | | | | | |
| 17 | 55162552 | 55198705 | AKAP1 | | | AKAP1 | | |
| 17 | 55333930 | 55710544 | MSI2 | | | | | |
| 17 | 55599608 | 55600958 | LOC101927557 | | | | | |
| 17 | 55678225 | 55685773 | LOC101927539 | | | | | |
| 17 | 55821841 | 55822690 | CCDC182 | | | | | |
| 17 | 55916286 | 55927433 | MRPS23 | | | | | |
| 17 | 55938603 | 56032684 | CUEDC1 | | | | | |
| 17 | 56048909 | 56065615 | VEZF1 | | | | | |
| 17 | 56078279 | 56084707 | SRSF1 | | | | | |
| 17 | 56154251 | 56160565 | LOC101927666 | | | | | |
| 17 | 56160779 | 56167618 | DYNLL2 | | | DYNLL2 | | |
| 17 | 56232514 | 56233447 | OR4D1 | | | | | |
| 17 | 56234319 | 56236480 | MSX2P1 | | | | | |
| 17 | 56247016 | 56247940 | OR4D2 | | | | | |
| 17 | 56270088 | 56282535 | EPX | | | | | |
| 17 | 56282796 | 56296966 | MKS1 | | | | | |
| 17 | 56315786 | 56345879 | LPO | | | | | |
| 17 | 56347216 | 56358296 | MPO | | | MPO | | |
| 17 | 56378587 | 56431088 | BZRAP1 | | | BZRAP1 | | |
| 17 | 56408592 | 56408679 | MIR142 | | | | | |
| 17 | 56413336 | 56413383 | MIR4736 | | | | | |
| 17 | 56414562 | 56431088 | BZRAP1 | | | BZRAP1 | | |
| 17 | 56422535 | 56429599 | SUPT4H1 | | | | | |
| 17 | 56429860 | 56494943 | RNF43 | | | | | |
| 17 | 56497527 | 56565762 | HSF5 | | | | | |
| 17 | 56566892 | 56595251 | MTMR4 | | | | | |
| 17 | 56597197 | 56618179 | SEPT4 | | SEPT4 | | | |
| 17 | 56618947 | 56621733 | C17orf47 | | | | | |

| | | | | | | | | |
|---|---|---|---|---|---|---|---|---|
| 17 | 56634037 | 56769416 | TEX14 | | | | | |
| 17 | 56769933 | 56811703 | RAD51C | | | | | |
| 17 | 56833229 | 57062540 | PPM1E | | | | | |
| 17 | 57059999 | 57184266 | TRIM37 | | TRIM37 | | | |
| 17 | 57187307 | 57232800 | SKA2 | | | | | |
| 17 | 57215118 | 57215233 | MIR454 | | | | | |
| 17 | 57228496 | 57228582 | MIR301A | | | | | |
| 17 | 57232859 | 57284070 | PRR11 | | | | | |
| 17 | 57287370 | 57292611 | SMG8 | | | | | |
| 17 | 57297827 | 57353330 | GDPD1 | | | | | |
| 17 | 57409052 | 57479095 | YPEL2 | | | | | |
| 17 | 57443443 | 57443515 | MIR4729 | | | | | |
| 17 | 57508229 | 57604218 | LINC01476 | | | | | |
| 17 | 57642885 | 57685713 | DHX40 | | | | | |
| 17 | 57697049 | 57774317 | CLTC | | CLTC | | CLTC | |
| 17 | 57774666 | 57784959 | PTRH2 | | | | | |
| 17 | 57784862 | 57917952 | VMP1 | | | | | |
| 17 | 57918626 | 57918698 | MIR21 | | | | | |
| 17 | 57936840 | 57970306 | TUBD1 | | | | | |
| 17 | 57970406 | 58027786 | RPS6KB1 | | | RPS6KB1 | | |
| 17 | 58029722 | 58042117 | RNFT1 | | | | | |
| 17 | 58039723 | 58096413 | TBC1D3P1-DHX40P1 | | | | | |
| 17 | 58042456 | 58074333 | LOC101927755 | | | | | |
| 17 | 58120385 | 58120466 | MIR4737 | | | | | |
| 17 | 58120551 | 58156292 | HEATR6 | | | | | |
| 17 | 58160926 | 58165828 | WFDC21P | | | | | |
| 17 | 58179120 | 58180280 | LOC653653 | | | | | |
| 17 | 58227301 | 58236906 | CA4 | | | | | |
| 17 | 58254690 | 58469586 | USP32 | | | | | |
| 17 | 58308876 | 58309006 | SCARNA20 | | | | | |
| 17 | 58499864 | 58508787 | C17orf64 | | | | | |
| 17 | 58520509 | 58603601 | APPBP2 | | | APPBP2 | | |
| 17 | 58641906 | 58663981 | LOC388406 | | | | | |
| 17 | 58677543 | 58743640 | PPM1D | | | | | |
| 17 | 58755171 | 59470199 | BCAS3 | | | | | |
| 17 | 59470816 | 59486827 | TBX2 | | | | | |
| 17 | 59489111 | 59490641 | C17orf82 | | | | | |
| 17 | 59533806 | 59561664 | TBX4 | | | | | |
| 17 | 59667793 | 59668563 | NACA2 | | | | | |
| 17 | 59756546 | 59940920 | BRIP1 | | | BRIP1 | | |
| 17 | 59942727 | 60005377 | INTS2 | | | | | |
| 17 | 60019965 | 60142643 | MED13 | | | | | |
| 17 | 60342066 | 60353016 | TBC1D3P2 | | | | | |
| 17 | 60447578 | 60493839 | EFCAB3 | | | | | |
| 17 | 60501245 | 60527454 | METTL2A | | | | | |
| 17 | 60556385 | 60692841 | TLK2 | | | | | |
| 17 | 60704761 | 60770962 | MRC2 | | | | | |
| 17 | 60778674 | 60885742 | MARCH10 | | | | | |
| 17 | 60798857 | 61268734 | MIR548W | | | | | |
| 17 | 61021575 | 61021673 | MIR633 | | | | | |
| 17 | 61086897 | 61505067 | TANC2 | | | | | |
| 17 | 61509664 | 61523722 | CYB561 | | | CYB561 | | |
| 17 | 61554421 | 61575741 | ACE | | | | | |
| 17 | 61600694 | 61626338 | KCNH6 | | | KCNH6 | | |
| 17 | 61627795 | 61671642 | DCAF7 | | | | | |
| 17 | 61678230 | 61685725 | TACO1 | | | | | |
| 17 | 61699800 | 61773670 | MAP3K3 | | | | | |
| 17 | 61773248 | 61777519 | LIMD2 | | | | | |
| 17 | 61777697 | 61780045 | LOC729683 | | | | | |
| 17 | 61780191 | 61819330 | STRADA | | | | | |
| 17 | 61822609 | 61851088 | CCDC47 | | | | | |
| 17 | 61851548 | 61896677 | DDX42 | | | | | |
| 17 | 61896792 | 61905031 | FTSJ3 | | | | | |
| 17 | 61904769 | 61909387 | PSMC5 | | | | | |
| 17 | 61909440 | 61920351 | SMARCD2 | | | | | |
| 17 | 61934375 | 61941739 | TCAM1P | | | | | |

| | | | | | | | | |
|---|---|---|---|---|---|---|---|---|
| 17 | 61949371 | 61951089 | CSH2 | | | | | |
| 17 | 61957571 | 61959302 | GH2 | | | | | |
| 17 | 61972267 | 61974021 | CSH1 | | | | CSH1 | |
| 17 | 61986964 | 61988618 | CSHL1 | | | | | |
| 17 | 61994552 | 61996212 | GH1 | | | | GH1 | |
| 17 | 62006097 | 62009704 | CD79B | | | | | |
| 17 | 62015913 | 62050278 | SCN4A | | | SCN4A | SCN4A | |
| 17 | 62073430 | 62081644 | PRR29 | | | | | |
| 17 | 62079954 | 62097994 | ICAM2 | | | | | |
| 17 | 62120389 | 62207502 | ERN1 | | | | | |
| 17 | 62223329 | 62223669 | SNHG25 | | | | | |
| 17 | 62223437 | 62223517 | SNORD104 | | | | | |
| 17 | 62223698 | 62223831 | SNORA50C | | | | | |
| 17 | 62224792 | 62340683 | TEX2 | | | | | |
| 17 | 62396776 | 62407083 | PECAM1 | | | | | |
| 17 | 62461568 | 62464760 | MILR1 | | | | | |
| 17 | 62473901 | 62493184 | POLG2 | | | | | |
| 17 | 62494373 | 62502484 | DDX5 | | | | | |
| 17 | 62496891 | 62496957 | MIR3064 | | | | | |
| 17 | 62497331 | 62497431 | MIR5047 | | | | | |
| 17 | 62502853 | 62534069 | CEP95 | | | | | |
| 17 | 62540734 | 62658386 | SMURF2 | | | | | |
| 17 | 62745779 | 62777622 | LOC146880 | | | | | |
| 17 | 62776876 | 62776942 | MIR6080 | | | | | |
| 17 | 62780958 | 62833302 | PLEKHM1P1 | | | | | |
| 17 | 62818147 | 62818220 | MIR4315 | | | | | |
| 17 | 62850429 | 62915598 | LRRC37A3 | | | | | |
| 17 | 62962667 | 62971703 | AMZ2P1 | | | | | |
| 17 | 63005406 | 63052920 | GNA13 | | | | | |
| 17 | 63096929 | 63107212 | LOC100507002 | | | | | |
| 17 | 63133455 | 63223821 | RGS9 | | RGS9 | | | |
| 17 | 63453658 | 63454526 | CRAT40 | | | | | |
| 17 | 63524682 | 63557740 | AXIN2 | | | | | |
| 17 | 63631657 | 64188249 | CEP112 | | | | | |
| 17 | 64208146 | 64225556 | APOH | | | | | |
| 17 | 64298925 | 64412972 | PRKCA | PRKCA | PRKCA | PRKCA | | |
| 17 | 64783189 | 64783286 | MIR634 | | | | | |
| 17 | 64873390 | 64881395 | CACNG5 | | | | | |
| 17 | 64960979 | 65029518 | CACNG4 | | | | | |
| 17 | 65040651 | 65052911 | CACNG1 | | | | | |
| 17 | 65066553 | 65241319 | HELZ | | | | | |
| 17 | 65334031 | 65362743 | PSMD12 | | | | | |
| 17 | 65373396 | 65693379 | PITPNC1 | | | | | |
| 17 | 65713948 | 65740324 | NOL11 | | | | | |
| 17 | 65736785 | 65736917 | SNORA38B | | | | | |
| 17 | 65821779 | 65980494 | BPTF | | | | | |
| 17 | 65987214 | 65989765 | C17orf58 | | | | | |
| 17 | 66031847 | 66042970 | KPNA2 | | | KPNA2 | | |
| 17 | 66097695 | 66132070 | LINC00674 | | | | | |
| 17 | 66194800 | 66196443 | LOC440461 | | | | | |
| 17 | 66244144 | 66253305 | AMZ2 | | | | | |
| 17 | 66255322 | 66417000 | ARSG | | | | | |
| 17 | 66263166 | 66287405 | SLC16A6 | | | | | |
| 17 | 66287635 | 66417000 | ARSG | | | | | |
| 17 | 66409763 | 66529570 | PRKAR1A | | | PRKAR1A | | PRKAR1A |
| 17 | 66417421 | 66453653 | WIPI1 | | | | | |
| 17 | 66420591 | 66420689 | MIR635 | | | | | |
| 17 | 66507920 | 66547457 | PRKAR1A | | | PRKAR1A | | PRKAR1A |
| 17 | 66531256 | 66597095 | FAM20A | | | | | |
| 17 | 66624294 | 66675747 | LINC01482 | | | | | |
| 17 | 66863427 | 66951533 | ABCA8 | | | | | |
| 17 | 66970772 | 67014464 | ABCA9 | | | | | |
| 17 | 67074846 | 67138015 | ABCA6 | | | | | |
| 17 | 67095682 | 67095797 | MIR4524B | | | | | |
| 17 | 67095704 | 67095773 | MIR4524A | | | | | |
| 17 | 67144147 | 67240956 | ABCA10 | | | | | |

| | | | | | | | | |
|---|---|---|---|---|---|---|---|---|
| 17 | 67229115 | 67231153 | PRO1804 | | | | | |
| 17 | 67240575 | 67323323 | ABCA5 | | | | | |
| 17 | 67410837 | 67538470 | MAP2K6 | | | | | |
| 17 | 67590127 | 67899141 | LINC01483 | | | | | |
| 17 | 67957847 | 67979685 | LINC01497 | | | | | |
| 17 | 68047411 | 68064236 | LINC01028 | | | | | |
| 17 | 68071365 | 68131746 | KCNJ16 | | | | | |
| 17 | 68163101 | 68176183 | KCNJ2 | | KCNJ2 | KCNJ2 | KCNJ2 | |
| 17 | 69093914 | 69198320 | CASC17 | | | | | |
| 17 | 70017991 | 70022120 | LOC102723505 | | | | | |
| 17 | 70026794 | 70037438 | LINC01152 | | | | | |
| 17 | 70030247 | 70053875 | LOC102723517 | | | | | |
| 17 | 70067182 | 70112157 | SOX9 | | SOX9 | SOX9 | | |
| 17 | 70068473 | 70089622 | LOC101928205 | | | | | |
| 17 | 70076078 | 70122560 | SOX9 | | SOX9 | SOX9 | | |
| 17 | 70399462 | 70588943 | LINC00673 | | | | | |
| 17 | 70594179 | 70636611 | LINC00511 | | | | | |
| 17 | 70642084 | 71088853 | SLC39A11 | | | | | |
| 17 | 71161159 | 71168062 | SSTR2 | | | SSTR2 | | |
| 17 | 71189172 | 71204645 | COG1 | | | COG1 | | |
| 17 | 71203491 | 71228533 | FAM104A | | | | | |
| 17 | 71228371 | 71245095 | C17orf80 | | | | | |
| 17 | 71244587 | 71258019 | CPSF4L | | | | | |
| 17 | 71279762 | 71308143 | CDC42EP4 | | | | | |
| 17 | 71330522 | 71640227 | SDK2 | | | | | |
| 17 | 71733992 | 71752696 | LOC100134391 | | | | | |
| 17 | 71745408 | 71824676 | LINC00469 | | | | | |
| 17 | 71782960 | 71796934 | LOC400620 | | | | | |
| 17 | 72199794 | 72206019 | RPL38 | | | | | |
| 17 | 72206134 | 72209460 | MGC16275 | | | | | |
| 17 | 72209695 | 72258157 | TTYH2 | | | | | |
| 17 | 72270385 | 72311023 | DNAI2 | | | | | |
| 17 | 72322350 | 72351959 | KIF19 | | | | | |
| 17 | 72352554 | 72357958 | BTBD17 | | | | | |
| 17 | 72363644 | 72368739 | GPR142 | | | | | |
| 17 | 72427666 | 72443568 | GPRC5C | | | | | |
| 17 | 72462521 | 72480937 | CD300A | | | | | |
| 17 | 72517312 | 72527613 | CD300LB | | | | | |
| 17 | 72537204 | 72542310 | CD300C | | | | | |
| 17 | 72576110 | 72588370 | CD300LD | | | | | |
| 17 | 72580817 | 72590348 | C17orf77 | | | | | |
| 17 | 72606021 | 72619897 | CD300E | | | | | |
| 17 | 72667255 | 72743474 | RAB37 | | | | | |
| 17 | 72690446 | 72709139 | CD300LF | | | | | |
| 17 | 72732958 | 72743474 | RAB37 | | | | | |
| 17 | 72744750 | 72765499 | SLC9A3R1 | | | | | |
| 17 | 72744751 | 72744838 | MIR3615 | | | | | |
| 17 | 72766667 | 72772564 | NAT9 | | | | | |
| 17 | 72772621 | 72835922 | TMEM104 | | | | | |
| 17 | 72838161 | 72856966 | GRIN2C | | | GRIN2C | | |
| 17 | 72858618 | 72869156 | FDXR | | | | | |
| 17 | 72873441 | 72889909 | FADS6 | | | | | |
| 17 | 72912175 | 72919358 | USH1G | | | | | |
| 17 | 72920369 | 72930006 | OTOP2 | | | | | |
| 17 | 72931896 | 72946087 | OTOP3 | | | | | |
| 17 | 72946838 | 72971823 | HID1 | | | | | |
| 17 | 72983726 | 73001892 | CDR2L | | | | | |
| 17 | 73008758 | 73017356 | ICT1 | | | | | |
| 17 | 73028661 | 73061984 | KCTD2 | | | | | |
| 17 | 73034954 | 73043074 | ATP5H | | | | | |
| 17 | 73043278 | 73061984 | KCTD2 | | | | | |
| 17 | 73083821 | 73102255 | SLC16A5 | | | | | |
| 17 | 73106046 | 73126360 | ARMC7 | | | | | |
| 17 | 73126319 | 73127890 | NT5C | | | NT5C | | |
| 17 | 73131337 | 73150778 | HN1 | | | | | |
| 17 | 73163824 | 73179098 | SUMO2 | | | | | |

| | | | | | | | | |
|---|---|---|---|---|---|---|---|---|
| 17 | 73201544 | 73231854 | NUP85 | | | | | |
| 17 | 73232686 | 73258474 | GGA3 | | | | | |
| 17 | 73257748 | 73262457 | MRPS7 | | | | | |
| 17 | 73262309 | 73267311 | MIF4GD | | | | | |
| 17 | 73267379 | 73269976 | LOC100287042 | | | | | |
| 17 | 73269060 | 73285530 | SLC25A19 | | | | | |
| 17 | 73314156 | 73401790 | GRB2 | | | | | |
| 17 | 73402149 | 73402243 | MIR3678 | | | | | |
| 17 | 73452663 | 73496533 | TMEM94 | | | | | |
| 17 | 73494628 | 73494709 | MIR6785 | | | | | |
| 17 | 73496340 | 73511664 | CASKIN2 | | | | | |
| 17 | 73512608 | 73520820 | TSEN54 | | | | | |
| 17 | 73521782 | 73571290 | LLGL2 | | | | | |
| 17 | 73584138 | 73622927 | MYO15B | | | | | |
| 17 | 73622924 | 73663269 | RECQL5 | | RECQL5 | | | |
| 17 | 73629513 | 73637486 | SMIM5 | | | | | |
| 17 | 73642645 | 73644057 | SMIM6 | | | | | |
| 17 | 73645793 | 73663269 | RECQL5 | | RECQL5 | | | |
| 17 | 73663195 | 73704142 | SAP30BP | | | | | |
| 17 | 73717515 | 73753899 | ITGB4 | | | | | |
| 17 | 73754017 | 73761280 | GALK1 | | | GALK1 | | |
| 17 | 73772514 | 73776016 | H3F3B | | | | | |
| 17 | 73780601 | 73780688 | MIR4738 | | | | | |
| 17 | 73780919 | 73821886 | UNK | | | | | |
| 17 | 73823307 | 73840798 | UNC13D | | | | | |
| 17 | 73841779 | 73851501 | WBP2 | | | | | |
| 17 | 73870244 | 73874656 | TRIM47 | | | | | |
| 17 | 73885040 | 73893084 | TRIM65 | | | | | |
| 17 | 73894723 | 73901181 | MRPL38 | | | | | |
| 17 | 73905654 | 73937119 | FBF1 | | | | | |
| 17 | 73937588 | 73975515 | ACOX1 | | | | | |
| 17 | 73975297 | 73996667 | TEN1 | | | | | |
| 17 | 73975297 | 74002080 | TEN1-CDK3 | | | | | |
| 17 | 73996986 | 74002080 | CDK3 | | | | | |
| 17 | 73997239 | 73997409 | MIR4538 | | | | | |
| 17 | 74002926 | 74023507 | EVPL | | | | | |
| 17 | 74034855 | 74068607 | SRP68 | | | SRP68 | | |
| 17 | 74070881 | 74073622 | GALR2 | | | GALR2 | | |
| 17 | 74075262 | 74078885 | ZACN | | | ZACN | | |
| 17 | 74077073 | 74099868 | EXOC7 | | | | | |
| 17 | 74094099 | 74094157 | MIR6868 | | | | | |
| 17 | 74097092 | 74099868 | EXOC7 | | | | | |
| 17 | 74132414 | 74137380 | FOXJ1 | | | | | |
| 17 | 74136636 | 74236390 | RNF157 | | | | | |
| 17 | 74261285 | 74267379 | UBALD2 | | | | | |
| 17 | 74270129 | 74303761 | QRICH2 | | | | | |
| 17 | 74306867 | 74350230 | PRPSAP1 | | | | | |
| 17 | 74380689 | 74383941 | SPHK1 | | | | | |
| 17 | 74385612 | 74449288 | UBE2O | | | | | |
| 17 | 74449432 | 74466199 | AANAT | | | | | |
| 17 | 74466974 | 74497509 | RHBDF2 | | | | | |
| 17 | 74523429 | 74533987 | CYGB | | | | | |
| 17 | 74523667 | 74541458 | PRCD | | | | | |
| 17 | 74553845 | 74561430 | SNHG16 | | | | | |
| 17 | 74554873 | 74554951 | SNORD1C | | | | | |
| 17 | 74557189 | 74557275 | SNORD1B | | | | | |
| 17 | 74557714 | 74557788 | SNORD1A | | | | | |
| 17 | 74561460 | 74582145 | ST6GALNAC2 | | | | | |
| 17 | 74620837 | 74639920 | ST6GALNAC1 | | | | | |
| 17 | 74668009 | 74669740 | LOC105274304 | | | | | |
| 17 | 74669731 | 74707087 | MXRA7 | | | | | |
| 17 | 74708913 | 74722881 | JMJD6 | | | | | |
| 17 | 74722911 | 74729963 | METTL23 | | | | | |
| 17 | 74730196 | 74733493 | SRSF2 | | | | | |
| 17 | 74732531 | 74732630 | MIR636 | | | | | |
| 17 | 74732646 | 74775336 | MFSD11 | | | | | |

| | | | | | | | | | |
|---|---|---|---|---|---|---|---|---|---|
| 17 | 74795125 | 74803184 | LOC101928514 | | | | | | |
| 17 | 74864797 | 74946471 | MGAT5B | | | | | | |
| 17 | 75084724 | 75091068 | SNHG20 | | | | | | |
| 17 | 75084724 | 75213181 | SEC14L1 | | | | | | |
| 17 | 75085388 | 75085575 | SCARNA16 | | | | | | |
| 17 | 75085498 | 75085579 | MIR6516 | | | | | | |
| 17 | 75137004 | 75213181 | SEC14L1 | | | | | | |
| 17 | 75277491 | 75496678 | SEPT9 | | | | | | |
| 17 | 75393065 | 75393136 | MIR4316 | | | | | | |
| 17 | 75401150 | 75496678 | SEPT9 | | | | | | |
| 17 | 75543022 | 75561103 | LOC100507351 | | | | | | |
| 17 | 75718953 | 75724641 | LOC100132174 | | | | | | |
| 17 | 75875082 | 75880169 | FLJ45079 | | | | | | |
| 17 | 76000317 | 76107880 | TNRC6C | | | | | | |
| 17 | 76108998 | 76128488 | TMC6 | | | | | | |
| 17 | 76126858 | 76139049 | TMC8 | | | | | | |
| 17 | 76142433 | 76162364 | C17orf99 | | | | | | |
| 17 | 76164670 | 76169009 | SYNGR2 | | | SYNGR2 | | | |
| 17 | 76170159 | 76183285 | TK1 | | | | | | |
| 17 | 76183397 | 76203782 | AFMID | | | | | | |
| 17 | 76210276 | 76221716 | BIRC5 | | | | | | |
| 17 | 76227390 | 76237068 | TMEM235 | | | | | | |
| 17 | 76257429 | 76274573 | LOC100996291 | | | | | | |
| 17 | 76352857 | 76356160 | SOCS3 | | | | | | |
| 17 | 76356528 | 76361012 | LOC101928674 | | | | | | |
| 17 | 76374698 | 76420740 | PGS1 | | | | | | |
| 17 | 76419777 | 76499138 | DNAH17 | | | | | | |
| 17 | 76613465 | 76628139 | LOC101928710 | | | | | | |
| 17 | 76670128 | 76778424 | CYTH1 | | | | | | |
| 17 | 76792964 | 76836969 | USP36 | | | | | | |
| 17 | 76849058 | 76921472 | TIMP2 | | | | | | |
| 17 | 76886661 | 76899299 | CEP295NL | | | | | | |
| 17 | 76967334 | 76976061 | LGALS3BP | | | | | | |
| 17 | 76987797 | 77005899 | CANT1 | | | CANT1 | | | |
| 17 | 77015290 | 77045870 | C1QTNF1 | | | | | | |
| 17 | 77071018 | 77084685 | ENGASE | | | | | | |
| 17 | 77085426 | 77512230 | RBFOX3 | | | | | | |
| 17 | 77680984 | 77681058 | MIR4739 | | | | | | |
| 17 | 77681074 | 77686081 | HP09025 | | | | | | |
| 17 | 77704864 | 77716021 | ENPP7 | | | ENPP7 | | | |
| 17 | 77751976 | 77761449 | CBX2 | | | | | | |
| 17 | 77768175 | 77770915 | CBX8 | | | | | | |
| 17 | 77806954 | 77813213 | CBX4 | | | | | | |
| 17 | 77889050 | 77900524 | LOC101928766 | | | | | | |
| 17 | 77893155 | 77897803 | LOC101928738 | | | | | | |
| 17 | 77906141 | 77924656 | TBC1D16 | | | | | | |
| 17 | 78010430 | 78074412 | CCDC40 | | | | | | |
| 17 | 78075324 | 78093681 | GAA | | | GAA | | | |
| 17 | 78109012 | 78120982 | EIF4A3 | | | | | | |
| 17 | 78143790 | 78172723 | CARD14 | | | | | | |
| 17 | 78183078 | 78194199 | SGSH | | | SGSH | | | |
| 17 | 78194199 | 78227308 | SLC26A11 | | | | | | |
| 17 | 78234659 | 78372581 | RNF213 | | | | | | |
| 17 | 78325630 | 78388968 | LOC100294362 | | | | | | |
| 17 | 78388966 | 78411884 | ENDOV | | | | | | |
| 17 | 78393217 | 78393293 | MIR4730 | | | | | | |
| 17 | 78440632 | 78450404 | NPTX1 | | | | | | |
| 17 | 78518624 | 78940173 | RPTOR | | | | | | |
| 17 | 78775439 | 78779432 | LOC101928855 | | | | | | |
| 17 | 78965640 | 78973933 | CHMP6 | | | | | | |
| 17 | 79002932 | 79091232 | BAIAP2 | | | | | | |
| 17 | 79091095 | 79139872 | AATK | | AATK | | | | |
| 17 | 79099075 | 79099173 | MIR657 | | | | | | |
| 17 | 79099676 | 79099755 | MIR3065 | | | | | | |
| 17 | 79099682 | 79099749 | MIR338 | | | | | | |
| 17 | 79106995 | 79107108 | MIR1250 | | | | | | |

| | | | | | | | | |
|---|---|---|---|---|---|---|---|---|
| 17 | 79139306 | 79156964 | AATK | | AATK | | | |
| 17 | 79163392 | 79196789 | CEP131 | | | | | |
| 17 | 79202076 | 79212891 | ENTHD2 | | | | | |
| 17 | 79213110 | 79215098 | C17orf89 | | | | | |
| 17 | 79218610 | 79269139 | SLC38A10 | | | | | |
| 17 | 79276623 | 79283048 | LINC00482 | | | | | |
| 17 | 79285071 | 79304474 | TMEM105 | | | | | |
| 17 | 79349698 | 79359160 | LOC100130370 | | | | | |
| 17 | 79373520 | 79433358 | BAHCC1 | | | | | |
| 17 | 79374515 | 79374578 | MIR4740 | | | | | |
| 17 | 79418129 | 79418214 | MIR3186 | | | | | |
| 17 | 79476996 | 79479892 | ACTG1 | | | | | |
| 17 | 79495416 | 79504156 | FSCN2 | | | | | |
| 17 | 79506910 | 79519429 | FAAP100 | | | | | |
| 17 | 79523910 | 79604179 | NPLOC4 | | | | | |
| 17 | 79604196 | 79615785 | TSPAN10 | | | | | |
| 17 | 79617488 | 79623607 | PDE6G | | | | | |
| 17 | 79632065 | 79633667 | OXLD1 | | | | | |
| 17 | 79633760 | 79640936 | CCDC137 | | | | | |
| 17 | 79648223 | 79650954 | ARL16 | | | | | |
| 17 | 79650961 | 79669151 | HGS | | | HGS | | |
| 17 | 79660786 | 79660899 | MIR6786 | | | | | |
| 17 | 79670399 | 79674556 | MRPL12 | | | | | |
| 17 | 79679265 | 79688046 | SLC25A10 | | | | | |
| 17 | 79762007 | 79771889 | GCGR | | | | | |
| 17 | 79780236 | 79791170 | FAM195B | | | | | |
| 17 | 79791367 | 79792926 | PPP1R27 | | | | | |
| 17 | 79801033 | 79818544 | P4HB | | | | | |
| 17 | 79825594 | 79829282 | ARHGDIA | | | | | |
| 17 | 79845710 | 79849462 | ALYREF | | | | | |
| 17 | 79848665 | 79858409 | ANAPC11 | | | | | |
| 17 | 79859984 | 79860781 | NPB | | | | | |
| 17 | 79860776 | 79869353 | PCYT2 | | | | | |
| 17 | 79869814 | 79876058 | SIRT7 | | | | | |
| 17 | 79876144 | 79888629 | MAFG | | | | | |
| 17 | 79890261 | 79895204 | PYCR1 | | | | | |
| 17 | 79897520 | 79905109 | MYADML2 | | | | | |
| 17 | 79910382 | 79919057 | NOTUM | | | | | |
| 17 | 79935425 | 79975282 | ASPSCR1 | | | | | |
| 17 | 79976578 | 79980785 | STRA13 | | | | | |
| 17 | 79981177 | 79989027 | LRRC45 | | | | | |
| 17 | 79989531 | 79992080 | RAC3 | | | | | |
| 17 | 79993756 | 79995573 | DCXR | | | | | |
| 17 | 80005777 | 80009650 | RFNG | | | | | |
| 17 | 80009762 | 80015346 | GPS1 | | | | | |
| 17 | 80015747 | 80023697 | DUS1L | | | | | |
| 17 | 80036213 | 80056106 | FASN | | | FASN | | |
| 17 | 80047835 | 80048016 | SNORD134 | | | | | |
| 17 | 80059345 | 80170705 | CCDC57 | | | | | |
| 17 | 80186281 | 80197375 | SLC16A3 | | | | | |
| 17 | 80194543 | 80194604 | MIR6787 | | | | | |
| 17 | 80200536 | 80231618 | CSNK1D | | | CSNK1D | | |
| 17 | 80272745 | 80275480 | CD7 | | | | | |
| 17 | 80278899 | 80291921 | SECTM1 | | | | | |
| 17 | 80317122 | 80321652 | TEX19 | | | | | |
| 17 | 80332152 | 80333462 | UTS2R | | | | | |
| 17 | 80347085 | 80376513 | OGFOD3 | | | | | |
| 17 | 80376251 | 80400516 | HEXDC | | | | | |
| 17 | 80400462 | 80408707 | C17orf62 | | | | | |
| 17 | 80416059 | 80446143 | NARF | | | | | |
| 17 | 80477593 | 80562483 | FOXK2 | | | | | |
| 17 | 80572437 | 80606411 | WDR45B | | | | | |
| 17 | 80614942 | 80656598 | RAB40B | | | RAB40B | | |
| 17 | 80626108 | 80626183 | MIR4525 | | | | | |
| 17 | 80674581 | 80685893 | FN3KRP | | | | | |
| 17 | 80693451 | 80709073 | FN3K | | | | | |

| | | | | | | | | |
|---|---|---|---|---|---|---|---|---|
| 17 | 80709939 | 80901062 | TBCD | | | | | |
| 17 | 80787309 | 80797931 | ZNF750 | | | | | |
| 17 | 80901666 | 81009686 | B3GNTL1 | | | | | |
| 17 | 81037566 | 81052871 | METRNL | | | | | |
| 17 | 81174665 | 81188573 | RPL23AP87 | | | | | |
| 18 | 12074 | 15930 | LOC102723376 | | | | | |
| 18 | 109064 | 122222 | ROCK1P1 | | | | | |
| 18 | 112255 | 112339 | MIR8078 | | | | | |
| 18 | 158482 | 213739 | USP14 | | | | | |
| 18 | 214519 | 268059 | THOC1 | | | | | |
| 18 | 319354 | 500729 | COLEC12 | | | | | |
| 18 | 580342 | 582020 | CETN1 | | | CETN1 | | |
| 18 | 596997 | 650293 | CLUL1 | | | | | |
| 18 | 649619 | 658340 | TYMSOS | | | | | |
| 18 | 657603 | 673499 | TYMS | | | | | |
| 18 | 670319 | 712664 | ENOSF1 | | | | | |
| 18 | 721591 | 812327 | YES1 | | | | | |
| 18 | 904943 | 912173 | ADCYAP1 | | | ADCYAP1 | | |
| 18 | 1268311 | 1359630 | LINC00470 | | | | | |
| 18 | 2537523 | 2571502 | METTL4 | | | | | |
| 18 | 2571509 | 2616634 | NDC80 | | | | | |
| 18 | 2652168 | 2655394 | CBX3P2 | | | | | |
| 18 | 2655885 | 2805015 | SMCHD1 | | | | | |
| 18 | 2847027 | 2914090 | EMILIN2 | | | | | |
| 18 | 2916991 | 3011945 | LPIN2 | | LPIN2 | | | |
| 18 | 2943212 | 2946621 | LOC727896 | | | | | |
| 18 | 3066804 | 3220106 | MYOM1 | | | | | |
| 18 | 3247479 | 3256235 | MYL12A | | | | | |
| 18 | 3255433 | 3261848 | LOC104968399 | | | | | |
| 18 | 3262110 | 3278282 | MYL12B | | | | | |
| 18 | 3411924 | 3458406 | TGIF1 | | | TGIF1 | | |
| 18 | 3466247 | 3478976 | GAPLINC | | | | | |
| 18 | 3496029 | 3897069 | DLGAP1 | | | DLGAP1 | | |
| 18 | 3885352 | 3885432 | MIR6718 | | | | | |
| 18 | 3962352 | 4296000 | DLGAP1 | | | DLGAP1 | | |
| 18 | 5143671 | 5197255 | C18orf42 | | | | | |
| 18 | 5236722 | 5238028 | LINC00526 | | | | | |
| 18 | 5238098 | 5246505 | LINC00667 | | | | | |
| 18 | 5289017 | 5297052 | ZBTB14 | | | | | |
| 18 | 5392379 | 5628990 | EPB41L3 | | | | | |
| 18 | 5748817 | 5795900 | MIR3976HG | | | | | |
| 18 | 5840693 | 5840832 | MIR3976 | | | | | |
| 18 | 5890183 | 5892103 | TMEM200C | | | | | |
| 18 | 5954704 | 6260933 | L3MBTL4 | | | | | |
| 18 | 6374359 | 6374424 | MIR4317 | | | | | |
| 18 | 6511414 | 6590652 | LINC01387 | | | | | |
| 18 | 6728925 | 6729861 | LOC101927168 | | | | | |
| 18 | 6834431 | 6915712 | ARHGAP28 | | | | | |
| 18 | 6925472 | 6929868 | LINC00668 | | | | | |
| 18 | 6941742 | 7117813 | LAMA1 | | LAMA1 | LAMA1 | | |
| 18 | 6954675 | 6957417 | LOC101927188 | | | | | |
| 18 | 7231136 | 7232042 | LRRC30 | | | | | |
| 18 | 7567313 | 8406859 | PTPRM | | | | | |
| 18 | 8360817 | 8367032 | LOC100192426 | | | | | |
| 18 | 8609442 | 8639380 | RAB12 | | | | | |
| 18 | 8695853 | 8707619 | GACAT2 | | | | | |
| 18 | 8717368 | 8832775 | MTCL1 | | | | | |
| 18 | 9102627 | 9136718 | NDUFV2 | | | NDUFV2 | | |
| 18 | 9136750 | 9285983 | ANKRD12 | | | | | |
| 18 | 9334764 | 9402418 | TWSG1 | | | | | |
| 18 | 9475529 | 9538106 | RALBP1 | | | | | |
| 18 | 9546788 | 9619361 | PPP4R1 | | | | | |
| 18 | 9708227 | 9862553 | RAB31 | | | RAB31 | | |
| 18 | 9885722 | 9888156 | TXNDC2 | | | | | |
| 18 | 9913954 | 9960018 | VAPA | | | | | |
| 18 | 10405129 | 10414367 | LINC01254 | | | | | |

| | | | | | | | | | |
|---|---|---|---|---|---|---|---|---|---|
| 18 | 10454624 | 10488698 | APCDD1 | | | | | | |
| 18 | 10525872 | 10552766 | NAPG | | | NAPG | | | |
| 18 | 10661929 | 10666884 | LOC101927410 | | | | | | |
| 18 | 10670243 | 11148761 | PIEZO2 | | PIEZO2 | | | | |
| 18 | 10759581 | 10759647 | MIR6788 | | | | | | |
| 18 | 11488568 | 11506982 | LINC01255 | | | | | | |
| 18 | 11609556 | 11610611 | SLC35G4 | | | | | | |
| 18 | 11654883 | 11654940 | MIR7153 | | | | | | |
| 18 | 11689013 | 11885683 | GNAL | | GNAL | GNAL | | | |
| 18 | 11851388 | 11854448 | CHMP1B | | | | | | |
| 18 | 11857436 | 11885683 | GNAL | | GNAL | GNAL | | | |
| 18 | 11883470 | 11908796 | MPPE1 | | | | | | |
| 18 | 11981426 | 12030885 | IMPA2 | | | | | | |
| 18 | 12093847 | 12129748 | ANKRD62 | | | | | | |
| 18 | 12200777 | 12224709 | C18orf61 | | | | | | |
| 18 | 12254317 | 12277594 | CIDEA | | | | | | |
| 18 | 12307667 | 12329824 | TUBB6 | | | | | | |
| 18 | 12328942 | 12377275 | AFG3L2 | | | AFG3L2 | | | |
| 18 | 12407894 | 12432236 | PRELID3A | | | | | | |
| 18 | 12446510 | 12657912 | SPIRE1 | | | | | | |
| 18 | 12658737 | 12725739 | PSMG2 | | | | | | |
| 18 | 12661954 | 12702776 | CEP76 | | | | | | |
| 18 | 12702986 | 12725739 | PSMG2 | | | | | | |
| 18 | 12739484 | 12749421 | LOC100996324 | | | | | | |
| 18 | 12785476 | 12884334 | PTPN2 | | | | | | |
| 18 | 12947982 | 12987536 | SEH1L | | | | | | |
| 18 | 12991360 | 13125051 | CEP192 | | | | | | |
| 18 | 13218728 | 13427533 | LDLRAD4 | | | | | | |
| 18 | 13459945 | 13460025 | MIR5190 | | | | | | |
| 18 | 13611112 | 13611199 | MIR4526 | | | | | | |
| 18 | 13611462 | 13652753 | LDLRAD4 | | | | | | |
| 18 | 13663345 | 13726591 | FAM210A | | | | | | |
| 18 | 13726658 | 13764555 | RNMT | | | | | | |
| 18 | 13825542 | 13826861 | MC5R | | | | | | |
| 18 | 13882042 | 13915706 | MC2R | | | | | | |
| 18 | 14075988 | 14132489 | ZNF519 | | | | | | |
| 18 | 14179095 | 14227049 | ANKRD20A5P | | | | | | |
| 18 | 14337421 | 14342523 | CYP4F35P | | | | | | |
| 18 | 14477953 | 14498705 | CXADRP3 | | | | | | |
| 18 | 14507348 | 14543599 | POTEC | | | | | | |
| 18 | 14748238 | 14852737 | ANKRD30B | | | | | | |
| 18 | 14830164 | 14830241 | MIR3156 | | | | | | |
| 18 | 14946265 | 14973755 | LINC01443 | | | | | | |
| 18 | 14969605 | 14970467 | LINC01444 | | | | | | |
| 18 | 15313554 | 15325918 | LOC644669 | | | | | | |
| 18 | 18529702 | 18691812 | ROCK1 | | | | | | |
| 18 | 18822202 | 19102791 | GREB1L | | | | | | |
| 18 | 19109261 | 19180693 | ESCO1 | | | | | | |
| 18 | 19192229 | 19210206 | SNRPD1 | | | | | | |
| 18 | 19230857 | 19284766 | ABHD3 | | | | | | |
| 18 | 19263470 | 19263558 | MIR320C1 | | | | | | |
| 18 | 19321289 | 19450918 | MIB1 | | | MIB1 | | | |
| 18 | 19405439 | 19411367 | MIR133A1HG | | | | | | |
| 18 | 19405658 | 19405746 | MIR133A1 | | | | | | |
| 18 | 19408964 | 19409049 | MIR1 | | | | | | |
| 18 | 19746858 | 19782491 | GATA6 | | | | | | |
| 18 | 19993563 | 19997878 | CTAGE1 | | | | | | |
| 18 | 20303453 | 20366800 | LOC101927571 | | | | | | |
| 18 | 20513294 | 20606449 | RBBP8 | | | | | | |
| 18 | 20513311 | 20513401 | MIR4741 | | | | | | |
| 18 | 20513838 | 20606449 | RBBP8 | | | | | | |
| 18 | 20714527 | 20840434 | CABLES1 | | | | | | |
| 18 | 20875978 | 21017933 | TMEM241 | | | | | | |
| 18 | 21032786 | 21063099 | RIOK3 | | | | | | |
| 18 | 21083433 | 21111771 | C18orf8 | | | | | | |
| 18 | 21111462 | 21166581 | NPC1 | | NPC1 | NPC1 | | | |

| | | | | | | | | |
|---|---|---|---|---|---|---|---|---|
| 18 | 21178889 | 21242849 | ANKRD29 | | | | | |
| 18 | 21269406 | 21535029 | LAMA3 | | | LAMA3 | | |
| 18 | 21572736 | 21715574 | TTC39C | | | | | |
| 18 | 21718919 | 21741564 | CABYR | | | | | |
| 18 | 21742010 | 21977833 | OSBPL1A | | OSBPL1A | | | |
| 18 | 21901649 | 21901699 | MIR320C2 | | | | | |
| 18 | 22006608 | 22033494 | IMPACT | | | | | |
| 18 | 22040592 | 22059921 | HRH4 | | | | | |
| 18 | 22208145 | 22242162 | LOC729950 | | | | | |
| 18 | 22305744 | 22346609 | LOC105372028 | | | | | |
| 18 | 22641887 | 22932214 | ZNF521 | | | | | |
| 18 | 23596216 | 23671181 | SS18 | | | | | |
| 18 | 23713810 | 23773319 | PSMA8 | | | | | |
| 18 | 23806846 | 23971649 | TAF4B | | | | | |
| 18 | 24002958 | 24018008 | LINC01543 | | | | | |
| 18 | 24034873 | 24237365 | KCTD1 | | | | | |
| 18 | 24171430 | 24171499 | MIR8057 | | | | | |
| 18 | 24267584 | 24283602 | PCAT18 | | | | | |
| 18 | 24432001 | 24515910 | AQP4 | | AQP4 | | | |
| 18 | 24495594 | 24765302 | CHST9 | | | | | |
| 18 | 24916954 | 24922620 | LOC105372038 | | | | | |
| 18 | 25530926 | 25757410 | CDH2 | | CDH2 | CDH2 | | CDH2 |
| 18 | 27878875 | 27878926 | MIR302F | | | | | |
| 18 | 28569330 | 28622781 | DSC3 | | | | | |
| 18 | 28645939 | 28682395 | DSC2 | | | DSC2 | | |
| 18 | 28681550 | 28742752 | DSCAS | | | | | |
| 18 | 28709213 | 28742819 | DSC1 | | | | | |
| 18 | 28898051 | 29006951 | DSG1 | | | | | |
| 18 | 28956739 | 28993880 | DSG4 | | | DSG4 | | |
| 18 | 29027731 | 29058665 | DSG3 | | | DSG3 | | |
| 18 | 29077966 | 29136874 | DSG2 | | | | | |
| 18 | 29171729 | 29178986 | TTR | | | | | |
| 18 | 29202208 | 29264686 | B4GALT6 | | | | | |
| 18 | 29339658 | 29340843 | SLC25A52 | | | | | |
| 18 | 29409135 | 29523091 | TRAPPC8 | | | | | |
| 18 | 29598444 | 29653154 | RNF125 | | | | | |
| 18 | 29671817 | 29711524 | RNF138 | | | | | |
| 18 | 29769986 | 29800366 | MEP1B | | | | | |
| 18 | 29843483 | 30050447 | GAREM1 | | | | | |
| 18 | 30091625 | 30094597 | WBP11P1 | | | | | |
| 18 | 30252635 | 30352974 | KLHL14 | | | | | |
| 18 | 30517365 | 31020685 | CCDC178 | | | | | |
| 18 | 31158540 | 31327399 | ASXL3 | | | | | |
| 18 | 31431063 | 31803515 | NOL4 | | | | | |
| 18 | 32073253 | 32471808 | DTNA | | DTNA | DTNA | DTNA | DTNA |
| 18 | 32556891 | 32723432 | MAPRE2 | | MAPRE2 | MAPRE2 | | |
| 18 | 32820993 | 32838397 | ZNF397 | | | | | |
| 18 | 32831021 | 32870209 | ZSCAN30 | | | | | |
| 18 | 32870235 | 32890730 | ZNF271P | | | | | |
| 18 | 32912171 | 32924431 | ZNF24 | | | | | |
| 18 | 32946660 | 32957301 | ZNF396 | | | | | |
| 18 | 33048290 | 33077955 | INO80C | | | | | |
| 18 | 33171700 | 33171770 | MIR3975 | | | | | |
| 18 | 33234532 | 33291798 | GALNT1 | | | | | |
| 18 | 33484780 | 33484889 | MIR187 | | | | | |
| 18 | 33514050 | 33514105 | MIR3929 | | | | | |
| 18 | 33552587 | 33559250 | C18orf21 | | | | | |
| 18 | 33569786 | 33647557 | RPRD1A | | | | | |
| 18 | 33688493 | 33709357 | SLC39A6 | | | | | |
| 18 | 33709836 | 33754688 | ELP2 | | | | | |
| 18 | 33759963 | 33767398 | LOC101927809 | | | | | |
| 18 | 33767479 | 33848685 | MOCOS | | | | | |
| 18 | 33877658 | 34360183 | FHOD3 | | | | | |
| 18 | 34240351 | 34243381 | LOC105372071 | | | | | |
| 18 | 34359987 | 34409179 | TPGS2 | | | | | |
| 18 | 34409079 | 34805288 | KIAA1328 | | | | | |

| | | | | | | | | |
|---|---|---|---|---|---|---|---|---|
| 18 | 34481907 | 34503777 | LOC105372069 | | | | | |
| 18 | 34823007 | 35146000 | CELF4 | | | | | |
| 18 | 34854422 | 34856363 | LOC105372068 | | | | | |
| 18 | 35046301 | 35046467 | SNORA111 | | | | | |
| 18 | 35237097 | 35237178 | MIR4318 | | | | | |
| 18 | 36786887 | 37331959 | MIR924HG | | | | | |
| 18 | 37202086 | 37202139 | MIR924 | | | | | |
| 18 | 37256682 | 37256743 | MIR5583 | | | | | |
| 18 | 37646249 | 37679197 | LINC01477 | | | | | |
| 18 | 39060235 | 39100561 | KC6 | | | | | |
| 18 | 39535162 | 39661446 | PIK3C3 | | | | | |
| 18 | 39766632 | 40271389 | LINC00907 | | | | | |
| 18 | 40323182 | 40695657 | RIT2 | | | | | |
| 18 | 40847856 | 40857615 | SYT4 | | | SYT4 | | |
| 18 | 41903399 | 42111662 | LINC01478 | | | | | |
| 18 | 42111743 | 42133868 | LOC105667213 | | | | | |
| 18 | 42260137 | 42648475 | SETBP1 | | | | | |
| 18 | 42550046 | 42550131 | MIR4319 | | | | | |
| 18 | 42792946 | 43263060 | SLC14A2 | | | | | |
| 18 | 43304087 | 43332485 | SLC14A1 | | SLC14A1 | | | |
| 18 | 43405544 | 43422521 | SIGLEC15 | | | | | |
| 18 | 43427573 | 43547305 | EPG5 | | | | | |
| 18 | 43563501 | 43652250 | PSTPIP2 | | | | | |
| 18 | 43664109 | 43684199 | ATP5A1 | | | ATP5A1 | | |
| 18 | 43684297 | 43708299 | HAUS1 | | | | | |
| 18 | 43753987 | 43846955 | C18orf25 | | | | | |
| 18 | 43914186 | 44040783 | RNF165 | | | | | |
| 18 | 44056934 | 44236996 | LOXHD1 | | | | | |
| 18 | 44259080 | 44337132 | ST8SIA5 | | | | | |
| 18 | 44390022 | 44497495 | PIAS2 | | PIAS2 | PIAS2 | | |
| 18 | 44526786 | 44628614 | KATNAL2 | | | | | |
| 18 | 44542730 | 44544371 | TCEB3CL | | | | | |
| 18 | 44542730 | 44544607 | TCEB3CL2 | | | | | |
| 18 | 44548657 | 44550298 | TCEB3CL | | | | | |
| 18 | 44548657 | 44550534 | TCEB3C | | | | | |
| 18 | 44548657 | 44550534 | TCEB3CL2 | | | | | |
| 18 | 44554572 | 44556213 | TCEB3CL | | | | | |
| 18 | 44554572 | 44556449 | TCEB3C | | | | | |
| 18 | 44558942 | 44561988 | TCEB3B | | | | | |
| 18 | 44633772 | 44676871 | HDHD2 | | | | | |
| 18 | 44681389 | 44702745 | IER3IP1 | | | | | |
| 18 | 44738459 | 44775554 | SKOR2 | | | | | |
| 18 | 44906866 | 44906936 | MIR4527 | | | | | |
| 18 | 45359465 | 45457517 | SMAD2 | | | SMAD2 | | |
| 18 | 45553638 | 45935793 | ZBTB7C | | | | | |
| 18 | 46065426 | 46389586 | CTIF | | | | | |
| 18 | 46196970 | 46197039 | MIR4743 | | | | | |
| 18 | 46446222 | 46477081 | SMAD7 | | | | | |
| 18 | 46570171 | 46987079 | DYM | | | DYM | | |
| 18 | 46576056 | 46576138 | MIR4744 | | | | | |
| 18 | 47007547 | 47013644 | C18orf32 | | | | | |
| 18 | 47007547 | 47018935 | RPL17-C18orf32 | | | | | |
| 18 | 47013742 | 47013792 | MIR1539 | | | | | |
| 18 | 47014850 | 47018935 | RPL17 | | | | | |
| 18 | 47015604 | 47015694 | SNORD58C | | | | | |
| 18 | 47017652 | 47017717 | SNORD58A | | | | | |
| 18 | 47018033 | 47018099 | SNORD58B | | | | | |
| 18 | 47088400 | 47119279 | LIPG | | | | | |
| 18 | 47309873 | 47340251 | ACAA2 | | | ACAA2 | | |
| 18 | 47340392 | 47340813 | SCARNA17 | | | | | |
| 18 | 47340392 | 47377429 | SNHG22 | | | | | |
| 18 | 47349155 | 47721451 | MYO5B | | | | | |
| 18 | 47652868 | 47652933 | MIR4320 | | | | | |
| 18 | 47753563 | 47792892 | CFAP53 | | | | | |
| 18 | 47793251 | 47806387 | MBD1 | | | | | |
| 18 | 47808712 | 47814692 | CXXC1 | | | | | |

| | | | | | | | | |
|---|---|---|---|---|---|---|---|---|
| 18 | 47901391 | 47920538 | SKA1 | | | | | |
| 18 | 48086483 | 48258196 | MAPK4 | | | | | |
| 18 | 48321489 | 48351754 | MRO | | | | | |
| 18 | 48405431 | 48476162 | ME2 | | | ME2 | | |
| 18 | 48494386 | 48514490 | ELAC1 | | | | | |
| 18 | 48556582 | 48611411 | SMAD4 | | | SMAD4 | | |
| 18 | 48700919 | 48724051 | MEX3C | | | | | |
| 18 | 48918411 | 49088839 | LOC100287225 | | | | | |
| 18 | 49866541 | 51062273 | DCC | | | | | |
| 18 | 50763470 | 50763560 | MIR4528 | | | | | |
| 18 | 51094816 | 51124221 | LOC102724651 | | | | | |
| 18 | 51105992 | 51107429 | LOC101928167 | | | | | |
| 18 | 51677970 | 51751158 | MBD2 | | | | | |
| 18 | 51748653 | 51748782 | SNORA37 | | | | | |
| 18 | 51795848 | 51824604 | POLI | | | POLI | | |
| 18 | 51851061 | 51880943 | STARD6 | | | | | |
| 18 | 51884286 | 51908405 | C18orf54 | | | | | |
| 18 | 52254989 | 52266724 | DYNAP | | | | | |
| 18 | 52495707 | 52562747 | RAB27B | | | RAB27B | | |
| 18 | 52568739 | 52626739 | CCDC68 | | | | | |
| 18 | 52773134 | 52791537 | LOC101927229 | | | | | |
| 18 | 52889561 | 53150171 | TCF4 | | | TCF4 | | |
| 18 | 53146451 | 53146529 | MIR4529 | | | | | |
| 18 | 53548918 | 53587430 | LINC01416 | | | | | |
| 18 | 53750586 | 53804767 | LINC01539 | | | | | |
| 18 | 54270052 | 54305920 | TXNL1 | | | | | |
| 18 | 54318615 | 54697036 | WDR7 | | | WDR7 | | |
| 18 | 54721803 | 54739350 | LINC-ROR | | | | | |
| 18 | 54814292 | 54817639 | BOD1L2 | | | | | |
| 18 | 55019720 | 55036161 | ST8SIA3 | | | | | |
| 18 | 55102916 | 55158530 | ONECUT2 | | | | | |
| 18 | 55212072 | 55253969 | FECH | | | | | |
| 18 | 55267893 | 55289177 | NARS | | | | | |
| 18 | 55297533 | 55336508 | LOC100505549 | | | | | |
| 18 | 55313658 | 55470327 | ATP8B1 | | | ATP8B1 | | |
| 18 | 55711609 | 56068772 | NEDD4L | | | NEDD4L | | |
| 18 | 56118305 | 56118390 | MIR122 | | | | | |
| 18 | 56118311 | 56118384 | MIR3591 | | | | | |
| 18 | 56148481 | 56296189 | ALPK2 | | | | | |
| 18 | 56267851 | 56267982 | SNORA108 | | | | | |
| 18 | 56337240 | 56339105 | LOC101927322 | | | | | |
| 18 | 56338617 | 56417371 | MALT1 | | | | | |
| 18 | 56529831 | 56653712 | ZNF532 | | | | | |
| 18 | 56702910 | 56720446 | OACYLP | | | | | |
| 18 | 56807088 | 56826069 | SEC11C | | | | | |
| 18 | 56887399 | 56898006 | GRP | | | | | |
| 18 | 56934266 | 56940625 | RAX | | | | | |
| 18 | 56962635 | 56985881 | CPLX4 | | | | | |
| 18 | 56995055 | 57026508 | LMAN1 | | | | | |
| 18 | 57098170 | 57364644 | CCBE1 | | | | | |
| 18 | 57567191 | 57571538 | PMAIP1 | | | | | |
| 18 | 58038563 | 58040001 | MC4R | | | | | |
| 18 | 59157774 | 59222365 | CDH20 | | | | | |
| 18 | 59415408 | 59421928 | LINC01544 | | | | | |
| 18 | 59482303 | 59560304 | RNF152 | | | | | |
| 18 | 59711457 | 59854289 | PIGN | | | | | |
| 18 | 59854523 | 59974355 | KIAA1468 | | | | | |
| 18 | 59992519 | 60054943 | TNFRSF11A | | | | | |
| 18 | 60190657 | 60253976 | ZCCHC2 | | | | | |
| 18 | 60382671 | 60647676 | PHLPP1 | | PHLPP1 | | | |
| 18 | 60790578 | 60986613 | BCL2 | | | | | |
| 18 | 60994970 | 61034506 | KDSR | | | | | |
| 18 | 61056424 | 61089752 | VPS4B | | | VPS4B | | |
| 18 | 61144143 | 61172318 | SERPINB5 | | | | | |
| 18 | 61223392 | 61236560 | SERPINB12 | | | | | |
| 18 | 61254533 | 61266433 | SERPINB13 | | | | | |

| | | | | | | | | |
|---|---|---|---|---|---|---|---|---|
| 18 | 61304492 | 61311553 | SERPINB4 | | | | | |
| 18 | 61322430 | 61329197 | SERPINB3 | | | | | |
| 18 | 61369537 | 61391127 | SERPINB11 | | | | | |
| 18 | 61420280 | 61472610 | SERPINB7 | | | | | |
| 18 | 61554938 | 61571124 | SERPINB2 | | | | | |
| 18 | 61575223 | 61603345 | SERPINB10 | | | | | |
| 18 | 61616587 | 61627645 | HMSD | | | | | |
| 18 | 61637262 | 61656608 | SERPINB8 | | | | | |
| 18 | 61747242 | 61816260 | LINC00305 | | | | | |
| 18 | 61771324 | 62090827 | LOC284294 | | | | | |
| 18 | 61880317 | 61927290 | LINC01538 | | | | | |
| 18 | 63417487 | 63549202 | CDH7 | | | | | |
| 18 | 64168423 | 64271375 | CDH19 | | | | | |
| 18 | 64748820 | 64748923 | MIR5011 | | | | | |
| 18 | 65173818 | 65183967 | DSEL | | | | | |
| 18 | 65183782 | 65566856 | LOC643542 | | | | | |
| 18 | 66340924 | 66382353 | TMX3 | | | | | |
| 18 | 66382490 | 66722426 | CCDC102B | | | | | |
| 18 | 67068283 | 67516322 | DOK6 | | | | | |
| 18 | 67137166 | 67138972 | LOC105372179 | | | | | |
| 18 | 67530192 | 67624412 | CD226 | | | | | |
| 18 | 67671042 | 67872962 | RTTN | | | | | |
| 18 | 67956136 | 67997434 | SOCS6 | | | | | |
| 18 | 68002674 | 68019695 | LOC101927481 | | | | | |
| 18 | 68047375 | 68051827 | LOC101060542 | | | | | |
| 18 | 68297754 | 68318093 | GTSCR1 | | | | | |
| 18 | 69187199 | 69246192 | LINC01541 | | | | | |
| 18 | 69399851 | 69449462 | LOC102724913 | | | | | |
| 18 | 70203914 | 70211723 | CBLN2 | | | | | |
| 18 | 70409548 | 70532934 | NETO1 | | | NETO1 | | |
| 18 | 70535622 | 70548634 | LOC100505797 | | | | | |
| 18 | 70821292 | 70931733 | LOC400655 | | | | | |
| 18 | 70992175 | 71017124 | LOC100505817 | | | | | |
| 18 | 71740587 | 71815100 | FBXO15 | | | | | |
| 18 | 71815745 | 71826204 | TIMM21 | | | | | |
| 18 | 71920526 | 71959251 | CYB5A | | | | | |
| 18 | 71983109 | 72026422 | C18orf63 | | | | | |
| 18 | 72076682 | 72080443 | LOC101927606 | | | | | |
| 18 | 72102962 | 72124503 | FAM69C | | | | | |
| 18 | 72163499 | 72190689 | CNDP2 | | | | | |
| 18 | 72201691 | 72252261 | CNDP1 | | CNDP1 | | | |
| 18 | 72259009 | 72265071 | LINC00909 | | | | | |
| 18 | 72342918 | 72777628 | ZNF407 | | | | | |
| 18 | 72907064 | 72921303 | ZADH2 | | | | | |
| 18 | 72922709 | 73001905 | TSHZ1 | | | | | |
| 18 | 73121430 | 73139658 | SMIM21 | | | | | |
| 18 | 73408037 | 73424358 | LOC100505853 | | | | | |
| 18 | 73834952 | 73857210 | LOC339298 | | | | | |
| 18 | 74069636 | 74207146 | ZNF516 | | | | | |
| 18 | 74207476 | 74210045 | C18orf65 | | | | | |
| 18 | 74240611 | 74271784 | LINC00908 | | | | | |
| 18 | 74331733 | 74335516 | LINC00683 | | | | | |
| 18 | 74334737 | 74350968 | LOC101927651 | | | | | |
| 18 | 74401985 | 74405592 | LOC400661 | | | | | |
| 18 | 74506687 | 74534251 | LOC100131655 | | | | | |
| 18 | 74534562 | 74682682 | ZNF236 | | | | | |
| 18 | 74690788 | 74844774 | MBP | | | | | |
| 18 | 74962007 | 74982096 | GALR1 | | | GALR1 | | |
| 18 | 75683252 | 75705683 | LINC01029 | | | | | |
| 18 | 76740274 | 76758969 | SALL3 | | SALL3 | SALL3 | | |
| 18 | 76829274 | 77138282 | ATP9B | | | ATP9B | | |
| 18 | 77155771 | 77289323 | NFATC1 | | | | | |
| 18 | 77439800 | 77514510 | CTDP1 | | | | | |
| 18 | 77623667 | 77659816 | KCNG2 | | | | | |
| 18 | 77662419 | 77711653 | PQLC1 | | | | | |
| 18 | 77724581 | 77730822 | HSBP1L1 | | | | | |

| | | | | | | | | |
|---|---|---|---|---|---|---|---|---|
| 18 | 77732866 | 77793935 | TXNL4A | | | | | |
| 18 | 77794345 | 77810652 | RBFA | | | | | |
| 18 | 77827255 | 77839206 | RBFADN | | | | | |
| 18 | 77866914 | 77898228 | ADNP2 | | | | | |
| 18 | 77905806 | 78005397 | PARD6G | | | PARD6G | | |
| 19 | 60950 | 70966 | WASH5P | | | | | |
| 19 | 71972 | 72110 | MIR1302 | | | | | |
| 19 | 76219 | 77690 | FAM138A | | | | | |
| 19 | 76219 | 77690 | FAM138F | | | | | |
| 19 | 110678 | 111596 | OR4F17 | | | | | |
| 19 | 197015 | 202209 | LINC01002 | | | | | |
| 19 | 281039 | 291435 | PLPP2 | | | | | |
| 19 | 305574 | 344791 | MIER2 | | | | | |
| 19 | 362056 | 376013 | THEG | | | | | |
| 19 | 405442 | 409170 | C2CD4C | | | | | |
| 19 | 416582 | 460996 | SHC2 | | | | | |
| 19 | 463345 | 474983 | ODF3L2 | | | | | |
| 19 | 496489 | 505343 | MADCAM1 | | | | | |
| 19 | 507496 | 519654 | TPGS1 | | | | | |
| 19 | 531732 | 542087 | CDC34 | | | | | |
| 19 | 544033 | 549920 | GZMM | | | | | |
| 19 | 571276 | 583493 | BSG | | | | | |
| 19 | 589892 | 617159 | HCN2 | | | HCN2 | | |
| 19 | 617222 | 633568 | POLRMT | | | POLRMT | | |
| 19 | 639894 | 643604 | FGF22 | | | | | |
| 19 | 647525 | 663233 | RNF126 | | | | | |
| 19 | 676388 | 683392 | FSTL3 | | | | | |
| 19 | 685520 | 695461 | PRSS57 | | | | | |
| 19 | 708952 | 748330 | PALM | | | | | |
| 19 | 751145 | 764318 | MISP | | | | | |
| 19 | 797391 | 812327 | PTBP1 | | | | | |
| 19 | 804939 | 805001 | MIR4745 | | | | | |
| 19 | 812487 | 821952 | PLPPR3 | | | | | |
| 19 | 813583 | 813653 | MIR3187 | | | | | |
| 19 | 827825 | 832018 | AZU1 | | | | | |
| 19 | 840984 | 848175 | PRTN3 | | | | | |
| 19 | 852208 | 856246 | ELANE | | | | | |
| 19 | 859658 | 863569 | CFD | | | | | |
| 19 | 867961 | 893218 | MED16 | | | | | |
| 19 | 893483 | 893590 | RNU6-2 | | | | | |
| 19 | 896502 | 913225 | R3HDM4 | | | | | |
| 19 | 917341 | 921015 | KISS1R | | KISS1R | KISS1R | KISS1R | |
| 19 | 926036 | 972803 | ARID3A | | | | | |
| 19 | 984327 | 994569 | WDR18 | | | | | |
| 19 | 1000417 | 1009731 | GRIN3B | | | | | |
| 19 | 1009649 | 1021141 | TMEM259 | | | | | |
| 19 | 1021520 | 1021627 | RNU6-2 | | | | | |
| 19 | 1026273 | 1039067 | CNN2 | | | | | |
| 19 | 1040101 | 1065570 | ABCA7 | | | | | |
| 19 | 1065921 | 1087830 | HMHA1 | | | | | |
| 19 | 1086577 | 1095391 | POLR2E | | | | | |
| 19 | 1103924 | 1106788 | GPX4 | | | | | |
| 19 | 1107632 | 1174282 | SBNO2 | | | | | |
| 19 | 1205797 | 1228434 | STK11 | | | | | |
| 19 | 1229946 | 1237990 | CBARP | | | | | |
| 19 | 1241748 | 1244824 | ATP5D | | | ATP5D | | |
| 19 | 1248551 | 1259142 | MIDN | | | | | |
| 19 | 1267469 | 1274808 | CIRBP | | | | | |
| 19 | 1275519 | 1279243 | C19orf24 | | | | | |
| 19 | 1286152 | 1301429 | EFNA2 | | | | | |
| 19 | 1354975 | 1378430 | MUM1 | | | | | |
| 19 | 1383882 | 1395588 | NDUFS7 | | NDUFS7 | | | |
| 19 | 1397024 | 1401569 | GAMT | | | | | |
| 19 | 1407567 | 1435686 | DAZAP1 | | | | | |
| 19 | 1438362 | 1440496 | RPS15 | | | | | |
| 19 | 1450147 | 1473243 | APC2 | | | | | |

| | | | | | | | | |
|---|---|---|---|---|---|---|---|---|
| 19 | 1473199 | 1479228 | C19orf25 | | | | | |
| 19 | 1481426 | 1490449 | PCSK4 | | | | | |
| 19 | 1491164 | 1497924 | REEP6 | | | | | |
| 19 | 1505016 | 1513188 | ADAMTSL5 | | | | | |
| 19 | 1524072 | 1535455 | PLK5 | | | | | |
| 19 | 1554667 | 1568057 | MEX3D | | | | | |
| 19 | 1576669 | 1592760 | MBD3 | | | | | |
| 19 | 1597153 | 1605483 | UQCR11 | | | | | |
| 19 | 1609288 | 1652328 | TCF3 | | | | | |
| 19 | 1753661 | 1775444 | ONECUT3 | | | | | |
| 19 | 1782073 | 1812275 | ATP8B3 | | | | | |
| 19 | 1815244 | 1848452 | REXO1 | | | | | |
| 19 | 1816157 | 1816237 | MIR1909 | | | | | |
| 19 | 1822087 | 1824542 | LOC100288123 | | | | | |
| 19 | 1852397 | 1863564 | KLF16 | | | | | |
| 19 | 1876974 | 1885518 | ABHD17A | | | | | |
| 19 | 1905370 | 1913446 | ADAT3 | | | | | |
| 19 | 1905372 | 1926012 | SCAMP4 | | | | | |
| 19 | 1941160 | 1954548 | CSNK1G2 | | | | | |
| 19 | 1985446 | 2015702 | BTBD2 | | | | | |
| 19 | 2037469 | 2051243 | MKNK2 | | | | | |
| 19 | 2071034 | 2096269 | MOB3A | | | | | |
| 19 | 2096867 | 2099583 | IZUMO4 | | | | | |
| 19 | 2100986 | 2151556 | AP3D1 | | | AP3D1 | | |
| 19 | 2164147 | 2232577 | DOT1L | | | | | |
| 19 | 2229950 | 2236352 | PLEKHJ1 | | | | | |
| 19 | 2234060 | 2234148 | MIR1227 | | | | | |
| 19 | 2235827 | 2235925 | MIR6789 | | | | | |
| 19 | 2236815 | 2248678 | SF3A2 | | | | | |
| 19 | 2249112 | 2252072 | AMH | | | | | |
| 19 | 2250637 | 2250717 | MIR4321 | | | | | |
| 19 | 2252249 | 2256422 | JSRP1 | | | | | |
| 19 | 2269484 | 2273487 | OAZ1 | | | | | |
| 19 | 2274630 | 2282181 | C19orf35 | | | | | |
| 19 | 2289773 | 2308156 | LINGO3 | | | | | |
| 19 | 2321519 | 2328614 | LSM7 | | | | | |
| 19 | 2328628 | 2355100 | SPPL2B | | | | | |
| 19 | 2389783 | 2426086 | TMPRSS9 | | | | | |
| 19 | 2425621 | 2427914 | TIMM13 | | | | | |
| 19 | 2428162 | 2456966 | LMNB2 | | | | | |
| 19 | 2434911 | 2434998 | MIR7108 | | | | | |
| 19 | 2458934 | 2462183 | LOC101928602 | | | | | |
| 19 | 2476122 | 2478257 | GADD45B | | | | | |
| 19 | 2511217 | 2702746 | GNG7 | | | | | |
| 19 | 2630712 | 2630791 | MIR7850 | | | | | |
| 19 | 2714564 | 2721390 | DIRAS1 | | | | | |
| 19 | 2732522 | 2740074 | SLC39A3 | | | | | |
| 19 | 2754711 | 2783354 | SGTA | | | | | |
| 19 | 2785457 | 2813602 | THOP1 | | | | | |
| 19 | 2819871 | 2836733 | ZNF554 | | | | | |
| 19 | 2841432 | 2860472 | ZNF555 | | | | | |
| 19 | 2867332 | 2878503 | ZNF556 | | | | | |
| 19 | 2900895 | 2918474 | ZNF57 | | | | | |
| 19 | 2933215 | 2944969 | ZNF77 | | | | | |
| 19 | 2977535 | 2995182 | TLE6 | | | | | |
| 19 | 2997635 | 3047633 | TLE2 | | | | | |
| 19 | 3052907 | 3062964 | AES | | | | | |
| 19 | 3094407 | 3124000 | GNA11 | | | | | |
| 19 | 3136029 | 3163767 | GNA15 | | | | | |
| 19 | 3141573 | 3155173 | LOC100996351 | | | | | |
| 19 | 3178735 | 3180330 | S1PR4 | | | | | |
| 19 | 3185874 | 3209573 | NCLN | | | | | |
| 19 | 3224700 | 3297073 | CELF5 | | | | | |
| 19 | 3359560 | 3469215 | NFIC | | | | | |
| 19 | 3474404 | 3480540 | SMIM24 | | | | | |
| 19 | 3490818 | 3500938 | DOHH | | | | | |

| | | | | | | | | |
|---|---|---|---|---|---|---|---|---|
| 19 | 3506294 | 3536755 | FZR1 | | | | | |
| 19 | 3539154 | 3544028 | C19orf71 | | | | | |
| 19 | 3544196 | 3557582 | MFSD12 | | | | | |
| 19 | 3572942 | 3579081 | HMG20B | | | | | |
| 19 | 3585568 | 3593539 | GIPC3 | | | | | |
| 19 | 3594503 | 3606831 | TBXA2R | | | | | |
| 19 | 3607244 | 3626813 | CACTIN | | | | | |
| 19 | 3630178 | 3700490 | PIP5K1C | | | | | |
| 19 | 3708334 | 3750811 | TJP3 | | | | | |
| 19 | 3742815 | 3987432 | MIR1268A | | | | | |
| 19 | 3750770 | 3761673 | APBA3 | | | | | |
| 19 | 3762664 | 3767563 | MRPL54 | | | | | |
| 19 | 3769088 | 3772219 | RAX2 | | | | | |
| 19 | 3777966 | 3801810 | MATK | | | MATK | | |
| 19 | 3804021 | 3869027 | ZFR2 | | | | | |
| 19 | 3880617 | 3928080 | ATCAY | | ATCAY | | | |
| 19 | 3933100 | 3942414 | NMRK2 | | | | | |
| 19 | 3958450 | 3971121 | DAPK3 | | | | | |
| 19 | 3961411 | 3961510 | MIR637 | | | | | |
| 19 | 3976053 | 3985461 | EEF2 | | | | | |
| 19 | 3982504 | 3982570 | SNORD37 | | | | | |
| 19 | 4007595 | 4039384 | PIAS4 | | | | | |
| 19 | 4043301 | 4066943 | ZBTB7A | | | | | |
| 19 | 4090319 | 4124126 | MAP2K2 | | | | | |
| 19 | 4153597 | 4173051 | CREB3L3 | | | | | |
| 19 | 4174105 | 4182596 | SIRT6 | | | | | |
| 19 | 4183350 | 4224811 | ANKRD24 | | | | | |
| 19 | 4229539 | 4237524 | EBI3 | | | | | |
| 19 | 4247110 | 4269085 | CCDC94 | | | | | |
| 19 | 4278597 | 4290720 | SHD | | | | | |
| 19 | 4292224 | 4302428 | TMIGD2 | | | | | |
| 19 | 4304590 | 4323843 | FSD1 | | | | | |
| 19 | 4324039 | 4338847 | STAP2 | | | | | |
| 19 | 4343523 | 4360083 | MPND | | | | | |
| 19 | 4360363 | 4400565 | SH3GL1 | | | SH3GL1 | SH3GL1 | |
| 19 | 4402659 | 4443394 | CHAF1A | | | | | |
| 19 | 4445002 | 4457791 | UBXN6 | | | | | |
| 19 | 4445974 | 4446045 | MIR4746 | | | | | |
| 19 | 4472192 | 4502232 | HDGFRP2 | | | | | |
| 19 | 4502191 | 4517716 | PLIN4 | | | | | |
| 19 | 4522543 | 4535208 | PLIN5 | | | | | |
| 19 | 4537226 | 4540036 | LRG1 | | | | | |
| 19 | 4542599 | 4559771 | SEMA6B | | | | | |
| 19 | 4639526 | 4655580 | TNFAIP8L1 | | | | | |
| 19 | 4657556 | 4670415 | MYDGF | | | | | |
| 19 | 4675243 | 4685960 | DPP9 | | | | | |
| 19 | 4769116 | 4770791 | MIR7 | | | | | |
| 19 | 4791727 | 4795571 | FEM1A | | | | | |
| 19 | 4815935 | 4831754 | TICAM1 | | | | | |
| 19 | 4838345 | 4867780 | PLIN3 | | | | | |
| 19 | 4890448 | 4902879 | ARRDC5 | | | | | |
| 19 | 4903091 | 4962165 | UHRF1 | | | | | |
| 19 | 4932698 | 4932752 | MIR4747 | | | | | |
| 19 | 4969123 | 5153608 | KDM4B | | | | | |
| 19 | 5205518 | 5340814 | PTPRS | | | PTPRS | | |
| 19 | 5455425 | 5456867 | ZNRF4 | | | | | |
| 19 | 5558177 | 5568045 | TINCR | | | | | |
| 19 | 5587009 | 5622938 | SAFB2 | | | | | |
| 19 | 5623045 | 5668489 | SAFB | | | | | |
| 19 | 5678424 | 5680907 | C19orf70 | | | | | |
| 19 | 5680775 | 5688534 | HSD11B1L | | | | | |
| 19 | 5690271 | 5691678 | RPL36 | | | | | |
| 19 | 5691844 | 5720463 | LONP1 | | | LONP1 | | |
| 19 | 5720687 | 5778742 | CATSPERD | | | | | |
| 19 | 5782970 | 5784776 | PRR22 | | | | | |
| 19 | 5785152 | 5791249 | DUS3L | | | | | |

| | | | | | | | | |
|---|---|---|---|---|---|---|---|---|
| 19 | 5823817 | 5828335 | NRTN | | | NRTN | NRTN | |
| 19 | 5830636 | 5839742 | FUT6 | | | | | |
| 19 | 5842898 | 5851485 | FUT3 | | | | | |
| 19 | 5847477 | 5858250 | LOC101928844 | | | | | |
| 19 | 5865836 | 5870551 | FUT5 | | | | | |
| 19 | 5891286 | 5904024 | NDUFA11 | | | | | |
| 19 | 5904851 | 5910263 | VMAC | | | | | |
| 19 | 5913654 | 5916222 | CAPS | | | | | |
| 19 | 5916150 | 5978320 | RANBP3 | | | | | |
| 19 | 5978413 | 6020374 | LOC100128568 | | | | | |
| 19 | 5993174 | 6110664 | RFX2 | | | | | |
| 19 | 6135643 | 6193114 | ACSBG2 | | | | | |
| 19 | 6210391 | 6279959 | MLLT1 | | | | | |
| 19 | 6306509 | 6333640 | ACER1 | | | | | |
| 19 | 6361462 | 6368915 | CLPP | | | CLPP | | |
| 19 | 6372443 | 6375261 | ALKBH7 | | | | | |
| 19 | 6375304 | 6375860 | PSPN | | | | | |
| 19 | 6379579 | 6393291 | GTF2F1 | | | | | |
| 19 | 6389648 | 6389714 | MIR6885 | | | | | |
| 19 | 6392931 | 6392994 | MIR6790 | | | | | |
| 19 | 6413118 | 6424822 | KHSRP | | | | | |
| 19 | 6416420 | 6416522 | MIR3940 | | | | | |
| 19 | 6426047 | 6433790 | SLC25A41 | | | | | |
| 19 | 6440074 | 6459781 | SLC25A23 | | | | | |
| 19 | 6464259 | 6467230 | CRB3 | | | | | |
| 19 | 6467214 | 6481819 | DENND1C | | | | | |
| 19 | 6494329 | 6502859 | TUBB4A | | TUBB4A | | | |
| 19 | 6531009 | 6535939 | TNFSF9 | | | | | |
| 19 | 6585849 | 6591163 | CD70 | | | | | |
| 19 | 6663147 | 6670599 | TNFSF14 | | | | | |
| 19 | 6677845 | 6720693 | C3 | | | C3 | | |
| 19 | 6729924 | 6737633 | GPR108 | | | | | |
| 19 | 6736722 | 6736789 | MIR6791 | | | | | |
| 19 | 6739692 | 6751537 | TRIP10 | | | | | |
| 19 | 6752172 | 6767523 | SH2D3A | | | | | |
| 19 | 6772678 | 6857377 | VAV1 | | | | | |
| 19 | 6887559 | 6940464 | ADGRE1 | | | | | |
| 19 | 6952510 | 6990857 | ADGRE4P | | | | | |
| 19 | 7004963 | 7006426 | FLJ25758 | | | | | |
| 19 | 7030593 | 7033022 | MBD3L5 | | | | | |
| 19 | 7037754 | 7040184 | MBD3L4 | | | | | |
| 19 | 7049350 | 7051746 | MBD3L2 | | | | | |
| 19 | 7056215 | 7058645 | MBD3L3 | | | | | |
| 19 | 7069470 | 7087978 | ZNF557 | | | | | |
| 19 | 7112265 | 7294313 | INSR | | | INSR | | |
| 19 | 7459998 | 7537371 | ARHGEF18 | | | | | |
| 19 | 7537722 | 7538247 | LOC100128573 | | | | | |
| 19 | 7541755 | 7554286 | PEX11G | | | | | |
| 19 | 7562444 | 7573336 | C19orf45 | | | | | |
| 19 | 7581003 | 7585911 | ZNF358 | | | | | |
| 19 | 7587495 | 7598895 | MCOLN1 | | | MCOLN1 | | |
| 19 | 7599037 | 7626653 | PNPLA6 | | PNPLA6 | | PNPLA6 | |
| 19 | 7660787 | 7683196 | CAMSAP3 | | | | | |
| 19 | 7682324 | 7682391 | MIR6792 | | | | | |
| 19 | 7684410 | 7694439 | XAB2 | | | | | |
| 19 | 7694670 | 7696510 | PET100 | | | | | |
| 19 | 7696500 | 7698634 | PCP2 | | | | | |
| 19 | 7701986 | 7712760 | STXBP2 | | | | STXBP2 | |
| 19 | 7733971 | 7735340 | RETN | | | | | |
| 19 | 7741942 | 7744719 | MCEMP1 | | | | | |
| 19 | 7745706 | 7747748 | TRAPPC5 | | | | | |
| 19 | 7753642 | 7767032 | FCER2 | | | | | |
| 19 | 7793842 | 7797057 | CLEC4G | | | | | |
| 19 | 7804880 | 7812464 | CD209 | | | | | |
| 19 | 7828034 | 7834491 | CLEC4M | | | | | |
| 19 | 7852369 | 7855898 | CLEC4GP1 | | | | | |

| | | | | | | | | | |
|---|---|---|---|---|---|---|---|---|---|
| 19 | 7895160 | 7929862 | EVI5L | | | | | | |
| 19 | 7933604 | 7939326 | PRR36 | | | | | | |
| 19 | 7943516 | 7945117 | LYPLA2P2 | | | | | | |
| 19 | 7953389 | 7966908 | LRRC8E | | | | | | |
| 19 | 7968664 | 7979368 | MAP2K7 | | | | | | |
| 19 | 7981031 | 7983980 | TGFBR3L | | | | | | |
| 19 | 7985193 | 7988136 | SNAPC2 | | | | | | |
| 19 | 7989380 | 7991051 | CTXN1 | | | | | | |
| 19 | 7991602 | 8008708 | TIMM44 | | | TIMM44 | | | |
| 19 | 8023456 | 8070529 | ELAVL1 | | | | | | |
| 19 | 8117645 | 8127547 | CCL25 | | | | | | |
| 19 | 8130286 | 8212385 | FBN3 | | | | | | |
| 19 | 8274216 | 8327304 | CERS4 | | | | | | |
| 19 | 8367010 | 8373240 | CD320 | | | | | | |
| 19 | 8376183 | 8386280 | NDUFA7 | | | | | | |
| 19 | 8386383 | 8387280 | RPS28 | | | | | | |
| 19 | 8387467 | 8408146 | KANK3 | | | | | | |
| 19 | 8429010 | 8439259 | ANGPTL4 | | | | | | |
| 19 | 8439259 | 8455575 | RAB11B | | | RAB11B | | | |
| 19 | 8454173 | 8454264 | MIR4999 | | | | | | |
| 19 | 8455204 | 8469317 | RAB11B | | | RAB11B | | | |
| 19 | 8478186 | 8503899 | MARCH2 | | | | | | |
| 19 | 8509711 | 8554002 | HNRNPM | | | | | | |
| 19 | 8554939 | 8567538 | PRAM1 | | | | | | |
| 19 | 8575461 | 8579048 | ZNF414 | | | | | | |
| 19 | 8585673 | 8642331 | MYO1F | | | | | | |
| 19 | 8645123 | 8675620 | ADAMTS10 | | | | | | |
| 19 | 8807746 | 8809180 | ACTL9 | | | | | | |
| 19 | 8841315 | 8842371 | OR2Z1 | | | | | | |
| 19 | 8920251 | 8942980 | ZNF558 | | | | | | |
| 19 | 8953268 | 8954016 | MBD3L1 | | | | | | |
| 19 | 8959519 | 9092018 | MUC16 | | | | | | |
| 19 | 9203920 | 9204862 | OR1M1 | | | | | | |
| 19 | 9212944 | 9213982 | OR7G2 | | | | | | |
| 19 | 9225503 | 9226439 | OR7G1 | | | | | | |
| 19 | 9236687 | 9237626 | OR7G3 | | | | | | |
| 19 | 9251055 | 9274091 | ZNF317 | | | | | | |
| 19 | 9296269 | 9299493 | OR7D2 | | | | | | |
| 19 | 9324525 | 9325547 | OR7D4 | | | | | | |
| 19 | 9361719 | 9362739 | OR7E24 | | | | | | |
| 19 | 9405985 | 9415795 | ZNF699 | | | | | | |
| 19 | 9434447 | 9454521 | ZNF559 | | | | | | |
| 19 | 9434901 | 9493293 | ZNF559-ZNF177 | | | | | | |
| 19 | 9473695 | 9493293 | ZNF177 | | | | | | |
| 19 | 9523101 | 9546254 | ZNF266 | | | | | | |
| 19 | 9577030 | 9609279 | ZNF560 | | | | | | |
| 19 | 9633899 | 9649321 | ZNF426 | | | | | | |
| 19 | 9649341 | 9650406 | LOC101928238 | | | | | | |
| 19 | 9676403 | 9695209 | ZNF121 | | | | | | |
| 19 | 9718001 | 9745538 | ZNF561 | | | | | | |
| 19 | 9759337 | 9785776 | ZNF562 | | | | | | |
| 19 | 9868150 | 9879410 | ZNF846 | | | | | | |
| 19 | 9920942 | 9929779 | FBXL12 | | | | | | |
| 19 | 9938555 | 9940797 | UBL5 | | | | | | |
| 19 | 9945882 | 9960365 | PIN1 | | PIN1 | | | | |
| 19 | 9964393 | 10047228 | OLFM2 | | | OLFM2 | | | |
| 19 | 10070236 | 10121147 | COL5A3 | | COL5A3 | | | | |
| 19 | 10123924 | 10132954 | RDH8 | | | | | | |
| 19 | 10149029 | 10149089 | MIR5589 | | | | | | |
| 19 | 10152031 | 10184813 | C3P1 | | | | | | |
| 19 | 10196794 | 10203928 | C19orf66 | | | | | | |
| 19 | 10203012 | 10213425 | ANGPTL6 | | | | | | |
| 19 | 10216898 | 10221975 | PPAN | | | | | | |
| 19 | 10216898 | 10226064 | PPAN-P2RY11 | | | | | | |
| 19 | 10218326 | 10218411 | SNORD105 | | | | | | |
| 19 | 10220424 | 10220516 | SNORD105B | | | | | | |

| | | | | | | | | |
|---|---|---|---|---|---|---|---|---|
| 19 | 10222196 | 10226064 | P2RY11 | | | | | |
| 19 | 10225689 | 10230599 | EIF3G | | | | | |
| 19 | 10244019 | 10305811 | DNMT1 | | | | | |
| 19 | 10332108 | 10341948 | S1PR2 | | | | | |
| 19 | 10341088 | 10341161 | MIR4322 | | | | | |
| 19 | 10362639 | 10370736 | MRPL4 | | | | | |
| 19 | 10381516 | 10397291 | ICAM1 | | | | | |
| 19 | 10397642 | 10399260 | ICAM4 | | | | | |
| 19 | 10400654 | 10407454 | ICAM5 | | | | | |
| 19 | 10415478 | 10420233 | ZGLP1 | | | | | |
| 19 | 10420886 | 10426691 | FDX1L | | | | | |
| 19 | 10426888 | 10444314 | RAVER1 | | | | | |
| 19 | 10444451 | 10450345 | ICAM3 | | | ICAM3 | | |
| 19 | 10461203 | 10491248 | TYK2 | | | | | |
| 19 | 10501808 | 10514271 | CDC37 | | | | | |
| 19 | 10514133 | 10514214 | MIR1181 | | | | | |
| 19 | 10527448 | 10580307 | PDE4A | | PDE4A | | | |
| 19 | 10596795 | 10614054 | KEAP1 | | | | | |
| 19 | 10623417 | 10628668 | S1PR5 | | | | | |
| 19 | 10654569 | 10664099 | ATG4D | | | | | |
| 19 | 10662797 | 10662880 | MIR1238 | | | | | |
| 19 | 10663760 | 10676702 | KRI1 | | | | | |
| 19 | 10677137 | 10679655 | CDKN2D | | | | | |
| 19 | 10683346 | 10697991 | AP1M2 | | | | | |
| 19 | 10713120 | 10755235 | SLC44A2 | | | | | |
| 19 | 10762537 | 10803095 | ILF3 | | | | | |
| 19 | 10812111 | 10824043 | QTRT1 | | | | | |
| 19 | 10828728 | 10942586 | DNM2 | | | DNM2 | | DNM2 |
| 19 | 10829079 | 10829179 | MIR638 | | | | | |
| 19 | 10890929 | 10891011 | MIR4748 | | | | | |
| 19 | 10928101 | 10928172 | MIR199A1 | | | | | |
| 19 | 10939648 | 10939711 | MIR6793 | | | | | |
| 19 | 10942742 | 10946983 | TMED1 | | | | | |
| 19 | 10959105 | 10980360 | C19orf38 | | | | | |
| 19 | 10982252 | 11033448 | CARM1 | | | | | |
| 19 | 11033443 | 11039688 | YIPF2 | | | | | |
| 19 | 11039423 | 11040916 | C19orf52 | | | | | |
| 19 | 11071597 | 11172958 | SMARCA4 | | | | | |
| 19 | 11200037 | 11244505 | LDLR | | | LDLR | | |
| 19 | 11224149 | 11224210 | MIR6886 | | | | | |
| 19 | 11256169 | 11266488 | SPC24 | | | | | |
| 19 | 11274942 | 11308243 | KANK2 | | | | | |
| 19 | 11309968 | 11373168 | DOCK6 | | | | | |
| 19 | 11314303 | 11326844 | LOC105372273 | | | | | |
| 19 | 11350294 | 11352619 | ANGPTL8 | | | | | |
| 19 | 11406814 | 11437672 | TSPAN16 | | | | | |
| 19 | 11432721 | 11450344 | RAB3D | | | RAB3D | | |
| 19 | 11453451 | 11456981 | TMEM205 | | | | | |
| 19 | 11457180 | 11465620 | CCDC159 | | | | | |
| 19 | 11466061 | 11476374 | PLPPR2 | | | | | |
| 19 | 11485382 | 11487627 | SWSAP1 | | | | | |
| 19 | 11487880 | 11495018 | EPOR | EPOR | | | | |
| 19 | 11504731 | 11530018 | RGL3 | | | | | |
| 19 | 11531271 | 11546603 | CCDC151 | | | | | |
| 19 | 11546077 | 11561782 | PRKCSH | | | | | |
| 19 | 11562142 | 11591803 | ELAVL3 | | | | | |
| 19 | 11594241 | 11616738 | ZNF653 | | | | | |
| 19 | 11606358 | 11606437 | MIR7974 | | | | | |
| 19 | 11616730 | 11639987 | ECSIT | | | | | |
| 19 | 11649531 | 11661138 | CNN1 | | | | | |
| 19 | 11663857 | 11670051 | ELOF1 | | | | | |
| 19 | 11685474 | 11689801 | ACP5 | | | | | |
| 19 | 11708234 | 11729974 | ZNF627 | | | | | |
| 19 | 11784812 | 11797384 | ZNF833P | | | | | |
| 19 | 11832079 | 11849824 | ZNF823 | | | | | |
| 19 | 11877814 | 11894893 | ZNF441 | | | | | |

| | | | | | | | | | |
|---|---|---|---|---|---|---|---|---|---|
| 19 | 11909390 | 11919306 | ZNF491 | | | | | | |
| 19 | 11925106 | 11946016 | ZNF440 | | | | | | |
| 19 | 11976843 | 11980306 | ZNF439 | | | | | | |
| 19 | 11998669 | 12025365 | ZNF69 | | | | | | |
| 19 | 12035882 | 12061588 | ZNF700 | | | | | | |
| 19 | 12075868 | 12091198 | ZNF763 | | | | | | |
| 19 | 12098431 | 12157090 | LOC101928464 | | | | | | |
| 19 | 12125531 | 12146556 | ZNF433 | | | | | | |
| 19 | 12154619 | 12163782 | ZNF878 | | | | | | |
| 19 | 12175513 | 12188631 | ZNF844 | | | | | | |
| 19 | 12203077 | 12225494 | ZNF788 | | | | | | |
| 19 | 12242167 | 12251222 | ZNF20 | | | | | | |
| 19 | 12242167 | 12267546 | ZNF625-ZNF20 | | | | | | |
| 19 | 12255708 | 12267546 | ZNF625 | | | | | | |
| 19 | 12273871 | 12300064 | ZNF136 | | | | | | |
| 19 | 12305829 | 12318391 | LOC100289333 | | | | | | |
| 19 | 12382624 | 12405714 | ZNF44 | | | | | | |
| 19 | 12428303 | 12444534 | ZNF563 | | | | | | |
| 19 | 12460184 | 12476475 | ZNF442 | | | | | | |
| 19 | 12500827 | 12512088 | ZNF799 | | | | | | |
| 19 | 12540519 | 12551926 | ZNF443 | | | | | | |
| 19 | 12571997 | 12595632 | ZNF709 | | | | | | |
| 19 | 12636183 | 12662356 | ZNF564 | | | | | | |
| 19 | 12686919 | 12721623 | ZNF490 | | | | | | |
| 19 | 12721731 | 12740676 | ZNF791 | | | | | | |
| 19 | 12757321 | 12777591 | MAN2B1 | MAN2B1 | | MAN2B1 | | | |
| 19 | 12777617 | 12786646 | WDR83 | | | | | | |
| 19 | 12778880 | 12780465 | WDR83OS | | | | | | |
| 19 | 12780516 | 12786646 | WDR83 | | | | | | |
| 19 | 12786530 | 12792701 | DHPS | | | | | | |
| 19 | 12798811 | 12799234 | LOC105372280 | | | | | | |
| 19 | 12799729 | 12807455 | FBXW9 | | | | | | |
| 19 | 12810007 | 12834810 | TNPO2 | | | | | | |
| 19 | 12814410 | 12814485 | SNORD135 | | | | | | |
| 19 | 12817262 | 12817332 | SNORD41 | | | | | | |
| 19 | 12841453 | 12845529 | C19orf43 | | | | | | |
| 19 | 12848305 | 12859137 | ASNA1 | | | | | | |
| 19 | 12863406 | 12869271 | BEST2 | | | | | | |
| 19 | 12873816 | 12886434 | HOOK2 | | | HOOK2 | | | |
| 19 | 12902309 | 12904125 | JUNB | | | | | | |
| 19 | 12907633 | 12912724 | PRDX2 | | | | | | |
| 19 | 12917427 | 12924462 | RNASEH2A | | | | | | |
| 19 | 12936290 | 12946242 | RTBDN | | | | | | |
| 19 | 12949258 | 12985766 | MAST1 | | | MAST1 | | | |
| 19 | 12963073 | 12963141 | MIR6794 | | | | | | |
| 19 | 12986024 | 12992335 | DNASE2 | | | | | | |
| 19 | 12995236 | 12998017 | KLF1 | | | | | | |
| 19 | 13001942 | 13010813 | GCDH | | | | | | |
| 19 | 13009893 | 13030086 | SYCE2 | | | | | | |
| 19 | 13033283 | 13044558 | FARSA | | | | | | |
| 19 | 13049413 | 13055304 | CALR | | | | | | |
| 19 | 13051297 | 13051354 | MIR6515 | | | | | | |
| 19 | 13056627 | 13064457 | RAD23A | | | | | | |
| 19 | 13064969 | 13068068 | GADD45GIP1 | | | | | | |
| 19 | 13080431 | 13085567 | DAND5 | | | | | | |
| 19 | 13106583 | 13209610 | NFIX | | | | | | |
| 19 | 13209841 | 13213974 | LYL1 | | | | | | |
| 19 | 13215713 | 13227563 | TRMT1 | | | | | | |
| 19 | 13229101 | 13251961 | NACC1 | | | | | | |
| 19 | 13254871 | 13261188 | STX10 | | | STX10 | | | |
| 19 | 13261281 | 13265718 | IER2 | | | | | | |
| 19 | 13317255 | 13617274 | CACNA1A | | CACNA1A | | CACNA1A | | |
| 19 | 13858752 | 13874106 | CCDC130 | | | | | | |
| 19 | 13875336 | 13885096 | MRI1 | | | | | | |
| 19 | 13885256 | 13889586 | C19orf53 | | | | | | |
| 19 | 13906273 | 13943044 | ZSWIM4 | | | | | | |

| | | | | | | | | |
|---|---|---|---|---|---|---|---|---|
| 19 | 13945329 | 13947103 | LOC284454 | | | | | |
| 19 | 13947100 | 13947173 | MIR24 | | | | | |
| 19 | 13947253 | 13947331 | MIR27A | | | | | |
| 19 | 13947400 | 13947473 | MIR23A | | | | | |
| 19 | 13985512 | 13985622 | MIR181C | | | | | |
| 19 | 13985688 | 13985825 | MIR181D | | | | | |
| 19 | 13988062 | 13991571 | NANOS3 | | | | | |
| 19 | 13993167 | 14016909 | C19orf57 | | | | | |
| 19 | 14016955 | 14041693 | CC2D1A | | | CC2D1A | | |
| 19 | 14041999 | 14064204 | PODNL1 | | | | | |
| 19 | 14063318 | 14072256 | DCAF15 | | | | | |
| 19 | 14072341 | 14117134 | RFX1 | | | | | |
| 19 | 14138959 | 14142370 | RLN3 | | | | | |
| 19 | 14142551 | 14163717 | IL27RA | | | | | |
| 19 | 14164178 | 14169971 | PALM3 | | | | | |
| 19 | 14183820 | 14185874 | LOC113230 | | | | | |
| 19 | 14184172 | 14184291 | MIR1199 | | | | | |
| 19 | 14192443 | 14196607 | C19orf67 | | | | | |
| 19 | 14199228 | 14201232 | SAMD1 | | | | | |
| 19 | 14202506 | 14228559 | PRKACA | | | | | PRKACA |
| 19 | 14230320 | 14247440 | ASF1B | | | | | |
| 19 | 14247963 | 14282075 | LOC100507373 | | | | | |
| 19 | 14258548 | 14316997 | ADGRL1 | | | | | |
| 19 | 14416269 | 14474802 | LOC105372288 | | | | | |
| 19 | 14444554 | 14454726 | LOC101928845 | | | | | |
| 19 | 14491955 | 14519537 | ADGRE5 | | | | | |
| 19 | 14519609 | 14530195 | DDX39A | | | | | |
| 19 | 14544165 | 14582679 | PKN1 | | | | | |
| 19 | 14583277 | 14586174 | PTGER1 | | | | | |
| 19 | 14588570 | 14606961 | GIPC1 | | | | | |
| 19 | 14625575 | 14640134 | DNAJB1 | | | | | |
| 19 | 14640354 | 14640452 | MIR639 | | | | | |
| 19 | 14640378 | 14676792 | TECR | | | | | |
| 19 | 14676889 | 14682889 | NDUFB7 | | | | | |
| 19 | 14693895 | 14721956 | CLEC17A | | | | | |
| 19 | 14729928 | 14785730 | ADGRE3 | | | | | |
| 19 | 14733024 | 14733163 | SNORA104 | | | | | |
| 19 | 14800851 | 14831772 | ZNF333 | | | | | |
| 19 | 14843508 | 14889353 | ADGRE2 | | | | | |
| 19 | 14909985 | 14910948 | OR7C1 | | | | | |
| 19 | 14937138 | 14939276 | OR7A5 | | | | | |
| 19 | 14951759 | 14952689 | OR7A10 | | | | | |
| 19 | 14991237 | 14992167 | OR7A17 | | | | | |
| 19 | 15052300 | 15053260 | OR7C2 | | | | | |
| 19 | 15060844 | 15090547 | SLC1A6 | | | SLC1A6 | | |
| 19 | 15121538 | 15134083 | CCDC105 | | | | | |
| 19 | 15160290 | 15169103 | CASP14 | | | | | |
| 19 | 15197876 | 15198944 | OR1I1 | | | | | |
| 19 | 15218174 | 15225799 | SYDE1 | | | | | |
| 19 | 15225784 | 15236610 | ILVBL | | | | | |
| 19 | 15270443 | 15311792 | NOTCH3 | | | | | |
| 19 | 15290093 | 15290161 | MIR6795 | | | | | |
| 19 | 15337729 | 15343858 | EPHX3 | | | | | |
| 19 | 15348300 | 15391262 | BRD4 | | | | | |
| 19 | 15464331 | 15490612 | AKAP8 | | | | | |
| 19 | 15490858 | 15529932 | AKAP8L | | | AKAP8L | | |
| 19 | 15532317 | 15560762 | WIZ | | | | | |
| 19 | 15560358 | 15560419 | MIR1470 | | | | | |
| 19 | 15562437 | 15575382 | RASAL3 | | | | | |
| 19 | 15579456 | 15590315 | PGLYRP2 | | | | | |
| 19 | 15619335 | 15663128 | CYP4F22 | | | | | |
| 19 | 15726028 | 15740447 | CYP4F8 | | | | | |
| 19 | 15751706 | 15771570 | CYP4F3 | | | | | |
| 19 | 15783827 | 15807984 | CYP4F12 | | | | | |
| 19 | 15838833 | 15839862 | OR10H2 | | | OR10H2 | | |
| 19 | 15852202 | 15853153 | OR10H3 | | | | | |

| | | | | | | | | |
|---|---|---|---|---|---|---|---|---|
| 19 | 15870143 | 15890798 | CYP4F24P | | | | | |
| 19 | 15904858 | 15905806 | OR10H5 | | | | | |
| 19 | 15917816 | 15918936 | OR10H1 | | | | | |
| 19 | 15939756 | 15947131 | UCA1 | | | | | |
| 19 | 15962803 | 15975714 | LOC102724279 | | | | | |
| 19 | 15988833 | 16008930 | CYP4F2 | | | | | |
| 19 | 16023179 | 16045676 | CYP4F11 | | | | | |
| 19 | 16059817 | 16060768 | OR10H4 | | | | | |
| 19 | 16126443 | 16138272 | LINC00661 | | | | | |
| 19 | 16144522 | 16152945 | LINC00905 | | | | | |
| 19 | 16178316 | 16213813 | TPM4 | | | | | |
| 19 | 16222489 | 16244445 | RAB8A | | | RAB8A | | |
| 19 | 16244837 | 16269384 | HSH2D | | | | | |
| 19 | 16272178 | 16284336 | CIB3 | | | | | |
| 19 | 16296211 | 16302857 | FAM32A | | | | | |
| 19 | 16308664 | 16346156 | AP1M1 | | | | | |
| 19 | 16435650 | 16438339 | KLF2 | | | | | |
| 19 | 16466054 | 16582823 | EPS15L1 | | | EPS15L1 | | |
| 19 | 16589867 | 16607003 | CALR3 | | | | | |
| 19 | 16607121 | 16632180 | C19orf44 | | | | | |
| 19 | 16628699 | 16653263 | CHERP | | | | | |
| 19 | 16660647 | 16683193 | SLC35E1 | | | | | |
| 19 | 16685717 | 16739015 | MED26 | | | | | |
| 19 | 16756958 | 16770968 | SMIM7 | | | | | |
| 19 | 16771937 | 16799816 | TMEM38A | | | | | |
| 19 | 16830786 | 16928774 | NWD1 | | | | | |
| 19 | 16940197 | 16991164 | SIN3B | | | | | |
| 19 | 16999825 | 17002830 | F2RL3 | | | | | |
| 19 | 17003761 | 17137625 | CPAMD8 | | | | | |
| 19 | 17160570 | 17186343 | HAUS8 | | | | | |
| 19 | 17186590 | 17324104 | MYO9B | | | | | |
| 19 | 17326154 | 17330638 | USE1 | | | USE1 | | |
| 19 | 17337012 | 17340028 | OCEL1 | | | | | |
| 19 | 17342693 | 17356151 | NR2F6 | | | NR2F6 | | |
| 19 | 17360829 | 17375544 | USHBP1 | | | | | |
| 19 | 17378184 | 17390162 | BABAM1 | | | | | |
| 19 | 17392453 | 17398455 | ANKLE1 | | | | | |
| 19 | 17402939 | 17414282 | ABHD8 | | | | | |
| 19 | 17416476 | 17417652 | MRPL34 | | | | | |
| 19 | 17420336 | 17434106 | DDA1 | | | | | |
| 19 | 17434031 | 17445638 | ANO8 | | | | | |
| 19 | 17445790 | 17453540 | GTPBP3 | | | | | |
| 19 | 17462256 | 17488158 | PLVAP | | | | | |
| 19 | 17513747 | 17516457 | BST2 | | | | | |
| 19 | 17516494 | 17526545 | BISPR | | | | | |
| 19 | 17516494 | 17536148 | MVB12A | | | | | |
| 19 | 17546317 | 17559376 | TMEM221 | | | | | |
| 19 | 17566233 | 17571725 | NXNL1 | | | | | |
| 19 | 17581252 | 17616977 | SLC27A1 | | | | | |
| 19 | 17622431 | 17632097 | PGLS | | | | | |
| 19 | 17634109 | 17664648 | FAM129C | | | | | |
| 19 | 17666402 | 17693971 | COLGALT1 | | | | | |
| 19 | 17712136 | 17799008 | UNC13A | | | | | |
| 19 | 17830260 | 17845324 | MAP1S | | | | | |
| 19 | 17858526 | 17899377 | FCHO1 | | | | | |
| 19 | 17905918 | 17924385 | B3GNT3 | | | | | |
| 19 | 17927321 | 17932383 | INSL3 | | | | | |
| 19 | 17935592 | 17958841 | JAK3 | | | JAK3 | | |
| 19 | 17970686 | 17974133 | RPL18A | | | | | |
| 19 | 17973396 | 17973529 | SNORA68 | | | | | |
| 19 | 17982781 | 18005983 | SLC5A5 | | | | | |
| 19 | 18043823 | 18054794 | CCDC124 | | | | | |
| 19 | 18062110 | 18109930 | KCNN1 | | KCNN1 | | | |
| 19 | 18111940 | 18124911 | ARRDC2 | | | | | |
| 19 | 18169804 | 18197813 | IL12RB1 | | | IL12RB1 | | |
| 19 | 18208602 | 18262499 | MAST3 | | | | | |

| | | | | | | | | |
|---|---|---|---|---|---|---|---|---|
| 19 | 18263987 | 18281343 | PIK3R2 | | | | | |
| 19 | 18284589 | 18288934 | IFI30 | | | | | |
| 19 | 18304039 | 18307550 | MPV17L2 | | | | | |
| 19 | 18307610 | 18314874 | RAB3A | | | RAB3A | | |
| 19 | 18315539 | 18316373 | LOC102725254 | | | | | |
| 19 | 18318770 | 18359010 | PDE4C | | | | | |
| 19 | 18360759 | 18366229 | LOC729966 | | | | | |
| 19 | 18367905 | 18385319 | KIAA1683 | | | | | |
| 19 | 18390503 | 18392466 | JUND | | | | | |
| 19 | 18392886 | 18392971 | MIR3188 | | | | | |
| 19 | 18417039 | 18434001 | LSM4 | | | | | |
| 19 | 18451396 | 18480763 | PGPEP1 | | | | | |
| 19 | 18496967 | 18499986 | GDF15 | | | | | |
| 19 | 18497371 | 18497444 | MIR3189 | | | | | |
| 19 | 18501953 | 18508415 | LRRC25 | | | | | |
| 19 | 18530145 | 18545372 | SSBP4 | | | | | |
| 19 | 18545197 | 18549111 | ISYNA1 | | | | | |
| 19 | 18553472 | 18632937 | ELL | | | | | |
| 19 | 18642561 | 18654406 | FKBP8 | | | | | |
| 19 | 18668571 | 18680197 | KXD1 | | | | | |
| 19 | 18682613 | 18688270 | UBA52 | | | | | |
| 19 | 18699494 | 18703147 | C19orf60 | | | | | |
| 19 | 18704034 | 18717660 | CRLF1 | | | | | |
| 19 | 18723681 | 18731849 | TMEM59L | | | | | |
| 19 | 18747837 | 18781302 | KLHL26 | | | | | |
| 19 | 18794424 | 18893143 | CRTC1 | | | CRTC1 | | |
| 19 | 18893582 | 18902114 | COMP | | | COMP | | |
| 19 | 18942743 | 18979041 | UPF1 | | | | | |
| 19 | 18979354 | 19006953 | CERS1 | | CERS1 | | | |
| 19 | 18979354 | 19006953 | GDF1 | | GDF1 | | | |
| 19 | 18988717 | 19007536 | CERS1 | | CERS1 | | | |
| 19 | 19010322 | 19030199 | COPE | | | | | |
| 19 | 19030483 | 19039442 | DDX49 | | | | | |
| 19 | 19040009 | 19052041 | HOMER3 | | | | HOMER3 | |
| 19 | 19051062 | 19057640 | LOC102724360 | | | | | |
| 19 | 19101696 | 19144380 | SUGP2 | | | | | |
| 19 | 19144386 | 19168987 | ARMC6 | | | | | |
| 19 | 19174802 | 19223841 | SLC25A42 | | | | | |
| 19 | 19230424 | 19249310 | TMEM161A | | | | | |
| 19 | 19256375 | 19281098 | MEF2B | | | | | |
| 19 | 19256375 | 19303400 | MEF2BNB-MEF2B | | | | | |
| 19 | 19287712 | 19303400 | BORCS8 | | | | | |
| 19 | 19303007 | 19312678 | RFXANK | | | | | |
| 19 | 19312219 | 19314238 | NR2C2AP | | | | | |
| 19 | 19322781 | 19363061 | NCAN | | | | | |
| 19 | 19366451 | 19373596 | HAPLN4 | | | | | |
| 19 | 19375173 | 19384074 | TM6SF2 | | | | | |
| 19 | 19387321 | 19431321 | SUGP1 | | | | | |
| 19 | 19431629 | 19469563 | MAU2 | | | | | |
| 19 | 19496641 | 19619741 | GATAD2A | | | | | |
| 19 | 19545871 | 19545967 | MIR640 | | | | | |
| 19 | 19576148 | 19619741 | GATAD2A | | | | | |
| 19 | 19625027 | 19626469 | TSSK6 | | | | | |
| 19 | 19627018 | 19639013 | NDUFA13 | | | | | |
| 19 | 19639669 | 19648393 | YJEFN3 | | | | | |
| 19 | 19649073 | 19657468 | CILP2 | | | | | |
| 19 | 19672515 | 19729725 | PBX4 | | | | | |
| 19 | 19734465 | 19739039 | LPAR2 | | | LPAR2 | | |
| 19 | 19740281 | 19754476 | GMIP | | | | | |
| 19 | 19756009 | 19774503 | ATP13A1 | | | | | |
| 19 | 19778961 | 19794315 | ZNF101 | | | | | |
| 19 | 19821280 | 19843921 | ZNF14 | | | | | |
| 19 | 19867180 | 19887222 | LINC00663 | | | | | |
| 19 | 19903519 | 19932560 | ZNF506 | | | | | |
| 19 | 19976713 | 20004293 | ZNF253 | | | | | |
| 19 | 20011721 | 20046382 | ZNF93 | | | | | |

| | | | | | | | | |
|---|---|---|---|---|---|---|---|---|
| 19 | 20115226 | 20150277 | ZNF682 | | | | | |
| 19 | 20188802 | 20231977 | ZNF90 | | | | | |
| 19 | 20278022 | 20311299 | ZNF486 | | | | | |
| 19 | 20510080 | 20510163 | MIR1270 | | | | | |
| 19 | 20578625 | 20607771 | ZNF826P | | | | | |
| 19 | 20579239 | 20579322 | MIR1270 | | | | | |
| 19 | 20720797 | 20748626 | ZNF737 | | | | | |
| 19 | 20802744 | 20844402 | ZNF626 | | | | | |
| 19 | 21106058 | 21133503 | ZNF85 | | | | | |
| 19 | 21203425 | 21242852 | ZNF430 | | | | | |
| 19 | 21264952 | 21307883 | ZNF714 | | | | | |
| 19 | 21324812 | 21368805 | ZNF431 | | | | | |
| 19 | 21473961 | 21512212 | ZNF708 | | | | | |
| 19 | 21541734 | 21571384 | ZNF738 | | | | | |
| 19 | 21579920 | 21610296 | ZNF493 | | | | | |
| 19 | 21666516 | 21686040 | LINC00664 | | | | | |
| 19 | 21688436 | 21721079 | ZNF429 | | | | | |
| 19 | 21906842 | 21950430 | ZNF100 | | | | | |
| 19 | 21933546 | 21936240 | LOC641367 | | | | | |
| 19 | 21987750 | 22034870 | ZNF43 | | | | | |
| 19 | 22148896 | 22193745 | ZNF208 | | | | | |
| 19 | 22235249 | 22273903 | ZNF257 | | | | | |
| 19 | 22361902 | 22379753 | ZNF676 | | | | | |
| 19 | 22469251 | 22499978 | ZNF729 | | | | | |
| 19 | 22573898 | 22605148 | ZNF98 | | | | | |
| 19 | 22703796 | 22710751 | LOC101929124 | | | | | |
| 19 | 22715427 | 22716296 | LINC01233 | | | | | |
| 19 | 22779058 | 22786352 | GOLGA2P9 | | | | | |
| 19 | 22790817 | 22791303 | LOC100996349 | | | | | |
| 19 | 22817125 | 22850472 | ZNF492 | | | | | |
| 19 | 22934984 | 22966973 | ZNF99 | | | | | |
| 19 | 23157684 | 23186010 | ZNF728 | | | | | |
| 19 | 23197876 | 23206499 | LOC101929164 | | | | | |
| 19 | 23237624 | 23244067 | LOC101929144 | | | | | |
| 19 | 23299776 | 23330014 | ZNF730 | | | | | |
| 19 | 23406606 | 23433172 | ZNF724P | | | | | |
| 19 | 23437855 | 23457053 | IPO5P1 | | | | | |
| 19 | 23540497 | 23578362 | ZNF91 | | | | | |
| 19 | 23582035 | 23598876 | LINC01224 | | | | | |
| 19 | 23835707 | 23870017 | ZNF675 | | | | | |
| 19 | 23921996 | 23941693 | ZNF681 | | | | | |
| 19 | 23945815 | 24010919 | RPSAP58 | | | | | |
| 19 | 24097683 | 24116769 | ZNF726 | | | | | |
| 19 | 24216206 | 24312769 | ZNF254 | | | | | |
| 19 | 24344994 | 24346249 | HAVCR1P1 | | | | | |
| 19 | 28281400 | 28284848 | LINC00662 | | | | | |
| 19 | 28284374 | 28297688 | LOC101927151 | | | | | |
| 19 | 28926295 | 29218601 | LOC100420587 | | | | | |
| 19 | 29456037 | 29460055 | LINC00906 | | | | | |
| 19 | 29459374 | 29461579 | LOC102724958 | | | | | |
| 19 | 29493461 | 29504862 | LINC01532 | | | | | |
| 19 | 29698166 | 29704136 | UQCRFS1 | | | UQCRFS1 | | |
| 19 | 29777917 | 30016659 | LOC284395 | | | | | |
| 19 | 30017490 | 30055226 | VSTM2B | | | | | |
| 19 | 30097169 | 30108162 | POP4 | | | | | |
| 19 | 30156326 | 30166383 | PLEKHF1 | | | | | |
| 19 | 30189792 | 30206696 | C19orf12 | | | | | |
| 19 | 30302900 | 30315215 | CCNE1 | | | CCNE1 | | |
| 19 | 30414550 | 30507519 | URI1 | | | | | |
| 19 | 30863299 | 31048966 | ZNF536 | | | | | |
| 19 | 31765850 | 31840190 | TSHZ3 | | | | | |
| 19 | 32079094 | 32084456 | THEG5 | | | | | |
| 19 | 32516767 | 32539805 | LINC01533 | | | | | |
| 19 | 32592997 | 32596822 | LOC101927411 | | | | | |
| 19 | 32836513 | 32878573 | ZNF507 | | | | | |
| 19 | 32880955 | 32896445 | LOC400684 | | | | | |

| | | | | | | | | |
|---|---|---|---|---|---|---|---|---|
| 19 | 32896654 | 32976799 | DPY19L3 | | | | | |
| 19 | 33072093 | 33078358 | PDCD5 | | | | | |
| 19 | 33087906 | 33166102 | ANKRD27 | | | | | |
| 19 | 33166312 | 33169206 | RGS9BP | | | | | |
| 19 | 33182866 | 33204702 | NUDT19 | | | | | |
| 19 | 33210678 | 33281714 | TDRD12 | | | | | |
| 19 | 33321418 | 33360683 | SLC7A9 | | | | | |
| 19 | 33369903 | 33462935 | CEP89 | | | | | |
| 19 | 33463122 | 33468401 | FAAP24 | | | | | |
| 19 | 33469497 | 33555824 | RHPN2 | | | | | |
| 19 | 33571785 | 33621318 | GPATCH1 | | | | | |
| 19 | 33622997 | 33666703 | WDR88 | | | | | |
| 19 | 33685598 | 33699773 | LRP3 | | | | | |
| 19 | 33699569 | 33716756 | SLC7A10 | | | | | |
| 19 | 33790839 | 33795963 | CEBPA | | | | | |
| 19 | 33864574 | 33873592 | CEBPG | | | | | |
| 19 | 33877854 | 34012799 | PEPD | | | | | |
| 19 | 34112860 | 34264414 | CHST8 | | CHST8 | | | |
| 19 | 34287750 | 34306666 | KCTD15 | | | | | |
| 19 | 34663351 | 34720420 | LSM14A | | | | | |
| 19 | 34745455 | 34846471 | KIAA0355 | | | | | |
| 19 | 34855644 | 34893318 | GPI | | | | | |
| 19 | 34895302 | 34917072 | PDCD2L | | | | | |
| 19 | 34919267 | 34960798 | UBA2 | | | | | |
| 19 | 34972879 | 34992085 | WTIP | | | | | |
| 19 | 35067637 | 35068596 | SCGB1B2P | | | | | |
| 19 | 35084345 | 35085490 | SCGB2B2 | | | | | |
| 19 | 35136777 | 35136957 | SCGB2B3P | | | | | |
| 19 | 35168543 | 35177302 | ZNF302 | | | | | |
| 19 | 35225479 | 35233774 | ZNF181 | | | | | |
| 19 | 35248978 | 35264134 | ZNF599 | | | | | |
| 19 | 35307057 | 35323773 | LOC400685 | | | | | |
| 19 | 35382932 | 35389847 | LINC00904 | | | | | |
| 19 | 35413918 | 35436076 | ZNF30 | | | | | |
| 19 | 35447257 | 35454953 | ZNF792 | | | | | |
| 19 | 35485687 | 35517375 | GRAMD1A | | | | | |
| 19 | 35521591 | 35531353 | SCN1B | | SCN1B | | SCN1B | |
| 19 | 35531409 | 35597175 | HPN | | | | | |
| 19 | 35606731 | 35615228 | FXYD3 | | | | | |
| 19 | 35613603 | 35613668 | MIR6887 | | | | | |
| 19 | 35615416 | 35626178 | LGI4 | | | | | |
| 19 | 35629692 | 35633959 | FXYD1 | | | | | |
| 19 | 35634153 | 35645205 | FXYD7 | | | | | |
| 19 | 35645624 | 35660788 | FXYD5 | | | | | |
| 19 | 35715703 | 35719628 | FAM187B | | | | | |
| 19 | 35739558 | 35758867 | LSR | | | | | |
| 19 | 35759895 | 35770718 | USF2 | | | | | |
| 19 | 35773248 | 35776045 | HAMP | | HAMP | | | |
| 19 | 35782988 | 35804709 | MAG | | MAG | | | |
| 19 | 35820068 | 35838264 | CD22 | | | | | |
| 19 | 35836415 | 35836530 | MIR5196 | | | | | |
| 19 | 35842444 | 35843367 | FFAR1 | | | | | |
| 19 | 35849487 | 35851389 | FFAR3 | | | | | |
| 19 | 35896508 | 35907742 | LINC01531 | | | | | |
| 19 | 35940616 | 35942669 | FFAR2 | | | | | |
| 19 | 35978225 | 35981433 | KRTDAP | | | | | |
| 19 | 35988118 | 36004560 | DMKN | | | | | |
| 19 | 36014268 | 36019253 | SBSN | | | | | |
| 19 | 36024313 | 36036221 | GAPDHS | | | | | |
| 19 | 36031639 | 36038429 | TMEM147 | | | | | |
| 19 | 36041094 | 36054560 | ATP4A | | | | | |
| 19 | 36088796 | 36092403 | LOC102723617 | | | | | |
| 19 | 36103645 | 36116251 | HAUS5 | | | | | |
| 19 | 36119917 | 36128588 | RBM42 | | | | | |
| 19 | 36132638 | 36135773 | ETV2 | | | | | |
| 19 | 36139124 | 36149686 | COX6B1 | | | | | |

| | | | | | | | | |
|---|---|---|---|---|---|---|---|---|
| 19 | 36157417 | 36164193 | UPK1A | | | | | |
| 19 | 36195388 | 36207940 | ZBTB32 | | | | | |
| 19 | 36208920 | 36229781 | KMT2B | | | | | |
| 19 | 36230150 | 36233351 | IGFLR1 | | | | | |
| 19 | 36233427 | 36236343 | U2AF1L4 | | | | | |
| 19 | 36236477 | 36238056 | PSENEN | | | | | |
| 19 | 36239261 | 36245420 | LIN37 | | | | | |
| 19 | 36245466 | 36247930 | HSPB6 | | | | | |
| 19 | 36249043 | 36260077 | PROSER3 | | | | | |
| 19 | 36266416 | 36279724 | ARHGAP33 | | | | | |
| 19 | 36279777 | 36288811 | LINC01529 | | | | | |
| 19 | 36290891 | 36304201 | PRODH2 | | | | | |
| 19 | 36316273 | 36342895 | NPHS1 | | | NPHS1 | | |
| 19 | 36347809 | 36358048 | KIRREL2 | | | | | |
| 19 | 36359400 | 36370699 | APLP1 | | | APLP1 | | |
| 19 | 36379142 | 36391552 | NFKBID | | | | | |
| 19 | 36393381 | 36395173 | HCST | | | | | |
| 19 | 36395302 | 36399211 | TYROBP | | | | | |
| 19 | 36428021 | 36436097 | LRFN3 | | | | | |
| 19 | 36486089 | 36487220 | SDHAF1 | | | | | |
| 19 | 36494200 | 36499695 | SYNE4 | | | | | |
| 19 | 36500017 | 36505141 | ALKBH6 | | | | | |
| 19 | 36505400 | 36536899 | LOC101927572 | | | | | |
| 19 | 36505561 | 36523797 | CLIP3 | | | | | |
| 19 | 36525886 | 36545664 | THAP8 | | | | | |
| 19 | 36545782 | 36596012 | WDR62 | | | | | |
| 19 | 36602104 | 36604613 | OVOL3 | | | | | |
| 19 | 36604610 | 36606206 | POLR2I | | | | | |
| 19 | 36605887 | 36616850 | TBCB | | | TBCB | | |
| 19 | 36630827 | 36641255 | CAPNS1 | | | | | |
| 19 | 36641823 | 36643771 | COX7A1 | | | | | |
| 19 | 36672961 | 36705986 | ZNF565 | | | | | |
| 19 | 36705503 | 36729675 | ZNF146 | | | | | |
| 19 | 36802244 | 36803570 | LOC100134317 | | | | | |
| 19 | 36803962 | 36822620 | LINC00665 | | | | | |
| 19 | 36825354 | 36870105 | ZFP14 | | | | | |
| 19 | 36882860 | 36909550 | ZFP82 | | | | | |
| 19 | 36912075 | 36913799 | LOC644189 | | | | | |
| 19 | 36936020 | 36980804 | ZNF566 | | | | | |
| 19 | 36980528 | 36981942 | LOC728752 | | | | | |
| 19 | 37001588 | 37019248 | ZNF260 | | | | | |
| 19 | 37034516 | 37096178 | ZNF529 | | | | | |
| 19 | 37096206 | 37119499 | ZNF382 | | | | | |
| 19 | 37128282 | 37157755 | ZNF461 | | | | | |
| 19 | 37176340 | 37178351 | LINC01534 | | | | | |
| 19 | 37178513 | 37212238 | ZNF567 | | | | | |
| 19 | 37234379 | 37263716 | ZNF850 | | | | | |
| 19 | 37264019 | 37267980 | LOC728485 | | | | | |
| 19 | 37288451 | 37341689 | ZNF790 | | | | | |
| 19 | 37341259 | 37370477 | ZNF345 | | | | | |
| 19 | 37379025 | 37407193 | ZNF829 | | | | | |
| 19 | 37407230 | 37488834 | ZNF568 | | | | | |
| 19 | 37569381 | 37620651 | ZNF420 | | | | | |
| 19 | 37638339 | 37663643 | ZNF585A | | | | | |
| 19 | 37672480 | 37701451 | ZNF585B | | | | | |
| 19 | 37717365 | 37734574 | ZNF383 | | | | | |
| 19 | 37742786 | 37756437 | LINC01535 | | | | | |
| 19 | 37756840 | 37759912 | LOC284412 | | | | | |
| 19 | 37825579 | 37855357 | HKR1 | | | | | |
| 19 | 37862058 | 37883966 | ZNF527 | | | | | |
| 19 | 37902059 | 37958339 | ZNF569 | | | | | |
| 19 | 37958673 | 37976260 | ZNF570 | | | | | |
| 19 | 37988062 | 38034239 | ZNF793 | | | | | |
| 19 | 38039850 | 38078248 | ZNF571 | | | | | |
| 19 | 38042272 | 38105079 | ZNF540 | | | | | |
| 19 | 38055154 | 38085693 | ZNF571 | | | | | |

| | | | | | | | | |
|---|---|---|---|---|---|---|---|---|
| 19 | 38085730 | 38105079 | ZNF540 | | | | | |
| 19 | 38123388 | 38146313 | ZFP30 | | | | | |
| 19 | 38158649 | 38183216 | ZNF781 | | | | | |
| 19 | 38187263 | 38210691 | ZNF607 | | | | | |
| 19 | 38229202 | 38270230 | ZNF573 | | | | | |
| 19 | 38308050 | 38317278 | LOC644554 | | | | | |
| 19 | 38314362 | 38345836 | LOC100631378 | | | | | |
| 19 | 38375462 | 38397346 | WDR87 | | | | | |
| 19 | 38397860 | 38699012 | SIPA1L3 | | | | | |
| 19 | 38701645 | 38720354 | DPF1 | | | | | |
| 19 | 38741876 | 38747231 | PPP1R14A | | | | | |
| 19 | 38755097 | 38783254 | SPINT2 | | | | | |
| 19 | 38794199 | 38806606 | YIF1B | | | | | |
| 19 | 38794800 | 38795646 | C19orf33 | | | | | |
| 19 | 38810483 | 38819649 | KCNK6 | | | | | |
| 19 | 38826442 | 38861589 | CATSPERG | | | | | |
| 19 | 38865189 | 38874464 | PSMD8 | | | | | |
| 19 | 38874991 | 38878668 | GGN | | | | | |
| 19 | 38880839 | 38890523 | SPRED3 | | | | | |
| 19 | 38893774 | 38899728 | FAM98C | | | | | |
| 19 | 38899697 | 38916945 | RASGRP4 | | | | | |
| 19 | 38924339 | 39078204 | RYR1 | | RYR1 | RYR1 | | |
| 19 | 39078279 | 39108675 | MAP4K1 | | | | | |
| 19 | 39086986 | 39092334 | LOC105372397 | | | | | |
| 19 | 39109711 | 39127599 | EIF3K | | | | | |
| 19 | 39138266 | 39221171 | ACTN4 | | | | | |
| 19 | 39220831 | 39235114 | CAPN12 | | | | | |
| 19 | 39261607 | 39264157 | LGALS7 | | | LGALS7 | | |
| 19 | 39279849 | 39282394 | LGALS7B | | | | | |
| 19 | 39292310 | 39303740 | LGALS4 | | | | | |
| 19 | 39306061 | 39322497 | ECH1 | | | | | |
| 19 | 39327027 | 39342979 | HNRNPL | | | | | |
| 19 | 39358471 | 39368919 | RINL | | | | | |
| 19 | 39369194 | 39390502 | SIRT2 | | | | | |
| 19 | 39390339 | 39399534 | NFKBIB | | | | | |
| 19 | 39399619 | 39402798 | CCER2 | | | | | |
| 19 | 39405903 | 39421536 | SARS2 | | | | | |
| 19 | 39421347 | 39423659 | MRPS12 | | | | | |
| 19 | 39432040 | 39466453 | FBXO17 | | | | | |
| 19 | 39514662 | 39523236 | FBXO27 | | | | | |
| 19 | 39574944 | 39602128 | ACP7 | | | | | |
| 19 | 39616419 | 39670046 | PAK4 | | | | | |
| 19 | 39687603 | 39692522 | NCCRP1 | | | | | |
| 19 | 39693561 | 39694906 | SYCN | | | | | |
| 19 | 39734271 | 39735611 | IFNL3 | | | | | |
| 19 | 39736953 | 39739496 | IFNL4 | | | | | |
| 19 | 39759156 | 39760732 | IFNL2 | | | | | |
| 19 | 39786964 | 39789312 | IFNL1 | | | | | |
| 19 | 39797456 | 39805976 | LRFN1 | | | | | |
| 19 | 39818996 | 39826726 | GMFG | | | | | |
| 19 | 39833098 | 39875537 | SAMD4B | | | | | |
| 19 | 39876269 | 39881835 | PAF1 | | | | | |
| 19 | 39881942 | 39891277 | MED29 | | | | | |
| 19 | 39897486 | 39900052 | ZFP36 | | | | | |
| 19 | 39900262 | 39900318 | MIR4530 | | | | | |
| 19 | 39903749 | 39919055 | PLEKHG2 | | | | | |
| 19 | 39923846 | 39926618 | RPS16 | | | | | |
| 19 | 39936185 | 39967308 | SUPT5H | | | | | |
| 19 | 39971051 | 39983659 | TIMM50 | | | | | |
| 19 | 39989556 | 39999121 | DLL3 | | DLL3 | | | |
| 19 | 40005752 | 40011326 | SELV | | | | | |
| 19 | 40022172 | 40023507 | EID2B | | | | | |
| 19 | 40029446 | 40030838 | EID2 | | | | | |
| 19 | 40093168 | 40098114 | LGALS13 | | | | | |
| 19 | 40129324 | 40133041 | LOC100129935 | | | | | |
| 19 | 40146533 | 40151287 | LGALS16 | | | | | |

| | | | | | | | | |
|---|---|---|---|---|---|---|---|---|
| 19 | 40170013 | 40177013 | LGALS17A | | | | | |
| 19 | 40194945 | 40200088 | LGALS14 | | | | | |
| 19 | 40221892 | 40228669 | CLC | | | | | |
| 19 | 40267233 | 40276775 | LEUTX | | | | | |
| 19 | 40315986 | 40324873 | DYRK1B | | | | | |
| 19 | 40320355 | 40320442 | MIR6719 | | | | | |
| 19 | 40325092 | 40337054 | FBL | | | | | |
| 19 | 40353962 | 40440533 | FCGBP | | | | | |
| 19 | 40476911 | 40487671 | PSMC4 | | | | | |
| 19 | 40502942 | 40526948 | ZNF546 | | | | | |
| 19 | 40534166 | 40562115 | ZNF780B | | | | | |
| 19 | 40575058 | 40596845 | ZNF780A | | | | | |
| 19 | 40697650 | 40721482 | MAP3K10 | | | | | |
| 19 | 40721964 | 40724306 | TTC9B | | | | | |
| 19 | 40728114 | 40732597 | CNTD2 | | | | | |
| 19 | 40736223 | 40791302 | AKT2 | | | AKT2 | | |
| 19 | 40788449 | 40788548 | MIR641 | | | | | |
| 19 | 40826966 | 40854434 | C19orf47 | | | | | |
| 19 | 40854331 | 40884397 | PLD3 | | PLD3 | | | |
| 19 | 40875752 | 40875814 | MIR6796 | | | | | |
| 19 | 40885177 | 40896128 | HIPK4 | | | | | |
| 19 | 40899670 | 40919271 | PRX | | | PRX | | |
| 19 | 40928408 | 40931932 | SERTAD1 | | | | | |
| 19 | 40946747 | 40950282 | SERTAD3 | | | | | |
| 19 | 40953690 | 40971725 | BLVRB | | | | | |
| 19 | 40973125 | 41066658 | SPTBN4 | | SPTBN4 | SPTBN4 | | |
| 19 | 41082756 | 41097305 | SHKBP1 | | | | | |
| 19 | 41099071 | 41135725 | LTBP4 | | | | | |
| 19 | 41171811 | 41196563 | NUMBL | | | | | |
| 19 | 41197433 | 41222790 | ADCK4 | | | | | |
| 19 | 41223007 | 41246765 | ITPKC | | | | | |
| 19 | 41246760 | 41255828 | C19orf54 | | | | | |
| 19 | 41256758 | 41271297 | SNRPA | | | | | |
| 19 | 41281081 | 41283398 | MIA | | | | | |
| 19 | 41281441 | 41302849 | MIA-RAB4B | | | | | |
| 19 | 41284123 | 41302849 | RAB4B | | | RAB4B | | |
| 19 | 41284123 | 41314346 | RAB4B-EGLN2 | | | | | |
| 19 | 41305047 | 41314346 | EGLN2 | | | | | |
| 19 | 41349442 | 41356352 | CYP2A6 | | | | | |
| 19 | 41381343 | 41388657 | CYP2A7 | | | | | |
| 19 | 41396730 | 41406413 | CYP2G1P | | | | | |
| 19 | 41430169 | 41456565 | CYP2B7P | | | | | |
| 19 | 41497203 | 41524301 | CYP2B6 | | | | | |
| 19 | 41594355 | 41602100 | CYP2A13 | | | | | |
| 19 | 41620352 | 41634281 | CYP2F1 | | | | | |
| 19 | 41699111 | 41713444 | CYP2S1 | | | | | |
| 19 | 41725103 | 41767672 | AXL | | | | | |
| 19 | 41768380 | 41813597 | HNRNPUL1 | | | | | |
| 19 | 41816093 | 41830788 | CCDC97 | | | | | |
| 19 | 41836435 | 41859838 | TGFB1 | | | TGFB1 | | |
| 19 | 41860321 | 41870078 | B9D2 | | | | | |
| 19 | 41869870 | 41889987 | TMEM91 | | | | | |
| 19 | 41892275 | 41903256 | EXOSC5 | | | | | |
| 19 | 41903693 | 41930910 | BCKDHA | | | BCKDHA | | |
| 19 | 41931263 | 41934635 | B3GNT8 | | | | | |
| 19 | 41937222 | 41945843 | ATP5SL | | | | | |
| 19 | 41949062 | 41950670 | ERICH4 | | | | | |
| 19 | 41960073 | 42006554 | PCAT19 | | | | | |
| 19 | 42041859 | 42043266 | LINC01480 | | | | | |
| 19 | 42055885 | 42093197 | CEACAM21 | | | | | |
| 19 | 42125343 | 42133442 | CEACAM4 | | | | | |
| 19 | 42177234 | 42192206 | CEACAM7 | | | | | |
| 19 | 42212503 | 42234341 | CEACAM5 | | | | | |
| 19 | 42259427 | 42276113 | CEACAM6 | | | | | |
| 19 | 42300521 | 42315591 | CEACAM3 | | | | | |
| 19 | 42341147 | 42348736 | LYPD4 | | | | | |

| | | | | | | | | |
|---|---|---|---|---|---|---|---|---|
| 19 | 42349085 | 42356397 | DMRTC2 | | | | | |
| 19 | 42363987 | 42375484 | RPS19 | | | | | |
| 19 | 42373696 | 42373768 | MIR6797 | | | | | |
| 19 | 42381189 | 42385439 | CD79A | | | | | |
| 19 | 42387266 | 42411604 | ARHGEF1 | | | | | |
| 19 | 42460832 | 42463528 | RABAC1 | | | RABAC1 | | |
| 19 | 42470733 | 42498428 | ATP1A3 | ATP1A3 | ATP1A3 | ATP1A3 | | |
| 19 | 42502467 | 42569967 | GRIK5 | | | | | |
| 19 | 42580289 | 42585720 | ZNF574 | | | | | |
| 19 | 42590261 | 42636625 | POU2F2 | | | POU2F2 | | |
| 19 | 42636780 | 42641251 | LOC100505622 | | | | | |
| 19 | 42637596 | 42637665 | MIR4323 | | | | | |
| 19 | 42702744 | 42724304 | DEDD2 | | | | | |
| 19 | 42724422 | 42731205 | ZNF526 | | | | | |
| 19 | 42734337 | 42746736 | GSK3A | | | | | |
| 19 | 42751712 | 42759316 | ERF | | | | | |
| 19 | 42772688 | 42799948 | CIC | | | | | |
| 19 | 42801184 | 42806952 | PAFAH1B3 | | | PAFAH1B3 | | |
| 19 | 42806283 | 42814973 | PRR19 | | | | | |
| 19 | 42817476 | 42829214 | TMEM145 | | | | | |
| 19 | 42829760 | 42882921 | MEGF8 | | MEGF8 | | | |
| 19 | 42855282 | 42855357 | MIR8077 | | | | | |
| 19 | 42891170 | 42894444 | CNFN | | | | | |
| 19 | 42901279 | 42912604 | LOC101930071 | | | | | |
| 19 | 42901299 | 43156507 | LIPE | | LIPE | | | |
| 19 | 42932689 | 42947200 | CXCL17 | | | | | |
| 19 | 43011457 | 43032661 | CEACAM1 | | | | | |
| 19 | 43084394 | 43099082 | CEACAM8 | | | | | |
| 19 | 43225793 | 43244668 | PSG3 | | | | | |
| 19 | 43256838 | 43269831 | PSG8 | | | | | |
| 19 | 43326014 | 43331030 | LOC100289650 | | | | | |
| 19 | 43341148 | 43359870 | PSG10P | | | | | |
| 19 | 43370612 | 43383871 | PSG1 | | | | | |
| 19 | 43406233 | 43422076 | PSG6 | | | | | |
| 19 | 43428283 | 43441330 | PSG7 | | | | | |
| 19 | 43511808 | 43530631 | PSG11 | | | | | |
| 19 | 43568361 | 43586893 | PSG2 | | | | | |
| 19 | 43671894 | 43690688 | PSG5 | | | | | |
| 19 | 43696853 | 43709926 | PSG4 | | | | | |
| 19 | 43715942 | 43752798 | LOC284344 | | | | | |
| 19 | 43757433 | 43773715 | PSG9 | | | | | |
| 19 | 43853207 | 43853700 | PRG1 | | | | | |
| 19 | 43857810 | 43867324 | CD177 | | | | | |
| 19 | 43892762 | 43922767 | TEX101 | | | | | |
| 19 | 43964945 | 43969831 | LYPD3 | | | | | |
| 19 | 43979254 | 44008985 | PHLDB3 | | | | | |
| 19 | 44010870 | 44031396 | ETHE1 | | | ETHE1 | | |
| 19 | 44037339 | 44040284 | ZNF575 | | | | | |
| 19 | 44047463 | 44079730 | XRCC1 | | | | | |
| 19 | 44080951 | 44086256 | PINLYP | | | | | |
| 19 | 44088518 | 44100287 | IRGQ | | | | | |
| 19 | 44100543 | 44104587 | ZNF576 | | | | | |
| 19 | 44111375 | 44124014 | ZNF428 | | | | | |
| 19 | 44116252 | 44118650 | SRRM5 | | | | | |
| 19 | 44126521 | 44143991 | CADM4 | | | | | |
| 19 | 44150246 | 44174498 | PLAUR | | | | | |
| 19 | 44220213 | 44224169 | IRGC | | | | | |
| 19 | 44235300 | 44259142 | SMG9 | | | | | |
| 19 | 44270684 | 44285409 | KCNN4 | | | | | |
| 19 | 44300078 | 44324808 | LYPD5 | | | | | |
| 19 | 44331443 | 44353050 | ZNF283 | | | | | |
| 19 | 44376514 | 44384288 | ZNF404 | | | | | |
| 19 | 44395955 | 44405955 | LOC100505715 | | | | | |
| 19 | 44416775 | 44439411 | ZNF45 | | | | | |
| 19 | 44455374 | 44471752 | ZNF221 | | | | | |
| 19 | 44488321 | 44502477 | ZNF155 | | | | | |

| | | | | | | | | |
|---|---|---|---|---|---|---|---|---|
| 19 | 44501047 | 44507022 | LOC101928063 | | | | | |
| 19 | 44507076 | 44518072 | ZNF230 | | | | | |
| 19 | 44529493 | 44537262 | ZNF222 | | | | | |
| 19 | 44556163 | 44572147 | ZNF223 | | | | | |
| 19 | 44576296 | 44591623 | ZNF284 | | | | | |
| 19 | 44598481 | 44612479 | ZNF224 | | | | | |
| 19 | 44609491 | 44617336 | LOC100379224 | | | | | |
| 19 | 44617547 | 44637255 | ZNF225 | | | | | |
| 19 | 44645709 | 44664462 | ZNF234 | | | | | |
| 19 | 44669214 | 44681838 | ZNF226 | | | | | |
| 19 | 44716680 | 44741421 | ZNF227 | | | | | |
| 19 | 44764032 | 44779470 | ZNF233 | | | | | |
| 19 | 44790500 | 44809178 | ZNF235 | | | | | |
| 19 | 44830705 | 44860856 | ZNF112 | | | | | |
| 19 | 44889807 | 44905777 | ZNF285 | | | | | |
| 19 | 44930422 | 44952766 | ZNF229 | | | | | |
| 19 | 44978644 | 45004575 | ZNF180 | | | | | |
| 19 | 45010210 | 45033548 | CEACAM20 | | | | | |
| 19 | 45041044 | 45060150 | CEACAM22P | | | | | |
| 19 | 45116939 | 45140081 | IGSF23 | | | | | |
| 19 | 45147097 | 45169428 | PVR | | | | | |
| 19 | 45156955 | 45157002 | MIR4531 | | | | | |
| 19 | 45174723 | 45187627 | CEACAM19 | | | | | |
| 19 | 45202420 | 45213986 | CEACAM16 | | | | | |
| 19 | 45251977 | 45263301 | BCL3 | | | | | |
| 19 | 45261913 | 45261978 | MIR8085 | | | | | |
| 19 | 45281125 | 45303903 | CBLC | | | | | |
| 19 | 45312315 | 45324678 | BCAM | | | BCAM | | |
| 19 | 45349392 | 45392485 | PVRL2 | | | | | |
| 19 | 45394476 | 45406946 | TOMM40 | | | | | |
| 19 | 45409005 | 45412652 | APOE | APOE | APOE | APOE | | |
| 19 | 45417920 | 45422606 | APOC1 | | APOC1 | | | |
| 19 | 45430059 | 45434643 | APOC1P1 | | | | | |
| 19 | 45445494 | 45448753 | APOC4 | | | | | |
| 19 | 45445494 | 45452822 | APOC4-APOC2 | | | | | |
| 19 | 45449238 | 45452822 | APOC2 | | APOC2 | | | |
| 19 | 45457841 | 45496604 | CLPTM1 | | | | | |
| 19 | 45504706 | 45541456 | RELB | | | | | |
| 19 | 45542297 | 45574214 | CLASRP | | | | | |
| 19 | 45574757 | 45579688 | ZNF296 | | | | | |
| 19 | 45582452 | 45594174 | GEMIN7 | | | | | |
| 19 | 45588586 | 45593649 | LOC105372419 | | | | | |
| 19 | 45596430 | 45650543 | PPP1R37 | | | | | |
| 19 | 45653007 | 45663408 | NKPD1 | | | | | |
| 19 | 45666185 | 45681501 | TRAPPC6A | | | | | |
| 19 | 45682002 | 45685059 | BLOC1S3 | | | | | |
| 19 | 45715878 | 45737469 | EXOC3L2 | | | | | |
| 19 | 45754515 | 45808541 | MARK4 | | | | | |
| 19 | 45809670 | 45826235 | CKM | | | | | |
| 19 | 45843997 | 45854778 | KLC3 | | | | | |
| 19 | 45854648 | 45873845 | ERCC2 | | | | | |
| 19 | 45882891 | 45909607 | PPP1R13L | | | | | |
| 19 | 45909466 | 45914024 | CD3EAP | | | | | |
| 19 | 45910590 | 45926820 | ERCC1 | | | | | |
| 19 | 45939911 | 45939962 | MIR6088 | | | | | |
| 19 | 45971252 | 45978437 | FOSB | | | | | |
| 19 | 45988545 | 46000313 | RTN2 | | RTN2 | | | |
| 19 | 46001730 | 46005764 | PPM1N | | | | | |
| 19 | 46010687 | 46030240 | VASP | | | | | |
| 19 | 46031024 | 46088122 | OPA3 | | | OPA3 | | |
| 19 | 46093022 | 46105466 | GPR4 | | | | | |
| 19 | 46112657 | 46148775 | EML2 | | | | | |
| 19 | 46142251 | 46142345 | MIR330 | | | | | |
| 19 | 46144751 | 46146092 | EML2 | | | | | |
| 19 | 46171501 | 46186982 | GIPR | | | GIPR | | |
| 19 | 46178185 | 46178282 | MIR642A | | | | | |

| | | | | | | | | |
|---|---|---|---|---|---|---|---|---|
| 19 | 46178189 | 46178266 | MIR642B | | | | | |
| 19 | 46190711 | 46195443 | SNRPD2 | | | | | |
| 19 | 46195740 | 46207248 | QPCTL | | | | | |
| 19 | 46213886 | 46234151 | FBXO46 | | | | | |
| 19 | 46236508 | 46267797 | BHMG1 | | | | | |
| 19 | 46268042 | 46272497 | SIX5 | | | | | |
| 19 | 46272966 | 46285815 | DMPK | | | DMPK | | |
| 19 | 46286263 | 46296060 | DMWD | | | | | |
| 19 | 46298967 | 46318605 | RSPH6A | | | | | |
| 19 | 46318699 | 46366548 | SYMPK | | | | | |
| 19 | 46367517 | 46377055 | FOXA3 | | | | | |
| 19 | 46386864 | 46389428 | IRF2BP1 | | | | | |
| 19 | 46393280 | 46405862 | MYPOP | | | | | |
| 19 | 46416472 | 46418036 | NANOS2 | | | | | |
| 19 | 46442770 | 46476657 | NOVA2 | | | NOVA2 | | |
| 19 | 46498718 | 46521874 | CCDC61 | | | | | |
| 19 | 46522189 | 46522307 | MIR769 | | | | | |
| 19 | 46522411 | 46526556 | PGLYRP1 | | | | | |
| 19 | 46543005 | 46544274 | IGFL4 | | | | | |
| 19 | 46560935 | 46580887 | LOC400706 | | | | | |
| 19 | 46623327 | 46627931 | IGFL3 | | | | | |
| 19 | 46651038 | 46664561 | IGFL2 | | | | | |
| 19 | 46713498 | 46718094 | DKFZp434J0226 | | | | | |
| 19 | 46733008 | 46734500 | IGFL1 | | | | | |
| 19 | 46800302 | 46846690 | HIF3A | | | | | |
| 19 | 46850250 | 46894232 | PPP5C | | | PPP5C | | |
| 19 | 46913585 | 46916919 | CCDC8 | | | | | |
| 19 | 46969747 | 46974820 | PNMAL1 | | | | | |
| 19 | 46984044 | 47104457 | PPP5D1 | | | | | |
| 19 | 46994447 | 46999169 | PNMAL2 | | | | | |
| 19 | 47104511 | 47114039 | CALM3 | | | | | |
| 19 | 47123724 | 47128354 | PTGIR | | | | | |
| 19 | 47137332 | 47137939 | GNG8 | | | | | |
| 19 | 47150820 | 47174598 | DACT3 | | | | | |
| 19 | 47177572 | 47220384 | PRKD2 | | | PRKD2 | | |
| 19 | 47212549 | 47212602 | MIR320E | | | | | |
| 19 | 47222767 | 47249720 | STRN4 | | | | | |
| 19 | 47249302 | 47261832 | FKRP | | | FKRP | | |
| 19 | 47278139 | 47291842 | SLC1A5 | | | | | |
| 19 | 47333841 | 47333961 | SNAR-E | | | | | |
| 19 | 47341414 | 47354252 | AP2S1 | | | AP2S1 | | |
| 19 | 47421932 | 47508333 | ARHGAP35 | | ARHGAP35 | | | |
| 19 | 47524142 | 47549017 | NPAS1 | | | | | |
| 19 | 47549166 | 47551882 | TMEM160 | | | | | |
| 19 | 47567446 | 47617009 | ZC3H4 | | | | | |
| 19 | 47634079 | 47713893 | SAE1 | | | | | |
| 19 | 47724078 | 47736023 | BBC3 | | | | | |
| 19 | 47730198 | 47730278 | MIR3190 | | | | | |
| 19 | 47730200 | 47730276 | MIR3191 | | | | | |
| 19 | 47759730 | 47775210 | CCDC9 | | | | | |
| 19 | 47777658 | 47778980 | INAFM1 | | | | | |
| 19 | 47813103 | 47825327 | C5AR1 | | | | | |
| 19 | 47835403 | 47845272 | C5AR2 | | | | | |
| 19 | 47852537 | 47885961 | DHX34 | | | DHX34 | | |
| 19 | 47906374 | 47922785 | MEIS3 | | | | | |
| 19 | 47931278 | 47975307 | SLC8A2 | | | | | |
| 19 | 47978397 | 47987521 | KPTN | | | | | |
| 19 | 47987538 | 48018515 | NAPA | | | NAPA | | |
| 19 | 48023941 | 48059113 | ZNF541 | | | | | |
| 19 | 48111452 | 48206534 | GLTSCR1 | | | | | |
| 19 | 48216600 | 48246391 | EHD2 | | | | | |
| 19 | 48248792 | 48260323 | GLTSCR2 | | | | | |
| 19 | 48259109 | 48259219 | SNORD23 | | | | | |
| 19 | 48260292 | 48272097 | GLTSCR2 | | | | | |
| 19 | 48281841 | 48287943 | SEPW1 | | | | | |
| 19 | 48304499 | 48306861 | TPRX1 | | | | | |

| | | | | | | | | |
|---|---|---|---|---|---|---|---|---|
| 19 | 48325098 | 48346586 | CRX | | | | | |
| 19 | 48373722 | 48389654 | SULT2A1 | | | | | |
| 19 | 48410934 | 48411056 | SNAR-A12 | | | | | |
| 19 | 48410934 | 48411056 | SNAR-A13 | | | | | |
| 19 | 48416307 | 48416427 | SNAR-C1 | | | | | |
| 19 | 48416307 | 48416427 | SNAR-C2 | | | | | |
| 19 | 48416307 | 48416427 | SNAR-C5 | | | | | |
| 19 | 48421685 | 48421807 | SNAR-A1 | | | | | |
| 19 | 48421685 | 48421807 | SNAR-A2 | | | | | |
| 19 | 48427035 | 48427156 | SNAR-A10 | | | | | |
| 19 | 48427035 | 48427156 | SNAR-A11 | | | | | |
| 19 | 48427035 | 48427156 | SNAR-A14 | | | | | |
| 19 | 48427035 | 48427156 | SNAR-A3 | | | | | |
| 19 | 48427035 | 48427156 | SNAR-A4 | | | | | |
| 19 | 48427035 | 48427156 | SNAR-A5 | | | | | |
| 19 | 48427035 | 48427156 | SNAR-A6 | | | | | |
| 19 | 48427035 | 48427156 | SNAR-A7 | | | | | |
| 19 | 48427035 | 48427156 | SNAR-A8 | | | | | |
| 19 | 48427035 | 48427156 | SNAR-A9 | | | | | |
| 19 | 48432074 | 48432194 | SNAR-C1 | | | | | |
| 19 | 48432074 | 48432194 | SNAR-C2 | | | | | |
| 19 | 48432074 | 48432194 | SNAR-C5 | | | | | |
| 19 | 48437437 | 48437559 | SNAR-A1 | | | | | |
| 19 | 48437437 | 48437559 | SNAR-A2 | | | | | |
| 19 | 48442797 | 48442917 | SNAR-C4 | | | | | |
| 19 | 48448178 | 48448300 | SNAR-A12 | | | | | |
| 19 | 48448178 | 48448300 | SNAR-A13 | | | | | |
| 19 | 48453552 | 48453671 | SNAR-C3 | | | | | |
| 19 | 48458938 | 48459058 | SNAR-C1 | | | | | |
| 19 | 48458938 | 48459058 | SNAR-C2 | | | | | |
| 19 | 48458938 | 48459058 | SNAR-C5 | | | | | |
| 19 | 48471302 | 48495427 | BSPH1 | | | | | |
| 19 | 48497907 | 48528410 | ELSPBP1 | | | | | |
| 19 | 48532639 | 48547311 | CABP5 | | | | | |
| 19 | 48551099 | 48568202 | PLA2G4C | | PLA2G4C | | | |
| 19 | 48618701 | 48673860 | LIG1 | | | LIG1 | | |
| 19 | 48673948 | 48700877 | C19orf68 | | | | | |
| 19 | 48711342 | 48761450 | CARD8 | | | | | |
| 19 | 48773355 | 48790865 | ZNF114 | | | | | |
| 19 | 48799708 | 48823332 | CCDC114 | | | | | |
| 19 | 48828628 | 48833810 | EMP3 | | | | | |
| 19 | 48835612 | 48867494 | TMEM143 | | | | | |
| 19 | 48867650 | 48879634 | SYNGR4 | | | SYNGR4 | | |
| 19 | 48885826 | 48894810 | KDELR1 | | | | | |
| 19 | 48898131 | 48948188 | GRIN2D | | | | | |
| 19 | 48949029 | 48957164 | GRWD1 | | | | | |
| 19 | 48958963 | 48969367 | KCNJ14 | | | | | |
| 19 | 48972464 | 48985571 | CYTH2 | | | | | |
| 19 | 48988527 | 49016446 | LMTK3 | | | | | |
| 19 | 49055428 | 49102684 | SULT2B1 | | | | | |
| 19 | 49103856 | 49116694 | FAM83E | | | | | |
| 19 | 49109999 | 49110971 | SPACA4 | | | | | |
| 19 | 49118583 | 49122675 | RPL18 | | | | | |
| 19 | 49122547 | 49133663 | SPHK2 | | | | | |
| 19 | 49133816 | 49140807 | DBP | | | | | |
| 19 | 49141271 | 49149451 | CA11 | | | CA11 | | |
| 19 | 49141295 | 49185502 | SEC1P | | | | | |
| 19 | 49164663 | 49176338 | NTN5 | | | | | |
| 19 | 49199227 | 49209191 | FUT2 | | | | | |
| 19 | 49205706 | 49207105 | LOC105447645 | | | | | |
| 19 | 49215978 | 49222976 | MAMSTR | | | | | |
| 19 | 49223841 | 49243970 | RASIP1 | | | | | |
| 19 | 49244144 | 49250166 | IZUMO1 | | | | | |
| 19 | 49251267 | 49258647 | FUT1 | | | | | |
| 19 | 49259343 | 49261582 | FGF21 | | | | | |
| 19 | 49298318 | 49314320 | BCAT2 | | | BCAT2 | | |

| | | | | | | | | |
|---|---|---|---|---|---|---|---|---|
| 19 | 49316273 | 49339934 | HSD17B14 | | | | | |
| 19 | 49340353 | 49371884 | PLEKHA4 | | | | | |
| 19 | 49375648 | 49379319 | PPP1R15A | | | | | |
| 19 | 49384221 | 49401996 | TULP2 | | | | | |
| 19 | 49403306 | 49422148 | NUCB1 | | | | | |
| 19 | 49436938 | 49448226 | DHDH | | | | | |
| 19 | 49458116 | 49465055 | BAX | | | | | |
| 19 | 49468565 | 49470136 | FTL | | | | | |
| 19 | 49471381 | 49496610 | GYS1 | | | | | |
| 19 | 49497155 | 49519182 | RUVBL2 | | | | | |
| 19 | 49513162 | 49513229 | MIR6798 | | | | | |
| 19 | 49519236 | 49520347 | LHB | | | | | |
| 19 | 49521504 | 49522741 | LOC101059948 | | | | | |
| 19 | 49526125 | 49527632 | CGB3 | | | | | |
| 19 | 49534925 | 49535044 | SNAR-G2 | | | | | |
| 19 | 49535129 | 49536495 | CGB2 | | | | | |
| 19 | 49538825 | 49540191 | CGB1 | | | | | |
| 19 | 49540276 | 49540404 | SNAR-G1 | | | | | |
| 19 | 49547101 | 49548568 | CGB5 | | | | | |
| 19 | 49550894 | 49552368 | CGB8 | | | | | |
| 19 | 49557530 | 49558997 | CGB7 | | | | | |
| 19 | 49564396 | 49567124 | NTF4 | NTF4 | | | | |
| 19 | 49570674 | 49576198 | KCNA7 | | | | | |
| 19 | 49588396 | 49611870 | SNRNP70 | | | | | |
| 19 | 49617617 | 49621717 | LIN7B | | | LIN7B | | |
| 19 | 49621653 | 49622397 | C19orf73 | | | | | |
| 19 | 49622645 | 49654287 | PPFIA3 | | | | | |
| 19 | 49654455 | 49658681 | HRC | | | | | |
| 19 | 49661015 | 49715098 | TRPM4 | | | | | |
| 19 | 49792891 | 49828474 | SLC6A16 | | | SLC6A16 | | |
| 19 | 49812053 | 49812125 | MIR4324 | | | | | |
| 19 | 49838676 | 49843861 | CD37 | | | | | |
| 19 | 49843852 | 49865714 | TEAD2 | | | | | |
| 19 | 49866986 | 49878373 | DKKL1 | | | | | |
| 19 | 49871961 | 49891338 | LOC101928295 | | | | | |
| 19 | 49891474 | 49921256 | CCDC155 | | | | | |
| 19 | 49925670 | 49926698 | PTH2 | | | | | |
| 19 | 49929687 | 49932075 | GFY | | | | | |
| 19 | 49932654 | 49944808 | SLC17A7 | | | | | |
| 19 | 49949549 | 49955115 | PIH1D1 | | | | | |
| 19 | 49956472 | 49974305 | ALDH16A1 | | | | | |
| 19 | 49977465 | 49989488 | FLT3LG | | | FLT3LG | | |
| 19 | 49990810 | 49995564 | RPL13A | | | | | |
| 19 | 49990864 | 49995096 | RPL13AP5 | | | | | |
| 19 | 49993222 | 49993304 | SNORD32A | | | | | |
| 19 | 49993873 | 49993956 | SNORD33 | | | | | |
| 19 | 49994163 | 49994229 | SNORD34 | | | | | |
| 19 | 49994431 | 49994517 | SNORD35A | | | | | |
| 19 | 49999621 | 50002969 | RPS11 | | | | | |
| 19 | 50000975 | 50001062 | SNORD35B | | | | | |
| 19 | 50004041 | 50004125 | MIR150 | | | | | |
| 19 | 50015535 | 50029685 | FCGRT | | | | | |
| 19 | 50030874 | 50046890 | RCN3 | | | | | |
| 19 | 50058724 | 50083829 | NOSIP | | | | | |
| 19 | 50084586 | 50094265 | PRRG2 | | | | | |
| 19 | 50094911 | 50129696 | PRR12 | | | | | |
| 19 | 50138548 | 50143400 | RRAS | | | | | |
| 19 | 50145381 | 50161906 | SCAF1 | | | | | |
| 19 | 50162825 | 50169132 | IRF3 | | | | | |
| 19 | 50168398 | 50177173 | BCL2L12 | | | | | |
| 19 | 50180408 | 50191707 | PRMT1 | | | | | |
| 19 | 50185373 | 50185452 | MIR5088 | | | | | |
| 19 | 50191941 | 50194247 | ADM5 | | | | | |
| 19 | 50194364 | 50216988 | CPT1C | | CPT1C | CPT1C | | |
| 19 | 50243010 | 50266515 | TSKS | | | | | |
| 19 | 50270179 | 50310369 | AP2A1 | | | | | |

| | | | | | | | | | |
|---|---|---|---|---|---|---|---|---|---|
| 19 | 50295122 | 50295191 | MIR6799 | | | | | | |
| 19 | 50310123 | 50316567 | FUZ | | | | | | |
| 19 | 50321535 | 50340237 | MED25 | | | | | | |
| 19 | 50335274 | 50335356 | MIR6800 | | | | | | |
| 19 | 50341902 | 50364001 | PTOV1 | | | | | | |
| 19 | 50357847 | 50357908 | MIR4749 | | | | | | |
| 19 | 50359721 | 50362546 | PTOV1 | | | | | | |
| 19 | 50364459 | 50370822 | PNKP | | | | | | |
| 19 | 50372289 | 50381613 | AKT1S1 | | | | | | |
| 19 | 50380681 | 50392007 | TBC1D17 | | | | | | |
| 19 | 50391431 | 50391487 | MIR4750 | | | | | | |
| 19 | 50392912 | 50400147 | IL4I1 | | | | | | |
| 19 | 50410083 | 50432988 | NUP62 | | | | | | |
| 19 | 50431958 | 50437193 | ATF5 | | | | | | |
| 19 | 50436320 | 50436394 | MIR4751 | | | | | | |
| 19 | 50452249 | 50464429 | SIGLEC11 | | | | | | |
| 19 | 50472856 | 50476788 | SIGLEC16 | | | | | | |
| 19 | 50479723 | 50528643 | VRK3 | | | | | | |
| 19 | 50529062 | 50552031 | ZNF473 | | | | | | |
| 19 | 50553936 | 50570052 | FLJ26850 | | | | | | |
| 19 | 50595745 | 50595866 | SNAR-A10 | | | | | | |
| 19 | 50595745 | 50595866 | SNAR-A11 | | | | | | |
| 19 | 50595745 | 50595866 | SNAR-A14 | | | | | | |
| 19 | 50595745 | 50595866 | SNAR-A3 | | | | | | |
| 19 | 50595745 | 50595866 | SNAR-A4 | | | | | | |
| 19 | 50595745 | 50595866 | SNAR-A5 | | | | | | |
| 19 | 50595745 | 50595866 | SNAR-A6 | | | | | | |
| 19 | 50595745 | 50595866 | SNAR-A7 | | | | | | |
| 19 | 50595745 | 50595866 | SNAR-A8 | | | | | | |
| 19 | 50595745 | 50595866 | SNAR-A9 | | | | | | |
| 19 | 50601082 | 50601203 | SNAR-A10 | | | | | | |
| 19 | 50601082 | 50601203 | SNAR-A11 | | | | | | |
| 19 | 50601082 | 50601203 | SNAR-A14 | | | | | | |
| 19 | 50601082 | 50601203 | SNAR-A3 | | | | | | |
| 19 | 50601082 | 50601203 | SNAR-A4 | | | | | | |
| 19 | 50601082 | 50601203 | SNAR-A5 | | | | | | |
| 19 | 50601082 | 50601203 | SNAR-A6 | | | | | | |
| 19 | 50601082 | 50601203 | SNAR-A7 | | | | | | |
| 19 | 50601082 | 50601203 | SNAR-A8 | | | | | | |
| 19 | 50601082 | 50601203 | SNAR-A9 | | | | | | |
| 19 | 50604147 | 50604268 | SNAR-A10 | | | | | | |
| 19 | 50604147 | 50604268 | SNAR-A11 | | | | | | |
| 19 | 50604147 | 50604268 | SNAR-A14 | | | | | | |
| 19 | 50604147 | 50604268 | SNAR-A3 | | | | | | |
| 19 | 50604147 | 50604268 | SNAR-A4 | | | | | | |
| 19 | 50604147 | 50604268 | SNAR-A5 | | | | | | |
| 19 | 50604147 | 50604268 | SNAR-A6 | | | | | | |
| 19 | 50604147 | 50604268 | SNAR-A7 | | | | | | |
| 19 | 50604147 | 50604268 | SNAR-A8 | | | | | | |
| 19 | 50604147 | 50604268 | SNAR-A9 | | | | | | |
| 19 | 50607211 | 50607332 | SNAR-A10 | | | | | | |
| 19 | 50607211 | 50607332 | SNAR-A11 | | | | | | |
| 19 | 50607211 | 50607332 | SNAR-A14 | | | | | | |
| 19 | 50607211 | 50607332 | SNAR-A3 | | | | | | |
| 19 | 50607211 | 50607332 | SNAR-A4 | | | | | | |
| 19 | 50607211 | 50607332 | SNAR-A5 | | | | | | |
| 19 | 50607211 | 50607332 | SNAR-A6 | | | | | | |
| 19 | 50607211 | 50607332 | SNAR-A7 | | | | | | |
| 19 | 50607211 | 50607332 | SNAR-A8 | | | | | | |
| 19 | 50607211 | 50607332 | SNAR-A9 | | | | | | |
| 19 | 50610270 | 50610391 | SNAR-A10 | | | | | | |
| 19 | 50610270 | 50610391 | SNAR-A11 | | | | | | |
| 19 | 50610270 | 50610391 | SNAR-A14 | | | | | | |
| 19 | 50610270 | 50610391 | SNAR-A3 | | | | | | |
| 19 | 50610270 | 50610391 | SNAR-A4 | | | | | | |
| 19 | 50610270 | 50610391 | SNAR-A5 | | | | | | |

| | | | | | | | | |
|---|---|---|---|---|---|---|---|---|
| 19 | 50610270 | 50610391 | SNAR-A6 | | | | | |
| 19 | 50610270 | 50610391 | SNAR-A7 | | | | | |
| 19 | 50610270 | 50610391 | SNAR-A8 | | | | | |
| 19 | 50610270 | 50610391 | SNAR-A9 | | | | | |
| 19 | 50615624 | 50615745 | SNAR-A10 | | | | | |
| 19 | 50615624 | 50615745 | SNAR-A11 | | | | | |
| 19 | 50615624 | 50615745 | SNAR-A14 | | | | | |
| 19 | 50615624 | 50615745 | SNAR-A3 | | | | | |
| 19 | 50615624 | 50615745 | SNAR-A4 | | | | | |
| 19 | 50615624 | 50615745 | SNAR-A5 | | | | | |
| 19 | 50615624 | 50615745 | SNAR-A6 | | | | | |
| 19 | 50615624 | 50615745 | SNAR-A7 | | | | | |
| 19 | 50615624 | 50615745 | SNAR-A8 | | | | | |
| 19 | 50615624 | 50615745 | SNAR-A9 | | | | | |
| 19 | 50620976 | 50621097 | SNAR-A10 | | | | | |
| 19 | 50620976 | 50621097 | SNAR-A11 | | | | | |
| 19 | 50620976 | 50621097 | SNAR-A14 | | | | | |
| 19 | 50620976 | 50621097 | SNAR-A3 | | | | | |
| 19 | 50620976 | 50621097 | SNAR-A4 | | | | | |
| 19 | 50620976 | 50621097 | SNAR-A5 | | | | | |
| 19 | 50620976 | 50621097 | SNAR-A6 | | | | | |
| 19 | 50620976 | 50621097 | SNAR-A7 | | | | | |
| 19 | 50620976 | 50621097 | SNAR-A8 | | | | | |
| 19 | 50620976 | 50621097 | SNAR-A9 | | | | | |
| 19 | 50626330 | 50626451 | SNAR-A10 | | | | | |
| 19 | 50626330 | 50626451 | SNAR-A11 | | | | | |
| 19 | 50626330 | 50626451 | SNAR-A14 | | | | | |
| 19 | 50626330 | 50626451 | SNAR-A3 | | | | | |
| 19 | 50626330 | 50626451 | SNAR-A4 | | | | | |
| 19 | 50626330 | 50626451 | SNAR-A5 | | | | | |
| 19 | 50626330 | 50626451 | SNAR-A6 | | | | | |
| 19 | 50626330 | 50626451 | SNAR-A7 | | | | | |
| 19 | 50626330 | 50626451 | SNAR-A8 | | | | | |
| 19 | 50626330 | 50626451 | SNAR-A9 | | | | | |
| 19 | 50631658 | 50631779 | SNAR-A10 | | | | | |
| 19 | 50631658 | 50631779 | SNAR-A11 | | | | | |
| 19 | 50631658 | 50631779 | SNAR-A14 | | | | | |
| 19 | 50631658 | 50631779 | SNAR-A3 | | | | | |
| 19 | 50631658 | 50631779 | SNAR-A4 | | | | | |
| 19 | 50631658 | 50631779 | SNAR-A5 | | | | | |
| 19 | 50631658 | 50631779 | SNAR-A6 | | | | | |
| 19 | 50631658 | 50631779 | SNAR-A7 | | | | | |
| 19 | 50631658 | 50631779 | SNAR-A8 | | | | | |
| 19 | 50631658 | 50631779 | SNAR-A9 | | | | | |
| 19 | 50637001 | 50637121 | SNAR-B1 | | | | | |
| 19 | 50637001 | 50637121 | SNAR-B2 | | | | | |
| 19 | 50642374 | 50642494 | SNAR-B1 | | | | | |
| 19 | 50642374 | 50642494 | SNAR-B2 | | | | | |
| 19 | 50643458 | 50643577 | SNAR-D | | | | | |
| 19 | 50655804 | 50666538 | IZUMO2 | | | | | |
| 19 | 50706884 | 50813801 | MYH14 | | | | | |
| 19 | 50815198 | 50832634 | KCNC3 | | KCNC3 | KCNC3 | KCNC3 | |
| 19 | 50837056 | 50848005 | NAPSB | | | | | |
| 19 | 50861733 | 50868931 | NAPSA | | | | | |
| 19 | 50879679 | 50886285 | NR1H2 | | | | | |
| 19 | 50887579 | 50921275 | POLD1 | | | | | |
| 19 | 50922194 | 50934309 | SPIB | | | | | |
| 19 | 50936159 | 50969583 | MYBPC2 | | | | | |
| 19 | 50970041 | 50980003 | FAM71E1 | | | | | |
| 19 | 50979733 | 50986783 | EMC10 | | | | | |
| 19 | 51009253 | 51014612 | JOSD2 | | | | | |
| 19 | 51014856 | 51017947 | ASPDH | | | | | |
| 19 | 51020149 | 51071302 | LRRC4B | | | | | |
| 19 | 51108219 | 51108342 | SNAR-F | | | | | |
| 19 | 51125233 | 51143092 | SYT3 | | | SYT3 | | |
| 19 | 51152701 | 51162567 | C19orf81 | | | | | |

| | | | | | | | | |
|---|---|---|---|---|---|---|---|---|
| 19 | 51165083 | 51220195 | SHANK1 | | | | | |
| 19 | 51226604 | 51228981 | CLEC11A | | | | | |
| 19 | 51273720 | 51274989 | GPR32 | | | | | |
| 19 | 51279402 | 51289467 | LOC105372440 | | | | | |
| 19 | 51293671 | 51298481 | ACPT | | | | | |
| 19 | 51300949 | 51308110 | C19orf48 | | | | | |
| 19 | 51302285 | 51302382 | SNORD88B | | | | | |
| 19 | 51302695 | 51302792 | SNORD88A | | | | | |
| 19 | 51305581 | 51305678 | SNORD88C | | | | | |
| 19 | 51320936 | 51322134 | MGC45922 | | | | | |
| 19 | 51322401 | 51327043 | KLK1 | | | | | |
| 19 | 51328544 | 51334779 | KLK15 | | | | | |
| 19 | 51333785 | 51354399 | LOC105372441 | | | | | |
| 19 | 51358170 | 51364020 | KLK3 | | | | | |
| 19 | 51376688 | 51383823 | KLK2 | | | | | |
| 19 | 51385351 | 51399654 | KLKP1 | | | | | |
| 19 | 51409607 | 51413994 | KLK4 | | | | | |
| 19 | 51446558 | 51456344 | KLK5 | | | | | |
| 19 | 51461886 | 51472929 | KLK6 | | KLK6 | | | |
| 19 | 51479734 | 51487320 | KLK7 | | | | | |
| 19 | 51499263 | 51504958 | KLK8 | | | | | |
| 19 | 51505768 | 51512890 | KLK9 | | | | | |
| 19 | 51515999 | 51523431 | KLK10 | | | | | |
| 19 | 51525486 | 51531290 | KLK11 | | | | | |
| 19 | 51532347 | 51538148 | KLK12 | | | | | |
| 19 | 51559462 | 51568367 | KLK13 | | | | | |
| 19 | 51580751 | 51587467 | KLK14 | | | | | |
| 19 | 51600862 | 51611647 | CTU1 | | | | | |
| 19 | 51628136 | 51639520 | SIGLEC9 | | | | | |
| 19 | 51645557 | 51656783 | SIGLEC7 | | | | | |
| 19 | 51656179 | 51685222 | LOC101928517 | | | | | |
| 19 | 51670584 | 51676780 | SIGLEC17P | | | | | |
| 19 | 51710184 | 51710265 | MIR8074 | | | | | |
| 19 | 51728334 | 51743274 | CD33 | | | | | |
| 19 | 51754331 | 51772582 | SIGLECL1 | | | | | |
| 19 | 51815101 | 51834102 | IGLON5 | | | | | |
| 19 | 51834794 | 51845378 | VSIG10L | | | | | |
| 19 | 51848408 | 51869672 | ETFB | | | ETFB | | |
| 19 | 51870351 | 51872257 | CLDND2 | | | | | |
| 19 | 51874873 | 51875960 | NKG7 | | | | | |
| 19 | 51883162 | 51891210 | LIM2 | | | | | |
| 19 | 51891542 | 51893828 | C19orf84 | | | | | |
| 19 | 51913274 | 51921057 | SIGLEC10 | | | | | |
| 19 | 51919159 | 51920679 | LOC100129083 | | | | | |
| 19 | 51954250 | 51961708 | SIGLEC8 | | | | | |
| 19 | 51981896 | 51993859 | CEACAM18 | | | | | |
| 19 | 51994480 | 52005043 | SIGLEC12 | | | | | |
| 19 | 52022783 | 52035110 | SIGLEC6 | | | | | |
| 19 | 52074530 | 52092991 | ZNF175 | | | | | |
| 19 | 52095035 | 52097633 | LINC01530 | | | | | |
| 19 | 52114755 | 52133727 | SIGLEC5 | | | | | |
| 19 | 52145805 | 52150132 | SIGLEC14 | | | | | |
| 19 | 52188615 | 52196709 | SPACA6P | | | | | |
| 19 | 52192686 | 52208443 | SPACA6 | | | | | |
| 19 | 52195864 | 52195934 | MIR99B | | | | | |
| 19 | 52196038 | 52196117 | MIRLET7E | | | | | |
| 19 | 52196506 | 52196592 | MIR125A | | | | | |
| 19 | 52196592 | 52208443 | SPACA6 | | | | | |
| 19 | 52216364 | 52227245 | HAS1 | | | | | |
| 19 | 52249022 | 52255150 | FPR1 | | | FPR1 | | |
| 19 | 52264452 | 52273779 | FPR2 | | | | | |
| 19 | 52298398 | 52329443 | FPR3 | | | | | |
| 19 | 52359055 | 52391229 | ZNF577 | | | | | |
| 19 | 52391277 | 52408305 | ZNF649 | | | | | |
| 19 | 52430687 | 52449011 | ZNF613 | | | | | |
| 19 | 52452386 | 52490079 | ZNF350 | | | | | |

| | | | | | | | | | |
|---|---|---|---|---|---|---|---|---|---|
| 19 | 52494586 | 52511483 | ZNF615 | | | | | | |
| 19 | 52516576 | 52531680 | ZNF614 | | | | | | |
| 19 | 52536676 | 52552073 | ZNF432 | | | | | | |
| 19 | 52567718 | 52599018 | ZNF841 | | | | | | |
| 19 | 52617652 | 52643191 | ZNF616 | | | | | | |
| 19 | 52658124 | 52674896 | ZNF836 | | | | | | |
| 19 | 52693054 | 52729678 | PPP2R1A | | | | PPP2R1A | | |
| 19 | 52725272 | 52725351 | MIR6801 | | | | | | |
| 19 | 52772823 | 52795976 | ZNF766 | | | | | | |
| 19 | 52785049 | 52785143 | MIR643 | | | | | | |
| 19 | 52800421 | 52829180 | ZNF480 | | | | | | |
| 19 | 52839497 | 52870376 | ZNF610 | | | | | | |
| 19 | 52873169 | 52889046 | ZNF880 | | | | | | |
| 19 | 52892092 | 52921657 | ZNF528 | | | | | | |
| 19 | 52934666 | 52955192 | ZNF534 | | | | | | |
| 19 | 52956828 | 53020131 | ZNF578 | | | | | | |
| 19 | 53030908 | 53059303 | ZNF808 | | | | | | |
| 19 | 53073525 | 53090427 | ZNF701 | | | | | | |
| 19 | 53099936 | 53103405 | ZNF137P | | | | | | |
| 19 | 53115617 | 53193834 | ZNF83 | | | | | | |
| 19 | 53206065 | 53238307 | ZNF611 | | | | | | |
| 19 | 53268747 | 53290034 | ZNF600 | | | | | | |
| 19 | 53300660 | 53324922 | ZNF28 | | | | | | |
| 19 | 53341784 | 53360902 | ZNF468 | | | | | | |
| 19 | 53379424 | 53394599 | ZNF320 | | | | | | |
| 19 | 53409145 | 53421184 | ZNF888 | | | | | | |
| 19 | 53430387 | 53445847 | ZNF321P | | | | | | |
| 19 | 53430387 | 53466164 | ZNF816-ZNF321P | | | | | | |
| 19 | 53452631 | 53466164 | ZNF816 | | | | | | |
| 19 | 53471503 | 53496784 | ZNF702P | | | | | | |
| 19 | 53517343 | 53519833 | ERVV-1 | | | | | | |
| 19 | 53547990 | 53554380 | ERVV-2 | | | | | | |
| 19 | 53569866 | 53606687 | ZNF160 | | | | | | |
| 19 | 53611131 | 53636173 | ZNF415 | | | | | | |
| 19 | 53641956 | 53662322 | ZNF347 | | | | | | |
| 19 | 53666551 | 53696619 | ZNF665 | | | | | | |
| 19 | 53716200 | 53719355 | ZNF818P | | | | | | |
| 19 | 53738633 | 53758156 | ZNF677 | | | | | | |
| 19 | 53761544 | 53762855 | VN1R2 | | | | | | |
| 19 | 53770012 | 53770918 | VN1R4 | | | | | | |
| 19 | 53785542 | 53787602 | FAM90A27P | | | | | | |
| 19 | 53792853 | 53794875 | BIRC8 | | | | | | |
| 19 | 53837001 | 53858122 | ZNF845 | | | | | | |
| 19 | 53868967 | 53889841 | ZNF525 | | | | | | |
| 19 | 53898396 | 53915262 | ZNF765 | | | | | | |
| 19 | 53935226 | 53947925 | TPM3P9 | | | | | | |
| 19 | 53935226 | 53953608 | ZNF761 | | | | | | |
| 19 | 53970988 | 53997546 | ZNF813 | | | | | | |
| 19 | 54024176 | 54083523 | ZNF331 | | | | | | |
| 19 | 54102884 | 54106751 | LOC284379 | | | | | | |
| 19 | 54135309 | 54140263 | DPRX | | | | | | |
| 19 | 54169926 | 54172500 | MIR512 | | | | | | |
| 19 | 54175221 | 54175294 | MIR1323 | | | | | | |
| 19 | 54177450 | 54177574 | MIR498 | | | | | | |
| 19 | 54178964 | 54179051 | MIR520E | | | | | | |
| 19 | 54182256 | 54182339 | MIR515 | | | | | | |
| 19 | 54183193 | 54183277 | MIR519E | | | | | | |
| 19 | 54185412 | 54185499 | MIR520F | | | | | | |
| 19 | 54188262 | 54188345 | MIR515 | | | | | | |
| 19 | 54189722 | 54189809 | MIR519C | | | | | | |
| 19 | 54191734 | 54191821 | MIR1283 | | | | | | |
| 19 | 54194134 | 54194219 | MIR520A | | | | | | |
| 19 | 54197646 | 54197729 | MIR526B | | | | | | |
| 19 | 54198466 | 54198547 | MIR519B | | | | | | |
| 19 | 54200786 | 54200871 | MIR525 | | | | | | |
| 19 | 54201638 | 54201725 | MIR523 | | | | | | |

| | | | | | | | | |
|---|---|---|---|---|---|---|---|---|
| 19 | 54203268 | 54203355 | MIR518F | | | | | |
| 19 | 54204480 | 54204541 | MIR520B | | | | | |
| 19 | 54205990 | 54206073 | MIR518B | | | | | |
| 19 | 54209505 | 54209590 | MIR526A1 | | | | | |
| 19 | 54210706 | 54210793 | MIR520C | | | | | |
| 19 | 54211988 | 54212089 | MIR518C | | | | | |
| 19 | 54214255 | 54214342 | MIR524 | | | | | |
| 19 | 54215521 | 54215608 | MIR517A | | | | | |
| 19 | 54216600 | 54216688 | MIR519D | | | | | |
| 19 | 54219847 | 54219934 | MIR521 | | | | | |
| 19 | 54223349 | 54223436 | MIR520D | | | | | |
| 19 | 54224329 | 54224396 | MIR517B | | | | | |
| 19 | 54225419 | 54225509 | MIR520G | | | | | |
| 19 | 54228695 | 54228780 | MIR516B2 | | | | | |
| 19 | 54230175 | 54230240 | MIR526A2 | | | | | |
| 19 | 54233091 | 54233179 | MIR518E | | | | | |
| 19 | 54234259 | 54234344 | MIR518A1 | | | | | |
| 19 | 54238130 | 54238217 | MIR518D | | | | | |
| 19 | 54240098 | 54240188 | MIR516B1 | | | | | |
| 19 | 54242586 | 54242673 | MIR518A2 | | | | | |
| 19 | 54244566 | 54244661 | MIR517C | | | | | |
| 19 | 54245765 | 54245853 | MIR520H | | | | | |
| 19 | 54251889 | 54251976 | MIR521 | | | | | |
| 19 | 54254464 | 54254551 | MIR522 | | | | | |
| 19 | 54255650 | 54255735 | MIR519A1 | | | | | |
| 19 | 54257271 | 54257356 | MIR527 | | | | | |
| 19 | 54259994 | 54260084 | MIR516A1 | | | | | |
| 19 | 54261485 | 54261572 | MIR1283 | | | | | |
| 19 | 54264386 | 54264476 | MIR516A2 | | | | | |
| 19 | 54265597 | 54265684 | MIR519A2 | | | | | |
| 19 | 54290928 | 54290995 | MIR371A | | | | | |
| 19 | 54290930 | 54290996 | MIR371B | | | | | |
| 19 | 54291143 | 54291210 | MIR372 | | | | | |
| 19 | 54291958 | 54292027 | MIR373 | | | | | |
| 19 | 54296854 | 54327657 | NLRP12 | | | | | |
| 19 | 54368837 | 54379689 | MYADM | | | | | |
| 19 | 54385437 | 54410901 | PRKCG | PRKCG | PRKCG | PRKCG | | |
| 19 | 54415990 | 54446969 | CACNG7 | | | | | |
| 19 | 54466289 | 54493469 | CACNG8 | | | CACNG8 | | |
| 19 | 54485560 | 54485651 | MIR935 | | | | | |
| 19 | 54494402 | 54515920 | CACNG6 | | | | | |
| 19 | 54544079 | 54567207 | VSTM1 | | | | | |
| 19 | 54573200 | 54584634 | TARM1 | | | | | |
| 19 | 54597932 | 54605994 | OSCAR | | | | | |
| 19 | 54606159 | 54610281 | NDUFA3 | | | | | |
| 19 | 54610319 | 54619055 | TFPT | | | | | |
| 19 | 54618789 | 54635150 | PRPF31 | | | | | |
| 19 | 54641435 | 54659446 | CNOT3 | | | | | |
| 19 | 54659377 | 54663481 | LENG1 | | | | | |
| 19 | 54663832 | 54676944 | TMC4 | | | | | |
| 19 | 54677105 | 54693733 | MBOAT7 | | MBOAT7 | | | |
| 19 | 54694118 | 54698394 | TSEN34 | | | | | |
| 19 | 54704725 | 54711515 | RPS9 | | | | | |
| 19 | 54720146 | 54746711 | LILRB3 | | | | | |
| 19 | 54740467 | 54746610 | LILRA6 | | | | | |
| 19 | 54754268 | 54761171 | LILRB5 | | | | | |
| 19 | 54777674 | 54784399 | LILRB2 | | | | | |
| 19 | 54785963 | 54786035 | MIR4752 | | | | | |
| 19 | 54799849 | 54804265 | LILRA3 | | | | | |
| 19 | 54818352 | 54824409 | LILRA5 | | | | | |
| 19 | 54844455 | 54850440 | LILRA4 | | | | | |
| 19 | 54865232 | 54882163 | LAIR1 | | | | | |
| 19 | 54926604 | 54947899 | TTYH1 | | | | | |
| 19 | 54955993 | 54973226 | LENG8 | | | | | |
| 19 | 54972977 | 54974894 | LENG9 | | | | | |
| 19 | 54976208 | 54984437 | CDC42EP5 | | | | | |

| | | | | | | | | | |
|---|---|---|---|---|---|---|---|---|---|
| 19 | 55014012 | 55021900 | LAIR2 | | | | | | |
| 19 | 55043908 | 55057024 | KIR3DX1 | | | | | | |
| 19 | 55084463 | 55099028 | LILRA2 | | | | | | |
| 19 | 55105040 | 55111185 | LILRA1 | | | | | | |
| 19 | 55128383 | 55148981 | LILRB1 | | | | | | |
| 19 | 55156759 | 55156834 | MIR8061 | | | | | | |
| 19 | 55174270 | 55181810 | LILRB4 | | | | | | |
| 19 | 55219699 | 55224955 | LILRP2 | | | | | | |
| 19 | 55235951 | 55248003 | KIR3DL3 | | | | | | |
| 19 | 55249973 | 55264504 | KIR2DL3 | | | | | | |
| 19 | 55280873 | 55282516 | LOC101928804 | | | | | | |
| 19 | 55281264 | 55295778 | KIR2DL1 | | | | | | |
| 19 | 55315066 | 55325972 | KIR2DL4 | | | | | | |
| 19 | 55327892 | 55342233 | KIR3DL1 | | | | | | |
| 19 | 55344130 | 55360024 | KIR2DS4 | | | | | | |
| 19 | 55361897 | 55378670 | KIR3DL2 | | | | | | |
| 19 | 55385548 | 55402786 | FCAR | | | | | | |
| 19 | 55417507 | 55424439 | NCR1 | | | NCR1 | | | |
| 19 | 55434876 | 55458873 | NLRP7 | | | | | | |
| 19 | 55476651 | 55512510 | NLRP2 | | | | | | |
| 19 | 55525074 | 55549632 | GP6 | | | | | | |
| 19 | 55555691 | 55580914 | RDH13 | | | | | | |
| 19 | 55587220 | 55599291 | EPS8L1 | | | | | | |
| 19 | 55602280 | 55628968 | PPP1R12C | | | | | | |
| 19 | 55634592 | 55634660 | MIR7975 | | | | | | |
| 19 | 55644065 | 55660722 | TNNT1 | | | | | | |
| 19 | 55663135 | 55669100 | TNNI3 | | | | | | |
| 19 | 55670028 | 55678090 | DNAAF3 | | | | | | |
| 19 | 55684463 | 55691720 | SYT5 | | | SYT5 | | | |
| 19 | 55692614 | 55720874 | PTPRH | | | | | | |
| 19 | 55738001 | 55740632 | TMEM86B | | | | | | |
| 19 | 55741146 | 55770038 | PPP6R1 | | | | | | |
| 19 | 55742252 | 55742320 | MIR6804 | | | | | | |
| 19 | 55751279 | 55751344 | MIR6802 | | | | | | |
| 19 | 55756553 | 55756618 | MIR6803 | | | | | | |
| 19 | 55773589 | 55791751 | HSPBP1 | | | | | | |
| 19 | 55795533 | 55823903 | BRSK1 | | | | | | |
| 19 | 55824168 | 55836708 | TMEM150B | | | | | | |
| 19 | 55851220 | 55859489 | KMT5C | | | | | | |
| 19 | 55861069 | 55866182 | COX6B2 | | | | | | |
| 19 | 55866275 | 55874620 | FAM71E2 | | | | | | |
| 19 | 55875749 | 55881831 | IL11 | | | | | | |
| 19 | 55888203 | 55889612 | TMEM190 | | | | | | |
| 19 | 55890611 | 55895627 | TMEM238 | | | | | | |
| 19 | 55897299 | 55903451 | RPL28 | | | | | | |
| 19 | 55899548 | 55899610 | MIR6805 | | | | | | |
| 19 | 55912649 | 55919325 | UBE2S | | | | | | |
| 19 | 55940104 | 55954230 | SHISA7 | | | | | | |
| 19 | 55964345 | 55973049 | ISOC2 | | | | | | |
| 19 | 55987698 | 55995854 | ZNF628 | | | | | | |
| 19 | 55996556 | 55998935 | NAT14 | | | | | | |
| 19 | 55999869 | 56030466 | SSC5D | | | | | | |
| 19 | 56041099 | 56048435 | SBK2 | | | | | | |
| 19 | 56052022 | 56056909 | SBK3 | | | | | | |
| 19 | 56088890 | 56092211 | ZNF579 | | | | | | |
| 19 | 56102736 | 56110893 | FIZ1 | | | | | | |
| 19 | 56111705 | 56114504 | ZNF524 | | | | | | |
| 19 | 56124958 | 56129907 | ZNF865 | | | | | | |
| 19 | 56132106 | 56135941 | ZNF784 | | | | | | |
| 19 | 56152391 | 56154836 | ZNF580 | | | | | | |
| 19 | 56154985 | 56156989 | ZNF581 | | | | | | |
| 19 | 56158953 | 56164526 | CCDC106 | | | | | | |
| 19 | 56165415 | 56186082 | U2AF2 | | | | | | |
| 19 | 56186560 | 56207133 | EPN1 | | | EPN1 | | | |
| 19 | 56219797 | 56249768 | NLRP9 | | | | | | |
| 19 | 56270506 | 56274541 | RFPL4A | | | | | | |

| | | | | | | | | |
|---|---|---|---|---|---|---|---|---|
| 19 | 56283170 | 56284545 | RFPL4AL1 | | | | | |
| 19 | 56296762 | 56348128 | NLRP11 | | | | | |
| 19 | 56347943 | 56393220 | NLRP4 | | | | | |
| 19 | 56407310 | 56443702 | NLRP13 | | | | | |
| 19 | 56459197 | 56499995 | NLRP8 | | | | | |
| 19 | 56511091 | 56573174 | NLRP5 | | | | | |
| 19 | 56578049 | 56590166 | LOC101928886 | | | | | |
| 19 | 56598728 | 56632742 | ZNF787 | | | | | |
| 19 | 56652534 | 56672262 | ZNF444 | | | | | |
| 19 | 56687388 | 56697144 | GALP | | | | | |
| 19 | 56701057 | 56704421 | ZSCAN5B | | | | | |
| 19 | 56732678 | 56739659 | ZSCAN5A | | | | | |
| 19 | 56879467 | 56891196 | ZNF542P | | | | | |
| 19 | 56894647 | 56910544 | ZNF582 | | | | | |
| 19 | 56915382 | 56936400 | ZNF583 | | | | | |
| 19 | 56950692 | 57006805 | ZNF667 | | | | | |
| 19 | 57019211 | 57040269 | ZNF471 | | | | | |
| 19 | 57050316 | 57068170 | ZFP28 | | | | | |
| 19 | 57078889 | 57094262 | ZNF470 | | | | | |
| 19 | 57106663 | 57135544 | ZNF71 | | | | | |
| 19 | 57154526 | 57168614 | SMIM17 | | | | | |
| 19 | 57174019 | 57183123 | ZNF835 | | | | | |
| 19 | 57276689 | 57352097 | ZIM2 | | | | | |
| 19 | 57321444 | 57325161 | PEG3 | | PEG3 | | | |
| 19 | 57352269 | 57359922 | MIMT1 | | | | | |
| 19 | 57631508 | 57643293 | USP29 | | | | | |
| 19 | 57645463 | 57656570 | ZIM3 | | | | | |
| 19 | 57663093 | 57678856 | DUXA | | | | | |
| 19 | 57702867 | 57734214 | ZNF264 | | | | | |
| 19 | 57742376 | 57746916 | AURKC | | | | | |
| 19 | 57752052 | 57774106 | ZNF805 | | | | | |
| 19 | 57791852 | 57805436 | ZNF460 | | | | | |
| 19 | 57831864 | 57842144 | ZNF543 | | | | | |
| 19 | 57862641 | 57871265 | ZNF304 | | | | | |
| 19 | 57874802 | 57890925 | ZNF547 | | | | | |
| 19 | 57874878 | 57876721 | TRAPPC2B | | | | | |
| 19 | 57901217 | 57913919 | ZNF548 | | | | | |
| 19 | 57922528 | 57933307 | ZNF17 | | | | | |
| 19 | 57946692 | 57957191 | ZNF749 | | | | | |
| 19 | 57966541 | 57968107 | VN1R1 | | | | | |
| 19 | 57980953 | 57988938 | ZNF772 | | | | | |
| 19 | 57999078 | 58006048 | ZNF419 | | | | | |
| 19 | 58011221 | 58027791 | ZNF773 | | | | | |
| 19 | 58038692 | 58052244 | ZNF549 | | | | | |
| 19 | 58053203 | 58071231 | ZNF550 | | | | | |
| 19 | 58082933 | 58090243 | ZNF416 | | | | | |
| 19 | 58095627 | 58103758 | ZIK1 | | | | | |
| 19 | 58111252 | 58119637 | ZNF530 | | | | | |
| 19 | 58125829 | 58133636 | ZNF134 | | | | | |
| 19 | 58144534 | 58154147 | ZNF211 | | | | | |
| 19 | 58180302 | 58190520 | ZSCAN4 | | | | | |
| 19 | 58193336 | 58201169 | ZNF551 | | | | | |
| 19 | 58207642 | 58220579 | ZNF154 | | | | | |
| 19 | 58231118 | 58238995 | ZNF671 | | | | | |
| 19 | 58258163 | 58269527 | ZNF776 | | | | | |
| 19 | 58281019 | 58291984 | ZNF586 | | | | | |
| 19 | 58318449 | 58326281 | ZNF552 | | | | | |
| 19 | 58336666 | 58338830 | FKBP1AP1 | | | | | |
| 19 | 58341661 | 58357606 | ZNF587B | | | | | |
| 19 | 58361180 | 58376491 | ZNF587 | | | | | |
| 19 | 58380746 | 58400442 | ZNF814 | | | | | |
| 19 | 58417141 | 58427984 | ZNF417 | | | | | |
| 19 | 58433251 | 58446755 | ZNF418 | | | | | |
| 19 | 58452200 | 58459077 | ZNF256 | | | | | |
| 19 | 58469804 | 58485902 | C19orf18 | | | | | |
| 19 | 58488440 | 58514714 | ZNF606 | | | | | |

| | | | | | | | | |
|---|---|---|---|---|---|---|---|---|
| 19 | 58514261 | 58518574 | LOC100128398 | | | | | |
| 19 | 58545433 | 58565999 | ZSCAN1 | | | | | |
| 19 | 58570606 | 58581110 | ZNF135 | | | | | |
| 19 | 58595208 | 58629793 | ZSCAN18 | | | | | |
| 19 | 58637694 | 58662148 | ZNF329 | | | | | |
| 19 | 58694355 | 58724928 | ZNF274 | | | | | |
| 19 | 58740069 | 58775008 | ZNF544 | | | | | |
| 19 | 58790317 | 58807254 | ZNF8 | | | | | |
| 19 | 58838384 | 58853712 | ZSCAN22 | | | | | |
| 19 | 58846053 | 58846117 | MIR6806 | | | | | |
| 19 | 58858171 | 58866549 | A1BG | | | | | |
| 19 | 58865722 | 58874214 | ZNF497 | | | | | |
| 19 | 58878989 | 58892389 | ZNF837 | | | | | |
| 19 | 58898136 | 58898225 | MIR4754 | | | | | |
| 19 | 58898635 | 58906171 | RPS5 | | | | | |
| 19 | 58907456 | 58908446 | RNF225 | | | | | |
| 19 | 58920040 | 58929692 | ZNF584 | | | | | |
| 19 | 58944180 | 58951589 | ZNF132 | | | | | |
| 19 | 58962970 | 58969199 | ZNF324B | | | | | |
| 19 | 58978411 | 58984945 | ZNF324 | | | | | |
| 19 | 58987530 | 59000900 | ZNF446 | | | | | |
| 19 | 59009699 | 59023432 | SLC27A5 | | SLC27A5 | | | |
| 19 | 59024896 | 59053079 | ZBTB45 | | | | | |
| 19 | 59055835 | 59062082 | TRIM28 | | | | | |
| 19 | 59061651 | 59061743 | MIR6807 | | | | | |
| 19 | 59062932 | 59066491 | CHMP2A | | | CHMP2A | | |
| 19 | 59067078 | 59070343 | UBE2M | | | | | |
| 19 | 59070552 | 59084942 | MZF1 | | | | | |
| 19 | 59086765 | 59095762 | CENPBD1P1 | | | | | |
| 20 | 68312 | 77214 | DEFB125 | | | | | |
| 20 | 123193 | 126392 | DEFB126 | | | | | |
| 20 | 138110 | 139804 | DEFB127 | | | | | |
| 20 | 168493 | 170322 | DEFB128 | | | | | |
| 20 | 207898 | 210527 | DEFB129 | | | | | |
| 20 | 238376 | 241736 | DEFB132 | | | | | |
| 20 | 251503 | 271419 | C20orf96 | | | | | |
| 20 | 278203 | 280963 | ZCCHC3 | | | | | |
| 20 | 300330 | 305896 | NRSN2 | | | | | |
| 20 | 306214 | 310872 | SOX12 | | | | | |
| 20 | 327369 | 335512 | NRSN2 | | | | | |
| 20 | 361272 | 378203 | TRIB3 | | | TRIB3 | | |
| 20 | 388708 | 411610 | RBCK1 | | | | | |
| 20 | 416120 | 443197 | TBC1D20 | | | | | |
| 20 | 463337 | 524482 | CSNK2A1 | | | CSNK2A1 | | |
| 20 | 584636 | 590910 | TCF15 | | | | | |
| 20 | 627267 | 634014 | SRXN1 | | | | | |
| 20 | 642239 | 656823 | SCRT2 | | | | | |
| 20 | 740723 | 749228 | SLC52A3 | | | | | |
| 20 | 814339 | 826922 | FAM110A | | | | | |
| 20 | 853296 | 896960 | ANGPT4 | | | | | |
| 20 | 939095 | 982907 | RSPO4 | | RSPO4 | | | |
| 20 | 1093905 | 1148426 | PSMF1 | | | | | |
| 20 | 1161204 | 1166077 | TMEM74B | | | | | |
| 20 | 1184097 | 1188918 | C20orf202 | | | | | |
| 20 | 1206763 | 1235145 | RAD21L1 | | | | | |
| 20 | 1246934 | 1289971 | SNPH | | | SNPH | | |
| 20 | 1290554 | 1309879 | SDCBP2 | | | | | |
| 20 | 1290554 | 1373816 | FKBP1A-SDCBP2 | | | | | |
| 20 | 1305986 | 1359379 | SDCBP2 | | | | | |
| 20 | 1349620 | 1373816 | FKBP1A | | | | | |
| 20 | 1373543 | 1373605 | MIR6869 | | | | | |
| 20 | 1422806 | 1448337 | NSFL1C | | | | | |
| 20 | 1455235 | 1472233 | SIRPB2 | | | | | |
| 20 | 1514896 | 1538343 | SIRPD | | | | | |
| 20 | 1545028 | 1600689 | SIRPB1 | | | | | |
| 20 | 1609797 | 1629119 | SIRPG | | | | | |

| | | | | | | | | |
|---|---|---|---|---|---|---|---|---|
| 20 | 1754010 | 1760392 | LOC100289473 | | | | | |
| 20 | 1874812 | 1920540 | SIRPA | | | SIRPA | | |
| 20 | 1927855 | 1988163 | LOC727993 | | | | | |
| 20 | 1959401 | 1974931 | PDYN | | PDYN | PDYN | PDYN | |
| 20 | 2082527 | 2129198 | STK35 | | | | | |
| 20 | 2187573 | 2193797 | LOC388780 | | | | | |
| 20 | 2276612 | 2321725 | TGM3 | | | | | |
| 20 | 2361553 | 2413399 | TGM6 | | | | | |
| 20 | 2442280 | 2451499 | SNRPB | | | | | |
| 20 | 2443597 | 2443693 | SNORD119 | | | | | |
| 20 | 2462465 | 2489778 | ZNF343 | | | | | |
| 20 | 2517252 | 2622430 | TMC2 | | | TMC2 | | |
| 20 | 2633177 | 2639039 | NOP56 | | | | | |
| 20 | 2633422 | 2633488 | MIR1292 | | | | | |
| 20 | 2634857 | 2634932 | SNORD110 | | | | | |
| 20 | 2635712 | 2635844 | SNORA51 | | | | | |
| 20 | 2636742 | 2636828 | SNORD86 | | | | | |
| 20 | 2637269 | 2637340 | SNORD56 | | | | | |
| 20 | 2637584 | 2637656 | SNORD57 | | | | | |
| 20 | 2639040 | 2644865 | IDH3B | | | | | |
| 20 | 2673523 | 2740754 | EBF4 | | | | | |
| 20 | 2774714 | 2781292 | CPXM1 | | | | | |
| 20 | 2795632 | 2796476 | C20orf141 | | | | | |
| 20 | 2796947 | 2800930 | TMEM239 | | | | | |
| 20 | 2815960 | 2821889 | PCED1A | | | | | |
| 20 | 2821342 | 2847378 | VPS16 | | | | | |
| 20 | 2844840 | 3019315 | PTPRA | | | | | |
| 20 | 3024267 | 3026391 | GNRH2 | | | | | |
| 20 | 3026674 | 3028896 | MRPS26 | | | | | |
| 20 | 3052265 | 3053163 | OXT | | | | | |
| 20 | 3063201 | 3065370 | AVP | AVP | | | | |
| 20 | 3087556 | 3140556 | UBOX5 | | | | | |
| 20 | 3127164 | 3140532 | FASTKD5 | | | | | |
| 20 | 3143262 | 3154238 | LZTS3 | | | | | |
| 20 | 3170995 | 3185343 | DDRGK1 | | | | | |
| 20 | 3189513 | 3204516 | ITPA | | | | | |
| 20 | 3208062 | 3219887 | SLC4A11 | | | | | |
| 20 | 3229947 | 3388309 | C20orf194 | | C20orf194 | | | |
| 20 | 3451664 | 3631769 | ATRN | | ATRN | | | |
| 20 | 3639938 | 3644046 | GFRA4 | | | | | |
| 20 | 3648619 | 3662778 | ADAM33 | | | | | |
| 20 | 3667616 | 3687775 | SIGLEC1 | | | | | |
| 20 | 3713316 | 3733758 | HSPA12B | | | | | |
| 20 | 3734145 | 3749035 | C20orf27 | | | | | |
| 20 | 3758150 | 3762102 | SPEF1 | | | | | |
| 20 | 3764497 | 3767337 | CENPB | | | | | |
| 20 | 3767418 | 3786768 | CDC25B | | | CDC25B | | |
| 20 | 3789140 | 3793081 | LOC101929125 | | | | | |
| 20 | 3801170 | 3805954 | AP5S1 | | | | | |
| 20 | 3827445 | 3856770 | MAVS | | | | | |
| 20 | 3869485 | 3904502 | PANK2 | | | | | |
| 20 | 3898140 | 3898218 | MIR103A2 | | | | | |
| 20 | 3898148 | 3898210 | MIR103B2 | | | | | |
| 20 | 3912068 | 3996216 | RNF24 | | | | | |
| 20 | 4129425 | 4168394 | SMOX | | | SMOX | | |
| 20 | 4173736 | 4176600 | LINC01433 | | | | | |
| 20 | 4201277 | 4229659 | ADRA1D | | | | | |
| 20 | 4666796 | 4682234 | PRNP | | PRNP | PRNP | | |
| 20 | 4702499 | 4709108 | PRND | | | | | |
| 20 | 4711927 | 4721314 | PRNT | | | | | |
| 20 | 4760668 | 4804291 | RASSF2 | | | | | |
| 20 | 4833001 | 4990939 | SLC23A2 | | SLC23A2 | | | |
| 20 | 5080483 | 5093733 | TMEM230 | | | | | |
| 20 | 5095598 | 5100615 | PCNA | | | | | |
| 20 | 5107406 | 5178533 | CDS2 | | | CDS2 | | |
| 20 | 5282685 | 5295015 | PROKR2 | | | PROKR2 | | |

| | | | | | | | | |
|---|---|---|---|---|---|---|---|---|
| 20 | 5412602 | 5426394 | LINC00658 | | | | | |
| 20 | 5451841 | 5457780 | LOC643406 | | | | | |
| 20 | 5479217 | 5485242 | LINC00654 | | | | | |
| 20 | 5488520 | 5490648 | LOC101929207 | | | | | |
| 20 | 5525079 | 5591672 | GPCPD1 | | | | | |
| 20 | 5731032 | 5845053 | C20orf196 | | | | | |
| 20 | 5891973 | 5906005 | CHGB | | CHGB | CHGB | | |
| 20 | 5918478 | 5931204 | TRMT6 | | | | | |
| 20 | 5931297 | 5986467 | MCM8 | | | | | |
| 20 | 5986738 | 6020697 | CRLS1 | | | | | |
| 20 | 6021191 | 6034726 | LRRN4 | | | | | |
| 20 | 6055491 | 6104191 | FERMT1 | | | | | |
| 20 | 6407378 | 6509106 | CASC20 | | | | | |
| 20 | 6748744 | 6760925 | BMP2 | | | | | |
| 20 | 7127113 | 7234849 | LINC01428 | | | | | |
| 20 | 7282735 | 7288079 | LOC101929288 | | | | | |
| 20 | 7328079 | 7349148 | LOC101929312 | | | | | |
| 20 | 7352254 | 7352339 | MIR8062 | | | | | |
| 20 | 7863630 | 7921093 | HAO1 | | | | | |
| 20 | 7957999 | 8000476 | TMX4 | | | | | |
| 20 | 8112911 | 8865547 | PLCB1 | | PLCB1 | PLCB1 | | |
| 20 | 9049700 | 9461462 | PLCB4 | | | PLCB4 | | |
| 20 | 9485826 | 9511171 | LAMP5 | | | | | |
| 20 | 9518036 | 9819687 | PAK7 | | | PAK7 | | |
| 20 | 9966980 | 9987764 | LOC101929371 | | | | | |
| 20 | 10004459 | 10200154 | SNAP25 | | SNAP25 | SNAP25 | | SNAP25 |
| 20 | 10015646 | 10037410 | ANKEF1 | | | | | |
| 20 | 10199476 | 10288066 | SNAP25 | | SNAP25 | SNAP25 | | SNAP25 |
| 20 | 10385427 | 10414887 | MKKS | | | MKKS | | |
| 20 | 10415950 | 10604027 | SLX4IP | | | | | |
| 20 | 10618331 | 10654694 | JAG1 | | | JAG1 | | |
| 20 | 10630283 | 10630343 | MIR6870 | | | | | |
| 20 | 10733737 | 10734675 | LOC101929395 | | | | | |
| 20 | 10855980 | 10889920 | LOC101929413 | | | | | |
| 20 | 11247306 | 11254031 | LOC339593 | | | | | |
| 20 | 11790634 | 11851363 | LINC00687 | | | | | |
| 20 | 11871370 | 11907243 | BTBD3 | | | | | |
| 20 | 12845851 | 12933167 | LOC101929486 | | | | | |
| 20 | 12915525 | 12917611 | LOC102606466 | | | | | |
| 20 | 12930987 | 12932275 | LOC100505515 | | | | | |
| 20 | 12989626 | 13147411 | SPTLC3 | | | | | |
| 20 | 13202417 | 13220334 | ISM1 | | | | | |
| 20 | 13370035 | 13619583 | TASP1 | | | | | |
| 20 | 13694968 | 13765579 | ESF1 | | | | | |
| 20 | 13765671 | 13799067 | NDUFAF5 | | | | | |
| 20 | 13830043 | 13975979 | SEL1L2 | | | | | |
| 20 | 13976145 | 16033841 | MACROD2 | | | | | |
| 20 | 14304638 | 14318313 | FLRT3 | | | | | |
| 20 | 14535029 | 14609554 | MACROD2-IT1 | | | | | |
| 20 | 14864898 | 16033841 | MACROD2 | | | | | |
| 20 | 15872978 | 15966527 | LOC613266 | | | | | |
| 20 | 16252748 | 16554079 | KIF16B | | | KIF16B | | |
| 20 | 16710608 | 16722417 | SNRPB2 | | | | | |
| 20 | 16728997 | 16732809 | OTOR | | | | | |
| 20 | 17206751 | 17465222 | PCSK2 | | | | | |
| 20 | 17474549 | 17549865 | BFSP1 | | | | | |
| 20 | 17550598 | 17588652 | DSTN | | | DSTN | | |
| 20 | 17594322 | 17662928 | RRBP1 | | | | | |
| 20 | 17674319 | 17716517 | BANF2 | | | | | |
| 20 | 17922239 | 17949634 | SNX5 | | | SNX5 | | |
| 20 | 17943352 | 17943589 | SNORD17 | | | | | |
| 20 | 17949533 | 17971765 | MGME1 | | | | | |
| 20 | 18004795 | 18039832 | OVOL2 | | | | | |
| 20 | 18118498 | 18123812 | PET117 | | | | | |
| 20 | 18122867 | 18169031 | CSRP2BP | | | | | |
| 20 | 18268926 | 18297640 | ZNF133 | | | | | |

| | | | | | | | | |
|---|---|---|---|---|---|---|---|---|
| 20 | 18359692 | 18362127 | LINC00851 | | | | | |
| 20 | 18364010 | 18447829 | DZANK1 | | | | | |
| 20 | 18448032 | 18465292 | POLR3F | | | | | |
| 20 | 18451258 | 18451335 | MIR3192 | | | | | |
| 20 | 18467187 | 18477887 | RBBP9 | | | | | |
| 20 | 18488187 | 18542059 | SEC23B | | | | | |
| 20 | 18548072 | 18550203 | LINC00493 | | | | | |
| 20 | 18568536 | 18744560 | DTD1 | | | | | |
| 20 | 18655038 | 18679352 | LOC101929526 | | | | | |
| 20 | 18768614 | 18775228 | LINC00652 | | | | | |
| 20 | 18774692 | 18776709 | LOC100270804 | | | | | |
| 20 | 18789994 | 18810847 | C20orf78 | | | | | |
| 20 | 18794369 | 18795035 | SCP2D1 | | | | | |
| 20 | 19193289 | 19703541 | SLC24A3 | | | | | |
| 20 | 19222945 | 19265240 | LOC100130264 | | | | | |
| 20 | 19867164 | 19983103 | RIN2 | | | | | |
| 20 | 19997933 | 20014273 | NAA20 | | | | | |
| 20 | 20015004 | 20036690 | CRNKL1 | | | | | |
| 20 | 20033157 | 20152977 | CFAP61 | | | | | |
| 20 | 20348764 | 20351592 | INSM1 | | | | | |
| 20 | 20370271 | 20693266 | RALGAPA2 | | | | | |
| 20 | 21106623 | 21198927 | KIZ | | | | | |
| 20 | 21283921 | 21370463 | XRN2 | | | XRN2 | | |
| 20 | 21376004 | 21378047 | NKX2-4 | | | | | |
| 20 | 21491659 | 21494664 | NKX2-2 | | NKX2-2 | | | |
| 20 | 21550661 | 21596360 | LOC101929625 | | | | | |
| 20 | 21591424 | 21684223 | LOC101929608 | | | | | |
| 20 | 21686296 | 21699124 | PAX1 | | | | | |
| 20 | 22034727 | 22055292 | LINC01432 | | | | | |
| 20 | 22260865 | 22263439 | LINC01427 | | | | | |
| 20 | 22380970 | 22401281 | LOC284788 | | | | | |
| 20 | 22541191 | 22559280 | LINC00261 | | | | | |
| 20 | 22561641 | 22566101 | FOXA2 | | | | | |
| 20 | 22568159 | 22588155 | LINC01384 | | | | | |
| 20 | 23016056 | 23017314 | SSTR4 | | | | | |
| 20 | 23026269 | 23030301 | THBD | | | | | |
| 20 | 23059992 | 23066977 | CD93 | | | | | |
| 20 | 23105704 | 23113273 | LINC00656 | | | | | |
| 20 | 23331372 | 23335408 | NXT1 | | | | | |
| 20 | 23337230 | 23338764 | LINC01431 | | | | | |
| 20 | 23342768 | 23353698 | GZF1 | | | | | |
| 20 | 23355155 | 23402156 | NAPB | | | | | |
| 20 | 23420321 | 23425567 | CSTL1 | | | | | |
| 20 | 23431040 | 23433482 | CST11 | | | | | |
| 20 | 23471737 | 23476655 | CST8 | | | | | |
| 20 | 23499782 | 23522655 | CST13P | | | | | |
| 20 | 23545369 | 23549386 | CST9L | | | | | |
| 20 | 23583046 | 23586610 | CST9 | | | | | |
| 20 | 23608533 | 23618685 | CST3 | | CST3 | | | |
| 20 | 23666276 | 23669662 | CST4 | | | | | |
| 20 | 23728189 | 23731574 | CST1 | | | | | |
| 20 | 23804403 | 23807312 | CST2 | | | | | |
| 20 | 23856571 | 23860380 | CST5 | | | | | |
| 20 | 23965689 | 23969416 | GGTLC1 | | | | | |
| 20 | 24180402 | 24205224 | FLJ33581 | | | | | |
| 20 | 24449834 | 24647253 | SYNDIG1 | | | | | |
| 20 | 24929865 | 24940564 | CST7 | | | | | |
| 20 | 24943579 | 24973425 | APMAP | | | | | |
| 20 | 24986865 | 25038818 | ACSS1 | | | ACSS1 | | |
| 20 | 25051520 | 25063015 | VSX1 | | VSX1 | | | |
| 20 | 25121433 | 25129426 | LOC284798 | | | | | |
| 20 | 25165307 | 25177528 | LOC101926889 | | | | | |
| 20 | 25176305 | 25207365 | ENTPD6 | | | | | |
| 20 | 25228705 | 25278648 | PYGB | | | | | |
| 20 | 25275378 | 25371618 | ABHD12 | | ABHD12 | | | |
| 20 | 25388318 | 25429191 | GINS1 | | | | | |

| | | | | | | | | | |
|---|---|---|---|---|---|---|---|---|---|
| 20 | 25433332 | 25566167 | NINL | | | | | | |
| 20 | 25593572 | 25604648 | NANP | | | | | | |
| 20 | 25604680 | 25677540 | ZNF337 | | | | | | |
| 20 | 25731843 | 25736686 | LOC105372582 | | | | | | |
| 20 | 25744101 | 25781927 | FAM182B | | | | | | |
| 20 | 25825302 | 25834657 | LOC101926935 | | | | | | |
| 20 | 25936447 | 25949924 | LOC101926955 | | | | | | |
| 20 | 25990434 | 26002430 | LOC100134868 | | | | | | |
| 20 | 26035249 | 26067553 | FAM182A | | | | | | |
| 20 | 26084051 | 26094677 | NCOR1P1 | | | | | | |
| 20 | 26167654 | 26189869 | MIR663AHG | | | | | | |
| 20 | 26188821 | 26188914 | MIR663A | | | | | | |
| 20 | 29558487 | 29570363 | LINC01598 | | | | | | |
| 20 | 29611878 | 29634007 | FRG1BP | | | | | | |
| 20 | 29612047 | 29628331 | FRG1DP | | | | | | |
| 20 | 29637583 | 29638138 | MLLT10P1 | | | | | | |
| 20 | 29845466 | 29847435 | DEFB115 | | | | | | |
| 20 | 29891014 | 29896388 | DEFB116 | | | | | | |
| 20 | 29956420 | 29961705 | DEFB118 | | | | | | |
| 20 | 29964965 | 29978452 | DEFB119 | | | | | | |
| 20 | 29992647 | 30000641 | DEFB121 | | | | | | |
| 20 | 30009241 | 30016983 | DEFB122 | | | | | | |
| 20 | 30028321 | 30038060 | DEFB123 | | | DEFB123 | | | |
| 20 | 30053308 | 30060816 | DEFB124 | | | | | | |
| 20 | 30063090 | 30072708 | REM1 | | | | | | |
| 20 | 30073580 | 30075377 | LINC00028 | | | | | | |
| 20 | 30102212 | 30161066 | HM13 | | | | | | |
| 20 | 30193085 | 30194317 | ID1 | | | | | | |
| 20 | 30194988 | 30195043 | MIR3193 | | | | | | |
| 20 | 30225690 | 30232800 | COX4I2 | | | COX4I2 | | | |
| 20 | 30252254 | 30310901 | BCL2L1 | | | BCL2L1 | | | |
| 20 | 30309309 | 30311212 | ABALON | | | | | | |
| 20 | 30326903 | 30389603 | TPX2 | | | | | | |
| 20 | 30407177 | 30422500 | MYLK2 | | | | | | |
| 20 | 30432102 | 30433420 | FOXS1 | | | | | | |
| 20 | 30448869 | 30458479 | DUSP15 | | | | | | |
| 20 | 30458504 | 30532762 | TTLL9 | | | | | | |
| 20 | 30532757 | 30539883 | PDRG1 | | | | | | |
| 20 | 30555804 | 30586256 | XKR7 | | | | | | |
| 20 | 30596189 | 30596239 | MIR7641 | | | | | | |
| 20 | 30598244 | 30619984 | CCM2L | | | | | | |
| 20 | 30639990 | 30689657 | HCK | | | | | | |
| 20 | 30697308 | 30755061 | TM9SF4 | | | | | | |
| 20 | 30776948 | 30778163 | TSPY26P | | | | | | |
| 20 | 30780306 | 30795546 | PLAGL2 | | | | | | |
| 20 | 30795695 | 30826467 | POFUT1 | | | | | | |
| 20 | 30825597 | 30825650 | MIR1825 | | | | | | |
| 20 | 30865453 | 30922811 | KIF3B | | | KIF3B | | | |
| 20 | 30946146 | 31027122 | ASXL1 | | | | | | |
| 20 | 31030861 | 31172875 | NOL4L | | | | | | |
| 20 | 31097761 | 31108088 | LOC101929698 | | | | | | |
| 20 | 31175280 | 31196694 | LOC149950 | | | | | | |
| 20 | 31219426 | 31239783 | C20orf203 | | | | | | |
| 20 | 31290492 | 31331814 | COMMD7 | | | | | | |
| 20 | 31350190 | 31397162 | DNMT3B | | | | | | |
| 20 | 31407698 | 31438211 | MAPRE1 | | | | | | |
| 20 | 31571580 | 31592239 | SUN5 | | | | | | |
| 20 | 31595383 | 31611515 | BPIFB2 | | | | | | |
| 20 | 31619453 | 31631853 | BPIFB6 | | | | | | |
| 20 | 31643136 | 31661443 | BPIFB3 | | | | | | |
| 20 | 31669317 | 31699557 | BPIFB4 | | | | | | |
| 20 | 31749653 | 31769223 | BPIFA2 | | | | | | |
| 20 | 31781410 | 31798268 | BPIFA4P | | | | | | |
| 20 | 31805115 | 31815612 | BPIFA3 | | | | | | |
| 20 | 31823801 | 31831115 | BPIFA1 | | | | | | |
| 20 | 31870940 | 31897684 | BPIFB1 | | | | | | |

| | | | | | | | | |
|---|---|---|---|---|---|---|---|---|
| 20 | 31946644 | 31989375 | CDK5RAP1 | | | CDK5RAP1 | | |
| 20 | 31995762 | 32031698 | SNTA1 | | SNTA1 | | SNTA1 | SNTA1 |
| 20 | 32077927 | 32237837 | CBFA2T2 | | | | | |
| 20 | 32244892 | 32262264 | NECAB3 | | | | | |
| 20 | 32250091 | 32251721 | C20orf144 | | | | | |
| 20 | 32254303 | 32256331 | ACTL10 | | | | | |
| 20 | 32263291 | 32274210 | E2F1 | | | E2F1 | | |
| 20 | 32290549 | 32308136 | PXMP4 | | | | | |
| 20 | 32319565 | 32398905 | ZNF341 | | | | | |
| 20 | 32399109 | 32442173 | CHMP4B | | | CHMP4B | | |
| 20 | 32580293 | 32670991 | RALY | | | | | |
| 20 | 32636924 | 32636996 | MIR4755 | | | | | |
| 20 | 32676103 | 32700162 | EIF2S2 | | | | | |
| 20 | 32848170 | 32857148 | ASIP | | | | | |
| 20 | 32868070 | 32899608 | AHCY | | | | | |
| 20 | 32951040 | 33099198 | ITCH | | | | | |
| 20 | 33054129 | 33054223 | MIR644A | | | | | |
| 20 | 33104188 | 33128762 | DYNLRB1 | | | DYNLRB1 | | |
| 20 | 33134687 | 33148149 | MAP1LC3A | | | | | |
| 20 | 33148345 | 33265089 | PIGU | | | | | |
| 20 | 33292147 | 33301237 | TP53INP2 | | | | | |
| 20 | 33302577 | 33409442 | NCOA6 | | | NCOA6 | | |
| 20 | 33421377 | 33422265 | HMGB3P1 | | | | | |
| 20 | 33432522 | 33460661 | GGT7 | | | | | |
| 20 | 33462765 | 33515769 | ACSS2 | | | | | |
| 20 | 33516235 | 33543601 | GSS | | | | | |
| 20 | 33543637 | 33590240 | MYH7B | | MYH7B | | | |
| 20 | 33578178 | 33578300 | MIR499A | | | | | |
| 20 | 33578202 | 33578275 | MIR499B | | | | | |
| 20 | 33590206 | 33680618 | TRPC4AP | | | | | |
| 20 | 33703159 | 33735161 | EDEM2 | | | | | |
| 20 | 33759739 | 33765165 | PROCR | | | | | |
| 20 | 33814538 | 33865960 | MMP24 | | | | | |
| 20 | 33866708 | 33872619 | EIF6 | | | | | |
| 20 | 33873053 | 33880225 | FAM83C | | | | | |
| 20 | 33890368 | 33999945 | UQCC1 | | | | | |
| 20 | 34021144 | 34042568 | GDF5 | | | GDF5 | | |
| 20 | 34041775 | 34041919 | MIR1289 | | | | | |
| 20 | 34043222 | 34099804 | CEP250 | | | | | |
| 20 | 34108569 | 34117481 | C20orf173 | | | | | |
| 20 | 34129777 | 34145405 | ERGIC3 | | | | | |
| 20 | 34146506 | 34195484 | FER1L4 | | | | | |
| 20 | 34203805 | 34209016 | SPAG4 | | | | | |
| 20 | 34213952 | 34252878 | CPNE1 | | | | | |
| 20 | 34236846 | 34252878 | RBM12 | | | | | |
| 20 | 34256609 | 34287287 | NFS1 | | | NFS1 | | |
| 20 | 34287231 | 34288902 | ROMO1 | | | | | |
| 20 | 34291530 | 34330258 | RBM39 | | | | | |
| 20 | 34359922 | 34538288 | PHF20 | | | | | |
| 20 | 34541538 | 34543281 | SCAND1 | | | | | |
| 20 | 34556502 | 34618622 | CNBD2 | | | | | |
| 20 | 34633539 | 34638882 | NORAD | | | | | |
| 20 | 34679425 | 34820721 | EPB41L1 | | EPB41L1 | EPB41L1 | | |
| 20 | 34824338 | 34844863 | AAR2 | | | | | |
| 20 | 34894244 | 35201678 | DLGAP4 | | | DLGAP4 | | |
| 20 | 35169886 | 35178226 | MYL9 | | | | | |
| 20 | 35201875 | 35222355 | TGIF2 | | | TGIF2 | | |
| 20 | 35202956 | 35240960 | TGIF2-C20orf24 | | | | | |
| 20 | 35234136 | 35240960 | C20orf24 | | | | | |
| 20 | 35240923 | 35274619 | SLA2 | | | | | |
| 20 | 35280168 | 35374541 | NDRG3 | | | | | |
| 20 | 35380193 | 35402230 | DSN1 | | | | | |
| 20 | 35405844 | 35492087 | SOGA1 | | | | | |
| 20 | 35504523 | 35522634 | TLDC2 | | | | | |
| 20 | 35520226 | 35580246 | SAMHD1 | | | | | |
| 20 | 35624754 | 35724403 | RBL1 | | | RBL1 | | |

| | | | | | | | | |
|---|---|---|---|---|---|---|---|---|
| 20 | 35729628 | 35807991 | MROH8 | | | | | |
| 20 | 35807455 | 35870025 | RPN2 | | | | | |
| 20 | 35879489 | 35885311 | GHRH | | | | | |
| 20 | 35918050 | 35945663 | MANBAL | | | | | |
| 20 | 35973087 | 36033835 | SRC | | | SRC | | |
| 20 | 36145818 | 36156333 | BLCAP | | | | | |
| 20 | 36149606 | 36152090 | NNAT | | NNAT | | | |
| 20 | 36247699 | 36251521 | LINC00489 | | | | | |
| 20 | 36305311 | 36311636 | LOC100287792 | | | | | |
| 20 | 36322356 | 36500531 | CTNNBL1 | | | | | |
| 20 | 36531498 | 36573747 | VSTM2L | | | | | |
| 20 | 36611408 | 36661870 | TTI1 | | | | | |
| 20 | 36661947 | 36720766 | RPRD1B | | | | | |
| 20 | 36756863 | 36793700 | TGM2 | | | | | |
| 20 | 36838906 | 36889174 | KIAA1755 | | | | | |
| 20 | 36888576 | 36917348 | LOC149684 | | | | | |
| 20 | 36932551 | 36965905 | BPI | | | | | |
| 20 | 36974813 | 37005653 | LBP | | | | | |
| 20 | 37049238 | 37063962 | SNHG17 | | | | | |
| 20 | 37053842 | 37053978 | SNORA71B | | | | | |
| 20 | 37055948 | 37056086 | SNORA71A | | | | | |
| 20 | 37058309 | 37058447 | SNORA71C | | | | | |
| 20 | 37062504 | 37062642 | SNORA71D | | | | | |
| 20 | 37075296 | 37079564 | SNHG11 | | | | | |
| 20 | 37076725 | 37076861 | SNORA71E | | | | | |
| 20 | 37078011 | 37078147 | SNORA60 | | | | | |
| 20 | 37101448 | 37207504 | RALGAPB | | | | | |
| 20 | 37209837 | 37217106 | ADIG | | | | | |
| 20 | 37230576 | 37279295 | ARHGAP40 | | | | | |
| 20 | 37353104 | 37358015 | SLC32A1 | | | SLC32A1 | | |
| 20 | 37377096 | 37401089 | ACTR5 | | | | | |
| 20 | 37434335 | 37551667 | PPP1R16B | | | | | |
| 20 | 37554954 | 37581703 | FAM83D | | | | | |
| 20 | 37590980 | 37668366 | DHX35 | | | | | |
| 20 | 37842423 | 37853391 | LOC339568 | | | | | |
| 20 | 38633102 | 38637171 | LINC01370 | | | | | |
| 20 | 39314487 | 39317880 | MAFB | | | | | |
| 20 | 39609400 | 39654015 | LOC100128988 | | | | | |
| 20 | 39657461 | 39753126 | TOP1 | | | | | |
| 20 | 39726633 | 39804357 | PLCG1 | | | PLCG1 | | |
| 20 | 39797662 | 39797718 | MIR6871 | | | | | |
| 20 | 39807088 | 39928739 | ZHX3 | | ZHX3 | | | |
| 20 | 39969492 | 39989225 | LPIN3 | | | | | |
| 20 | 39988605 | 39995498 | EMILIN3 | | | | | |
| 20 | 40030742 | 40247133 | CHD6 | | | CHD6 | | |
| 20 | 40701391 | 41818557 | PTPRT | | PTPRT | | | |
| 20 | 41314122 | 41317202 | LOC101927159 | | | | | |
| 20 | 42086503 | 42092244 | SRSF6 | | | | | |
| 20 | 42136319 | 42170535 | L3MBTL1 | | L3MBTL1 | | | |
| 20 | 42187634 | 42214273 | SGK2 | | SGK2 | SGK2 | | |
| 20 | 42219252 | 42275862 | IFT52 | | | | | |
| 20 | 42295658 | 42345136 | MYBL2 | | | | | |
| 20 | 42354800 | 42355642 | GTSF1L | | | | | |
| 20 | 42523357 | 42524110 | LOC105372626 | | | | | |
| 20 | 42543491 | 42698254 | TOX2 | | | | | |
| 20 | 42740336 | 42816218 | JPH2 | | | | | |
| 20 | 42824580 | 42854667 | OSER1 | | | | | |
| 20 | 42875738 | 42909557 | GDAP1L1 | | | | | |
| 20 | 42935196 | 42939889 | FITM2 | | | | | |
| 20 | 42965797 | 42979432 | R3HDML | | | | | |
| 20 | 42984440 | 43061485 | HNF4A | | | | | |
| 20 | 43036759 | 43036843 | MIR3646 | | | | | |
| 20 | 43077416 | 43079197 | LINC01430 | | | | | |
| 20 | 43080623 | 43093984 | LINC01620 | | | | | |
| 20 | 43104525 | 43123244 | TTPAL | | | | | |
| 20 | 43124863 | 43150726 | SERINC3 | | | | | |

| | | | | | | | | |
|---|---|---|---|---|---|---|---|---|
| 20 | 43160421 | 43247678 | PKIG | | | | | |
| 20 | 43248162 | 43280376 | ADA | | | ADA | | |
| 20 | 43285091 | 43300380 | LINC01260 | | | | | |
| 20 | 43340502 | 43374868 | KCNK15 | | | | | |
| 20 | 43343884 | 43356452 | WISP2 | | | | | |
| 20 | 43374487 | 43380954 | KCNK15 | | | | | |
| 20 | 43380444 | 43438979 | RIMS4 | | | RIMS4 | | |
| 20 | 43514239 | 43537175 | YWHAB | | | YWHAB | | |
| 20 | 43538700 | 43587799 | PABPC1L | | | | | |
| 20 | 43570770 | 43589114 | TOMM34 | | | | | |
| 20 | 43592439 | 43708593 | STK4 | | | STK4 | | |
| 20 | 43720949 | 43729753 | KCNS1 | | | KCNS1 | | |
| 20 | 43738092 | 43743813 | WFDC5 | | | | | |
| 20 | 43752066 | 43753106 | WFDC12 | | | | | |
| 20 | 43803539 | 43805185 | PI3 | | | | | |
| 20 | 43835637 | 43838414 | SEMG1 | | | | | |
| 20 | 43850009 | 43853099 | SEMG2 | | | | | |
| 20 | 43880879 | 43883205 | SLPI | | | | | |
| 20 | 43922085 | 43937169 | MATN4 | | | | | |
| 20 | 43935482 | 43946464 | RBPJL | | | | | |
| 20 | 43953928 | 43977064 | SDC4 | | | | | |
| 20 | 43990576 | 44005442 | SYS1 | | | | | |
| 20 | 43991808 | 44039250 | SYS1-DBNDD2 | | | | | |
| 20 | 44002519 | 44006957 | TP53TG5 | | | | | |
| 20 | 44034632 | 44039250 | DBNDD2 | | | | | |
| 20 | 44044706 | 44054885 | PIGT | | | PIGT | | |
| 20 | 44054149 | 44054213 | MIR6812 | | | | | |
| 20 | 44098393 | 44110172 | WFDC2 | | | | | |
| 20 | 44141100 | 44144264 | SPINT3 | | | | | |
| 20 | 44162835 | 44168134 | WFDC6 | | | | | |
| 20 | 44164918 | 44176065 | EPPIN-WFDC6 | | | | | |
| 20 | 44169264 | 44176065 | EPPIN | | | | | |
| 20 | 44179790 | 44207965 | WFDC8 | | | | | |
| 20 | 44236577 | 44259907 | WFDC9 | | | | | |
| 20 | 44258384 | 44259831 | WFDC10A | | | | | |
| 20 | 44277201 | 44298878 | WFDC11 | | | | | |
| 20 | 44313289 | 44333658 | WFDC10B | | | | | |
| 20 | 44330654 | 44337456 | WFDC13 | | | | | |
| 20 | 44333740 | 44333819 | MIR3617 | | | | | |
| 20 | 44350987 | 44354335 | SPINT4 | | | | | |
| 20 | 44402846 | 44420547 | WFDC3 | | | | | |
| 20 | 44420575 | 44440066 | DNTTIP1 | | | | | |
| 20 | 44441214 | 44445596 | UBE2C | | | | | |
| 20 | 44451854 | 44455953 | TNNC2 | | | | | |
| 20 | 44462469 | 44471914 | SNX21 | | | | | |
| 20 | 44470359 | 44486048 | ACOT8 | | | | | |
| 20 | 44486219 | 44507769 | ZSWIM3 | | | | | |
| 20 | 44509847 | 44513905 | ZSWIM1 | | | | | |
| 20 | 44515129 | 44516238 | SPATA25 | | | | | |
| 20 | 44517110 | 44519926 | NEURL2 | | | | | |
| 20 | 44518782 | 44527458 | CTSA | | | | | |
| 20 | 44527258 | 44541003 | PLTP | | | | | |
| 20 | 44563316 | 44576662 | PCIF1 | | | | | |
| 20 | 44577291 | 44600833 | ZNF335 | | | | | |
| 20 | 44637546 | 44645200 | MMP9 | | | | | |
| 20 | 44650328 | 44688789 | SLC12A5 | | SLC12A5 | SLC12A5 | | |
| 20 | 44689625 | 44718580 | NCOA5 | | | NCOA5 | | |
| 20 | 44746892 | 44758384 | CD40 | | | | | |
| 20 | 44802371 | 44937137 | CDH22 | | | | | |
| 20 | 44978166 | 44993097 | SLC35C2 | | | | | |
| 20 | 44994683 | 45035690 | ELMO2 | | | | | |
| 20 | 45085131 | 45086282 | ZNF663P | | | | | |
| 20 | 45092298 | 45093931 | MKRN7P | | | | | |
| 20 | 45128268 | 45142198 | ZNF334 | | | | | |
| 20 | 45169669 | 45179213 | OCSTAMP | | | | | |
| 20 | 45186461 | 45313124 | SLC13A3 | | SLC13A3 | | | |

| | | | | | | | | |
|---|---|---|---|---|---|---|---|---|
| 20 | 45313003 | 45318276 | TP53RK | | | | | |
| 20 | 45338278 | 45364985 | SLC2A10 | | | | | |
| 20 | 45523262 | 45817492 | EYA2 | | | EYA2 | | |
| 20 | 45795608 | 45795700 | MIR3616 | | | | | |
| 20 | 45837858 | 45985633 | ZMYND8 | | | ZMYND8 | | |
| 20 | 45947245 | 45949498 | LOC100131496 | | | | | |
| 20 | 45981304 | 45983376 | LOC101927377 | | | | | |
| 20 | 46130600 | 46285621 | NCOA3 | | | NCOA3 | | |
| 20 | 46286149 | 46415360 | SULF2 | | | | | |
| 20 | 46608836 | 46618861 | LINC01522 | | | | | |
| 20 | 46611969 | 46613057 | LINC01523 | | | | | |
| 20 | 46988653 | 46999381 | LINC00494 | | | | | |
| 20 | 47240792 | 47444420 | PREX1 | | | PREX1 | | |
| 20 | 47538274 | 47653230 | ARFGEF2 | | | | | |
| 20 | 47656999 | 47713497 | CSE1L | | | | | |
| 20 | 47729875 | 47835832 | STAU1 | | | | | |
| 20 | 47835831 | 47860614 | DDX27 | | | | | |
| 20 | 47862438 | 47894756 | ZNFX1 | | | | | |
| 20 | 47894714 | 47905795 | ZFAS1 | | | | | |
| 20 | 47895481 | 47895560 | SNORD12C | | | | | |
| 20 | 47896849 | 47896952 | SNORD12B | | | | | |
| 20 | 47897219 | 47897309 | SNORD12 | | | | | |
| 20 | 47988504 | 48099181 | KCNB1 | | KCNB1 | KCNB1 | | |
| 20 | 48120410 | 48184707 | PTGIS | | | | | |
| 20 | 48249482 | 48330421 | B4GALT5 | | | | | |
| 20 | 48429249 | 48508779 | SLC9A8 | | | SLC9A8 | | |
| 20 | 48519928 | 48532080 | SPATA2 | | | | | |
| 20 | 48532358 | 48545358 | LOC105372653 | | | | | |
| 20 | 48552913 | 48570422 | RNF114 | | | | | |
| 20 | 48599512 | 48605420 | SNAI1 | | | | | |
| 20 | 48657243 | 48658166 | TRERNA1 | | | | | |
| 20 | 48697660 | 48732496 | UBE2V1 | | | UBE2V1 | | |
| 20 | 48697660 | 48770335 | TMEM189-UBE2V1 | | | | | |
| 20 | 48711955 | 48729735 | UBE2V1 | | | UBE2V1 | | |
| 20 | 48740273 | 48770335 | TMEM189 | | | | | |
| 20 | 48789106 | 48793208 | LINC01273 | | | | | |
| 20 | 48801139 | 48809227 | CEBPB | | | | | |
| 20 | 48884022 | 48896332 | LINC01272 | | | | | |
| 20 | 48909256 | 48931456 | LINC01270 | | | | | |
| 20 | 48927247 | 48937879 | LINC01271 | | | | | |
| 20 | 49126857 | 49201300 | PTPN1 | | | PTPN1 | | |
| 20 | 49202322 | 49202416 | MIR645 | | | | | |
| 20 | 49202644 | 49308067 | FAM65C | | | | | |
| 20 | 49231172 | 49231322 | MIR1302 | | | | | |
| 20 | 49262007 | 49276828 | LOC100506175 | | | | | |
| 20 | 49348080 | 49370278 | PARD6B | | | PARD6B | | |
| 20 | 49411430 | 49493714 | BCAS4 | | | | | |
| 20 | 49505454 | 49561671 | ADNP | | | ADNP | | |
| 20 | 49551391 | 49575101 | DPM1 | | | | | |
| 20 | 49575350 | 49578399 | MOCS3 | | | | | |
| 20 | 49620192 | 49639675 | KCNG1 | | | | | |
| 20 | 50003493 | 50179370 | NFATC2 | | | NFATC2 | | |
| 20 | 50069441 | 50069514 | MIR3194 | | | | | |
| 20 | 50213313 | 50384950 | ATP9A | | | | | |
| 20 | 50399255 | 50419062 | SALL4 | | | SALL4 | | |
| 20 | 50448335 | 50479452 | LINC01429 | | | | | |
| 20 | 50700549 | 50808524 | ZFP64 | | | | | |
| 20 | 51237376 | 51266965 | LINC01524 | | | | | |
| 20 | 51588945 | 52111869 | TSHZ2 | | | | | |
| 20 | 52169308 | 52191847 | LOC101927770 | | | | | |
| 20 | 52183609 | 52199636 | ZNF217 | | | | | |
| 20 | 52224892 | 52251129 | LOC105372672 | | | | | |
| 20 | 52491039 | 52492248 | SUMO1P1 | | | | | |
| 20 | 52560078 | 52687304 | BCAS1 | | | | | |
| 20 | 52684946 | 52685024 | MIR4756 | | | | | |
| 20 | 52769987 | 52790516 | CYP24A1 | | | CYP24A1 | | |

| | | | | | | | | |
|---|---|---|---|---|---|---|---|---|
| 20 | 52824501 | 52836492 | PFDN4 | | | | | |
| 20 | 53092010 | 53267710 | DOK5 | | | DOK5 | | |
| 20 | 54036873 | 54043735 | LINC01441 | | | | | |
| 20 | 54039580 | 54043329 | LINC01440 | | | | | |
| 20 | 54572412 | 54580528 | CBLN4 | | | | | |
| 20 | 54823787 | 54824871 | MC3R | | | | | |
| 20 | 54933982 | 54943718 | FAM210B | | | | | |
| 20 | 54944444 | 54967351 | AURKA | | | AURKA | | |
| 20 | 54967426 | 54979582 | CSTF1 | | | | | |
| 20 | 54987167 | 55034396 | CASS4 | | | | | |
| 20 | 55043640 | 55093942 | RTFDC1 | | | | | |
| 20 | 55066547 | 55100981 | GCNT7 | | | | | |
| 20 | 55099784 | 55101208 | FAM209A | | | | | |
| 20 | 55108301 | 55111574 | FAM209B | | | | | |
| 20 | 55204357 | 55214338 | TFAP2C | | | TFAP2C | | |
| 20 | 55743808 | 55790922 | BMP7 | | BMP7 | | | |
| 20 | 55896557 | 55896647 | MIR4325 | | | | | |
| 20 | 55904830 | 55919049 | SPO11 | | | | | |
| 20 | 55926144 | 55953519 | RAE1 | | | | | |
| 20 | 55933495 | 55934878 | MTRNR2L3 | | | | | |
| 20 | 55966453 | 55984386 | RBM38 | | | | | |
| 20 | 56071020 | 56100708 | CTCFL | | | | | |
| 20 | 56136136 | 56141513 | PCK1 | | | PCK1 | | |
| 20 | 56178901 | 56195632 | ZBP1 | | | | | |
| 20 | 56223447 | 56286592 | PMEPA1 | | | | | |
| 20 | 56285238 | 56287836 | NKILA | | | | | |
| 20 | 56470449 | 56470500 | MIR4532 | | | | | |
| 20 | 56725982 | 56736183 | C20orf85 | | | | | |
| 20 | 56791181 | 56803709 | ANKRD60 | | | | | |
| 20 | 56807832 | 56884495 | PPP4R1L | | | | | |
| 20 | 56884770 | 56942563 | RAB22A | | | RAB22A | | |
| 20 | 56964174 | 57026156 | VAPB | | | | | |
| 20 | 57034159 | 57194948 | APCDD1L | | | | | |
| 20 | 57209827 | 57210794 | LOC79160 | | | | | |
| 20 | 57226308 | 57254582 | STX16 | | | | | |
| 20 | 57226308 | 57290900 | STX16-NPEPL1 | | | | | |
| 20 | 57264186 | 57290900 | NPEPL1 | | | | | |
| 20 | 57348480 | 57349867 | LOC105372695 | | | | | |
| 20 | 57392669 | 57392749 | MIR296 | | | | | |
| 20 | 57393280 | 57393368 | MIR298 | | | | | |
| 20 | 57393972 | 57486251 | GNAS | | | | | |
| 20 | 57438810 | 57463865 | LOC101927932 | | | | | |
| 20 | 57463467 | 57475554 | GNAS | | | | | |
| 20 | 57556262 | 57570188 | NELFCD | | | | | |
| 20 | 57570241 | 57582309 | CTSZ | | | | | |
| 20 | 57594308 | 57601709 | TUBB1 | | | | | |
| 20 | 57603732 | 57607422 | ATP5E | | | | | |
| 20 | 57603732 | 57617901 | SLMO2-ATP5E | | | | | |
| 20 | 57608199 | 57617901 | PRELID3B | | | | | |
| 20 | 57766074 | 57834167 | ZNF831 | | | | | |
| 20 | 57875481 | 57901047 | EDN3 | | EDN3 | EDN3 | | |
| 20 | 58152563 | 58422766 | PHACTR3 | | PHACTR3 | PHACTR3 | PHACTR3 | |
| 20 | 58201518 | 58203344 | LOC100506384 | | | | | |
| 20 | 58203663 | 58422766 | PHACTR3 | | PHACTR3 | PHACTR3 | PHACTR3 | |
| 20 | 58438611 | 58508718 | SYCP2 | | | | | |
| 20 | 58508818 | 58523702 | FAM217B | | | | | |
| 20 | 58511886 | 58515352 | PPP1R3D | | | | | |
| 20 | 58515443 | 58523702 | FAM217B | | | | | |
| 20 | 58533470 | 58588168 | CDH26 | | | | | |
| 20 | 58630979 | 58648008 | C20orf197 | | | | | |
| 20 | 58662740 | 58676442 | LOC729296 | | | | | |
| 20 | 58713547 | 58897314 | MIR646HG | | | | | |
| 20 | 58883531 | 58883625 | MIR646 | | | | | |
| 20 | 59044666 | 59048034 | LOC101928048 | | | | | |
| 20 | 59053168 | 59053239 | MIR4533 | | | | | |
| 20 | 59139619 | 59139683 | MIR548AG2 | | | | | |

| | | | | | | | | |
|---|---|---|---|---|---|---|---|---|
| 20 | 59654119 | 59655235 | LOC100506470 | | | | | |
| 20 | 59827481 | 60515673 | CDH4 | | | CDH4 | | |
| 20 | 60528601 | 60528718 | MIR1257 | | | | | |
| 20 | 60549853 | 60640866 | TAF4 | | | | | |
| 20 | 60639857 | 60639941 | MIR3195 | | | | | |
| 20 | 60697516 | 60710434 | LSM14B | | | | | |
| 20 | 60711782 | 60718514 | PSMA7 | | | | | |
| 20 | 60718775 | 60757566 | SS18L1 | | | | | |
| 20 | 60758080 | 60777810 | MTG2 | | | | | |
| 20 | 60790016 | 60795323 | HRH3 | | | HRH3 | | |
| 20 | 60813540 | 60871269 | OSBPL2 | | | | | |
| 20 | 60877951 | 60883918 | ADRM1 | | | | | |
| 20 | 60884115 | 60942368 | LAMA5 | | | | | |
| 20 | 60907542 | 60907613 | MIR4758 | | | | | |
| 20 | 60928065 | 60931534 | LAMA5 | | | | | |
| 20 | 60962120 | 60963576 | RPS21 | | | | | |
| 20 | 60963685 | 60982339 | CABLES2 | | | | | |
| 20 | 60985292 | 61002629 | RBBP8NL | | | | | |
| 20 | 61038552 | 61051026 | GATA5 | | | | | |
| 20 | 61141437 | 61148768 | C20orf166 | | | | | |
| 20 | 61147659 | 61151583 | MIR1 | | | | | |
| 20 | 61162118 | 61162220 | MIR133A2 | | | | | |
| 20 | 61273796 | 61297973 | SLCO4A1 | | | | | |
| 20 | 61340188 | 61394123 | NTSR1 | | | NTSR1 | | |
| 20 | 61405472 | 61408208 | LINC00659 | | | | | |
| 20 | 61427804 | 61431945 | MRGBP | | | | | |
| 20 | 61431978 | 61445352 | OGFR | | | | OGFR | |
| 20 | 61448413 | 61472511 | COL9A3 | | COL9A3 | COL9A3 | | |
| 20 | 61472365 | 61493115 | TCFL5 | | | | | |
| 20 | 61475917 | 61477543 | DPH3P1 | | | | | |
| 20 | 61482845 | 61493115 | TCFL5 | | | | | |
| 20 | 61509089 | 61569304 | DIDO1 | | | | | |
| 20 | 61569440 | 61579827 | GID8 | | | | | |
| 20 | 61583998 | 61599949 | SLC17A9 | | | | | |
| 20 | 61637330 | 61638387 | BHLHE23 | | | | | |
| 20 | 61640734 | 61716423 | LOC63930 | | | | | |
| 20 | 61665568 | 61668380 | LINC00029 | | | | | |
| 20 | 61669362 | 61685215 | LINC01056 | | | | | |
| 20 | 61726844 | 61733671 | HAR1B | | | | | |
| 20 | 61732643 | 61735737 | HAR1A | | | | | |
| 20 | 61809851 | 61809938 | MIR124 | | | | | |
| 20 | 61826781 | 61847538 | YTHDF1 | | | | | |
| 20 | 61867234 | 61871859 | BIRC7 | | BIRC7 | BIRC7 | | |
| 20 | 61870130 | 61870194 | MIR3196 | | | | | |
| 20 | 61872135 | 61885892 | NKAIN4 | | | | | |
| 20 | 61885329 | 61892967 | FLJ16779 | | | | | |
| 20 | 61904136 | 61921142 | ARFGAP1 | | | | | |
| 20 | 61918159 | 61918218 | MIR4326 | | | | | |
| 20 | 61924537 | 61962285 | COL20A1 | | | | | |
| 20 | 61974661 | 61992748 | CHRNA4 | | | CHRNA4 | CHRNA4 | CHRNA4 |
| 20 | 61991339 | 62002529 | LOC100130587 | | | | | |
| 20 | 62037541 | 62103993 | KCNQ2 | | KCNQ2 | KCNQ2 | | |
| 20 | 62119364 | 62130668 | EEF1A2 | | EEF1A2 | | | |
| 20 | 62152132 | 62153524 | PPDPF | | | | | |
| 20 | 62159775 | 62168723 | PTK6 | | | | | |
| 20 | 62171276 | 62178857 | SRMS | | | | | |
| 20 | 62184372 | 62188060 | C20orf195 | | | | | |
| 20 | 62189438 | 62205592 | HELZ2 | | | | | |
| 20 | 62218946 | 62258454 | GMEB2 | | | | | |
| 20 | 62258587 | 62260177 | LOC100505771 | | | | | |
| 20 | 62271057 | 62284963 | STMN3 | | | | | |
| 20 | 62289162 | 62327606 | RTEL1 | | | | | |
| 20 | 62289162 | 62330051 | RTEL1-TNFRSF6B | | | | | |
| 20 | 62328003 | 62330051 | TNFRSF6B | | | | | |
| 20 | 62329994 | 62339365 | ARFRP1 | | | | | |
| 20 | 62338793 | 62367494 | ZGPAT | | | | | |

| | | | | | | | | |
|---|---|---|---|---|---|---|---|---|
| 20 | 62367052 | 62370460 | LIME1 | | | | | |
| 20 | 62371210 | 62375403 | SLC2A4RG | | | | | |
| 20 | 62375020 | 62454360 | ZBTB46 | | | | | |
| 20 | 62492565 | 62494341 | ABHD16B | | | | | |
| 20 | 62496580 | 62522898 | TPD52L2 | | | | | |
| 20 | 62526454 | 62567384 | DNAJC5 | | DNAJC5 | DNAJC5 | DNAJC5 | |
| 20 | 62550801 | 62551292 | MIR941 | | | | | |
| 20 | 62571181 | 62587800 | UCKL1 | | | | | |
| 20 | 62572817 | 62572897 | MIR1914 | | | | | |
| 20 | 62573983 | 62574079 | MIR647 | | | | | |
| 20 | 62584736 | 62588338 | UCKL1 | | | | | |
| 20 | 62588056 | 62601223 | ZNF512B | | | | | |
| 20 | 62605465 | 62610995 | SAMD10 | | | | | |
| 20 | 62612430 | 62664453 | PRPF6 | | | | | |
| 20 | 62665696 | 62671315 | LINC00176 | | | | | |
| 20 | 62679078 | 62680979 | SOX18 | | | | | |
| 20 | 62688438 | 62703700 | TCEA2 | | | | | |
| 20 | 62704534 | 62711324 | RGS19 | | | | | |
| 20 | 62708307 | 62708363 | MIR6813 | | | | | |
| 20 | 62711434 | 62731996 | OPRL1 | | | | | |
| 20 | 62714732 | 62715712 | LKAAEAR1 | | | | | |
| 20 | 62716368 | 62731996 | OPRL1 | | | | | |
| 20 | 62737182 | 62738184 | NPBWR2 | | | | | |
| 20 | 62795826 | 62873606 | MYT1 | | | | | |
| 20 | 62887047 | 62907579 | PCMTD2 | | | | | |
| 20 | 62921737 | 62934707 | LINC00266-1 | | | | | |
| 21 | 9825831 | 9826011 | MIR3648 | | | | | |
| 21 | 9826202 | 9826263 | MIR3687 | | | | | |
| 21 | 9907188 | 9968594 | TEKT4P2 | | | | | |
| 21 | 10906186 | 10990943 | TPTE | | | | | |
| 21 | 11020841 | 11098925 | BAGE2 | | | | | |
| 21 | 11020841 | 11098925 | BAGE3 | | | | | |
| 21 | 11020841 | 11098925 | BAGE4 | | | | | |
| 21 | 11020841 | 11098925 | BAGE5 | | | | | |
| 21 | 11057795 | 11098937 | BAGE | | | | | |
| 21 | 14410486 | 14490571 | ANKRD30BP2 | | | | | |
| 21 | 14778704 | 14778781 | MIR3156 | | | | | |
| 21 | 14918353 | 14930781 | LOC102724188 | | | | | |
| 21 | 14982497 | 15013906 | POTED | | | | | |
| 21 | 15017095 | 15017171 | MIR3118 | | | | | |
| 21 | 15096509 | 15096595 | MIR8069 | | | | | |
| 21 | 15215453 | 15220685 | CYP4F29P | | | | | |
| 21 | 15316095 | 15352765 | ANKRD20A11P | | | | | |
| 21 | 15481134 | 15583212 | LIPI | | | | | |
| 21 | 15588465 | 15600693 | RBM11 | | | | | |
| 21 | 15646119 | 15673692 | ABCC13 | | | | | |
| 21 | 15743436 | 15755509 | HSPA13 | | | | | |
| 21 | 15857548 | 15970624 | SAMSN1 | | | | | |
| 21 | 15963712 | 16015428 | LOC388813 | | | | | |
| 21 | 16333555 | 16437126 | NRIP1 | | | | | |
| 21 | 17102343 | 17252390 | USP25 | | | | | |
| 21 | 17442841 | 17982094 | MIR99AHG | | | | | |
| 21 | 17911408 | 17911489 | MIR99A | | | | | |
| 21 | 17912147 | 17912231 | MIRLET7C | | | | | |
| 21 | 17962556 | 17962645 | MIR125B2 | | | | | |
| 21 | 18811207 | 18821503 | LINC01549 | | | | | |
| 21 | 18885223 | 18965897 | CXADR | | | | | |
| 21 | 18965967 | 18985268 | BTG3 | | | | | |
| 21 | 19149720 | 19164826 | C21orf91-OT1 | | | | | |
| 21 | 19161283 | 19191703 | C21orf91 | | | | | |
| 21 | 19207988 | 19639687 | CHODL | | | | | |
| 21 | 19641432 | 19775970 | TMPRSS15 | | | | | |
| 21 | 19933582 | 20132130 | MIR548XHG | | | | | |
| 21 | 22114907 | 22175426 | LINC00320 | | | | | |
| 21 | 22370632 | 22912517 | NCAM2 | | | | | |
| 21 | 23095612 | 23109639 | LINC00317 | | | | | |

| | | | | | | | | | |
|---|---|---|---|---|---|---|---|---|---|
| 21 | 23119304 | 23169735 | LINC01425 | | | | | |
| 21 | 23381476 | 23470778 | LOC101927843 | | | | | |
| 21 | 23470935 | 23488847 | LINC00308 | | | | | |
| 21 | 24733425 | 24757156 | D21S2088E | | | | | |
| 21 | 25676862 | 25693690 | LOC101927869 | | | | | |
| 21 | 26212863 | 26430056 | LOC339622 | | | | | |
| 21 | 26758132 | 26804013 | LINC00158 | | | | | |
| 21 | 26934456 | 26947480 | MIR155HG | | | | | |
| 21 | 26946291 | 26946356 | MIR155 | | | | | |
| 21 | 26955086 | 26955536 | LINC00515 | | | | | |
| 21 | 26957967 | 26979801 | MRPL39 | | | MRPL39 | | |
| 21 | 27011593 | 27089874 | JAM2 | | | | | |
| 21 | 27096790 | 27107965 | ATP5J | | | | | |
| 21 | 27107257 | 27144771 | GABPA | | | | | |
| 21 | 27252860 | 27543446 | APP | APP | APP | APP | | APP |
| 21 | 27838527 | 27945581 | CYYR1 | | | | | |
| 21 | 28208605 | 28217728 | ADAMTS1 | | | ADAMTS1 | | |
| 21 | 28290230 | 28339439 | ADAMTS5 | | | | | |
| 21 | 28326279 | 28326362 | MIR4759 | | | | | |
| 21 | 28659821 | 29283529 | MIR5009 | | | | | |
| 21 | 29094697 | 29123552 | LINC00113 | | | | | |
| 21 | 29385681 | 29395528 | LINC00314 | | | | | |
| 21 | 29420732 | 29475583 | LOC284825 | | | | | |
| 21 | 29488412 | 29600986 | LOC101927973 | | | | | |
| 21 | 29911639 | 29912677 | LINC00161 | | | | | |
| 21 | 30244512 | 30257695 | N6AMT1 | | | | | |
| 21 | 30300465 | 30365277 | LTN1 | | | | | |
| 21 | 30378079 | 30391685 | RWDD2B | | | | | |
| 21 | 30396937 | 30426807 | USP16 | | | | | |
| 21 | 30428642 | 30446118 | CCT8 | | | | | |
| 21 | 30449791 | 30548210 | MAP3K7CL | | | | | |
| 21 | 30565814 | 30660526 | LINC00189 | | | | | |
| 21 | 30671115 | 30734217 | BACH1 | | | | | |
| 21 | 30744820 | 30746300 | BACH1-IT2 | | | | | |
| 21 | 30909253 | 31136325 | GRIK1 | | | GRIK1 | | |
| 21 | 31538240 | 31538971 | CLDN17 | | | | | |
| 21 | 31581468 | 31584101 | LINC00307 | | | | | |
| 21 | 31586323 | 31588469 | CLDN8 | | | | | |
| 21 | 31653626 | 31655276 | KRTAP24-1 | | | | | |
| 21 | 31661462 | 31661832 | KRTAP25-1 | | | | | |
| 21 | 31691449 | 31692607 | KRTAP26-1 | | | | | |
| 21 | 31709330 | 31710012 | KRTAP27-1 | | | | | |
| 21 | 31720716 | 31720924 | KRTAP23-1 | | | | | |
| 21 | 31743708 | 31744557 | KRTAP13-2 | | | | | |
| 21 | 31747611 | 31747696 | MIR4327 | | | | | |
| 21 | 31768391 | 31769138 | KRTAP13-1 | | | | | |
| 21 | 31797710 | 31798230 | KRTAP13-3 | | | | | |
| 21 | 31802593 | 31803076 | KRTAP13-4 | | | | | |
| 21 | 31812645 | 31813098 | KRTAP15-1 | | | | | |
| 21 | 31852363 | 31852636 | KRTAP19-1 | | | | | |
| 21 | 31859508 | 31859667 | KRTAP19-2 | | | | | |
| 21 | 31863781 | 31864275 | KRTAP19-3 | | | | | |
| 21 | 31869173 | 31869428 | KRTAP19-4 | | | | | |
| 21 | 31874189 | 31874408 | KRTAP19-5 | | | | | |
| 21 | 31913853 | 31914183 | KRTAP19-6 | | | | | |
| 21 | 31933416 | 31933608 | KRTAP19-7 | | | | | |
| 21 | 31962423 | 31962716 | KRTAP22-2 | | | | | |
| 21 | 31964758 | 31965374 | KRTAP6-3 | | | | | |
| 21 | 31971004 | 31971193 | KRTAP6-2 | | | | | |
| 21 | 31973439 | 31973586 | KRTAP22-1 | | | | | |
| 21 | 31986004 | 31986223 | KRTAP6-1 | | | | | |
| 21 | 31988773 | 31988944 | KRTAP20-1 | | | | | |
| 21 | 31992945 | 31993169 | KRTAP20-4 | | | | | |
| 21 | 32007582 | 32007780 | KRTAP20-2 | | | | | |
| 21 | 32015182 | 32015455 | KRTAP20-3 | | | | | |
| 21 | 32090842 | 32091095 | KRTAP21-3 | | | | | |

| | | | | | | | | | |
|---|---|---|---|---|---|---|---|---|---|
| 21 | 32119268 | 32119520 | KRTAP21-2 | | | | | | |
| 21 | 32127456 | 32127696 | KRTAP21-1 | | | | | | |
| 21 | 32185014 | 32185570 | KRTAP8-1 | | | | | | |
| 21 | 32201357 | 32202051 | KRTAP7-1 | | | | | | |
| 21 | 32252963 | 32253874 | KRTAP11-1 | | | | | | |
| 21 | 32410477 | 32410795 | KRTAP19-8 | | | | | | |
| 21 | 32490735 | 32931290 | TIAM1 | | | | | | |
| 21 | 33031934 | 33041243 | SOD1 | | | | SOD1 | | |
| 21 | 33043312 | 33104431 | SCAF4 | | | | | | |
| 21 | 33245627 | 33376377 | HUNK | | | | | | |
| 21 | 33452793 | 33458608 | LINC00159 | | | | | | |
| 21 | 33640529 | 33651376 | MIS18A | | | | | | |
| 21 | 33664123 | 33687094 | MRAP | | | | | | |
| 21 | 33683329 | 33765312 | URB1 | | | | | | |
| 21 | 33749495 | 33749631 | SNORA80A | | | | | | |
| 21 | 33765441 | 33766266 | URB1 | | | | | | |
| 21 | 33784744 | 33887710 | EVA1C | | | | | | |
| 21 | 33947150 | 33957845 | TCP10L | | | | | | |
| 21 | 33973983 | 33984918 | C21orf59 | | | | | | |
| 21 | 34001068 | 34100351 | SYNJ1 | | | SYNJ1 | SYNJ1 | | |
| 21 | 34100424 | 34144169 | PAXBP1 | | | | | | |
| 21 | 34144410 | 34186053 | C21orf62 | | | | | | |
| 21 | 34398215 | 34401503 | OLIG2 | | | OLIG2 | OLIG2 | OLIG2 | |
| 21 | 34430134 | 34437385 | LINC00945 | | | | | | |
| 21 | 34442449 | 34444728 | OLIG1 | | | | | | |
| 21 | 34484009 | 34496006 | LOC101928107 | | | | | | |
| 21 | 34537775 | 34542541 | LINC01548 | | | | | | |
| 21 | 34602199 | 34636818 | IFNAR2 | | | | | | |
| 21 | 34637936 | 34669539 | IL10RB | | | | | | |
| 21 | 34697213 | 34732128 | IFNAR1 | | | | | | |
| 21 | 34775201 | 34809828 | IFNGR2 | | | | | | |
| 21 | 34804792 | 34852316 | TMEM50B | | | | | | |
| 21 | 34860361 | 34864030 | DNAJC28 | | | | | | |
| 21 | 34876237 | 34914464 | GART | | | | | | |
| 21 | 34915343 | 34949820 | SON | | | | | | |
| 21 | 34922967 | 34923034 | MIR6501 | | | | | | |
| 21 | 34949858 | 34961014 | DONSON | | | | | | |
| 21 | 34961647 | 35014160 | CRYZL1 | | | | | | |
| 21 | 35014783 | 35261609 | ITSN1 | | | | ITSN1 | ITSN1 | ITSN1 |
| 21 | 35275756 | 35288158 | ATP5O | | | | ATP5O | | |
| 21 | 35303425 | 35343487 | LINC00649 | | | | | | |
| 21 | 35348682 | 35349992 | LOC101928126 | | | | | | |
| 21 | 35445822 | 35478561 | SLC5A3 | | | | | | |
| 21 | 35445822 | 35515334 | MRPS6 | | | | MRPS6 | | |
| 21 | 35552977 | 35562220 | LINC00310 | | | | | | |
| 21 | 35736322 | 35743440 | KCNE2 | | | | | KCNE2 | |
| 21 | 35747748 | 35761452 | SMIM11A | | | | | | |
| 21 | 35747778 | 35761452 | SMIM11B | | | | | | |
| 21 | 35772409 | 35773370 | C21orf140 | | | | | | |
| 21 | 35818985 | 35884573 | KCNE1 | | | | KCNE1 | | |
| 21 | 35888739 | 35987441 | RCAN1 | | | RCAN1 | | | |
| 21 | 36041461 | 36090525 | CLIC6 | | | | | | |
| 21 | 36096104 | 36109479 | LINC00160 | | | | | | |
| 21 | 36118121 | 36157168 | LINC01426 | | | | | | |
| 21 | 36160097 | 36260987 | RUNX1 | | | | | | |
| 21 | 36410232 | 36411723 | RUNX1-IT1 | | | | | | |
| 21 | 36744804 | 36953062 | LOC100506403 | | | | | | |
| 21 | 37093012 | 37093106 | MIR802 | | | | | | |
| 21 | 37326976 | 37376965 | LOC101928269 | | | | | | |
| 21 | 37377617 | 37380593 | LINC01436 | | | | | | |
| 21 | 37406838 | 37432662 | SETD4 | | | | | | |
| 21 | 37441939 | 37498938 | LOC100133286 | | | | | | |
| 21 | 37442221 | 37445475 | CBR1 | | | | | | |
| 21 | 37504064 | 37528606 | CBR3 | | | | | | |
| 21 | 37536838 | 37666572 | DOPEY2 | | | | DOPEY2 | | |
| 21 | 37692486 | 37748944 | MORC3 | | | | | | |

| | | | | | | | | |
|---|---|---|---|---|---|---|---|---|
| 21 | 37757688 | 37789125 | CHAF1B | | | | | |
| 21 | 37832919 | 37948867 | CLDN14 | | | | | |
| 21 | 38071432 | 38122218 | SIM2 | | | | | |
| 21 | 38123188 | 38362545 | HLCS | | | HLCS | | |
| 21 | 38378449 | 38391958 | RIPPLY3 | | | | | |
| 21 | 38435340 | 38436366 | LOC105372795 | | | | | |
| 21 | 38437663 | 38445458 | PIGP | | | | | |
| 21 | 38445570 | 38575408 | TTC3 | | | | | |
| 21 | 38580803 | 38594037 | DSCR9 | | | | | |
| 21 | 38595725 | 38639833 | DSCR3 | | | DSCR3 | | |
| 21 | 38739858 | 38887679 | DYRK1A | | | | | |
| 21 | 38996524 | 39288741 | KCNJ6 | | KCNJ6 | KCNJ6 | | |
| 21 | 39426312 | 39493454 | DSCR4 | | | | | |
| 21 | 39493544 | 39528605 | DSCR8 | | | | | |
| 21 | 39578249 | 39580738 | DSCR10 | | | | | |
| 21 | 39601836 | 39673746 | KCNJ15 | | | KCNJ15 | | |
| 21 | 39698280 | 39717998 | LINC01423 | | | | | |
| 21 | 39739182 | 40033704 | ERG | | | | | |
| 21 | 40110878 | 40145401 | LINC00114 | | | | | |
| 21 | 40177230 | 40196878 | ETS2 | | | | | |
| 21 | 40229462 | 40230786 | LOC101928398 | | | | | |
| 21 | 40249214 | 40310353 | LOC400867 | | | | | |
| 21 | 40346354 | 40349700 | LOC101928435 | | | | | |
| 21 | 40547371 | 40555440 | PSMG1 | | | | | |
| 21 | 40557403 | 40685712 | BRWD1 | | BRWD1 | | | |
| 21 | 40685832 | 40686841 | BRWD1-IT2 | | | | | |
| 21 | 40687632 | 40695144 | BRWD1 | | BRWD1 | | | |
| 21 | 40714240 | 40721047 | HMGN1 | | | | | |
| 21 | 40752169 | 40887433 | WRB-SH3BGR | | | | | |
| 21 | 40752212 | 40769815 | WRB | | WRB | | | |
| 21 | 40777769 | 40816128 | LCA5L | | | | | |
| 21 | 40817790 | 40887433 | SH3BGR | | | | | |
| 21 | 40818935 | 40818995 | MIR6508 | | | | | |
| 21 | 40823755 | 40887433 | SH3BGR | | | | | |
| 21 | 40969074 | 41034816 | B3GALT5 | | | | | |
| 21 | 41117333 | 41174023 | IGSF5 | | | | | |
| 21 | 41239346 | 41301322 | PCP4 | | | | | |
| 21 | 41384342 | 42219039 | DSCAM | | | | | |
| 21 | 41584278 | 41584358 | MIR4760 | | | | | |
| 21 | 41755009 | 41757285 | DSCAM | | | | | |
| 21 | 41990432 | 42002693 | DSCAM-IT1 | | | | | |
| 21 | 42513426 | 42519991 | LINC00323 | | | | | |
| 21 | 42539483 | 42539556 | MIR3197 | | | | | |
| 21 | 42539727 | 42654461 | BACE2 | | | | | |
| 21 | 42547157 | 42557166 | PLAC4 | | | | | |
| 21 | 42688660 | 42729654 | FAM3B | | | | | |
| 21 | 42733949 | 42780869 | MX2 | | | | | |
| 21 | 42792484 | 42831141 | MX1 | | | | | |
| 21 | 42836477 | 42880085 | TMPRSS2 | | | | | |
| 21 | 43099461 | 43117496 | LINC00111 | | | | | |
| 21 | 43131679 | 43135935 | LINC00479 | | | | | |
| 21 | 43136595 | 43137742 | LINC00112 | | | | | |
| 21 | 43159528 | 43187249 | RIPK4 | | | | | |
| 21 | 43166931 | 43167001 | MIR6814 | | | | | |
| 21 | 43218384 | 43299591 | PRDM15 | | | | | |
| 21 | 43305218 | 43373999 | C2CD2 | | | | | |
| 21 | 43368213 | 43368391 | SNORA91 | | | | | |
| 21 | 43406939 | 43430496 | ZBTB21 | | | | | |
| 21 | 43429302 | 43445029 | ZNF295 | | | | | |
| 21 | 43483067 | 43528644 | UMODL1 | | | | | |
| 21 | 43619798 | 43717354 | ABCG1 | | | | | |
| 21 | 43731776 | 43735706 | TFF3 | | | | | |
| 21 | 43766466 | 43771208 | TFF2 | | | | | |
| 21 | 43782390 | 43786644 | TFF1 | | | | | |
| 21 | 43791995 | 43816200 | TMPRSS3 | | | TMPRSS3 | | |
| 21 | 43823970 | 43867790 | UBASH3A | | | | | |

| | | | | | | | | | |
|---|---|---|---|---|---|---|---|---|---|
| 21 | 43892596 | 43916464 | RSPH1 | | RSPH1 | | | | |
| 21 | 43919741 | 44001550 | SLC37A1 | | | | | | |
| 21 | 44019513 | 44035205 | LOC101928233 | | | | | | |
| 21 | 44073861 | 44195618 | PDE9A | | PDE9A | | | | |
| 21 | 44263189 | 44299699 | WDR4 | | | | | | |
| 21 | 44313377 | 44329773 | NDUFV3 | | | NDUFV3 | | | |
| 21 | 44337360 | 44345708 | ERVH48-1 | | | | | | |
| 21 | 44394619 | 44454041 | PKNOX1 | | | | | | |
| 21 | 44473300 | 44496472 | CBS | | CBS | CBS | | | |
| 21 | 44513065 | 44527688 | U2AF1 | | | | | | |
| 21 | 44560632 | 44561202 | FRGCA | | | | | | |
| 21 | 44560632 | 44561202 | LOC106780825 | | | | | | |
| 21 | 44589117 | 44592920 | CRYAA | | | | | | |
| 21 | 44589159 | 44592431 | LOC102724652 | | | | | | |
| 21 | 44742088 | 44751919 | LINC00322 | | | | | | |
| 21 | 44777934 | 44782229 | TCONS_00029157 | | | | | | |
| 21 | 44834397 | 44847002 | SIK1 | | | | | | |
| 21 | 44869903 | 44873771 | LINC00319 | | | | | | |
| 21 | 44881973 | 44898103 | LINC00313 | | | | | | |
| 21 | 44949071 | 45079374 | HSF2BP | | | | | | |
| 21 | 45029767 | 45029870 | MIR6070 | | | | | | |
| 21 | 45079431 | 45115960 | RRP1B | | | | | | |
| 21 | 45138977 | 45182188 | PDXK | | | | | | |
| 21 | 45193545 | 45196256 | CSTB | | | | | | |
| 21 | 45209417 | 45223983 | RRP1 | | | | | | |
| 21 | 45225638 | 45232448 | AATBC | | | | | | |
| 21 | 45285115 | 45407475 | AGPAT3 | | | | | | |
| 21 | 45432205 | 45526432 | TRAPPC10 | | | | | | |
| 21 | 45527207 | 45551063 | PWP2 | | | PWP2 | | | |
| 21 | 45553493 | 45565605 | C21orf33 | | | | | | |
| 21 | 45642877 | 45660887 | ICOSLG | | | | | | |
| 21 | 45666221 | 45682099 | DNMT3L | | | | | | |
| 21 | 45705720 | 45718102 | AIRE | | | AIRE | | | |
| 21 | 45719916 | 45747264 | PFKL | | | | | | |
| 21 | 45748826 | 45759285 | C21orf2 | | | | | | |
| 21 | 45773483 | 45845446 | TRPM2 | | TRPM2 | TRPM2 | | | |
| 21 | 45870868 | 45878739 | LRRC3 | | | | | | |
| 21 | 45917774 | 45938860 | TSPEAR | | | | | | |
| 21 | 45959067 | 45960078 | KRTAP10-1 | | | | | | |
| 21 | 45970239 | 45971388 | KRTAP10-2 | | | | | | |
| 21 | 45977905 | 45978643 | KRTAP10-3 | | | | | | |
| 21 | 45993605 | 45995248 | KRTAP10-4 | | | | | | |
| 21 | 45999331 | 46000481 | KRTAP10-5 | | | | | | |
| 21 | 46011148 | 46012386 | KRTAP10-6 | | | | | | |
| 21 | 46020496 | 46022091 | KRTAP10-7 | | | | | | |
| 21 | 46031995 | 46032871 | KRTAP10-8 | | | | | | |
| 21 | 46047039 | 46048295 | KRTAP10-9 | | | | | | |
| 21 | 46057272 | 46058372 | KRTAP10-10 | | | | | | |
| 21 | 46066330 | 46067566 | KRTAP10-11 | | | | | | |
| 21 | 46074129 | 46074576 | KRTAP12-4 | | | | | | |
| 21 | 46077848 | 46078258 | KRTAP12-3 | | | | | | |
| 21 | 46086105 | 46086844 | KRTAP12-2 | | | | | | |
| 21 | 46101490 | 46102078 | KRTAP12-1 | | | | | | |
| 21 | 46117086 | 46117959 | KRTAP10-12 | | | | | | |
| 21 | 46188494 | 46221751 | UBE2G2 | | | | | | |
| 21 | 46222155 | 46224631 | LINC01424 | | | | | | |
| 21 | 46225531 | 46238044 | SUMO3 | | | | | | |
| 21 | 46269499 | 46293818 | PTTG1IP | | | | | | |
| 21 | 46305868 | 46349595 | ITGB2 | | | ITGB2 | | | |
| 21 | 46353198 | 46359828 | LINC01547 | | | | | | |
| 21 | 46359611 | 46396904 | FAM207A | | | | | | |
| 21 | 46409778 | 46414001 | LINC00163 | | | | | | |
| 21 | 46419121 | 46424642 | LINC00162 | | | | | | |
| 21 | 46490869 | 46493126 | SSR4P1 | | | | | | |
| 21 | 46494492 | 46646478 | ADARB1 | | | | | | |
| 21 | 46683842 | 46707811 | POFUT2 | | | | | | |

| | | | | | | | | |
|---|---|---|---|---|---|---|---|---|
| 21 | 46707966 | 46717269 | LOC642852 | | | | | |
| 21 | 46758504 | 46761905 | LINC00316 | | | | | |
| 21 | 46825051 | 46933634 | COL18A1 | | | | | |
| 21 | 46898179 | 46898240 | MIR6815 | | | | | |
| 21 | 46934628 | 46962385 | SLC19A1 | | | | | |
| 21 | 47247754 | 47256333 | LOC100129027 | | | | | |
| 21 | 47269874 | 47362368 | PCBP3 | | | | | |
| 21 | 47392883 | 47394867 | LOC101928796 | | | | | |
| 21 | 47401662 | 47424963 | COL6A1 | | | | | |
| 21 | 47518032 | 47552763 | COL6A2 | | | | | |
| 21 | 47556175 | 47575481 | FTCD | | FTCD | | | |
| 21 | 47581061 | 47604373 | SPATC1L | | | | | |
| 21 | 47608359 | 47648738 | LSS | | LSS | | | |
| 21 | 47649144 | 47705308 | MCM3AP | | | | | |
| 21 | 47706243 | 47717665 | YBEY | | | | | |
| 21 | 47720343 | 47743813 | C21orf58 | | | | | |
| 21 | 47744035 | 47865682 | PCNT | | | | | |
| 21 | 47878861 | 47989926 | DIP2A | | | | | |
| 21 | 47882383 | 47889218 | DIP2A-IT1 | | | | | |
| 21 | 48018530 | 48025035 | S100B | | | | | |
| 21 | 48055506 | 48085036 | PRMT2 | | | | | |
| 22 | 16150528 | 16193009 | DUXAP8 | | | | | |
| 22 | 16157078 | 16172265 | BMS1P22 | | | | | |
| 22 | 16162065 | 16172265 | BMS1P17 | | | | | |
| 22 | 16162065 | 16172265 | BMS1P18 | | | | | |
| 22 | 16199673 | 16231289 | LINC01297 | | | | | |
| 22 | 16256331 | 16277577 | POTEH | | | | | |
| 22 | 16448823 | 16449804 | OR11H1 | | | | | |
| 22 | 17071647 | 17073700 | CCT8L2 | | | | | |
| 22 | 17082800 | 17129720 | TPTEP1 | | | | | |
| 22 | 17134598 | 17156430 | ANKRD62P1-PARP4P3 | | | | | |
| 22 | 17227758 | 17229328 | LOC101929350 | | | | | |
| 22 | 17264305 | 17302589 | XKR3 | | | | | |
| 22 | 17308363 | 17310225 | HSFY1P1 | | | | | |
| 22 | 17442826 | 17489112 | GAB4 | | | | | |
| 22 | 17517459 | 17539682 | CECR7 | | | | | |
| 22 | 17565848 | 17596584 | IL17RA | | | | | |
| 22 | 17597188 | 17602257 | CECR6 | | | | | |
| 22 | 17602484 | 17612994 | LOC100996342 | | | | | |
| 22 | 17618409 | 17646335 | CECR5 | | | | | |
| 22 | 17659679 | 17702744 | CECR1 | | | | | |
| 22 | 17737749 | 17747623 | CECR3 | | | | | |
| 22 | 17840838 | 18037856 | CECR2 | | CECR2 | | | |
| 22 | 18043138 | 18073656 | SLC25A18 | | | SLC25A18 | | |
| 22 | 18062922 | 18071958 | LOC101929372 | | | | | |
| 22 | 18074902 | 18111588 | ATP6V1E1 | | ATP6V1E1 | | | |
| 22 | 18121349 | 18213621 | BCL2L13 | | | | | |
| 22 | 18216905 | 18257431 | BID | | | BID | | |
| 22 | 18246945 | 18247025 | MIR3198 | | | | | |
| 22 | 18260055 | 18262247 | LINC00528 | | | | | |
| 22 | 18270415 | 18389652 | MICAL3 | | | | | |
| 22 | 18463633 | 18463727 | MIR648 | | | | | |
| 22 | 18512150 | 18520734 | FLJ41941 | | | | | |
| 22 | 18560685 | 18573797 | PEX26 | | | PEX26 | | |
| 22 | 18593452 | 18614498 | TUBA8 | | | TUBA8 | | |
| 22 | 18632757 | 18660162 | USP18 | | | | | |
| 22 | 18761201 | 18779474 | GGT3P | | | | | |
| 22 | 18893735 | 18899601 | DGCR6 | | | | | |
| 22 | 18900286 | 18924066 | PRODH | | PRODH | PRODH | | |
| 22 | 18958010 | 19018755 | DGCR5 | | | | | |
| 22 | 19005346 | 19007761 | DGCR9 | | | | | |
| 22 | 19010136 | 19011063 | DGCR10 | | | | | |
| 22 | 19023794 | 19109967 | DGCR2 | | | | | |
| 22 | 19033674 | 19035888 | DGCR11 | | | | | |
| 22 | 19117791 | 19132190 | DGCR14 | | | | | |
| 22 | 19118320 | 19120136 | TSSK2 | | | | | |

| | | | | | | | | |
|---|---|---|---|---|---|---|---|---|
| 22 | 19136503 | 19137796 | GSC2 | | | | | |
| 22 | 19159218 | 19160345 | LINC01311 | | | | | |
| 22 | 19163087 | 19166376 | SLC25A1 | | | | | |
| 22 | 19166986 | 19279239 | CLTCL1 | | | | CLTCL1 | |
| 22 | 19318223 | 19419219 | HIRA | | | HIRA | | |
| 22 | 19419424 | 19423601 | MRPL40 | | | | | |
| 22 | 19428409 | 19435755 | C22orf39 | | | | | |
| 22 | 19437463 | 19466738 | UFD1L | | | | | |
| 22 | 19467348 | 19508135 | CDC45 | | | | | |
| 22 | 19510546 | 19512860 | CLDN5 | | | | | |
| 22 | 19553652 | 19554362 | LINC00895 | | | | | |
| 22 | 19701986 | 19710845 | SEPT5 | | | | | |
| 22 | 19704742 | 19712297 | SEPT5-GP1BB | | | | | |
| 22 | 19705957 | 19710845 | SEPT5 | | | | | |
| 22 | 19705991 | 19712297 | SEPT5-GP1BB | | | | | |
| 22 | 19711065 | 19712297 | GP1BB | | | | | |
| 22 | 19744225 | 19771112 | TBX1 | | | TBX1 | | |
| 22 | 19775933 | 19842462 | GNB1L | | | | | |
| 22 | 19833660 | 19842371 | C22orf29 | | | | | |
| 22 | 19863040 | 19929515 | TXNRD2 | | | TXNRD2 | | |
| 22 | 19929262 | 19957498 | COMT | COMT | | COMT | COMT | |
| 22 | 19951275 | 19951357 | MIR4761 | | | | | |
| 22 | 19957401 | 20004309 | ARVCF | | | | | |
| 22 | 20004522 | 20053449 | TANGO2 | | | | | |
| 22 | 20020661 | 20020743 | MIR185 | | | | | |
| 22 | 20024282 | 20053449 | TANGO2 | | | | | |
| 22 | 20067754 | 20099400 | DGCR8 | | | | | |
| 22 | 20073268 | 20073356 | MIR3618 | | | | | |
| 22 | 20073580 | 20073665 | MIR1306 | | | | | |
| 22 | 20099388 | 20104818 | TRMT2A | | | | | |
| 22 | 20102208 | 20102274 | MIR6816 | | | | | |
| 22 | 20103460 | 20114880 | RANBP1 | | | | | |
| 22 | 20119363 | 20135530 | ZDHHC8 | | | | | |
| 22 | 20136108 | 20137431 | CCDC188 | | | | | |
| 22 | 20186252 | 20192441 | LOC284865 | | | | | |
| 22 | 20193854 | 20196060 | LINC00896 | | | | | |
| 22 | 20228937 | 20255816 | RTN4R | | RTN4R | | | |
| 22 | 20236656 | 20236734 | MIR1286 | | | | | |
| 22 | 20301760 | 20307628 | DGCR6L | | | | | |
| 22 | 20377668 | 20380440 | TMEM191B | | | | | |
| 22 | 20383730 | 20398695 | PI4KAP1 | | | | | |
| 22 | 20455993 | 20461786 | RIMBP3 | | | | | |
| 22 | 20748404 | 20762753 | ZNF74 | | | | | |
| 22 | 20778873 | 20792146 | SCARF2 | | | | | |
| 22 | 20795805 | 20850170 | KLHL22 | | | | | |
| 22 | 20861828 | 20941919 | MED15 | | | MED15 | | |
| 22 | 21043842 | 21046009 | POM121L4P | | | | | |
| 22 | 21055401 | 21058891 | TMEM191A | | | | | |
| 22 | 21061978 | 21213100 | PI4KA | | PI4KA | | | |
| 22 | 21128382 | 21142008 | SERPIND1 | | | | | |
| 22 | 21213291 | 21245501 | SNAP29 | | | | | SNAP29 |
| 22 | 21271713 | 21308037 | CRKL | | | CRKL | | |
| 22 | 21311379 | 21318968 | LOC101928891 | | | | | |
| 22 | 21319417 | 21335649 | AIFM3 | | | | | |
| 22 | 21336557 | 21353326 | LZTR1 | | | | | |
| 22 | 21354060 | 21364663 | THAP7 | | | | | |
| 22 | 21362495 | 21368576 | TUBA3FP | | | | | |
| 22 | 21369441 | 21382302 | P2RX6 | | | | | |
| 22 | 21383006 | 21386847 | SLC7A4 | | | | | |
| 22 | 21388464 | 21388561 | MIR649 | | | | | |
| 22 | 21396680 | 21398538 | P2RX6P | | | | | |
| 22 | 21400248 | 21418457 | LRRC74B | | | | | |
| 22 | 21457304 | 21476575 | BCRP2 | | | | | |
| 22 | 21521191 | 21546445 | FAM230B | | | | | |
| 22 | 21636713 | 21652015 | POM121L8P | | | | | |
| 22 | 21737662 | 21743455 | RIMBP3C | | | | | |

| | | | | | | | | | |
|---|---|---|---|---|---|---|---|---|---|
| 22 | 21738039 | 21743455 | RIMBP3B | | | | | | |
| 22 | 21771692 | 21805750 | HIC2 | | | | | | |
| 22 | 21821458 | 21824224 | TMEM191C | | | | | | |
| 22 | 21827286 | 21871780 | PI4KAP2 | | | | | | |
| 22 | 21899957 | 21905373 | RIMBP3B | | | | | | |
| 22 | 21899957 | 21905750 | RIMBP3C | | | | | | |
| 22 | 21903735 | 21978323 | UBE2L3 | | | | UBE2L3 | | |
| 22 | 21982377 | 21984340 | YDJC | | | | | | |
| 22 | 21987085 | 21991616 | CCDC116 | | | | | | |
| 22 | 21996541 | 21998588 | SDF2L1 | | | | | | |
| 22 | 22007269 | 22007347 | MIR301B | | | | | | |
| 22 | 22007592 | 22007674 | MIR130B | | | | | | |
| 22 | 22020272 | 22052202 | PPIL2 | | | | | | |
| 22 | 22051825 | 22090123 | YPEL1 | | | | | | |
| 22 | 22113946 | 22221970 | MAPK1 | | | MAPK1 | MAPK1 | | |
| 22 | 22273791 | 22307250 | PPM1F | | | | | | |
| 22 | 22311396 | 22337240 | TOP3B | | | | | | |
| 22 | 22599191 | 22599927 | VPREB1 | | | | | | |
| 22 | 22652462 | 22677324 | BMS1P20 | | | | | | |
| 22 | 22838770 | 22863505 | ZNF280B | | | | | | |
| 22 | 22868060 | 22874624 | ZNF280A | | | | | | |
| 22 | 22890117 | 22901768 | PRAME | | | | | | |
| 22 | 22901749 | 22909004 | LL22NC03-63E9.3 | | | | | | |
| 22 | 22974027 | 22987012 | POM121L1P | | | | | | |
| 22 | 22987090 | 22990368 | GGTLC2 | | | | | | |
| 22 | 23165269 | 23165365 | MIR650 | | | | | | |
| 22 | 23228446 | 23228559 | MIR5571 | | | | | | |
| 22 | 23229959 | 23238013 | IGLL5 | | | | | | |
| 22 | 23401592 | 23484241 | RSPH14 | | | | | | |
| 22 | 23412668 | 23467221 | GNAZ | | | | | | |
| 22 | 23487512 | 23506531 | RAB36 | | | | RAB36 | | |
| 22 | 23522551 | 23660224 | BCR | | | BCR | BCR | | |
| 22 | 23604953 | 23607186 | FBXW4P1 | | | | | | |
| 22 | 23701792 | 23724313 | CES5AP1 | | | | | | |
| 22 | 23732791 | 23744799 | ZDHHC8P1 | | | | | | |
| 22 | 23775750 | 23777258 | LOC101929374 | | | | | | |
| 22 | 23804268 | 23829167 | LOC388882 | | | | | | |
| 22 | 23915312 | 23922495 | IGLL1 | | | | | | |
| 22 | 23950638 | 23974508 | DRICH1 | | | | | | |
| 22 | 23980674 | 24059610 | GUSBP11 | | | | | | |
| 22 | 24033047 | 24041363 | RGL4 | | | | | | |
| 22 | 24083770 | 24093279 | ZNF70 | | | | | | |
| 22 | 24094929 | 24096630 | VPREB3 | | | | | | |
| 22 | 24105207 | 24108050 | C22orf15 | | | | | | |
| 22 | 24108020 | 24110159 | CHCHD10 | | | | | | |
| 22 | 24115005 | 24126503 | MMP11 | | | | | | |
| 22 | 24129117 | 24176705 | SMARCB1 | | | | | | |
| 22 | 24176689 | 24181199 | DERL3 | | | | | | |
| 22 | 24198889 | 24228299 | SLC2A11 | | | | | | |
| 22 | 24235896 | 24237409 | MIF | | | | MIF | | |
| 22 | 24299600 | 24303368 | GSTT2B | | | | | | |
| 22 | 24299600 | 24303488 | GSTT2 | | | | | | |
| 22 | 24309025 | 24314748 | DDTL | | | | | | |
| 22 | 24313553 | 24322019 | DDT | | | | | | |
| 22 | 24322218 | 24326106 | GSTT2 | | | | | | |
| 22 | 24322338 | 24326106 | GSTT2B | | | | | | |
| 22 | 24340594 | 24347258 | GSTTP1 | | | | | | |
| 22 | 24373116 | 24374043 | LOC391322 | | | | | | |
| 22 | 24375962 | 24384311 | GSTT1 | | | | | | |
| 22 | 24385937 | 24401899 | GSTTP2 | | | | | | |
| 22 | 24407764 | 24574596 | CABIN1 | | | | | | |
| 22 | 24577443 | 24585074 | SUSD2 | | | | | | |
| 22 | 24615621 | 24641110 | GGT5 | | | | | | |
| 22 | 24647588 | 24661492 | POM121L9P | | | | | | |
| 22 | 24666784 | 24813708 | SPECC1L | | | | | | |
| 22 | 24666784 | 24838328 | SPECC1L-ADORA2A | | | | | | |

| | | | | | | | | |
|---|---|---|---|---|---|---|---|---|
| 22 | 24738075 | 24813708 | SPECC1L | | | | | |
| 22 | 24819564 | 24891042 | ADORA2A | | | ADORA2A | | |
| 22 | 24891250 | 24922553 | UPB1 | | | | | |
| 22 | 24936390 | 24951903 | GUCD1 | | | | | |
| 22 | 24951617 | 24970932 | SNRPD3 | | | | | |
| 22 | 24979717 | 25024972 | GGT1 | | | | | |
| 22 | 24981590 | 24989035 | LRRC75B | | | | | |
| 22 | 24999123 | 25024972 | GGT1 | | | | | |
| 22 | 25028881 | 25049327 | BCRP3 | | | | | |
| 22 | 25041132 | 25055114 | POM121L10P | | | | | |
| 22 | 25115000 | 25170687 | PIWIL3 | | | | | |
| 22 | 25160467 | 25161986 | TOP1P2 | | | | | |
| 22 | 25202187 | 25322813 | SGSM1 | | | | | |
| 22 | 25331207 | 25335314 | TMEM211 | | | | | |
| 22 | 25423940 | 25593415 | KIAA1671 | | | | | |
| 22 | 25498383 | 25508659 | LOC100128531 | | | | | |
| 22 | 25595816 | 25603326 | CRYBB3 | | | | | |
| 22 | 25615611 | 25627836 | CRYBB2 | | | | | |
| 22 | 25714223 | 25716193 | IGLL3P | | | | | |
| 22 | 25747384 | 25777544 | LRP5L | | | | | |
| 22 | 25844053 | 25857645 | CRYBB2P1 | | | | | |
| 22 | 25851612 | 25851678 | MIR6817 | | | | | |
| 22 | 25960860 | 26125258 | ADRBK2 | | | | | |
| 22 | 26138110 | 26427007 | MYO18B | | | | | |
| 22 | 26565439 | 26779563 | SEZ6L | | | | | |
| 22 | 26825279 | 26840978 | ASPHD2 | | | | | |
| 22 | 26846848 | 26879829 | HPS4 | | | | | |
| 22 | 26879849 | 26887904 | SRRD | | | | | |
| 22 | 26887893 | 26908437 | TFIP11 | | | | | |
| 22 | 26921713 | 26986089 | TPST2 | | | | | |
| 22 | 26951177 | 26951289 | MIR548J | | | | | |
| 22 | 26995361 | 27013991 | CRYBB1 | | | | | |
| 22 | 27017927 | 27026636 | CRYBA4 | | | | | |
| 22 | 27053445 | 27072440 | MIAT | | | | | |
| 22 | 27068805 | 27176856 | MIATNB | | | | | |
| 22 | 27299254 | 27316573 | LINC01422 | | | | | |
| 22 | 27444105 | 27456480 | LOC284898 | | | | | |
| 22 | 27583477 | 27585177 | LOC105372977 | | | | | |
| 22 | 27706611 | 27713801 | LOC100507657 | | | | | |
| 22 | 28144264 | 28197486 | MN1 | | | | | |
| 22 | 28247656 | 28315294 | PITPNB | | | | | |
| 22 | 28315363 | 28398667 | TTC28 | | | | | |
| 22 | 28316512 | 28316599 | MIR3199 | | | | | |
| 22 | 28374001 | 29075853 | TTC28 | | | | | |
| 22 | 28855856 | 28855936 | MIR5739 | | | | | |
| 22 | 29083730 | 29137822 | CHEK2 | | | CHEK2 | | |
| 22 | 29138019 | 29153506 | HSCB | | | | | |
| 22 | 29168661 | 29185289 | CCDC117 | | | | | |
| 22 | 29190547 | 29196560 | XBP1 | | | | | |
| 22 | 29279754 | 29427464 | ZNRF3 | | | | | |
| 22 | 29454659 | 29457907 | C22orf31 | | | | | |
| 22 | 29469065 | 29564321 | KREMEN1 | | | | | |
| 22 | 29601900 | 29655586 | EMID1 | | | | | |
| 22 | 29655843 | 29663914 | RHBDD3 | | | | | |
| 22 | 29663997 | 29696515 | EWSR1 | | | EWSR1 | | |
| 22 | 29702984 | 29708778 | GAS2L1 | | GAS2L1 | | | |
| 22 | 29708921 | 29711745 | RASL10A | | | | | |
| 22 | 29723668 | 29784572 | AP1B1 | | | AP1B1 | | |
| 22 | 29729146 | 29729256 | MIR3653 | | | | | |
| 22 | 29729151 | 29729247 | SNORD125 | | | | | |
| 22 | 29833003 | 29838118 | RFPL1S | | | | | |
| 22 | 29834571 | 29838444 | RFPL1 | | | | | |
| 22 | 29876180 | 29887277 | NEFH | | NEFH | NEFH | | |
| 22 | 29904155 | 29949736 | THOC5 | | | | | |
| 22 | 29950797 | 29977326 | NIPSNAP1 | | | | | |
| 22 | 29999544 | 30094589 | NF2 | | | NF2 | | |

| | | | | | | | | |
|---|---|---|---|---|---|---|---|---|
| 22 | 30116343 | 30127820 | CABP7 | | | | | |
| 22 | 30126944 | 30163000 | ZMAT5 | | | | | |
| 22 | 30163357 | 30166402 | UQCR10 | | | | | |
| 22 | 30184596 | 30234293 | ASCC2 | | | | | |
| 22 | 30279157 | 30426857 | MTMR3 | | | | | |
| 22 | 30403037 | 30403102 | MIR6818 | | | | | |
| 22 | 30404730 | 30573062 | HORMAD2 | | | | | |
| 22 | 30636435 | 30642840 | LIF | | | | LIF | |
| 22 | 30658816 | 30662832 | OSM | | | | | |
| 22 | 30681106 | 30685616 | GATSL3 | | | | | |
| 22 | 30687978 | 30722955 | TBC1D10A | | | | | |
| 22 | 30727976 | 30752936 | SF3A1 | | | | | |
| 22 | 30752623 | 30774644 | CCDC157 | | | | | |
| 22 | 30764799 | 30773894 | KIAA1656 | | | | | |
| 22 | 30774802 | 30783302 | RNF215 | | | | | |
| 22 | 30792929 | 30821291 | SEC14L2 | | | | | |
| 22 | 30821610 | 30825041 | MTFP1 | | | | | |
| 22 | 30855215 | 30868034 | SEC14L3 | | | | | |
| 22 | 30877276 | 30877743 | SDC4P | | | | | |
| 22 | 30884897 | 30901698 | SEC14L4 | | | | | |
| 22 | 30920916 | 30942669 | SEC14L6 | | | | | |
| 22 | 30950621 | 30970574 | GAL3ST1 | | | | | |
| 22 | 30972611 | 31003000 | PES1 | | | | | |
| 22 | 31003069 | 31023047 | TCN2 | | | | | |
| 22 | 31031792 | 31043862 | SLC35E4 | | | | | |
| 22 | 31048037 | 31063877 | DUSP18 | | | | | |
| 22 | 31089768 | 31303811 | OSBP2 | | | | | |
| 22 | 31127543 | 31127628 | MIR3200 | | | | | |
| 22 | 31160239 | 31303811 | OSBP2 | | | | | |
| 22 | 31318294 | 31364483 | MORC2 | | | | | |
| 22 | 31365196 | 31375380 | TUG1 | | | | | |
| 22 | 31477281 | 31500610 | SMTN | | | | | |
| 22 | 31500762 | 31503551 | SELM | | | | | |
| 22 | 31518908 | 31530683 | INPP5J | | | | | |
| 22 | 31530792 | 31536593 | PLA2G3 | | | | | |
| 22 | 31556047 | 31556105 | MIR3928 | | | | | |
| 22 | 31556137 | 31603005 | RNF185 | | | | | |
| 22 | 31608249 | 31676066 | LIMK2 | | | | | |
| 22 | 31677578 | 31688520 | PIK3IP1 | | | | | |
| 22 | 31721789 | 31742249 | PATZ1 | | | | | |
| 22 | 31731334 | 31734007 | PIK3IP1 | | | | | |
| 22 | 31736664 | 31742249 | PATZ1 | | | | | |
| 22 | 31742744 | 31747141 | LINC01521 | | | | | |
| 22 | 31795538 | 31830172 | DRG1 | | | | | |
| 22 | 31835344 | 31885874 | EIF4ENIF1 | | | | | |
| 22 | 31892124 | 32014537 | SFI1 | | | | | |
| 22 | 32014476 | 32026810 | PISD | | | PISD | | |
| 22 | 32017452 | 32017517 | MIR7109 | | | | | |
| 22 | 32077333 | 32146120 | PRR14L | | | | | |
| 22 | 32149936 | 32303020 | DEPDC5 | | | DEPDC5 | | |
| 22 | 32329506 | 32341348 | C22orf24 | | | | | |
| 22 | 32340478 | 32353590 | YWHAH | | | YWHAH | | |
| 22 | 32439018 | 32509011 | SLC5A1 | | | | | |
| 22 | 32517963 | 32529456 | AP1B1P1 | | | | | |
| 22 | 32545518 | 32555243 | C22orf42 | | | | | |
| 22 | 32586421 | 32600718 | RFPL2 | | | | | |
| 22 | 32614462 | 32651318 | SLC5A4 | | | | | |
| 22 | 32750871 | 32757148 | RFPL3 | | | | | |
| 22 | 32755892 | 32767251 | RFPL3S | | | | | |
| 22 | 32772650 | 32780329 | LOC339666 | | | | | |
| 22 | 32783561 | 32808274 | RTCB | | | | | |
| 22 | 32809833 | 32853373 | BPIFC | | | | | |
| 22 | 32870706 | 32894818 | FBXO7 | | | | | |
| 22 | 32908539 | 33454377 | SYN3 | | | SYN3 | SYN3 | SYN3 |
| 22 | 33196801 | 33259028 | TIMP3 | | | | | |
| 22 | 33504514 | 33512280 | LOC105373006 | | | | | |

| | | | | | | | | |
|---|---|---|---|---|---|---|---|---|
| 22 | 33669061 | 34316416 | LARGE | | | LARGE | | |
| 22 | 33832567 | 33832655 | MIR4764 | | | | | |
| 22 | 34120971 | 34146803 | LARGE | | | LARGE | | |
| 22 | 35462128 | 35483380 | ISX | | | | | |
| 22 | 35515816 | 35627049 | LINC01399 | | | | | |
| 22 | 35653444 | 35691800 | HMGXB4 | | | | | |
| 22 | 35695267 | 35743987 | TOM1 | | | TOM1 | | |
| 22 | 35731632 | 35731751 | MIR3909 | | | | | |
| 22 | 35732713 | 35732792 | MIR6069 | | | | | |
| 22 | 35777059 | 35790207 | HMOX1 | | | | | |
| 22 | 35796115 | 35820495 | MCM5 | | | | | |
| 22 | 35937351 | 35950045 | RASD2 | | | | | |
| 22 | 36002810 | 36019401 | MB | | | | | |
| 22 | 36044423 | 36064456 | APOL6 | | | | | |
| 22 | 36113918 | 36125529 | APOL5 | | | | | |
| 22 | 36134782 | 36424585 | RBFOX2 | | | | | |
| 22 | 36536370 | 36556977 | APOL3 | | | | | |
| 22 | 36585175 | 36600879 | APOL4 | | | | | |
| 22 | 36622254 | 36636000 | APOL2 | | | | | |
| 22 | 36649116 | 36663577 | APOL1 | | | | | |
| 22 | 36677322 | 36784112 | MYH9 | | | | | |
| 22 | 36682892 | 36682953 | MIR6819 | | | | | |
| 22 | 36863092 | 36877687 | TXN2 | | | | | |
| 22 | 36883232 | 36903148 | FOXRED2 | | | | | |
| 22 | 36906896 | 36925277 | EIF3D | | | | | |
| 22 | 36956915 | 37098690 | CACNG2 | | CACNG2 | CACNG2 | | |
| 22 | 37099920 | 37163133 | LOC105373021 | | | | | |
| 22 | 37154245 | 37172177 | IFT27 | | | | | |
| 22 | 37196744 | 37215517 | PVALB | | | | | |
| 22 | 37257029 | 37274059 | NCF4 | | | | | |
| 22 | 37309674 | 37336479 | CSF2RB | | | | | |
| 22 | 37361288 | 37364213 | LL22NC01-81G9.3 | | | | | |
| 22 | 37387159 | 37403877 | TEX33 | | | | | |
| 22 | 37406899 | 37416224 | TST | | | | | |
| 22 | 37415682 | 37425863 | MPST | | | | | |
| 22 | 37447775 | 37459430 | KCTD17 | | | | | |
| 22 | 37461475 | 37505603 | TMPRSS6 | | | | | |
| 22 | 37521879 | 37545962 | IL2RB | | | | | |
| 22 | 37576205 | 37584330 | C1QTNF6 | | | | | |
| 22 | 37600276 | 37608416 | SSTR3 | | | SSTR3 | | |
| 22 | 37621300 | 37640339 | RAC2 | | | RAC2 | | |
| 22 | 37678423 | 37711389 | CYTH4 | | | | | |
| 22 | 37736684 | 37823505 | ELFN2 | | | | | |
| 22 | 37748230 | 37750879 | LOC100506271 | | | | | |
| 22 | 37763999 | 37823505 | ELFN2 | | | | | |
| 22 | 37865100 | 37882478 | MFNG | | | | | |
| 22 | 37886399 | 37915210 | CARD10 | | | | | |
| 22 | 37956470 | 37965410 | CDC42EP1 | | | | | |
| 22 | 37966252 | 37976024 | LGALS2 | | | | | |
| 22 | 38004480 | 38029571 | GGA1 | | | GGA1 | | |
| 22 | 38035683 | 38052050 | SH3BP1 | | | | | |
| 22 | 38037838 | 38054384 | LOC101927051 | | | | | |
| 22 | 38054736 | 38062939 | PDXP | | | | | |
| 22 | 38071612 | 38075809 | LGALS1 | | | | | |
| 22 | 38082343 | 38089485 | NOL12 | | | | | |
| 22 | 38092994 | 38172563 | TRIOBP | | | | | |
| 22 | 38201113 | 38203443 | H1F0 | | | | | |
| 22 | 38203911 | 38213183 | GCAT | | | | | |
| 22 | 38219388 | 38221502 | GALR3 | | | | | |
| 22 | 38226861 | 38240353 | ANKRD54 | | | | | |
| 22 | 38240278 | 38240378 | MIR658 | | | | | |
| 22 | 38243684 | 38243781 | MIR659 | | | | | |
| 22 | 38245378 | 38284789 | EIF3L | | | | | |
| 22 | 38302154 | 38338465 | MICALL1 | | | | | |
| 22 | 38339056 | 38349676 | C22orf23 | | | | | |
| 22 | 38349669 | 38437922 | POLR2F | | | | | |

| | | | | | | | | |
|---|---|---|---|---|---|---|---|---|
| 22 | 38363569 | 38363631 | MIR6820 | | | | | |
| 22 | 38368318 | 38380539 | SOX10 | | SOX10 | | | |
| 22 | 38384800 | 38384860 | MIR4534 | | | | | |
| 22 | 38453261 | 38471708 | PICK1 | | | PICK1 | | |
| 22 | 38474143 | 38479170 | SLC16A8 | | | | | |
| 22 | 38480895 | 38506676 | BAIAP2L2 | | | | | |
| 22 | 38507501 | 38577836 | PLA2G6 | | | | | |
| 22 | 38597938 | 38612517 | MAFF | | | | | |
| 22 | 38615297 | 38669040 | TMEM184B | | | | | |
| 22 | 38620485 | 38620727 | SNORA92 | | | | | |
| 22 | 38686696 | 38714089 | CSNK1E | | | | | |
| 22 | 38686696 | 38794527 | LOC400927-CSNK1E | | | | | |
| 22 | 38740669 | 38794931 | LOC400927 | | | | | |
| 22 | 38822332 | 38851205 | KCNJ4 | | KCNJ4 | | | |
| 22 | 38864066 | 38879452 | KDELR3 | | | | | |
| 22 | 38879442 | 38902345 | DDX17 | | | | | |
| 22 | 38914953 | 38966201 | DMC1 | | | | | |
| 22 | 38966504 | 38967363 | LOC105373031 | | | | | |
| 22 | 38974124 | 39052634 | FAM227A | | | | | |
| 22 | 39052657 | 39069855 | CBY1 | | | | | |
| 22 | 39077953 | 39080766 | TOMM22 | | | | | |
| 22 | 39081547 | 39096459 | JOSD1 | | | | | |
| 22 | 39101806 | 39129592 | GTPBP1 | | | | | |
| 22 | 39130718 | 39152024 | SUN2 | | | | | |
| 22 | 39174512 | 39190161 | DNAL4 | | | | | |
| 22 | 39214455 | 39240017 | NPTXR | | | | | |
| 22 | 39257431 | 39268339 | CBX6 | | | | | |
| 22 | 39353526 | 39359188 | APOBEC3A | | | | | |
| 22 | 39353526 | 39388783 | APOBEC3A_B | | | | | |
| 22 | 39378403 | 39394225 | APOBEC3B | | | | | |
| 22 | 39410264 | 39414825 | APOBEC3C | | | | | |
| 22 | 39417117 | 39429256 | APOBEC3D | | | | | |
| 22 | 39436672 | 39451975 | APOBEC3F | | | | | |
| 22 | 39473009 | 39483748 | APOBEC3G | | | | | |
| 22 | 39493228 | 39500072 | APOBEC3H | | | | | |
| 22 | 39526778 | 39548538 | CBX7 | | | | | |
| 22 | 39619363 | 39641060 | PDGFB | | | PDGFB | | |
| 22 | 39708886 | 39715670 | RPL3 | | | | | |
| 22 | 39709823 | 39709916 | SNORD83B | | | | | |
| 22 | 39711217 | 39711312 | SNORD83A | | | | | |
| 22 | 39712846 | 39712901 | RNU86 | | | | | |
| 22 | 39715056 | 39715118 | SNORD43 | | | | | |
| 22 | 39745953 | 39774394 | SYNGR1 | | SYNGR1 | SYNGR1 | SYNGR1 | SYNGR1 |
| 22 | 39795758 | 39833132 | TAB1 | | | | | |
| 22 | 39828164 | 39833133 | LOC100506472 | | | | | |
| 22 | 39853324 | 39888199 | MGAT3 | | | | | |
| 22 | 39896104 | 39914139 | MIEF1 | | | | | |
| 22 | 39916563 | 39918691 | ATF4 | | | | | |
| 22 | 39925097 | 39928860 | RPS19BP1 | | | | | |
| 22 | 39966757 | 40085740 | CACNA1I | | | CACNA1I | | |
| 22 | 40139048 | 40289794 | ENTHD1 | | | | | |
| 22 | 40297085 | 40369346 | GRAP2 | | | | | |
| 22 | 40390952 | 40426043 | FAM83F | | | | | |
| 22 | 40428335 | 40432581 | LOC100130899 | | | | | |
| 22 | 40440820 | 40731812 | TNRC6B | | | | | |
| 22 | 40742503 | 40762752 | ADSL | | | | | |
| 22 | 40766565 | 40806293 | SGSM3 | | | | | |
| 22 | 40806284 | 41032723 | MKL1 | | | | | |
| 22 | 40917803 | 40922711 | LOC101927257 | | | | | |
| 22 | 41075181 | 41078818 | MCHR1 | | MCHR1 | | | |
| 22 | 41165633 | 41215403 | SLC25A17 | | | | | |
| 22 | 41209886 | 41209962 | MIR4766 | | | | | |
| 22 | 41220538 | 41253012 | ST13 | | | | | |
| 22 | 41253084 | 41328823 | XPNPEP3 | | | | | |
| 22 | 41255553 | 41258130 | DNAJB7 | | | | | |
| 22 | 41347350 | 41369019 | RBX1 | | | | | |

| | | | | | | | | |
|---|---|---|---|---|---|---|---|---|
| 22 | 41469611 | 41469725 | SNORD140 | | | | | |
| 22 | 41488516 | 41488570 | MIR1281 | | | | | |
| 22 | 41488613 | 41593505 | EP300 | | | EP300 | | |
| 22 | 41601312 | 41627275 | L3MBTL2 | | | | | |
| 22 | 41625513 | 41636935 | CHADL | | | | | |
| 22 | 41640780 | 41698217 | RANGAP1 | | | | | |
| 22 | 41648995 | 41649054 | MIR6889 | | | | | |
| 22 | 41697506 | 41756151 | ZC3H7B | | | | | |
| 22 | 41763336 | 41795332 | TEF | | | | | |
| 22 | 41829491 | 41843027 | TOB2 | | | | | |
| 22 | 41855720 | 41864708 | PHF5A | | | | | |
| 22 | 41865128 | 41924993 | ACO2 | | ACO2 | | | |
| 22 | 41921805 | 41940610 | POLR3H | | | | | |
| 22 | 41947712 | 41956887 | LOC105373044 | | | | | |
| 22 | 41957013 | 41972670 | CSDC2 | | | | | |
| 22 | 41972889 | 41985871 | PMM1 | | | | | |
| 22 | 41994031 | 42017061 | DESI1 | | | | | |
| 22 | 42017166 | 42060052 | XRCC6 | | | XRCC6 | | |
| 22 | 42069936 | 42084913 | SNU13 | | | | | |
| 22 | 42086546 | 42094140 | C22orf46 | | | | | |
| 22 | 42095517 | 42195459 | MEI1 | | | | | |
| 22 | 42196625 | 42222303 | CCDC134 | | | | | |
| 22 | 42229082 | 42303312 | SREBF2 | | | | | |
| 22 | 42296947 | 42297016 | MIR33A | | | | | |
| 22 | 42305557 | 42310671 | SHISA8 | | | | | |
| 22 | 42319225 | 42319301 | MIR378I | | | | | |
| 22 | 42321035 | 42322821 | TNFRSF13C | | | | | |
| 22 | 42334724 | 42343168 | CENPM | | | | | |
| 22 | 42348190 | 42354946 | LINC00634 | | | | | |
| 22 | 42372930 | 42394225 | SEPT3 | | | | | |
| 22 | 42394728 | 42424477 | WBP2NL | | | | | |
| 22 | 42454337 | 42466846 | NAGA | | | | | |
| 22 | 42470254 | 42475442 | FAM109B | | | | | |
| 22 | 42475694 | 42480288 | SMDT1 | | | | | |
| 22 | 42481529 | 42521354 | NDUFA6 | | | | | |
| 22 | 42522500 | 42526883 | CYP2D6 | | | | | |
| 22 | 42522500 | 42526883 | LOC101929829 | | | | | |
| 22 | 42536213 | 42540576 | CYP2D7 | | | | | |
| 22 | 42556018 | 42679933 | TCF20 | | | TCF20 | | |
| 22 | 42665758 | 42670868 | OGFRP1 | | | | | |
| 22 | 42760405 | 42765214 | LINC01315 | | | | | |
| 22 | 42776413 | 42828401 | NFAM1 | | | | | |
| 22 | 42896584 | 42908566 | SERHL | | | | | |
| 22 | 42904340 | 42915829 | RRP7A | | | | | |
| 22 | 42949867 | 42970388 | SERHL2 | | | | | |
| 22 | 42969265 | 42978017 | RRP7BP | | | | | |
| 22 | 42979726 | 43010968 | POLDIP3 | | | | | |
| 22 | 43011250 | 43011399 | RNU12 | | | | | |
| 22 | 43013845 | 43045405 | CYB5R3 | | | CYB5R3 | | |
| 22 | 43035808 | 43036607 | ATP5L2 | | | | | |
| 22 | 43088120 | 43117307 | A4GALT | | | | | |
| 22 | 43192531 | 43253408 | ARFGAP3 | | | | | |
| 22 | 43265771 | 43411184 | PACSIN2 | | | PACSIN2 | | |
| 22 | 43434590 | 43448371 | LOC100506679 | | | | | |
| 22 | 43435522 | 43485434 | TTLL1 | | | | | |
| 22 | 43506753 | 43525718 | BIK | | | | | |
| 22 | 43528211 | 43539403 | MCAT | | | | | |
| 22 | 43547519 | 43559248 | TSPO | | | TSPO | | |
| 22 | 43562627 | 43583137 | TTLL12 | | | | | |
| 22 | 43599228 | 43739394 | SCUBE1 | | | | | |
| 22 | 43608679 | 43609667 | LOC105373051 | | | | | |
| 22 | 43671947 | 43679832 | LOC101927447 | | | | | |
| 22 | 43808019 | 43902800 | MPPED1 | | | | | |
| 22 | 43912133 | 44208217 | EFCAB6 | | | | | |
| 22 | 44220386 | 44258378 | SULT4A1 | | | SULT4A1 | | |
| 22 | 44275557 | 44287893 | PNPLA5 | | | | | |

| | | | | | | | | |
|---|---|---|---|---|---|---|---|---|
| 22 | 44319618 | 44343448 | PNPLA3 | | | PNPLA3 | | |
| 22 | 44351260 | 44392412 | SAMM50 | | | | | |
| 22 | 44395090 | 44565112 | PARVB | | | | | |
| 22 | 44568835 | 44604349 | PARVG | | | | | |
| 22 | 44639556 | 44708731 | KIAA1644 | | | | | |
| 22 | 44839206 | 44840668 | LOC101927526 | | | | | |
| 22 | 44888449 | 44894005 | LDOC1L | | | | | |
| 22 | 44965219 | 44968329 | LINC00207 | | | | | |
| 22 | 45002207 | 45021299 | LINC00229 | | | | | |
| 22 | 45064426 | 45133561 | PRR5 | | | | | |
| 22 | 45098077 | 45258664 | PRR5-ARHGAP8 | | | | | |
| 22 | 45148437 | 45258664 | ARHGAP8 | | | | | |
| 22 | 45277042 | 45405809 | PHF21B | | | | | |
| 22 | 45529638 | 45583890 | NUP50 | | | | | |
| 22 | 45588122 | 45636650 | KIAA0930 | | | | | |
| 22 | 45596834 | 45596900 | MIR1249 | | | | | |
| 22 | 45680867 | 45691755 | UPK3A | | | | | |
| 22 | 45705080 | 45737836 | FAM118A | | | | | |
| 22 | 45739944 | 45809500 | SMC1B | | | | | |
| 22 | 45809571 | 45828302 | RIBC2 | | | | | |
| 22 | 45898718 | 45997014 | FBLN1 | | | | | |
| 22 | 46000311 | 46001527 | LINC01589 | | | | | |
| 22 | 46067677 | 46241187 | ATXN10 | | | | | |
| 22 | 46156403 | 46156478 | MIR4762 | | | | | |
| 22 | 46316247 | 46373008 | WNT7B | | | | | |
| 22 | 46402495 | 46406657 | LOC730668 | | | | | |
| 22 | 46435786 | 46440748 | LINC00899 | | | | | |
| 22 | 46446338 | 46454402 | PRR34 | | | | | |
| 22 | 46481876 | 46509808 | MIRLET7BHG | | | | | |
| 22 | 46486923 | 46487006 | MIR3619 | | | | | |
| 22 | 46508628 | 46508702 | MIRLET7A3 | | | | | |
| 22 | 46509445 | 46509537 | MIR4763 | | | | | |
| 22 | 46509565 | 46509648 | MIRLET7B | | | | | |
| 22 | 46546498 | 46639653 | PPARA | | | | | |
| 22 | 46639909 | 46646193 | CDPF1 | | | | | |
| 22 | 46651559 | 46659219 | PKDREJ | | | | | |
| 22 | 46663860 | 46689905 | TTC38 | | | | | |
| 22 | 46691039 | 46726707 | GTSE1 | | | | | |
| 22 | 46731297 | 46753237 | TRMU | | | | | |
| 22 | 46756730 | 46933067 | CELSR1 | | | CELSR1 | | |
| 22 | 47016298 | 47075688 | GRAMD4 | | | | | |
| 22 | 47080306 | 47134152 | CERK | | | | | |
| 22 | 47158513 | 47311938 | TBC1D22A | | | | | |
| 22 | 47857047 | 47882860 | LL22NC03-75H12.2 | | | | | |
| 22 | 48016791 | 48027318 | LINC00898 | | | | | |
| 22 | 48027422 | 48251349 | LOC284930 | | | | | |
| 22 | 48670175 | 48670227 | MIR3201 | | | | | |
| 22 | 48885271 | 49147747 | FAM19A5 | | | | | |
| 22 | 48934711 | 48943199 | LOC284933 | | | | | |
| 22 | 48972117 | 49147747 | FAM19A5 | | | | | |
| 22 | 49176106 | 49176165 | MIR4535 | | | | | |
| 22 | 49262581 | 49294198 | LINC01310 | | | | | |
| 22 | 49808173 | 50051190 | C22orf34 | | | | | |
| 22 | 49937040 | 49937114 | MIR3667 | | | | | |
| 22 | 50014103 | 50051190 | C22orf34 | | | | | |
| 22 | 50166925 | 50221196 | BRD1 | | | | | |
| 22 | 50247496 | 50283726 | ZBED4 | | | | | |
| 22 | 50296853 | 50312106 | ALG12 | | | ALG12 | | |
| 22 | 50312277 | 50321188 | CRELD2 | | | | | |
| 22 | 50354142 | 50357720 | PIM3 | | | | | |
| 22 | 50356513 | 50356587 | MIR6821 | | | | | |
| 22 | 50432941 | 50451055 | IL17REL | | | | | |
| 22 | 50497819 | 50524358 | MLC1 | | MLC1 | | | |
| 22 | 50528434 | 50600116 | MOV10L1 | | | | | |
| 22 | 50609159 | 50618724 | PANX2 | | | | | |
| 22 | 50624359 | 50638027 | TRABD | | | | | |

| | | | | | | | | |
|---|---|---|---|---|---|---|---|---|
| 22 | 50639407 | 50656045 | SELO | | | | | |
| 22 | 50656117 | 50683400 | TUBGCP6 | | | TUBGCP6 | | |
| 22 | 50683612 | 50689834 | HDAC10 | | | | | |
| 22 | 50691330 | 50700188 | MAPK12 | | | | | |
| 22 | 50702141 | 50708822 | MAPK11 | | MAPK11 | | | |
| 22 | 50713407 | 50746001 | PLXNB2 | | | | | |
| 22 | 50750391 | 50765489 | DENND6B | | | | | |
| 22 | 50781745 | 50883518 | PPP6R2 | | | | | |
| 22 | 50883430 | 50913500 | SBF1 | | SBF1 | | | |
| 22 | 50920152 | 50924866 | ADM2 | | | | | |
| 22 | 50925212 | 50928750 | MIOX | | | | | |
| 22 | 50941375 | 50946135 | LMF2 | | | | | |
| 22 | 50946644 | 50963209 | NCAPH2 | | | | | |
| 22 | 50961996 | 50964868 | SCO2 | | | SCO2 | | |
| 22 | 50964180 | 50968514 | TYMP | | | | | |
| 22 | 50968837 | 50971008 | ODF3B | | | | | |
| 22 | 50986461 | 50989452 | KLHDC7B | | | | | |
| 22 | 50989540 | 51001328 | SYCE3 | | | | | |
| 22 | 51007289 | 51017096 | CPT1B | | | | | |
| 22 | 51007289 | 51021428 | CHKB-CPT1B | | | | | |
| 22 | 51017386 | 51033710 | CHKB | | | | | |
| 22 | 51039113 | 51049979 | MAPK8IP2 | | MAPK8IP2 | | | |
| 22 | 51061181 | 51066601 | ARSA | ARSA | | ARSA | | |
| 22 | 51113069 | 51171640 | SHANK3 | | SHANK3 | SHANK3 | | SHANK3 |
| 22 | 51174256 | 51176597 | LOC105373100 | | | | | |
| 22 | 51176651 | 51183727 | ACR | | | | | |
| 22 | 51195513 | 51238065 | RPL23AP82 | | | | | |
| 22 | 51205919 | 51222087 | RABL2B | | | | | |
| 22 | 51222156 | 51238065 | RPL23AP82 | | | | | |
| 23 | 192990 | 220022 | PLCXD1 | | | | | |
| 23 | 221416 | 230887 | GTPBP6 | | | | | |
| 23 | 281384 | 282054 | LINC00685 | | | | | |
| 23 | 294667 | 347690 | PPP2R3B | | | | | |
| 23 | 585078 | 620146 | SHOX | | | SHOX | | |
| 23 | 1314893 | 1331616 | CRLF2 | | | | | |
| 23 | 1387692 | 1428828 | CSF2RA | | | | | |
| 23 | 1412810 | 1412885 | MIR3690 | | | | | |
| 23 | 1455508 | 1501582 | IL3RA | | | | | |
| 23 | 1505044 | 1511039 | SLC25A6 | | | SLC25A6 | | |
| 23 | 1515917 | 1518291 | LINC00106 | | | | | |
| 23 | 1519423 | 1572655 | ASMTL | | | | | |
| 23 | 1581465 | 1656037 | P2RY8 | | | | | |
| 23 | 1710485 | 1721411 | AKAP17A | | | | | |
| 23 | 1714347 | 1761974 | ASMT | | | | | |
| 23 | 2137554 | 2419015 | DHRSX | | | | | |
| 23 | 2404454 | 2419008 | ZBED1 | | | | | |
| 23 | 2527231 | 2527295 | MIR6089 | | | | | |
| 23 | 2527305 | 2575270 | CD99P1 | | | | | |
| 23 | 2531031 | 2533388 | LINC00102 | | | | | |
| 23 | 2609227 | 2659350 | CD99 | | | | | |
| 23 | 2670092 | 2734541 | XG | | | | | |
| 23 | 2670336 | 2693037 | XGY2 | | | | | |
| 23 | 2746862 | 2800861 | GYG2 | | | | | |
| 23 | 2822010 | 2847416 | ARSD | | | | | |
| 23 | 2852672 | 2886351 | ARSE | | | ARSE | | |
| 23 | 2924653 | 2951426 | ARSH | | | | | |
| 23 | 2958274 | 3030770 | ARSF | | | | | |
| 23 | 3189860 | 3202694 | LINC01546 | | | | | |
| 23 | 3226608 | 3264684 | MXRA5 | | | | | |
| 23 | 3522383 | 3586231 | PRKX | | | PRKX | | |
| 23 | 3735575 | 3761935 | LOC389906 | | | | | |
| 23 | 4545240 | 4551613 | LOC101928201 | | | | | |
| 23 | 5808066 | 6146923 | NLGN4X | | | NLGN4X | NLGN4X | |
| 23 | 6301946 | 6302004 | MIR4770 | | | | | |
| 23 | 6451658 | 6453159 | VCX3A | | | VCX3A | | |
| 23 | 6966960 | 7066231 | PUDP | | | | | |

| | | | | | | | | |
|---|---|---|---|---|---|---|---|---|
| 23 | 7065900 | 7065978 | MIR4767 | | | | | |
| 23 | 7137471 | 7272682 | STS | STS | | STS | | |
| 23 | 7810302 | 7812184 | VCX | | | | | |
| 23 | 7866803 | 7895780 | PNPLA4 | | | | | |
| 23 | 8095005 | 8095102 | MIR651 | | | | | |
| 23 | 8137984 | 8139308 | VCX2 | | | | | |
| 23 | 8432870 | 8434551 | VCX3B | | | | | |
| 23 | 8496914 | 8700227 | ANOS1 | | | | ANOS1 | |
| 23 | 8758836 | 8769424 | FAM9A | | | | | |
| 23 | 8992272 | 9002168 | FAM9B | | | | | |
| 23 | 9431334 | 9687780 | TBL1X | | | | | |
| 23 | 9693452 | 9734005 | GPR143 | | | GPR143 | | |
| 23 | 9754495 | 9917481 | SHROOM2 | | | SHROOM2 | | |
| 23 | 9935397 | 9936042 | CLDN34 | | | | | |
| 23 | 9983794 | 10112518 | WWC3 | | | | | |
| 23 | 10124984 | 10205699 | CLCN4 | | | | | |
| 23 | 10413349 | 10535643 | MID1 | | | MID1 | | |
| 23 | 11129405 | 11141204 | HCCS | | | HCCS | | |
| 23 | 11155662 | 11683821 | ARHGAP6 | | | | | |
| 23 | 11311532 | 11318881 | AMELX | | | | | |
| 23 | 11776277 | 11793872 | MSL3 | | | | | |
| 23 | 12156584 | 12742642 | FRMPD4 | | | | | |
| 23 | 12809473 | 12842346 | PRPS2 | | | | | |
| 23 | 12885201 | 12908480 | TLR7 | | | | | |
| 23 | 12920935 | 12941288 | TLR8 | | | | | |
| 23 | 12993225 | 12995346 | TMSB4X | | | | | |
| 23 | 13053735 | 13062917 | FAM9C | | | | | |
| 23 | 13328770 | 13338052 | GS1-600G8.3 | | | | | |
| 23 | 13336767 | 13338518 | ATXN3L | | | | | |
| 23 | 13353359 | 13359944 | LINC01203 | | | | | |
| 23 | 13587693 | 13651694 | EGFL6 | | | | | |
| 23 | 13608410 | 13608465 | MIR6086 | | | | | |
| 23 | 13671224 | 13683527 | TCEANC | | | | | |
| 23 | 13707239 | 13727944 | RAB9A | | | RAB9A | | |
| 23 | 13730360 | 13752754 | TRAPPC2 | | | TRAPPC2 | | |
| 23 | 13752831 | 13787480 | OFD1 | | | OFD1 | | |
| 23 | 13789040 | 13956943 | GPM6B | | | | GPM6B | |
| 23 | 14024844 | 14048035 | GEMIN8 | | | | | |
| 23 | 14262386 | 14263545 | UBE2E4P | | | | | |
| 23 | 14547419 | 14749933 | GLRA2 | | | GLRA2 | GLRA2 | |
| 23 | 14861528 | 14891184 | FANCB | | | FANCB | | |
| 23 | 14891526 | 14939459 | MOSPD2 | | | | | |
| 23 | 15262108 | 15288589 | ASB9 | | | | | |
| 23 | 15299830 | 15333746 | ASB11 | | | | | |
| 23 | 15337572 | 15353676 | PIGA | | | | | |
| 23 | 15363712 | 15402535 | FIGF | | | | | |
| 23 | 15363712 | 15509432 | PIR-FIGF | | | | | |
| 23 | 15402923 | 15511711 | PIR | | | | | |
| 23 | 15518899 | 15574652 | BMX | | | | | |
| 23 | 15579155 | 15620192 | ACE2 | | | | | |
| 23 | 15621003 | 15639607 | GS1-594A7.3 | | | | | |
| 23 | 15645438 | 15683154 | TMEM27 | | | | | |
| 23 | 15693038 | 15721474 | CA5BP1 | | | | | |
| 23 | 15756411 | 15805748 | CA5B | | | | | |
| 23 | 15803838 | 15805712 | INE2 | | | | | |
| 23 | 15808573 | 15841382 | ZRSR2 | | | | | |
| 23 | 15843928 | 15873137 | AP1S2 | | | AP1S2 | | |
| 23 | 16141423 | 16171641 | GRPR | GRPR | | GRPR | | |
| 23 | 16185603 | 16189516 | MAGEB17 | | | | | |
| 23 | 16606121 | 16731102 | CTPS2 | | | | | |
| 23 | 16645134 | 16645208 | MIR548AM | | | | | |
| 23 | 16668280 | 16672791 | S100G | | | | | |
| 23 | 16737706 | 16780807 | SYAP1 | | | SYAP1 | SYAP1 | |
| 23 | 16804554 | 16862642 | TXLNG | | | | | |
| 23 | 16862774 | 16888534 | RBBP7 | | | | | |
| 23 | 16964813 | 17171403 | REPS2 | | | REPS2 | REPS2 | |

| | | | | | | | | |
|---|---|---|---|---|---|---|---|---|
| 23 | 17393542 | 17754113 | NHS | | | NHS | | |
| 23 | 17444003 | 17444077 | MIR4768 | | | | | |
| 23 | 17570469 | 17754113 | NHS | | | NHS | | |
| 23 | 17755568 | 17773108 | SCML1 | | | | | |
| 23 | 17818168 | 17879457 | RAI2 | | | | | |
| 23 | 17988292 | 18122764 | LINC01456 | | | | | |
| 23 | 18181050 | 18239024 | BEND2 | | | | | |
| 23 | 18257432 | 18372844 | SCML2 | | | | | |
| 23 | 18443724 | 18671749 | CDKL5 | | | CDKL5 | | |
| 23 | 18657807 | 18690223 | RS1 | | | RS1 | | |
| 23 | 18706762 | 18846034 | PPEF1 | | | PPEF1 | | |
| 23 | 18908413 | 19002480 | PHKA2 | | | | | |
| 23 | 19007424 | 19140755 | ADGRG2 | | | | | |
| 23 | 19362010 | 19379825 | PDHA1 | | | PDHA1 | | |
| 23 | 19378175 | 19533379 | MAP3K15 | | | | | |
| 23 | 19552082 | 19905744 | SH3KBP1 | | | SH3KBP1 | | |
| 23 | 19930979 | 19988382 | CXorf23 | | | | | |
| 23 | 20004934 | 20007897 | LOC729609 | | | | | |
| 23 | 20024830 | 20135114 | MAP7D2 | | | | | |
| 23 | 20035205 | 20035305 | MIR23C | | | | | |
| 23 | 20142635 | 20159966 | EIF1AX | | | | | |
| 23 | 20154183 | 20154531 | SCARNA9L | | | | | |
| 23 | 20158085 | 20158562 | EIF1AX | | | | | |
| 23 | 20168028 | 20284750 | RPS6KA3 | | | RPS6KA3 | | |
| 23 | 21392535 | 21672813 | CNKSR2 | | | | | |
| 23 | 21673466 | 21676505 | KLHL34 | | | | | |
| 23 | 21724089 | 21776278 | SMPX | | | | | |
| 23 | 21857655 | 21903541 | MBTPS2 | | | | | |
| 23 | 21874104 | 21876845 | YY2 | | | | | |
| 23 | 21958690 | 22012955 | SMS | | | SMS | | |
| 23 | 22050561 | 22191100 | PHEX | | | PHEX | | |
| 23 | 22277913 | 23311263 | PTCHD1 | | | | | |
| 23 | 22291029 | 22292576 | ZNF645 | | | | | |
| 23 | 23018077 | 23020206 | DDX53 | | | | | |
| 23 | 23352984 | 23414918 | PTCHD1 | | | | | |
| 23 | 23685644 | 23704514 | PRDX4 | | | | | |
| 23 | 23721776 | 23761407 | ACOT9 | | | | | |
| 23 | 23801274 | 23804340 | SAT1 | | | | | |
| 23 | 23851464 | 23926057 | APOO | | | | | |
| 23 | 23926122 | 23957624 | CXorf58 | | | | | |
| 23 | 24001832 | 24045303 | KLHL15 | | | | | |
| 23 | 24073064 | 24096927 | EIF2S3 | | | | | |
| 23 | 24164341 | 24234372 | ZFX | | | | | |
| 23 | 24328978 | 24331432 | SUPT20HL2 | | | | | |
| 23 | 24380877 | 24383541 | SUPT20HL1 | | | | | |
| 23 | 24483343 | 24568583 | PDK3 | | | | | |
| 23 | 24576203 | 24676354 | PCYT1B | | | | | |
| 23 | 24712063 | 25015102 | POLA1 | | | | | |
| 23 | 24762557 | 24762687 | SCARNA23 | | | | | |
| 23 | 25021812 | 25034065 | ARX | | | ARX | | |
| 23 | 26156459 | 26158853 | MAGEB18 | | | | | |
| 23 | 26210556 | 26213763 | MAGEB6 | | | | | |
| 23 | 26234285 | 26236387 | MAGEB5 | | | | | |
| 23 | 26576453 | 26579169 | VENTXP1 | | | | | |
| 23 | 27478327 | 27481458 | PPP4R3CP | | | | | |
| 23 | 27764925 | 27766938 | DCAF8L2 | | | | | |
| 23 | 27826106 | 27841131 | MAGEB10 | | | | | |
| 23 | 27996109 | 27999566 | DCAF8L1 | | | | | |
| 23 | 28513671 | 28513780 | MIR6134 | | | | | |
| 23 | 28605680 | 29974017 | IL1RAPL1 | IL1RAPL1 | | IL1RAPL1 | | |
| 23 | 29592394 | 29592475 | MIR4666B | | | | | |
| 23 | 30233674 | 30238206 | MAGEB2 | | | | | |
| 23 | 30248552 | 30255610 | MAGEB3 | | | | | |
| 23 | 30260056 | 30262308 | MAGEB4 | | | | | |
| 23 | 30261847 | 30270155 | MAGEB1 | | | | | |
| 23 | 30322538 | 30327495 | NR0B1 | | | NR0B1 | | |

| | | | | | | | | |
|---|---|---|---|---|---|---|---|---|
| 23 | 30576940 | 30596033 | CXorf21 | | | | | |
| 23 | 30671475 | 30749577 | GK | | | GK | | |
| 23 | 30845558 | 30907511 | TAB3 | | | | | |
| 23 | 31089357 | 31090170 | FTHL17 | | | | | |
| 23 | 31137344 | 31285024 | DMD | | | DMD | DMD | |
| 23 | 32601772 | 32601869 | MIR3915 | | | | | |
| 23 | 34147868 | 34150447 | FAM47A | | | | | |
| 23 | 34645180 | 34675405 | TMEM47 | | | | | |
| 23 | 34960912 | 34963034 | FAM47B | | | | | |
| 23 | 35816458 | 35821852 | MAGEB16 | | | | | |
| 23 | 35937850 | 36403434 | CFAP47 | | | | | |
| 23 | 36383740 | 36458375 | RP11-87M18.2 | | | | | |
| 23 | 37026431 | 37029739 | FAM47C | | | | | |
| 23 | 37060954 | 37061867 | FTH1P18 | | | | | |
| 23 | 37208527 | 37316548 | PRRG1 | | | | | |
| 23 | 37430821 | 37536750 | LANCL3 | | | | | |
| 23 | 37545132 | 37591383 | XK | | | | | |
| 23 | 37639269 | 37672714 | CYBB | | | CYBB | | |
| 23 | 37698088 | 37706889 | DYNLT3 | | | | | |
| 23 | 37850069 | 37850570 | HYPM | | | | | |
| 23 | 37865834 | 37988073 | SYTL5 | | | SYTL5 | | |
| 23 | 38008587 | 38080177 | SRPX | | | | | |
| 23 | 38128422 | 38186788 | RPGR | | | | | |
| 23 | 38211735 | 38280703 | OTC | | | OTC | | |
| 23 | 38420730 | 38548172 | TSPAN7 | | | TSPAN7 | TSPAN7 | |
| 23 | 38660500 | 38665783 | MID1IP1 | | | | | |
| 23 | 39164209 | 39186616 | LINC01281 | | | | | |
| 23 | 39226538 | 39251028 | LINC01282 | | | | | |
| 23 | 39520469 | 39520575 | MIR3937 | | | | | |
| 23 | 39910498 | 40036582 | BCOR | | | BCOR | | |
| 23 | 40122169 | 40146974 | LOC101927476 | | | | | |
| 23 | 40440215 | 40465888 | ATP6AP2 | | | ATP6AP2 | | |
| 23 | 40482817 | 40483391 | MPC1L | | | | | |
| 23 | 40486172 | 40506819 | CXorf38 | | | | | |
| 23 | 40508794 | 40594804 | MED14 | | | | | |
| 23 | 40594647 | 40597953 | MED14OS | | | | | |
| 23 | 40690469 | 40692449 | LOC100132831 | | | | | |
| 23 | 40944887 | 41095832 | USP9X | | | | | |
| 23 | 41152089 | 41152139 | MIR7641 | | | | | |
| 23 | 41192560 | 41209540 | DDX3X | | | | | |
| 23 | 41306712 | 41334905 | NYX | | | | | |
| 23 | 41374188 | 41782287 | CASK | | | CASK | | |
| 23 | 41548225 | 41556530 | GPR34 | | | | | |
| 23 | 41583407 | 41589388 | GPR82 | | | | | |
| 23 | 42636616 | 42637486 | PPP1R2P9 | | | | | |
| 23 | 43036242 | 43085847 | LOC101927501 | | | | | |
| 23 | 43514154 | 43606071 | MAOA | MAOA | | MAOA | | |
| 23 | 43625856 | 43741721 | MAOB | | | MAOB | | |
| 23 | 43808023 | 43832921 | NDP | | | NDP | | |
| 23 | 44007127 | 44202923 | EFHC2 | | | | | |
| 23 | 44382884 | 44402221 | FUNDC1 | | | | | |
| 23 | 44703248 | 44704134 | DUSP21 | | | | | |
| 23 | 44732420 | 44971857 | KDM6A | | | | | |
| 23 | 45007617 | 45060146 | CXorf36 | | | | | |
| 23 | 45364632 | 45386484 | LINC01204 | | | | | |
| 23 | 45590576 | 45591246 | LOC392452 | | | | | |
| 23 | 45605584 | 45605694 | MIR221 | | | | | |
| 23 | 45606420 | 45606530 | MIR222 | | | | | |
| 23 | 45707508 | 45710920 | LOC401585 | | | | | |
| 23 | 46185358 | 46187109 | LINC01186 | | | | | |
| 23 | 46306623 | 46334074 | KRBOX4 | | | | | |
| 23 | 46357159 | 46407910 | ZNF674 | | | ZNF674 | | |
| 23 | 46433121 | 46457931 | CHST7 | | | | | |
| 23 | 46458685 | 46618607 | SLC9A7 | | | | | |
| 23 | 46696346 | 46741791 | RP2 | | | | | |
| 23 | 46746853 | 46759139 | LINC01545 | | | | | |

| | | | | | | | | |
|---|---|---|---|---|---|---|---|---|
| 23 | 46771710 | 46920641 | JADE3 | | | | | |
| 23 | 46937753 | 46952713 | RGN | | | | | |
| 23 | 47001614 | 47004609 | NDUFB11 | | | NDUFB11 | | |
| 23 | 47004616 | 47046214 | RBM10 | | | | | |
| 23 | 47050198 | 47074527 | UBA1 | | | | | |
| 23 | 47064246 | 47065254 | INE1 | | | | | |
| 23 | 47077527 | 47089394 | CDK16 | | | | | |
| 23 | 47092313 | 47107727 | USP11 | | | | | |
| 23 | 47229998 | 47273098 | ZNF157 | | | | | |
| 23 | 47248048 | 47248175 | SNORA11C | | | | | |
| 23 | 47305560 | 47342345 | ZNF41 | | | ZNF41 | | |
| 23 | 47342114 | 47344626 | LINC01560 | | | | | |
| 23 | 47420498 | 47431320 | ARAF | | | | | |
| 23 | 47431299 | 47479256 | SYN1 | | | SYN1 | SYN1 | |
| 23 | 47441689 | 47446190 | TIMP1 | | | | | |
| 23 | 47446827 | 47446904 | MIR4769 | | | | | |
| 23 | 47483611 | 47489704 | CFP | | | | | |
| 23 | 47494918 | 47510003 | ELK1 | | | ELK1 | | |
| 23 | 47511190 | 47519510 | UXT | | | | | |
| 23 | 47566589 | 47596027 | CXXC1P1 | | | | | |
| 23 | 47696300 | 47781655 | ZNF81 | | | ZNF81 | | |
| 23 | 47834249 | 47863394 | ZNF182 | | | | | |
| 23 | 47863733 | 47869126 | SPACA5 | | | | | |
| 23 | 47867169 | 47869126 | SPACA5B | | | | | |
| 23 | 47915698 | 47931025 | ZNF630 | | | | | |
| 23 | 47967366 | 47980068 | SSX6 | | | | | |
| 23 | 47986602 | 47991995 | SPACA5 | | | | | |
| 23 | 47990038 | 47991995 | SPACA5B | | | | | |
| 23 | 48045655 | 48056199 | SSX5 | | | | | |
| 23 | 48114751 | 48126879 | SSX1 | | | | | |
| 23 | 48160984 | 48165614 | SSX9 | | | | | |
| 23 | 48205862 | 48216188 | SSX3 | | | | | |
| 23 | 48242956 | 48252785 | SSX4B | | | | | |
| 23 | 48242967 | 48271344 | SSX4 | | | | | |
| 23 | 48261523 | 48271355 | SSX4B | | | | | |
| 23 | 48316919 | 48328644 | SLC38A5 | | | SLC38A5 | | |
| 23 | 48334408 | 48344752 | FTSJ1 | | | FTSJ1 | | |
| 23 | 48367346 | 48379202 | PORCN | | | PORCN | | |
| 23 | 48380163 | 48387104 | EBP | | | | | |
| 23 | 48398074 | 48420997 | TBC1D25 | | | | | |
| 23 | 48432740 | 48439553 | RBM3 | | | | | |
| 23 | 48455879 | 48463582 | WDR13 | | | | | |
| 23 | 48542185 | 48549817 | WAS | | | | | |
| 23 | 48553944 | 48567406 | SUV39H1 | | | | | |
| 23 | 48620153 | 48632064 | GLOD5 | | | | | |
| 23 | 48644981 | 48652717 | GATA1 | | | | | |
| 23 | 48660486 | 48683380 | HDAC6 | | | HDAC6 | | |
| 23 | 48684922 | 48688279 | ERAS | | | | | |
| 23 | 48689501 | 48694040 | PCSK1N | | | | | |
| 23 | 48750729 | 48755426 | TIMM17B | | | TIMM17B | | |
| 23 | 48755194 | 48760420 | PQBP1 | | | PQBP1 | | |
| 23 | 48760456 | 48769235 | SLC35A2 | | | | | |
| 23 | 48770458 | 48776413 | PIM2 | | | | | |
| 23 | 48779302 | 48815648 | OTUD5 | | | | | |
| 23 | 48818638 | 48828251 | KCND1 | | | KCND1 | | |
| 23 | 48830130 | 48858675 | GRIPAP1 | | | | | |
| 23 | 48886237 | 48901043 | TFE3 | | | | | |
| 23 | 48910960 | 48927510 | CCDC120 | | | | | |
| 23 | 48928812 | 48931704 | PRAF2 | | | | | |
| 23 | 48932091 | 48958059 | WDR45 | | | | | |
| 23 | 48970322 | 48980151 | GPKOW | | | | | |
| 23 | 49019180 | 49024495 | MAGIX | | | | | |
| 23 | 49028183 | 49031468 | PLP2 | | | PLP2 | PLP2 | |
| 23 | 49031150 | 49042853 | PRICKLE3 | | | | | |
| 23 | 49044264 | 49058913 | SYP | | | | SYP | |
| 23 | 49061522 | 49089833 | CACNA1F | | | | CACNA1F | |

| | | | | | | | | |
|---|---|---|---|---|---|---|---|---|
| 23 | 49091926 | 49106987 | CCDC22 | | | | | |
| 23 | 49106896 | 49121288 | FOXP3 | | | | | |
| 23 | 49126305 | 49144555 | PPP1R3F | | | | | |
| 23 | 49160124 | 49176323 | GAGE10 | | | | | |
| 23 | 49178508 | 49294588 | GAGE12J | | | | | |
| 23 | 49178515 | 49294598 | GAGE2E | | | | | |
| 23 | 49188080 | 49294588 | GAGE13 | | | | | |
| 23 | 49188103 | 49313700 | GAGE8 | | | | | |
| 23 | 49197562 | 49223943 | GAGE2D | | | | | |
| 23 | 49197581 | 49313700 | GAGE2A | | | | | |
| 23 | 49197595 | 49214430 | GAGE2C | | | | | |
| 23 | 49197606 | 49294588 | GAGE8 | | | | | |
| 23 | 49207115 | 49214420 | GAGE2D | | | | | |
| 23 | 49207134 | 49304144 | GAGE2A | | | | | |
| 23 | 49207148 | 49223953 | GAGE2C | | | | | |
| 23 | 49207159 | 49214420 | GAGE8 | | | | | |
| 23 | 49216633 | 49294588 | GAGE2A | | | | | |
| 23 | 49216647 | 49294598 | GAGE2C | | | | | |
| 23 | 49216648 | 49223939 | GAGE4 | | | | | |
| 23 | 49216656 | 49223943 | GAGE5 | | | | | |
| 23 | 49216658 | 49304144 | GAGE8 | | | | | |
| 23 | 49216676 | 49223943 | GAGE12I | | | | | |
| 23 | 49216677 | 49223939 | GAGE7 | | | | | |
| 23 | 49226137 | 49233481 | GAGE2D | | | | | |
| 23 | 49226170 | 49233491 | GAGE2C | | | | | |
| 23 | 49235707 | 49242997 | GAGE2B | | | | | |
| 23 | 49296745 | 49304144 | GAGE12F | | | | | |
| 23 | 49296768 | 49304154 | GAGE12D | | | | | |
| 23 | 49296779 | 49304144 | GAGE12C | | | | | |
| 23 | 49296779 | 49304144 | GAGE12E | | | | | |
| 23 | 49296779 | 49304144 | GAGE12G | | | | | |
| 23 | 49296779 | 49304144 | GAGE12H | | | | | |
| 23 | 49296814 | 49304080 | GAGE12B | | | | | |
| 23 | 49306301 | 49313700 | GAGE12F | | | | | |
| 23 | 49306324 | 49313710 | GAGE12D | | | | | |
| 23 | 49306335 | 49313700 | GAGE12C | | | | | |
| 23 | 49306335 | 49313700 | GAGE12E | | | | | |
| 23 | 49306335 | 49313700 | GAGE12G | | | | | |
| 23 | 49306335 | 49313700 | GAGE12H | | | | | |
| 23 | 49306370 | 49313636 | GAGE12B | | | | | |
| 23 | 49315857 | 49323252 | GAGE12F | | | | | |
| 23 | 49315880 | 49323262 | GAGE12D | | | | | |
| 23 | 49315891 | 49323252 | GAGE12C | | | | | |
| 23 | 49315891 | 49323252 | GAGE12E | | | | | |
| 23 | 49315891 | 49323252 | GAGE12G | | | | | |
| 23 | 49315891 | 49323252 | GAGE12H | | | | | |
| 23 | 49315926 | 49323188 | GAGE12B | | | | | |
| 23 | 49325410 | 49332811 | GAGE12F | | | | | |
| 23 | 49325433 | 49332821 | GAGE12D | | | | | |
| 23 | 49325444 | 49332811 | GAGE12C | | | | | |
| 23 | 49325444 | 49332811 | GAGE12E | | | | | |
| 23 | 49325444 | 49332811 | GAGE12G | | | | | |
| 23 | 49325479 | 49332807 | GAGE6 | | | | | |
| 23 | 49325506 | 49332811 | GAGE12I | | | | | |
| 23 | 49334968 | 49342360 | GAGE12F | | | | | |
| 23 | 49334991 | 49342370 | GAGE12D | | | | | |
| 23 | 49335002 | 49342360 | GAGE12C | | | | | |
| 23 | 49335002 | 49342360 | GAGE12E | | | | | |
| 23 | 49335002 | 49342360 | GAGE12G | | | | | |
| 23 | 49335037 | 49342356 | GAGE6 | | | | | |
| 23 | 49335064 | 49342360 | GAGE12I | | | | | |
| 23 | 49344540 | 49351917 | GAGE12D | | | | | |
| 23 | 49344551 | 49351907 | GAGE12C | | | | | |
| 23 | 49344551 | 49351907 | GAGE12E | | | | | |
| 23 | 49344551 | 49351907 | GAGE12H | | | | | |
| 23 | 49354117 | 49361430 | GAGE2A | | | | | |

| | | | | | | | | | |
|---|---|---|---|---|---|---|---|---|---|
| 23 | 49363615 | 49373139 | GAGE1 | | | | | | |
| 23 | 49452053 | 49460596 | PAGE1 | | | | | | |
| 23 | 49593862 | 49598867 | PAGE4 | | | | | | |
| 23 | 49641326 | 49647168 | USP27X | | | | | | |
| 23 | 49687224 | 49863892 | CLCN5 | | | CLCN5 | | | |
| 23 | 49767753 | 49767844 | MIR532 | | | | | | |
| 23 | 49768108 | 49768194 | MIR188 | | | | | | |
| 23 | 49773038 | 49773122 | MIR500A | | | | | | |
| 23 | 49773571 | 49773635 | MIR362 | | | | | | |
| 23 | 49774329 | 49774413 | MIR501 | | | | | | |
| 23 | 49775279 | 49775358 | MIR500B | | | | | | |
| 23 | 49777848 | 49777945 | MIR660 | | | | | | |
| 23 | 49779205 | 49779291 | MIR502 | | | | | | |
| 23 | 49832214 | 49863892 | CLCN5 | | | CLCN5 | | | |
| 23 | 49955410 | 49965004 | AKAP4 | | | | | | |
| 23 | 50027539 | 50094911 | CCNB3 | | | | | | |
| 23 | 50108405 | 50213737 | DGKK | | | | | | |
| 23 | 50334642 | 50557044 | SHROOM4 | | | SHROOM4 | | | |
| 23 | 50653734 | 50659641 | BMP15 | | | | | | |
| 23 | 50838681 | 50914232 | LINC01284 | | | | | | |
| 23 | 51075082 | 51080377 | NUDT10 | | | | | | |
| 23 | 51149766 | 51151689 | CXorf67 | | | | | | |
| 23 | 51232862 | 51239459 | NUDT11 | | | | | | |
| 23 | 51242760 | 51250293 | LINC01496 | | | | | | |
| 23 | 51424145 | 51425447 | CENPVP1 | | | | | | |
| 23 | 51424145 | 51425447 | CENPVP2 | | | | | | |
| 23 | 51453924 | 51455226 | CENPVP1 | | | | | | |
| 23 | 51453924 | 51455226 | CENPVP2 | | | | | | |
| 23 | 51486480 | 51489326 | GSPT2 | | | | | | |
| 23 | 51546154 | 51645450 | MAGED1 | | | | | | |
| 23 | 51804922 | 51812368 | MAGED4 | | | | | | |
| 23 | 51804922 | 51812368 | MAGED4B | | | | | | |
| 23 | 51806442 | 51806569 | SNORA11D | | | | | | |
| 23 | 51806442 | 51806569 | SNORA11E | | | | | | |
| 23 | 51927918 | 51935364 | MAGED4B | | | | | | |
| 23 | 51927918 | 51935366 | MAGED4 | | | | | | |
| 23 | 51933717 | 51933844 | SNORA11D | | | | | | |
| 23 | 51933717 | 51933844 | SNORA11E | | | | | | |
| 23 | 52079698 | 52079784 | MIR8088 | | | | | | |
| 23 | 52112152 | 52118826 | XAGE2 | | | | | | |
| 23 | 52238809 | 52243953 | XAGE1B | | | | | | |
| 23 | 52238809 | 52243953 | XAGE1E | | | | | | |
| 23 | 52238962 | 52243953 | XAGE1B | | | | | | |
| 23 | 52238962 | 52243953 | XAGE1E | | | | | | |
| 23 | 52239018 | 52243953 | XAGE1B | | | | | | |
| 23 | 52239018 | 52243953 | XAGE1E | | | | | | |
| 23 | 52255219 | 52260154 | XAGE1B | | | | | | |
| 23 | 52255219 | 52260154 | XAGE1E | | | | | | |
| 23 | 52255219 | 52260210 | XAGE1B | | | | | | |
| 23 | 52255219 | 52260210 | XAGE1E | | | | | | |
| 23 | 52255219 | 52260363 | XAGE1B | | | | | | |
| 23 | 52255219 | 52260363 | XAGE1E | | | | | | |
| 23 | 52380347 | 52387021 | XAGE2 | | | | | | |
| 23 | 52511760 | 52516904 | XAGE1B | | | | | | |
| 23 | 52511760 | 52516904 | XAGE1E | | | | | | |
| 23 | 52511913 | 52516904 | XAGE1B | | | | | | |
| 23 | 52511913 | 52516904 | XAGE1E | | | | | | |
| 23 | 52511969 | 52516904 | XAGE1B | | | | | | |
| 23 | 52511969 | 52516904 | XAGE1E | | | | | | |
| 23 | 52528159 | 52533094 | XAGE1B | | | | | | |
| 23 | 52528159 | 52533094 | XAGE1E | | | | | | |
| 23 | 52528159 | 52533150 | XAGE1B | | | | | | |
| 23 | 52528159 | 52533150 | XAGE1E | | | | | | |
| 23 | 52528159 | 52533303 | XAGE1B | | | | | | |
| 23 | 52528159 | 52533303 | XAGE1E | | | | | | |
| 23 | 52541053 | 52545988 | XAGE1B | | | | | | |

| | | | | | | | | | |
|---|---|---|---|---|---|---|---|---|---|
| 23 | 52541053 | 52545988 | XAGE1E | | | | | | |
| 23 | 52541053 | 52546044 | XAGE1B | | | | | | |
| 23 | 52541053 | 52546044 | XAGE1E | | | | | | |
| 23 | 52541053 | 52546197 | XAGE1B | | | | | | |
| 23 | 52541053 | 52546197 | XAGE1E | | | | | | |
| 23 | 52651984 | 52662998 | SSX8 | | | | | | |
| 23 | 52673110 | 52683950 | SSX7 | | | | | | |
| 23 | 52725945 | 52736276 | SSX2B | | | | | | |
| 23 | 52725945 | 52790617 | SSX2 | | | | | | |
| 23 | 52780280 | 52790617 | SSX2B | | | | | | |
| 23 | 52825185 | 52826388 | SPANXN5 | | | | | | |
| 23 | 52841227 | 52847322 | XAGE5 | | | | | | |
| 23 | 52891557 | 52897119 | XAGE3 | | | | | | |
| 23 | 52926321 | 52937585 | FAM156A | | | | | | |
| 23 | 52928408 | 52985629 | FAM156B | | | | | | |
| 23 | 52976463 | 53024651 | FAM156A | | | | | | |
| 23 | 53078505 | 53109796 | GPR173 | | | | | | |
| 23 | 53111541 | 53117728 | TSPYL2 | | | | | | |
| 23 | 53123338 | 53173249 | KANTR | | | | | | |
| 23 | 53220502 | 53254604 | KDM5C | | | | | | |
| 23 | 53224592 | 53224670 | MIR6895 | | | | | | |
| 23 | 53228070 | 53228127 | MIR6894 | | | | | | |
| 23 | 53262057 | 53310796 | IQSEC2 | | | | | | |
| 23 | 53401069 | 53449677 | SMC1A | | | | SMC1A | | |
| 23 | 53432604 | 53432697 | MIR6857 | | | | | | |
| 23 | 53449804 | 53458068 | RIBC1 | | | | | | |
| 23 | 53458205 | 53461323 | HSD17B10 | | | | HSD17B10 | | |
| 23 | 53559056 | 53713674 | HUWE1 | | | | | | |
| 23 | 53583183 | 53583302 | MIR98 | | | | | | |
| 23 | 53584152 | 53584235 | MIRLET7F2 | | | | | | |
| 23 | 53963112 | 54069627 | PHF8 | | | | PHF8 | | |
| 23 | 54094756 | 54209714 | FAM120C | | | | | | |
| 23 | 54219255 | 54384438 | WNK3 | | | | WNK3 | | |
| 23 | 54466852 | 54471731 | TSR2 | | | | | | |
| 23 | 54471886 | 54522599 | FGD1 | | | | FGD1 | | |
| 23 | 54556643 | 54593720 | GNL3L | | | | | | |
| 23 | 54775331 | 54824673 | ITIH6 | | | | | | |
| 23 | 54834031 | 54842448 | MAGED2 | | | | | | |
| 23 | 54840802 | 54840933 | SNORA11 | | | | | | |
| 23 | 54946995 | 54957866 | TRO | | | | | | |
| 23 | 54959566 | 55024967 | PFKFB1 | | | | | | |
| 23 | 55026755 | 55034306 | APEX2 | | | | APEX2 | | |
| 23 | 55035487 | 55057497 | ALAS2 | | | | | | |
| 23 | 55101488 | 55105336 | PAGE2B | | | | | | |
| 23 | 55115484 | 55119269 | PAGE2 | | | | | | |
| 23 | 55169534 | 55187628 | FAM104B | | | | | | |
| 23 | 55207823 | 55208944 | MTRNR2L10 | | | | | | |
| 23 | 55209914 | 55210040 | SNORA109 | | | | | | |
| 23 | 55246779 | 55250541 | PAGE5 | | | | | | |
| 23 | 55284848 | 55291349 | PAGE3 | | | | | | |
| 23 | 55306520 | 55315219 | LOC100421746 | | | | | | |
| 23 | 55477927 | 55478015 | MIR4536 | | | | | | |
| 23 | 55478521 | 55480001 | MAGEH1 | | | | | | |
| 23 | 55511048 | 55515631 | USP51 | | | | | | |
| 23 | 55649832 | 55652621 | FOXR2 | | | | | | |
| 23 | 55744109 | 55785207 | RRAGB | | | | | | |
| 23 | 56258821 | 56314322 | KLF8 | | | | KLF8 | | |
| 23 | 56590025 | 56593443 | UBQLN2 | | | | | | |
| 23 | 56755717 | 56844004 | LINC01420 | | | | | | |
| 23 | 56763220 | 56764017 | UQCRBP1 | | | | | | |
| 23 | 57002802 | 57021988 | SPIN3 | | | | | | |
| 23 | 57146114 | 57147989 | SPIN2B | | | | | | |
| 23 | 57162082 | 57164058 | SPIN2A | | | | | | |
| 23 | 57313109 | 57515629 | FAAH2 | | | | | | |
| 23 | 57618268 | 57623910 | ZXDB | | | | | | |
| 23 | 57703499 | 57706693 | NLRP2P | | | | | | |

| | | | | | | | | |
|---|---|---|---|---|---|---|---|---|
| 23 | 57931863 | 57937067 | ZXDA | | | | | |
| 23 | 62567106 | 62571218 | SPIN4 | | | | | |
| 23 | 62646438 | 62780873 | LINC01278 | | | | | |
| 23 | 62854847 | 63005426 | ARHGEF9 | | | | ARHGEF9 | |
| 23 | 62890075 | 62891382 | ARHGEF9-IT1 | | | | | |
| 23 | 63005881 | 63005967 | MIR1468 | | | | | |
| 23 | 63404996 | 63425624 | AMER1 | | | | | |
| 23 | 63444071 | 63450511 | ASB12 | | | | | |
| 23 | 63487960 | 63615333 | MTMR8 | | | | | |
| 23 | 64135681 | 64254624 | ZC4H2 | | | | | |
| 23 | 64708614 | 64727767 | ZC3H12B | | | | | |
| 23 | 64732461 | 64754686 | LAS1L | | | | | |
| 23 | 64770501 | 64772301 | FRMD8P1 | | | | | |
| 23 | 64887510 | 64961793 | MSN | | | | | |
| 23 | 65238711 | 65238821 | MIR223 | | | | | |
| 23 | 65241579 | 65259967 | VSIG4 | | | | | |
| 23 | 65382390 | 65487230 | HEPH | | | | | |
| 23 | 65815481 | 65859140 | EDA2R | | | | | |
| 23 | 66763873 | 66950461 | AR | | | AR | AR | |
| 23 | 67262185 | 67653299 | OPHN1 | | | OPHN1 | | |
| 23 | 67718623 | 67757127 | YIPF6 | | | | | |
| 23 | 67867510 | 67945684 | STARD8 | | | | | |
| 23 | 68048839 | 68062006 | EFNB1 | | | | | |
| 23 | 68380580 | 68385365 | PJA1 | | | | | |
| 23 | 68399399 | 68429767 | LINC00269 | | | | | |
| 23 | 68725077 | 68752351 | FAM155B | | | | | |
| 23 | 68835910 | 69259321 | EDA | | | EDA | | |
| 23 | 69242706 | 69242773 | MIR676 | | | | | |
| 23 | 69260391 | 69269788 | AWAT2 | | | | | |
| 23 | 69282340 | 69284029 | OTUD6A | | | | | |
| 23 | 69353317 | 69386173 | IGBP1 | | | IGBP1 | | |
| 23 | 69397332 | 69425553 | DGAT2L6 | | | | | |
| 23 | 69454504 | 69460511 | AWAT1 | | | | | |
| 23 | 69478015 | 69479654 | P2RY4 | | | | | |
| 23 | 69488184 | 69501690 | ARR3 | | | | | |
| 23 | 69502021 | 69504852 | RAB41 | | | | | |
| 23 | 69506210 | 69509798 | PDZD11 | | | | | |
| 23 | 69509878 | 69640774 | KIF4A | | | | | |
| 23 | 69642880 | 69653241 | GDPD2 | | | | | |
| 23 | 69664704 | 69725339 | DLG3 | | | DLG3 | DLG3 | |
| 23 | 69748789 | 70128567 | TEX11 | | | | | |
| 23 | 70145429 | 70150975 | SLC7A3 | | | SLC7A3 | | |
| 23 | 70279096 | 70293276 | SNX12 | | | SNX12 | | |
| 23 | 70315998 | 70323384 | FOXO4 | | | | | |
| 23 | 70323738 | 70326638 | CXorf65 | | | | | |
| 23 | 70327253 | 70331481 | IL2RG | | | | | |
| 23 | 70338405 | 70362304 | MED12 | | | | | |
| 23 | 70364680 | 70391051 | NLGN3 | NLGN3 | | NLGN3 | | |
| 23 | 70430034 | 70948962 | BCYRN1 | | | | | |
| 23 | 70435061 | 70445065 | GJB1 | | | | | |
| 23 | 70459473 | 70474499 | ZMYM3 | | | ZMYM3 | | |
| 23 | 70503041 | 70521018 | NONO | | | | | |
| 23 | 70521597 | 70525221 | ITGB1BP2 | | | | | |
| 23 | 70586088 | 70750211 | TAF1 | | | | | |
| 23 | 70711530 | 70712299 | INGX | | | | | |
| 23 | 70711845 | 70750375 | TAF1 | | | | | |
| 23 | 70752911 | 70795747 | OGT | | | | | |
| 23 | 70797873 | 70833433 | ACRC | | | | | |
| 23 | 70835765 | 70838367 | CXCR3 | | | | | |
| 23 | 70917045 | 70923256 | LINC00891 | | | | | |
| 23 | 70917045 | 70923256 | LOC100132741 | | | | | |
| 23 | 70934223 | 70938135 | CXorf49 | | | | | |
| 23 | 70934223 | 70938135 | CXorf49B | | | | | |
| 23 | 70983139 | 70987051 | CXorf49 | | | | | |
| 23 | 70983139 | 70987051 | CXorf49B | | | | | |
| 23 | 71130937 | 71363424 | NHSL2 | | | | | |

| | | | | | | | | |
|---|---|---|---|---|---|---|---|---|
| 23 | 71264258 | 71264811 | RPS26P11 | | | | | |
| 23 | 71346960 | 71351751 | RGAG4 | | | | | |
| 23 | 71364033 | 71381600 | FLJ44635 | | | | | |
| 23 | 71401525 | 71483814 | PIN4 | | | | | |
| 23 | 71424506 | 71458858 | ERCC6L | | | | | |
| 23 | 71492452 | 71497141 | RPS4X | | | | | |
| 23 | 71521487 | 71527037 | CITED1 | | | | | |
| 23 | 71549365 | 71792953 | HDAC8 | | | | | |
| 23 | 71798663 | 71934029 | PHKA1 | | | | | |
| 23 | 71908798 | 71932190 | LOC101928259 | | | | | |
| 23 | 72001688 | 72002484 | LINC00684 | | | | | |
| 23 | 72001688 | 72002484 | LOC100132304 | | | | | |
| 23 | 72064875 | 72068636 | DMRTC1 | | | | | |
| 23 | 72064875 | 72068636 | DMRTC1B | | | | | |
| 23 | 72091858 | 72095622 | DMRTC1 | | | | | |
| 23 | 72091858 | 72095622 | DMRTC1B | | | | | |
| 23 | 72158002 | 72158798 | LINC00684 | | | | | |
| 23 | 72158002 | 72158798 | LOC100132304 | | | | | |
| 23 | 72161567 | 72163589 | FAM226A | | | | | |
| 23 | 72161567 | 72163589 | FAM226B | | | | | |
| 23 | 72218226 | 72225551 | PABPC1L2B | | | | | |
| 23 | 72297176 | 72299351 | PABPC1L2A | | | | | |
| 23 | 72300005 | 72304474 | PABPC1L2B | | | | | |
| 23 | 72345875 | 72347919 | NAP1L6 | | | | | |
| 23 | 72432136 | 72434710 | NAP1L2 | | | | | |
| 23 | 72667089 | 72674421 | CDX4 | | | | | |
| 23 | 72744110 | 72782921 | MAP2K4P1 | | | | | |
| 23 | 72782983 | 72906944 | CHIC1 | | | | | |
| 23 | 73012039 | 73049066 | TSIX | | | | | |
| 23 | 73040485 | 73072588 | XIST | | | XIST | | |
| 23 | 73164158 | 73290217 | JPX | | | | | |
| 23 | 73247970 | 73513409 | FTX | | | | | |
| 23 | 73438211 | 73438296 | MIR421 | | | | | |
| 23 | 73438381 | 73438453 | MIR374B | | | | | |
| 23 | 73438383 | 73438453 | MIR374C | | | | | |
| 23 | 73506938 | 73507044 | MIR545 | | | | | |
| 23 | 73507120 | 73507192 | MIR374A | | | | | |
| 23 | 73524024 | 73524869 | ZCCHC13 | | | | | |
| 23 | 73641327 | 73753764 | SLC16A2 | | | | | |
| 23 | 73802810 | 73834461 | RLIM | | | | | |
| 23 | 73952690 | 74145287 | KIAA2022 | | | KIAA2022 | | |
| 23 | 74273006 | 74376175 | ABCB7 | | | | | |
| 23 | 74493893 | 74524732 | UPRT | | | | | |
| 23 | 74588261 | 74743337 | ZDHHC15 | | | ZDHHC15 | | |
| 23 | 74960372 | 74962914 | TTC3P1 | | | | | |
| 23 | 75002822 | 75005079 | MAGEE2 | | | | | |
| 23 | 75392763 | 75398033 | PBDC1 | | | | | |
| 23 | 75648045 | 75651746 | MAGEE1 | | | | | |
| 23 | 75878198 | 76234957 | MIR325HG | | | | | |
| 23 | 76139697 | 76139785 | MIR384 | | | | | |
| 23 | 76225828 | 76225926 | MIR325 | | | | | |
| 23 | 76709646 | 76712013 | FGF16 | | | | | |
| 23 | 76760355 | 77041755 | ATRX | | | ATRX | | |
| 23 | 77081861 | 77151065 | MAGT1 | | | | | |
| 23 | 77154960 | 77160881 | COX7B | | | | | |
| 23 | 77166152 | 77305892 | ATP7A | | | ATP7A | | |
| 23 | 77223457 | 77225135 | PGAM4 | | | | | |
| 23 | 77359665 | 77382324 | PGK1 | | | PGK1 | | |
| 23 | 77385244 | 77395179 | TAF9B | | | | | |
| 23 | 77526968 | 77583188 | CYSLTR1 | | | | | |
| 23 | 77911565 | 77914825 | ZCCHC5 | | | | | |
| 23 | 78003205 | 78012578 | LPAR4 | | | | | |
| 23 | 78156690 | 78156746 | MIR4328 | | | | | |
| 23 | 78200828 | 78217438 | P2RY10 | | | | P2RY10 | |
| 23 | 78426468 | 78427726 | GPR174 | | | | | |
| 23 | 78615880 | 78623049 | ITM2A | | | | | |

| | | | | | | | | |
|---|---|---|---|---|---|---|---|---|
| 23 | 79270254 | 79287268 | TBX22 | | | | | |
| 23 | 79483987 | 79590817 | CHMP1B2P | | | | | |
| 23 | 79591002 | 79700810 | FAM46D | | | | | |
| 23 | 79924986 | 80065233 | BRWD3 | | | | | |
| 23 | 80369199 | 80457441 | HMGN5 | | | | | |
| 23 | 80457302 | 80554046 | SH3BGRL | | | | | |
| 23 | 82763268 | 82764775 | POU3F4 | | | POU3F4 | | |
| 23 | 83116133 | 83141708 | CYLC1 | | | | | |
| 23 | 83313353 | 83442943 | RPS6KA6 | | | | | |
| 23 | 83572881 | 83757487 | HDX | | | | | |
| 23 | 84189156 | 84189896 | UBE2DNL | | | | | |
| 23 | 84258897 | 84348323 | APOOL | | | | | |
| 23 | 84347291 | 84363974 | SATL1 | | | | | |
| 23 | 84465711 | 84474295 | LOC101928128 | | | | | |
| 23 | 84498996 | 84528368 | ZNF711 | | | | | |
| 23 | 84532394 | 84634748 | POF1B | | | | | |
| 23 | 85090784 | 85090863 | MIR1321 | | | | | |
| 23 | 85116184 | 85302566 | CHM | | | CHM | CHM | |
| 23 | 85158640 | 85158712 | MIR361 | | | | | |
| 23 | 85224098 | 85302566 | CHM | | | CHM | CHM | |
| 23 | 85403454 | 86087605 | DACH2 | | | DACH2 | | |
| 23 | 86772714 | 86925050 | KLHL4 | | | | | |
| 23 | 88002225 | 88009785 | CPXCR1 | | | | | |
| 23 | 89176939 | 89177882 | TGIF2LX | | | TGIF2LX | | |
| 23 | 90669901 | 90693583 | PABPC5 | | | | | |
| 23 | 91090459 | 91878228 | PCDH11X | | | | | |
| 23 | 92079850 | 92079902 | MIR4454 | | | | | |
| 23 | 92925924 | 92928682 | NAP1L3 | | | | | |
| 23 | 92929011 | 92967273 | FAM133A | | | | | |
| 23 | 95592084 | 95592901 | BRDTP1 | | | | | |
| 23 | 95939661 | 96855597 | DIAPH2 | | | | | |
| 23 | 96138906 | 96140450 | RPA4 | | | | | |
| 23 | 96783362 | 96819534 | DIAPH2 | | | | | |
| 23 | 98716599 | 99194841 | XRCC6P5 | | | | | |
| 23 | 99546641 | 99665271 | PCDH19 | | | | | |
| 23 | 99839789 | 99854882 | TNMD | | | | | |
| 23 | 99882104 | 99892101 | TSPAN6 | | | | TSPAN6 | |
| 23 | 99899162 | 99926296 | SRPX2 | | | | | |
| 23 | 99929488 | 99987135 | SYTL4 | | | SYTL4 | | |
| 23 | 100075347 | 100096509 | CSTF2 | | | | | |
| 23 | 100098312 | 100129334 | NOX1 | | | | | |
| 23 | 100168430 | 100183898 | XKRX | | | | | |
| 23 | 100224696 | 100245820 | ARL13A | | | | | |
| 23 | 100264333 | 100307105 | TRMT2B | | | | | |
| 23 | 100333835 | 100351355 | TMEM35 | | | | | |
| 23 | 100353170 | 100396279 | CENPI | | | | | |
| 23 | 100474932 | 100519485 | DRP2 | | | | DRP2 | |
| 23 | 100523240 | 100548059 | TAF7L | | | | | |
| 23 | 100600643 | 100603708 | TIMM8A | | | TIMM8A | | |
| 23 | 100604434 | 100645784 | BTK | | | BTK | | |
| 23 | 100645877 | 100651142 | RPL36A | | | | | |
| 23 | 100645877 | 100669128 | RPL36A-HNRNPH2 | | | | | |
| 23 | 100652778 | 100663001 | GLA | | | GLA | | |
| 23 | 100663120 | 100669128 | HNRNPH2 | | | | | |
| 23 | 100673250 | 100750798 | ARMCX4 | | | | | |
| 23 | 100805513 | 100809675 | ARMCX1 | | | | | |
| 23 | 100870107 | 100872991 | ARMCX6 | | | | | |
| 23 | 100878119 | 100882831 | ARMCX3 | | | | | |
| 23 | 100910267 | 100914876 | ARMCX2 | | | | | |
| 23 | 101087084 | 101112549 | NXF5 | | | NXF5 | | |
| 23 | 101137259 | 101187039 | ZMAT1 | | | | | |
| 23 | 101380659 | 101382684 | TCEAL2 | | | | | |
| 23 | 101394932 | 101397388 | TCEAL6 | | | | | |
| 23 | 101408678 | 101410986 | BEX5 | | | | | |
| 23 | 101470279 | 101481785 | TCP11X2 | | | | | |
| 23 | 101502093 | 101581634 | NXF2 | | | | | |

| | | | | | | | | |
|---|---|---|---|---|---|---|---|---|
| 23 | 101502093 | 101581634 | NXF2B | | | | | |
| 23 | 101615315 | 101694929 | NXF2 | | | | | |
| 23 | 101615315 | 101694929 | NXF2B | | | | | |
| 23 | 101715239 | 101726732 | TCP11X2 | | | | | |
| 23 | 101768609 | 101771699 | TMSB15A | | | | | |
| 23 | 101804892 | 101826621 | NXF4 | | | | | |
| 23 | 101854095 | 101859085 | ARMCX5 | | | | | |
| 23 | 101854275 | 101972661 | ARMCX5-GPRASP2 | | | | | |
| 23 | 101854425 | 101859085 | ARMCX5 | | | | | |
| 23 | 101906293 | 101914010 | GPRASP1 | | | | | |
| 23 | 101967103 | 101972661 | GPRASP2 | | | | | |
| 23 | 101975641 | 102007369 | BHLHB9 | | | | | |
| 23 | 102024094 | 102140338 | LINC00630 | | | | | |
| 23 | 102192199 | 102193228 | RAB40AL | | | RAB40AL | | |
| 23 | 102317580 | 102319168 | BEX1 | | | | | |
| 23 | 102330749 | 102348022 | NXF3 | | | | | |
| 23 | 102470019 | 102472128 | BEX4 | | | | | |
| 23 | 102507922 | 102510121 | TCEAL8 | | | | | |
| 23 | 102528617 | 102531797 | TCEAL5 | | | | | |
| 23 | 102564273 | 102565974 | BEX2 | | | | | |
| 23 | 102585113 | 102587251 | TCEAL7 | | | | | |
| 23 | 102611379 | 102613397 | WBP5 | | | | | |
| 23 | 102631250 | 102633092 | NGFRAP1 | | | | | |
| 23 | 102754680 | 102774417 | RAB40A | | | RAB40A | | |
| 23 | 102785694 | 102809881 | LOC105373300 | | | | | |
| 23 | 102831158 | 102842664 | TCEAL4 | | | | | |
| 23 | 102862833 | 102864855 | TCEAL3 | | | | | |
| 23 | 102883647 | 102885876 | TCEAL1 | | | | | |
| 23 | 102930425 | 102947484 | MORF4L2 | | | | | |
| 23 | 102962271 | 102983552 | GLRA4 | | | | | |
| 23 | 102965836 | 102968960 | TMEM31 | | | | | |
| 23 | 102973501 | 102983552 | GLRA4 | | | | | |
| 23 | 103031433 | 103047547 | PLP1 | | | PLP1 | PLP1 | |
| 23 | 103077254 | 103087212 | RAB9B | | | RAB9B | | |
| 23 | 103217199 | 103220563 | TMSB15B | | | | | |
| 23 | 103230501 | 103232935 | H2BFXP | | | | | |
| 23 | 103230501 | 103232935 | LOC100101478 | | | | | |
| 23 | 103265718 | 103268259 | H2BFWT | | | | | |
| 23 | 103294515 | 103297021 | H2BFM | | | | | |
| 23 | 103315068 | 103317502 | H2BFXP | | | | | |
| 23 | 103315068 | 103317502 | LOC100101478 | | | | | |
| 23 | 103343897 | 103401708 | SLC25A53 | | | | | |
| 23 | 103357216 | 103360538 | ZCCHC18 | | | | | |
| 23 | 103366999 | 103369317 | LOC286437 | | | | | |
| 23 | 103411155 | 103440582 | FAM199X | | | | | |
| 23 | 103494718 | 103499599 | ESX1 | | | | | |
| 23 | 103810995 | 105011822 | IL1RAPL2 | | | | | |
| 23 | 104463610 | 104465377 | TEX13A | | | | | |
| 23 | 105066535 | 105202602 | NRK | | | | | |
| 23 | 105277189 | 105282718 | SERPINA7 | | | | | |
| 23 | 105412297 | 105452949 | MUM1L1 | | | | | |
| 23 | 105855159 | 105922673 | CXorf57 | | | | | |
| 23 | 105937067 | 106040246 | RNF128 | | | | | |
| 23 | 106045918 | 106119377 | TBC1D8B | | | | | |
| 23 | 106143292 | 106146561 | RIPPLY1 | | | | | |
| 23 | 106143393 | 106174091 | CLDN2 | | | | | |
| 23 | 106183963 | 106243474 | MORC4 | | | | | |
| 23 | 106305117 | 106362057 | RBM41 | | | | | |
| 23 | 106366656 | 106449670 | NUP62CL | | | | | |
| 23 | 106449861 | 106487473 | PIH1D3 | | | | | |
| 23 | 106756212 | 106848474 | FRMPD3 | | | | | |
| 23 | 106871653 | 106894256 | PRPS1 | | | PRPS1 | | |
| 23 | 106956451 | 107019218 | TSC22D3 | | | | | |
| 23 | 107069083 | 107174867 | MID2 | | | MID2 | | |
| 23 | 107137826 | 107179210 | LOC101928335 | | | | | |
| 23 | 107224093 | 107225600 | TEX13B | | | | | |

| | | | | | | | | |
|---|---|---|---|---|---|---|---|---|
| 23 | 107288199 | 107322414 | VSIG1 | | | | | |
| 23 | 107327434 | 107334874 | PSMD10 | | | | | |
| 23 | 107334898 | 107397901 | ATG4A | | | | | |
| 23 | 107398836 | 107682727 | COL4A6 | | | | | |
| 23 | 107683073 | 107940775 | COL4A5 | | | COL4A5 | | |
| 23 | 107975726 | 107979607 | IRS4 | | | | | |
| 23 | 107979769 | 107982133 | LOC101928358 | | | | | |
| 23 | 108297771 | 108297820 | MIR6087 | | | | | |
| 23 | 108616134 | 108725285 | GUCY2F | | | | | |
| 23 | 108779009 | 108787927 | NXT2 | | | | | |
| 23 | 108866928 | 108868393 | KCNE5 | | | | | |
| 23 | 108884558 | 108976621 | ACSL4 | ACSL4 | | ACSL4 | | |
| 23 | 109113629 | 109113681 | MIR4454 | | | | | |
| 23 | 109245862 | 109421016 | TMEM164 | | | | | |
| 23 | 109298556 | 109298654 | MIR652 | | | | | |
| 23 | 109325345 | 109325446 | MIR3978 | | | | | |
| 23 | 109437413 | 109683461 | AMMECR1 | | | | | |
| 23 | 109468216 | 109468288 | SNORD96B | | | | | |
| 23 | 109662284 | 109699562 | RGAG1 | | | | | |
| 23 | 109763539 | 109766249 | TDGF1P3 | | | | | |
| 23 | 109917083 | 110039286 | CHRDL1 | | | | | |
| 23 | 110187512 | 110464173 | PAK3 | | | PAK3 | | |
| 23 | 110488326 | 110513774 | CAPN6 | | | | | |
| 23 | 110537006 | 110655460 | DCX | | | DCX | | |
| 23 | 110754889 | 110765627 | LINC00890 | | | | | |
| 23 | 110924345 | 110933623 | ALG13 | | | | | |
| 23 | 111017541 | 111326004 | TRPC5 | | | | | |
| 23 | 111119278 | 111147213 | TRPC5OS | | | | | |
| 23 | 111326252 | 111700473 | ZCCHC16 | | | | | |
| 23 | 111873878 | 111923375 | LHFPL1 | | | | | |
| 23 | 112018104 | 112084043 | AMOT | | | | | |
| 23 | 112023945 | 112024016 | MIR4329 | | | | | |
| 23 | 112285954 | 112763885 | LOC101928437 | | | | | |
| 23 | 112859586 | 113181506 | XACT | | | | | |
| 23 | 113818550 | 114144624 | HTR2C | | | HTR2C | HTR2C | |
| 23 | 113865258 | 113865386 | SNORA35 | | | | | |
| 23 | 113873917 | 113874002 | MIR764 | | | | | |
| 23 | 113886018 | 113886098 | MIR1912 | | | | | |
| 23 | 113887129 | 113887198 | MIR1264 | | | | | |
| 23 | 113949649 | 113949761 | MIR1298 | | | | | |
| 23 | 113997743 | 113997823 | MIR1911 | | | | | |
| 23 | 114058016 | 114058127 | MIR448 | | | | | |
| 23 | 114238537 | 114252207 | IL13RA2 | | | | | |
| 23 | 114345182 | 114468635 | LRCH2 | | | | | |
| 23 | 114423962 | 114427431 | RBMXL3 | | | | | |
| 23 | 114524282 | 114542123 | LUZP4 | | | | | |
| 23 | 114752496 | 114885179 | PLS3 | | | | | |
| 23 | 114956728 | 115085444 | DANT2 | | | | | |
| 23 | 115301957 | 115306225 | AGTR2 | | | AGTR2 | | |
| 23 | 115567746 | 115592625 | SLC6A14 | | | SLC6A14 | | |
| 23 | 115592852 | 115594194 | CT83 | | | | | |
| 23 | 117031775 | 117251303 | KLHL13 | | | | | |
| 23 | 117480035 | 117583923 | WDR44 | | | | | |
| 23 | 117520356 | 117520434 | MIR1277 | | | | | |
| 23 | 117629871 | 117820123 | DOCK11 | | | | | |
| 23 | 117861558 | 117928496 | IL13RA1 | | | | | |
| 23 | 117957705 | 117960936 | ZCCHC12 | | | | | |
| 23 | 117973518 | 118015977 | LINC01285 | | | | | |
| 23 | 118108576 | 118152318 | LONRF3 | | | | | |
| 23 | 118212597 | 118284542 | KIAA1210 | | | | | |
| 23 | 118370207 | 118378429 | PGRMC1 | | | | | |
| 23 | 118425491 | 118469573 | LOC101928336 | | | | | |
| 23 | 118533257 | 118588437 | SLC25A43 | | | | | |
| 23 | 118599995 | 118605359 | SLC25A5 | | | | | |
| 23 | 118672111 | 118699397 | CXorf56 | | | | | |
| 23 | 118708429 | 118718392 | UBE2A | | | | | |

| | | | | | | | | |
|---|---|---|---|---|---|---|---|---|
| 23 | 118722299 | 118739846 | NKRF | | | | | |
| 23 | 118749687 | 118827333 | SEPT6 | | | | | |
| 23 | 118780700 | 118780811 | MIR766 | | | | | |
| 23 | 118892575 | 118894165 | SOWAHD | | | | | |
| 23 | 118920466 | 118925622 | RPL39 | | | | | |
| 23 | 118921315 | 118921447 | SNORA69 | | | | | |
| 23 | 118967988 | 118986991 | UPF3B | | | | | |
| 23 | 119004494 | 119005791 | RNF113A | | | | | |
| 23 | 119005733 | 119010629 | NDUFA1 | | | | | |
| 23 | 119029935 | 119054679 | AKAP14 | | | | | |
| 23 | 119059012 | 119077735 | NKAP | | | | | |
| 23 | 119144673 | 119149508 | RHOXF1P1 | | | | | |
| 23 | 119170200 | 119280761 | RHOXF1 | | | | | |
| 23 | 119206228 | 119211707 | RHOXF2 | | | | | |
| 23 | 119206228 | 119211707 | RHOXF2B | | | | | |
| 23 | 119243010 | 119249847 | RHOXF1 | | | | | |
| 23 | 119251551 | 119253610 | LINC01402 | | | | | |
| 23 | 119292466 | 119297945 | RHOXF2 | | | | | |
| 23 | 119292466 | 119297945 | RHOXF2B | | | | | |
| 23 | 119370308 | 119379122 | NKAPP1 | | | | | |
| 23 | 119384606 | 119392251 | ZBTB33 | | | | | |
| 23 | 119392504 | 119445391 | TMEM255A | | | | | |
| 23 | 119495939 | 119517104 | ATP1B4 | | | | | |
| 23 | 119560002 | 119603204 | LAMP2 | | | LAMP2 | | |
| 23 | 119658445 | 119709684 | CUL4B | | | CUL4B | | |
| 23 | 119737743 | 119755016 | MCTS1 | | | | | |
| 23 | 119759528 | 119764005 | C1GALT1C1 | | | | | |
| 23 | 120006451 | 120009779 | CT47B1 | | | | | |
| 23 | 120011344 | 120066151 | CT47A1 | | | | | |
| 23 | 120011344 | 120066151 | CT47A10 | | | | | |
| 23 | 120011344 | 120066151 | CT47A11 | | | | | |
| 23 | 120011344 | 120066151 | CT47A12 | | | | | |
| 23 | 120011344 | 120066151 | CT47A2 | | | | | |
| 23 | 120011344 | 120066151 | CT47A3 | | | | | |
| 23 | 120011344 | 120066151 | CT47A4 | | | | | |
| 23 | 120011344 | 120066151 | CT47A5 | | | | | |
| 23 | 120011344 | 120066151 | CT47A8 | | | | | |
| 23 | 120011344 | 120066151 | CT47A9 | | | | | |
| 23 | 120067694 | 120071012 | CT47A1 | | | | | |
| 23 | 120067694 | 120071012 | CT47A10 | | | | | |
| 23 | 120067694 | 120071012 | CT47A11 | | | | | |
| 23 | 120067694 | 120071012 | CT47A12 | | | | | |
| 23 | 120067694 | 120071012 | CT47A2 | | | | | |
| 23 | 120067694 | 120071012 | CT47A3 | | | | | |
| 23 | 120067694 | 120071012 | CT47A4 | | | | | |
| 23 | 120067694 | 120071012 | CT47A5 | | | | | |
| 23 | 120067694 | 120071012 | CT47A6 | | | | | |
| 23 | 120067694 | 120071012 | CT47A8 | | | | | |
| 23 | 120067694 | 120071012 | CT47A9 | | | | | |
| 23 | 120072555 | 120075873 | CT47A1 | | | | | |
| 23 | 120072555 | 120075873 | CT47A10 | | | | | |
| 23 | 120072555 | 120075873 | CT47A11 | | | | | |
| 23 | 120072555 | 120075873 | CT47A12 | | | | | |
| 23 | 120072555 | 120075873 | CT47A2 | | | | | |
| 23 | 120072555 | 120075873 | CT47A3 | | | | | |
| 23 | 120072555 | 120075873 | CT47A4 | | | | | |
| 23 | 120072555 | 120075873 | CT47A5 | | | | | |
| 23 | 120072555 | 120075873 | CT47A6 | | | | | |
| 23 | 120072555 | 120075873 | CT47A8 | | | | | |
| 23 | 120072555 | 120075873 | CT47A9 | | | | | |
| 23 | 120077415 | 120080733 | CT47A1 | | | | | |
| 23 | 120077415 | 120080733 | CT47A10 | | | | | |
| 23 | 120077415 | 120080733 | CT47A11 | | | | | |
| 23 | 120077415 | 120080733 | CT47A12 | | | | | |
| 23 | 120077415 | 120080733 | CT47A2 | | | | | |
| 23 | 120077415 | 120080733 | CT47A3 | | | | | |

| | | | | | | | | |
|---|---|---|---|---|---|---|---|---|
| 23 | 120077415 | 120080733 | CT47A4 | | | | | |
| 23 | 120077415 | 120080733 | CT47A5 | | | | | |
| 23 | 120077415 | 120080733 | CT47A6 | | | | | |
| 23 | 120077415 | 120080733 | CT47A8 | | | | | |
| 23 | 120077415 | 120080733 | CT47A9 | | | | | |
| 23 | 120082276 | 120085594 | CT47A1 | | | | | |
| 23 | 120082276 | 120085594 | CT47A10 | | | | | |
| 23 | 120082276 | 120085594 | CT47A11 | | | | | |
| 23 | 120082276 | 120085594 | CT47A12 | | | | | |
| 23 | 120082276 | 120085594 | CT47A2 | | | | | |
| 23 | 120082276 | 120085594 | CT47A3 | | | | | |
| 23 | 120082276 | 120085594 | CT47A4 | | | | | |
| 23 | 120082276 | 120085594 | CT47A5 | | | | | |
| 23 | 120082276 | 120085594 | CT47A6 | | | | | |
| 23 | 120082276 | 120085594 | CT47A8 | | | | | |
| 23 | 120082276 | 120085594 | CT47A9 | | | | | |
| 23 | 120087136 | 120090454 | CT47A7 | | | | | |
| 23 | 120092019 | 120095337 | CT47A6 | | | | | |
| 23 | 120092019 | 120100198 | CT47A1 | | | | | |
| 23 | 120096880 | 120100198 | CT47A10 | | | | | |
| 23 | 120096880 | 120100198 | CT47A11 | | | | | |
| 23 | 120096880 | 120100198 | CT47A12 | | | | | |
| 23 | 120096880 | 120100198 | CT47A2 | | | | | |
| 23 | 120096880 | 120100198 | CT47A3 | | | | | |
| 23 | 120096880 | 120100198 | CT47A4 | | | | | |
| 23 | 120096880 | 120100198 | CT47A5 | | | | | |
| 23 | 120096880 | 120100198 | CT47A6 | | | | | |
| 23 | 120096880 | 120100198 | CT47A8 | | | | | |
| 23 | 120096880 | 120100198 | CT47A9 | | | | | |
| 23 | 120101740 | 120105058 | CT47A1 | | | | | |
| 23 | 120101740 | 120105058 | CT47A10 | | | | | |
| 23 | 120101740 | 120105058 | CT47A11 | | | | | |
| 23 | 120101740 | 120105058 | CT47A12 | | | | | |
| 23 | 120101740 | 120105058 | CT47A2 | | | | | |
| 23 | 120101740 | 120105058 | CT47A3 | | | | | |
| 23 | 120101740 | 120105058 | CT47A4 | | | | | |
| 23 | 120101740 | 120105058 | CT47A5 | | | | | |
| 23 | 120101740 | 120105058 | CT47A6 | | | | | |
| 23 | 120101740 | 120105058 | CT47A8 | | | | | |
| 23 | 120101740 | 120105058 | CT47A9 | | | | | |
| 23 | 120106600 | 120109918 | CT47A1 | | | | | |
| 23 | 120106600 | 120109918 | CT47A10 | | | | | |
| 23 | 120106600 | 120109918 | CT47A11 | | | | | |
| 23 | 120106600 | 120109918 | CT47A12 | | | | | |
| 23 | 120106600 | 120109918 | CT47A2 | | | | | |
| 23 | 120106600 | 120109918 | CT47A3 | | | | | |
| 23 | 120106600 | 120109918 | CT47A4 | | | | | |
| 23 | 120106600 | 120109918 | CT47A5 | | | | | |
| 23 | 120106600 | 120109918 | CT47A6 | | | | | |
| 23 | 120106600 | 120109918 | CT47A8 | | | | | |
| 23 | 120106600 | 120109918 | CT47A9 | | | | | |
| 23 | 120111460 | 120114778 | CT47A1 | | | | | |
| 23 | 120111460 | 120114778 | CT47A10 | | | | | |
| 23 | 120111460 | 120114778 | CT47A11 | | | | | |
| 23 | 120111460 | 120114778 | CT47A12 | | | | | |
| 23 | 120111460 | 120114778 | CT47A2 | | | | | |
| 23 | 120111460 | 120114778 | CT47A3 | | | | | |
| 23 | 120111460 | 120114778 | CT47A4 | | | | | |
| 23 | 120111460 | 120114778 | CT47A5 | | | | | |
| 23 | 120111460 | 120114778 | CT47A6 | | | | | |
| 23 | 120111460 | 120114778 | CT47A8 | | | | | |
| 23 | 120111460 | 120114778 | CT47A9 | | | | | |
| 23 | 120116320 | 120119638 | CT47A1 | | | | | |
| 23 | 120116320 | 120119638 | CT47A6 | | | | | |
| 23 | 120181461 | 120183796 | GLUD2 | | | GLUD2 | | |
| 23 | 120504825 | 120504907 | MIR3672 | | | | | |

| | | | | | | | | |
|---|---|---|---|---|---|---|---|---|
| 23 | 122318095 | 122624766 | GRIA3 | | | GRIA3 | GRIA3 | |
| 23 | 122734411 | 122866904 | THOC2 | | | | | |
| 23 | 122993661 | 123047829 | XIAP | | | | | |
| 23 | 123093082 | 123095191 | LOC101928402 | | | | | |
| 23 | 123094409 | 123236505 | STAG2 | | | STAG2 | | |
| 23 | 123480131 | 123507010 | SH2D1A | | | SH2D1A | | |
| 23 | 123509755 | 124097666 | TENM1 | | | | | |
| 23 | 124453968 | 124456950 | TEX13C | | | | | |
| 23 | 125243744 | 125249545 | LOC101928495 | | | | | |
| 23 | 125297481 | 125300080 | DCAF12L2 | | | | | |
| 23 | 125683365 | 125686842 | DCAF12L1 | | | | | |
| 23 | 125953746 | 125955768 | PRR32 | | | | | |
| 23 | 127184940 | 127186382 | ACTRT1 | | | | | |
| 23 | 128580477 | 128657482 | SMARCA1 | | | | | |
| 23 | 128674231 | 128726530 | OCRL | | | OCRL | OCRL | |
| 23 | 128779235 | 128788933 | APLN | | | | | |
| 23 | 128872945 | 128903525 | XPNPEP2 | | | | | |
| 23 | 128913891 | 128929176 | SASH3 | | | | | |
| 23 | 128937263 | 128977910 | ZDHHC9 | | | ZDHHC9 | | |
| 23 | 129040096 | 129063738 | UTP14A | | | | | |
| 23 | 129139163 | 129192058 | BCORL1 | | | | | |
| 23 | 129198894 | 129244688 | ELF4 | | | | | |
| 23 | 129263337 | 129299861 | AIFM1 | | | AIFM1 | | |
| 23 | 129305772 | 129318844 | RAB33A | | | RAB33A | | |
| 23 | 129336672 | 129402922 | ZNF280C | | | | | |
| 23 | 129473861 | 129507335 | SLC25A14 | | | SLC25A14 | | |
| 23 | 129518318 | 129519511 | GPR119 | | | | | |
| 23 | 129535942 | 129547317 | RBMX2 | | | | | |
| 23 | 129628914 | 129631421 | FAM45BP | | | | | |
| 23 | 129757356 | 130037291 | ENOX2 | | | | | |
| 23 | 130150442 | 130192120 | LINC01201 | | | | | |
| 23 | 130192215 | 130223859 | ARHGAP36 | | | | | |
| 23 | 130407482 | 130423403 | IGSF1 | | | | | |
| 23 | 130678047 | 130678974 | OR13H1 | | | | | |
| 23 | 130836677 | 130964671 | FIRRE | | | | | |
| 23 | 131157244 | 131209971 | STK26 | | | | | |
| 23 | 131211013 | 131262050 | FRMD7 | | | | | |
| 23 | 131337051 | 131566839 | RAP2C | | | | | |
| 23 | 131503342 | 131623996 | MBNL3 | | | | | |
| 23 | 131760037 | 131803915 | HS6ST2 | | | | | |
| 23 | 132159506 | 132162300 | USP26 | | | | | |
| 23 | 132350696 | 132352376 | TFDP3 | | | | | |
| 23 | 132435063 | 132549205 | GPC4 | | | | | |
| 23 | 132669775 | 133119673 | GPC3 | | | GPC3 | | |
| 23 | 133303407 | 133303482 | MIR363 | | | | | |
| 23 | 133303567 | 133303642 | MIR92A2 | | | | | |
| 23 | 133303700 | 133303796 | MIR19B2 | | | | | |
| 23 | 133303838 | 133303907 | MIR20B | | | | | |
| 23 | 133304070 | 133304141 | MIR18B | | | | | |
| 23 | 133304227 | 133304308 | MIR106A | | | | | |
| 23 | 133371076 | 133379808 | CCDC160 | | | | | |
| 23 | 133507341 | 133562822 | PHF6 | | | PHF6 | | |
| 23 | 133594174 | 133634698 | HPRT1 | | | HPRT1 | | |
| 23 | 133674214 | 133674292 | MIR450B | | | | | |
| 23 | 133674370 | 133674461 | MIR450A1 | | | | | |
| 23 | 133674537 | 133674637 | MIR450A2 | | | | | |
| 23 | 133675370 | 133675467 | MIR542 | | | | | |
| 23 | 133677406 | 133680660 | MIR503HG | | | | | |
| 23 | 133680357 | 133680428 | MIR503 | | | | | |
| 23 | 133680643 | 133680741 | MIR424 | | | | | |
| 23 | 133684053 | 133694428 | LINC00629 | | | | | |
| 23 | 133699872 | 133898352 | PLAC1 | | | | | |
| 23 | 133903595 | 133931262 | FAM122B | | | | | |
| 23 | 133930442 | 133988641 | FAM122C | | | | | |
| 23 | 134021655 | 134049374 | MOSPD1 | | | | | |
| 23 | 134083578 | 134087411 | LOC340581 | | | | | |

| | | | | | | | | |
|---|---|---|---|---|---|---|---|---|
| 23 | 134124967 | 134126503 | SMIM10 | | | | | |
| 23 | 134154533 | 134156566 | FAM127C | | | | | |
| 23 | 134166332 | 134167575 | FAM127A | | | | | |
| 23 | 134184962 | 134186221 | FAM127B | | | | | |
| 23 | 134229014 | 134232664 | SMIM10L2B | | | | | |
| 23 | 134252881 | 134254405 | LINC00633 | | | | | |
| 23 | 134254548 | 134257529 | LOC100287728 | | | | | |
| 23 | 134290460 | 134305751 | CT55 | | | | | |
| 23 | 134382535 | 134478012 | ZNF75D | | | | | |
| 23 | 134478695 | 134497338 | ZNF449 | | | | | |
| 23 | 134530353 | 134531672 | LOC100506790 | | | | | |
| 23 | 134555857 | 134561999 | SMIM10L2A | | | | | |
| 23 | 134654007 | 134654599 | DDX26B | | | | | |
| 23 | 134654554 | 134716460 | INTS6L | | | | | |
| 23 | 134847184 | 134891539 | CT45A1 | | | | | |
| 23 | 134847385 | 134856984 | CT45A7 | | | | | |
| 23 | 134866213 | 134874248 | CT45A6 | | | | | |
| 23 | 134866213 | 134874249 | CT45A2 | | | | | |
| 23 | 134866213 | 134874249 | CT45A8 | | | | | |
| 23 | 134866213 | 134874249 | CT45A9 | | | | | |
| 23 | 134866213 | 134874561 | CT45A5 | | | | | |
| 23 | 134866213 | 134891519 | CT45A4 | | | | | |
| 23 | 134866414 | 134874244 | CT45A7 | | | | | |
| 23 | 134883487 | 134891519 | CT45A3 | | | | | |
| 23 | 134883487 | 134891519 | CT45A6 | | | | | |
| 23 | 134883688 | 134891515 | CT45A7 | | | | | |
| 23 | 134928696 | 134936735 | CT45A4 | | | | | |
| 23 | 134945650 | 134975579 | CT45A10 | | | | | |
| 23 | 134963218 | 134971043 | CT45A7 | | | | | |
| 23 | 134975784 | 134995220 | SAGE1 | | | | | |
| 23 | 135044230 | 135056134 | MMGT1 | | | | | |
| 23 | 135067582 | 135129428 | SLC9A6 | | | SLC9A6 | | |
| 23 | 135228860 | 135293518 | FHL1 | | | | | |
| 23 | 135295378 | 135338641 | MAP7D3 | | | | | |
| 23 | 135383121 | 135499047 | ADGRG4 | | | | | |
| 23 | 135570124 | 135574598 | BRS3 | | | BRS3 | | |
| 23 | 135579237 | 135594503 | HTATSF1 | | | | | |
| 23 | 135614310 | 135638966 | VGLL1 | | | | | |
| 23 | 135633036 | 135633119 | MIR934 | | | | | |
| 23 | 135721701 | 135724588 | LINC00892 | | | | | |
| 23 | 135730335 | 135742549 | CD40LG | | | | | |
| 23 | 135747711 | 135863503 | ARHGEF6 | | | ARHGEF6 | | |
| 23 | 135951352 | 135962939 | RBMX | | | | | |
| 23 | 135961357 | 135961430 | SNORD61 | | | | | |
| 23 | 136112306 | 136113833 | GPR101 | | | | | |
| 23 | 136648345 | 136654259 | ZIC3 | | | ZIC3 | | |
| 23 | 137696891 | 137699799 | LINC00889 | | | | | |
| 23 | 137713733 | 138287185 | FGF13 | | | | | |
| 23 | 137749871 | 137749954 | MIR504 | | | | | |
| 23 | 137794268 | 137798763 | FGF13 | | | | | |
| 23 | 138528977 | 138531132 | SRD5A1P1 | | | | | |
| 23 | 138612894 | 138645617 | F9 | | | F9 | | |
| 23 | 138663929 | 138790381 | MCF2 | | | | | |
| 23 | 138808504 | 138914447 | ATP11C | | | | | |
| 23 | 139006306 | 139006390 | MIR505 | | | | | |
| 23 | 139037883 | 139047677 | CXorf66 | | | | | |
| 23 | 139173825 | 139175070 | LOC389895 | | | | | |
| 23 | 139585151 | 139587225 | SOX3 | | | SOX3 | | |
| 23 | 139791923 | 139854841 | LINC00632 | | | | | |
| 23 | 139865424 | 139866723 | CDR1 | | | | | |
| 23 | 140008336 | 140008384 | MIR320D2 | | | | | |
| 23 | 140084755 | 140097876 | SPANXB1 | | | | | |
| 23 | 140212214 | 140212321 | RNU6-2 | | | | | |
| 23 | 140269930 | 140271399 | LDOC1 | | | | | |
| 23 | 140590842 | 140738069 | SPANXA2-OT1 | | | | | |
| 23 | 140671795 | 140672860 | SPANXA1 | | | | | |

| | | | | | | | | |
|---|---|---|---|---|---|---|---|---|
| 23 | 140671795 | 140672860 | SPANXA2 | | | | | |
| 23 | 140677834 | 140678899 | SPANXA1 | | | | | |
| 23 | 140677834 | 140678899 | SPANXA2 | | | | | |
| 23 | 140713993 | 140714861 | LOC645188 | | | | | |
| 23 | 140785557 | 140786896 | SPANXD | | | | | |
| 23 | 140785567 | 140786655 | SPANXC | | | | | |
| 23 | 140926101 | 140985618 | MAGEC3 | | | | | |
| 23 | 140991641 | 140997187 | MAGEC1 | | | | | |
| 23 | 141290127 | 141293076 | MAGEC2 | | | | | |
| 23 | 142113703 | 142122066 | SPANXN4 | | | | | |
| 23 | 142596563 | 142605303 | SPANXN3 | | | | | |
| 23 | 142710594 | 142723926 | SLITRK4 | | | | | |
| 23 | 142795134 | 142803762 | SPANXN2 | | | | | |
| 23 | 142967172 | 142968357 | UBE2NL | | | | | |
| 23 | 144329106 | 144337728 | SPANXN1 | | | | | |
| 23 | 144899346 | 144907360 | SLITRK2 | | | SLITRK2 | | |
| 23 | 144908927 | 144911370 | TMEM257 | | | | | |
| 23 | 145074267 | 145074344 | MIR892C | | | | | |
| 23 | 145075792 | 145075869 | MIR890 | | | | | |
| 23 | 145076301 | 145076378 | MIR888 | | | | | |
| 23 | 145078186 | 145078261 | MIR892A | | | | | |
| 23 | 145078715 | 145078792 | MIR892B | | | | | |
| 23 | 145082570 | 145082649 | MIR891B | | | | | |
| 23 | 145109311 | 145109390 | MIR891A | | | | | |
| 23 | 145891301 | 145891929 | CXorf51A | | | | | |
| 23 | 145891301 | 145891929 | CXorf51B | | | | | |
| 23 | 145895621 | 145896249 | CXorf51A | | | | | |
| 23 | 145895621 | 145896249 | CXorf51B | | | | | |
| 23 | 146271221 | 146271305 | MIR513C | | | | | |
| 23 | 146280561 | 146280645 | MIR513B | | | | | |
| 23 | 146294980 | 146295109 | MIR513A1 | | | | | |
| 23 | 146307343 | 146307470 | MIR513A2 | | | | | |
| 23 | 146312237 | 146312361 | MIR506 | | | | | |
| 23 | 146312501 | 146312595 | MIR507 | | | | | |
| 23 | 146318430 | 146318545 | MIR508 | | | | | |
| 23 | 146331668 | 146331748 | MIR514B | | | | | |
| 23 | 146340277 | 146342133 | MIR509 | | | | | |
| 23 | 146353852 | 146353926 | MIR510 | | | | | |
| 23 | 146360764 | 146360862 | MIR514A1 | | | | | |
| 23 | 146363460 | 146363548 | MIR514A2 | | | | | |
| 23 | 146363460 | 146363548 | MIR514A3 | | | | | |
| 23 | 146366158 | 146366246 | MIR514A2 | | | | | |
| 23 | 146366158 | 146366246 | MIR514A3 | | | | | |
| 23 | 146990948 | 147032647 | FMR1 | FMR1 | | FMR1 | | |
| 23 | 147062848 | 147108187 | FMR1NB | | | FMR1NB | | |
| 23 | 147582138 | 148082193 | AFF2 | AFF2 | | AFF2 | | |
| 23 | 148558520 | 148586884 | IDS | | | IDS | | |
| 23 | 148609131 | 148621312 | LINC00893 | | | | | |
| 23 | 148622518 | 148632086 | CXorf40A | | | | | |
| 23 | 148663308 | 148669109 | MAGEA9 | | | | | |
| 23 | 148663308 | 148669116 | MAGEA9B | | | | | |
| 23 | 148674171 | 148676970 | HSFX2 | | | | | |
| 23 | 148674182 | 148676974 | HSFX1 | | | | | |
| 23 | 148678215 | 148713568 | TMEM185A | | | | | |
| 23 | 148769902 | 148798928 | MAGEA11 | | | | | |
| 23 | 148855725 | 148858517 | HSFX1 | | | | | |
| 23 | 148855729 | 148858528 | HSFX2 | | | | | |
| 23 | 148863592 | 148869399 | MAGEA9B | | | | | |
| 23 | 148863599 | 148869399 | MAGEA9 | | | | | |
| 23 | 148958632 | 149008599 | LINC00850 | | | | | |
| 23 | 149007562 | 149014609 | MAGEA8 | | | | | |
| 23 | 149100414 | 149106716 | CXorf40B | | | | | |
| 23 | 149106765 | 149185018 | LINC00894 | | | | | |
| 23 | 149396238 | 149396318 | MIR2114 | | | | | |
| 23 | 149531544 | 149682448 | MAMLD1 | | | | | |
| 23 | 149737046 | 149841616 | MTM1 | | | | | |

| | | | | | | | | |
|---|---|---|---|---|---|---|---|---|
| 23 | 149861836 | 149933576 | MTMR1 | | | | | |
| 23 | 149934808 | 150067289 | CD99L2 | | | | | |
| 23 | 150148980 | 150159248 | HMGB3 | | | | | |
| 23 | 150336693 | 150336798 | MIR4330 | | | | | |
| 23 | 150345055 | 150349937 | GPR50 | | | GPR50 | | |
| 23 | 150565656 | 150577836 | VMA21 | | | | | |
| 23 | 150732006 | 150845211 | PASD1 | | | | | |
| 23 | 150863729 | 150870063 | PRRG3 | | | | | |
| 23 | 150884507 | 150891664 | FATE1 | | | | | |
| 23 | 150903217 | 150914036 | CNGA2 | | | | | |
| 23 | 151081360 | 151093642 | MAGEA4 | | | | | |
| 23 | 151121595 | 151143151 | GABRE | | | GABRE | GABRE | |
| 23 | 151127049 | 151127130 | MIR224 | | | | | |
| 23 | 151128099 | 151128184 | MIR452 | | | | | |
| 23 | 151282520 | 151286411 | MAGEA5 | | | | | |
| 23 | 151282520 | 151307050 | MAGEA10-MAGEA5 | | | | | |
| 23 | 151301782 | 151307050 | MAGEA10 | | | | | |
| 23 | 151335633 | 151619831 | GABRA3 | | | GABRA3 | GABRA3 | |
| 23 | 151560690 | 151560771 | MIR105 | | | | | |
| 23 | 151561892 | 151562001 | MIR767 | | | | | |
| 23 | 151562883 | 151562964 | MIR105 | | | | | |
| 23 | 151806636 | 151821825 | GABRQ | | | GABRQ | GABRQ | |
| 23 | 151867231 | 151870825 | MAGEA6 | | | | | |
| 23 | 151876742 | 151883038 | CSAG3 | | | | | |
| 23 | 151883074 | 151887096 | MAGEA2 | | | | | |
| 23 | 151885383 | 151887079 | MAGEA2B | | | | | |
| 23 | 151895977 | 151903136 | CSAG4 | | | | | |
| 23 | 151899292 | 151903184 | MAGEA12 | | | | | |
| 23 | 151903226 | 151909518 | CSAG1 | | | | | |
| 23 | 151918386 | 151922408 | MAGEA2 | | | | | |
| 23 | 151918403 | 151920099 | MAGEA2B | | | | | |
| 23 | 151922444 | 151928738 | CSAG3 | | | | | |
| 23 | 151934651 | 151938240 | MAGEA3 | | | | | |
| 23 | 151995870 | 151999301 | CETN2 | | | CETN2 | | |
| 23 | 151999510 | 152037907 | NSDHL | | | | | |
| 23 | 152082985 | 152142025 | ZNF185 | | | | | |
| 23 | 152157367 | 152162671 | PNMA5 | | | | | |
| 23 | 152224765 | 152228827 | PNMA3 | | | | | |
| 23 | 152240796 | 152340864 | PNMA6A | | | | | |
| 23 | 152481521 | 152486116 | MAGEA1 | | | | | |
| 23 | 152599612 | 152618384 | ZNF275 | | | | | |
| 23 | 152683780 | 152687086 | ZFP92 | | | | | |
| 23 | 152710177 | 152711945 | TREX2 | | | | | |
| 23 | 152713122 | 152760983 | HAUS7 | | | | | |
| 23 | 152760346 | 152775012 | BGN | | | | | |
| 23 | 152801579 | 152848387 | ATP2B3 | | | | | |
| 23 | 152853382 | 152864632 | FAM58A | | | | | |
| 23 | 152864794 | 152865348 | LOC105373383 | | | | | |
| 23 | 152907896 | 152916781 | DUSP9 | | | | | |
| 23 | 152935187 | 152939816 | PNCK | | | PNCK | | |
| 23 | 152953751 | 152962048 | SLC6A8 | | | SLC6A8 | | |
| 23 | 152965946 | 152990201 | BCAP31 | | | | | |
| 23 | 152990322 | 153010216 | ABCD1 | | | ABCD1 | | |
| 23 | 153029650 | 153044801 | PLXNB3 | | | | | |
| 23 | 153046455 | 153051187 | SRPK3 | | | | | |
| 23 | 153051220 | 153059978 | IDH3G | | | | | |
| 23 | 153059629 | 153063967 | SSR4 | | | | | |
| 23 | 153067620 | 153096022 | PDZD4 | | | | | |
| 23 | 153126968 | 153151628 | L1CAM | | | L1CAM | | |
| 23 | 153146126 | 153154427 | LCA10 | | | | | |
| 23 | 153167984 | 153172620 | AVPR2 | | | AVPR2 | AVPR2 | |
| 23 | 153172829 | 153191714 | ARHGAP4 | | | | | |
| 23 | 153195279 | 153200607 | NAA10 | | | | | |
| 23 | 153200721 | 153210232 | RENBP | | | | | |
| 23 | 153213007 | 153235543 | HCFC1 | | | | | |
| 23 | 153237990 | 153248646 | TMEM187 | | | | | |

| | | | | | | | | |
|---|---|---|---|---|---|---|---|---|
| 23 | 153246547 | 153246627 | MIR3202 | | | | | |
| 23 | 153275956 | 153285342 | IRAK1 | | | | | |
| 23 | 153285370 | 153285440 | MIR718 | | | | | |
| 23 | 153287263 | 153363188 | MECP2 | MECP2 | | MECP2 | | |
| 23 | 153409697 | 153424507 | OPN1LW | | | | | |
| 23 | 153448084 | 153461637 | OPN1MW2 | | | | | |
| 23 | 153448084 | 153462352 | OPN1MW | | | | | |
| 23 | 153485202 | 153498755 | OPN1MW2 | | | | | |
| 23 | 153485202 | 153499470 | OPN1MW | | | | | |
| 23 | 153498929 | 153523564 | TEX28 | | | | | |
| 23 | 153524026 | 153558713 | TKTL1 | | | | | |
| 23 | 153576899 | 153603006 | FLNA | | | FLNA | | |
| 23 | 153607596 | 153609883 | EMD | | | | | |
| 23 | 153626405 | 153630680 | RPL10 | | | | | |
| 23 | 153628621 | 153628756 | SNORA70 | | | | | |
| 23 | 153629576 | 153640428 | DNASE1L1 | | | | | |
| 23 | 153639853 | 153650063 | TAZ | | | | | |
| 23 | 153652722 | 153656825 | CH17-340M24.3 | | | | | |
| 23 | 153656977 | 153664863 | ATP6AP1 | | | | | |
| 23 | 153665258 | 153671814 | GDI1 | | | GDI1 | GDI1 | |
| 23 | 153672472 | 153679002 | FAM50A | | | | | |
| 23 | 153678667 | 153678734 | MIR6858 | | | | | |
| 23 | 153686620 | 153701989 | PLXNA3 | | | | | |
| 23 | 153706107 | 153707596 | LAGE3 | | | | | |
| 23 | 153712055 | 153715009 | UBL4A | | | | | |
| 23 | 153715644 | 153719029 | SLC10A3 | | | | | |
| 23 | 153734489 | 153744566 | FAM3A | | | | | |
| 23 | 153759604 | 153775801 | G6PD | | | G6PD | | |
| 23 | 153770458 | 153793261 | IKBKG | | | IKBKG | | |
| 23 | 153799478 | 153800188 | FAM223A | | | | | |
| 23 | 153799478 | 153800188 | FAM223B | | | | | |
| 23 | 153813417 | 153815075 | CTAG1A | | | | | |
| 23 | 153813417 | 153815075 | CTAG1B | | | | | |
| 23 | 153845864 | 153847522 | CTAG1A | | | | | |
| 23 | 153845864 | 153847522 | CTAG1B | | | | | |
| 23 | 153860738 | 153861448 | FAM223A | | | | | |
| 23 | 153860738 | 153861448 | FAM223B | | | | | |
| 23 | 153880245 | 153881853 | CTAG2 | | | | | |
| 23 | 153903526 | 153979858 | GAB3 | | | | | |
| 23 | 153991016 | 154005964 | DKC1 | | | DKC1 | | |
| 23 | 153996802 | 153996932 | SNORA36A | | | | | |
| 23 | 153996870 | 153996931 | MIR664B | | | | | |
| 23 | 154003272 | 154003401 | SNORA56 | | | | | |
| 23 | 154006958 | 154033802 | MPP1 | | | | MPP1 | |
| 23 | 154051622 | 154062937 | SMIM9 | | | | | |
| 23 | 154064063 | 154250998 | F8 | | | F8 | | |
| 23 | 154113243 | 154113837 | H2AFB2 | | | | | |
| 23 | 154113316 | 154113833 | H2AFB1 | | | | | |
| 23 | 154113316 | 154113833 | H2AFB3 | | | | | |
| 23 | 154114634 | 154116336 | F8A1 | | | F8A1 | | |
| 23 | 154114649 | 154115765 | F8A2 | | | | | |
| 23 | 154114649 | 154115765 | F8A3 | | | | | |
| 23 | 154115634 | 154115733 | MIR1184 | | | | | |
| 23 | 154255063 | 154285191 | FUNDC2 | | | | | |
| 23 | 154289899 | 154299547 | CMC4 | | | | | |
| 23 | 154292308 | 154299547 | MTCP1 | | | MTCP1 | | |
| 23 | 154299694 | 154351349 | BRCC3 | | | | | |
| 23 | 154425283 | 154468122 | VBP1 | | | | | |
| 23 | 154487518 | 154493874 | RAB39B | | | | | |
| 23 | 154505495 | 154563990 | CLIC2 | | | | | |
| 23 | 154576449 | 154603823 | LOC101927830 | | | | | |
| 23 | 154576449 | 154603823 | TMLHE | | | | | |
| 23 | 154610354 | 154610948 | H2AFB2 | | | | | |
| 23 | 154610427 | 154610944 | H2AFB1 | | | | | |
| 23 | 154610427 | 154610944 | H2AFB3 | | | | | |
| 23 | 154611748 | 154613450 | F8A1 | | | F8A1 | | |

| | | | | | | | | |
|---|---|---|---|---|---|---|---|---|
| 23 | 154611763 | 154612879 | F8A2 | | | | | |
| 23 | 154611763 | 154612879 | F8A3 | | | | | |
| 23 | 154612748 | 154612847 | MIR1184 | | | | | |
| 23 | 154686574 | 154688276 | F8A1 | | | F8A1 | | |
| 23 | 154687145 | 154688261 | F8A2 | | | | | |
| 23 | 154687145 | 154688261 | F8A3 | | | | | |
| 23 | 154687177 | 154687276 | MIR1184 | | | | | |
| 23 | 154689075 | 154689669 | H2AFB2 | | | | | |
| 23 | 154689079 | 154689596 | H2AFB1 | | | | | |
| 23 | 154689079 | 154689596 | H2AFB3 | | | | | |
| 23 | 154696200 | 154723771 | LOC101927830 | | | | | |
| 23 | 154696200 | 154842622 | TMLHE | | | | | |
| 23 | 154842225 | 155012119 | SPRY3 | | | | | |
| 23 | 155110942 | 155173433 | VAMP7 | | | VAMP7 | | |
| 23 | 155227245 | 155240482 | IL9R | | | | | |
| 23 | 155255322 | 155257848 | DDX11L16 | | | | | |
| 24 | 142990 | 170022 | PLCXD1 | | | | | |
| 24 | 171416 | 180887 | GTPBP6 | | | | | |
| 24 | 231384 | 232054 | LINC00685 | | | | | |
| 24 | 244667 | 297690 | PPP2R3B | | | | | |
| 24 | 535078 | 570146 | SHOX | | | SHOX | | |
| 24 | 1264893 | 1281616 | CRLF2 | | | | | |
| 24 | 1337692 | 1378828 | CSF2RA | | | | | |
| 24 | 1362810 | 1362885 | MIR3690 | | | | | |
| 24 | 1405508 | 1451582 | IL3RA | | | | | |
| 24 | 1455044 | 1461039 | SLC25A6 | | | SLC25A6 | | |
| 24 | 1465917 | 1468291 | LINC00106 | | | | | |
| 24 | 1469423 | 1522655 | ASMTL | | | | | |
| 24 | 1531465 | 1606037 | P2RY8 | | | | | |
| 24 | 1660485 | 1671411 | AKAP17A | | | | | |
| 24 | 1664347 | 1711974 | ASMT | | | | | |
| 24 | 2087554 | 2369015 | DHRSX | | | | | |
| 24 | 2354454 | 2369008 | ZBED1 | | | | | |
| 24 | 2477231 | 2477295 | MIR6089 | | | | | |
| 24 | 2477305 | 2525270 | CD99P1 | | | | | |
| 24 | 2559227 | 2609350 | CD99 | | | | | |
| 24 | 2620336 | 2643037 | XGY2 | | | | | |
| 24 | 2654895 | 2655782 | SRY | | | SRY | | |
| 24 | 2709622 | 2734997 | RPS4Y1 | | | | | |
| 24 | 2803111 | 2850547 | ZFY | | | | | |
| 24 | 2871036 | 2970313 | LINC00278 | | | | | |
| 24 | 3447125 | 3448082 | TGIF2LY | | | TGIF2LY | | |
| 24 | 4868266 | 5610269 | PCDH11Y | | PCDH11Y | | | |
| 24 | 6110486 | 6111651 | TTTY23 | | | | | |
| 24 | 6110486 | 6111651 | TTTY23B | | | | | |
| 24 | 6114263 | 6117053 | TSPY2 | | | | | |
| 24 | 6225259 | 6229454 | LINC00280 | | | | | |
| 24 | 6258441 | 6279605 | TTTY1 | | | | | |
| 24 | 6258441 | 6279605 | TTTY1B | | | | | |
| 24 | 6274284 | 6296485 | TTTY2 | | | | | |
| 24 | 6274284 | 6296485 | TTTY2B | | | | | |
| 24 | 6311474 | 6315118 | TTTY21 | | | | | |
| 24 | 6311474 | 6315118 | TTTY21B | | | | | |
| 24 | 6317508 | 6325947 | TTTY7 | | | | | |
| 24 | 6317508 | 6325947 | TTTY7B | | | | | |
| 24 | 6339071 | 6341671 | TTTY8 | | | | | |
| 24 | 6339071 | 6341671 | TTTY8B | | | | | |
| 24 | 6733958 | 6742068 | AMELY | | | | | |
| 24 | 6778726 | 6959724 | TBL1Y | | | | | |
| 24 | 7142012 | 7249588 | PRKY | | | PRKY | | |
| 24 | 7567397 | 7569288 | TTTY16 | | | | | |
| 24 | 7672964 | 7678723 | TTTY12 | | | | | |
| 24 | 8551410 | 8551919 | TTTY18 | | | | | |
| 24 | 8572512 | 8573324 | TTTY19 | | | | | |
| 24 | 8651358 | 8685423 | TTTY11 | | | | | |
| 24 | 9154669 | 9160483 | RBMY1A3P | | | | | |

| | | | | | | | | |
|---|---|---|---|---|---|---|---|---|
| 24 | 9167488 | 9172441 | TTTY20 | | | | | |
| 24 | 9187188 | 9192791 | FAM197Y2P | | | | | |
| 24 | 9187188 | 9192791 | FAM197Y5P | | | | | |
| 24 | 9195451 | 9198202 | TSPY8 | | | | | |
| 24 | 9195451 | 9218480 | TSPY4 | | | | | |
| 24 | 9207465 | 9213071 | FAM197Y2P | | | | | |
| 24 | 9207465 | 9213071 | FAM197Y5P | | | | | |
| 24 | 9215730 | 9238826 | TSPY4 | | | | | |
| 24 | 9236029 | 9238826 | TSPY3 | | | | | |
| 24 | 9236048 | 9238663 | TSPY10 | | | | | |
| 24 | 9236075 | 9368285 | TSPY4 | | | | | |
| 24 | 9296351 | 9301954 | FAM197Y2P | | | | | |
| 24 | 9296351 | 9301954 | FAM197Y5P | | | | | |
| 24 | 9304563 | 9307358 | TSPY1 | | | | | |
| 24 | 9316661 | 9322263 | FAM197Y2P | | | | | |
| 24 | 9316661 | 9322263 | FAM197Y5P | | | | | |
| 24 | 9357274 | 9362877 | FAM197Y2P | | | | | |
| 24 | 9357274 | 9362877 | FAM197Y5P | | | | | |
| 24 | 9365488 | 9368285 | TSPY3 | | | | | |
| 24 | 9365507 | 9368122 | TSPY10 | | | | | |
| 24 | 9448329 | 9452762 | RBMY3AP | | | | | |
| 24 | 9528708 | 9531308 | TTTY8 | | | | | |
| 24 | 9528708 | 9531308 | TTTY8B | | | | | |
| 24 | 9544432 | 9552871 | TTTY7 | | | | | |
| 24 | 9544432 | 9552871 | TTTY7B | | | | | |
| 24 | 9555261 | 9558905 | TTTY21 | | | | | |
| 24 | 9555261 | 9558905 | TTTY21B | | | | | |
| 24 | 9573894 | 9596085 | TTTY2 | | | | | |
| 24 | 9573894 | 9596085 | TTTY2B | | | | | |
| 24 | 9590764 | 9611928 | TTTY1 | | | | | |
| 24 | 9590764 | 9611928 | TTTY1B | | | | | |
| 24 | 9638761 | 9650854 | TTTY22 | | | | | |
| 24 | 9748406 | 9749571 | TTTY23 | | | | | |
| 24 | 9748406 | 9749571 | TTTY23B | | | | | |
| 24 | 14517914 | 14533389 | GYG2P1 | | | | | |
| 24 | 14774297 | 14804153 | TTTY15 | | | | | |
| 24 | 14813159 | 14972768 | USP9Y | | | | | |
| 24 | 15016018 | 15030439 | DDX3Y | | | | | |
| 24 | 15360258 | 15592550 | UTY | | | | | |
| 24 | 15815446 | 15817902 | TMSB4Y | | | | | |
| 24 | 16097651 | 16098388 | VCY1B | | | | | |
| 24 | 16097651 | 16168838 | VCY | | | | | |
| 24 | 16168101 | 16168838 | VCY1B | | | | | |
| 24 | 16634487 | 16915913 | NLGN4Y | | NLGN4Y | NLGN4Y | NLGN4Y | |
| 24 | 19612837 | 19628622 | FAM41AY1 | | | | | |
| 24 | 19612837 | 19628622 | FAM41AY2 | | | | | |
| 24 | 19687040 | 19691332 | FAM224B | | | | | |
| 24 | 19687063 | 19691353 | FAM224A | | | | | |
| 24 | 19880859 | 19882440 | XKRY | | | | | |
| 24 | 19880859 | 19882440 | XKRY2 | | | | | |
| 24 | 19990139 | 19992099 | CDY2B | | | | | |
| 24 | 19990140 | 20139626 | CDY2A | | | | | |
| 24 | 20137667 | 20139627 | CDY2B | | | | | |
| 24 | 20297334 | 20298915 | XKRY | | | | | |
| 24 | 20297334 | 20298915 | XKRY2 | | | | | |
| 24 | 20488418 | 20492712 | FAM224A | | | | | |
| 24 | 20488439 | 20492736 | FAM224B | | | | | |
| 24 | 20551155 | 20566932 | FAM41AY1 | | | | | |
| 24 | 20551155 | 20566932 | FAM41AY2 | | | | | |
| 24 | 20708556 | 20750849 | HSFY2 | | | | | |
| 24 | 20708573 | 20750849 | HSFY1 | | | | | |
| 24 | 20708576 | 20750849 | HSFY2 | | | | | |
| 24 | 20743091 | 20752407 | TTTY9A | | | | | |
| 24 | 20743091 | 20752407 | TTTY9B | | | | | |
| 24 | 20891767 | 20901083 | TTTY9A | | | | | |
| 24 | 20891767 | 20901083 | TTTY9B | | | | | |

| | | | | | | | | | |
|---|---|---|---|---|---|---|---|---|---|
| 24 | 20893325 | 20935601 | HSFY1 | | | | | | |
| 24 | 20893325 | 20935621 | HSFY2 | | | | | | |
| 24 | 20933699 | 20935604 | HSFY1 | | | | | | |
| 24 | 20933700 | 20935621 | HSFY2 | | | | | | |
| 24 | 21034386 | 21239433 | TTTY14 | | | | | | |
| 24 | 21152525 | 21438350 | CD24 | | | | | | |
| 24 | 21617316 | 21665039 | BCORP1 | | | | | | |
| 24 | 21729243 | 21769056 | TXLNGY | | | | | | |
| 24 | 21867300 | 21906825 | KDM5D | | | | | | |
| 24 | 22627553 | 22681114 | TTTY10 | | | | | | |
| 24 | 22737596 | 22755040 | EIF1AY | | | | | | |
| 24 | 22917953 | 22942918 | RPS4Y2 | | | | | | |
| 24 | 23544859 | 23548246 | PRORY | | | | | | |
| 24 | 23557033 | 23563448 | RBMY2EP | | | | | | |
| 24 | 23673223 | 23687672 | RBMY1B | | | | | | |
| 24 | 23673257 | 23687672 | RBMY1A1 | | | | | | |
| 24 | 23673263 | 23687672 | RBMY1D | | | | | | |
| 24 | 23673263 | 23687672 | RBMY1E | | | | | | |
| 24 | 23696764 | 23711212 | RBMY1B | | | | | | |
| 24 | 23696798 | 23711212 | RBMY1A1 | | | | | | |
| 24 | 23696804 | 23711212 | RBMY1D | | | | | | |
| 24 | 23745485 | 23756552 | TTTY13 | | | | | | |
| 24 | 24026222 | 24040633 | RBMY1D | | | | | | |
| 24 | 24026222 | 24040633 | RBMY1E | | | | | | |
| 24 | 24026222 | 24040639 | RBMY1A1 | | | | | | |
| 24 | 24026222 | 24040673 | RBMY1B | | | | | | |
| 24 | 24049764 | 24064174 | RBMY1D | | | | | | |
| 24 | 24049764 | 24064174 | RBMY1E | | | | | | |
| 24 | 24049764 | 24064214 | RBMY1B | | | | | | |
| 24 | 24217902 | 24242154 | PRY | | | | | | |
| 24 | 24217902 | 24242154 | PRY2 | | | | | | |
| 24 | 24247838 | 24293631 | LOC101929148 | | | | | | |
| 24 | 24291111 | 24292978 | TTTY6 | | | | | | |
| 24 | 24291133 | 24292981 | TTTY6B | | | | | | |
| 24 | 24314688 | 24329089 | RBMY1F | | | | | | |
| 24 | 24314688 | 24329089 | RBMY1J | | | | | | |
| 24 | 24442944 | 24445023 | TTTY5 | | | | | | |
| 24 | 24455005 | 24462352 | RBMY2FP | | | | | | |
| 24 | 24455009 | 24564028 | RBMY1F | | | | | | |
| 24 | 24462824 | 24466531 | LOC100652931 | | | | | | |
| 24 | 24549622 | 24564028 | RBMY1J | | | | | | |
| 24 | 24585086 | 24630861 | LOC101929148 | | | | | | |
| 24 | 24585736 | 24587584 | TTTY6B | | | | | | |
| 24 | 24585739 | 24587606 | TTTY6 | | | | | | |
| 24 | 24636543 | 24660784 | PRY | | | | | | |
| 24 | 24636543 | 24660784 | PRY2 | | | | | | |
| 24 | 24997730 | 24998862 | TTTY17A | | | | | | |
| 24 | 24997730 | 24998862 | TTTY17B | | | | | | |
| 24 | 24997730 | 24998862 | TTTY17C | | | | | | |
| 24 | 25082601 | 25119431 | TTTY4 | | | | | | |
| 24 | 25082601 | 25119431 | TTTY4B | | | | | | |
| 24 | 25082601 | 25119431 | TTTY4C | | | | | | |
| 24 | 25130409 | 25151610 | BPY2 | | | | | | |
| 24 | 25130409 | 25151610 | BPY2B | | | | | | |
| 24 | 25130409 | 25151610 | BPY2C | | | | | | |
| 24 | 25275501 | 25345239 | DAZ1 | | | | | | |
| 24 | 25275501 | 25345254 | DAZ4 | | | | | | |
| 24 | 25365580 | 25437503 | DAZ3 | | | | | | |
| 24 | 25365580 | 25437503 | DAZ4 | | | | | | |
| 24 | 25365603 | 25437503 | DAZ2 | | | | | | |
| 24 | 26082873 | 26087769 | TTTY3 | | | | | | |
| 24 | 26082873 | 26087769 | TTTY3B | | | | | | |
| 24 | 26191376 | 26194161 | CDY1 | | | | | | |
| 24 | 26191376 | 26194161 | CDY1B | | | | | | |
| 24 | 26191939 | 26194161 | CDY1 | | | | | | |
| 24 | 26191939 | 26194161 | CDY1B | | | | | | |

| | | | | | | | | |
|---|---|---|---|---|---|---|---|---|
| 24 | 26329580 | 26333378 | CSPG4P1Y | | | | | |
| 24 | 26356113 | 26360978 | GOLGA2P2Y | | | | | |
| 24 | 26356113 | 26360978 | GOLGA2P3Y | | | | | |
| 24 | 26631478 | 26632610 | TTTY17A | | | | | |
| 24 | 26631478 | 26632610 | TTTY17B | | | | | |
| 24 | 26631478 | 26632610 | TTTY17C | | | | | |
| 24 | 26716348 | 26753172 | TTTY4 | | | | | |
| 24 | 26716348 | 26753172 | TTTY4B | | | | | |
| 24 | 26716348 | 26753172 | TTTY4C | | | | | |
| 24 | 26764150 | 26785352 | BPY2 | | | | | |
| 24 | 26764150 | 26785352 | BPY2B | | | | | |
| 24 | 26764150 | 26785352 | BPY2C | | | | | |
| 24 | 26909215 | 26959639 | DAZ3 | | | | | |
| 24 | 26909215 | 26959639 | DAZ4 | | | | | |
| 24 | 26979966 | 27053187 | DAZ3 | | | | | |
| 24 | 26979966 | 27053187 | DAZ4 | | | | | |
| 24 | 26979989 | 27053187 | DAZ2 | | | | | |
| 24 | 27177049 | 27198251 | BPY2 | | | | | |
| 24 | 27177049 | 27198251 | BPY2B | | | | | |
| 24 | 27177049 | 27198251 | BPY2C | | | | | |
| 24 | 27209229 | 27246039 | TTTY4 | | | | | |
| 24 | 27209229 | 27246039 | TTTY4B | | | | | |
| 24 | 27209229 | 27246039 | TTTY4C | | | | | |
| 24 | 27329789 | 27330920 | TTTY17A | | | | | |
| 24 | 27329789 | 27330920 | TTTY17B | | | | | |
| 24 | 27329789 | 27330920 | TTTY17C | | | | | |
| 24 | 27601457 | 27606322 | GOLGA2P2Y | | | | | |
| 24 | 27601457 | 27606322 | GOLGA2P3Y | | | | | |
| 24 | 27629054 | 27632852 | CSPG4P1Y | | | | | |
| 24 | 27768263 | 27770485 | CDY1 | | | | | |
| 24 | 27768263 | 27770485 | CDY1B | | | | | |
| 24 | 27768263 | 27771048 | CDY1 | | | | | |
| 24 | 27768263 | 27771048 | CDY1B | | | | | |
| 24 | 27874636 | 27879535 | TTTY3 | | | | | |
| 24 | 27874636 | 27879535 | TTTY3B | | | | | |
| 24 | 59100402 | 59115125 | SPRY3 | | | | | |
| 24 | 59213948 | 59276439 | VAMP7 | | | VAMP7 | | |
| 24 | 59330251 | 59343488 | IL9R | | | | | |
| 24 | 59358328 | 59360854 | DDX11L16 | | | | | |
